# MetaMeta: integrating metagenome analysis tools to improve taxonomic profiling

**DOI:** 10.1186/s40168-017-0318-y

**Published:** 2017-08-14

**Authors:** Vitor C. Piro, Marcel Matschkowski, Bernhard Y. Renard

**Affiliations:** 1 Research Group Bioinformatics (NG4), Robert Koch Institute, Nordufer 20, Berlin, 13353 Germany; 2 CAPES Foundation, Ministry of Education of Brazil, Brasília, 70040-020 DF Brazil

**Keywords:** Metagenomics, Taxonomic profiling, Binning, Pipeline

## Abstract

**Background:**

Many metagenome analysis tools are presently available to classify sequences and profile environmental samples. In particular, taxonomic profiling and binning methods are commonly used for such tasks. Tools available among these two categories make use of several techniques, e.g., read mapping, k-mer alignment, and composition analysis. Variations on the construction of the corresponding reference sequence databases are also common. In addition, different tools provide good results in different datasets and configurations. All this variation creates a complicated scenario to researchers to decide which methods to use. Installation, configuration and execution can also be difficult especially when dealing with multiple datasets and tools.

**Results:**

We propose MetaMeta: a pipeline to execute and integrate results from metagenome analysis tools. MetaMeta provides an easy workflow to run multiple tools with multiple samples, producing a single enhanced output profile for each sample. MetaMeta includes a database generation, pre-processing, execution, and integration steps, allowing easy execution and parallelization. The integration relies on the co-occurrence of organisms from different methods as the main feature to improve community profiling while accounting for differences in their databases.

**Conclusions:**

In a controlled case with simulated and real data, we show that the integrated profiles of MetaMeta overcome the best single profile. Using the same input data, it provides more sensitive and reliable results with the presence of each organism being supported by several methods. MetaMeta uses Snakemake and has six pre-configured tools, all available at BioConda channel for easy installation (conda install -c bioconda metameta). The MetaMeta pipeline is open-source and can be downloaded at: https://gitlab.com/rki_bioinformatics.

**Electronic supplementary material:**

The online version of this article (doi:10.1186/s40168-017-0318-y) contains supplementary material, which is available to authorized users.

## Background

A large and increasing number of metagenome analysis tools are presently available aiming to characterize environmental samples [1–4]. Motivated by the large amounts of data produced from whole metagenome shotgun (WMS) sequencing technologies, profiling of metagenomes has become more accessible, faster and applicable in real scenarios and tends to become the standard method for metagenomics analysis [5–7]. Tools which perform sequence classification based on WMS sequencing data come in different flavors. One basic approach is the *de novo* sequence assembly [8–10], which aims to reconstruct complete or near complete genomes from fragmented short sequences without any reference or prior knowledge. It is the method which provides the best resolution to assess the community composition. However, it is very difficult to produce meaningful assemblies from metagenomics data due to short read length, insufficient coverage, similar DNA sequences, and low abundant strains [11].

More commonly, methods use the WMS reads directly without assembly and are in general reference-based, meaning that they rely on previously obtained genome sequences to perform their analysis. In this category of applications, two standard definitions are employed: taxonomic profiling and binning tools. Profilers aim to analyze WMS sequences as a whole, predicting organisms and their relative abundances based on a given set of reference sequences. Binning tools aim to classify each sequence in a given sample individually, linking each one of them to the most probable organism of the reference set. Regardless of their conceptual differences, both groups of tools could be used to characterize microbial communities. Yet binning tools produce individual classification for each sequence and should be converted and normalized to be used as a taxonomic profiler.

Methods available among these two categories make use of several techniques, e.g. read mapping, k-mer alignment, and composition analysis. Variations on the construction of the reference databases, e.g., complete genome sequences, marker genes, protein sequences, are also common. Many of those techniques were developed to overcome the computational cost of dealing with the high throughput of modern sequencing technologies as well as the large number of reference genome sequences available.

The availability of several options for tools, parameters, databases, and techniques create a complicated scenario to researchers to decide which methods to use. Different tools provide good results in different scenarios, being more or less precise or sensitive in multiple configurations. It is hard to rely on their output for every study or sample variation. In addition, when more than one method is used, inconsistent results between tools using different reference sets are difficult to be integrated. Furthermore, installation, parameterization, and database creation as well as the lack of standard outputs are challenges not easily overcome.

We propose MetaMeta, a new pipeline for the joint execution and integration of metagenomic sequence classification tools. MetaMeta has several strengths: easy installation and set-up, support for multiple tools, samples and databases, improved final profile combining multiple results, out-of-the-box parallelization and high performance computing (HPC) integration, automated database download and set-up, custom database creation, integrated pre-processing step (read trimming, error correction, and sub-sampling) as well as standardized rules for integration of new tools. MetaMeta achieves more sensitive profiling results than single tools alone by merging their correct identifications and properly filtering out false identifications. MetaMeta was built with SnakeMake [12] and is open-source. The pipeline has six pre-configured tools that are automatically installed using Conda through the BioConda channel (https://bioconda.github.io). We encourage the integration of new tools, making it available to the community through a participative Git repository (via pull request). MetaMeta source-code is available at: https://github.com/pirovc/metameta.

## Implementation

MetaMeta executes and integrates metagenomic sequence classification tools. The integration is based on several tools’ output profiles and aims to improve organism identification and quantification. An optional pre-processing and sub-sampling step is included. The pipeline is generalized for binning and profiling tools, categories that were previously described in the CAMI (Critical Assessment of Metagenome Interpretation) challenge (http://www.cami-challenge.org). MetaMeta provides a pre-defined set of standardized rules to facilitate the integration of tools, easy parallelization and execution in high performance computing infrastructure. The pre-configured tools are available at the BioConda channel to facilitate download and installation, avoiding set-up problems and broken dependencies.

**Fig. 1:**
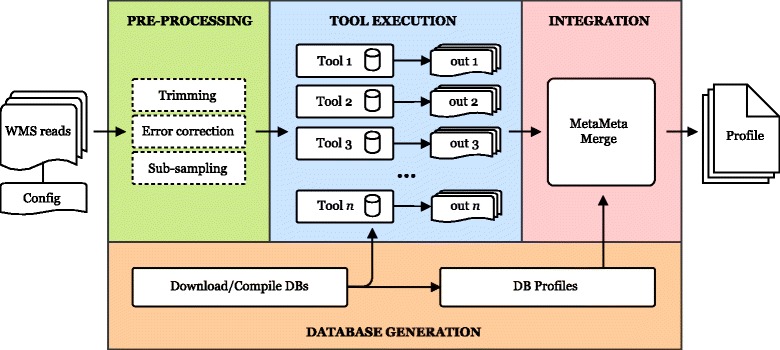
MetaMeta Pipeline. The MetaMeta Pipeline: one or more WMS read samples and a configuration file are the input. The pipeline consists of four main modules: *Database Generation* (only on the first run), *Pre-processing* (optional), *Tool Execution* and *Integration*. The output is a unified taxonomic profile integrating the results from all configured tools for each sample, generated by the MetaMetaMerge module

The pipeline accepts one or multiple WMS samples as well as one or more databases and the output is an integrated taxonomic profile for each sample per database (as well as a separated output from each executed tool). The MetaMeta pipeline can be described in four modules: database generation, pre-processing, tool execution, and integration (Fig. 1).

### Database generation

On the first run, the pipeline downloads and builds the databases for each of the configured tools. Pre-configured databases (Additional file 1: Table S1) are provided as well as a custom database creation option based on reference sequences. Since each tool has its own database with a specific version of reference sequences, database profiles are generated, collecting which taxonomic groups each tool can identify. Given a list of accession version identifiers for each sequence on the reference set, MetaMeta automatically generates a taxonomic profile for each tool’s database.

### Pre-processing

An optional pre-processing step is provided to remove errors and improve sequence classification: Trimommatic [13] for read trimming and BayesHammer [14] for error correction. A sub-sampling step is also included, allowing the sub division of large read sets among several tools by equally dividing them or by taking smaller random samples with or without replacement, to reduce overall run-time.

### Tool execution

In this step, the pre-processed reads are analyzed by the configured tools. Tools can be added to the pipeline if they follow a minimum set of requirements. They should output their results based on the NCBI Taxonomy database [15] (by name or taxonomic id). Profiling tools should output a rank separated taxonomic profile with relative abundances while binning tools should provide an output with sequence id, length used in the assignment and taxon. The BioBoxes [16] data format for binning and profiling (https://github.com/bioboxes/rfc/tree/master/data-format) is directly accepted. Tools which provide non-standard output should be configured with an additional step converting their output to be correctly integrated into the pipeline (More details are given in the Additional file 1: File Formats).

### Integration

The integration step will merge identified taxonomic groups and abundances and provide a unified profile for each sample. MetaMeta aims to improve the final results based on the assumption that the more identifications of the same taxon by different tools are reported, the higher its chance to be correct. This task is performed by the MetaMetaMerge module. This module accepts binning and profiling results and relies on previously generated database profiles. Taxonomic classification can change over time and each tool can use a different version/definition of it. For that reason, a recent taxonomy database version is used to solve name and rank conflicts (e.g., changing name specification, species turning into sub-species, etc.).

#### Abundance estimation - binning tools

Binning tools provide a single classification for each sequence in the dataset instead of relative abundances for taxons. An abundance estimation step is necessary for a correct interpretation of such data and posterior integration. The lengths of the binned sequences are summed up for each identified taxonomic group and normalized by the length of their respective reference sequences, estimating the *abundance* for each identified taxon *n* as:

1 $$ {abundance}_{n} = \sum_{i=1}^{r} \frac{\sum_{j=1}^{t_{i}}b_{j}}{l_{i}}  $$

where *r* is the number of reference sequences belonging to the taxonomic group *n*, *t*_*i*_ is the total of reads classified to the reference *i*, *b*_*j*_ is the number of aligned bases of a read *j* and *l*_*i*_ is the length of the reference *i*. The abundance of the parent nodes is based on the cumulative sum of their children nodes’ abundance.

#### Merging approach

The first step on the merging approach is to normalize estimated abundances to 100% for each taxonomic level. That is necessary because some tools do account for the unclassified reads and others do not. MetaMetaMerge only considers classified reads. Once normalized, all profiles are then integrated to a single profile. In this step, MetaMetaMerge saves the number of occurrences of each taxon among all profiles. This occurrence count is used to better select taxons that are more often identified, assuming that they have higher chances of being a correct identification. MetaMetaMerge also calculates an integrated value for the relative abundance estimation, defined as the harmonic mean of all normalized abundances for each taxon, avoiding outliers and obtaining a general trend among the estimated abundances. All steps taken in the merging process are performed for each taxonomic level independently, from super kingdom to species by default.

Since tools use different databases of reference sequences it is necessary to account for this bias. Previously generated database profiles provide which taxons are available for each tool. By merging all database profiles, it is possible to anticipate how many times each taxon could be identified among all tools used. The number of occurrences of each taxon from the tools’ output and the database presence number are integrated to generate a score *S* for each taxon, defined as:

2 $$ S_{ij} = \frac{(i+1)^{2}}{j+1}  $$

where *i* is the number of times the taxon was identified and *j* the number of times it is contained in the databases. This score calculation accounts for the presence/absence of taxonomic groups on different databases. It gives higher scores to the most identified taxons present in more databases. At the same time, lower scores are assigned to taxons present in many databases but not identified too many times. The score calculation is purposely biased for higher scores when *i*=*j* (Additional file 1: Figure S1), given the benefit of the doubt for taxons with low identification that are available only in few databases.

Commonly, metagenome analysis methods have to deal with a moderate to high number of false positive identifications at lower taxonomic levels. That occurs mainly because metagenomes can contain very low abundant organisms with similar genome sequences. This problem is even extended in our merged profile by collecting all false positives from different methods, generating a long tail of false positives with lower scores mixed together with true identifications. A filtering step is therefore necessary to avoid wrong assignments. This step is usually performed by an abundance cutoff value. Setting up this value is subject to uncertainty since the real abundances are usually not known and the separation between low abundant organisms and false identifications is not clear [17]. A simple cutoff would not provide a good separation between true and false results in this scenario.

To overcome this problem, MetaMetaMerge classifies each taxon in a set of bins (four by default) based on the calculated score (Eq. 2). Bins are defined by equally dividing the range of scores in the numbers of bins selected. Now each taxon has a score and a bin assigned to it. Taxons with higher scores are more likely to be true identifications and are going to be grouped together in the same bin. With this strategy it is possible to obtain a general separation among taxons which are prone to be true or false identifications.

Within each taxon grouped in a bin (sorted by relative abundance) a cutoff is applied to remove possible false identifications with low abundance. Here, the cutoff value is a percentile relative to the number of taxons on each bin and it is selected based on predefined functions, which can achieve more sensitive or precise results (Additional file 1: Mode functions). Each bin will have a different cutoff value depending on the chosen function.

If precision is chosen, a gradually more stringent cutoff will be used, selecting only the most abundant taxa for each bin. If sensitivity is selected, cutoffs will be set higher, allowing more identifications to be kept. Sensitive results have an increased chance of containing more true positives but at the same time they will likely have more false identifications due to less strict cutoffs.

Based on this percentile cutoff, MetaMetaMerge keeps only the top abundant taxa on each bin and removes taxons below it. After this step, the remaining taxons on each bin are re-grouped and sorted by relative abundance to generate the final profile.

At the end, MetaMeta will provide a final taxonomic profile, integrating all tools results, a detailed profile with co-occurrence and individual abundances, an interactive Krona pie chart [18] to easily compare taxonomic abundances among the tools as well as single profiles for each executed tool.

## Results

### Tool selection

MetaMeta was evaluated with a set of six tools: CLARK [19], DUDes [20], GOTTCHA [21], Kraken [22], Kaiju [23], and mOTUs [24]. The choice was partially motivated by recent publications comparing the performance of such tools [3, 4, 25]. CLARK, GOTTCHA, Kraken, and mOTUs achieved very low false positive numbers according to [4]. DUDes was an in-house developed tool which achieves good trade-off between precision and sensitivity according to [25]. Kaiju uses a translated database, bringing diversity to the current whole genome-based methods. We also considered the amount of data/run time performance for each tool, selecting only the ones that can handle large amounts of data as commonly used today in metagenomics analysis in an acceptable time (less than 1 day for our largest CAMI dataset −7.4 Gbp). MetaPhlAn [26] a widely used metagenomics tool could not be included due to taxonomic incompatibility. Any other sequence classification tool could be configured and used in MetaMeta, as long as it fits with our pipeline requirements described in the Methods - Tool execution section. We selected an equal number of tools for each category: DUDes, GOTTCHA, and mOTUs are taxonomic profiling tools, while CLARK, Kraken, and Kaiju are binning tools. Databases were created following the default guidelines for each tool, considering only bacteria and archaea as targets (Additional file 1: Table S1).

### Datasets and evaluation

The pipeline was evaluated with a set of simulated and real samples (Table 1). The simulated data were provided as part of the CAMI Challenge (toy samples) and the real samples were obtained from the Human Microbiome Project (HMP) [27, 28]. MetaMeta was compared to each single result from each tool configured in the pipeline. Although the pipeline can work on the strain level, we evaluate the results until species levels since most of the tools still do not provide strain level identifications. We compare the results to the ground truth in a binary (true and false positives, sensitivity, and precision) and quantitative way with the *L*_1_ norm, which is the sum of absolute differences between predicted and real abundances, when abundance profiles are available. Computer specifications and parameters can be found on the Additional file 1.

**Table 1:** Samples used in this study and run-time (based on the computer specifications on Additional file 1)

Sets	# Samples	Total bases	# Species	Cpu time/sample	Estimated wall time/sample
CAMI toy low	1	14.8 Gbp	30	31:04:52	02:35:24
CAMI toy medium	4	31.3 Gbp	199	15:18:16	01:16:31
CAMI toy high	5	74.5 Gbp	375	33:20:30	02:46:42
HMP stool	147	1.44 Tbp	299*	19:39:39	01:38:18

cpu time/sample stands for the mean cpu time for each sample without paralellization. Estimated wall time/sample considers a double speed-up by using 12 threads and concurrently running all six tools (when computational resources are available the pipeline can run all tools/samples/databases at the same time). *expected number of species from isolated genomes from the gastrointestinal tract

#### CAMI data

The CAMI challenge provided three toy datasets of different complexity (Table 1) with known composition and abundances. From low to high complexity, they provide an increasing number of organisms and samples. The samples within a complexity group contain the same organisms with variable abundances among samples. The sets contain real and simulated strains from complete and draft bacterial and archaeal genome sequences. The simulated CAMI datasets, especially those of medium and high complexity, provide a very challenging and realistic data in terms of complexity and size.

**Fig. 2:**
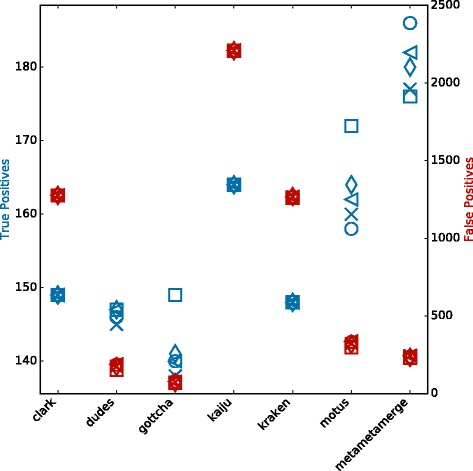
True and False Positives - CAMI high complexity set. In *blue* (left *y* axis): True Positives. In *red* (right *y* axis): False Positives. Results at species level. Each *marker* represents one out of five samples from the CAMI high complexity set

**Fig. 3:**
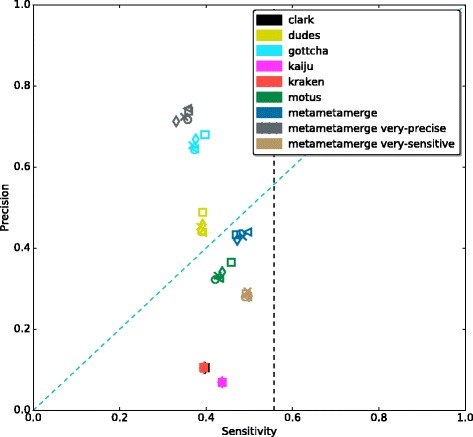
Precision and Sensitivity - CAMI high complexity set. *Dotted black line*marks the maximum possible sensitivity value (0.57) that could be achieved with the given tools and databases. Results at species level. Each *marker* represents one out of five samples from the CAMI high complexity set

In Fig. 2, it is possible to observe the tools performance in terms of true and false positives for the CAMI high complexity set. All configured tools perform similarly in the true positive identifications but vary among the false positives. Binning tools have a higher number of false positive identifications due to the fact that even single classified reads are considered. The MetaMetaMerge profile surpassed all other methods in true positive identifications while keeping the false positive number low. The same trend occurs in the other complexity sets (Additional file 1: Figures S3–S8). Figure 3 shows the trade-off between precision and sensitivity for all high complexity samples. MetaMetaMerge achieved the best sensitivity while GOTTCHA the best precision among the compared tools with default parameters. Those results show how the merging module of the MetaMeta pipeline is capable of better selecting and identifying true positives based on the co-occurrence information. MetaMetaMerge also has the flexibility to provide more precise or sensitive results (Fig. 3) just by changing the *mode* parameter (details are given in the Additional file 1: Mode functions). In the very precise mode, the merged profile outperformed all tools in terms of precision, but with the cost of losing sensitivity. In the very sensitive mode, the merged profile could improve the sensitivity compared to the run with default parameters, with some loss of precision. It is important to notice that the trade-off between precision and sensitivity could also be explored by the *cutoff* parameter (default 0.0001), depending on what is expected to be the lowest abundant organism in the sample. The MetaMetaMerge *mode* parameter will give more precise or sensitive results based on this cutoff value.

**Fig. 4:**
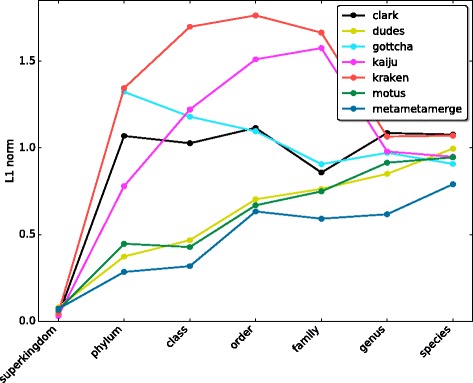
*L*_1_ norm error. Mean of the *L*_1_ norm measure at each taxonomic level for five samples from the high complexity CAMI set

In terms of relative abundance, MetaMetaMerge provides the most reliable predictions with smaller difference from the real abundances, as shown in Fig. 4 with regard to the *L*_1_ norm measure. By taking the harmonic mean, we succeed in reducing the effect of outliers that occur among the tools and capture the trend of the estimated relative abundances, providing a new, more robust estimate (Additional file 2).

#### Pre-processing and sub-sampling effects

**Fig. 5:**
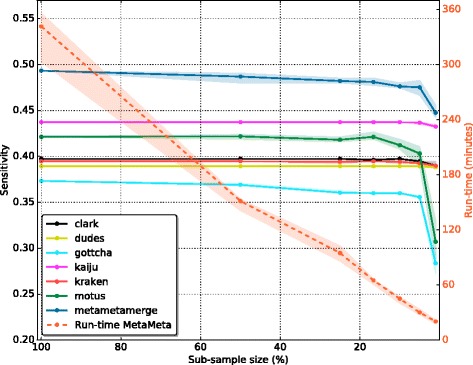
Sub-sampling. Sensitivity (left *y* axis) and run-time (right *y* axis) at species level for one randomly selected CAMI high complexity sample. Each sub-sample was executed five times. *Lines*represent the mean and the area around it the maximum and minimum achieved values. Run-time stands for the time to execute the MetaMeta pipeline. The evaluated sample sizes are 100, 50, 25, 16.6, 10, 5, and 1%. 16.6% is the exact division among six tools, using the the whole sample. Sub-samples above that value were taken with replacement and below without replacement. The plot is limited to a value of 0.57 (left *y* axis) that is the maximum possible sensitivity value that could be achieved with the given tools and databases

We explore here the effects of pre-processing and sub-sampling on the CAMI toy sets. Results shown in this section were trimmed and sub-sampled in several sizes, with and without replacement and executed five times for each sub-sample. Trimming effects were small on this set, slightly increasing precision (data not shown). Figure 5 shows the effects of sub-sampling in terms of sensitivity and run-time (wall time for the full pipeline) for one of the high complexity CAMI sets. Sub-sampling provides a high decrease on run-time for every tool and consequently for the whole pipeline. However, only below 5% it is possible to see a significant but still small decrease on sensitivity. All tools behave similarly on the sub-sampled sets, with GOTTCHA and mOTUs having a high decrease of sensitivity when using only 1% of the data. With the same sub-sample configuration (1%), MetaMetaMerge achieved a sensitivity higher than any other tool alone using 100% of the set. It also runs the whole pipeline approximately 17 times faster than with the full set (from 05 h 41 min 36 s to 20 min 19 s on average), being faster than the fastest tool with 100% of the data (kraken 29 min 26 s on average) and the second best sensitive tool (kaiju 1 h 47 min 44 s on average). As expected, precision is slightly increased in small sub-samples due to less data (Additional file 1: Figure S9).

#### Human Microbiome Project data

The HMP provided several resources to characterize the microbial communities at different sites of the human body. MetaMeta was tested on stool samples to evaluate the performance of the pipeline on real data. For evaluation we used a list of reference genome sequences that were isolated from specific body sites and sequenced as part of the HMP. They do not represent the complete content of microbial diversity in each community but serve as a guide to check how well the tools are performing. Stool samples were compared against the isolated genomes obtained from the gastrointestinal tract.

**Fig. 6:**
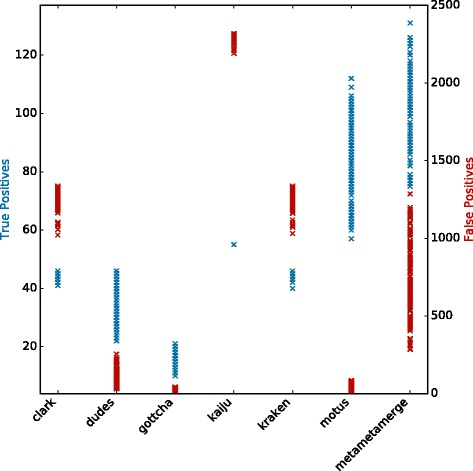
True and False Positives - HMP stool samples. In *blue* (left *y* axis): True Positives. In *red* (right *y* axis): False Positives. Results at species level. Each marker represents one out of 147 stool samples from the HMP

Figure 6 shows the results for 147 samples. In sensitive mode, MetaMetaMerge achieved the highest number of true positive identifications with a moderate number of false positives, below all binning tools but above all taxonomic profilers. mOTUs produced good results in the selected samples mainly because its database is based on the isolated genomes from the HMP (the same as the ground truth used here). Since mOTUs is the only tool with a distinct set of reference sequences that could classify this set, the scores (from Eq. 2) attributed to mOTUs’ unique identifications were low. Still, MetaMetaMerge could improve the true identifications keeping a lower rate of false positives by incorporating the true identifications from other methods.

## Discussion

MetaMeta is a complete pipeline for classification of metagenomic datasets. It provides improved profiles over the tested tools by merging their results. In addition, the pipeline provides easy configuration, execution and parallelization. With simulated and real data, MetaMeta is capable to achieve higher sensitivity. That is possible due to the MetaMetaMerge module, which extracts information of co-occurrence of taxons on databases and profiles, collecting complementary results from different methods. Further, the guided cutoff approach avoids false positives and keeps most of the true identifications, enhancing final sensitivity and exploring the complementarity of currently available methods.

By running several tools, MetaMeta has an apparently prohibitive execution time. In reality, the parallelization provided by Snakemake makes the pipeline run in a reasonable time using most of the computational resources (Table 1). That is possible by the way the rules are chained and executed among several cores, lasting not more than the slowest tool plus pre- and post-processing time, which are very small in comparison to the analysis time. In addition, sub-sampling allows the reduction of input data and a high decrease of execution time with small if any impact on the final result. That is viable due to redundant data contained in many metagenomic samples as well as redundant execution by several tools provided in the MetaMeta environment. However sub-sampling should be used with caution, taking in consideration the coverage of low abundant organisms.

All tools presented here are available at the BioConda channel and are automatically installed in the first MetaMeta run, working out-of-the-box for several computer environments and avoiding conflicts and broken dependencies. MetaMeta can also handle multiple large samples and databases at the same time, with options to delete intermediate files and keep only necessary ones, being well suited to large scale projects. It also reduces idle computational time by smartly parallelizing samples among one or more tools (Additional file 1: Figures S10–S13). The parallelization noticeably decreases the run time when computational power is available and manages to serialize and control the run when access to computational power is limited. Integration into HPC systems is also possible and we provide a pre-configured file for queuing systems (e.g., slurm). As stated by Lee et al. [29], solid-state drives accelerate the run time of many bioinformatics tools. Such drives were used in some evaluations shown in this paper and are beneficial for the MetaMeta pipeline.

MetaMeta makes it easier for the user to obtain more precise or sensitive results by providing a single default parameter as well as advanced options for more refined results. This parameter when set towards sensitivity tends to output an extensive list of taxons, being at the same time less stringent with the minimum abundance cutoff. When set towards precision it will apply a more strict abundance cutoff and provide a smaller but more accurate list of predicted taxons. Since all tools were used in default mode, it is possible to obtain problem-centric optimized results only by changing the way MetaMeta works. That facilitates and simplifies the task for researchers that are in search for a specific goal.

MetaMeta supports strain level identification. Nevertheless all evaluations were made at species level due to lack of support to strain identification in some tools. Also the lack of standard was a limiting factor. Taxonomic IDs are no longer assigned to strain levels [30] and tools output them in different ways. With standard output definitions, the use of strain classification on the pipeline is straight forward.

Related in parts, a method called WEVOTE was developed in parallel and recently published [31] where five classification tools were used to generate a merged taxonomic profile. Although the two methods present distinct ways of achieving better taxonomic profiling, they are not built for the same use case. WEVOTE relies on BLAST based tools and thereby is not suited for the large scale WMS applications, since the dataset sizes practically prohibit analyses via BLAST based approaches. Differently, MetaMeta was built accounting for high throughput data. Moreover, we supply an easy way to install tools and MetaMeta provides a complete pipeline which can configure databases and run classification tools with an integration module at the end, where WEVOTE provides only the integration method. As a result a comparison among the pipelines is hard to perform and interpret since they both use a different set of tools and databases.

## Conclusion

In conclusion, MetaMeta is an easy way to execute and generate improved taxonomic profiles for WMS samples with multiple tool support. We believe the method can be very useful for researchers that are dealing with multiple metagenomic samples and want to standardize their analysis. The MetaMeta pipeline was built in a way to facilitate the execution in many computational environments using Snakemake and BioConda. That diminishes the burden of installing and configuring multiple tools. The pipeline also gives control over the storage of the results and has an easy set of parameters which makes it possible to obtain more precise or sensitive results. MetaMeta was coded in a standardized manner, allowing easy expansion to more tools, also collectively in the MetaMeta git repository (https://github.com/pirovc/metameta). We believe that the final profile could be even further improved with novel tools configured into the pipeline.

## Availability and requirements

**Project name:** MetaMeta**Project home page:** https://github.com/pirovc/metameta**Operating systems:** Linux**Programming language:** Python**Other requirements:** Snakemake**Licence:** MIT

## Additional files

Additional file 1 Additional file with supplementary figures and information. (PDF 1024 kb)

Additional file 2 Additional File with interactive charts for all CAMI toy set results on default, very-precise and very-sensitive mode. File prefix S, M, and H for low, medium and high complexity, respectively. (TAR 3573 kb)

## Abbreviations

CAMI: Critical assessment of metagenome interpretation
HMP: Human Microbiome Project
HPC: High performance computing
WMS: Whole metagenome shotgun
