# Supplementary material for: MetaMeta: integrating metagenome analysis tools to improve taxonomic profiling
Source: Microbiome. 2017 Aug 14;5:101. doi: 10.1186/s40168-017-0318-y (PMC5557516; doi:10.1186/s40168-017-0318-y)
Supplement: Supplementary file 1 — Additional file with supplementary figures and information. (PDF 1024 kb) [file 40168_2017_318_MOESM1_ESM.pdf]

Additional File 1
MetaMeta: Integrating metagenome analysis tools to improve taxonomic profiling

Vitor C. Piro, Marcel Matschkowski, Bernhard Y. Renard

1 Implementation

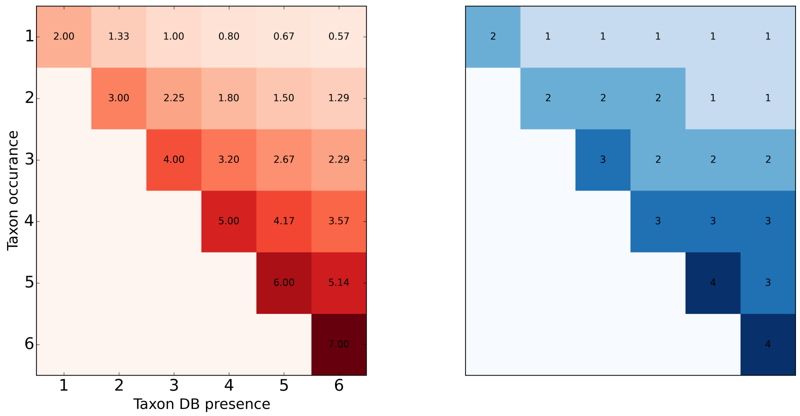

Figure 1: Score and bin matrices: Left: Matrix with an example of calculated scores for 6 tools. Right: matrix showing the division of the scores into 4 bins

1.1 File formats

MetaMeta accepts BioBoxes format directly (https://github.com/bioboxes/rfc/tree/master/data-format) or a .tsv file in the following format:
Profiling: rank, taxon name or taxid, abundance
Example:

genus	Methanospirillum	0.0029
genus	Thermus	0.0029
genus	568394	0.0029
species	Arthrobacter sp. FB24	0.0835
species	195	0.0582
species	Mycoplasma gallisepticum	0.0536

Binning: readid, taxon name or taxid, lenght of sequence assigned
Example:

M2—S1—R140	354	201
M2—S1—R142	195	201
M2—S1—R145	457425	201
M2—S1—R146	562	201
M2—S1—R147	1245471	201
M2—S1—R150	354	201

1.2 Mode functions

The mode parameter can be selected among 5 different functions, that would generate more precise or sensitive results (Figure 2). Each *bin* will have a cut-off value C defined as:

Very-sensitive: $C_{bin} = log(bin + 3)/log(maxbins + 3)$
Sensitive: $C_{bin} = log(bin + 1)/log(maxbins + 1)$
Linear: $C_{bin} = bin/maxbins$
Precise: $C_{bin} = 2^{bin}/2^{maxbins}$
Very-precise: $C_{bin} = 4^{bin}/4^{maxbins}$
where *maxbins* is the total number of bins.

2 Results

2.1 Databases

Table 1: MetaMeta pre-configured databases

Tool	Archaea + Bacteria (v1)	Custom
CLARK	Yes (https://doi.org/10.5281/zenodo.819305)	Yes
DUDes	Yes (https://doi.org/10.5281/zenodo.819343)	Yes
GOTTCHA	Yes (https://doi.org/10.5281/zenodo.819341)	No
kaiju	Yes (https://doi.org/10.5281/zenodo.819425)	Yes
kraken	Yes (https://doi.org/10.5281/zenodo.819363)	Yes
mOTUs	Yes (https://doi.org/10.5281/zenodo.819365)	No

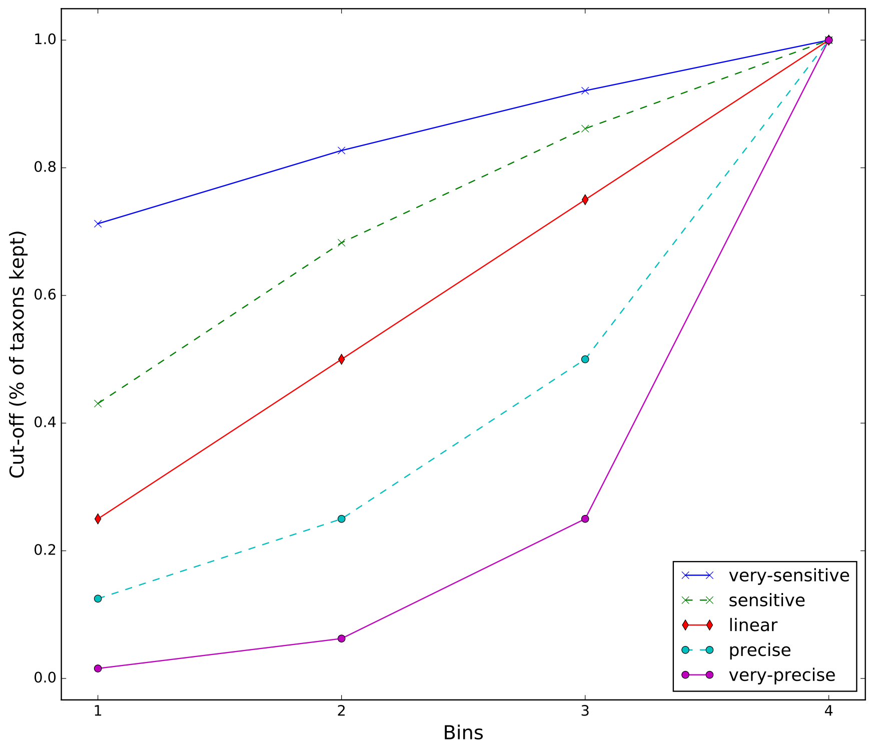

Figure 2: Example of cut-off values for 4 bins in each mode

2.2 Computer specifications

The main evaluations were performed with MetaMeta v1.1 on a x86 cluster consisting of of a total of 1000 cores and roughly 3.5 TB RAM. The subsampling evaluations on CAMI data were performed with MetaMeta v1.0 on: 60 CPUs x Intel(R) Xeon(R) CPU E7-4890 v2 @ 2.80GHz, 1056 GB RAM, Debian GNU/Linux 8.4, 2.8 TB SSD.

2.3 Datasets and Parameters

MetaMeta pipeline was executed with all 6 pre-configured tools using the archaea and bacteria database (Table 1).
All CAMI toy sets (low, medium and high complexity) were obtained from https://data.cami-challenge.org/
148 stool samples from HMP were obtained at: http://hmpdacc.org/

List of analyzed samples: SRS011061, SRS011134, SRS011239, SRS011271, SRS011302, SRS011405, SRS011452, SRS011529, SRS011586, SRS012273, SRS012902, SRS013158, SRS013215, SRS013476, SRS013521, SRS013687, SRS013800, SRS013951, SRS014235, SRS014287, SRS014313, SRS014459, SRS014613, SRS014683, SRS014923, SRS014979, SRS015065, SRS015133, SRS015190, SRS015217, SRS015264, SRS015369, SRS015578, SRS015663, SRS015782, SRS015794, SRS015854, SRS015960, SRS016018, SRS016056, SRS016095, SRS016203, SRS016267, SRS016335, SRS016495, SRS016517, SRS016585, SRS016753, SRS016954, SRS016989, SRS017103, SRS017191, SRS017247, SRS017307, SRS017433, SRS017521, SRS017701, SRS017821, SRS018133, SRS018313, SRS018351, SRS018427, SRS018575, SRS018656, SRS018817, SRS019030, SRS019161, SRS019267, SRS019397, SRS019582, SRS019601, SRS019685, SRS019787, SRS019910, SRS019968, SRS020233, SRS020328, SRS020869, SRS021484, SRS021948, SRS022071, SRS022137, SRS022524, SRS022609, SRS022713, SRS023346, SRS023526, SRS023583, SRS023829, SRS023914, SRS023971, SRS024009, SRS024075, SRS024132, SRS024265, SRS024331, SRS024388, SRS024435, SRS024549, SRS024625, SRS042284, SRS042628, SRS043001, SRS043411, SRS043701, SRS045004, SRS045645, SRS045713, SRS047014, SRS047044, SRS048164, SRS048870, SRS049164, SRS049712, SRS049900, SRS049959, SRS049995, SRS050299, SRS050422, SRS050752, SRS050925, SRS051031, SRS051882, SRS052027, SRS052697, SRS053214, SRS053335, SRS053398, SRS054590, SRS054956, SRS055982, SRS056259, SRS056519, SRS057478, SRS057717, SRS058723, SRS058770, SRS062427, SRS063040, SRS063985, SRS064276, SRS064557, SRS064645, SRS065504, SRS075398, SRS077730, SRS078176
The sample SRS023176 couldn't be properly analyzed due to inconsistent read pairs.

Table 2: MetaMeta (v1.1) parameters used for the CAMI and HMP data. Default parameters were used when not stated below.

	Default	CAMI low/med./high	HMP
trimming	0	-	-
desiredminlen	70	-	-
subsample	0	-	-
mode	linear	-	sensitive
cutoff	0.0001	-	0.00001
bins	4	-	-
ranks	species	-	-

2.4 Results

Table 3: MetaMeta (v1.0) parameters used for the sub-sampled CAMI data. Default parameters were used when not stated below. N/A: not applicable

	Default	CAMI 1%	CAMI 5%	CAMI 10%	CAMI 16.6%	CAMI 25%	CAMI 50%	CAMI 100%
trimming	0	1	1	1	1	1	1	1
desiredminlen	70	-	-	-	-	-	-	-
strictness	0.8	-	-	-	-	-	-	-
errorcorr	0	-	-	-	-	-	-	-
subsample	0	1	1	1	1	1	1	-
samplesize	1	0.01	0.05	0.1	-	0.25	0.5	N/A
replacement	0	-	-	-	-	1	1	N/A
mode	linear	precise	precise	precise	precise	precise	precise	precise
cutoff	0.0001	0.00001	0.00001	0.00001	0.00001	0.00001	0.00001	0.00001
bins	4	3	3	3	3	3	3	3
ranks	species	-	-	-	-	-	-	-

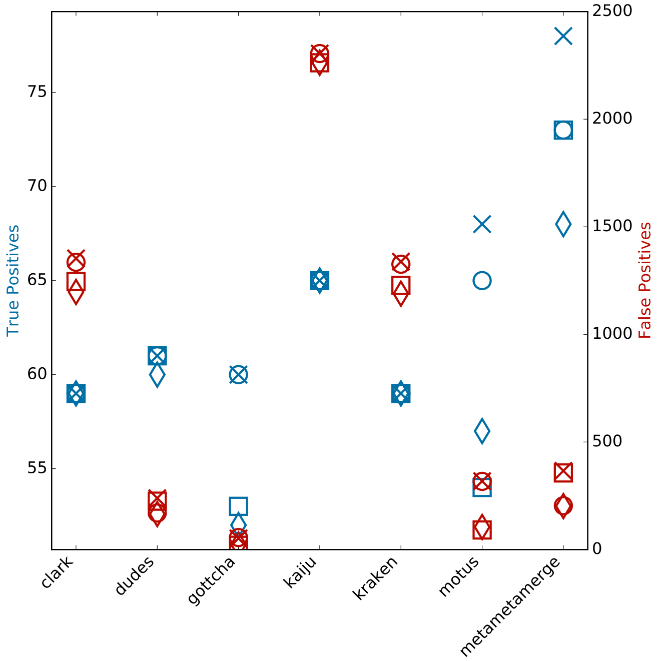

Figure 3: **True and False Positives - CAMI medium complexity set** In blue (left y axis): True Positives. In red (right y axis): False Positives. Results at species level. Each marker represents one out of four samples from the CAMI medium complexity set.

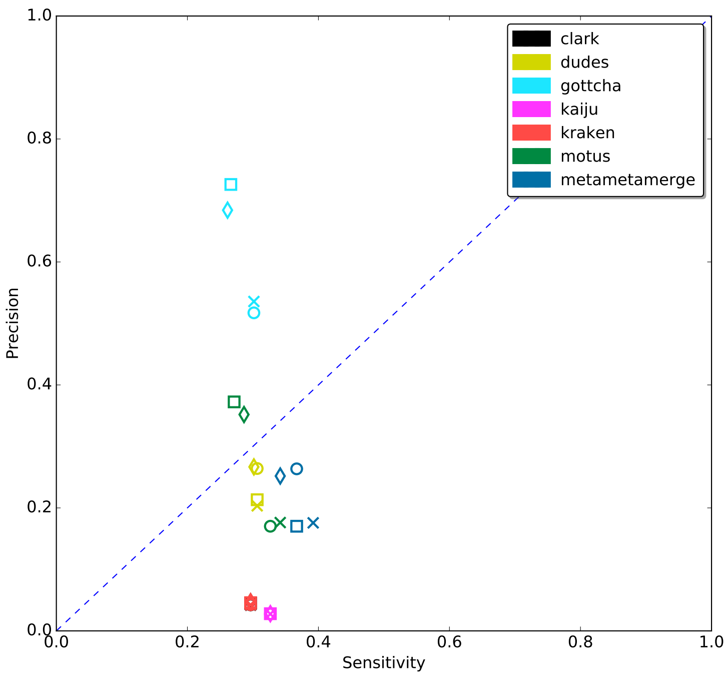

Figure 4: **Precision and Sensitivity - CAMI medium complexity set** Results at species level. Each marker represents one out of four samples from the CAMI medium complexity set.

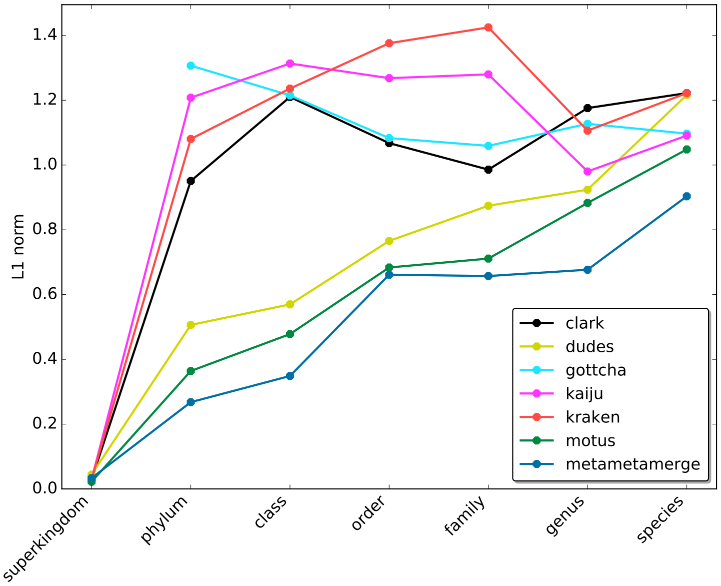

Figure 5: L_1 **norm error** Mean of the L_1 norm measure at each taxonomic level for four samples from the medium complexity CAMI set.

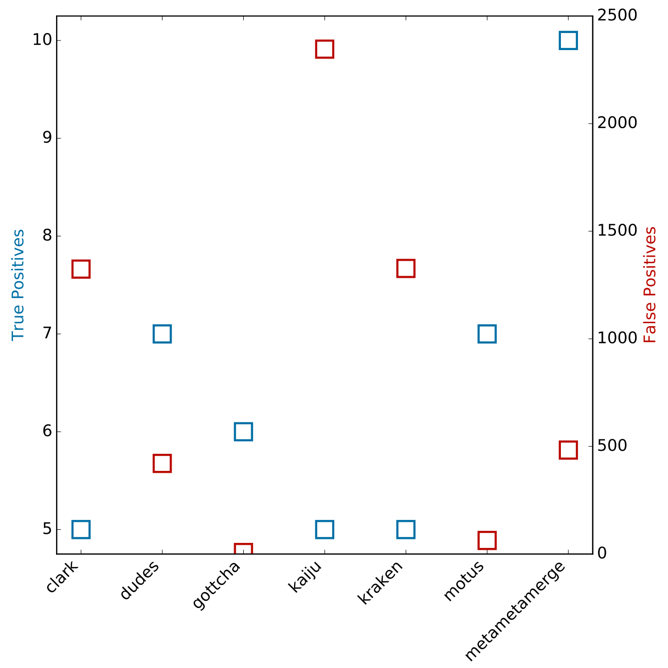

Figure 6: **True and False Positives - CAMI low complexity set** In blue (left y axis): True Positives. In red (right y axis): False Positives. Results at species level.

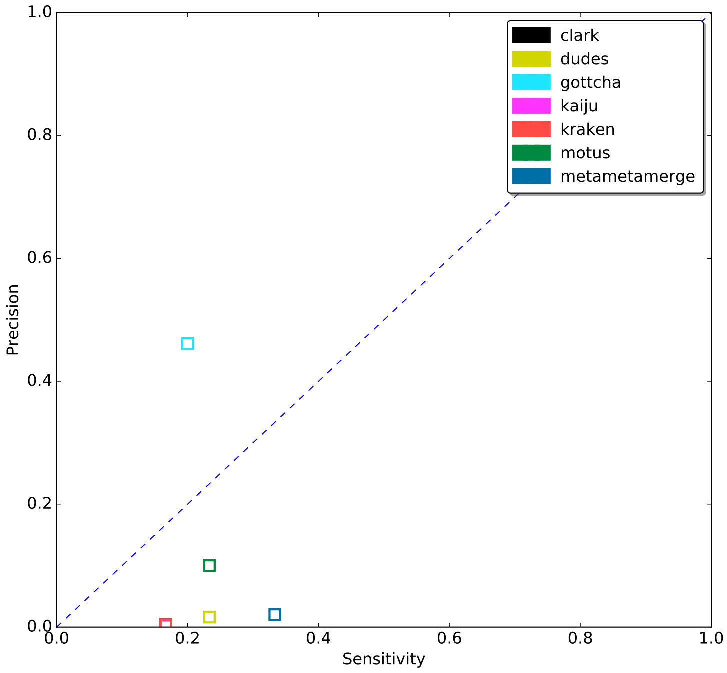

Figure 7: **Precision and Sensitivity - CAMI low complexity set** Results at species level.

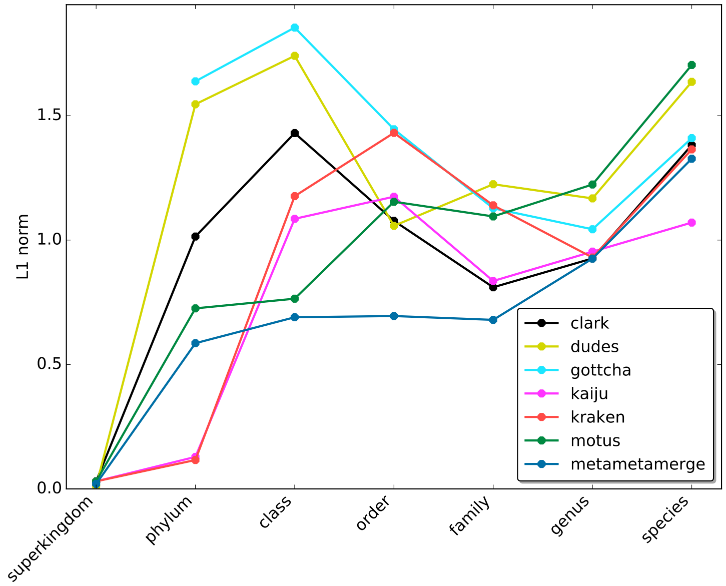

Figure 8: L_1 **norm error** L_1 norm measure at each taxonomic level for one sample from the low complexity CAMI set.

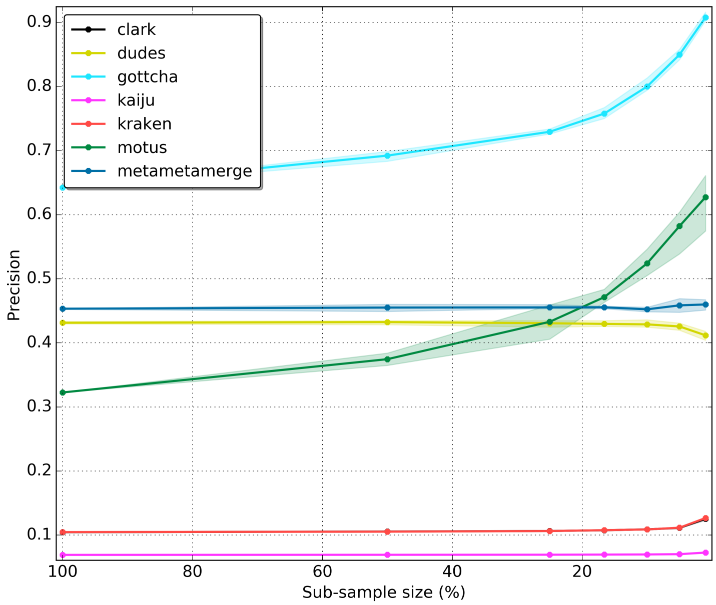

Figure 9: **Sub-sampling** Precision at species level for one randomly selected CAMI high complexity sample. Each sub-sample was executed five times. Lines represent the mean and the area around it the maximum and minimum achieved values. The evaluated sample sizes are: 100%, 50%, 25%, 16.6%, 10%, 5%, 1%. 16.6% is the exact division among 6 tools, using the the whole sample. Sub-samples above that value were taken with replacement and below without replacement.

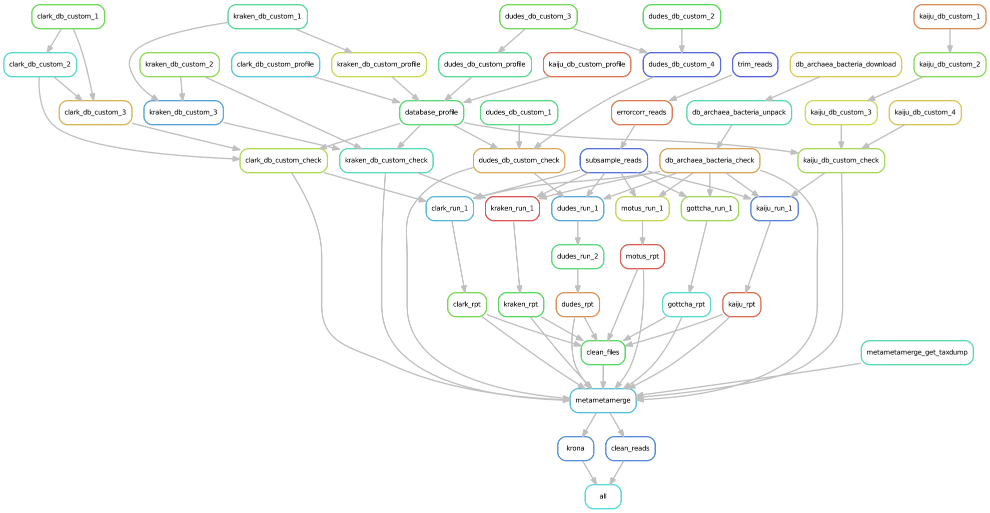

Figure 10: **Rulegraph** Overview of the rules and their dependencies on MetaMeta pipeline.

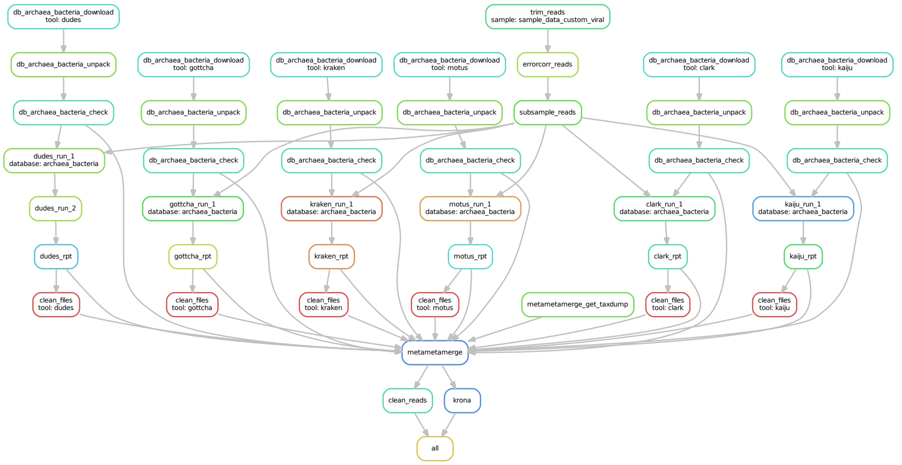

Figure 11: **DAG - pre-configured database** Directed acyclic graph of the MetaMeta pipeline for one sample, one database (pre-configured) and six tools.

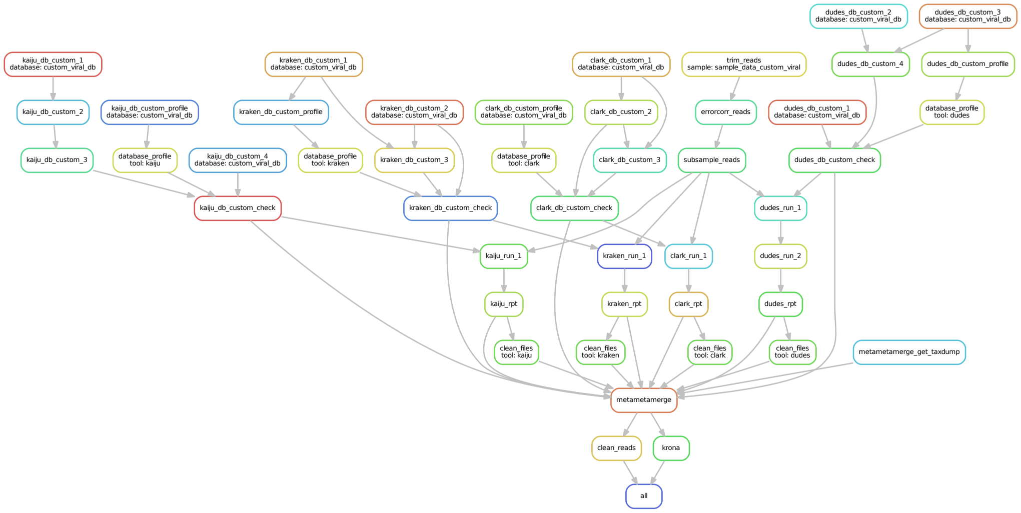

Figure 12: **DAG - custom database** Directed acyclic graph of the MetaMeta pipeline for one sample, one database (custom) and 4 tools.

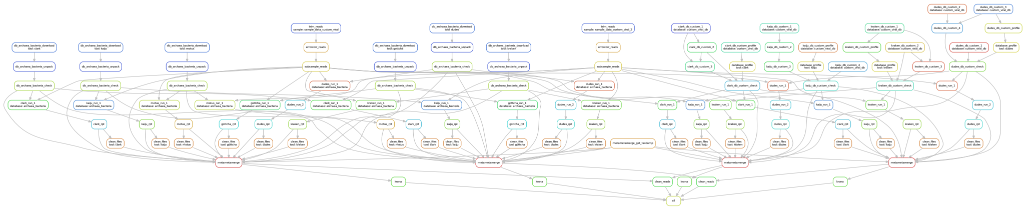

Figure 13: **DAG - multiple samples** Directed acyclic graph of the MetaMeta pipeline for two samples, two databases (pre-configured and custom) and six tools.
