# Supplementary material for: MetaMeta: integrating metagenome analysis tools to improve taxonomic profiling
Source: Microbiome. 2017 Aug 14;5:101. doi: 10.1186/s40168-017-0318-y (PMC5557516; doi:10.1186/s40168-017-0318-y)
Supplement: Supplementary file 2 — Additional File with interactive charts for all CAMI toy set results on default, very-precise and very-sensitive mode. File prefix S, M, and H for low, medium and high complexity, respectively. (TAR 3573 kb) [file 40168_2017_318_MOESM2_ESM.tar › H_S001__insert_180_default.html]

Javascript must be enabled to view this page.

magnitude
magnitudeUnassigned

clark.parsed\_profile
dudes.parsed\_profile
final.metametamerge.profile
gottcha.parsed\_profile
kaiju.parsed\_profile
kraken.parsed\_profile
motus.parsed\_profile

0.9999999999999971.0000061.0000011.0000170.9999990.9999959999999981.000003

0.0854340.058110.0550250.052560.0802790.0822080.050763

5e-060.0001234e-06

5e-060.0001234e-06

5e-063.3e-054e-06

5e-063.3e-054e-06

5e-063.3e-054e-06

9e-06

5e-062.4e-054e-06

9e-05

9e-05

3.7e-05

3.7e-05

5.3e-05

5.3e-05

0.0444870.0243570.0255910.0243740.0406570.0429240.021351

0.0039430.0023370.0024170.0025080.0038190.0037930.001542

1.4e-050.000241.4e-05

4e-065.7e-055e-06

4e-065.7e-055e-06

3e-062.8e-053e-06

1e-062.9e-052e-06

1e-050.0001839e-06

03e-050

03e-050

01.2e-050

01.2e-050

7e-061.6e-056e-06

7e-061.6e-056e-06

07.2e-050

06e-060

1.3e-05

3e-06

1.5e-05

02e-060

02e-060

1e-05

4e-06

1e-05

1e-06

1e-06

5e-06

2e-061.6e-052e-06

2e-061.6e-052e-06

1e-061.4e-051e-06

1e-061.4e-051e-06

2.3e-05

1.4e-05

9e-06

0.0038920.0023370.0024170.0025080.0034560.0037310.001542

0.0038920.0023370.0024170.0025080.0034560.0037310.001542

0.0038920.0023370.0024170.0025080.0034560.0037310.001542

1e-062.2e-051e-06

0.0038910.0023370.0024170.0025080.0034340.003730.001542

3.7e-050.0001234.8e-05

1.1e-054.6e-051.1e-05

1.1e-052.9e-051e-05

4e-061.1e-054e-06

4e-06

7e-061.4e-056e-06

01.7e-051e-06

01.7e-051e-06

1.8e-053.9e-052.8e-05

1.8e-052.6e-052.8e-05

1e-061.2e-051.2e-05

1.7e-051.4e-051.6e-05

01.3e-050

01.3e-050

8e-062.5e-059e-06

8e-062.5e-059e-06

8e-062.5e-059e-06

01.3e-05

01.3e-05

01.3e-05

1e-058.2e-051.1e-05

1e-058.2e-051.1e-05

7e-064.9e-058e-06

1e-067e-061e-06

1e-067e-061e-06

6e-064.2e-057e-06

1e-061.6e-051e-06

2e-061e-052e-06

01.1e-051e-06

3e-065e-063e-06

3e-063.3e-053e-06

1e-067e-061e-06

1e-067e-061e-06

2e-062.6e-052e-06

2e-067e-062e-06

2e-06

07e-060

2e-06

7e-06

01e-060

6e-068e-056e-06

6e-068e-056e-06

6e-068e-056e-06

2.2e-05

1.2e-05

1e-05

01e-05

01e-05

6e-064.8e-056e-06

3e-061.6e-053e-06

1e-061.6e-051e-06

1e-061e-051e-06

1e-066e-061e-06

0.0030490.0022670.0022220.0038110.0024040.0029280.001695

0.0030490.0022670.0022220.0038110.0024040.0029280.001695

0.0030490.0022670.0022220.0038110.0024040.0029280.001695

5e-06

5e-06

0.0022450.0012820.0014180.0020060.0016980.0021540.000909

7e-06

06e-060

3e-06

3e-060

4e-06

2e-065e-061e-06

4e-068e-064e-06

2e-061e-06

04e-060

5e-06

4e-06

2e-06

5e-06

0.0022390.0012820.0014180.0020060.0016230.0021480.000909

6e-06

8e-06

3e-06

0.0008040.0009850.0008040.0018050.0007010.0007740.000786

8e-06

03e-060

03e-060

4e-06

09e-060

0.0008030.0009850.0008040.0018050.0006630.0007730.000786

1e-061.1e-051e-06

6.3e-050.0002826.2e-05

5.9e-050.0001756e-05

2e-062.6e-053e-06

2e-062.6e-053e-06

2e-062.6e-053e-06

5.5e-050.0001125.5e-05

5.5e-050.0001125.5e-05

4.8e-057.1e-054.8e-05

7e-064.1e-057e-06

2e-061.6e-052e-06

2e-061.6e-052e-06

2e-061.6e-052e-06

02.1e-050

02.1e-050

02.1e-050

4e-067.2e-052e-06

4e-067.2e-052e-06

1e-063.2e-05

1e-063.2e-05

2.3e-05

2.3e-05

3e-061.7e-052e-06

3e-061.7e-052e-06

3.5e-05

3.5e-05

3.5e-05

3.5e-05

0.0015250.0007430.0008020.003210.0019450.0014830.000666

0.0002640.0001120.0015050.000430.0002590.0001

1.8e-050.0001652e-05

1.8e-050.0001652e-05

1.8e-050.0001652e-05

0.0002460.0001120.0015050.0002650.0002390.0001

7e-065.5e-058e-06

7e-065.5e-058e-06

1.2e-054.7e-051.1e-05

1.2e-054.7e-051.1e-05

0.0002270.0001120.0015050.0001630.000220.0001

2.6e-05

0.0002180.0001120.0015050.0001090.000210.0001

9e-062.8e-051e-05

0.0011870.0006310.0008020.0017050.0013010.0011490.000566

0.0011730.0006310.0008020.0017050.0011420.0011340.000566

5e-050.000155e-05

2.3e-057.2e-052.2e-05

2.7e-057.8e-052.8e-05

1e-051.7e-059e-06

4e-061e-063e-06

3e-06

6e-061.3e-056e-06

1e-054.5e-051.4e-05

1e-054.5e-051.4e-05

0.0011030.0006310.0008020.0017050.000930.0010610.000566

0.0010960.0006310.0008020.0017050.0009030.0010520.000566

7e-062.7e-059e-06

1.4e-050.0001361.5e-05

6e-064.8e-056e-06

6e-064.8e-056e-06

8e-068.8e-059e-06

8e-061.9e-059e-06

3.5e-05

3.4e-05

2.3e-05

2.3e-05

2.3e-05

7.4e-050.0002147.5e-05

7.4e-050.0002147.5e-05

1.4e-052.9e-051.6e-05

1.4e-052.9e-051.6e-05

7e-062.3e-058e-06

7e-062.3e-058e-06

1.3e-054.1e-051.1e-05

1.3e-054.1e-051.1e-05

1e-052.5e-051e-05

1e-052.5e-051e-05

2.3e-05

2.3e-05

1.8e-053.8e-051.8e-05

9e-062.1e-051e-05

9e-061.7e-058e-06

6e-061.8e-056e-06

6e-061.8e-056e-06

6e-061.7e-056e-06

6e-061.7e-056e-06

0.0352560.0186360.0195840.013240.0314150.0340310.017448

0.0352560.0186360.0195840.013240.0314150.0340310.017448

2e-061.4e-052e-06

2e-061.4e-052e-06

2e-061.4e-052e-06

0.0352540.0186360.0195840.013240.0314010.0340290.017448

1e-061.6e-050

1e-06

1.2e-05

1e-063e-060

0.0339870.0178360.0187950.0115350.0301970.0328150.017076

9.5e-05

7e-063e-057e-06

0.0339680.0178360.0187950.0115350.0298640.0327970.017076

1.2e-053.4e-051.1e-05

3.1e-05

0.000143

5e-064.7e-054e-06

3e-061.1e-053e-06

02.3e-05

3e-06

2e-061e-051e-06

0.0012610.00080.0007890.0017050.0011410.001210.000372

0.0012610.00080.0007890.0017050.0011410.001210.000372

0.0006350.0003740.0005660.0016050.000630.00061

0.0006350.0003740.0005660.0016050.000630.00061

0.0006350.0003740.0005660.0016050.000630.00061

0.0006350.0003740.0005660.0016050.000630.00061

0.0006340.0003740.0005660.0016050.0006090.000609

1e-062.1e-051e-06

0.0409240.0337530.0294340.0281860.0393950.0392670.029412

0.0409240.0337530.0294340.0281860.0393950.0392670.029412

3e-062.4e-051e-06

1e-061.2e-050

1e-061.2e-050

1e-061.2e-050

2e-061.2e-051e-06

2e-061.2e-051e-06

2e-061.2e-051e-06

6.7e-050.0015080.0034130.0050160.0006476.5e-050.001412

6e-062.1e-054e-06

6e-062.1e-054e-06

0

9e-06

1e-06

6e-061.1e-054e-06

6.1e-050.0015080.0034130.0050160.0006266.1e-050.001412

3.6e-050.0013890.0015055.6e-053.4e-05

3.6e-050.0013890.0015055.6e-053.4e-05

1e-061.8e-051e-06

1e-069e-061e-06

09e-060

1.3e-050.0001361.4e-05

1e-057.9e-051e-05

3e-065.7e-054e-06

1.1e-050.0015080.0020240.0035110.0004161.2e-050.001412

0.00040.0005250.0016050.000465

1.1e-050.0003840.0004161.2e-05

0.0011080.0011150.0019060.000947

0.0019940.0100160.0027780.0063190.0017170.0019160.009601

0.0019940.0100160.0027780.0063190.0017170.0019160.009601

01.1e-050

2e-06

09e-060

0.0019940.0100160.0027780.0063190.0016890.0019160.009601

1.5e-05

0.0019910.0100160.0027780.0063190.0016480.0019130.009567

2e-068e-062e-063.4e-05

1e-061.5e-051e-06

03e-060

01.7e-05

01.7e-05

0.038860.0222290.0232430.0168510.0370070.0372850.018399

2.7e-05

1.1e-05

1.1e-05

7e-06

7e-06

9e-06

9e-06

0.038860.0222290.0232430.0168510.036980.0372850.018399

0.0388580.0222290.0232430.0168510.0369040.0372820.018399

0.0388580.0222290.0232430.0168510.0369040.0372820.018399

7e-06

5e-06

2e-06

1e-061.9e-051e-06

1e-061.9e-051e-06

03e-051e-06

02e-050

01e-051e-06

1e-062e-051e-06

1e-062e-051e-06

1e-062.5e-050

1e-062.5e-050

1e-062.5e-050

1e-062.5e-050

1e-062.5e-050

1e-062.5e-050

1.7e-057.9e-051.3e-05

1.7e-057.9e-051.3e-05

1.7e-057.9e-051.3e-05

1.7e-057.9e-051.3e-05

1.7e-057.9e-051.3e-05

1.7e-057.9e-051.3e-05

0.9142079999999970.9418960000000010.9449760.9474569999999980.919720.9174769999999980.949240000000001

0.0002850.0004480.001605

0.0002850.0004480.001605

0.0002850.0004480.001605

0.0002850.0004480.001605

0.0002850.0004480.001605

0.0002850.0004480.001605

6.1e-050.00037.1e-05

6.1e-050.00037.1e-05

6.1e-050.00037.1e-05

4e-050.0002345.1e-05

1.9e-054.9e-052.2e-05

1.9e-054.9e-052.2e-05

4e-064.1e-059e-06

4e-064.1e-059e-06

4.8e-05

4.8e-05

1.7e-059.6e-052e-05

3.9e-05

1.7e-052.5e-052e-05

3.2e-05

2.1e-056.6e-052e-05

8e-064.8e-056e-06

8e-064.8e-056e-06

1.3e-051.8e-051.4e-05

1.3e-051.5e-051.4e-05

3e-06

3.1e-055.5e-052.4e-05

3.1e-055.5e-052.4e-05

3.1e-055.5e-052.4e-05

3.1e-055.5e-052.4e-05

3.1e-055.5e-052.4e-05

3.1e-055.5e-052.4e-05

6.4e-05

6.4e-05

6.4e-05

6.4e-05

6.4e-05

6.4e-05

1.6e-050.0001021.5e-05

1.6e-050.0001021.5e-05

1.6e-050.0001021.5e-05

1.6e-050.0001021.5e-05

1.6e-050.0001021.5e-05

1.6e-050.0001021.5e-05

0.4099770.4185510.4509690.3589840.4417969999999990.4270580.373223

0.0092390.0052260.0055560.006620.0100590.0088010.003792

2.3e-050.0001761.9e-05

6e-064e-056e-06

6e-064e-056e-06

6e-064e-056e-06

1.7e-050.0001361.3e-05

2e-063e-051e-06

2e-063e-051e-06

3e-063.1e-053e-06

3e-063.1e-053e-06

1.2e-057.5e-059e-06

1.2e-057.5e-059e-06

0.0001490.000470.0001313e-05

4e-05

4e-05

4e-05

4.8e-05

4.8e-05

4.8e-05

2.2e-054.1e-051.6e-05

2.2e-054.1e-051.6e-05

2.2e-054.1e-051.6e-05

1.3e-057.3e-051.1e-05

2.8e-05

2.8e-05

1.3e-054.5e-051.1e-05

1.3e-054.5e-051.1e-05

5e-059.7e-054.6e-05

1.7e-052.6e-051.6e-05

1.7e-052.6e-051.6e-05

3.3e-057.1e-053e-05

1.1e-051.9e-051.1e-05

1.4e-051.9e-051.1e-05

1.4e-05

8e-061.9e-058e-06

1.7e-058.1e-051.3e-05

1.7e-053.7e-051.3e-05

1.7e-053.7e-051.3e-05

4.4e-05

4.4e-05

4.7e-059e-054.5e-053e-05

4.7e-059e-054.5e-053e-05

1.1e-052.5e-051.1e-053e-05

2.9e-055.1e-052.7e-05

7e-061.4e-057e-06

0.0001560.0003740.000142

0.0001250.0002340.000115

0.0001250.0001930.000115

1.4e-053.8e-051.1e-05

1.9e-052.1e-051.7e-05

2.3e-05

7e-067e-063e-06

1.4e-052.6e-051.4e-05

7e-062.3e-058e-06

2.8e-056e-062.6e-05

2.1e-051e-051.9e-05

6e-06

1.5e-053.3e-051.7e-05

4.1e-05

4.1e-05

3.1e-050.000142.7e-05

3.1e-056.3e-052.7e-05

1.9e-052.9e-051.9e-05

1.2e-053.4e-058e-06

7.7e-05

3.4e-05

4.3e-05

3e-053.4e-052.6e-05

3e-053.4e-052.6e-05

3e-053.4e-052.6e-05

3e-053.4e-052.6e-05

2.1e-050.0001012e-05

2.1e-050.0001012e-05

4.3e-05

4.3e-05

2.1e-055.8e-052e-05

2.1e-055.8e-052e-05

0.0079610.0048160.0050950.0050150.0077220.0076320.003527

0.0028930.0017040.0019740.0022070.0028030.0027760.001452

7e-063.7e-055e-06

7e-063.7e-055e-06

1e-063.4e-050

1e-063.4e-050

0.0028780.0017040.0019740.0022070.0027120.0027630.001452

0.0028780.0017040.0019740.0022070.0027120.0027630.001452

7e-062e-058e-06

7e-062e-058e-06

0.0050680.0031120.0031210.0028080.0049190.0048560.002075

2.5e-054.9e-051.9e-05

2.5e-054.9e-051.9e-05

0.0050160.0031120.0031210.0028080.0047880.0048160.002075

0.0050160.0031120.0031210.0028080.0047880.0048160.002075

6e-063.8e-054e-06

6e-063.8e-054e-06

2.1e-054.4e-051.7e-05

2.1e-054.4e-051.7e-05

0.0008430.000410.0004610.0016050.0009680.000790.000235

0.000810.000410.0004610.0016050.0008620.000760.000235

6e-062.3e-056e-06

6e-062.3e-056e-06

0.0008040.000410.0004610.0016050.0008390.0007540.000235

0.0006710.000410.0004610.0016050.0006050.0006430.000235

9e-062.8e-057e-06

2.5e-053.1e-052.2e-05

2e-05

1.6e-051.3e-05

3e-052.9e-052.6e-05

4.6e-05

1e-061e-06

1e-055e-06

2.3e-053.2e-052.2e-05

5e-063.1e-055e-06

1.4e-051.7e-051e-05

2.6e-058.3e-052.2e-05

2.6e-058.3e-052.2e-05

5e-05

2.6e-053.3e-052.2e-05

7e-062.3e-058e-06

7e-062.3e-058e-06

7e-062.3e-058e-06

3.5e-053e-052.1e-05

3.5e-053e-052.1e-05

3.5e-053e-052.1e-05

3.5e-053e-052.1e-05

2.1e-050.0001842e-05

1.1e-050.0001211.2e-05

1.1e-050.0001211.2e-05

1.1e-050.0001211.2e-05

1e-056.3e-058e-06

1e-056.3e-058e-06

6e-062.2e-056e-06

4e-064.1e-052e-06

0.2024520.2093010.1311950.1055190.1487060.1946210.091142

0.0011859.8e-050.0015520.0034110.0026590.0011950.000127

0.0010639.8e-050.0015520.0019060.0023280.0010770.000127

0.0002520.0002860.0002461.6e-05

5.4e-05

2.6e-05

7.8e-054.7e-057.5e-05

2.7e-05

9.3e-051.4e-059.1e-05

2.2e-05

1.9e-05

8.1e-052.2e-058e-051.6e-05

5.5e-05

5e-05

5e-05

2e-054.8e-056.5e-05

2e-054.8e-056.5e-05

0.0002650.0003720.0002496.4e-05

1.6e-05

5.4e-05

1.6e-05

6.9e-05

7.4e-05

5.1e-05

0.0001345.5e-050.0001223.2e-05

0.0001316.9e-050.000127

0.0003919.8e-050.0015520.0019060.0013440.0003974.7e-05

3.3e-05

5.4e-05

0.0010570.001145

1.7e-05

3.5e-05

0.0003919.8e-050.0004950.0019066e-050.0003974.7e-05

4.3e-05

4.3e-05

3.4e-05

3.4e-05

0.0001350.0001510.00012

5.8e-051.7e-055.5e-05

7.7e-053.9e-056.5e-05

8e-05

1.5e-05

0.0001220.0015050.0003310.000118

0.0001220.0015056.4e-050.000118

2.3e-05

0.0001220.0015054.1e-050.000118

4e-05

4e-05

4.9e-05

2.1e-05

2.8e-05

0.000178

3.1e-05

1.3e-05

3.8e-05

2.7e-05

2.8e-05

4.1e-05

0.0001660.0003140.0001523.1e-05

0.0001660.0003140.0001523.1e-05

2.5e-059.8e-052.3e-05

2.5e-059.8e-052.3e-05

1.6e-050.0001151.3e-05

1.6e-050.0001151.3e-05

0.0001250.0001010.0001161.5e-05

0.0001250.0001010.0001161.5e-05

1.6e-05

1.6e-05

0.000254

0.000132

0.000132

0.000132

0.000122

0.000122

0.000122

2.3e-050.00011.8e-05

2.3e-050.00011.8e-05

2.3e-050.00011.8e-05

2.3e-050.00011.8e-05

0.0896050.0605940.0521730.0469410.0537520.0863040.032527

0.000204

9.1e-05

4.8e-05

4.3e-05

0.000113

0.000113

0.0004420.0003940.000419

0.0001540.0001750.00014

0.0001540.0001750.00014

0.0001289e-050.000125

0.0001289e-050.000125

0.000160.0001290.000154

0.000160.0001290.000154

6.3e-057.8e-056e-05

6.3e-057.8e-056e-05

6.3e-057.8e-056e-05

8.8e-050.0001159.6e-05

8.8e-050.0001159.6e-05

3.7e-052.8e-053.5e-05

3e-062e-061.2e-05

5e-066e-065e-06

6e-061.3e-058e-06

3e-066e-062e-06

8e-062.2e-054e-06

2.7e-05

1.1e-056e-061.1e-05

1.5e-055e-061.9e-05

0.0002280.0006230.000212

6.1e-057.7e-055.8e-05

6.1e-057.7e-055.8e-05

0.0001180.0001780.000107

4.9e-056.9e-054.1e-05

3e-055.7e-053.2e-05

3.9e-055.2e-053.4e-05

0.000134

6.9e-05

6.5e-05

3.4e-05

3.4e-05

0.000103

0.000103

2.9e-05

2.9e-05

4.9e-056.8e-054.7e-05

4.9e-056.8e-054.7e-05

0.0008050.0094360.0030790.010130.0002230.0007690.009795

6.3e-050.0001054e-051.7e-05

6.3e-053.3e-054e-05

1.7e-05

7.2e-05

0.0007420.0094360.0030790.010130.0001180.0007290.009778

2e-061e-052e-06

4e-060.0009720.0008973e-064e-06

0

5e-065e-065e-06

1.6e-052.5e-051.7e-05

8e-061.7e-054e-06

5e-060.0008080.0007465e-065e-06

0.0006990.0076560.0014360.010138e-060.0006880.009778

4.4e-05

3e-061e-064e-06

7.9e-05

7.9e-05

7.9e-05

0.0714140.0414490.0402860.0253760.0446140.0686290.018727

0.000364

0.000178

9.1e-05

9.5e-05

0.0001990.000170.0001790.000104

2.3e-05

0.0001140.0001070.0001088.1e-05

8.5e-056.3e-057.1e-05

0.0685070.0393250.0375610.0214640.0417520.0658630.009073

0.000370.0001430.0002380.0001460.0003850.004243

0.0002070.000225

0.0673610.0390080.0362810.0214640.0404730.06467

0.0003890.000421

0.0001543.2e-057.2e-050.00016

0.0001363e-050.00010.0001447.7e-05

0.0001583.3e-057e-050.0001659.7e-05

0.000161

0.0003287.9e-050.0004468.4e-050.0003390.004656

0.0026730.0021240.0027250.0039120.0022950.0025580.009461

0.0026730.0021240.0027250.0039120.0022950.0025580.009461

3.5e-053.3e-052.9e-057e-05

3.5e-053.3e-052.9e-057e-05

1.9e-05

1.9e-05

0.0002368.4e-050.0015050.0004910.0002154.7e-05

0.0001788.4e-050.0015050.0002110.0001663.1e-05

0.0001788.4e-050.0015050.0002110.0001663.1e-05

0.000188

0.000188

5.8e-059.2e-054.9e-051.6e-05

5.8e-059.2e-054.9e-051.6e-05

0.0104320.0060070.0047140.0061190.0027780.0100190.001614

6.5e-05

6.5e-05

0.0002220.0003240.0002130.0002150.000893

6.3e-05

4.3e-051.5e-054.8e-050.000177

7.8e-050.0003243.6e-057.2e-050.000351

5.3e-055.1e-054.7e-05

4.8e-054.8e-054.8e-050.000208

0.000157

7.4e-05

7.4e-05

4.5e-05

4.5e-05

0.0100070.0059740.004390.0061190.0020780.0096240.000667

6.3e-054.1e-055.7e-05

0.0092310.0054160.0037420.0039120.0017480.00886

7.3e-05

8.8e-051.4e-054.4e-050.0001043.3e-05

2.6e-05

0.0006250.0005440.0006480.0022071.9e-050.0006030.000634

1.9e-05

0

0

2.1e-05

0

8.7e-05

0.0001513.3e-050.0001750.0001365.4e-05

5.2e-053.8e-054.5e-05

4.9e-05

1.4e-051.1e-051.5e-051.2e-052.2e-05

8.5e-052.2e-057.3e-057.9e-053.2e-05

5.2e-050.0001284.4e-05

4e-069e-062e-06

2.2e-052.6e-051.8e-05

4.3e-05

1.9e-052.8e-051.7e-05

7e-062.2e-057e-06

0.0010640.0013760.0007030.0021060.0007910.0010170.001416

0.0010640.0013760.0007030.0021060.0007910.0010170.001416

0.0006960.0013450.0007030.0021060.0003320.0006670.00139

8.3e-057.5e-057.2e-05

0.000119

0.0001423.1e-054.8e-050.000139

9.9e-05

6.1e-053.2e-055.8e-05

8.2e-057e-058.1e-052.6e-05

1.6e-05

7.4e-050.0001047.4e-057e-06

7.4e-050.0001047.4e-057e-06

7.4e-050.0001047.4e-057e-06

0.0047590.0022420.0033910.0017050.0032580.0047940.000921

0.000203

0.000203

9.4e-051.5e-05

1.5e-05

9.4e-05

7.1e-050.0001565.9e-057.5e-05

7.1e-050.0001565.9e-057.5e-05

0.0046880.0022420.0033910.0017050.0028050.0047350.000831

0.0008760.0003570.0003890.0002520.000890.000355

0.0010320.001117

0.0007830.0003320.0002670.0003020.0008070.000119

0.0020470.0011770.0013020.0017050.0008870.002031

0.0009820.0003760.0004010.0002470.0010070.000357

0.0044110.005770.0067010.0106330.0031630.0042380.005359

5.2e-058.2e-053.3e-05

5.2e-058.2e-053.3e-05

5.2e-058.2e-053.3e-05

0.0008380.0004610.0023790.0020060.000360.000810.000722

0.0008350.0004610.0023790.0020060.0003440.0008050.000722

1e-056e-063e-06

6e-064e-066e-06

7.4e-051.7e-057.7e-057.2e-05

1.7e-050.000334e-061.7e-050.000217

1.6e-050.0001311e-051.8e-050.000105

1.7e-056e-061.8e-05

4e-061e-061e-06

9.5e-05

1.5e-050.0018520.0020067e-061.6e-05

4e-062e-066e-06

6e-063e-068e-06

0.0002350.000255

1.1e-052.1e-057e-06

3e-062e-063e-06

3.7e-051.5e-053.9e-05

0.0001123.1e-053.9e-053.9e-05

9e-063e-061.1e-05

1e-054e-061.1e-05

0.0003060.0002926.8e-050.0003283.4e-05

0.0001293.5e-050.000147

4.9e-051e-055e-05

3e-061.6e-055e-06

3e-061.6e-055e-06

0.0035210.0053090.0043220.0086270.0026290.0033950.004637

0.0013180.0033440.0017950.0029090.0017830.0012670.003471

0.0013170.0033440.0017950.0029090.0017010.0012660.003253

1e-067.4e-051e-060.000218

08e-060

0.0021870.0019650.0025270.0057180.000770.0021070.001166

0

3.3e-05

5e-065.4e-055e-06

0.0019290.0019650.0015530.0020060.0018590.0011

0.0002410.0002530.0015050.0001830.0002316.6e-05

02.6e-050

1e-050.0007210.0022070.0004741.1e-05

2e-0601e-06

6e-064.1e-055e-06

2.7e-05

6e-061.1e-055e-06

03e-06

1e-053.5e-051.6e-05

1e-061e-061e-06

5e-069e-066e-06

4e-06

3e-061.2e-058e-06

1e-069e-061e-06

9.2e-05

9.2e-05

9.2e-05

0.000490.0006940.0010670.0004740.000483

0.000490.0006940.0010670.0004740.000483

8e-05

8e-05

0.0001740.0001480.0001721.6e-05

7e-053.9e-056.9e-051.6e-05

3.5e-05

7.5e-053.6e-056.8e-05

2.9e-053.8e-053.5e-05

0.0001720.0006940.000690.0001680.00045

0.0001729.3e-050.000168

0.00011

0.0004160.00045

0.000186

0.0002780.000301

0.0001047.9e-059.8e-05

0.0001047.9e-059.8e-05

4e-057e-053.6e-051.7e-05

4e-057e-053.6e-051.7e-05

1.1e-054.9e-057e-06

1.1e-054.9e-057e-06

1.1e-054.9e-057e-06

1.1e-054.9e-057e-06

6.3e-050.0003885.2e-05

6.3e-050.0003885.2e-05

2.7e-050.0002022.2e-05

8e-060.0001055e-06

1.9e-059.7e-051.7e-05

3.6e-050.0001863e-05

2e-058.9e-051.6e-05

1.6e-059.7e-051.4e-05

0.1030840.1403420.0678230.0420260.0825810.0989110.050627

0.000110.0002079.6e-056.2e-05

4.8e-056.3e-053.4e-05

4.8e-056.3e-053.4e-05

9e-068.1e-051.7e-05

9e-068.1e-051.7e-05

5.3e-056.3e-054.5e-05

5.3e-056.3e-054.5e-05

6.2e-05

6.2e-05

1.1e-05

1.1e-05

1.1e-05

0.1029740.1403420.0678230.0420260.0823740.0988150.050554

4.4e-056.5e-053.8e-05

2.2e-055.8e-052.6e-05

2.2e-057e-061.2e-05

4.5e-054e-054.1e-05

4.5e-054e-054.1e-05

4.5e-050.0001063.8e-05

4.5e-050.0001063.8e-05

1.1e-05

1.1e-05

3.4e-05

3.4e-05

5e-06

5e-06

1.9e-051.6e-05

1.9e-051.6e-05

0.00015

0.00015

9.5e-057.6e-059.2e-05

9.5e-057.6e-059.2e-05

7.4e-05

7.4e-05

0.000109

0.000109

1e-05

1e-05

0.000118

0.000118

0.000164

0.000164

0.0001070.0001180.000101

0.0001070.0001180.000101

7.8e-05

7.8e-05

4.5e-05

4.5e-05

0.00016

0.00016

0.0002220.0001920.0002121.6e-05

0.000150.0001110.0001461.6e-05

7.2e-058.1e-056.6e-05

0.000279

0.000104

0.000175

1e-05

1e-05

0.000199

0.000199

4e-050.0001183.9e-052.1e-05

2.3e-053.5e-052.2e-051e-05

1.7e-058.3e-051.7e-051.1e-05

0.000160.0001780.000164

0.0001130.0001090.000116

4.7e-053.5e-054.8e-05

3.4e-05

0.000128

0.000128

0.0010110.0013220.0023120.0022070.0007410.0009790.002876

0.0013770.001492

0.000860.0013220.0009350.0022070.0006180.0008260.001384

0.0001510.0001230.000153

0.1011860.139020.0655110.0398190.0792360.0970950.047571

0.1011610.139020.0641330.0398190.0777440.0970690.047571

2.5e-050.0010430.0011292.6e-05

0.0003350.000363

0.0034140.0024970.0022520.0025080.0043790.003270.001988

0.0004463.8e-050.0008150.000433

0.0001480.0002090.000143

0.0001480.0002090.000143

2e-060.000143e-06

2.3e-05

0.000114

2e-063e-063e-06

9.7e-053.8e-059.8e-050.000101

9.7e-053.8e-059.8e-050.000101

6.1e-053.7e-056.2e-05

2.4e-051.7e-052.5e-05

3.7e-052e-053.7e-05

0.000147

0.000147

7e-050.000126.2e-05

5.7e-05

7e-056.3e-056.2e-05

6.8e-056.4e-056.2e-05

6.8e-056.4e-056.2e-05

0.0029680.0024590.0022520.0025080.0035640.0028370.001988

0.0002460.000266

0.0002460.000266

2.2e-059.5e-051.4e-05

2.2e-059.5e-051.4e-05

0.000164

0.000164

0.0023250.0024460.0020060.0025080.0022060.0022320.001976

0.0001910.0001710.000184

0.0021340.0024460.0020060.0025080.0020350.0020480.001976

0.0002331.3e-050.000170.0002321.2e-05

0.0001186.5e-050.000121.2e-05

0.0001151.3e-053.7e-050.000112

1.8e-05

5e-05

0.000173

0.000173

0.0001380.0001350.000139

0.0001380.0001350.000139

5.3e-050.0001264.6e-05

5.3e-050.0001264.6e-05

0.0001970.0002290.000174

5.2e-05

9e-050.0001277.9e-05

0.0001075e-059.5e-05

7e-059e-056.6e-05

7e-059e-056.6e-05

7e-059e-056.6e-05

7e-059e-056.6e-05

4.3e-052.8e-053.8e-05

1.3e-052.2e-051.1e-05

1.4e-054e-051.7e-05

0.0725680.0465080.1248450.0604820.0940580.069550.094741

0.0005360.0001650.000270.0015050.0004550.0005016.4e-05

0.0005360.0001650.000270.0015050.0004550.0005016.4e-05

0.0003090.0001650.000270.0015050.0002670.0002936.4e-05

0.0003090.0001650.000270.0015050.0002670.0002936.4e-05

9.8e-050.0001418.6e-05

2.5e-055.3e-052.3e-05

1e-052.1e-057e-06

9e-062.2e-057e-06

3.5e-051.7e-052.9e-05

1.9e-052.8e-052e-05

5.2e-051.8e-054.7e-05

5.2e-051.8e-054.7e-05

7.7e-052.9e-057.5e-05

7.7e-052.9e-057.5e-05

0.0021720.000370.0019590.0024870.0019570.00042

0.0001240.0001099.9e-05

0.0001240.0001099.9e-05

0.0001240.0001099.9e-05

0.0015910.000370.0019590.0021510.0014760.000388

0.0008570.000280.0017030.0018370.0007980.000241

0.0008570.000280.0003630.0003850.0007980.000241

0.001340.001452

0.0007349e-050.0002560.0003140.0006780.000147

0.0005219e-050.0002568e-060.0004750.000147

0.000102

0.0002130.0001890.000203

1.5e-05

0.0004570.0002270.0003823.2e-05

0.0001840.0001220.0001651.6e-05

0.0001840.0001220.0001651.6e-05

0.0002730.0001050.0002171.6e-05

0.0002730.0001050.0002171.6e-05

0.0334790.020850.077360.0294880.0517150.0324080.048688

0.0125150.013555

0.0125150.013555

0.0125150.013555

0.0010620.0006690.0033870.0042130.0012840.0008260.000722

0.0005590.0006170.0031190.0042130.0005180.0005060.000643

1.3e-050.00053501.2e-050.00058

7e-06

3.1e-05

3.1e-05

3.5e-05

0.0001184.1e-050.0001121.6e-05

9.6e-05

0.0001780.0005890.0002690.0017053.4e-050.0001711.6e-05

8.9e-05

0.0002298.9e-050.0001891.6e-05

2.1e-052.8e-050.0023150.0025086e-062.2e-051.5e-05

5.9e-05

0.0003785.2e-050.0002680.0001210.0002367.9e-05

3.9e-05

9e-06

0.0003785.2e-050.0002688.2e-050.0002367e-05

4.7e-050.0001242.8e-05

7.7e-05

4.7e-054.7e-052.8e-05

4.9e-058.9e-054.3e-05

4.9e-058.9e-054.3e-05

0.000106

0.000106

0.000126

0.000126

0.000124

0.000124

2.9e-057.6e-051.3e-05

2.5e-055.6e-051e-05

4e-062e-053e-06

0.0008270.0204910.009360.000690.013481

4.3e-05

4.3e-05

0.0003010.0002040.0001610.0002471.7e-05

0.0003010.0002040.0001610.0002471.7e-05

6.3e-05

6.3e-05

0.0001037.7e-058.9e-05

6.1e-055.2e-05

4.2e-057.7e-053.7e-05

5.3e-05

5.3e-05

0.0202870.0087820.013367

0.000119

5.7e-05

0.0123410.013367

0.0079460.008606

0.0002439.7e-050.000207

0.0002439.7e-050.000207

0.000188.4e-050.0001479.7e-05

9.7e-05

0.000188.4e-050.000147

0.0104510.008530.0084980.0109330.008210.009970.009803

0.0005057.8e-050.0003270.0004640.0004716.4e-05

0.0001519.7e-050.0001284.7e-05

0.0003547.8e-050.0003270.0003670.0003431.7e-05

0.0065390.0062880.0061770.0072220.005820.0062630.008879

7.8e-05

0.0065390.0062880.0061770.0072220.0057420.0062630.008879

0.000133

5.7e-05

7.6e-05

2.5e-055.6e-051.9e-05

2.5e-055.6e-051.9e-05

0.0001382.4e-054.9e-050.000144

7.5e-051.6e-057.4e-05

6.3e-052.4e-051.2e-057e-05

2.1e-05

0.000122

7.5e-05

4.7e-05

0.0001654.7e-055.2e-050.000147

0.0001654.7e-055.2e-050.000147

7.7e-05

3.7e-05

4e-05

8.5e-057.7e-057.3e-05

8.5e-057.7e-057.3e-05

0.0001940.0001710.000182

0.0001940.0001710.000182

0.0023960.0020930.0019940.0037110.0009630.0023210.000735

0.0001254.3e-050.000112

0.0004650.0002040.0003490.0018050.0002940.000449

4e-05

0.0001778.4e-050.0001693.5e-05

0.0015340.0010580.0008780.0019060.0004790.0014980.0007

9.5e-050.0008310.0007672.3e-059.3e-05

0.0001254.7e-050.000112

0.0001254.7e-050.000112

0.000102

0.000102

0.0002797.7e-050.0002380.000125

0.0002797.7e-050.0002380.000125

0.0162370.0093920.0298950.010330.0304390.0163330.009475

0.0016150.0005410.0019930.0018050.0013570.0015390.000522

0.0015820.0013510.0013140.001522

1.5e-05

3.3e-050.0005410.0006420.0018052.8e-051.7e-050.000522

0.0001350.0004940.000119

2.6e-05

1.6e-05

8.7e-05

2.7e-05

2.8e-05

2.5e-05

9.1e-05

0.0001351.5e-050.000119

0.000179

0.0017440.0009010.0009110.0016050.0007430.0017140.000707

0.000112

1.9e-05

0.0012640.0004940.0005960.000390.0012312.8e-05

0.000480.0004070.0003150.0016050.0001130.0004830.00066

0.000128

0.0031340.0033030.0013190.006920.0014820.0031070.003284

3.5e-05

4e-06

5.4e-051.4e-051.1e-055.8e-05

6.1e-05

4.6e-053.2e-056e-064.6e-051.7e-05

0.0011490.0009250.0007020.0018050.0003020.0011180.000853

0.000114

2.1e-05

9.3e-053.2e-059.6e-057e-06

2.3e-055e-062.4e-056e-06

3.8e-058e-063.6e-05

8e-050.0018490.0003990.0018051.3e-057.9e-050.000171

1e-06

0.0001310.0002680.0018051.1e-050.0001270.001864

0.0002022.1e-050.0001981.8e-05

0.0001333.8e-050.000125

0.0002863.2e-054.1e-050.0002826.1e-05

5.7e-05

2.2e-05

1e-06

7.7e-05

0.0003540.0001190.0002180.0015050.0002230.000350.000161

1.7e-05

1.6e-05

0.0001452.6e-052.7e-050.000153

5e-06

0.0001081.2e-050.000111

1e-05

2.8e-05

6e-05

4.9e-05

0.0001011.4e-053e-060.0001060.000103

2.4e-05

2.1e-05

1.2e-05

2.3e-05

0.0001236.3e-050.000129

6.8e-052.4e-058e-066.9e-052.3e-05

0.0004910.0003480.0004471e-06

0.0001393.8e-050.000135

0.0001214e-050.000112

9.2e-055.1e-057.5e-05

3.3e-052.8e-053e-05

3.7e-05

9.3e-05

0.0001066.1e-059.5e-051e-06

0.0091180.0046470.0256720.0260150.0094070.004961

0.0197870.021431

0.0018870.0002770.0006480.001290.0019320.000591

0.0027870.0011710.0017320.0028750.001747

0.002250.0027710.0016410.0009230.0023260.002024

0.0010570.001145

0.0021940.0004280.0008070.0012260.0022740.000599

0.0049020.0022590.0025740.0040120.0024220.0045890.001652

0.0036640.0019890.0017780.0023070.0012020.0034790.001372

0.0036640.0019890.0017780.0023070.0011240.0034790.001372

4.4e-05

3.4e-05

8e-050.000225.4e-05

8e-058e-055.4e-05

5.8e-05

8.2e-05

0.000174

8.2e-05

9.2e-05

0.0010510.000270.0007960.0017050.0007620.0009650.00028

0.0010510.000270.0003380.0017050.0001530.0009650.00028

0.0004580.000496

0.000113

0.0001076.4e-059.1e-05

0.0001076.4e-059.1e-05

0.0092630.0098560.0288980.0186570.0116220.0087690.032796

0.0083220.0096510.0285830.0171520.0110540.0080060.032508

0.0003510.00038

0.0003510.00038

0.0014720.001594

0.0014720.001594

0.0002860.000309

0.0002860.000309

0.0019760.0011660.000988

0.0010770.0011661.5e-05

0.0008990.000973

0.0083220.0096510.0244980.0171520.0095790.0080060.029546

0.0006890.000746

0.0022660.002454

0.0022040.0014260.0006950.0023070.0002010.0014650.001378

0.0073190.007928

0.0046120.0018910.0013320.0106320.0018780.0045820.000389

0.0029050.003146

0.0017320.001876

0.0018520.0049010.001261

0.0015060.0063340.0006460.0042130.0001450.0019590.007209

0.0002450.000266

0.00013

0.0030890.003345

0.0017280.001872

0.0009410.0002050.0003150.0015050.0005680.0007630.000288

0.0005660.0002050.0003150.0015050.0003730.0004910.000179

0.0005660.0002050.0003150.0015050.0003730.0004910.000179

0.0001730.00010.0001127.5e-05

0.0001730.00010.0001127.5e-05

0.0002029.5e-050.000163.4e-05

0.0002029.5e-050.000163.4e-05

0.0271180.0152670.0163580.0108320.0277790.0259150.012773

0.0001450.0003270.000121

2.6e-050.0001132.5e-05

5.7e-05

2.6e-055.6e-052.5e-05

0.0001190.0002149.6e-05

4e-054.6e-051.1e-05

4.7e-05

1.9e-05

3.5e-054.4e-052.7e-05

3.2e-054.5e-054.1e-05

1.2e-051.3e-051.7e-05

0.0007120.0005180.000348

9.6e-05

9.6e-05

0.0007120.0004220.000348

0.000390.000422

0.0003220.000348

0.0256520.0150310.015130.0108320.0231490.0245960.012012

7.3e-051.7e-056.3e-05

4.1e-058e-063.7e-05

3.2e-059e-062.6e-05

0.000137

7.1e-05

6.6e-05

6e-05

6e-05

6.3e-056.2e-054.5e-05

6.3e-056.2e-054.5e-05

0.0255160.0150310.015130.0108320.0228730.0244880.012012

4.1e-059.4e-053.9e-05

0.0254750.0150310.015130.0108320.0227790.0244490.012012

0.0010150.0002360.0005160.0034890.0009760.000413

0.0010150.0002360.0005160.0034890.0009760.000413

0.0010150.0002360.0005160.0034890.0009760.000413

0.0003060.0002960.000222

0.0001120.0001267.8e-05

0.0001120.0001267.8e-05

0.0001339.3e-050.000101

0.0001339.3e-050.000101

6.1e-057.7e-054.3e-05

6.1e-057.7e-054.3e-05

0.0016690.0008320.0040630.0052150.0018580.0015360.000492

1.3e-055.1e-051e-05

1.3e-055.1e-051e-05

3e-062.7e-055e-06

3e-062.7e-055e-06

1e-052.4e-055e-06

1.1e-05

1e-051.3e-055e-06

0.0016480.0008320.0040630.0052150.0017660.0015180.000492

0.0015060.0008320.0009140.0018050.0013990.001430.000492

3e-062.8e-053e-06

3e-062.8e-053e-06

0.001460.0008320.0009140.0018050.0013070.0013990.000492

0.0014370.0008320.0009140.0018050.0011780.0013780.000492

2.4e-05

000

3e-05

9e-069e-06

7e-061e-067e-06

1e-062.8e-050

2e-068e-062e-06

1.4e-05

1e-067e-061e-06

3e-061.7e-052e-06

1.6e-051.9e-058e-06

1.6e-051.9e-058e-06

2.7e-054.5e-052e-05

8e-062.3e-057e-06

1.9e-052.2e-051.3e-05

1.6e-053.2e-051.4e-05

1.6e-053.2e-051.4e-05

1.6e-053.2e-051.4e-05

0.0001260.0031490.003410.0003357.4e-05

6.6e-056.2e-052.9e-05

1.4e-052.2e-051.3e-05

7e-061.1e-057e-06

4.5e-052.9e-059e-06

5.2e-050.0031490.003410.0002183.9e-05

1e-051.6e-05

2.8e-053e-062.8e-05

4e-06

1e-060.0031490.003411e-061e-06

8e-06

1e-064e-061e-06

1e-061.4e-05

1.2e-05

5e-062e-065e-06

6e-063.5e-054e-06

4e-06

2.1e-05

1.8e-05

5.2e-05

1.1e-05

1.3e-05

8e-065.5e-056e-06

1.1e-05

5e-06

1e-05

7e-061.3e-054e-06

1e-061e-052e-06

6e-06

8e-064.1e-058e-06

8e-064.1e-058e-06

8e-064.1e-058e-06

8e-064.1e-058e-06

0.1239790.1566840.185310.1811480.1870260.1524840.183056

0.000117

0.000117

4.9e-05

4.9e-05

6.8e-05

6.8e-05

0.0009594.9e-050.0013330.0026460.0007844.7e-05

9.7e-050.0001339.1e-05

9.7e-050.0001339.1e-05

5.6e-05

6.9e-057.1e-056.2e-05

4e-06

2.8e-052e-062.9e-05

3.8e-05

3.8e-05

3.8e-05

0.0001660.0002230.0001253.2e-05

6.5e-053.2e-054.5e-05

6.5e-053.2e-054.5e-05

4.5e-05

4.5e-05

0.0001018.8e-058e-053.2e-05

4.1e-052.6e-053.1e-05

3.4e-053.5e-053.1e-051.6e-05

2.6e-052.7e-051.8e-051.6e-05

5.8e-05

5.8e-05

0.0005894.9e-050.0013330.0021080.0004831.5e-05

0.0001660.0001210.000154

0.0001660.0001210.000154

7.6e-051.3e-054e-06

7.6e-051.3e-054e-06

4.7e-05

4.7e-05

9.3e-05

9.3e-05

5.7e-051.3e-054.8e-05

5.7e-051.3e-054.8e-05

0.000294.9e-050.0013330.0018210.0002771.5e-05

0.000132

0.000141

0.0010930.001184

0.000294.9e-050.000240.0002240.0002771.5e-05

0.00014

6.6e-059.8e-055.3e-05

6.6e-059.8e-055.3e-05

3.3e-05

6.6e-053.6e-055.3e-05

2.9e-05

4.1e-054.6e-053.2e-05

4.1e-054.6e-053.2e-05

4.1e-054.6e-053.2e-05

0.0068830.0110110.0098860.0138430.0026120.0053540.011795

0.0068830.0110110.0098860.0138430.0026120.0053540.011795

0.000324.4e-050.0002510.0003279.3e-05

7.6e-050.0001017.4e-05

0.0001834.8e-050.000193

5.8e-05

6.1e-054.4e-054.4e-056e-05

9.3e-05

0.0003480.0003510.0003470.0016050.0002850.0003410.000313

0.0003480.0003510.0003470.0016050.0002850.0003410.000313

7.6e-050.0001135.7e-051.5e-05

7.6e-050.0001135.7e-051.5e-05

2.2e-053.4e-053.1e-05

2.2e-053.4e-053.1e-05

7e-050.0001115.9e-05

7e-050.0001115.9e-05

0.0031690.0030340.001520.0025080.0007180.0019980.002838

0.000260.0001070.0002742.1e-05

2.1e-05

0.0029090.0030340.001520.0025080.0006110.0017240.002796

3.9e-050.0001130.0015050.0001333.6e-053.1e-05

7.3e-05

3.7e-05

3.9e-050.0001130.0015052.3e-053.6e-053.1e-05

8.3e-055.3e-058e-05

8.3e-055.3e-058e-05

0.0027560.0074690.0080190.0082250.0009140.0024250.008505

0.0001191.9e-050.000115

0.0018520.0072660.0009620.0050150.0003750.0016010.000628

0.0002016.2e-059e-057e-05

0.0005840.0002030.0004380.003210.0004580.0006190.000524

0.006310.006835

0.0003090.000335

0.000113

0.0205190.0438120.0201360.0338020.0082140.0420780.029286

0.0202840.0438120.0201360.0338020.0079460.0418990.029286

0.0002134.8e-050.0001050.000160.000237

4.4e-05

2e-05

1e-06

0.0002134.8e-051.7e-050.000160.000217

0

4.3e-05

6.7e-05

1.7e-05

2.2e-05

2.8e-05

6.8e-050.0001570.0015054.9e-056.6e-057.9e-05

6.8e-050.0001570.0015054.9e-056.6e-057.9e-05

0.000690.0003130.0001130.0005179.7e-05

0.0002996.4e-050.0002175e-05

0.0003910.0003134.9e-050.00034.7e-05

0.0193130.0436070.0198230.0322970.0076120.0411560.028873

0.0010420.0003190.0004520.0024070.0001950.0010860.000592

0.0008610.0013670.0009730.0019060.0004780.0019730.001482

0.0023910.0006050.0008630.003410.0003870.002518

6e-05

8.1e-05

0.0005760.0001650.0001210.000616

5e-060.00250.0027082e-067e-06

0.0132550.0359270.0106650.0164490.0035030.0336980.024847

3.4e-050.0010810.0013310.0025087e-064.3e-050.001321

0.0023320.002526

0.0011490.0041430.0007070.0029090.0002520.0012150.000631

0.0002350.0002680.000179

0.0001614.4e-050.000124

0.0001614.4e-050.000124

0.000103

3.6e-05

2.5e-05

4.2e-05

7.4e-054.8e-055.5e-05

7.4e-054.8e-055.5e-05

7.3e-05

7.3e-05

0.0001620.0004210.000135

0.0001620.0004210.000135

1.2e-050.0001961.5e-05

5.2e-05

1.2e-057.4e-051.5e-05

7e-05

2.1e-050.0001322e-05

2.1e-050.0001322e-05

0.0001299.3e-050.0001

0.0001299.3e-050.0001

0.0007370.0030430.0005030.0030090.000330.0006580.003116

0.0007340.0030430.0005030.0030090.0002690.0006570.003116

3.6e-05

3.6e-05

0.0007340.0030430.0005030.0030090.0002330.0006570.003116

2.2e-05

2.8e-05

0.0007230.0030430.0005030.0030090.0001410.0006450.003116

2e-05

1.1e-052.2e-051.2e-05

3e-066.1e-051e-06

2e-05

2e-05

3e-064.1e-051e-06

3.1e-05

3e-061e-051e-06

0.0016340.0009340.0012460.0018050.0003590.0014830.001757

0.0016340.0009340.0003640.0018050.0003590.0014830.000802

0.0013590.0009340.0003640.0018050.0002140.0013010.000786

9e-06

6.3e-05

0.0010220.0008960.0003640.0018050.0001060.0009880.000483

0.000185

0.0002023.8e-051.3e-050.0001890.000118

0.0001352.3e-050.000124

0.000178.1e-050.0001071.6e-05

0.000178.1e-050.0001071.6e-05

0.0001056.4e-057.5e-05

0.0001056.4e-057.5e-05

0.0008820.000955

0.0008820.000955

0.0008820.000955

0.0137640.0330820.0542760.0285880.0727560.012510.079704

0.0029910.0064480.0021220.0114360.0015760.0027250.009192

0.0028660.0062950.0021220.0099310.0014360.0026110.00917

3.7e-05

2e-06

6.3e-051.3e-056.8e-05

3.7e-055e-063.6e-05

5e-06

1.6e-05

4.4e-053.4e-050.0012960.0014041.1e-053.8e-059.5e-05

7.8e-053.9e-051.4e-057.7e-057.1e-05

7e-06

2.2e-05

7.9e-051.2e-057.1e-05

5e-06

4.7e-05

1e-058e-061e-05

4.5e-05

4e-06

4.4e-05

4e-06

1.5e-05

0.000121.9e-050.000119

3.1e-05

1.7e-05

7e-05

0.00012

0.0010940.0058710.0008260.0055170.0002290.0010490.008638

8.7e-052e-058.3e-05

0.0001093.3e-050.000102

4e-06

6.2e-052e-065.8e-05

3.8e-05

1.3e-05

8e-06

4.6e-051.1e-054.4e-05

9.5e-05

6.8e-052.4e-051.2e-056.5e-057.2e-05

7e-06

3e-06

1e-05

1e-05

3.2e-05

1.1e-05

1.1e-05

2.7e-05

3e-06

0.0001313.8e-050.000116

8.1e-051.3e-052.1e-057.3e-051.6e-05

4.5e-05

3.1e-05

1.6e-05

0.0002050.0001040.0015057.2e-050.0001930.00017

2.2e-05

2.6e-05

0.0003840.000210.0015050.0001080.0003676.3e-05

5.4e-05

4.8e-051.1e-054.2e-05

5e-06

1.4e-05

1.4e-05

0.0001250.0001530.0015050.0001260.0001142.2e-05

0.0001250.0001530.0015057.7e-050.0001142.2e-05

4.9e-05

0.0107730.0266340.0521540.0171520.071180.0097850.070512

0.0099020.0258590.0517180.0155470.0703240.0089270.07033

0.001080.00117

0.0007110.00077

0.0054870.0240570.0032970.0155470.0008850.0043040.033454

0.0421310.06470.035245

0.0020950.0008630.0009570.0005670.002177

3.2e-05

0.0013040.0009390.0004490.0001770.0013519.4e-05

0.0005620.000609

0.0010160.0002860.0002050.0010950.000163

2e-05

7.3e-05

0.000880.000953

5.9e-05

0.000340.000368

0.0008060.000873

0.0002190.000237

0.0001020.0002230.0001580.0001

6.9e-05

0.0001020.0002232.2e-050.0001

6.7e-05

0.0007690.0005520.0004360.0016050.0006980.0007580.000182

2.8e-05

4.4e-05

2.9e-059e-062.7e-05

1e-05

2.8e-05

3.7e-051.8e-054.5e-05

0.0006830.0005520.0004360.0016050.0005520.0006650.000182

2e-059e-062.1e-05

0.0006930.0022370.0005430.0030090.0006220.0009120.002827

0.0002830.0002780.000209

3.7e-054.6e-051.6e-05

3.7e-051.9e-051.6e-05

2.7e-05

5.2e-054.3e-055.5e-05

5.2e-054.3e-055.5e-05

6e-06

6e-06

0.0001790.0001530.000128

0.0001399.4e-050.000128

4e-055.9e-05

1.5e-053e-051e-05

01.9e-050

1.5e-051.1e-051e-05

8.9e-05

3.7e-05

3.7e-05

5.2e-05

5.2e-05

0.000410.0022370.0005430.0030090.0002550.0007030.002827

0.000410.0022370.0005430.0030090.0002340.0007030.002827

1.8e-05

7e-064e-061e-05

0.0003960.0022370.0005430.0030090.0001970.0006820.002827

5e-068e-065e-06

7e-06

2e-0606e-06

2.1e-05

2.1e-05

0.0391090.0343750.0655430.0674040.0578710.0502090.036207

8.4e-05

8.4e-05

8.4e-05

0.000173

0.000173

0.000173

0.0081170.0106050.0094560.0203630.0033550.0112580.013804

0.000101

0.000101

0.0003540.0002060.0001270.000363

0.0003540.0002060.0001270.000363

4e-05

4e-05

6e-05

6e-05

0.0015590.0047030.0028750.0081250.0002630.0015160.004543

6.1e-050.0005693.7e-053.1e-050.000616

5.3e-053.9e-050.0013890.0015057e-065.4e-05

0.0001530.0002480.0002620.0020062.1e-050.0001580.003927

0.0012920.0044160.0006550.0046140.0001980.001273

0.0017680.0001980.0009030.0015050.0003190.0016735e-05

1e-06

7e-06

7e-06

2e-06

1e-06

2e-06

0.0003140.0002931.3e-050.000321.6e-05

1.3e-05

0.0002031.6e-057e-060.000176

0.0003750.0003345.6e-050.000349

4.2e-05

0.0006410.0001820.0002760.0015050.0001370.000593

5e-06

9e-06

0.0002351.7e-050.000235

3.4e-05

4.6e-054.2e-055.8e-05

4.6e-054.2e-055.8e-05

9e-05

9e-05

0.000103

0.000103

7.9e-05

7.9e-05

8.7e-05

8.7e-05

4.5e-050.0002244.9e-05

6e-062e-065e-06

2.1e-055.2e-052.6e-05

5.8e-05

5.8e-05

9e-062e-069e-06

9e-065.2e-059e-06

0.0002760.0007090.0019910.0025086.5e-050.0002620.003283

1.9e-050.000131

7.9e-050.0007090.0012380.0025089e-067.6e-050.002336

0.0001973.7e-050.000186

0.0007530.000816

0.0014110.0006280.0009820.0017050.0006760.0013990.000509

0.000310.0002954.1e-050.00033

2.8e-05

3.7e-05

0.0011010.0006280.0006870.0017050.0005410.0010690.000455

2.5e-05

2.6e-05

3.2e-05

0.0002232.5e-050.000234

0.0002232.5e-050.000234

2.8e-05

2.8e-05

0.0003770.0004420.0004110.0016050.0001250.0003770.000563

2e-05

0.0003080.0004420.0004110.0016053.8e-050.0003010.000563

5e-06

1.5e-05

6.9e-052.4e-057.6e-05

2.1e-05

2e-06

0.0010690.0035310.0013120.0029090.0001630.0010530.003091

2.8e-052e-062.3e-05

0.0007670.0035160.0013120.0029093e-050.0007930.003091

0.000129e-068.7e-05

0.0001541.5e-051.2e-050.00015

1e-06

0.000107

2e-06

7.9e-05

7.9e-05

9.1e-05

9.1e-05

4.3e-054.1e-057.6e-05

4.3e-054.1e-057.6e-05

7.4e-05

5.7e-05

1.7e-05

5.6e-055.4e-05

5.6e-055.4e-05

0.0006590.0002910.0002070.0005963e-05

3e-05

9.1e-056.8e-055.9e-05

0.0003030.0002915.2e-050.000328

4.9e-056e-052.3e-05

0.0002162.7e-050.000186

6e-05

6e-05

0.0002310.0003940.0004850.0020060.0001860.0035480.001735

0.0001428e-060.0001289e-06

8.9e-050.0003940.0004850.0020060.0001780.003420.001726

0.0021430.0008280.00190.0031090.0010560.0023020.000685

0.0014510.0008140.0016350.0031090.0007760.001710.000609

0.0005548.7e-050.0003351.7e-050.0005380.000217

0.0004220.0006320.000358

0.0001952.5e-050.000197

0.0004925.8e-050.0005539.7e-050.0007653.4e-05

0.000210.0006690.0003250.0031095e-060.00021

0.0001410.0001450.000106

0.0001414.2e-050.000106

3.7e-05

6.6e-05

0.0005511.4e-050.0002650.0001350.0004867.6e-05

0.0003140.0002658.6e-050.000265

0.0002371.4e-052.3e-050.0002217.6e-05

2.6e-05

0.0016651.2e-050.0008320.0008250.0014510.00053

0.0007861.2e-050.0002690.0003510.0006710.000119

8.7e-051.2e-052.6e-057.3e-057.9e-05

2.3e-05

0.000261.2e-050.0002091.6e-05

0.0003010.0002698.2e-050.0002822.4e-05

0.0001383e-050.000107

5e-05

4.2e-05

8.6e-05

2e-055e-052.3e-05

2e-055e-052.3e-05

2.5e-053.2e-052.8e-05

2.5e-053.2e-052.8e-05

0.0008340.0005630.0003920.0007290.000411

0.00040.0003428.9e-050.0003340.000385

3.3e-056e-063.7e-051e-05

5.6e-055e-066.3e-055e-06

0.0002515.3e-050.00021.1e-05

0.0002210.000239

9.4e-0509.5e-05

0.0014110.0005780.0012676.9e-05

0.000108

0.000108

0.000247e-050.000196

0.000247e-050.000196

0.0002082.9e-050.000189

0.0002082.9e-050.000189

0.0004330.0001540.0003864.7e-05

4.1e-05

0.0002636e-050.000224

0.000175.3e-050.0001624.7e-05

0.0004210.0002080.0004012.2e-05

0.0002333.9e-050.0002141.6e-05

4.1e-05

2.8e-05

4.6e-05

0.0001885.4e-050.0001876e-06

0.0001099e-069.5e-05

0

0.0001099e-069.5e-05

0.0255590.0228820.0533550.0439320.0515220.0337430.021104

0.0016410.0002860.0015860.0008480.0015860.000515

4.8e-05

0.0004382.9e-050.0003744.2e-050.000378

3.8e-05

0.0005665.4e-050.00056.1e-050.00052

9e-06

6.9e-05

0.0005237e-050.0002510.0001360.000561.9e-05

0.0001148.5e-053.3e-050.0001280.000152

0.000206

0.0004610.000499

06.9e-05

1.9e-05

1.1e-05

0.0011520.001248

0.0011520.001248

0.023630.0224390.0506170.0439320.0493740.0318770.020445

8.9e-05

6.9e-050.0048320.0006520.0162491.5e-056.9e-050.000251

0.0095380.01033

0.0012860.0007890.0006210.0056170.0001730.0013060.006557

0.0222750.0168180.0051430.0220660.0012170.0305020.013637

0.0014040.00152

0.0055240.005983

0.0011920.001291

0.0097260.010534

8e-06

0.0168170.018214

0.0002880.0001575.2e-050.000280.000144

0.0002547.4e-051e-050.000240.000144

3.4e-058.3e-054.2e-054e-05

0.0002144.8e-050.0002780.0001881.5e-05

0.000112

0.000112

0.0002144.8e-057e-050.0001881.5e-05

2e-06

0.0001894.8e-053.5e-050.0001681.5e-05

5e-06

1.6e-052.2e-051.3e-05

9e-066e-067e-06

9.6e-05

9.6e-05

2e-057.4e-051.7e-05

2e-057.4e-051.7e-05

2e-057.4e-051.7e-05

2e-057.4e-051.7e-05

8.7e-05

8.7e-05

8.7e-05

8.7e-05

7.5e-05

7.5e-05

7.5e-05

7.5e-05

0.0089550.0053910.0057330.0082240.0078410.0084850.00428

3.7e-052.7e-051.8e-05

3.7e-052.7e-051.8e-05

3.7e-052.7e-051.8e-05

5.2e-053.6e-054.4e-05

5.2e-053.6e-054.4e-05

5.2e-053.6e-054.4e-05

0.0002230.0006220.000212

0.0002230.0006220.000212

7e-06

3e-06

2.3e-05

2.4e-05

2.7e-05

3.6e-054e-062.7e-05

1.7e-05

0.0001330.0005080.000134

5.4e-053e-065.1e-05

6e-06

0.0044690.003030.0033490.0058170.0038530.0042310.002594

0.0038640.002320.0024770.0024070.0032540.0037090.001823

0.003850.002320.0024770.0024070.0032090.0037020.001823

1.4e-054.5e-057e-06

0.0002590.0004970.0006540.0018050.0001490.0002090.000598

1e-06

8e-06

0

2.2e-050.0004970.0006540.0018058e-061.9e-050.000598

0.0002070.0001050.000155

1.4e-05

3e-051.3e-053.5e-05

4.7e-05

4.7e-05

4.2e-05

4.2e-05

0.0003160.0002130.0002180.0016050.0002940.0002860.000173

0.0002390.0002130.0002180.0016050.0001740.000230.000173

3.7e-051.3e-052.6e-05

1.5e-05

1.1e-05

3.5e-05

2.1e-05

1.1e-05

4e-051.4e-053e-05

3e-056.7e-052.7e-05

3e-056.7e-052.7e-05

7.4e-057.6e-056.8e-05

7.4e-057.6e-056.8e-05

4.3e-054.1e-054.4e-05

3.1e-053.5e-052.4e-05

1.6e-054.2e-051.5e-05

1.6e-054.2e-051.5e-05

2.1e-05

1.6e-052.1e-051.5e-05

3.3e-05

3.3e-05

3.3e-05

0.0040840.0023610.0023840.0024070.0031520.0038970.001686

0.0040840.0023610.0023840.0024070.0031520.0038970.001686

2.1e-057e-061.7e-05

3.1e-052.1e-052.8e-05

5.4e-054.9e-05

0.0036780.0023610.0023840.0024070.0029770.0035240.001686

3.8e-051.5e-053.5e-05

2.9e-051.3e-053.5e-05

6.2e-056e-065.2e-05

7e-061.8e-051.2e-05

1.3e-051.2e-051.2e-05

6e-062e-066e-06

5e-052.7e-054.4e-05

3.1e-052.5e-052.5e-05

1e-052e-069e-06

1.4e-055e-061.3e-05

1e-054e-069e-06

2.1e-051.6e-051.8e-05

9e-062e-069e-06

0.0142430.0052230.0100790.0047140.0180440.0137610.003283

0.0138910.0052230.0100790.0047140.0175080.0134480.003283

0.0001540.000180.000117

8.2e-059.1e-056.5e-05

7.2e-058.9e-055.2e-05

0.0001560.0002120.00016

0.0001560.0002120.00016

0.0001310.000150.000119

0.0001310.000150.000119

8.8e-05

8.8e-05

0.000177

0.000177

0.013450.0052230.0100790.0047140.0167010.0130520.003283

0.0003390.000196

0.0127220.0052230.0058270.0047140.0120040.0126460.003267

0.0003890.0002550.0001720.0004061.6e-05

0.0002060.000223

0.0037910.004106

5.5e-05

5.5e-05

5.5e-05

0.0002930.0003040.000263

6.3e-05

6.3e-05

0.0001135.8e-050.000105

0.0001135.8e-050.000105

8e-055.4e-057.3e-05

8e-055.4e-057.3e-05

4e-057.7e-053.5e-05

1e-051.7e-057e-06

1.2e-054.3e-051.2e-05

1.8e-051.7e-051.6e-05

6e-055.2e-055e-05

6e-055.2e-055e-05

5.4e-05

5.4e-05

5.4e-05

5.9e-050.0001235e-05

5.9e-050.0001235e-05

5.9e-056e-055e-05

6.3e-05

0.0159720.0175270.0150380.016750.0134410.0158060.010338

0.0159720.0175270.0150380.016750.0134410.0158060.010338

0.00018.6e-059.8e-051.6e-05

2.5e-05

3.9e-053.6e-054.3e-051.6e-05

6.1e-052.5e-055.5e-05

0.0158370.0175270.0143540.016750.0125120.0156770.010298

3.7e-051.4e-050.0015740.0017051.2e-052.9e-052.4e-05

5.3e-05

4.2e-058e-06

0.0023540.0102820.0021590.0058170.0007160.0026610.01

3.4e-054e-06

2e-050.0025930.0028084e-061.7e-05

3.2e-05

2.9e-051e-052.7e-05

2e-057e-062e-051.6e-05

3.9e-051.9e-05

3.7e-053e-062.4e-052.4e-05

5.9e-05

2.3e-056e-063e-05

0.0129380.0071280.0078030.0049150.0110910.012432

5e-06

0.000107

2e-05

0.0002860.0001030.0002250.0015050.0003210.000284

1e-052.3e-05

3.2e-052.2e-058.5e-055.3e-05

4e-06

6.1e-052.6e-056.8e-051.6e-05

3.5e-050.0001023.1e-052.4e-05

8e-06

3.5e-054.5e-053.1e-05

5.7e-05

1.6e-05

0.0006840.000741

0.0006840.000741

0.0001070.0003840.0003240.0001020.000416

8.7e-05

4.5e-05

4.5e-05

4.2e-05

4.2e-05

0.0003840.000416

0.0003840.000416

0.0003840.000416

0.0001070.000170.000102

2.9e-053.5e-053.1e-05

2.9e-053.5e-053.1e-05

4e-055.5e-054e-05

4e-055.5e-054e-05

2.2e-054.4e-052.1e-05

2.2e-054.4e-052.1e-05

1.6e-053.6e-051e-05

1.6e-053.6e-051e-05

6.7e-05

6.7e-05

2.8e-05

3.9e-05

0.0002220.000610.0011920.00019

0.0002220.000610.0011920.00019

0.000610.000702

0.000610.000661

4.1e-05

7.2e-05

7.2e-05

5.4e-05

5.4e-05

0.000170.0002770.000139

1.8e-053.5e-053.4e-05

1.2e-055.3e-051.1e-05

4.1e-05

0.000148.4e-059.4e-05

6.4e-05

5.2e-052.7e-055.1e-05

5.2e-052.7e-055.1e-05

6e-05

6e-05

0.0032870.0019030.0020630.0021060.0031260.0031060.001649

0.0032870.0019030.0020630.0021060.0031260.0031060.001649

0.0031180.0019030.0020630.0021060.0027910.0029830.001633

0.0031180.0019030.0020630.0021060.0027910.0029830.001633

2.3e-055.1e-051.9e-05

2.3e-055.1e-051.9e-05

0.0030660.0019030.0020630.0021060.0026490.0029410.001633

1.4e-054.2e-05

3e-069e-063e-06

6e-062.2e-055e-06

3e-062.2e-053e-06

2e-06

0.0030540.0019030.0020630.0021060.002580.002930.001591

1.2e-053.1e-051e-05

1.2e-053.1e-051e-05

1.7e-056e-051.3e-05

6e-063e-056e-06

1.1e-053e-057e-06

0.0001690.0003350.0001231.6e-05

2.8e-055e-052.5e-05

2.8e-055e-052.5e-05

2.8e-055e-052.5e-05

0.0001410.0002859.8e-051.6e-05

0.0001410.0002859.8e-051.6e-05

2.5e-05

1.1e-053.4e-058e-06

9e-06

1.9e-05

2.9e-053e-052.1e-05

1.9e-053.9e-051.4e-05

2.1e-053.7e-051.3e-05

1.9e-051.8e-051.4e-05

1.1e-052.4e-058e-06

3.1e-053.9e-052e-051.6e-05

1.1e-05

0.1504860.2146310.1917550.219660.1104690.1329040.241852

0.0223460.0305190.0346260.0310930.0240150.0179730.044827

2.2e-050.0002263.6e-05

1.1e-058.8e-051.4e-05

8e-064.9e-051.2e-05

8e-064.9e-051.2e-05

3e-063.9e-052e-06

3e-063.9e-052e-06

1.1e-050.0001382.2e-05

7e-068.1e-051.9e-05

4e-063.8e-051e-05

3e-064.3e-059e-06

4e-065.7e-053e-06

4e-065.7e-053e-06

7e-063.6e-056e-06

7e-063.6e-056e-06

7e-063.6e-056e-06

7e-063.6e-056e-06

0.0016120.0011070.0009830.003410.0015380.0015410.000597

5e-065.2e-054e-06

5e-065.2e-054e-06

5e-065.2e-054e-06

1.4e-058.5e-054e-06

1e-055.1e-052e-06

1e-055.1e-052e-06

4e-063.4e-052e-06

4e-063.4e-052e-06

0.0006090.0004690.0004140.0016050.0004040.0005940.000349

8e-065.4e-055e-06

8e-065.4e-055e-06

1e-054.1e-051.7e-05

5e-061.5e-055e-06

5e-062.6e-051.2e-05

0.0005910.0004690.0004140.0016050.0003090.0005720.000349

2e-064e-062e-06

0.000570.0004690.0004140.0016050.0002390.0005520.000349

1e-068e-061e-06

2e-066e-063e-06

1e-064e-061e-06

2e-061e-052e-06

6e-061.1e-056e-06

7e-062.7e-055e-06

0.0009840.0006380.0005690.0018050.0009970.0009390.000248

3.6e-056.7e-054.2e-05

00

1.7e-052.3e-051.8e-05

1.1e-058e-061.2e-05

8e-067e-061.2e-05

2.9e-05

7e-064.8e-057e-06

7e-064.8e-057e-06

1e-055.5e-059e-06

1e-055.5e-059e-06

1.1e-054.1e-056e-06

1.1e-054.1e-056e-06

3e-062.7e-052e-06

3e-062.7e-052e-06

0.000910.0006380.0005690.0018050.0007450.0008680.000248

0.000910.0006380.0005690.0018050.0007450.0008680.000248

7e-061.4e-055e-06

7e-061.4e-055e-06

0.0207050.0294120.0336430.0276830.0222150.016390.04423

4.3e-055e-053.2e-05

4.3e-055e-053.2e-05

4.3e-055e-053.2e-05

0.001220.0010850.007890.006720.0034180.0009850.007322

0.0001460.0002720.000294

7.2e-05

7.4e-05

0.0002720.000294

0.000156

0.000156

1.6e-050.000212.2e-054.9e-05

4.9e-05

1.6e-050.000212.2e-05

1.9e-058.4e-051.9e-05

1.9e-058.4e-051.9e-05

3.2e-05

3.2e-05

0.0004560.0004520.000490.000135

0.00013

0.0004520.00049

0.0001775e-06

0.000279

0.0001942e-050.0058010.0004586.1e-050.006295

0.0001942e-050.0004586.1e-05

4e-06

4e-06

4e-06

0.0041480.004492

0.0007890.000855

0.0008640.000936

0.0002367.7e-050.0007790.0010010.0001420.000517

0.0003020.000327

0.0002117.7e-050.0004430.000116

2.5e-050.0002312.6e-05

0.0004770.000517

0.0007550.0003860.0005860.006720.0010190.000741

0.0007550.0002460.0005860.006720.0010190.000741

0.00014

3.9e-050.0001453.8e-05

3.9e-050.0001453.8e-05

3.9e-050.0001453.8e-05

2.1e-050.0012280.0014021.9e-053.3e-05

0.0003180.000345

0.0003180.000345

0.000910.000986

0.000910.000986

2.1e-057.1e-051.9e-05

2.1e-057.1e-051.9e-05

3.3e-05

3.3e-05

0.0112850.0146440.0105260.0093280.0059710.0108350.020021

6.3e-050.0001216.2e-05

6.3e-050.0001216.2e-05

3.3e-05

3.3e-05

6.5e-050.0001520.0002856.2e-05

5.1e-054.8e-05

0.000152

0.000179

1.4e-050.0001061.4e-05

5.7e-050.0001165.5e-05

5.7e-050.0001165.5e-05

0.001310.0021130.00908

0.001310.0021130.00908

0.01110.0131820.0084130.0093280.0054490.0106560.010908

0.0110730.0131820.0084130.0093280.0050180.0106260.010908

2e-052.3e-052e-05

4e-062.5e-056e-06

0.000112

0.000214

3e-065.7e-054e-06

0.000159

0.000159

0.000159

0.0001870.0006020.0001920.000114

0.0001750.0005420.0001760.000114

0.0001270.0004620.00013

0.000114

4.8e-058e-054.6e-05

1.2e-056e-051.6e-05

1.2e-056e-051.6e-05

0.0014410.0008980.0058420.0051150.0056670.0014220.001192

1.1e-050.0001061.4e-05

5e-065.3e-057e-06

6e-065.3e-057e-06

1.5e-053.1e-051.5e-05

6e-062e-066e-06

1e-06

9e-062.8e-059e-06

0.0014150.0008980.0058420.0051150.0054850.0013930.001192

7e-06

2.4e-052.5e-052.6e-05

1.2e-052.7e-051.4e-05

8e-068e-061.1e-05

3.3e-053.3e-053.4e-05

6.3e-05

2.4e-05

9e-063.3e-059e-06

1.7e-052.4e-051.9e-05

0.0009720.0005630.0007390.0017050.001160.000940.000459

1.6e-057e-062.3e-05

0.0001940.0003350.0002350.0016050.0001860.0001870.000248

0.0005440.0007510.000485

5.8e-05

3e-050.0016670.0018056e-063.1e-05

2.3e-05

2.1e-051.6e-051.4e-05

4.9e-05

0.0026570.002878

3.3e-051.9e-053.4e-05

1.4e-054.4e-051.5e-05

1e-051e-061e-05

2.2e-052.4e-052.6e-05

1.9e-05

4.5e-05

4.5e-05

3.7e-059e-052.5e-05

3.7e-059e-052.5e-05

3.7e-059e-052.5e-05

3.8e-055.4e-052.8e-05

3.8e-055.4e-052.8e-05

3.8e-055.4e-052.8e-05

0.0062540.0127850.0081570.006520.0039690.0026970.015548

6.3e-050.0001426.1e-05

6.3e-050.0001426.1e-05

0.0061690.0127850.0048880.006520.0036460.0026170.012008

0.0061690.0127850.0048880.006520.0036460.0026170.012008

2.2e-050.00011.9e-05

2.2e-050.00011.9e-05

8.1e-05

8.1e-05

0.0032690.00354

0.0032690.00354

0.0001280.0005840.000108

1e-055.1e-059e-06

1e-055.1e-059e-06

9e-06

9e-06

1.6e-059.2e-051.6e-05

9e-063.2e-059e-06

6e-063.2e-055e-06

1e-062.8e-052e-06

1.3e-050.0001271.5e-05

7e-062.3e-057e-06

2e-061.6e-053e-06

5.2e-05

4e-063.6e-055e-06

1.4e-056.6e-051.2e-05

1.4e-056.6e-051.2e-05

01.7e-050

000

1.5e-05

2e-06

5.5e-050.0001854.6e-05

1.3e-052.8e-051.3e-05

1.5e-054.7e-052e-05

8e-065.1e-056e-06

9e-06

5e-062.8e-055e-06

5e-063.1e-052e-06

1.1e-054.6e-051e-05

1.1e-054.6e-051e-05

1.2e-050.0001049e-06

1e-054.9e-058e-06

1e-054.9e-058e-06

2e-065.5e-051e-06

2e-065.5e-051e-06

6.7e-05

6.7e-05

6.7e-05

6.7e-05

6.7e-05

0.1243460.1823130.1232080.1802420.0724970.1112960.170305

0.1013760.1581960.1022630.1447360.0502670.0966770.135303

0.0532170.0544230.0385860.0509530.0235220.0448380.040939

1.2e-050.0020370.0022078.1e-052.8e-05

5e-060.0020370.0022072e-052.1e-05

7e-061.9e-057e-06

4.2e-05

0.0532050.0544230.0365490.0487460.0234410.044810.040939

8.7e-057.3e-058.9e-05

6.2e-05

0.0087080.0081240.005120.0050150.0028960.008396

3.7e-052.3e-050.0023075.4e-050.00012

3.2e-05

7.5e-05

0.0001214.3e-050.0001235.3e-05

0.0106930.0056070.004870.0036110.0066280.0103610.00293

2.2e-05

7.7e-053.3e-057.6e-053.8e-05

3.1e-054e-063.4e-05

0.0025030.0012360.001110.0018050.0005980.00240.000877

0.0005790.000627

0.0005110.0010870.0005650.0019060.0003110.0004910.000861

1.8e-05

7.1e-05

5e-062.1e-052e-065e-061.8e-05

0.000110.0004560.0002830.0018056.3e-059.3e-050.000809

0.0263830.0308760.0143960.0139420.0067660.0171180.027348

8.4e-05

0.0002070.000224

2.6e-051e-052.7e-051.8e-05

1.8e-054e-061.8e-05

8e-060.001760.0019062.6e-052.2e-05

0.0011470.001243

0.0003050.0004040.0069215.2e-050.0002915.1e-05

0.000440.0006140.0004040.0017050.0002170.000426

7e-066e-061e-067e-06

0.0001020.0001320.000108

0.0013350.002660.0019280.0024070.001760.0029990.002323

3.5e-05

0.0020.0020430.002477

6.5e-052.1e-056.2e-05

8.1e-050.0003423.5e-058.2e-050.000371

8.2e-057.9e-057.8e-05

8.2e-05

2.4e-052.6e-056.9e-050.000264

0.00030.0001710.0002950.000341

4.5e-051e-054.4e-05

0.0004080.0015390.0006230.0021069.6e-050.000310.001631

8.8e-05

0.0006930.0001740.0002980.003310.0003470.0006660.000148

2.3e-05

0.000470.000509

4e-05

9.5e-05

3.4e-05

0.0038720.0023290.0041820.0072220.0015710.0037220.00222

0.0001280.0001350.0001330.0015050.0001110.0001240.000116

0.0001280.0001350.0001330.0015050.0001110.0001240.000116

0.0037290.0021940.0040490.0057170.0014330.0035840.002104

9.9e-050.00020.0003290.0016053.6e-059.5e-059.8e-05

0.0002840.000307

3.5e-054e-053.5e-05

1e-05

2.5e-051.4e-052.5e-05

0.0035560.0019940.0018620.0024070.0010140.0034190.002006

1.4e-050.0015740.0017051.2e-051e-05

1.5e-052.7e-051.4e-05

1.5e-052.7e-051.4e-05

0.0415760.1007060.0548540.0804420.0222920.045540.091394

9.5e-050.004630.0050150.0001048.8e-05

7e-050.004630.0050153.9e-056.3e-05

2.5e-054e-052.5e-05

2.5e-05

0.0414810.1007060.0502240.0754270.0221880.0454520.091394

8.7e-057.3e-058.8e-05

9e-05

2.3e-059.3e-051.8e-05

0.0041930.009820.0025450.0060180.000770.0040350.008028

2.3e-05

5e-053.9e-050.0019450.0021061.9e-055.1e-05

1.6e-05

3.9e-053.3e-054.1e-05

1.6e-05

0.0010870.001178

5.2e-05

9e-056.3e-050.0032410.0035115.1e-058.4e-05

2.6e-05

5.5e-05

3e-050.0035190.0038115.7e-052.7e-05

0.0258620.0653310.0265150.0361080.014250.024820.05956

6.4e-050.0001680.0016051.1e-056e-050.000109

2.6e-05

0.0015190.0005660.0005550.0051150.0001560.0015210.003475

9.7e-05

0.000320.0077030.0005080.0035110.0002580.0003090.003921

6.9e-050.0001197.5e-05

0.000203

0.0007730.0009630.0007350.0019060.0003980.0007450.001221

5.9e-056.2e-051.6e-05

7.7e-056.3e-050.0013890.0015054.5e-057.7e-059.6e-05

0.0024490.0050890.0032690.0036110.0019980.0078840.005391

9.3e-05

0.0057020.0109010.0035690.006620.0012020.0054770.008362

0.0013470.001459

6.6e-05

0.000148

6.7e-05

9.8e-05

2.5e-053.4e-052.6e-05

4e-063.8e-054e-062.1e-05

4.6e-056.4e-054.8e-05

0.0013420.0007380.0036070.0061190.0015450.0012870.000734

2.4e-055.6e-052e-05

2.4e-055.6e-052e-05

0.0005980.0003660.0004420.0016050.0006230.0005710.000251

4.2e-05

1.3e-05

0.0005980.0003660.0004420.0016050.0005680.0005710.000251

0.000720.0003720.0031650.0045140.0008660.0006960.000483

1.6e-051.5e-051.2e-05

9e-060.0013890.0015051.5e-051.1e-05

0.000640.0003720.000480.0016050.0005180.000620.00032

1.4e-051.9e-051.2e-05

3.4e-052.1e-053.4e-05

0.0002710.000163

7e-060.0012960.0014047e-067e-06

0.0013150.0010340.0010810.0012521.6e-05

0.0013150.0010340.0010810.0012521.6e-05

1.6e-05

5.2e-05

2.8e-054.6e-052.8e-05

0.0012570.0010340.0009490.001207

3e-053.4e-051.7e-05

5.4e-050.0002563.8e-05

5.4e-050.0002563.8e-05

5.4e-053.8e-053.8e-05

2.1e-05

7.9e-05

5.7e-05

7e-06

5.4e-05

0.022970.0241170.0209450.0355060.022230.0146190.035002

0.0009460.0008970.0010430.0019060.0021330.0018170.000634

0.0009420.0008970.0010430.0019060.002070.0018130.000634

0.0009420.0008970.0010430.0019060.002070.0018130.000634

4e-066.3e-054e-06

4e-066.3e-054e-06

3.2e-056.1e-052.5e-05

3.2e-056.1e-052.5e-05

3.2e-056.1e-052.5e-05

0.005040.002790.0047030.0031090.0073790.0048520.001166

0.0050010.002790.0033460.0031090.004840.0048178.5e-05

1.6e-05

5.9e-055.4e-051.6e-052.9e-054.1e-05

6.3e-05

5.1e-05

1.4e-05

3.5e-05

1.6e-057.2e-051.7e-05

1e-05

4e-06

7e-06

9e-06

6.9e-05

2.3e-05

2.3e-05

4.5e-05

6e-062.6e-055e-06

1.1e-05

1.3e-05

9e-05

2.9e-055.6e-052.6e-05

8.4e-05

5e-06

2.2e-05

0.0001444.2e-059.1e-050.0001764.4e-05

1.5e-05

5.8e-05

2.1e-05

0.0047470.0026940.0033460.0031090.0038910.004564

0.000117

0.000117

1.6e-054.9e-051.5e-05

1.6e-054.9e-051.5e-05

2.3e-050.0013570.0023732e-050.001081

2.3e-057.9e-052e-05

0.0013570.0022940.001081

4.4e-050.0006170.0014154.8e-053.3e-05

0.000352

3e-05

5e-05

5.1e-05

2.2e-05

6.1e-05

5.5e-05

2.7e-05

2.8e-05

2.8e-05

4.4e-057.6e-054.8e-05

4.4e-057.6e-054.8e-05

0.0002890.000313

0.0002890.000313

0.0003280.0003553.3e-05

0.0003280.000355

3.3e-05

0.000164

0.000164

0.000155

0.000155

0.0004425e-060.0031110.0021060.0006020.0004640.001262

0.0003975e-060.0031110.0021060.0004090.000430.001262

1.7e-051.2e-051.9e-05

2.4e-052.5e-052.7e-05

0.0011660.001262

2e-065e-060.0019450.0021065e-061.2e-05

3.6e-05

3.4e-051.4e-053.6e-05

3.5e-051.9e-053.8e-05

1e-06

9e-06

0.0001540.0001580.000161

5.9e-05

1.3e-05

4.7e-057e-064.7e-05

6e-06

1.2e-059e-061.5e-05

1.2e-05

3.1e-053e-063.3e-05

4.1e-059e-064.2e-05

4e-06

8e-06

4.5e-058.8e-053.4e-05

4.5e-058.8e-053.4e-05

0.000105

0.000105

0.0159820.0203210.0111840.0263790.0086990.0068980.031891

1.3e-050.000121.6e-05

1.3e-050.000121.6e-05

0.000101

0.000101

0.0134840.0190250.00930.0213640.0050610.004420.031794

0.0012350.0053980.0004880.0024070.0001090.0017260.006186

4.9e-050.0002435.9e-05

0.0004330.0058260.0007510.0048141.8e-050.0004210.000922

3.3e-05

3.7e-051.7e-055.2e-05

1e-053e-061.7e-05

5.5e-05

2.5e-050.0001233.8e-05

3.8e-053e-054.7e-05

0.000260.000281

1.7e-05

0.0003390.000367

0

0.0003070.000332

2.5e-059.2e-052.8e-05

0.0002220.000241

0.0005640.000611

0.0006620.0001890.0004260.0028088.3e-050.000661

0.0001831.8e-050.000201

0.0002940.000319

8.4e-05

0.0001950.0017740.0004480.0020062.1e-050.000260.003707

0.000141e-060.000202

7e-06

0.0097880.0045310.0016250.0035110.0004860.005671

0.0001615.2e-050.0003120.000171

2e-06

1.1e-05

9.4e-055e-069.5e-05

0.000207

2.1e-054.5e-052.5e-05

7.4e-05

2e-06

0.000135.6e-050.0019061.4e-050.000130.013506

0.0002380.000258

1e-05

5.5e-050.0011990.0014950.0022078e-065.7e-050.001769

0

4.5e-050.0015740.0017051e-054.6e-05

7.2e-054.7e-058.1e-05

0.0002690.000291

3.9e-055.4e-055.4e-05

4.7e-050.0001184.9e-05

3e-05

5e-06

0.000131

0.000131

0.0001040.0002580.00011

0.0001045.7e-050.00011

0

8.9e-05

0.000112

0.000175

9.8e-05

7.7e-05

0.0010420.0006450.0007630.0017050.000710.0010191.5e-05

0.0009480.0006450.0007630.0017050.0005660.000916

7e-061e-065e-06

1e-066e-063e-063e-06

6e-06

1e-05

2.1e-05

2e-06

3e-063e-063e-063e-06

3.8e-052.9e-053.8e-05

03e-06

2e-06

1e-064e-061e-063e-06

3e-06

6e-065e-068e-06

3.8e-055.5e-054.5e-05

0.000164

3.5e-05

0.00012

9e-06

0.0007150.0002350.0005450.0017050.0011450.0007348.2e-05

0.0007150.0002350.0005450.0017050.0011450.0007348.2e-05

0.000116

0.000116

0.0006150.0004160.0005760.0016050.0005980.000591

0.0006150.0004160.0005760.0016050.0005980.000591

9e-067e-068e-06

9e-067e-068e-06

0.000113

0.000113

4.6e-051e-063.8e-056e-05

4.6e-051e-063.8e-056e-05

1.6e-059e-062.2e-05

3e-063e-066e-06

4e-066e-065e-06

2.2e-051.9e-052.6e-05

1e-061e-061e-061e-06

0.0004380.0001030.0002870.0020060.0019030.0004551.6e-05

0.0004380.0001030.0002870.0020060.0018340.0004551.6e-05

1.9e-052.3e-052.1e-05

2.7e-051.7e-052.7e-05

8e-06

2.4e-052e-052.5e-05

0.0003680.0001030.0002870.0020060.0017660.0003821.6e-05

6.9e-05

6.9e-05

0.0028760.0014720.0207980.0036110.0105380.0027510.014551

0.0025140.0014720.0018840.0036110.0023940.0024140.001581

0.0025140.0014720.0018840.0036110.0023940.0024140.001581

0.0025140.0014720.0018840.0036110.0023940.0024140.001581

0.0022880.0013470.0016910.0021060.0021030.0021940.00145

0.0002260.0001250.0001930.0015050.0002910.000220.000131

0.0003020.0186420.0077720.0002810.012675

0.0003020.0180280.0070150.0002810.012675

0.0003020.0180280.0070150.0002810.012675

0.0005780.000626

0.0049850.005399

0.0117030.012675

0.0005530.000599

7e-050.0001427.8e-05

0.0002320.0002090.0002490.000203

0.0006140.000757

0.0006140.000757

9.2e-05

0.0006140.000665

6e-050.0002720.0003725.6e-050.000295

6e-050.0002720.0003725.6e-050.000295

0.000103

0.000103

2.4e-058.2e-052.4e-05

2.4e-058.2e-052.4e-05

3.6e-050.0002720.0001873.2e-050.000295

3.6e-050.0001873.2e-05

0.0002720.000295

0.0002160.0079460.0018460.0002120.00707

0.0002160.0079460.0018460.0002120.00707

0.0002160.0079460.0018460.0002120.00707

8.5e-05

8.5e-05

8e-067.3e-057e-06

5.8e-05

8e-061.5e-057e-06

0.0002080.000205

0.0002080.000205

0.000152

0.000152

0.0079460.0015360.00707

0.0014180.001536

0.0065280.00707

0.0007020.0003270.0051770.0047140.0015060.0006720.005099

0.0007020.0003270.0051770.0047140.0015060.0006720.005099

0.0007020.0003270.0051770.0047140.0015060.0006720.005099

0.000103

0.000103

0.0004050.0002030.0049320.0027080.0010090.0003930.005067

0.000167

0.0004050.0002030.0004080.0027080.0010090.000393

0.0045240.0049

9.2e-05

9.2e-05

0.0002970.0001240.0002450.0020060.0003020.0002793.2e-05

0.0002970.0001240.0002450.0020060.0003020.0002793.2e-05

0.0061220.0049730.0052750.0082250.0054580.0059510.003589

0.0061220.0049730.0052750.0082250.0053950.0059510.003589

0.0028220.0015610.0016870.0021060.0027210.0026930.000968

0.0028220.0015610.0016870.0021060.0027210.0026930.000968

0.0027780.0015610.0016870.0021060.0026750.0026570.000968

8e-064.3e-058e-06

3e-054.5e-051.6e-05

0.002740.0015610.0016870.0021060.0025570.0026330.000968

3e-05

4.4e-054.6e-053.6e-05

2e-061e-062e-06

3.8e-052.4e-053.2e-05

0

3e-061.4e-051e-06

1e-067e-061e-06

0.0032450.0034120.0035880.0061190.0025710.0032040.002621

0.0032450.0034120.0035880.0061190.0025710.0032040.002621

0.0005640.00180.0018970.0040130.0005760.0004920.001471

6e-06

2.3e-054e-062.2e-05

1.3e-051.2e-052e-06

2e-062e-06

4e-06

9e-063e-069e-06

2e-062e-062e-06

8e-061e-067e-06

07e-061e-06

1.8e-05

9e-060

07e-06

2.2e-051.1e-051.7e-05

0.0003490.00180.0005080.0025080.0003190.0003070.001453

2e-06

02e-061e-06

3e-063e-063e-06

2.2e-05

8e-061.1e-058e-06

3e-061e-063e-06

3.5e-056e-063.5e-05

8e-062.5e-058e-06

2.2e-051.4e-051.6e-05

01.3e-051e-06

3.1e-054e-061.5e-05

1e-062e-061e-06

1.3e-050.0013890.0015051e-051.3e-051.8e-05

2e-05

4e-06

4e-061e-06

2e-063e-062e-06

1e-061e-061e-06

01e-067e-06

1.6e-05

2e-06

5e-061.1e-058e-06

0.0026810.0016120.0016910.0021060.0019950.0027120.00115

0.0026030.0016120.0016910.0021060.0018980.0024980.00115

7.8e-059.7e-050.000214

5.5e-050.0001035.4e-05

4e-061.4e-054e-06

4e-061.4e-054e-06

4e-061.4e-054e-06

5.1e-058.9e-055e-05

5.1e-058.9e-055e-05

3e-06

1.3e-05

2.3e-055e-062.5e-05

1e-061.3e-051e-06

8e-068e-065e-06

1.2e-05

1e-052e-061e-05

9e-068e-069e-06

1e-05

9e-06

6e-06

6.3e-05

6.3e-05

6.3e-05

6.3e-05

6.3e-05

1.7e-050.0001961.1e-05

7.7e-05

7.7e-05

7.7e-05

7.7e-05

7.7e-05

1.1e-057.8e-051e-05

1.1e-057.8e-051e-05

1.1e-057.8e-051e-05

1.1e-057.8e-051e-05

1.1e-057.8e-051e-05

6e-064.1e-051e-06

6e-064.1e-051e-06

6e-064.1e-051e-06

6e-064.1e-051e-06

6e-064.1e-051e-06

0.2928220.240960.1842560.2293920.2434360.2935110.213011

4.3e-050.0001283.3e-05

4.3e-050.0001283.3e-05

4.3e-050.0001283.3e-05

4.3e-050.0001283.3e-05

5.8e-05

4.3e-057e-053.3e-05

0.0002060.0006020.00016

0.0001010.0002998e-05

0.0001010.0002998e-05

9e-062.9e-055e-06

9e-062.9e-055e-06

1.6e-059e-06

1.6e-059e-06

1.5e-057.1e-051.5e-05

1.5e-057.1e-051.5e-05

2.9e-054.1e-052.5e-05

2.9e-054.1e-052.5e-05

5.3e-05

5.3e-05

3.2e-050.0001052.6e-05

1e-055.8e-058e-06

2.2e-054.7e-051.8e-05

0.0001050.0003038e-05

8.5e-050.0001716.6e-05

2.8e-050.0001112.4e-05

5.6e-05

2.8e-055.5e-052.4e-05

5.7e-056e-054.2e-05

5.7e-056e-054.2e-05

2e-050.0001321.4e-05

2e-058.4e-051.4e-05

2e-058.4e-051.4e-05

4.8e-05

4.8e-05

0.2922510.2409310.1839770.2293920.2421680.2930240.212994

4.7e-055.9e-054e-054.3e-05

4.7e-055.9e-054e-054.3e-05

4.7e-055.9e-054e-054.3e-05

4.7e-055.9e-054e-054.3e-05

0.2169540.1280980.1213050.0788370.1856340.2082320.099499

0.2169540.1280980.1213050.0788370.1856340.2082320.099499

7.9e-050.0001757.7e-051.6e-05

7.9e-050.0001757.7e-051.6e-05

0.0429140.0242830.0236730.0171520.0330660.0412320.021204

0.0420930.0240050.0230550.0139420.032160.0404250.02114

0.0008210.0002780.0006180.003210.0009060.0008076.4e-05

3.5e-055.7e-053.6e-05

2.3e-053.1e-052.3e-05

1.2e-052.6e-051.3e-05

0.1739260.1038150.0976320.0616850.1523360.1668870.078279

3.8e-050.0001523.7e-05

0.1738070.1038150.0972470.0616850.1515920.1667720.078279

4e-050.0001753.8e-05

4.1e-050.0003850.0004174e-05

5.7e-057.3e-055.1e-05

5.7e-057.3e-055.1e-05

5.7e-057.3e-055.1e-05

5.7e-057.3e-055.1e-05

0.0001240.0002560.000117

0.0001240.0002560.000117

5.6e-05

5.6e-05

0.0001240.00020.000117

0.0001240.00020.000117

0.0040510.0018260.0033010.0065210.0052580.0038460.002662

4.9e-050.000154.3e-051.6e-05

4.9e-05

4.9e-05

4.9e-055.2e-054.3e-051.6e-05

4.9e-055.2e-054.3e-051.6e-05

4.9e-05

4.9e-05

5e-058.5e-054.8e-05

3.4e-05

3.4e-05

5e-055.1e-054.8e-05

5e-055.1e-054.8e-05

0.000960.0003930.0005010.003010.0011330.000890.000358

3.7e-052.2e-053.1e-05

3.7e-052.2e-053.1e-05

6.2e-057.2e-055.3e-05

2.5e-05

3.3e-052.2e-052.7e-05

2.9e-052.5e-052.6e-05

5.6e-05

5.6e-05

0.000390.0002230.0003020.0015050.0004810.0003730.000219

7e-06

2.3e-05

1.8e-05

0.0003870.0002230.0003020.0015050.00030.000370.000219

3.6e-05

2.8e-05

2e-05

2.1e-05

5e-06

1.7e-05

3e-066e-063e-06

6.3e-050.0001525.1e-051.6e-05

6.3e-054.4e-055.1e-051.6e-05

5e-05

5.8e-05

0.0002490.000170.0001990.0015050.0002050.0002390.000123

0.0002490.000170.0001990.0015050.0002050.0002390.000123

8.8e-058.4e-057.6e-05

6.5e-056.1e-055.6e-05

2.3e-052.3e-052e-05

5e-068e-065e-06

5e-068e-065e-06

6.6e-055.3e-056.2e-05

6.6e-055.3e-056.2e-05

2e-061.2e-051e-06

2e-061.2e-051e-06

2e-061.2e-051e-06

0.0010820.0003930.000779

0.0010820.0003930.000779

0.0007190.000779

0.0003630.000393

5e-056.2e-054.8e-051.5e-05

5e-056.2e-054.8e-051.5e-05

5e-056.2e-054.8e-051.5e-05

0.002160.0011790.001460.0019060.0019620.002070.001113

0.0020880.0011790.001460.0019060.0019020.0020030.001113

0.0020880.0011790.001460.0019060.0018380.0020030.001113

6.4e-05

7.2e-056e-056.7e-05

7.2e-056e-056.7e-05

1.1e-053.8e-058e-06

1.1e-053.8e-058e-06

1.1e-053.8e-058e-06

0.0004540.0002540.0002580.0016050.0010610.0004380.00029

5e-05

5e-05

4.8e-05

4.8e-05

3.3e-05

3.3e-05

4.4e-05

4.4e-05

3.2e-05

3.2e-05

4.5e-052.3e-054.3e-05

4.5e-052.3e-054.3e-05

0.0002870.0002540.0002580.0016050.0001580.0002740.00029

0.0002870.0002540.0002580.0016050.0001580.0002740.00029

0.0001220.0005790.000121

7e-05

0.0001220.0001070.000121

0.000101

0.000129

0.000103

6.9e-05

5.1e-05

3.9e-05

1.2e-05

4.3e-05

4.3e-05

0.0001940.0001630.0001851e-05

0.0001940.0001630.0001851e-05

7.2e-056e-056.8e-05

5.9e-054.5e-055.7e-05

6.3e-055.8e-056e-051e-05

6e-050.0001455.6e-056.6e-05

4.7e-05

4.7e-05

5.1e-05

5.1e-05

6.6e-05

6.6e-05

6e-054.7e-055.6e-05

6e-054.7e-055.6e-05

6.1e-055.4e-055.9e-051.5e-05

6.1e-055.4e-055.9e-051.5e-05

6.1e-055.4e-055.9e-051.5e-05

0.0002630.0004040.000245

0.0001580.0001470.000148

2.7e-051.3e-052.6e-05

2e-062e-062e-06

2.5e-051.1e-052.4e-05

3.9e-051.9e-054e-05

3.9e-051.9e-054e-05

2.6e-053.6e-052.2e-05

2.6e-053.6e-052.2e-05

3.2e-052.8e-053.2e-05

9e-06

3.2e-051.9e-053.2e-05

3.4e-055.1e-052.8e-05

3.4e-055.1e-052.8e-05

0.0001050.0002579.7e-05

4.9e-055.2e-054.6e-05

4.9e-055.2e-054.6e-05

5.5e-05

5.5e-05

5.6e-059.5e-055.1e-05

5.6e-055e-055.1e-05

4.5e-05

5.5e-05

5.5e-05

0.0016160.0007080.0010630.0017050.0016130.0015620.000616

0.0013580.0006690.0008620.0017050.0011630.0013010.000568

3.8e-055.7e-053.1e-051.6e-05

3.8e-055.7e-053.1e-051.6e-05

0.001320.0006690.0008620.0017050.0011060.001270.000552

3.5e-055e-053.3e-05

0.0012850.0006690.0008620.0017050.0010560.0012370.000552

7.9e-058.1e-057.8e-05

7.9e-058.1e-057.8e-05

7.9e-058.1e-057.8e-05

0.0001793.9e-050.0002010.0003690.0001834.8e-05

0.0001793.9e-050.0002010.0003690.0001834.8e-05

0.0001793.9e-050.0002010.0003690.0001834.8e-05

1.5e-055e-051.3e-05

1.5e-055e-051.3e-05

1.5e-055e-051.3e-05

1.5e-055e-051.3e-05

0.0004120.0001530.0002580.0015050.0004990.00040.000111

0.0004120.0001530.0002580.0015050.0004990.00040.000111

0.0004120.0001530.0002580.0015050.0004990.00040.000111

2.1e-054.5e-052.1e-05

3.5e-056.2e-053.6e-05

2.5e-056.2e-052.6e-05

0.0002890.0001530.0002580.0015050.0002750.0002799.6e-05

4.2e-055.5e-053.8e-051.5e-05

0.0174520.0101580.0106330.0075230.0167370.0167330.008943

0.0174520.0101580.0106330.0075230.0167370.0167330.008943

0.0174310.0101580.0106330.0075230.0164970.0167220.008943

0.0174310.0101580.0106330.0075230.0164970.0167220.008943

1.1e-05

1.1e-05

2.1e-054e-051.1e-05

2.1e-054e-051.1e-05

0.000141

3.3e-05

3.6e-05

3.9e-05

3.3e-05

4.8e-05

4.8e-05

0.000160.0001690.0001596.2e-05

0.000160.0001690.0001596.2e-05

5.4e-055.4e-055.2e-056.2e-05

5.4e-055.4e-055.2e-056.2e-05

5.2e-055.4e-055.3e-05

5.2e-055.4e-055.3e-05

5.4e-056.1e-055.4e-05

5.4e-056.1e-055.4e-05

0.0063190.0035510.0043780.0029090.0063290.0060980.003331

0.0063190.0035510.0043780.0029090.0063290.0060980.003331

0.006270.0035510.0043780.0029090.0062750.0060510.0033

2.4e-05

1.6e-05

3e-064e-064e-06

3.5e-05

1.5e-05

3e-05

4.8e-05

1.7e-05

4e-053.6e-054.4e-05

2.5e-05

2.3e-05

2.6e-05

0.0057680.0035250.0035530.0029090.004550.0055410.002794

3e-061e-052e-06

3.8e-052.5e-053.8e-05

0.000340.000368

4.1e-054.2e-05

1.7e-05

2.3e-05

2.8e-051.3e-052.3e-052.9e-05

0.0001184.6e-05

0.0004850.000525

3.2e-05

3.8e-052.2e-054.3e-05

2.7e-05

2.1e-05

1.3e-05

2.7e-05

5.2e-051.4e-055.4e-05

3.6e-05

4.8e-05

2.2e-051.3e-051.9e-054.6e-05

3.7e-056e-053.9e-05

5.6e-05

3.9e-053.2e-054.2e-05

3e-05

4e-053.2e-05

5e-06

2.3e-05

3.2e-05

3.5e-05

2.8e-05

4.7e-058e-065e-05

1.6e-05

1.6e-05

4.3e-05

2.8e-051.9e-053e-05

5e-06

2.4e-05

3.8e-05

4.6e-051.6e-054.7e-05

4.9e-055.4e-054.7e-053.1e-05

4.9e-055.4e-054.7e-053.1e-05

3.6e-055.1e-053.4e-05

3.6e-055.1e-053.4e-05

3.6e-055.1e-053.4e-05

3.6e-055.1e-053.4e-05

0.0126070.0527020.0136610.0955870.0058630.0165840.055993

0.0126070.0527020.0136610.0955870.0058630.0165840.055993

0.001320.001410.0012420.0023070.0008870.0012690.001554

0.001320.001410.0012420.0023070.0008870.0012690.001554

0.0001054.4e-05

0.0001054.4e-05

7.3e-05

7.3e-05

0.0112870.0512920.0124190.093280.0047980.0153150.054395

2e-06

0.0105120.0505420.0120150.0422270.0042490.0145630.05413

2.1e-053.2e-052.4e-05

2.8e-054.6e-053.2e-05

1.3e-05

2.2e-05

1.4e-051.4e-051.3e-05

2.9e-05

6.5e-05

2e-062e-063e-06

1.2e-05

0.0004810.0005730.0004040.0510530.0001850.00046

2.6e-051e-052.3e-05

2e-06

0.0002030.0001779.3e-050.0001970.000265

4e-06

1.8e-05

3.8e-055.7e-053.5e-05

3.8e-055.7e-053.5e-05

3.8e-055.7e-053.5e-05

3.8e-055.7e-053.5e-05

0.0026990.0030470.0021090.0041120.0024120.0025990.001937

0.0026990.0030470.0021090.0041120.0024120.0025990.001937

0.0001974.2e-050.0002160.0004020.0001913.1e-05

0.0001974.2e-050.0002160.0004020.0001913.1e-05

7.8e-057.6e-05

7.8e-057.6e-05

8.6e-050.0001978.1e-051.5e-05

4.8e-05

0

2.9e-05

3.2e-05

4e-06

1e-06

8.6e-058.3e-058.1e-051.5e-05

0.0021990.0030050.0018930.0041120.0014480.0021180.001833

0.00015

0.0010420.0023880.001270.0024070.0009740.0010020.001833

0.0011570.0006170.0006230.0017050.0002910.001116

3.3e-05

5.3e-05

5.3e-05

7.3e-055.8e-057.1e-05

7.3e-055.8e-057.1e-05

5.5e-05

5.5e-05

7e-05

7e-05

6.4e-05

6.4e-05

6.6e-056.5e-056.2e-055.8e-05

6.6e-056.5e-056.2e-055.8e-05

0.0294010.0406880.0272690.0306930.0167040.0362760.039797

5.5e-056.2e-055e-05

5.5e-056.2e-055e-05

5.5e-056.2e-055e-05

0.0001160.0001010.000108

0.0001160.0001010.000108

0.0001160.0001010.000108

0.0030750.0019250.00140.0021060.0024430.0030620.001345

0.002260.0017570.0011440.0021060.0014330.0022380.001296

3.4e-05

0.0001667.1e-056.6e-050.000169

3.6e-05

2.8e-05

1e-05

5.9e-05

0.0001256.1e-052.9e-050.000133.3e-05

2.6e-05

0.0003770.0001340.0003770.000390.000132

0.0002960.0001073.2e-050.000299

0.0002730.000296

0.0002140.000231

0.0012960.0013840.0006570.0021060.0002090.001250.001131

0.0008150.0001680.0002560.001010.0008244.9e-05

0.0001840.0001790.0001841.8e-05

0.0002354e-050.0001010.0002373.1e-05

0.000119

0.0003960.0001280.0002560.0006110.000403

3.3e-05

3.3e-05

3.3e-05

0.0220910.0311210.0103230.0190580.0050080.0289990.023188

4.4e-050.0001453.6e-055e-06

4.4e-050.0001453.6e-055e-06

0.0220470.0311210.0103230.0190580.0048630.0289630.023183

1e-056e-061.1e-055.3e-05

4e-066e-065e-06

4.3e-05

3.2e-05

0.0162150.0253160.0048890.0122370.0011790.0202590.018862

0.0024080.0039290.0018790.003210.0006510.0054440.004055

4.3e-05

3.3e-05

8e-062e-067e-06

4.6e-056e-064.5e-053.8e-05

000

5.7e-054.8e-056.6e-05

0005.1e-05

5e-069e-065e-06

4.4e-052.1e-054.5e-052.3e-05

2e-062e-062e-06

4e-053.2e-054.1e-05

2.8e-05

3e-062e-063e-06

4.3e-05

4e-06

0.0029730.0017910.0021660.0021060.0024290.002856

4e-06

00

6.5e-05

2e-064e-062e-06

2.7e-051.6e-053e-05

0

6.5e-052.4e-057e-053.6e-05

5e-052.9e-055.6e-05

1.5e-058.5e-050.0013890.0015054e-061.3e-05

2.1e-05

5.5e-05

0

1.2e-05

5e-067e-063e-06

3.5e-05

3.3e-05

6.8e-050

00

0.0003410.0004640.0019060.0007890.000335.1e-05

0.0003410.0004640.0019060.0007890.000335.1e-05

1.7e-05

0.0004640.000503

1.9e-05

0.0001130.0001130.0001121.5e-05

0.0001148.9e-050.00011

0.0001140.0019063.7e-050.000108

4.7e-05

0.0037230.0076420.0150820.0076230.0082680.0037270.015213

0.0037230.0076420.0150820.0076230.0082680.0037270.015213

0.000147

0.000250.000271

7.2e-05

2.9e-05

0.000121

0.000142

0.0008190.000887

7.5e-055.2e-057.7e-05

7.7e-057.1e-057.6e-05

0.0003350.0002060.0002020.000326

4.3e-05

5.6e-05

1.2e-052.4e-052e-061.3e-05

0.0071470.007741

0.0001015.7e-055.2e-050.0001021.3e-05

3e-056.8e-050.002130.0023071.9e-053e-05

0.0002460.000267

0.000119

0.0002930.000318

0.0015150.001641

6.3e-055.8e-056.3e-05

6.6e-058.5e-057.1e-05

7.4e-05

0.000128

8.4e-05

0.00013

0.0011240.0063430.0009230.0053160.000270.0010790.007355

1e-059e-061.2e-05

8.3e-05

6.6e-05

0.0001670.000160.000175

6e-053.9e-055.7e-05

0.000290.000314

0.0001157e-050.0001131.5e-05

0.00014

0.000182

0.0006860.000743

0.000106

1.5e-05

0.0011160.000560.0007830.0008550.001154

0.0002250.0001360.000227

0.0001470.0003849.9e-050.000152

5.7e-059.3e-053.9e-05

5.7e-059.3e-053.9e-05

5.7e-059.3e-053.9e-05

2.4e-054.4e-051.9e-05

2.4e-054.4e-051.9e-05

3.3e-054.9e-052e-05

3.3e-054.9e-052e-05

0.0002652.9e-050.0002790.0004450.0002551.7e-05

0.0002652.9e-050.0002790.0004450.0002551.7e-05

0.0002652.9e-050.0002790.0004450.0002551.7e-05

0.0002652.9e-050.0002790.0004450.0002551.7e-05

0.0002652.9e-050.0002790.0004450.0002551.7e-05

0.0187240.0144670.0261340.0235720.0351070.0182850.012406

0.0040560.0024690.0039920.0025080.0055250.0038950.001816

0.0040560.0024690.0039920.0025080.0055250.0038950.001816

0.0040560.0024690.0039920.0025080.0055250.0038950.001816

0.0013810.001496

0.0007170.000776

0.0006640.00072

8.8e-05

8.8e-05

0.0040420.0024690.0026110.0025080.0038110.003880.001816

0.0040420.0024690.0026110.0025080.0038110.003880.001816

6.5e-05

6.5e-05

1.4e-056.5e-051.5e-05

1.4e-056.5e-051.5e-05

0.0047230.0025180.0073750.0116350.0092890.0046380.00378

0.000237

0.000141

0.000141

0.000141

9.6e-05

9.6e-05

9.6e-05

0.0047230.0025180.0073750.0116350.0090520.0046380.00378

0.0028170.0015450.0037470.0050150.0014590.0027050.001941

0.0002160.000234

0.0002160.000234

0.0028170.0015450.0035310.0050150.0012250.0027050.001941

5.6e-050.0015390.0026080.0012255.4e-05

0.0027610.0015450.0019920.0024070.0026510.001941

0.0004010.0006080.0003610.0017050.0005440.0003870.000511

0.00021

0.00021

0.0004010.0006080.0003610.0017050.0003340.0003870.000511

0.0003780.0006080.0003610.0017050.0001730.0003640.000511

2.3e-050.0001612.3e-05

0.0006350.0001440.0011050.0016050.0017550.0006610.00066

0.0006350.0001440.0011050.0016050.0017550.0006610.00066

0.0003610.0001440.0002230.0016050.0003810.0003680.00011

0.0004930.000534

1.6e-05

7.9e-050.0001578.3e-05

4.3e-050.0003890.0004215e-05

4.9e-050.0001625.3e-05

0.000209

6e-050.0002286.5e-05

4.3e-056.9e-054.2e-05

0.000128

8.8e-050.0005980.0010348.8e-051.6e-05

3e-050.0001742.5e-05

3e-055.7e-052.5e-05

0.000117

5.8e-050.0005980.000866.3e-051.6e-05

5.8e-050.0005980.0006486.3e-05

1.6e-05

0.000212

0.0007150.0002210.0002520.003310.0022660.0007290.000652

0.0007150.0002210.0002520.003310.0022660.0007290.000652

0.000127

2.8e-05

1.7e-05

1e-06

1.6e-05

6.8e-05

0.0003530.0001250.0002520.0016050.0002450.0003533.3e-05

2.9e-050.0001683.3e-05

0.000194

3.3e-05

5.7e-05

9e-05

0.0001129.6e-050.0017050.0001190.0001110.000397

0.0001410.0012110.000149

8e-050.0001148.3e-05

1e-050.0010330.0012441.2e-05

0.0010330.001119

0.0010330.001119

1e-050.0001251.2e-05

1e-050.0001251.2e-05

0.0002790.000302

0.0002790.000302

0.0002790.000302

1e-050.0002331e-05

1e-050.0002331e-05

1e-050.0002331e-05

4.7e-050.0002154.6e-05

4.7e-050.0002154.6e-05

4.7e-050.0002154.6e-05

0.0066990.0076950.012270.0072220.0148880.006610.00681

0.0066990.0076950.012270.0072220.0148880.006610.00681

2.2e-056.6e-052.1e-05

2.2e-056.6e-052.1e-05

2.2e-056.6e-052.1e-05

8.3e-05

8.3e-05

8.3e-05

0.0065070.0076950.012270.0072220.0144580.006420.00681

1.6e-057.2e-051.8e-05

1.6e-057.2e-051.8e-05

0.0003040.0003950.000348

4.5e-058.7e-055.2e-05

4.6e-053.1e-055.6e-05

7.6e-054.3e-058.4e-05

4.3e-059.2e-054.8e-05

9.4e-050.0001420.000108

0.000106

0.000106

1.6e-050.0001211.9e-05

1.6e-055.7e-051.9e-05

6.4e-05

0.0002770.0003

0.0002770.0003

2e-050.0001132.1e-05

2e-050.0001132.1e-05

0.0001960.000210.0003020.00021

0.0001960.000210.0003020.00021

6.4e-05

6.4e-05

1.7e-050.0002071.8e-05

1.7e-050.0001061.8e-05

0.000101

1.8e-058.5e-051.6e-05

1.8e-058.5e-051.6e-05

8.1e-05

8.1e-05

0.0002270.000452

0.0002270.000246

8.2e-05

0.000124

0.000111

2.1e-05

9e-05

0.0009480.001027

0.0009480.001027

8.3e-059.9e-058.8e-05

3.3e-052.9e-053.6e-05

5e-054.6e-055.2e-05

2.4e-05

3e-050.0001553.6e-05

3e-050.0001553.6e-05

2.1e-050.0001672.3e-05

2.1e-054.9e-052.3e-05

0.000109

9e-06

6.1e-050.0001086.8e-05

6.1e-050.0001086.8e-05

1.8e-056.4e-051.7e-05

1.8e-056.4e-051.7e-05

9e-05

9e-05

5.5e-059.3e-055.6e-05

5.5e-059.3e-055.6e-05

8.1e-05

8.1e-05

0.0065540.0070990.000144

0.003260.003531

0.0008470.000918

0.0016130.001747

0.000144

0.0008340.000903

3.6e-055e-054.3e-05

3.6e-055e-054.3e-05

6e-067.6e-057e-06

6e-067.6e-057e-06

0.0031980.0016470.0016260.0022070.0011940.0030990.001198

0.0031610.0016470.0011840.0022070.0004760.0030590.001148

0.0004420.000479

3.3e-05

1.7e-05

3.7e-050.000114e-05

0.000129

0.000157

6.8e-05

8.9e-05

9.4e-05

9.4e-05

2.9e-050.0001683.1e-05

2.9e-050.0001683.1e-05

0.0023030.0060480.0024280.0050150.0011940.0022140.005168

0.0023030.0060480.0024280.0050150.0011940.0022140.005168

3.7e-050.000153.6e-05

3.7e-050.000153.6e-05

4.3e-050.0002835.2e-05

4.3e-050.0002835.2e-05

0.0001550.0002160.000154

0.0001550.0002160.000154

1.5e-052.7e-051.5e-05

3e-051.8e-053.4e-05

2.9e-052.9e-053e-05

2.2e-054.7e-052.1e-05

1.4e-051.4e-051.9e-05

6e-062.1e-056e-06

3.3e-055e-052.3e-05

6e-061e-056e-06

1.5e-056.5e-051.5e-05

1.5e-056.5e-051.5e-05

1.5e-056.5e-051.5e-05

4.4e-050.0006044.8e-05

4.4e-050.0006044.8e-05

4.4e-050.0006044.8e-05

8e-060.000191e-05

8e-066.2e-051e-05

4.5e-05

3.7e-05

4.6e-05

1.2e-057.5e-051.2e-05

1.2e-057.5e-051.2e-05

1.4e-050.0001021.5e-05

1.4e-050.0001021.5e-05

6.4e-05

6.4e-05

1e-050.0001731.1e-05

1e-058.1e-051.1e-05

9.2e-05

0.0031660.0017850.0024970.0022070.0045810.003062

0.0031660.0017850.0024970.0022070.0045810.003062

1.3e-050.0001391e-05

8e-066.8e-055e-06

8e-066.8e-055e-06

5e-067.1e-055e-06

5e-067.1e-055e-06

1.3e-053.7e-051.6e-05

1.3e-053.7e-051.6e-05

1.3e-053.7e-051.6e-05

0.0030680.0017850.0024970.0022070.0034430.002951

3.3e-053.2e-05

3.3e-053.2e-05

1.8e-058.9e-051.9e-05

1.8e-058.9e-051.9e-05

8e-060.0001068e-06

8e-060.0001068e-06

2e-055.5e-051.9e-05

2e-055.5e-051.9e-05

0.0002450.000265

0.0002450.000265

0.0029770.0017850.0022520.0022070.0028510.00286

0.0029770.0017850.0022520.0022070.0028510.00286

1.2e-057.7e-051.3e-05

1.2e-057.7e-051.3e-05

1.7e-050.0001582.3e-05

4.9e-05

4.9e-05

1.7e-055.8e-052.3e-05

1.7e-055.8e-052.3e-05

5.1e-05

5.1e-05

5.1e-05

5.1e-05

5.1e-05

5.5e-050.0004146.2e-05

9e-064.5e-051.1e-05

9e-064.5e-051.1e-05

3.9e-050.0002694.2e-05

0.000181

3.9e-058.8e-054.2e-05

4.5e-05

4.5e-05

7e-065.5e-059e-06

7e-065.5e-059e-06

0.000339

7.8e-05

3.9e-05

3.9e-05

7.9e-05

2.8e-05

2.6e-05

2.5e-05

0.000182

1.2e-05

3.1e-05

3.8e-05

3.2e-05

1.3e-05

2.3e-05

3.3e-05

9e-069.2e-051.3e-05

9e-069.2e-051.3e-05

3e-065.4e-054e-06

3e-065.4e-054e-06

3e-065.4e-054e-06

6e-063.8e-059e-06

6e-063.8e-059e-06

6e-063.8e-059e-06

2.7e-050.0001281.9e-05

2.7e-050.0001281.9e-05

2.7e-050.0001281.9e-05

2e-058.7e-051.3e-05

2e-058.7e-051.3e-05

7e-064.1e-056e-06

7e-064.1e-056e-06

5.5e-050.000333.1e-05

5.5e-050.000333.1e-05

2.5e-050.0002711.7e-05

1.4e-057.2e-051e-05

7e-064.2e-057e-06

3e-062.4e-054e-06

4e-061.8e-053e-06

7e-063e-053e-06

7e-063e-053e-06

6.7e-05

6.7e-05

6.7e-05

1.1e-050.0001327e-06

01e-050

01e-050

2e-065.2e-051e-06

2e-065.2e-051e-06

06.4e-05

03.9e-05

2.5e-05

9e-066e-066e-06

8e-066e-066e-06

1e-06

3e-055.9e-051.4e-05

3e-055.9e-051.4e-05

1e-062.1e-050

1e-062.1e-050

2.9e-053.8e-051.4e-05

2.9e-053.8e-051.4e-05

0.0063650.0012740.0470140.003210.0557120.0066420.000502

0.0063320.0012740.0470140.003210.0555690.0066090.000502

0.0002280.0003180.000235

0.0001060.0001680.000111

0.0001060.0001680.000111

0.0001060.0001680.000111

0.0001220.000150.000124

0.0001220.000150.000124

0.0001220.000150.000124

0.0011180.0006880.0003490.003210.0018410.0010940.000394

2e-050.0003061.9e-05

2e-050.0003061.9e-05

0.000147

2e-055.1e-051.9e-05

0.000108

2e-057.3e-051.6e-05

2e-057.3e-051.6e-05

2e-057.3e-051.6e-05

0.0005650.0002580.0003490.0016050.0005970.0005540.000163

0.0005650.0002580.0003490.0016050.0005970.0005540.000163

0.0005650.0002580.0003490.0016050.0005970.0005540.000163

0.0003180.0007380.000317

5.4e-058.5e-054.8e-05

3.9e-058.5e-053.4e-05

1.5e-051.4e-05

0.0001980.0005180.000216

7e-061.3e-051e-05

1.3e-056.7e-051.1e-05

1.2e-05

2e-05

2.6e-059e-062.8e-05

3e-06

2.3e-051e-062.3e-05

2.2e-058.7e-052.3e-05

4e-06

1.9e-05

1.5e-051.7e-051.5e-05

2e-051e-062.3e-05

2e-06

2e-06

6e-063.3e-058e-06

1.2e-051.6e-051.2e-05

2.3e-058.2e-052.6e-05

2.6e-050.00013.1e-05

7e-06

1.3e-05

5e-061e-056e-06

3.3e-058e-053.4e-05

3.3e-058e-053.4e-05

3.3e-055.5e-051.9e-05

3.3e-055.5e-051.9e-05

0.0001320.000430.0016050.0001120.0001270.000231

0.0001320.000430.0016050.0001120.0001270.000231

2.8e-05

0.0001320.000430.0016058.4e-050.0001270.000231

7e-061.5e-057e-06

7e-061.5e-057e-06

7e-069e-067e-06

4e-06

2e-06

5.6e-055.4e-05

5.6e-055.4e-05

5.6e-055.4e-05

0.0040020.0005860.0466650.0506830.0042413e-05

0.0009423.6e-050.0003070.0007390.001008

0.0006963.6e-050.0003070.0005810.000749

0.0002280.0001760.000241

0.000201

0.0004683.6e-050.0003070.0002040.000508

0.0002460.0001580.000259

0.0002460.0001580.000259

0.0449490.048684

0.0449490.048684

0.0449490.048684

0.003060.000550.0014090.001260.0032333e-05

0.0007290.0001710.0002630.0003450.0006711.5e-05

0.0001160.00010201.5e-05

0.0003616.9e-050.0002630.0001910.000396

0.0002526.2e-050.000275

9.2e-05

0.0002286.6e-050.000252

0.0002286.6e-050.000252

0.0003894.3e-050.0003810.000438

0.0003894.3e-050.0003810.000438

0.0017140.0003360.0007650.0008490.0018721.5e-05

0.0004369.4e-050.0002610.0001610.000478

0.0004860.0001030.0002120.000531

0.00015

0.0004317e-050.0002780.0001840.0004531.5e-05

0.0003616.9e-050.0002260.0001420.00041

3.6e-05

3.6e-05

3.6e-05

3.6e-05

0.0002680.0008690.000276

2.4e-055.2e-052.8e-05

2.4e-055.2e-052.8e-05

2.4e-055.2e-052.8e-05

4.5e-052.9e-054.9e-05

4.5e-052.9e-054.9e-05

1.3e-05

4.5e-051.6e-054.9e-05

6.6e-050.0001226.2e-05

3.1e-057.3e-053.5e-05

3.1e-057.3e-053.5e-05

3.5e-054.9e-052.7e-05

3.5e-054.9e-052.7e-05

0.0001330.0006660.000137

9e-05

4.5e-05

4.5e-05

0.0001330.0005760.000137

0.0001330.0005760.000137

0.0001560.0004780.000164

0.0001560.0004780.000164

0.0001560.0004780.000164

0.0001560.0004780.000164

0.000560.0013440.0005997.8e-05

9.4e-050.0002499.3e-05

9.4e-050.0002499.3e-05

5.8e-050.0001455.7e-05

3.6e-050.0001043.6e-05

0.0001230.000250.000128

3.4e-053.7e-05

3.4e-053.7e-05

2.5e-055.6e-052.7e-05

2.5e-055.6e-052.7e-05

6.4e-050.0001946.4e-05

6.4e-050.0001946.4e-05

2.1e-050.0001471.9e-05

2.1e-050.0001471.9e-05

2.1e-050.0001471.9e-05

0.0001930.0004470.0002137.8e-05

0.0001930.0004470.0002137.8e-05

2.9e-058.8e-053.5e-05

7.8e-05

1.7e-050.000122.1e-05

4e-061.3e-056e-06

4.7e-056.4e-055e-05

1.1e-057e-061.4e-05

8.5e-050.0001558.7e-05

0.0001290.0002510.000146

0.0001290.0002510.000146

0.0001290.0002510.000146

3.3e-050.0001433.3e-05

3.3e-050.0001433.3e-05

3.3e-050.0001433.3e-05

3.3e-050.0001433.3e-05

1.6e-056.7e-051.5e-05

1.7e-057.6e-051.8e-05

3e-064.6e-052e-06

3e-064.6e-052e-06

3e-064.6e-052e-06

3e-064.6e-052e-06

3e-064.6e-052e-06

3e-064.6e-052e-06

9.5e-050.0047490.0005258.2e-050.005144

4.5e-050.0001423.7e-05

3.8e-058.2e-053.1e-05

3.8e-058.2e-053.1e-05

3.8e-058.2e-053.1e-05

3.8e-058.2e-053.1e-05

7e-066e-056e-06

7e-066e-056e-06

7e-066e-056e-06

7e-066e-056e-06

1.5e-057.1e-051.1e-05

1.5e-057.1e-051.1e-05

3e-062.4e-052e-06

3e-062.4e-052e-06

0

1e-06

3e-062.3e-052e-06

1.2e-054.7e-059e-06

1.2e-054.7e-059e-06

5e-062.3e-053e-06

7e-062.4e-056e-06

0.0047490.005144

0.0047490.005144

0.0047490.005144

0.0047490.005144

0.0047490.005144

9e-060.0001931.4e-05

9e-060.0001931.4e-05

9e-060.0001931.4e-05

9e-063.5e-051.4e-05

9e-063.5e-051.4e-05

0.000158

0.000158

1.9e-056e-051.5e-05

3e-064e-064e-06

3e-064e-064e-06

3e-064e-064e-06

3e-064e-064e-06

0

1.6e-055.6e-051.1e-05

1.6e-055.6e-051.1e-05

1.6e-055.6e-051.1e-05

1.6e-055.6e-051.1e-05

7e-065.9e-055e-06

7e-065.9e-055e-06

7e-065.9e-055e-06

7e-065.9e-055e-06

7e-065.9e-055e-06

2.2e-050.0001612.8e-05

2.2e-050.0001612.8e-05

2.2e-050.0001612.8e-05

2.2e-050.0001612.8e-05

6e-063.8e-056e-06

6e-063.8e-056e-06

3e-063.6e-052e-06

3e-063.6e-052e-06

9e-064.3e-051.8e-05

9e-064.3e-051.8e-05

4e-064.4e-052e-06

4e-064.4e-052e-06

0.0053050.0076740.0044750.0074220.0031250.0097020.006606

0.0053050.0076740.0044750.0074220.0031250.0097020.006606

0.0038610.0050260.0034290.0050150.0018810.0083850.004079

0.0038610.0050260.0034290.0050150.0018810.0083850.004079

0.0038510.0050260.0034290.0050150.0018340.0083760.004079

0.0003790.000411

0.0024830.0027190.0020420.0026080.0010020.0067880.002413

1e-063e-051e-06

1.8e-05

0.0013670.0023070.0010080.0024070.0003730.0015870.001666

1e-054.7e-059e-06

1e-054.7e-059e-06

0.0001999.1e-050.0001713.3e-05

0.0001999.1e-050.0001713.3e-05

0.0001999.1e-050.0001713.3e-05

5.6e-054.9e-055.5e-053.3e-05

8.8e-0506e-05

5e-061.1e-055e-06

5e-053.1e-055.1e-05

0.0012450.0026480.0010460.0024070.0011530.0011460.002494

0.0011150.0026480.0010460.0024070.0005660.0010320.002494

7.7e-051.5e-053.9e-05

2e-06

2e-0600

1e-051e-05

4.3e-051e-05

1.7e-0502.4e-05

5e-062e-065e-06

1e-06

0.0010380.0026480.0010460.0024070.0005510.0009930.002494

5e-068e-065e-06

2.8e-059e-062.5e-05

0.0009610.0026480.0010460.0024070.0005050.0009230.002494

1e-063e-061e-06

1.5e-052e-061.5e-05

2.8e-052.4e-052.4e-05

0.000130.0005870.000114

6.6e-050.0003456.2e-05

4.7e-05

2.2e-05

3.1e-057.1e-053.1e-05

4e-065.5e-053e-06

1e-06

3e-062.9e-053e-06

3e-063.1e-053e-06

1.5e-051.5e-051.4e-05

1e-06

4e-063.2e-054e-06

6e-064.1e-054e-06

1.3e-053.7e-059e-06

1.3e-053.7e-059e-06

4e-063.2e-054e-06

4e-063.2e-054e-06

1.3e-055.6e-051.1e-05

6e-063e-055e-06

7e-062.6e-056e-06

3.4e-050.0001172.8e-05

5e-062.6e-055e-06

2.1e-053.4e-051.4e-05

8e-065.7e-059e-06

0.0014970.0008740.0010250.0018050.0013270.0014390.000637

0.0014970.0008740.0010250.0018050.0013270.0014390.000637

0.0014970.0008740.0010250.0018050.0013270.0014390.000637

0.0014970.0008740.0010250.0018050.0013270.0014390.000637

0.0014970.0008740.0010250.0018050.0013270.0014390.000637

1e-063.4e-052e-06

0.0014960.0008740.0010250.0018050.0012930.0014370.000637

3.6e-050.0003083.7e-05

3.6e-050.0003083.7e-05

3.6e-050.0003083.7e-05

3.6e-050.0003083.7e-05

1.2e-050.0002069e-06

1.2e-056.9e-059e-06

7.8e-05

5.9e-05

1.6e-055.7e-052.4e-05

1.6e-055.7e-052.4e-05

8e-064.5e-054e-06

2e-064e-061e-06

6e-063.7e-053e-06

4e-06

9.1e-050.0007587.9e-05

2.2e-050.0001931.9e-05

8e-065e-057e-06

8e-065e-057e-06

8e-065e-057e-06

8e-065e-057e-06

1.4e-050.0001431.2e-05

1.4e-050.0001431.2e-05

3.6e-05

3.6e-05

5.1e-05

5.1e-05

1.4e-055.6e-051.2e-05

1.4e-055.6e-051.2e-05

3.7e-050.0003953.7e-05

3.7e-050.0003953.7e-05

3.7e-050.0003953.7e-05

3.7e-050.0003953.7e-05

0.000207

3.7e-050.0001883.7e-05

3.3e-05

3.3e-05

3.3e-05

3.3e-05

3.3e-05

9.8e-05

9.8e-05

9.8e-05

9.8e-05

9.8e-05

3.2e-053.9e-052.3e-05

3.2e-053.9e-052.3e-05

3.2e-053.9e-052.3e-05

3.2e-053.9e-052.3e-05

3.2e-053.9e-052.3e-05

3e-064.2e-053e-06

4.2e-05

4.2e-05

4.2e-05

4.2e-05

4.2e-05

3e-063e-06

3e-063e-06

3e-063e-06

3e-063e-06

3e-063e-06

0.0103390.0210990.0186370.0334010.0071210.0100870.015241

0.0103390.0210990.0186370.0334010.0071210.0100870.015241

4e-066.4e-053e-06

4e-066.4e-053e-06

2e-063.5e-052e-06

1.7e-05

2e-061.8e-052e-06

2e-062.9e-051e-06

2e-062.9e-051e-06

0.0103180.0210990.0186370.0334010.0069650.0100630.015241

0.01030.0210990.0186370.0334010.0068820.0100480.015241

0.0022550.0042920.004830.0035110.0008920.0021880.003345

3e-062.7e-053e-06

8e-068e-068e-06

8e-060.0030884e-058e-060.003345

0.0022360.0042920.0017420.0035110.0008170.002169

0.0080450.0168070.0138070.029890.005990.007860.011896

0.0004310.0001540.0003280.0090276e-060.000431

7e-06

0.0040290.0125960.0055260.0064194.9e-050.0039060.01042

1.8e-05

8e-06

0.0009820.0012430.0005990.0052162.8e-050.0009760.000214

0.000910.0013240.0010860.0043131e-050.0008970.00095

0.0016930.001490.0008740.0049151e-050.001650.000312

1.2e-05

0.0053940.005842

1.8e-058.3e-051.5e-05

7e-064.1e-055e-06

5e-062.4e-054e-06

8e-06

2e-069e-061e-06

1.1e-054.2e-051e-05

4e-062.1e-053e-06

7e-062.1e-057e-06

1.7e-059.2e-052.1e-05

1.7e-059.2e-052.1e-05

2.9e-05

2.9e-05

1.3e-052.8e-051.2e-05

1.3e-052.8e-051.2e-05

4e-063.5e-059e-06

4e-063.5e-059e-06

0.0034870.0017990.0020480.0022070.0035680.0033580.001247

1.9e-055.3e-051.3e-05

1.9e-055.3e-051.3e-05

1.9e-055.3e-051.3e-05

1.9e-055.3e-051.3e-05

1.9e-055.3e-051.3e-05

0.0034680.0017990.0020480.0022070.0035150.0033450.001247

0.0034680.0017990.0020480.0022070.0035150.0033450.001247

9e-065.7e-051e-05

6e-062.8e-057e-06

6e-062.8e-057e-06

3e-062.9e-053e-06

3e-062.9e-053e-06

0.0034590.0017990.0020480.0022070.0034580.0033350.001247

0.0034450.0017990.0020480.0022070.0033070.0033220.001247

0.0034450.0017990.0020480.0022070.0033070.0033220.001247

7e-065.1e-056e-06

7e-065.1e-056e-06

4.1e-05

4.1e-05

2e-063.2e-053e-06

2e-063.2e-053e-06

5e-062.7e-054e-06

5e-062.7e-054e-06

2.6e-050.000211.2e-05

2.6e-050.000211.2e-05

2.6e-050.000211.2e-05

2.6e-050.000211.2e-05

5e-05

5e-05

6e-065.1e-054e-06

6e-065.1e-054e-06

5e-063.8e-051e-06

5e-063.8e-051e-06

5e-063.2e-051e-06

5e-063.2e-051e-06

1e-053.9e-056e-06

1e-053.9e-056e-06

1.7e-055.5e-051.2e-05

1.7e-055.5e-051.2e-05

1.7e-055.5e-051.2e-05

1.7e-055.5e-051.2e-05

1.7e-055.5e-051.2e-05

1.7e-055.5e-051.2e-05

3e-063.8e-052e-06

3e-063.8e-052e-06

3e-063.8e-052e-06

3e-063.8e-052e-06

3e-063.8e-052e-06

3e-063.8e-052e-06

2.3e-050.0001451.4e-05

2.3e-050.0001451.4e-05

2.3e-050.0001451.4e-05

2.3e-050.0001451.4e-05

8e-063.5e-057e-06

8e-063.5e-057e-06

3.5e-05

3.5e-05

1.5e-057.5e-057e-06

3.4e-05

1.5e-054.1e-057e-06

0.0001110.0003639.1e-05

0.0001110.0003639.1e-05

0.0001110.0003639.1e-05

0.0001110.0003639.1e-05

4.6e-050.0001113.5e-05

1.4e-052.5e-058e-06

1.9e-052.5e-051.1e-05

9e-062.8e-058e-06

4e-063.3e-058e-06

5e-066.3e-056e-06

5e-066.3e-056e-06

3.5e-055.6e-052.6e-05

1.3e-05

2.1e-051.8e-051.6e-05

1.4e-052.5e-051e-05

1.7e-054.9e-051.6e-05

3e-062.7e-059e-06

1.4e-052.2e-057e-06

8e-068.4e-058e-06

8e-063.4e-058e-06

5e-05

0.0021260.0115760.0038460.0536610.0022670.0020040.074133

0.0021260.0115760.0038460.0536610.0022670.0020040.074133

0.0021060.0115760.0038460.0536610.0021420.0019870.074133

0.0021060.0115760.0038460.0536610.0021420.0019870.074133

0.0021060.0115760.0038460.0536610.0021420.0019870.074133

1.9e-051.1e-052.1e-05

1e-063e-061e-06

6.5e-050.0003310.0004880.0016054.1e-055.5e-050.000492

2e-068e-062.2e-05

0.0020140.0112450.0033580.0520560.0019970.0018850.073641

2.2e-05

03e-060

4e-062.1e-052e-06

1e-061.1e-051e-06

2.5e-05

2e-050.0001251.7e-05

1e-054.7e-059e-06

1e-062.4e-050

1e-062.4e-050

9e-062.3e-059e-06

9e-069e-06

2.3e-05

7e-063.7e-056e-06

7e-063.7e-056e-06

7e-063.7e-056e-06

3e-064.1e-052e-06

3e-064.1e-052e-06

3e-064.1e-052e-06

7.5e-050.0002786.4e-05

7e-066.5e-055e-06

7e-066.5e-055e-06

7e-066.5e-055e-06

7e-066.5e-055e-06

7e-066.5e-055e-06

6.1e-050.0001765.3e-05

6.1e-050.0001765.3e-05

6.1e-050.0001765.3e-05

1.9e-055.2e-051.9e-05

1e-052.4e-051.1e-05

9e-062.8e-058e-06

8e-063.6e-056e-06

8e-063.6e-056e-06

1.3e-053.5e-051e-05

1.3e-053.5e-051e-05

2.1e-055.3e-051.8e-05

8e-062.6e-057e-06

1.3e-052.7e-051.1e-05

7e-063.7e-056e-06

7e-063.7e-056e-06

7e-063.7e-056e-06

7e-063.7e-056e-06

7e-063.7e-056e-06

3e-058.2e-051.2e-05

3e-058.2e-051.2e-05

3e-058.2e-051.2e-05

3e-058.2e-051.2e-05

3e-058.2e-051.2e-05

3e-058.2e-051.2e-05

0.0029560.001830.0022820.0022070.0031490.00284

0.0029560.001830.0022820.0022070.0031490.00284

0.0029560.001830.0022820.0022070.0031490.00284

3e-060.0001353e-06

3e-060.0001353e-06

3e-060.0001353e-06

0.0029530.001830.0022820.0022070.0030140.002837

0.0029530.001830.0022820.0022070.0030140.002837

0.0029530.001830.0022820.0022070.0030140.002837

0.0003580.000311

0.0003580.000311

0.0003580.000311

0.0003580.000311

0.0001770.000166

0.0001770.000166

0.0001770.000166

0.0001810.000145

0.0001810.000145

0.0001810.000145
