# Supplementary material for: MetaMeta: integrating metagenome analysis tools to improve taxonomic profiling
Source: Microbiome. 2017 Aug 14;5:101. doi: 10.1186/s40168-017-0318-y (PMC5557516; doi:10.1186/s40168-017-0318-y)
Supplement: Supplementary file 2 — Additional File with interactive charts for all CAMI toy set results on default, very-precise and very-sensitive mode. File prefix S, M, and H for low, medium and high complexity, respectively. (TAR 3573 kb) [file 40168_2017_318_MOESM2_ESM.tar › H_S001__insert_180_very-sensitive.html]

Javascript must be enabled to view this page.

magnitude
magnitudeUnassigned

clark.parsed\_profile
dudes.parsed\_profile
final.metametamerge.profile
gottcha.parsed\_profile
kaiju.parsed\_profile
kraken.parsed\_profile
motus.parsed\_profile

0.9999999999999971.0000060.9999850000000011.0000170.9999990.9999959999999981.000003

0.0003580.0002960.000311

0.0003580.0002960.000311

0.0003580.0002960.000311

0.0003580.0002960.000311

0.0001810.0001430.000145

0.0001810.0001430.000145

0.0001810.0001430.000145

0.0001770.0001530.000166

0.0001770.0001530.000166

0.0001770.0001530.000166

0.0854340.058110.0533270.052560.0802790.0822080.050763

5e-060.0001234e-06

5e-060.0001234e-06

9e-05

9e-05

5.3e-05

5.3e-05

3.7e-05

3.7e-05

5e-063.3e-054e-06

5e-063.3e-054e-06

5e-063.3e-054e-06

9e-06

5e-062.4e-054e-06

1.7e-057.9e-051.3e-05

1.7e-057.9e-051.3e-05

1.7e-057.9e-051.3e-05

1.7e-057.9e-051.3e-05

1.7e-057.9e-051.3e-05

1.7e-057.9e-051.3e-05

0.0444870.0243570.0249470.0243740.0406570.0429240.021351

0.0030490.0022670.0021420.0038110.0024040.0029280.001695

0.0030490.0022670.0021420.0038110.0024040.0029280.001695

0.0030490.0022670.0021420.0038110.0024040.0029280.001695

5e-06

5e-06

0.0008040.0009850.0007750.0018050.0007010.0007740.000786

4e-06

8e-06

03e-060

0.0008030.0009850.0007750.0018050.0006630.0007730.000786

09e-060

03e-060

1e-061.1e-051e-06

0.0022450.0012820.0013670.0020060.0016980.0021540.000909

0.0022390.0012820.0013670.0020060.0016230.0021480.000909

6e-06

4e-06

5e-06

04e-060

2e-061e-06

3e-06

3e-06

5e-06

3e-060

4e-06

4e-068e-064e-06

2e-06

2e-065e-061e-06

06e-060

8e-06

7e-06

1e-058.2e-051.1e-05

1e-058.2e-051.1e-05

3e-063.3e-053e-06

1e-067e-061e-06

1e-067e-061e-06

2e-062.6e-052e-06

2e-06

2e-06

01e-060

7e-06

2e-067e-062e-06

07e-060

7e-064.9e-058e-06

6e-064.2e-057e-06

2e-061e-052e-06

1e-061.6e-051e-06

01.1e-051e-06

3e-065e-063e-06

1e-067e-061e-06

1e-067e-061e-06

0.0006350.0003740.0005450.0016050.000630.00061

0.0006350.0003740.0005450.0016050.000630.00061

0.0006350.0003740.0005450.0016050.000630.00061

0.0006350.0003740.0005450.0016050.000630.00061

0.0006340.0003740.0005450.0016050.0006090.000609

1e-062.1e-051e-06

0.0039430.0023370.0023310.0025080.0038190.0037930.001542

0.0038920.0023370.0023310.0025080.0034560.0037310.001542

0.0038920.0023370.0023310.0025080.0034560.0037310.001542

0.0038920.0023370.0023310.0025080.0034560.0037310.001542

0.0038910.0023370.0023310.0025080.0034340.003730.001542

1e-062.2e-051e-06

1.4e-050.000241.4e-05

4e-065.7e-055e-06

4e-065.7e-055e-06

3e-062.8e-053e-06

1e-062.9e-052e-06

1e-050.0001839e-06

07.2e-050

1.3e-05

3e-06

1e-06

02e-060

1e-05

4e-06

02e-060

5e-06

06e-060

1.5e-05

1e-06

1e-05

1e-061.4e-051e-06

1e-061.4e-051e-06

2e-061.6e-052e-06

2e-061.6e-052e-06

2.3e-05

9e-06

1.4e-05

01.2e-050

01.2e-050

7e-061.6e-056e-06

7e-061.6e-056e-06

03e-050

03e-050

3.7e-050.0001234.8e-05

1.8e-053.9e-052.8e-05

1.8e-052.6e-052.8e-05

1.7e-051.4e-051.6e-05

1e-061.2e-051.2e-05

01.3e-050

01.3e-050

1.1e-054.6e-051.1e-05

01.7e-051e-06

01.7e-051e-06

1.1e-052.9e-051e-05

4e-06

7e-061.4e-056e-06

4e-061.1e-054e-06

8e-062.5e-059e-06

8e-062.5e-059e-06

8e-062.5e-059e-06

01.3e-05

01.3e-05

01.3e-05

0.0015250.0007430.0009190.003210.0019450.0014830.000666

0.0011870.0006310.0007730.0017050.0013010.0011490.000566

0.0011730.0006310.0007730.0017050.0011420.0011340.000566

0.0011030.0006310.0007730.0017050.000930.0010610.000566

0.0010960.0006310.0007730.0017050.0009030.0010520.000566

7e-062.7e-059e-06

1e-051.7e-059e-06

4e-061e-063e-06

6e-061.3e-056e-06

3e-06

1e-054.5e-051.4e-05

1e-054.5e-051.4e-05

5e-050.000155e-05

2.7e-057.8e-052.8e-05

2.3e-057.2e-052.2e-05

2.3e-05

2.3e-05

2.3e-05

1.4e-050.0001361.5e-05

8e-068.8e-059e-06

3.5e-05

3.4e-05

8e-061.9e-059e-06

6e-064.8e-056e-06

6e-064.8e-056e-06

7.4e-050.0002147.5e-05

7.4e-050.0002147.5e-05

7e-062.3e-058e-06

7e-062.3e-058e-06

6e-061.8e-056e-06

6e-061.8e-056e-06

2.3e-05

2.3e-05

1.3e-054.1e-051.1e-05

1.3e-054.1e-051.1e-05

1.8e-053.8e-051.8e-05

9e-061.7e-058e-06

9e-062.1e-051e-05

1.4e-052.9e-051.6e-05

1.4e-052.9e-051.6e-05

1e-052.5e-051e-05

1e-052.5e-051e-05

6e-061.7e-056e-06

6e-061.7e-056e-06

0.0002640.0001120.0001460.0015050.000430.0002590.0001

0.0002460.0001120.0015050.0002650.0002390.0001

1.2e-054.7e-051.1e-05

1.2e-054.7e-051.1e-05

7e-065.5e-058e-06

7e-065.5e-058e-06

0.0002270.0001120.0015050.0001630.000220.0001

2.6e-05

0.0002180.0001120.0015050.0001090.000210.0001

9e-062.8e-051e-05

1.8e-050.0001460.0001652e-05

1.8e-050.0001460.0001652e-05

1.8e-050.0001460.0001652e-05

6e-068e-056e-06

6e-068e-056e-06

6e-068e-056e-06

2.2e-05

1.2e-05

1e-05

01e-05

01e-05

6e-064.8e-056e-06

1e-066e-061e-06

1e-061e-051e-06

1e-061.6e-051e-06

3e-061.6e-053e-06

0.0352560.0186360.019010.013240.0314150.0340310.017448

0.0352560.0186360.019010.013240.0314150.0340310.017448

2e-061.4e-052e-06

2e-061.4e-052e-06

2e-061.4e-052e-06

0.0352540.0186360.019010.013240.0314010.0340290.017448

0.0012610.00080.0007610.0017050.0011410.001210.000372

0.0012610.00080.0007610.0017050.0011410.001210.000372

1e-061.6e-050

1e-063e-060

1.2e-05

1e-06

0.0339870.0178360.0182490.0115350.0301970.0328150.017076

9.5e-05

1.2e-053.4e-051.1e-05

0.0339680.0178360.0181220.0115350.0298640.0327970.017076

0.0001270.000143

7e-063e-057e-06

3.1e-05

5e-064.7e-054e-06

02.3e-05

3e-061.1e-053e-06

2e-061e-051e-06

3e-06

6.3e-050.0002826.2e-05

3.5e-05

3.5e-05

3.5e-05

3.5e-05

4e-067.2e-052e-06

4e-067.2e-052e-06

3e-061.7e-052e-06

3e-061.7e-052e-06

2.3e-05

2.3e-05

1e-063.2e-05

1e-063.2e-05

5.9e-050.0001756e-05

2e-061.6e-052e-06

2e-061.6e-052e-06

2e-061.6e-052e-06

5.5e-050.0001125.5e-05

5.5e-050.0001125.5e-05

7e-064.1e-057e-06

4.8e-057.1e-054.8e-05

2e-062.6e-053e-06

2e-062.6e-053e-06

2e-062.6e-053e-06

02.1e-050

02.1e-050

02.1e-050

0.0409240.0337530.028380.0281860.0393950.0392670.029412

0.0409240.0337530.028380.0281860.0393950.0392670.029412

3e-062.4e-051e-06

2e-061.2e-051e-06

2e-061.2e-051e-06

2e-061.2e-051e-06

1e-061.2e-050

1e-061.2e-050

1e-061.2e-050

6.7e-050.0015080.0032920.0050160.0006476.5e-050.001412

6e-062.1e-054e-06

6e-062.1e-054e-06

6e-061.1e-054e-06

1e-06

0

9e-06

6.1e-050.0015080.0032920.0050160.0006266.1e-050.001412

1.1e-050.0015080.0019530.0035110.0004161.2e-050.001412

0.00040.0005070.0016050.000465

1.1e-050.0003710.0004161.2e-05

0.0011080.0010750.0019060.000947

1e-061.8e-051e-06

1e-069e-061e-06

09e-060

3.6e-050.0013390.0015055.6e-053.4e-05

3.6e-050.0013390.0015055.6e-053.4e-05

1.3e-050.0001361.4e-05

3e-065.7e-054e-06

1e-057.9e-051e-05

0.0019940.0100160.0026780.0063190.0017170.0019160.009601

0.0019940.0100160.0026780.0063190.0017170.0019160.009601

0.0019940.0100160.0026780.0063190.0016890.0019160.009601

0.0019910.0100160.0026780.0063190.0016480.0019130.009567

1.5e-05

03e-060

1e-061.5e-051e-06

2e-068e-062e-063.4e-05

01.1e-050

2e-06

09e-060

01.7e-05

01.7e-05

0.038860.0222290.022410.0168510.0370070.0372850.018399

2.7e-05

1.1e-05

1.1e-05

9e-06

9e-06

7e-06

7e-06

0.038860.0222290.022410.0168510.036980.0372850.018399

1e-062e-051e-06

1e-062e-051e-06

1e-061.9e-051e-06

1e-061.9e-051e-06

03e-051e-06

01e-051e-06

02e-050

7e-06

5e-06

2e-06

0.0388580.0222290.022410.0168510.0369040.0372820.018399

0.0388580.0222290.022410.0168510.0369040.0372820.018399

1e-062.5e-050

1e-062.5e-050

1e-062.5e-050

1e-062.5e-050

1e-062.5e-050

1e-062.5e-050

0.9142079999999970.9418960000000010.9463620000000010.9474569999999980.919720.9174769999999980.949240000000001

3.6e-050.0003083.7e-05

3.6e-050.0003083.7e-05

3.6e-050.0003083.7e-05

3.6e-050.0003083.7e-05

8e-064.5e-054e-06

2e-064e-061e-06

6e-063.7e-053e-06

4e-06

1.2e-050.0002069e-06

1.2e-056.9e-059e-06

7.8e-05

5.9e-05

1.6e-055.7e-052.4e-05

1.6e-055.7e-052.4e-05

3e-063.8e-052e-06

3e-063.8e-052e-06

3e-063.8e-052e-06

3e-063.8e-052e-06

3e-063.8e-052e-06

3e-063.8e-052e-06

0.2928220.240960.1813370.2293920.2434360.2935110.213011

0.0002060.0006020.00016

0.0001010.0002998e-05

0.0001010.0002998e-05

1.6e-059e-06

1.6e-059e-06

5.3e-05

5.3e-05

2.9e-054.1e-052.5e-05

2.9e-054.1e-052.5e-05

9e-062.9e-055e-06

9e-062.9e-055e-06

3.2e-050.0001052.6e-05

1e-055.8e-058e-06

2.2e-054.7e-051.8e-05

1.5e-057.1e-051.5e-05

1.5e-057.1e-051.5e-05

0.0001050.0003038e-05

8.5e-050.0001716.6e-05

2.8e-050.0001112.4e-05

5.6e-05

2.8e-055.5e-052.4e-05

5.7e-056e-054.2e-05

5.7e-056e-054.2e-05

2e-050.0001321.4e-05

2e-058.4e-051.4e-05

2e-058.4e-051.4e-05

4.8e-05

4.8e-05

4.3e-050.0001283.3e-05

4.3e-050.0001283.3e-05

4.3e-050.0001283.3e-05

4.3e-050.0001283.3e-05

5.8e-05

4.3e-057e-053.3e-05

0.2922510.2409310.1810680.2293920.2421680.2930240.212994

0.0004120.0001530.0002490.0015050.0004990.00040.000111

0.0004120.0001530.0002490.0015050.0004990.00040.000111

0.0004120.0001530.0002490.0015050.0004990.00040.000111

2.5e-056.2e-052.6e-05

4.2e-055.5e-053.8e-051.5e-05

3.5e-056.2e-053.6e-05

0.0002890.0001530.0002490.0015050.0002750.0002799.6e-05

2.1e-054.5e-052.1e-05

0.0026990.0030470.0021670.0041120.0024120.0025990.001937

0.0026990.0030470.0021670.0041120.0024120.0025990.001937

6.6e-056.5e-056.2e-055.8e-05

6.6e-056.5e-056.2e-055.8e-05

7.3e-055.8e-057.1e-05

7.3e-055.8e-057.1e-05

0.0021990.0030050.0019580.0041120.0014480.0021180.001833

0.0011570.0006170.0006010.0017050.0002910.001116

0.0010420.0023880.0012240.0024070.0009740.0010020.001833

0.0001330.00015

3.3e-05

7e-05

7e-05

6.4e-05

6.4e-05

7.8e-057.6e-05

7.8e-057.6e-05

5.3e-05

5.3e-05

0.0001974.2e-050.0002090.0004020.0001913.1e-05

0.0001974.2e-050.0002090.0004020.0001913.1e-05

5.5e-05

5.5e-05

8.6e-050.0001978.1e-051.5e-05

4.8e-05

4e-06

2.9e-05

1e-06

0

8.6e-058.3e-058.1e-051.5e-05

3.2e-05

3.6e-055.1e-053.4e-05

3.6e-055.1e-053.4e-05

3.6e-055.1e-053.4e-05

3.6e-055.1e-053.4e-05

0.0294010.0406880.0289720.0306930.0167040.0362760.039797

3.3e-05

3.3e-05

3.3e-05

0.0220910.0311210.0100810.0190580.0050080.0289990.023188

4.4e-050.0001290.0001453.6e-055e-06

4.4e-050.0001290.0001453.6e-055e-06

0.0220470.0311210.0099520.0190580.0048630.0289630.023183

4e-066e-065e-06

5.7e-054.8e-056.6e-05

0.0024080.0039290.0018110.003210.0006510.0054440.004055

6.5e-05

5e-052.9e-055.6e-05

4e-06

4.3e-05

4.3e-05

8e-062e-067e-06

1.5e-058.5e-050.0013390.0015054e-061.3e-05

0

2e-064e-062e-06

5e-067e-063e-06

000

2.8e-05

2.7e-051.6e-053e-05

4e-06

6.5e-052.4e-057e-053.6e-05

0.0029730.0017910.0020880.0021060.0024290.002856

1e-056e-061.1e-055.3e-05

2.1e-05

3.3e-05

5e-069e-065e-06

0.0162150.0253160.0047140.0122370.0011790.0202590.018862

0

4.6e-056e-064.5e-053.8e-05

0005.1e-05

4.3e-05

3.2e-05

6.8e-050

4e-053.2e-054.1e-05

3.3e-05

2e-062e-062e-06

3e-062e-063e-06

5.5e-05

00

1.2e-05

00

4.4e-052.1e-054.5e-052.3e-05

3.5e-05

5.5e-056.2e-055e-05

5.5e-056.2e-055e-05

5.5e-056.2e-055e-05

0.0001160.0001010.000108

0.0001160.0001010.000108

0.0001160.0001010.000108

0.0030750.0019250.0022780.0021060.0024430.0030620.001345

0.002260.0017570.0017240.0021060.0014330.0022380.001296

0.0002960.0001070.0001663.2e-050.000299

1e-05

2.8e-05

0.0003770.0001340.0001940.0003770.000390.000132

0.0012960.0013840.0006330.0021060.0002090.001250.001131

0.0001256.1e-050.0001132.9e-050.000133.3e-05

3.6e-05

0.0002630.000296

3.4e-05

0.0001667.1e-050.0001496.6e-050.000169

5.9e-05

2.6e-05

0.0002060.000231

0.0008150.0001680.0005540.001010.0008244.9e-05

0.0002354e-050.0001450.0001010.0002373.1e-05

0.0003960.0001280.0002460.0006110.000403

0.0001840.0001630.0001790.0001841.8e-05

0.000119

0.0003410.0005920.0019060.0007890.000335.1e-05

0.0003410.0005920.0019060.0007890.000335.1e-05

1.7e-05

1.9e-05

0.0004480.000503

0.0001148.9e-050.00011

0.0001140.0001440.0019063.7e-050.000108

4.7e-05

0.0001130.0001130.0001121.5e-05

0.0037230.0076420.0160210.0076230.0082680.0037270.015213

0.0037230.0076420.0160210.0076230.0082680.0037270.015213

6.3e-055.8e-056.3e-05

5.6e-05

0.0002250.0001650.0001360.000227

0.000121

0.0001670.0001490.000160.000175

0.0001310.000147

0.0001260.000142

2.9e-05

3e-056.8e-050.0020540.0023071.9e-053e-05

0.000119

4.3e-05

0.0014610.001641

0.0001160.00013

6e-053.9e-055.7e-05

0.000106

0.0011160.000560.0007550.0008550.001154

7.5e-055.2e-057.7e-05

0.0003350.0002060.0002240.0002020.000326

1e-059e-061.2e-05

6.6e-05

0.0002830.000318

1.5e-05

0.0006610.000743

7.7e-057.1e-057.6e-05

8.3e-05

0.0001015.7e-055.2e-050.0001021.3e-05

7.2e-05

0.0001470.0003840.0001679.9e-050.000152

0.0001240.00014

0.0068910.007741

0.000280.000314

7.4e-05

6.6e-058.5e-057.1e-05

0.0011240.0063430.000890.0053160.000270.0010790.007355

1.2e-052.4e-052e-061.3e-05

0.0001140.000128

8.4e-05

0.0001620.000182

0.0002410.000271

0.0002380.000267

0.0001157e-050.0001131.5e-05

0.0007890.000887

0.0063190.0035510.0042210.0029090.0063290.0060980.003331

0.0063190.0035510.0042210.0029090.0063290.0060980.003331

0.006270.0035510.0042210.0029090.0062750.0060510.0033

3e-05

2.4e-05

2.4e-05

1.7e-05

2.6e-05

0.0003280.000368

4.6e-051.6e-054.7e-05

3.7e-056e-053.9e-05

3.2e-05

3.2e-05

3e-05

4.3e-05

4e-053.6e-054.4e-05

2.3e-05

4e-053.2e-05

0.0057680.0035250.0034260.0029090.004550.0055410.002794

2.3e-05

3.8e-052.2e-054.3e-05

0.0004670.000525

2.1e-05

2.5e-05

3.5e-05

3.8e-05

1.3e-05

2.7e-05

1.5e-05

4.8e-05

0.0001184.6e-05

2.8e-05

3.6e-05

2.2e-051.3e-051.9e-054.6e-05

4.1e-054.2e-05

2.7e-05

2.8e-051.3e-052.3e-052.9e-05

5e-06

5.2e-051.4e-055.4e-05

3.5e-05

1.6e-05

2.3e-05

1.6e-05

5e-06

4.8e-05

1.6e-05

4.7e-058e-065e-05

3.8e-052.5e-053.8e-05

3e-061e-052e-06

3e-064e-064e-06

3.9e-053.2e-054.2e-05

2.8e-051.9e-053e-05

1.7e-05

5.6e-05

4.9e-055.4e-054.7e-053.1e-05

4.9e-055.4e-054.7e-053.1e-05

0.0174520.0101580.0102520.0075230.0167370.0167330.008943

0.0174520.0101580.0102520.0075230.0167370.0167330.008943

2.1e-054e-051.1e-05

2.1e-054e-051.1e-05

1.1e-05

1.1e-05

4.8e-05

4.8e-05

0.0174310.0101580.0102520.0075230.0164970.0167220.008943

0.0174310.0101580.0102520.0075230.0164970.0167220.008943

0.000141

3.3e-05

3.9e-05

3.6e-05

3.3e-05

0.0126070.0527020.0133550.0955870.0058630.0165840.055993

0.0126070.0527020.0133550.0955870.0058630.0165840.055993

0.0001054.4e-05

0.0001054.4e-05

0.0112870.0512920.0121570.093280.0047980.0153150.054395

2.8e-054.6e-053.2e-05

1.4e-051.4e-051.3e-05

1.3e-05

2.9e-05

0.0105120.0505420.0115840.0422270.0042490.0145630.05413

2e-062e-063e-06

2e-06

0.0004810.0005730.000390.0510530.0001850.00046

2.1e-053.2e-052.4e-05

6.5e-05

0.0002030.0001770.0001839.3e-050.0001970.000265

2.2e-05

2e-06

2.6e-051e-052.3e-05

1.8e-05

1.2e-05

4e-06

7.3e-05

7.3e-05

0.001320.001410.0011980.0023070.0008870.0012690.001554

0.001320.001410.0011980.0023070.0008870.0012690.001554

4.7e-055.9e-054e-054.3e-05

4.7e-055.9e-054e-054.3e-05

4.7e-055.9e-054e-054.3e-05

4.7e-055.9e-054e-054.3e-05

0.2169540.1280980.1174050.0788370.1856340.2082320.099499

0.2169540.1280980.1174050.0788370.1856340.2082320.099499

3.5e-055.7e-053.6e-05

2.3e-053.1e-052.3e-05

1.2e-052.6e-051.3e-05

0.0429140.0242830.0228250.0171520.0330660.0412320.021204

0.0420930.0240050.0222290.0139420.032160.0404250.02114

0.0008210.0002780.0005960.003210.0009060.0008076.4e-05

0.1739260.1038150.0944250.0616850.1523360.1668870.078279

4e-050.0001560.0001753.8e-05

0.1738070.1038150.0937620.0616850.1515920.1667720.078279

4.1e-050.0003710.0004174e-05

3.8e-050.0001360.0001523.7e-05

7.9e-050.0001550.0001757.7e-051.6e-05

7.9e-050.0001550.0001757.7e-051.6e-05

5.7e-057.3e-055.1e-05

5.7e-057.3e-055.1e-05

5.7e-057.3e-055.1e-05

5.7e-057.3e-055.1e-05

0.0040510.0018260.0032990.0065210.0052580.0038460.002662

0.0001940.0001630.0001851e-05

0.0001940.0001630.0001851e-05

5.9e-054.5e-055.7e-05

6.3e-055.8e-056e-051e-05

7.2e-056e-056.8e-05

6.1e-055.4e-055.9e-051.5e-05

6.1e-055.4e-055.9e-051.5e-05

6.1e-055.4e-055.9e-051.5e-05

0.000960.0003930.0004830.003010.0011330.000890.000358

6.2e-057.2e-055.3e-05

3.3e-052.2e-052.7e-05

2.5e-05

2.9e-052.5e-052.6e-05

6.3e-050.0001525.1e-051.6e-05

6.3e-054.4e-055.1e-051.6e-05

5e-05

5.8e-05

5.6e-05

5.6e-05

0.000390.0002230.0002910.0015050.0004810.0003730.000219

5e-06

3e-066e-063e-06

2e-05

7e-06

1.8e-05

0.0003870.0002230.0002910.0015050.00030.000370.000219

2.8e-05

3.6e-05

2.3e-05

1.7e-05

2.1e-05

8.8e-058.4e-057.6e-05

2.3e-052.3e-052e-05

6.5e-056.1e-055.6e-05

3.7e-052.2e-053.1e-05

3.7e-052.2e-053.1e-05

5e-068e-065e-06

5e-068e-065e-06

0.0002490.000170.0001920.0015050.0002050.0002390.000123

0.0002490.000170.0001920.0015050.0002050.0002390.000123

6.6e-055.3e-056.2e-05

6.6e-055.3e-056.2e-05

0.002160.0011790.0014080.0019060.0019620.002070.001113

0.0020880.0011790.0014080.0019060.0019020.0020030.001113

6.4e-05

0.0020880.0011790.0014080.0019060.0018380.0020030.001113

7.2e-056e-056.7e-05

7.2e-056e-056.7e-05

5e-058.5e-054.8e-05

3.4e-05

3.4e-05

5e-055.1e-054.8e-05

5e-055.1e-054.8e-05

0.0004540.0002540.0003640.0016050.0010610.0004380.00029

0.0001220.0001150.0005790.000121

6.9e-05

0.0001150.000129

0.0001220.0001070.000121

7e-05

0.000103

0.000101

3.3e-05

3.3e-05

4.8e-05

4.8e-05

0.0002870.0002540.0002490.0016050.0001580.0002740.00029

0.0002870.0002540.0002490.0016050.0001580.0002740.00029

5.1e-05

1.2e-05

3.9e-05

5e-05

5e-05

4.3e-05

4.3e-05

3.2e-05

3.2e-05

4.4e-05

4.4e-05

4.5e-052.3e-054.3e-05

4.5e-052.3e-054.3e-05

2e-061.2e-051e-06

2e-061.2e-051e-06

2e-061.2e-051e-06

1.1e-053.8e-058e-06

1.1e-053.8e-058e-06

1.1e-053.8e-058e-06

0.0010440.0003930.000779

0.0010440.0003930.000779

0.0006940.000779

0.000350.000393

4.9e-050.000154.3e-051.6e-05

4.9e-05

4.9e-05

4.9e-05

4.9e-05

4.9e-055.2e-054.3e-051.6e-05

4.9e-055.2e-054.3e-051.6e-05

6e-050.0001455.6e-056.6e-05

5.1e-05

5.1e-05

6e-054.7e-055.6e-05

6e-054.7e-055.6e-05

4.7e-05

4.7e-05

6.6e-05

6.6e-05

5e-056.2e-054.8e-051.5e-05

5e-056.2e-054.8e-051.5e-05

5e-056.2e-054.8e-051.5e-05

3.8e-055.7e-053.5e-05

3.8e-055.7e-053.5e-05

3.8e-055.7e-053.5e-05

3.8e-055.7e-053.5e-05

0.0001240.0001230.0002560.000117

0.0001240.0001230.0002560.000117

0.0001240.0001230.00020.000117

0.0001240.0001230.00020.000117

5.6e-05

5.6e-05

1.5e-055e-051.3e-05

1.5e-055e-051.3e-05

1.5e-055e-051.3e-05

1.5e-055e-051.3e-05

0.000160.0001690.0001596.2e-05

0.000160.0001690.0001596.2e-05

5.4e-055.4e-055.2e-056.2e-05

5.4e-055.4e-055.2e-056.2e-05

5.4e-056.1e-055.4e-05

5.4e-056.1e-055.4e-05

5.2e-055.4e-055.3e-05

5.2e-055.4e-055.3e-05

0.0016160.0007080.0010250.0017050.0016130.0015620.000616

7.9e-058.1e-057.8e-05

7.9e-058.1e-057.8e-05

7.9e-058.1e-057.8e-05

0.0013580.0006690.0008310.0017050.0011630.0013010.000568

0.001320.0006690.0008310.0017050.0011060.001270.000552

3.5e-055e-053.3e-05

0.0012850.0006690.0008310.0017050.0010560.0012370.000552

3.8e-055.7e-053.1e-051.6e-05

3.8e-055.7e-053.1e-051.6e-05

0.0001793.9e-050.0001940.0003690.0001834.8e-05

0.0001793.9e-050.0001940.0003690.0001834.8e-05

0.0001793.9e-050.0001940.0003690.0001834.8e-05

0.0002630.0004040.000245

0.0001050.0002579.7e-05

5.5e-05

5.5e-05

5.6e-059.5e-055.1e-05

5.6e-055e-055.1e-05

4.5e-05

5.5e-05

5.5e-05

4.9e-055.2e-054.6e-05

4.9e-055.2e-054.6e-05

0.0001580.0001470.000148

3.9e-051.9e-054e-05

3.9e-051.9e-054e-05

2.6e-053.6e-052.2e-05

2.6e-053.6e-052.2e-05

2.7e-051.3e-052.6e-05

2.5e-051.1e-052.4e-05

2e-062e-062e-06

3.2e-052.8e-053.2e-05

3.2e-051.9e-053.2e-05

9e-06

3.4e-055.1e-052.8e-05

3.4e-055.1e-052.8e-05

0.0002652.9e-050.0002690.0004450.0002551.7e-05

0.0002652.9e-050.0002690.0004450.0002551.7e-05

0.0002652.9e-050.0002690.0004450.0002551.7e-05

0.0002652.9e-050.0002690.0004450.0002551.7e-05

0.0002652.9e-050.0002690.0004450.0002551.7e-05

5.7e-059.3e-053.9e-05

5.7e-059.3e-053.9e-05

5.7e-059.3e-053.9e-05

2.4e-054.4e-051.9e-05

2.4e-054.4e-051.9e-05

3.3e-054.9e-052e-05

3.3e-054.9e-052e-05

6.4e-05

6.4e-05

6.4e-05

6.4e-05

6.4e-05

6.4e-05

0.0014970.0008740.0009880.0018050.0013270.0014390.000637

0.0014970.0008740.0009880.0018050.0013270.0014390.000637

0.0014970.0008740.0009880.0018050.0013270.0014390.000637

0.0014970.0008740.0009880.0018050.0013270.0014390.000637

0.0014970.0008740.0009880.0018050.0013270.0014390.000637

0.0014960.0008740.0009880.0018050.0012930.0014370.000637

1e-063.4e-052e-06

2.2e-050.0001612.8e-05

2.2e-050.0001612.8e-05

2.2e-050.0001612.8e-05

2.2e-050.0001612.8e-05

4e-064.4e-052e-06

4e-064.4e-052e-06

6e-063.8e-056e-06

6e-063.8e-056e-06

9e-064.3e-051.8e-05

9e-064.3e-051.8e-05

3e-063.6e-052e-06

3e-063.6e-052e-06

3e-058.2e-051.2e-05

3e-058.2e-051.2e-05

3e-058.2e-051.2e-05

3e-058.2e-051.2e-05

3e-058.2e-051.2e-05

3e-058.2e-051.2e-05

1.7e-055.5e-051.2e-05

1.7e-055.5e-051.2e-05

1.7e-055.5e-051.2e-05

1.7e-055.5e-051.2e-05

1.7e-055.5e-051.2e-05

1.7e-055.5e-051.2e-05

7.5e-050.0002786.4e-05

6.1e-050.0001765.3e-05

6.1e-050.0001765.3e-05

6.1e-050.0001765.3e-05

8e-063.6e-056e-06

8e-063.6e-056e-06

1.3e-053.5e-051e-05

1.3e-053.5e-051e-05

2.1e-055.3e-051.8e-05

1.3e-052.7e-051.1e-05

8e-062.6e-057e-06

1.9e-055.2e-051.9e-05

9e-062.8e-058e-06

1e-052.4e-051.1e-05

7e-066.5e-055e-06

7e-066.5e-055e-06

7e-066.5e-055e-06

7e-066.5e-055e-06

7e-066.5e-055e-06

7e-063.7e-056e-06

7e-063.7e-056e-06

7e-063.7e-056e-06

7e-063.7e-056e-06

7e-063.7e-056e-06

0.0053050.0076740.0043150.0074220.0031250.0097020.006606

0.0053050.0076740.0043150.0074220.0031250.0097020.006606

0.0001999.1e-050.0001713.3e-05

0.0001999.1e-050.0001713.3e-05

0.0001999.1e-050.0001713.3e-05

5e-053.1e-055.1e-05

5e-061.1e-055e-06

8.8e-0506e-05

5.6e-054.9e-055.5e-053.3e-05

0.0012450.0026480.0010080.0024070.0011530.0011460.002494

0.0011150.0026480.0010080.0024070.0005660.0010320.002494

7.7e-051.5e-053.9e-05

1.7e-0502.4e-05

5e-062e-065e-06

1e-06

4.3e-051e-05

2e-0600

2e-06

1e-051e-05

0.0010380.0026480.0010080.0024070.0005510.0009930.002494

2.8e-052.4e-052.4e-05

0.0009610.0026480.0010080.0024070.0005050.0009230.002494

2.8e-059e-062.5e-05

1e-063e-061e-06

1.5e-052e-061.5e-05

5e-068e-065e-06

0.000130.0005870.000114

1.3e-053.7e-059e-06

1.3e-053.7e-059e-06

1.3e-055.6e-051.1e-05

6e-063e-055e-06

7e-062.6e-056e-06

6.6e-050.0003456.2e-05

1e-06

1e-06

3e-063.1e-053e-06

3.1e-057.1e-053.1e-05

2.2e-05

4.7e-05

3e-062.9e-053e-06

4e-065.5e-053e-06

6e-064.1e-054e-06

4e-063.2e-054e-06

1.5e-051.5e-051.4e-05

3.4e-050.0001172.8e-05

8e-065.7e-059e-06

5e-062.6e-055e-06

2.1e-053.4e-051.4e-05

4e-063.2e-054e-06

4e-063.2e-054e-06

0.0038610.0050260.0033070.0050150.0018810.0083850.004079

0.0038610.0050260.0033070.0050150.0018810.0083850.004079

1e-054.7e-059e-06

1e-054.7e-059e-06

0.0038510.0050260.0033070.0050150.0018340.0083760.004079

1.8e-05

0.0013670.0023070.0009720.0024070.0003730.0015870.001666

1e-063e-051e-06

0.0003660.000411

0.0024830.0027190.0019690.0026080.0010020.0067880.002413

0.0034870.0017990.0019750.0022070.0035680.0033580.001247

1.9e-055.3e-051.3e-05

1.9e-055.3e-051.3e-05

1.9e-055.3e-051.3e-05

1.9e-055.3e-051.3e-05

1.9e-055.3e-051.3e-05

0.0034680.0017990.0019750.0022070.0035150.0033450.001247

0.0034680.0017990.0019750.0022070.0035150.0033450.001247

0.0034590.0017990.0019750.0022070.0034580.0033350.001247

7e-065.1e-056e-06

7e-065.1e-056e-06

0.0034450.0017990.0019750.0022070.0033070.0033220.001247

0.0034450.0017990.0019750.0022070.0033070.0033220.001247

5e-062.7e-054e-06

5e-062.7e-054e-06

2e-063.2e-053e-06

2e-063.2e-053e-06

4.1e-05

4.1e-05

9e-065.7e-051e-05

3e-062.9e-053e-06

3e-062.9e-053e-06

6e-062.8e-057e-06

6e-062.8e-057e-06

0.0029560.001830.002320.0022070.0031490.00284

0.0029560.001830.002320.0022070.0031490.00284

0.0029560.001830.002320.0022070.0031490.00284

3e-060.000120.0001353e-06

3e-060.000120.0001353e-06

3e-060.000120.0001353e-06

0.0029530.001830.00220.0022070.0030140.002837

0.0029530.001830.00220.0022070.0030140.002837

0.0029530.001830.00220.0022070.0030140.002837

9.5e-050.0047190.0005258.2e-050.005144

0.0045790.005144

0.0045790.005144

0.0045790.005144

0.0045790.005144

0.0045790.005144

9e-060.000140.0001931.4e-05

9e-060.000140.0001931.4e-05

9e-060.000140.0001931.4e-05

9e-063.5e-051.4e-05

9e-063.5e-051.4e-05

0.000140.000158

0.000140.000158

1.5e-057.1e-051.1e-05

1.5e-057.1e-051.1e-05

3e-062.4e-052e-06

3e-062.4e-052e-06

0

1e-06

3e-062.3e-052e-06

1.2e-054.7e-059e-06

1.2e-054.7e-059e-06

7e-062.4e-056e-06

5e-062.3e-053e-06

7e-065.9e-055e-06

7e-065.9e-055e-06

7e-065.9e-055e-06

7e-065.9e-055e-06

7e-065.9e-055e-06

1.9e-056e-051.5e-05

3e-064e-064e-06

3e-064e-064e-06

3e-064e-064e-06

0

3e-064e-064e-06

1.6e-055.6e-051.1e-05

1.6e-055.6e-051.1e-05

1.6e-055.6e-051.1e-05

1.6e-055.6e-051.1e-05

4.5e-050.0001423.7e-05

7e-066e-056e-06

7e-066e-056e-06

7e-066e-056e-06

7e-066e-056e-06

3.8e-058.2e-053.1e-05

3.8e-058.2e-053.1e-05

3.8e-058.2e-053.1e-05

3.8e-058.2e-053.1e-05

0.4099770.4185510.4527070.3589840.4417969999999990.4270580.373223

0.1239790.1566840.186980.1811480.1870260.1524840.183056

0.0159720.0175270.0144990.016750.0134410.0158060.010338

0.0159720.0175270.0144990.016750.0134410.0158060.010338

0.00018.6e-059.8e-051.6e-05

3.9e-053.6e-054.3e-051.6e-05

6.1e-052.5e-055.5e-05

2.5e-05

0.000660.000741

0.000660.000741

0.0158370.0175270.0138390.016750.0125120.0156770.010298

2e-05

5.9e-05

0.0023540.0102820.0020810.0058170.0007160.0026610.01

3.2e-052.2e-058.5e-055.3e-05

0.000107

2e-050.00250.0028084e-061.7e-05

3.9e-051.9e-05

3.7e-051.4e-050.0015180.0017051.2e-052.9e-052.4e-05

1e-052.3e-05

5.3e-05

2.3e-056e-063e-05

3.7e-053e-062.4e-052.4e-05

0.0002860.0001030.0002170.0015050.0003210.000284

5e-06

2.9e-051e-052.7e-05

3.4e-054e-06

3.2e-05

2e-057e-062e-051.6e-05

6.1e-052.6e-056.8e-051.6e-05

4e-06

0.0129380.0071280.0075230.0049150.0110910.012432

4.2e-058e-06

3.5e-050.0001023.1e-052.4e-05

1.6e-05

8e-06

5.7e-05

3.5e-054.5e-053.1e-05

0.0068830.0110110.0102220.0138430.0026120.0053540.011795

0.0068830.0110110.0102220.0138430.0026120.0053540.011795

7e-050.0001115.9e-05

7e-050.0001115.9e-05

2.2e-053.4e-053.1e-05

2.2e-053.4e-053.1e-05

0.0003480.0003510.0003340.0016050.0002850.0003410.000313

0.0003480.0003510.0003340.0016050.0002850.0003410.000313

3.9e-050.0001130.0001880.0015050.0001333.6e-053.1e-05

7.3e-05

3.9e-050.0001130.0001880.0015052.3e-053.6e-053.1e-05

3.7e-05

7.6e-050.0001135.7e-051.5e-05

7.6e-050.0001135.7e-051.5e-05

0.0031690.0030340.0016230.0025080.0007180.0019980.002838

0.0029090.0030340.0014650.0025080.0006110.0017240.002796

0.000260.0001580.0001070.0002742.1e-05

2.1e-05

8.3e-055.3e-058e-05

8.3e-055.3e-058e-05

0.000324.4e-050.0001670.0002510.0003279.3e-05

5.8e-05

9.3e-05

7.6e-050.0001017.4e-05

0.0001830.0001674.8e-050.000193

6.1e-054.4e-054.4e-056e-05

0.0027560.0074690.007910.0082250.0009140.0024250.008505

0.0002010.0001796.2e-059e-057e-05

0.0002980.000335

0.000113

0.0001191.9e-050.000115

0.0018520.0072660.0009270.0050150.0003750.0016010.000628

0.0005840.0002030.0004220.003210.0004580.0006190.000524

0.0060840.006835

0.0391090.0343750.0671570.0674040.0578710.0502090.036207

0.0081170.0106050.0104140.0203630.0033550.0112580.013804

0.0017680.0001980.0012470.0015050.0003190.0016735e-05

0.0006410.0001820.0002660.0015050.0001370.000593

1e-06

3.4e-05

7e-06

0.0002031.6e-050.0001687e-060.000176

1e-06

0.0002350.0002091.7e-050.000235

1.3e-05

2e-06

0.0003750.0003225.6e-050.000349

9e-06

0.0003140.0002821.3e-050.000321.6e-05

5e-06

7e-06

2e-06

4.2e-05

0.0002760.0007090.0022060.0025086.5e-050.0002620.003283

0.0001171.9e-050.000131

7.9e-050.0007090.0011930.0025089e-067.6e-050.002336

0.0001970.000173.7e-050.000186

0.0007260.000816

0.0010690.0035310.00140.0029090.0001630.0010530.003091

0.0001541.5e-050.0001351.2e-050.00015

0.000129e-068.7e-05

0.000107

2.8e-052e-062.3e-05

2e-06

1e-06

0.0007670.0035160.0012650.0029093e-050.0007930.003091

0.0014110.0006280.0009470.0017050.0006760.0013990.000509

3.2e-05

0.000310.0002854.1e-050.00033

2.5e-05

0.0011010.0006280.0006620.0017050.0005410.0010690.000455

2.6e-05

2.8e-05

3.7e-05

4.6e-054.2e-055.8e-05

4.6e-054.2e-055.8e-05

4.3e-054.1e-057.6e-05

4.3e-054.1e-057.6e-05

7.9e-05

7.9e-05

6e-05

6e-05

0.0002310.0003940.0005880.0020060.0001860.0035480.001735

8.9e-050.0003940.0004680.0020060.0001780.003420.001726

0.0001420.000128e-060.0001289e-06

7.4e-05

5.7e-05

1.7e-05

0.0006590.0004580.0002070.0005963e-05

0.0002160.0001782.7e-050.000186

4.9e-056e-052.3e-05

0.0003030.000285.2e-050.000328

9.1e-056.8e-055.9e-05

3e-05

0.000103

0.000103

0.000101

0.000101

8.7e-05

8.7e-05

9.1e-05

9.1e-05

0.0003770.0004420.0003960.0016050.0001250.0003770.000563

2.1e-05

2e-05

5e-06

6.9e-052.4e-057.6e-05

0.0003080.0004420.0003960.0016053.8e-050.0003010.000563

2e-06

1.5e-05

7.9e-05

7.9e-05

9e-05

9e-05

4e-05

4e-05

6e-05

6e-05

2.8e-05

2.8e-05

5.6e-055.4e-05

5.6e-055.4e-05

4.5e-050.0002244.9e-05

2.1e-055.2e-052.6e-05

6e-062e-065e-06

9e-065.2e-059e-06

5.8e-05

5.8e-05

9e-062e-069e-06

0.0015590.0047030.002770.0081250.0002630.0015160.004543

0.0012920.0044160.0006310.0046140.0001980.001273

0.0001530.0002480.0002520.0020062.1e-050.0001580.003927

6.1e-050.0005483.7e-053.1e-050.000616

5.3e-053.9e-050.0013390.0015057e-065.4e-05

0.0003540.0001990.0001270.000363

0.0003540.0001990.0001270.000363

0.0002230.0002032.5e-050.000234

0.0002230.0002032.5e-050.000234

0.0001540.000173

0.0001540.000173

0.0001540.000173

0.0255590.0228820.0519160.0439320.0515220.0337430.021104

0.0016410.0002860.0018290.0008480.0015860.000515

6.9e-05

0.0001148.5e-050.0001153.3e-050.0001280.000152

0.0004382.9e-050.0003614.2e-050.000378

1.1e-05

06.9e-05

0.0001830.000206

1.9e-05

3.8e-05

4.8e-05

0.0004450.000499

0.0005665.4e-050.0004836.1e-050.00052

9e-06

0.0005237e-050.0002420.0001360.000561.9e-05

0.023630.0224390.0487990.0439320.0493740.0318770.020445

0.0011490.001291

8.9e-05

0.0222750.0168180.0049580.0220660.0012170.0305020.013637

0.0093770.010534

8e-06

0.0091960.01033

0.0012860.0007890.0005980.0056170.0001730.0013060.006557

6.9e-050.0048320.0006280.0162491.5e-056.9e-050.000251

0.0053260.005983

0.0162140.018214

0.0013530.00152

0.0011110.001248

0.0011110.001248

0.0002880.0001570.0001775.2e-050.000280.000144

3.4e-058.3e-054.2e-054e-05

0.0002547.4e-050.0001771e-050.000240.000144

8.4e-05

8.4e-05

8.4e-05

0.0021430.0008280.0022120.0031090.0010560.0023020.000685

0.0001410.0001450.000106

6.6e-05

3.7e-05

0.0001414.2e-050.000106

0.0005511.4e-050.000460.0001350.0004867.6e-05

0.0002371.4e-050.0002042.3e-050.0002217.6e-05

2.6e-05

0.0003140.0002568.6e-050.000265

0.0014510.0008140.0017520.0031090.0007760.001710.000609

0.0001950.0001752.5e-050.000197

0.0004070.0006320.000358

0.0005548.7e-050.0003231.7e-050.0005380.000217

0.000210.0006690.0003140.0031095e-060.00021

0.0004925.8e-050.0005339.7e-050.0007653.4e-05

0.0002144.8e-050.0001580.0002780.0001881.5e-05

9.6e-05

9.6e-05

0.000112

0.000112

0.0002144.8e-050.0001587e-050.0001881.5e-05

2e-06

1.6e-052.2e-051.3e-05

9e-066e-067e-06

5e-06

0.0001894.8e-050.0001583.5e-050.0001681.5e-05

0.0014110.0010970.0005780.0012676.9e-05

0.000240.0001927e-050.000196

0.000240.0001927e-050.000196

0.0002080.0001762.9e-050.000189

0.0002080.0001762.9e-050.000189

0.0004210.0003650.0002080.0004012.2e-05

4.1e-05

0.0002330.0001983.9e-050.0002141.6e-05

4.6e-05

2.8e-05

0.0001880.0001675.4e-050.0001876e-06

0.0004330.0003640.0001540.0003864.7e-05

4.1e-05

0.0002630.0002166e-050.000224

0.000170.0001485.3e-050.0001624.7e-05

0.0001099e-069.5e-05

0.0001099e-069.5e-05

0

0.000108

0.000108

0.0016651.2e-050.0012060.0008250.0014510.00053

0.0007861.2e-050.0004650.0003510.0006710.000119

2.3e-05

0.0003010.0002598.2e-050.0002822.4e-05

4.2e-05

8.6e-05

0.0001383e-050.000107

5e-05

8.7e-051.2e-052.6e-057.3e-057.9e-05

0.000260.0002061.2e-050.0002091.6e-05

0.0008340.0007410.0003920.0007290.000411

3.3e-056e-063.7e-051e-05

9.4e-0509.5e-05

0.0002130.000239

0.00040.000338.9e-050.0003340.000385

0.0002510.0001985.3e-050.00021.1e-05

5.6e-055e-066.3e-055e-06

2e-055e-052.3e-05

2e-055e-052.3e-05

2.5e-053.2e-052.8e-05

2.5e-053.2e-052.8e-05

0.0006930.0022370.0006430.0030090.0006220.0009120.002827

0.0002830.0001190.0002780.000209

0.0001790.0001190.0001530.000128

4e-055.9e-05

0.0001390.0001199.4e-050.000128

1.5e-053e-051e-05

01.9e-050

1.5e-051.1e-051e-05

6e-06

6e-06

5.2e-054.3e-055.5e-05

5.2e-054.3e-055.5e-05

3.7e-054.6e-051.6e-05

2.7e-05

3.7e-051.9e-051.6e-05

8.9e-05

5.2e-05

5.2e-05

3.7e-05

3.7e-05

0.000410.0022370.0005240.0030090.0002550.0007030.002827

0.000410.0022370.0005240.0030090.0002340.0007030.002827

1.8e-05

2e-0606e-06

7e-064e-061e-05

5e-068e-065e-06

0.0003960.0022370.0005240.0030090.0001970.0006820.002827

7e-06

2.1e-05

2.1e-05

0.000117

0.000117

4.9e-05

4.9e-05

6.8e-05

6.8e-05

7.5e-05

7.5e-05

7.5e-05

7.5e-05

8.7e-05

8.7e-05

8.7e-05

8.7e-05

0.0001070.000370.0003240.0001020.000416

0.0001070.000170.000102

1.6e-053.6e-051e-05

1.6e-053.6e-051e-05

4e-055.5e-054e-05

4e-055.5e-054e-05

2.9e-053.5e-053.1e-05

2.9e-053.5e-053.1e-05

2.2e-054.4e-052.1e-05

2.2e-054.4e-052.1e-05

0.000370.000416

0.000370.000416

0.000370.000416

6.7e-05

6.7e-05

2.8e-05

3.9e-05

8.7e-05

4.2e-05

4.2e-05

4.5e-05

4.5e-05

0.0137640.0330820.0526780.0285880.0727560.012510.079704

0.0029910.0064480.0022680.0114360.0015760.0027250.009192

0.0028660.0062950.0022680.0099310.0014360.0026110.00917

4e-06

2.6e-05

0.0001313.8e-050.000116

1.7e-05

0.000121.9e-050.000119

2.2e-05

3.2e-05

1.1e-05

6.8e-052.4e-051.2e-056.5e-057.2e-05

7e-06

6.3e-051.3e-056.8e-05

3.1e-05

5.4e-05

3.7e-055e-063.6e-05

8.1e-051.3e-052.1e-057.3e-051.6e-05

7.8e-053.9e-051.4e-057.7e-057.1e-05

1.1e-05

1e-058e-061e-05

0.0002050.0001040.0015057.2e-050.0001930.00017

3.7e-05

9.5e-05

2e-06

1.5e-05

0.0003840.000210.0002220.0015050.0001080.0003676.3e-05

6.2e-052e-065.8e-05

4e-06

2.7e-05

4.4e-05

4.5e-05

5e-06

0.0010940.0058710.0007960.0055170.0002290.0010490.008638

4.4e-053.4e-050.001250.0014041.1e-053.8e-059.5e-05

1e-05

0.00012

1.3e-05

4.7e-05

3.1e-05

8.7e-052e-058.3e-05

4e-06

1.6e-05

5e-06

3e-06

3.8e-05

8e-06

3e-06

5e-06

7e-05

0.0001093.3e-050.000102

7.9e-051.2e-057.1e-05

7e-06

4.5e-05

4.6e-051.1e-054.4e-05

1e-05

2.2e-05

1.6e-05

4.8e-051.1e-054.2e-05

1.4e-05

1.4e-05

0.0001250.0001530.0015050.0001260.0001142.2e-05

4.9e-05

0.0001250.0001530.0015057.7e-050.0001142.2e-05

0.0107730.0266340.050410.0171520.071180.0097850.070512

0.0099020.0258590.0498650.0155470.0703240.0089270.07033

0.0002110.000237

0.0005420.000609

0.0010160.0002760.0002050.0010950.000163

0.0054870.0240570.0031790.0155470.0008850.0043040.033454

2e-05

0.0003270.000368

0.0406210.06470.035245

3.2e-05

0.0007770.000873

5.9e-05

0.0010420.00117

0.0020950.0008630.0009230.0005670.002177

7.3e-05

0.0008480.000953

0.0006860.00077

0.0013040.0009390.0004330.0001770.0013519.4e-05

0.0007690.0005520.000420.0016050.0006980.0007580.000182

3.7e-051.8e-054.5e-05

2e-059e-062.1e-05

2.8e-05

4.4e-05

2.9e-059e-062.7e-05

2.8e-05

1e-05

0.0006830.0005520.000420.0016050.0005520.0006650.000182

0.0001020.0002230.0001250.0001580.0001

6.7e-05

0.0001020.0002230.0001252.2e-050.0001

6.9e-05

0.0089550.0053910.0058130.0082240.0078410.0084850.00428

3.7e-052.7e-051.8e-05

3.7e-052.7e-051.8e-05

3.7e-052.7e-051.8e-05

5.2e-053.6e-054.4e-05

5.2e-053.6e-054.4e-05

5.2e-053.6e-054.4e-05

7.4e-057.6e-056.8e-05

7.4e-057.6e-056.8e-05

3.1e-053.5e-052.4e-05

4.3e-054.1e-054.4e-05

0.0044690.003030.0033560.0058170.0038530.0042310.002594

0.0003160.0002130.000210.0016050.0002940.0002860.000173

1.1e-05

0.0002390.0002130.000210.0016050.0001740.000230.000173

1.1e-05

3.7e-051.3e-052.6e-05

3.5e-05

4e-051.4e-053e-05

1.5e-05

2.1e-05

4.2e-05

4.2e-05

0.0002590.0004970.0007580.0018050.0001490.0002090.000598

2.2e-050.0004970.000630.0018058e-061.9e-050.000598

0

1e-06

8e-06

3e-051.3e-053.5e-05

0.0002070.0001280.0001050.000155

1.4e-05

4.7e-05

4.7e-05

0.0038640.002320.0023880.0024070.0032540.0037090.001823

0.003850.002320.0023880.0024070.0032090.0037020.001823

1.4e-054.5e-057e-06

3e-056.7e-052.7e-05

3e-056.7e-052.7e-05

0.0040840.0023610.0022990.0024070.0031520.0038970.001686

0.0040840.0023610.0022990.0024070.0031520.0038970.001686

2.1e-051.6e-051.8e-05

2.1e-057e-061.7e-05

7e-061.8e-051.2e-05

3.1e-052.1e-052.8e-05

1.4e-055e-061.3e-05

1e-052e-069e-06

0.0036780.0023610.0022990.0024070.0029770.0035240.001686

2.9e-051.3e-053.5e-05

5e-052.7e-054.4e-05

6.2e-056e-065.2e-05

6e-062e-066e-06

5.4e-054.9e-05

1.3e-051.2e-051.2e-05

3.1e-052.5e-052.5e-05

3.8e-051.5e-053.5e-05

1e-054e-069e-06

9e-062e-069e-06

3.3e-05

3.3e-05

3.3e-05

0.0002230.0001580.0006220.000212

0.0002230.0001580.0006220.000212

7e-06

3.6e-054e-062.7e-05

3e-06

1.7e-05

2.3e-05

0.0001330.0001580.0005080.000134

6e-06

2.7e-05

5.4e-053e-065.1e-05

2.4e-05

1.6e-054.2e-051.5e-05

1.6e-054.2e-051.5e-05

1.6e-052.1e-051.5e-05

2.1e-05

2e-057.4e-051.7e-05

2e-057.4e-051.7e-05

2e-057.4e-051.7e-05

2e-057.4e-051.7e-05

0.0007370.0030430.0004850.0030090.000330.0006580.003116

0.0007340.0030430.0004850.0030090.0002690.0006570.003116

3.6e-05

3.6e-05

0.0007340.0030430.0004850.0030090.0002330.0006570.003116

2e-05

2.8e-05

1.1e-052.2e-051.2e-05

0.0007230.0030430.0004850.0030090.0001410.0006450.003116

2.2e-05

3e-066.1e-051e-06

2e-05

2e-05

3e-064.1e-051e-06

3e-061e-051e-06

3.1e-05

0.0016340.0009340.0017410.0018050.0003590.0014830.001757

0.000850.000955

0.000850.000955

0.000850.000955

0.0016340.0009340.0008910.0018050.0003590.0014830.000802

0.0001056.4e-057.5e-05

0.0001056.4e-057.5e-05

0.0013590.0009340.0007740.0018050.0002140.0013010.000786

0.0010220.0008960.0003510.0018050.0001060.0009880.000483

9e-06

0.0001350.0001152.3e-050.000124

0.0001650.000185

0.0002023.8e-050.0001431.3e-050.0001890.000118

6.3e-05

0.000170.0001178.1e-050.0001071.6e-05

0.000170.0001178.1e-050.0001071.6e-05

0.0002220.0007130.0011920.00019

0.0002220.0007130.0011920.00019

0.000170.0001240.0002770.000139

1.2e-055.3e-051.1e-05

4.1e-05

6.4e-05

0.000140.0001248.4e-059.4e-05

1.8e-053.5e-053.4e-05

6e-05

6e-05

7.2e-05

7.2e-05

5.4e-05

5.4e-05

5.2e-052.7e-055.1e-05

5.2e-052.7e-055.1e-05

0.0005890.000702

4.1e-05

0.0005890.000661

0.0001620.0001170.0004210.000135

0.0001620.0001170.0004210.000135

1.2e-050.0001961.5e-05

7e-05

5.2e-05

1.2e-057.4e-051.5e-05

0.0001299.3e-050.0001

0.0001299.3e-050.0001

2.1e-050.0001170.0001322e-05

2.1e-050.0001170.0001322e-05

0.0009594.9e-050.0017820.0026460.0007844.7e-05

9.7e-050.0001339.1e-05

9.7e-050.0001339.1e-05

4e-06

5.6e-05

6.9e-057.1e-056.2e-05

2.8e-052e-062.9e-05

3.8e-05

3.8e-05

3.8e-05

4.1e-054.6e-053.2e-05

4.1e-054.6e-053.2e-05

4.1e-054.6e-053.2e-05

0.0001660.0002230.0001253.2e-05

0.0001018.8e-058e-053.2e-05

2.6e-052.7e-051.8e-051.6e-05

3.4e-053.5e-053.1e-051.6e-05

4.1e-052.6e-053.1e-05

4.5e-05

4.5e-05

6.5e-053.2e-054.5e-05

6.5e-053.2e-054.5e-05

5.8e-05

5.8e-05

0.0005894.9e-050.0017820.0021080.0004831.5e-05

7.6e-051.3e-054e-06

7.6e-051.3e-054e-06

0.0001660.0001290.0001210.000154

0.0001660.0001290.0001210.000154

4.7e-05

4.7e-05

0.000294.9e-050.0016530.0018210.0002771.5e-05

0.0001250.00014

0.0001170.000132

0.0010540.001184

0.0001250.000141

0.000294.9e-050.0002320.0002240.0002771.5e-05

5.7e-051.3e-054.8e-05

5.7e-051.3e-054.8e-05

9.3e-05

9.3e-05

6.6e-059.8e-055.3e-05

6.6e-059.8e-055.3e-05

3.3e-05

6.6e-053.6e-055.3e-05

2.9e-05

0.0205190.0438120.0203910.0338020.0082140.0420780.029286

0.0202840.0438120.0202660.0338020.0079460.0418990.029286

0.000690.0005260.0001130.0005179.7e-05

0.0002990.0002246.4e-050.0002175e-05

0.0003910.0003024.9e-050.00034.7e-05

6.7e-05

1.7e-05

2.2e-05

2.8e-05

6.8e-050.0001570.0002530.0015054.9e-056.6e-057.9e-05

6.8e-050.0001570.0002530.0015054.9e-056.6e-057.9e-05

0.0002134.8e-050.0001710.0001050.000160.000237

4.3e-05

2e-05

4.4e-05

0

0.0002134.8e-050.0001711.7e-050.000160.000217

1e-06

0.0193130.0436070.0193160.0322970.0076120.0411560.028873

0.0011490.0041430.0006820.0029090.0002520.0012150.000631

0.0022480.002526

0.0023910.0006050.0008320.003410.0003870.002518

0.0005760.0001650.0002020.0001210.000616

0.0132550.0359270.0102830.0164490.0035030.0336980.024847

6e-05

0.0010420.0003190.0004360.0024070.0001950.0010860.000592

5e-060.0024110.0027082e-067e-06

8.1e-05

0.0008610.0013670.0009390.0019060.0004780.0019730.001482

3.4e-050.0010810.0012830.0025087e-064.3e-050.001321

0.0002350.0001250.0002680.000179

0.000103

4.2e-05

2.5e-05

3.6e-05

7.4e-054.8e-055.5e-05

7.4e-054.8e-055.5e-05

0.0001610.0001254.4e-050.000124

0.0001610.0001254.4e-050.000124

7.3e-05

7.3e-05

0.0142430.0052230.0103690.0047140.0180440.0137610.003283

5.4e-05

5.4e-05

5.4e-05

0.0138910.0052230.0103690.0047140.0175080.0134480.003283

0.0001540.000180.000117

8.2e-059.1e-056.5e-05

7.2e-058.9e-055.2e-05

0.0001580.000177

0.0001580.000177

0.0001560.0001540.0002120.00016

0.0001560.0001540.0002120.00016

8.8e-05

8.8e-05

0.013450.0052230.0099390.0047140.0167010.0130520.003283

0.0127220.0052230.0056180.0047140.0120040.0126460.003267

0.0003390.0002210.000196

0.0036550.004106

0.0003890.0002460.0001720.0004061.6e-05

0.0001990.000223

0.0001310.0001180.000150.000119

0.0001310.0001180.000150.000119

5.9e-050.0001235e-05

5.9e-050.0001235e-05

6.3e-05

5.9e-056e-055e-05

5.5e-05

5.5e-05

5.5e-05

0.0002930.0003040.000263

6e-055.2e-055e-05

6e-055.2e-055e-05

8e-055.4e-057.3e-05

8e-055.4e-057.3e-05

4e-057.7e-053.5e-05

1e-051.7e-057e-06

1.2e-054.3e-051.2e-05

1.8e-051.7e-051.6e-05

6.3e-05

6.3e-05

0.0001135.8e-050.000105

0.0001135.8e-050.000105

0.0016690.0008320.0039170.0052150.0018580.0015360.000492

1.3e-055.1e-051e-05

1.3e-055.1e-051e-05

3e-062.7e-055e-06

3e-062.7e-055e-06

1e-052.4e-055e-06

1.1e-05

1e-051.3e-055e-06

0.0016480.0008320.0039170.0052150.0017660.0015180.000492

0.0015060.0008320.0008810.0018050.0013990.001430.000492

3e-062.8e-053e-06

3e-062.8e-053e-06

2.7e-054.5e-052e-05

1.9e-052.2e-051.3e-05

8e-062.3e-057e-06

0.001460.0008320.0008810.0018050.0013070.0013990.000492

1e-067e-061e-06

0.0014370.0008320.0008810.0018050.0011780.0013780.000492

1e-062.8e-050

2.4e-05

3e-061.7e-052e-06

3e-05

9e-069e-06

000

1.4e-05

2e-068e-062e-06

7e-061e-067e-06

1.6e-051.9e-058e-06

1.6e-051.9e-058e-06

1.6e-053.2e-051.4e-05

1.6e-053.2e-051.4e-05

1.6e-053.2e-051.4e-05

0.0001260.0030360.003410.0003357.4e-05

6.6e-056.2e-052.9e-05

7e-061.1e-057e-06

1.4e-052.2e-051.3e-05

4.5e-052.9e-059e-06

5.2e-050.0030360.003410.0002183.9e-05

1.1e-05

5e-062e-065e-06

1.8e-05

6e-063.5e-054e-06

1.2e-05

8e-06

4e-06

1e-061.4e-05

4e-06

1e-051.6e-05

5.2e-05

1.3e-05

2.8e-053e-062.8e-05

2.1e-05

1e-060.0030360.003411e-061e-06

1e-064e-061e-06

8e-065.5e-056e-06

1e-061e-052e-06

6e-06

5e-06

1e-05

1.1e-05

7e-061.3e-054e-06

8e-064.1e-058e-06

8e-064.1e-058e-06

8e-064.1e-058e-06

8e-064.1e-058e-06

7e-059e-056.6e-05

7e-059e-056.6e-05

7e-059e-056.6e-05

7e-059e-056.6e-05

1.4e-054e-051.7e-05

1.3e-052.2e-051.1e-05

4.3e-052.8e-053.8e-05

0.0725680.0465080.1241280.0604820.0940580.069550.094741

0.0334790.020850.0774890.0294880.0517150.0324080.048688

0.0049020.0022590.0024820.0040120.0024220.0045890.001652

0.0001076.4e-059.1e-05

0.0001076.4e-059.1e-05

8e-050.000225.4e-05

5.8e-05

8.2e-05

8e-058e-055.4e-05

0.000174

9.2e-05

8.2e-05

0.0010510.000270.0007670.0017050.0007620.0009650.00028

0.0010510.000270.0003260.0017050.0001530.0009650.00028

0.0004410.000496

0.000113

0.0036640.0019890.0017150.0023070.0012020.0034790.001372

4.4e-05

3.4e-05

0.0036640.0019890.0017150.0023070.0011240.0034790.001372

0.0008270.02010.009360.000690.013481

0.0001037.7e-058.9e-05

4.2e-057.7e-053.7e-05

6.1e-055.2e-05

0.019560.0087820.013367

0.0076610.008606

5.7e-05

0.0118990.013367

0.000119

5.3e-05

5.3e-05

4.3e-05

4.3e-05

0.0002430.0001999.7e-050.000207

0.0002430.0001999.7e-050.000207

0.0003010.0001970.0001610.0002471.7e-05

0.0003010.0001970.0001610.0002471.7e-05

0.000180.0001448.4e-050.0001479.7e-05

9.7e-05

0.000180.0001448.4e-050.000147

6.3e-05

6.3e-05

0.0162370.0093920.0302270.010330.0304390.0163330.009475

0.0017440.0009010.0009930.0016050.0007430.0017140.000707

1.9e-05

0.000112

0.0012640.0004940.0005750.000390.0012312.8e-05

0.0001140.000128

0.000480.0004070.0003040.0016050.0001130.0004830.00066

0.0004910.0001220.0003480.0004471e-06

3.7e-05

0.0001390.0001223.8e-050.000135

3.3e-052.8e-053e-05

9.3e-05

0.0001066.1e-059.5e-051e-06

0.0001214e-050.000112

9.2e-055.1e-057.5e-05

0.0001350.0002720.0004940.000119

2.7e-05

0.000160.000179

2.6e-05

1.6e-05

0.0001350.0001121.5e-050.000119

2.8e-05

8.7e-05

9.1e-05

2.5e-05

0.0031340.0033030.0021670.006920.0014820.0031070.003284

5e-06

0.0001236.3e-050.000129

0.0001081.2e-050.000111

0.0002863.2e-050.0002524.1e-050.0002826.1e-05

1e-05

0.0001310.0002680.0002190.0018051.1e-050.0001270.001864

2.1e-05

0.000114

0.0011490.0009250.0006770.0018050.0003020.0011180.000853

4e-06

2.2e-05

2.8e-05

0.0001011.4e-053e-060.0001060.000103

1.6e-05

6.8e-052.4e-058e-066.9e-052.3e-05

2.3e-055e-062.4e-056e-06

1.7e-05

6.1e-05

2.4e-05

7.7e-05

3.8e-058e-063.6e-05

5.4e-051.4e-051.1e-055.8e-05

9.3e-053.2e-059.6e-057e-06

8e-050.0018490.0003840.0018051.3e-057.9e-050.000171

1e-06

0.0002020.0001782.1e-050.0001981.8e-05

3.5e-05

1.2e-05

4.6e-053.2e-056e-064.6e-051.7e-05

1e-06

2.3e-05

6e-05

4.9e-05

0.0001330.0001153.8e-050.000125

2.1e-05

5.7e-05

0.0001452.6e-050.0001322.7e-050.000153

0.0003540.0001190.000210.0015050.0002230.000350.000161

0.0016150.0005410.0019210.0018050.0013570.0015390.000522

0.0015820.0013020.0013140.001522

3.3e-050.0005410.0006190.0018052.8e-051.7e-050.000522

1.5e-05

0.0091180.0046470.0247520.0260150.0094070.004961

0.0018870.0002770.0006240.001290.0019320.000591

0.0021940.0004280.0007780.0012260.0022740.000599

0.0027870.0011710.001670.0028750.001747

0.002250.0027710.0015830.0009230.0023260.002024

0.0190780.021431

0.0010190.001145

0.0120670.013555

0.0120670.013555

0.0120670.013555

0.0104510.008530.008940.0109330.008210.009970.009803

0.0005057.8e-050.0004380.0004640.0004716.4e-05

0.0001510.0001239.7e-050.0001284.7e-05

0.0003547.8e-050.0003150.0003670.0003431.7e-05

0.0001654.7e-050.0001385.2e-050.000147

0.0001654.7e-050.0001385.2e-050.000147

0.000122

4.7e-05

7.5e-05

0.000133

5.7e-05

7.6e-05

2.5e-055.6e-051.9e-05

2.5e-055.6e-051.9e-05

8.5e-057.7e-057.3e-05

8.5e-057.7e-057.3e-05

0.0001940.0001620.0001710.000182

0.0001940.0001620.0001710.000182

7.7e-05

4e-05

3.7e-05

0.0001254.7e-050.000112

0.0001254.7e-050.000112

0.000102

0.000102

0.0002790.0001697.7e-050.0002380.000125

0.0002790.0001697.7e-050.0002380.000125

0.0001382.4e-054.9e-050.000144

6.3e-052.4e-051.2e-057e-05

2.1e-05

7.5e-051.6e-057.4e-05

0.0023960.0020930.0020780.0037110.0009630.0023210.000735

4e-05

0.0001254.3e-050.000112

0.0004650.0002040.0003370.0018050.0002940.000449

0.0015340.0010580.0008470.0019060.0004790.0014980.0007

9.5e-050.0008310.000742.3e-059.3e-05

0.0001770.0001548.4e-050.0001693.5e-05

0.0065390.0062880.0059550.0072220.005820.0062630.008879

0.0065390.0062880.0059550.0072220.0057420.0062630.008879

7.8e-05

0.0010620.0006690.0036730.0042130.0012840.0008260.000722

0.0005590.0006170.0031920.0042130.0005180.0005060.000643

9.6e-05

0.0001184.1e-050.0001121.6e-05

2.1e-052.8e-050.0022320.0025086e-062.2e-051.5e-05

1.3e-050.00051601.2e-050.00058

5.9e-05

8.9e-05

3.5e-05

7e-06

3.1e-05

3.1e-05

0.0002290.0001858.9e-050.0001891.6e-05

0.0001780.0005890.0002590.0017053.4e-050.0001711.6e-05

0.0001120.000126

0.0001120.000126

4.9e-058.9e-054.3e-05

4.9e-058.9e-054.3e-05

4.7e-050.0001242.8e-05

7.7e-05

4.7e-054.7e-052.8e-05

0.0003785.2e-050.0002590.0001210.0002367.9e-05

3.9e-05

0.0003785.2e-050.0002598.2e-050.0002367e-05

9e-06

2.9e-057.6e-051.3e-05

2.5e-055.6e-051e-05

4e-062e-053e-06

0.000110.000124

0.000110.000124

0.000106

0.000106

0.0021720.000370.0023540.0024870.0019570.00042

0.0015910.000370.0020680.0021510.0014760.000388

0.0008570.000280.0016420.0018370.0007980.000241

0.0008570.000280.000350.0003850.0007980.000241

0.0012920.001452

0.0007349e-050.0004260.0003140.0006780.000147

1.5e-05

0.0005219e-050.0002478e-060.0004750.000147

0.0002130.0001790.0001890.000203

0.000102

0.0004570.0002860.0002270.0003823.2e-05

0.0002730.000150.0001050.0002171.6e-05

0.0002730.000150.0001050.0002171.6e-05

0.0001840.0001360.0001220.0001651.6e-05

0.0001840.0001360.0001220.0001651.6e-05

0.0001240.0001099.9e-05

0.0001240.0001099.9e-05

0.0001240.0001099.9e-05

0.0005360.0001650.000260.0015050.0004550.0005016.4e-05

0.0005360.0001650.000260.0015050.0004550.0005016.4e-05

9.8e-050.0001418.6e-05

2.5e-055.3e-052.3e-05

1e-052.1e-057e-06

9e-062.2e-057e-06

1.9e-052.8e-052e-05

3.5e-051.7e-052.9e-05

0.0003090.0001650.000260.0015050.0002670.0002936.4e-05

0.0003090.0001650.000260.0015050.0002670.0002936.4e-05

5.2e-051.8e-054.7e-05

5.2e-051.8e-054.7e-05

7.7e-052.9e-057.5e-05

7.7e-052.9e-057.5e-05

0.0092630.0098560.0282550.0186570.0116220.0087690.032796

0.0009410.0002050.0005830.0015050.0005680.0007630.000288

0.0002020.0001599.5e-050.000163.4e-05

0.0002020.0001599.5e-050.000163.4e-05

0.0005660.0002050.0003030.0015050.0003730.0004910.000179

0.0005660.0002050.0003030.0015050.0003730.0004910.000179

0.0001730.0001210.00010.0001127.5e-05

0.0001730.0001210.00010.0001127.5e-05

0.0083220.0096510.0276720.0171520.0110540.0080060.032508

0.0003390.00038

0.0003390.00038

0.0083220.0096510.0237350.0171520.0095790.0080060.029546

0.0021850.002454

0.0002370.000266

0.0028010.003146

0.0046120.0018910.0012840.0106320.0018780.0045820.000389

0.0001160.00013

0.0017850.0049010.001261

0.0070570.007928

0.0015060.0063340.0006220.0042130.0001450.0019590.007209

0.0029780.003345

0.0006640.000746

0.0022040.0014260.000670.0023070.0002010.0014650.001378

0.001670.001876

0.0016660.001872

0.0014190.001594

0.0014190.001594

0.0019040.0011660.000988

0.0008660.000973

0.0010380.0011661.5e-05

0.0002750.000309

0.0002750.000309

0.0271180.0152670.015770.0108320.0277790.0259150.012773

0.0001450.0003270.000121

0.0001190.0002149.6e-05

3.5e-054.4e-052.7e-05

1.9e-05

1.2e-051.3e-051.7e-05

4.7e-05

3.2e-054.5e-054.1e-05

4e-054.6e-051.1e-05

2.6e-050.0001132.5e-05

5.7e-05

2.6e-055.6e-052.5e-05

0.0003060.0002960.000222

6.1e-057.7e-054.3e-05

6.1e-057.7e-054.3e-05

0.0001339.3e-050.000101

0.0001339.3e-050.000101

0.0001120.0001267.8e-05

0.0001120.0001267.8e-05

0.0006860.0005180.000348

9.6e-05

9.6e-05

0.0006860.0004220.000348

0.000310.000348

0.0003760.000422

0.0256520.0150310.0145870.0108320.0231490.0245960.012012

0.0255160.0150310.0145870.0108320.0228730.0244880.012012

4.1e-059.4e-053.9e-05

0.0254750.0150310.0145870.0108320.0227790.0244490.012012

6.3e-056.2e-054.5e-05

6.3e-056.2e-054.5e-05

0.000137

7.1e-05

6.6e-05

7.3e-051.7e-056.3e-05

3.2e-059e-062.6e-05

4.1e-058e-063.7e-05

6e-05

6e-05

0.0010150.0002360.0004970.0034890.0009760.000413

0.0010150.0002360.0004970.0034890.0009760.000413

0.0010150.0002360.0004970.0034890.0009760.000413

0.2024520.2093010.1323250.1055190.1487060.1946210.091142

6.3e-050.0003885.2e-05

6.3e-050.0003885.2e-05

2.7e-050.0002022.2e-05

1.9e-059.7e-051.7e-05

8e-060.0001055e-06

3.6e-050.0001863e-05

2e-058.9e-051.6e-05

1.6e-059.7e-051.4e-05

0.0001170.000254

0.0001170.000132

0.0001170.000132

0.0001170.000132

0.000122

0.000122

0.000122

0.0896050.0605940.0525730.0469410.0537520.0863040.032527

7.9e-05

7.9e-05

7.9e-05

0.0002368.4e-050.0003770.0015050.0004910.0002154.7e-05

0.0001788.4e-050.0002090.0015050.0002110.0001663.1e-05

0.0001788.4e-050.0002090.0015050.0002110.0001663.1e-05

0.0001680.000188

0.0001680.000188

5.8e-059.2e-054.9e-051.6e-05

5.8e-059.2e-054.9e-051.6e-05

0.0714140.0414490.0394280.0253760.0446140.0686290.018727

0.0001990.000170.0001790.000104

2.3e-05

0.0001140.0001070.0001088.1e-05

8.5e-056.3e-057.1e-05

3.5e-053.3e-052.9e-057e-05

3.5e-053.3e-052.9e-057e-05

0.0685070.0393250.0366430.0214640.0417520.0658630.009073

0.0003287.9e-050.000438.4e-050.0003390.004656

0.0001583.3e-050.0001437e-050.0001659.7e-05

0.0673610.0390080.0349810.0214640.0404730.06467

0.0001363e-050.00010.0001447.7e-05

0.0003750.000421

0.0001543.2e-050.000147.2e-050.00016

0.0001440.000161

0.000370.0001430.000230.0001460.0003850.004243

0.00020.000225

0.0001580.000364

9.5e-05

0.0001580.000178

9.1e-05

1.9e-05

1.9e-05

0.0026730.0021240.0026270.0039120.0022950.0025580.009461

0.0026730.0021240.0026270.0039120.0022950.0025580.009461

8.8e-050.0001159.6e-05

8.8e-050.0001159.6e-05

3e-066e-062e-06

3e-062e-061.2e-05

6e-061.3e-058e-06

8e-062.2e-054e-06

5e-066e-065e-06

1.1e-056e-061.1e-05

2.7e-05

1.5e-055e-061.9e-05

3.7e-052.8e-053.5e-05

0.0008050.0094360.0029680.010130.0002230.0007690.009795

0.0007420.0094360.0029680.010130.0001180.0007290.009778

4e-060.0009720.0008653e-064e-06

1.6e-052.5e-051.7e-05

5e-065e-065e-06

4.4e-05

0.0006990.0076560.0013840.010138e-060.0006880.009778

8e-061.7e-054e-06

2e-061e-052e-06

3e-061e-064e-06

0

5e-060.0008080.0007195e-065e-06

6.3e-050.0001054e-051.7e-05

1.7e-05

6.3e-053.3e-054e-05

7.2e-05

0.000204

9.1e-05

4.8e-05

4.3e-05

0.000113

0.000113

0.0010640.0013760.0008020.0021060.0007910.0010170.001416

0.0010640.0013760.0008020.0021060.0007910.0010170.001416

8.3e-057.5e-057.2e-05

0.000119

0.0006960.0013450.0006770.0021060.0003320.0006670.00139

8.2e-057e-058.1e-052.6e-05

0.0001423.1e-050.0001254.8e-050.000139

1.6e-05

6.1e-053.2e-055.8e-05

9.9e-05

0.0047590.0022420.0035910.0017050.0032580.0047940.000921

9.4e-051.5e-05

9.4e-05

1.5e-05

0.0001810.000203

0.0001810.000203

0.0046880.0022420.0032710.0017050.0028050.0047350.000831

0.0008760.0003570.0003750.0002520.000890.000355

0.0020470.0011770.0012560.0017050.0008870.002031

0.0009820.0003760.0003870.0002470.0010070.000357

0.0009950.001117

0.0007830.0003320.0002580.0003020.0008070.000119

7.1e-050.0001390.0001565.9e-057.5e-05

7.1e-050.0001390.0001565.9e-057.5e-05

0.0104320.0060070.0050260.0061190.0027780.0100190.001614

6.5e-05

6.5e-05

7.4e-05

7.4e-05

0.0001513.3e-050.0001750.0001365.4e-05

5.2e-053.8e-054.5e-05

4.9e-05

8.5e-052.2e-057.3e-057.9e-053.2e-05

1.4e-051.1e-051.5e-051.2e-052.2e-05

4.5e-05

4.5e-05

5.2e-050.0001284.4e-05

4e-069e-062e-06

1.9e-052.8e-051.7e-05

4.3e-05

7e-062.2e-057e-06

2.2e-052.6e-051.8e-05

0.0100070.0059740.0042320.0061190.0020780.0096240.000667

8.8e-051.4e-054.4e-050.0001043.3e-05

6.3e-054.1e-055.7e-05

8.7e-05

2.6e-05

2.1e-05

0

0.0092310.0054160.0036080.0039120.0017480.00886

0.0006250.0005440.0006240.0022071.9e-050.0006030.000634

0

7.3e-05

0

1.9e-05

0.0002220.0007940.0002130.0002150.000893

4.3e-050.0001571.5e-054.8e-050.000177

7.8e-050.0003123.6e-057.2e-050.000351

4.8e-050.0001854.8e-054.8e-050.000208

0.000140.000157

5.3e-055.1e-054.7e-05

6.3e-05

0.0002280.0006230.000212

2.9e-05

2.9e-05

0.0001180.0001780.000107

4.9e-056.9e-054.1e-05

3e-055.7e-053.2e-05

3.9e-055.2e-053.4e-05

0.000103

0.000103

6.1e-057.7e-055.8e-05

6.1e-057.7e-055.8e-05

0.000134

6.5e-05

6.9e-05

3.4e-05

3.4e-05

4.9e-056.8e-054.7e-05

4.9e-056.8e-054.7e-05

6.3e-057.8e-056e-05

6.3e-057.8e-056e-05

6.3e-057.8e-056e-05

0.0004420.0003810.0003940.000419

0.0001280.0001139e-050.000125

0.0001280.0001139e-050.000125

0.000160.000130.0001290.000154

0.000160.000130.0001290.000154

0.0001540.0001380.0001750.00014

0.0001540.0001380.0001750.00014

7.4e-050.0001047.4e-057e-06

7.4e-050.0001047.4e-057e-06

7.4e-050.0001047.4e-057e-06

0.000490.0009850.0010670.0004740.000483

0.000490.0009850.0010670.0004740.000483

0.0001720.0009850.000690.0001680.00045

0.0001720.0001519.3e-050.000168

0.0004010.00045

0.0001650.000186

0.0002680.000301

0.00011

0.0001740.0001480.0001721.6e-05

3.5e-05

7e-053.9e-056.9e-051.6e-05

2.9e-053.8e-053.5e-05

7.5e-053.6e-056.8e-05

0.0001047.9e-059.8e-05

0.0001047.9e-059.8e-05

8e-05

8e-05

4e-057e-053.6e-051.7e-05

4e-057e-053.6e-051.7e-05

1.1e-054.9e-057e-06

1.1e-054.9e-057e-06

1.1e-054.9e-057e-06

1.1e-054.9e-057e-06

0.0034140.0024970.0032550.0025080.0043790.003270.001988

0.0029680.0024590.002980.0025080.0035640.0028370.001988

0.0002331.3e-050.000170.0002321.2e-05

5e-05

0.0001186.5e-050.000121.2e-05

0.0001151.3e-053.7e-050.000112

1.8e-05

0.0001380.0001220.0001350.000139

0.0001380.0001220.0001350.000139

0.0001970.0001130.0002290.000174

5.2e-05

0.0001075e-059.5e-05

9e-050.0001130.0001277.9e-05

2.2e-059.5e-051.4e-05

2.2e-059.5e-051.4e-05

0.0001460.000164

0.0001460.000164

0.0001540.000173

0.0001540.000173

0.0002370.000266

0.0002370.000266

5.3e-050.0001120.0001264.6e-05

5.3e-050.0001120.0001264.6e-05

0.0023250.0024460.0020960.0025080.0022060.0022320.001976

0.0021340.0024460.0019340.0025080.0020350.0020480.001976

0.0001910.0001620.0001710.000184

0.0004463.8e-050.0002750.0008150.000433

0.0001310.000147

0.0001310.000147

0.0001480.0001440.0002090.000143

0.0001480.0001440.0002090.000143

6.8e-056.4e-056.2e-05

6.8e-056.4e-056.2e-05

9.7e-053.8e-059.8e-050.000101

9.7e-053.8e-059.8e-050.000101

7e-050.000126.2e-05

7e-056.3e-056.2e-05

5.7e-05

6.1e-053.7e-056.2e-05

3.7e-052e-053.7e-05

2.4e-051.7e-052.5e-05

2e-060.000143e-06

2.3e-05

0.000114

2e-063e-063e-06

0.0011859.8e-050.0018790.0034110.0026590.0011950.000127

0.0010639.8e-050.0017250.0019060.0023280.0010770.000127

3.4e-05

3.4e-05

0.0003919.8e-050.0014960.0019060.0013440.0003974.7e-05

1.7e-05

3.3e-05

3.5e-05

5.4e-05

0.0003919.8e-050.0004770.0019066e-050.0003974.7e-05

0.0010190.001145

5e-05

5e-05

0.0001350.0001510.00012

7.7e-053.9e-056.5e-05

8e-05

1.5e-05

5.8e-051.7e-055.5e-05

0.0002650.0002290.0003720.0002496.4e-05

7.4e-05

0.0001310.0001156.9e-050.000127

5.4e-05

1.6e-05

0.0001340.0001145.5e-050.0001223.2e-05

1.6e-05

6.9e-05

5.1e-05

2e-054.8e-056.5e-05

2e-054.8e-056.5e-05

4.3e-05

4.3e-05

0.0002520.0002860.0002461.6e-05

2.6e-05

5.5e-05

8.1e-052.2e-058e-051.6e-05

9.3e-051.4e-059.1e-05

5.4e-05

1.9e-05

2.2e-05

7.8e-054.7e-057.5e-05

2.7e-05

0.0001220.0001540.0015050.0003310.000118

0.000178

2.7e-05

3.8e-05

3.1e-05

1.3e-05

4.1e-05

2.8e-05

0.0001220.0001540.0015056.4e-050.000118

2.3e-05

0.0001220.0001540.0015054.1e-050.000118

4e-05

4e-05

4.9e-05

2.8e-05

2.1e-05

0.0044110.005770.007010.0106330.0031630.0042380.005359

0.0035210.0053090.004360.0086270.0026290.0033950.004637

6e-064.1e-055e-06

2.7e-05

6e-061.1e-055e-06

03e-06

0.0021870.0019650.0024360.0057180.000770.0021070.001166

2e-0601e-06

0.0019290.0019650.0014970.0020060.0018590.0011

5e-065.4e-055e-06

0.0002410.0002440.0015050.0001830.0002316.6e-05

02.6e-050

1e-050.0006950.0022070.0004741.1e-05

3.3e-05

0

0.0013180.0033440.0019240.0029090.0017830.0012670.003471

1e-060.0001947.4e-051e-060.000218

08e-060

0.0013170.0033440.001730.0029090.0017010.0012660.003253

1e-053.5e-051.6e-05

3e-061.2e-058e-06

1e-069e-061e-06

1e-061e-061e-06

5e-069e-066e-06

4e-06

9.2e-05

9.2e-05

9.2e-05

5.2e-058.2e-053.3e-05

5.2e-058.2e-053.3e-05

5.2e-058.2e-053.3e-05

0.0008380.0004610.002650.0020060.000360.000810.000722

0.0008350.0004610.002650.0020060.0003440.0008050.000722

1.5e-050.0017860.0020067e-061.6e-05

0.0001123.1e-053.9e-053.9e-05

1.7e-050.000330.0002334e-061.7e-050.000217

0.0001290.0001223.5e-050.000147

4e-062e-066e-06

1e-054e-061.1e-05

1.1e-052.1e-057e-06

3e-062e-063e-06

9e-063e-061.1e-05

4e-061e-061e-06

0.0003060.0002826.8e-050.0003283.4e-05

6e-063e-068e-06

0.0002270.000255

7.4e-051.7e-057.7e-057.2e-05

9.5e-05

1.7e-056e-061.8e-05

4.9e-051e-055e-05

1e-056e-063e-06

6e-064e-066e-06

1.6e-050.0001311e-051.8e-050.000105

3.7e-051.5e-053.9e-05

3e-061.6e-055e-06

3e-061.6e-055e-06

2.3e-050.00011.8e-05

2.3e-050.00011.8e-05

2.3e-050.00011.8e-05

2.3e-050.00011.8e-05

0.1030840.1403420.0665060.0420260.0825810.0989110.050627

1.1e-05

1.1e-05

1.1e-05

0.000110.0002079.6e-056.2e-05

9e-068.1e-051.7e-05

9e-068.1e-051.7e-05

6.2e-05

6.2e-05

5.3e-056.3e-054.5e-05

5.3e-056.3e-054.5e-05

4.8e-056.3e-053.4e-05

4.8e-056.3e-053.4e-05

0.1029740.1403420.0665060.0420260.0823740.0988150.050554

4.5e-050.0001063.8e-05

4.5e-050.0001063.8e-05

0.0010110.0013220.0023550.0022070.0007410.0009790.002876

0.0013280.001492

0.0001510.0001250.0001230.000153

0.000860.0013220.0009020.0022070.0006180.0008260.001384

4.5e-054e-054.1e-05

4.5e-054e-054.1e-05

1.9e-051.6e-05

1.9e-051.6e-05

0.0001330.00015

0.0001330.00015

0.000109

0.000109

7.8e-05

7.8e-05

4e-050.0001183.9e-052.1e-05

2.3e-053.5e-052.2e-051e-05

1.7e-058.3e-051.7e-051.1e-05

0.0001070.0001180.000101

0.0001070.0001180.000101

0.0001780.000199

0.0001780.000199

0.0001430.00016

0.0001430.00016

0.000160.0001780.000164

3.4e-05

4.7e-053.5e-054.8e-05

0.0001130.0001090.000116

0.0001140.000128

0.0001140.000128

0.0001460.000164

0.0001460.000164

4.5e-05

4.5e-05

3.4e-05

3.4e-05

1.1e-05

1.1e-05

1e-05

1e-05

1e-05

1e-05

9.5e-057.6e-059.2e-05

9.5e-057.6e-059.2e-05

5e-06

5e-06

0.1011860.139020.0631630.0398190.0792360.0970950.047571

2.5e-050.0010050.0011292.6e-05

0.1011610.139020.0618350.0398190.0777440.0970690.047571

0.0003230.000363

7.4e-05

7.4e-05

0.0002220.0001180.0001920.0002121.6e-05

7.2e-058.1e-056.6e-05

0.000150.0001180.0001110.0001461.6e-05

0.0001560.000279

0.000104

0.0001560.000175

0.000118

0.000118

4.4e-056.5e-053.8e-05

2.2e-057e-061.2e-05

2.2e-055.8e-052.6e-05

0.0001660.0003140.0001523.1e-05

0.0001660.0003140.0001523.1e-05

1.6e-050.0001151.3e-05

1.6e-050.0001151.3e-05

1.6e-05

1.6e-05

0.0001250.0001010.0001161.5e-05

0.0001250.0001010.0001161.5e-05

2.5e-059.8e-052.3e-05

2.5e-059.8e-052.3e-05

0.0092390.0052260.0053570.006620.0100590.0088010.003792

0.0008430.000410.0004450.0016050.0009680.000790.000235

2.6e-058.3e-052.2e-05

2.6e-058.3e-052.2e-05

2.6e-053.3e-052.2e-05

5e-05

0.000810.000410.0004450.0016050.0008620.000760.000235

6e-062.3e-056e-06

6e-062.3e-056e-06

0.0008040.000410.0004450.0016050.0008390.0007540.000235

2.5e-053.1e-052.2e-05

4.6e-05

1e-061e-06

2.3e-053.2e-052.2e-05

1e-055e-06

0.0006710.000410.0004450.0016050.0006050.0006430.000235

5e-063.1e-055e-06

1.4e-051.7e-051e-05

1.6e-051.3e-05

9e-062.8e-057e-06

3e-052.9e-052.6e-05

2e-05

7e-062.3e-058e-06

7e-062.3e-058e-06

7e-062.3e-058e-06

0.0001560.0003740.000142

3.1e-050.000142.7e-05

7.7e-05

3.4e-05

4.3e-05

3.1e-056.3e-052.7e-05

1.2e-053.4e-058e-06

1.9e-052.9e-051.9e-05

0.0001250.0002340.000115

0.0001250.0001930.000115

6e-06

7e-067e-063e-06

2.8e-056e-062.6e-05

1.5e-053.3e-051.7e-05

7e-062.3e-058e-06

1.4e-053.8e-051.1e-05

1.4e-052.6e-051.4e-05

2.1e-051e-051.9e-05

1.9e-052.1e-051.7e-05

2.3e-05

4.1e-05

4.1e-05

2.3e-050.0001761.9e-05

6e-064e-056e-06

6e-064e-056e-06

6e-064e-056e-06

1.7e-050.0001361.3e-05

1.2e-057.5e-059e-06

1.2e-057.5e-059e-06

3e-063.1e-053e-06

3e-063.1e-053e-06

2e-063e-051e-06

2e-063e-051e-06

0.0079610.0048160.0049120.0050150.0077220.0076320.003527

0.0050680.0031120.0030090.0028080.0049190.0048560.002075

2.1e-054.4e-051.7e-05

2.1e-054.4e-051.7e-05

2.5e-054.9e-051.9e-05

2.5e-054.9e-051.9e-05

6e-063.8e-054e-06

6e-063.8e-054e-06

0.0050160.0031120.0030090.0028080.0047880.0048160.002075

0.0050160.0031120.0030090.0028080.0047880.0048160.002075

0.0028930.0017040.0019030.0022070.0028030.0027760.001452

7e-063.7e-055e-06

7e-063.7e-055e-06

7e-062e-058e-06

7e-062e-058e-06

0.0028780.0017040.0019030.0022070.0027120.0027630.001452

0.0028780.0017040.0019030.0022070.0027120.0027630.001452

1e-063.4e-050

1e-063.4e-050

2.1e-050.0001012e-05

2.1e-050.0001012e-05

4.3e-05

4.3e-05

2.1e-055.8e-052e-05

2.1e-055.8e-052e-05

0.0001490.000470.0001313e-05

2.2e-054.1e-051.6e-05

2.2e-054.1e-051.6e-05

2.2e-054.1e-051.6e-05

4e-05

4e-05

4e-05

1.7e-058.1e-051.3e-05

1.7e-053.7e-051.3e-05

1.7e-053.7e-051.3e-05

4.4e-05

4.4e-05

4.7e-059e-054.5e-053e-05

4.7e-059e-054.5e-053e-05

2.9e-055.1e-052.7e-05

7e-061.4e-057e-06

1.1e-052.5e-051.1e-053e-05

5e-059.7e-054.6e-05

3.3e-057.1e-053e-05

8e-061.9e-058e-06

1.1e-051.9e-051.1e-05

1.4e-05

1.4e-051.9e-051.1e-05

1.7e-052.6e-051.6e-05

1.7e-052.6e-051.6e-05

1.3e-057.3e-051.1e-05

2.8e-05

2.8e-05

1.3e-054.5e-051.1e-05

1.3e-054.5e-051.1e-05

4.8e-05

4.8e-05

4.8e-05

3.5e-053e-052.1e-05

3.5e-053e-052.1e-05

3.5e-053e-052.1e-05

3.5e-053e-052.1e-05

2.1e-050.0001842e-05

1e-056.3e-058e-06

1e-056.3e-058e-06

6e-062.2e-056e-06

4e-064.1e-052e-06

1.1e-050.0001211.2e-05

1.1e-050.0001211.2e-05

1.1e-050.0001211.2e-05

3e-053.4e-052.6e-05

3e-053.4e-052.6e-05

3e-053.4e-052.6e-05

3e-053.4e-052.6e-05

0.0061220.0049730.0052750.0082250.0054580.0059510.003589

0.0061220.0049730.0052750.0082250.0053950.0059510.003589

5.5e-050.0001035.4e-05

5.1e-058.9e-055e-05

5.1e-058.9e-055e-05

1e-052e-061e-05

9e-06

6e-06

9e-068e-069e-06

1.3e-05

2.3e-055e-062.5e-05

1e-061.3e-051e-06

8e-068e-065e-06

1.2e-05

3e-06

1e-05

4e-061.4e-054e-06

4e-061.4e-054e-06

4e-061.4e-054e-06

0.0032450.0034120.0036490.0061190.0025710.0032040.002621

0.0032450.0034120.0036490.0061190.0025710.0032040.002621

0.0005640.00180.0018290.0040130.0005760.0004920.001471

1.3e-050.0013390.0015051e-051.3e-051.8e-05

9e-060

2e-06

2e-062e-062e-06

9e-063e-069e-06

1.3e-051.2e-052e-06

6e-06

1.6e-05

2.2e-05

2e-062e-06

07e-061e-06

2e-06

4e-06

3e-061e-063e-06

3.1e-054e-061.5e-05

3.5e-056e-063.5e-05

0.0003490.00180.000490.0025080.0003190.0003070.001453

2e-05

1e-062e-061e-06

4e-061e-06

2.2e-051.1e-051.7e-05

2.2e-051.4e-051.6e-05

07e-06

8e-062.5e-058e-06

5e-061.1e-058e-06

2.3e-054e-062.2e-05

01e-067e-06

01.3e-051e-06

8e-061.1e-058e-06

4e-06

2e-063e-062e-06

3e-063e-063e-06

1e-061e-061e-06

02e-061e-06

1.8e-05

8e-061e-067e-06

0.0026810.0016120.001820.0021060.0019950.0027120.00115

0.0026030.0016120.001630.0021060.0018980.0024980.00115

7.8e-050.000199.7e-050.000214

0.0028220.0015610.0016260.0021060.0027210.0026930.000968

0.0028220.0015610.0016260.0021060.0027210.0026930.000968

4.4e-054.6e-053.6e-05

3e-061.4e-051e-06

3.8e-052.4e-053.2e-05

1e-067e-061e-06

0

2e-061e-062e-06

0.0027780.0015610.0016260.0021060.0026750.0026570.000968

3e-05

8e-064.3e-058e-06

0.002740.0015610.0016260.0021060.0025570.0026330.000968

3e-054.5e-051.6e-05

6.3e-05

6.3e-05

6.3e-05

6.3e-05

6.3e-05

0.0063650.0012740.0481580.003210.0557120.0066420.000502

3.3e-050.0001433.3e-05

3.3e-050.0001433.3e-05

3.3e-050.0001433.3e-05

3.3e-050.0001433.3e-05

1.7e-057.6e-051.8e-05

1.6e-056.7e-051.5e-05

0.0063320.0012740.0481580.003210.0555690.0066090.000502

0.000560.0007140.0013440.0005997.8e-05

9.4e-050.0001290.0002499.3e-05

9.4e-050.0001290.0002499.3e-05

5.8e-050.0001290.0001455.7e-05

3.6e-050.0001043.6e-05

0.0001930.0001380.0004470.0002137.8e-05

0.0001930.0001380.0004470.0002137.8e-05

8.5e-050.0001380.0001558.7e-05

2.9e-058.8e-053.5e-05

4e-061.3e-056e-06

1.1e-057e-061.4e-05

1.7e-050.000122.1e-05

4.7e-056.4e-055e-05

7.8e-05

0.0001230.0001730.000250.000128

6.4e-050.0001730.0001946.4e-05

6.4e-050.0001730.0001946.4e-05

2.5e-055.6e-052.7e-05

2.5e-055.6e-052.7e-05

3.4e-053.7e-05

3.4e-053.7e-05

2.1e-050.0001310.0001471.9e-05

2.1e-050.0001310.0001471.9e-05

2.1e-050.0001310.0001471.9e-05

0.0001290.0001430.0002510.000146

0.0001290.0001430.0002510.000146

0.0001290.0001430.0002510.000146

0.0002280.0001170.0003180.000235

0.0001220.0001170.000150.000124

0.0001220.0001170.000150.000124

0.0001220.0001170.000150.000124

0.0001060.0001680.000111

0.0001060.0001680.000111

0.0001060.0001680.000111

0.0001560.0001830.0004780.000164

0.0001560.0001830.0004780.000164

0.0001560.0001830.0004780.000164

0.0001560.0001830.0004780.000164

0.0011180.0006880.0006630.003210.0018410.0010940.000394

0.0003180.0007380.000317

0.0001980.0005180.000216

7e-061.3e-051e-05

1.5e-051.7e-051.5e-05

1.2e-05

7e-06

2e-06

2e-05

1.3e-05

2.6e-059e-062.8e-05

2.3e-051e-062.3e-05

1.2e-051.6e-051.2e-05

4e-06

2.3e-058.2e-052.6e-05

3e-06

5e-061e-056e-06

1.9e-05

1.3e-056.7e-051.1e-05

6e-063.3e-058e-06

2e-051e-062.3e-05

2e-06

2.6e-050.00013.1e-05

2.2e-058.7e-052.3e-05

5.4e-058.5e-054.8e-05

3.9e-058.5e-053.4e-05

1.5e-051.4e-05

3.3e-055.5e-051.9e-05

3.3e-055.5e-051.9e-05

3.3e-058e-053.4e-05

3.3e-058e-053.4e-05

0.0005650.0002580.0003360.0016050.0005970.0005540.000163

0.0005650.0002580.0003360.0016050.0005970.0005540.000163

0.0005650.0002580.0003360.0016050.0005970.0005540.000163

7e-061.5e-057e-06

7e-061.5e-057e-06

2e-06

7e-069e-067e-06

4e-06

2e-050.0001310.0003061.9e-05

2e-050.0001310.0003061.9e-05

2e-055.1e-051.9e-05

0.0001310.000147

0.000108

5.6e-055.4e-05

5.6e-055.4e-05

5.6e-055.4e-05

0.0001320.000430.0001960.0016050.0001120.0001270.000231

0.0001320.000430.0001960.0016050.0001120.0001270.000231

0.0001320.000430.0001960.0016058.4e-050.0001270.000231

2.8e-05

2e-057.3e-051.6e-05

2e-057.3e-051.6e-05

2e-057.3e-051.6e-05

0.0040020.0005860.046320.0506830.0042413e-05

0.0009423.6e-050.000850.0007390.001008

0.0006963.6e-050.0006630.0005810.000749

0.0002280.0001880.0001760.000241

0.0001790.000201

0.0004683.6e-050.0002960.0002040.000508

0.0002460.0001870.0001580.000259

0.0002460.0001870.0001580.000259

0.0433380.048684

0.0433380.048684

0.0433380.048684

0.003060.000550.0021320.001260.0032333e-05

0.0007290.0001710.0004870.0003450.0006711.5e-05

0.0001160.00010201.5e-05

0.0003616.9e-050.0002530.0001910.000396

9.2e-05

0.0002520.0002346.2e-050.000275

0.0017140.0003360.0010650.0008490.0018721.5e-05

0.0004317e-050.0002680.0001840.0004531.5e-05

0.0003616.9e-050.0002180.0001420.00041

0.0001330.00015

0.0004860.0001030.0001940.0002120.000531

0.0004369.4e-050.0002520.0001610.000478

0.0002280.0002136.6e-050.000252

0.0002280.0002136.6e-050.000252

0.0003894.3e-050.0003670.000438

0.0003894.3e-050.0003670.000438

0.0002680.0001610.0008690.000276

2.4e-055.2e-052.8e-05

2.4e-055.2e-052.8e-05

2.4e-055.2e-052.8e-05

4.5e-052.9e-054.9e-05

4.5e-052.9e-054.9e-05

4.5e-051.6e-054.9e-05

1.3e-05

0.0001330.0001610.0006660.000137

9e-05

4.5e-05

4.5e-05

0.0001330.0001610.0005760.000137

0.0001330.0001610.0005760.000137

6.6e-050.0001226.2e-05

3.5e-054.9e-052.7e-05

3.5e-054.9e-052.7e-05

3.1e-057.3e-053.5e-05

3.1e-057.3e-053.5e-05

3.6e-05

3.6e-05

3.6e-05

3.6e-05

0.0103390.0210990.0179690.0334010.0071210.0100870.015241

0.0103390.0210990.0179690.0334010.0071210.0100870.015241

1.7e-059.2e-052.1e-05

1.7e-059.2e-052.1e-05

1.3e-052.8e-051.2e-05

1.3e-052.8e-051.2e-05

4e-063.5e-059e-06

4e-063.5e-059e-06

2.9e-05

2.9e-05

4e-066.4e-053e-06

4e-066.4e-053e-06

2e-063.5e-052e-06

1.7e-05

2e-061.8e-052e-06

2e-062.9e-051e-06

2e-062.9e-051e-06

0.0103180.0210990.0179690.0334010.0069650.0100630.015241

1.8e-058.3e-051.5e-05

7e-064.1e-055e-06

8e-06

5e-062.4e-054e-06

2e-069e-061e-06

1.1e-054.2e-051e-05

4e-062.1e-053e-06

7e-062.1e-057e-06

0.01030.0210990.0179690.0334010.0068820.0100480.015241

0.0080450.0168070.0133120.029890.005990.007860.011896

0.0009820.0012430.0005780.0052162.8e-050.0009760.000214

8e-06

0.0040290.0125960.0053280.0064194.9e-050.0039060.01042

0.00520.005842

0.0016930.001490.0008430.0049151e-050.001650.000312

0.000910.0013240.0010470.0043131e-050.0008970.00095

1.8e-05

1.2e-05

0.0004310.0001540.0003160.0090276e-060.000431

7e-06

0.0022550.0042920.0046570.0035110.0008920.0021880.003345

8e-068e-068e-06

8e-060.0029774e-058e-060.003345

0.0022360.0042920.001680.0035110.0008170.002169

3e-062.7e-053e-06

5.5e-050.000333.1e-05

5.5e-050.000333.1e-05

2.5e-050.0002711.7e-05

6.7e-05

6.7e-05

6.7e-05

1.4e-057.2e-051e-05

7e-064.2e-057e-06

4e-061.8e-053e-06

3e-062.4e-054e-06

7e-063e-053e-06

7e-063e-053e-06

1.1e-050.0001327e-06

9e-066e-066e-06

8e-066e-066e-06

1e-06

01e-050

01e-050

06.4e-05

2.5e-05

03.9e-05

2e-065.2e-051e-06

2e-065.2e-051e-06

3e-055.9e-051.4e-05

3e-055.9e-051.4e-05

2.9e-053.8e-051.4e-05

2.9e-053.8e-051.4e-05

1e-062.1e-050

1e-062.1e-050

0.1504860.2146310.1913920.219660.1104690.1329040.241852

0.0028760.0014720.0203450.0036110.0105380.0027510.014551

0.0025140.0014720.0018170.0036110.0023940.0024140.001581

0.0025140.0014720.0018170.0036110.0023940.0024140.001581

0.0025140.0014720.0018170.0036110.0023940.0024140.001581

0.0002260.0001250.0001860.0015050.0002910.000220.000131

0.0022880.0013470.0016310.0021060.0021030.0021940.00145

0.0003020.0180990.0077720.0002810.012675

0.0005920.000757

0.0005920.000757

0.0005920.000665

9.2e-05

0.0003020.0175070.0070150.0002810.012675

0.0003020.0175070.0070150.0002810.012675

0.0002320.0002010.0002490.000203

0.0005570.000626

0.0005330.000599

7e-050.0001270.0001427.8e-05

0.0048060.005399

0.0112830.012675

6e-050.0004290.0003725.6e-050.000295

6e-050.0004290.0003725.6e-050.000295

0.000103

0.000103

2.4e-058.2e-052.4e-05

2.4e-058.2e-052.4e-05

3.6e-050.0004290.0001873.2e-050.000295

3.6e-050.0001660.0001873.2e-05

0.0002630.000295

0.1243460.1823130.1219150.1802420.0724970.1112960.170305

0.022970.0241170.0219870.0355060.022230.0146190.035002

0.0004380.0001030.0002760.0020060.0019030.0004551.6e-05

6.9e-05

6.9e-05

0.0004380.0001030.0002760.0020060.0018340.0004551.6e-05

1.9e-052.3e-052.1e-05

2.7e-051.7e-052.7e-05

2.4e-052e-052.5e-05

8e-06

0.0003680.0001030.0002760.0020060.0017660.0003821.6e-05

0.0004425e-060.0031390.0021060.0006020.0004640.001262

0.000105

0.000105

4.5e-058.8e-053.4e-05

4.5e-058.8e-053.4e-05

0.0003975e-060.0031390.0021060.0004090.000430.001262

4e-06

1.7e-051.2e-051.9e-05

6e-06

3.4e-051.4e-053.6e-05

1e-06

3.6e-05

3.1e-053e-063.3e-05

2e-065e-060.0018750.0021065e-061.2e-05

1.2e-05

0.0001540.000140.0001580.000161

0.0011240.001262

9e-06

1.2e-059e-061.5e-05

8e-06

4.7e-057e-064.7e-05

4.1e-059e-064.2e-05

5.9e-05

3.5e-051.9e-053.8e-05

2.4e-052.5e-052.7e-05

1.3e-05

4.6e-051e-063.8e-056e-05

4.6e-051e-063.8e-056e-05

3e-063e-066e-06

1.6e-059e-062.2e-05

1e-061e-061e-061e-06

4e-066e-065e-06

2.2e-051.9e-052.6e-05

4.4e-050.0008780.0014154.8e-053.3e-05

0.0001380.000155

0.0001380.000155

0.0001460.000164

0.0001460.000164

0.000352

5.5e-05

5.1e-05

2.8e-05

2.8e-05

6.1e-05

2.7e-05

2.2e-05

3e-05

5e-05

0.0003160.0003553.3e-05

3.3e-05

0.0003160.000355

4.4e-057.6e-054.8e-05

4.4e-057.6e-054.8e-05

0.0002780.000313

0.0002780.000313

3.2e-056.1e-052.5e-05

3.2e-056.1e-052.5e-05

3.2e-056.1e-052.5e-05

0.0159820.0203210.0120140.0263790.0086990.0068980.031891

0.0007150.0002350.0005250.0017050.0011450.0007348.2e-05

0.0007150.0002350.0005250.0017050.0011450.0007348.2e-05

0.000164

3.5e-05

0.00012

9e-06

0.000116

0.000116

0.0001160.000131

0.0001160.000131

1.3e-050.000121.6e-05

1.3e-050.000121.6e-05

0.0010420.0006450.0007360.0017050.000710.0010191.5e-05

2.1e-05

6e-065e-068e-06

2e-06

3.8e-052.9e-053.8e-05

1e-05

3e-063e-063e-063e-06

0.0009480.0006450.0007360.0017050.0005660.000916

03e-06

2e-06

7e-061e-065e-06

3.8e-055.5e-054.5e-05

1e-066e-063e-063e-06

3e-06

1e-064e-061e-063e-06

6e-06

0.0006150.0004160.0005550.0016050.0005980.000591

0.0006150.0004160.0005550.0016050.0005980.000591

0.000101

0.000101

0.0001040.0002580.00011

8.9e-05

0

0.000112

0.0001045.7e-050.00011

0.0134840.0190250.0100820.0213640.0050610.004420.031794

2.5e-059.2e-052.8e-05

7.2e-054.7e-058.1e-05

4.5e-050.0015180.0017051e-054.6e-05

4.7e-050.0001184.9e-05

5.5e-05

0.0001840.000207

0.0097880.0045310.0015660.0035110.0004860.005671

1.7e-05

0.0002140.000241

3.7e-051.7e-055.2e-05

0.0006620.0001890.0004110.0028088.3e-050.000661

2.5e-050.0001233.8e-05

3.9e-055.4e-055.4e-05

0.0002590.000291

0.0004330.0058260.0007240.0048141.8e-050.0004210.000922

0.0001615.2e-050.0001750.0003120.000171

2e-06

5.5e-050.0011990.0014410.0022078e-065.7e-050.001769

3e-05

4.9e-050.0002160.0002435.9e-05

3.8e-053e-054.7e-05

0.0001950.0017740.0004320.0020062.1e-050.000260.003707

0.0002290.000258

0.0012350.0053980.0004710.0024070.0001090.0017260.006186

0.0001830.000171.8e-050.000201

2e-06

0.0005440.000611

1e-053e-061.7e-05

0.000135.6e-050.0002240.0019061.4e-050.000130.013506

0

3.3e-05

0.0002960.000332

0.000250.000281

1.1e-05

7e-06

9.4e-055e-069.5e-05

0.000140.0001471e-060.000202

5e-06

2.1e-054.5e-052.5e-05

7.4e-05

8.4e-05

0.0002840.000319

1e-05

0

0.0003270.000367

0.000113

0.000113

9e-067e-068e-06

9e-067e-068e-06

0.000175

9.8e-05

7.7e-05

0.0009460.0008970.0010050.0019060.0021330.0018170.000634

0.0009420.0008970.0010050.0019060.002070.0018130.000634

0.0009420.0008970.0010050.0019060.002070.0018130.000634

4e-066.3e-054e-06

4e-066.3e-054e-06

0.005040.002790.0046750.0031090.0073790.0048520.001166

2.3e-050.0013080.0023732e-050.001081

2.3e-057.9e-052e-05

0.0013080.0022940.001081

1.6e-054.9e-051.5e-05

1.6e-054.9e-051.5e-05

0.000117

0.000117

0.0050010.002790.0033670.0031090.004840.0048178.5e-05

1e-05

7e-06

5e-06

2.2e-05

9e-05

0.0001444.2e-050.0001419.1e-050.0001764.4e-05

8.4e-05

5.8e-05

1.6e-057.2e-051.7e-05

3.5e-05

6.3e-05

2.1e-05

1.1e-05

4e-06

4.5e-05

2.9e-055.6e-052.6e-05

5.9e-055.4e-051.6e-052.9e-054.1e-05

6.9e-05

0.0047470.0026940.0032260.0031090.0038910.004564

6e-062.6e-055e-06

1.6e-05

2.3e-05

1.4e-05

5.1e-05

2.3e-05

1.3e-05

9e-06

1.5e-05

0.1013760.1581960.0999280.1447360.0502670.0966770.135303

0.0038720.0023290.0040320.0072220.0015710.0037220.00222

0.0037290.0021940.0039040.0057170.0014330.0035840.002104

9.9e-050.00020.0003170.0016053.6e-059.5e-059.8e-05

1e-05

2.5e-051.4e-052.5e-05

0.0002740.000307

0.0035560.0019940.0017950.0024070.0010140.0034190.002006

3.5e-054e-053.5e-05

1.4e-050.0015180.0017051.2e-051e-05

0.0001280.0001350.0001280.0015050.0001110.0001240.000116

0.0001280.0001350.0001280.0015050.0001110.0001240.000116

1.5e-052.7e-051.4e-05

1.5e-052.7e-051.4e-05

0.0415760.1007060.0533710.0804420.0222920.045540.091394

9.5e-050.0044640.0050150.0001048.8e-05

7e-050.0044640.0050153.9e-056.3e-05

2.5e-05

2.5e-054e-052.5e-05

0.0414810.1007060.0489070.0754270.0221880.0454520.091394

5e-053.9e-050.0018750.0021061.9e-055.1e-05

0.0015190.0005660.0005350.0051150.0001560.0015210.003475

9.3e-05

2.6e-05

0.0012990.001459

5.2e-05

1.6e-05

9.8e-05

0.0001810.000203

9.7e-05

0.0041930.009820.0024530.0060180.000770.0040350.008028

0.000320.0077030.000490.0035110.0002580.0003090.003921

0.0024490.0050890.0031520.0036110.0019980.0078840.005391

2.3e-05

5.9e-056.2e-051.6e-05

6.4e-050.0001680.000170.0016051.1e-056e-050.000109

9e-05

6.6e-05

0.0258620.0653310.0255650.0361080.014250.024820.05956

6.9e-050.0001197.5e-05

0.0001320.000148

3.9e-053.3e-054.1e-05

4e-063.8e-054e-062.1e-05

2.3e-059.3e-051.8e-05

1.6e-05

3e-050.0033930.0038115.7e-052.7e-05

2.5e-053.4e-052.6e-05

7.7e-056.3e-050.0013390.0015054.5e-057.7e-059.6e-05

2.6e-05

0.0007730.0009630.0007090.0019060.0003980.0007450.001221

4.6e-056.4e-054.8e-05

9e-056.3e-050.0031250.0035115.1e-058.4e-05

0.0057020.0109010.0034410.006620.0012020.0054770.008362

0.0010480.001178

8.7e-057.3e-058.8e-05

5.5e-05

6.7e-05

5.4e-050.0002563.8e-05

5.4e-050.0002563.8e-05

7.9e-05

7e-06

5.7e-05

5.4e-05

2.1e-05

5.4e-053.8e-053.8e-05

0.0013150.0009970.0010810.0012521.6e-05

0.0013150.0009970.0010810.0012521.6e-05

0.0012570.0009970.0009490.001207

1.6e-05

5.2e-05

3e-053.4e-051.7e-05

2.8e-054.6e-052.8e-05

0.0532170.0544230.0378680.0509530.0235220.0448380.040939

0.0532050.0544230.0359040.0487460.0234410.044810.040939

7e-066e-061e-067e-06

5e-062.1e-052e-065e-061.8e-05

4.5e-051e-054.4e-05

2.6e-051e-052.7e-051.8e-05

0.0006930.0001740.0002870.003310.0003470.0006660.000148

0.0106930.0056070.0046960.0036110.0066280.0103610.00293

0.00020.000224

6.2e-05

7.7e-053.3e-057.6e-053.8e-05

2.3e-05

0.0263830.0308760.013880.0139420.0067660.0171180.027348

9.5e-05

6.5e-052.1e-056.2e-05

7.1e-05

0.000110.0004560.0002730.0018056.3e-059.3e-050.000809

0.0004080.0015390.00060.0021069.6e-050.000310.001631

3.1e-054e-063.4e-05

8.7e-057.3e-058.9e-05

4e-05

0.0025030.0012360.001070.0018050.0005980.00240.000877

0.000440.0006140.0003890.0017050.0002170.000426

3.7e-052.3e-050.0002040.0023075.4e-050.00012

2.4e-050.0002352.6e-056.9e-050.000264

1.8e-05

8.2e-057.9e-057.8e-05

7.5e-05

8.1e-050.000333.5e-058.2e-050.000371

8e-060.0016960.0019062.6e-052.2e-05

0.0005580.000627

0.0004530.000509

0.0013350.002660.0018590.0024070.001760.0029990.002323

0.0003050.0003890.0069215.2e-050.0002915.1e-05

0.0001020.0001320.000108

2.2e-05

3.2e-05

0.0011060.001243

8.4e-05

0.0005110.0010870.0005440.0019060.0003110.0004910.000861

0.0020.001970.002477

8.8e-05

1.8e-054e-061.8e-05

0.00030.0002290.0001710.0002950.000341

0.0087080.0081240.0049360.0050150.0028960.008396

8.2e-05

0.0001214.3e-050.0001235.3e-05

3.4e-05

3.5e-05

1.2e-050.0019640.0022078.1e-052.8e-05

4.2e-05

7e-061.9e-057e-06

5e-060.0019640.0022072e-052.1e-05

0.0013420.0007380.003660.0061190.0015450.0012870.000734

0.0005980.0003660.0004270.0016050.0006230.0005710.000251

4.2e-05

0.0005980.0003660.0004270.0016050.0005680.0005710.000251

1.3e-05

0.000720.0003720.0032330.0045140.0008660.0006960.000483

7e-060.001250.0014047e-067e-06

0.000640.0003720.0004630.0016050.0005180.000620.00032

9e-060.0013390.0015051.5e-051.1e-05

1.4e-051.9e-051.2e-05

3.4e-052.1e-053.4e-05

1.6e-051.5e-051.2e-05

0.0001810.0002710.000163

2.4e-055.6e-052e-05

2.4e-055.6e-052e-05

0.0223460.0305190.0360110.0310930.0240150.0179730.044827

7e-063.6e-056e-06

7e-063.6e-056e-06

7e-063.6e-056e-06

7e-063.6e-056e-06

2.2e-050.0002263.6e-05

1.1e-050.0001382.2e-05

4e-065.7e-053e-06

4e-065.7e-053e-06

7e-068.1e-051.9e-05

4e-063.8e-051e-05

3e-064.3e-059e-06

1.1e-058.8e-051.4e-05

3e-063.9e-052e-06

3e-063.9e-052e-06

8e-064.9e-051.2e-05

8e-064.9e-051.2e-05

0.0016120.0011070.0009470.003410.0015380.0015410.000597

0.0009840.0006380.0005480.0018050.0009970.0009390.000248

3e-062.7e-052e-06

3e-062.7e-052e-06

7e-061.4e-055e-06

7e-061.4e-055e-06

7e-064.8e-057e-06

7e-064.8e-057e-06

1.1e-054.1e-056e-06

1.1e-054.1e-056e-06

0.000910.0006380.0005480.0018050.0007450.0008680.000248

0.000910.0006380.0005480.0018050.0007450.0008680.000248

3.6e-056.7e-054.2e-05

1.7e-052.3e-051.8e-05

8e-067e-061.2e-05

00

2.9e-05

1.1e-058e-061.2e-05

1e-055.5e-059e-06

1e-055.5e-059e-06

0.0006090.0004690.0003990.0016050.0004040.0005940.000349

8e-065.4e-055e-06

8e-065.4e-055e-06

1e-054.1e-051.7e-05

5e-062.6e-051.2e-05

5e-061.5e-055e-06

0.0005910.0004690.0003990.0016050.0003090.0005720.000349

2e-066e-063e-06

1e-064e-061e-06

6e-061.1e-056e-06

2e-064e-062e-06

1e-068e-061e-06

0.000570.0004690.0003990.0016050.0002390.0005520.000349

2e-061e-052e-06

7e-062.7e-055e-06

1.4e-058.5e-054e-06

1e-055.1e-052e-06

1e-055.1e-052e-06

4e-063.4e-052e-06

4e-063.4e-052e-06

5e-065.2e-054e-06

5e-065.2e-054e-06

5e-065.2e-054e-06

0.0207050.0294120.0350640.0276830.0222150.016390.04423

3.7e-059e-052.5e-05

3.7e-059e-052.5e-05

3.7e-059e-052.5e-05

3.8e-055.4e-052.8e-05

3.8e-055.4e-052.8e-05

3.8e-055.4e-052.8e-05

0.0014410.0008980.0056340.0051150.0056670.0014220.001192

1.5e-053.1e-051.5e-05

1e-06

6e-062e-066e-06

9e-062.8e-059e-06

4.5e-05

4.5e-05

0.0014150.0008980.0056340.0051150.0054850.0013930.001192

7e-06

2.3e-05

2.4e-05

3.3e-051.9e-053.4e-05

1.2e-052.7e-051.4e-05

2.1e-051.6e-051.4e-05

5.8e-05

6.3e-05

0.0005250.0007510.000485

9e-063.3e-059e-06

1.9e-05

0.0001940.0003350.0002270.0016050.0001860.0001870.000248

1.7e-052.4e-051.9e-05

0.0009720.0005630.0007130.0017050.001160.000940.000459

4.9e-05

3.3e-053.3e-053.4e-05

2.2e-052.4e-052.6e-05

1.6e-057e-062.3e-05

0.0025620.002878

1e-051e-061e-05

1.4e-054.4e-051.5e-05

8e-068e-061.1e-05

2.4e-052.5e-052.6e-05

3e-050.0016070.0018056e-063.1e-05

1.1e-050.0001061.4e-05

5e-065.3e-057e-06

6e-065.3e-057e-06

3.9e-050.0001290.0001453.8e-05

3.9e-050.0001290.0001453.8e-05

3.9e-050.0001290.0001453.8e-05

2.1e-050.0011850.0014021.9e-053.3e-05

0.0003070.000345

0.0003070.000345

0.0008780.000986

0.0008780.000986

3.3e-05

3.3e-05

2.1e-057.1e-051.9e-05

2.1e-057.1e-051.9e-05

0.0001870.0001510.0006020.0001920.000114

0.0001750.0001510.0005420.0001760.000114

0.000114

4.8e-058e-054.6e-05

0.0001270.0001510.0004620.00013

1.2e-056e-051.6e-05

1.2e-056e-051.6e-05

0.0001280.0005840.000108

1.4e-056.6e-051.2e-05

1.4e-056.6e-051.2e-05

1e-055.1e-059e-06

1e-055.1e-059e-06

1.3e-050.0001271.5e-05

7e-062.3e-057e-06

4e-063.6e-055e-06

2e-061.6e-053e-06

5.2e-05

1.6e-059.2e-051.6e-05

6e-063.2e-055e-06

9e-063.2e-059e-06

1e-062.8e-052e-06

9e-06

9e-06

1.1e-054.6e-051e-05

1.1e-054.6e-051e-05

5.5e-050.0001854.6e-05

8e-065.1e-056e-06

1.5e-054.7e-052e-05

5e-062.8e-055e-06

1.3e-052.8e-051.3e-05

9e-06

5e-063.1e-052e-06

01.7e-050

2e-06

1.5e-05

000

1.2e-050.0001049e-06

1e-054.9e-058e-06

1e-054.9e-058e-06

2e-065.5e-051e-06

2e-065.5e-051e-06

4.3e-055e-053.2e-05

4.3e-055e-053.2e-05

4.3e-055e-053.2e-05

0.0112850.0146440.0106350.0093280.0059710.0108350.020021

0.001310.0020380.00908

0.001310.0020380.00908

6.3e-050.0001216.2e-05

6.3e-050.0001216.2e-05

6.5e-050.0001520.0002950.0002856.2e-05

1.4e-050.0001061.4e-05

5.1e-054.8e-05

0.0001520.000135

0.000160.000179

3.3e-05

3.3e-05

5.7e-050.0001165.5e-05

5.7e-050.0001165.5e-05

0.01110.0131820.0083020.0093280.0054490.0106560.010908

0.000190.000214

4e-062.5e-056e-06

3e-065.7e-054e-06

0.0110730.0131820.0081120.0093280.0050180.0106260.010908

2e-052.3e-052e-05

0.000112

0.0001420.000159

0.0001420.000159

0.0001420.000159

0.0062540.0127850.0079920.006520.0039690.0026970.015548

6.3e-050.0001270.0001426.1e-05

6.3e-050.0001270.0001426.1e-05

0.0061690.0127850.0047130.006520.0036460.0026170.012008

0.0061690.0127850.0047130.006520.0036460.0026170.012008

0.0031520.00354

0.0031520.00354

2.2e-050.00011.9e-05

2.2e-050.00011.9e-05

8.1e-05

8.1e-05

0.001220.0010850.0091960.006720.0034180.0009850.007322

0.0002367.7e-050.0011270.0010010.0001420.000517

0.0002117.7e-050.0001710.0004430.000116

0.000460.000517

0.0002910.000327

2.5e-050.0002050.0002312.6e-05

0.0001460.0002620.000294

7.4e-05

0.0002620.000294

7.2e-05

0.0001942e-050.0058350.0004586.1e-050.006295

0.0001942e-050.0002420.0004586.1e-05

0.0008330.000936

4e-06

4e-06

0.0039990.004492

4e-06

0.0007610.000855

3.2e-05

3.2e-05

1.6e-050.0001870.000212.2e-054.9e-05

4.9e-05

1.6e-050.0001870.000212.2e-05

0.0004560.0009570.000490.000135

0.0004360.00049

0.0001770.0001575e-06

0.0002790.000248

0.0001160.00013

1.9e-058.4e-051.9e-05

1.9e-058.4e-051.9e-05

0.0007550.0003860.0006890.006720.0010190.000741

0.0007550.0002460.0005650.006720.0010190.000741

0.000140.000124

0.0001390.000156

0.0001390.000156

0.0007020.0003270.0051410.0047140.0015060.0006720.005099

0.0007020.0003270.0051410.0047140.0015060.0006720.005099

0.0007020.0003270.0051410.0047140.0015060.0006720.005099

0.0002970.0001240.0002360.0020060.0003020.0002793.2e-05

0.0002970.0001240.0002360.0020060.0003020.0002793.2e-05

0.000103

0.000103

9.2e-05

9.2e-05

0.0004050.0002030.0049050.0027080.0010090.0003930.005067

0.0004050.0002030.0003940.0027080.0010090.000393

0.0001490.000167

0.0043620.0049

6.7e-05

6.7e-05

6.7e-05

6.7e-05

6.7e-05

0.0002160.007980.0018460.0002120.00707

0.0002160.007980.0018460.0002120.00707

0.0002160.007980.0018460.0002120.00707

8.5e-05

8.5e-05

0.0002080.0001840.000205

0.0002080.0001840.000205

0.0076610.0015360.00707

0.0013670.001536

0.0062940.00707

8e-067.3e-057e-06

5.8e-05

8e-061.5e-057e-06

0.0001350.000152

0.0001350.000152

0.0032870.0019030.0019890.0021060.0031260.0031060.001649

0.0032870.0019030.0019890.0021060.0031260.0031060.001649

0.0001690.0003350.0001231.6e-05

0.0001410.0002859.8e-051.6e-05

0.0001410.0002859.8e-051.6e-05

1.9e-05

2.5e-05

2.9e-053e-052.1e-05

1.1e-05

1.1e-053.4e-058e-06

1.1e-052.4e-058e-06

9e-06

2.1e-053.7e-051.3e-05

3.1e-053.9e-052e-051.6e-05

1.9e-051.8e-051.4e-05

1.9e-053.9e-051.4e-05

2.8e-055e-052.5e-05

2.8e-055e-052.5e-05

2.8e-055e-052.5e-05

0.0031180.0019030.0019890.0021060.0027910.0029830.001633

0.0031180.0019030.0019890.0021060.0027910.0029830.001633

1.7e-056e-051.3e-05

1.1e-053e-057e-06

6e-063e-056e-06

0.0030660.0019030.0019890.0021060.0026490.0029410.001633

1.4e-054.2e-05

6e-062.2e-055e-06

3e-069e-063e-06

3e-062.2e-053e-06

2e-06

0.0030540.0019030.0019890.0021060.002580.002930.001591

1.2e-053.1e-051e-05

1.2e-053.1e-051e-05

2.3e-055.1e-051.9e-05

2.3e-055.1e-051.9e-05

9.1e-050.0003510.0007587.9e-05

2.2e-050.0001931.9e-05

8e-065e-057e-06

8e-065e-057e-06

8e-065e-057e-06

8e-065e-057e-06

1.4e-050.0001431.2e-05

1.4e-050.0001431.2e-05

1.4e-055.6e-051.2e-05

1.4e-055.6e-051.2e-05

5.1e-05

5.1e-05

3.6e-05

3.6e-05

3.3e-05

3.3e-05

3.3e-05

3.3e-05

3.3e-05

3.2e-053.9e-052.3e-05

3.2e-053.9e-052.3e-05

3.2e-053.9e-052.3e-05

3.2e-053.9e-052.3e-05

3.2e-053.9e-052.3e-05

9.8e-05

9.8e-05

9.8e-05

9.8e-05

9.8e-05

3.7e-050.0003510.0003953.7e-05

3.7e-050.0003510.0003953.7e-05

3.7e-050.0003510.0003953.7e-05

3.7e-050.0003510.0003953.7e-05

3.7e-050.0001670.0001883.7e-05

0.0001840.000207

1.6e-050.0001021.5e-05

1.6e-050.0001021.5e-05

1.6e-050.0001021.5e-05

1.6e-050.0001021.5e-05

1.6e-050.0001021.5e-05

1.6e-050.0001021.5e-05

0.0021260.0115760.0037090.0536610.0022670.0020040.074133

0.0021260.0115760.0037090.0536610.0022670.0020040.074133

0.0021060.0115760.0037090.0536610.0021420.0019870.074133

0.0021060.0115760.0037090.0536610.0021420.0019870.074133

0.0021060.0115760.0037090.0536610.0021420.0019870.074133

1.9e-051.1e-052.1e-05

6.5e-050.0003310.0004710.0016054.1e-055.5e-050.000492

2e-068e-062.2e-05

03e-060

4e-062.1e-052e-06

0.0020140.0112450.0032380.0520560.0019970.0018850.073641

1e-061.1e-051e-06

2.5e-05

1e-063e-061e-06

2.2e-05

2e-050.0001251.7e-05

7e-063.7e-056e-06

7e-063.7e-056e-06

7e-063.7e-056e-06

3e-064.1e-052e-06

3e-064.1e-052e-06

3e-064.1e-052e-06

1e-054.7e-059e-06

9e-062.3e-059e-06

2.3e-05

9e-069e-06

1e-062.4e-050

1e-062.4e-050

2.3e-050.0001451.4e-05

2.3e-050.0001451.4e-05

2.3e-050.0001451.4e-05

2.3e-050.0001451.4e-05

1.5e-057.5e-057e-06

3.4e-05

1.5e-054.1e-057e-06

8e-063.5e-057e-06

8e-063.5e-057e-06

3.5e-05

3.5e-05

0.0002850.0004320.001605

0.0002850.0004320.001605

0.0002850.0004320.001605

0.0002850.0004320.001605

0.0002850.0004320.001605

0.0002850.0004320.001605

3.1e-055.5e-052.4e-05

3.1e-055.5e-052.4e-05

3.1e-055.5e-052.4e-05

3.1e-055.5e-052.4e-05

3.1e-055.5e-052.4e-05

3.1e-055.5e-052.4e-05

0.0001110.0003639.1e-05

0.0001110.0003639.1e-05

0.0001110.0003639.1e-05

0.0001110.0003639.1e-05

5e-066.3e-056e-06

5e-066.3e-056e-06

3.5e-055.6e-052.6e-05

1.3e-05

1.4e-052.5e-051e-05

2.1e-051.8e-051.6e-05

4.6e-050.0001113.5e-05

4e-063.3e-058e-06

9e-062.8e-058e-06

1.9e-052.5e-051.1e-05

1.4e-052.5e-058e-06

8e-068.4e-058e-06

8e-063.4e-058e-06

5e-05

1.7e-054.9e-051.6e-05

3e-062.7e-059e-06

1.4e-052.2e-057e-06

2.6e-050.000211.2e-05

2.6e-050.000211.2e-05

2.6e-050.000211.2e-05

2.6e-050.000211.2e-05

5e-063.8e-051e-06

5e-063.8e-051e-06

6e-065.1e-054e-06

6e-065.1e-054e-06

5e-063.2e-051e-06

5e-063.2e-051e-06

5e-05

5e-05

1e-053.9e-056e-06

1e-053.9e-056e-06

3e-064.6e-052e-06

3e-064.6e-052e-06

3e-064.6e-052e-06

3e-064.6e-052e-06

3e-064.6e-052e-06

3e-064.6e-052e-06

3e-064.2e-053e-06

3e-063e-06

3e-063e-06

3e-063e-06

3e-063e-06

3e-063e-06

4.2e-05

4.2e-05

4.2e-05

4.2e-05

4.2e-05

6.1e-050.00037.1e-05

6.1e-050.00037.1e-05

6.1e-050.00037.1e-05

2.1e-056.6e-052e-05

1.3e-051.8e-051.4e-05

3e-06

1.3e-051.5e-051.4e-05

8e-064.8e-056e-06

8e-064.8e-056e-06

4e-050.0002345.1e-05

1.7e-059.6e-052e-05

3.9e-05

1.7e-052.5e-052e-05

3.2e-05

4.8e-05

4.8e-05

1.9e-054.9e-052.2e-05

1.9e-054.9e-052.2e-05

4e-064.1e-059e-06

4e-064.1e-059e-06

1.7e-050.0001961.1e-05

7.7e-05

7.7e-05

7.7e-05

7.7e-05

7.7e-05

1.1e-057.8e-051e-05

1.1e-057.8e-051e-05

1.1e-057.8e-051e-05

1.1e-057.8e-051e-05

1.1e-057.8e-051e-05

6e-064.1e-051e-06

6e-064.1e-051e-06

6e-064.1e-051e-06

6e-064.1e-051e-06

6e-064.1e-051e-06

0.0187240.0144670.0287260.0235720.0351070.0182850.012406

0.0040560.0024690.0038490.0025080.0055250.0038950.001816

0.0040560.0024690.0038490.0025080.0055250.0038950.001816

0.0040560.0024690.0038490.0025080.0055250.0038950.001816

0.0013320.001496

0.0006910.000776

0.0006410.00072

0.0040420.0024690.0025170.0025080.0038110.003880.001816

0.0040420.0024690.0025170.0025080.0038110.003880.001816

6.5e-05

6.5e-05

1.4e-056.5e-051.5e-05

1.4e-056.5e-051.5e-05

8.8e-05

8.8e-05

9e-069.2e-051.3e-05

9e-069.2e-051.3e-05

3e-065.4e-054e-06

3e-065.4e-054e-06

3e-065.4e-054e-06

6e-063.8e-059e-06

6e-063.8e-059e-06

6e-063.8e-059e-06

4.4e-050.0006044.8e-05

4.4e-050.0006044.8e-05

4.4e-050.0006044.8e-05

1e-050.0001731.1e-05

1e-058.1e-051.1e-05

9.2e-05

8e-060.000191e-05

8e-066.2e-051e-05

3.7e-05

4.5e-05

4.6e-05

6.4e-05

6.4e-05

1.4e-050.0001021.5e-05

1.4e-050.0001021.5e-05

1.2e-057.5e-051.2e-05

1.2e-057.5e-051.2e-05

2.7e-050.0001281.9e-05

2.7e-050.0001281.9e-05

2.7e-050.0001281.9e-05

7e-064.1e-056e-06

7e-064.1e-056e-06

2e-058.7e-051.3e-05

2e-058.7e-051.3e-05

0.0031660.0017850.0025690.0022070.0045810.003062

0.0031660.0017850.0025690.0022070.0045810.003062

1.7e-050.0001582.3e-05

5.1e-05

5.1e-05

4.9e-05

4.9e-05

1.7e-055.8e-052.3e-05

1.7e-055.8e-052.3e-05

1.3e-050.0001391e-05

5e-067.1e-055e-06

5e-067.1e-055e-06

8e-066.8e-055e-06

8e-066.8e-055e-06

0.0030680.0017850.0024080.0022070.0034430.002951

2e-055.5e-051.9e-05

2e-055.5e-051.9e-05

1.8e-058.9e-051.9e-05

1.8e-058.9e-051.9e-05

3.3e-053.2e-05

3.3e-053.2e-05

0.0002360.000265

0.0002360.000265

8e-060.0001068e-06

8e-060.0001068e-06

1.2e-057.7e-051.3e-05

1.2e-057.7e-051.3e-05

0.0029770.0017850.0021720.0022070.0028510.00286

0.0029770.0017850.0021720.0022070.0028510.00286

1.3e-053.7e-051.6e-05

1.3e-053.7e-051.6e-05

1.3e-053.7e-051.6e-05

5.1e-05

5.1e-05

5.1e-05

0.000339

7.9e-05

2.5e-05

2.6e-05

2.8e-05

7.8e-05

3.9e-05

3.9e-05

0.000182

3.8e-05

3.1e-05

3.3e-05

2.3e-05

1.3e-05

3.2e-05

1.2e-05

5.5e-050.0001610.0004146.2e-05

4.5e-05

4.5e-05

9e-064.5e-051.1e-05

9e-064.5e-051.1e-05

7e-065.5e-059e-06

7e-065.5e-059e-06

3.9e-050.0001610.0002694.2e-05

0.0001610.000181

3.9e-058.8e-054.2e-05

0.0047230.0025180.0095610.0116350.0092890.0046380.00378

0.0001250.000237

0.0001250.000141

0.0001250.000141

0.0001250.000141

9.6e-05

9.6e-05

9.6e-05

0.0047230.0025180.0094360.0116350.0090520.0046380.00378

0.0006350.0001440.0018530.0016050.0017550.0006610.00066

0.0006350.0001440.0018530.0016050.0017550.0006610.00066

4.9e-050.0001440.0001625.3e-05

6e-050.0002030.0002286.5e-05

4.3e-056.9e-054.2e-05

0.0004760.000534

1.6e-05

0.0001860.000209

0.0001140.000128

7.9e-050.000140.0001578.3e-05

4.3e-050.0003750.0004215e-05

0.0003610.0001440.0002150.0016050.0003810.0003680.00011

0.0002690.000302

0.0002690.000302

0.0002690.000302

4.7e-050.0001920.0002154.6e-05

4.7e-050.0001920.0002154.6e-05

4.7e-050.0001920.0002154.6e-05

0.0004010.0006080.0006780.0017050.0005440.0003870.000511

0.0004010.0006080.0004910.0017050.0003340.0003870.000511

2.3e-050.0001430.0001612.3e-05

0.0003780.0006080.0003480.0017050.0001730.0003640.000511

0.0001870.00021

0.0001870.00021

1e-050.0009960.0012441.2e-05

0.0009960.001119

0.0009960.001119

1e-050.0001251.2e-05

1e-050.0001251.2e-05

8.8e-050.0007660.0010348.8e-051.6e-05

5.8e-050.0007660.000866.3e-051.6e-05

1.6e-05

5.8e-050.0005770.0006486.3e-05

0.0001890.000212

3e-050.0001742.5e-05

3e-055.7e-052.5e-05

0.000117

0.0007150.0002210.0008610.003310.0022660.0007290.000652

0.0007150.0002210.0008610.003310.0022660.0007290.000652

0.0003530.0001250.0002430.0016050.0002450.0003533.3e-05

1.6e-05

8e-050.0001148.3e-05

0.0001129.6e-050.0017050.0001190.0001110.000397

2.9e-050.000150.0001683.3e-05

0.0001720.000194

2.8e-05

1.7e-05

3.3e-05

6.8e-05

5.7e-05

9e-05

0.0001410.0001830.0012110.000149

0.0001130.000127

1e-06

0.0028170.0015450.0036130.0050150.0014590.0027050.001941

0.0028170.0015450.0034050.0050150.0012250.0027050.001941

5.6e-050.0014840.0026080.0012255.4e-05

0.0027610.0015450.0019210.0024070.0026510.001941

0.0002080.000234

0.0002080.000234

1e-050.0002080.0002331e-05

1e-050.0002080.0002331e-05

1e-050.0002080.0002331e-05

0.0066990.0076950.0127470.0072220.0148880.006610.00681

0.0066990.0076950.0127470.0072220.0148880.006610.00681

8.3e-05

8.3e-05

8.3e-05

2.2e-056.6e-052.1e-05

2.2e-056.6e-052.1e-05

2.2e-056.6e-052.1e-05

1.5e-056.5e-051.5e-05

1.5e-056.5e-051.5e-05

1.5e-056.5e-051.5e-05

0.0001550.0002160.000154

0.0001550.0002160.000154

3.3e-055e-052.3e-05

6e-061e-056e-06

2.2e-054.7e-052.1e-05

3e-051.8e-053.4e-05

2.9e-052.9e-053e-05

1.4e-051.4e-051.9e-05

6e-062.1e-056e-06

1.5e-052.7e-051.5e-05

0.0065070.0076950.0127470.0072220.0144580.006420.00681

3.7e-050.0001340.000153.6e-05

3.7e-050.0001340.000153.6e-05

6e-067.6e-057e-06

6e-067.6e-057e-06

0.0002190.000452

8.2e-05

0.0002190.000246

0.000124

1.8e-058.5e-051.6e-05

1.8e-058.5e-051.6e-05

3.6e-055e-054.3e-05

3.6e-055e-054.3e-05

8.1e-05

8.1e-05

1.8e-056.4e-051.7e-05

1.8e-056.4e-051.7e-05

2.9e-050.0001490.0001683.1e-05

2.9e-050.0001490.0001683.1e-05

3e-050.0001380.0001553.6e-05

3e-050.0001380.0001553.6e-05

1.7e-050.0002071.8e-05

1.7e-050.0001061.8e-05

0.000101

0.0031980.0016470.0016830.0022070.0011940.0030990.001198

1.7e-05

0.0001150.000129

0.0004260.000479

0.0031610.0016470.0011420.0022070.0004760.0030590.001148

3.3e-05

3.7e-050.000114e-05

0.0023030.0060480.0023410.0050150.0011940.0022140.005168

0.0023030.0060480.0023410.0050150.0011940.0022140.005168

0.0064470.0070990.000144

0.0015550.001747

0.0031430.003531

0.0001280.000144

0.0008040.000903

0.0008170.000918

0.0003040.0003950.000348

4.6e-053.1e-055.6e-05

9.4e-050.0001420.000108

4.5e-058.7e-055.2e-05

4.3e-059.2e-054.8e-05

7.6e-054.3e-058.4e-05

0.000157

6.8e-05

8.9e-05

6.4e-05

6.4e-05

2e-050.0001132.1e-05

2e-050.0001132.1e-05

0.0001960.0002030.0003020.00021

0.0001960.0002030.0003020.00021

5.5e-059.3e-055.6e-05

5.5e-059.3e-055.6e-05

9.4e-05

9.4e-05

8.1e-05

8.1e-05

0.000106

0.000106

6.1e-050.0001086.8e-05

6.1e-050.0001086.8e-05

1.6e-057.2e-051.8e-05

1.6e-057.2e-051.8e-05

2.1e-050.0001672.3e-05

0.000109

2.1e-054.9e-052.3e-05

9e-06

0.0009140.001027

0.0009140.001027

0.000111

2.1e-05

9e-05

1.6e-050.0001211.9e-05

1.6e-055.7e-051.9e-05

6.4e-05

9e-05

9e-05

8.3e-059.9e-058.8e-05

3.3e-052.9e-053.6e-05

2.4e-05

5e-054.6e-055.2e-05

4.3e-050.0002520.0002835.2e-05

4.3e-050.0002520.0002835.2e-05

0.0002670.0003

0.0002670.0003
