# Supplementary material for: MetaMeta: integrating metagenome analysis tools to improve taxonomic profiling
Source: Microbiome. 2017 Aug 14;5:101. doi: 10.1186/s40168-017-0318-y (PMC5557516; doi:10.1186/s40168-017-0318-y)
Supplement: Supplementary file 2 — Additional File with interactive charts for all CAMI toy set results on default, very-precise and very-sensitive mode. File prefix S, M, and H for low, medium and high complexity, respectively. (TAR 3573 kb) [file 40168_2017_318_MOESM2_ESM.tar › H_S002__insert_180_default.html]

Javascript must be enabled to view this page.

magnitude
magnitudeUnassigned

clark.parsed\_profile
dudes.parsed\_profile
final.metametamerge.profile
gottcha.parsed\_profile
kaiju.parsed\_profile
kraken.parsed\_profile
motus.parsed\_profile

0.9999999999999980.9999969999999991.0000080.9999930000000021.0000020.9999959999999980.999994

0.08440800000000010.0569950.0532220.0508010.0796220.08121400000000010.050449

1e-062.6e-050

1e-062.6e-050

1e-062.6e-050

1e-062.6e-050

1e-062.6e-050

1e-062.6e-050

3e-060.000123e-06

3e-060.000123e-06

8.7e-05

8.7e-05

5.4e-05

5.4e-05

3.3e-05

3.3e-05

3e-063.3e-053e-06

3e-063.3e-053e-06

3e-063.3e-053e-06

8e-06

3e-062.5e-053e-06

0.0448140.0248270.0265760.0246970.0411920.0432410.022455

6.3e-050.0002886.2e-05

6.1e-050.0001766e-05

5.7e-050.0001125.5e-05

5.7e-050.0001125.5e-05

9e-063.9e-058e-06

4.8e-057.3e-054.7e-05

2e-062.7e-053e-06

2e-062.7e-053e-06

2e-062.7e-053e-06

02.2e-050

02.2e-050

02.2e-050

2e-061.5e-052e-06

2e-061.5e-052e-06

2e-061.5e-052e-06

3.4e-05

3.4e-05

3.4e-05

3.4e-05

2e-067.8e-052e-06

2e-067.8e-052e-06

1e-061.8e-051e-06

1e-061.8e-051e-06

2.6e-05

2.6e-05

1e-063.4e-051e-06

1e-063.4e-051e-06

9e-068.4e-051.2e-05

9e-068.4e-051.2e-05

6e-065.1e-058e-06

08e-060

08e-060

6e-064.3e-058e-06

2e-065e-063e-06

2e-061.6e-052e-06

1e-061.1e-052e-06

1e-061.1e-051e-06

3e-063.3e-054e-06

1e-067e-061e-06

1e-067e-061e-06

2e-062.6e-053e-06

2e-06

8e-06

01e-060

2e-067e-062e-06

01e-060

07e-061e-06

0.0035090.0017310.0018480.0036140.0035980.0034060.001124

0.0024570.0013490.0014820.0020080.0023720.0023650.000911

2.6e-05

2.6e-05

2.6e-05

1.8e-050.0001481.9e-05

8e-065.3e-058e-06

8e-065.3e-058e-06

1e-059.5e-051.1e-05

3.9e-05

3.5e-05

1e-052.1e-051.1e-05

0.0024390.0013490.0014820.0020080.0021980.0023460.000911

1.6e-054.7e-052e-05

1.6e-054.7e-052e-05

6.3e-050.000166.1e-05

2.9e-057.7e-052.7e-05

3.4e-058.3e-053.4e-05

1.2e-051.9e-051.1e-05

6e-061e-065e-06

6e-061.5e-056e-06

3e-06

0.0023480.0013490.0014820.0020080.0019720.0022540.000911

1e-053e-051.2e-05

0.0023380.0013490.0014820.0020080.0019420.0022420.000911

0.0003634.7e-050.0005440.0003741.4e-05

0.0003634.7e-050.0005440.0003741.4e-05

3.6e-055e-053.7e-05

3.6e-055e-053.7e-05

3e-054.3e-053.1e-05

3e-054.3e-053.1e-05

3.2e-055e-053.3e-05

3.2e-055e-053.3e-05

4.1e-055.3e-054.2e-051.4e-05

4.1e-055.3e-054.2e-051.4e-05

3e-054.3e-052.9e-05

3e-054.3e-052.9e-05

1.4e-054.5e-051.4e-05

1.4e-054.5e-051.4e-05

0.000184.7e-050.0002220.000188

7.9e-050.0001038.4e-05

0.0001014.7e-050.0001190.000104

3.8e-05

3.8e-05

0.0006890.0003350.0003660.0016060.0006820.0006670.000199

0.0006680.0003350.0003660.0016060.0005090.0006440.000199

9e-065.8e-051e-05

9e-065.8e-051e-05

1.1e-055.1e-051.1e-05

1.1e-055.1e-051.1e-05

0.0006480.0003350.0003660.0016060.00040.0006230.000199

5.3e-05

0.0006320.0003350.0003660.0016060.0003110.0006070.000199

1.6e-053.6e-051.6e-05

2.1e-050.0001732.3e-05

2.1e-050.0001732.3e-05

2.1e-050.0001732.3e-05

0.0027150.0023170.0022310.0039150.002210.0026070.002049

0.0027150.0023170.0022310.0039150.002210.0026070.002049

0.0027150.0023170.0022310.0039150.002210.0026070.002049

4e-06

4e-06

0.0011760.0014380.0011740.0021080.0010250.0011310.001279

02e-060

01.2e-050

5e-06

9e-06

3e-06

9e-06

0.0011760.0014380.0011740.0021080.0009850.0011310.001279

0.0015390.0008790.0010570.0018070.0011810.0014760.00077

0.0015320.0008790.0010570.0018070.0011090.0014690.00077

03e-060

5e-06

4e-060

03e-060

3e-06

6e-06

4e-06

06e-060

03e-060

3e-06

5e-068e-065e-06

3e-06

2e-065e-062e-06

6e-06

4e-06

6e-06

6e-067.7e-055e-06

6e-067.7e-055e-06

6e-067.7e-055e-06

1e-05

1e-05

6e-064.4e-055e-06

06e-060

1e-061e-051e-06

1e-061.4e-050

4e-061.4e-054e-06

2.3e-05

1.1e-05

1.2e-05

0.0012510.0007360.0010510.0018070.0012240.001199

0.0012510.0007360.0010510.0018070.0012240.001199

0.0012510.0007360.0010510.0018070.0012240.001199

0.0012510.0007360.0010510.0018070.0012240.001199

1e-062.2e-050

0.001250.0007360.0010510.0018070.0012020.001199

0.0040010.0023850.0026020.002510.00390.0038460.001986

3.7e-050.0001244.5e-05

01.3e-050

01.3e-050

01.3e-050

9e-064.6e-058e-06

9e-062.9e-058e-06

4e-06

2e-061.1e-052e-06

7e-061.4e-056e-06

01.7e-050

01.7e-050

2.1e-054.1e-053.1e-05

2e-052.8e-053.1e-05

1e-061.2e-051.4e-05

1.9e-051.6e-051.7e-05

1e-061.3e-050

1e-061.3e-050

7e-062.4e-056e-06

7e-062.4e-056e-06

7e-062.4e-056e-06

1.3e-050.0002321.4e-05

9e-060.0001761e-05

03e-050

03e-050

1e-061.2e-051e-06

1e-061.2e-051e-06

2e-061.7e-052e-06

2e-061.7e-052e-06

01.2e-050

01.2e-050

6e-061.5e-056e-06

6e-061.5e-056e-06

02.2e-051e-06

01.4e-051e-06

8e-06

06.8e-050

1e-06

02e-060

4e-06

1e-05

1e-06

9e-06

1.2e-05

3e-06

06e-060

5e-06

1.3e-05

02e-060

4e-065.6e-054e-06

4e-065.6e-054e-06

3e-062.8e-053e-06

1e-062.8e-051e-06

0.0039510.0023850.0026020.002510.0035440.0037870.001986

0.0039510.0023850.0026020.002510.0035440.0037870.001986

0.0039510.0023850.0026020.002510.0035440.0037870.001986

1e-062.1e-051e-06

0.003950.0023850.0026020.002510.0035230.0037860.001986

0.033260.0176580.0188440.0128510.0298110.0321040.017296

0.033260.0176580.0188440.0128510.0298110.0321040.017296

3e-061.4e-053e-06

3e-061.4e-053e-06

3e-061.4e-053e-06

0.0332570.0176580.0188440.0128510.0297970.0321010.017296

1e-061.9e-050

1e-064e-060

1.4e-05

1e-06

0.0014310.0009080.0010310.0018070.0012830.0013740.00067

0.0014310.0009080.0010310.0018070.0012830.0013740.00067

4e-064.8e-054e-06

1e-061.1e-051e-06

3e-061.1e-053e-06

02.3e-050

3e-06

0.0318210.016750.0178130.0110440.0284470.0307230.016626

2.8e-05

9.3e-05

1.1e-053.5e-051e-05

6e-063.1e-056e-06

0.0318040.016750.0178130.0110440.0281270.0307070.016626

0.000133

2.2e-058.1e-051.5e-05

2.2e-058.1e-051.5e-05

2.2e-058.1e-051.5e-05

2.2e-058.1e-051.5e-05

2.2e-058.1e-051.5e-05

2.2e-058.1e-051.5e-05

0.0395680.0321680.0266460.0261040.0382030.0379550.027994

0.0395680.0321680.0266460.0261040.0382030.0379550.027994

0.0018840.0095020.0026310.0061240.0016350.001810.009248

0.0018840.0095020.0026310.0061240.0016350.001810.009248

01.1e-050

09e-060

2e-06

01.4e-050

01.4e-050

0.0018840.0095020.0026310.0061240.001610.001810.009248

1e-061.5e-051e-06

1.5e-05

04e-060

1e-067e-061e-061.7e-05

0.0018820.0095020.0026310.0061240.0015690.0018080.009231

2e-062.5e-052e-06

01.2e-050

01.2e-050

01.2e-050

2e-061.3e-052e-06

2e-061.3e-052e-06

2e-061.3e-052e-06

0.000440.0013110.0016910.0035140.0008280.000420.001155

0.0004340.0013110.0016910.0035140.0008070.0004160.001155

9e-060.0010730.001360.0019080.0002949e-060.000928

9e-060.000270.0002949e-06

0.0010730.001090.0019080.000928

1.3e-059.5e-051.2e-05

1.1e-056e-051e-05

2e-063.5e-052e-06

0.0004120.0002380.0003310.0016060.0004010.0003950.000227

0.0004120.0002380.0003310.0016060.0004010.0003950.000227

01.7e-050

9e-060

08e-060

6e-062.1e-054e-06

6e-062.1e-054e-06

1e-06

1e-060

6e-061.1e-054e-06

8e-06

0.0372420.0213550.0223240.0164660.0357150.0357230.017591

0.0372420.0213550.0223240.0164660.0356880.0357230.017591

0.037240.0213550.0223240.0164660.0356160.0357220.017591

0.037240.0213550.0223240.0164660.0356160.0357220.017591

1e-061.7e-050

1e-061.7e-050

07e-060

05e-060

2e-06

02.9e-050

01.1e-050

01.8e-050

1e-061.9e-051e-06

1e-061.9e-051e-06

2.7e-05

1e-05

1e-05

7e-06

7e-06

1e-05

1e-05

0.9153339999999990.9430019999999990.9467860.9491920000000020.9203799999999980.9185639999999980.949545

0.0001090.0003528.6e-05

0.0001090.0003528.6e-05

0.0001090.0003528.6e-05

0.0001090.0003528.6e-05

4e-066.2e-055e-06

4e-066.2e-055e-06

3.7e-055.3e-052.6e-05

1.5e-052.2e-051.1e-05

2.2e-051.8e-051.5e-05

1.3e-05

1.4e-054.7e-051.6e-05

4e-062.7e-051e-05

1e-052e-056e-06

9e-068.1e-058e-06

9e-063.3e-058e-06

4.8e-05

4.5e-050.0001093.1e-05

4e-063.3e-058e-06

1.5e-052.5e-058e-06

1.8e-052.3e-059e-06

8e-062.8e-056e-06

6.1e-050.0003026.9e-05

6.1e-050.0003026.9e-05

6.1e-050.0003026.9e-05

3.9e-050.0002364.9e-05

4.7e-05

4.7e-05

2e-055.2e-052.3e-05

2e-055.2e-052.3e-05

4e-064.2e-059e-06

4e-064.2e-059e-06

1.5e-059.5e-051.7e-05

3e-05

3.8e-05

1.5e-052.7e-051.7e-05

2.2e-056.6e-052e-05

9e-064.8e-056e-06

9e-064.8e-056e-06

1.3e-051.8e-051.4e-05

1.3e-051.4e-051.4e-05

4e-06

3e-055e-052.3e-05

3e-055e-052.3e-05

3e-055e-052.3e-05

3e-055e-052.3e-05

3e-055e-052.3e-05

3e-055e-052.3e-05

0.1563380.2171620.1975120.2212860.1175420.1388120.248789

0.0002270.0080120.0019250.0002240.00711

0.0002270.0080120.0019250.0002240.00711

0.0002270.0080120.0019250.0002240.00711

1.6e-05

1.6e-05

8.6e-05

8.6e-05

0.0080120.0016130.007094

0.0065280.007094

0.0014840.001613

8e-067.3e-057e-06

8e-061.4e-057e-06

5.9e-05

0.000153

0.000153

0.0002190.000217

0.0002190.000217

6.8e-05

6.8e-05

6.8e-05

6.8e-05

6.8e-05

0.0015560.0007750.0049010.0044170.0022550.0014960.004713

0.0015560.0007750.0049010.0044170.0022550.0014960.004713

0.0015560.0007750.0049010.0044170.0022550.0014960.004713

9.4e-05

9.4e-05

0.0011910.0005930.0008580.0018070.0011540.001140.00062

0.0011910.0005930.0008580.0018070.0011540.001140.00062

0.000102

0.000102

0.0003650.0001820.0040430.002610.0009050.0003560.004093

0.0003650.0001820.0003670.002610.0009050.000356

0.0036760.003995

9.8e-05

0.1260060.1822360.1288970.1832350.0767780.1129450.17593

0.0242150.0264970.0223670.0336330.0238480.0164360.041819

0.0166930.0220470.0101410.0257020.0091120.0075560.036713

0.0001829.4e-050.0015060.0002150.000175

0.0001829.4e-050.0015060.0002150.000175

7.3e-050.0002147.7e-05

5.8e-05

0.000107

7.3e-054.9e-057.7e-05

0

1.3e-050.0001161.6e-05

1.3e-050.0001161.6e-05

0.0015790.0010270.0011350.0018070.0010090.0015331.6e-05

1e-05

2e-065e-064e-06

2.1e-05

1e-064e-061e-06

2e-06

6e-06

8e-06

3.6e-053e-053.6e-05

6e-066e-067e-06

2e-063e-062e-06

0.0014880.0010270.0011350.0018070.0008640.001434

08e-06

8e-061e-066e-06

2e-06

3.6e-055.5e-054.3e-05

0.000169

7.5e-05

9.4e-05

8e-067e-067e-06

8e-067e-067e-06

0.000112

0.000112

0.000116

0.000116

0.0137760.0205770.0082230.0205820.0050850.004660.036599

0.000230.00025

2.4e-05

0.0002650.000288

0.0003290.000358

0.0001411e-060.000203

1.1e-05

3.6e-051.8e-055.2e-05

7.2e-05

4e-055.4e-055.2e-05

5e-06

2e-06

0.0098870.0045810.0016330.0035140.0004970.004987

0.0001295.6e-050.0019081.5e-050.0001290.018927

1.6e-05

0.0002220.000241

3.7e-052.9e-054.4e-05

0.0013270.0058820.0005080.002610.0001130.0018360.006707

3e-06

0.000199

5.2e-050.0002426.1e-05

8.4e-05

4.2e-059e-064.3e-05

2.5e-059.2e-052.9e-05

0

0.0001711.7e-050.000189

3.2e-05

0.0005480.000596

6e-050.0013460.0017040.0023099e-066.2e-050.002252

4.7e-050.0001134.8e-05

0.0001066e-060.000107

9e-063e-061.7e-05

0.0002970.000323

9e-06

0.0004920.0064940.0009350.0054222.1e-050.0004790.002158

0.0003140.000341

0.0006710.0001930.0004310.0027119.2e-050.000667

1.2e-05

0

0.0001575.5e-050.0003020.000167

7e-054.9e-057.9e-05

5.6e-05

2.1e-054.7e-052.6e-05

2.2e-050.0001173.5e-05

0.0002890.000314

0.0002340.001970.0005180.0021081.9e-050.0003350.001552

0.000165

3.4e-05

9e-06

0.000122

0.0010620.0003490.0007830.0018070.0016650.0010889.8e-05

0.0010620.0003490.0007830.0018070.0016650.0010889.8e-05

0.000102

0.000102

0.000137

0.000137

0.0002656e-050.0002710.0011870.00028

0.0002656e-050.0002710.0011190.00028

1.8e-051.8e-051.9e-05

2.2e-051.1e-052.2e-05

1.4e-052.1e-051.6e-05

6e-06

0.0002116e-050.0002710.0010630.000223

6.8e-05

6.8e-05

0.0050010.0027820.0048570.0031120.0077360.0048190.001287

1.7e-055.3e-051.7e-05

1.7e-055.3e-051.7e-05

0.0049580.0027820.0033080.0031120.004930.0047793.3e-05

2.2e-05

1.2e-05

0.0001664.9e-050.0001080.000206

6.8e-05

5e-06

0.0046670.0026660.0033080.0031120.0038870.004486

6e-062.8e-055e-06

8.8e-05

6.5e-05

2.4e-05

5.1e-05

7.1e-056.7e-051.9e-053.7e-053.3e-05

1.8e-057.8e-051.7e-05

3e-056.7e-052.8e-05

2.4e-05

1.6e-05

6e-06

5.5e-05

7.4e-05

1.4e-05

1e-05

1.9e-05

1.2e-05

3.5e-05

8e-06

2.4e-05

9e-06

0.000102

0.000112

0.000112

2.6e-050.0015490.0026412.3e-050.001254

0.0015490.0025580.001254

2.6e-058.3e-052.3e-05

4.7e-050.0012960.001455.3e-050.000718

0.000164

0.000164

4.7e-057.6e-055.3e-05

4.7e-057.6e-055.3e-05

0.0002810.000306

0.0002810.000306

0.000167

0.000167

0.0010150.0003840.000718

0.0006610.000718

0.0003540.000384

0.000353

3.2e-05

2.2e-05

5.7e-05

2.7e-05

2.6e-05

5.8e-05

2.9e-05

5.1e-05

5.1e-05

4.8e-051e-063.8e-055.9e-05

4.8e-051e-063.8e-055.9e-05

4e-066e-064e-06

4e-064e-067e-06

2.1e-051.8e-052.5e-05

1.8e-059e-062.2e-05

1e-061e-061e-061e-06

0.0005227e-050.0040050.002610.0007060.0005530.001742

0.0004797e-050.0040050.002610.0005040.0005180.001742

6e-06

3e-053.1e-053.3e-05

7.4e-05

2e-06

1.2e-05

0.0016030.001742

4.1e-054e-064.3e-05

4.2e-052.1e-054.6e-05

1.2e-05

4.1e-051.7e-054.4e-05

2e-068e-060.0024020.002616e-061.8e-05

1.1e-05

1e-05

4e-05

4.7e-051.1e-055e-05

2.4e-051.3e-052.6e-05

6e-06

4.7e-056e-064.5e-05

1.5e-059e-061.8e-05

0.000196.2e-050.0002130.000195

0.00011

0.00011

4.3e-059.2e-053.5e-05

4.3e-059.2e-053.5e-05

0.0016080.0015370.0017970.0022090.0035610.003090.001359

0.0016020.0015370.0017970.0022090.0035010.0030860.001359

0.0016020.0015370.0017970.0022090.0035010.0030860.001359

6e-066e-054e-06

6e-066e-054e-06

3.1e-055.8e-052.6e-05

3.1e-055.8e-052.6e-05

3.1e-055.8e-052.6e-05

0.1017910.1557390.106530.1496020.052930.0965090.134111

0.0027550.0015610.0059120.0071280.0035060.0026530.001931

0.0015730.000840.0051020.0054210.0023390.0015220.001462

0.0014170.000840.001050.0018070.0011120.001370.000846

2.2e-050.0016630.0018071.9e-052.1e-05

4.3e-053e-055.1e-05

2.8e-050.0016630.0018072.4e-053e-05

3.7e-052.5e-052.6e-05

2.6e-053.4e-052.4e-05

0.0007260.0010950.000616

2.7e-055.3e-052.3e-05

2.7e-055.3e-052.3e-05

0.0011550.0007210.000810.0017070.0011140.0011080.000469

1.5e-05

0.0011550.0007210.000810.0017070.0010550.0011080.000469

4.4e-05

0.0390380.095260.0525420.0759050.0207750.0419440.087634

0.038940.095260.0525420.0759050.0206710.0418530.087634

0.000152

2.5e-05

5.4e-05

0.0051630.0098490.0032510.0062250.0011050.0049580.007371

4.3e-056.2e-054.6e-05

4e-050.0015710.0017073.3e-054.2e-05

2.4e-05

3e-053.4e-053e-05

7.6e-05

0.0016040.0006230.0005850.0055220.0001620.0016110.004234

8.3e-056e-050.0036030.0039164.8e-057.8e-051.6e-05

6.6e-05

2.7e-052.7e-052.9e-05

0.000104

2.5e-05

2.2e-053.7e-050.0048040.0052215e-061.9e-05

0.0041430.0097060.0025130.0059240.0007660.0039870.007812

2.7e-05

0.001960.0040690.002470.0031120.0012940.0062550.004434

0.0243680.0616660.0249880.0339360.013520.0233810.057136

0.0013520.00147

9.8e-05

2.9e-050.0001112.4e-05

0.0008240.0010960.0007820.0019080.0004090.0007950.001411

5.7e-056.2e-05

9.5e-057.3e-059.2e-05

9.3e-05

8.8e-05

0.000187

0.0003110.0081170.0004810.0037150.0002370.0002990.003265

0.0017990.001955

3.1e-050.0024950.0027115.8e-052.9e-05

6e-05

6.2e-050.0001096.7e-05

5e-065.2e-055e-06

4.3e-053.7e-050.0018480.0020081.7e-054.4e-05

9.8e-050.0001049.1e-05

7.3e-054e-056.5e-05

2.5e-053.9e-052.6e-05

2.5e-05

4.5e-050.0002723.4e-05

4.5e-050.0002723.4e-05

8e-06

8.4e-05

4.5e-053.8e-053.4e-05

6.1e-05

1.9e-05

6.2e-05

0.0537590.0559660.0393680.0574320.0248840.0459380.04175

0.0537440.0559660.0377050.0556250.0247890.0459070.04175

9.4e-056.5e-059.1e-05

0.0001314.7e-050.0001310.000106

0.0006790.000738

4e-05

0.000101

1.8e-05

0.0006780.0010860.000620.0019080.0003360.000656

5.8e-05

0.0002950.0066275.1e-050.0002810.000105

0.0004660.0009820.0005270.0019080.0003050.0004490.00078

9e-060.0015710.0017072.9e-052.7e-05

6.3e-052.6e-056e-05

0.0006950.0026450.000520.0027110.0001610.0005280.002858

0.0019340.0008340.0008290.0017070.0004890.0018520.000565

0.0007670.0001940.000290.0037150.0003970.0007410.000118

8e-05

5.2e-051.5e-055.1e-058e-06

0.000189

5.4e-054.8e-050.000290.0017074.5e-050.0001540.00041

9.3e-05

0.0002620.0011140.0003280.002410.0001490.0002230.001936

0.000430.0003340.0003450.0017070.0001620.000420.000659

0.0004120.000448

0.0020920.002140.002619

8.8e-05

6e-05

8.3e-050.0005193.9e-058.2e-050.000564

0.0002880.000312

3e-056e-063.4e-05

7.8e-055.9e-058.1e-05

4.5e-05

0.0251590.0294920.0137260.0135540.006510.0163210.025043

0.000127

2.8e-059e-062.7e-051.9e-05

0.0013990.0028110.0020490.002510.001920.0032650.002514

0.0002970.0002430.0001790.0002870.000367

1.7e-05

2.3e-057e-062.4e-058e-06

6e-062.1e-052e-065e-065.1e-05

0.0085230.008010.005030.004920.0028760.008223

8.1e-057.7e-057.6e-05

7e-065e-060.0016630.0018071e-067e-06

3.8e-051.8e-050.0027114.9e-050.000115

0.0120620.006280.0056360.0040160.0075890.0116960.003687

0.000108

1.5e-050.0016630.0018079.5e-053.1e-05

6e-060.0016630.0018072.4e-052.2e-05

5e-05

9e-062.1e-059e-06

0.0017060.0013510.0013890.0016273.2e-05

0.0017060.0013510.0013890.0016273.2e-05

5.6e-05

3.2e-053.6e-051.8e-051.6e-05

3e-055e-053e-05

0.0016440.0013510.0012470.001579

1.6e-05

0.0044880.0029520.0073570.0091370.0021040.0043130.002764

1.5e-053.2e-051.5e-05

1.5e-053.2e-051.5e-05

0.0007640.0008860.0007150.0018070.0006340.0007350.000558

0.0007640.0008860.0007150.0018070.0006340.0007350.000558

0.0037090.0020660.0066420.007330.0014380.0035630.002206

3.2e-053.7e-053.2e-05

0.0036170.0020240.0019270.002510.0010460.0034760.002206

0.0002810.000305

9e-06

2e-052.3e-050.0026790.0029121.5e-051.9e-05

2.5e-051.3e-052.5e-05

1.5e-051.9e-050.0017550.0019081.3e-051.1e-05

0.0026410.0013710.0192510.0035140.0098180.0025230.013223

0.0022980.0013710.0018750.0035140.0022220.0022070.00118

0.0022980.0013710.0018750.0035140.0022220.0022070.00118

0.0022980.0013710.0018750.0035140.0022220.0022070.00118

0.0019040.0011320.0011090.0020080.0017840.0018270.00051

0.0003940.0002390.00030.0015060.0004380.000380.000164

0.0004660.000506

0.000290.0173760.0073030.0002670.012043

0.000290.0168470.0066420.0002670.012043

0.000290.0168470.0066420.0002670.012043

0.000510.000554

0.0046970.005104

6.7e-050.0001387.4e-05

0.0005580.000606

0.0110820.012043

0.0002230.000240.000193

0.0005290.000661

0.0005290.000661

0.0005290.000575

8.6e-05

5.3e-050.0002934.9e-05

5.3e-050.0002934.9e-05

2.2e-057.6e-052.3e-05

2.2e-057.6e-052.3e-05

3.1e-050.0001132.6e-05

3.1e-050.0001132.6e-05

0.000104

0.000104

0.0259080.032780.0364510.030120.0266980.0216240.047813

7e-063.5e-055e-06

7e-063.5e-055e-06

7e-063.5e-055e-06

7e-063.5e-055e-06

2.5e-050.0002253.8e-05

1.3e-050.0001342.5e-05

9e-067.9e-052.1e-05

3e-064.4e-051e-05

6e-063.5e-051.1e-05

4e-065.5e-054e-06

4e-065.5e-054e-06

1.2e-059.1e-051.3e-05

9e-065e-051.2e-05

9e-065e-051.2e-05

3e-064.1e-051e-06

3e-064.1e-051e-06

0.004380.0032420.0028970.0042160.0031450.004210.002397

0.0023220.0018520.0015080.0021080.0011410.0022490.001473

7e-065.6e-055e-06

7e-065.6e-055e-06

0.0023030.0018520.0015080.0021080.0010420.0022270.001473

0.002270.0018520.0015080.0021080.0009330.0021960.001473

1e-053.4e-057e-06

4e-061.9e-053e-06

1e-069e-061e-06

5e-061e-055e-06

4e-061.6e-054e-06

3e-068e-064e-06

6e-061.3e-057e-06

1.2e-054.3e-051.7e-05

6e-062.8e-051.2e-05

6e-061.5e-055e-06

0.0020360.001390.0013890.0021080.0018640.0019480.000924

7e-064.7e-057e-06

7e-064.7e-057e-06

0.0019260.001390.0013890.0021080.0015690.0018410.000924

0.0019260.001390.0013890.0021080.0015690.0018410.000924

3e-062.6e-052e-06

3e-062.6e-052e-06

7.2e-050.0001117.8e-05

1.4e-051.3e-051.8e-05

000

3.8e-053.8e-054e-05

4.6e-05

2e-051.4e-052e-05

1.2e-055.6e-059e-06

1.2e-055.6e-059e-06

9e-064.2e-055e-06

9e-064.2e-055e-06

7e-061.3e-056e-06

7e-061.3e-056e-06

1.6e-059e-057e-06

6e-063.8e-054e-06

6e-063.8e-054e-06

1e-055.2e-053e-06

1e-055.2e-053e-06

6e-065e-056e-06

6e-065e-056e-06

6e-065e-056e-06

0.0214960.0295380.0335540.0259040.0232930.0173710.045416

0.0033340.0025590.0052180.0040160.0071990.0032430.003354

4.8e-05

4.8e-05

1.2e-050.0001121.4e-05

6e-065.4e-057e-06

6e-065.8e-057e-06

1.5e-053.3e-051.6e-05

9e-063e-051e-05

1e-06

6e-062e-066e-06

0.0033070.0025590.0052180.0040160.0070060.0032130.003354

5.1e-05

6.5e-05

2e-052.6e-052.3e-05

3.3e-051.9e-053.5e-05

0.000610.0010790.0007120.0019080.0005680.0005860.000841

9.7e-05

3.1e-05

2.6e-051e-062.7e-05

2.4e-05

0.0015240.0018980.001469

2.1e-051.7e-051.4e-05

3.7e-053.9e-056e-063.8e-05

0.0023980.0014380.0015870.0021080.0024870.0023060.000947

9e-063.5e-059e-06

3.2e-052.6e-053.6e-05

1.9e-057e-062.6e-05

1e-053e-069e-061.4e-05

6e-05

7e-06

1.8e-054.6e-052e-05

1.9e-05

2.4e-052.3e-052.5e-05

0.0013950.001516

1.8e-052.9e-052e-05

3.2e-053.6e-053.4e-05

0.0001280.0005810.000108

5.3e-050.0001824.5e-05

4e-062.8e-054e-06

1.3e-052.8e-051.3e-05

1.5e-054.7e-052e-05

9e-06

5e-063.1e-053e-06

7e-064.8e-055e-06

9e-06

9e-06

1.3e-050.0001251.7e-05

5.1e-05

2e-061.6e-053e-06

4e-063.4e-056e-06

7e-062.4e-058e-06

1.7e-059.4e-051.6e-05

7e-063.4e-056e-06

2e-062.9e-052e-06

8e-063.1e-058e-06

1.2e-054.4e-051e-05

1.2e-054.4e-051e-05

1e-055.4e-058e-06

1e-055.4e-058e-06

1.9e-05

0

2e-06

1.7e-05

1.4e-056.3e-051.2e-05

1.4e-056.3e-051.2e-05

3.5e-059.7e-052.4e-05

3.5e-059.7e-052.4e-05

3.5e-059.7e-052.4e-05

1.4e-050.00011.2e-05

3e-065.1e-052e-06

3e-065.1e-052e-06

1.1e-054.9e-051e-05

1.1e-054.9e-051e-05

4.4e-050.0001754.3e-05

4.4e-050.0001754.3e-05

4.4e-050.0001754.3e-05

4.3e-055e-053.2e-05

4.3e-055e-053.2e-05

4.3e-055e-053.2e-05

0.000164

0.000164

0.000164

0.0002030.0006240.0002088.1e-05

0.000190.0005640.0001928.1e-05

5.4e-058.2e-055.3e-05

8.1e-05

0.0001360.0004820.000139

1.3e-056e-051.6e-05

1.3e-056e-051.6e-05

2e-050.0010120.0011731.9e-05

2e-057.2e-051.9e-05

2e-057.2e-051.9e-05

0.0006640.000722

0.0006640.000722

0.0003480.000379

0.0003480.000379

0.010590.0137940.0103490.0089360.0058250.0101680.01872

7.4e-050.0001377.2e-05

7.4e-050.0001377.2e-05

5.1e-050.0001245e-05

5.1e-050.0001245e-05

0.0012270.0019630.008148

0.0012270.0019630.008148

0.0103940.0123930.0081510.0089360.0051820.0099760.010572

0.0002130.000232

0.0103670.0123930.0079380.0089360.0047320.0099460.010572

1.9e-052.4e-051.9e-05

4e-065.5e-055e-06

4e-062.6e-056e-06

0.000113

7.1e-050.0001740.0002350.0003827e-05

0.0002350.000255

0.000174

5.3e-055.1e-05

1.8e-050.0001271.9e-05

0.0058490.0119580.008370.0062250.0037740.0025270.01576

0.0057610.0119580.0045880.0062250.0034410.0024440.01165

0.0057610.0119580.0045880.0062250.0034410.0024440.01165

8.5e-05

8.5e-05

6.5e-050.0001466.3e-05

6.5e-050.0001466.3e-05

2.3e-050.0001022e-05

2.3e-050.0001022e-05

0.0037820.00411

0.0037820.00411

0.0011990.0012270.0086050.0067270.0034760.0009610.007501

0.0007340.0005640.0007130.0067270.0010190.000719

0.000175

0.0007340.0003890.0007130.0067270.0010190.000719

0.000158

0.000158

0.0002042.3e-050.0055130.0004756.6e-050.005918

0.0002042.3e-050.0002620.0004756.6e-05

0.0002

0.0002710.000295

4e-06

4e-06

4e-06

0.0038510.004185

0.0011290.001226

0.0002269.7e-050.0009710.0010120.0001370.000722

0.0002019.7e-050.0004410.000111

0.0003060.000332

2.5e-050.0002392.6e-05

0.0006650.000722

7.7e-050.0003240.000352

0.0003240.000352

7.7e-05

0.0004660.0007260.0005095e-06

0.0001865e-06

0.0004680.000509

0.000280.000258

2e-058.6e-051.9e-05

2e-058.6e-051.9e-05

0.000115

1.9e-05

9.6e-05

1.5e-050.0003580.0002172e-050.000389

0.0003580.000389

1.5e-050.0002172e-05

3.7e-055.5e-052.6e-05

3.7e-055.5e-052.6e-05

3.7e-055.5e-052.6e-05

0.0073040.0067570.005080.0091360.0065930.0070710.005621

0.0073040.0067570.005080.0091360.0065310.0070710.005621

0.0036370.0020410.0022870.002410.0035350.0034740.001563

0.0036370.0020410.0022870.002410.0035350.0034740.001563

0.0035980.0020410.0022870.002410.0034920.0034420.001563

2.5e-054.2e-051.3e-05

0.0035630.0020410.0022870.002410.0033720.003420.001563

3.3e-05

1e-054.5e-059e-06

3.9e-054.3e-053.2e-05

0

3e-068e-062e-06

2e-0603e-06

3e-061.2e-050

3.1e-052.3e-052.7e-05

5e-050.0001075e-05

4e-061.4e-054e-06

4e-061.4e-054e-06

4e-061.4e-054e-06

4.6e-059.3e-054.6e-05

4.6e-059.3e-054.6e-05

1e-061.3e-051e-06

3e-06

1.2e-05

1.3e-05

1.1e-053e-061.1e-05

8e-068e-065e-06

8e-06

8e-06

9e-069e-061e-05

1e-05

1.7e-056e-061.9e-05

0.0036170.0047160.0027930.0067260.0028890.0035470.004058

0.0036170.0047160.0027930.0067260.0028890.0035470.004058

0.0008950.0030740.0009770.0046180.0008560.0007980.002563

07e-060

2.1e-05

1.3e-051.3e-053e-06

4e-062e-06

8e-061e-051.1e-05

3e-064e-063e-06

2.2e-051.3e-051.8e-05

02e-061e-06

2e-05

01e-065e-06

3e-061e-062e-06

5e-06

1.3e-05

2e-062e-062e-06

1e-062e-061e-06

1e-061.3e-052e-06

3e-06

8e-063e-068e-06

4e-06

1e-066e-061e-06

7e-06

2.6e-054e-062.3e-05

6e-061e-065e-06

01.3e-05

0.0002580.0008460.0003540.0018070.0002150.0002470.000783

7e-061e-057e-06

1e-062e-060

3e-056e-063.2e-05

0.0004310.0022280.0006230.0028110.0003960.0003780.00178

9e-062.3e-057e-06

3.1e-053e-061.6e-05

01e-060

4e-06

2.7e-051.3e-052.2e-05

01.5e-05

3e-062e-06

0.0027220.0016420.0018160.0021080.0020330.0027490.001495

7.7e-059.5e-050.000212

0.0026450.0016420.0018160.0021080.0019380.0025370.001495

6.2e-05

6.2e-05

6.2e-05

6.2e-05

6.2e-05

0.0037810.0019550.0022760.0023090.0038670.0036390.001517

2e-055.4e-051.3e-05

2e-055.4e-051.3e-05

2e-055.4e-051.3e-05

2e-055.4e-051.3e-05

2e-055.4e-051.3e-05

0.0037610.0019550.0022760.0023090.0038130.0036260.001517

0.0037610.0019550.0022760.0023090.0038130.0036260.001517

0.003750.0019550.0022760.0023090.0037560.0036150.001517

7e-064.9e-056e-06

7e-064.9e-056e-06

0.0037360.0019550.0022760.0023090.0036070.0036020.001517

0.0037360.0019550.0022760.0023090.0036070.0036020.001517

2e-063.2e-053e-06

2e-063.2e-053e-06

4.1e-05

4.1e-05

5e-062.7e-054e-06

5e-062.7e-054e-06

1.1e-055.7e-051.1e-05

7e-062.8e-057e-06

7e-062.8e-057e-06

4e-062.9e-054e-06

4e-062.9e-054e-06

0.0200810.0144530.0295180.0240950.0373270.0196030.013057

1.1e-059.4e-051.4e-05

1.1e-059.4e-051.4e-05

4e-065.5e-054e-06

4e-065.5e-054e-06

4e-065.5e-054e-06

7e-063.9e-051e-05

7e-063.9e-051e-05

7e-063.9e-051e-05

0.0034430.001930.0029850.0023090.0050750.003325

0.0034430.001930.0029850.0023090.0050750.003325

0.000343

7.7e-05

3.7e-05

4e-05

0.000185

3.8e-05

2.3e-05

3.2e-05

3.5e-05

1.1e-05

1.3e-05

3.3e-05

8.1e-05

2.4e-05

2.9e-05

2.8e-05

0.0001070.000370.0007340.000114

9e-050.000370.0005779.4e-05

0.000370.000402

9e-050.0001759.4e-05

4.8e-05

4.8e-05

1e-055e-051.1e-05

1e-055e-051.1e-05

7e-065.9e-059e-06

7e-065.9e-059e-06

1.6e-050.0001311.1e-05

6e-066.7e-055e-06

6e-066.7e-055e-06

1e-056.4e-056e-06

1e-056.4e-056e-06

1.6e-050.0001582e-05

1.6e-055.8e-052e-05

1.6e-055.8e-052e-05

4.9e-05

4.9e-05

5.1e-05

5.1e-05

0.003290.001930.0026150.0023090.003620.003164

0.00320.001930.0024040.0023090.0030780.003074

0.00320.001930.0024040.0023090.0030780.003074

0.0002110.00023

0.0002110.00023

1.3e-057.4e-051.3e-05

1.3e-057.4e-051.3e-05

2.9e-052.8e-05

2.9e-052.8e-05

8e-069.4e-058e-06

8e-069.4e-058e-06

1.7e-058.7e-051.9e-05

1.7e-058.7e-051.9e-05

2.3e-055.7e-052.2e-05

2.3e-055.7e-052.2e-05

1.4e-053.9e-051.6e-05

1.4e-053.9e-051.6e-05

1.4e-053.9e-051.6e-05

5e-05

5e-05

5e-05

0.0053610.0026930.0091670.0122490.010620.0052760.003879

0.0053610.0026930.0091670.0122490.0103830.0052760.003879

0.0030960.0017030.0039770.0053210.0015310.0029760.002

0.0002160.000234

0.0002160.000234

0.0030960.0017030.0037610.0053210.0012970.0029760.002

6.2e-050.0016140.0027110.0012976.2e-05

0.0030340.0017030.0021470.002610.0029140.002

0.0005157.8e-050.0015070.0016060.0018520.0005390.000516

0.0005157.8e-050.0015070.0016060.0018520.0005390.000516

0.0003030.000329

6.5e-059.2e-056.3e-05

0.000125

0.0002710.000295

5.1e-050.0002145.5e-05

0.0001887.8e-050.0002370.0016060.0002270.0001936.2e-05

4.7e-050.000440.0004785.3e-05

0.000104

7.3e-050.0002560.0002787.7e-05

9.1e-050.0001649.8e-05

0.0003740.0005570.0003350.0017070.0005680.000360.000445

0.000221

0.000221

0.0003740.0005570.0003350.0017070.0003470.000360.000445

2.7e-050.000182.6e-05

0.0003470.0005570.0003350.0017070.0001670.0003340.000445

0.0011940.0003550.0010080.0036150.0030840.0012150.000902

0.0011940.0003550.0010080.0036150.0030840.0012150.000902

0.0001020.0001390.000105

8.3e-05

2.1e-05

4.7e-05

3.7e-050.0002214.3e-05

9.1e-05

1.6e-05

8.7e-05

0.0002330.000253

0.0001017.5e-050.0017070.0001110.00010.000396

0.0001820.0002420.0015310.000191

0.000174

1.6e-05

5.3e-05

0.000141

2.1e-05

0.0007720.000280.0005330.0019080.0005140.0007767.1e-05

1.3e-050.0002351.4e-05

1.3e-050.0002351.4e-05

1.3e-050.0002351.4e-05

1.3e-050.0009910.0012161.4e-05

1.3e-050.0001391.4e-05

1.3e-050.0001391.4e-05

0.0009910.001077

0.0009910.001077

0.0001110.0010320.0013270.0001131.6e-05

3.4e-050.0002063e-05

0.000138

3.4e-056.8e-053e-05

7.7e-050.0010320.0011218.3e-051.6e-05

7.7e-050.0007960.0008658.3e-05

0.0002360.000256

1.6e-05

4.5e-050.0002264.5e-05

4.5e-050.0002264.5e-05

4.5e-050.0002264.5e-05

0.0003170.000344

0.0003170.000344

0.0003170.000344

0.000237

0.00014

0.00014

0.00014

9.7e-05

9.7e-05

9.7e-05

0.0070840.0073140.0130830.0069270.0149180.0069740.007289

0.0070840.0073140.0130830.0069270.0149180.0069740.007289

2e-056.5e-051.9e-05

2e-056.5e-051.9e-05

2e-056.5e-051.9e-05

0.0001630.0001990.00016

0.0001630.0001990.00016

8e-061.9e-058e-06

1e-051.4e-051.6e-05

2.4e-053.8e-052e-05

7e-069e-066e-06

3.5e-052.9e-053.7e-05

1.3e-052.9e-051.4e-05

3.3e-051.7e-053.3e-05

3.3e-054.4e-052.6e-05

0.0068860.0073140.0130830.0069270.0144860.0067790.007289

3.2e-050.0001523.7e-05

3.2e-050.0001523.7e-05

0.000108

8.8e-05

2e-05

0.0002270.000458

0.000126

8.6e-05

0.0002270.000246

3.3e-050.0001763.4e-05

3.3e-050.0001763.4e-05

0.0037860.0019670.0018860.0023090.0013720.0036670.001367

3.2e-05

0.0037490.0019670.0013640.0023090.0005540.0036260.001319

3.7e-050.0001164.1e-05

1.6e-05

0.0005220.000568

0.000134

0.0013250.00144

0.0013250.00144

0.00020.0002110.00030.00021

0.00020.0002110.00030.00021

8.8e-05

8.8e-05

4.2e-050.0002680.0002914.8e-05

4.2e-050.0002680.0002914.8e-05

7.7e-050.0001717.8e-05

7.7e-050.0001717.8e-05

1.9e-050.0002612.1e-05

0.000129

1.9e-050.0001322.1e-05

8.1e-05

8.1e-05

0.000134

0.000134

0.0062710.0068149.5e-05

0.0008080.000878

0.0007910.00086

9.5e-05

0.0031230.003393

0.0015490.001683

4.1e-050.0001624.1e-05

4.1e-050.0001624.1e-05

6e-068.9e-058e-06

6e-068.9e-058e-06

0.0020320.0053470.0021430.0046180.0010620.0019530.004387

0.0020320.0053470.0021430.0046180.0010620.0019530.004387

2.6e-050.0001382.8e-05

2.6e-050.0001382.8e-05

1.7e-058e-052e-05

1.7e-058e-052e-05

0.000134

0.000134

0.000122

0.000122

0.0007520.000817

0.0007520.000817

2.1e-057.2e-052e-05

2.1e-057.2e-052e-05

6.1e-050.0001116.7e-05

6.1e-050.0001116.7e-05

0.000201

0.000106

9.5e-05

0.0003030.0004080.000341

9.5e-050.0001450.000106

4.4e-059.9e-054.8e-05

7.5e-054.3e-058.2e-05

4.3e-058.9e-054.9e-05

4.6e-053.2e-055.6e-05

2.8e-050.0001772.8e-05

2.8e-055.8e-052.8e-05

0.00011

9e-06

0.000114

0.000114

1.4e-050.0001291.7e-05

6.8e-05

1.4e-056.1e-051.7e-05

3.5e-055.5e-054e-05

3.5e-055.5e-054e-05

2.1e-059.2e-051.9e-05

2.1e-059.2e-051.9e-05

9.2e-050.0001170.000102

2.8e-05

4.1e-053.7e-054.5e-05

5.1e-055.2e-055.7e-05

1.5e-056.8e-051.6e-05

1.5e-056.8e-051.6e-05

1.5e-056.8e-051.6e-05

0.0001

0.0001

0.0001

0.0041110.0025160.0042830.002610.0058720.0039470.001889

0.0041110.0025160.0042830.002610.0058720.0039470.001889

0.0041110.0025160.0042830.002610.0058720.0039470.001889

1.7e-056.9e-051.8e-05

1.7e-056.9e-051.8e-05

9.3e-05

9.3e-05

0.0016110.001752

0.0007750.000843

0.0008360.000909

0.0040940.0025160.0026720.002610.0038890.0039290.001889

0.0040940.0025160.0026720.002610.0038890.0039290.001889

6.9e-05

6.9e-05

4.5e-050.0006264.9e-05

4.5e-050.0006264.9e-05

4.5e-050.0006264.9e-05

1.6e-050.0001061.7e-05

1.6e-050.0001061.7e-05

8e-060.0002031e-05

4e-05

4.6e-05

4.7e-05

8e-067e-051e-05

1.1e-057.5e-051.1e-05

1.1e-057.5e-051.1e-05

1e-050.0001771.1e-05

1e-058.3e-051.1e-05

9.4e-05

6.5e-05

6.5e-05

2.6e-050.0001221.8e-05

2.6e-050.0001221.8e-05

2.6e-050.0001221.8e-05

1.9e-058.1e-051.3e-05

1.9e-058.1e-051.3e-05

7e-064.1e-055e-06

7e-064.1e-055e-06

1.9e-050.000221e-05

1.9e-050.000221e-05

1.9e-050.000221e-05

1.9e-050.000221e-05

5.4e-05

5.4e-05

4e-063.3e-051e-06

4e-063.3e-051e-06

8e-064.2e-055e-06

8e-064.2e-055e-06

3e-063.9e-051e-06

3e-063.9e-051e-06

4e-065.2e-053e-06

4e-065.2e-053e-06

1.5e-055.5e-051.2e-05

1.5e-055.5e-051.2e-05

1.5e-055.5e-051.2e-05

1.5e-055.5e-051.2e-05

1.5e-055.5e-051.2e-05

1.5e-055.5e-051.2e-05

0.0102960.0209920.0181740.0344370.0069760.0100280.013624

0.0102960.0209920.0181740.0344370.0069760.0100280.013624

1.8e-059.1e-052.3e-05

1.8e-059.1e-052.3e-05

4e-063.5e-059e-06

4e-063.5e-059e-06

3e-05

3e-05

1.4e-052.6e-051.4e-05

1.4e-052.6e-051.4e-05

3e-066.8e-053e-06

3e-066.8e-053e-06

2e-063.8e-051e-06

2e-061.9e-051e-06

1.9e-05

1e-063e-052e-06

1e-063e-052e-06

0.0102750.0209920.0181740.0344370.0068170.0100020.013624

0.0102560.0209920.0181740.0344370.0067350.0099870.013624

0.0076370.0159480.0128050.0304210.0057090.0074440.009974

0.0037880.0119240.0051890.0064264.7e-050.0036730.009008

0.0004170.000150.0003170.0090365e-060.000413

1.2e-05

7e-06

0.0009430.0011860.0006390.0048192.2e-050.0009270.000249

1.9e-05

0.0016110.0014280.0006610.0055228e-060.0015690.000207

0.0008780.001260.0008730.0046189e-060.0008620.00051

0.0051260.005571

9e-06

0.0026190.0050440.0053690.0040160.0010260.0025430.00365

8e-060.0033594.6e-059e-060.00365

2e-063e-053e-06

6e-068e-066e-06

0.0026030.0050440.002010.0040160.0009420.002525

1.9e-058.2e-051.5e-05

1.1e-054.4e-051e-05

4e-062.4e-053e-06

7e-062e-057e-06

8e-063.8e-055e-06

2e-068e-061e-06

8e-06

6e-062.2e-054e-06

2.1e-050.0001951.4e-05

1.2e-057.6e-051.2e-05

1.2e-057.6e-051.2e-05

1.2e-057.6e-051.2e-05

1.2e-057.6e-051.2e-05

1.2e-057.6e-051.2e-05

9e-064.1e-052e-06

9e-064.1e-052e-06

9e-064.1e-052e-06

9e-064.1e-052e-06

9e-064.1e-052e-06

7.8e-05

7.8e-05

7.8e-05

7.8e-05

7.8e-05

0.0018650.0010910.0012390.0019080.0016510.001790.000769

0.0018650.0010910.0012390.0019080.0016510.001790.000769

0.0018650.0010910.0012390.0019080.0016510.001790.000769

0.0018650.0010910.0012390.0019080.0016510.001790.000769

0.0018650.0010910.0012390.0019080.0016510.001790.000769

1e-063.3e-052e-06

0.0018640.0010910.0012390.0019080.0016180.0017880.000769

6.4e-05

6.4e-05

6.4e-05

6.4e-05

6.4e-05

6.4e-05

0.0001030.0042060.0004888.8e-050.004571

5.3e-050.0001984.4e-05

4.5e-050.0001293.8e-05

4.5e-050.0001293.8e-05

4.5e-050.0001293.8e-05

4.5e-050.0001293.8e-05

8e-066.9e-056e-06

8e-066.9e-056e-06

8e-066.9e-056e-06

8e-066.9e-056e-06

2e-056.1e-051.6e-05

1.7e-055.7e-051.2e-05

1.7e-055.7e-051.2e-05

1.7e-055.7e-051.2e-05

1.7e-055.7e-051.2e-05

3e-064e-064e-06

3e-064e-064e-06

3e-064e-064e-06

3e-064e-064e-06

0

1.4e-057.1e-051e-05

1.4e-057.1e-051e-05

1.2e-054.6e-058e-06

1.2e-054.6e-058e-06

7e-062.4e-055e-06

5e-062.2e-053e-06

2e-062.5e-052e-06

2e-062.5e-052e-06

2e-062.4e-052e-06

0

1e-06

9e-060.00011.3e-05

9e-060.00011.3e-05

9e-060.00011.3e-05

6.4e-05

6.4e-05

9e-063.6e-051.3e-05

9e-063.6e-051.3e-05

7e-065.8e-055e-06

7e-065.8e-055e-06

7e-065.8e-055e-06

7e-065.8e-055e-06

7e-065.8e-055e-06

0.0042060.004571

0.0042060.004571

0.0042060.004571

0.0042060.004571

0.0042060.004571

6.6e-050.0003263.5e-05

6.6e-050.0003263.5e-05

3.3e-056.4e-051.6e-05

3.3e-056.4e-051.6e-05

1e-062.2e-051e-06

1e-062.2e-051e-06

3.2e-054.2e-051.5e-05

3.2e-054.2e-051.5e-05

3.3e-050.0002621.9e-05

1.5e-050.0001279e-06

3e-065.1e-051e-06

3e-065.1e-051e-06

1e-066e-050

2.5e-05

1e-063.5e-050

1e-061.1e-051e-06

1e-061.1e-051e-06

1e-055e-067e-06

9e-065e-067e-06

1e-060

6.5e-05

6.5e-05

6.5e-05

1.8e-057e-051e-05

9e-062.8e-053e-06

9e-062.8e-053e-06

9e-064.2e-057e-06

4e-062.4e-053e-06

5e-061.8e-054e-06

3e-064.2e-052e-06

4.2e-05

4.2e-05

4.2e-05

4.2e-05

4.2e-05

3e-062e-06

3e-062e-06

3e-062e-06

3e-062e-06

3e-062e-06

0.0054440.0078820.0073260.010040.0037340.0103250.006266

0.0054440.0078820.0073260.010040.0037340.0103250.006266

0.0013330.0028840.0034350.005020.0012220.0012340.002547

0.000140.0006050.000125

3.6e-050.0001233e-05

2.1e-053.8e-051.3e-05

9e-065.7e-051.1e-05

6e-062.8e-056e-06

7.5e-050.0003547.2e-05

2e-063.1e-053e-06

3.6e-057.3e-053.6e-05

1.9e-05

3e-063.2e-054e-06

5e-064.2e-054e-06

5e-065.6e-054e-06

2e-051.8e-051.8e-05

5.1e-05

2e-06

1e-06

4e-062.9e-053e-06

4e-063.2e-054e-06

4e-063.2e-054e-06

1.1e-053.6e-058e-06

1.1e-053.6e-058e-06

1.4e-056e-051.1e-05

6e-062.8e-055e-06

8e-063.2e-056e-06

0.0011930.0028840.0034350.005020.0006170.0011090.002547

7.2e-051.9e-053.4e-05

1e-061e-060

3.7e-051e-05

7e-067e-06

6e-063e-065e-06

3e-06

2.1e-051e-062.2e-05

1e-06

0.0011210.0028840.0034350.005020.0005980.0010750.002547

2.7e-058e-062.4e-05

2.8e-050.002310.002512.4e-052.6e-05

1.6e-052e-061.6e-05

7e-068e-067e-06

1e-063e-061e-06

0.0010420.0028840.0011250.002510.0005530.0010010.002547

0.0001356.5e-050.0001091.6e-05

0.0001356.5e-050.0001091.6e-05

0.0001356.5e-050.0001091.6e-05

3.3e-053.4e-053.3e-058e-06

4e-061.1e-054e-068e-06

6.9e-0504.3e-05

2.9e-052e-052.9e-05

0.0039760.0049980.0038910.005020.0024470.0089820.003703

0.0039760.0049980.0038910.005020.0024470.0089820.003703

0.0039670.0049980.0038910.005020.0024010.0089740.003703

2e-063.1e-051e-06

0.0008540.000928

1.8e-05

0.0011630.0019720.0008720.0023090.0003240.001280.001508

0.0028020.0030260.0021650.0027110.00110.0076930.002195

9e-064.6e-058e-06

9e-064.6e-058e-06

3e-064.5e-051e-06

3e-064.5e-051e-06

3e-064.5e-051e-06

3e-064.5e-051e-06

3e-064.5e-051e-06

3e-064.5e-051e-06

3.7e-050.0002973.7e-05

3.7e-050.0002973.7e-05

3.7e-050.0002973.7e-05

3.7e-050.0002973.7e-05

1.2e-050.0001958e-06

7.5e-05

5.6e-05

1.2e-056.4e-058e-06

1e-054.6e-056e-06

7e-063.8e-054e-06

4e-06

3e-064e-062e-06

1.5e-055.6e-052.3e-05

1.5e-055.6e-052.3e-05

7.5e-050.0002716.3e-05

6.3e-050.0001735.5e-05

6.3e-050.0001735.5e-05

6.3e-050.0001735.5e-05

1.3e-053.4e-051e-05

1.3e-053.4e-051e-05

9e-063.4e-056e-06

9e-063.4e-056e-06

2.1e-055.3e-052e-05

1e-052.5e-051.2e-05

1.1e-052.8e-058e-06

2e-055.2e-051.9e-05

8e-062.5e-058e-06

1.2e-052.7e-051.1e-05

6e-066.2e-053e-06

6e-066.2e-053e-06

6e-066.2e-053e-06

6e-066.2e-053e-06

6e-066.2e-053e-06

6e-063.6e-055e-06

6e-063.6e-055e-06

6e-063.6e-055e-06

6e-063.6e-055e-06

6e-063.6e-055e-06

2e-063.6e-051e-06

2e-063.6e-051e-06

2e-063.6e-051e-06

2e-063.6e-051e-06

2e-063.6e-051e-06

2e-063.6e-051e-06

0.2873390.2375810.1808550.227510.2413640.2879820.209421

5.4e-059.5e-053.7e-05

5.4e-059.5e-053.7e-05

5.4e-059.5e-053.7e-05

2.5e-054.4e-051.9e-05

2.5e-054.4e-051.9e-05

2.9e-055.1e-051.8e-05

2.9e-055.1e-051.8e-05

0.0002923.4e-050.0003110.0005050.0002871.7e-05

0.0002923.4e-050.0003110.0005050.0002871.7e-05

0.0002923.4e-050.0003110.0005050.0002871.7e-05

0.0002923.4e-050.0003110.0005050.0002871.7e-05

0.0002923.4e-050.0003110.0005050.0002871.7e-05

0.2867470.2375470.1805440.227510.2400190.2874670.209404

5.1e-056.3e-054.4e-05

5.1e-056.3e-054.4e-05

5.1e-056.3e-054.4e-05

5.1e-056.3e-054.4e-05

0.0003480.0001070.0001630.0015060.0004470.0003440.000149

0.0003480.0001070.0001630.0015060.0004470.0003440.000149

0.0003480.0001070.0001630.0015060.0004470.0003440.000149

4.2e-055.6e-054.1e-05

2.4e-054.8e-052.4e-05

2.7e-056.6e-052.9e-054.5e-05

0.0002140.0001070.0001630.0015060.0002110.0002070.000104

4.1e-056.6e-054.3e-05

0.2090090.123520.117270.0764050.1799030.2005830.097396

0.2090090.123520.117270.0764050.1799030.2005830.097396

0.1653020.0988940.0934580.0598390.1458960.1585760.075539

4.3e-050.0001514.1e-05

4.7e-050.0001724.4e-05

4.4e-050.0003750.0004084.4e-05

0.1651680.0988940.0930830.0598390.1451650.1584470.075539

0.0435630.0246260.0238120.0165660.0337540.0418670.021775

0.0422680.024170.023060.0138550.0324910.0405910.021363

0.0012950.0004560.0007520.0027110.0012630.0012760.000412

4.8e-056.7e-055e-053.3e-05

1.6e-052.7e-051.8e-05

1.7e-05

3.2e-054e-053.2e-051.6e-05

9.6e-050.0001869e-054.9e-05

9.6e-050.0001869e-054.9e-05

0.001810.0007750.0008920.0017070.001840.0017550.000644

9.5e-050.00019.6e-05

9.5e-050.00019.6e-05

9.5e-050.00019.6e-05

0.0014780.0007220.0008920.0017070.0012640.0014180.000533

0.0014320.0007220.0008920.0017070.0012010.0013770.000533

4.3e-055.4e-054.1e-05

0.0013890.0007220.0008920.0017070.0011470.0013360.000533

4.6e-056.3e-054.1e-05

4.6e-056.3e-054.1e-05

0.0002375.3e-050.0004760.0002410.000111

0.0002375.3e-050.0004760.0002410.000111

0.0002375.3e-050.0004760.0002410.000111

4.4e-056.2e-054.1e-05

4.4e-056.2e-054.1e-05

4.4e-056.2e-054.1e-05

4.4e-056.2e-054.1e-05

0.0001720.0001760.0001716.2e-05

0.0001720.0001760.0001716.2e-05

5.6e-055.4e-055.8e-05

5.6e-055.4e-055.8e-05

5.6e-056.6e-055.6e-05

5.6e-056.6e-055.6e-05

6e-055.6e-055.7e-056.2e-05

6e-055.6e-055.7e-056.2e-05

0.0290330.0412280.0257520.0302220.0166370.0357980.041044

0.0031230.0020850.0012520.0022090.0022690.0030980.001506

0.0023760.0019050.0012520.0022090.0013420.0023460.00143

0.0001195.7e-052.9e-050.0001227e-06

0.0002859.9e-050.0002653e-050.000291

6.2e-05

3.6e-05

0.0002510.000273

2.6e-05

0.0014480.0015550.0007360.0022090.0002330.0013940.001405

2.1e-05

2.6e-05

0.0003610.0001290.0003640.000373

0.000167

1.1e-05

0.0001636.5e-056.4e-050.0001661.8e-05

0.0007470.000180.0009270.0007527.6e-05

0.000118

0.000223.5e-059.5e-050.0002195.7e-05

0.0001732.8e-050.0001680.0001731.9e-05

0.0003540.0001170.0005460.00036

0.0038590.0086030.015290.0084340.008250.0038620.016729

0.0038590.0086030.015290.0084340.008250.0038620.016729

0.0069790.007584

3e-05

0.0002380.000258

0.0001127.2e-050.0001111.5e-05

7.4e-057e-057e-05

8.6e-05

6.3e-056e-056.1e-05

0.0002240.0001350.000225

0.000126

0.000680.000738

5.7e-05

1e-059e-061.2e-05

6.5e-058.8e-056.9e-05

1.5e-05

0.0003380.0002070.0002350.0002070.000329

0.0002820.000306

0.0002490.00027

0.00013

0.0002820.000307

0.0001570.0003819.4e-050.0001634e-06

5.7e-054.5e-055.6e-05

0.0014970.001627

0.000119

7.5e-055e-057.8e-05

3.2e-05

0.000122

7.5e-05

6.8e-05

0.000146

0.0010880.000550.0007650.0008410.001129

0.000138

0.000104

8.1e-05

0.000144

0.0001620.0001540.000175

0.0001016e-055.5e-050.000101

1.1e-052.4e-052e-061.2e-05

2.9e-057e-050.0022170.002412e-053e-05

4.3e-05

0.0012930.0073110.0010640.0060240.0003130.0012410.009081

0.0008020.000872

0.000186

3.4e-05

3.4e-05

3.4e-05

0.0001190.0001160.000112

0.0001190.0001160.000112

0.0001190.0001160.000112

0.0215110.030540.0086790.0175710.0049470.0283220.022727

4.3e-050.0001413.6e-05

4.3e-050.0001413.6e-05

0.0214680.030540.0086790.0175710.0048060.0282860.022727

6.9e-050

6.2e-052.5e-056.6e-051.5e-05

3.9e-053.3e-054e-05

5e-067e-063e-06

0

4.7e-056e-064.6e-051e-06

4e-06

4.9e-053e-055.4e-05

4.4e-05

00

0008.3e-05

4e-05

00.000107005e-06

3.5e-05

4e-06

5e-066e-065e-06

0.0155850.0244170.0047260.0121490.001140.0195410.019031

5.6e-05

0.0030340.0018320.002220.0022090.002490.002916

2e-05

1.2e-05

0.00011305e-06

2.9e-051.7e-053.3e-05

2.6e-05

0.0024040.0039220.0017330.0032130.0005770.0054360.003486

3.1e-05

1.1e-050.0001086e-061.1e-055e-06

0

3e-062e-063e-06

4.2e-052.1e-054.2e-051.6e-05

3e-062e-063e-06

7e-061e-066e-06

3.5e-05

6.4e-054.9e-057.1e-051.5e-05

2e-064e-062e-06

4.4e-05

3.2e-05

5e-069e-066e-063e-05

3.2e-05

3e-064.1e-051e-062e-06

6e-056.4e-055.2e-05

6e-056.4e-055.2e-05

6e-056.4e-055.2e-05

0.0003610.0005310.0020080.0009570.0003528.2e-05

0.0003610.0005310.0020080.0009570.0003528.2e-05

1.9e-05

0.0005310.000577

0.0001160.0001510.0001153e-05

3.3e-05

0.0001270.0001340.000122

0.0001180.0020084.5e-050.000115

5e-05

0.0002810.000420.0002631.6e-05

0.0001190.0002730.000111.6e-05

6.1e-050.0001015.6e-05

6.1e-055.5e-055.6e-05

4.6e-05

5.8e-055.7e-055.4e-051.6e-05

5.8e-055.7e-055.4e-051.6e-05

5.6e-05

5.6e-05

5.9e-05

5.9e-05

0.0001620.0001470.000153

2.5e-053.8e-052.1e-05

2.5e-053.8e-052.1e-05

3.7e-055e-053e-05

3.7e-055e-053e-05

3.9e-051.8e-053.9e-05

3.9e-051.8e-053.9e-05

2.6e-051.3e-052.6e-05

2e-062e-062e-06

2.4e-051.1e-052.4e-05

3.5e-052.8e-053.7e-05

3.5e-051.9e-053.7e-05

9e-06

6.4e-057.4e-055.8e-055e-06

6.4e-057.4e-055.8e-055e-06

6.4e-057.4e-055.8e-055e-06

6.4e-057.4e-055.8e-055e-06

0.0125760.0510820.0139460.0956830.006670.0163770.052873

0.0125760.0510820.0139460.0956830.006670.0163770.052873

0.0017410.0017730.0015450.002410.0011960.0016780.001697

0.0017410.0017730.0015450.002410.0011960.0016780.001697

0.000490.0007610.000409

0.000490.0007610.000409

0.0108350.0493090.0119110.0932730.004640.0146990.050767

2.8e-054.6e-053e-05

3.2e-05

2.3e-05

2.7e-051.1e-052.5e-05

6e-05

0.0004610.0005520.0003880.0520080.0001790.000441

0.0100790.0485840.0115230.0412650.0041040.0139620.050407

2e-06

1.8e-05

2.6e-053.1e-053e-05

2e-063e-063e-06

1.1e-05

0.0001970.0001738.9e-050.0001930.00036

1.2e-05

4e-06

2e-06

1.5e-051.3e-051.5e-05

7.3e-05

7.3e-05

0.0055710.0029860.0045460.002610.0066310.0054230.002907

0.0055710.0029860.0045460.002610.0066310.0054230.002907

0.0055020.0029860.0045460.002610.0065690.0053570.002907

6e-05

0.0001012.4e-051.8e-050.000109

6.7e-052e-052.9e-057e-05

3.5e-05

3e-05

8.4e-052e-059e-069e-051e-06

7.3e-054.1e-057.9e-05

3.7e-05

2.2e-05

2.5e-05

7.5e-05

6.8e-053.5e-050

1.7e-05

0.0004550.0006840.000387

4.9e-05

1.8e-05

5e-061e-06

6.2e-056.7e-05

3.5e-05

5e-065e-066e-06

0.0008990.000977

0.0003610.000392

1.5e-05

5.8e-052.1e-052.1e-056.3e-05

3.8e-05

5e-06

1.8e-05

4.6e-05

6.3e-054e-057.2e-05

4.6e-05

2.5e-05

3.1e-05

6.7e-056.7e-057e-051e-05

5.7e-05

2.8e-05

5e-061e-055e-06

4e-05

3.4e-053e-052.2e-057.3e-051e-06

0.0001043.1e-052e-050.00011

4.5e-052.3e-053.2e-054.7e-05

2.6e-05

1.6e-05

3.1e-05

2.4e-05

0.0046020.0028170.0028310.002610.0036470.0044250.002021

1.9e-05

6.4e-052.4e-057.1e-05

4.4e-05

3e-06

2.8e-05

3.4e-05

6.9e-056.2e-056.6e-05

6.9e-056.2e-056.6e-05

1.6e-055.2e-051.5e-05

1.6e-055.2e-051.5e-05

1.6e-055.2e-051.5e-05

1.6e-055.2e-051.5e-05

4.4e-055.4e-054e-05

4.4e-055.4e-054e-05

4.4e-055.4e-054e-05

4.4e-055.4e-054e-05

0.0069760.0037250.0051790.0072280.0075620.0066510.003726

5.7e-058.6e-055.2e-05

3.5e-05

3.5e-05

5.7e-055.1e-055.2e-05

5.7e-055.1e-055.2e-05

0.0031690.001760.0020080.0021080.0028940.0030360.001503

0.0030850.001760.0020080.0021080.0028230.0029580.001473

8.8e-05

0.0030850.001760.0020080.0021080.0027350.0029580.001473

8.4e-057.1e-057.8e-053e-05

8.4e-057.1e-057.8e-053e-05

0.0004750.0003110.0002770.0016060.0008660.0004570.000215

3.3e-05

3.3e-05

3.4e-05

3.4e-05

4.6e-05

4.6e-05

4.2e-05

4.2e-05

5.3e-05

5.3e-05

4.6e-05

4.6e-05

8.7e-050.000358.6e-056e-06

8e-05

2.8e-05

4.7e-05

7.4e-05

8.7e-057.9e-058.6e-056e-06

4.2e-05

0.0003430.0003110.0002770.0016060.0001870.0003280.000209

0.0003430.0003110.0002770.0016060.0001870.0003280.000209

4.5e-052.3e-054.3e-05

4.5e-052.3e-054.3e-05

5.2e-05

1.2e-05

4e-05

1.3e-054.3e-059e-06

1.3e-054.3e-059e-06

1.3e-054.3e-059e-06

7.4e-056.2e-057.2e-051.5e-05

7.4e-056.2e-057.2e-051.5e-05

7.4e-056.2e-057.2e-051.5e-05

5.9e-057.1e-055.6e-05

5.9e-057.1e-055.6e-05

5.9e-057.1e-055.6e-05

5.4e-050.0001644.9e-053.3e-05

5.4e-05

5.4e-05

5.4e-055.7e-054.9e-053.3e-05

5.4e-055.7e-054.9e-053.3e-05

5.3e-05

5.3e-05

2e-061.2e-051e-06

2e-061.2e-051e-06

2e-061.2e-051e-06

6.9e-050.0001516.4e-056.8e-05

6.8e-05

6.8e-05

5.1e-05

5.1e-05

5.1e-05

5.1e-05

6.9e-054.9e-056.4e-05

6.9e-054.9e-056.4e-05

0.0002310.0001890.0002242e-05

0.0002310.0001890.0002242e-05

8.5e-057.1e-058.2e-05

7e-055.3e-057e-055e-06

7.6e-056.5e-057.2e-051.5e-05

0.0012390.0004450.000931

0.0012390.0004450.000931

0.0008290.0009

0.000410.0004453.1e-05

0.0027730.0016540.0016550.0035140.0025790.0026310.000941

6.6e-057.6e-055.7e-05

3.6e-052.4e-053e-05

2.7e-05

3e-052.5e-052.7e-05

0.0009390.0005580.0006570.0017070.000940.0008980.000405

3e-066e-063e-06

6e-06

2.2e-05

1.8e-05

6e-06

2.4e-05

5.7e-05

1.9e-05

2.9e-05

0.0009360.0005580.0006570.0017070.0007320.0008950.000405

2.1e-05

5.8e-050.0001584.9e-05

5.1e-05

5.8e-054.7e-054.9e-05

6e-05

5e-068e-065e-06

5e-068e-065e-06

7.3e-055.8e-057e-05

7.3e-055.8e-057e-05

0.001510.0010960.0009980.0018070.0011780.0014480.000536

0.001510.0010960.0009980.0018070.0011780.0014480.000536

5.8e-05

5.8e-05

3.7e-052.2e-053.1e-05

3.7e-052.2e-053.1e-05

8.5e-058.1e-057.3e-05

2.2e-052.3e-051.9e-05

6.3e-055.8e-055.4e-05

0.0001570.0003070.000151.6e-05

0.0001570.0003070.000151.6e-05

5.5e-05

5.5e-05

0.0001570.0002520.000151.6e-05

0.0001570.0002520.000151.6e-05

0.016830.0098230.0101280.007430.016280.0161330.008011

0.016830.0098230.0101280.007430.016280.0161330.008011

2.2e-054.4e-051.2e-05

2.2e-054.4e-051.2e-05

0.0168080.0098230.0101280.007430.0160320.0161210.008011

0.0168080.0098230.0101280.007430.0160320.0161210.008011

1.2e-05

1.2e-05

4.8e-05

4.8e-05

0.000144

4e-05

3.5e-05

3.6e-05

3.3e-05

0.0037650.0043010.0026680.0047190.0028410.0036210.002555

0.0037650.0043010.0026680.0047190.0028410.0036210.002555

6.1e-05

6.1e-05

7.3e-056.9e-056.9e-051.8e-05

7.3e-056.9e-056.9e-051.8e-05

0.0032910.0043010.0026680.0047190.0019870.0031650.002445

0.0014160.0032680.001690.0028110.0013250.0013610.002445

3.9e-05

0.0018750.0010330.0009780.0019080.000470.001804

0.000153

7.7e-056e-057.5e-05

7.7e-056e-057.5e-05

7.6e-05

7.6e-05

5.9e-05

5.9e-05

0.0001370.0002610.0001334.6e-05

0.0001370.0002610.0001334.6e-05

9.3e-059e-053.1e-05

9.3e-059e-053.1e-05

6.9e-05

6.9e-05

9.4e-050.0001998.9e-051.5e-05

9.4e-058.4e-058.9e-051.5e-05

3e-06

0

3e-05

1e-06

3.3e-05

4.8e-05

0.0001990.0006110.000155

9.8e-050.0003077.4e-05

8.1e-050.0001786.2e-05

5.4e-056.5e-053.8e-05

5.4e-056.5e-053.8e-05

2.7e-050.0001132.4e-05

2.7e-055.7e-052.4e-05

5.6e-05

1.7e-050.0001291.2e-05

1.7e-058e-051.2e-05

1.7e-058e-051.2e-05

4.9e-05

4.9e-05

0.0001010.0003048.1e-05

0.0001010.0003048.1e-05

1.6e-057.2e-051.6e-05

1.6e-057.2e-051.6e-05

2.5e-054.2e-052.4e-05

2.5e-054.2e-052.4e-05

1e-053.5e-054e-06

1e-053.5e-054e-06

1.6e-051.1e-05

1.6e-051.1e-05

5.1e-05

5.1e-05

3.4e-050.0001042.6e-05

1.3e-055.8e-051e-05

2.1e-054.6e-051.6e-05

4.7e-050.0001343.6e-05

4.7e-050.0001343.6e-05

4.7e-050.0001343.6e-05

4.7e-050.0001343.6e-05

4.7e-057.2e-053.6e-05

6.2e-05

1.5e-059.9e-051.4e-05

1.5e-059.9e-051.4e-05

1.5e-059.9e-051.4e-05

1.5e-059.9e-051.4e-05

1.5e-059.9e-051.4e-05

1.5e-059.9e-051.4e-05

2.7e-050.0001643.4e-05

2.7e-050.0001643.4e-05

2.7e-050.0001643.4e-05

2.7e-050.0001643.4e-05

4e-063.8e-053e-06

4e-063.8e-053e-06

7e-063.9e-058e-06

7e-063.9e-058e-06

1.2e-054.2e-052.1e-05

1.2e-054.2e-052.1e-05

4e-064.5e-052e-06

4e-064.5e-052e-06

0.002150.0021310.00251

0.002150.0021310.00251

0.002150.0021310.00251

0.002150.0021310.00251

0.002150.0021310.00251

0.002150.0021310.00251

0.0021420.0116610.0034330.052610.0022680.002010.067467

0.0021420.0116610.0034330.052610.0022680.002010.067467

2e-050.0001241.6e-05

1.1e-054.8e-051e-05

1e-062.6e-050

1e-062.6e-050

1e-052.2e-051e-05

2.2e-05

1e-051e-05

3e-064.1e-051e-06

3e-064.1e-051e-06

3e-064.1e-051e-06

6e-063.5e-055e-06

6e-063.5e-055e-06

6e-063.5e-055e-06

0.0021220.0116610.0034330.052610.0021440.0019940.067467

0.0021220.0116610.0034330.052610.0021440.0019940.067467

0.0021220.0116610.0034330.052610.0021440.0019940.067467

1.8e-059e-061.9e-05

0.0001770.0009050.0002340.0020080.0001130.0001510.001144

0.0019210.0107560.0031990.0506020.0019180.0017980.066323

04e-060

2e-061e-052.2e-05

1e-063e-061e-06

2.7e-05

01.2e-050

2.4e-05

3e-062.4e-053e-06

0.0069860.0040920.0453810.0047190.0531890.0071960.002835

3.6e-050.000143.5e-05

3.6e-050.000143.5e-05

3.6e-050.000143.5e-05

3.6e-050.000143.5e-05

1.7e-056.6e-051.6e-05

1.9e-057.4e-051.9e-05

0.006950.0040920.0453810.0047190.0530490.0071610.002835

0.0021030.003550.0014660.0047190.0025540.0020420.00282

5.3e-055.2e-05

5.3e-055.2e-05

5.3e-055.2e-05

1.7e-057.4e-051.4e-05

1.7e-057.4e-051.4e-05

1.7e-057.4e-051.4e-05

2e-050.000311.7e-05

2e-050.000311.7e-05

2e-055e-051.7e-05

0.000111

0.000149

0.000950.0031990.0011420.0030120.0006330.0009110.002717

0.000950.0031990.0011420.0030120.0006330.0009110.002717

0.000950.0031990.0011420.0030120.0005980.0009110.002717

3.5e-05

0.0007610.0003510.0003240.0017070.0007840.0007450.000103

0.0007610.0003510.0003240.0017070.0007840.0007450.000103

0.0007610.0003510.0003240.0017070.0007840.0007450.000103

0.0002950.0007380.000296

5e-058.6e-054.9e-05

3.6e-058.6e-053.4e-05

1.4e-051.5e-05

0.0001870.0005240.000201

2e-06

2.1e-05

2.6e-057e-062.7e-05

1.4e-056.7e-051.1e-05

1e-05

2e-05

2e-0502.3e-05

1.4e-051.8e-051.2e-05

1.7e-05

1.9e-058.3e-052.1e-05

4e-06

2.6e-050.0001083.1e-05

5e-061e-054e-06

3e-06

4e-06

0

4e-063.4e-056e-06

2e-058e-052.3e-05

7e-06

7e-061.1e-059e-06

1.9e-051e-062.1e-05

1.3e-051.7e-051.3e-05

2.8e-057.7e-052.9e-05

2.8e-057.7e-052.9e-05

3e-055.1e-051.7e-05

3e-055.1e-051.7e-05

7e-061.5e-057e-06

7e-061.5e-057e-06

2e-06

7e-069e-067e-06

4e-06

0.0002150.000310.000225

0.00010.0001690.000107

0.00010.0001690.000107

0.00010.0001690.000107

0.0001150.0001410.000118

0.0001150.0001410.000118

0.0001150.0001410.000118

0.0037510.0005420.0439150.0475670.0039561.5e-05

0.0420030.045644

0.0420030.045644

0.0420030.045644

0.0028630.0005040.0016240.0012140.003011.5e-05

0.0006970.0001560.0002480.0003330.000619

8.7e-05

0.0002286e-050.000254

0.0001379.2e-050

0.0003326.4e-050.0002480.0001860.000365

0.0003593.9e-050.000350.000404

0.0003593.9e-050.000350.000404

0.0002066.4e-050.000228

0.0002066.4e-050.000228

0.0016010.0003090.0010260.0008170.0017591.5e-05

0.000146

0.0004549.6e-050.0003040.0002050.000501

0.0004178.7e-050.0002480.0001530.000457

0.0003296.4e-050.0002110.0001350.000377

0.0004016.2e-050.0002630.0001780.0004241.5e-05

0.0008883.8e-050.0002880.0007090.000946

0.000653.8e-050.0002880.0005560.000697

0.000194

0.0002150.0001680.000227

0.0004353.8e-050.0002880.0001940.00047

0.0002380.0001530.000249

0.0002380.0001530.000249

0.0004860.0012560.000529

9.3e-050.0002469.2e-05

9.3e-050.0002469.2e-05

3.7e-050.0001033.7e-05

5.6e-050.0001435.5e-05

0.0001120.0002480.000118

5.8e-050.0001936e-05

5.8e-050.0001936e-05

3.1e-053.3e-05

3.1e-053.3e-05

2.3e-055.5e-052.5e-05

2.3e-055.5e-052.5e-05

0.0001350.0003650.000156

0.0001350.0003650.000156

4.5e-056.1e-054.8e-05

2.9e-058.9e-053.6e-05

8e-065e-061e-05

4e-061.2e-055e-06

1.8e-050.0001182.3e-05

3.1e-058e-053.4e-05

2.3e-050.0001482e-05

2.3e-050.0001482e-05

2.3e-050.0001482e-05

0.0001230.0002490.000143

0.0001230.0002490.000143

0.0001230.0002490.000143

0.0001480.0004760.000156

0.0001480.0004760.000156

0.0001480.0004760.000156

0.0001480.0004760.000156

3.4e-05

3.4e-05

3.4e-05

3.4e-05

0.0002470.0008520.000253

4.3e-052.6e-054.6e-05

4.3e-052.6e-054.6e-05

4.3e-051.5e-054.6e-05

1.1e-05

2.3e-055.1e-052.6e-05

2.3e-055.1e-052.6e-05

2.3e-055.1e-052.6e-05

0.0001170.0006580.000119

0.0001170.0005720.000119

0.0001170.0005720.000119

8.6e-05

4.3e-05

4.3e-05

6.4e-050.0001176.2e-05

3.5e-054.6e-052.8e-05

3.5e-054.6e-052.8e-05

2.9e-057.1e-053.4e-05

2.9e-057.1e-053.4e-05

8.1e-050.0007037.1e-05

3.1e-05

3.1e-05

3.1e-05

3.1e-05

3.1e-05

9.3e-05

9.3e-05

9.3e-05

9.3e-05

9.3e-05

3.1e-053.8e-052.4e-05

3.1e-053.8e-052.4e-05

3.1e-053.8e-052.4e-05

3.1e-053.8e-052.4e-05

3.1e-053.8e-052.4e-05

2e-050.0001951.7e-05

7e-065.3e-056e-06

7e-065.3e-056e-06

7e-065.3e-056e-06

7e-065.3e-056e-06

1.3e-050.0001421.1e-05

1.3e-050.0001421.1e-05

3.6e-05

3.6e-05

1.3e-055.5e-051.1e-05

1.3e-055.5e-051.1e-05

5.1e-05

5.1e-05

3e-050.0003463e-05

3e-050.0003463e-05

3e-050.0003463e-05

3e-050.0003463e-05

3e-050.0001693e-05

0.000177

0.0033640.0020870.002540.0023090.0035550.003229

0.0033640.0020870.002540.0023090.0035550.003229

0.0033640.0020870.002540.0023090.0035550.003229

4e-060.0001273e-06

4e-060.0001273e-06

4e-060.0001273e-06

0.003360.0020870.002540.0023090.0034280.003226

0.003360.0020870.002540.0023090.0034280.003226

0.003360.0020870.002540.0023090.0034280.003226

2.2e-050.0001441.2e-05

2.2e-050.0001441.2e-05

2.2e-050.0001441.2e-05

2.2e-050.0001441.2e-05

9e-063.5e-057e-06

9e-063.5e-057e-06

1.3e-057.3e-055e-06

3.2e-05

1.3e-054.1e-055e-06

3.6e-05

3.6e-05

0.4065460.4133270.4451730.3542150000000010.4354010.4233410.374129

0.1256250.1552380.1863440.1791150.1844710.1536780.185449

0.0007460.002550.0005970.0033130.0006380.0010010.003188

0.0004670.002550.0005970.0033130.000270.0007980.003188

0.0004670.002550.0005970.0033130.0002470.0007980.003188

2e-061e-066e-06

7e-068e-066e-06

8e-064e-061.1e-05

0.000450.002550.0005970.0033130.0002110.0007750.003188

6e-06

1.7e-05

2.3e-05

2.3e-05

0.0002790.0002810.000203

0.0001760.0001540.000124

0.0001369.5e-050.000124

4e-055.9e-05

5e-054.7e-055.4e-05

5e-054.7e-055.4e-05

5e-06

5e-06

1.5e-052.9e-051e-05

1.5e-051.1e-051e-05

01.8e-050

3.8e-054.6e-051.5e-05

2.7e-05

3.8e-051.9e-051.5e-05

8.7e-05

4.9e-05

4.9e-05

3.8e-05

3.8e-05

0.016570.0176870.016060.0163650.0141070.0163670.009575

1.7e-05

1.7e-05

1.7e-05

0.016570.0176870.016060.0163650.0141070.0163670.009558

0.0007240.0007871.5e-05

0.0007240.0007871.5e-05

9.4e-058.7e-059.1e-05

3.7e-054e-054.1e-05

5.7e-052.5e-055e-05

2.2e-05

0.0164420.0176870.0153360.0163650.0131290.0162450.009537

6e-06

0.0022970.0100610.0021440.0058230.0007260.0026030.009283

4.1e-052.2e-05

8e-06

3.8e-051.5e-050.0021250.0023091.3e-053.1e-052.6e-05

7e-05

1.8e-057e-061.8e-052.1e-05

3.5e-05

4e-06

4.8e-05

3.4e-058e-06

2.9e-051.1e-052.7e-05

7.1e-058.7e-056.9e-05

0.0138170.0076020.0082950.0052210.011920.013275

2.2e-05

1.9e-059e-060.0027720.0030124e-061.6e-05

2.5e-056e-063.3e-05

1e-05

3.9e-05

1.6e-05

3.5e-053e-062.2e-05

3.2e-052.2e-058.5e-054.5e-05

2.3e-05

1.9e-056e-06

6.1e-052.8e-056.6e-054.7e-05

6e-06

3.4e-050.0001043.1e-056e-06

6e-06

5.7e-05

3.4e-054.7e-053.1e-05

2e-057.7e-051.5e-051.6e-05

2e-057.7e-051.5e-051.6e-05

2e-057.7e-051.5e-05

2e-057.7e-051.5e-05

1.6e-05

1.6e-05

0.0008130.003380.0005370.0033130.0003310.0007220.003878

0.000810.003380.0005370.0033130.000270.0007210.003878

0.000810.003380.0005370.0033130.0002380.0007210.003878

2.1e-05

1.1e-052.2e-051.2e-05

0.0007990.003380.0005370.0033130.0001480.0007090.003878

2.9e-05

1.8e-05

3.2e-05

3.2e-05

3e-066.1e-051e-06

3e-064.4e-051e-06

3.4e-05

3e-061e-051e-06

1.7e-05

1.7e-05

0.000116

0.000116

4.7e-05

4.7e-05

6.9e-05

6.9e-05

6.7e-05

6.7e-05

6.7e-05

6.7e-05

0.0403940.0355420.0661920.0682740.0570340.0527590.040516

0.0016274.6e-050.0005470.0008450.0014120.000662

0.0007824.6e-050.0003810.0006720.000106

0.0003143.3e-050.0001060.0002921.8e-05

8.5e-051.3e-052.5e-057.1e-058.8e-05

4.3e-05

5.2e-05

0.0002461.4e-050.0002

2.2e-05

0.0001373.1e-050.000109

8.8e-05

2.2e-054.7e-052.4e-05

2.2e-054.7e-052.4e-05

0.0007990.0005470.0003870.0006920.000556

3e-056e-063.3e-05

0.0002425.3e-050.0001880.000171

0.0002130.000232

5.2e-056e-065.9e-05

0.0003860.0003349e-050.0003240.000385

8.9e-0508.8e-05

2.4e-053e-052.4e-05

2.4e-053e-052.4e-05

0.000174

0.000174

0.000174

0.0002020.0002660.0001793.1e-05

0.000108

0.000108

0.0002026.8e-050.0001793.1e-05

1e-056e-068e-06

1.4e-052.2e-051.2e-05

2e-06

5e-06

0.0001783.3e-050.0001593.1e-05

9e-05

9e-05

0.0013660.0005710.0012217e-05

0.0001099e-069.6e-05

0.0001099e-069.6e-05

0.000112

0.000112

0.0002276.8e-050.000185

0.0002276.8e-050.000185

0.0004060.0002050.0003826.2e-05

0.0002223.9e-050.0002056.2e-05

0.0001845.1e-050.000177

2.5e-05

4.9e-05

4.1e-05

0.0004190.0001490.0003738e-06

0.0001595.1e-050.0001528e-06

4.1e-05

0.000265.7e-050.000221

0.0002052.8e-050.000185

0.0002052.8e-050.000185

0.0242080.0217380.0509950.0434740.0490770.0319120.021396

0.0015720.0002660.001520.0008130.0015210.000336

05.8e-05

9e-06

4.2e-05

0.0005425e-050.000485.9e-050.000502

0.0001098.2e-053.2e-050.0001210.000115

0.0004956.2e-050.0002410.0001320.000533

0.000105

0.0004370.000475

0.0004263e-050.0003624.1e-050.000365

1.9e-05

3.5e-05

5.8e-05

1.1e-05

0.0223590.0213190.0483760.0434740.0470170.0301220.020957

7e-06

0.001140.001239

0.0013250.00144

0.001220.0007490.0005890.0055220.0001650.0012410.006004

8.1e-05

0.0092320.010033

0.009030.009812

6.6e-050.0045830.0009090.0165661.5e-056.5e-050.000362

0.0159750.017361

0.0052470.005702

0.0210730.0159870.0049290.0213860.0011620.0288160.014591

0.0002770.0001535.3e-050.0002690.000103

3.4e-058.1e-054.2e-054e-05

0.0002437.2e-051.1e-050.0002290.000103

0.0010990.001194

0.0010990.001194

0.0102090.0123450.0118850.0219890.0044290.0150220.01729

4.7e-050.0002225.2e-05

5.4e-05

5.9e-05

1e-052e-069e-06

2.1e-055e-052.6e-05

6e-063e-067e-06

1e-055.4e-051e-05

7.7e-05

5.9e-05

1.8e-05

6.1e-05

6.1e-05

7.6e-05

7.6e-05

4.3e-054.2e-055.4e-05

4.3e-054.2e-055.4e-05

0.0006630.0002940.0002150.0005974e-05

0.0002142.9e-050.0001842.3e-05

5.3e-056.1e-052.3e-05

1.7e-05

0.0003080.0002946e-050.000331

8.8e-056.5e-055.9e-05

8.2e-05

8.2e-05

0.0002780.0006350.0004090.002410.0003020.0053480.002633

0.0001441.1e-050.0001313.7e-05

0.0001340.0006350.0004090.002410.0002910.0052170.002596

7.6e-05

7.6e-05

5.9e-05

5.9e-05

0.000101

0.000101

0.0010460.0034470.0012920.0029120.0001570.0010260.003652

2.8e-052e-062.5e-05

2e-06

0.0001248e-068.7e-051.8e-05

1e-06

0.0007420.0034310.0012920.0029122.9e-050.0007680.003617

0.000103

0.0001521.6e-051.2e-050.0001461.7e-05

9.8e-05

9.8e-05

0.0001711.9e-059.2e-050.0001816.6e-05

1.3e-05

9.8e-051.9e-056e-060.00015.4e-05

7.3e-052.5e-058.1e-051.2e-05

2.2e-05

4e-06

0

2.2e-05

4.1e-054.3e-057.7e-05

4.1e-054.3e-057.7e-05

4.5e-05

4.5e-05

0.0001

0.0001

0.0017170.0052930.0047970.0105420.0002930.0016670.005379

0.0013150.0048240.0006630.0046180.0002010.001297

6.1e-054.6e-050.0015710.0017077e-066.2e-05

7.7e-050.0021330.0019084.5e-053.9e-050.002952

0.0002640.0004230.000430.0023094e-050.0002690.002427

0.0003460.0002160.0001420.000352

0.0003460.0002160.0001420.000352

0.0002162.6e-050.000231

0.0002162.6e-050.000231

2.9e-05

2.9e-05

0.0026010.0013970.0017210.0019080.0013290.0025410.001232

4.4e-05

0.002260.0013970.00140.0019080.0011560.0021830.001066

4.3e-05

0.0003410.0003214.5e-050.000358

0.000122

3.5e-05

5e-05

0.0026970.0007540.0014480.0017070.0006270.0025658.6e-05

0.0003580.0003185.6e-050.0003341.6e-05

1e-06

0.0002471.6e-050.0002482.4e-05

4.7e-05

1.4e-05

0.0003240.0002991.3e-050.000326

9e-06

7e-06

1e-06

0.0015640.0007340.0008310.0017070.0004390.001481

0.0002042e-057e-060.0001764.6e-05

2e-06

3e-06

5e-06

7e-06

0.0002870.00080.0017080.002516.9e-050.0002780.004202

0.0002064e-050.0002

8.1e-050.00080.001440.002519e-067.8e-050.003711

2e-050.0002

0.0002680.000291

6.6e-05

6.6e-05

5.6e-055.3e-05

5.6e-055.3e-05

8.6e-05

8.6e-05

8.6e-05

0.0027820.0014130.0027650.0028110.0015860.0030130.001067

0.0020890.0013990.0025050.0028110.0013230.0024320.000856

0.0007180.0011740.000584

0.0006256.1e-050.0007049.9e-050.0009855.9e-05

0.0007940.0001440.0002591.9e-050.0007810.000213

0.0003490.0011940.0005290.0028116e-060.000346

0.0003210.0002952.5e-050.00032

0.0001430.0001330.000106

0.0001433.7e-050.000106

3.4e-05

6.2e-05

0.000551.4e-050.000260.000130.0004750.000211

0.0003090.000268.4e-050.00026

2.4e-05

0.0002411.4e-052.2e-050.0002150.000211

0.0016240.0009280.001260.0018070.0003620.0014820.00179

0.0016240.0009280.0003560.0018070.0003620.0014820.000808

0.0001096.3e-057.9e-054e-06

0.0001096.3e-057.9e-054e-06

0.0001548e-059.9e-053.5e-05

0.0001548e-059.9e-053.5e-05

0.0013610.0009280.0003560.0018070.0002190.0013040.000769

0.000146

0.0002013.8e-051.4e-050.000199.9e-05

6.6e-05

0.0010240.000890.0003560.0018070.0001060.0009890.000416

1e-05

0.0001362.3e-050.0001250.000108

0.0009040.000982

0.0009040.000982

0.0009040.000982

0.0132820.0313630.0532480.0260040.0697820.0120430.077275

0.003190.0055920.001430.0084340.0017670.002910.008875

1.5e-05

1.5e-05

6e-051.6e-057.5e-055.1e-05

5e-05

6e-051.6e-052.5e-055.1e-05

0.003130.0055760.001430.0084340.0016770.0028590.008875

0.000132

4.1e-05

5e-06

6e-06

1.6e-05

1.3e-05

0.00013.5e-051.6e-059.7e-050.000109

5e-06

0.0009210.004950.0006980.005020.0001940.0008840.007881

0.0001514.3e-050.000132

8.4e-051.5e-052.2e-057.6e-05

3.7e-05

1.7e-05

4.8e-05

4e-06

3.4e-05

7.2e-05

1.4e-05

6.1e-05

0.0001032e-059.3e-05

5.2e-05

2.3e-05

0.000118

7.7e-059.9e-050.0003060.0017071.6e-056.8e-050.000184

0.0001312.4e-050.000127

8e-06

9.7e-051.9e-050.000101

0.0005720.0003660.0004260.0017070.000230.0005480.000574

3.3e-05

4e-06

7e-06

1.1e-051e-051.2e-05

3e-06

5e-06

2.4e-05

9e-06

3e-05

1.6e-05

2.1e-05

0.0001512.3e-050.0001471.8e-05

0.000134.2e-050.000122

0.0001550.0001112.5e-050.0001545.2e-05

2.8e-05

5.4e-051e-054.8e-05

2e-05

3.1e-05

5.6e-051.3e-055.4e-05

8e-06

5.6e-056e-065.6e-054.2e-05

6e-06

4.8e-05

5.3e-05

5.6e-059e-065.4e-058e-06

1.4e-05

9.3e-052e-068.6e-05

2.6e-05

0.0100920.0257710.0518180.017570.0680150.0091330.0684

0.0098450.0253420.0518180.017570.0676820.0088810.0684

0.000590.000641

8.3e-05

0.0005250.000571

0.0010820.001176

0.0010980.0004180.0002060.0011867e-05

0.0003330.000362

3.8e-05

7.9e-05

0.0053790.023550.0032470.015060.000880.0042190.031115

0.0008440.000917

0.0019930.0008480.0009290.0005570.002066

5.7e-05

0.0018770.00204

0.040610.0618830.034294

0.0008010.000871

0.0013750.0009440.0005620.002510.0001890.001415.3e-05

0.0001470.0002350.0001790.000157

6.9e-050.0001173.7e-057.4e-05

2.5e-055.8e-057e-062.4e-05

2.5e-05

3.1e-05

1e-05

3.4e-051.7e-053.9e-05

1.9e-056e-058e-062e-05

4.4e-05

0.00010.0001940.0001549.5e-05

0.00010.0001942.1e-059.5e-05

6.4e-05

6.9e-05

8.6e-05

8.6e-05

8.6e-05

8.6e-05

0.0089350.005940.006080.0086340.0081170.0084480.004504

3.4e-052.6e-051.8e-05

3.4e-052.6e-051.8e-05

3.4e-052.6e-051.8e-05

0.004670.0027460.0027390.002610.0036680.0044540.001982

0.004670.0027460.0027390.002610.0036680.0044540.001982

5.8e-057e-064.7e-05

9e-064e-069e-06

2.1e-059e-061.7e-05

3.4e-051.5e-053.3e-05

2.7e-052.4e-052.1e-05

8e-061.7e-051.2e-05

5e-062e-065e-06

4.5e-052.9e-054e-05

0.0042840.0027460.0027390.002610.0034870.0041040.001982

9e-064e-068e-06

2.8e-051.4e-053.1e-05

1.5e-055e-061.3e-05

3e-052.2e-052.8e-05

2.2e-051.6e-051.9e-05

5.3e-054.7e-05

9e-062e-067e-06

1.3e-051.1e-051.3e-05

1.5e-054.1e-051.5e-05

1.5e-054.1e-051.5e-05

1.5e-052.2e-051.5e-05

1.9e-05

5e-053.8e-054e-05

5e-053.8e-054e-05

5e-053.8e-054e-05

0.0038540.0031940.0033410.0060240.0035130.003620.002507

4.5e-05

4.5e-05

0.0003740.0007880.0009340.0020080.0002140.0002950.000843

0

1.5e-05

1e-06

0.0003230.0001660.000241

3.1e-051.4e-053.6e-05

8e-06

2e-050.0007880.0009340.0020081e-051.8e-050.000843

3.3e-057.6e-053e-05

3.3e-057.6e-053e-05

4.7e-05

4.7e-05

0.0012080.0010790.0009870.0020080.001010.0011450.000857

1.8e-05

4.4e-051.7e-053.5e-05

1.4e-05

4.2e-051.5e-053.1e-05

2.3e-05

6.4e-05

0.0011220.0010790.0009870.0020080.0008440.0010790.000857

1.5e-05

0.0022390.0013270.001420.0020080.0021210.002150.000807

1.5e-054.6e-058e-06

0.0022240.0013270.001420.0020080.0020750.0021420.000807

7e-057.6e-056.6e-05

7e-057.6e-056.6e-05

4.1e-053.9e-054.2e-05

2.9e-053.7e-052.4e-05

3.3e-05

3.3e-05

3.3e-05

0.0002420.0007220.0002351.5e-05

0.0002420.0007220.0002351.5e-05

7e-06

0.0001580.0006060.00016

7e-06

3e-06

2.7e-05

1.8e-05

5.1e-053e-064.9e-05

3.3e-054e-062.6e-051.5e-05

2.3e-05

2.4e-05

0.0150340.005580.0106180.005020.0194430.0145250.003174

0.0146860.005580.0106180.005020.0189090.0142190.003143

8.7e-05

8.7e-05

0.0001490.0001790.000114

7.3e-059.5e-055.4e-05

7.6e-058.4e-056e-05

0.000150.0002130.0001541.6e-05

0.000150.0002130.0001541.6e-05

0.000181

0.000181

0.0001280.0001480.000117

0.0001280.0001480.000117

0.0142590.005580.0106180.005020.0181010.0138340.003127

0.0135090.005580.0059560.005020.0127180.0134210.003111

0.0044040.004786

0.0003510.000196

0.0003990.0002580.0001740.0004131.6e-05

0.000227

5.7e-05

5.7e-05

5.7e-05

5.5e-05

5.5e-05

5.5e-05

0.0002880.0002980.0002553.1e-05

8e-055.4e-057.1e-05

8e-055.4e-057.1e-05

3.9e-057.6e-053.3e-05

1.7e-051.7e-051.4e-05

9e-061.8e-056e-06

1.3e-054.1e-051.3e-05

6e-05

6e-05

0.0001085.7e-050.0001013.1e-05

0.0001085.7e-050.0001013.1e-05

6.1e-055.1e-055e-05

6.1e-055.1e-055e-05

6e-050.0001245.1e-05

6e-050.0001245.1e-05

6e-056.1e-055.1e-05

6.3e-05

0.0001030.0004550.0003319.6e-050.000494

9.2e-05

4.4e-05

4.4e-05

4.8e-05

4.8e-05

0.0001030.000179.6e-05

2.6e-053.5e-052.8e-05

2.6e-053.5e-052.8e-05

4e-055.7e-053.9e-05

4e-055.7e-053.9e-05

2.2e-054.5e-051.9e-05

2.2e-054.5e-051.9e-05

1.5e-053.3e-051e-05

1.5e-053.3e-051e-05

6.9e-05

6.9e-05

4e-05

2.9e-05

0.0004550.000494

0.0004550.000494

0.0004550.000494

0.0001550.0004030.000131

0.0001550.0004030.000131

0.0001218.8e-059.3e-05

0.0001218.8e-059.3e-05

1.3e-050.0001881.7e-05

5.1e-05

6.5e-05

1.3e-057.2e-051.7e-05

2.1e-050.0001272.1e-05

2.1e-050.0001272.1e-05

0.0195790.0413990.0203360.0322290.0079740.0397360.028394

0.0002330.0002660.000179

7.4e-05

7.4e-05

0.0001

2.5e-05

4.1e-05

3.4e-05

0.0001614.3e-050.000126

0.0001614.3e-050.000126

7.2e-054.9e-055.3e-05

7.2e-054.9e-055.3e-05

0.0193460.0413990.0203360.0322290.0077080.0395570.028394

6.6e-05

2.7e-05

2.3e-05

1.6e-05

0.0002114.9e-050.0001040.0001570.000145

4.1e-05

1e-06

1e-06

0.0002114.9e-051.7e-050.0001579.7e-05

4.4e-05

4.8e-05

0.0006980.0003220.0001080.0005219.4e-05

0.0002966.2e-050.000211.8e-05

0.0004020.0003224.6e-050.0003117.6e-05

0.0184320.041350.0200140.0322290.0074190.0388740.028155

0.0005710.0001650.0001230.000602

6.1e-05

8e-05

0.0011560.0041620.0007550.0028110.0002570.0012220.000876

0.0007840.001190.0008960.0019080.0004440.001770.001409

5e-060.0033260.0036142e-066e-06

0.0124210.0338030.0100410.0156630.0033070.0316030.023865

0.0023370.002539

0.001050.0003240.0004660.002510.00020.0010930.000672

3.4e-050.0010960.0013170.0023098e-064e-050.001333

0.0024110.000610.0008760.0034140.0003980.002538

5e-061.1e-055e-06

5e-061.1e-055e-06

0.0072450.0108290.0100730.0141560.0026960.005450.01259

0.0072450.0108290.0100730.0141560.0026960.005450.01259

0.0036170.0034750.0017280.0028110.0008170.0022260.003104

0.0002540.0001090.000283

0.0033630.0034750.0017280.0028110.0007080.0019430.003062

4.2e-05

2.3e-053.3e-050.0001552.2e-05

4.6e-05

9.1e-05

2.3e-053.3e-051.8e-052.2e-05

0.0001188e-050.0015069.3e-050.0001197.9e-05

0.0001188e-050.0015069.3e-050.0001197.9e-05

0.0003615.7e-050.0009190.0003060.0003790.001014

7.9e-050.0001118.1e-05

0.0009190.000999

0.000216.3e-050.000225

7.4e-05

7.2e-055.7e-055.8e-057.3e-051.5e-05

7.8e-050.0001186.2e-05

7.8e-050.0001186.2e-05

7.4e-050.0001296.1e-05

7.4e-050.0001296.1e-05

9.7e-056.2e-059.3e-05

9.7e-056.2e-059.3e-05

2.4e-053.7e-053.3e-051.6e-05

2.4e-053.7e-053.3e-051.6e-05

0.0028530.0071840.0074260.0098390.0009790.0024550.008377

0.0002690.000292

0.0006270.0002280.0004720.0031120.0004870.0006550.000555

0.000118

0.0001272.2e-050.000127

0.0003560.0001050.0019080.0001110.0001661.7e-05

0.0017430.0068510.0010720.0048190.0003590.0015070.001295

0.0056130.0061

0.000230.0005810.000183

0.000230.0005810.000183

0.000104

4e-05

6.4e-05

5.8e-052.5e-055.4e-05

5.8e-052.5e-055.4e-05

5.2e-05

5.2e-05

7.3e-05

7.3e-05

5.9e-05

5.9e-05

0.0001720.0002680.000129

0.0001458.3e-059e-05

3.9e-05

1.3e-055.2e-051e-05

1.4e-052.9e-052.9e-05

6.5e-05

0.0008954e-050.0008880.0023260.000725.5e-05

7.1e-059.4e-055.4e-05

7.1e-059.4e-055.4e-05

7.1e-053.6e-055.4e-05

2.7e-05

3.1e-05

4e-054.7e-053.2e-05

4e-054.7e-053.2e-05

4e-054.7e-053.2e-05

9.8e-050.0001278.6e-05

9.8e-050.0001278.6e-05

5.4e-05

4e-06

2.5e-052e-062.5e-05

7.3e-056.7e-056.1e-05

4.1e-05

4.1e-05

4.1e-05

0.0005264e-050.0008880.0017850.0004283.1e-05

0.0002594e-050.0008880.0015070.000243

0.000116

0.000122

0.000117

0.0002594e-050.0001870.000243

0.0008880.000965

5.2e-05

5.2e-05

5e-051.3e-054.2e-05

5e-051.3e-054.2e-05

6.7e-051.7e-053e-06

6.7e-051.7e-053e-06

0.000150.0001070.000143.1e-05

0.000150.0001070.000143.1e-05

8.9e-05

8.9e-05

0.000160.0002320.000122.4e-05

4.7e-05

4.7e-05

5.8e-05

5.8e-05

6e-053.3e-054.2e-05

6e-053.3e-054.2e-05

0.00019.4e-057.8e-052.4e-05

3.4e-053.7e-053e-051.6e-05

4.1e-052.8e-053.2e-05

2.5e-052.9e-051.6e-058e-06

0.1969940.2035010.1287030.1040170.1460770.1893460.089329

0.0001670.0003110.0001511.7e-05

0.0001670.0003110.0001511.7e-05

2.6e-059.4e-052.3e-05

2.6e-059.4e-052.3e-05

1.5e-050.0001151.2e-05

1.5e-050.0001151.2e-05

0.0001260.0001020.0001161.7e-05

0.0001260.0001020.0001161.7e-05

0.0041690.0064360.0061270.0095380.0035260.0039880.005535

8e-05

8e-05

8e-05

0.0013240.000940.0025490.0022090.0005350.0012690.000714

3e-061.7e-053e-06

3e-061.7e-053e-06

0.0013210.000940.0025490.0022090.0005180.0012660.000714

5e-062e-061e-06

0.000151

0.0002450.000266

2e-060.0002940.0002711e-063e-06

0.0001192.1e-050.000136.5e-05

1.5e-053e-061.7e-05

0.000215.7e-050.000229

6e-063e-068e-064.8e-05

0.0004990.0001720.0001130.0005418e-05

1.3e-055e-061.4e-05

7e-051.9e-057.2e-05

3.4e-05

1.1e-053e-061.1e-05

4e-061e-065e-06

1.6e-052.5e-058e-06

2.4e-050.0020330.0022091.1e-052.6e-05

5.9e-052.6e-056.4e-05

1.6e-056e-061.6e-05

0.0001844.3e-055.9e-056.1e-05

2.8e-051e-053.1e-05

2.4e-050.0004741.2e-052.7e-050.00016

1.6e-056e-064e-06

5.4e-057.9e-053.6e-05

5.4e-057.9e-053.6e-05

5.4e-057.9e-053.6e-05

0.0027910.0054960.0035780.0073290.0028320.0026830.004821

1e-053.2e-051.3e-05

3e-061.1e-057e-06

1e-068e-060

5e-069e-065e-06

1e-061e-061e-06

3e-06

7e-064.4e-054e-06

3e-05

1e-063e-060

6e-061.1e-054e-06

0.0017490.0044490.0023570.0036140.0024020.0016810.004372

0.0017460.0044490.0023570.0036140.0022910.0016790.004232

2e-060.0001032e-060.00014

1e-068e-060

0.0010250.0010470.0012210.0037150.0003540.0009850.000449

2e-062.9e-052e-06

3e-062.9e-052e-06

3e-061e-06

1.9e-05

1.6e-050

0

0.001010.0010470.0007950.0017070.0009720.000449

7e-060.0004260.0020080.0002618e-06

0.0005754.9e-050.0015850.0014160.0005990.000961

0.0005754.9e-050.0015850.0014160.0005990.000961

0.0001850.0001520.0002233.2e-05

7.6e-053.7e-057.3e-05

3.3e-05

7.7e-054.1e-057.7e-051.6e-05

3.2e-054.1e-057.3e-051.6e-05

0.0001068.2e-059.8e-05

0.0001068.2e-059.8e-05

0.0002424.9e-050.0015850.0010320.000240.000929

0.000126

0.0004550.000495

0.0002424.9e-050.0001120.00024

0.0008550.000929

0.0002750.000299

4.2e-056.9e-053.8e-05

4.2e-056.9e-053.8e-05

8.1e-05

8.1e-05

0.0977780.1331070.0650690.0405630.0793590.0938110.04793

0.0976640.1331070.064560.0405630.0791170.093710.047377

2.2e-05

2.2e-05

0.0960180.1322210.0622610.0386550.0758510.0921150.044467

2.6e-050.001010.0010982.7e-05

0.0959920.1322210.0609130.0386550.0743860.0920880.044467

0.0003380.000367

1.5e-05

1.5e-05

0.0007640.0008860.001850.0019080.000570.0007440.002285

0.0011840.001287

0.0006070.0008860.0006660.0019080.000440.0005830.000998

0.0001570.000130.000161

0.0001099.5e-050.000106

0.0001099.5e-050.000106

4.9e-054.2e-054.5e-05

4.9e-054.2e-054.5e-05

0.000144

0.000144

1.9e-051.6e-05

1.9e-051.6e-05

0.000171

0.000171

1.5e-05

1.5e-05

4.8e-05

4.8e-05

7.7e-05

7.7e-05

0.0002420.0002010.0002332.4e-05

0.0001650.0001130.0001612.4e-05

7.7e-058.8e-057.2e-05

0.0004490.000488

0.0004490.000488

0.00021

0.00021

0.000296

0.000184

0.000112

3.1e-05

3.1e-05

0.0001920.0002580.00021.5e-05

5.3e-054.3e-055.4e-05

0.0001390.0001740.0001461.5e-05

4.1e-05

0.000146

0.000146

0.000172

0.000172

8.1e-05

8.1e-05

4.3e-050.000134.3e-05

2.4e-054.2e-052.5e-05

1.9e-058.8e-051.8e-05

0.00012

0.00012

0.000176

0.000176

0.0001220.0001370.0001151.5e-05

0.0001220.0001370.0001151.5e-05

5.5e-057.7e-054.9e-05

2.7e-056.9e-053.4e-05

2.8e-058e-061.5e-05

5.1e-050.0001154.4e-05

5.1e-050.0001154.4e-05

0.0001140.0005090.0002420.0001010.000553

5.9e-059.7e-055.1e-05

5.9e-059.7e-055.1e-05

9e-067.9e-051.5e-05

9e-067.9e-051.5e-05

4.6e-056.6e-053.5e-05

4.6e-056.6e-053.5e-05

0.0005090.000553

0.0005090.000553

2.4e-050.0001051.9e-05

2.4e-050.0001051.9e-05

2.4e-050.0001051.9e-05

2.4e-050.0001051.9e-05

0.0885820.0600680.05130.047390.0529430.0852870.031858

9.1e-050.0001219.7e-05

9.1e-050.0001219.7e-05

3.3e-052.8e-053.3e-05

1.5e-056e-062e-05

3e-066e-063e-06

1.3e-055e-061.3e-05

1.2e-052.3e-055e-06

5e-062e-061.2e-05

6e-061.3e-057e-06

4e-067e-064e-06

3.1e-05

0.0002670.0001040.0001640.0015060.0005090.0002440.000107

0.0002090.0001040.0001640.0015060.0002350.0001950.000107

0.0002090.0001040.0001640.0015060.0002350.0001950.000107

5.8e-059e-054.9e-05

5.8e-059e-054.9e-05

0.000184

0.000184

0.0689950.0401620.038650.0252010.0433010.0662720.016651

3.2e-052.9e-052.6e-054.8e-05

3.2e-052.9e-052.6e-054.8e-05

0.0024520.001960.0025060.0038150.0021230.0023450.007933

0.0024520.001960.0025060.0038150.0021230.0023450.007933

0.0001790.0001550.0001629.3e-05

7.8e-055.8e-056.6e-056e-06

1e-05

0.0001019.7e-059.6e-057.7e-05

0.000345

9.2e-05

0.000167

8.6e-05

0.0663320.0382020.0361440.0213860.0406490.0637390.008577

0.0654830.0379830.0355220.0213860.0395890.06285

0.0001182.7e-058.8e-050.0001225.6e-05

0.000131

0.0003250.000353

0.0001352.7e-056.1e-050.0001435.4e-05

0.0002155e-050.0002976.8e-050.0002250.004406

0.000167

0.0002498.9e-050.0001290.0002620.004061

0.0001322.6e-056.3e-050.000137

0.0110510.0064920.0048560.0063250.0028590.010610.00188

7.3e-05

7.3e-05

6.3e-05

6.3e-05

5.2e-050.0001224.3e-05

9e-062.1e-057e-06

1.7e-053e-051.4e-05

5e-068e-062e-06

3.9e-05

2.1e-052.4e-052e-05

0.0001533.4e-050.0001740.0001382e-05

1.4e-051.1e-051.5e-051.2e-05

4.6e-05

5.1e-054.1e-054.6e-05

8.8e-052.3e-057.2e-058e-052e-05

4.3e-05

4.3e-05

0.0106240.0064580.0048560.0063250.0021680.0102180.001192

3.9e-05

7.4e-05

2.5e-05

0

3e-06

8.7e-05

0.0009730.0008650.0010090.0023092.3e-050.0009390.00114

1.9e-05

0

6.9e-054.2e-056.1e-05

0.0094790.0055750.0038470.0040160.0018110.009096

0.0001031.8e-054.5e-050.0001225.2e-05

0.0002220.0002160.0002110.000668

0.000202

6.6e-05

7.6e-053.7e-056.8e-050.000234

5.5e-055.1e-054.7e-051.6e-05

4.6e-054.8e-054.6e-050.000148

4.5e-051.4e-055e-056.8e-05

6.1e-057.7e-056e-051.6e-05

6.1e-057.7e-056e-05

6.1e-057.7e-056e-05

1.6e-05

1.6e-05

0.000208

9.4e-05

4.4e-05

5e-05

0.000114

0.000114

0.0012880.0018560.0009440.002410.0008990.0012330.002131

0.0012880.0018560.0009440.002410.0008990.0012330.002131

0.0001362.9e-054.7e-050.0001331.5e-05

0.000116

9.5e-05

1.7e-05

5.9e-053.4e-055.4e-05

7.8e-056.7e-057.9e-051.5e-05

0.0009320.0018270.0009440.002410.000450.0008940.002101

8.3e-057.3e-057.3e-05

0.0004230.0003730.000403

0.0001228.7e-050.000121

0.0001228.7e-050.000121

0.0001480.0001670.000136

0.0001480.0001670.000136

0.0001530.0001190.000146

0.0001530.0001190.000146

0.0007590.0089140.0029030.0101410.0002120.0007220.009959

0.0006950.0089140.0029030.0101410.0001090.0006820.009959

3e-061e-064e-06

5e-064e-065e-06

1e-06

4e-05

2e-061e-052e-06

1.5e-052.4e-051.5e-050.000178

4e-060.0007690.0007085e-064e-06

3e-060.0009210.0008483e-063e-06

0.0006560.0072240.0013470.0101417e-060.0006450.009781

7e-061.4e-054e-06

6.4e-050.0001034e-05

6.4e-053.3e-054e-05

7e-05

7.1e-050.0001047.2e-051.6e-05

7.1e-050.0001047.2e-051.6e-05

7.1e-050.0001047.2e-051.6e-05

0.0053470.002540.0037830.0018070.0035810.0053610.001065

7.6e-050.000186.3e-054.1e-05

7.6e-050.000186.3e-054.1e-05

0.0052710.002540.0037830.0018070.0030920.0052980.00098

0.0024650.0014090.0015290.0018070.0010750.002435

0.0009320.0003770.000430.0002740.0009480.000439

0.0010630.001155

0.0008440.0003580.0004140.0003280.0008650.00034

0.001030.0003960.0003470.000260.001050.000201

9.8e-054.4e-05

9.8e-05

4.4e-05

0.000211

0.000211

7.8e-05

7.8e-05

7.8e-05

0.0002290.0006210.0002133.3e-05

2.7e-05

2.7e-05

9.7e-05

9.7e-05

3.4e-05

3.4e-05

6.1e-057.7e-055.7e-051.8e-05

6.1e-057.7e-055.7e-051.8e-05

0.0001170.0001750.0001061.5e-05

4.1e-055e-053.5e-051.5e-05

2.8e-055.9e-053e-05

4.8e-056.6e-054.1e-05

5.1e-057.2e-055e-05

5.1e-057.2e-055e-05

0.000139

7e-05

6.9e-05

0.0012840.000210.0015690.0034140.0025350.0012740.000226

0.0010018.7e-050.0014010.0019080.0021360.0010059.6e-05

4.5e-05

4.5e-05

3.5e-05

3.5e-05

0.0002680.0003710.0002546.4e-05

4.8e-05

5.3e-05

7.9e-05

0.0001296.7e-050.000127

6.9e-05

0.0001395.5e-050.0001271.6e-05

4.8e-05

0.0003538.7e-050.0014010.0019080.0012260.0003591.6e-05

0.0009520.001034

3.2e-05

5.8e-05

3.3e-05

0.0003538.7e-050.0004490.0019085.2e-050.0003591.6e-05

1.7e-05

4.1e-05

4.1e-05

1.9e-054e-055.7e-05

1.9e-054e-055.7e-05

0.0002310.0002340.000221.6e-05

3.4e-05

8.1e-051.2e-057.9e-05

2.2e-05

1.8e-05

7.3e-052e-056.9e-05

1.4e-05

2.3e-05

4.6e-05

7.7e-054.5e-057.2e-051.6e-05

0.000130.0001440.000115

5.8e-051.7e-055.4e-05

7.5e-05

1.5e-05

7.2e-053.7e-056.1e-05

0.0002830.0001230.0001680.0015060.0003990.0002690.00013

0.000175

2.8e-05

3.9e-05

3e-05

3.7e-05

1.4e-05

2.7e-05

0.0002830.0001230.0001680.0015060.0001320.0002690.00013

2.2e-05

0.0002830.0001230.0001680.0015060.000110.0002690.00013

4.9e-05

2.1e-05

2.8e-05

4.3e-05

4.3e-05

1.2e-054.7e-057e-06

1.2e-054.7e-057e-06

1.2e-054.7e-057e-06

1.2e-054.7e-057e-06

6.8e-050.0003865.8e-05

6.8e-050.0003865.8e-05

4.2e-050.0001873.6e-05

2e-059.5e-051.7e-05

2.2e-059.2e-051.9e-05

2.6e-050.0001992.2e-05

1.8e-059.7e-051.8e-05

8e-060.0001024e-06

0.000239

0.000113

0.000113

0.000113

0.000126

0.000126

0.000126

0.0043350.0036310.0030530.0031120.005210.0041520.002802

0.0038990.0035960.0030530.0031120.0044280.0037320.002802

0.0002340.000254

0.0002340.000254

0.000169

0.000169

0.00016

0.00016

0.0001310.0001320.000128

0.0001310.0001320.000128

0.000190.0002170.0001721.6e-05

8.5e-050.0001197.7e-05

0.0001054.8e-059.5e-051.6e-05

5e-05

0.0032810.0035830.0028190.0031120.0031180.003150.002768

0.0031010.0035830.0028190.0031120.0029530.0029750.002768

0.000180.0001650.000175

0.0002221.3e-050.0001650.0002241.8e-05

0.000116.3e-050.000116

1.8e-05

0.0001121.3e-053.5e-050.0001081.8e-05

4.9e-05

5.1e-050.0001234.3e-05

5.1e-050.0001234.3e-05

2.4e-059e-051.5e-05

2.4e-059e-051.5e-05

0.0004363.5e-050.0007820.00042

7.1e-050.0001146.1e-05

5.3e-05

7.1e-056.1e-056.1e-05

0.0001450.0001990.000141

0.0001450.0001990.000141

0.000143

0.000143

6.7e-056.3e-055.9e-05

6.7e-056.3e-055.9e-05

9.3e-053.5e-059.6e-059.6e-05

9.3e-053.5e-059.6e-059.6e-05

2e-060.0001313e-06

2e-05

0.000108

2e-063e-063e-06

5.8e-053.6e-056e-05

3.3e-051.9e-053.5e-05

2.5e-051.7e-052.5e-05

0.0030830.0016620.0046030.0052210.0030490.0029030.001209

0.003060.0016620.0046030.0052210.002960.0028830.001209

0.0001190.0027720.0030120.0003287.3e-05

8e-065.3e-057e-06

1e-05

5e-06

6e-06

1e-069e-061e-06

7e-061.4e-056e-06

9e-06

5e-050.0027720.0030120.0002154e-05

5.2e-05

1.8e-05

2e-064e-062e-06

2.5e-053e-062.5e-05

5e-062e-065e-06

1.2e-05

4e-06

2e-05

1e-060.0027720.0030121e-062e-06

1e-061.5e-05

7e-063.1e-056e-06

8e-06

4e-06

1.1e-05

9e-061.7e-05

1.3e-05

6.1e-056e-052.6e-05

7e-061e-057e-06

4.1e-053e-057e-06

1.3e-052e-051.2e-05

0.0029260.0016620.0018310.0022090.00260.0027970.001209

1.2e-052e-056e-06

1.2e-052e-056e-06

4e-063.1e-054e-06

4e-063.1e-054e-06

2.1e-054.4e-051.8e-05

1.5e-052.1e-051.2e-05

6e-062.3e-056e-06

0.0028890.0016620.0018310.0022090.0025050.0027690.001209

1e-062.9e-050

0.0028670.0016620.0018310.0022090.0023690.002750.001209

2.9e-05

2e-069e-062e-06

2.4e-05

1e-069e-061e-06

000

8e-068e-06

7e-0606e-06

3e-061.7e-052e-06

1.9e-05

1.5e-053.2e-051.3e-05

1.5e-053.2e-051.3e-05

1.5e-053.2e-051.3e-05

1.3e-055e-051.1e-05

1.3e-055e-051.1e-05

1e-052.4e-055e-06

1e-051.3e-055e-06

1.1e-05

3e-062.6e-056e-06

3e-062.6e-056e-06

1e-053.9e-059e-06

1e-053.9e-059e-06

1e-053.9e-059e-06

1e-053.9e-059e-06

6.7e-059e-056.1e-05

6.7e-059e-056.1e-05

6.7e-059e-056.1e-05

6.7e-059e-056.1e-05

1.3e-053.9e-051.6e-05

4.2e-052.8e-053.5e-05

1.2e-052.3e-051e-05

0.0714470.0476650.1198050.0592350.0914310.0684690.094072

0.0019520.0003160.0016290.0021660.0017360.000278

0.0004260.000220.000354

0.0002580.0001080.000206

0.0002580.0001080.000206

0.0001680.0001120.000148

0.0001680.0001120.000148

0.0014060.0003160.0016290.0018420.0012890.000278

0.0006577.6e-050.0002350.0002920.0005960.000141

1.3e-05

0.0004587.6e-050.0002357e-060.000410.000141

9.5e-05

0.0001990.0001770.000186

0.0007490.000240.0013940.001550.0006930.000137

0.0007490.000240.0002660.0003240.0006930.000137

0.0011280.001226

0.000120.0001049.3e-05

0.000120.0001049.3e-05

0.000120.0001049.3e-05

0.0349330.0227750.074470.0307220.051520.0337980.049102

0.0170730.009880.0304710.0113450.0303570.0171290.010492

0.0006226e-050.0003870.0005774.7e-05

0.0001752e-055.8e-050.000166

9.1e-055.3e-057.4e-051.5e-05

0.000101

3.9e-05

0.0001216.2e-050.0001113.2e-05

0.0001764e-054.2e-050.000171

5.9e-053.2e-055.5e-05

0.0086490.0044010.0245490.0249750.0089430.004866

0.0189220.020563

0.0020910.0004120.000770.0011840.0021690.000566

0.0026480.0010410.0016720.0027420.002006

0.0021340.0026530.001540.0008850.0022040.001791

0.0017760.0002950.0006310.0012410.0018280.000503

0.0010140.001102

0.0025480.0008650.0032390.0021080.0021470.0024340.00112

0.0025140.0021450.0021040.002416

1.6e-05

3.4e-050.0008650.0010940.0021082.7e-051.8e-050.00112

0.0001320.000480.000114

1.5e-05

2.4e-05

2.6e-05

8.4e-05

2.7e-05

2.7e-05

0.000174

8.8e-05

0.0001321.5e-050.000114

0.0018030.0009950.0009710.0017070.00080.0017690.001192

0.0012030.0003220.0004940.0003750.0011759.3e-05

3.1e-05

0.00060.0006730.0004770.0017070.0001910.0005940.001068

0.00011

0.000124

0.0033190.0035590.0017120.007530.0015680.0032920.003267

1.3e-05

2.2e-05

0.000265.3e-059.5e-050.000254

0.0002320.0021560.0004530.0020081.8e-050.0002260.002109

5.9e-05

6e-06

9.3e-053e-059.5e-058e-06

0.0001041.5e-053e-060.0001110.000122

2.6e-05

0.0012540.0010640.0007240.0018070.0003310.001220.000668

6.6e-052.4e-059e-066.7e-051e-05

0.000137

0.0001326.4e-050.000137

0.0002773e-050.0002544.2e-050.0002763.4e-05

6.4e-059.8e-050.0002810.0019081.5e-056.3e-050.000166

0.0001191.3e-050.000122

5.5e-05

2.1e-055e-062.2e-055e-06

6e-05

2.1e-05

7.7e-05

3.5e-058e-063.5e-05

0.000212.1e-050.000203

1e-06

0.000214.9e-050.0018070.0001260.0002070.000128

1.6e-05

0.000119

2.5e-05

2.4e-05

0.0001422.6e-052.6e-050.000151

3.4e-05

4e-06

5.4e-051.4e-051e-055.8e-05

2.1e-05

1e-05

0

4.6e-053e-056e-064.5e-051.7e-05

1e-06

1.5e-05

0.0120640.01311

0.0120640.01311

0.0120640.01311

0.001240.0026160.0018690.0041160.0012760.000990.001349

4.8e-058.4e-053.9e-05

4.8e-058.4e-053.9e-05

0.000106

0.000106

0.000118

0.000118

0.0007510.0025670.001610.0041160.0005440.0006860.001303

0.0002158.7e-050.000173

0.0001224.2e-050.0001111.8e-05

3.2e-05

9.3e-05

8.7e-05

3.4e-05

2.6e-050.00086202.5e-050.000937

7e-06

3e-05

5.8e-05

2.2e-050.0012950.0003720.0021087e-062.2e-050.000162

0.0003660.0012720.0003760.0020086.7e-050.0003550.000186

4.4e-050.0001182.3e-05

7.3e-05

4.4e-054.5e-052.3e-05

0.0003674.9e-050.0002590.0001170.0002284.6e-05

3.9e-05

8e-06

0.0003674.9e-050.0002597.8e-050.0002283.8e-05

0.000122

0.000122

3e-056.7e-051.4e-05

2.6e-055.1e-051.1e-05

4e-061.6e-053e-06

0.0053560.0024790.0029850.0042170.0027420.005040.00197

0.0001056.2e-058.9e-05

0.0001056.2e-058.9e-05

0.0013410.0003780.0010780.0019080.0010250.0012580.000327

0.000122

0.0013410.0003780.0004420.0019080.0002120.0012580.000327

0.0006360.000691

0.000171

8e-05

9.1e-05

0.0038370.0021010.0019070.0023090.0012660.0036440.001643

4.5e-05

0.0038370.0021010.0019070.0023090.0011870.0036440.001643

3.4e-05

7.3e-050.0002184.9e-05

5.8e-05

7.9e-05

7.3e-058.1e-054.9e-05

0.0008020.0194110.0090080.0006690.012827

0.0001768e-050.000143

0.0001768e-050.000143

0.0002379.5e-050.000201

0.0002379.5e-050.000201

0.0002910.000150.000241.9e-05

0.0002910.000150.000241.9e-05

6e-05

6e-05

4.1e-05

4.1e-05

0.0194110.0084560.012808

0.0076250.008286

0.000113

0.0117860.012808

5.7e-05

5.3e-05

5.3e-05

9.8e-057.3e-058.5e-05

4.2e-057.3e-053.7e-05

5.6e-054.8e-05

0.0104620.00780.007670.0110440.0081370.009970.009354

0.0063870.0061460.0060120.0072290.0057290.0061150.008306

0.0063870.0061460.0060120.0072290.0056540.0061150.008306

7.5e-05

0.0002647.4e-050.0002241.7e-05

0.0002647.4e-050.0002241.7e-05

0.000119

4.4e-05

7.5e-05

7.6e-05

3.9e-05

3.7e-05

2.7e-055.5e-052.1e-05

2.7e-055.5e-052.1e-05

0.000132.4e-055.1e-050.000134

6.9e-051.6e-056.8e-05

2.3e-05

6.1e-052.4e-051.2e-056.6e-05

0.0004847.3e-050.0003150.0004530.0004496.4e-05

0.0003417.3e-050.0003150.0003550.0003313.1e-05

0.0001439.8e-050.0001183.3e-05

0.002620.0015130.0013430.0038150.0010170.0025340.000967

9.1e-056.3e-052.5e-058.8e-054.2e-05

0.0018450.0012850.0010520.0020080.0005780.0017980.000875

0.0003920.0001650.0002910.0018070.0002510.000376

0.0001688.2e-050.000165e-05

3.9e-05

0.0001244.2e-050.000112

0.0001860.0001680.000173

0.0001860.0001680.000173

8e-057.2e-056.7e-05

8e-057.2e-056.7e-05

9.9e-05

9.9e-05

0.000128

5.7e-05

7.1e-05

0.0001574.4e-055e-050.000139

0.0001574.4e-055e-050.000139

0.0001274.6e-050.000114

0.0001274.6e-050.000114

0.0087210.0099940.0278090.0168670.0109310.0082710.032402

0.008070.0099940.0278090.0168670.0106290.0077950.032337

0.0003840.000417

0.0003840.000417

0.0002760.0003

0.0002760.0003

0.0018220.0010910.000889

0.0010040.001091

0.0008180.000889

0.008070.0099940.0238660.0168670.0092380.0077950.029443

0.0006840.000743

0.0022510.002447

0.0068630.007458

0.0021910.0014430.0007090.0022090.0002050.0014540.001586

0.0043130.0018040.0015120.0105420.0017970.0043050.000505

0.000158

2.2e-05

0.0031030.003372

0.0022980.002497

0.000116

0.0019790.002151

0.0021940.002384

0.0015660.0067470.000670.0041160.0001520.0020360.007319

0.0016030.00470.001069

0.0014610.001588

0.0014610.001588

0.0006510.0003020.0004766.5e-05

0.0002790.0001180.0002131.6e-05

8e-06

0.0002790.0001180.0002138e-06

0.0001739.3e-050.000111.7e-05

0.0001739.3e-050.000111.7e-05

0.0001999.1e-050.0001533.2e-05

0.0001999.1e-050.0001533.2e-05

0.0015750.0009310.0009540.0018070.0014120.0014950.00054

0.0015750.0009310.0009540.0018070.0014120.0014950.00054

0.0013680.0009310.0009540.0018070.0012320.0013060.00054

0.0013680.0009310.0009540.0018070.0012320.0013060.00054

4.9e-051.6e-054.6e-05

4.9e-051.6e-054.6e-05

5.9e-052.8e-056.3e-05

5.9e-052.8e-056.3e-05

9.9e-050.0001368e-05

4.1e-051.6e-053e-05

1.9e-055e-051.7e-05

1e-052e-056e-06

1e-052.1e-057e-06

1.9e-052.9e-052e-05

0.0242660.0136490.0149430.0098390.0254020.0231690.01175

0.0008680.0006770.000356

9.1e-05

9.1e-05

0.0008680.0005860.000356

0.0003280.000356

0.000540.000586

0.000960.0002210.0004460.0033290.0009220.000297

0.000960.0002210.0004460.0033290.0009220.000297

0.000960.0002210.0004460.0033290.0009220.000297

0.0002930.0002870.00021

5.6e-057.5e-053.8e-05

5.6e-057.5e-053.8e-05

0.0001299e-059.5e-05

0.0001299e-059.5e-05

0.0001080.0001227.7e-05

0.0001080.0001227.7e-05

0.0001450.0003230.000119

0.000120.0002129.5e-05

2.9e-054.2e-053.7e-05

4.8e-05

2.1e-05

4.2e-054.6e-051e-05

3.6e-054.2e-052.9e-05

1.3e-051.3e-051.9e-05

2.5e-050.0001112.4e-05

5.4e-05

2.5e-055.7e-052.4e-05

0.0228680.0134280.0136290.0098390.0207860.0219180.011097

5.9e-056.1e-054.5e-05

5.9e-056.1e-054.5e-05

0.0227360.0134280.0136290.0098390.0205230.0218110.011097

0.0226990.0134280.0136290.0098390.0204380.0217780.011097

3.7e-058.5e-053.3e-05

5.9e-05

5.9e-05

7.3e-051.5e-056.2e-05

4.3e-057e-063.8e-05

3e-058e-062.4e-05

0.000128

6.3e-05

6.5e-05

0.009330.0052610.0057180.0066270.0102830.0088840.00407

2.9e-053.5e-052.4e-05

2.9e-053.5e-052.4e-05

2.9e-053.5e-052.4e-05

2.9e-053.5e-052.4e-05

3.3e-050.0003083.2e-05

1.1e-056.4e-051e-05

1.1e-056.4e-051e-05

7e-062.2e-057e-06

4e-064.2e-053e-06

2.2e-050.0002442.2e-05

2.2e-050.0002442.2e-05

2.2e-050.0002442.2e-05

0.0078460.004730.0051050.004920.0076420.0075180.00371

0.0040460.00240.0025980.002510.0038960.0038820.001849

8e-062.1e-058e-06

8e-062.1e-058e-06

1e-063.2e-051e-06

1e-063.2e-051e-06

6e-063.6e-055e-06

6e-063.6e-055e-06

0.0040310.00240.0025980.002510.0038070.0038680.001849

0.0040310.00240.0025980.002510.0038070.0038680.001849

0.00380.002330.0025070.002410.0037460.0036360.001861

0.0037510.002330.0025070.002410.003620.0035970.001861

0.0037510.002330.0025070.002410.003620.0035970.001861

2e-054.3e-051.8e-05

2e-054.3e-051.8e-05

5e-063.6e-054e-06

5e-063.6e-054e-06

2.4e-054.7e-051.7e-05

2.4e-054.7e-051.7e-05

0.0001550.000370.000136

0.0001230.0002350.000109

0.0001230.0001950.000109

2.1e-052e-051.7e-05

7e-067e-063e-06

1.2e-054e-051.1e-05

7e-06

1.6e-053.2e-051.7e-05

1.2e-052.7e-051.1e-05

2.3e-05

2.2e-051e-051.7e-05

6e-062.3e-057e-06

2.7e-056e-062.6e-05

4e-05

4e-05

3.2e-050.0001352.7e-05

7.4e-05

3.4e-05

4e-05

3.2e-056.1e-052.7e-05

1.9e-052.8e-051.8e-05

1.3e-053.3e-059e-06

2.5e-050.0001692e-05

6e-063.8e-056e-06

6e-063.8e-056e-06

6e-063.8e-056e-06

1.9e-050.0001311.4e-05

3e-063e-052e-06

3e-063e-052e-06

1.2e-057.1e-051e-05

1.2e-057.1e-051e-05

4e-063e-052e-06

4e-063e-052e-06

3.4e-053e-052.4e-05

3.4e-053e-052.4e-05

3.4e-053e-052.4e-05

3.4e-053e-052.4e-05

1.9e-059.8e-052.1e-05

1.9e-059.8e-052.1e-05

4.1e-05

4.1e-05

1.9e-055.7e-052.1e-05

1.9e-055.7e-052.1e-05

0.0010430.0005310.0006130.0017070.0011550.000980.00036

0.0010080.0005310.0006130.0017070.001050.0009490.00036

6e-062.5e-056e-06

6e-062.5e-056e-06

0.0010020.0005310.0006130.0017070.0010250.0009430.00036

1.6e-051.3e-05

2.2e-053.2e-052.1e-051.6e-05

4.8e-05

9e-064e-06

0.000870.0005310.0006130.0017070.0007820.0008330.000344

1e-052.8e-056e-06

2.5e-053.1e-052.2e-05

1.4e-051.7e-051.1e-05

1e-061e-06

2.2e-05

6e-063.3e-057e-06

2.9e-053.2e-052.5e-05

9e-062.2e-051e-05

9e-062.2e-051e-05

9e-062.2e-051e-05

2.6e-058.3e-052.1e-05

2.6e-058.3e-052.1e-05

4.9e-05

2.6e-053.4e-052.1e-05

0.0001460.0004760.000129

4.8e-059.3e-054.7e-05

4.8e-059.3e-054.7e-05

2.9e-055.3e-052.8e-05

1.2e-052.5e-051.3e-05

7e-061.5e-056e-06

1.5e-058.2e-051.2e-05

4.5e-05

4.5e-05

1.5e-053.7e-051.2e-05

1.5e-053.7e-051.2e-05

4.8e-059.9e-054.3e-05

1.6e-052.7e-051.4e-05

1.6e-052.7e-051.4e-05

3.2e-057.2e-052.9e-05

1.3e-052e-051e-05

1.1e-051.8e-051.1e-05

1.4e-05

8e-062e-058e-06

2.2e-054.1e-051.6e-05

2.2e-054.1e-051.6e-05

2.2e-054.1e-051.6e-05

1.3e-057.4e-051.1e-05

1.3e-054.6e-051.1e-05

1.3e-054.6e-051.1e-05

2.8e-05

2.8e-05

4.1e-05

4.1e-05

4.1e-05

4.6e-05

4.6e-05

4.6e-05

2.5e-057.8e-051.1e-05

2.5e-057.8e-051.1e-05

2.5e-057.8e-051.1e-05

2.5e-057.8e-051.1e-05

2.5e-057.8e-051.1e-05

2.5e-057.8e-051.1e-05

0.0031340.0018120.0019420.0021080.0029820.0029550.001479

0.0031340.0018120.0019420.0021080.0029820.0029550.001479

0.002960.0018120.0019420.0021080.002650.0028310.001479

0.002960.0018120.0019420.0021080.002650.0028310.001479

1.3e-053.1e-051.1e-05

1.3e-053.1e-051.1e-05

0.0029110.0018120.0019420.0021080.0025170.0027920.001479

2e-06

6e-062.3e-054e-06

3e-069e-063e-06

1.4e-055.6e-05

0.0028960.0018120.0019420.0021080.0024490.0027780.001423

6e-062e-057e-06

1.6e-055.5e-051.1e-05

6e-062.7e-055e-06

1e-052.8e-056e-06

2e-054.7e-051.7e-05

2e-054.7e-051.7e-05

0.0001740.0003320.000124

0.0001440.0002819.9e-05

0.0001440.0002819.9e-05

2.9e-053.1e-052.2e-05

1e-052.2e-057e-06

3.3e-053.9e-051.9e-05

1.9e-053.7e-051.5e-05

2e-051.9e-051.3e-05

1.1e-053.4e-058e-06

1e-05

1.1e-05

2.4e-05

1.7e-05

2.2e-053.7e-051.5e-05

3e-055.1e-052.5e-05

3e-055.1e-052.5e-05

3e-055.1e-052.5e-05

0.0002580.000218

0.0002580.000218

0.0002580.000218

0.0002580.000218

0.0001650.00012

0.0001650.00012

0.0001650.00012

9.3e-059.8e-05

9.3e-059.8e-05

9.3e-059.8e-05
