# Supplementary material for: MetaMeta: integrating metagenome analysis tools to improve taxonomic profiling
Source: Microbiome. 2017 Aug 14;5:101. doi: 10.1186/s40168-017-0318-y (PMC5557516; doi:10.1186/s40168-017-0318-y)
Supplement: Supplementary file 2 — Additional File with interactive charts for all CAMI toy set results on default, very-precise and very-sensitive mode. File prefix S, M, and H for low, medium and high complexity, respectively. (TAR 3573 kb) [file 40168_2017_318_MOESM2_ESM.tar › H_S002__insert_180_very-sensitive.html]

Javascript must be enabled to view this page.

magnitude
magnitudeUnassigned

clark.parsed\_profile
dudes.parsed\_profile
final.metametamerge.profile
gottcha.parsed\_profile
kaiju.parsed\_profile
kraken.parsed\_profile
motus.parsed\_profile

0.9999999999999980.9999969999999991.0000120.9999930000000021.0000020.9999959999999980.999994

0.9153339999999990.9430019999999990.9482860.9491920000000020.9203799999999980.9185639999999980.949545

3e-064.2e-052e-06

4.2e-05

4.2e-05

4.2e-05

4.2e-05

4.2e-05

3e-062e-06

3e-062e-06

3e-062e-06

3e-062e-06

3e-062e-06

2e-063.6e-051e-06

2e-063.6e-051e-06

2e-063.6e-051e-06

2e-063.6e-051e-06

2e-063.6e-051e-06

2e-063.6e-051e-06

2.1e-050.0001951.4e-05

9e-064.1e-052e-06

9e-064.1e-052e-06

9e-064.1e-052e-06

9e-064.1e-052e-06

9e-064.1e-052e-06

1.2e-057.6e-051.2e-05

1.2e-057.6e-051.2e-05

1.2e-057.6e-051.2e-05

1.2e-057.6e-051.2e-05

1.2e-057.6e-051.2e-05

7.8e-05

7.8e-05

7.8e-05

7.8e-05

7.8e-05

0.0037810.0019550.0021950.0023090.0038670.0036390.001517

0.0037610.0019550.0021950.0023090.0038130.0036260.001517

0.0037610.0019550.0021950.0023090.0038130.0036260.001517

0.003750.0019550.0021950.0023090.0037560.0036150.001517

2e-063.2e-053e-06

2e-063.2e-053e-06

7e-064.9e-056e-06

7e-064.9e-056e-06

4.1e-05

4.1e-05

5e-062.7e-054e-06

5e-062.7e-054e-06

0.0037360.0019550.0021950.0023090.0036070.0036020.001517

0.0037360.0019550.0021950.0023090.0036070.0036020.001517

1.1e-055.7e-051.1e-05

7e-062.8e-057e-06

7e-062.8e-057e-06

4e-062.9e-054e-06

4e-062.9e-054e-06

2e-055.4e-051.3e-05

2e-055.4e-051.3e-05

2e-055.4e-051.3e-05

2e-055.4e-051.3e-05

2e-055.4e-051.3e-05

3.7e-050.0002973.7e-05

3.7e-050.0002973.7e-05

3.7e-050.0002973.7e-05

3.7e-050.0002973.7e-05

1e-054.6e-056e-06

4e-06

3e-064e-062e-06

7e-063.8e-054e-06

1.5e-055.6e-052.3e-05

1.5e-055.6e-052.3e-05

1.2e-050.0001958e-06

1.2e-056.4e-058e-06

5.6e-05

7.5e-05

1.9e-050.000221e-05

1.9e-050.000221e-05

1.9e-050.000221e-05

1.9e-050.000221e-05

8e-064.2e-055e-06

8e-064.2e-055e-06

5.4e-05

5.4e-05

4e-063.3e-051e-06

4e-063.3e-051e-06

3e-063.9e-051e-06

3e-063.9e-051e-06

4e-065.2e-053e-06

4e-065.2e-053e-06

0.1563380.2171620.196350.2212860.1175420.1388120.248789

0.0026410.0013710.0188820.0035140.0098180.0025230.013223

0.0022980.0013710.0018090.0035140.0022220.0022070.00118

0.0022980.0013710.0018090.0035140.0022220.0022070.00118

0.0022980.0013710.0018090.0035140.0022220.0022070.00118

0.0019040.0011320.001070.0020080.0017840.0018270.00051

0.000450.000506

0.0003940.0002390.0002890.0015060.0004380.000380.000164

0.000290.0170730.0073030.0002670.012043

0.000290.0165630.0066420.0002670.012043

0.000290.0165630.0066420.0002670.012043

0.0005380.000606

0.004530.005104

6.7e-050.0001220.0001387.4e-05

0.0106890.012043

0.0002230.0001920.000240.000193

0.0004920.000554

0.000510.000661

0.000510.000661

8.6e-05

0.000510.000575

5.3e-050.0002934.9e-05

5.3e-050.0002934.9e-05

3.1e-050.0001132.6e-05

3.1e-050.0001132.6e-05

0.000104

0.000104

2.2e-057.6e-052.3e-05

2.2e-057.6e-052.3e-05

0.0259080.032780.0373460.030120.0266980.0216240.047813

0.0214960.0295380.0345520.0259040.0232930.0173710.045416

0.0001460.000164

0.0001460.000164

0.0001460.000164

0.0058490.0119580.0082020.0062250.0037740.0025270.01576

8.5e-05

8.5e-05

0.0057610.0119580.0044250.0062250.0034410.0024440.01165

0.0057610.0119580.0044250.0062250.0034410.0024440.01165

6.5e-050.0001290.0001466.3e-05

6.5e-050.0001290.0001466.3e-05

2.3e-050.0001022e-05

2.3e-050.0001022e-05

0.0036480.00411

0.0036480.00411

0.0001280.0005810.000108

1.7e-059.4e-051.6e-05

8e-063.1e-058e-06

2e-062.9e-052e-06

7e-063.4e-056e-06

1.9e-05

1.7e-05

2e-06

0

1.2e-054.4e-051e-05

1.2e-054.4e-051e-05

1.3e-050.0001251.7e-05

2e-061.6e-053e-06

5.1e-05

7e-062.4e-058e-06

4e-063.4e-056e-06

9e-06

9e-06

1.4e-056.3e-051.2e-05

1.4e-056.3e-051.2e-05

1e-055.4e-058e-06

1e-055.4e-058e-06

5.3e-050.0001824.5e-05

1.3e-052.8e-051.3e-05

5e-063.1e-053e-06

4e-062.8e-054e-06

9e-06

1.5e-054.7e-052e-05

7e-064.8e-055e-06

4.4e-050.0001560.0001754.3e-05

4.4e-050.0001560.0001754.3e-05

4.4e-050.0001560.0001754.3e-05

4.3e-055e-053.2e-05

4.3e-055e-053.2e-05

4.3e-055e-053.2e-05

1.4e-050.00011.2e-05

3e-065.1e-052e-06

3e-065.1e-052e-06

1.1e-054.9e-051e-05

1.1e-054.9e-051e-05

2e-050.0009770.0011731.9e-05

0.0003360.000379

0.0003360.000379

0.0006410.000722

0.0006410.000722

2e-057.2e-051.9e-05

2e-057.2e-051.9e-05

0.0002030.0001610.0006240.0002088.1e-05

1.3e-056e-051.6e-05

1.3e-056e-051.6e-05

0.000190.0001610.0005640.0001928.1e-05

5.4e-058.2e-055.3e-05

0.0001360.0001610.0004820.000139

8.1e-05

0.0011990.0012270.0095060.0067270.0034760.0009610.007501

0.0002269.7e-050.0013120.0010120.0001370.000722

0.0006410.000722

2.5e-050.0002120.0002392.6e-05

0.0002950.000332

0.0002019.7e-050.0001640.0004410.000111

7.7e-050.0003120.000352

7.7e-05

0.0003120.000352

2e-058.6e-051.9e-05

2e-058.6e-051.9e-05

0.0002042.3e-050.0054960.0004756.6e-050.005918

0.0001780.0002

0.0010890.001226

0.0037140.004185

0.0002042.3e-050.0002530.0004756.6e-05

4e-06

0.0002620.000295

4e-06

4e-06

1.5e-050.0005380.0002172e-050.000389

0.0003450.000389

1.5e-050.0001930.0002172e-05

0.0004660.0008650.0005095e-06

0.0001860.0001655e-06

0.0004510.000509

0.000280.000249

0.0007340.0005640.0008430.0067270.0010190.000719

0.0007340.0003890.0006880.0067270.0010190.000719

0.0001750.000155

0.000115

1.9e-05

9.6e-05

0.000140.000158

0.000140.000158

3.7e-055.5e-052.6e-05

3.7e-055.5e-052.6e-05

3.7e-055.5e-052.6e-05

0.010590.0137940.0103710.0089360.0058250.0101680.01872

0.0012270.0018930.008148

0.0012270.0018930.008148

5.1e-050.0001245e-05

5.1e-050.0001245e-05

7.1e-050.0001740.0004940.0003827e-05

0.0002270.000255

1.8e-050.0001120.0001271.9e-05

0.0001740.000155

5.3e-055.1e-05

7.4e-050.0001220.0001377.2e-05

7.4e-050.0001220.0001377.2e-05

0.0103940.0123930.0078620.0089360.0051820.0099760.010572

4e-062.6e-056e-06

0.0103670.0123930.0076570.0089360.0047320.0099460.010572

0.0002050.000232

4e-065.5e-055e-06

1.9e-052.4e-051.9e-05

0.000113

3.5e-059.7e-052.4e-05

3.5e-059.7e-052.4e-05

3.5e-059.7e-052.4e-05

0.0033340.0025590.0050330.0040160.0071990.0032430.003354

0.0033070.0025590.0050330.0040160.0070060.0032130.003354

6.5e-05

0.0013450.001516

1.9e-057e-062.6e-05

2.4e-052.3e-052.5e-05

1e-053e-069e-061.4e-05

2.6e-051e-062.7e-05

5.1e-05

3.2e-053.6e-053.4e-05

3.2e-052.6e-053.6e-05

6e-05

9e-063.5e-059e-06

9.7e-05

1.8e-052.9e-052e-05

3.1e-05

0.001470.0018980.001469

1.9e-05

0.0023980.0014380.0015310.0021080.0024870.0023060.000947

2e-052.6e-052.3e-05

7e-06

1.8e-054.6e-052e-05

2.1e-051.7e-051.4e-05

0.000610.0010790.0006870.0019080.0005680.0005860.000841

2.4e-05

3.7e-053.9e-056e-063.8e-05

3.3e-051.9e-053.5e-05

1.2e-050.0001121.4e-05

6e-065.4e-057e-06

6e-065.8e-057e-06

4.8e-05

4.8e-05

1.5e-053.3e-051.6e-05

6e-062e-066e-06

9e-063e-051e-05

1e-06

0.004380.0032420.0027940.0042160.0031450.004210.002397

0.0020360.001390.0013390.0021080.0018640.0019480.000924

7.2e-050.0001117.8e-05

3.8e-053.8e-054e-05

4.6e-05

2e-051.4e-052e-05

1.4e-051.3e-051.8e-05

000

3e-062.6e-052e-06

3e-062.6e-052e-06

7e-064.7e-057e-06

7e-064.7e-057e-06

1.2e-055.6e-059e-06

1.2e-055.6e-059e-06

9e-064.2e-055e-06

9e-064.2e-055e-06

7e-061.3e-056e-06

7e-061.3e-056e-06

0.0019260.001390.0013390.0021080.0015690.0018410.000924

0.0019260.001390.0013390.0021080.0015690.0018410.000924

0.0023220.0018520.0014550.0021080.0011410.0022490.001473

0.0023030.0018520.0014550.0021080.0010420.0022270.001473

4e-061.6e-054e-06

5e-061e-055e-06

1e-069e-061e-06

0.002270.0018520.0014550.0021080.0009330.0021960.001473

3e-068e-064e-06

6e-061.3e-057e-06

1e-053.4e-057e-06

4e-061.9e-053e-06

1.2e-054.3e-051.7e-05

6e-061.5e-055e-06

6e-062.8e-051.2e-05

7e-065.6e-055e-06

7e-065.6e-055e-06

6e-065e-056e-06

6e-065e-056e-06

6e-065e-056e-06

1.6e-059e-057e-06

1e-055.2e-053e-06

1e-055.2e-053e-06

6e-063.8e-054e-06

6e-063.8e-054e-06

7e-063.5e-055e-06

7e-063.5e-055e-06

7e-063.5e-055e-06

7e-063.5e-055e-06

2.5e-050.0002253.8e-05

1.2e-059.1e-051.3e-05

9e-065e-051.2e-05

9e-065e-051.2e-05

3e-064.1e-051e-06

3e-064.1e-051e-06

1.3e-050.0001342.5e-05

9e-067.9e-052.1e-05

6e-063.5e-051.1e-05

3e-064.4e-051e-05

4e-065.5e-054e-06

4e-065.5e-054e-06

0.0015560.0007750.0047270.0044170.0022550.0014960.004713

0.0015560.0007750.0047270.0044170.0022550.0014960.004713

0.0015560.0007750.0047270.0044170.0022550.0014960.004713

9.4e-05

9.4e-05

0.000102

0.000102

0.0011910.0005930.0008270.0018070.0011540.001140.00062

0.0011910.0005930.0008270.0018070.0011540.001140.00062

0.0003650.0001820.00390.002610.0009050.0003560.004093

9.8e-05

0.0035460.003995

0.0003650.0001820.0003540.002610.0009050.000356

0.1260060.1822360.1273380.1832350.0767780.1129450.17593

0.1017910.1557390.1037370.1496020.052930.0965090.134111

0.0044880.0029520.0070960.0091370.0021040.0043130.002764

1.5e-053.2e-051.5e-05

1.5e-053.2e-051.5e-05

0.0037090.0020660.0064060.007330.0014380.0035630.002206

1.5e-051.9e-050.0016930.0019081.3e-051.1e-05

0.0036170.0020240.0018580.002510.0010460.0034760.002206

2e-052.3e-050.0025840.0029121.5e-051.9e-05

0.0002710.000305

3.2e-053.7e-053.2e-05

2.5e-051.3e-052.5e-05

9e-06

0.0007640.0008860.000690.0018070.0006340.0007350.000558

0.0007640.0008860.000690.0018070.0006340.0007350.000558

0.0027550.0015610.0057010.0071280.0035060.0026530.001931

0.0015730.000840.004920.0054210.0023390.0015220.001462

2.8e-050.0016040.0018072.4e-053e-05

0.00070.0010950.000616

3.7e-052.5e-052.6e-05

4.3e-053e-055.1e-05

0.0014170.000840.0010120.0018070.0011120.001370.000846

2.6e-053.4e-052.4e-05

2.2e-050.0016040.0018071.9e-052.1e-05

0.0011550.0007210.0007810.0017070.0011140.0011080.000469

4.4e-05

1.5e-05

0.0011550.0007210.0007810.0017070.0010550.0011080.000469

2.7e-055.3e-052.3e-05

2.7e-055.3e-052.3e-05

0.0017060.0013030.0013890.0016273.2e-05

0.0017060.0013030.0013890.0016273.2e-05

0.0016440.0013030.0012470.001579

5.6e-05

1.6e-05

3.2e-053.6e-051.8e-051.6e-05

3e-055e-053e-05

0.0537590.0559660.0386590.0574320.0248840.0459380.04175

0.0537440.0559660.0370550.0556250.0247890.0459070.04175

0.0013990.0028110.0019770.002510.001920.0032650.002514

7e-065e-060.0016040.0018071e-067e-06

3.8e-051.8e-050.0001960.0027114.9e-050.000115

0.000430.0003340.0003330.0017070.0001620.000420.000659

0.0007670.0001940.000280.0037150.0003970.0007410.000118

0.0085230.008010.0048510.004920.0028760.008223

6.3e-052.6e-056e-05

2.8e-059e-062.7e-051.9e-05

0.0120620.006280.0054360.0040160.0075890.0116960.003687

2.3e-057e-062.4e-058e-06

7.8e-055.9e-058.1e-05

5.8e-05

8.8e-05

5.4e-054.8e-050.0002790.0017074.5e-050.0001540.00041

0.0002620.0011140.0003160.002410.0001490.0002230.001936

1.8e-05

0.0003980.000448

0.0001670.000189

9.4e-056.5e-059.1e-05

0.0001314.7e-050.0001310.000106

0.0002950.0002140.0066275.1e-050.0002810.000105

0.0002970.0002340.0001790.0002870.000367

0.0006550.000738

0.0002770.000312

0.0019340.0008340.00080.0017070.0004890.0018520.000565

1.7e-05

0.000108

0.0001120.000127

8.1e-057.7e-057.6e-05

4e-05

3e-056e-063.4e-05

0.0251590.0294920.0132390.0135540.006510.0163210.025043

0.0006950.0026450.0005010.0027110.0001610.0005280.002858

9.3e-05

8e-05

5.2e-051.5e-055.1e-058e-06

8.3e-050.0005013.9e-058.2e-050.000564

0.0004660.0009820.0005080.0019080.0003050.0004490.00078

4.5e-05

6e-05

0.0006780.0010860.0005980.0019080.0003360.000656

6e-062.1e-052e-065e-065.1e-05

0.0020920.0020640.002619

9e-060.0015150.0017072.9e-052.7e-05

0.000101

1.5e-050.0016040.0018079.5e-053.1e-05

5e-05

9e-062.1e-059e-06

6e-060.0016040.0018072.4e-052.2e-05

0.0390380.095260.0509780.0759050.0207750.0419440.087634

9.8e-050.0001049.1e-05

2.5e-053.9e-052.6e-05

7.3e-054e-056.5e-05

2.5e-05

0.038940.095260.0509780.0759050.0206710.0418530.087634

3e-053.4e-053e-05

0.0001660.000187

0.0016040.0006230.0005640.0055220.0001620.0016110.004234

4.3e-053.7e-050.0017820.0020081.7e-054.4e-05

5.4e-05

9.8e-05

2.5e-05

0.0051630.0098490.0031360.0062250.0011050.0049580.007371

9.3e-05

4.3e-056.2e-054.6e-05

8.3e-056e-050.0034750.0039164.8e-057.8e-051.6e-05

7.6e-05

0.0003110.0081170.0004640.0037150.0002370.0002990.003265

0.000104

2.7e-05

0.0013040.00147

0.0017360.001955

0.0008240.0010960.0007540.0019080.0004090.0007950.001411

0.0243680.0616660.0241010.0339360.013520.0233810.057136

8.8e-05

0.001960.0040690.0023820.0031120.0012940.0062550.004434

2.5e-05

0.0041430.0097060.0024240.0059240.0007660.0039870.007812

2.9e-050.0001112.4e-05

2.2e-053.7e-050.0046340.0052215e-061.9e-05

5.7e-056.2e-05

0.0001350.000152

9.5e-057.3e-059.2e-05

6.2e-050.0001096.7e-05

5e-065.2e-055e-06

6.6e-05

6e-05

3.1e-050.0024060.0027115.8e-052.9e-05

4e-050.0015150.0017073.3e-054.2e-05

2.7e-052.7e-052.9e-05

2.4e-05

4.5e-050.0002723.4e-05

4.5e-050.0002723.4e-05

6.2e-05

4.5e-053.8e-053.4e-05

6.1e-05

8.4e-05

1.9e-05

8e-06

0.0242150.0264970.0236010.0336330.0238480.0164360.041819

0.0016080.0015370.0017330.0022090.0035610.003090.001359

6e-066e-054e-06

6e-066e-054e-06

0.0016020.0015370.0017330.0022090.0035010.0030860.001359

0.0016020.0015370.0017330.0022090.0035010.0030860.001359

0.0002656e-050.0002620.0011870.00028

6.8e-05

6.8e-05

0.0002656e-050.0002620.0011190.00028

6e-06

1.4e-052.1e-051.6e-05

2.2e-051.1e-052.2e-05

0.0002116e-050.0002620.0010630.000223

1.8e-051.8e-051.9e-05

0.0166930.0220470.0112070.0257020.0091120.0075560.036713

0.000116

0.000116

0.000169

9.4e-05

7.5e-05

0.000165

9e-06

3.4e-05

0.000122

0.000102

0.000102

7.3e-050.0002147.7e-05

7.3e-054.9e-057.7e-05

0.000107

5.8e-05

0

0.0137760.0205770.009020.0205820.0050850.004660.036599

5.6e-05

2e-06

0

2.5e-059.2e-052.9e-05

0.0001410.0001481e-060.000203

3.7e-052.9e-054.4e-05

2.2e-050.0001173.5e-05

0.0002340.001970.0004990.0021081.9e-050.0003350.001552

0.0001575.5e-050.000170.0003020.000167

0.0002790.000314

1.2e-05

2.4e-05

0.0002220.00025

3e-06

0

0.0003030.000341

1.6e-05

0.0013270.0058820.000490.002610.0001130.0018360.006707

0.0006710.0001930.0004160.0027119.2e-050.000667

0.0098870.0045810.0015750.0035140.0004970.004987

0.0001770.000199

0.0001295.6e-050.0002220.0019081.5e-050.0001290.018927

5e-06

0.0002140.000241

9e-063e-061.7e-05

5.2e-050.0002140.0002426.1e-05

4.2e-059e-064.3e-05

0.0005290.000596

0.0003170.000358

6e-050.0013460.0016430.0023099e-066.2e-050.002252

2.1e-054.7e-052.6e-05

0.0002550.000288

0.0001066e-060.000107

4.7e-050.0001134.8e-05

7e-054.9e-057.9e-05

0.0002860.000323

1.1e-05

0.0004920.0064940.0009020.0054222.1e-050.0004790.002158

3.6e-051.8e-055.2e-05

9e-06

3.2e-05

8.4e-05

4e-055.4e-055.2e-05

0.0001710.0001591.7e-050.000189

7.2e-05

8e-067e-067e-06

8e-067e-067e-06

0.000112

0.000112

0.0010620.0003490.0007550.0018070.0016650.0010889.8e-05

0.0010620.0003490.0007550.0018070.0016650.0010889.8e-05

0.0001220.000137

0.0001220.000137

0.0015790.0010270.0010950.0018070.0010090.0015331.6e-05

2e-065e-064e-06

3.6e-055.5e-054.3e-05

3.6e-053e-053.6e-05

6e-066e-067e-06

2e-063e-062e-06

8e-06

2e-06

1e-05

1e-064e-061e-06

8e-061e-066e-06

6e-06

08e-06

0.0014880.0010270.0010950.0018070.0008640.001434

2.1e-05

2e-06

1.3e-050.0001161.6e-05

1.3e-050.0001161.6e-05

0.0001829.4e-050.0002150.0015060.0002150.000175

0.0001829.4e-050.0002150.0015060.0002150.000175

0.0005227e-050.004040.002610.0007060.0005530.001742

0.0004797e-050.004040.002610.0005040.0005180.001742

1e-05

2e-068e-060.0023170.002616e-061.8e-05

1.2e-05

0.000196.2e-050.0001770.0002130.000195

0.0015460.001742

4.1e-054e-064.3e-05

2e-06

4.7e-056e-064.5e-05

4.7e-051.1e-055e-05

3e-053.1e-053.3e-05

1.1e-05

6e-06

1.5e-059e-061.8e-05

1.2e-05

2.4e-051.3e-052.6e-05

4.1e-051.7e-054.4e-05

6e-06

4.2e-052.1e-054.6e-05

7.4e-05

4e-05

4.3e-059.2e-053.5e-05

4.3e-059.2e-053.5e-05

0.00011

0.00011

3.1e-055.8e-052.6e-05

3.1e-055.8e-052.6e-05

3.1e-055.8e-052.6e-05

4.7e-050.0015420.001455.3e-050.000718

0.0009780.0003840.000718

0.0003410.000384

0.0006370.000718

0.0002710.000306

0.0002710.000306

0.0001450.000164

0.0001450.000164

0.000353

5.1e-05

5.8e-05

2.9e-05

3.2e-05

2.7e-05

2.2e-05

5.1e-05

2.6e-05

5.7e-05

0.0001480.000167

0.0001480.000167

4.7e-057.6e-055.3e-05

4.7e-057.6e-055.3e-05

4.8e-051e-063.8e-055.9e-05

4.8e-051e-063.8e-055.9e-05

1e-061e-061e-061e-06

4e-066e-064e-06

2.1e-051.8e-052.5e-05

4e-064e-067e-06

1.8e-059e-062.2e-05

0.0050010.0027820.0048170.0031120.0077360.0048190.001287

1.7e-055.3e-051.7e-05

1.7e-055.3e-051.7e-05

0.0049580.0027820.0033230.0031120.004930.0047793.3e-05

2.4e-05

5.5e-05

6.5e-05

3.5e-05

7.1e-056.7e-051.9e-053.7e-053.3e-05

0.0046670.0026660.0031910.0031120.0038870.004486

1.2e-05

7.4e-05

6e-062.8e-055e-06

6e-06

6.8e-05

1.2e-05

1.9e-05

1.8e-057.8e-051.7e-05

5.1e-05

2.4e-05

0.0001664.9e-050.0001320.0001080.000206

1.4e-05

8.8e-05

9e-06

1e-05

1.6e-05

0.000102

3e-056.7e-052.8e-05

5e-06

2.4e-05

2.2e-05

8e-06

0.000112

0.000112

2.6e-050.0014940.0026412.3e-050.001254

0.0014940.0025580.001254

2.6e-058.3e-052.3e-05

6.8e-05

6.8e-05

6.8e-05

6.8e-05

6.8e-05

0.0002270.0080570.0019250.0002240.00711

0.0002270.0080570.0019250.0002240.00711

0.0002270.0080570.0019250.0002240.00711

1.6e-05

1.6e-05

8e-067.3e-057e-06

8e-061.4e-057e-06

5.9e-05

8.6e-05

8.6e-05

0.0002190.0001940.000217

0.0002190.0001940.000217

0.0077280.0016130.007094

0.0062960.007094

0.0014320.001613

0.0001350.000153

0.0001350.000153

0.0069860.0040920.0460880.0047190.0531890.0071960.002835

0.006950.0040920.0460880.0047190.0530490.0071610.002835

3.4e-05

3.4e-05

3.4e-05

3.4e-05

0.0021030.003550.0015460.0047190.0025540.0020420.00282

0.000950.0031990.0011020.0030120.0006330.0009110.002717

0.000950.0031990.0011020.0030120.0006330.0009110.002717

3.5e-05

0.000950.0031990.0011020.0030120.0005980.0009110.002717

0.0002950.0007380.000296

0.0001870.0005240.000201

4e-06

1.4e-051.8e-051.2e-05

2e-06

1.4e-056.7e-051.1e-05

1.9e-051e-062.1e-05

1.3e-051.7e-051.3e-05

0

2.1e-05

2e-0502.3e-05

4e-063.4e-056e-06

7e-06

2e-058e-052.3e-05

1.7e-05

2e-05

5e-061e-054e-06

3e-06

1.9e-058.3e-052.1e-05

4e-06

2.6e-050.0001083.1e-05

1e-05

7e-061.1e-059e-06

2.6e-057e-062.7e-05

2.8e-057.7e-052.9e-05

2.8e-057.7e-052.9e-05

5e-058.6e-054.9e-05

3.6e-058.6e-053.4e-05

1.4e-051.5e-05

3e-055.1e-051.7e-05

3e-055.1e-051.7e-05

0.0007610.0003510.0003120.0017070.0007840.0007450.000103

0.0007610.0003510.0003120.0017070.0007840.0007450.000103

0.0007610.0003510.0003120.0017070.0007840.0007450.000103

2e-050.0001320.000311.7e-05

2e-050.0001320.000311.7e-05

2e-055e-051.7e-05

0.0001320.000149

0.000111

7e-061.5e-057e-06

7e-061.5e-057e-06

7e-069e-067e-06

4e-06

2e-06

1.7e-057.4e-051.4e-05

1.7e-057.4e-051.4e-05

1.7e-057.4e-051.4e-05

5.3e-055.2e-05

5.3e-055.2e-05

5.3e-055.2e-05

0.0002150.0001160.000310.000225

0.00010.0001160.0001690.000107

0.00010.0001160.0001690.000107

0.00010.0001160.0001690.000107

0.0001150.0001410.000118

0.0001150.0001410.000118

0.0001150.0001410.000118

0.0004860.0005670.0012560.000529

0.0001350.0003650.000156

0.0001350.0003650.000156

1.8e-050.0001182.3e-05

3.1e-058e-053.4e-05

8e-065e-061e-05

4.5e-056.1e-054.8e-05

4e-061.2e-055e-06

2.9e-058.9e-053.6e-05

0.0001120.0001710.0002480.000118

2.3e-055.5e-052.5e-05

2.3e-055.5e-052.5e-05

5.8e-050.0001710.0001936e-05

5.8e-050.0001710.0001936e-05

3.1e-053.3e-05

3.1e-053.3e-05

0.0001230.0001390.0002490.000143

0.0001230.0001390.0002490.000143

0.0001230.0001390.0002490.000143

9.3e-050.0001260.0002469.2e-05

9.3e-050.0001260.0002469.2e-05

5.6e-050.0001260.0001435.5e-05

3.7e-050.0001033.7e-05

2.3e-050.0001310.0001482e-05

2.3e-050.0001310.0001482e-05

2.3e-050.0001310.0001482e-05

0.0037510.0005420.0435420.0475670.0039561.5e-05

0.0028630.0005040.0022220.0012140.003011.5e-05

0.0006970.0001560.0005740.0003330.000619

0.0003326.4e-050.0002390.0001860.000365

8.7e-05

0.0001379.2e-050.0001220

0.0002280.0002136e-050.000254

0.0003593.9e-050.0003370.000404

0.0003593.9e-050.0003370.000404

0.0016010.0003090.0011190.0008170.0017591.5e-05

0.0001290.000146

0.0003296.4e-050.0002030.0001350.000377

0.0004549.6e-050.0002930.0002050.000501

0.0004016.2e-050.0002540.0001780.0004241.5e-05

0.0004178.7e-050.000240.0001530.000457

0.0002060.0001926.4e-050.000228

0.0002060.0001926.4e-050.000228

0.0008883.8e-050.0008080.0007090.000946

0.000653.8e-050.0006270.0005560.000697

0.0002150.0001770.0001680.000227

0.0004353.8e-050.0002780.0001940.00047

0.0001720.000194

0.0002380.0001810.0001530.000249

0.0002380.0001810.0001530.000249

0.0405120.045644

0.0405120.045644

0.0405120.045644

0.0002470.0001420.0008520.000253

6.4e-050.0001176.2e-05

2.9e-057.1e-053.4e-05

2.9e-057.1e-053.4e-05

3.5e-054.6e-052.8e-05

3.5e-054.6e-052.8e-05

4.3e-052.6e-054.6e-05

4.3e-052.6e-054.6e-05

4.3e-051.5e-054.6e-05

1.1e-05

2.3e-055.1e-052.6e-05

2.3e-055.1e-052.6e-05

2.3e-055.1e-052.6e-05

0.0001170.0001420.0006580.000119

8.6e-05

4.3e-05

4.3e-05

0.0001170.0001420.0005720.000119

0.0001170.0001420.0005720.000119

0.0001480.0001750.0004760.000156

0.0001480.0001750.0004760.000156

0.0001480.0001750.0004760.000156

0.0001480.0001750.0004760.000156

3.6e-050.000143.5e-05

3.6e-050.000143.5e-05

3.6e-050.000143.5e-05

3.6e-050.000143.5e-05

1.9e-057.4e-051.9e-05

1.7e-056.6e-051.6e-05

8.1e-050.0003070.0007037.1e-05

3.1e-053.8e-052.4e-05

3.1e-053.8e-052.4e-05

3.1e-053.8e-052.4e-05

3.1e-053.8e-052.4e-05

3.1e-053.8e-052.4e-05

9.3e-05

9.3e-05

9.3e-05

9.3e-05

9.3e-05

3e-050.0003070.0003463e-05

3e-050.0003070.0003463e-05

3e-050.0003070.0003463e-05

3e-050.0003070.0003463e-05

3e-050.000150.0001693e-05

0.0001570.000177

3.1e-05

3.1e-05

3.1e-05

3.1e-05

3.1e-05

2e-050.0001951.7e-05

7e-065.3e-056e-06

7e-065.3e-056e-06

7e-065.3e-056e-06

7e-065.3e-056e-06

1.3e-050.0001421.1e-05

1.3e-050.0001421.1e-05

3.6e-05

3.6e-05

1.3e-055.5e-051.1e-05

1.3e-055.5e-051.1e-05

5.1e-05

5.1e-05

0.0021420.0116610.0033120.052610.0022680.002010.067467

0.0021420.0116610.0033120.052610.0022680.002010.067467

2e-050.0001241.6e-05

1.1e-054.8e-051e-05

1e-052.2e-051e-05

1e-051e-05

2.2e-05

1e-062.6e-050

1e-062.6e-050

6e-063.5e-055e-06

6e-063.5e-055e-06

6e-063.5e-055e-06

3e-064.1e-051e-06

3e-064.1e-051e-06

3e-064.1e-051e-06

0.0021220.0116610.0033120.052610.0021440.0019940.067467

0.0021220.0116610.0033120.052610.0021440.0019940.067467

0.0021220.0116610.0033120.052610.0021440.0019940.067467

0.0019210.0107560.0030860.0506020.0019180.0017980.066323

01.2e-050

2e-061e-052.2e-05

04e-060

0.0001770.0009050.0002260.0020080.0001130.0001510.001144

2.7e-05

3e-062.4e-053e-06

1e-063e-061e-06

2.4e-05

1.8e-059e-061.9e-05

0.002150.0020560.00251

0.002150.0020560.00251

0.002150.0020560.00251

0.002150.0020560.00251

0.002150.0020560.00251

0.002150.0020560.00251

0.0200810.0144530.0320960.0240950.0373270.0196030.013057

0.0041110.0025160.0041330.002610.0058720.0039470.001889

0.0041110.0025160.0041330.002610.0058720.0039470.001889

0.0041110.0025160.0041330.002610.0058720.0039470.001889

1.7e-056.9e-051.8e-05

1.7e-056.9e-051.8e-05

0.0040940.0025160.0025780.002610.0038890.0039290.001889

0.0040940.0025160.0025780.002610.0038890.0039290.001889

6.9e-05

6.9e-05

9.3e-05

9.3e-05

0.0015550.001752

0.0007480.000843

0.0008070.000909

1.1e-059.4e-051.4e-05

1.1e-059.4e-051.4e-05

4e-065.5e-054e-06

4e-065.5e-054e-06

4e-065.5e-054e-06

7e-063.9e-051e-05

7e-063.9e-051e-05

7e-063.9e-051e-05

2.6e-050.0001221.8e-05

2.6e-050.0001221.8e-05

2.6e-050.0001221.8e-05

1.9e-058.1e-051.3e-05

1.9e-058.1e-051.3e-05

7e-064.1e-055e-06

7e-064.1e-055e-06

0.0070840.0073140.0140290.0069270.0149180.0069740.007289

0.0070840.0073140.0140290.0069270.0149180.0069740.007289

0.0001

0.0001

0.0001

0.0001630.0001990.00016

0.0001630.0001990.00016

2.4e-053.8e-052e-05

1.3e-052.9e-051.4e-05

3.3e-054.4e-052.6e-05

3.3e-051.7e-053.3e-05

7e-069e-066e-06

8e-061.9e-058e-06

3.5e-052.9e-053.7e-05

1e-051.4e-051.6e-05

0.0068860.0073140.0140290.0069270.0144860.0067790.007289

3.2e-050.0001350.0001523.7e-05

3.2e-050.0001350.0001523.7e-05

6.1e-050.0001116.7e-05

6.1e-050.0001116.7e-05

0.0060480.0068149.5e-05

9.5e-05

0.0007790.000878

0.0007630.00086

0.0030120.003393

0.0014940.001683

0.0001190.000134

0.0001190.000134

1.9e-050.0002310.0002612.1e-05

1.9e-050.0001170.0001322.1e-05

0.0001140.000129

0.0020320.0053470.0020670.0046180.0010620.0019530.004387

0.0020320.0053470.0020670.0046180.0010620.0019530.004387

0.000122

0.000122

8.1e-05

8.1e-05

0.000201

0.000106

9.5e-05

0.0012780.00144

0.0012780.00144

2.1e-057.2e-052e-05

2.1e-057.2e-052e-05

2.1e-059.2e-051.9e-05

2.1e-059.2e-051.9e-05

3.5e-055.5e-054e-05

3.5e-055.5e-054e-05

1.7e-058e-052e-05

1.7e-058e-052e-05

0.0003030.0004080.000341

7.5e-054.3e-058.2e-05

4.3e-058.9e-054.9e-05

4.4e-059.9e-054.8e-05

9.5e-050.0001450.000106

4.6e-053.2e-055.6e-05

0.0037860.0019670.0019390.0023090.0013720.0036670.001367

3.2e-05

1.6e-05

0.0001190.000134

0.0037490.0019670.0013160.0023090.0005540.0036260.001319

3.7e-050.0001164.1e-05

0.0005040.000568

0.000114

0.000114

8.8e-05

8.8e-05

0.0001190.000134

0.0001190.000134

4.2e-050.0002580.0002914.8e-05

4.2e-050.0002580.0002914.8e-05

6e-068.9e-058e-06

6e-068.9e-058e-06

2.6e-050.0001230.0001382.8e-05

2.6e-050.0001230.0001382.8e-05

3.3e-050.0001570.0001763.4e-05

3.3e-050.0001570.0001763.4e-05

7.7e-050.0001520.0001717.8e-05

7.7e-050.0001520.0001717.8e-05

0.000108

2e-05

8.8e-05

1.4e-050.0001291.7e-05

1.4e-056.1e-051.7e-05

6.8e-05

0.0003310.000458

0.0002190.000246

8.6e-05

0.0001120.000126

4.1e-050.0001440.0001624.1e-05

4.1e-050.0001440.0001624.1e-05

0.0007250.000817

0.0007250.000817

9.2e-050.0001170.000102

5.1e-055.2e-055.7e-05

2.8e-05

4.1e-053.7e-054.5e-05

2.8e-050.0001772.8e-05

9e-06

2.8e-055.8e-052.8e-05

0.00011

0.00020.0002030.00030.00021

0.00020.0002030.00030.00021

2e-056.5e-051.9e-05

2e-056.5e-051.9e-05

2e-056.5e-051.9e-05

1.5e-056.8e-051.6e-05

1.5e-056.8e-051.6e-05

1.5e-056.8e-051.6e-05

0.0053610.0026930.0108990.0122490.010620.0052760.003879

0.0001240.000237

9.7e-05

9.7e-05

9.7e-05

0.0001240.00014

0.0001240.00014

0.0001240.00014

0.0053610.0026930.0107750.0122490.0103830.0052760.003879

0.0011940.0003550.0014480.0036150.0030840.0012150.000902

0.0011940.0003550.0014480.0036150.0030840.0012150.000902

0.0001820.0002340.0015310.000191

0.0001017.5e-050.0017070.0001110.00010.000396

0.0001250.000141

1.6e-05

0.0001550.000174

0.0001020.0001390.000105

2.1e-05

5.3e-05

0.0002240.000253

0.0007720.000280.0005140.0019080.0005140.0007767.1e-05

3.7e-050.0001960.0002214.3e-05

8.3e-05

8.7e-05

9.1e-05

2.1e-05

4.7e-05

1.6e-05

1.3e-050.0010790.0012161.4e-05

1.3e-050.0001230.0001391.4e-05

1.3e-050.0001230.0001391.4e-05

0.0009560.001077

0.0009560.001077

1.3e-050.0002090.0002351.4e-05

1.3e-050.0002090.0002351.4e-05

1.3e-050.0002090.0002351.4e-05

0.0003740.0005570.0006790.0017070.0005680.000360.000445

0.0003740.0005570.0004830.0017070.0003470.000360.000445

2.7e-050.000160.000182.6e-05

0.0003470.0005570.0003230.0017070.0001670.0003340.000445

0.0001960.000221

0.0001960.000221

4.5e-050.0002010.0002264.5e-05

4.5e-050.0002010.0002264.5e-05

4.5e-050.0002010.0002264.5e-05

0.0030960.0017030.0038360.0053210.0015310.0029760.002

0.0002080.000234

0.0002080.000234

0.0030960.0017030.0036280.0053210.0012970.0029760.002

6.2e-050.0015570.0027110.0012976.2e-05

0.0030340.0017030.0020710.002610.0029140.002

0.0003050.000344

0.0003050.000344

0.0003050.000344

0.0001110.0011180.0013270.0001131.6e-05

7.7e-050.0009950.0011218.3e-051.6e-05

0.0002270.000256

1.6e-05

7.7e-050.0007680.0008658.3e-05

3.4e-050.0001230.0002063e-05

3.4e-056.8e-053e-05

0.0001230.000138

0.0005157.8e-050.00190.0016060.0018520.0005390.000516

0.0005157.8e-050.00190.0016060.0018520.0005390.000516

6.5e-059.2e-056.3e-05

0.0002620.000295

0.0001110.000125

5.1e-050.000190.0002145.5e-05

7.3e-050.0002470.0002787.7e-05

4.7e-050.0004240.0004785.3e-05

0.0002920.000329

9.1e-050.0001450.0001649.8e-05

0.0001887.8e-050.0002290.0016060.0002270.0001936.2e-05

0.000104

4.5e-050.0006264.9e-05

4.5e-050.0006264.9e-05

4.5e-050.0006264.9e-05

1.1e-057.5e-051.1e-05

1.1e-057.5e-051.1e-05

1e-050.0001771.1e-05

1e-058.3e-051.1e-05

9.4e-05

8e-060.0002031e-05

4.7e-05

4e-05

8e-067e-051e-05

4.6e-05

1.6e-050.0001061.7e-05

1.6e-050.0001061.7e-05

6.5e-05

6.5e-05

0.0034430.001930.0030350.0023090.0050750.003325

0.0034430.001930.0030350.0023090.0050750.003325

1.4e-053.9e-051.6e-05

1.4e-053.9e-051.6e-05

1.4e-053.9e-051.6e-05

5e-05

5e-05

5e-05

1.6e-050.0001582e-05

1.6e-055.8e-052e-05

1.6e-055.8e-052e-05

4.9e-05

4.9e-05

5.1e-05

5.1e-05

0.000343

7.7e-05

4e-05

3.7e-05

0.000185

2.3e-05

1.1e-05

3.5e-05

3.3e-05

3.8e-05

1.3e-05

3.2e-05

8.1e-05

2.9e-05

2.8e-05

2.4e-05

1.6e-050.0001311.1e-05

6e-066.7e-055e-06

6e-066.7e-055e-06

1e-056.4e-056e-06

1e-056.4e-056e-06

0.003290.001930.0025230.0023090.003620.003164

1.7e-058.7e-051.9e-05

1.7e-058.7e-051.9e-05

1.3e-057.4e-051.3e-05

1.3e-057.4e-051.3e-05

2.3e-055.7e-052.2e-05

2.3e-055.7e-052.2e-05

8e-069.4e-058e-06

8e-069.4e-058e-06

0.0002040.00023

0.0002040.00023

0.00320.001930.0023190.0023090.0030780.003074

0.00320.001930.0023190.0023090.0030780.003074

2.9e-052.8e-05

2.9e-052.8e-05

0.0001070.0005120.0007340.000114

4.8e-05

4.8e-05

9e-050.0005120.0005779.4e-05

9e-050.0001550.0001759.4e-05

0.0003570.000402

7e-065.9e-059e-06

7e-065.9e-059e-06

1e-055e-051.1e-05

1e-055e-051.1e-05

3e-055e-052.3e-05

3e-055e-052.3e-05

3e-055e-052.3e-05

3e-055e-052.3e-05

3e-055e-052.3e-05

3e-055e-052.3e-05

0.0001090.0003528.6e-05

0.0001090.0003528.6e-05

0.0001090.0003528.6e-05

0.0001090.0003528.6e-05

9e-068.1e-058e-06

9e-063.3e-058e-06

4.8e-05

1.4e-054.7e-051.6e-05

4e-062.7e-051e-05

1e-052e-056e-06

4.5e-050.0001093.1e-05

4e-063.3e-058e-06

8e-062.8e-056e-06

1.5e-052.5e-058e-06

1.8e-052.3e-059e-06

4e-066.2e-055e-06

4e-066.2e-055e-06

3.7e-055.3e-052.6e-05

1.5e-052.2e-051.1e-05

1.3e-05

2.2e-051.8e-051.5e-05

0.0073040.0067570.0050890.0091360.0065930.0070710.005621

6.2e-05

6.2e-05

6.2e-05

6.2e-05

6.2e-05

0.0073040.0067570.0050890.0091360.0065310.0070710.005621

0.0036170.0047160.0028830.0067260.0028890.0035470.004058

0.0036170.0047160.0028830.0067260.0028890.0035470.004058

0.0027220.0016420.001940.0021080.0020330.0027490.001495

7.7e-050.0001889.5e-050.000212

0.0026450.0016420.0017520.0021080.0019380.0025370.001495

0.0008950.0030740.0009430.0046180.0008560.0007980.002563

01.3e-05

4e-06

1.3e-05

2e-062e-062e-06

01.5e-05

0.0002580.0008460.0003420.0018070.0002150.0002470.000783

2.2e-051.3e-051.8e-05

9e-062.3e-057e-06

1e-066e-061e-06

3e-06

1e-062e-061e-06

07e-060

2e-05

4e-06

1.3e-051.3e-053e-06

3e-062e-06

8e-061e-051.1e-05

02e-061e-06

3e-056e-063.2e-05

1e-061.3e-052e-06

4e-062e-06

5e-06

7e-061e-057e-06

2.6e-054e-062.3e-05

2.1e-05

01e-065e-06

3.1e-053e-061.6e-05

3e-064e-063e-06

8e-063e-068e-06

6e-061e-065e-06

3e-061e-062e-06

7e-06

01e-060

2.7e-051.3e-052.2e-05

1e-062e-060

0.0004310.0022280.0006010.0028110.0003960.0003780.00178

5e-050.0001075e-05

4.6e-059.3e-054.6e-05

4.6e-059.3e-054.6e-05

1.7e-056e-061.9e-05

8e-068e-065e-06

9e-069e-061e-05

8e-06

1e-05

8e-06

3e-06

1e-061.3e-051e-06

1.3e-05

1.2e-05

1.1e-053e-061.1e-05

4e-061.4e-054e-06

4e-061.4e-054e-06

4e-061.4e-054e-06

0.0036370.0020410.0022060.002410.0035350.0034740.001563

0.0036370.0020410.0022060.002410.0035350.0034740.001563

0.0035980.0020410.0022060.002410.0034920.0034420.001563

2.5e-054.2e-051.3e-05

1e-054.5e-059e-06

3.3e-05

0.0035630.0020410.0022060.002410.0033720.003420.001563

3.9e-054.3e-053.2e-05

3e-068e-062e-06

3.1e-052.3e-052.7e-05

3e-061.2e-050

0

2e-0603e-06

7.5e-050.0002716.3e-05

6.3e-050.0001735.5e-05

6.3e-050.0001735.5e-05

6.3e-050.0001735.5e-05

9e-063.4e-056e-06

9e-063.4e-056e-06

2e-055.2e-051.9e-05

8e-062.5e-058e-06

1.2e-052.7e-051.1e-05

1.3e-053.4e-051e-05

1.3e-053.4e-051e-05

2.1e-055.3e-052e-05

1.1e-052.8e-058e-06

1e-052.5e-051.2e-05

6e-066.2e-053e-06

6e-066.2e-053e-06

6e-066.2e-053e-06

6e-066.2e-053e-06

6e-066.2e-053e-06

6e-063.6e-055e-06

6e-063.6e-055e-06

6e-063.6e-055e-06

6e-063.6e-055e-06

6e-063.6e-055e-06

6.6e-050.0003263.5e-05

6.6e-050.0003263.5e-05

3.3e-056.4e-051.6e-05

3.3e-056.4e-051.6e-05

3.2e-054.2e-051.5e-05

3.2e-054.2e-051.5e-05

1e-062.2e-051e-06

1e-062.2e-051e-06

3.3e-050.0002621.9e-05

6.5e-05

6.5e-05

6.5e-05

1.8e-057e-051e-05

9e-062.8e-053e-06

9e-062.8e-053e-06

9e-064.2e-057e-06

4e-062.4e-053e-06

5e-061.8e-054e-06

1.5e-050.0001279e-06

1e-061.1e-051e-06

1e-061.1e-051e-06

1e-066e-050

1e-063.5e-050

2.5e-05

1e-055e-067e-06

1e-060

9e-065e-067e-06

3e-065.1e-051e-06

3e-065.1e-051e-06

0.0033640.0020870.0025630.0023090.0035550.003229

0.0033640.0020870.0025630.0023090.0035550.003229

0.0033640.0020870.0025630.0023090.0035550.003229

4e-060.0001130.0001273e-06

4e-060.0001130.0001273e-06

4e-060.0001130.0001273e-06

0.003360.0020870.002450.0023090.0034280.003226

0.003360.0020870.002450.0023090.0034280.003226

0.003360.0020870.002450.0023090.0034280.003226

0.2873390.2375810.1783570.227510.2413640.2879820.209421

0.0001990.0006110.000155

9.8e-050.0003077.4e-05

1.7e-050.0001291.2e-05

4.9e-05

4.9e-05

1.7e-058e-051.2e-05

1.7e-058e-051.2e-05

8.1e-050.0001786.2e-05

5.4e-056.5e-053.8e-05

5.4e-056.5e-053.8e-05

2.7e-050.0001132.4e-05

2.7e-055.7e-052.4e-05

5.6e-05

0.0001010.0003048.1e-05

0.0001010.0003048.1e-05

3.4e-050.0001042.6e-05

2.1e-054.6e-051.6e-05

1.3e-055.8e-051e-05

1e-053.5e-054e-06

1e-053.5e-054e-06

2.5e-054.2e-052.4e-05

2.5e-054.2e-052.4e-05

1.6e-057.2e-051.6e-05

1.6e-057.2e-051.6e-05

5.1e-05

5.1e-05

1.6e-051.1e-05

1.6e-051.1e-05

0.2867470.2375470.1780570.227510.2400190.2874670.209404

0.0002810.000420.0002631.6e-05

0.0001620.0001470.000153

3.9e-051.8e-053.9e-05

3.9e-051.8e-053.9e-05

3.7e-055e-053e-05

3.7e-055e-053e-05

3.5e-052.8e-053.7e-05

3.5e-051.9e-053.7e-05

9e-06

2.6e-051.3e-052.6e-05

2e-062e-062e-06

2.4e-051.1e-052.4e-05

2.5e-053.8e-052.1e-05

2.5e-053.8e-052.1e-05

0.0001190.0002730.000111.6e-05

5.8e-055.7e-055.4e-051.6e-05

5.8e-055.7e-055.4e-051.6e-05

6.1e-050.0001015.6e-05

6.1e-055.5e-055.6e-05

4.6e-05

5.6e-05

5.6e-05

5.9e-05

5.9e-05

4.4e-055.4e-054e-05

4.4e-055.4e-054e-05

4.4e-055.4e-054e-05

4.4e-055.4e-054e-05

0.0125760.0510820.013640.0956830.006670.0163770.052873

0.0125760.0510820.013640.0956830.006670.0163770.052873

7.3e-05

7.3e-05

0.0108350.0493090.0116780.0932730.004640.0146990.050767

2.8e-054.6e-053e-05

1.5e-051.3e-051.5e-05

1.8e-05

1.1e-05

2.7e-051.1e-052.5e-05

2.6e-053.1e-053e-05

0.0004610.0005520.0003750.0520080.0001790.000441

0.0100790.0485840.0111140.0412650.0041040.0139620.050407

0.0001970.0001730.0001898.9e-050.0001930.00036

4e-06

3.2e-05

2e-063e-063e-06

1.2e-05

2e-06

2.3e-05

2e-06

6e-05

0.0017410.0017730.001490.002410.0011960.0016780.001697

0.0017410.0017730.001490.002410.0011960.0016780.001697

0.0004720.0007610.000409

0.0004720.0007610.000409

0.0003480.0001070.0001570.0015060.0004470.0003440.000149

0.0003480.0001070.0001570.0015060.0004470.0003440.000149

0.0003480.0001070.0001570.0015060.0004470.0003440.000149

4.1e-056.6e-054.3e-05

2.7e-056.6e-052.9e-054.5e-05

0.0002140.0001070.0001570.0015060.0002110.0002070.000104

4.2e-055.6e-054.1e-05

2.4e-054.8e-052.4e-05

4.4e-056.2e-054.1e-05

4.4e-056.2e-054.1e-05

4.4e-056.2e-054.1e-05

4.4e-056.2e-054.1e-05

0.016830.0098230.0097690.007430.016280.0161330.008011

0.016830.0098230.0097690.007430.016280.0161330.008011

0.000144

4e-05

3.5e-05

3.6e-05

3.3e-05

0.0168080.0098230.0097690.007430.0160320.0161210.008011

0.0168080.0098230.0097690.007430.0160320.0161210.008011

2.2e-054.4e-051.2e-05

2.2e-054.4e-051.2e-05

1.2e-05

1.2e-05

4.8e-05

4.8e-05

6.4e-057.4e-055.8e-055e-06

6.4e-057.4e-055.8e-055e-06

6.4e-057.4e-055.8e-055e-06

6.4e-057.4e-055.8e-055e-06

0.0001570.0001570.0003070.000151.6e-05

0.0001570.0001570.0003070.000151.6e-05

5.5e-05

5.5e-05

0.0001570.0001570.0002520.000151.6e-05

0.0001570.0001570.0002520.000151.6e-05

0.0037650.0043010.0028520.0047190.0028410.0036210.002555

0.0037650.0043010.0028520.0047190.0028410.0036210.002555

9.4e-050.0001998.9e-051.5e-05

9.4e-058.4e-058.9e-051.5e-05

4.8e-05

1e-06

3.3e-05

0

3e-05

3e-06

7.3e-056.9e-056.9e-051.8e-05

7.3e-056.9e-056.9e-051.8e-05

0.0001370.0001430.0002610.0001334.6e-05

0.0001370.0001430.0002610.0001334.6e-05

7.7e-056e-057.5e-05

7.7e-056e-057.5e-05

6.9e-05

6.9e-05

0.0032910.0043010.0027090.0047190.0019870.0031650.002445

0.0018750.0010330.0009430.0019080.000470.001804

3.9e-05

0.0014160.0032680.001630.0028110.0013250.0013610.002445

0.0001360.000153

6.1e-05

6.1e-05

5.9e-05

5.9e-05

7.6e-05

7.6e-05

9.3e-059e-053.1e-05

9.3e-059e-053.1e-05

0.0069760.0037250.0049940.0072280.0075620.0066510.003726

0.0031690.001760.0019370.0021080.0028940.0030360.001503

8.4e-057.1e-057.8e-053e-05

8.4e-057.1e-057.8e-053e-05

0.0030850.001760.0019370.0021080.0028230.0029580.001473

0.0030850.001760.0019370.0021080.0027350.0029580.001473

8.8e-05

5.7e-058.6e-055.2e-05

5.7e-055.1e-055.2e-05

5.7e-055.1e-055.2e-05

3.5e-05

3.5e-05

6.9e-050.0001516.4e-056.8e-05

6.9e-054.9e-056.4e-05

6.9e-054.9e-056.4e-05

5.1e-05

5.1e-05

6.8e-05

6.8e-05

5.1e-05

5.1e-05

1.3e-054.3e-059e-06

1.3e-054.3e-059e-06

1.3e-054.3e-059e-06

2e-061.2e-051e-06

2e-061.2e-051e-06

2e-061.2e-051e-06

0.0004750.0003110.0002670.0016060.0008660.0004570.000215

5.3e-05

5.3e-05

3.4e-05

3.4e-05

4.6e-05

4.6e-05

4.5e-052.3e-054.3e-05

4.5e-052.3e-054.3e-05

4.6e-05

4.6e-05

4.2e-05

4.2e-05

8.7e-050.000358.6e-056e-06

2.8e-05

4.7e-05

8.7e-057.9e-058.6e-056e-06

7.4e-05

8e-05

4.2e-05

3.3e-05

3.3e-05

5.2e-05

1.2e-05

4e-05

0.0003430.0003110.0002670.0016060.0001870.0003280.000209

0.0003430.0003110.0002670.0016060.0001870.0003280.000209

7.4e-056.2e-057.2e-051.5e-05

7.4e-056.2e-057.2e-051.5e-05

7.4e-056.2e-057.2e-051.5e-05

5.4e-050.0001644.9e-053.3e-05

5.4e-055.7e-054.9e-053.3e-05

5.4e-055.7e-054.9e-053.3e-05

5.3e-05

5.3e-05

5.4e-05

5.4e-05

0.0011940.0004450.000931

0.0011940.0004450.000931

0.0003950.0004453.1e-05

0.0007990.0009

5.9e-057.1e-055.6e-05

5.9e-057.1e-055.6e-05

5.9e-057.1e-055.6e-05

0.0002310.0001890.0002242e-05

0.0002310.0001890.0002242e-05

8.5e-057.1e-058.2e-05

7.6e-056.5e-057.2e-051.5e-05

7e-055.3e-057e-055e-06

0.0027730.0016540.0015960.0035140.0025790.0026310.000941

3.7e-052.2e-053.1e-05

3.7e-052.2e-053.1e-05

5.8e-050.0001584.9e-05

6e-05

5.1e-05

5.8e-054.7e-054.9e-05

5.8e-05

5.8e-05

5e-068e-065e-06

5e-068e-065e-06

7.3e-055.8e-057e-05

7.3e-055.8e-057e-05

8.5e-058.1e-057.3e-05

6.3e-055.8e-055.4e-05

2.2e-052.3e-051.9e-05

0.0009390.0005580.0006340.0017070.000940.0008980.000405

0.0009360.0005580.0006340.0017070.0007320.0008950.000405

6e-06

3e-066e-063e-06

1.8e-05

1.9e-05

2.9e-05

6e-06

2.4e-05

5.7e-05

2.1e-05

2.2e-05

6.6e-057.6e-055.7e-05

3.6e-052.4e-053e-05

3e-052.5e-052.7e-05

2.7e-05

0.001510.0010960.0009620.0018070.0011780.0014480.000536

0.001510.0010960.0009620.0018070.0011780.0014480.000536

5.1e-056.3e-054.4e-05

5.1e-056.3e-054.4e-05

5.1e-056.3e-054.4e-05

5.1e-056.3e-054.4e-05

0.0055710.0029860.0043840.002610.0066310.0054230.002907

0.0055710.0029860.0043840.002610.0066310.0054230.002907

0.0055020.0029860.0043840.002610.0065690.0053570.002907

4.9e-05

2.5e-05

5.8e-052.1e-052.1e-056.3e-05

6.2e-056.7e-05

1.6e-05

1.8e-05

0.0001012.4e-051.8e-050.000109

3.7e-05

0.0001043.1e-052e-050.00011

1.7e-05

5e-061e-055e-06

5e-065e-066e-06

8.4e-052e-059e-069e-051e-06

4.4e-05

3.4e-05

1.8e-05

0.0004390.0006840.000387

4.6e-05

5e-06

7.3e-054.1e-057.9e-05

0.0046020.0028170.002730.002610.0036470.0044250.002021

2.8e-05

3.5e-05

4.6e-05

4e-05

2.4e-05

5.7e-05

1.5e-05

3e-05

1.9e-05

0.0003480.000392

3.4e-053e-052.2e-057.3e-051e-06

3.8e-05

6.7e-056.7e-057e-051e-05

3.1e-05

3.1e-05

5e-061e-06

6e-05

2.6e-05

6.7e-052e-052.9e-057e-05

4.5e-052.3e-053.2e-054.7e-05

7.5e-05

0.0008670.000977

3.5e-05

2.5e-05

6.4e-052.4e-057.1e-05

2.8e-05

6.3e-054e-057.2e-05

2.2e-05

6.8e-053.5e-050

3e-06

6.9e-056.2e-056.6e-05

6.9e-056.2e-056.6e-05

0.0290330.0412280.0276820.0302220.0166370.0357980.041044

0.0001190.0001160.000112

0.0001190.0001160.000112

0.0001190.0001160.000112

0.0215110.030540.0084950.0175710.0049470.0283220.022727

0.0214680.030540.008370.0175710.0048060.0282860.022727

4e-06

4.4e-05

4e-06

6.2e-052.5e-056.6e-051.5e-05

0

5e-067e-063e-06

6.9e-050

2e-064e-062e-06

1.2e-05

7e-061e-066e-06

4.7e-056e-064.6e-051e-06

2.9e-051.7e-053.3e-05

6.4e-054.9e-057.1e-051.5e-05

4.4e-05

3e-064.1e-051e-062e-06

3e-062e-063e-06

2.6e-05

3.5e-05

0.0155850.0244170.0045580.0121490.001140.0195410.019031

1.1e-050.0001086e-061.1e-055e-06

5e-069e-066e-063e-05

3.5e-05

00

4e-05

5e-066e-065e-06

0008.3e-05

2e-05

3.1e-05

0.0024040.0039220.0016710.0032130.0005770.0054360.003486

3.2e-05

5.6e-05

0.00011305e-06

00.000107005e-06

4.9e-053e-055.4e-05

0.0030340.0018320.0021410.0022090.002490.002916

3e-062e-063e-06

4.2e-052.1e-054.2e-051.6e-05

0

3.2e-05

3.9e-053.3e-054e-05

4.3e-050.0001250.0001413.6e-05

4.3e-050.0001250.0001413.6e-05

0.0003610.0008870.0020080.0009570.0003528.2e-05

0.0003610.0008870.0020080.0009570.0003528.2e-05

5e-05

0.0005120.000577

1.9e-05

3.3e-05

0.0001180.0001510.0020084.5e-050.000115

0.0001160.0001110.0001510.0001153e-05

0.0001270.0001130.0001340.000122

0.0031230.0020850.0022940.0022090.0022690.0030980.001506

0.0007470.000180.0005690.0009270.0007527.6e-05

0.0001732.8e-050.0001520.0001680.0001731.9e-05

0.000118

0.000223.5e-050.0001959.5e-050.0002195.7e-05

0.0003540.0001170.0002220.0005460.00036

0.0023760.0019050.0017250.0022090.0013420.0023460.00143

0.0001480.000167

2.1e-05

0.0001636.5e-050.0001466.4e-050.0001661.8e-05

0.0002859.9e-050.0002563e-050.000291

2.6e-05

2.6e-05

0.0003610.0001290.0002230.0003640.000373

0.0001195.7e-052.9e-050.0001227e-06

0.0002420.000273

1.1e-05

3.6e-05

0.0014480.0015550.000710.0022090.0002330.0013940.001405

6.2e-05

3.4e-05

3.4e-05

3.4e-05

6e-056.4e-055.2e-05

6e-056.4e-055.2e-05

6e-056.4e-055.2e-05

0.0038590.0086030.0160060.0084340.008250.0038620.016729

0.0038590.0086030.0160060.0084340.008250.0038620.016729

0.000122

6.8e-05

0.0001280.000144

0.0001120.000126

3.2e-05

0.000240.00027

0.0002240.0001630.0001350.000225

0.0010880.000550.0007380.0008410.001129

0.0001127.2e-050.0001111.5e-05

0.0001650.000186

0.000119

0.0001016e-055.5e-050.000101

0.0001150.00013

8.6e-05

0.0012930.0073110.0010270.0060240.0003130.0012410.009081

0.0002720.000307

4.3e-05

7.4e-057e-057e-05

0.0002290.000258

1e-059e-061.2e-05

7.5e-05

5.7e-05

1.5e-05

0.0014440.001627

0.0007740.000872

0.0001620.0001450.0001540.000175

7.5e-055e-057.8e-05

0.0001570.0003810.0001769.4e-050.0001634e-06

0.0003380.0002070.0002270.0002070.000329

0.0001230.000138

8.1e-05

0.0006550.000738

0.000104

5.7e-054.5e-055.6e-05

6.5e-058.8e-056.9e-05

0.000130.000146

3e-05

2.9e-057e-050.0021390.002412e-053e-05

6.3e-056e-056.1e-05

0.0067320.007584

0.0002720.000306

1.1e-052.4e-052e-061.2e-05

0.001810.0007750.0008610.0017070.001840.0017550.000644

9.5e-050.00019.6e-05

9.5e-050.00019.6e-05

9.5e-050.00019.6e-05

0.0002375.3e-050.0004760.0002410.000111

0.0002375.3e-050.0004760.0002410.000111

0.0002375.3e-050.0004760.0002410.000111

0.0014780.0007220.0008610.0017070.0012640.0014180.000533

4.6e-056.3e-054.1e-05

4.6e-056.3e-054.1e-05

0.0014320.0007220.0008610.0017070.0012010.0013770.000533

0.0013890.0007220.0008610.0017070.0011470.0013360.000533

4.3e-055.4e-054.1e-05

1.6e-055.2e-051.5e-05

1.6e-055.2e-051.5e-05

1.6e-055.2e-051.5e-05

1.6e-055.2e-051.5e-05

0.0001720.0001760.0001716.2e-05

0.0001720.0001760.0001716.2e-05

5.6e-056.6e-055.6e-05

5.6e-056.6e-055.6e-05

6e-055.6e-055.7e-056.2e-05

6e-055.6e-055.7e-056.2e-05

5.6e-055.4e-055.8e-05

5.6e-055.4e-055.8e-05

0.2090090.123520.1135610.0764050.1799030.2005830.097396

0.2090090.123520.1135610.0764050.1799030.2005830.097396

4.8e-056.7e-055e-053.3e-05

3.2e-054e-053.2e-051.6e-05

1.7e-05

1.6e-052.7e-051.8e-05

9.6e-050.0001650.0001869e-054.9e-05

9.6e-050.0001650.0001869e-054.9e-05

0.0435630.0246260.0229680.0165660.0337540.0418670.021775

0.0012950.0004560.0007260.0027110.0012630.0012760.000412

0.0422680.024170.0222420.0138550.0324910.0405910.021363

0.1653020.0988940.0904280.0598390.1458960.1585760.075539

0.1651680.0988940.0897790.0598390.1451650.1584470.075539

4.3e-050.0001340.0001514.1e-05

4.7e-050.0001530.0001724.4e-05

4.4e-050.0003620.0004084.4e-05

4.7e-050.0001343.6e-05

4.7e-050.0001343.6e-05

4.7e-050.0001343.6e-05

4.7e-050.0001343.6e-05

6.2e-05

4.7e-057.2e-053.6e-05

5.4e-059.5e-053.7e-05

5.4e-059.5e-053.7e-05

5.4e-059.5e-053.7e-05

2.9e-055.1e-051.8e-05

2.9e-055.1e-051.8e-05

2.5e-054.4e-051.9e-05

2.5e-054.4e-051.9e-05

0.0002923.4e-050.00030.0005050.0002871.7e-05

0.0002923.4e-050.00030.0005050.0002871.7e-05

0.0002923.4e-050.00030.0005050.0002871.7e-05

0.0002923.4e-050.00030.0005050.0002871.7e-05

0.0002923.4e-050.00030.0005050.0002871.7e-05

1.5e-059.9e-051.4e-05

1.5e-059.9e-051.4e-05

1.5e-059.9e-051.4e-05

1.5e-059.9e-051.4e-05

1.5e-059.9e-051.4e-05

1.5e-059.9e-051.4e-05

0.0001030.0041710.0004888.8e-050.004571

1.4e-057.1e-051e-05

1.4e-057.1e-051e-05

2e-062.5e-052e-06

2e-062.5e-052e-06

0

2e-062.4e-052e-06

1e-06

1.2e-054.6e-058e-06

1.2e-054.6e-058e-06

5e-062.2e-053e-06

7e-062.4e-055e-06

9e-060.00011.3e-05

9e-060.00011.3e-05

9e-060.00011.3e-05

6.4e-05

6.4e-05

9e-063.6e-051.3e-05

9e-063.6e-051.3e-05

2e-056.1e-051.6e-05

3e-064e-064e-06

3e-064e-064e-06

3e-064e-064e-06

0

3e-064e-064e-06

1.7e-055.7e-051.2e-05

1.7e-055.7e-051.2e-05

1.7e-055.7e-051.2e-05

1.7e-055.7e-051.2e-05

0.0040570.004571

0.0040570.004571

0.0040570.004571

0.0040570.004571

0.0040570.004571

7e-065.8e-055e-06

7e-065.8e-055e-06

7e-065.8e-055e-06

7e-065.8e-055e-06

7e-065.8e-055e-06

5.3e-050.0001140.0001984.4e-05

4.5e-050.0001140.0001293.8e-05

4.5e-050.0001140.0001293.8e-05

4.5e-050.0001140.0001293.8e-05

4.5e-050.0001140.0001293.8e-05

8e-066.9e-056e-06

8e-066.9e-056e-06

8e-066.9e-056e-06

8e-066.9e-056e-06

0.4065460.4133270.4480389999999990.3542150000000010.4354010.4233410.374129

0.0714470.0476650.1194780.0592350.0914310.0684690.094072

0.0349330.0227750.0746310.0307220.051520.0337980.049102

0.0053560.0024790.0028790.0042170.0027420.005040.00197

0.000171

9.1e-05

8e-05

0.0038370.0021010.0018390.0023090.0012660.0036440.001643

4.5e-05

0.0038370.0021010.0018390.0023090.0011870.0036440.001643

3.4e-05

0.0013410.0003780.001040.0019080.0010250.0012580.000327

0.0006140.000691

0.0013410.0003780.0004260.0019080.0002120.0012580.000327

0.000122

0.0001056.2e-058.9e-05

0.0001056.2e-058.9e-05

7.3e-050.0002184.9e-05

7.3e-058.1e-054.9e-05

7.9e-05

5.8e-05

0.0170730.009880.0307420.0113450.0303570.0171290.010492

0.0086490.0044010.0236770.0249750.0089430.004866

0.0021340.0026530.0014860.0008850.0022040.001791

0.0009780.001102

0.018250.020563

0.0017760.0002950.0006080.0012410.0018280.000503

0.0020910.0004120.0007420.0011840.0021690.000566

0.0026480.0010410.0016130.0027420.002006

0.0001320.0001550.000480.000114

0.0001321.5e-050.000114

8.4e-05

8.8e-05

2.4e-05

2.7e-05

1.5e-05

2.6e-05

0.0001550.000174

2.7e-05

0.0025480.0008650.0031240.0021080.0021470.0024340.00112

1.6e-05

0.0025140.0020690.0021040.002416

3.4e-050.0008650.0010550.0021082.7e-051.8e-050.00112

0.0006226e-050.0003050.0003870.0005774.7e-05

0.0001216.2e-050.0001113.2e-05

9.1e-055.3e-057.4e-051.5e-05

5.9e-053.2e-055.5e-05

3.9e-05

0.000101

0.0001752e-050.0001515.8e-050.000166

0.0001764e-050.0001544.2e-050.000171

0.0033190.0035590.0024340.007530.0015680.0032920.003267

0.000210.0001832.1e-050.000203

0.0002320.0021560.0004370.0020081.8e-050.0002260.002109

0.0001041.5e-053e-060.0001110.000122

2.1e-05

0.0012540.0010640.0006980.0018070.0003310.001220.000668

0.000214.9e-050.0018070.0001260.0002070.000128

3.4e-05

1.6e-05

1e-06

6e-06

6.6e-052.4e-059e-066.7e-051e-05

0.000265.3e-050.0002289.5e-050.000254

0

0.0002773e-050.0002454.2e-050.0002763.4e-05

0.0001220.000137

2.6e-05

0.0001422.6e-050.000132.6e-050.000151

1.5e-05

5.5e-05

0.0001191.3e-050.000122

6.4e-059.8e-050.0002710.0019081.5e-056.3e-050.000166

7.7e-05

0.0001320.000126.4e-050.000137

1.3e-05

6e-05

5.9e-05

2.1e-05

1e-06

9.3e-053e-059.5e-058e-06

1e-05

4e-06

2.5e-05

2.2e-05

3.5e-058e-063.5e-05

2.4e-05

2.1e-055e-062.2e-055e-06

4.6e-053e-056e-064.5e-051.7e-05

5.4e-051.4e-051e-055.8e-05

0.000119

0.0018030.0009950.0010470.0017070.00080.0017690.001192

3.1e-05

0.00060.0006730.000460.0017070.0001910.0005940.001068

0.000110.000124

0.0012030.0003220.0004770.0003750.0011759.3e-05

0.00011

0.0104620.00780.008160.0110440.0081370.009970.009354

0.000119

4.4e-05

7.5e-05

0.000132.4e-055.1e-050.000134

2.3e-05

6.9e-051.6e-056.8e-05

6.1e-052.4e-051.2e-056.6e-05

7.6e-05

3.9e-05

3.7e-05

2.7e-055.5e-052.1e-05

2.7e-055.5e-052.1e-05

8e-057.2e-056.7e-05

8e-057.2e-056.7e-05

0.0004847.3e-050.0004190.0004530.0004496.4e-05

0.0001430.0001159.8e-050.0001183.3e-05

0.0003417.3e-050.0003040.0003550.0003313.1e-05

0.002620.0015130.0014410.0038150.0010170.0025340.000967

0.0001244.2e-050.000112

9.1e-056.3e-052.5e-058.8e-054.2e-05

0.0003920.0001650.000280.0018070.0002510.000376

3.9e-05

0.0001680.0001468.2e-050.000165e-05

0.0018450.0012850.0010150.0020080.0005780.0017980.000875

0.0001274.6e-050.000114

0.0001274.6e-050.000114

9.9e-05

9.9e-05

0.0063870.0061460.0057980.0072290.0057290.0061150.008306

0.0063870.0061460.0057980.0072290.0056540.0061150.008306

7.5e-05

0.0001860.0001560.0001680.000173

0.0001860.0001560.0001680.000173

0.0001574.4e-050.0001315e-050.000139

0.0001574.4e-050.0001315e-050.000139

0.0002640.0002157.4e-050.0002241.7e-05

0.0002640.0002157.4e-050.0002241.7e-05

0.000128

7.1e-05

5.7e-05

0.0116360.01311

0.0116360.01311

0.0116360.01311

0.001240.0026160.0019730.0041160.0012760.000990.001349

0.000118

0.000118

0.000106

0.000106

0.000122

0.000122

4.8e-058.4e-053.9e-05

4.8e-058.4e-053.9e-05

0.0007510.0025670.0017240.0041160.0005440.0006860.001303

3e-05

0.0001224.2e-050.0001111.8e-05

7e-06

0.0002150.000178.7e-050.000173

8.7e-05

0.0003660.0012720.0003630.0020086.7e-050.0003550.000186

3.4e-05

9.3e-05

5.8e-05

3.2e-05

2.2e-050.0012950.0003590.0021087e-062.2e-050.000162

2.6e-050.00083202.5e-050.000937

4.4e-050.0001182.3e-05

7.3e-05

4.4e-054.5e-052.3e-05

0.0003674.9e-050.0002490.0001170.0002284.6e-05

0.0003674.9e-050.0002497.8e-050.0002283.8e-05

8e-06

3.9e-05

3e-056.7e-051.4e-05

2.6e-055.1e-051.1e-05

4e-061.6e-053e-06

0.0008020.0192410.0090080.0006690.012827

4.1e-05

4.1e-05

6e-05

6e-05

0.0187220.0084560.012808

5.7e-05

0.000113

0.0073540.008286

0.0113680.012808

0.0002370.0001939.5e-050.000201

0.0002370.0001939.5e-050.000201

0.0002910.0001860.000150.000241.9e-05

0.0002910.0001860.000150.000241.9e-05

9.8e-057.3e-058.5e-05

4.2e-057.3e-053.7e-05

5.6e-054.8e-05

0.0001760.000148e-050.000143

0.0001760.000148e-050.000143

5.3e-05

5.3e-05

0.0019520.0003160.0020080.0021660.0017360.000278

0.000120.0001049.3e-05

0.000120.0001049.3e-05

0.000120.0001049.3e-05

0.0004260.0002710.000220.000354

0.0001680.0001230.0001120.000148

0.0001680.0001230.0001120.000148

0.0002580.0001480.0001080.000206

0.0002580.0001480.0001080.000206

0.0014060.0003160.0017370.0018420.0012890.000278

0.0006577.6e-050.0003930.0002920.0005960.000141

0.0001990.0001660.0001770.000186

0.0004587.6e-050.0002277e-060.000410.000141

1.3e-05

9.5e-05

0.0007490.000240.0013440.001550.0006930.000137

0.0010880.001226

0.0007490.000240.0002560.0003240.0006930.000137

0.0087210.0099940.0273940.0168670.0109310.0082710.032402

0.0006510.0004320.0003020.0004766.5e-05

0.0002790.0001590.0001180.0002131.6e-05

8e-06

0.0002790.0001590.0001180.0002138e-06

0.0001990.0001549.1e-050.0001533.2e-05

0.0001990.0001549.1e-050.0001533.2e-05

0.0001730.0001199.3e-050.000111.7e-05

0.0001730.0001199.3e-050.000111.7e-05

0.008070.0099940.0269620.0168670.0106290.0077950.032337

0.0002670.0003

0.0002670.0003

0.0017570.0010910.000889

0.0007890.000889

0.0009680.001091

0.000370.000417

0.000370.000417

0.008070.0099940.0231580.0168670.0092380.0077950.029443

0.0006590.000743

0.0015660.0067470.0006460.0041160.0001520.0020360.007319

0.0043130.0018040.0014590.0105420.0017970.0043050.000505

0.0021710.002447

0.0022160.002497

0.0019090.002151

0.0066190.007458

0.000116

0.0029930.003372

0.000140.000158

0.0015460.00470.001069

2.2e-05

0.0021160.002384

0.0021910.0014430.0006840.0022090.0002050.0014540.001586

0.001410.001588

0.001410.001588

0.0242660.0136490.0145250.0098390.0254020.0231690.01175

0.0008360.0006770.000356

9.1e-05

9.1e-05

0.0008360.0005860.000356

0.0003160.000356

0.000520.000586

0.0228680.0134280.0131450.0098390.0207860.0219180.011097

0.000128

6.3e-05

6.5e-05

0.0227360.0134280.0131450.0098390.0205230.0218110.011097

0.0226990.0134280.0131450.0098390.0204380.0217780.011097

3.7e-058.5e-053.3e-05

7.3e-051.5e-056.2e-05

3e-058e-062.4e-05

4.3e-057e-063.8e-05

5.9e-05

5.9e-05

5.9e-056.1e-054.5e-05

5.9e-056.1e-054.5e-05

0.0001450.0003230.000119

2.5e-050.0001112.4e-05

2.5e-055.7e-052.4e-05

5.4e-05

0.000120.0002129.5e-05

4.8e-05

3.6e-054.2e-052.9e-05

2.9e-054.2e-053.7e-05

1.3e-051.3e-051.9e-05

4.2e-054.6e-051e-05

2.1e-05

0.000960.0002210.000430.0033290.0009220.000297

0.000960.0002210.000430.0033290.0009220.000297

0.000960.0002210.000430.0033290.0009220.000297

0.0002930.0001140.0002870.00021

0.0001080.0001227.7e-05

0.0001080.0001227.7e-05

0.0001290.0001149e-059.5e-05

0.0001290.0001149e-059.5e-05

5.6e-057.5e-053.8e-05

5.6e-057.5e-053.8e-05

0.0015750.0009310.000920.0018070.0014120.0014950.00054

0.0015750.0009310.000920.0018070.0014120.0014950.00054

0.0013680.0009310.000920.0018070.0012320.0013060.00054

0.0013680.0009310.000920.0018070.0012320.0013060.00054

4.9e-051.6e-054.6e-05

4.9e-051.6e-054.6e-05

5.9e-052.8e-056.3e-05

5.9e-052.8e-056.3e-05

9.9e-050.0001368e-05

4.1e-051.6e-053e-05

1.9e-052.9e-052e-05

1.9e-055e-051.7e-05

1e-052.1e-057e-06

1e-052e-056e-06

6.7e-059e-056.1e-05

6.7e-059e-056.1e-05

6.7e-059e-056.1e-05

6.7e-059e-056.1e-05

4.2e-052.8e-053.5e-05

1.2e-052.3e-051e-05

1.3e-053.9e-051.6e-05

0.1256250.1552380.1879140.1791150.1844710.1536780.185449

0.0016240.0009280.0016550.0018070.0003620.0014820.00179

0.0008720.000982

0.0008720.000982

0.0008720.000982

0.0016240.0009280.0007830.0018070.0003620.0014820.000808

0.0013610.0009280.0006460.0018070.0002190.0013040.000769

1e-05

0.0001362.3e-050.0001250.000108

0.0010240.000890.0003440.0018070.0001060.0009890.000416

0.0002013.8e-050.0001731.4e-050.000199.9e-05

6.6e-05

0.0001290.000146

0.0001540.0001378e-059.9e-053.5e-05

0.0001540.0001378e-059.9e-053.5e-05

0.0001096.3e-057.9e-054e-06

0.0001096.3e-057.9e-054e-06

0.0008130.003380.0005180.0033130.0003310.0007220.003878

0.000810.003380.0005180.0033130.000270.0007210.003878

3.2e-05

3.2e-05

0.000810.003380.0005180.0033130.0002380.0007210.003878

0.0007990.003380.0005180.0033130.0001480.0007090.003878

2.1e-05

2.9e-05

1.1e-052.2e-051.2e-05

1.8e-05

3e-066.1e-051e-06

1.7e-05

1.7e-05

3e-064.4e-051e-06

3.4e-05

3e-061e-051e-06

0.0150340.005580.0110920.005020.0194430.0145250.003174

5.5e-05

5.5e-05

5.5e-05

0.0002880.0002980.0002553.1e-05

3.9e-057.6e-053.3e-05

1.7e-051.7e-051.4e-05

9e-061.8e-056e-06

1.3e-054.1e-051.3e-05

0.0001085.7e-050.0001013.1e-05

0.0001085.7e-050.0001013.1e-05

8e-055.4e-057.1e-05

8e-055.4e-057.1e-05

6.1e-055.1e-055e-05

6.1e-055.1e-055e-05

6e-05

6e-05

0.0146860.005580.0110920.005020.0189090.0142190.003143

0.0142590.005580.0106670.005020.0181010.0138340.003127

0.0002020.000227

0.0042480.004786

0.0003510.0002230.000196

0.0135090.005580.0057450.005020.0127180.0134210.003111

0.0003990.0002490.0001740.0004131.6e-05

0.000150.0001490.0002130.0001541.6e-05

0.000150.0001490.0002130.0001541.6e-05

0.0001490.0001790.000114

7.3e-059.5e-055.4e-05

7.6e-058.4e-056e-05

0.0001280.0001150.0001480.000117

0.0001280.0001150.0001480.000117

8.7e-05

8.7e-05

0.0001610.000181

0.0001610.000181

6e-050.0001245.1e-05

6e-050.0001245.1e-05

6.3e-05

6e-056.1e-055.1e-05

5.7e-05

5.7e-05

5.7e-05

6.7e-05

6.7e-05

6.7e-05

6.7e-05

0.0072450.0108290.0104460.0141560.0026960.005450.01259

0.0072450.0108290.0104460.0141560.0026960.005450.01259

0.0001188e-050.0001510.0015069.3e-050.0001197.9e-05

0.0001188e-050.0001510.0015069.3e-050.0001197.9e-05

2.3e-053.3e-050.0001552.2e-05

2.3e-053.3e-051.8e-052.2e-05

4.6e-05

9.1e-05

2.4e-053.7e-053.3e-051.6e-05

2.4e-053.7e-053.3e-051.6e-05

0.0003615.7e-050.0010790.0003060.0003790.001014

7.4e-05

0.0008860.000999

7.9e-050.0001118.1e-05

0.000210.0001936.3e-050.000225

7.2e-055.7e-055.8e-057.3e-051.5e-05

7.4e-050.0001140.0001296.1e-05

7.4e-050.0001140.0001296.1e-05

9.7e-056.2e-059.3e-05

9.7e-056.2e-059.3e-05

7.8e-050.0001186.2e-05

7.8e-050.0001186.2e-05

0.0036170.0034750.0018270.0028110.0008170.0022260.003104

0.0033630.0034750.0016670.0028110.0007080.0019430.003062

4.2e-05

0.0002540.000160.0001090.000283

0.0028530.0071840.0072750.0098390.0009790.0024550.008377

0.0054140.0061

0.0006270.0002280.0004560.0031120.0004870.0006550.000555

0.0003560.0001050.0019080.0001110.0001661.7e-05

0.0001270.0001122.2e-050.000127

0.000118

0.0017430.0068510.0010340.0048190.0003590.0015070.001295

0.0002590.000292

0.0001030.0004390.0003319.6e-050.000494

6.9e-05

6.9e-05

4e-05

2.9e-05

9.2e-05

4.4e-05

4.4e-05

4.8e-05

4.8e-05

0.0004390.000494

0.0004390.000494

0.0004390.000494

0.0001030.000179.6e-05

2.6e-053.5e-052.8e-05

2.6e-053.5e-052.8e-05

2.2e-054.5e-051.9e-05

2.2e-054.5e-051.9e-05

1.5e-053.3e-051e-05

1.5e-053.3e-051e-05

4e-055.7e-053.9e-05

4e-055.7e-053.9e-05

0.0008954e-050.0011710.0023260.000725.5e-05

7.1e-059.4e-055.4e-05

7.1e-059.4e-055.4e-05

7.1e-053.6e-055.4e-05

2.7e-05

3.1e-05

0.0005264e-050.0011710.0017850.0004283.1e-05

8.9e-05

8.9e-05

0.0002594e-050.0010560.0015070.000243

0.000117

0.000116

0.0008560.000965

0.0002594e-050.00020.0001870.000243

0.000122

0.000150.0001150.0001070.000143.1e-05

0.000150.0001150.0001070.000143.1e-05

5.2e-05

5.2e-05

5e-051.3e-054.2e-05

5e-051.3e-054.2e-05

6.7e-051.7e-053e-06

6.7e-051.7e-053e-06

9.8e-050.0001278.6e-05

9.8e-050.0001278.6e-05

4e-06

2.5e-052e-062.5e-05

7.3e-056.7e-056.1e-05

5.4e-05

4.1e-05

4.1e-05

4.1e-05

4e-054.7e-053.2e-05

4e-054.7e-053.2e-05

4e-054.7e-053.2e-05

0.000160.0002320.000122.4e-05

6e-053.3e-054.2e-05

6e-053.3e-054.2e-05

5.8e-05

5.8e-05

4.7e-05

4.7e-05

0.00019.4e-057.8e-052.4e-05

4.1e-052.8e-053.2e-05

3.4e-053.7e-053e-051.6e-05

2.5e-052.9e-051.6e-058e-06

0.0007460.002550.0006910.0033130.0006380.0010010.003188

0.0002790.0001150.0002810.000203

1.5e-052.9e-051e-05

1.5e-051.1e-051e-05

01.8e-050

5e-054.7e-055.4e-05

5e-054.7e-055.4e-05

0.0001760.0001150.0001540.000124

0.0001360.0001159.5e-050.000124

4e-055.9e-05

3.8e-054.6e-051.5e-05

2.7e-05

3.8e-051.9e-051.5e-05

5e-06

5e-06

8.7e-05

4.9e-05

4.9e-05

3.8e-05

3.8e-05

0.0004670.002550.0005760.0033130.000270.0007980.003188

0.0004670.002550.0005760.0033130.0002470.0007980.003188

0.000450.002550.0005760.0033130.0002110.0007750.003188

8e-064e-061.1e-05

7e-068e-066e-06

1.7e-05

6e-06

2e-061e-066e-06

2.3e-05

2.3e-05

0.0001550.0001120.0004030.000131

0.0001550.0001120.0004030.000131

2.1e-050.0001120.0001272.1e-05

2.1e-050.0001120.0001272.1e-05

1.3e-050.0001881.7e-05

1.3e-057.2e-051.7e-05

6.5e-05

5.1e-05

0.0001218.8e-059.3e-05

0.0001218.8e-059.3e-05

0.016570.0176870.0154910.0163650.0141070.0163670.009575

1.7e-05

1.7e-05

1.7e-05

0.016570.0176870.0154910.0163650.0141070.0163670.009558

3.4e-050.0001043.1e-056e-06

5.7e-05

6e-06

3.4e-054.7e-053.1e-05

9.4e-058.7e-059.1e-05

3.7e-054e-054.1e-05

2.2e-05

5.7e-052.5e-055e-05

0.0006990.0007871.5e-05

0.0006990.0007871.5e-05

0.0164420.0176870.0147920.0163650.0131290.0162450.009537

1.9e-059e-060.0026730.0030124e-061.6e-05

1e-05

4e-06

0.0138170.0076020.0080010.0052210.011920.013275

3.2e-052.2e-058.5e-054.5e-05

6e-06

4.8e-05

3.4e-058e-06

1.6e-05

0.0022970.0100610.0020680.0058230.0007260.0026030.009283

3.5e-053e-062.2e-05

1.8e-057e-061.8e-052.1e-05

7.1e-058.7e-056.9e-05

6.1e-052.8e-056.6e-054.7e-05

3.9e-05

3.8e-051.5e-050.002050.0023091.3e-053.1e-052.6e-05

2.3e-05

2.2e-05

4.1e-052.2e-05

3.5e-05

2.9e-051.1e-052.7e-05

6e-06

1.9e-056e-06

7e-05

8e-06

2.5e-056e-063.3e-05

0.000116

0.000116

6.9e-05

6.9e-05

4.7e-05

4.7e-05

0.0089350.005940.0062520.0086340.0081170.0084480.004504

7e-057.6e-056.6e-05

7e-057.6e-056.6e-05

2.9e-053.7e-052.4e-05

4.1e-053.9e-054.2e-05

0.0002420.0001870.0007220.0002351.5e-05

0.0002420.0001870.0007220.0002351.5e-05

2.4e-05

2.7e-05

7e-06

0.0001580.0001870.0006060.00016

3.3e-054e-062.6e-051.5e-05

1.8e-05

7e-06

2.3e-05

5.1e-053e-064.9e-05

3e-06

0.004670.0027460.0026420.002610.0036680.0044540.001982

0.004670.0027460.0026420.002610.0036680.0044540.001982

9e-064e-068e-06

3.4e-051.5e-053.3e-05

4.5e-052.9e-054e-05

2.2e-051.6e-051.9e-05

5e-062e-065e-06

1.3e-051.1e-051.3e-05

1.5e-055e-061.3e-05

2.8e-051.4e-053.1e-05

0.0042840.0027460.0026420.002610.0034870.0041040.001982

2.1e-059e-061.7e-05

2.7e-052.4e-052.1e-05

5.3e-054.7e-05

8e-061.7e-051.2e-05

5.8e-057e-064.7e-05

9e-062e-067e-06

9e-064e-069e-06

3e-052.2e-052.8e-05

5e-053.8e-054e-05

5e-053.8e-054e-05

5e-053.8e-054e-05

3.4e-052.6e-051.8e-05

3.4e-052.6e-051.8e-05

3.4e-052.6e-051.8e-05

3.3e-05

3.3e-05

3.3e-05

0.0038540.0031940.0034230.0060240.0035130.003620.002507

4.7e-05

4.7e-05

4.5e-05

4.5e-05

0.0022390.0013270.0013690.0020080.0021210.002150.000807

0.0022240.0013270.0013690.0020080.0020750.0021420.000807

1.5e-054.6e-058e-06

0.0003740.0007880.0011020.0020080.0002140.0002950.000843

3.1e-051.4e-053.6e-05

1e-06

0

8e-06

2e-050.0007880.0009010.0020081e-051.8e-050.000843

1.5e-05

0.0003230.0002010.0001660.000241

0.0012080.0010790.0009520.0020080.001010.0011450.000857

1.8e-05

6.4e-05

1.5e-05

1.4e-05

2.3e-05

4.4e-051.7e-053.5e-05

0.0011220.0010790.0009520.0020080.0008440.0010790.000857

4.2e-051.5e-053.1e-05

3.3e-057.6e-053e-05

3.3e-057.6e-053e-05

1.5e-054.1e-051.5e-05

1.5e-054.1e-051.5e-05

1.9e-05

1.5e-052.2e-051.5e-05

0.000230.0001290.0005810.000183

0.000230.0001290.0005810.000183

0.000104

6.4e-05

4e-05

5.8e-052.5e-055.4e-05

5.8e-052.5e-055.4e-05

0.0001720.0001290.0002680.000129

0.0001450.0001298.3e-059e-05

1.4e-052.9e-052.9e-05

6.5e-05

3.9e-05

1.3e-055.2e-051e-05

5.9e-05

5.9e-05

5.2e-05

5.2e-05

7.3e-05

7.3e-05

2e-057.7e-051.5e-051.6e-05

2e-057.7e-051.5e-051.6e-05

2e-057.7e-051.5e-05

2e-057.7e-051.5e-05

1.6e-05

1.6e-05

8.6e-05

8.6e-05

8.6e-05

8.6e-05

0.0403940.0355420.0674550.0682740.0570340.0527590.040516

0.0016274.6e-050.0010640.0008450.0014120.000662

0.0007990.0007020.0003870.0006920.000556

3e-056e-063.3e-05

5.2e-056e-065.9e-05

0.0002420.0001745.3e-050.0001880.000171

8.9e-0508.8e-05

0.0003860.0003229e-050.0003240.000385

0.0002060.000232

0.0007824.6e-050.0003620.0003810.0006720.000106

2.2e-05

5.2e-05

0.0003143.3e-050.0001660.0001060.0002921.8e-05

8.5e-051.3e-052.5e-057.1e-058.8e-05

0.0002460.0001961.4e-050.0002

0.0001373.1e-050.000109

8.8e-05

4.3e-05

2.2e-054.7e-052.4e-05

2.2e-054.7e-052.4e-05

2.4e-053e-052.4e-05

2.4e-053e-052.4e-05

0.0102090.0123450.0128380.0219890.0044290.0150220.01729

6.1e-05

6.1e-05

8.2e-05

8.2e-05

0.0026010.0013970.001660.0019080.0013290.0025410.001232

4.3e-05

4.4e-05

0.0003410.000314.5e-050.000358

0.002260.0013970.001350.0019080.0011560.0021830.001066

3.5e-05

5e-05

0.000122

5.9e-05

5.9e-05

9.8e-05

9.8e-05

0.0002160.0001992.6e-050.000231

0.0002160.0001992.6e-050.000231

0.0006630.0004590.0002150.0005974e-05

1.7e-05

5.3e-056.1e-052.3e-05

0.0002140.0001762.9e-050.0001842.3e-05

8.8e-056.5e-055.9e-05

0.0003080.0002836e-050.000331

0.000101

0.000101

0.0002870.00080.0020050.002516.9e-050.0002780.004202

0.0002590.000291

8.1e-050.00080.0013890.002519e-067.8e-050.003711

0.0002060.000184e-050.0002

0.0001772e-050.0002

7.7e-05

1.8e-05

5.9e-05

4.3e-054.2e-055.4e-05

4.3e-054.2e-055.4e-05

0.0026970.0007540.0017850.0017070.0006270.0025658.6e-05

7e-06

0.0002470.000221.6e-050.0002482.4e-05

1e-06

1e-06

0.0003240.0002891.3e-050.000326

2e-06

0.0003580.0003075.6e-050.0003341.6e-05

7e-06

3e-06

5e-06

1.4e-05

0.0015640.0007340.0008010.0017070.0004390.001481

0.0002042e-050.0001687e-060.0001764.6e-05

4.7e-05

9e-06

0.0001

0.0001

4.5e-05

4.5e-05

0.0017170.0052930.0046260.0105420.0002930.0016670.005379

0.0013150.0048240.000640.0046180.0002010.001297

6.1e-054.6e-050.0015150.0017077e-066.2e-05

7.7e-050.0020570.0019084.5e-053.9e-050.002952

0.0002640.0004230.0004140.0023094e-050.0002690.002427

6.6e-05

6.6e-05

7.6e-05

7.6e-05

5.6e-055.3e-05

5.6e-055.3e-05

2.9e-05

2.9e-05

4.1e-054.3e-057.7e-05

4.1e-054.3e-057.7e-05

0.0001711.9e-059.2e-050.0001816.6e-05

1.3e-05

7.3e-052.5e-058.1e-051.2e-05

9.8e-051.9e-056e-060.00015.4e-05

4e-06

2.2e-05

0

2.2e-05

0.0010460.0034470.0013790.0029120.0001570.0010260.003652

0.0001521.6e-050.0001331.2e-050.0001461.7e-05

0.0007420.0034310.0012460.0029122.9e-050.0007680.003617

2e-06

1e-06

2.8e-052e-062.5e-05

0.000103

0.0001248e-068.7e-051.8e-05

0.0002780.0006350.0005170.002410.0003020.0053480.002633

0.0001340.0006350.0003950.002410.0002910.0052170.002596

0.0001440.0001221.1e-050.0001313.7e-05

7.6e-05

7.6e-05

4.7e-050.0002225.2e-05

5.4e-05

5.9e-05

6e-063e-067e-06

1e-052e-069e-06

1e-055.4e-051e-05

2.1e-055e-052.6e-05

0.0003460.0002080.0001420.000352

0.0003460.0002080.0001420.000352

8.6e-05

8.6e-05

8.6e-05

0.0013660.0010530.0005710.0012217e-05

0.0002270.0001816.8e-050.000185

0.0002270.0001816.8e-050.000185

0.000112

0.000112

0.0004060.0003490.0002050.0003826.2e-05

4.1e-05

2.5e-05

0.0001840.000165.1e-050.000177

4.9e-05

0.0002220.0001893.9e-050.0002056.2e-05

0.0001099e-069.6e-05

0.0001099e-069.6e-05

0.0004190.000350.0001490.0003738e-06

0.0001590.0001385.1e-050.0001528e-06

4.1e-05

0.000260.0002125.7e-050.000221

0.0002050.0001732.8e-050.000185

0.0002050.0001732.8e-050.000185

0.0027820.0014130.0028640.0028110.0015860.0030130.001067

0.0020890.0013990.0024170.0028110.0013230.0024320.000856

0.0006920.0011740.000584

0.0003210.0002852.5e-050.00032

0.0003490.0011940.0005110.0028116e-060.000346

0.0006256.1e-050.0006799.9e-050.0009855.9e-05

0.0007940.0001440.000251.9e-050.0007810.000213

0.0001430.0001330.000106

6.2e-05

3.4e-05

0.0001433.7e-050.000106

0.000551.4e-050.0004470.000130.0004750.000211

0.0002411.4e-050.0001972.2e-050.0002150.000211

2.4e-05

0.0003090.000258.4e-050.00026

0.0002020.0001490.0002660.0001793.1e-05

9e-05

9e-05

0.0002020.0001496.8e-050.0001793.1e-05

2e-06

0.0001780.0001493.3e-050.0001593.1e-05

1e-056e-068e-06

5e-06

1.4e-052.2e-051.2e-05

0.000108

0.000108

0.0242080.0217380.0493320.0434740.0490770.0319120.021396

0.0015720.0002660.0014660.0008130.0015210.000336

3.5e-05

0.0001098.2e-053.2e-050.0001210.000115

5.8e-05

0.0004263e-050.0003494.1e-050.000365

0.000105

1.9e-05

4.2e-05

9e-06

1.1e-05

05.8e-05

0.0005425e-050.0004635.9e-050.000502

0.0004220.000475

0.0004956.2e-050.0002320.0001320.000533

0.0002770.0001530.0001465.3e-050.0002690.000103

3.4e-058.1e-054.2e-054e-05

0.0002437.2e-050.0001461.1e-050.0002290.000103

0.0223590.0213190.046660.0434740.0470170.0301220.020957

6.6e-050.0045830.0008770.0165661.5e-056.5e-050.000362

0.0050610.005702

0.0154080.017361

8.1e-05

0.0087090.009812

0.00110.001239

0.0089050.010033

0.0210730.0159870.0047540.0213860.0011620.0288160.014591

7e-06

0.0012780.00144

0.001220.0007490.0005680.0055220.0001650.0012410.006004

0.001060.001194

0.001060.001194

0.0001550.000174

0.0001550.000174

0.0001550.000174

0.0195790.0413990.0203220.0322290.0079740.0397360.028394

0.0002330.0001250.0002660.000179

7.2e-054.9e-055.3e-05

7.2e-054.9e-055.3e-05

0.0001

4.1e-05

2.5e-05

3.4e-05

0.0001610.0001254.3e-050.000126

0.0001610.0001254.3e-050.000126

7.4e-05

7.4e-05

0.0193460.0413990.0201970.0322290.0077080.0395570.028394

0.0184320.041350.0195080.0322290.0074190.0388740.028155

0.0007840.001190.0008640.0019080.0004440.001770.001409

0.0022540.002539

6.1e-05

3.4e-050.0010960.0012710.0023098e-064e-050.001333

8e-05

0.0124210.0338030.0096850.0156630.0033070.0316030.023865

0.001050.0003240.000450.002510.00020.0010930.000672

0.0011560.0041620.0007290.0028110.0002570.0012220.000876

5e-060.0032080.0036142e-066e-06

0.0024110.000610.0008450.0034140.0003980.002538

0.0005710.0001650.0002020.0001230.000602

5e-061.1e-055e-06

5e-061.1e-055e-06

0.0006980.0005290.0001080.0005219.4e-05

0.0004020.0003114.6e-050.0003117.6e-05

0.0002960.0002186.2e-050.000211.8e-05

0.0002114.9e-050.000160.0001040.0001570.000145

0.0002114.9e-050.000161.7e-050.0001579.7e-05

1e-06

1e-06

4.8e-05

4.1e-05

4.4e-05

6.6e-05

2.7e-05

1.6e-05

2.3e-05

0.0132820.0313630.0521410.0260040.0697820.0120430.077275

0.0100920.0257710.0501530.017570.0680150.0091330.0684

0.0098450.0253420.0499810.017570.0676820.0088810.0684

0.0010440.001176

0.0003210.000362

0.0010980.0004030.0002060.0011867e-05

7.9e-05

8.3e-05

0.0053790.023550.0031320.015060.000880.0042190.031115

0.0391690.0618830.034294

0.0013750.0009440.0005420.002510.0001890.001415.3e-05

0.0005070.000571

0.0008140.000917

3.8e-05

5.7e-05

0.0019930.0008480.0008960.0005570.002066

0.0005690.000641

0.0007730.000871

0.0018110.00204

0.00010.0001940.0001720.0001549.5e-05

6.4e-05

6.9e-05

0.00010.0001940.0001722.1e-059.5e-05

0.0001470.0002350.0001790.000157

2.5e-055.8e-057e-062.4e-05

3.1e-05

2.5e-05

4.4e-05

3.4e-051.7e-053.9e-05

1.9e-056e-058e-062e-05

6.9e-050.0001173.7e-057.4e-05

1e-05

0.003190.0055920.0019880.0084340.0017670.002910.008875

1.5e-05

1.5e-05

0.003130.0055760.0019880.0084340.0016770.0028590.008875

0.0001032e-059.3e-05

5.6e-056e-065.6e-054.2e-05

8e-06

0.000118

0.0001510.0001322.3e-050.0001471.8e-05

1.4e-05

8e-06

6e-06

4e-06

5.4e-051e-054.8e-05

5.2e-05

0.0001310.0001142.4e-050.000127

3.3e-05

1.6e-05

2.4e-05

5.3e-05

2.3e-05

2.6e-05

5e-06

4.8e-05

9.3e-052e-068.6e-05

5e-06

2e-05

0.0001510.0001254.3e-050.000132

2.1e-05

0.0001550.0001110.0001212.5e-050.0001545.2e-05

1.1e-051e-051.2e-05

0.000134.2e-050.000122

6e-06

3.1e-05

0.0001320.000117

4.8e-05

5.6e-051.3e-055.4e-05

7e-06

7.7e-059.9e-050.0002960.0017071.6e-056.8e-050.000184

9.7e-051.9e-050.000101

0.0009210.004950.0006730.005020.0001940.0008840.007881

5e-06

3e-05

9e-06

6.1e-05

7.2e-05

3.4e-05

0.00013.5e-051.6e-059.7e-050.000109

1.7e-05

1.3e-05

1.6e-05

2.8e-05

3e-06

4.1e-05

3.7e-05

5.6e-059e-065.4e-058e-06

4e-06

8.4e-051.5e-052.2e-057.6e-05

0.0005720.0003660.000410.0017070.000230.0005480.000574

1.4e-05

6e-051.6e-057.5e-055.1e-05

5e-05

6e-051.6e-052.5e-055.1e-05

0.0030830.0016620.0044390.0052210.0030490.0029030.001209

1.3e-055e-051.1e-05

1.3e-055e-051.1e-05

1e-052.4e-055e-06

1.1e-05

1e-051.3e-055e-06

3e-062.6e-056e-06

3e-062.6e-056e-06

1e-053.9e-059e-06

1e-053.9e-059e-06

1e-053.9e-059e-06

1e-053.9e-059e-06

0.003060.0016620.0044390.0052210.002960.0028830.001209

0.0029260.0016620.0017660.0022090.00260.0027970.001209

2.1e-054.4e-051.8e-05

6e-062.3e-056e-06

1.5e-052.1e-051.2e-05

1.2e-052e-056e-06

1.2e-052e-056e-06

4e-063.1e-054e-06

4e-063.1e-054e-06

0.0028890.0016620.0017660.0022090.0025050.0027690.001209

1.9e-05

0.0028670.0016620.0017660.0022090.0023690.002750.001209

1e-062.9e-050

7e-0606e-06

2.4e-05

2.9e-05

2e-069e-062e-06

3e-061.7e-052e-06

1e-069e-061e-06

8e-068e-06

000

0.0001190.0026730.0030120.0003287.3e-05

8e-065.3e-057e-06

1e-069e-061e-06

7e-061.4e-056e-06

1e-05

5e-06

9e-06

6e-06

6.1e-056e-052.6e-05

4.1e-053e-057e-06

7e-061e-057e-06

1.3e-052e-051.2e-05

5e-050.0026730.0030120.0002154e-05

4e-06

5e-062e-065e-06

1.3e-05

4e-06

1.8e-05

2e-064e-062e-06

1.2e-05

1e-060.0026730.0030121e-062e-06

9e-061.7e-05

2e-05

7e-063.1e-056e-06

8e-06

1e-061.5e-05

5.2e-05

2.5e-053e-062.5e-05

1.1e-05

1.5e-053.2e-051.3e-05

1.5e-053.2e-051.3e-05

1.5e-053.2e-051.3e-05

0.009330.0052610.0057320.0066270.0102830.0088840.00407

2.5e-050.0001692e-05

6e-063.8e-056e-06

6e-063.8e-056e-06

6e-063.8e-056e-06

1.9e-050.0001311.4e-05

3e-063e-052e-06

3e-063e-052e-06

1.2e-057.1e-051e-05

1.2e-057.1e-051e-05

4e-063e-052e-06

4e-063e-052e-06

3.4e-053e-052.4e-05

3.4e-053e-052.4e-05

3.4e-053e-052.4e-05

3.4e-053e-052.4e-05

0.0001460.0004760.000129

4.8e-059.3e-054.7e-05

4.8e-059.3e-054.7e-05

1.2e-052.5e-051.3e-05

7e-061.5e-056e-06

2.9e-055.3e-052.8e-05

4.1e-05

4.1e-05

4.1e-05

4.6e-05

4.6e-05

4.6e-05

4.8e-059.9e-054.3e-05

1.6e-052.7e-051.4e-05

1.6e-052.7e-051.4e-05

3.2e-057.2e-052.9e-05

8e-062e-058e-06

1.4e-05

1.3e-052e-051e-05

1.1e-051.8e-051.1e-05

2.2e-054.1e-051.6e-05

2.2e-054.1e-051.6e-05

2.2e-054.1e-051.6e-05

1.3e-057.4e-051.1e-05

1.3e-054.6e-051.1e-05

1.3e-054.6e-051.1e-05

2.8e-05

2.8e-05

1.5e-058.2e-051.2e-05

4.5e-05

4.5e-05

1.5e-053.7e-051.2e-05

1.5e-053.7e-051.2e-05

2.9e-053.5e-052.4e-05

2.9e-053.5e-052.4e-05

2.9e-053.5e-052.4e-05

2.9e-053.5e-052.4e-05

0.0010430.0005310.0005910.0017070.0011550.000980.00036

0.0010080.0005310.0005910.0017070.001050.0009490.00036

6e-062.5e-056e-06

6e-062.5e-056e-06

0.0010020.0005310.0005910.0017070.0010250.0009430.00036

1.6e-051.3e-05

9e-064e-06

2.2e-053.2e-052.1e-051.6e-05

1.4e-051.7e-051.1e-05

4.8e-05

2.2e-05

1e-061e-06

2.5e-053.1e-052.2e-05

2.9e-053.2e-052.5e-05

6e-063.3e-057e-06

0.000870.0005310.0005910.0017070.0007820.0008330.000344

1e-052.8e-056e-06

9e-062.2e-051e-05

9e-062.2e-051e-05

9e-062.2e-051e-05

2.6e-058.3e-052.1e-05

2.6e-058.3e-052.1e-05

4.9e-05

2.6e-053.4e-052.1e-05

0.0078460.004730.0049240.004920.0076420.0075180.00371

0.00380.002330.0024180.002410.0037460.0036360.001861

2e-054.3e-051.8e-05

2e-054.3e-051.8e-05

5e-063.6e-054e-06

5e-063.6e-054e-06

2.4e-054.7e-051.7e-05

2.4e-054.7e-051.7e-05

0.0037510.002330.0024180.002410.003620.0035970.001861

0.0037510.002330.0024180.002410.003620.0035970.001861

0.0040460.00240.0025060.002510.0038960.0038820.001849

6e-063.6e-055e-06

6e-063.6e-055e-06

8e-062.1e-058e-06

8e-062.1e-058e-06

1e-063.2e-051e-06

1e-063.2e-051e-06

0.0040310.00240.0025060.002510.0038070.0038680.001849

0.0040310.00240.0025060.002510.0038070.0038680.001849

0.0001550.000370.000136

3.2e-050.0001352.7e-05

7.4e-05

4e-05

3.4e-05

3.2e-056.1e-052.7e-05

1.9e-052.8e-051.8e-05

1.3e-053.3e-059e-06

0.0001230.0002350.000109

4e-05

4e-05

0.0001230.0001950.000109

1.2e-052.7e-051.1e-05

6e-062.3e-057e-06

2.1e-052e-051.7e-05

2.7e-056e-062.6e-05

2.2e-051e-051.7e-05

7e-067e-063e-06

1.6e-053.2e-051.7e-05

1.2e-054e-051.1e-05

7e-06

2.3e-05

1.9e-059.8e-052.1e-05

1.9e-059.8e-052.1e-05

4.1e-05

4.1e-05

1.9e-055.7e-052.1e-05

1.9e-055.7e-052.1e-05

3.3e-050.0002170.0003083.2e-05

2.2e-050.0002170.0002442.2e-05

2.2e-050.0002170.0002442.2e-05

2.2e-050.0002170.0002442.2e-05

1.1e-056.4e-051e-05

1.1e-056.4e-051e-05

4e-064.2e-053e-06

7e-062.2e-057e-06

0.1969940.2035010.1304760.1040170.1460770.1893460.089329

0.0885820.0600680.0519210.047390.0529430.0852870.031858

6.1e-057.7e-056e-051.6e-05

6.1e-057.7e-056e-05

6.1e-057.7e-056e-05

1.6e-05

1.6e-05

0.0053470.002540.0039940.0018070.0035810.0053610.001065

0.0001870.000211

0.0001870.000211

7.6e-050.000160.000186.3e-054.1e-05

7.6e-050.000160.000186.3e-054.1e-05

9.8e-054.4e-05

4.4e-05

9.8e-05

0.0052710.002540.0036470.0018070.0030920.0052980.00098

0.001030.0003960.0003350.000260.001050.000201

0.0008440.0003580.0003990.0003280.0008650.00034

0.0009320.0003770.0004140.0002740.0009480.000439

0.0024650.0014090.0014740.0018070.0010750.002435

0.0010250.001155

7.1e-050.0001047.2e-051.6e-05

7.1e-050.0001047.2e-051.6e-05

7.1e-050.0001047.2e-051.6e-05

0.000208

0.000114

0.000114

9.4e-05

5e-05

4.4e-05

0.0002670.0001040.0003220.0015060.0005090.0002440.000107

0.0001640.000184

0.0001640.000184

0.0002090.0001040.0001580.0015060.0002350.0001950.000107

0.0002090.0001040.0001580.0015060.0002350.0001950.000107

5.8e-059e-054.9e-05

5.8e-059e-054.9e-05

0.0012880.0018560.0010290.002410.0008990.0012330.002131

0.0012880.0018560.0010290.002410.0008990.0012330.002131

0.0009320.0018270.000910.002410.000450.0008940.002101

0.000116

0.0001362.9e-050.0001194.7e-050.0001331.5e-05

8.3e-057.3e-057.3e-05

1.7e-05

9.5e-05

5.9e-053.4e-055.4e-05

7.8e-056.7e-057.9e-051.5e-05

0.0110510.0064920.0052040.0063250.0028590.010610.00188

4.3e-05

4.3e-05

0.0001533.4e-050.0001740.0001382e-05

5.1e-054.1e-054.6e-05

4.6e-05

8.8e-052.3e-057.2e-058e-052e-05

1.4e-051.1e-051.5e-051.2e-05

7.3e-05

7.3e-05

0.0002220.0005190.0002160.0002110.000668

0.000180.000202

4.6e-050.0001314.8e-054.6e-050.000148

4.5e-051.4e-055e-056.8e-05

5.5e-055.1e-054.7e-051.6e-05

7.6e-050.0002083.7e-056.8e-050.000234

6.6e-05

0.0106240.0064580.0046850.0063250.0021680.0102180.001192

0.0094790.0055750.0037110.0040160.0018110.009096

7.4e-05

2.5e-05

3e-06

6.9e-054.2e-056.1e-05

1.9e-05

8.7e-05

0

0.0009730.0008650.0009740.0023092.3e-050.0009390.00114

0

3.9e-05

0.0001031.8e-054.5e-050.0001225.2e-05

5.2e-050.0001224.3e-05

3.9e-05

5e-068e-062e-06

1.7e-053e-051.4e-05

2.1e-052.4e-052e-05

9e-062.1e-057e-06

6.3e-05

6.3e-05

9.1e-050.0001219.7e-05

9.1e-050.0001219.7e-05

3.1e-05

1.2e-052.3e-055e-06

3.3e-052.8e-053.3e-05

1.5e-056e-062e-05

3e-066e-063e-06

5e-062e-061.2e-05

4e-067e-064e-06

6e-061.3e-057e-06

1.3e-055e-061.3e-05

0.0002290.0006210.0002133.3e-05

6.1e-057.7e-055.7e-051.8e-05

6.1e-057.7e-055.7e-051.8e-05

3.4e-05

3.4e-05

0.000139

7e-05

6.9e-05

9.7e-05

9.7e-05

5.1e-057.2e-055e-05

5.1e-057.2e-055e-05

2.7e-05

2.7e-05

0.0001170.0001750.0001061.5e-05

4.8e-056.6e-054.1e-05

4.1e-055e-053.5e-051.5e-05

2.8e-055.9e-053e-05

7.8e-05

7.8e-05

7.8e-05

0.0007590.0089140.0029590.0101410.0002120.0007220.009959

0.0006950.0089140.0029590.0101410.0001090.0006820.009959

1.5e-050.0001582.4e-051.5e-050.000178

4e-05

2e-061e-052e-06

1e-06

3e-061e-064e-06

3e-060.0009210.0008183e-063e-06

4e-060.0007690.0006835e-064e-06

7e-061.4e-054e-06

0.0006560.0072240.00130.0101417e-060.0006450.009781

5e-064e-065e-06

6.4e-050.0001034e-05

6.4e-053.3e-054e-05

7e-05

0.0689950.0401620.0381590.0252010.0433010.0662720.016651

3.2e-052.9e-052.6e-054.8e-05

3.2e-052.9e-052.6e-054.8e-05

0.0024520.001960.0024170.0038150.0021230.0023450.007933

0.0024520.001960.0024170.0038150.0021230.0023450.007933

0.0663320.0382020.0355940.0213860.0406490.0637390.008577

0.0001182.7e-058.8e-050.0001225.6e-05

0.0001352.7e-050.0001236.1e-050.0001435.4e-05

0.0001160.000131

0.0002155e-050.0002866.8e-050.0002250.004406

0.0654830.0379830.0342610.0213860.0395890.06285

0.0001490.000167

0.0001322.6e-050.000126.3e-050.000137

0.0003140.000353

0.0002498.9e-050.0002250.0001290.0002620.004061

0.0001790.0001550.0001629.3e-05

1e-05

7.8e-055.8e-056.6e-056e-06

0.0001019.7e-059.6e-057.7e-05

0.0001480.000345

8.6e-05

0.0001480.000167

9.2e-05

0.0004230.0002540.0003730.000403

0.0001228.7e-050.000121

0.0001228.7e-050.000121

0.0001530.0001220.0001190.000146

0.0001530.0001220.0001190.000146

0.0001480.0001320.0001670.000136

0.0001480.0001320.0001670.000136

2.4e-050.0001051.9e-05

2.4e-050.0001051.9e-05

2.4e-050.0001051.9e-05

2.4e-050.0001051.9e-05

0.0005754.9e-050.0017950.0014160.0005990.000961

0.0005754.9e-050.0017950.0014160.0005990.000961

0.0001068.2e-059.8e-05

0.0001068.2e-059.8e-05

4.2e-056.9e-053.8e-05

4.2e-056.9e-053.8e-05

0.0001850.0001520.0002233.2e-05

3.2e-054.1e-057.3e-051.6e-05

3.3e-05

7.6e-053.7e-057.3e-05

7.7e-054.1e-057.7e-051.6e-05

0.0002424.9e-050.0017950.0010320.000240.000929

0.0001120.000126

0.0008250.000929

0.0004390.000495

0.0002650.000299

0.0002424.9e-050.0001540.0001120.00024

8.1e-05

8.1e-05

0.0041690.0064360.0068020.0095380.0035260.0039880.005535

8e-05

8e-05

8e-05

0.0013240.000940.0033520.0022090.0005350.0012690.000714

0.0013210.000940.0033520.0022090.0005180.0012660.000714

0.0001840.0001634.3e-055.9e-056.1e-05

1.6e-052.5e-058e-06

4e-061e-065e-06

0.0001192.1e-050.000136.5e-05

5e-062e-061e-06

1.6e-056e-061.6e-05

7e-051.9e-057.2e-05

2.4e-050.0004740.0002121.2e-052.7e-050.00016

1.1e-053e-061.1e-05

0.0001340.000151

2.4e-050.001960.0022091.1e-052.6e-05

1.5e-053e-061.7e-05

3.4e-05

0.0004990.0001720.0001910.0001130.0005418e-05

5.9e-052.6e-056.4e-05

0.0002360.000266

1.3e-055e-061.4e-05

2e-060.0002940.0002611e-063e-06

0.000210.0001955.7e-050.000229

6e-063e-068e-064.8e-05

2.8e-051e-053.1e-05

1.6e-056e-064e-06

3e-061.7e-053e-06

3e-061.7e-053e-06

0.0027910.0054960.003450.0073290.0028320.0026830.004821

0.0010250.0010470.0011770.0037150.0003540.0009850.000449

0

3e-062.9e-052e-06

3e-061e-06

1.6e-050

7e-060.000410.0020080.0002618e-06

2e-062.9e-052e-06

0.001010.0010470.0007670.0017070.0009720.000449

1.9e-05

0.0017490.0044490.0022730.0036140.0024020.0016810.004372

0.0017460.0044490.0022730.0036140.0022910.0016790.004232

1e-068e-060

2e-060.0001032e-060.00014

1e-053.2e-051.3e-05

1e-068e-060

1e-061e-061e-06

5e-069e-065e-06

3e-061.1e-057e-06

3e-06

7e-064.4e-054e-06

6e-061.1e-054e-06

3e-05

1e-063e-060

5.4e-057.9e-053.6e-05

5.4e-057.9e-053.6e-05

5.4e-057.9e-053.6e-05

0.0001670.0003110.0001511.7e-05

0.0001670.0003110.0001511.7e-05

1.5e-050.0001151.2e-05

1.5e-050.0001151.2e-05

0.0001260.0001020.0001161.7e-05

0.0001260.0001020.0001161.7e-05

2.6e-059.4e-052.3e-05

2.6e-059.4e-052.3e-05

0.0012840.000210.0017450.0034140.0025350.0012740.000226

0.0002830.0001230.0001620.0015060.0003990.0002690.00013

0.0002830.0001230.0001620.0015060.0001320.0002690.00013

0.0002830.0001230.0001620.0015060.000110.0002690.00013

2.2e-05

4.3e-05

4.3e-05

4.9e-05

2.1e-05

2.8e-05

0.000175

3.7e-05

3e-05

2.8e-05

3.9e-05

1.4e-05

2.7e-05

0.0010018.7e-050.0015830.0019080.0021360.0010059.6e-05

3.5e-05

3.5e-05

1.9e-054e-055.7e-05

1.9e-054e-055.7e-05

0.0003538.7e-050.0013510.0019080.0012260.0003591.6e-05

3.2e-05

3.3e-05

5.8e-05

0.0003538.7e-050.0004330.0019085.2e-050.0003591.6e-05

1.7e-05

0.0009180.001034

0.0002680.0002320.0003710.0002546.4e-05

0.0001290.0001146.7e-050.000127

0.0001390.0001185.5e-050.0001271.6e-05

4.8e-05

4.8e-05

7.9e-05

6.9e-05

5.3e-05

0.0002310.0002340.000221.6e-05

7.3e-052e-056.9e-05

4.6e-05

1.8e-05

1.4e-05

3.4e-05

2.3e-05

8.1e-051.2e-057.9e-05

7.7e-054.5e-057.2e-051.6e-05

2.2e-05

4.5e-05

4.5e-05

0.000130.0001440.000115

7.2e-053.7e-056.1e-05

5.8e-051.7e-055.4e-05

7.5e-05

1.5e-05

4.1e-05

4.1e-05

0.0977780.1331070.0643270.0405630.0793590.0938110.04793

0.0001140.0004910.0002420.0001010.000553

5.9e-059.7e-055.1e-05

5.9e-059.7e-055.1e-05

9e-067.9e-051.5e-05

9e-067.9e-051.5e-05

0.0004910.000553

0.0004910.000553

4.6e-056.6e-053.5e-05

4.6e-056.6e-053.5e-05

0.0976640.1331070.0638360.0405630.0791170.093710.047377

8.1e-05

8.1e-05

0.0001630.000296

0.000112

0.0001630.000184

5.1e-050.0001154.4e-05

5.1e-050.0001154.4e-05

0.0007640.0008860.0019150.0019080.000570.0007440.002285

0.0001570.0001310.000130.000161

0.0011420.001287

0.0006070.0008860.0006420.0019080.000440.0005830.000998

0.0001860.00021

0.0001860.00021

2.2e-05

2.2e-05

0.0001280.000144

0.0001280.000144

5.5e-057.7e-054.9e-05

2.8e-058e-061.5e-05

2.7e-056.9e-053.4e-05

0.00012

0.00012

0.0001560.000176

0.0001560.000176

1.9e-051.6e-05

1.9e-051.6e-05

0.0001530.000172

0.0001530.000172

0.0001099.5e-050.000106

0.0001099.5e-050.000106

1.5e-05

1.5e-05

4.3e-050.000134.3e-05

2.4e-054.2e-052.5e-05

1.9e-058.8e-051.8e-05

4.8e-05

4.8e-05

1.5e-05

1.5e-05

7.7e-05

7.7e-05

0.0004330.000488

0.0004330.000488

0.0960180.1322210.0600510.0386550.0758510.0921150.044467

2.6e-050.0009740.0010982.7e-05

0.0959920.1322210.0587510.0386550.0743860.0920880.044467

0.0003260.000367

0.0001510.000171

0.0001510.000171

4.9e-054.2e-054.5e-05

4.9e-054.2e-054.5e-05

0.0001220.000110.0001370.0001151.5e-05

0.0001220.000110.0001370.0001151.5e-05

0.000130.000146

0.000130.000146

0.0002420.0001260.0002010.0002332.4e-05

7.7e-058.8e-057.2e-05

0.0001650.0001260.0001130.0001612.4e-05

0.0001920.0001340.0002580.00021.5e-05

4.1e-05

5.3e-054.3e-055.4e-05

0.0001390.0001340.0001740.0001461.5e-05

3.1e-05

3.1e-05

1.2e-054.7e-057e-06

1.2e-054.7e-057e-06

1.2e-054.7e-057e-06

1.2e-054.7e-057e-06

6.8e-050.0003865.8e-05

6.8e-050.0003865.8e-05

2.6e-050.0001992.2e-05

1.8e-059.7e-051.8e-05

8e-060.0001024e-06

4.2e-050.0001873.6e-05

2e-059.5e-051.7e-05

2.2e-059.2e-051.9e-05

0.0001120.000239

0.000113

0.000113

0.000113

0.0001120.000126

0.0001120.000126

0.0001120.000126

0.0043350.0036310.0037740.0031120.005210.0041520.002802

0.0004363.5e-050.0002670.0007820.00042

2e-060.0001313e-06

2e-063e-063e-06

0.000108

2e-05

5.8e-053.6e-056e-05

3.3e-051.9e-053.5e-05

2.5e-051.7e-052.5e-05

6.7e-056.3e-055.9e-05

6.7e-056.3e-055.9e-05

7.1e-050.0001146.1e-05

7.1e-056.1e-056.1e-05

5.3e-05

0.0001450.000140.0001990.000141

0.0001450.000140.0001990.000141

0.0001270.000143

0.0001270.000143

9.3e-053.5e-059.6e-059.6e-05

9.3e-053.5e-059.6e-059.6e-05

0.0038990.0035960.0035070.0031120.0044280.0037320.002802

0.000150.000169

0.000150.000169

0.000190.0002170.0001721.6e-05

5e-05

8.5e-050.0001197.7e-05

0.0001054.8e-059.5e-051.6e-05

0.0001310.0001160.0001320.000128

0.0001310.0001160.0001320.000128

0.0002260.000254

0.0002260.000254

2.4e-059e-051.5e-05

2.4e-059e-051.5e-05

0.0032810.0035830.0028730.0031120.0031180.003150.002768

0.000180.0001540.0001650.000175

0.0031010.0035830.0027190.0031120.0029530.0029750.002768

5.1e-050.0001234.3e-05

5.1e-050.0001234.3e-05

0.0001420.00016

0.0001420.00016

0.0002221.3e-050.0001650.0002241.8e-05

4.9e-05

1.8e-05

0.000116.3e-050.000116

0.0001121.3e-053.5e-050.0001081.8e-05

2.7e-050.0001643.4e-05

2.7e-050.0001643.4e-05

2.7e-050.0001643.4e-05

2.7e-050.0001643.4e-05

1.2e-054.2e-052.1e-05

1.2e-054.2e-052.1e-05

4e-063.8e-053e-06

4e-063.8e-053e-06

7e-063.9e-058e-06

7e-063.9e-058e-06

4e-064.5e-052e-06

4e-064.5e-052e-06

0.0031340.0018120.0018730.0021080.0029820.0029550.001479

0.0031340.0018120.0018730.0021080.0029820.0029550.001479

0.002960.0018120.0018730.0021080.002650.0028310.001479

0.002960.0018120.0018730.0021080.002650.0028310.001479

1.3e-053.1e-051.1e-05

1.3e-053.1e-051.1e-05

1.6e-055.5e-051.1e-05

1e-052.8e-056e-06

6e-062.7e-055e-06

2e-054.7e-051.7e-05

2e-054.7e-051.7e-05

0.0029110.0018120.0018730.0021080.0025170.0027920.001479

2e-06

1.4e-055.6e-05

0.0028960.0018120.0018730.0021080.0024490.0027780.001423

6e-062e-057e-06

3e-069e-063e-06

6e-062.3e-054e-06

0.0001740.0003320.000124

3e-055.1e-052.5e-05

3e-055.1e-052.5e-05

3e-055.1e-052.5e-05

0.0001440.0002819.9e-05

0.0001440.0002819.9e-05

1.9e-053.7e-051.5e-05

2.9e-053.1e-052.2e-05

1.7e-05

1e-052.2e-057e-06

1.1e-05

3.3e-053.9e-051.9e-05

1.1e-053.4e-058e-06

2.2e-053.7e-051.5e-05

2e-051.9e-051.3e-05

1e-05

2.4e-05

3e-064.5e-051e-06

3e-064.5e-051e-06

3e-064.5e-051e-06

3e-064.5e-051e-06

3e-064.5e-051e-06

3e-064.5e-051e-06

0.0102960.0209920.0175290.0344370.0069760.0100280.013624

0.0102960.0209920.0175290.0344370.0069760.0100280.013624

3e-066.8e-053e-06

3e-066.8e-053e-06

2e-063.8e-051e-06

2e-061.9e-051e-06

1.9e-05

1e-063e-052e-06

1e-063e-052e-06

0.0102750.0209920.0175290.0344370.0068170.0100020.013624

1.9e-058.2e-051.5e-05

1.1e-054.4e-051e-05

4e-062.4e-053e-06

7e-062e-057e-06

8e-063.8e-055e-06

8e-06

6e-062.2e-054e-06

2e-068e-061e-06

0.0102560.0209920.0175290.0344370.0067350.0099870.013624

0.0076370.0159480.012350.0304210.0057090.0074440.009974

0.0009430.0011860.0006160.0048192.2e-050.0009270.000249

0.0049440.005571

7e-06

0.0004170.000150.0003060.0090365e-060.000413

1.2e-05

0.0016110.0014280.0006380.0055228e-060.0015690.000207

9e-06

0.0008780.001260.0008420.0046189e-060.0008620.00051

0.0037880.0119240.0050040.0064264.7e-050.0036730.009008

1.9e-05

0.0026190.0050440.0051790.0040160.0010260.0025430.00365

2e-063e-053e-06

6e-068e-066e-06

8e-060.003244.6e-059e-060.00365

0.0026030.0050440.0019390.0040160.0009420.002525

1.8e-059.1e-052.3e-05

1.8e-059.1e-052.3e-05

1.4e-052.6e-051.4e-05

1.4e-052.6e-051.4e-05

3e-05

3e-05

4e-063.5e-059e-06

4e-063.5e-059e-06

2.2e-050.0001441.2e-05

2.2e-050.0001441.2e-05

2.2e-050.0001441.2e-05

2.2e-050.0001441.2e-05

3.6e-05

3.6e-05

1.3e-057.3e-055e-06

3.2e-05

1.3e-054.1e-055e-06

9e-063.5e-057e-06

9e-063.5e-057e-06

2.5e-057.8e-051.1e-05

2.5e-057.8e-051.1e-05

2.5e-057.8e-051.1e-05

2.5e-057.8e-051.1e-05

2.5e-057.8e-051.1e-05

2.5e-057.8e-051.1e-05

0.0054440.0078820.0070660.010040.0037340.0103250.006266

0.0054440.0078820.0070660.010040.0037340.0103250.006266

0.0013330.0028840.0033130.005020.0012220.0012340.002547

0.000140.0006050.000125

7.5e-050.0003547.2e-05

5e-064.2e-054e-06

3.6e-057.3e-053.6e-05

1.9e-05

4e-062.9e-053e-06

2e-06

5.1e-05

5e-065.6e-054e-06

1e-06

2e-051.8e-051.8e-05

2e-063.1e-053e-06

3e-063.2e-054e-06

1.4e-056e-051.1e-05

6e-062.8e-055e-06

8e-063.2e-056e-06

1.1e-053.6e-058e-06

1.1e-053.6e-058e-06

4e-063.2e-054e-06

4e-063.2e-054e-06

3.6e-050.0001233e-05

2.1e-053.8e-051.3e-05

9e-065.7e-051.1e-05

6e-062.8e-056e-06

0.0011930.0028840.0033130.005020.0006170.0011090.002547

7.2e-051.9e-053.4e-05

7e-067e-06

3e-06

1e-06

3.7e-051e-05

2.1e-051e-062.2e-05

1e-061e-060

6e-063e-065e-06

0.0011210.0028840.0033130.005020.0005980.0010750.002547

1e-063e-061e-06

2.8e-050.0022280.002512.4e-052.6e-05

2.7e-058e-062.4e-05

7e-068e-067e-06

0.0010420.0028840.0010850.002510.0005530.0010010.002547

1.6e-052e-061.6e-05

0.0001356.5e-050.0001091.6e-05

0.0001356.5e-050.0001091.6e-05

0.0001356.5e-050.0001091.6e-05

3.3e-053.4e-053.3e-058e-06

4e-061.1e-054e-068e-06

6.9e-0504.3e-05

2.9e-052e-052.9e-05

0.0039760.0049980.0037530.005020.0024470.0089820.003703

0.0039760.0049980.0037530.005020.0024470.0089820.003703

9e-064.6e-058e-06

9e-064.6e-058e-06

0.0039670.0049980.0037530.005020.0024010.0089740.003703

2e-063.1e-051e-06

1.8e-05

0.0011630.0019720.0008410.0023090.0003240.001280.001508

0.0008240.000928

0.0028020.0030260.0020880.0027110.00110.0076930.002195

1.5e-055.5e-051.2e-05

1.5e-055.5e-051.2e-05

1.5e-055.5e-051.2e-05

1.5e-055.5e-051.2e-05

1.5e-055.5e-051.2e-05

1.5e-055.5e-051.2e-05

6.1e-050.0003026.9e-05

6.1e-050.0003026.9e-05

6.1e-050.0003026.9e-05

2.2e-056.6e-052e-05

9e-064.8e-056e-06

9e-064.8e-056e-06

1.3e-051.8e-051.4e-05

1.3e-051.4e-051.4e-05

4e-06

3.9e-050.0002364.9e-05

1.5e-059.5e-051.7e-05

3.8e-05

1.5e-052.7e-051.7e-05

3e-05

4.7e-05

4.7e-05

2e-055.2e-052.3e-05

2e-055.2e-052.3e-05

4e-064.2e-059e-06

4e-064.2e-059e-06

6.4e-05

6.4e-05

6.4e-05

6.4e-05

6.4e-05

6.4e-05

0.0018650.0010910.0011950.0019080.0016510.001790.000769

0.0018650.0010910.0011950.0019080.0016510.001790.000769

0.0018650.0010910.0011950.0019080.0016510.001790.000769

0.0018650.0010910.0011950.0019080.0016510.001790.000769

0.0018650.0010910.0011950.0019080.0016510.001790.000769

0.0018640.0010910.0011950.0019080.0016180.0017880.000769

1e-063.3e-052e-06

0.08440800000000010.0569950.0516030.0508010.0796220.08121400000000010.050449

0.0395680.0321680.0256990.0261040.0382030.0379550.027994

0.0395680.0321680.0256990.0261040.0382030.0379550.027994

0.0372420.0213550.0215310.0164660.0357150.0357230.017591

2.7e-05

1e-05

1e-05

7e-06

7e-06

1e-05

1e-05

0.0372420.0213550.0215310.0164660.0356880.0357230.017591

1e-061.7e-050

1e-061.7e-050

07e-060

05e-060

2e-06

02.9e-050

01.1e-050

01.8e-050

0.037240.0213550.0215310.0164660.0356160.0357220.017591

0.037240.0213550.0215310.0164660.0356160.0357220.017591

1e-061.9e-051e-06

1e-061.9e-051e-06

2e-062.5e-052e-06

2e-061.3e-052e-06

2e-061.3e-052e-06

2e-061.3e-052e-06

01.2e-050

01.2e-050

01.2e-050

0.0018840.0095020.0025370.0061240.0016350.001810.009248

0.0018840.0095020.0025370.0061240.0016350.001810.009248

01.1e-050

2e-06

09e-060

0.0018840.0095020.0025370.0061240.001610.001810.009248

1e-061.5e-051e-06

1.5e-05

0.0018820.0095020.0025370.0061240.0015690.0018080.009231

04e-060

1e-067e-061e-061.7e-05

01.4e-050

01.4e-050

0.000440.0013110.0016310.0035140.0008280.000420.001155

0.0004340.0013110.0016310.0035140.0008070.0004160.001155

9e-060.0010730.0013120.0019080.0002949e-060.000928

9e-060.0002610.0002949e-06

0.0010730.0010510.0019080.000928

1.3e-059.5e-051.2e-05

2e-063.5e-052e-06

1.1e-056e-051e-05

01.7e-050

08e-060

9e-060

0.0004120.0002380.0003190.0016060.0004010.0003950.000227

0.0004120.0002380.0003190.0016060.0004010.0003950.000227

6e-062.1e-054e-06

6e-062.1e-054e-06

1e-06

6e-061.1e-054e-06

8e-06

1e-060

3e-060.000123e-06

3e-060.000123e-06

8.7e-05

8.7e-05

5.4e-05

5.4e-05

3.3e-05

3.3e-05

3e-063.3e-053e-06

3e-063.3e-053e-06

3e-063.3e-053e-06

3e-062.5e-053e-06

8e-06

2.2e-058.1e-051.5e-05

2.2e-058.1e-051.5e-05

2.2e-058.1e-051.5e-05

2.2e-058.1e-051.5e-05

2.2e-058.1e-051.5e-05

2.2e-058.1e-051.5e-05

1e-062.6e-050

1e-062.6e-050

1e-062.6e-050

1e-062.6e-050

1e-062.6e-050

1e-062.6e-050

0.0448140.0248270.0259040.0246970.0411920.0432410.022455

6.3e-050.0002886.2e-05

2e-067.8e-052e-06

2e-067.8e-052e-06

2.6e-05

2.6e-05

1e-063.4e-051e-06

1e-063.4e-051e-06

1e-061.8e-051e-06

1e-061.8e-051e-06

6.1e-050.0001766e-05

2e-062.7e-053e-06

2e-062.7e-053e-06

2e-062.7e-053e-06

5.7e-050.0001125.5e-05

5.7e-050.0001125.5e-05

4.8e-057.3e-054.7e-05

9e-063.9e-058e-06

2e-061.5e-052e-06

2e-061.5e-052e-06

2e-061.5e-052e-06

02.2e-050

02.2e-050

02.2e-050

3.4e-05

3.4e-05

3.4e-05

3.4e-05

6e-067.7e-055e-06

6e-067.7e-055e-06

6e-067.7e-055e-06

6e-064.4e-055e-06

1e-061.4e-050

4e-061.4e-054e-06

1e-061e-051e-06

06e-060

2.3e-05

1.1e-05

1.2e-05

1e-05

1e-05

0.0040010.0023850.0025090.002510.00390.0038460.001986

3.7e-050.0001244.5e-05

7e-062.4e-056e-06

7e-062.4e-056e-06

7e-062.4e-056e-06

2.1e-054.1e-053.1e-05

2e-052.8e-053.1e-05

1.9e-051.6e-051.7e-05

1e-061.2e-051.4e-05

1e-061.3e-050

1e-061.3e-050

01.3e-050

01.3e-050

01.3e-050

9e-064.6e-058e-06

9e-062.9e-058e-06

7e-061.4e-056e-06

4e-06

2e-061.1e-052e-06

01.7e-050

01.7e-050

1.3e-050.0002321.4e-05

9e-060.0001761e-05

1e-061.2e-051e-06

1e-061.2e-051e-06

02.2e-051e-06

01.4e-051e-06

8e-06

6e-061.5e-056e-06

6e-061.5e-056e-06

03e-050

03e-050

2e-061.7e-052e-06

2e-061.7e-052e-06

06.8e-050

02e-060

06e-060

02e-060

1e-05

3e-06

9e-06

1e-06

1.3e-05

1e-06

1.2e-05

4e-06

5e-06

01.2e-050

01.2e-050

4e-065.6e-054e-06

4e-065.6e-054e-06

1e-062.8e-051e-06

3e-062.8e-053e-06

0.0039510.0023850.0025090.002510.0035440.0037870.001986

0.0039510.0023850.0025090.002510.0035440.0037870.001986

0.0039510.0023850.0025090.002510.0035440.0037870.001986

0.003950.0023850.0025090.002510.0035230.0037860.001986

1e-062.1e-051e-06

0.0012510.0007360.0010140.0018070.0012240.001199

0.0012510.0007360.0010140.0018070.0012240.001199

0.0012510.0007360.0010140.0018070.0012240.001199

0.0012510.0007360.0010140.0018070.0012240.001199

0.001250.0007360.0010140.0018070.0012020.001199

1e-062.2e-050

0.0027150.0023170.0021520.0039150.002210.0026070.002049

0.0027150.0023170.0021520.0039150.002210.0026070.002049

0.0027150.0023170.0021520.0039150.002210.0026070.002049

4e-06

4e-06

0.0015390.0008790.0010190.0018070.0011810.0014760.00077

6e-06

03e-060

03e-060

3e-06

4e-060

4e-06

4e-06

6e-06

5e-068e-065e-06

3e-06

2e-065e-062e-06

5e-06

03e-060

06e-060

3e-06

0.0015320.0008790.0010190.0018070.0011090.0014690.00077

6e-06

0.0011760.0014380.0011330.0021080.0010250.0011310.001279

0.0011760.0014380.0011330.0021080.0009850.0011310.001279

9e-06

02e-060

5e-06

3e-06

9e-06

01.2e-050

0.0035090.0017310.0019350.0036140.0035980.0034060.001124

0.0003634.7e-050.0005440.0003741.4e-05

0.0003634.7e-050.0005440.0003741.4e-05

3e-054.3e-053.1e-05

3e-054.3e-053.1e-05

4.1e-055.3e-054.2e-051.4e-05

4.1e-055.3e-054.2e-051.4e-05

3.8e-05

3.8e-05

3.6e-055e-053.7e-05

3.6e-055e-053.7e-05

0.000184.7e-050.0002220.000188

0.0001014.7e-050.0001190.000104

7.9e-050.0001038.4e-05

1.4e-054.5e-051.4e-05

1.4e-054.5e-051.4e-05

3.2e-055e-053.3e-05

3.2e-055e-053.3e-05

3e-054.3e-052.9e-05

3e-054.3e-052.9e-05

0.0006890.0003350.0005060.0016060.0006820.0006670.000199

0.0006680.0003350.0003530.0016060.0005090.0006440.000199

1.1e-055.1e-051.1e-05

1.1e-055.1e-051.1e-05

9e-065.8e-051e-05

9e-065.8e-051e-05

0.0006480.0003350.0003530.0016060.00040.0006230.000199

5.3e-05

1.6e-053.6e-051.6e-05

0.0006320.0003350.0003530.0016060.0003110.0006070.000199

2.1e-050.0001530.0001732.3e-05

2.1e-050.0001530.0001732.3e-05

2.1e-050.0001530.0001732.3e-05

0.0024570.0013490.0014290.0020080.0023720.0023650.000911

0.0024390.0013490.0014290.0020080.0021980.0023460.000911

1.2e-051.9e-051.1e-05

6e-061e-065e-06

3e-06

6e-061.5e-056e-06

0.0023480.0013490.0014290.0020080.0019720.0022540.000911

0.0023380.0013490.0014290.0020080.0019420.0022420.000911

1e-053e-051.2e-05

6.3e-050.000166.1e-05

3.4e-058.3e-053.4e-05

2.9e-057.7e-052.7e-05

1.6e-054.7e-052e-05

1.6e-054.7e-052e-05

1.8e-050.0001481.9e-05

8e-065.3e-058e-06

8e-065.3e-058e-06

1e-059.5e-051.1e-05

3.5e-05

1e-052.1e-051.1e-05

3.9e-05

2.6e-05

2.6e-05

2.6e-05

9e-068.4e-051.2e-05

9e-068.4e-051.2e-05

3e-063.3e-054e-06

2e-062.6e-053e-06

07e-061e-06

2e-067e-062e-06

01e-060

8e-06

2e-06

01e-060

1e-067e-061e-06

1e-067e-061e-06

6e-065.1e-058e-06

08e-060

08e-060

6e-064.3e-058e-06

1e-061.1e-051e-06

2e-065e-063e-06

2e-061.6e-052e-06

1e-061.1e-052e-06

0.033260.0176580.0182940.0128510.0298110.0321040.017296

0.033260.0176580.0182940.0128510.0298110.0321040.017296

0.0332570.0176580.0182940.0128510.0297970.0321010.017296

4e-064.8e-054e-06

3e-061.1e-053e-06

02.3e-050

3e-06

1e-061.1e-051e-06

0.0318210.016750.0172990.0110440.0284470.0307230.016626

9.3e-05

6e-063.1e-056e-06

0.0318040.016750.0171810.0110440.0281270.0307070.016626

1.1e-053.5e-051e-05

2.8e-05

0.0001180.000133

1e-061.9e-050

1e-064e-060

1.4e-05

1e-06

0.0014310.0009080.0009950.0018070.0012830.0013740.00067

0.0014310.0009080.0009950.0018070.0012830.0013740.00067

3e-061.4e-053e-06

3e-061.4e-053e-06

3e-061.4e-053e-06

0.0002580.0001230.000218

0.0002580.0001230.000218

0.0002580.0001230.000218

0.0002580.0001230.000218

0.0001650.0001230.00012

0.0001650.0001230.00012

0.0001650.0001230.00012

9.3e-059.8e-05

9.3e-059.8e-05

9.3e-059.8e-05
