# Supplementary material for: MetaMeta: integrating metagenome analysis tools to improve taxonomic profiling
Source: Microbiome. 2017 Aug 14;5:101. doi: 10.1186/s40168-017-0318-y (PMC5557516; doi:10.1186/s40168-017-0318-y)
Supplement: Supplementary file 2 — Additional File with interactive charts for all CAMI toy set results on default, very-precise and very-sensitive mode. File prefix S, M, and H for low, medium and high complexity, respectively. (TAR 3573 kb) [file 40168_2017_318_MOESM2_ESM.tar › H_S003__insert_180_very-sensitive.html]

Javascript must be enabled to view this page.

magnitude
magnitudeUnassigned

clark.parsed\_profile
dudes.parsed\_profile
final.metametamerge.profile
gottcha.parsed\_profile
kaiju.parsed\_profile
kraken.parsed\_profile
motus.parsed\_profile

1.0000150.9999900000000011.0000070.9999970.9999999999999970.9999959999999990.999999999999999

0.9192459999999980.9464020000000010.9515000000000010.9527190.9237199999999980.9225109999999990.955089999999999

2.3e-050.0001411.4e-05

2.3e-050.0001411.4e-05

2.3e-050.0001411.4e-05

2.3e-050.0001411.4e-05

1.3e-057.1e-056e-06

3.2e-05

1.3e-053.9e-056e-06

3.5e-05

3.5e-05

1e-053.5e-058e-06

1e-053.5e-058e-06

3e-064.4e-052e-06

3e-064.4e-052e-06

3e-064.4e-052e-06

3e-064.4e-052e-06

3e-064.4e-052e-06

3e-064.4e-052e-06

0.0071350.0051670.0438170.005320.051060.007320.003275

3.3e-050.0001393.2e-05

3.3e-050.0001393.2e-05

3.3e-050.0001393.2e-05

3.3e-050.0001393.2e-05

1.7e-057.1e-051.7e-05

1.6e-056.8e-051.5e-05

0.0071020.0051670.0438170.005320.0509210.0072880.003275

0.0002420.0001410.000830.000248

3.9e-052.6e-054.2e-05

3.9e-052.6e-054.2e-05

3.9e-051.4e-054.2e-05

1.2e-05

2.4e-055e-052.6e-05

2.4e-055e-052.6e-05

2.4e-055e-052.6e-05

0.0001160.0001410.0006350.00012

0.0001160.0001410.0005490.00012

0.0001160.0001410.0005490.00012

8.6e-05

4.3e-05

4.3e-05

6.3e-050.0001196e-05

2.9e-056.8e-053.3e-05

2.9e-056.8e-053.3e-05

3.4e-055.1e-052.7e-05

3.4e-055.1e-052.7e-05

3.4e-05

3.4e-05

3.4e-05

3.4e-05

0.0025230.004650.0018980.005320.002860.002440.00326

1.7e-057.5e-051.4e-05

1.7e-057.5e-051.4e-05

1.7e-057.5e-051.4e-05

0.0002980.0007330.000293

2.9e-057.8e-053e-05

2.9e-057.8e-053e-05

0.0001880.0005150.000195

1.5e-05

1.9e-05

2e-06

2.2e-058.3e-052.4e-05

1.5e-056.9e-051.1e-05

4e-06

8e-06

1.3e-051.6e-051.2e-05

2.1e-05

2e-0502.1e-05

4e-061e-053e-06

7e-061e-059e-06

5e-063.3e-056e-06

1.3e-051.6e-051.3e-05

0

2e-06

2.5e-059e-062.6e-05

9e-06

2e-0502.1e-05

2.6e-050.0001022.9e-05

1.8e-058.4e-052e-05

3e-06

3.1e-055.3e-052.1e-05

3.1e-055.3e-052.1e-05

5e-058.7e-054.7e-05

1.4e-051.4e-05

3.6e-058.7e-053.3e-05

4.8e-054.8e-05

4.8e-054.8e-05

4.8e-054.8e-05

0.0012530.0042430.0014210.0036140.0008350.0011990.003144

0.0012530.0042430.0014210.0036140.0008350.0011990.003144

3.8e-05

0.0012530.0042430.0014210.0036140.0007970.0011990.003144

0.0008840.0004070.0003510.0017060.0009010.0008640.000116

0.0008840.0004070.0003510.0017060.0009010.0008640.000116

0.0008840.0004070.0003510.0017060.0009010.0008640.000116

1.7e-050.0001260.0003011.6e-05

1.7e-050.0001260.0003011.6e-05

0.0001260.000144

1.7e-055.1e-051.6e-05

0.000106

6e-061.5e-056e-06

6e-061.5e-056e-06

6e-069e-066e-06

3e-06

3e-06

0.0001430.0001670.0004530.000151

0.0001430.0001670.0004530.000151

0.0001430.0001670.0004530.000151

0.0001430.0001670.0004530.000151

0.0035260.0005170.0409630.0452390.0037391.5e-05

0.0008423.1e-050.0007670.0006850.000892

0.0002230.0001710.000150.000232

0.0002230.0001710.000150.000232

0.0006193.1e-050.0005960.0005350.00066

0.0002050.0001670.000160.000214

0.0004143.1e-050.0002620.0001850.000446

0.0001670.00019

0.0382020.043381

0.0382020.043381

0.0382020.043381

0.0026840.0004860.0019940.0011730.0028471.5e-05

0.0015090.0002980.0010660.0007930.0016561.5e-05

0.0003166.2e-050.0001960.0001320.000362

0.0001250.000142

0.0003858e-050.0002280.0001510.000422

0.0003796.3e-050.0002390.000170.0003981.5e-05

0.0004299.3e-050.0002780.0001980.000474

0.0003453.8e-050.0003220.000388

0.0003453.8e-050.0003220.000388

0.0006350.000150.0004250.0003150.000586

0.0002180.0002025.8e-050.000241

8.3e-05

0.0001039e-050

0.0003146e-050.0002230.0001740.000345

0.0001950.0001816.5e-050.000217

0.0001950.0001816.5e-050.000217

0.0002060.0001090.0002930.000214

9.7e-050.0001090.0001570.000103

9.7e-050.0001090.0001570.000103

9.7e-050.0001090.0001570.000103

0.0001090.0001360.000111

0.0001090.0001360.000111

0.0001090.0001360.000111

0.0004620.0005390.0012120.000496

0.0001090.0001640.000240.000114

2.2e-055.3e-052.4e-05

2.2e-055.3e-052.4e-05

5.6e-050.0001640.0001875.7e-05

5.6e-050.0001640.0001875.7e-05

3.1e-053.3e-05

3.1e-053.3e-05

2.1e-050.0001270.0001441.9e-05

2.1e-050.0001270.0001441.9e-05

2.1e-050.0001270.0001441.9e-05

8.9e-050.000120.0002388.8e-05

8.9e-050.000120.0002388.8e-05

3.5e-050.0001023.5e-05

5.4e-050.000120.0001365.3e-05

0.0001140.0001280.0002350.000131

0.0001140.0001280.0002350.000131

0.0001140.0001280.0002350.000131

0.0001290.0003550.000144

0.0001290.0003550.000144

1.8e-050.0001162.1e-05

4e-061.1e-055e-06

8e-065e-069e-06

3.1e-058e-053.3e-05

2.6e-058.5e-053.1e-05

4.2e-055.8e-054.5e-05

2e-063.7e-051e-06

2e-063.7e-051e-06

2e-063.7e-051e-06

2e-063.7e-051e-06

2e-063.7e-051e-06

2e-063.7e-051e-06

0.0047250.0029330.0033950.0028110.0048850.004526

0.0047250.0029330.0033950.0028110.0048850.004526

0.0047250.0029330.0033950.0028110.0048850.004526

0.0047220.0029330.0032870.0028110.0047620.004522

0.0047220.0029330.0032870.0028110.0047620.004522

0.0047220.0029330.0032870.0028110.0047620.004522

3e-060.0001080.0001234e-06

3e-060.0001080.0001234e-06

3e-060.0001080.0001234e-06

0.0221850.0159840.0347250.0276040.0393160.0216290.01679

0.0049510.0030430.0045460.0028110.0066960.004740.0022

0.0049510.0030430.0045460.0028110.0066960.004740.0022

0.0049510.0030430.0045460.0028110.0066960.004740.0022

0.0049350.0030430.0029910.0028110.004690.0047230.0022

0.0049350.0030430.0029910.0028110.004690.0047230.0022

9.8e-05

9.8e-05

1.6e-056.9e-051.7e-05

1.6e-056.9e-051.7e-05

0.0015550.001766

0.0007510.000853

0.0008040.000913

7.3e-05

7.3e-05

1.1e-059.3e-051.4e-05

1.1e-059.3e-051.4e-05

7e-063.8e-059e-06

7e-063.8e-059e-06

7e-063.8e-059e-06

4e-065.5e-055e-06

4e-065.5e-055e-06

4e-065.5e-055e-06

0.0029090.001620.0027990.0021080.0048430.002811.6e-05

0.0029090.001620.0027990.0021080.0048430.002811.6e-05

0.000348

0.000186

3.4e-05

1.3e-05

3.1e-05

2.3e-05

3.9e-05

3.5e-05

1.1e-05

8.5e-05

2.8e-05

3e-05

2.7e-05

7.7e-05

3.8e-05

3.9e-05

1.4e-053.9e-051.7e-05

1.4e-053.9e-051.7e-05

1.4e-053.9e-051.7e-05

1.7e-050.0001582e-05

1.7e-055.7e-052e-05

1.7e-055.7e-052e-05

4.9e-05

4.9e-05

5.2e-05

5.2e-05

1.3e-050.000131.1e-05

7e-066.2e-055e-06

7e-066.2e-055e-06

6e-066.8e-056e-06

6e-066.8e-056e-06

0.0001483.1e-050.0006160.0009490.0001551.6e-05

1.2e-055.1e-051.3e-05

1.2e-055.1e-051.3e-05

5e-05

5e-05

8e-066.2e-051e-05

8e-066.2e-051e-05

0.0001283.1e-050.0006160.0007860.0001321.6e-05

0.0004810.000546

0.0001283.1e-050.0001350.000240.0001321.6e-05

0.0027170.0015890.0021830.0021080.0031680.002607

1.3e-058.3e-051.3e-05

1.3e-058.3e-051.3e-05

2.2e-055.6e-052e-05

2.2e-055.6e-052e-05

3.4e-053.4e-05

3.4e-053.4e-05

8e-060.0001078e-06

8e-060.0001078e-06

1.5e-058.8e-051.6e-05

1.5e-058.8e-051.6e-05

0.0026250.0015890.0019360.0021080.0025530.002516

0.0026250.0015890.0019360.0021080.0025530.002516

0.0002470.000281

0.0002470.000281

5.1e-05

5.1e-05

5.1e-05

0.0062820.0065330.0134560.0065240.0144920.006190.007506

0.0062820.0065330.0134560.0065240.0144920.006190.007506

0.0001490.0001950.000143

0.0001490.0001950.000143

1.8e-052.5e-051.9e-05

3.6e-054.8e-052.5e-05

2.6e-053.9e-052.2e-05

7e-061.3e-051.2e-05

2.1e-053e-052.3e-05

9e-061.6e-058e-06

2.5e-051.6e-052.8e-05

7e-068e-066e-06

2.1e-056.5e-052.1e-05

2.1e-056.5e-052.1e-05

2.1e-056.5e-052.1e-05

0.0060960.0065330.0134560.0065240.0140750.0060090.007498

0.0001290.000147

0.0001290.000147

0.0001810.0001850.000280.000194

0.0001810.0001850.000280.000194

1.7e-059.7e-052e-05

1.7e-059.7e-052e-05

2.2e-058.2e-052e-05

2.2e-058.2e-052e-05

3.2e-055.8e-054e-05

3.2e-055.8e-054e-05

3.2e-050.0001430.0001633.4e-05

3.2e-050.0001430.0001633.4e-05

0.00140.00159

0.00140.00159

1.7e-050.0001321.8e-05

7.2e-05

1.7e-056e-051.8e-05

8e-06

8e-06

8.1e-050.0001660.0001898.5e-05

8.1e-050.0001660.0001898.5e-05

0.000107

2e-05

8.7e-05

0.0002220.000462

0.0002220.000252

8.4e-05

0.000126

9.6e-05

9.6e-05

0.0032020.0016760.0016040.0022080.0012130.0030910.00144

1.6e-05

0.0031660.0016760.0011730.0022080.0004820.0030530.001356

3.6e-050.0001153.8e-05

6.8e-05

0.000127

0.0004310.000489

3e-050.0001350.0001543.7e-05

3e-050.0001350.0001543.7e-05

8.8e-05

8.8e-05

2.5e-050.0001792.6e-05

1e-05

0.000111

2.5e-055.8e-052.6e-05

2.8e-050.000120.0001373e-05

2.8e-050.000120.0001373e-05

2.1e-058.9e-051.9e-05

2.1e-058.9e-051.9e-05

0.0007740.000879

0.0007740.000879

4e-050.0002280.0002594.7e-05

4e-050.0002280.0002594.7e-05

0.000206

0.000106

0.0001

0.0056870.0064586.3e-05

6.3e-05

0.0028240.003207

0.0007350.000835

0.0014050.001595

0.0007230.000821

0.0018380.0048570.0018770.0043160.0009620.0017630.004397

0.0018380.0048570.0018770.0043160.0009620.0017630.004397

4.1e-050.0001360.0001544.2e-05

4.1e-050.0001360.0001544.2e-05

5.7e-050.0001156.2e-05

5.7e-050.0001156.2e-05

0.0003090.0001080.0004070.000348

4.4e-059.7e-054.9e-05

4.7e-053.3e-055.6e-05

8.1e-054.3e-058.5e-05

9.4e-050.0001080.0001440.000107

4.3e-059e-055.1e-05

0.0001320.00015

0.0001320.00015

0.000115

0.000115

9.2e-050.0001310.0001

3.1e-05

5.5e-055.7e-055.8e-05

3.7e-054.3e-054.2e-05

2.3e-050.0002970.0003372.4e-05

0.0001470.000167

2.3e-050.000150.000172.4e-05

8e-060.0001019e-06

8e-060.0001019e-06

0.0001130.000128

0.0001130.000128

1.6e-056.6e-051.7e-05

1.6e-056.6e-051.7e-05

1.6e-056.6e-051.7e-05

9.1e-058e-06

8e-06

8e-06

9.1e-05

9.1e-05

2.5e-050.0001181.9e-051.5e-05

1.5e-05

1.5e-05

1.5e-05

1.5e-05

2.5e-050.0001181.9e-05

2.5e-050.0001181.9e-05

7e-063.9e-056e-06

7e-063.9e-056e-06

1.8e-057.9e-051.3e-05

1.8e-057.9e-051.3e-05

4.3e-050.0006314.9e-05

4.3e-050.0006314.9e-05

4.3e-050.0006314.9e-05

6.3e-05

6.3e-05

1e-057.8e-051e-05

1e-057.8e-051e-05

1.4e-050.0001051.6e-05

1.4e-050.0001051.6e-05

1e-050.0001791.1e-05

9.5e-05

1e-058.4e-051.1e-05

9e-060.0002061.2e-05

9e-067e-051.2e-05

4.8e-05

4.1e-05

4.7e-05

0.0079640.0047880.0139240.0161610.0124430.0078070.007053

0.0001270.000239

9.5e-05

9.5e-05

9.5e-05

0.0001270.000144

0.0001270.000144

0.0001270.000144

0.0079640.0047880.0137970.0161610.0122040.0078070.007053

0.0011230.0002730.0032340.0036140.0027250.0011460.001616

0.0011230.0002730.0032340.0036140.0027250.0011460.001616

0.0006680.0002730.0004640.0018070.0006830.0006720.000369

0.0003790.00043

0.0007190.000817

0.0002020.000229

5.2e-050.0004460.0005076e-05

9.1e-050.000230.0002619.6e-05

0.0001470.0001980.0018070.000260.000151

8.3e-050.0001088.2e-05

0.0003030.000344

8.2e-050.0002930.0003338.5e-05

0.0003160.000359

0.0003160.000359

0.0003160.000359

0.003840.0021290.0045480.0062240.0017380.0036820.00256

0.0002010.000229

0.0002010.000229

0.003840.0021290.0043470.0062240.0015090.0036820.00256

0.0037660.0021290.0025210.0029110.0036080.00256

7.4e-050.0018260.0033130.0015097.4e-05

1.4e-050.0010280.0011661.4e-05

0.0009110.001034

0.0009110.001034

1.4e-050.0001170.0001321.4e-05

1.4e-050.0001170.0001321.4e-05

0.000120.0010110.0012180.000118

7.6e-050.0008890.0010097.9e-05

0.0001950.000221

7.6e-050.0006940.0007887.9e-05

4.4e-050.0001220.0002093.9e-05

0.0001220.000139

4.4e-057e-053.9e-05

4.8e-050.0002060.0002344.9e-05

4.8e-050.0002060.0002344.9e-05

4.8e-050.0002060.0002344.9e-05

0.0010980.0017530.0012540.0022080.0008470.0010530.001456

0.0010980.0017530.0010610.0022080.0006280.0010530.001456

0.0010720.0017530.0009070.0022080.0004530.0010270.001456

2.6e-050.0001540.0001752.6e-05

0.0001930.000219

0.0001930.000219

0.001710.0006330.0019930.0041150.0036820.0017320.001421

0.001710.0006330.0019930.0041150.0036820.0017320.001421

0.0001540.000174

0.001130.0004130.0007340.0022080.0007370.0011347e-05

9.6e-05

1.6e-05

1.6e-05

0.000110.0001450.000111

0.0002044.1e-050.000260.0016680.000215

7.8e-05

0.000108

0.0001240.00014

7e-05

0.0002180.0001790.0002540.0019070.0002240.0002180.000921

1.6e-05

9.4e-05

0.0002510.000285

4.8e-050.0002160.0002455.4e-05

1.1e-050.0002070.0002351.3e-05

1.1e-050.0002070.0002351.3e-05

1.1e-050.0002070.0002351.3e-05

0.0087150.0074220.0056580.0082310.0079260.0083970.00609

6.7e-05

6.7e-05

6.7e-05

6.7e-05

6.7e-05

0.0087150.0074220.0056580.0082310.0078590.0083970.00609

5.6e-050.0001065.5e-05

5.2e-059.2e-054.9e-05

5.2e-059.2e-054.9e-05

2e-057e-062.1e-05

3e-061.3e-051e-06

9e-067e-067e-06

1.1e-05

0

1.1e-053e-061.1e-05

9e-061e-059e-06

1e-05

9e-06

3e-06

1.1e-05

8e-06

4e-061.4e-056e-06

4e-061.4e-056e-06

4e-061.4e-056e-06

0.0049570.0028150.0029580.0028110.0047750.0047290.002275

0.0049570.0028150.0029580.0028110.0047750.0047290.002275

0.0049180.0028150.0029580.0028110.0047340.0046950.002275

2.8e-054e-051.5e-05

3.8e-05

0.0048830.0028150.0029580.0028110.0046120.0046740.002275

7e-064.4e-056e-06

3.9e-054.1e-053.4e-05

3e-061.4e-052e-06

1e-061e-062e-06

2e-066e-062e-06

1e-06

3.3e-051.9e-052.8e-05

0.0037020.0046070.00270.005420.0029780.0036130.003815

0.0037020.0046070.00270.005420.0029780.0036130.003815

0.0029540.0017930.0019480.0022080.0022420.0029680.001423

0.0028740.0017930.0018270.0022080.0021410.0027510.001423

8e-050.0001210.0001010.000217

0.0007480.0028140.0007520.0032120.0007360.0006450.002392

6e-06

1e-061.2e-051e-06

2e-05

2e-062e-062e-06

1.8e-051.2e-051.4e-05

3e-0603e-06

1.6e-05

4e-061e-06

3e-056e-063.2e-05

4e-06

2e-06

2.1e-054e-062e-05

5e-063e-065e-06

1.3e-052.1e-051e-05

6e-06

8e-062e-068e-06

3e-06

09e-06

7e-060

7e-061.1e-057e-06

3e-062e-06

0.0005410.0028140.0007520.0032120.00050.0004770.002392

01e-060

1.7e-051.1e-053e-06

1e-066e-061e-06

2e-063e-062e-06

2.6e-051.3e-052.2e-05

3.1e-054e-061.4e-05

6e-061.1e-059e-06

1.5e-05

3e-06

02e-061e-06

6e-061e-065e-06

3e-063e-062e-06

1.6e-05

01e-064e-06

0.1631730.221880.2090010.231380.1255630.1461950.255769

0.0041850.0023530.0208880.0040150.0116940.0039990.013582

0.0002860.0169350.0077980.0002660.011568

0.0008580.001084

0.0008580.001084

0.00011

0.0008580.000974

0.0002860.0160770.0067140.0002660.011568

0.0002860.0160770.0067140.0002660.011568

6.8e-050.0001240.000147.6e-05

0.0045610.00518

0.0002180.0001880.0002380.00019

0.0004890.000556

0.0005280.0006

0.0101870.011568

5.2e-050.000284.8e-05

5.2e-050.000284.8e-05

2.2e-057.4e-052.3e-05

2.2e-057.4e-052.3e-05

3e-050.0001092.5e-05

3e-050.0001092.5e-05

9.7e-05

9.7e-05

0.0038470.0023530.0039530.0040150.0036160.0036850.002014

0.0038470.0023530.0039530.0040150.0036160.0036850.002014

0.0038470.0023530.0039530.0040150.0036160.0036850.002014

0.0011490.001305

0.0023360.0014040.001760.0022080.0021680.002235

0.0015110.0009490.0010440.0018070.0014480.001450.000709

0.0024340.0012130.0060910.0048190.0031610.0023350.006257

0.0024340.0012130.0060910.0048190.0031610.0023350.006257

0.0024340.0012130.0060910.0048190.0031610.0023350.006257

0.000107

0.000107

0.0019650.0010.0013690.0020080.0018480.0018790.001283

0.0019650.0010.0013690.0020080.0018480.0018790.001283

0.000108

0.000108

0.0004690.0002130.0047220.0028110.0010980.0004560.004974

0.0004690.0002130.0004280.0028110.0010980.000456

0.0042940.004876

9.8e-05

0.1285970.1832660.1305230.1901230.07904299999999990.1164320.178934

0.0251560.0266750.0244810.0373410.0245720.0178870.041644

4.4e-051e-063.7e-055.4e-05

4.4e-051e-063.7e-055.4e-05

1e-061e-061e-061e-06

1.8e-051.8e-052.2e-05

1.6e-059e-061.9e-05

4e-063e-066e-06

5e-066e-066e-06

0.0006088.7e-050.0042220.0024090.0008030.0006370.002143

0.0005688.7e-050.0042220.0024090.0005980.0006060.002143

1.2e-05

1.1e-05

3e-069e-060.0021210.0024096e-062.3e-05

4.7e-052.4e-055e-05

1.1e-05

1e-06

4.5e-055e-064.7e-05

4.4e-05

6.1e-051.2e-056.3e-05

6e-06

9.1e-05

3.5e-053.8e-053.6e-05

0.0002317.8e-050.0002140.0002660.000235

2.7e-051.3e-052.9e-05

5.2e-052.1e-055.4e-05

1.6e-051e-051.8e-05

0.0018870.002143

7e-06

1.2e-05

5.1e-058e-065.1e-05

4e-059.6e-053.1e-05

4e-059.6e-053.1e-05

0.000109

0.000109

0.0057150.0031980.0047850.0049190.0076820.0055050.000905

0.0056740.0031980.0037770.0049190.0055450.0054668.2e-05

1e-05

7e-06

0.0052750.0030270.0035290.0033130.0043830.005058

1.3e-05

3.8e-05

2.5e-05

1.3e-05

2.6e-05

1.1e-05

1.7e-05

5.5e-05

0.000111

9e-06

3e-057.7e-052.8e-05

0.0002447.2e-050.0002480.0016060.0001620.0003043.4e-05

1.8e-058e-051.9e-05

1.4e-05

1.9e-05

6.8e-05

2.2e-05

0.0001019.9e-052.7e-055.2e-054.8e-05

7.2e-05

6e-06

6e-062.8e-055e-06

9.3e-05

5.6e-05

7.8e-05

2.5e-05

0.000113

0.000113

1.7e-055.9e-051.7e-05

1.7e-055.9e-051.7e-05

2.4e-050.0010080.0019652.2e-050.000823

0.0010080.0018780.000823

2.4e-058.7e-052.2e-05

4.5e-050.0014730.0014034.9e-050.000675

0.0002610.000296

0.0002610.000296

0.0009240.0003750.000675

0.000330.000375

0.0005940.000675

0.0001420.000161

0.0001420.000161

4.5e-057.5e-054.9e-05

4.5e-057.5e-054.9e-05

0.0001460.000165

0.0001460.000165

0.000331

4.8e-05

2.4e-05

2.6e-05

2.8e-05

4.7e-05

2.9e-05

2e-05

5.6e-05

5.3e-05

3e-055.5e-052.6e-05

3e-055.5e-052.6e-05

3e-055.5e-052.6e-05

0.0002230.0002120.0009790.0002311.6e-05

0.0002230.0002120.00090.0002311.6e-05

0.0001740.0002120.000850.0001811.6e-05

1.6e-051.6e-051.7e-05

1.5e-052e-051.6e-05

1.8e-058e-061.7e-05

6e-06

7.9e-05

7.9e-05

0.0166890.0216540.0118210.0277040.0096230.0079320.036161

0.0133720.0199560.0099440.0224850.0049430.0046470.036

0.0013680.0061310.0004920.002610.0001140.0018540.006758

4.1e-050.0001124.5e-05

1e-053e-061.8e-05

0.0001650.0001521.7e-050.000181

2.3e-050.0001143.4e-05

0.0002040.000231

2.3e-058.7e-052.5e-05

7.2e-055.2e-058.1e-05

8e-05

0

1e-06

3.5e-051.7e-055e-05

8e-06

5.5e-05

7.1e-05

1e-06

2.1e-054.6e-052.5e-05

5.1e-050.0010090.0012030.0021087e-065.3e-050.001368

0.0002150.000244

0.0001750.000198

0.0002840.0023260.0006050.0023092.2e-050.0004220.001908

0.0003040.000345

0.0006750.0001940.0004160.0029119.5e-050.000673

3.3e-055.3e-054.8e-05

0.0005040.000572

4.4e-050.0015910.0018071e-054.6e-05

0.0002440.000277

8e-055e-068.1e-05

0.0004320.0057590.0007440.0051191.8e-050.0004190.001171

2.4e-05

2.7e-05

4.7e-050.0002050.0002335.7e-05

0.0001525e-050.0001640.0002940.000163

4e-06

3.7e-052.8e-054.5e-05

1.6e-05

0.0001360.0001411e-060.000195

0.0002980.000339

0.0002690.000306

0.0001325.5e-050.0002240.0021081.5e-050.0001320.019592

0.0002740.000311

0.0095110.0044320.001520.0035130.0004790.005203

1.1e-05

9e-066e-068e-06

9e-066e-068e-06

0.000105

0.000105

6.8e-050.0002097.2e-05

6.8e-054.8e-057.2e-05

0.000103

5.8e-05

0

0.0013010.0004270.0003850.0019070.002040.0013290.000113

0.0013010.0004270.0003850.0019070.002040.0013290.000113

1.2e-050.0001131.6e-05

1.2e-050.0001131.6e-05

0.000163

9e-06

0.00012

3.4e-05

0.000790.000550.0006940.0016060.0007610.000757

0.000790.000550.0006940.0016060.0007610.000757

0.0011370.0007210.0007980.0017060.000760.0011034.8e-05

2e-06

2e-06

1e-05

3.8e-055e-054.5e-05

2.1e-05

0.0010490.0007210.0007980.0017060.0006250.001007

5e-064e-066e-06

3.3e-052.8e-053.2e-05

6e-06

2e-065e-064e-06

7e-061e-066e-06

1e-064e-061e-064.8e-05

0

2e-062e-062e-06

0.000125

0.000125

0.000115

0.000115

0.000113

0.000113

0.00017

7.4e-05

9.6e-05

0.0018020.0017350.0019680.0023090.003990.0034530.001744

6e-065.9e-055e-06

6e-065.9e-055e-06

0.0017960.0017350.0019680.0023090.0039310.0034480.001744

0.0017960.0017350.0019680.0023090.0039310.0034480.001744

0.1034410.1565910.1060420.1527820.0544710.0985450.13729

0.0547880.0582150.0410520.0609330.0259320.0471520.044935

1.4e-050.0019450.0022080.0001023.1e-05

8e-062.2e-058e-06

5.6e-05

6e-060.0019450.0022082.4e-052.3e-05

0.0547740.0582150.0391070.0587250.025830.0471210.044935

5.2e-05

6.7e-052.9e-056.5e-05

0.0011770.0003750.000390.0028110.000320.0011170.000177

7.9e-057.7e-057.7e-05

1.9e-05

8.9e-050.0003633.8e-058.9e-050.000412

0.0002820.0003560.0066255e-050.0002688.7e-05

0.0002470.00028

5.6e-05

0.0010.001136

0.0010320.0008470.0007480.0020080.0003750.0010030.001331

0.0007420.0012270.0006370.0020080.000350.000716

0.0020680.0020210.002578

0.0001390.000158

0.000290.0002460.0001850.0002820.000522

0.001330.0029280.0013730.0028110.0008130.0012740.002608

8e-066e-060.0015030.0017061e-068e-063.2e-05

3.2e-057e-063.3e-05

0.0001450.0001285.1e-050.0001454.6e-05

0.0003240.000368

1.8e-05

9.3e-058e-059.9e-05

3.5e-05

8.4e-057.1e-058.6e-05

0.024440.0287360.0128850.0134510.0063410.0158160.025604

0.0123140.0063750.005580.0041160.0077440.0119090.003957

7.2e-05

5.3e-051.9e-055.1e-05

0.00020.000227

3.8e-051.3e-053.7e-05

4e-057e-064.2e-05

8.3e-05

0.0002390.000271

0.0001230.00014

1.2e-050.0017680.0020083.4e-053.2e-05

0.000107

0.00140.0028240.0019950.002610.0019720.00330.002585

2.2e-05

7e-062.3e-053e-066e-063.6e-05

0.0007460.0028440.0005340.0028110.0001750.0005650.002802

0.0002580.0011020.0003110.0024090.0001480.0002190.001977

8.9e-058.6e-050.0003560.0019075.4e-050.0002580.000331

0.0090660.0085460.0051560.005320.0030860.008719

0.0008180.0002070.000280.0036140.0004180.0007830.000113

4.3e-052.1e-050.0002050.002516.2e-050.000122

0.0380060.0928740.0504920.0754880.0205060.0412080.086923

0.0379110.0928740.0504920.0754880.0204060.0411210.086923

0.0001960.000223

3.5e-050.001680.0019073.1e-053.6e-05

2.1e-053.7e-050.0037130.0042165e-061.8e-05

0.000310.0080090.0004660.0036140.0002440.0002980.003757

5.6e-05

6.3e-05

2.3e-05

6.1e-056.6e-051.6e-05

6.4e-050.0001167.1e-05

1e-05

0.00017.3e-059.8e-05

2.5e-05

0.0001150.000131

3.2e-050.0001240.0001412.6e-05

3.2e-053.3e-053.1e-05

0.0020850.004360.0026540.0033130.0017450.006710.00388

0.0001240.000141

0.0038890.0091470.0022580.0058220.0007150.0037350.007358

0.00070.0009380.0006610.0018070.0003560.0006760.001527

8.3e-056.1e-050.0032710.0037144.8e-057.8e-05

4.9e-054.5e-050.0017680.0020082e-055e-056e-06

0.0055880.0107410.0033630.0067260.0011970.0053540.007813

7.1e-05

3e-053.3e-053.3e-05

4e-064.8e-054e-06

8.4e-05

0.0030740.003491

9.3e-05

0.0011420.001296

0.0015660.0006120.0005470.0055210.0001580.0015660.004122

6e-05

2.7e-050.0023870.002715.7e-052.6e-05

2.2e-05

8.7e-05

0.0231780.0589240.0229490.034130.0128960.0221860.054943

5.7e-058.3e-055.9e-05

3.2e-05

9.5e-050.00018.7e-05

7e-053.8e-056.2e-05

2.5e-053.8e-052.5e-05

2.4e-05

0.0019930.0015240.0016250.00196.4e-05

0.0019930.0015240.0016250.00194.8e-05

5.9e-05

2.9e-055.2e-052.9e-05

3.2e-05

0.0019360.0015240.0014770.001853

2.8e-053.7e-051.8e-051.6e-05

1.6e-05

1.6e-05

6e-050.00034.6e-05

6e-050.00034.6e-05

9.7e-05

6.6e-05

9e-06

6.2e-05

2.4e-05

6e-054.2e-054.6e-05

0.0034320.0020340.0055640.0071270.0036520.003290.002055

2.8e-055.6e-052.1e-05

2.8e-055.6e-052.1e-05

0.0013340.0007160.0041320.0051190.0016560.0012880.000962

0.0003630.000580.000319

9e-060.0014140.0016068e-068e-06

2.7e-052.1e-052e-05

2e-052.4e-051.9e-05

3.7e-052.6e-053.9e-05

2.3e-050.0015030.0017062.1e-052.6e-05

0.0012180.0007160.0008520.0018070.0009760.0011760.000643

0.002070.0013180.0014320.0020080.001940.0019810.001093

4.4e-05

1.4e-05

0.002070.0013180.0014320.0020080.0018820.0019810.001093

0.0051620.0034680.007410.0092340.0024560.0049490.003313

0.0009470.0011140.0009020.0019070.0007920.0009070.000984

0.0009470.0011140.0009020.0019070.0007920.0009070.000984

1.6e-053.3e-051.6e-05

1.6e-053.3e-051.6e-05

0.0041990.0023540.0065080.0073270.0016310.0040260.002329

3.6e-054.3e-053.5e-05

2.6e-051.4e-052.6e-05

1.5e-051.9e-050.0015030.0017061.4e-051.1e-05

0.0041020.002310.0020380.002610.0011770.0039350.002329

2e-052.5e-050.0026520.0030111.6e-051.9e-05

0.0003150.000357

1e-05

6.6e-05

6.6e-05

6.6e-05

6.6e-05

6.6e-05

0.0277230.0350480.0437860.0324230.0295920.02320.050304

0.0066010.0049850.0039190.0050190.0042850.0063340.003223

0.0024590.0016830.0015410.0023090.0022120.0023440.001007

1e-054.6e-055e-06

1e-054.6e-055e-06

8.4e-050.0001329.2e-05

4.4e-054.8e-054.6e-05

5.2e-05

1.6e-051.6e-052.1e-05

0

2.4e-051.6e-052.5e-05

000

1.1e-055.4e-057e-06

1.1e-055.4e-057e-06

0.0023350.0016830.0015410.0023090.001890.0022240.001007

0.0023350.0016830.0015410.0023090.001890.0022240.001007

7e-064.9e-057e-06

7e-064.9e-057e-06

3e-062.7e-052e-06

3e-062.7e-052e-06

9e-061.4e-057e-06

9e-061.4e-057e-06

1.8e-059.1e-059e-06

1.2e-055.4e-054e-06

1.2e-055.4e-054e-06

6e-063.7e-055e-06

6e-063.7e-055e-06

0.0041170.0033020.0023780.002710.001930.0039750.002216

8e-065.6e-056e-06

8e-065.6e-056e-06

1.3e-054.1e-051.8e-05

6e-061.5e-055e-06

7e-062.6e-051.3e-05

0.0040960.0033020.0023780.002710.0018330.0039510.002216

6e-062.4e-055e-06

5e-062.9e-055e-06

3e-061.3e-053e-06

1e-054.5e-057e-06

0.0040540.0033020.0023780.002710.0016770.0039130.002216

2e-061.4e-052e-06

9e-061.5e-059e-06

7e-061.6e-057e-06

7e-065.2e-056e-06

7e-065.2e-056e-06

7e-065.2e-056e-06

6e-063.5e-056e-06

6e-063.5e-056e-06

6e-063.5e-056e-06

6e-063.5e-056e-06

2.3e-050.0002243.6e-05

1.3e-050.0001352.3e-05

5e-065.6e-054e-06

5e-065.6e-054e-06

8e-067.9e-051.9e-05

5e-063.6e-051.2e-05

3e-064.3e-057e-06

1e-058.9e-051.3e-05

2e-064.1e-051e-06

2e-064.1e-051e-06

8e-064.8e-051.2e-05

8e-064.8e-051.2e-05

0.0210930.0300630.0398670.0274040.0250480.0168240.047081

0.010510.0136940.0103070.0089340.0058780.0100670.018096

7.6e-050.0001670.0005370.0004427.5e-051.6e-05

1.6e-05

0.000270.000306

5.5e-055.4e-05

0.0001670.000147

2.1e-050.000120.0001362.1e-05

9.6e-050.0001350.0001539.3e-05

9.6e-050.0001350.0001539.3e-05

0.0011770.0018010.007801

0.0011770.0018010.007801

0.0102890.012350.0077220.0089340.0051560.0098520.010263

4e-065.7e-055e-06

0.0001990.000226

0.000121

4e-062.7e-055e-06

0.0102640.012350.0075230.0089340.00470.0098240.010263

1.7e-052.5e-051.8e-05

4.9e-050.0001120.0001274.7e-05

4.9e-050.0001120.0001274.7e-05

1.6e-05

1.6e-05

2.4e-050.0003120.0019530.0019812.2e-054.8e-05

4.8e-05

4.8e-05

0.0013220.001501

0.0013220.001501

0.0003570.000405

0.0003570.000405

0.0003120.000274

0.0003120.000274

2.4e-057.5e-052.2e-05

2.4e-057.5e-052.2e-05

4.6e-050.0001620.0001844.5e-05

4.6e-050.0001620.0001844.5e-05

4.6e-050.0001620.0001844.5e-05

0.0060880.0125070.009180.0064240.003950.002630.016667

7.8e-050.0001450.0001647.7e-05

7.8e-050.0001450.0001647.7e-05

2.2e-050.0001061.9e-05

2.2e-050.0001061.9e-05

0.0059880.0125070.0045460.0064240.003590.0025340.01157

0.0059880.0125070.0045460.0064240.003590.0025340.01157

9e-05

9e-05

0.0044890.005097

0.0044890.005097

0.0027910.0023290.0079680.0058220.0078210.0027170.003747

0.0027610.0023290.0079680.0058220.0076280.0026830.003747

4.2e-053.8e-050.001680.0019077e-064.3e-05

2.1e-051e-062.2e-05

1.9e-058e-062.6e-05

0.0003670.000416

0.0025820.002932

2.4e-05

7e-06

1.9e-052.7e-052.1e-05

2.8e-05

1.7e-053e-051.9e-05

5e-05

0.0007050.0012520.0007870.0020080.0006570.0006760.001006

2.1e-051.7e-051.4e-05

6.6e-05

3.1e-052.7e-053.5e-05

0.0017510.0010360.0012420.0019070.00190.0016820.000932

2e-05

1e-053e-068e-061.4e-05

0.001310.0015960.001393

3.6e-053.6e-053.7e-05

5.9e-05

1e-053.5e-059e-06

2.8e-052.5e-053.1e-05

1.7e-054.8e-051.8e-05

3.4e-052e-053.6e-05

4.8e-05

4.8e-05

1.3e-050.0001141.5e-05

6e-065.4e-057e-06

7e-066e-058e-06

1.7e-053.1e-051.9e-05

1e-052.7e-051.1e-05

1e-06

7e-063e-068e-06

0.0001430.000163

0.0001430.000163

0.0001430.000163

1.5e-050.0001021e-05

1.1e-054.8e-059e-06

1.1e-054.8e-059e-06

4e-065.4e-051e-06

4e-065.4e-051e-06

0.000130.0005820.000108

9e-06

9e-06

9e-065.1e-059e-06

9e-065.1e-059e-06

1.8e-059.3e-051.7e-05

7e-063.2e-056e-06

8e-063.2e-058e-06

3e-062.9e-053e-06

01.9e-050

02e-060

1.7e-05

000

1.3e-050.0001271.6e-05

4e-063.5e-056e-06

3e-061.7e-053e-06

5.1e-05

6e-062.4e-057e-06

1.5e-056.9e-051.2e-05

1.5e-056.9e-051.2e-05

1.3e-054.4e-051.1e-05

1.3e-054.4e-051.1e-05

5.3e-050.0001794.3e-05

1.4e-052.7e-051.2e-05

4e-062.7e-054e-06

8e-06

1.4e-054.5e-051.9e-05

8e-064.8e-055e-06

5e-063.2e-053e-06

0.0001890.0001510.000630.0001950.000112

1.1e-056e-051.4e-05

1.1e-056e-051.4e-05

0.0001780.0001510.000570.0001810.000112

0.0001280.0001510.0004870.000132

0.000112

5e-058.3e-054.9e-05

3.7e-059.4e-052.5e-05

3.7e-059.4e-052.5e-05

3.7e-059.4e-052.5e-05

4.4e-055e-053e-05

4.4e-055e-053e-05

4.4e-055e-053e-05

0.0011830.0012210.0100030.0062240.0035580.000950.008411

0.0007260.000360.0006440.0062240.0010160.000709

0.0001370.000121

0.0007260.0002230.0005230.0062240.0010160.000709

0.0001530.000174

0.0001530.000174

0.0002220.0003250.0011250.0010650.0001360.000575

0.0003110.000354

0.000420.000477

0.0001960.0003250.000180.0004680.0001089.8e-05

2.6e-050.0002140.0002432.8e-05

0.0001420.000161

0.0001420.000161

2e-058.9e-051.9e-05

2e-058.9e-051.9e-05

7.5e-050.0003050.000347

7.5e-05

0.0003050.000347

0.0001982.1e-050.0059130.0004826.4e-050.006545

0.0038650.004389

0.0001982.1e-050.0002470.0004826.4e-05

0.0011340.001288

0.0006670.000758

0.00011

0.000440.0012180.0005250.000419

0.000180.000158

0.000260.000229

0.0003690.000419

0.0004620.000525

1.7e-050.0005030.000222.2e-050.000351

1.7e-050.0001940.000222.2e-05

0.0003090.000351

3.6e-055.5e-052.5e-05

3.6e-055.5e-052.5e-05

3.6e-055.5e-052.5e-05

0.0002340.0077130.0020070.0002290.006692

0.0002340.0077130.0020070.0002290.006692

0.0002340.0077130.0020070.0002290.006692

0.0001340.000153

0.0001340.000153

8.7e-05

8.7e-05

0.0002240.0001960.000221

0.0002240.0001960.000221

0.0073830.0016920.006692

0.001490.001692

0.0058930.006692

1e-057.5e-058e-06

6e-05

1e-051.5e-058e-06

7.3e-050.0002696.4e-05

6e-066.2e-054e-06

6e-066.2e-054e-06

6e-066.2e-054e-06

6e-066.2e-054e-06

6e-066.2e-054e-06

6e-050.0001715.4e-05

6e-050.0001715.4e-05

6e-050.0001715.4e-05

2e-055.3e-051.9e-05

1e-052.8e-058e-06

1e-052.5e-051.1e-05

2e-055e-051.9e-05

1.3e-052.5e-051.2e-05

7e-062.5e-057e-06

1.3e-053.4e-051.1e-05

1.3e-053.4e-051.1e-05

7e-063.4e-055e-06

7e-063.4e-055e-06

7e-063.6e-056e-06

7e-063.6e-056e-06

7e-063.6e-056e-06

7e-063.6e-056e-06

7e-063.6e-056e-06

8.3e-050.0003140.0007026.9e-05

3.2e-05

3.2e-05

3.2e-05

3.2e-05

3.2e-05

9.2e-05

9.2e-05

9.2e-05

9.2e-05

9.2e-05

3e-050.0003140.0003572.9e-05

3e-050.0003140.0003572.9e-05

3e-050.0003140.0003572.9e-05

3e-050.0003140.0003572.9e-05

3e-050.0001520.0001732.9e-05

0.0001620.000184

2.2e-050.0001831.8e-05

1.4e-050.0001341.2e-05

1.4e-050.0001341.2e-05

1.4e-055.2e-051.2e-05

1.4e-055.2e-051.2e-05

4.7e-05

4.7e-05

3.5e-05

3.5e-05

8e-064.9e-056e-06

8e-064.9e-056e-06

8e-064.9e-056e-06

8e-064.9e-056e-06

3.1e-053.8e-052.2e-05

3.1e-053.8e-052.2e-05

3.1e-053.8e-052.2e-05

3.1e-053.8e-052.2e-05

3.1e-053.8e-052.2e-05

0.0057370.0086310.0049660.0078290.0038210.0103450.007058

0.0057370.0086310.0049660.0078290.0038210.0103450.007058

0.0037460.0047080.0035370.0049180.0022490.0084970.003926

0.0037460.0047080.0035370.0049180.0022490.0084970.003926

0.0037350.0047080.0035370.0049180.0022050.0084880.003926

2e-05

0.001060.0018010.0007690.0022080.0002950.0011710.001484

0.0007140.000811

0.0026730.0029070.0020540.002710.0010640.0073160.002442

2e-061.5e-051e-06

1.1e-054.4e-059e-06

1.1e-054.4e-059e-06

0.0017160.0039230.0014290.0029110.0014470.0015990.003068

0.0015780.0039230.0014290.0029110.0008270.0014690.003068

0.0015030.0039230.0014290.0029110.0008120.0014340.003068

3e-051e-052.5e-05

1e-064e-060

0.0014150.0039230.0014290.0029110.0007530.0013550.003068

1.4e-051e-061.3e-05

3.4e-053.2e-053.3e-05

9e-061.2e-058e-06

7.5e-051.5e-053.5e-05

4.1e-051e-05

1.8e-0502.2e-05

2e-0600

2e-06

5e-062e-064e-06

9e-069e-06

1e-06

0.0001380.000620.00013

4e-063.1e-054e-06

4e-063.1e-054e-06

3.1e-050.000132.8e-05

7e-066.2e-051e-05

1.8e-053.7e-051.3e-05

6e-063.1e-055e-06

7.8e-050.0003617.8e-05

2.3e-051.9e-052.1e-05

2e-06

1e-06

2.2e-05

4e-063.3e-055e-06

6e-064e-055e-06

3e-063.2e-053e-06

4e-065.9e-054e-06

3.4e-057.2e-053.6e-05

5e-05

4e-063.1e-054e-06

1.3e-056.1e-051.1e-05

7e-063.2e-055e-06

6e-062.9e-056e-06

1.2e-053.7e-059e-06

1.2e-053.7e-059e-06

0.0002750.0001250.0002496.4e-05

0.0002750.0001250.0002496.4e-05

0.0002750.0001250.0002496.4e-05

7.7e-054.7e-057.6e-053.2e-05

7.9e-056.5e-057.8e-053.2e-05

0.00011308.9e-05

6e-061.3e-056e-06

3e-055.5e-052.2e-05

3e-055.5e-052.2e-05

3e-055.5e-052.2e-05

3e-055.5e-052.2e-05

3e-055.5e-052.2e-05

3e-055.5e-052.2e-05

0.4068990.4130570.4423380.3542460.4342150.4240240.369069999999999

0.0117730.0068050.0073260.0073280.0125630.01120.005652

3e-053.4e-052.6e-05

3e-053.4e-052.6e-05

3e-053.4e-052.6e-05

3e-053.4e-052.6e-05

0.0001450.0004620.0001272.9e-05

4.3e-05

4.3e-05

4.3e-05

1.5e-057.9e-051.2e-05

4.3e-05

4.3e-05

1.5e-053.6e-051.2e-05

1.5e-053.6e-051.2e-05

2.1e-054.1e-051.7e-05

2.1e-054.1e-051.7e-05

2.1e-054.1e-051.7e-05

1.3e-057.5e-051e-05

1.3e-054.6e-051e-05

1.3e-054.6e-051e-05

2.9e-05

2.9e-05

4.5e-059e-054.3e-052.9e-05

4.5e-059e-054.3e-052.9e-05

1.2e-052.5e-051.2e-052.9e-05

2.7e-055e-052.5e-05

6e-061.5e-056e-06

5.1e-059.5e-054.5e-05

1.7e-052.6e-051.5e-05

1.7e-052.6e-051.5e-05

3.4e-056.9e-053e-05

1.4e-051.9e-051.1e-05

9e-061.9e-058e-06

1.1e-051.8e-051.1e-05

1.3e-05

3.9e-05

3.9e-05

3.9e-05

2.1e-059.6e-052e-05

2.1e-059.6e-052e-05

3.9e-05

3.9e-05

2.1e-055.7e-052e-05

2.1e-055.7e-052e-05

3.3e-050.000240.0003343.2e-05

1.1e-056.2e-059e-06

1.1e-056.2e-059e-06

7e-062.2e-055e-06

4e-064e-054e-06

2.2e-050.000240.0002722.3e-05

2.2e-050.000240.0002722.3e-05

2.2e-050.000240.0002722.3e-05

0.0001540.0003680.000138

0.0001230.000230.000112

0.0001230.0001910.000112

2.1e-052e-051.7e-05

1.4e-052.5e-051.3e-05

8e-067e-064e-06

2.6e-057e-062.3e-05

6e-06

7e-062.3e-059e-06

2.1e-05

2e-051e-051.7e-05

1.4e-053.2e-051.7e-05

1.3e-054e-051.2e-05

3.9e-05

3.9e-05

3.1e-050.0001382.6e-05

7.5e-05

3.5e-05

4e-05

3.1e-056.3e-052.6e-05

1.2e-053.5e-058e-06

1.9e-052.8e-051.8e-05

3.1e-052.9e-051.8e-05

3.1e-052.9e-051.8e-05

3.1e-052.9e-051.8e-05

3.1e-052.9e-051.8e-05

0.0088050.0053370.0055940.005320.0085860.0084180.004642

0.0041810.0024850.002650.002610.0040470.0040010.002153

2e-063.2e-051e-06

2e-063.2e-051e-06

0.0041660.0024850.002650.002610.0039590.0039870.002153

0.0041660.0024850.002650.002610.0039590.0039870.002153

7e-062e-058e-06

7e-062e-058e-06

6e-063.6e-055e-06

6e-063.6e-055e-06

0.0046240.0028520.0029440.002710.0045390.0044170.002489

5e-063.5e-055e-06

5e-063.5e-055e-06

2e-054.4e-051.6e-05

2e-054.4e-051.6e-05

2.3e-054.6e-051.7e-05

2.3e-054.6e-051.7e-05

0.0045760.0028520.0029440.002710.0044140.0043790.002489

0.0045760.0028520.0029440.002710.0044140.0043790.002489

0.0025270.0014680.0014920.0020080.0024850.00240.000981

2.4e-058.5e-051.9e-05

2.4e-058.5e-051.9e-05

2.4e-053.5e-051.9e-05

5e-05

0.0024950.0014680.0014920.0020080.0023770.0023720.000981

6e-062.2e-056e-06

6e-062.2e-056e-06

0.0024890.0014680.0014920.0020080.0023550.0023660.000981

1.4e-051.7e-051.1e-05

1.7e-051.3e-05

1.2e-052.9e-058e-06

2.8e-053e-052.4e-05

2.4e-05

4.4e-05

9e-065e-06

2e-062e-06

2.5e-053.1e-052.2e-05

2.3e-053.1e-052.3e-05

6e-063.3e-056e-06

0.0023530.0014680.0014920.0020080.0021160.0022520.000981

8e-062.3e-059e-06

8e-062.3e-059e-06

8e-062.3e-059e-06

2.7e-050.0001692.1e-05

2e-050.0001321.5e-05

3e-062.9e-052e-06

3e-062.9e-052e-06

4e-063.2e-053e-06

4e-063.2e-053e-06

1.3e-057.1e-051e-05

1.3e-057.1e-051e-05

7e-063.7e-056e-06

7e-063.7e-056e-06

7e-063.7e-056e-06

6.6e-059.1e-056.4e-05

6.6e-059.1e-056.4e-05

6.6e-059.1e-056.4e-05

6.6e-059.1e-056.4e-05

1.1e-052.2e-051e-05

1.4e-054.1e-051.9e-05

4.1e-052.8e-053.5e-05

0.0732830.0506780.1174490.0602280.09173300000000010.0702210.094568

0.0018420.0002750.0020260.0019770.0016420.000263

0.000120.0001069.4e-05

0.000120.0001069.4e-05

0.000120.0001069.4e-05

0.0013070.0002750.0017640.0016580.0012030.000263

0.0006317.2e-050.000530.0002850.0005781.7e-05

1.3e-05

0.0004447.2e-050.0003725e-060.0004031.7e-05

0.0001870.0001580.0001750.000175

9.2e-05

0.0006760.0002030.0012340.0013730.0006250.000246

0.0009490.001078

0.0006760.0002030.0002850.0002950.0006250.000246

0.0004150.0002620.0002130.000345

0.0001640.000120.0001090.000146

0.0001640.000120.0001090.000146

0.0002510.0001420.0001040.000199

0.0002510.0001420.0001040.000199

0.0250750.014190.0149650.0103390.0264280.0238920.01167

0.0010080.0008340.000405

9.4e-05

9.4e-05

0.0010080.000740.000405

0.0003570.000405

0.0006510.00074

0.023660.013960.0133910.0103390.0215320.0226230.010877

0.000129

6.8e-05

6.1e-05

6.4e-056.1e-055e-05

6.4e-056.1e-055e-05

5.9e-05

5.9e-05

0.0235230.013960.0133910.0103390.0212680.0225120.010877

3.7e-058.8e-053.4e-05

0.0234860.013960.0133910.0103390.021180.0224780.010877

7.3e-051.5e-056.1e-05

4.6e-057e-063.9e-05

2.7e-058e-062.2e-05

0.0009860.000230.0004490.0034470.0009490.000323

0.0009860.000230.0004490.0034470.0009490.000323

0.0009860.000230.0004490.0034470.0009490.000323

0.0002970.0001170.0002880.0002114.9e-05

0.0001330.0001179.1e-059.7e-051.6e-05

0.0001330.0001179.1e-059.7e-051.6e-05

0.0001080.0001247.5e-05

0.0001080.0001247.5e-05

5.6e-057.3e-053.9e-053.3e-05

5.6e-057.3e-053.9e-053.3e-05

0.0001320.0003270.0001091.6e-05

0.0001080.0002168.7e-05

1.9e-05

3.4e-054.4e-052.8e-05

2.6e-054.5e-053.3e-05

1.1e-051.3e-051.5e-05

3.7e-054.8e-051.1e-05

4.7e-05

2.4e-050.0001112.2e-051.6e-05

2.4e-055.7e-052.2e-051.6e-05

5.4e-05

0.0023320.001480.0014280.0020080.0021140.0022180.000954

0.0023320.001480.0014280.0020080.0021140.0022180.000954

5e-051.9e-054.4e-05

5e-051.9e-054.4e-05

9.4e-050.0001338e-05

1.6e-052.7e-051.6e-05

9e-061.9e-056e-06

2.4e-055.3e-052.2e-05

1.1e-052e-057e-06

3.4e-051.4e-052.9e-05

6e-052.9e-056.1e-05

6e-052.9e-056.1e-05

0.0021280.001480.0014280.0020080.0019330.0020330.000954

0.0021280.001480.0014280.0020080.0019330.0020330.000954

0.0358290.0247720.073890.0311170.0506710.0345530.049223

0.0123230.013994

0.0123230.013994

0.0123230.013994

0.0015120.0023430.0028740.0043160.0013030.0012670.002306

0.000364.9e-050.000240.0001150.0002190.0001

0.000364.9e-050.000247.7e-050.0002190.0001

3.8e-05

4.5e-050.0001162.6e-05

7.3e-05

4.5e-054.3e-052.6e-05

0.000103

0.000103

0.000112

0.000112

0.000119

0.000119

0.0010350.0022940.0026340.0043160.0005840.000970.002206

5.7e-05

4.2e-052.6e-050.0015711e-064e-050.001785

3.2e-05

0.0001194.1e-050.000115.2e-05

8.3e-05

8.8e-05

3e-05

3.4e-05

0.0006340.0022430.0003490.0024090.0001170.000616

7e-06

2.9e-052.5e-050.0005450.0019078e-062.9e-050.000369

0.0002110.0001698.6e-050.000175

2.7e-057e-051.3e-05

2.3e-055.1e-051e-05

4e-061.9e-053e-06

4.5e-058.4e-053.9e-05

4.5e-058.4e-053.9e-05

0.0007830.01650.0086170.0006440.010362

0.0159960.0080680.010271

0.000116

0.0090440.010271

5.7e-05

0.0069520.007895

5.1e-05

5.1e-05

0.0002820.0001810.0001490.0002291.6e-05

0.0002820.0001810.0001490.0002291.6e-05

0.0001720.0001368.1e-050.000145.8e-05

5.8e-05

0.0001720.0001368.1e-050.00014

5.9e-05

5.9e-05

9.7e-057.1e-058e-05

4e-057.1e-053.4e-05

5.7e-054.6e-05

4.3e-05

4.3e-05

0.0002320.0001879.5e-050.0001951.7e-05

0.0002320.0001879.5e-050.0001951.7e-05

0.0180570.011440.0305460.0120460.0298780.0179940.011579

0.0006950.0001020.0003550.0004170.0006490.000166

4.2e-05

8.5e-055e-057.1e-052.9e-05

0.0001266.5e-050.0001151.5e-05

7.3e-053.9e-056.8e-05

0.0002125.3e-050.0001827e-050.0002016.5e-05

0.000107

0.0001994.9e-050.0001734.4e-050.0001945.7e-05

0.0082970.0042360.0224310.0239480.0085480.004216

0.0173520.019705

0.0017190.0002520.000570.0011970.0017560.000562

0.0020490.0025380.0014140.0008510.002110.001714

0.0019980.0004540.0007770.0011340.0020710.000626

0.0025310.0009920.0013830.0026110.001314

0.0009350.001061

0.0001290.000150.0004650.000111

2.6e-05

8.1e-05

2.6e-05

0.000150.000171

1.4e-05

2.5e-05

0.0001291.5e-050.000111

8.4e-05

2.3e-05

0.0043420.0053340.0032220.0082320.0018470.0042690.005109

5.4e-051.3e-051e-055.5e-05

9e-053e-059.1e-05

0.0002672.9e-050.0002354.2e-050.0002689.1e-05

2e-05

0.0021180.0018020.0011920.0021080.0005510.0020530.001594

0.000337.5e-050.0001890.0001280.0003222.7e-05

0.0001670.00019

0.000180.0020080.0001010.0001740.000114

0.000119

1e-06

0.0001350.0001226.4e-050.000141

1.5e-05

0.0001131.3e-053e-060.000126e-06

0.0003510.0032160.0006350.0023092.6e-050.0003410.002426

0.0001350.0001222.6e-050.000142

2e-05

8.8e-050.0001330.0002820.0018072e-058.7e-050.000763

2e-05

4.5e-053.2e-056e-064.4e-054.4e-05

1e-06

2.4e-05

5.9e-05

6.5e-052.1e-059e-066.6e-054e-06

0.0001980.0001712e-050.000191.7e-05

3.3e-058e-063.2e-05

1.9e-055e-062e-052.3e-05

1e-05

5.8e-05

3.3e-05

1.1e-05

7.3e-05

1.7e-05

0

5e-06

5.4e-05

2.5e-05

6e-06

0.0001210.0001071.3e-050.0001230

2.4e-05

0.0016370.0007570.0007740.0016060.000710.0016020.000675

0.000107

0.000118

3.1e-05

0.0011580.0003120.0004550.0003590.001124

0.0004790.0004450.0003190.0016060.0001260.0004780.000644

0.0029570.0010110.0036140.0022080.0024910.0028150.001413

3.2e-050.0010110.0012290.0022083e-051.3e-050.001413

0.0029250.0023850.0024450.002802

1.6e-05

0.0106580.0087580.0091340.010740.0083730.0101320.009348

0.0002640.0002137.2e-050.0002231.7e-05

0.0002640.0002137.2e-050.0002231.7e-05

2.5e-055.1e-052e-05

2.5e-055.1e-052e-05

8.4e-057.4e-057e-05

8.4e-057.4e-057e-05

0.0029940.0025540.0025930.0036130.0014150.0028810.000995

8.4e-050.0008770.0007722.2e-057.7e-05

0.0009870.0005280.0007370.0017060.0007050.000942

0.0001164.1e-050.0001042.4e-05

0.0016450.0011490.0009440.0019070.0005220.0016010.000949

4.2e-05

0.0001620.000148.3e-050.0001572.2e-05

0.0001535.2e-050.0001264.8e-050.0001351.5e-05

0.0001535.2e-050.0001264.8e-050.0001351.5e-05

0.0001810.000150.0001620.000168

0.0001810.000150.0001620.000168

9.4e-05

9.4e-05

0.0004686.9e-050.0004010.0004270.0004336.2e-05

0.0003266.9e-050.0002870.0003370.0003150

0.0001420.0001149e-050.0001186.2e-05

0.0001214.4e-050.00011

0.0001214.4e-050.00011

0.0001252.4e-055e-050.000129

2.2e-05

6.8e-051.6e-056.7e-05

5.7e-052.4e-051.2e-056.2e-05

0.000118

7.5e-05

4.3e-05

7.9e-05

3.8e-05

4.1e-05

0.0062430.0060590.0056510.0071270.005610.0059630.008259

7.3e-05

0.0062430.0060590.0056510.0071270.0055370.0059630.008259

0.000129

7.3e-05

5.6e-05

0.0048190.0022310.0025130.0040150.00250.0045160.001634

0.0001056.1e-058.7e-05

0.0001056.1e-058.7e-05

0.0011870.0003320.0008570.0018070.000910.001110.000172

0.0005350.000608

0.0011870.0003320.0003220.0018070.0001880.001110.000172

0.000114

0.000166

7.8e-05

8.8e-05

0.0034570.0018990.0016560.0022080.0011480.0032710.001462

0.0034570.0018990.0016560.0022080.0010720.0032710.001462

3.4e-05

4.2e-05

7e-050.0002154.8e-05

7e-057.8e-054.8e-05

7.9e-05

5.8e-05

0.0082050.0099610.025140.0167640.0105430.0079160.032458

0.0006280.0003050.0002990.0004620.000134

0.0002670.0001540.0001160.0002061.6e-05

8e-06

0.0002670.0001540.0001160.0002068e-06

0.0001649.4e-050.0001050.000118

0.0001649.4e-050.0001050.000118

0.0001970.0001518.9e-050.000151

0.0001970.0001518.9e-050.000151

0.0075770.0099610.0248350.0167640.0102440.0074540.032324

0.0002490.000283

0.0002490.000283

0.0018890.001160.000986

0.0008680.000986

0.0010210.00116

0.0003160.000359

0.0003160.000359

0.0075770.0099610.020510.0167640.0088010.0074540.028854

0.0001560.000177

0.0013620.0044560.000936

0.0058020.006589

0.0026190.002974

0.0017650.0010810.0005370.0024090.0001680.0011730.00088

0.000660.000749

4.2e-05

0.0020170.00229

0.004160.0016990.0011250.0101390.0017260.0041340.000339

0.0016520.0071810.0006810.0042160.0001610.0021470.009863

0.0001240.000141

0.0016720.001899

0.0016040.001822

0.0021510.002443

0.0018710.002125

0.0018710.002125

0.003260.0017740.0047970.0055210.0031870.0030580.001383

1.2e-054.8e-059e-06

1.2e-054.8e-059e-06

3e-062.6e-055e-06

3e-062.6e-055e-06

9e-062.2e-054e-06

9e-061.4e-054e-06

8e-06

0.003240.0017740.0047970.0055210.0030980.0030420.001383

0.0001250.0029170.0033130.0003287.5e-05

5.3e-050.0029170.0033130.0002114e-05

2.5e-053e-062.5e-05

8e-06

1.2e-05

1e-05

2e-05

8e-063.2e-057e-06

1e-064e-061e-06

1e-060.0029170.0033131e-062e-06

1e-05

5.2e-05

6e-062e-065e-06

4e-06

1.6e-05

4e-06

1e-051.6e-05

2e-061.7e-05

8e-065.4e-056e-06

6e-06

1e-069e-061e-06

1e-05

7e-061.4e-055e-06

1e-05

5e-06

6.4e-056.3e-052.9e-05

8e-061.1e-058e-06

1.3e-052.2e-051.2e-05

4.3e-053e-059e-06

0.00310.0017740.001880.0022080.0027390.0029550.001383

2e-054.2e-051.6e-05

5e-062.2e-055e-06

1.5e-052e-051.1e-05

1.4e-052.1e-057e-06

1.4e-052.1e-057e-06

0.0030630.0017740.001880.0022080.0026450.0029290.001383

2e-061e-051e-06

9e-061e-068e-06

1e-062.7e-050

000

2.9e-05

0.0030370.0017740.001880.0022080.002510.0029070.001383

9e-061e-05

2e-068e-062e-06

2.2e-05

3e-061.8e-051e-06

2e-05

3e-063.1e-053e-06

3e-063.1e-053e-06

1.5e-053.1e-051.2e-05

1.5e-053.1e-051.2e-05

1.5e-053.1e-051.2e-05

8e-064.1e-057e-06

8e-064.1e-057e-06

8e-064.1e-057e-06

8e-064.1e-057e-06

0.1893310.1969080.1257820.1009820.141650.1815450.086521

0.0848230.0585520.0491260.047580.0506430.081440.030436

0.0016350.0025960.001350.0029110.0010640.0015660.002843

0.0016350.0025960.001350.0029110.0010640.0015660.002843

0.0012960.0025680.0012390.0029110.0006250.001240.002828

0.000111

7.5e-056.6e-057.6e-058e-06

7.9e-057.2e-057e-057e-06

0.0001272.8e-050.0001114.5e-050.000124

9.3e-05

1.9e-05

5.8e-053.3e-055.6e-05

8.2e-050.0001139e-05

8.2e-050.0001139e-05

5e-066e-065e-06

4e-061.1e-058e-06

3e-066e-063e-06

1.4e-055e-061.7e-05

9e-062.1e-054e-06

1.2e-055e-061.2e-05

3e-052.8e-052.9e-05

5e-062e-061.2e-05

2.9e-05

0.00021

0.000111

0.000111

9.9e-05

4.9e-05

5e-05

6.1e-057.4e-056e-05

6.1e-057.4e-056e-05

6.1e-057.4e-056e-05

0.0004230.0002530.0003740.00043e-05

0.0001480.000130.0001650.0001341.5e-05

0.0001480.000130.0001650.0001341.5e-05

0.0001530.0001230.0001230.000147

0.0001530.0001230.0001230.000147

0.0001228.6e-050.0001191.5e-05

0.0001228.6e-050.0001191.5e-05

0.000740.0086680.0026750.0102390.0002080.0007030.008907

0.000680.0086680.0026750.0102390.0001050.0006650.008907

1e-06

4e-060.0007340.0006465e-064e-06

1e-069e-062e-06

7e-061.3e-053e-06

3e-061e-063e-06

1.5e-052.2e-051.6e-05

0.0006410.0070570.0012570.0102397e-060.0006290.008907

6e-064e-065e-06

4e-05

3e-060.0008770.0007723e-063e-06

6e-050.0001033.8e-05

6e-053.3e-053.8e-05

7e-05

0.0004760.0002320.0004740.0016060.0006830.0004470.000226

0.000420.0002320.0003170.0016060.0004170.0003990.000226

0.000420.0002320.0003170.0016060.0004170.0003990.000226

0.0001570.000178

0.0001570.000178

5.6e-058.8e-054.8e-05

5.6e-058.8e-054.8e-05

0.0038870.0018040.00310.0017060.002820.0039110.000811

0.000109

0.000109

7.6e-050.0001980.0002256.6e-056.6e-05

7.6e-050.0001980.0002256.6e-056.6e-05

0.0038110.0018040.0027250.0017060.0022850.0038450.000745

0.0006430.0002710.0003070.0002470.0006620.000283

0.0007260.0002920.0002960.0002080.0007360.000254

0.0008160.000927

0.0016320.0009350.0010230.0017060.0007050.001619

0.000810.0003060.0002830.0001980.0008280.000208

0.0001770.000201

0.0001770.000201

0.0114590.0067980.00560.0068260.0029230.0109860.002157

0.0110520.0067670.0048530.0068260.0022420.0106050.001185

2e-05

8e-05

0.0010040.0009050.0010030.002612.3e-050.0009670.001147

7.4e-05

0

6.4e-054e-055.7e-05

1e-06

0.0098810.0058420.003850.0042160.0018920.009459

4.1e-05

2.6e-05

0.0001032e-054.5e-050.0001223.8e-05

0.0002130.0007470.0002170.000210.000933

4.2e-051.6e-054.9e-058.6e-05

6.5e-05

4.6e-050.0001744.9e-054.6e-050.000197

7.2e-050.0002923.5e-056.8e-050.000331

0.0002810.000319

5.3e-055.2e-054.7e-05

5.2e-050.0001224.3e-05

1e-052.1e-057e-06

1.7e-052.7e-051.4e-05

3e-069e-061e-06

3.9e-05

2.2e-052.6e-052.1e-05

7e-05

7e-05

0.0001423.1e-050.0001670.0001283.9e-05

4.6e-05

1.4e-051e-051.4e-051.1e-051.7e-05

8.1e-052.1e-056.9e-057.4e-052.2e-05

4.7e-053.8e-054.3e-05

6.2e-05

6.2e-05

4.3e-05

4.3e-05

8e-05

8e-05

8e-05

7.3e-050.0001027.1e-05

7.3e-050.0001027.1e-05

7.3e-050.0001027.1e-05

0.0002320.0006080.000215

0.0001150.0001720.000105

3.9e-055e-053.4e-05

2.7e-055.7e-053e-05

4.9e-056.5e-054.1e-05

6.4e-057.4e-055.8e-05

6.4e-057.4e-055.8e-05

0.000135

6.7e-05

6.8e-05

2.7e-05

2.7e-05

5.3e-057.6e-055.2e-05

5.3e-057.6e-055.2e-05

9e-05

9e-05

3.4e-05

3.4e-05

0.0657550.0384540.0356740.0242920.0413840.0629910.015462

0.0633760.0367030.0332290.0207790.0389770.060730.007648

0.000105

0.0001454.4e-050.0001110.0001540.003451

0.000112

0.0627680.0365590.0327860.0207790.0381070.060095

0.0001322.6e-050.0001765.1e-050.0001390.003971

0.0001012.4e-057.6e-050.0001050.000109

0.0002670.000303

0.0001132.5e-055.7e-050.000115

0.0001172.5e-055.5e-050.0001220.000117

0.002190.0017510.0021580.0035130.0019010.0020920.007555

0.002190.0017510.0021580.0035130.0019010.0020920.007555

3e-05

3e-05

0.0001430.000336

9e-05

0.0001430.000163

8.3e-05

0.000160.0001440.0001410.0001450.000199

6.9e-055.5e-055.7e-052e-05

1.6e-05

9.1e-050.0001448.6e-058.8e-050.000163

1.5e-05

1.5e-05

2.9e-052.9e-052.4e-051.5e-05

2.9e-052.9e-052.4e-051.5e-05

2.6e-050.00012.1e-05

2.6e-050.00012.1e-05

2.6e-050.00012.1e-05

2.6e-050.00012.1e-05

0.0935160.1277710.061580.0390480.0761450.0895120.045601

3.5e-05

3.5e-05

5e-06

3e-05

0.0933940.1277710.0606820.0390480.0758530.0894010.044688

4.5e-050.0001214.3e-051e-05

2.7e-054e-052.6e-055e-06

1.8e-058.1e-051.7e-055e-06

1.7e-05

1.7e-05

0.0001270.0001370.0001183.3e-05

0.0001270.0001370.0001183.3e-05

0.000120.000136

0.000120.000136

0.0001530.000174

0.0001530.000174

5e-05

5e-05

5.6e-057.3e-055.3e-05

2.7e-058e-061.6e-05

2.9e-056.5e-053.7e-05

8.1e-05

8.1e-05

0.0001820.000207

0.0001820.000207

0.0919740.1273150.0570220.0373420.0727960.0880260.041972

0.0919490.1273150.055790.0373420.0713970.0879990.041972

0.000310.000352

2.5e-050.0009220.0010472.7e-05

0.0001560.000292

0.000115

0.0001560.000177

0.0002440.0001270.0002040.0002394.1e-05

7.9e-058.8e-057.6e-05

0.0001650.0001270.0001160.0001634.1e-05

0.0001530.000174

0.0001530.000174

0.0001280.000145

0.0001280.000145

5e-06

5e-06

0.000118.7e-050.000105

0.000118.7e-050.000105

5.8e-05

5.8e-05

4.8e-054.2e-054.4e-05

4.8e-054.2e-054.4e-05

7.6e-05

7.6e-05

5e-050.0001144.3e-05

5e-050.0001144.3e-05

0.000119

0.000119

0.0005250.0004560.0020910.0017060.00040.0005110.002217

0.000350.0004560.0003550.0017060.0002610.0003370.000407

0.0015940.00181

0.0001750.0001420.0001390.000174

0.0002510.00029

0.0002510.000285

5e-06

2e-051.6e-05

2e-051.6e-05

4.5e-05

4.5e-05

0.0001950.000130.0002330.000203

0.0001430.000130.0001520.000148

5.2e-054.1e-055.5e-05

4e-05

0.0001690.000192

0.0001690.000192

0.0001220.0008980.0002920.0001110.000878

4.8e-056.8e-053.7e-05

4.8e-056.8e-053.7e-05

6.5e-050.0001250.0001425.9e-05

6.5e-050.0001250.0001425.9e-05

0.0007730.000878

0.0007730.000878

9e-068.2e-051.5e-05

9e-068.2e-051.5e-05

0.0001630.000310.0001484.6e-05

0.0001630.000310.0001484.6e-05

2.5e-059.1e-052.2e-05

2.5e-059.1e-052.2e-05

0.0001220.00010.0001134.6e-05

0.0001220.00010.0001134.6e-05

1.6e-050.0001191.3e-05

1.6e-050.0001191.3e-05

0.0006696.8e-050.0026360.0017780.0006920.001557

0.0006696.8e-050.0026360.0017780.0006920.001557

4.5e-056.9e-053.8e-051.7e-05

4.5e-056.9e-053.8e-051.7e-05

0.0002030.0001590.0002431.5e-05

3.5e-05

8.4e-053.8e-057.8e-05

3.5e-054.3e-058.1e-05

8.4e-054.3e-058.4e-051.5e-05

0.0001188e-050.000111

0.0001188e-050.000111

0.0003036.8e-050.0026360.001390.00030.001525

0.0006140.000697

0.0013430.001525

0.0003036.8e-050.0001890.0001360.0003

0.0001240.000141

0.0003660.000416

8e-05

8e-05

5.4e-050.0003644.8e-05

5.4e-050.0003644.8e-05

2.3e-050.0001941.9e-05

5e-060.0001032e-06

1.8e-059.1e-051.7e-05

3.1e-050.000172.9e-05

1.6e-058.1e-051.5e-05

1.5e-058.9e-051.4e-05

0.004510.0038860.0038730.0032120.0053590.004310.002981

0.0004233.5e-050.0002590.0007640.000404

0.0001230.000139

0.0001230.000139

2e-060.0001292e-06

2e-063e-062e-06

0.000104

2.2e-05

7e-050.0001156.2e-05

5.5e-05

7e-056e-056.2e-05

0.0001450.0001360.0001920.000137

0.0001450.0001360.0001920.000137

5.5e-053.6e-055.6e-05

2.4e-051.8e-052.4e-05

3.1e-051.8e-053.2e-05

6.3e-056.1e-055.7e-05

6.3e-056.1e-055.7e-05

8.8e-053.5e-059.2e-059e-05

8.8e-053.5e-059.2e-059e-05

0.0040870.0038510.0036140.0032120.0045950.0039060.002981

0.000221.2e-050.0001610.0002174.3e-05

0.0001116.2e-050.0001124.3e-05

0.0001091.2e-053.6e-050.000105

1.7e-05

4.6e-05

1.9e-058.5e-051.2e-05

1.9e-058.5e-051.2e-05

5e-050.0001090.0001244.4e-05

5e-050.0001090.0001244.4e-05

0.0001930.0002140.0001741.6e-05

0.0001074.7e-059.7e-051.6e-05

8.6e-050.000127.7e-05

4.7e-05

0.0002210.000251

0.0002210.000251

0.0001430.000162

0.0001430.000162

0.0034790.0038390.0030050.0032120.0033160.0033320.002922

0.0001770.0001490.0001610.000172

0.0033020.0038390.0028560.0032120.0031550.003160.002922

0.0001360.000154

0.0001360.000154

0.0001260.0001280.000127

0.0001260.0001280.000127

1.2e-054.6e-058e-06

1.2e-054.6e-058e-06

1.2e-054.6e-058e-06

1.2e-054.6e-058e-06

0.000238

0.000124

0.000124

0.000124

0.000114

0.000114

0.000114

0.0034150.0059510.0062110.0074280.0033740.0032540.00534

7.8e-05

7.8e-05

7.8e-05

0.0023140.0052110.0028980.005320.002790.002220.004752

1e-053e-051.3e-05

4e-061.1e-057e-06

1e-061e-061e-06

1e-067e-061e-06

4e-068e-064e-06

3e-06

6e-064.5e-055e-06

03e-06

6e-061.3e-055e-06

2.9e-05

0.0018780.0047860.0025240.0037140.0025550.00180.00449

0.0018750.0047860.0023820.0037140.0024410.0017980.00416

1e-068e-061e-06

2e-060.0001420.0001061e-060.00033

0.000420.0004250.0003740.0016060.000160.0004020.000262

1e-05

0.0004110.0004250.0003740.0016060.0003940.000262

3e-062.2e-052e-06

1e-061.1e-051e-06

3e-060.0001114e-06

06e-060

2e-0601e-06

0.0010540.000740.0033130.0021080.000430.0010030.000588

0.0010510.000740.0033130.0021080.0004150.0010.000588

0.0001130.000128

1.9e-050.0018560.0021088e-062.1e-05

4e-062e-066e-06

2e-060.0002320.00020402e-066.8e-05

1.3e-056e-064e-06

0.0001870.000212

5.2e-051.2e-055.4e-05

1e-055e-061.2e-05

1.1e-053e-061.3e-05

9.2e-051.9e-050.0001027e-06

0.0003890.0001360.0002148.4e-050.0004133.3e-05

4.5e-051.9e-054.9e-05

1.2e-050.0001136e-061.4e-050.000128

0.0001770.0001644.3e-050.000196

1.9e-050.0003720.0003281.1e-052.1e-057.1e-05

0.0001520.0001343.4e-054.8e-059e-06

4e-062e-064e-066e-05

2.4e-057e-062.3e-05

1e-053e-061e-05

1.2e-052.2e-057e-06

4e-061e-061e-06

3e-061.5e-053e-06

3e-061.5e-053e-06

4.7e-057.6e-053.1e-05

4.7e-057.6e-053.1e-05

4.7e-057.6e-053.1e-05

0.0021430.000680.0023560.0037140.0032930.0021120.00056

0.0010170.0005590.0005780.0017060.0007220.0009740.000417

0.000181

1.6e-05

2.7e-05

3.2e-05

4e-05

3.8e-05

2.8e-05

4.3e-05

4.3e-05

0.0010170.0005590.0005780.0017060.0004470.0009740.000417

2.3e-05

0.0010170.0005590.0005780.0017060.0004240.0009740.000417

5.1e-05

2.7e-05

2.4e-05

0.0011260.0001210.0017780.0020080.0025710.0011380.000143

3.5e-05

3.5e-05

0.0002680.000230.0003680.0002566.6e-05

3.2e-05

7.7e-05

4.8e-05

0.000130.0001146.8e-050.000129

5.4e-05

6.6e-05

0.0001380.0001165.5e-050.0001273.4e-05

0.0004730.0001210.0015480.0020080.0016660.0004796.2e-05

6e-05

0.0004730.0001210.0002720.0020086.8e-050.0004796.2e-05

3.5e-05

3.5e-05

0.0012760.001449

1.9e-05

4.6e-05

4.6e-05

0.0001320.0001420.0001181.5e-05

7.2e-05

1.5e-05

5.6e-051.7e-055.2e-05

1.6e-05

7.6e-053.7e-056.6e-05

4.4e-05

4.4e-05

0.0002350.000230.000229

1.7e-05

8e-054.6e-057.7e-05

2.2e-05

3.4e-05

1.4e-05

8.4e-051.1e-058.2e-05

2.1e-05

4.6e-05

7.1e-051.9e-057e-05

1.8e-054e-055.6e-05

1.8e-054e-055.6e-05

0.1291860.1568920.1869840.1801870.1849910.1579360.180946

0.0167590.017250.0131120.0136520.0143530.016520.009367

0.0167590.017250.0131120.0136520.0143530.016520.009367

9.5e-058.5e-059e-051.5e-05

2.3e-05

5.8e-052.4e-055.2e-05

3.7e-053.8e-053.8e-051.5e-05

0.0006030.0006854.6e-05

0.0006030.0006854.6e-05

0.0166310.017250.0125090.0136520.0134790.0164020.00926

7e-06

2.7e-051.1e-052.5e-05

3.3e-052.5e-059.1e-051.6e-05

1.5e-05

4.6e-056e-06

6.1e-053.4e-056.8e-05

3e-06

9.3e-05

2.1e-055e-062.9e-05

1.3e-05

1.7e-05

0.0021240.0093540.0019160.0055210.0006770.0024350.009086

0.0142180.0078710.0082060.0054210.0122980.013627

1.7e-058e-061.7e-05

5.3e-05

1.9e-059e-064e-061.6e-055e-06

2e-051e-05

3.9e-051.6e-050.0023870.002711.4e-053.2e-051.1e-05

3.8e-055.5e-053.9e-05

4.4e-05

3.4e-051.9e-05

3.4e-053e-062.3e-054.6e-05

1.1e-05

3.7e-051.3e-05

3.3e-050.0001042.8e-054.6e-05

3.1e-05

1.5e-05

3.3e-054.6e-052.8e-05

5.8e-05

0.0009475.4e-050.0018360.0027560.0007869.6e-05

0.0001520.0002460.0001181.6e-05

9.5e-050.0001047.7e-051.6e-05

3.4e-054.1e-053.1e-051.6e-05

3.8e-053e-053e-05

2.3e-053.3e-051.6e-05

6.3e-05

6.3e-05

5.7e-053.2e-054.1e-05

5.7e-053.2e-054.1e-05

4.7e-05

4.7e-05

4.6e-05

4.6e-05

4.6e-05

3.9e-054.9e-053e-05

3.9e-054.9e-053e-05

3.9e-054.9e-053e-05

6.5e-059.7e-055.3e-05

6.5e-059.7e-055.3e-05

2.9e-05

3.2e-05

6.5e-053.6e-055.3e-05

9.8e-050.0001329e-05

9.8e-050.0001329e-05

7.2e-057e-056.3e-05

2.6e-051e-062.7e-05

5.7e-05

4e-06

0.0005935.4e-050.0018360.0021860.0004958e-05

0.0003045.4e-050.0017040.0018980.000291.9e-05

0.0001250.000142

0.0003045.4e-050.000240.0002350.000291.5e-05

0.0001250.000142

4e-06

0.001090.001238

0.0001240.000141

0.0001720.0001320.0001240.0001616.1e-05

0.0001720.0001320.0001240.0001616.1e-05

4.7e-05

4.7e-05

5.2e-051.2e-054.2e-05

5.2e-051.2e-054.2e-05

6.5e-051.5e-052e-06

6.5e-051.5e-052e-06

9e-05

9e-05

0.0202250.0419740.0212520.0327240.0092780.0401560.027562

0.0199760.0419740.021120.0327240.0090140.0399670.027562

0.0002536.7e-050.0001920.0001120.0001920.000109

4.6e-05

4.6e-05

0.0002536.7e-050.0001921.8e-050.0001927.7e-05

1e-06

3.2e-05

1e-06

0.0188830.0418470.0204270.0327240.0087120.0391330.027264

6e-060.0029170.0033133e-067e-06

3.6e-050.0011820.0014070.0024098e-064.2e-050.001623

0.0031620.003591

0.000104

0.0012250.0045330.0007640.0032120.0002710.001290.00091

0.0011340.0003440.0004640.002610.0002140.0011690.000592

0.0007740.0002350.0002640.0001520.000816

0.0025950.0006610.0008890.0035130.0004160.00272

0.0119980.0330160.0093620.0155590.0032350.0305030.022428

0.0011150.0018760.0011980.0021080.0006520.0025860.001711

6.6e-05

6e-061.2e-056e-06

6e-061.2e-056e-06

6.6e-05

2.3e-05

2.7e-05

1.6e-05

0.0008346e-050.0005010.0001120.0006360.000189

0.0004816e-050.0002384.8e-050.0003770.000157

0.0003530.0002636.4e-050.0002593.2e-05

0.0002490.0001320.0002640.000189

7e-05

7e-05

0.000102

4.3e-05

3.5e-05

2.4e-05

0.0001720.0001324.4e-050.000133

0.0001720.0001324.4e-050.000133

7.7e-054.8e-055.6e-05

7.7e-054.8e-055.6e-05

7.1e-05

7.1e-05

7.1e-05

7.1e-05

0.0013520.0007050.0024980.0017060.0003370.0012190.00227

0.0013520.0007050.0011010.0017060.0003370.0012190.000683

0.00110.0007050.000970.0017060.0001830.0010470.000683

8e-06

0.0001172.1e-050.0001073.3e-05

5.8e-05

0.0008140.0007050.0006570.0017068.5e-050.0007830.000456

0.0001690.0001431.1e-050.000157

0.000170.000194

0.0001036.6e-057.4e-05

0.0001036.6e-057.4e-05

0.0001490.0001318.8e-059.8e-05

0.0001490.0001318.8e-059.8e-05

0.0013970.001587

0.0013970.001587

0.0013970.001587

0.0161020.006030.0120.005220.0210260.0155520.003499

0.0157540.006030.0120.005220.0204920.0152480.003499

0.0001590.00018

0.0001590.00018

8.5e-05

8.5e-05

0.0153320.006030.011580.005220.0196890.0148650.003499

0.0001950.000222

0.0003450.0002150.000189

0.0145980.006030.0061890.005220.0137270.0144620.003499

0.0047390.005381

0.0003890.0002420.000170.000403

0.0001450.0001790.000114

7.2e-059.3e-055.6e-05

7.3e-058.6e-055.8e-05

0.0001240.0001130.0001490.000117

0.0001240.0001130.0001490.000117

0.0001530.0001480.000210.000152

0.0001530.0001480.000210.000152

5.4e-050.000124.7e-05

5.4e-050.000124.7e-05

5.4e-056e-054.7e-05

6e-05

5.4e-05

5.4e-05

5.4e-05

5.8e-05

5.8e-05

5.8e-05

0.0002940.0003020.000257

6e-055.2e-055.1e-05

6e-055.2e-055.1e-05

0.000115.8e-050.000101

0.000115.8e-050.000101

8.3e-055.4e-057.1e-05

8.3e-055.4e-057.1e-05

6e-05

6e-05

4.1e-057.8e-053.4e-05

1e-051.9e-056e-06

1.7e-051.9e-051.4e-05

1.4e-054e-051.4e-05

0.0100740.0065830.006710.0089340.0090120.0095110.004884

3.4e-05

3.4e-05

3.4e-05

3.4e-052.8e-051.8e-05

3.4e-052.8e-051.8e-05

3.4e-052.8e-051.8e-05

0.00370.0029840.0031890.0059230.0033680.0034650.002247

5e-05

5e-05

0.0023090.0013780.0014430.0020080.0021490.0022110.000932

0.0022950.0013780.0014430.0020080.0021030.0022020.000932

1.4e-054.6e-059e-06

0.0003420.0007130.0009870.0020080.0001960.0002680.000729

1e-06

7e-06

3e-051.4e-053.4e-05

0

2e-050.0007130.0008070.0020089e-061.7e-050.000729

1.4e-05

0.0002920.000180.0001510.000217

4.4e-05

4.4e-05

0.0010180.0008930.0007590.0019070.0008590.0009580.000586

5.7e-05

1.4e-05

4e-051.6e-052.9e-05

1.8e-05

2.3e-05

0.0009350.0008930.0007590.0019070.0007040.0008960.000586

1.2e-05

4.3e-051.5e-053.3e-05

3.1e-057e-052.8e-05

3.1e-057e-052.8e-05

7.1e-058e-056.8e-05

7.1e-058e-056.8e-05

4.2e-054.3e-054.4e-05

2.9e-053.7e-052.4e-05

0.0002260.0001690.0006710.000217

0.0002260.0001690.0006710.000217

2.8e-05

3e-06

4.9e-053e-064.7e-05

7e-06

7e-06

0.0001450.0001690.0005530.000144

1.9e-05

2.3e-05

2.4e-05

3.2e-054e-062.6e-05

0.0059730.0035990.0033520.0030110.0047530.0056840.002637

0.0059730.0035990.0033520.0030110.0047530.0056840.002637

1.3e-056e-061.2e-053e-06

8e-063e-068e-061e-06

3.2e-051.4e-053.5e-05

5e-054.4e-05

3e-052.2e-052.7e-05

5.6e-058e-064.5e-05

9e-064e-068e-061.1e-05

0.0055880.0035990.0033520.0030110.0045660.0053420.002622

3.3e-051.6e-052.9e-05

2.7e-052.6e-052e-05

9e-062e-051.3e-05

1.4e-051.1e-051.3e-05

2.1e-051.6e-051.9e-05

2.1e-058e-061.7e-05

6e-062e-065e-06

8e-062e-066e-06

4.8e-052.9e-054.1e-05

1.6e-054.3e-051.5e-05

1.6e-054.3e-051.5e-05

1.6e-052.3e-051.5e-05

2e-05

5.4e-053.5e-054.4e-05

5.4e-053.5e-054.4e-05

5.4e-053.5e-054.4e-05

0.000940.0039450.0006020.0037140.0003560.0008360.00415

2e-065.9e-051e-06

2e-064.3e-051e-06

2e-061e-051e-06

3.3e-05

1.6e-05

1.6e-05

0.0009380.0039450.0006020.0037140.0002970.0008350.00415

0.0009380.0039450.0006020.0037140.0002640.0008350.00415

2.8e-05

0.0009280.0039450.0006020.0037140.0001750.0008240.00415

2.2e-05

1e-052.1e-051.1e-05

1.8e-05

3.3e-05

3.3e-05

0.000116

0.000116

4.9e-05

4.9e-05

6.7e-05

6.7e-05

0.000910.0035260.0029620.0064250.0007450.0012870.004478

8.7e-05

3.6e-05

3.6e-05

5.1e-05

5.1e-05

0.0002750.0021470.0023090.0002810.000201

4.9e-054.5e-055.1e-05

4.9e-054.5e-055.1e-05

6e-06

6e-06

1.4e-050.0020330.0023093.3e-051e-05

1.4e-050.0020330.0023091.3e-051e-05

02e-050

3.9e-054.7e-051.5e-05

2.9e-05

3.9e-051.8e-051.5e-05

0.0001730.0001140.000150.000125

0.0001350.0001149.1e-050.000125

3.8e-055.9e-05

0.0006350.0035260.0008150.0041160.0003770.0010860.004478

0.0006350.0035260.0008150.0041160.0003530.0010860.004478

2e-061e-065e-06

7e-064e-061e-05

0.0006190.0035260.0008150.0041160.0003150.0010650.004478

7e-068e-066e-06

1.8e-05

7e-06

2.4e-05

2.4e-05

1.8e-057.8e-051.3e-05

1.8e-057.8e-051.3e-05

1.8e-057.8e-051.3e-05

1.8e-057.8e-051.3e-05

0.0060870.0092190.0090240.0120470.0024780.0047190.010207

0.0060870.0092190.0090240.0120470.0024780.0047190.010207

8.5e-050.000110.0001256.4e-05

8.5e-050.000110.0001256.4e-05

5.2e-054e-055.6e-051.5e-05

5.2e-054e-055.6e-051.5e-05

6.8e-050.0001120.0001275.7e-05

6.8e-050.0001120.0001275.7e-05

0.00016.5e-050.000101

0.00016.5e-050.000101

0.0026220.002460.0013370.0023090.0006080.001640.002093

0.0023710.002460.0011820.0023090.00050.0013780.00206

0.0002510.0001550.0001080.000262

3.3e-05

2.5e-052.4e-054.1e-053.1e-05

2.5e-052.4e-054.1e-053.1e-05

0.000380.0001580.0013140.0003160.0003960.001259

0.0002278.2e-050.0002056.8e-050.000238

7.2e-05

7.4e-057.6e-056.1e-057.6e-05

7.9e-050.0001158.2e-05

0.0011090.001259

0.002730.0065580.0061510.0097380.0010090.0023410.006809

0.0044080.005005

0.0001232.4e-050.00012

0.0001980.000225

0.0015830.0062030.0008890.0046180.0003250.0013650.000892

0.0006550.0002440.0004610.0033130.000540.0006840.00045

0.0003690.0001110.0018070.000120.0001721.6e-05

0.0001950.000221

2.5e-054.3e-050.0001642.3e-05

4.8e-05

2.5e-054.3e-051.9e-052.3e-05

9.7e-05

9.7e-050.000470.0003538.7e-050.000534

9.9e-05

4.9e-05

4.9e-05

5e-05

5e-05

7.3e-05

7.3e-05

4e-05

3.3e-05

0.000470.000534

0.000470.000534

0.000470.000534

9.7e-050.0001818.7e-05

3.8e-055.7e-053.6e-05

3.8e-055.7e-053.6e-05

1.4e-053.8e-058e-06

1.4e-053.8e-058e-06

2.1e-054.9e-051.8e-05

2.1e-054.9e-051.8e-05

2.4e-053.7e-052.5e-05

2.4e-053.7e-052.5e-05

0.0419990.0371410.0642480.0670550.0559540.0549270.041128

0.0133310.01470.0127830.0230880.0058940.019160.019716

5.8e-05

5.8e-05

5.5e-054.1e-056.6e-05

5.5e-054.1e-056.6e-05

0.0047180.0030050.0028060.002510.0024620.0045580.002404

0.000123

3.9e-05

4.4e-05

0.0044120.0030050.0025280.002510.00230.0042320.00217

3.5e-05

0.0003060.0002784.4e-050.0003262.1e-05

9e-05

0.0001611.6e-058.6e-050.000167

6.9e-052.1e-057.5e-05

2e-05

1.5e-05

4e-06

1.9e-05

1e-06

9.2e-051.6e-056e-069.2e-05

7.5e-05

7.5e-05

3.9e-054e-057e-05

3.9e-054e-057e-05

9.3e-05

9.3e-05

7.7e-05

7.7e-05

0.0002120.0001912.5e-050.000222

0.0002120.0001912.5e-050.000222

0.0002970.0009450.0017620.002716.8e-050.0002850.004082

2e-05

9.6e-050.0009450.0015570.002711.1e-059.2e-050.003715

0.0002010.0002053.7e-050.0001930.000367

4.6e-050.0002045.3e-05

1.1e-051e-061e-05

1e-055.5e-059e-06

5e-062e-065e-06

2e-054.5e-052.9e-05

4.9e-05

5.2e-05

5.4e-055.1e-05

5.4e-055.1e-05

0.0003220.0001860.0001240.00033

0.0003220.0001860.0001240.00033

3.9e-05

3.9e-05

9e-05

9e-05

2.8e-05

2.8e-05

9.1e-05

9.1e-05

0.0018130.0054840.0033330.010540.0003030.0017540.006351

0.0012210.0047810.0005970.0045170.0001890.001201

0

0.0003460.0005630.0005270.0024094.9e-050.000350.002386

8.8e-050.0020040.0018074.9e-054.5e-050.003073

0.0001580.000140.0002050.0018071.6e-050.0001580.000892

5.8e-05

5.8e-05

8.9e-05

8.9e-05

0.0006170.0004240.0001990.0005573.6e-05

0.0002830.000265.4e-050.000308

1.5e-05

5.2e-055.7e-052.1e-05

0.00020.0001642.5e-050.0001752.1e-05

8.2e-056.3e-055.3e-05

7.8e-05

2.1e-05

5.7e-05

0.0009390.0030530.0012220.002710.0001520.0009170.003306

0.0006570.0030530.0011010.002712.6e-050.0006770.00328

0.0001390.0001211.2e-050.0001351.9e-05

2e-06

0.000101

1e-06

2.8e-052e-062.4e-05

0.0001158e-068.1e-057e-06

0.000290.0007340.000560.002610.0003340.0065450.003443

0.0001520.0007340.0004450.002610.0003240.0064210.003432

0.0001380.0001151e-050.0001241.1e-05

7.7e-05

7.7e-05

0.0037680.0014630.0022990.0020080.0010030.0035859.4e-05

9e-06

0.0003020.0002671.3e-050.0003035.1e-05

4.3e-05

1e-06

2e-06

3e-061.7e-05

0.0003370.0002885.1e-050.000316

0.000191.8e-050.0001557e-060.0001641e-05

7e-06

6e-06

1.3e-05

0.0002230.0001941.6e-050.0002191.6e-05

1e-06

5e-06

0.0027160.0014450.0013950.0020080.0008260.002583

0.0025940.0013510.0028880.002610.0014840.0028060.001126

0.0005191.4e-050.0003730.0001230.000440.000109

0.0002880.0002297.9e-050.000237

0.0002311.4e-050.0001442.1e-050.0002030.000109

2.3e-05

0.0001310.0001150.0001339.8e-05

6e-05

0.0001310.0001153.7e-059.8e-05

3.6e-05

0.0019440.0013370.00240.002610.0012280.0022680.001017

0.0003170.0011190.0004630.002615e-060.000314

0.000810.0010880.000796

0.0005918.4e-050.0006369.4e-050.0009293.9e-05

0.0007340.0001340.0002241.8e-050.0007210.000182

0.0003020.0002672.3e-050.000304

0.0001430.000163

0.0001430.000163

0.0001430.000163

0.0015615.1e-050.0009950.000830.0013590.000354

2e-054.7e-052.4e-05

2e-054.7e-052.4e-05

2.2e-052.7e-052.5e-05

2.2e-052.7e-052.5e-05

0.0007510.0006290.0003730.000650.000241

0.0003540.0002488.4e-050.0002970.000224

0.0002360.0001835.2e-050.0001855e-06

4.8e-056e-065.4e-054e-06

2.8e-056e-063.1e-058e-06

8.5e-0508.3e-05

0.0001980.000225

0.0007685.1e-050.0003660.0003830.000660.000113

8.1e-05

2.2e-05

5e-05

0.0003193.9e-050.0001810.0001230.0002983.7e-05

8.1e-051.2e-052.4e-056.6e-057.6e-05

0.0002350.0001851.1e-050.000191

0.0001333.1e-050.000105

4.1e-05

0.00024.4e-050.0001460.0002530.0001731.5e-05

8.7e-05

8.7e-05

0.000103

0.000103

0.00024.4e-050.0001466.3e-050.0001731.5e-05

1.4e-052e-051.2e-05

0.0001774.4e-050.0001463e-050.0001551.5e-05

9e-066e-066e-06

2e-06

5e-06

0.0230390.0209950.046320.0413570.0467020.0302890.019883

0.0002560.0001430.0001954.9e-050.0002529.3e-05

3e-057.5e-053.8e-053.6e-05

0.0002266.8e-050.0001951.1e-050.0002169.3e-05

0.0014960.0002520.0016510.0007720.0014460.000477

0.0001460.000166

1.8e-05

0.0004022.7e-050.0003283.8e-050.000347

0.0005184.9e-050.0004375.5e-050.000477

0.0003970.00045

0.0004725.9e-050.000220.0001270.000507

8e-06

1.2e-05

3.4e-05

4.2e-05

0.0001047.5e-050.0001233e-050.0001150.000311

0

0.0009980.001134

0.0009980.001134

0.0212870.02060.0434760.0413570.0447470.0285910.019313

0.0082710.009392

0.0047830.005432

8.2e-05

0.0012110.0007540.000550.005320.0001590.0012260.006173

0.0145290.016499

0.0012160.001381

0.0010380.001179

0.0083650.0095

7e-06

6.4e-050.0046030.0002670.015661.4e-056.3e-050.000104

0.0200120.0152430.0044570.0203770.0011020.0273020.013036

8.6e-05

8.6e-05

8.6e-05

0.0012740.0009730.0005420.001143.4e-05

0.0001880.0001572.8e-050.000169

0.0001880.0001572.8e-050.000169

0.0002160.0001696.5e-050.000174

0.0002160.0001696.5e-050.000174

0.0003760.0003220.0001940.0003571.9e-05

0.0002080.0001753.7e-050.000191.5e-05

3.9e-05

4.3e-05

2.4e-05

0.0001680.0001475.1e-050.0001674e-06

0.000103

0.000103

0.0003940.0003250.0001440.000351.5e-05

0.0001530.0001324.9e-050.0001481.5e-05

4.1e-05

0.0002410.0001935.4e-050.000202

0.00018e-069e-05

0

0.00018e-069e-05

0.01330.0304650.0519330.028710.0669870.0120180.072771

0.0096370.0247680.046830.0172660.0649680.008690.064825

8.8e-050.0001820.0001610.0001538.4e-05

7.4e-05

8.8e-050.0001820.0001612e-058.4e-05

5.9e-05

0.0094030.024360.0466690.0172660.0646370.0084470.064825

9.8e-05

0.005160.0226860.002990.0146560.0008470.0040380.030361

0.0010280.001167

0.0007410.000841

0.0005310.000603

0.0019050.0007810.0008520.000550.0019671.5e-05

0.00130.0008930.0005130.002610.000180.0013315.6e-05

0.0360650.0589680.031372

0.0017790.002021

9.4e-05

0.0006580.000747

1.5e-05

0.0008220.000933

0.0010380.0003820.0001980.0011114.6e-05

0.0003080.00035

0.0001460.0002260.0001780.000159

4.2e-05

2.5e-05

1e-05

3.4e-051.8e-054.1e-05

6.8e-050.0001063.7e-057.4e-05

3e-05

1.8e-056e-058e-062e-05

2.6e-056e-058e-062.4e-05

0.0036630.0056970.0051030.0114440.0020190.0033280.007946

6e-051.7e-057.4e-055.2e-05

4.9e-05

6e-051.7e-052.5e-055.2e-05

0.0036030.005680.0051030.0114440.001930.0032760.007946

2.5e-05

5.9e-05

5e-06

1.4e-05

0.000170.0001424.3e-050.000154

1.1e-05

0.0001612.8e-050.0001382.9e-050.000152

1.8e-05

9e-06

5.3e-05

2.3e-05

7.3e-057e-066.9e-051.8e-05

7.1e-05

0.0001284.8e-050.0001221.8e-050.0001240.000176

0.0001983.4e-050.0001682.7e-050.0001841.9e-05

5.7e-05

1.5e-051.4e-051.5e-05

5e-05

6.7e-051.4e-056.2e-056e-06

3.9e-05

0.0001360.0002180.0001910.0020082.7e-050.0001210.000417

3.5e-05

0.000140.0001163.2e-050.000124

0.0008820.0047560.000640.0049190.0001860.0008440.006706

2.1e-05

5e-06

3.8e-05

4.1e-05

0.0001510.0001324.7e-050.000148

0.0006650.0004250.0004490.0017060.0002640.0006370.000507

8e-06

4e-06

6e-06

0.0001240.000141

1.5e-05

5e-06

3e-05

1.6e-05

9.1e-051.8e-050.0024750.0028112.4e-058.2e-051.5e-05

1.1e-05

5.9e-051.1e-055.3e-05

6e-06

0.0001193e-060.000111

4e-05

1.7e-05

3.4e-05

2.6e-05

0.0001290.0001142.2e-050.000131

2.6e-05

7.4e-05

6.6e-051e-056.2e-053e-06

5.6e-05

0.0001490.000131

6e-06

0.0002040.0001530.0001613.3e-050.0002037.3e-05

3e-05

1.5e-05

1.5e-05

8.8e-05

8.8e-05

8.8e-05

8.8e-05

0.0002170.0001160.0005960.000173

0.0002170.0001160.0005960.000173

6.3e-05

6.3e-05

0.00011

6.7e-05

4.3e-05

5.4e-052.6e-055e-05

5.4e-052.6e-055e-05

0.0001630.0001160.0002670.000123

6.6e-05

0.0001320.0001167.8e-057.9e-05

1.2e-055.4e-051e-05

1.9e-052.9e-053.4e-05

4e-05

7.5e-05

7.5e-05

5.5e-05

5.5e-05

0.0001590.0002210.0004070.000132

0.0001590.0002210.0004070.000132

0.0001230.0001099e-059.5e-05

0.0001230.0001099e-059.5e-05

1.5e-050.000191.6e-05

6.6e-05

1.5e-057.1e-051.6e-05

5.3e-05

2.1e-050.0001120.0001272.1e-05

2.1e-050.0001120.0001272.1e-05

0.0001140.0003518.7e-05

0.0001140.0003518.7e-05

0.0001140.0003518.7e-05

0.0001140.0003518.7e-05

3.8e-055.5e-052.6e-05

2.3e-052e-051.7e-05

1.2e-05

1.5e-052.3e-059e-06

7e-068e-057e-06

4.7e-05

7e-063.3e-057e-06

4.8e-050.0001093.4e-05

1.6e-052.6e-058e-06

3e-063.3e-058e-06

2e-052.3e-051e-05

9e-062.7e-058e-06

5e-066.1e-055e-06

5e-066.1e-055e-06

1.6e-054.6e-051.5e-05

1.2e-052e-057e-06

4e-062.6e-058e-06

5.9e-050.0003193.5e-05

5.9e-050.0003193.5e-05

2.9e-050.0002632.1e-05

1.4e-050.0001291.1e-05

1e-061e-051e-06

1e-061e-051e-06

1e-057e-068e-06

1e-06

9e-067e-068e-06

2e-065.2e-051e-06

2e-065.2e-051e-06

1e-066e-051e-06

2.3e-05

1e-063.7e-051e-06

1.5e-056.8e-051e-05

7e-064e-057e-06

3e-062.3e-054e-06

4e-061.7e-053e-06

8e-062.8e-053e-06

8e-062.8e-053e-06

6.6e-05

6.6e-05

6.6e-05

3e-055.6e-051.4e-05

3e-055.6e-051.4e-05

2.9e-053.6e-051.4e-05

2.9e-053.6e-051.4e-05

1e-062e-050

1e-062e-050

1.6e-050.0001051.5e-05

1.6e-050.0001051.5e-05

1.6e-050.0001051.5e-05

1.6e-050.0001051.5e-05

1.6e-050.0001051.5e-05

1.6e-050.0001051.5e-05

3.3e-050.0002873.1e-05

3.3e-050.0002873.1e-05

3.3e-050.0002873.1e-05

3.3e-050.0002873.1e-05

8e-064.6e-054e-06

2e-064e-062e-06

6e-063.7e-052e-06

5e-06

1.5e-055.2e-052e-05

1.5e-055.2e-052e-05

1e-050.0001897e-06

5.2e-05

7.2e-05

1e-056.5e-057e-06

2e-050.0001849e-06

7e-064e-051e-06

7e-064e-051e-06

7e-064e-051e-06

7e-064e-051e-06

7e-064e-051e-06

1.3e-057.2e-058e-06

1.3e-057.2e-058e-06

1.3e-057.2e-058e-06

1.3e-057.2e-058e-06

1.3e-057.2e-058e-06

7.2e-05

7.2e-05

7.2e-05

7.2e-05

7.2e-05

3e-064.1e-052e-06

4.1e-05

4.1e-05

4.1e-05

4.1e-05

4.1e-05

3e-062e-06

3e-062e-06

3e-062e-06

3e-062e-06

3e-062e-06

0.0021120.0114920.003220.0509930.0022190.0019720.070092

0.0021120.0114920.003220.0509930.0022190.0019720.070092

0.0020910.0114920.003220.0509930.0020980.0019550.070092

0.0020910.0114920.003220.0509930.0020980.0019550.070092

0.0020910.0114920.003220.0509930.0020980.0019550.070092

1e-064e-061e-06

5e-062.2e-053e-06

1e-061.1e-051e-06

1e-065e-061e-06

0.0018420.0103590.0029380.0487850.0018440.001720.068596

1.8e-051e-052e-05

2.8e-05

2e-068e-062.1e-05

0.0002210.0011330.0002820.0022080.0001460.0001880.001496

2e-05

2.1e-050.0001211.7e-05

1.1e-054.7e-051e-05

1e-052.3e-051e-05

1e-051e-05

2.3e-05

1e-062.4e-050

1e-062.4e-050

7e-063.4e-055e-06

7e-063.4e-055e-06

7e-063.4e-055e-06

3e-064e-052e-06

3e-064e-052e-06

3e-064e-052e-06

0.0020730.0019620.002409

0.0020730.0019620.002409

0.0020730.0019620.002409

0.0020730.0019620.002409

0.0020730.0019620.002409

0.0020730.0019620.002409

6.3e-05

6.3e-05

6.3e-05

6.3e-05

6.3e-05

6.3e-05

2.6e-057.4e-051.1e-05

2.6e-057.4e-051.1e-05

2.6e-057.4e-051.1e-05

2.6e-057.4e-051.1e-05

2.6e-057.4e-051.1e-05

2.6e-057.4e-051.1e-05

0.0038850.0020190.002220.0024090.0039820.003730.001494

2.1e-055.4e-051.4e-05

2.1e-055.4e-051.4e-05

2.1e-055.4e-051.4e-05

2.1e-055.4e-051.4e-05

2.1e-055.4e-051.4e-05

0.0038640.0020190.002220.0024090.0039280.0037160.001494

0.0038640.0020190.002220.0024090.0039280.0037160.001494

1e-055.6e-051.1e-05

6e-062.7e-057e-06

6e-062.7e-057e-06

4e-062.9e-054e-06

4e-062.9e-054e-06

0.0038540.0020190.002220.0024090.0038720.0037050.001494

7e-065.1e-056e-06

7e-065.1e-056e-06

5e-062.8e-054e-06

5e-062.8e-054e-06

0.003840.0020190.002220.0024090.0037210.0036920.001494

0.003840.0020190.002220.0024090.0037210.0036920.001494

4.2e-05

4.2e-05

2e-063e-053e-06

2e-063e-053e-06

0.0042550.0025340.0024610.0024090.0039710.0040230.00189

0.0042550.0025340.0024610.0024090.0039710.0040230.00189

0.0001670.0003380.00012

0.0001390.0002859.6e-05

0.0001390.0002859.6e-05

2.5e-05

3.2e-054e-052e-05

1e-053.4e-058e-06

1e-05

1.9e-05

2e-051.8e-051.3e-05

2.8e-053e-052.1e-05

2.2e-053.6e-051.3e-05

1.1e-05

1.6e-053.9e-051.3e-05

1.1e-052.3e-058e-06

2.8e-055.3e-052.4e-05

2.8e-055.3e-052.4e-05

2.8e-055.3e-052.4e-05

0.0040880.0025340.0024610.0024090.0036330.0039030.00189

0.0040880.0025340.0024610.0024090.0036330.0039030.00189

1.3e-053e-051.1e-05

1.3e-053e-051.1e-05

0.0040360.0025340.0024610.0024090.0034960.0038610.00189

0.004020.0025340.0024610.0024090.0034250.0038470.001863

2e-06

1.4e-052.7e-05

6e-062.2e-056e-06

4e-061e-054e-06

6e-062.3e-054e-06

1.7e-056e-051.2e-05

1e-053e-056e-06

7e-063e-056e-06

2.2e-054.7e-051.9e-05

2.2e-054.7e-051.9e-05

9.9e-050.0044480.0004948.6e-050.004924

6e-065.8e-055e-06

6e-065.8e-055e-06

6e-065.8e-055e-06

6e-065.8e-055e-06

6e-065.8e-055e-06

5e-050.0001120.0001994.3e-05

4.1e-050.0001120.0001273.7e-05

4.1e-050.0001120.0001273.7e-05

4.1e-050.0001120.0001273.7e-05

4.1e-050.0001120.0001273.7e-05

9e-067.2e-056e-06

9e-067.2e-056e-06

9e-067.2e-056e-06

9e-067.2e-056e-06

1e-050.0001011.2e-05

1e-050.0001011.2e-05

1e-050.0001011.2e-05

1e-053.7e-051.2e-05

1e-053.7e-051.2e-05

6.4e-05

6.4e-05

1.9e-056.3e-051.5e-05

3e-065e-064e-06

3e-065e-064e-06

3e-065e-064e-06

0

3e-065e-064e-06

1.6e-055.8e-051.1e-05

1.6e-055.8e-051.1e-05

1.6e-055.8e-051.1e-05

1.6e-055.8e-051.1e-05

1.4e-057.3e-051.1e-05

1.4e-057.3e-051.1e-05

1.2e-054.9e-051e-05

1.2e-054.9e-051e-05

5e-062.4e-054e-06

7e-062.5e-056e-06

2e-062.4e-051e-06

2e-062.4e-051e-06

1e-06

2e-062.3e-051e-06

0

0.0043360.004924

0.0043360.004924

0.0043360.004924

0.0043360.004924

0.0043360.004924

2.5e-050.0002161.6e-05

2.5e-050.0002161.6e-05

2.5e-050.0002161.6e-05

2.5e-050.0002161.6e-05

5e-063.8e-052e-06

5e-063.8e-052e-06

6e-065.1e-056e-06

6e-065.1e-056e-06

5e-05

5e-05

9e-064.3e-056e-06

9e-064.3e-056e-06

5e-063.4e-052e-06

5e-063.4e-052e-06

1.6e-055.4e-051e-05

1.6e-055.4e-051e-05

1.6e-055.4e-051e-05

1.6e-055.4e-051e-05

1.6e-055.4e-051e-05

1.6e-055.4e-051e-05

0.0029930.0017660.0019250.0022080.0026170.0028650.00153

0.0029930.0017660.0019250.0022080.0026170.0028650.00153

0.0029930.0017660.0019250.0022080.0026170.0028650.00153

0.0029930.0017660.0019250.0022080.0026170.0028650.00153

0.0029930.0017660.0019250.0022080.0026170.0028650.00153

0.0029920.0017660.0019250.0022080.0025780.0028630.00153

1e-063.9e-052e-06

0.2770010.2315150.1732320.2219450.2336610.2774540.203546

5.5e-059.5e-053.8e-05

5.5e-059.5e-053.8e-05

5.5e-059.5e-053.8e-05

3e-054.9e-052e-05

3e-054.9e-052e-05

2.5e-054.6e-051.8e-05

2.5e-054.6e-051.8e-05

4.5e-050.0001353.6e-05

4.5e-050.0001353.6e-05

4.5e-050.0001353.6e-05

4.5e-050.0001353.6e-05

6.1e-05

4.5e-057.4e-053.6e-05

0.2764110.2314840.1729520.2219450.232330.2769450.20353

4.1e-055.7e-053.9e-05

4.1e-055.7e-053.9e-05

4.1e-055.7e-053.9e-05

4.1e-055.7e-053.9e-05

0.0084450.0048040.0063330.0077290.008950.0080620.005305

1.4e-054.6e-051.1e-05

1.4e-054.6e-051.1e-05

1.4e-054.6e-051.1e-05

6.8e-050.0001676.4e-050.000122

5.7e-05

5.7e-05

0.000122

0.000122

6.8e-055.4e-056.4e-05

6.8e-055.4e-056.4e-05

5.6e-05

5.6e-05

0.000230.0001940.000224

0.000230.0001940.000224

6.8e-055.4e-056.8e-05

7.7e-057e-057.4e-05

8.5e-057e-058.2e-05

0.0015360.0005370.001207

0.0015360.0005370.001207

0.0004730.000537

0.0010630.001207

0.0026710.0016160.0015820.0035130.0025230.0025280.001045

7.6e-056.2e-056.9e-05

7.6e-056.2e-056.9e-05

6e-05

6e-05

3.6e-052.5e-053e-05

3.6e-052.5e-053e-05

6.3e-050.0001625.4e-05

5.2e-05

6.1e-05

6.3e-054.9e-055.4e-05

0.0005850.0003470.0004120.0016060.0006840.0005570.000276

3e-067e-063e-06

2.6e-05

0.0005820.0003470.0004120.0016060.0004580.0005540.000276

6e-05

2.2e-05

1.9e-05

3.1e-05

7e-06

8e-06

2.2e-05

2.4e-05

9.1e-057.9e-058.3e-05

2.3e-052.3e-052.3e-05

6.8e-055.6e-056e-05

6.8e-057.6e-055.9e-05

2.6e-05

3.3e-052.7e-052.9e-05

3.5e-052.3e-053e-05

0.0017460.0012690.001170.0019070.0013680.001670.000769

0.0017460.0012690.001170.0019070.0013680.001670.000769

6e-067e-066e-06

6e-067e-066e-06

0.0042680.0024210.0025660.0024090.003890.0040840.00211

0.0041810.0024210.0025660.0024090.0038170.0040020.00211

0.0041810.0024210.0025660.0024090.0037310.0040020.00211

8.6e-05

8.7e-057.3e-058.2e-05

8.7e-057.3e-058.2e-05

5.8e-057.4e-055.7e-05

5.8e-057.4e-055.7e-05

5.8e-057.4e-055.7e-05

7.2e-056.7e-057.2e-05

7.2e-056.7e-057.2e-05

7.2e-056.7e-057.2e-05

2e-061.1e-053e-06

2e-061.1e-053e-06

2e-061.1e-053e-06

6e-050.0001715.5e-051.5e-05

5.4e-05

5.4e-05

6e-056e-055.5e-051.5e-05

6e-056e-055.5e-051.5e-05

5.7e-05

5.7e-05

5.6e-059.6e-055.4e-05

5.6e-055.3e-055.4e-05

5.6e-055.3e-055.4e-05

4.3e-05

4.3e-05

0.0009460.0007670.0006490.0018070.0011740.000910.000806

4.7e-05

4.7e-05

3.4e-05

3.4e-05

5.2e-05

4e-05

1.2e-05

4.3e-05

4.3e-05

4.7e-052.4e-054.5e-051.5e-05

4.7e-052.4e-054.5e-051.5e-05

0.0007960.0007670.0006490.0018070.000410.0007590.000791

0.0007960.0007670.0006490.0018070.000410.0007590.000791

4.9e-05

4.9e-05

3.6e-05

3.6e-05

0.0001030.0004260.000106

9.2e-05

3.6e-05

9.9e-05

5.2e-05

5e-05

0.0001039.7e-050.000106

5.3e-05

5.3e-05

0.00030.0004450.0002834e-06

0.000180.0001570.000171

3.1e-051.5e-053.1e-05

2.9e-051.3e-052.9e-05

2e-062e-062e-06

2.9e-054e-052.5e-05

2.9e-054e-052.5e-05

3.6e-052.9e-053.8e-05

1e-05

3.6e-051.9e-053.8e-05

4e-055.4e-053.3e-05

4e-055.4e-053.3e-05

4.4e-051.9e-054.4e-05

4.4e-051.9e-054.4e-05

0.000120.0002880.0001124e-06

5.6e-056e-055.3e-05

5.6e-056e-055.3e-05

6.3e-052e-06

6.3e-05

2e-06

6.4e-050.0001055.9e-052e-06

4.9e-05

6.4e-055.6e-055.9e-052e-06

6e-05

6e-05

5.3e-056.6e-054.9e-05

5.3e-056.6e-054.9e-05

5.3e-056.6e-054.9e-05

5.3e-056.6e-054.9e-05

6.8e-057.6e-056e-051.5e-05

6.8e-057.6e-056e-051.5e-05

6.8e-057.6e-056e-051.5e-05

6.8e-057.6e-056e-051.5e-05

0.000180.0001820.0001784.6e-05

0.000180.0001820.0001784.6e-05

5.8e-055.6e-055.9e-05

5.8e-055.6e-055.9e-05

6.1e-056.6e-056.1e-05

6.1e-056.6e-056.1e-05

6.1e-056e-055.8e-054.6e-05

6.1e-056e-055.8e-054.6e-05

0.0009740.0004740.0005770.0016060.0009760.0009340.000409

0.0009740.0004740.0005770.0016060.0009760.0009340.000409

0.0009740.0004740.0005770.0016060.0009760.0009340.000409

2.3e-055.2e-052.1e-05

4.4e-055.7e-054e-052.9e-05

2.9e-056.6e-052.9e-05

3.9e-056.5e-054e-05

0.0008390.0004740.0005770.0016060.0007360.0008040.00038

0.0001950.0001930.0003640.000188

0.0001950.0001930.0003640.000188

0.0001950.0001930.0003090.000188

0.0001950.0001930.0003090.000188

5.5e-05

5.5e-05

0.0051740.0027990.0034730.002510.0052610.0050330.003144

0.0051740.0027990.0034730.002510.0052610.0050330.003144

0.0051110.0027990.0034730.002510.0051990.0049730.003144

2.3e-05

5e-06

3.1e-05

4.2e-05

3.5e-05

5e-052.2e-055.5e-05

1.7e-05

3.4e-05

5e-05

8.3e-052e-058.7e-05

2e-05

5.2e-056.5e-055.3e-051.7e-05

2.6e-05

8e-05

3.7e-05

4.8e-05

6e-06

5.4e-052.7e-055.6e-051e-05

2e-05

0.0001760.0003720.000137

3.5e-05

7e-067e-068e-06

5.6e-05

2.3e-05

0.0001134.5e-051e-050.000125

0.0002280.000259

4.8e-055.5e-052.5e-050.000103

4.8e-05

0.004120.0025350.0025320.002510.003280.0039520.002029

4.5e-05

3.7e-051.1e-053.1e-054e-05

3e-06

8.9e-051.8e-059.7e-05

6.4e-05

4e-06

0.0001064.1e-057.1e-050.0001171.5e-05

1.7e-05

4.9e-055e-06

1.7e-05

6e-05

6.6e-054e-057e-05

0.0005370.000609

4e-06

5e-06

1.5e-05

6e-05

6.6e-05

9.1e-053.6e-050.000101

1.5e-05

3.2e-05

5e-061e-055e-06

9.7e-053.7e-055.7e-050

9.3e-053.9e-053.6e-050.000104

5.9e-05

2e-05

6.3e-056.2e-056e-05

6.3e-056.2e-056e-05

0.0028890.0013330.0016590.0020080.0028360.0027890.001131

0.0024920.0012670.0014340.0020080.0021060.0023890.000971

4.8e-057.1e-054.2e-057e-06

4.8e-057.1e-054.2e-057e-06

0.0024440.0012670.0014340.0020080.0020350.0023470.000964

4.8e-056.5e-054.6e-05

0.0023960.0012670.0014340.0020080.001970.0023010.000964

0.0001010.0001170.0001012.3e-05

0.0001010.0001170.0001012.3e-05

0.0001010.0001170.0001012.3e-05

0.0002966.6e-050.0002250.0006130.0002990.000137

0.0002966.6e-050.0002250.0006130.0002990.000137

0.0002966.6e-050.0002250.0006130.0002990.000137

0.012280.0490170.0133520.0909460.006770.0159750.051595

0.012280.0490170.0133520.0909460.006770.0159750.051595

0.0006180.0009350.000561

0.0006180.0009350.000561

0.0103040.0469970.0110490.0884360.0044310.0140740.048866

4e-06

5.9e-05

2.3e-05

0.0001880.0001610.0001838.7e-050.0001850.000433

2.9e-053e-053e-05

1.8e-05

0.0004410.000530.0003560.0486850.0001720.000422

1e-05

2e-062e-062e-06

3e-06

1.2e-05

1.4e-051.4e-051.4e-05

3.3e-054.8e-053.5e-05

0.0095670.0463060.010510.0397510.0039070.0133590.04842

3e-05

2e-06

3e-051e-052.7e-051.3e-05

0.0019760.002020.0016850.002510.0013340.0019010.002168

0.0019760.002020.0016850.002510.0013340.0019010.002168

7e-05

7e-05

4.5e-056.3e-054.3e-05

4.5e-056.3e-054.3e-05

4.5e-056.3e-054.3e-05

4.5e-056.3e-054.3e-05

0.1972960.117240.1068310.0740820.17040.1888680.092026

0.1972960.117240.1068310.0740820.17040.1888680.092026

8.4e-050.0001540.0001748.1e-051.6e-05

8.4e-050.0001540.0001748.1e-051.6e-05

0.1571280.094420.085520.0575190.1390170.1503790.071403

4.5e-050.0003450.0003914.5e-05

4.5e-050.0001330.0001524.4e-05

0.1569930.094420.0848940.0575190.1383060.1502460.071403

4.5e-050.0001480.0001684.4e-05

0.0400430.022820.0211570.0165630.0311490.0383680.020592

0.0006490.0002160.0003060.0033130.000790.0006340.000126

0.0393940.0226040.0208510.013250.0303590.0377340.020466

4.1e-056e-054e-051.5e-05

1.3e-052.5e-051.3e-051.5e-05

2.8e-053.5e-052.7e-05

0.015130.0088710.008620.0068260.0146960.0144660.006723

0.015130.0088710.008620.0068260.0146960.0144660.006723

4.9e-05

4.9e-05

0.0001461.5e-05

3.6e-05

4.3e-05

3.3e-05

1.5e-05

3.4e-05

1.3e-05

1.3e-05

0.0151080.0088710.008620.0068260.014440.0144550.006708

0.0151080.0088710.008620.0068260.014440.0144550.006708

2.2e-054.8e-051.1e-05

2.2e-054.8e-051.1e-05

1.9e-056e-051.7e-05

1.9e-056e-051.7e-05

1.9e-056e-051.7e-05

1.9e-056e-051.7e-05

0.02940.0419450.0287920.0311180.0179850.03620.039927

0.0004052.8e-050.001240.0021080.001370.0004010.000116

0.0004052.8e-050.001240.0021080.001370.0004010.000116

0.0001332.8e-050.0001690.0021085.2e-050.000131

0.000101

1.5e-05

0.0008170.000928

5.8e-05

0.0001290.0001250.0001760.00013

0.0001430.0001290.0001560.00014

0.0001220.0001260.000116

0.0001220.0001260.000116

0.0001220.0001260.000116

0.0041330.0094820.0165430.0091350.0087220.0041320.01757

0.0041330.0094820.0165430.0091350.0087220.0041320.01757

6.3e-056.2e-056.4e-05

3.1e-057.4e-050.002210.002512.2e-053.1e-05

0.0001560.0003870.0001010.000167

7.3e-059.1e-057.5e-05

0.0002390.0001730.0001430.000244

0.0001330.000151

0.0001760.0001560.0001680.000189

0.0001310.000148

0.0014890.001691

8.8e-05

7.8e-05

0.0002510.000285

4.1e-05

7.3e-05

8.9e-05

0.0006880.000781

0.0069890.007937

1.1e-051e-051.3e-05

0.0001190.000135

0.000170.000193

5.9e-05

6.1e-054.3e-055.7e-05

0.0001290.000146

0.0008110.000921

3.5e-05

0.0014270.008110.0011240.0066250.0003460.0013660.009589

0.0011560.000610.0007910.0008990.001194

0.0001197.6e-050.000118

0.000114

0.0003550.0002140.0002350.0002180.000346

0.0001036.2e-055.8e-050.000105

2.9e-05

7.5e-057.2e-057.3e-05

0.0002920.000331

1.5e-05

0.00030.000341

1.2e-052.5e-052e-061.3e-05

0.000127

7.6e-055.7e-057.7e-05

0.0001160.000132

0.0002360.000268

0.000127

3.6e-05

3.6e-05

3.6e-05

5.3e-056.5e-054.7e-051.5e-05

5.3e-056.5e-054.7e-051.5e-05

5.3e-056.5e-054.7e-051.5e-05

0.0222240.0309560.008990.0178670.0057230.0290580.021237

4.3e-050.0001270.0001443.7e-05

4.3e-050.0001270.0001443.7e-05

0.0221810.0309560.0088630.0178670.0055790.0290210.021237

2e-064e-062e-06

6e-062e-066e-06

4.2e-052.1e-054.3e-058e-06

3.4e-05

00

3e-062e-063e-06

5.8e-05

0

7e-050

5e-068e-064e-06

0.0151990.0238980.0044140.0119450.0011160.0189130.017525

2e-05

3.1e-05

4.7e-056e-064.6e-059e-06

4.3e-05

1.5e-05

0.0026460.0043410.0018170.0035130.0006380.0059810.003573

4.2e-05

2.8e-05

4e-069e-065e-06

2.6e-051.7e-053e-05

0003.3e-05

2e-062e-062e-06

3e-063.9e-051e-062e-06

4e-06

0.0038980.0023660.0026320.0024090.0032190.003735

5e-065e-065e-06

4.3e-05

6e-055e-056.8e-056e-06

3.2e-05

4e-053.3e-054.1e-05

0.00010702.7e-05

00.000102002.7e-05

1e-050.0001036e-061.2e-055e-06

4e-06

3.4e-05

0

1.1e-05

5e-053e-055.6e-05

6.3e-052.6e-056.7e-059e-06

0.0024630.0014790.0020190.0020080.0019430.0024460.000989

0.0019180.0014210.0015220.0020080.0013140.0019010.000893

0.0003530.0001230.0002130.0003550.000363

0.0010170.0010820.0004960.0020080.0001670.0009780.00081

0.0001556.4e-050.0001376.3e-050.0001562.3e-05

3.9e-05

0.0002799.6e-050.0002483e-050.000285

0.0001145.6e-052.8e-050.0001196e-05

2.5e-05

5.4e-05

2.5e-05

9e-06

0.0002450.000278

0.0001830.000208

3.3e-05

0.0005455.8e-050.0004970.0006290.0005459.6e-05

0.000105

0.0001450.0001250.0001360.000145

0.0002175.8e-050.0002130.0003090.000221

0.0001830.0001597.9e-050.0001799.6e-05

0.0039220.0050010.0031220.005120.0031430.0037610.003205

0.0039220.0050010.0031220.005120.0031430.0037610.003205

7e-05

7e-05

7.5e-057.3e-057e-052e-06

7.5e-057.3e-057e-052e-06

0.0001130.0001125e-06

0.0001130.0001125e-06

0.0034220.0050010.0029790.005120.0022560.0032830.003168

0.001660.0009120.0008340.0018070.0004170.001594

3.8e-05

0.0001350.000153

0.0017620.0040890.002010.0033130.0016480.0016890.003168

6.1e-05

6.1e-05

8.1e-05

8.1e-05

6.6e-05

6.6e-05

8e-056.3e-057.7e-05

8e-056.3e-057.7e-05

0.000140.0001430.0002670.0001323e-05

0.000140.0001430.0002670.0001323e-05

9.2e-050.0002068.7e-05

1e-06

3.2e-05

0

3e-06

9.2e-059e-058.7e-05

3.1e-05

4.9e-05

0.0002753.1e-050.000280.0004850.0002681.6e-05

0.0002753.1e-050.000280.0004850.0002681.6e-05

0.0002753.1e-050.000280.0004850.0002681.6e-05

0.0002753.1e-050.000280.0004850.0002681.6e-05

0.0002753.1e-050.000280.0004850.0002681.6e-05

0.0002150.0006160.000167

0.000110.0003098.5e-05

0.000110.0003098.5e-05

2.9e-054e-052.5e-05

2.9e-054e-052.5e-05

1.7e-051e-05

1.7e-051e-05

5.4e-05

5.4e-05

3.7e-050.000113e-05

2.1e-055e-051.7e-05

1.6e-056e-051.3e-05

9e-063.3e-054e-06

9e-063.3e-054e-06

1.8e-057.2e-051.6e-05

1.8e-057.2e-051.6e-05

0.0001050.0003078.2e-05

8.5e-050.0001756.7e-05

2.9e-050.0001132.6e-05

5.6e-05

2.9e-055.7e-052.6e-05

5.6e-056.2e-054.1e-05

5.6e-056.2e-054.1e-05

2e-050.0001321.5e-05

4.8e-05

4.8e-05

2e-058.4e-051.5e-05

2e-058.4e-051.5e-05

2.5e-050.0001583.2e-05

2.5e-050.0001583.2e-05

2.5e-050.0001583.2e-05

2.5e-050.0001583.2e-05

1.1e-054.3e-052e-05

1.1e-054.3e-052e-05

4e-064e-052e-06

4e-064e-052e-06

4e-063.6e-053e-06

4e-063.6e-053e-06

6e-063.9e-057e-06

6e-063.9e-057e-06

6.3e-050.0003117e-05

6.3e-050.0003117e-05

6.3e-050.0003117e-05

2.3e-056.9e-052e-05

8e-064.9e-055e-06

8e-064.9e-055e-06

1.5e-052e-051.5e-05

4e-06

1.5e-051.6e-051.5e-05

4e-050.0002425e-05

1.9e-055.4e-052.2e-05

1.9e-055.4e-052.2e-05

5.2e-05

5.2e-05

1.7e-059.7e-051.9e-05

4.2e-05

2.9e-05

1.7e-052.6e-051.9e-05

4e-063.9e-059e-06

4e-063.9e-059e-06

0.0097180.0199290.0178180.0329250.0065790.0094550.013562

0.0097180.0199290.0178180.0329250.0065790.0094550.013562

1.8e-058.9e-052.2e-05

1.8e-058.9e-052.2e-05

4e-063.3e-059e-06

4e-063.3e-059e-06

1.4e-052.7e-051.3e-05

1.4e-052.7e-051.3e-05

2.9e-05

2.9e-05

0.0096970.0199290.0178180.0329250.0064230.0094310.013562

1.8e-058.3e-051.7e-05

1.2e-054.4e-051.2e-05

8e-062e-058e-06

4e-062.4e-054e-06

6e-063.9e-055e-06

1e-068e-061e-06

8e-06

5e-062.3e-054e-06

0.0096790.0199290.0178180.0329250.006340.0094140.013562

0.0071760.0151210.0126240.0291110.0053560.0069880.009754

0.0035940.0113170.0047050.0060234.4e-050.0034760.008663

7e-06

0.0008580.0011190.0005920.0054212.6e-050.0008460.000249

1.2e-05

0.0008240.0011970.0009020.0039151e-050.0008070.000747

6e-06

1.9e-05

0.0003880.000140.0002830.0085325e-060.000387

0.0045930.005216

0.0015120.0013480.0015490.005221.1e-050.0014729.5e-05

0.0025030.0048080.0051940.0038140.0009840.0024260.003808

8e-060.0033534.3e-059e-060.003808

0.0024850.0048080.0018410.0038140.0009070.002407

7e-067e-067e-06

3e-062.7e-053e-06

3e-066.7e-052e-06

3e-066.7e-052e-06

2e-064e-051e-06

1.9e-05

2e-062.1e-051e-06

1e-062.7e-051e-06

1e-062.7e-051e-06

0.000310.0001420.000216

0.000310.0001420.000216

0.000310.0001420.000216

0.000310.0001420.000216

0.0002180.0001420.000128

0.0002180.0001420.000128

0.0002180.0001420.000128

9.2e-058.8e-05

9.2e-058.8e-05

9.2e-058.8e-05

0.08045900000000010.0535880.0483650.0472780.07628000000000010.0772690.04491

3e-060.0001143e-06

3e-060.0001143e-06

8.4e-05

8.4e-05

3.3e-05

3.3e-05

5.1e-05

5.1e-05

3e-063e-053e-06

3e-063e-053e-06

3e-063e-053e-06

3e-062.2e-053e-06

8e-06

1e-062.5e-050

1e-062.5e-050

1e-062.5e-050

1e-062.5e-050

1e-062.5e-050

1e-062.5e-050

2.2e-058.4e-051.6e-05

2.2e-058.4e-051.6e-05

2.2e-058.4e-051.6e-05

2.2e-058.4e-051.6e-05

2.2e-058.4e-051.6e-05

2.2e-058.4e-051.6e-05

0.0370510.0300010.023960.0232880.0360950.0354620.024104

0.0370510.0300010.023960.0232880.0360950.0354620.024104

3.1e-050.0015070.0017530.0021080.00063.3e-050.001325

4e-062.2e-053e-06

4e-062.2e-053e-06

1e-06

1e-06

9e-06

4e-061.1e-053e-06

2.7e-050.0015070.0017530.0021080.0005783e-050.001325

1e-061.9e-051e-06

1e-061e-050

9e-061e-06

1e-050.0015070.0017530.0021080.0004051.1e-050.001325

1e-050.0003570.0004051.1e-05

0.0015070.0013960.0021080.001325

3e-062.5e-053e-06

3e-062.5e-053e-06

1.3e-050.0001291.5e-05

1e-057.7e-051.1e-05

3e-065.2e-054e-06

2e-062.5e-052e-06

2e-061.3e-052e-06

2e-061.3e-052e-06

2e-061.3e-052e-06

01.2e-05

01.2e-05

01.2e-05

0.0354220.0204080.0200820.015860.0340780.0338970.015655

03e-050

1.1e-05

1.1e-05

1e-05

1e-05

09e-060

09e-060

0.0354220.0204080.0200820.015860.0340480.0338970.015655

01.9e-050

01.9e-050

8e-06

2e-06

6e-06

03.1e-050

02.1e-050

01e-050

0.0354210.0204080.0200820.015860.0339710.0338960.015655

0.0354210.0204080.0200820.015860.0339710.0338960.015655

1e-061.9e-051e-06

1e-061.9e-051e-06

0.0015960.0080860.0021250.005320.0013920.001530.007124

0.0015960.0080860.0021250.005320.0013920.001530.007124

01.1e-050

09e-060

2e-06

0.0015960.0080860.0021250.005320.0013660.001530.007124

04e-060

2e-066e-062e-06

1.2e-05

1e-061.4e-051e-06

0.0015930.0080860.0021250.005320.001330.0015270.007124

01.5e-05

01.5e-05

0.0433820.0235870.0244050.023990.0399620.0417880.020806

9e-068.3e-051e-05

9e-068.3e-051e-05

7e-064.9e-058e-06

06e-060

06e-060

7e-064.3e-058e-06

01.2e-050

3e-061.6e-053e-06

2e-061e-052e-06

2e-065e-063e-06

2e-063.4e-052e-06

2e-062.8e-051e-06

1e-06

02e-060

2e-06

08e-060

08e-060

2e-067e-061e-06

06e-061e-06

06e-061e-06

0.0313080.0164080.0167310.0121460.0283160.0301930.01559

0.0313080.0164080.0167310.0121460.0283160.0301930.01559

2e-061.6e-052e-06

2e-061.6e-052e-06

2e-061.6e-052e-06

0.0313060.0164080.0167310.0121460.02830.0301910.01559

5e-065e-054e-06

2.2e-050

3e-061.2e-053e-06

3e-06

2e-061.3e-051e-06

01.6e-050

1e-06

03e-060

01.2e-050

0.0001860.0001170.0001710.0015060.0001790.0001798.4e-05

0.0001860.0001170.0001710.0015060.0001790.0001798.4e-05

0.0311150.0162910.016560.010640.0280550.0300080.015506

0.0001220.000138

0.000104

1.1e-053.8e-059e-06

7e-062.9e-056e-06

0.0310970.0162910.0164380.010640.0277170.0299930.015506

2.9e-05

0.0008970.000530.0007450.0017060.0008860.000857

0.0008970.000530.0007450.0017060.0008860.000857

0.0008970.000530.0007450.0017060.0008860.000857

0.0008970.000530.0007450.0017060.0008860.000857

0.0008960.000530.0007450.0017060.0008640.000857

1e-062.2e-050

6e-067.6e-056e-06

6e-067.6e-056e-06

6e-067.6e-056e-06

01.1e-050

01.1e-050

6e-064.4e-056e-06

2e-061.4e-052e-06

1e-061e-051e-06

3e-061.4e-053e-06

06e-060

2.1e-05

1.2e-05

9e-06

6e-050.0002846.1e-05

5.8e-050.0001765.9e-05

1e-061.7e-052e-06

1e-061.7e-052e-06

1e-061.7e-052e-06

2e-063.1e-053e-06

2e-063.1e-053e-06

2e-063.1e-053e-06

1e-062e-051e-06

1e-062e-051e-06

1e-062e-051e-06

5.4e-050.0001085.3e-05

5.4e-050.0001085.3e-05

9e-063.9e-059e-06

4.5e-056.9e-054.4e-05

2e-067.5e-052e-06

2e-067.5e-052e-06

1e-063.2e-050

1e-063.2e-050

1e-061.9e-052e-06

1e-061.9e-052e-06

2.4e-05

2.4e-05

3.3e-05

3.3e-05

3.3e-05

3.3e-05

0.0058320.0031160.0033970.0042160.005360.0056030.002425

0.0043540.0024630.0025280.002510.0039490.0041730.001935

1.8e-050.0001551.7e-05

9e-060.0001049e-06

3.9e-05

9e-062.3e-059e-06

4.2e-05

9e-065.1e-058e-06

9e-065.1e-058e-06

2.6e-05

2.6e-05

2.6e-05

0.0043360.0024630.0025280.002510.0037680.0041560.001935

6.5e-050.0001656.4e-05

3.4e-058.8e-053.3e-05

3.1e-057.7e-053.1e-05

1.3e-054.6e-051.6e-05

1.3e-054.6e-051.6e-05

0.0042440.0024630.0025280.002510.0035380.0040640.001935

0.0042340.0024630.0025280.002510.0035010.0040520.001935

1e-053.7e-051.2e-05

1.4e-051.9e-051.2e-05

8e-061.4e-057e-06

6e-061e-065e-06

4e-06

0.0001970.0003640.000196

0.0001970.0003640.000196

2.1e-053.1e-052.1e-05

2.1e-053.1e-052.1e-05

1.7e-053.4e-051.6e-05

1.7e-053.4e-051.6e-05

2.1e-053.8e-052.1e-05

2.1e-053.8e-052.1e-05

3.2e-05

3.2e-05

1.4e-054.4e-051.3e-05

1.4e-054.4e-051.3e-05

2e-053.4e-051.9e-05

2e-053.4e-051.9e-05

1.9e-053.3e-051.9e-05

1.9e-053.3e-051.9e-05

8.5e-050.0001188.7e-05

3.8e-055.5e-053.9e-05

4.7e-056.3e-054.8e-05

0.0012810.0006530.0008690.0017060.0010470.0012340.00049

0.0012620.0006530.0007060.0017060.0008620.0012140.00049

9e-066.3e-051.1e-05

9e-066.3e-051.1e-05

1.2e-055.4e-051.2e-05

1.2e-055.4e-051.2e-05

0.0012410.0006530.0007060.0017060.0007450.0011910.00049

2.1e-054.3e-052.1e-05

9.7e-05

0.001220.0006530.0007060.0017060.0006050.001170.00049

1.9e-050.0001630.0001852e-05

1.9e-050.0001630.0001852e-05

1.9e-050.0001630.0001852e-05

0.0036770.0021960.0022320.0024090.0036140.0035310.001604

1.4e-050.0002391.4e-05

5e-065.9e-055e-06

5e-065.9e-055e-06

3e-062.8e-053e-06

2e-063.1e-052e-06

9e-060.000189e-06

03e-050

03e-050

1e-061.3e-051e-06

1e-061.3e-051e-06

5e-061.8e-055e-06

5e-061.8e-055e-06

1e-061.2e-051e-06

1e-061.2e-051e-06

06.7e-050

06e-060

9e-06

1e-06

9e-06

1.3e-05

4e-06

02e-060

4e-06

1.3e-05

02e-060

3e-06

1e-06

02.3e-050

9e-06

01.4e-050

2e-061.7e-052e-06

2e-061.7e-052e-06

3.5e-050.000124.6e-05

1.7e-054.1e-052.8e-05

01.2e-051e-06

01.2e-051e-06

1.7e-052.9e-052.7e-05

1e-061.4e-051.4e-05

1.6e-051.5e-051.3e-05

1e-061.3e-050

1e-061.3e-050

1e-061.3e-050

9e-064.2e-051e-05

01.5e-051e-06

01.5e-051e-06

9e-062.7e-059e-06

6e-061.3e-055e-06

4e-06

3e-061e-054e-06

8e-062.4e-058e-06

8e-062.4e-058e-06

8e-062.4e-058e-06

0.0036280.0021960.0022320.0024090.0032550.0034710.001604

0.0036280.0021960.0022320.0024090.0032550.0034710.001604

0.0036280.0021960.0022320.0024090.0032550.0034710.001604

0.0036270.0021960.0022320.0024090.0032350.0034690.001604

1e-062e-052e-06

0.0015930.0013370.00130.0035130.0013430.0015270.001187

0.0015930.0013370.00130.0035130.0013430.0015270.001187

0.0015930.0013370.00130.0035130.0013430.0015270.001187

0.0009440.000540.0006690.0017060.000760.0009030.00055

0.0009360.000540.0006690.0017060.0006880.0008960.00055

6e-06

3e-06

3e-060

1e-065e-061e-06

4e-06

05e-060

5e-06

6e-06

3e-06

4e-06

3e-06

1e-063e-060

5e-06

4e-060

6e-067e-066e-06

06e-060

0.0006490.0007970.0006310.0018070.0005790.0006240.000637

0.0006480.0007970.0006310.0018070.0005430.0006220.000637

03e-060

8e-06

3e-06

8e-06

1e-061.1e-051e-06

03e-061e-06

4e-06

4e-06
