# Supplementary material for: MetaMeta: integrating metagenome analysis tools to improve taxonomic profiling
Source: Microbiome. 2017 Aug 14;5:101. doi: 10.1186/s40168-017-0318-y (PMC5557516; doi:10.1186/s40168-017-0318-y)
Supplement: Supplementary file 2 — Additional File with interactive charts for all CAMI toy set results on default, very-precise and very-sensitive mode. File prefix S, M, and H for low, medium and high complexity, respectively. (TAR 3573 kb) [file 40168_2017_318_MOESM2_ESM.tar › H_S004__insert_180_very-precise.html]

Javascript must be enabled to view this page.

magnitude
magnitudeUnassigned

clark.parsed\_profile
dudes.parsed\_profile
final.metametamerge.profile
gottcha.parsed\_profile
kaiju.parsed\_profile
kraken.parsed\_profile
motus.parsed\_profile

0.9999929999999971.0000030.9999970000000010.9999900000000020.9999840.9999869999999981.000001

0.9171409999999980.9448980.9382620000000010.9508940000000020.9216990.9205749999999990.952588999999999

3e-064.1e-052e-06

4.1e-05

4.1e-05

4.1e-05

4.1e-05

4.1e-05

3e-062e-06

3e-062e-06

3e-062e-06

3e-062e-06

3e-062e-06

0.0001170.0003519e-05

0.0001170.0003519e-05

0.0001170.0003519e-05

0.0001170.0003519e-05

3.7e-055.4e-052.7e-05

1.2e-05

2.1e-051.9e-051.6e-05

1.6e-052.3e-051.1e-05

7e-067.9e-057e-06

7e-063.3e-057e-06

4.6e-05

5e-066.3e-055e-06

5e-066.3e-055e-06

4.8e-050.0001073.3e-05

1e-052.7e-058e-06

1.8e-052.3e-051e-05

4e-063.2e-057e-06

1.6e-052.5e-058e-06

2e-054.8e-051.8e-05

1.4e-052.1e-058e-06

6e-062.7e-051e-05

0.0081580.0069760.0067420.0079310.0075020.0078660.005832

0.0081580.0069760.0067420.0079310.0074420.0078660.005832

5.7e-050.0001075.6e-05

5.2e-059.4e-055e-05

5.2e-059.4e-055e-05

1e-061.2e-051e-06

1.2e-05

0

1.2e-054e-061.1e-05

4e-06

9e-068e-068e-06

1e-05

1e-05

2.1e-056e-062.3e-05

9e-067e-067e-06

1.2e-05

9e-06

5e-061.3e-056e-06

5e-061.3e-056e-06

5e-061.3e-056e-06

0.0046190.0026110.0035470.0027110.0044750.0044050.001992

0.0046190.0026110.0035470.0027110.0044750.0044050.001992

3.6e-054.1e-053.5e-05

2e-067e-061e-06

001e-06

2e-061.3e-051e-06

0

3.2e-052.1e-053.2e-05

0.0045830.0026110.0035470.0027110.0044340.004370.001992

2.9e-054e-051.6e-05

3.4e-05

9e-064.3e-057e-06

0.0045450.0026110.0035470.0027110.0043170.0043470.001992

0.0034820.0043650.0031950.005220.002860.0034050.00384

0.0034820.0043650.0031950.005220.002860.0034050.00384

0.0027730.0016790.0022550.0021080.0021260.0027870.00138

7.6e-059.4e-050.000208

0.0026970.0016790.0022550.0021080.0020320.0025790.00138

0.0007090.0026860.000940.0031120.0007340.0006180.00246

2.1e-05

0.0005170.0026860.000940.0031120.0004830.0004540.00246

2e-063e-061e-06

3e-064e-062e-06

5e-06

2e-061.5e-052e-06

2e-06

2.1e-054e-061.9e-05

1e-067e-061e-06

2.2e-054e-061.3e-05

5e-063e-064e-06

1.5e-051.2e-054e-06

2e-06

1e-052.4e-051e-05

1e-061e-060

1.5e-051.4e-051.1e-05

1e-066e-061e-06

7e-06

1e-061.5e-051e-06

2.3e-051.3e-052e-05

6e-06

3e-063e-06

3e-0603e-06

1.3e-05

1e-063e-060

6e-061e-065e-06

5e-061.2e-057e-06

3e-056e-063.2e-05

2e-062e-062e-06

9e-063e-069e-06

1.9e-05

6e-061.2e-056e-06

1e-062e-061e-06

4e-063e-06

01e-064e-06

9e-06

6e-05

6e-05

6e-05

6e-05

6e-05

2.5e-057.6e-051e-05

2.5e-057.6e-051e-05

2.5e-057.6e-051e-05

2.5e-057.6e-051e-05

2.5e-057.6e-051e-05

2.5e-057.6e-051e-05

0.0055650.0028920.0040880.0028110.0056130.0053370.002374

2e-055.2e-051.3e-05

2e-055.2e-051.3e-05

2e-055.2e-051.3e-05

2e-055.2e-051.3e-05

2e-055.2e-051.3e-05

0.0055450.0028920.0040880.0028110.0055610.0053240.002374

0.0055450.0028920.0040880.0028110.0055610.0053240.002374

0.0055340.0028920.0040880.0028110.0055040.0053130.002374

5e-062.8e-054e-06

5e-062.8e-054e-06

4e-05

4e-05

3e-063e-053e-06

3e-063e-053e-06

8e-064.7e-056e-06

8e-064.7e-056e-06

0.0055180.0028920.0040880.0028110.0053590.00530.002374

0.0055180.0028920.0040880.0028110.0053590.00530.002374

1.1e-055.7e-051.1e-05

4e-063e-054e-06

4e-063e-054e-06

7e-062.7e-057e-06

7e-062.7e-057e-06

0.0021280.0012530.0018740.0020080.0019040.0020380.001146

0.0021280.0012530.0018740.0020080.0019040.0020380.001146

0.0021280.0012530.0018740.0020080.0019040.0020380.001146

0.0021280.0012530.0018740.0020080.0019040.0020380.001146

0.0021280.0012530.0018740.0020080.0019040.0020380.001146

1e-063.2e-053e-06

0.0021270.0012530.0018740.0020080.0018720.0020350.001146

6.1e-050.0003193.6e-05

6.1e-050.0003193.6e-05

2.9e-055.8e-051.6e-05

2.9e-055.8e-051.6e-05

2e-062.2e-052e-06

2e-062.2e-052e-06

2.7e-053.6e-051.4e-05

2.7e-053.6e-051.4e-05

3.2e-050.0002612e-05

6.6e-05

6.6e-05

6.6e-05

1.5e-050.0001261e-05

1e-061e-051e-06

1e-061e-051e-06

1.2e-056e-068e-06

1.1e-056e-068e-06

1e-06

2e-065.1e-051e-06

2e-065.1e-051e-06

05.9e-050

03.5e-050

2.4e-05

1.7e-056.9e-051e-05

9e-064.1e-058e-06

5e-061.6e-054e-06

4e-062.5e-054e-06

8e-062.8e-052e-06

8e-062.8e-052e-06

2.9e-055e-052.1e-05

2.9e-055e-052.1e-05

2.9e-055e-052.1e-05

2.9e-055e-052.1e-05

2.9e-055e-052.1e-05

2.9e-055e-052.1e-05

6e-05

6e-05

6e-05

6e-05

6e-05

6e-05

0.0018930.002309

0.0018930.002309

0.0018930.002309

0.0018930.002309

0.0018930.002309

0.0018930.002309

3e-064.5e-052e-06

3e-064.5e-052e-06

3e-064.5e-052e-06

3e-064.5e-052e-06

3e-064.5e-052e-06

3e-064.5e-052e-06

8.1e-050.0007146.8e-05

3e-05

3e-05

3e-05

3e-05

3e-05

2.9e-053.9e-052e-05

2.9e-053.9e-052e-05

2.9e-053.9e-052e-05

2.9e-053.9e-052e-05

2.9e-053.9e-052e-05

8.9e-05

8.9e-05

8.9e-05

8.9e-05

8.9e-05

3e-050.0003743.1e-05

3e-050.0003743.1e-05

3e-050.0003743.1e-05

3e-050.0003743.1e-05

0.000191

3e-050.0001833.1e-05

2.2e-050.0001821.7e-05

1.5e-050.0001341.1e-05

1.5e-050.0001341.1e-05

1.5e-055.2e-051.1e-05

1.5e-055.2e-051.1e-05

3.6e-05

3.6e-05

4.6e-05

4.6e-05

7e-064.8e-056e-06

7e-064.8e-056e-06

7e-064.8e-056e-06

7e-064.8e-056e-06

2.6e-050.0001341.5e-05

2.6e-050.0001341.5e-05

2.6e-050.0001341.5e-05

2.6e-050.0001341.5e-05

3e-05

3e-05

9e-063.3e-057e-06

9e-063.3e-057e-06

1.7e-057.1e-058e-06

3.2e-05

1.7e-053.9e-058e-06

6.6e-050.0003156.9e-05

6.6e-050.0003156.9e-05

6.6e-050.0003156.9e-05

2.3e-056.7e-051.9e-05

1.5e-052.1e-051.4e-05

1.5e-051.7e-051.4e-05

4e-06

8e-064.6e-055e-06

8e-064.6e-055e-06

4.3e-050.0002485e-05

3e-064e-057e-06

3e-064e-057e-06

1.8e-050.0001021.9e-05

4.1e-05

1.8e-053e-051.9e-05

3.1e-05

2.2e-055.4e-052.4e-05

2.2e-055.4e-052.4e-05

5.2e-05

5.2e-05

7.4e-050.000276.6e-05

6.1e-050.0001715.6e-05

6.1e-050.0001715.6e-05

6.1e-050.0001715.6e-05

1.9e-055.1e-052e-05

1e-052.5e-051.2e-05

9e-062.6e-058e-06

7e-063.2e-055e-06

7e-063.2e-055e-06

2.1e-055.2e-051.9e-05

1.3e-052.7e-051.1e-05

8e-062.5e-058e-06

1.4e-053.6e-051.2e-05

1.4e-053.6e-051.2e-05

7e-063.7e-056e-06

7e-063.7e-056e-06

7e-063.7e-056e-06

7e-063.7e-056e-06

7e-063.7e-056e-06

6e-066.2e-054e-06

6e-066.2e-054e-06

6e-066.2e-054e-06

6e-066.2e-054e-06

6e-066.2e-054e-06

0.0001020.0055820.0005379e-050.004878

5.1e-050.0002354.3e-05

8e-068e-056e-06

8e-068e-056e-06

8e-068e-056e-06

8e-068e-056e-06

4.3e-050.0001553.7e-05

4.3e-050.0001553.7e-05

4.3e-050.0001553.7e-05

4.3e-050.0001553.7e-05

1.5e-057.1e-051.2e-05

1.5e-057.1e-051.2e-05

1.2e-054.8e-051e-05

1.2e-054.8e-051e-05

7e-062.4e-056e-06

5e-062.4e-054e-06

3e-062.3e-052e-06

3e-062.3e-052e-06

3e-062.2e-052e-06

1e-06

000

0.0055820.004878

0.0055820.004878

0.0055820.004878

0.0055820.004878

0.0055820.004878

1e-050.0001041.3e-05

1e-050.0001041.3e-05

1e-050.0001041.3e-05

1e-053.8e-051.3e-05

1e-053.8e-051.3e-05

6.6e-05

6.6e-05

7e-065.9e-055e-06

7e-065.9e-055e-06

7e-065.9e-055e-06

7e-065.9e-055e-06

7e-065.9e-055e-06

1.9e-056.8e-051.7e-05

1.6e-056.3e-051.3e-05

1.6e-056.3e-051.3e-05

1.6e-056.3e-051.3e-05

1.6e-056.3e-051.3e-05

3e-065e-064e-06

3e-065e-064e-06

3e-065e-064e-06

3e-065e-064e-06

0

0.005030.0031220.0044680.0028110.0051840.004813

0.005030.0031220.0044680.0028110.0051840.004813

0.005030.0031220.0044680.0028110.0051840.004813

3e-060.0001233e-06

3e-060.0001233e-06

3e-060.0001233e-06

0.0050270.0031220.0044680.0028110.0050610.00481

0.0050270.0031220.0044680.0028110.0050610.00481

0.0050270.0031220.0044680.0028110.0050610.00481

0.0020140.0109240.0039920.0486950.0021320.001880.062696

0.0020140.0109240.0039920.0486950.0021320.001880.062696

1.9e-050.0001191.6e-05

1e-054.8e-058e-06

1e-062.6e-050

1e-062.6e-050

9e-062.2e-058e-06

9e-068e-06

2.2e-05

3e-063.9e-052e-06

3e-063.9e-052e-06

3e-063.9e-052e-06

6e-063.2e-056e-06

6e-063.2e-056e-06

6e-063.2e-056e-06

0.0019950.0109240.0039920.0486950.0020130.0018640.062696

0.0019950.0109240.0039920.0486950.0020130.0018640.062696

0.0019950.0109240.0039920.0486950.0020130.0018640.062696

3e-069e-062.2e-05

3e-062e-052e-06

1.6e-051.1e-051.8e-05

1e-063e-061e-06

2.6e-05

1e-064e-061e-06

2.1e-05

01.2e-051e-06

0.0002220.001130.0003640.0022090.0001450.0001880.001311

0.0017490.0097940.0036280.0464860.0017620.0016310.061385

2.4e-050.0001573.1e-05

2.4e-050.0001573.1e-05

2.4e-050.0001573.1e-05

2.4e-050.0001573.1e-05

5e-064.3e-053e-06

5e-064.3e-053e-06

3e-063.6e-052e-06

3e-063.6e-052e-06

6e-063.9e-057e-06

6e-063.9e-057e-06

1e-053.9e-051.9e-05

1e-053.9e-051.9e-05

0.0235080.0169040.0176450.0279110.039910.0228910.018168

0.002470.0013340.0021240.0020080.0046660.0023871.5e-05

0.002470.0013340.0021240.0020080.0046660.0023871.5e-05

1.6e-050.0001552e-05

4.8e-05

4.8e-05

5e-05

5e-05

1.6e-055.7e-052e-05

1.6e-055.7e-052e-05

5e-05

5e-05

5e-05

1.7e-050.0001299e-06

1.2e-055.9e-055e-06

1.2e-055.9e-055e-06

5e-067e-054e-06

5e-067e-054e-06

1.3e-053.8e-051.4e-05

1.3e-053.8e-051.4e-05

1.3e-053.8e-051.4e-05

0.000336

7.9e-05

2.6e-05

2.8e-05

2.5e-05

0.000185

1.3e-05

3.2e-05

3.3e-05

1.2e-05

2.3e-05

3.4e-05

3.8e-05

7.2e-05

3.5e-05

3.7e-05

0.0022430.0012940.0021240.0020080.0027710.002152

2.1e-055.4e-051.9e-05

2.1e-055.4e-051.9e-05

1.5e-058.7e-051.6e-05

1.5e-058.7e-051.6e-05

3.7e-053.7e-05

3.7e-053.7e-05

8e-060.0001148e-06

8e-060.0001148e-06

0.000314

0.000314

1.3e-058.3e-051.3e-05

1.3e-058.3e-051.3e-05

0.0021490.0012940.0021240.0020080.0021190.002059

0.0021490.0012940.0021240.0020080.0021190.002059

0.0001814e-050.0011870.0001921.5e-05

1e-055e-051.2e-05

1e-055e-051.2e-05

5.2e-05

5.2e-05

0.0001644e-050.0010240.0001711.5e-05

0.000719

0.0001644e-050.0003050.0001711.5e-05

7e-066.1e-059e-06

7e-066.1e-059e-06

2.5e-050.0001161.7e-05

2.5e-050.0001161.7e-05

2.5e-050.0001161.7e-05

1.8e-057.8e-051.2e-05

1.8e-057.8e-051.2e-05

7e-063.8e-055e-06

7e-063.8e-055e-06

1.1e-059.2e-051.4e-05

1.1e-059.2e-051.4e-05

7e-063.7e-051e-05

7e-063.7e-051e-05

7e-063.7e-051e-05

4e-065.5e-054e-06

4e-065.5e-054e-06

4e-065.5e-054e-06

4.5e-050.0005924.8e-05

4.5e-050.0005924.8e-05

4.5e-050.0005924.8e-05

6.3e-05

6.3e-05

1.1e-057.5e-051.1e-05

1.1e-057.5e-051.1e-05

1.1e-050.0001691.1e-05

1.1e-057.9e-051.1e-05

9e-05

9e-060.0001851.1e-05

4.4e-05

4.3e-05

3.7e-05

9e-066.1e-051.1e-05

1.4e-050.00011.5e-05

1.4e-050.00011.5e-05

0.0063470.0062990.0038620.0063250.0142270.0062530.00645

0.0063470.0062990.0038620.0063250.0142270.0062530.00645

0.0001540.0001970.000154

0.0001540.0001970.000154

3.4e-054.5e-052.6e-05

1.4e-052.6e-051.8e-05

2.9e-053.2e-052.9e-05

7e-061.5e-058e-06

7e-061e-056e-06

3e-051.8e-053e-05

2.3e-053.8e-052.1e-05

1e-051.3e-051.6e-05

0.0061570.0062990.0038620.0063250.0138180.0060640.006435

7.7e-050.0001737.8e-05

7.7e-050.0001737.8e-05

3.6e-050.0001283.9e-05

3.6e-050.0001283.9e-05

0.000108

0.000108

1.4e-050.0001261.7e-05

6.8e-05

1.4e-055.8e-051.7e-05

2.3e-050.000122.5e-05

2.3e-050.000122.5e-05

0.000427

0.000115

0.000233

7.9e-05

0.000152

0.000152

9.3e-050.0001359.8e-05

3.2e-05

3.8e-054.3e-054e-05

5.5e-056e-055.8e-05

2.3e-050.0003832.5e-05

2.3e-050.0001872.5e-05

0.000196

2.3e-058.6e-052.1e-05

2.3e-058.6e-052.1e-05

0.000137

0.000137

2.2e-050.0001712.4e-05

2.2e-055.2e-052.4e-05

0.00011

9e-06

0.0034150.001780.0016010.0022090.0012740.00330.001443

3.4e-050.0001093.7e-05

1.6e-05

0.000125

6.3e-05

0.000524

0.0033810.001780.0016010.0022090.0005160.0032630.001364

0.000102

1.9e-05

8.3e-05

8e-060.0001059e-06

8e-060.0001059e-06

0.000103

0.000103

0.000889

0.000889

3.3e-050.0001443.8e-05

3.3e-050.0001443.8e-05

0.000192

9e-05

0.000102

8.5e-05

8.5e-05

0.000121

0.000121

0.0001850.0002770.000199

0.0001850.0002770.000199

0.0064324.6e-05

0.001584

0.000814

0.000834

4.6e-05

0.0032

3.2e-055.7e-053.8e-05

3.2e-055.7e-053.8e-05

1.7e-059.5e-052.1e-05

1.7e-059.5e-052.1e-05

0.001306

0.001306

1.9e-058.4e-051.6e-05

1.9e-058.4e-051.6e-05

3.3e-050.0001863.8e-05

3.3e-050.0001863.8e-05

2.8e-050.0001313e-05

2.8e-050.0001313e-05

5.7e-050.0001116.3e-05

5.7e-050.0001116.3e-05

0.0017170.0045190.0022610.0041160.0009040.0016460.00364

0.0017170.0045190.0022610.0041160.0009040.0016460.00364

0.0003020.000380.000339

9.9e-050.0001330.000109

4.6e-053e-055.7e-05

4.3e-058.6e-054.9e-05

7.3e-054.1e-057.9e-05

4.1e-059e-054.5e-05

8.1e-051.5e-05

8.1e-05

8.1e-05

1.5e-05

1.5e-05

2e-056.3e-051.9e-05

2e-056.3e-051.9e-05

2e-056.3e-051.9e-05

1.6e-056.8e-051.6e-05

1.6e-056.8e-051.6e-05

1.6e-056.8e-051.6e-05

0.0055810.003420.0044710.0030120.0068490.0053390.002814

0.0055810.003420.0044710.0030120.0068490.0053390.002814

0.0055810.003420.0044710.0030120.0068490.0053390.002814

6e-05

6e-05

1.3e-056.2e-051.4e-05

1.3e-056.2e-051.4e-05

0.001332

0.000643

0.000689

0.0055680.003420.0044710.0030120.0053110.0053250.002814

0.0055680.003420.0044710.0030120.0053110.0053250.002814

8.4e-05

8.4e-05

0.0090290.0058510.0071880.0165660.0133680.0088330.008889

0.000224

8.8e-05

8.8e-05

8.8e-05

0.000136

0.000136

0.000136

0.0090290.0058510.0071880.0165660.0131440.0088330.008889

0.00037

0.00037

0.00037

4.6e-050.0002254.7e-05

4.6e-050.0002254.7e-05

4.6e-050.0002254.7e-05

0.0012580.000310.0006040.0038160.0029720.0012850.002039

0.0012580.000310.0006040.0038160.0029720.0012850.002039

0.000253

3e-06

0.00092

3e-06

4.7e-050.0005035.4e-05

0.0001650.0019080.0003010.000176e-06

0.000832

0.0001110.0002930.000115

0.000366

0.0007550.000310.0006040.0019080.0007720.0007590.000272

3e-06

8.9e-050.0003679.6e-05

9.1e-050.0001179.1e-05

0.0041110.0022780.0035190.0061240.001820.0039370.002877

0.000231

0.000231

0.0041110.0022780.0035190.0061240.0015890.0039370.002877

0.0040330.0022780.0035190.0030120.0038610.002877

7.8e-050.0031120.0015897.6e-05

1.4e-050.0011151.4e-05

1.4e-050.000131.4e-05

1.4e-050.000131.4e-05

0.000985

0.000985

0.0016010.0025590.0016920.002610.0010510.0015310.002167

0.000222

0.000222

0.0016010.0025590.0016920.002610.0008290.0015310.002167

0.0015720.0025590.0016920.002610.0006570.0015040.002167

2.9e-050.0001722.7e-05

0.0001390.0013350.000134

5.7e-050.0002164.8e-05

5.7e-057.4e-054.8e-05

0.000142

8.2e-050.0011198.6e-05

8.2e-050.0008588.6e-05

0.000261

0.0018490.0007040.0013730.0040160.0040290.0018740.001806

0.0018490.0007040.0013730.0040160.0040290.0018740.001806

0.00019

1.5e-05

0.000369

0.0002174.4e-050.0016770.000226

1.7e-05

0.000105

0.000153

0.0001018e-06

0.000596

0.000117

4e-06

0.0011510.0004190.0009670.0021080.0007550.001156

0.0001210.0001530.000125

0.0002660.0002410.0004060.0019080.0002770.0002660.000889

9.4e-050.000390.000101

4e-06

1.5e-05

1.1e-050.0002271.1e-05

1.1e-050.0002271.1e-05

1.1e-050.0002271.1e-05

2e-063.3e-050

2e-063.3e-050

2e-063.3e-050

2e-063.3e-050

2e-063.3e-050

2e-063.3e-050

0.0079090.0064120.0510530.0061250.0501750.0080310.004542

0.0078730.0064120.0510530.0061250.0500410.0079970.004542

3.6e-05

3.6e-05

3.6e-05

3.6e-05

0.0034460.0059140.0032090.0061250.0036370.0033340.004528

1.8e-050.0002951.7e-05

1.8e-050.0002951.7e-05

0.000143

1.8e-054.9e-051.7e-05

0.000103

7e-061.5e-057e-06

7e-061.5e-057e-06

2e-06

4e-06

7e-069e-067e-06

1.5e-057.1e-051.2e-05

1.5e-057.1e-051.2e-05

1.5e-057.1e-051.2e-05

4.9e-054.8e-05

4.9e-054.8e-05

4.9e-054.8e-05

0.0015350.0007120.0009270.0019080.0015260.0014980.000285

0.0015350.0007120.0009270.0019080.0015260.0014980.000285

0.0015350.0007120.0009270.0019080.0015260.0014980.000285

0.0002810.0007040.000278

3.2e-054.8e-052e-05

3.2e-054.8e-052e-05

4.3e-058.4e-054e-05

1.2e-051e-05

3.1e-058.4e-053e-05

3e-057.5e-053.2e-05

3e-057.5e-053.2e-05

0.0001760.0004970.000186

2.3e-059e-062.4e-05

8e-06

0

1.3e-056.3e-051.1e-05

4e-061e-054e-06

1.8e-0502.1e-05

2.5e-059.9e-052.9e-05

1.3e-051.8e-051.1e-05

1.9e-05

5e-063.3e-056e-06

1.9e-051e-062.1e-05

2e-06

2e-05

3e-06

4e-06

1.6e-057.9e-051.7e-05

2.2e-057.5e-052.3e-05

1.2e-051.6e-051.1e-05

3e-06

1.5e-05

6e-061.1e-058e-06

9e-06

0.0015410.0052020.0022820.0042170.0010260.0014740.004243

0.0015410.0052020.0022820.0042170.0010260.0014740.004243

4.1e-05

0.0015410.0052020.0022820.0042170.0009850.0014740.004243

0.000440.0012020.000478

2e-050.0001411.8e-05

2e-050.0001411.8e-05

2e-050.0001411.8e-05

0.0001110.0002370.000128

0.0001110.0002370.000128

0.0001110.0002370.000128

0.0001210.0003510.000141

0.0001210.0003510.000141

4.1e-055.8e-054.4e-05

1.7e-050.0001162.1e-05

3e-061.1e-056e-06

7e-066e-069e-06

2.6e-058.4e-053.2e-05

2.7e-057.6e-052.9e-05

0.0001060.000240.000109

3e-053.2e-05

3e-053.2e-05

5.5e-050.0001865.5e-05

5.5e-050.0001865.5e-05

2.1e-055.4e-052.2e-05

2.1e-055.4e-052.2e-05

8.2e-050.0002338.2e-05

8.2e-050.0002338.2e-05

3.2e-059.9e-053.2e-05

5e-050.0001345e-05

0.0034180.0004980.0478440.0436250.0035951.4e-05

0.0478440.041816

0.0478440.041816

0.0478440.041816

0.0008092.9e-050.0006690.000858

0.0002140.0001450.000223

0.0002140.0001450.000223

0.0005952.9e-050.0005240.000635

0.000181

0.0001930.0001580.000203

0.0004022.9e-050.0001850.000432

0.0026090.0004690.001140.0027371.4e-05

0.0001886.2e-050.000206

0.0001886.2e-050.000206

0.0006380.0001460.0003110.000574

8.1e-05

0.0002175.7e-050.000241

0.0003035.8e-050.0001730.000333

0.0001188.8e-050

0.0003293.6e-050.000369

0.0003293.6e-050.000369

0.0014540.0002870.0007670.0015881.4e-05

0.000139

0.0003065.9e-050.0001280.000349

0.0003595.9e-050.0001660.0003791.4e-05

0.0004239e-050.0001890.000459

0.0003667.9e-050.0001450.000401

0.0002050.0002860.00021

9.6e-050.0001510.000101

9.6e-050.0001510.000101

9.6e-050.0001510.000101

0.0001090.0001350.000109

0.0001090.0001350.000109

0.0001090.0001350.000109

0.0001360.0004440.000144

0.0001360.0004440.000144

0.0001360.0004440.000144

0.0001360.0004440.000144

0.0002280.0008110.000236

0.0001120.0006230.000114

8.1e-05

4.1e-05

4e-05

0.0001120.0005420.000114

0.0001120.0005420.000114

2.2e-054.5e-052.5e-05

2.2e-054.5e-052.5e-05

2.2e-054.5e-052.5e-05

5.5e-050.0001165.5e-05

2.8e-056.9e-053.2e-05

2.8e-056.9e-053.2e-05

2.7e-054.7e-052.3e-05

2.7e-054.7e-052.3e-05

3.9e-052.7e-054.2e-05

3.9e-052.7e-054.2e-05

3.9e-051.5e-054.2e-05

1.2e-05

3.6e-050.0001343.4e-05

3.6e-050.0001343.4e-05

3.6e-050.0001343.4e-05

3.6e-050.0001343.4e-05

1.7e-056.4e-051.5e-05

1.9e-057e-051.9e-05

0.005190.0031090.0039380.0027110.0047650.0049160.00254

0.005190.0031090.0039380.0027110.0047650.0049160.00254

0.0050210.0031090.0039380.0027110.0044360.0047930.00254

0.0050210.0031090.0039380.0027110.0044360.0047930.00254

1.7e-056.1e-051.2e-05

7e-063.1e-056e-06

1e-053e-056e-06

2.3e-054.5e-052e-05

2.3e-054.5e-052e-05

1.4e-053e-051.4e-05

1.4e-053e-051.4e-05

0.0049670.0031090.0039380.0027110.00430.0047470.00254

0.0049490.0031090.0039380.0027110.0042180.0047310.002486

4e-061.1e-054e-06

9e-062.4e-059e-06

1.7e-055.4e-05

5e-062.7e-053e-06

3e-06

0.0001690.0003290.000123

2.9e-055.2e-052.5e-05

2.9e-055.2e-052.5e-05

2.9e-055.2e-052.5e-05

0.000140.0002779.8e-05

0.000140.0002779.8e-05

1.9e-051.7e-051.4e-05

1.1e-052.3e-059e-06

1.8e-053.6e-051.3e-05

2.1e-053.6e-051.4e-05

1.9e-05

3e-053.1e-052.2e-05

2.4e-05

1e-05

3.1e-053.8e-051.8e-05

1e-053.4e-058e-06

9e-06

0.2679360.2236590.1977070.2128510.2274770.2678180.199102

4.4e-050.0001323.4e-05

4.4e-050.0001323.4e-05

4.4e-050.0001323.4e-05

4.4e-050.0001323.4e-05

4.4e-057.1e-053.4e-05

6.1e-05

0.0002833.2e-050.0004930.0002731.6e-05

0.0002833.2e-050.0004930.0002731.6e-05

0.0002833.2e-050.0004930.0002731.6e-05

0.0002833.2e-050.0004930.0002731.6e-05

0.0002833.2e-050.0004930.0002731.6e-05

0.2673430.2236270.1977070.2128510.2261380.2673040.199086

1.9e-056e-051.7e-05

1.9e-056e-051.7e-05

1.9e-056e-051.7e-05

1.9e-056e-051.7e-05

0.0001840.0003510.000174

0.0001840.0003510.000174

5.3e-05

5.3e-05

0.0001840.0002980.000174

0.0001840.0002980.000174

0.1879370.1111360.131150.069980.1630190.1797780.087419

0.1879370.1111360.131150.069980.1630190.1797780.087419

4.2e-056.2e-054.3e-054.1e-05

2.9e-053.6e-052.9e-053.1e-05

5e-06

1.3e-052.6e-051.4e-055e-06

0.1481620.088680.103970.0541160.131810.1416760.067446

4.5e-050.0001444.5e-05

4.3e-050.0003684.3e-05

4.7e-050.0001654.5e-05

0.1480270.088680.103970.0541160.1311330.1415430.067446

0.0396430.0224560.027180.0158640.0309670.037970.019932

0.0386860.0221270.0264270.0129520.0299690.0370280.019551

0.0009570.0003290.0007530.0029120.0009980.0009420.000381

9e-050.000188.9e-05

9e-050.000188.9e-05

0.0041250.0055220.0039850.0054210.0035660.003960.003808

0.0041250.0055220.0039850.0054210.0035660.003960.003808

9.6e-050.0002139e-05

1e-06

9.6e-059.1e-059e-05

0

3.3e-05

4e-06

5.2e-05

3.2e-05

8.1e-05

8.1e-05

7.7e-056.5e-057.5e-05

7.7e-056.5e-057.5e-05

7.3e-057.4e-057e-051.5e-05

7.3e-057.4e-057e-051.5e-05

0.0002235e-050.0004670.0002171.7e-05

0.0002235e-050.0004670.0002171.7e-05

0.0035360.0054720.0039850.0054210.0024620.0033890.003759

0.000156

0.0020020.0046320.002970.0036140.0018790.0019180.003759

0.0015340.000840.0010150.0018070.0003910.001471

3.6e-05

7.1e-05

7.1e-05

7e-05

7e-05

6.3e-05

6.3e-05

0.000120.0001191.7e-05

0.000120.0001191.7e-05

0.0091950.0051980.00650.0078310.0096890.0087670.005799

0.0011280.0009110.0009980.0019080.0013530.0010840.000978

3.8e-05

3.8e-05

0.0001270.0005140.000125

0.000113

0.000119

5.8e-05

4.4e-05

0.0001270.000120.000125

6e-05

0.000950.0009110.0009980.0019080.0004880.0009090.000948

0.000950.0009110.0009980.0019080.0004880.0009090.000948

5.5e-05

5.5e-05

5.5e-05

1.4e-05

4.1e-05

4.5e-05

4.5e-05

5.1e-052.5e-055e-053e-05

5.1e-052.5e-055e-053e-05

5e-05

5e-05

4.8e-05

4.8e-05

3.5e-05

3.5e-05

0.0026830.0015790.0019160.0034130.0025390.0025330.000834

3.8e-052.4e-053.2e-05

3.8e-052.4e-053.2e-05

7.2e-057.6e-056.2e-05

3.4e-052.6e-053e-05

3.8e-052.3e-053.2e-05

2.7e-05

0.0015930.001140.0012740.0018070.0012550.0015230.000533

0.0015930.001140.0012740.0018070.0012550.0015230.000533

6.6e-050.0001695.7e-05

5.4e-05

6.2e-05

6.6e-055.3e-055.7e-05

7e-068e-066e-06

7e-068e-066e-06

8.1e-056.6e-057.3e-05

8.1e-056.6e-057.3e-05

5.9e-05

5.9e-05

0.0007410.0004390.0006420.0016060.0008040.0007060.000301

2.1e-05

0.0007380.0004390.0006420.0016060.0005860.0007030.000301

3e-066e-063e-06

2.6e-05

2.3e-05

7e-06

5.9e-05

1.9e-05

3.1e-05

6e-06

2e-05

8.5e-057.8e-057.4e-05

6.2e-055.5e-055.3e-05

2.3e-052.3e-052.1e-05

0.0005150.001455

0.0005150.001455

0.001425

0.0005153e-05

6.1e-057.6e-056e-05

6.1e-057.6e-056e-05

6.1e-057.6e-056e-05

2e-061e-052e-06

2e-061e-052e-06

2e-061e-052e-06

6.4e-050.0001895.9e-053e-06

6.4e-056.5e-055.9e-05

6.4e-056.5e-055.9e-05

6e-053e-06

6e-05

3e-06

6.4e-05

6.4e-05

5.6e-050.0001015.5e-05

4.4e-05

4.4e-05

5.6e-055.7e-055.5e-05

5.6e-055.7e-055.5e-05

1.1e-054.9e-059e-06

1.1e-054.9e-059e-06

1.1e-054.9e-059e-06

7.8e-056.9e-057.5e-05

7.8e-056.9e-057.5e-05

7.8e-056.9e-057.5e-05

0.0047790.0027080.0035860.002510.0043940.0045690.002115

0.0046850.0027080.0035860.002510.0043130.0044820.002115

0.0046850.0027080.0035860.002510.0042170.0044820.002115

9.6e-05

9.4e-058.1e-058.7e-05

9.4e-058.1e-058.7e-05

0.0002510.0002020.0002441.5e-05

0.0002510.0002020.0002441.5e-05

7.9e-057.1e-057.8e-051.5e-05

9.5e-057.6e-059.1e-05

7.7e-055.5e-057.5e-05

8.2e-050.0001927.7e-050.000399

6.2e-05

6.2e-05

6.1e-05

6.1e-05

8.2e-056.9e-057.7e-05

8.2e-056.9e-057.7e-05

0.000399

0.000399

0.0002980.0004560.00028

0.0001740.0001610.000165

3.1e-051.4e-053.1e-05

2.9e-051.2e-052.9e-05

2e-062e-062e-06

2.9e-054e-052.5e-05

2.9e-054e-052.5e-05

4e-052e-054e-05

4e-052e-054e-05

3.6e-053.1e-053.8e-05

3.6e-052.1e-053.8e-05

1e-05

3.8e-055.6e-053.1e-05

3.8e-055.6e-053.1e-05

0.0001240.0002950.000115

6.2e-05

6.2e-05

6.2e-05

6.2e-05

5.8e-056e-055.5e-05

5.8e-056e-055.5e-05

6.6e-050.0001116e-05

6.6e-055.9e-056e-05

5.2e-05

4.3e-055.8e-054.1e-05

4.3e-055.8e-054.1e-05

4.3e-055.8e-054.1e-05

4.3e-055.8e-054.1e-05

5.4e-056.9e-054.8e-05

5.4e-056.9e-054.8e-05

5.4e-056.9e-054.8e-05

5.4e-056.9e-054.8e-05

0.012110.0467560.0154690.0862460.0072390.0155560.048751

0.012110.0467560.0154690.0862460.0072390.0155560.048751

0.0013580.000594

0.0013580.000594

0.0097840.0443630.0129660.0835350.0042390.0133210.045884

3.3e-053.2e-053.6e-05

2e-06

2.3e-05

2.8e-051e-052.6e-05

1.2e-05

0.0001860.0001578.2e-050.0001820.000486

3e-05

3.3e-054.4e-053.4e-05

2e-062e-064e-06

0.000420.0004950.0458840.0001650.0004

2e-06

1.2e-05

6.2e-05

1.6e-051.5e-051.5e-05

1.7e-05

4e-06

0.0090660.0437110.0129660.0376510.0037250.0126240.045398

7.6e-05

7.6e-05

0.0023260.0023930.0025030.0027110.0015660.0022350.002273

0.0023260.0023930.0025030.0027110.0015660.0022350.002273

4.5e-056.4e-054.2e-05

4.5e-056.4e-054.2e-05

4.5e-056.4e-054.2e-05

4.5e-056.4e-054.2e-05

0.0014210.0007330.0011090.0017070.0013630.0013610.000602

0.0014210.0007330.0011090.0017070.0013630.0013610.000602

0.0014210.0007330.0011090.0017070.0013630.0013610.000602

2.5e-055.7e-052.4e-05

4.2e-056.5e-054.3e-05

2.8e-056.8e-052.8e-051.5e-05

0.001280.0007330.0011090.0017070.0011150.0012240.000573

4.6e-055.8e-054.2e-051.4e-05

0.0133440.0077830.0099380.0060240.0130770.0127460.006074

0.0133440.0077830.0099380.0060240.0130770.0127460.006074

0.0133180.0077830.0099380.0060240.0128090.0127330.006074

0.0133180.0077830.0099380.0060240.0128090.0127330.006074

1.3e-05

1.3e-05

5.1e-05

5.1e-05

2.6e-054.8e-051.3e-05

2.6e-054.8e-051.3e-05

0.000156

3.4e-05

3.7e-05

3.8e-05

4.7e-05

7.1e-057.7e-056.2e-05

7.1e-057.7e-056.2e-05

7.1e-057.7e-056.2e-05

7.1e-057.7e-056.2e-05

0.0057080.0030170.0034220.002510.0057920.0055450.004092

0.0057080.0030170.0034220.002510.0057920.0055450.004092

0.005640.0030170.0034220.002510.0057280.0054820.004089

0.0001052e-050.000116

1.5e-05

6.3e-056.9e-056.5e-051.2e-05

7.8e-05

7.7e-05

6.3e-05

0.0001263.8e-057e-050

6.5e-053e-056.7e-05

0.0004840.000387

6e-05

1.8e-05

6.3e-052.2e-056.9e-05

3e-05

7e-06

0.0001162.1e-050.000123

0.0001042.1e-052.1e-050.000108

2.4e-05

3.7e-05

0.00021

5.6e-057.2e-052.9e-050.000122

0.0001143.5e-056.7e-050.000128

0.0044250.0027130.0034220.002510.0035490.0042410.002018

2.3e-05

5.2e-05

2e-06

2.5e-05

9e-067e-061e-05

5.1e-05

0.001388

2.3e-05

1.5e-05

5.8e-05

6e-061e-056e-06

0.0001164e-053.7e-050.00013

4.2e-05

4.6e-051.5e-053.6e-055e-051.2e-05

5.1e-05

3.9e-05

2.7e-05

4.5e-05

3.9e-05

5e-06

8.9e-05

2.2e-05

6.3e-05

3.3e-05

1.8e-05

6.5e-05

5e-05

3.4e-05

0.0001466.3e-051.2e-050.000161

8e-054.6e-058.6e-05

6.8e-056.4e-056.3e-053e-06

6.8e-056.4e-056.3e-053e-06

0.0001860.0001880.000187.5e-05

0.0001860.0001880.000187.5e-05

6.1e-056e-055.9e-057.5e-05

6.1e-056e-055.9e-057.5e-05

6.1e-055.9e-055.9e-05

6.1e-055.9e-055.9e-05

6.4e-056.9e-056.2e-05

6.4e-056.9e-056.2e-05

0.0036290.0017190.0024980.0021080.003420.0034910.001607

0.000286.1e-050.0005760.000283

0.000286.1e-050.0005760.000283

0.000286.1e-050.0005760.000283

0.0032430.0016580.0024980.0021080.0027320.0031040.001592

0.0031930.0016580.0024980.0021080.002660.0030620.001577

5.3e-056.8e-055e-05

0.003140.0016580.0024980.0021080.0025920.0030120.001577

5e-057.2e-054.2e-051.5e-05

5e-057.2e-054.2e-051.5e-05

0.0001060.0001120.0001041.5e-05

0.0001060.0001120.0001041.5e-05

0.0001060.0001120.0001041.5e-05

0.0289740.0417630.0236360.0310240.017650.0352560.040859

5.2e-056.8e-054.7e-05

5.2e-056.8e-054.7e-05

5.2e-056.8e-054.7e-05

0.0001190.0001370.0001111.5e-05

0.0001190.0001370.0001111.5e-05

0.0001190.0001370.0001111.5e-05

0.0004082.9e-050.0020080.001410.0004055.1e-05

0.0004082.9e-050.0020080.001410.0004055.1e-05

0.0001342.9e-050.0020085.8e-050.000134

0.0001330.0002060.0001331.5e-05

0.00089

0.0001410.0001920.000138

6.4e-05

3.6e-05

0.021470.029880.0110580.0172690.0054370.0278180.020811

0.0214290.029880.0110580.0172690.0052990.0277830.020811

3.3e-05

00.000101006e-06

0009.5e-05

4.2e-05

2e-05

4e-06

1.1e-050.0001016e-061.2e-056e-06

0.0036040.002180.003190.0023090.0029920.003452

4.4e-05

4e-066e-065e-06

5e-069e-066e-06

4.6e-056e-064.4e-051.5e-05

3e-064e-062e-06

3e-063.9e-051e-061e-06

6.2e-055.1e-056.7e-05

6.8e-050

0

6.4e-052.7e-056.7e-053e-05

4.2e-052.1e-054.3e-051e-06

0.0149190.023330.0056450.0116470.0011020.0183850.017093

0.00010606e-06

3e-062e-063e-06

2.6e-05

3.1e-05

4e-06

5.1e-053.1e-055.5e-05

1.1e-05

5e-066e-064e-06

5.7e-05

0.0024620.0040230.0022230.0033130.0005990.0055580.003544

0

4e-053.4e-054e-05

3.4e-05

2.8e-051.7e-053.1e-051.5e-05

3.2e-05

7e-061e-066e-06

2e-062e-062e-06

00

4.4e-05

4.1e-050.0001383.5e-05

4.1e-050.0001383.5e-05

3.4e-05

3.4e-05

3.4e-05

0.0042240.0100230.0117130.0095380.0087610.0042090.018483

0.0042240.0100230.0117130.0095380.0087610.0042090.018483

9.1e-05

5.6e-05

9e-061e-051.2e-05

0.0001790.0001710.000189

0.0001217.4e-050.000117

0.000132

0.000149

0.000354

1.4e-05

0.001694

0.000149

0.000283

0.000198

0.000765

0.0001076.3e-055.8e-050.0001072.6e-05

6.8e-059.3e-057.5e-05

0.000366

6.3e-056.2e-056.5e-05

0.000230.0001460.000234

0.0001530.0003770.00010.000161

0.000132

0.000261

7.4e-057.3e-057.4e-05

0.000350.0002110.0002150.000341

6.1e-05

1.5e-05

3.2e-05

8e-05

0.0015360.0086920.0015760.0070280.0003750.0014690.010384

6.5e-054.6e-056.1e-05

0.000109

0.0091390.007988

3.2e-057.5e-050.002512.1e-053.2e-05

4.4e-05

0.000126

8.8e-05

0.0011480.0005790.0009980.0008780.00118

0.000911

7.1e-05

0.000151

7.7e-055.2e-057.9e-05

0.000138

1.2e-052.6e-052e-061.3e-05

0.0027010.0018310.0008650.0022090.0018030.0026660.001499

0.0004543.9e-050.0005120.0004569.9e-05

0.0001330.0001190.000131

0.000103

0.0001637.1e-050.0001649.9e-05

0.0001583.9e-050.0002190.000161

0.0022470.0017920.0008650.0022090.0012910.002210.0014

0.0003460.0001210.0003390.0003576.9e-05

0.0001115.2e-052.8e-050.0001157.9e-05

6.3e-05

0.000174

0.0001555.9e-056e-050.000156

2.6e-05

2e-05

0.0002669.3e-052.8e-050.000267

3.7e-05

0.000258

2.7e-05

9e-06

0.0013690.0014670.0008650.0022090.0002220.0013150.001252

5.9e-059.4e-053.8e-05

5.9e-059.4e-053.8e-05

5.9e-059.4e-053.8e-05

2.5e-054.4e-051.8e-05

2.5e-054.4e-051.8e-05

3.4e-055e-052e-05

3.4e-055e-052e-05

0.0002070.000620.000169

0.0001080.0003078.7e-05

0.0001080.0003078.7e-05

3e-054.1e-052.7e-05

3e-054.1e-052.7e-05

8e-063.5e-053e-06

8e-063.5e-053e-06

1.6e-057.2e-051.6e-05

1.6e-057.2e-051.6e-05

1.7e-051.2e-05

1.7e-051.2e-05

5.5e-05

5.5e-05

3.7e-050.0001042.9e-05

2.2e-054.7e-051.8e-05

1.5e-055.7e-051.1e-05

9.9e-050.0003138.2e-05

8.2e-050.0001796.9e-05

3.2e-050.000112.9e-05

3.2e-055.6e-052.9e-05

5.4e-05

5e-056.9e-054e-05

5e-056.9e-054e-05

1.7e-050.0001341.3e-05

1.7e-058.4e-051.3e-05

1.7e-058.4e-051.3e-05

5e-05

5e-05

0.1663210.2231910.1919120.2370440.13260.1511980.262611

0.0002530.0079720.0019940.000250.006967

0.0002530.0079720.0019940.000250.006967

0.0002530.0079720.0019940.000250.006967

0.00015

0.00015

8.3e-05

8.3e-05

0.0002430.000242

0.0002430.000242

1e-057.5e-058e-06

6e-05

1e-051.5e-058e-06

0.0079720.0016860.006967

0.001686

0.0079720.006967

6.9e-05

6.9e-05

6.9e-05

6.9e-05

6.9e-05

0.0056970.0033720.0252490.0044170.0131770.005440.015239

0.0053610.0033720.0049110.0044170.0050180.0051280.00272

0.0053610.0033720.0049110.0044170.0050180.0051280.00272

0.0053610.0033720.0049110.0044170.0050180.0051280.00272

0.002760.0017950.0023870.0021080.0025970.0026410.001392

0.0026010.0015770.0025240.0023090.0024210.002487

0.001328

0.0002870.0203380.0078610.0002690.012519

0.0002870.0203380.0068260.0002690.012519

0.0002870.0203380.0068260.0002690.012519

0.000605

0.000577

0.0002180.0002480.000194

6.9e-050.000147.5e-05

0.0143240.012519

0.0060140.005256

0.001035

0.001035

0.000929

0.000106

4.9e-050.0002984.3e-05

4.9e-050.0002984.3e-05

2.8e-050.0001162.3e-05

2.8e-050.0001162.3e-05

0.000102

0.000102

2.1e-058e-052e-05

2.1e-058e-052e-05

0.0283150.0351860.0353780.0321290.0322560.0239910.051827

0.0208820.0295780.0296420.0269080.0272640.0168450.047929

0.0056820.0115670.011160.0060240.0037360.0024610.015715

8.7e-050.0001758.5e-05

8.7e-050.0001758.5e-05

1.5e-05

1.5e-05

9.2e-05

9.2e-05

0.0055730.0115670.0055060.0060240.0033610.0023560.010759

0.0055730.0115670.0055060.0060240.0033610.0023560.010759

0.0056540.004941

0.0056540.004941

2.2e-050.0001082e-05

2.2e-050.0001082e-05

4.3e-055.1e-053e-05

4.3e-055.1e-053e-05

4.3e-055.1e-053e-05

0.0108880.0140960.0100730.0091370.0062880.0104270.017868

5.3e-050.0001345.3e-05

5.3e-050.0001345.3e-05

0.0106420.0127860.0100730.0091370.0053540.0101820.010392

5e-065.8e-056e-06

1.7e-052.5e-051.8e-05

5e-062.6e-056e-06

0.0106150.0127860.0100730.0091370.004880.0101520.010392

0.000122

0.000243

6.7e-05

6.7e-05

9.9e-050.0001649.8e-05

9.9e-050.0001649.8e-05

0.0011370.007409

0.0011370.007409

9.4e-050.0001730.0006369.4e-05

6.5e-056.5e-05

0.000173

0.000473

2.9e-050.0001632.9e-05

1.2e-050.0001031e-05

9e-065e-059e-06

9e-065e-059e-06

3e-065.3e-051e-06

3e-065.3e-051e-06

0.0001960.0006450.0002019.4e-05

0.0001850.0005810.0001879.4e-05

5.7e-058.7e-055.5e-05

0.0001280.0004940.000132

9.4e-05

1.1e-056.4e-051.4e-05

1.1e-056.4e-051.4e-05

2.3e-050.0004460.0028232.2e-050.000153

2.3e-057.5e-052.2e-05

2.3e-057.5e-052.2e-05

0.002312

0.002312

0.000436

0.000436

0.000153

0.000153

0.000446

0.000446

0.000177

0.000177

0.000177

0.0011960.0014930.0061390.0059240.0037760.0009640.010951

0.000559

0.000559

5.3e-050.000586

5.3e-05

0.000586

0.0002022.3e-050.0061390.0005026.6e-050.008991

4e-06

0.000554

4e-06

0.0061390.005365

0.0002022.3e-050.0005026.6e-05

0.000342

4e-06

0.002718

2.3e-059.4e-052.3e-05

2.3e-059.4e-052.3e-05

0.0002160.0004220.001140.0001320.000219

2.6e-050.0002612.7e-05

0.000385

0.000190.0004220.0004940.000105

0.000219

1.8e-050.000232.4e-050.000376

0.000376

1.8e-050.000232.4e-05

0.0004370.0005750.00022

0.000575

0.000251

0.0001864e-06

0.000216

0.000169

0.000169

0.0007370.0005580.0059240.0010660.000719

0.000173

0.0007370.0003850.0059240.0010660.000719

3.9e-055.6e-052.9e-05

3.9e-055.6e-052.9e-05

3.9e-055.6e-052.9e-05

0.0001270.0005780.000105

1.1e-054.9e-051e-05

1.1e-054.9e-051e-05

1.4e-050.0001281.6e-05

5e-05

7e-062.5e-057e-06

4e-063.5e-055e-06

3e-061.8e-054e-06

5.1e-050.0001794.1e-05

1e-05

1.4e-054.2e-051.8e-05

3e-062.9e-054e-06

4e-063.1e-052e-06

1.4e-053e-051.3e-05

6e-064.7e-054e-06

1.5e-059.4e-051.6e-05

6e-063.3e-055e-06

2e-062.9e-053e-06

7e-063.2e-058e-06

01.9e-050

1.6e-05

03e-060

000

1e-054.3e-059e-06

1e-054.3e-059e-06

1.5e-056.6e-051.3e-05

1.5e-056.6e-051.3e-05

1.1e-05

1.1e-05

3.9e-059.4e-052.9e-05

3.9e-059.4e-052.9e-05

3.9e-059.4e-052.9e-05

5.1e-050.0001984.9e-05

5.1e-050.0001984.9e-05

5.1e-050.0001984.9e-05

0.0025860.0019760.002270.0058230.0087390.0025180.003148

0.0025550.0019760.002270.0058230.0085460.0024850.003148

1e-058e-061.5e-05

0.00058

3.9e-053.7e-053.9e-05

2e-051e-062e-05

3.1e-052.7e-053.5e-05

2.1e-05

1.7e-054.8e-051.9e-05

2.9e-05

4e-052e-054.2e-05

8e-06

1.6e-05

4.9e-053.9e-050.0021088e-064.9e-051.6e-05

6.8e-05

2.5e-05

0.004165

2.2e-051.8e-051.5e-05

1.5e-053e-051.7e-05

2.2e-052.9e-052.5e-05

9e-063.6e-059e-06

0.0017080.0010180.0015110.0019080.0018890.0016410.000762

3.2e-052.6e-053.4e-05

0.0005210.0009190.0007590.0018070.0004890.0004990.000701

0.0014380.001073

6.4e-05

5.4e-05

2e-058e-062.6e-05

1.8e-052.9e-051.8e-05

0

1.1e-052.7e-051.1e-05

7e-062e-067e-06

4.9e-05

4.9e-05

1.3e-050.0001151.5e-05

6e-065.4e-057e-06

7e-066.1e-058e-06

0.0074020.0056080.0057360.0052210.0047360.0071030.003898

1.5e-059e-057e-06

5e-064.2e-054e-06

5e-064.2e-054e-06

1e-054.8e-053e-06

1e-054.8e-053e-06

6e-065.6e-055e-06

6e-065.6e-055e-06

6e-065.6e-055e-06

0.0026570.0018120.0022050.002410.0023930.0025330.001179

3e-062.6e-053e-06

3e-062.6e-053e-06

1.1e-054.3e-056e-06

1.1e-054.3e-056e-06

8e-064.9e-057e-06

8e-064.9e-057e-06

1.3e-055.3e-059e-06

1.3e-055.3e-059e-06

0.0025220.0018120.0022050.002410.0020610.0024010.001179

0.0025220.0018120.0022050.002410.0020610.0024010.001179

7e-061.4e-055e-06

7e-061.4e-055e-06

9.3e-050.0001470.000102

5.1e-055.1e-055.3e-05

2.5e-051.9e-052.7e-05

1.7e-051.8e-052.2e-05

5.9e-05

0.0047240.0037960.0035310.0028110.0021970.0045580.002719

0.0047040.0037960.0035310.0028110.0020960.0045350.002719

9e-062.7e-057e-06

4e-061.4e-054e-06

3e-061.6e-053e-06

6e-063e-056e-06

0.0046550.0037960.0035310.0028110.0019230.0044910.002719

6e-061.8e-056e-06

1.1e-051.8e-051.1e-05

1e-055e-057e-06

1.1e-054.5e-051.7e-05

7e-062.9e-051.3e-05

4e-061.6e-054e-06

9e-065.6e-056e-06

9e-065.6e-056e-06

6e-063.6e-055e-06

6e-063.6e-055e-06

6e-063.6e-055e-06

6e-063.6e-055e-06

2.5e-050.000223.8e-05

1.2e-058.9e-051.4e-05

9e-064.7e-051.2e-05

9e-064.7e-051.2e-05

3e-064.2e-052e-06

3e-064.2e-052e-06

1.3e-050.0001312.4e-05

4e-065.6e-053e-06

4e-065.6e-053e-06

9e-067.5e-052.1e-05

4e-064.2e-059e-06

5e-063.3e-051.2e-05

0.0036030.0018170.0085290.0053210.0043420.003460.006865

0.0036030.0018170.0085290.0053210.0043420.003460.006865

0.0036030.0018170.0085290.0053210.0043420.003460.006865

0.0005530.0002470.006040.0030120.0012480.0005360.005375

0.0005530.0002470.0030120.0012480.000536

9.6e-05

0.006040.005279

0.000105

0.000105

0.000111

0.000111

0.003050.001570.0024890.0023090.0028780.0029240.00149

0.003050.001570.0024890.0023090.0028780.0029240.00149

0.1284530.1828160.1147840.1951770.08076199999999990.1180570.181713

0.1022540.153740.1004950.1553210.0553310.09850100000000010.133219

0.0013550.0011250.0012884.8e-05

1.6e-05

1.6e-05

0.0013550.0011250.0012883.2e-05

1.6e-05

2.9e-055.1e-052.9e-05

3.1e-053.5e-051.9e-051.6e-05

0.0012950.0009840.00124

5.5e-05

0.0052330.0030390.004090.0082330.0052330.0050180.002557

2.8e-055.7e-052.3e-05

2.8e-055.7e-052.3e-05

0.0030990.001710.0023140.0062250.0032020.0029830.001665

4.3e-053.4e-055e-05

5.4e-050.0023094e-055.9e-05

0.0029210.001710.0023140.0022090.0023520.002810.001242

4.5e-053.3e-053.1e-05

1.3e-050.0017071.3e-051.2e-05

0.0006950.000423

2.3e-053.5e-052.1e-05

0.0021060.0013290.0017760.0020080.0019740.0020120.000892

1.4e-05

0.0021060.0013290.0017760.0020080.0019160.0020120.000892

4.4e-05

0.0361610.0878970.0536650.07460.0199160.0397670.080831

9.5e-050.0037910.0033130.0001068.7e-05

6.7e-050.0037910.0033134e-056e-05

2.8e-054.2e-052.7e-05

2.4e-05

0.0360660.0878970.0498740.0712870.019810.039680.080831

8.5e-05

0.0014290.0005490.0006530.004920.0001480.0014290.003213

7.2e-055e-050.0039060.0034144.2e-056.7e-05

0.0002920.0072890.0005460.0034140.0002070.000280.003217

3.6e-050.0020083.2e-053.9e-05

2.7e-050.002415.8e-052.6e-05

3.5e-053.4e-053.4e-05

2.5e-05

8.3e-05

7e-050.0001297.5e-051.7e-05

2.6e-05

6.4e-05

3.2e-050.0001512.5e-05

6.3e-055.2e-050.0023092.3e-056.3e-05

3.5e-05

2.4e-05

9.8e-05

0.0001078.2e-050.000103

0.000262

0.000146

5.7e-05

5.9e-05

0.000149

4.9e-05

0.0019940.0041470.0033620.0032130.0017110.0069870.003839

2e-053.6e-050.0053990.0047195e-061.8e-05

0.001303

3.1e-053.6e-053.2e-05

7.4e-05

0.0005080.000680.0006450.0017070.0002730.0004920.001086

5.8e-058.9e-056.1e-054.7e-05

5e-064.8e-055e-06

0.002866

0.0221780.0561970.0283590.0310240.0124170.021210.051875

7.7e-058.2e-05

0.0038780.0090270.0029480.0059240.0007220.003720.007253

0.0051540.009870.0040560.0062250.0011130.0049320.007369

8.1e-050.00036.6e-05

8.1e-050.00036.6e-05

6.2e-05

2.3e-05

8e-06

8.1e-054e-056.6e-05

6.1e-05

0.000106

0.052960.0584420.0381410.0642550.0256850.0461730.046245

0.0529440.0584420.0381410.0624480.0255760.046140.046245

0.0001120.000110.0021085.9e-050.0003240.000471

1e-058e-060.0022092e-061e-051.4e-05

5.3e-059e-065.3e-05

0.0002860.0001770.0002810.000296

1.5e-050.0022093.5e-054e-05

1.5e-05

0.0013610.0011230.00130.0022090.0004950.0013220.002344

0.000147

1.8e-05

3.4e-05

0.000118

5e-05

4.6e-05

0.0010430.0003320.0003920.002610.0002890.0009920.000115

8.3e-05

0.000349

5.7e-05

0.0019610.002468

8e-063.2e-050.0031123e-068e-064e-05

0.0001184.2e-050.0001210.00027

0.0013360.0022430.0014760.002510.0006640.001285

0.000311

0.0002350.0009870.000370.0023090.0001350.0001990.002248

1.7e-05

0.0082630.0077660.0061150.004920.0028040.007941

2.2e-05

4.7e-051.5e-054.6e-05

0.0001835.1e-050.0001853.4e-05

0.0109690.0056860.0064660.0038150.0069270.0106060.003402

0.000757

8.2e-057.9e-058.4e-05

0.000537

0.000186

5.7e-052.3e-055.5e-05

0.0010390.0039410.0009570.0033130.0002440.0007860.003532

0.0007510.0001840.0035140.000390.0007197.8e-05

0.000929

7.5e-053e-057.2e-05

0.0230130.0270160.0158150.0126510.0060130.0148770.024361

0.0016520.003630.002160.0031120.0010030.0015820.00288

4.2e-057e-064.5e-05

6.7e-05

0.00170.0034010.003090.0028110.0023310.0039460.003322

9.3e-058.5e-059.7e-05

8.8e-057.5e-058.3e-05

4.6e-052.2e-050.0027116.4e-050.0001271e-06

0.0002670.0063254.8e-050.0002548.1e-05

2.2e-05

1.6e-050.0018070.0001093.3e-05

1e-052.3e-059e-06

6e-060.0018072.5e-052.4e-05

6.1e-05

0.0064640.0043620.0045990.0082330.0030720.0061890.003538

0.0012470.001460.001440.0021080.0010290.0011920.001003

0.0012470.001460.001440.0021080.0010290.0011920.001003

2e-053.5e-051.9e-05

2e-053.5e-051.9e-05

0.0051970.0029020.0031590.0061250.0020080.0049780.002535

2.8e-051.5e-052.8e-05

4.1e-054.9e-054e-05

1.6e-052e-051.4e-051.2e-05

0.0050870.0028520.0031590.0029120.0014650.0048740.002535

2.5e-053e-050.0032131.7e-052.4e-05

0.000437

1.1e-05

0.0261990.0290760.0142890.0398560.0254310.0195560.048494

3e-055.7e-052.5e-05

3e-055.7e-052.5e-05

3e-055.7e-052.5e-05

0.0054170.0030120.0043490.0048190.0075590.0052130.001076

0.000112

0.000112

2.3e-050.0021442.1e-050.000948

0.0020650.000948

2.3e-057.9e-052.1e-05

1.8e-055.6e-051.7e-05

1.8e-055.6e-051.7e-05

0.0053760.0030120.0043490.0048190.0052470.0051750.000128

6e-06

9e-06

5.4e-05

6.9e-05

9.7e-05

7.2e-05

6.3e-05

2.3e-05

2.3e-05

6e-062.6e-055e-06

8.5e-05

3.6e-05

0.0002527.4e-050.0016060.0001690.0003082.3e-05

0.00019.7e-052.6e-055.2e-050.000105

1.7e-05

1.4e-05

2.4e-05

1.8e-057.6e-051.8e-05

0.0049710.0028410.0043490.0032130.004150.004764

1.3e-05

9e-06

8e-06

1.6e-05

2.9e-056.8e-052.8e-05

1.2e-05

2.1e-05

5.1e-05

1e-05

4.1e-050.0013154.5e-050.000252

0.0003380.000252

0.000338

0.000252

0.000283

0.000283

0.000152

0.000152

0.00015

0.00015

4.1e-057e-054.5e-05

4.1e-057e-054.5e-05

0.000322

2.6e-05

4.9e-05

4.6e-05

5.4e-05

2.7e-05

2.1e-05

2.8e-05

2.4e-05

4.7e-05

0.0002360.0010380.000241

0.0002360.000960.000241

0.0001820.0009060.000188

5e-06

1.6e-052e-051.6e-05

1.7e-051.9e-051.7e-05

2.1e-051e-052e-05

7.8e-05

7.8e-05

0.0178650.0241160.0072820.028010.0103540.0096240.043118

0.0013970.000460.0006660.0019080.0021960.0014250.000174

0.0013970.000460.0006660.0019080.0021960.0014250.000174

6.8e-050.0001987e-05

0

6.8e-054.6e-057e-05

5.4e-05

9.8e-05

9.7e-05

9.7e-05

0.0008250.0005790.0009410.0016060.0007990.00079

0.0008250.0005790.0009410.0016060.0007990.00079

0.0129640.0213460.0034520.0224880.004690.0048250.042849

6.7e-05

3.2e-051.6e-054.9e-05

1.9e-05

3.2e-05

3.5e-055e-054.8e-05

0.000183

0

6e-050.0013350.0023099e-066.2e-050.001827

2e-06

2.4e-05

3.6e-052.7e-054.3e-05

0.000277

0.000297

7.8e-05

1e-053e-061.8e-05

0.0001717.3e-050.0022091.9e-050.0001690.02729

0.000223

5.9e-050.0021081.1e-056.1e-05

0.0001414.5e-050.0002840.00015

4.6e-050.0002195.5e-05

4e-06

2.2e-058.3e-052.5e-05

0.0006480.0001910.0028119.1e-050.000643

0.0001046e-060.000105

0.0014110.0063560.0006350.002610.0001120.0018870.007038

0.0001962.9e-051.9e-050.00021

1.3e-05

2.1e-054.2e-052.6e-05

2e-06

0.000231

8e-06

0.000263

0.000536

0.000370.0036830.0009490.002513e-050.0005480.001203

0.000297

7.7e-054.8e-058.6e-05

0.0003860.0054880.0046181.7e-050.0003740.000534

5.4e-05

0.0089380.0041460.0018680.0033130.0004530.004957

4.3e-050.0001074.4e-05

0.000323

2.3e-050.000113.3e-05

0.0001351e-060.000189

0.000123

0.000123

0.000156

8.7e-05

6.9e-05

0.000154

0.000115

8e-06

3.1e-05

0.0025910.0017310.0022230.0020080.0016110.0024939.5e-05

4e-065e-066e-06

3e-06

2e-06

7e-06

08e-06

3.3e-052.9e-053.2e-058e-06

3e-064e-063e-06

1e-066e-063e-06

8e-06

0.0025080.0017310.0022230.0020080.0014650.002404

8e-062e-065e-06

8e-06

2.1e-05

3.3e-055.2e-053.9e-05

1e-064e-061e-066.3e-05

1.1e-05

1.2e-050.0001071.4e-05

1.2e-050.0001071.4e-05

8e-066e-067e-06

8e-066e-067e-06

0.000111

0.000111

0.000106

0.000106

0.0006570.0001280.002610.0008460.0006990.00226

4.3e-059e-053.5e-05

4.3e-059e-053.5e-05

0.0006140.0001280.002610.0006530.0006640.00226

6e-06

4.8e-052.5e-055.1e-05

1.3e-05

6.1e-052.3e-056.4e-05

6.2e-051.4e-056.5e-05

3.5e-054.2e-053.9e-05

0.0001

0.0002548.6e-050.0002940.00026

2e-06

5.2e-053.2e-055e-065.4e-05

2.8e-051.6e-052.9e-05

4.7e-05

3e-061e-050.002615e-062.5e-05

0.00226

1.2e-05

5.5e-058e-065.8e-05

7e-06

1.2e-05

1.6e-059e-061.9e-05

1.3e-05

0.000103

0.000103

4.7e-052e-060.0021083.5e-055.7e-05

4.7e-052e-060.0021083.5e-055.7e-05

4e-063e-068e-06

1e-062e-060.0021081e-061e-06

1.7e-059e-062e-05

5e-066e-066e-06

2e-051.6e-052.2e-05

0.0019060.0018180.0026580.0023090.0042270.0036520.001788

0.0019010.0018180.0026580.0023090.0041670.0036480.001788

0.0019010.0018180.0026580.0023090.0041670.0036480.001788

5e-066e-054e-06

5e-066e-054e-06

3.2e-050.0002883.3e-05

3.2e-050.0002883.3e-05

3.2e-050.0002883.3e-05

3.2e-050.0002883.3e-05

9e-064.5e-055e-06

6e-063.5e-053e-06

5e-06

3e-065e-062e-06

1.1e-055.6e-052e-05

1.1e-055.6e-052e-05

1.2e-050.0001878e-06

1.2e-056.3e-058e-06

5.3e-05

7.1e-05

0.0064480.0092880.0062070.0081320.0044510.0125440.008324

0.0064480.0092880.0062070.0081320.0044510.0125440.008324

0.0044070.0052090.0043090.005220.002860.0106380.004666

0.0044070.0052090.0043090.005220.002860.0106380.004666

1e-054.4e-058e-06

1e-054.4e-058e-06

0.0043970.0052090.0043090.005220.0028160.010630.004666

0.0007840.0013230.000750.0021080.0002280.0007640.001051

2.1e-05

0.001113

0.0036110.0038860.0035590.0031120.0014380.0098650.003615

2e-061.6e-051e-06

0.0003080.0001110.000140.0002820.000127

0.0003080.0001110.000140.0002820.000127

0.0003080.0001110.000140.0002820.000127

6e-061.4e-056e-06

0.0001245.7e-0500.0001

9.4e-057.6e-059.2e-050.000111

8.4e-055.4e-055e-058.4e-051.6e-05

0.0017330.0039680.0018980.0029120.0014510.0016240.003531

0.0015960.0039680.0018980.0029120.0008350.0014990.003531

7.1e-051.7e-053.5e-05

3.9e-059e-06

2e-061e-061e-06

5e-062e-065e-06

1.7e-051e-062.2e-05

3e-06

1e-06

8e-067e-06

0.0015250.0039680.0018980.0029120.0008180.0014640.003531

2e-063e-062e-06

1.2e-052e-061.2e-05

2.4e-05

8e-061.2e-058e-06

0.0014360.0039680.0018980.0029120.0007620.0013750.003507

3.3e-053.2e-053.2e-05

3.4e-057e-063.5e-05

0.0001370.0006160.000125

3.3e-050.0001252.8e-05

7e-065.8e-058e-06

2e-053.7e-051.4e-05

6e-063e-056e-06

1.2e-053.7e-058e-06

1.2e-053.7e-058e-06

4e-063.1e-054e-06

4e-063.1e-054e-06

1.2e-055.8e-051.1e-05

6e-062.9e-056e-06

6e-062.9e-055e-06

7.6e-050.0003657.4e-05

2.2e-05

4e-063e-053e-06

5e-066.1e-053e-06

5e-05

3e-063.3e-054e-06

3.5e-057.2e-053.6e-05

3e-063.3e-054e-06

2.1e-052.2e-052e-05

5e-064e-054e-06

0

2e-06

1.6e-055.4e-051e-05

1.6e-055.4e-051e-05

1.6e-055.4e-051e-05

1.6e-055.4e-051e-05

1.6e-055.4e-051e-05

1.6e-055.4e-051e-05

0.0097110.0248990.0194370.0317280.0065450.0094260.013345

0.0097110.0248990.0194370.0317280.0065450.0094260.013345

3e-066.8e-052e-06

3e-066.8e-052e-06

1e-062.9e-051e-06

1e-062.9e-051e-06

2e-063.9e-051e-06

1.8e-05

2e-062.1e-051e-06

0.009690.0248990.0194370.0317280.0063890.0094040.013345

0.009670.0248990.0194370.0317280.0063010.0093870.013345

0.0028360.0105680.0077910.0042170.0011060.002740.003932

0.0028190.005430.0026940.0042170.001020.002723

6e-067e-066e-06

3e-062.7e-052e-06

8e-060.0051380.0050975.2e-059e-060.003932

0.0068340.0143310.0116460.0275110.0051950.0066470.009413

2.1e-05

0.0014470.0012840.004929e-060.0014080.000212

0.0007710.0011390.0038151.1e-050.0007560.000379

7e-06

0.0008430.0010690.004922.3e-050.000830.000273

0.0003680.0001280.0079324e-060.0003621.6e-05

6e-06

0.0034050.0107110.0058560.0059244.2e-050.0032910.008533

0.005790.005061

1.1e-05

2e-058.8e-051.7e-05

7e-064.2e-054e-06

5e-062.5e-053e-06

2e-068e-061e-06

9e-06

1.3e-054.6e-051.3e-05

4e-062.5e-054e-06

9e-062.1e-059e-06

1.8e-058.8e-052e-05

1.8e-058.8e-052e-05

2.9e-05

2.9e-05

5e-063.3e-058e-06

5e-063.3e-058e-06

1.3e-052.6e-051.2e-05

1.3e-052.6e-051.2e-05

2.4e-050.0002121.6e-05

2.4e-050.0002121.6e-05

2.4e-050.0002121.6e-05

2.4e-050.0002121.6e-05

7e-064.8e-057e-06

7e-064.8e-057e-06

5.3e-05

5.3e-05

4e-063.8e-051e-06

4e-063.8e-051e-06

5e-063.4e-052e-06

5e-063.4e-052e-06

8e-063.9e-056e-06

8e-063.9e-056e-06

1.4e-050.0001081.3e-05

1.4e-050.0001081.3e-05

1.4e-050.0001081.3e-05

1.4e-050.0001081.3e-05

1.4e-050.0001081.3e-05

1.4e-050.0001081.3e-05

2e-050.0001899e-06

1.1e-057.2e-058e-06

1.1e-057.2e-058e-06

1.1e-057.2e-058e-06

1.1e-057.2e-058e-06

1.1e-057.2e-058e-06

9e-064e-051e-06

9e-064e-051e-06

9e-064e-051e-06

9e-064e-051e-06

9e-064e-051e-06

7.7e-05

7.7e-05

7.7e-05

7.7e-05

7.7e-05

0.4065040.4103760.4236170.3578270.4294879999999990.4212360.367031

0.1834460.1907040.1336360.0993960.1382570.1757910.087176

0.0006546.4e-050.0017930.0006760.001377

0.0006546.4e-050.0017930.0006760.001377

0.0001127.7e-050.000104

0.0001127.7e-050.000104

0.0002946.4e-050.0014130.0002910.001377

0.0002946.4e-050.000110.000291

0.001377

0.000109

0.000753

0.000441

8.3e-05

8.3e-05

4.4e-056.7e-053.8e-05

4.4e-056.7e-053.8e-05

0.0002040.0001530.000243

3.5e-054e-057.8e-05

3.3e-05

8.2e-053.9e-057.9e-05

8.7e-054.1e-058.6e-05

0.0001840.0003150.0001646e-05

0.0001840.0003150.0001646e-05

1.7e-050.0001191.4e-05

1.7e-050.0001191.4e-05

0.0001420.0001090.0001274.4e-05

0.0001420.0001090.0001274.4e-05

2.5e-058.7e-052.3e-05

2.5e-058.7e-052.3e-05

1.6e-05

1.6e-05

0.00023

0.000119

0.000119

0.000119

0.000111

0.000111

0.000111

2.6e-050.0001032e-05

2.6e-050.0001032e-05

2.6e-050.0001032e-05

2.6e-050.0001032e-05

0.0057980.0053670.005070.0041160.006550.0055420.004032

0.0004153.3e-050.0007510.000401

0.000139

0.000139

6.2e-055.9e-055.5e-05

6.2e-055.9e-055.5e-05

0.0001430.0001890.000136

0.0001430.0001890.000136

5.2e-053.5e-055.4e-05

2.1e-051.7e-052.2e-05

3.1e-051.8e-053.2e-05

6.7e-050.0001116.2e-05

5.2e-05

6.7e-055.9e-056.2e-05

8.9e-053.3e-059e-059.2e-05

8.9e-053.3e-059e-059.2e-05

2e-060.0001282e-06

0.000105

2e-063e-062e-06

2e-05

0.0053830.0053340.005070.0041160.0057990.0051410.004032

0.0001260.0001260.000127

0.0001260.0001260.000127

0.0047710.0053220.005070.0041160.0045530.0045640.003997

0.000180.000160.000173

0.0045910.0053220.005070.0041160.0043930.0043910.003997

0.000153

0.000153

0.000157

0.000157

0.000236

0.000236

0.0001960.0002090.0001741.6e-05

8.7e-050.0001147.7e-05

0.0001094.7e-059.7e-051.6e-05

4.8e-05

1.9e-058.7e-051.1e-05

1.9e-058.7e-051.1e-05

5.2e-050.0001174.4e-05

5.2e-050.0001174.4e-05

0.0002191.2e-050.0001610.0002211.9e-05

4.8e-05

1.6e-05

0.0001111.2e-053.6e-050.0001087e-06

0.0001086.1e-050.0001131.2e-05

1e-054.5e-056e-06

1e-054.5e-056e-06

1e-054.5e-056e-06

1e-054.5e-056e-06

5.6e-050.0003484.9e-05

5.6e-050.0003484.9e-05

3e-050.000172.6e-05

1.5e-058.7e-051.3e-05

1.5e-058.3e-051.3e-05

2.6e-050.0001782.3e-05

6e-069.5e-054e-06

2e-058.3e-051.9e-05

0.0885040.1200610.0690990.0369470.072630.0846370.044288

0.0883780.1200610.0690990.0369470.0723280.0845240.042974

0.00016

0.00016

0.0002050.0002140.0002121.5e-05

0.0001480.000130.000152

1.5e-05

5.7e-054.1e-056e-05

4.3e-05

0.000307

0.000184

0.000123

5.4e-05

5.4e-05

0.0869320.1196980.0686840.0353410.0691920.0831270.039967

0.0869070.1196980.0686840.0353410.0678440.0831010.039967

2.5e-050.0010032.6e-05

0.000345

0.0004980.0003630.0004150.0016060.0003860.0004890.002843

0.0001940.0001560.000195

0.002369

0.0003040.0003630.0004150.0016060.000230.0002940.000474

0.0001360.000140.0001273e-05

0.0001360.000140.0001273e-05

8.5e-05

8.5e-05

0.000177

0.000177

0.000203

0.000203

5e-054.4e-054.6e-05

5e-054.4e-054.6e-05

0.0002

0.0002

5.6e-057.1e-054.8e-05

2.7e-057e-061.5e-05

2.9e-056.4e-053.3e-05

0.000191

0.000191

6e-06

6e-06

2.3e-05

2.3e-05

0.0001168.7e-050.000112

0.0001168.7e-050.000112

5.9e-050.0001165.1e-05

5.9e-050.0001165.1e-05

0.0002590.0002170.00025

8.3e-059.4e-057.7e-05

0.0001760.0001230.000173

0.000128

0.000128

4.8e-050.0001254.7e-056.1e-05

1.5e-05

2e-058.3e-051.9e-051.6e-05

2.8e-054.2e-052.8e-053e-05

7e-06

7e-06

8e-05

8e-05

0.000151

0.000151

2.2e-05

2.2e-05

1.9e-051.5e-05

1.9e-051.5e-05

7e-06

7e-06

7e-06

0.0001260.0003020.0001130.001307

4.8e-056.6e-053.6e-05

4.8e-056.6e-053.6e-05

0.001307

0.001307

1.1e-058.3e-051.6e-05

1.1e-058.3e-051.6e-05

6.7e-050.0001536.1e-05

6.7e-050.0001536.1e-05

0.0049990.0078240.0049660.0102410.0042410.0047740.006795

0.0012210.0006780.0022090.0004960.0011640.000496

3e-061.6e-054e-06

3e-061.6e-054e-06

0.0012180.0006780.0022090.000480.001160.000496

2e-060.0002661e-063e-067.5e-05

2.7e-058e-062.9e-05

4e-061e-065e-062e-06

1.6e-057e-064e-06

1.3e-056e-061.4e-051e-06

0.000201

1.2e-052e-061.3e-052e-06

0.000137

0.0002036e-050.000227

1.4e-053e-061.5e-05

2.3e-050.0022091.1e-052.4e-05

1.2e-054e-061.3e-05

6.4e-051.4e-056.9e-05

4e-06

0.0001132.4e-050.000125.1e-05

5e-062e-068e-062e-06

1.6e-052.3e-058e-06

5e-061e-061e-06

2.2e-050.0001911.1e-052.3e-055e-06

0.0001723.7e-055.5e-053.7e-05

5.2e-052.2e-055.6e-05

0.0004430.0002210.0001060.0004730.000116

4.1e-057.5e-052.6e-05

4.1e-057.5e-052.6e-05

4.1e-057.5e-052.6e-05

0.0037370.0071460.0049660.0080320.0035920.0035840.006299

0.0022040.0056060.0036660.0042170.0030090.0021120.005753

0.0022010.0056060.0036660.0042170.0028910.0021090.005477

2e-060.000112e-060.000276

1e-068e-061e-06

0.0015180.001540.00130.0038150.0005090.0014560.000546

0.0014980.001540.00130.0018070.0014370.000546

2e-063.4e-052e-06

5e-064.8e-055e-06

7e-060.0020080.0003739e-06

0

2.9e-05

1e-062.4e-051e-06

5e-061e-062e-06

7e-064.6e-054e-06

6e-061.4e-054e-06

1e-063e-06

2.9e-05

8e-062.8e-051.2e-05

4e-068e-065e-06

3e-069e-067e-06

1e-067e-060

3e-06

01e-060

7.8e-05

7.8e-05

7.8e-05

0.0808230.0566040.0536390.0442770.048580.0775720.030014

7.5e-050.0001027.4e-05

7.5e-050.0001027.4e-05

7.5e-050.0001027.4e-05

0.0002370.0006140.000215

6.2e-057.5e-055.7e-05

6.2e-057.5e-055.7e-05

0.000136

6.9e-05

6.7e-05

5.6e-058.2e-055.3e-05

5.6e-058.2e-055.3e-05

0.0001190.0001710.000105

4.1e-055.1e-053.5e-05

4.9e-056.5e-054.1e-05

2.9e-055.5e-052.9e-05

2.7e-05

2.7e-05

3.3e-05

3.3e-05

9e-05

9e-05

0.0609870.0354690.0420020.0226910.0387030.0583960.014889

0.0001730.0001550.0001587.5e-05

1.5e-05

7.4e-055.9e-056.3e-051.5e-05

9.9e-059.6e-059.5e-054.5e-05

3e-05

3e-05

4.4e-05

4.4e-05

0.000331

0.000159

9e-05

8.2e-05

0.0020480.0016270.0026360.0035140.001780.0019530.006994

0.0020480.0016270.0026360.0035140.001780.0019530.006994

0.0587330.0338420.0393660.0191770.0364060.0562580.007701

0.0001342.8e-056.1e-050.0001415.3e-05

0.0001162.7e-058.8e-050.0001240.000104

0.000121

0.0001564.7e-055.4e-050.0001640.003761

0.0001775.4e-050.000110.000190.003783

0.000132.6e-056.5e-050.000134

0.058020.033660.0393660.0191770.0354410.055505

0.000364

0.000102

3.3e-053.1e-052.7e-054.5e-05

3.3e-053.1e-052.7e-054.5e-05

0.0007560.0088780.0016490.0089360.0002120.0007160.009427

6.5e-050.0001044e-05

6.5e-053.4e-054e-05

7e-05

0.0006910.0088780.0016490.0089360.0001080.0006760.009427

1.4e-052.4e-051.5e-050.000246

1e-06

0.0006490.0072050.0016490.0089367e-060.0006370.009181

3e-061e-053e-06

4e-060.0007595e-064e-06

4e-060.0009143e-064e-06

5e-065e-065e-06

4e-061e-064e-06

4e-05

8e-061.2e-054e-06

0.0004230.000370.0004032.9e-05

0.0001590.000120.0001521.5e-05

0.0001590.000120.0001521.5e-05

0.0001218.9e-050.0001181.4e-05

0.0001218.9e-050.0001181.4e-05

0.0001430.0001610.000133

0.0001430.0001610.000133

0.0012510.0006850.001020.0017070.0013570.0011830.000488

5.7e-058.9e-054.6e-05

5.7e-058.9e-054.6e-05

0.0011940.0006850.001020.0017070.001090.0011370.000488

0.0011940.0006850.001020.0017070.001090.0011370.000488

0.000178

0.000178

0.0014410.0021730.0013420.002610.0009650.0013780.001997

0.0014410.0021730.0013420.002610.0009650.0013780.001997

7.6e-056.5e-057.5e-05

8e-057.2e-057.3e-05

9.2e-05

6.3e-053.3e-056e-05

1.7e-05

0.000109

0.0001312.8e-054.7e-050.000128

0.0010910.0021450.0013420.002610.000530.0010420.001997

7.8e-05

7.8e-05

7.8e-05

8.3e-050.0001249e-05

8.3e-050.0001249e-05

2e-066e-062e-06

4e-062e-061.1e-05

5e-061.3e-059e-06

6e-066e-066e-06

3.2e-053.1e-053e-05

3e-05

1.1e-056e-061e-05

1e-052.4e-055e-06

1.3e-056e-061.7e-05

0.0002164.4e-05

0.000113

0.000113

1.4e-05

1.4e-05

3e-05

3e-05

0.000103

5e-05

5.3e-05

6.1e-057.6e-055.8e-053.5e-05

1.5e-05

1.5e-05

6.1e-057.6e-055.8e-052e-05

6.1e-057.6e-055.8e-05

2e-05

0.0035220.0022440.0008780.0015060.0027420.0035830.000642

0.00022

0.00022

0.000117e-06

0.00011

7e-06

8.7e-050.0002657.7e-054.4e-05

8.7e-050.0002657.7e-054.4e-05

0.0034350.0022440.0008780.0015060.0021470.0035060.000591

0.0008980.0005580.0002230.0009220.000179

0.001013

0.0007940.0005130.0002220.0008060.000208

0.0010270.0006470.0008780.0015060.0004230.001036

0.0007160.0005260.0002660.0007420.000204

0.0119870.0071550.0067480.0068270.0030210.0114760.002463

0.0115720.0071230.0067480.0068270.0023360.0110940.001634

7e-054.2e-056.1e-05

0.0001142.3e-054.5e-050.0001350.00012

0.0101340.0059710.0051470.0043170.0019580.009693

7.7e-05

5e-05

2.2e-05

0.0012540.0011290.0016010.002512.8e-050.0012050.001514

3e-05

0

4e-06

0

8e-05

6.5e-05

6.5e-05

7.4e-05

7.4e-05

0.0002210.000220.0002110.000751

4.8e-055.2e-054.8e-050.000215

5.2e-055.1e-054.4e-05

6.6e-05

4.4e-051.5e-054.8e-050.000148

7.7e-053.6e-057.1e-050.000166

0.000222

4.5e-050.0001193.9e-05

1.4e-052.7e-051.2e-05

3.7e-05

4e-069e-062e-06

1.9e-052.5e-051.8e-05

8e-062.1e-057e-06

0.0001493.2e-050.0001640.0001327.8e-05

1.4e-051.1e-051.4e-051.2e-05

4.6e-05

8.5e-052.1e-056.8e-057.7e-057.8e-05

5e-053.6e-054.3e-05

4.3e-05

4.3e-05

0.0023920.0007840.0008620.0038150.0034220.0023510.00061

0.0011870.0006570.0008620.0017070.000790.0011370.000472

4.4e-05

4.4e-05

0.0011870.0006570.0008620.0017070.0005220.0011370.000472

2.5e-05

0.0011870.0006570.0008620.0017070.0004970.0011370.000472

0.000174

3.7e-05

1.5e-05

2.5e-05

3.9e-05

3e-05

2.8e-05

5e-05

2.8e-05

2.2e-05

0.0012050.0001270.0021080.0026320.0012140.000138

0.0004970.0001270.0021080.0017070.0005034.6e-05

0.0004970.0001270.0021087.3e-050.0005034.6e-05

6.7e-05

0.001476

3.7e-05

3.5e-05

1.9e-05

0.0002520.0002380.000247

5.2e-05

9e-051.1e-058.9e-05

2.3e-05

1.8e-05

8.5e-054.5e-058.2e-05

3.3e-05

2.3e-05

7.7e-051.8e-057.6e-05

1.5e-05

0.0002950.0003840.0002836.2e-05

0.0001436.5e-050.00014

5.3e-05

7.1e-05

5e-05

9e-05

2.1e-05

0.0001525.5e-050.0001434.1e-05

4.4e-05

4.4e-05

4.5e-05

4.5e-05

0.0001440.000140.0001313e-05

7.8e-053.8e-057e-05

6.6e-051.6e-056.1e-05

1.5e-05

3e-05

7.1e-05

3.5e-05

3.5e-05

1.7e-053.9e-055e-05

1.7e-053.9e-055e-05

0.0043670.0024120.0032460.0066260.0041260.0041170.001926

0.0043470.0024120.0032460.0066260.0040390.0041010.001926

1.5e-053.1e-051.2e-05

1.5e-053.1e-051.2e-05

1.5e-053.1e-051.2e-05

0.0042130.0024120.0032460.002510.0036830.0040170.001926

2e-054.3e-051.7e-05

1.5e-052e-051.1e-05

5e-062.3e-056e-06

0.0041780.0024120.0032460.002510.0035920.0039920.001926

000

7e-061e-066e-06

3e-05

1e-062.6e-051e-06

4e-061.9e-052e-06

0.0041550.0024120.0032460.002510.0034490.0039710.001926

2e-061.2e-052e-06

2.4e-05

8e-068e-06

2.3e-05

1e-068e-062e-06

3e-062.8e-053e-06

3e-062.8e-053e-06

1.2e-052e-055e-06

1.2e-052e-055e-06

0.0001190.0041160.0003257.2e-05

6e-065.2e-055e-06

1e-068e-061e-06

5e-06

5e-061.3e-054e-06

5e-06

1.1e-05

1e-05

5.9e-056e-052.5e-05

4.1e-052.9e-057e-06

7e-061e-057e-06

1.1e-052.1e-051.1e-05

5.4e-050.0041160.0002134.2e-05

1.2e-05

2e-061.6e-05

1e-05

4e-06

5e-060.0016062e-065e-06

8e-06

2.2e-05

8e-061.7e-05

1e-060.002511e-061e-06

2e-064e-061e-06

1.6e-05

9e-063e-057e-06

4e-06

1.2e-05

2.7e-053e-062.8e-05

5.2e-05

6e-064e-055e-06

6e-064e-055e-06

6e-064e-055e-06

6e-064e-055e-06

1.4e-054.7e-051.1e-05

1.4e-054.7e-051.1e-05

5e-062.6e-056e-06

5e-062.6e-056e-06

9e-062.1e-055e-06

9e-061.3e-055e-06

8e-06

6.6e-058.9e-056e-05

6.6e-058.9e-056e-05

6.6e-058.9e-056e-05

6.6e-058.9e-056e-05

1.4e-054e-051.6e-05

3.9e-052.7e-053.4e-05

1.3e-052.2e-051e-05

0.0755750.0523030.1064960.0611430.09161499999999990.0723490.093229

0.0022030.0013930.0018160.0019080.0019930.0020940.001029

0.0022030.0013930.0018160.0019080.0019930.0020940.001029

9e-050.0001197.8e-05

9e-061.8e-056e-06

3.3e-051.7e-052.8e-05

1.2e-051.4e-059e-06

2.1e-054.6e-051.9e-05

1.5e-052.4e-051.6e-05

0.0020090.0013930.0018160.0019080.0018290.0019160.001029

0.0020090.0013930.0018160.0019080.0018290.0019160.001029

4.1e-051.7e-053.8e-05

4.1e-051.7e-053.8e-05

6.3e-052.8e-056.2e-05

6.3e-052.8e-056.2e-05

0.0080350.0097610.0093910.0163650.0103870.007710.030609

0.0006250.000290.0004621.6e-05

0.0001938.5e-050.000153

0.0001938.5e-050.000153

0.0001699.1e-050.0001091.6e-05

0.0001699.1e-050.0001091.6e-05

0.0002630.0001140.0002

0.0002630.0001140.0002

0.007410.0097610.0093910.0163650.0100970.0072480.030593

0.007410.0097610.0093910.0163650.008620.0072480.027706

0.001536

0.0063990.005593

0.002229

0.000179

0.0044070.001047

0.004030.0016570.0014220.009940.0016590.0039980.000331

0.0017730.0011030.0007120.0023090.0001690.0011770.00104

0.00093

0.002314

0.0016070.0070010.0008580.0041160.0001560.0020730.009647

6.1e-05

0.002258

2.5e-05

0.002745

0.001848

0.001848

0.000286

0.000286

0.00035

0.00035

0.0011910.000689

0.000689

0.001191

0.0237740.0133840.016090.0097390.0252670.0226380.010172

0.0002770.0002850.0001991.5e-05

0.0001020.000127e-05

0.0001020.000127e-05

0.0001229.3e-059.1e-05

0.0001229.3e-059.1e-05

5.3e-057.2e-053.8e-051.5e-05

5.3e-057.2e-053.8e-051.5e-05

0.0001290.0003140.000107

0.0001040.0002058.5e-05

1.9e-05

1.1e-051.1e-051.6e-05

2.7e-054.2e-053.4e-05

3.5e-054.4e-051e-05

4.5e-05

3.1e-054.4e-052.5e-05

2.5e-050.0001092.2e-05

5.2e-05

2.5e-055.7e-052.2e-05

0.0224060.0131640.016090.0097390.0205640.0214050.009384

7e-051.7e-056e-05

3e-059e-062.5e-05

4e-058e-063.5e-05

0.0222740.0131640.016090.0097390.0203060.0212990.009384

0.0222380.0131640.016090.0097390.0202170.0212650.009384

3.6e-058.9e-053.4e-05

0.000124

6.6e-05

5.8e-05

5.9e-05

5.9e-05

6.2e-055.8e-054.6e-05

6.2e-055.8e-054.6e-05

0.0009620.000220.0033250.0009270.000431

0.0009620.000220.0033250.0009270.000431

0.0009620.000220.0033250.0009270.000431

0.0007790.000342

9.2e-05

9.2e-05

0.0006870.000342

0.000687

0.000342

0.0019020.0003010.0021470.0016990.000258

0.0013640.0003010.0018310.0012620.000197

0.0006387.7e-050.0002880.0005877.9e-05

1.3e-05

0.0001890.0001740.000177

0.0004497.7e-057e-060.000417.9e-05

9.4e-05

0.0007260.0002240.0015430.0006750.000118

0.0007260.0002240.0003310.0006750.000118

0.001212

0.0004190.0002140.0003456.1e-05

0.0002520.0001040.0001961.5e-05

0.0002520.0001040.0001961.5e-05

0.0001670.000110.0001494.6e-05

0.0001670.000110.0001494.6e-05

0.0001190.0001029.2e-05

0.0001190.0001029.2e-05

0.0001190.0001029.2e-05

0.0396610.0274640.0791990.0331310.0518210.0382080.051161

0.0014330.0021380.0003170.0044170.0012710.0011930.002092

0.000114

0.000114

0.0009690.0020910.0003170.0044170.0005680.0009060.00194

3e-05

0.0001114.1e-050.0001023.1e-05

0.0002078.2e-050.0001731.5e-05

5.7e-05

4e-052.4e-0503.8e-050.001432

3.3e-05

7e-06

2.7e-052.5e-050.0021088e-062.7e-050.000339

2.9e-05

8.4e-05

8.8e-05

0.0005840.0020420.0003170.0023090.0001090.0005660.000123

0.0001

0.0001

0.0003464.7e-050.000110.0002120.000152

8e-06

3.6e-05

0.0003464.7e-057.4e-050.0002120.000144

0.000116

0.000116

2.7e-056.7e-051.2e-05

2.3e-055e-059e-06

4e-061.7e-053e-06

4.6e-050.0001142.5e-05

4.6e-054.3e-052.5e-05

7.1e-05

4.5e-058.2e-053.8e-05

4.5e-058.2e-053.8e-05

0.0158470.013851

0.0158470.013851

0.0158470.013851

0.0195090.0128330.0295460.0130510.0303010.0193460.013321

0.0045820.005910.0026260.0085330.0019730.00450.005944

6.8e-052.4e-058e-066.8e-05

2.8e-05

0.0021560.0018750.0015650.0021080.000560.0020860.001533

5.7e-05

5.1e-05

0.0004170.0001070.0001680.0004090.000102

0.0001131.4e-054e-060.000119

0.0002672.9e-054.1e-050.0002648.8e-05

7.4e-05

0.0002042.6e-050.0021080.0001120.0002025.1e-05

1.5e-05

0.0001992e-050.000191

1e-06

3.3e-05

1.5e-05

0.000131.3e-050.000131

0.0001396.6e-050.000146

1.2e-05

0

7e-06

9.1e-052.9e-059.2e-057e-06

0.0001362.5e-050.000142

1.1e-05

5.6e-05

0.0004520.0037030.0010610.002613.2e-050.0004390.004063

1e-06

0.000251

5.6e-051.5e-051e-055.8e-05

2e-055e-062e-05

2.4e-05

5.5e-058.7e-050.0017072.1e-055.4e-05

2.2e-05

0.00012

2.4e-05

5e-06

1.9e-05

4.6e-053e-056e-064.5e-050.0001

2e-05

3.3e-057e-063.4e-05

0.0018260.0012280.000770.0019080.0008390.0017830.001302

0.000116

0.0007120.000930.000770.0019080.0002730.0006980.001226

0.0011140.0002980.0003470.0010857.6e-05

0.000103

0.0001240.0004620.000107

0.000164

1.5e-05

8.1e-05

2.6e-05

8.5e-05

2.5e-05

2.6e-05

2.5e-05

0.0001241.5e-050.000107

0.0007950.0001210.0004560.0007487.6e-05

4.3e-05

0.0001377e-050.000126

9e-052.9e-054.2e-058.6e-05

0.000254e-058.4e-050.0002397.6e-05

0.000116

9.2e-055.3e-057.5e-05

0.0002265.2e-054.8e-050.000222

0.0079430.0041190.026150.0230190.0081660.004231

0.0016390.0003160.0011520.001670.000518

0.0019190.0004320.0009620.0010950.0019870.00059

0.001017

0.0024280.0009470.0017220.0024950.001268

0.0019570.0024240.0017980.0008170.0020140.001855

0.0216680.018938

0.0042390.0014550.002610.0035520.0040420.001768

2.8e-050.0014550.002612.9e-051.2e-050.001768

1.6e-05

0.0042110.0035070.00403

0.0069190.0033330.0038390.0047190.003460.0065350.002632

7.1e-050.0002114.8e-05

7.1e-057.7e-054.8e-05

5.7e-05

7.7e-05

0.0016860.0005070.000760.0020080.0013390.0016020.000536

0.000134

0.0016860.0005070.000760.0020080.0002880.0016020.000536

0.000917

0.0050580.0028260.0030790.0027110.001680.0047990.002096

0.0050580.0028260.0030790.0027110.0016030.0047990.002096

4.3e-05

3.4e-05

0.000168

7.9e-05

8.9e-05

0.0001046.2e-058.6e-05

0.0001046.2e-058.6e-05

0.0110220.009160.0097880.0109440.0085530.0104860.009306

0.0001184.5e-050.000104

0.0001184.5e-050.000104

0.0001495.3e-054.9e-050.0001341.5e-05

0.0001495.3e-054.9e-050.0001341.5e-05

7.9e-057.5e-056.9e-05

7.9e-057.5e-056.9e-05

0.0004556.8e-050.0004170.0004249.4e-05

0.0001378.8e-050.0001184.9e-05

0.0003186.8e-050.0003290.0003064.5e-05

0.0001252.4e-055e-050.000129

6e-052.4e-051.2e-056.3e-05

2.2e-05

6.5e-051.6e-056.6e-05

2.6e-054.9e-052e-05

2.6e-054.9e-052e-05

0.000267.2e-050.0002217e-05

0.000267.2e-050.0002217e-05

0.0001770.0001590.0001661.8e-05

0.0001770.0001590.0001661.8e-05

0.0033480.0029480.002420.0037150.0015470.0032190.00108

0.0001164e-050.000104

0.0019430.0013590.0014080.0020080.0006080.0018850.00105

0.0010450.0005640.0010120.0017070.0007480.000997

4.2e-05

0.0001598.1e-050.000153e-05

8.5e-050.0010252.8e-058.3e-05

7.5e-05

3.8e-05

3.7e-05

9.4e-05

9.4e-05

0.000112

4.2e-05

7e-05

0.0062850.0060670.0073680.0072290.0056820.0060.008029

0.0062850.0060670.0073680.0072290.0056110.0060.008029

7.1e-05

0.000127

5.6e-05

7.1e-05

0.0007780.0198620.0082360.0006480.009959

0.0001777.8e-050.0001454.1e-05

0.0001777.8e-050.000145

4.1e-05

9.5e-057.4e-058.1e-05

5.6e-054.6e-05

3.9e-057.4e-053.5e-05

0.0002299.3e-050.0001963.5e-05

0.0002299.3e-050.0001963.5e-05

0.0198620.0076930.009829

0.000108

0.0112470.009829

0.0086150.007529

5.6e-05

4.4e-05

4.4e-05

5.7e-05

5.7e-05

5.2e-05

5.2e-05

0.0002770.0001450.0002265.4e-05

0.0002770.0001450.0002265.4e-05

0.012020.006910.0092230.0073290.0128620.0114290.00536

3.5e-053e-052.4e-05

3.5e-053e-052.4e-05

3.5e-053e-052.4e-05

3.5e-053e-052.4e-05

0.0001520.0004610.00013

4.4e-05

4.4e-05

4.4e-05

1.6e-057.8e-051.2e-05

4.2e-05

4.2e-05

1.6e-053.6e-051.2e-05

1.6e-053.6e-051.2e-05

5.1e-059.6e-054.5e-05

1.7e-052.7e-051.5e-05

1.7e-052.7e-051.5e-05

3.4e-056.9e-053e-05

9e-061.8e-058e-06

1.3e-05

1.4e-052e-051.1e-05

1.1e-051.8e-051.1e-05

2.2e-054e-051.7e-05

2.2e-054e-051.7e-05

2.2e-054e-051.7e-05

4.9e-059e-054.5e-05

4.9e-059e-054.5e-05

3.1e-054.9e-052.7e-05

1.2e-052.6e-051.2e-05

6e-061.5e-056e-06

1.4e-057.3e-051.1e-05

1.4e-054.5e-051.1e-05

1.4e-054.5e-051.1e-05

2.8e-05

2.8e-05

4e-05

4e-05

4e-05

2.6e-050.0001642.2e-05

1.9e-050.0001271.6e-05

4e-063e-053e-06

4e-063e-053e-06

1.3e-056.9e-051.1e-05

1.3e-056.9e-051.1e-05

2e-062.8e-052e-06

2e-062.8e-052e-06

7e-063.7e-056e-06

7e-063.7e-056e-06

7e-063.7e-056e-06

0.0001540.0003660.000136

3.1e-050.0001382.5e-05

7.5e-05

4.1e-05

3.4e-05

3.1e-056.3e-052.5e-05

1.3e-053.5e-059e-06

1.8e-052.8e-051.6e-05

0.0001230.0002280.000111

0.0001230.0001880.000111

1.4e-053e-051.5e-05

2.1e-052e-051.8e-05

2.7e-057e-062.4e-05

6e-06

1.9e-051e-051.7e-05

1.4e-053.9e-051.2e-05

7e-062.2e-059e-06

2.1e-05

1.4e-052.6e-051.2e-05

7e-067e-064e-06

4e-05

4e-05

0.0028150.0016310.0022570.0021080.0027580.0026760.001328

0.0027810.0016310.0022570.0021080.0026580.0026450.001328

6e-062.1e-056e-06

6e-062.1e-056e-06

0.0027750.0016310.0022570.0021080.0026370.0026390.001328

1.3e-051.6e-051.1e-05

2.3e-05

2.5e-053.1e-052.3e-05

7e-064e-06

2e-062e-06

1.2e-052.9e-058e-06

6e-063.5e-057e-06

2.4e-053.1e-052.3e-051.5e-05

1.8e-051.4e-05

0.0026380.0016310.0022570.0021080.0023970.0025210.001313

3e-053.1e-052.6e-05

4.4e-05

2.6e-057.8e-052.2e-05

2.6e-057.8e-052.2e-05

4.6e-05

2.6e-053.2e-052.2e-05

8e-062.2e-059e-06

8e-062.2e-059e-06

8e-062.2e-059e-06

3.5e-050.0003923.6e-05

2.5e-050.0003332.7e-05

2.5e-050.0003332.7e-05

2.5e-050.0003332.7e-05

1e-055.9e-059e-06

1e-055.9e-059e-06

6e-062.1e-056e-06

4e-063.8e-053e-06

2.1e-058.8e-051.9e-05

2.1e-058.8e-051.9e-05

3.9e-05

3.9e-05

2.1e-054.9e-051.9e-05

2.1e-054.9e-051.9e-05

0.0087520.0052790.0069660.0052210.0085680.008360.004032

0.004640.0027560.0035830.0027110.0044920.0044350.001942

6e-063.6e-054e-06

6e-063.6e-054e-06

0.0046250.0027560.0035830.0027110.0044030.0044220.001942

0.0046250.0027560.0035830.0027110.0044030.0044220.001942

1e-063.3e-051e-06

1e-063.3e-051e-06

8e-062e-058e-06

8e-062e-058e-06

0.0041120.0025230.0033830.002510.0040760.0039250.00209

2.1e-054.3e-051.8e-05

2.1e-054.3e-051.8e-05

5e-063.5e-055e-06

5e-063.5e-055e-06

2.2e-054.5e-051.6e-05

2.2e-054.5e-051.6e-05

0.0040640.0025230.0033830.002510.0039530.0038860.00209

0.0040640.0025230.0033830.002510.0039530.0038860.00209

3e-053.5e-052.6e-05

3e-053.5e-052.6e-05

3e-053.5e-052.6e-05

3e-053.5e-052.6e-05

0.131030.1580470.1710160.1833330.1825390.157490.17934

0.0414280.0379090.0577790.0706830.0543960.0525170.039601

0.0015858.8e-050.0016060.0008920.0013810.000206

0.0007230.000350.0006210.000113

2.8e-056e-063.1e-051e-05

4.8e-056e-065.2e-055e-06

0.000209

0.000224.8e-050.0001727e-06

8.2e-0508e-05

0.0003458.1e-050.0002869.1e-05

2.1e-052.7e-052.4e-05

2.1e-052.7e-052.4e-05

1.9e-054.5e-051.9e-05

1.9e-054.5e-051.9e-05

0.0008228.8e-050.0016060.000470.0007179.3e-05

2e-05

7.7e-059e-062.3e-056.4e-051.5e-05

0.0002251.2e-050.00018

7.8e-05

4.2e-05

0.0001313.1e-050.000104

0.0003897.9e-050.0016060.0002140.0003697.8e-05

5e-05

8.1e-05

8.1e-05

8.1e-05

0.0133210.0158820.0071380.0236950.0059750.0176560.019178

0.0019950.0059920.0110450.0003280.0019310.006721

0.0012770.0051540.0044180.0001960.001255

9.1e-050.0022095.2e-054.6e-050.001881

0.0002450.0002250.0019082.6e-050.0002430.001324

0.0003820.0006130.002515.4e-050.0003870.003516

0.0057130.0036790.0038530.0028110.0030480.0055030.002534

0.0003114.4e-050.0003283.3e-05

3.5e-05

5e-05

9.5e-05

6.7e-05

0.0054020.0036790.0038530.0028110.0028690.0051750.002339

5e-05

3.9e-05

3.9e-05

4.7e-050.0001995e-05

1.1e-052e-061e-05

9e-065.7e-058e-06

6e-061e-064e-06

2.1e-054.2e-052.8e-05

4.9e-05

4.8e-05

9.4e-05

9.4e-05

3.9e-054e-057.2e-05

3.9e-054e-057.2e-05

6.7e-05

6.7e-05

0.0011070.0038960.0017690.0030120.0001510.0010940.003506

1e-06

2e-06

0.0008360.003880.0017690.0030123.3e-050.0008610.003485

0.0001331.6e-051.1e-050.0001292.1e-05

9.4e-05

2.9e-052e-062.6e-05

0.0001098e-067.8e-05

5.7e-05

5.7e-05

0.0005970.0001890.0005364.4e-05

8.3e-056.1e-055.4e-05

4.4e-05

0.0001892.5e-050.000165

0.0002794.8e-050.000299

4.6e-055.5e-051.8e-05

0.0002012.3e-050.000213

0.0002012.3e-050.000213

7.2e-05

7.2e-05

0.0025230.0007510.0010230.0017070.0006040.0023920.000128

1.6e-05

4.1e-05

2e-061.7e-05

1.3e-05

7e-06

0.0015230.0007310.0010230.0017070.0004380.001439

8e-06

1e-06

7e-06

0.0003214.7e-050.0003

0.0001822e-056e-060.000157

0.0002121.5e-050.0002063.1e-05

0.0002851.1e-050.000296.4e-05

1e-06

2e-06

5e-06

0.0003040.0001130.000309

0.0003040.0001130.000309

7.1e-05

7.1e-05

5.2e-05

5.2e-05

0.0001481.2e-057.6e-050.0001564.6e-05

1.2e-05

8.6e-051.2e-056e-068.6e-052.7e-05

1.7e-05

1.6e-05

4e-06

0

6.2e-052.1e-057e-051.9e-05

5.3e-055.1e-05

5.3e-055.1e-05

0.0002590.0006050.0004930.002510.0003010.0050160.002304

0.0001311e-050.0001181.7e-05

0.0001280.0006050.0004930.002510.0002910.0048980.002287

2.6e-05

2.6e-05

4.6e-053.5e-055.4e-05

4.6e-053.5e-055.4e-05

9.1e-05

9.1e-05

7.4e-05

7.4e-05

7.1e-05

5.4e-05

1.7e-05

0.0002890.0009470.002616.8e-050.0002790.003895

8.9e-050.0009470.002611.1e-058.6e-050.003451

0.00023.7e-050.0001930.00022

2e-050.000224

8.6e-05

8.6e-05

0.0012160.000520.0010839.1e-05

0.0002046.1e-050.000163

0.0002046.1e-050.000163

0.000182.6e-050.000162

0.000182.6e-050.000162

9.5e-058e-068.5e-05

9.5e-058e-068.5e-05

0

9.7e-05

9.7e-05

0.0003620.0001890.0003411.5e-05

4.2e-05

0.0001635e-050.000158

3.7e-05

2.4e-05

0.0001993.6e-050.0001831.5e-05

0.0003750.0001390.0003327.6e-05

3.7e-05

0.0001474.7e-050.0001397.6e-05

0.0002285.5e-050.000193

0.0218770.0199340.0506410.0406630.0444430.0287080.018788

0.020210.0195520.0506410.0406630.0425830.0271150.018382

0.001127

0.0059070.005163

0.0189750.0143930.005510.0196790.0010520.0258680.011986

7.7e-05

0.0011730.0007280.0006870.0053210.0001520.0011860.006292

0.0101820.008899

0.0103920.009082

0.0179630.0157

6.2e-050.0044310.0156631.4e-056.1e-050.000104

0.00131

7e-06

0.0014220.0002470.0007330.0013570.000374

0.0004485.9e-050.0001210.000472

007.9e-05

0.0003862.7e-053.6e-050.000329

0.0001017.2e-052.9e-050.0001110.000154

1.1e-05

0.0004874.7e-055.4e-050.000445

4.2e-05

8e-06

0.000426

1.7e-05

6.2e-05

3.1e-05

7.9e-05

0.0002450.0001354.5e-050.0002363.2e-05

0.0002166.4e-051e-050.0002033.2e-05

2.9e-057.1e-053.5e-053.3e-05

0.001082

0.001082

0.000159

0.000159

0.000159

0.0001864.2e-050.0002430.00016

0.0001864.2e-056.2e-050.00016

2e-06

0.0001644.2e-053.1e-050.000143

1.4e-051.9e-051.2e-05

8e-065e-065e-06

5e-06

9.9e-05

9.9e-05

8.2e-05

8.2e-05

0.0032430.0019630.0047190.0020830.0035290.001338

0.0026170.0019490.0047190.0018320.0030150.001213

0.0009980.0001960.0020081.9e-050.0009880.000224

0.0007238.1e-059.6e-050.0011369.7e-05

0.0004392.6e-050.000439

0.0004570.0016720.0027117e-060.000452

0.0016840.000892

0.0001290.0001289.4e-05

3.4e-05

5.7e-05

0.0001293.7e-059.4e-05

0.0004971.4e-050.0001230.000420.000125

2.4e-05

0.0002757.7e-050.0002233.1e-05

0.0002221.4e-052.2e-050.0001979.4e-05

0.0001540.0004150.000129

0.0001540.0004150.000129

2e-050.0001312e-05

2e-050.0001312e-05

0.0001189.1e-059.2e-05

0.0001189.1e-059.2e-05

1.6e-050.0001931.7e-05

1.6e-057.3e-051.7e-05

5.4e-05

6.6e-05

0.0009135e-050.0026030.0007443.1e-05

6.3e-059e-055.2e-05

6.3e-059e-055.2e-05

3.2e-05

2.7e-05

6.3e-053.1e-055.2e-05

3.9e-054.7e-053.1e-05

3.9e-054.7e-053.1e-05

3.9e-054.7e-053.1e-05

4.8e-05

4.8e-05

4.8e-05

9e-050.000138.1e-05

9e-050.000138.1e-05

2.2e-052e-062.3e-05

4e-06

6.8e-056.8e-055.8e-05

5.6e-05

0.0001530.0002410.0001181.5e-05

4.7e-05

4.7e-05

9.5e-050.0001027.5e-051.5e-05

3.8e-052.8e-052.9e-05

2.4e-053e-051.6e-05

3.3e-054.4e-053e-051.5e-05

5.8e-053.2e-054.3e-05

5.8e-053.2e-054.3e-05

6e-05

6e-05

0.0005685e-050.0020470.0004621.6e-05

8.8e-05

8.8e-05

5e-05

5e-05

5.1e-051.3e-054.1e-05

5.1e-051.3e-054.1e-05

0.0002835e-050.0017620.0002711.6e-05

0.000131

0.000132

0.001139

0.0002835e-050.0002220.0002711.6e-05

0.000138

0.0001580.0001180.000147

0.0001580.0001180.000147

7.6e-051.6e-053e-06

7.6e-051.6e-053e-06

0.0163850.0061310.0140970.0052210.0211950.0158050.003613

5.7e-050.0001134.7e-05

5.7e-050.0001134.7e-05

5.8e-05

5.7e-055.5e-054.7e-05

0.000280.00030.000252

7.8e-055.4e-056.9e-05

7.8e-055.4e-056.9e-05

3.6e-057.9e-053e-05

8e-061.8e-055e-06

1.2e-054.2e-051.2e-05

1.6e-051.9e-051.3e-05

5.6e-055.1e-055e-05

5.6e-055.1e-055e-05

0.000115.8e-050.000103

0.000115.8e-050.000103

5.8e-05

5.8e-05

5.7e-05

5.7e-05

5.7e-05

0.0160480.0061310.0140970.0052210.0206710.0155060.003613

0.000176

0.000176

0.0001280.000150.0001171.6e-05

0.0001280.000150.0001171.6e-05

8.5e-05

8.5e-05

0.0156220.0061310.0140970.0052210.0198840.0151260.003582

0.0059210.005175

0.0148940.0061310.0081760.0052210.0141280.0147290.003582

0.000218

0.0003440.000194

0.0003840.0001690.000397

0.0001470.0001980.0001471.5e-05

0.0001470.0001980.0001471.5e-05

0.0001510.0001780.000116

7.5e-059.1e-056.1e-05

7.6e-058.7e-055.5e-05

5.4e-05

5.4e-05

5.4e-05

8.6e-05

8.6e-05

8.6e-05

8.6e-05

2e-057.4e-051.3e-051.5e-05

2e-057.4e-051.3e-051.5e-05

2e-057.4e-051.3e-05

2e-057.4e-051.3e-05

1.5e-05

1.5e-05

0.0002050.0005760.000169

0.0002050.0005760.000169

0.0001580.0002650.000126

1.2e-055.2e-051.2e-05

4.1e-05

0.0001317.8e-058.7e-05

1.5e-053e-052.7e-05

6.4e-05

5.9e-05

5.9e-05

0.000104

3.8e-05

6.6e-05

5.2e-05

5.2e-05

4.7e-052.4e-054.3e-05

4.7e-052.4e-054.3e-05

7.2e-05

7.2e-05

0.0011810.0051060.0015630.0055220.0008820.001980.006461

0.0002650.0002710.0002

6e-06

6e-06

3.8e-054.4e-051.6e-05

3.8e-051.7e-051.6e-05

2.7e-05

4.1e-054e-054.4e-05

4.1e-054e-054.4e-05

1.5e-053e-051.3e-05

1e-061.8e-051e-06

1.4e-051.2e-051.2e-05

0.0001710.0001510.000127

0.0001369.3e-050.000127

3.5e-055.8e-05

0.0009160.0051060.0015630.0055220.0005260.001780.006461

2.5e-05

2.5e-05

0.0009160.0051060.0015630.0055220.0005010.001780.006461

9e-06

0.0008990.0051060.0015630.0055220.0004580.0017620.006461

1.9e-05

3e-061e-063e-06

7e-065e-069e-06

7e-069e-066e-06

8.5e-05

3.6e-05

3.6e-05

4.9e-05

4.9e-05

0.014260.0306840.0511170.0284140.0648130.012890.071114

0.0048780.0067140.0020760.0119480.0026680.0044510.00962

0.0048150.0066970.0020760.0119480.0025780.0043980.00962

6e-05

6.6e-051.2e-056e-051.5e-05

2.1e-05

5e-06

2.6e-05

6.1e-05

0.0001923.7e-053.3e-050.000182

2.5e-05

4.3e-05

0.000220.0003850.002414.3e-050.0001970.000932

8.3e-051.6e-057.9e-05

0.000193

9.3e-05

1.4e-05

0.0002320.0001763.4e-050.0002298.8e-05

0.0001422.4e-053e-060.000132

4.9e-05

3.3e-05

2.3e-05

7.3e-05

6e-06

4.6e-05

0.000930.0049990.0008770.005020.0001970.0008890.007096

4e-05

1.5e-05

8.6e-058e-068.2e-058.1e-05

8e-06

1.6e-05

0.0013790.000950.0011990.0020080.0005910.0013190.001001

5.7e-05

3e-05

1.7e-051.9e-051.8e-05

0.000152.5e-050.00015

8.2e-051.2e-057.8e-054.8e-05

7e-06

3.4e-05

3.7e-05

7.7e-05

7e-06

0.0001715.1e-050.000166

2.6e-05

7e-05

0.0001012e-050.002512.5e-059e-053e-05

0.0001535.9e-052.1e-050.0001470.000278

6e-05

1.7e-05

0.000164

8e-06

0.0002624.7e-053.7e-050.0002395.1e-05

2e-05

0.0001925e-050.000168

1.5e-05

2.8e-05

7e-06

0.0001935.1e-050.000173

1.5e-05

1.5e-05

6.3e-051.7e-057.5e-055.3e-05

6.3e-051.7e-052.5e-055.3e-05

5e-05

0.0093820.023970.0490410.0164660.0621450.0084390.061494

9.5e-050.0001850.0001658.9e-05

6.3e-05

8e-05

9.5e-050.0001852.2e-058.9e-05

0.0001390.0002260.000170.000149

4.1e-05

9e-06

1.6e-056.1e-058e-061.8e-05

2.7e-05

2.2e-055.7e-058e-062.1e-05

3.5e-051.6e-054e-05

2.5e-05

6.6e-050.0001083.6e-057e-05

0.0091480.0235590.0490410.0164660.061810.0082010.061494

3.1e-05

7.1e-05

0.001108

0.000637

0.001059

0.0050130.0219270.0037840.0140560.0008270.0039180.029042

0.0018440.0007690.0010810.0005360.001898

0.001753

0.001169

0.000837

0.0010210.0001920.001097.6e-05

0.000339

0.001270.0008630.002410.0001710.001295

0.0441760.0559940.029462

0.0059120.0081220.0081970.0141570.002560.0045470.010216

0.0059120.0081220.0081970.0141570.002560.0045470.010216

2.9e-054.3e-050.0022090.0001842.7e-05

5.2e-05

0.00011

2.9e-054.3e-050.0022092.2e-052.7e-05

0.0026410.0056580.006740.0095380.0010180.0022330.006627

0.0050570.00442

0.0013520.005250.001070.0040160.0002810.0011640.001226

0.000303

0.0001392.7e-050.000138

0.0006910.0002530.0006130.0035140.0005610.0007150.000428

0.000233

0.0004590.0001550.0020080.0001490.0002161.7e-05

0.0003980.0001570.0003660.0004150.001684

7.8e-057.5e-057.2e-058.1e-051.5e-05

9e-05

0.001669

0.0002358.2e-057.6e-050.000249

8.5e-050.0001288.5e-05

0.0024880.0022640.0014570.002410.0005880.001530.001897

0.0002490.0001080.000273

0.0022390.0022640.0014570.002410.000480.0012570.001897

5.7e-054.6e-056e-05

5.7e-054.6e-056e-05

2.6e-052.8e-054.1e-05

2.6e-052.8e-054.1e-05

8.4e-050.0001196.5e-058e-06

8.4e-050.0001196.5e-058e-06

0.0001167.5e-050.000112

0.0001167.5e-050.000112

7.3e-050.0001366.4e-05

7.3e-050.0001366.4e-05

0.000118

0.000118

6.7e-05

6.7e-05

5.1e-05

5.1e-05

0.017950.0179740.0137640.0138550.0154030.0176360.009341

0.017950.0179740.0137640.0138550.0154030.0176360.009341

3.5e-050.0001033.2e-057.5e-05

5.8e-05

1.5e-05

6e-05

3.5e-054.5e-053.2e-05

0.0178280.0179740.0137640.0138550.0145360.017520.009251

4.1e-051.8e-050.0027111.5e-053.4e-053.8e-05

6e-053.5e-056.7e-052.8e-05

8e-06

3.8e-058e-06

3e-06

3.6e-055.8e-053.7e-05

2.1e-05

3.7e-051e-05

2.7e-051.1e-052.5e-05

0.0153660.0084020.0112590.0055220.0133370.014719

2.1e-056e-062.8e-05

1.8e-059e-064e-061.6e-05

1.7e-058e-061.8e-051.5e-05

3.3e-052.6e-059e-055.1e-05

0.0021760.0095450.0025050.0056220.0006750.0024630.008987

3.3e-054e-062.3e-05

1.1e-051.5e-05

6e-06

1.5e-05

4.5e-052.4e-05

4.6e-05

3e-06

9.5e-05

4.6e-05

5.8e-05

8.7e-058.5e-058.4e-051.5e-05

3.4e-053.8e-053.7e-051.5e-05

5.3e-052.3e-054.7e-05

2.4e-05

0.000679

0.000679

0.0105290.0068840.0073660.0091370.0094030.0099310.005078

0.0002250.0006820.000216

0.0002250.0006820.000216

3e-06

2.4e-05

3.3e-054e-062.5e-05

2.6e-05

1.8e-05

0.0001450.0005690.000145

7e-06

6e-06

4.7e-052e-064.6e-05

2.3e-05

0.005970.0036160.0042360.0030120.00480.005680.002325

0.005970.0036160.0042360.0030120.00480.005680.002325

4.7e-054.2e-05

0.0056060.0036110.0042360.0030120.0046170.0053540.002325

1.3e-051e-051.3e-05

2.7e-051.4e-053e-05

2.1e-051.6e-051.8e-05

8e-061.9e-051.1e-05

8e-065e-064e-068e-06

1.9e-059e-061.5e-05

2.5e-052.7e-052e-05

9e-062e-067e-06

8e-063e-069e-06

6e-062e-065e-06

5.5e-057e-064.5e-05

1.4e-055e-061.2e-05

4.4e-052.9e-053.8e-05

2.9e-052.1e-052.5e-05

3.1e-051.5e-052.8e-05

0.0041660.0032680.003130.0061250.0037070.0038980.002753

0.0004250.0009240.0022090.0002450.0003310.000874

7e-06

1.4e-05

1.9e-050.0009240.0022091.1e-051.7e-050.000874

0

2.9e-051.4e-053.3e-05

1e-06

0.0003770.0001980.000281

4.8e-05

4.8e-05

4.4e-05

4.4e-05

0.0005760.0004670.000580.0017070.000510.0005380.000424

3.8e-051.4e-053e-05

1.3e-05

1.1e-05

1.6e-05

0.0004990.0004670.000580.0017070.0003780.0004780.000424

2.1e-05

3.9e-051.4e-053e-05

4.3e-05

0.0031360.0018770.002550.0022090.0027880.0030030.001455

1.5e-054.5e-051e-05

0.0031210.0018770.002550.0022090.0027430.0029930.001455

2.9e-057.2e-052.6e-05

2.9e-057.2e-052.6e-05

3.2e-05

3.2e-05

3.2e-05

5.1e-053.6e-054.3e-05

5.1e-053.6e-054.3e-05

5.1e-053.6e-054.3e-05

1.7e-054.2e-051.5e-05

1.7e-054.2e-051.5e-05

2e-05

1.7e-052.2e-051.5e-05

6.6e-057.8e-056.1e-05

6.6e-057.8e-056.1e-05

2.7e-053.7e-052.3e-05

3.9e-054.1e-053.8e-05

3.4e-052.6e-051.8e-05

3.4e-052.6e-051.8e-05

3.4e-052.6e-051.8e-05

7e-05

7e-05

7e-05

7e-05

0.0001010.0003299.1e-050.000339

0.000339

0.000339

0.000339

7.2e-05

7.2e-05

3.2e-05

4e-05

0.0001010.0001699.1e-05

2.4e-054.3e-052.1e-05

2.4e-054.3e-052.1e-05

3.7e-055.5e-053.6e-05

3.7e-055.5e-053.6e-05

1.6e-053.6e-051e-05

1.6e-053.6e-051e-05

2.4e-053.5e-052.4e-05

2.4e-053.5e-052.4e-05

8.8e-05

4.6e-05

4.6e-05

4.2e-05

4.2e-05

0.0194670.0403420.0159220.0308220.0089010.0387680.026498

0.0192250.0403420.0159220.0308220.0086370.0385880.026498

0.0002386.3e-050.0001080.000180.000175

0

4.5e-05

4.4e-05

1e-06

0.0002386.3e-051.8e-050.000180.000175

6.7e-05

1.7e-05

2.7e-05

2.3e-05

0.0008095.7e-050.0001140.0006160.000145

0.000475.7e-054.9e-050.0003699.3e-05

0.0003396.5e-050.0002475.2e-05

0.0181720.0402220.0159220.0308220.0083370.0377860.026178

0.0115860.0316880.011720.014960.0031210.0294120.021447

9.6e-05

0.0007320.0002170.0001440.000772

3.4e-050.0011160.0022098e-064.1e-050.001543

0.0011590.0042790.0009080.0030120.0002640.001220.000657

0.0011490.0019730.0016110.0021080.0006840.002670.001744

6.3e-05

0.0010610.0003240.0005940.002610.0002010.0010950.000787

6e-060.002612e-066e-06

0.0024450.0006250.0010890.0033130.0003920.00257

0.003362

6e-061.1e-056e-06

6e-061.1e-056e-06

0.0002420.0002640.00018

7.4e-054.7e-055.3e-05

7.4e-054.7e-055.3e-05

0.0001684.3e-050.000127

0.0001684.3e-050.000127

0.000101

4e-05

2.6e-05

3.5e-05

7.3e-05

7.3e-05

0.0009510.0039810.0007790.0037150.0003560.0008370.003887

0.0009490.0039810.0007790.0037150.00030.0008360.003887

0.0009490.0039810.0007790.0037150.0002670.0008360.003887

0.0009390.0039810.0007790.0037150.0001740.0008250.003887

2.2e-05

2.8e-05

1e-052.4e-051.1e-05

1.9e-05

3.3e-05

3.3e-05

2e-065.6e-051e-06

2e-063.9e-051e-06

2e-061e-051e-06

2.9e-05

1.7e-05

1.7e-05

0.0015740.0008640.0004320.0018070.0003590.0014330.003146

0.0015740.0008640.0004320.0018070.0003590.0014330.000787

0.0001458.4e-059.4e-051.5e-05

0.0001458.4e-059.4e-051.5e-05

0.0013230.0008640.0004320.0018070.000210.0012640.000772

0.000248

1e-05

0.0001961.4e-050.0001821.9e-05

0.0001342.2e-050.0001250.000114

5.9e-05

0.0009930.0008640.0004320.0018070.0001050.0009570.000391

0.0001066.5e-057.5e-05

0.0001066.5e-057.5e-05

0.002359

0.002359

0.002359

0.0001720.000117

0.0001720.000117

0.0001720.000117

0.0001720.000117

0.0001720.000117

0.0001720.000117

0.0001720.000117

0.08268000000000010.0551050.0617350.0490960.07828500000000010.0792950.047412

02.6e-050

02.6e-050

02.6e-050

02.6e-050

02.6e-050

02.6e-050

5e-060.0001115e-06

5e-060.0001115e-06

5e-063e-055e-06

5e-063e-055e-06

5e-063e-055e-06

5e-062.1e-055e-06

9e-06

8.1e-05

8.1e-05

3.5e-05

3.5e-05

4.6e-05

4.6e-05

2.4e-059.1e-051.7e-05

2.4e-059.1e-051.7e-05

2.4e-059.1e-051.7e-05

2.4e-059.1e-051.7e-05

2.4e-059.1e-051.7e-05

2.4e-059.1e-051.7e-05

0.0466620.0251720.0335660.0245980.0426820.0448610.022414

0.0048280.0028950.0037860.0028110.0046720.0046240.002219

0.0047780.0028950.0037860.0028110.0043110.0045660.002219

0.0047780.0028950.0037860.0028110.0043110.0045660.002219

0.0047780.0028950.0037860.0028110.0043110.0045660.002219

0.0047760.0028950.0037860.0028110.0042890.0045640.002219

2e-062.2e-052e-06

1.3e-050.0002351.3e-05

7e-060.000187e-06

02.9e-050

02.9e-050

1e-061.7e-051e-06

1e-061.7e-051e-06

1e-061.2e-051e-06

1e-061.2e-051e-06

2.4e-050

1.4e-050

1e-05

4e-061.6e-054e-06

4e-061.6e-054e-06

06.7e-050

3e-06

1e-06

9e-06

8e-06

1.3e-05

1.4e-05

1e-06

02e-060

06e-060

4e-06

02e-060

4e-06

1e-061.5e-051e-06

1e-061.5e-051e-06

6e-065.5e-056e-06

6e-065.5e-056e-06

2e-062.9e-053e-06

4e-062.6e-053e-06

3.7e-050.0001264.5e-05

1.8e-054.1e-052.8e-05

1.7e-052.8e-052.7e-05

1.6e-051.6e-051.4e-05

1e-061.2e-051.3e-05

1e-061.3e-051e-06

1e-061.3e-051e-06

8e-062.4e-058e-06

8e-062.4e-058e-06

8e-062.4e-058e-06

1e-061.4e-050

1e-061.4e-050

1e-061.4e-050

1e-054.7e-059e-06

9e-063e-058e-06

6e-061.5e-055e-06

4e-06

3e-061.1e-053e-06

1e-061.7e-051e-06

1e-061.7e-051e-06

6e-067.8e-055e-06

6e-067.8e-055e-06

6e-067.8e-055e-06

6e-064.6e-055e-06

3e-061.3e-053e-06

2e-061.5e-051e-06

1e-061.2e-051e-06

06e-060

2.2e-05

1.2e-05

1e-05

01e-050

01e-050

0.0008170.0004830.0008850.0016060.0008140.000781

0.0008170.0004830.0008850.0016060.0008140.000781

0.0008170.0004830.0008850.0016060.0008140.000781

0.0008170.0004830.0008850.0016060.0008140.000781

0.0008170.0004830.0008850.0016060.0007920.000781

2.2e-05

6.2e-050.0002796.2e-05

2e-067.7e-053e-06

2e-067.7e-053e-06

03.4e-051e-06

03.4e-051e-06

2e-061.9e-052e-06

2e-061.9e-052e-06

2.4e-05

2.4e-05

3.3e-05

3.3e-05

3.3e-05

3.3e-05

6e-050.0001695.9e-05

5.5e-050.0001045.4e-05

5.5e-050.0001045.4e-05

7e-063.6e-057e-06

4.8e-056.8e-054.7e-05

2e-061.8e-053e-06

2e-061.8e-053e-06

2e-061.8e-053e-06

2e-062.6e-052e-06

2e-062.6e-052e-06

2e-062.6e-052e-06

1e-062.1e-050

1e-062.1e-050

1e-062.1e-050

0.0020460.0013460.0018730.0035140.0016260.0019580.001239

0.0020460.0013460.0018730.0035140.0016260.0019580.001239

0.0020460.0013460.0018730.0035140.0016260.0019580.001239

0.000270.0003310.0003740.0016060.0002570.000260.000364

3e-06

03e-060

7e-06

6e-06

0.0002690.0003310.0003740.0016060.0002280.0002590.000364

1e-067e-061e-06

03e-06

0.0017760.0010150.0014990.0019080.0013650.0016980.000875

03e-060

4e-066e-064e-06

7e-06

4e-06

03e-061e-06

5e-06

03e-060

1e-063e-060

0.0017690.0010150.0014990.0019080.0012960.0016910.000875

3e-06

4e-06

2e-06

2e-065e-062e-06

06e-060

06e-060

5e-06

4e-06

4e-06

4e-06

0.0070720.0038060.0052340.0045180.006220.0067860.003417

0.000150.000320.0001491.4e-05

0.000150.000320.0001491.4e-05

2.8e-05

2.8e-05

1.9e-053.6e-051.9e-05

1.9e-053.6e-051.9e-05

1.5e-054.6e-051.4e-05

1.5e-054.6e-051.4e-05

1.3e-052.9e-051.4e-05

1.3e-052.9e-051.4e-05

1.5e-053.3e-051.4e-05

1.5e-053.3e-051.4e-05

1.5e-052.8e-051.6e-051.4e-05

1.5e-052.8e-051.6e-051.4e-05

5.9e-058.9e-055.8e-05

2.8e-054.2e-052.6e-05

3.1e-054.7e-053.2e-05

1.4e-053.1e-051.4e-05

1.4e-053.1e-051.4e-05

0.0021260.0010960.0015870.0019080.0015270.0020410.001112

0.0021070.0010960.0015870.0019080.0013330.002020.001112

0.0020840.0010960.0015870.0019080.0012160.0019970.001112

2.8e-055.4e-052.7e-05

0.000158

0.0020560.0010960.0015870.0019080.0010040.001970.001112

1e-056.2e-051.1e-05

1e-056.2e-051.1e-05

1.3e-055.5e-051.2e-05

1.3e-055.5e-051.2e-05

1.9e-050.0001942.1e-05

1.9e-050.0001942.1e-05

1.9e-050.0001942.1e-05

0.0047960.002710.0036470.002610.0043730.0045960.002291

0.0047790.002710.0036470.002610.0041840.0045790.002291

1.5e-055e-051.8e-05

1.5e-055e-051.8e-05

0.0046870.002710.0036470.002610.0039420.0044850.002291

9e-063.9e-051.2e-05

0.0046780.002710.0036470.002610.0039030.0044730.002291

1.2e-052.2e-051.1e-05

5e-061e-064e-06

7e-061.7e-057e-06

4e-06

6.5e-050.000176.5e-05

3.4e-059e-053.5e-05

3.1e-058e-053e-05

1.7e-050.000161.7e-05

6e-065.3e-056e-06

6e-065.3e-056e-06

1.1e-050.0001071.1e-05

4.2e-05

1.1e-052.4e-051.1e-05

4.1e-05

2.9e-05

2.9e-05

2.9e-05

0.0318230.0166420.0217880.0121490.0289110.0306380.015539

0.0318230.0166420.0217880.0121490.0289110.0306380.015539

2e-061.6e-052e-06

2e-061.6e-052e-06

2e-061.6e-052e-06

0.0318210.0166420.0217880.0121490.0288950.0306360.015539

4e-064.6e-054e-06

3e-06

02.1e-050

1e-061.1e-051e-06

3e-061.1e-053e-06

0.0314560.0164150.0214240.0106430.0284880.0302880.015346

0.000105

0.0314380.0164150.0214240.0106430.0281390.0302720.015346

8e-063.2e-057e-06

1e-053.6e-059e-06

0.000145

3.1e-05

1e-061.8e-050

1e-064e-060

01.2e-050

2e-06

0.000360.0002270.0003640.0015060.0003430.0003440.000193

0.000360.0002270.0003640.0015060.0003430.0003440.000193

8e-068.2e-057e-06

8e-068.2e-057e-06

6e-064.8e-056e-06

07e-060

07e-060

6e-064.1e-056e-06

2e-061.4e-052e-06

01.1e-050

2e-061.2e-051e-06

2e-064e-063e-06

2e-063.4e-051e-06

1e-067e-060

1e-067e-060

1e-062.7e-051e-06

1e-068e-061e-06

1e-06

01e-060

2e-06

07e-060

8e-06

0.0359890.0299330.0281690.0244980.0353750.0344120.024998

0.0359890.0299330.0281690.0244980.0353750.0344120.024998

2e-062.7e-051e-06

01.4e-050

01.4e-050

01.4e-050

2e-061.3e-051e-06

2e-061.3e-051e-06

2e-061.3e-051e-06

0.001660.0084130.0028910.0054220.0014520.0015890.008541

0.001660.0084130.0028910.0054220.0014520.0015890.008541

01.5e-05

01.5e-05

01.1e-050

2e-06

09e-060

0.001660.0084130.0028910.0054220.0014260.0015890.008541

0.0016570.0084130.0028910.0054220.001390.0015860.008492

1.4e-05

2e-066e-062e-064.9e-05

1e-061.3e-051e-06

03e-060

0.000240.0019590.0002040.0038150.0009160.0002320.001462

6e-062.2e-054e-06

6e-062.2e-054e-06

6e-061.1e-054e-06

9e-06

1e-06

01e-06

0.0002340.0019590.0002040.0038150.0008940.0002280.001462

1.5e-050.0018420.0023090.0004951.6e-050.00136

1.5e-050.0004951.6e-05

0.0018420.0023090.00136

1.6e-050.000161.7e-05

5e-066.6e-055e-06

1.1e-059.4e-051.2e-05

02.1e-051e-06

01.1e-050

1e-051e-06

0.0002030.0001170.0002040.0015060.0002180.0001940.000102

0.0002030.0001170.0002040.0015060.0002180.0001940.000102

0.0340870.0195610.0250740.0152610.032980.032590.014995

03.2e-05

01e-05

01e-05

1.1e-05

1.1e-05

1.1e-05

1.1e-05

0.0340870.0195610.0250740.0152610.0329480.032590.014995

03.1e-050

01.1e-050

2e-05

7e-06

2e-06

5e-06

2e-061.9e-051e-06

2e-061.9e-051e-06

0.0340840.0195610.0250740.0152610.0328720.0325880.014995

0.0340840.0195610.0250740.0152610.0328720.0325880.014995

1e-061.9e-051e-06

1e-061.9e-051e-06
