# Supplementary material for: MetaMeta: integrating metagenome analysis tools to improve taxonomic profiling
Source: Microbiome. 2017 Aug 14;5:101. doi: 10.1186/s40168-017-0318-y (PMC5557516; doi:10.1186/s40168-017-0318-y)
Supplement: Supplementary file 2 — Additional File with interactive charts for all CAMI toy set results on default, very-precise and very-sensitive mode. File prefix S, M, and H for low, medium and high complexity, respectively. (TAR 3573 kb) [file 40168_2017_318_MOESM2_ESM.tar › H_S005__insert_180_very-precise.html]

Javascript must be enabled to view this page.

magnitude
magnitudeUnassigned

clark.parsed\_profile
dudes.parsed\_profile
final.metametamerge.profile
gottcha.parsed\_profile
kaiju.parsed\_profile
kraken.parsed\_profile
motus.parsed\_profile

0.9999979999999980.9999959999999990.9999991.0000041.0000070.9999939999999990.999996999999999

0.9145769999999990.9432759999999990.935890.9503209999999990.9188880.9176549999999990.951827999999999

0.0009550.0005570.0008160.0017060.0008820.0009190.000372

0.0009550.0005570.0008160.0017060.0008820.0009190.000372

0.0009550.0005570.0008160.0017060.0008820.0009190.000372

0.0009550.0005570.0008160.0017060.0008820.0009190.000372

0.0009550.0005570.0008160.0017060.0008820.0009190.000372

0.0009540.0005570.0008160.0017060.0008560.0009170.000372

1e-062.6e-052e-06

0.0088780.0085610.0075370.0091340.008240.008570.006863

0.0088780.0085610.0075370.0091340.008180.008570.006863

0.0036440.0056340.0035780.0062230.0030260.0035490.004576

0.0036440.0056340.0035780.0062230.0030260.0035490.004576

0.0026670.0016120.0021860.0021080.0020350.0026950.001368

7.3e-059e-050.000199

0.0025940.0016120.0021860.0021080.0019450.0024960.001368

0.0009770.0040220.0013920.0041150.0009910000000000010.0008540.003208

2e-0602e-06

1.9e-05

7e-06

1.3e-051.1e-054e-06

2e-05

2e-061.7e-052e-06

02e-061e-06

01e-061e-06

6e-06

1.6e-05

5e-062e-06

1e-064e-061e-06

1e-05

8e-061.2e-051.1e-05

02e-065e-06

5e-063e-064e-06

9e-062.5e-059e-06

7e-061.1e-057e-06

2.8e-058e-062.8e-05

3e-06

3e-064e-06

8e-062e-068e-06

0.000780.0040220.0013920.0041150.0007330.0006860.003208

2e-062e-062e-06

1.9e-054e-061.8e-05

1e-066e-061e-06

2.4e-051.5e-051.8e-05

1e-061.5e-05

1.7e-051.3e-051.4e-05

3.1e-054e-061.6e-05

2e-063e-062e-06

2e-06

4e-06

6e-061e-065e-06

1e-066e-061e-06

2e-064e-062e-06

0.0051750.0029270.0039590.0029110.0050480.0049620.002287

0.0051750.0029270.0039590.0029110.0050480.0049620.002287

3.3e-054e-052.6e-05

2e-061e-062e-06

2e-068e-062e-06

0

2.7e-051.9e-052.1e-05

2e-061.2e-051e-06

0.0051420.0029270.0039590.0029110.0050080.0049360.002287

2.1e-054.3e-051.3e-05

0.0051130.0029270.0039590.0029110.0048860.0049160.002287

8e-064.4e-057e-06

3.5e-05

5.9e-050.0001065.9e-05

5.4e-059.3e-055.4e-05

5.4e-059.3e-055.4e-05

1.1e-059e-061.1e-05

2e-061.3e-051e-06

2e-056e-062.4e-05

3e-06

8e-06

1.2e-05

9e-06

1.1e-053e-061.1e-05

1e-056e-067e-06

1.3e-05

1.1e-05

0

5e-061.3e-055e-06

5e-061.3e-055e-06

5e-061.3e-055e-06

6e-05

6e-05

6e-05

6e-05

6e-05

1.5e-050.0001731.1e-05

5e-063.5e-051e-06

5e-063.5e-051e-06

5e-063.5e-051e-06

5e-063.5e-051e-06

5e-063.5e-051e-06

1e-057.1e-051e-05

1e-057.1e-051e-05

1e-057.1e-051e-05

1e-057.1e-051e-05

1e-057.1e-051e-05

6.7e-05

6.7e-05

6.7e-05

6.7e-05

6.7e-05

5.9e-050.0003073.8e-05

5.9e-050.0003073.8e-05

2.9e-050.0002521.9e-05

1.5e-056.6e-059e-06

6e-062.7e-052e-06

6e-062.7e-052e-06

9e-063.9e-057e-06

5e-061.5e-054e-06

4e-062.4e-053e-06

1.4e-050.0001241e-05

1e-061.1e-051e-06

1e-061.1e-051e-06

2e-065.1e-051e-06

2e-065.1e-051e-06

1e-055e-067e-06

9e-065e-067e-06

1e-060

1e-065.7e-051e-06

2.3e-05

1e-063.4e-051e-06

6.2e-05

6.2e-05

6.2e-05

3e-055.5e-051.9e-05

3e-055.5e-051.9e-05

2.9e-053.4e-051.8e-05

2.9e-053.4e-051.8e-05

1e-062.1e-051e-06

1e-062.1e-051e-06

0.02320.0166280.0162390.0257950.0400750.0226930.017236

1.1e-059e-051.3e-05

1.1e-059e-051.3e-05

7e-063.7e-059e-06

7e-063.7e-059e-06

7e-063.7e-059e-06

4e-065.3e-054e-06

4e-065.3e-054e-06

4e-065.3e-054e-06

0.0086170.005590.0059820.0144530.0130320.0084730.008794

0.000223

8.6e-05

8.6e-05

8.6e-05

0.000137

0.000137

0.000137

0.0086170.005590.0059820.0144530.0128090.0084730.008794

0.0001260.0012240.0001241.5e-05

5.5e-050.000214.9e-05

5.5e-057e-054.9e-05

0.00014

7.1e-050.0010147.5e-051.5e-05

0.000248

1.5e-05

7.1e-050.0007667.5e-05

1.8e-050.001081.9e-05

0.000959

0.000959

1.8e-050.0001211.9e-05

1.8e-050.0001211.9e-05

0.0041150.0022680.0033830.0062230.0018190.0039620.00247

0.0041150.0022680.0033830.0062230.0015950.0039620.00247

8.3e-050.0032120.0015958.3e-05

0.0040320.0022680.0033830.0030110.0038790.00247

0.000224

0.000224

0.0016340.0006090.0003850.0039140.0040910.0016750.002426

0.0016340.0006090.0003850.0039140.0040910.0016750.002426

0.0002144.4e-050.0016480.000227

0.000447

0.0001360.0005560.000149

7.7e-05

0.001303

3e-06

3e-06

4.9e-05

0.0009190.0003380.0020070.0006230.0009275.6e-05

0.000250.0002270.0003850.0019070.000270.0002520.000876

0.0001087e-06

0.000183

0.000105

3e-06

0.0001150.0001510.00012

4.9e-05

0.000365

0.000365

0.000365

0.0011330.0002720.000570.0018070.0027650.0011570.001526

0.0011330.0002720.000570.0018070.0027650.0011570.001526

0.0001370.0002510.000141

0.000235

0.001088

4.8e-050.0005045.3e-05

8.5e-050.000329.1e-05

0.000121

1.5e-05

0.0001120.0002990.000115

8.5e-050.0001138.4e-05

0.000363

0.0006660.0002720.000570.0018070.000680.0006730.000302

4.4e-050.0002194.6e-05

4.4e-050.0002194.6e-05

4.4e-050.0002194.6e-05

1.2e-050.0002271.3e-05

1.2e-050.0002271.3e-05

1.2e-050.0002271.3e-05

0.0015350.0024410.0016440.0025090.0010190.0014770.002357

0.0015350.0024410.0016440.0025090.0007980.0014770.002357

0.0015080.0024410.0016440.0025090.0006360.001450.002357

2.7e-050.0001622.7e-05

0.000221

0.000221

2.7e-050.0001141.9e-05

2.7e-050.0001141.9e-05

2.7e-050.0001141.9e-05

1.9e-057.6e-051.3e-05

1.9e-057.6e-051.3e-05

8e-063.8e-056e-06

8e-063.8e-056e-06

0.0068920.0066030.0041090.0065240.0146650.0067960.006383

0.0068920.0066030.0041090.0065240.0146650.0067960.006383

0.0001350.0001910.000132

0.0001350.0001910.000132

7e-061.8e-057e-06

7e-061e-057e-06

1.6e-052.3e-051.9e-05

1.7e-053.8e-051.3e-05

9e-061.1e-051.2e-05

2.2e-053.3e-052.2e-05

2.6e-051.4e-052.8e-05

3.1e-054.4e-052.4e-05

0.0067210.0066030.0041090.0065240.0142660.0066280.006383

0.000816

0.000816

2.7e-050.0001292.8e-05

2.7e-050.0001292.8e-05

3e-050.000183.7e-05

3e-050.000183.7e-05

0.000169

0.000169

1.4e-050.0001271.7e-05

1.4e-055.8e-051.7e-05

6.9e-05

0.000428

0.000231

7.8e-05

0.000119

1.9e-058.1e-051.7e-05

1.9e-058.1e-051.7e-05

2.7e-050.000122.7e-05

2.7e-050.000122.7e-05

3.9e-050.0001314.1e-05

3.9e-050.0001314.1e-05

3.3e-055.8e-053.9e-05

3.3e-055.8e-053.9e-05

2.4e-050.0001812.4e-05

1.3e-05

2.4e-055.5e-052.4e-05

0.000113

9.5e-050.0001410.000102

3.3e-05

3.7e-054.5e-054.1e-05

5.8e-056.3e-056.1e-05

0.000722

0.000722

8.4e-05

8.4e-05

2.1e-050.0001072.4e-05

2.1e-050.0001072.4e-05

3.1e-050.0001423.6e-05

3.1e-050.0001423.6e-05

0.000213

0.00011

0.000103

0.00017

0.00017

0.000180.0002750.000191

0.000180.0002750.000191

8e-050.0002058.4e-05

8e-050.0002058.4e-05

2.1e-059.8e-052e-05

2.1e-059.8e-052e-05

0.00011

9.1e-05

1.9e-05

0.000142

0.000142

0.0039440.0020330.0018020.0024090.0014590.0038240.001563

1.5e-05

9.1e-05

0.0039080.0020330.0018020.0024090.0006010.0037850.001421

0.000612

3.6e-05

3.6e-050.0001163.9e-05

0.00013

0.00011

0.00011

0.000111

0.000111

8e-060.0001111e-05

8e-060.0001111e-05

0.0017460.004570.0023070.0041150.0009290.0016810.003896

0.0017460.004570.0023070.0041150.0009290.0016810.003896

5.6e-050.0001116.3e-05

5.6e-050.0001116.3e-05

0.0002940.0003980.00033

4.4e-053.1e-055.2e-05

4.3e-059.4e-054.7e-05

4.2e-058.8e-054.8e-05

7.5e-054.2e-058.1e-05

9e-050.0001430.000102

0.0065770.000108

0.000852

0.001613

0.003289

0.000108

0.000823

3.2e-050.0004473.3e-05

0.000233

3.2e-050.0002143.3e-05

1.6e-056.4e-051.7e-05

1.6e-056.4e-051.7e-05

1.6e-056.4e-051.7e-05

8.1e-05

8.1e-05

8.1e-05

2e-056.3e-051.9e-05

2e-056.3e-051.9e-05

2e-056.3e-051.9e-05

0.0046240.0028040.0036170.002710.0063770.0044450.002013

0.0046240.0028040.0036170.002710.0063770.0044450.002013

0.0046240.0028040.0036170.002710.0063770.0044450.002013

9.3e-05

9.3e-05

7.1e-05

7.1e-05

1.6e-056.9e-051.7e-05

1.6e-056.9e-051.7e-05

0.001688

0.000877

0.000811

0.0046080.0028040.0036170.002710.0044560.0044280.002013

0.0046080.0028040.0036170.002710.0044560.0044280.002013

0.0029870.0016310.0025310.0021080.0051830.0029014.6e-05

0.0029870.0016310.0025310.0021080.0051830.0029014.6e-05

1.3e-050.000131e-05

6e-066.4e-055e-06

6e-066.4e-055e-06

7e-066.6e-055e-06

7e-066.6e-055e-06

0.0027550.0015880.0025310.0021080.0032230.002656

3.3e-053.3e-05

3.3e-053.3e-05

0.000271

0.000271

2.1e-055.3e-052e-05

2.1e-055.3e-052e-05

8e-060.0001049e-06

8e-060.0001049e-06

1.6e-059e-051.6e-05

1.6e-059e-051.6e-05

1.3e-058.2e-051.3e-05

1.3e-058.2e-051.3e-05

0.0026640.0015880.0025310.0021080.0026230.002565

0.0026640.0015880.0025310.0021080.0026230.002565

1.6e-050.0001521.8e-05

1.6e-055.7e-051.8e-05

1.6e-055.7e-051.8e-05

4.7e-05

4.7e-05

4.8e-05

4.8e-05

4.7e-05

4.7e-05

4.7e-05

1.3e-053.7e-051.5e-05

1.3e-053.7e-051.5e-05

1.3e-053.7e-051.5e-05

0.000194.3e-050.0012570.0002024.6e-05

8e-066.4e-059e-06

8e-066.4e-059e-06

0.0001714.3e-050.001090.000184.6e-05

0.000766

0.0001714.3e-050.0003240.000184.6e-05

1.1e-055.2e-051.3e-05

1.1e-055.2e-051.3e-05

5.1e-05

5.1e-05

0.000337

7.7e-05

3.8e-05

3.9e-05

0.000178

3e-05

3.7e-05

2.2e-05

3.3e-05

3.3e-05

1.2e-05

1.1e-05

8.2e-05

2.6e-05

2.9e-05

2.7e-05

4.2e-050.0006144.6e-05

4.2e-050.0006144.6e-05

4.2e-050.0006144.6e-05

6.2e-05

6.2e-05

1.3e-050.0001021.4e-05

1.3e-050.0001021.4e-05

1e-057.4e-051e-05

1e-057.4e-051e-05

9e-060.00021.1e-05

4.6e-05

9e-066.9e-051.1e-05

3.8e-05

4.7e-05

1e-050.0001761.1e-05

1e-058.3e-051.1e-05

9.3e-05

0.0020060.002409

0.0020060.002409

0.0020060.002409

0.0020060.002409

0.0020060.002409

0.0020060.002409

2.3e-050.0002121.4e-05

2.3e-050.0002121.4e-05

2.3e-050.0002121.4e-05

2.3e-050.0002121.4e-05

4e-063.3e-052e-06

4e-063.3e-052e-06

8e-064e-055e-06

8e-064e-055e-06

4e-063.6e-052e-06

4e-063.6e-052e-06

5.3e-05

5.3e-05

7e-065e-055e-06

7e-065e-055e-06

1.7e-055.3e-051.3e-05

1.7e-055.3e-051.3e-05

1.7e-055.3e-051.3e-05

1.7e-055.3e-051.3e-05

1.7e-055.3e-051.3e-05

1.7e-055.3e-051.3e-05

6e-05

6e-05

6e-05

6e-05

6e-05

6e-05

0.0077550.0073510.0495340.0067250.0483330.0078210.005325

0.0077210.0073510.0495340.0067250.04820.0077890.005325

0.000130.0004250.000137

0.000130.0004250.000137

0.000130.0004250.000137

0.000130.0004250.000137

0.0001930.0002770.000201

0.0001040.000130.000105

0.0001040.000130.000105

0.0001040.000130.000105

8.9e-050.0001479.6e-05

8.9e-050.0001479.6e-05

8.9e-050.0001479.6e-05

0.0002280.0007820.000233

5.9e-050.0001125.5e-05

3.1e-054.8e-052.4e-05

3.1e-054.8e-052.4e-05

2.8e-056.4e-053.1e-05

2.8e-056.4e-053.1e-05

2.2e-054.4e-052.4e-05

2.2e-054.4e-052.4e-05

2.2e-054.4e-052.4e-05

3.7e-052.5e-054.1e-05

3.7e-052.5e-054.1e-05

3.7e-051.4e-054.1e-05

1.1e-05

0.000110.0006010.000113

0.000110.0005210.000113

0.000110.0005210.000113

8e-05

4.1e-05

3.9e-05

3.3e-05

3.3e-05

3.3e-05

3.3e-05

0.0004210.0011330.000457

9.8e-050.0002230.000104

1.9e-055.1e-052.1e-05

1.9e-055.1e-052.1e-05

2.8e-053.1e-05

2.8e-053.1e-05

5.1e-050.0001725.2e-05

5.1e-050.0001725.2e-05

0.0001070.0002250.000124

0.0001070.0002250.000124

0.0001070.0002250.000124

8.2e-050.0002218.2e-05

8.2e-050.0002218.2e-05

5e-050.0001285e-05

3.2e-059.3e-053.2e-05

0.0001140.000330.000129

0.0001140.000330.000129

7e-065e-068e-06

2.4e-057.9e-052.8e-05

2.7e-057e-052.9e-05

3.7e-055.5e-054e-05

3e-061.1e-055e-06

1.6e-050.000111.9e-05

2e-050.0001341.8e-05

2e-050.0001341.8e-05

2e-050.0001341.8e-05

0.0034370.0068750.0036310.0067250.0035110.0033280.005267

0.0002720.0006680.0002651.5e-05

2.7e-057.2e-052.9e-05

2.7e-057.2e-052.9e-05

0.0001720.0004680.0001791.5e-05

0

2.5e-058e-062.5e-05

2e-06

1.7e-057.1e-052e-05

3e-06

4e-06

5e-061e-055e-06

2e-05

1.5e-05

9e-06

1.8e-05

4e-063.3e-054e-06

1.9e-057.1e-052e-05

2.4e-059.4e-052.6e-05

1.2e-055.8e-051e-05

3e-06

1.3e-051.5e-051.2e-05

1.9e-051e-061.9e-05

6e-061e-059e-06

7e-06

1.1e-051.8e-051e-05

1.3e-05

1.7e-0501.9e-05

3e-054.9e-051.8e-05

3e-054.9e-051.8e-05

4.3e-057.9e-053.9e-05

1.1e-051e-05

3.2e-057.9e-052.9e-05

0.00120.0005520.0008640.0018070.0012160.0011770.000329

0.00120.0005520.0008640.0018070.0012160.0011770.000329

0.00120.0005520.0008640.0018070.0012160.0011770.000329

0.0018840.0063230.0027670.0049180.0012610.001810.004923

0.0018840.0063230.0027670.0049180.0012610.001810.004923

4.5e-05

0.0018840.0063230.0027670.0049180.0012160.001810.004923

6e-061.5e-057e-06

6e-061.5e-057e-06

4e-06

2e-06

6e-069e-067e-06

1.6e-050.0002851.5e-05

1.6e-050.0002851.5e-05

0.000138

1.6e-054.8e-051.5e-05

9.9e-05

4.5e-054.4e-05

4.5e-054.4e-05

4.5e-054.4e-05

1.4e-056.6e-051e-05

1.4e-056.6e-051e-05

1.4e-056.6e-051e-05

0.0033120.0004760.0459030.0420390.0034335.8e-05

0.0459030.040299

0.0459030.040299

0.0459030.040299

1.5e-05

1.5e-05

1.5e-05

0.0007663.3e-050.0006430.000816

0.0005633.3e-050.0005040.000604

0.0001860.0001520.000197

0.0003773.3e-050.0001740.000407

0.000178

0.0002030.0001390.000212

0.0002030.0001390.000212

0.0025460.0004430.0010970.0026174.3e-05

0.0003093.4e-050.00035

0.0003093.4e-050.00035

0.0001845.7e-050.000202

0.0001845.7e-050.000202

0.0013860.0002710.0007370.0015231.4e-05

0.0003465.5e-050.0001620.0003671.4e-05

0.0003537.5e-050.000140.000387

0.0003958.5e-050.0001830.000436

0.0002925.6e-050.0001220.000333

0.00013

0.0006670.0001380.0003030.0005422.9e-05

0.0001768.4e-0502.9e-05

7.8e-05

0.000295.4e-050.0001690.00032

0.0002015.6e-050.000222

3.4e-050.0001333.2e-05

3.4e-050.0001333.2e-05

3.4e-050.0001333.2e-05

3.4e-050.0001333.2e-05

1.8e-057e-051.7e-05

1.6e-056.3e-051.5e-05

2.4e-050.0001562.8e-05

2.4e-050.0001562.8e-05

2.4e-050.0001562.8e-05

2.4e-050.0001562.8e-05

1e-054e-051.7e-05

1e-054e-051.7e-05

5e-064.1e-052e-06

5e-064.1e-052e-06

4e-063.6e-053e-06

4e-063.6e-053e-06

5e-063.9e-056e-06

5e-063.9e-056e-06

0.262860.2165390.1919320.2071680.2254520.263450.193459

0.2622470.2165050.1919320.2071680.2240560.2629050.193427

0.0042860.0020680.0029890.0023090.0039850.0041410.002056

0.0001030.0001130.0001023.1e-05

0.0001030.0001130.0001023.1e-05

0.0001030.0001130.0001023.1e-05

0.0039260.0020110.0029890.0023090.0033370.0037770.001964

4.9e-057.7e-054.2e-05

4.9e-057.7e-054.2e-05

0.0038770.0020110.0029890.0023090.003260.0037350.001964

0.0038240.0020110.0029890.0023090.0031850.0036840.001964

5.3e-057.5e-055.1e-05

0.0002575.7e-050.0005350.0002626.1e-05

0.0002575.7e-050.0005350.0002626.1e-05

0.0002575.7e-050.0005350.0002626.1e-05

0.0001780.0003370.000173.1e-05

0.0001780.0003370.000173.1e-05

0.0001780.0002810.000173.1e-05

0.0001780.0002810.000173.1e-05

5.6e-05

5.6e-05

0.0003140.0004690.0002934.6e-05

0.0001820.0001620.000169

4.4e-052e-054.3e-05

4.4e-052e-054.3e-05

4.2e-055.8e-053.6e-05

4.2e-055.8e-053.6e-05

2.9e-054e-052.4e-05

2.9e-054e-052.4e-05

3.8e-053e-053.8e-05

3.8e-052e-053.8e-05

1e-05

2.9e-051.4e-052.8e-05

2.7e-051.2e-052.6e-05

2e-062e-062e-06

0.0001320.0003070.0001244.6e-05

6.4e-05

6.4e-05

6.1e-056.4e-055.7e-054.6e-05

6.1e-056.4e-055.7e-054.6e-05

7.1e-050.0001176.7e-05

5.6e-05

7.1e-056.1e-056.7e-05

6.2e-05

6.2e-05

5.6e-056.8e-054.9e-051.5e-05

5.6e-056.8e-054.9e-051.5e-05

5.6e-056.8e-054.9e-051.5e-05

5.6e-056.8e-054.9e-051.5e-05

0.0001990.0001930.0001964.5e-05

0.0001990.0001930.0001964.5e-05

6.8e-056.3e-056.5e-054.5e-05

6.8e-056.3e-056.5e-054.5e-05

6.8e-057e-056.7e-05

6.8e-057e-056.7e-05

6.3e-056e-056.4e-05

6.3e-056e-056.4e-05

5e-056.6e-054.6e-05

5e-056.6e-054.6e-05

5e-056.6e-054.6e-05

5e-056.6e-054.6e-05

7.5e-057.9e-056.9e-05

7.5e-057.9e-056.9e-05

7.5e-057.9e-056.9e-05

7.5e-057.9e-056.9e-05

2.1e-056.1e-051.7e-05

2.1e-056.1e-051.7e-05

2.1e-056.1e-051.7e-05

2.1e-056.1e-051.7e-05

0.1808320.1062030.1252350.0673490.158380.1738320.082045

0.1808320.1062030.1252350.0673490.158380.1738320.082045

0.0395060.0222070.0265280.0153570.0311690.0380340.018335

0.0381250.0217120.0256570.0128480.0298560.0366680.018024

0.0013810.0004950.0008710.0025090.0013130.0013660.000311

4.5e-056.5e-054.7e-053.2e-05

2.9e-053.8e-053e-058e-06

1.6e-052.7e-051.7e-058e-06

1.6e-05

9.8e-050.0001849.4e-053.1e-05

9.8e-050.0001849.4e-053.1e-05

0.1411830.0839960.0987070.0519920.1269620.1356570.063647

4.8e-050.0003634.8e-05

5.1e-050.0001614.9e-05

4.9e-050.0001474.9e-05

0.1410350.0839960.0987070.0519920.1262910.1355110.063647

0.0020310.0010740.0015550.0019070.0018970.0019530.000847

0.0020310.0010740.0015550.0019070.0018970.0019530.000847

0.0020310.0010740.0015550.0019070.0018970.0019530.000847

2.8e-055.9e-052.6e-051.4e-05

3.2e-057.1e-053.3e-05

4.5e-056.8e-054.5e-051.5e-05

4.8e-055.9e-054.4e-051.4e-05

0.0018780.0010740.0015550.0019070.001640.0018050.000804

4.6e-055.9e-054.3e-05

4.6e-055.9e-054.3e-05

4.6e-055.9e-054.3e-05

4.6e-055.9e-054.3e-05

0.0109020.0063270.0078180.0083310.0112920.0104560.006877

0.0004590.001339

0.0004590.001339

0.001309

0.0004593e-05

6.7e-050.0002096.3e-051.4e-05

7.5e-05

7.5e-05

6.7e-056.8e-056.3e-051.4e-05

6.7e-056.8e-056.3e-051.4e-05

6.6e-05

6.6e-05

6e-050.0001055.7e-05

6e-056e-055.7e-05

6e-056e-055.7e-05

4.5e-05

4.5e-05

3e-061.2e-052e-06

3e-061.2e-052e-06

3e-061.2e-052e-06

6.7e-058.1e-056.4e-05

6.7e-058.1e-056.4e-05

6.7e-058.1e-056.4e-05

8.6e-050.0002268.4e-050.000727

7.2e-05

7.2e-05

6.8e-05

6.8e-05

0.000727

0.000727

8.6e-058.6e-058.4e-05

8.6e-058.6e-058.4e-05

1.3e-055.2e-051e-05

1.3e-055.2e-051e-05

1.3e-055.2e-051e-05

8.9e-058e-058.8e-051.5e-05

8.9e-058e-058.8e-051.5e-05

8.9e-058e-058.8e-051.5e-05

0.0016330.0013340.0013920.0021080.0018580.0015810.001231

4.6e-05

4.6e-05

5.9e-052.8e-055.7e-05

5.9e-052.8e-055.7e-05

0.0013910.0013340.0013920.0021080.0007110.0013360.001231

0.0013910.0013340.0013920.0021080.0007110.0013360.001231

5.7e-05

5.7e-05

4e-05

4e-05

4.7e-05

4.7e-05

0.0001830.0007470.000188

7.4e-05

0.000181

0.000167

8e-05

5.9e-05

0.0001830.0001860.000188

6.2e-05

4.8e-05

1.4e-05

5.9e-05

5.9e-05

6.1e-05

6.1e-05

0.005050.002840.0036570.002610.0047080.0048530.002002

0.0001048.8e-059.7e-05

0.0001048.8e-059.7e-05

0.0049460.002840.0036570.002610.004620.0047560.002002

0.0049460.002840.0036570.002610.004510.0047560.002002

0.00011

0.0035520.0021530.0027690.0036130.0032810.0033840.001481

7.9e-056.4e-057.3e-051.6e-05

7.9e-056.4e-057.3e-051.6e-05

7e-068e-067e-06

7e-068e-067e-06

9.1e-058.3e-058.1e-05

2.3e-052.5e-052.3e-05

6.8e-055.8e-055.8e-05

3.8e-052.5e-053.3e-05

3.8e-052.5e-053.3e-05

7.3e-050.000185.8e-05

7.3e-055.3e-055.8e-05

5.7e-05

7e-05

0.0019150.0013850.0016390.0019070.001530.001840.000831

0.0019150.0013850.0016390.0019070.001530.001840.000831

0.0012790.0007680.001130.0017060.0012470.0012290.000634

0.0012760.0007680.001130.0017060.0010140.0012260.000634

6.3e-05

2e-05

2.3e-05

2.4e-05

6e-06

3e-067e-063e-06

2.4e-05

3.3e-05

6e-06

2.7e-05

7e-058e-056.3e-05

3.7e-052.4e-053.3e-05

2.8e-05

3.3e-052.8e-053e-05

6.4e-05

6.4e-05

0.0002820.0002210.000276.8e-05

0.0002820.0002210.000276.8e-05

9.2e-057.8e-058.8e-057e-06

0.0001058.1e-050.0001014.5e-05

8.5e-056.2e-058.1e-051.6e-05

0.0057610.0065360.0049120.0057210.0041330.0055540.0036

0.0057610.0065360.0049120.0057210.0041330.0055540.0036

7.6e-05

7.6e-05

8.7e-05

8.7e-05

6.6e-05

6.6e-05

8.3e-057e-058.3e-05

8.3e-057e-058.3e-05

0.0051380.0064860.0049120.0057210.0029710.0049470.003555

0.0020690.0047770.0030220.0036130.0019670.0019910.003555

0.00018

4.8e-05

0.0030690.0017090.001890.0021080.0007760.002956

0.0001260.0001253e-05

0.0001260.0001253e-05

7.7e-05

7.7e-05

0.0001030.000229.9e-05

3.2e-05

5.4e-05

4e-06

0

3.5e-05

0.0001039.4e-059.9e-05

1e-06

0.0002295e-050.0004880.000222

0.0002295e-050.0004880.000222

8.2e-057.8e-057.8e-051.5e-05

8.2e-057.8e-057.8e-051.5e-05

0.0124630.0072260.0092850.0057210.0123460.011960.005688

0.0124630.0072260.0092850.0057210.0123460.011960.005688

2.5e-054.5e-051.2e-05

2.5e-054.5e-051.2e-05

0.0001587e-06

3.6e-05

3.8e-05

3.8e-05

4.6e-05

7e-06

0.0124380.0072260.0092850.0057210.0120780.0119480.005681

0.0124380.0072260.0092850.0057210.0120780.0119480.005681

5.1e-05

5.1e-05

1.4e-05

1.4e-05

0.0055510.0028970.0031240.0024090.0059110.005440.003512

0.0055510.0028970.0031240.0024090.0059110.005440.003512

7.5e-057e-056.8e-051.6e-05

7.5e-057e-056.8e-051.6e-05

0.0054760.0028970.0031240.0024090.0058410.0053720.003496

5.6e-05

1e-058e-061.2e-05

2.4e-05

9e-055.1e-059.7e-05

6.6e-05

4.5e-05

4.4e-05

0.0001384.4e-057.9e-050.000157

1.1e-05

0.0001312.5e-052.2e-050.0001421.5e-05

1.2e-05

3.7e-05

7e-05

0.000877

1e-06

5.7e-05

2.4e-05

6e-06

2e-05

6.4e-058.2e-053e-050.0001415e-06

1e-06

9.4e-05

2.6e-05

7e-061.1e-058e-06

8e-061e-06

5.1e-051.6e-054e-055.5e-05

0.0040780.0024820.0031240.0024090.0032910.0039280.001767

0.0001244.1e-050.000138

6.7e-05

0.0007070.000376

1.5e-05

4.2e-05

6.7e-05

0.000134.5e-054.3e-050.000145

0.0001434.5e-057.5e-050

2.5e-05

5.7e-05

0.0001232.5e-052.2e-050.000132

3e-05

4.7e-05

4e-06

8.7e-05

0.0001657.2e-051.2e-050.0001841e-06

0.000404

7e-05

7.3e-052.5e-057.9e-05

4.1e-05

6e-06

7.8e-052e-053.1e-058e-05

8e-05

1.9e-05

5.6e-05

7.1e-057.3e-057.4e-05

2.4e-05

3.2e-05

0.0277820.0398210.0221140.0304130.0173290.0337470.041015

0.0202640.0284030.0103010.0166620.0048980.0262650.020621

0.0202210.0284030.0103010.0166620.0047570.0262270.020621

5e-067e-064e-06

5e-065e-065e-06

6e-062e-065e-06

0.00012e-06

2e-064e-062e-06

09.5e-05002e-06

0

3e-063.7e-051e-062e-06

0.0147370.0229030.0056030.0115430.0010990.0182530.017457

3.5e-05

2.8e-05

6.8e-050

3e-051.7e-053.3e-05

5e-06

1.9e-05

4.9e-053.1e-055.4e-05

4.4e-05

4.2e-052.1e-054.4e-058e-06

1e-059.5e-056e-061.1e-052e-06

0.00210.0034090.0020390.0030110.0005820.0047490.003118

3.6e-05

4.5e-057e-064.5e-051.5e-05

0001.7e-05

1e-05

6.2e-052.6e-056.5e-05

4.5e-05

4.1e-053.5e-054.2e-05

4e-06

5e-069e-066e-06

3.2e-05

4.2e-05

3.2e-05

6.6e-055.5e-057.2e-05

5.7e-05

0

2e-061e-062e-06

0

3e-062e-063e-06

0.002940.0017640.0026590.0021080.0024580.00283

4.3e-050.0001413.8e-05

4.3e-050.0001413.8e-05

0.0004183e-050.0021080.001420.0004136.2e-05

0.0004183e-050.0021080.001420.0004136.2e-05

0.000143e-050.0021085.8e-050.000137

1.8e-05

0.000891

0.0001350.0002140.0001372.9e-05

0.0001430.0001950.0001391.5e-05

6.2e-05

0.0001180.0001350.0001091.5e-05

0.0001180.0001350.0001091.5e-05

0.0001180.0001350.0001091.5e-05

0.004180.0096390.0110030.0095350.0087620.0041820.018674

0.004180.0096390.0110030.0095350.0087620.0041820.018674

1e-051e-051.3e-051.5e-05

6.4e-054.6e-056.2e-05

0.000131

0.000148

7.4e-059e-057.6e-05

3e-06

7.9e-05

0.000771

0.000109

0.0001560.0003690.0001020.000166

6.3e-056.2e-056.4e-05

3.2e-057.7e-050.002712.1e-053.2e-05

0.0002330.0001450.000235

9e-05

0.0014790.0083240.0015260.0068250.0003650.0014210.01112

1.4e-05

0.000281

0.0003380.0002040.0002170.000331

0.000351

8e-055.3e-058.3e-051.3e-05

0.0011570.0005780.0009970.0008790.0011961.1e-05

1e-06

6.1e-05

3.2e-05

0.000180.0001650.00019

0.000271

0.001682

0.0001197.4e-050.000119

4.4e-05

0.000364

0.000135

0.000128

7.4e-057.8e-057.3e-05

0.000136

8.3e-05

0.000154

1.2e-052.4e-052e-061.3e-05

0.008480.007445

7.6e-05

0.0001096.3e-055.8e-050.0001085.2e-05

0.0002

0.000149

0.00092

0.0027440.0017490.000810.0021080.0020120.0027270.001643

0.0006190.0001040.0007510.0006258e-05

0.0002647.4e-050.0004050.000272

0.000113

0.0001580.0001470.0001581.5e-05

0.0001973e-058.6e-050.0001956.5e-05

0.0021250.0016450.000810.0021080.0012610.0021020.001563

1e-05

0.0003510.0001180.0003390.0003640.000112

0.0002669.2e-052.7e-050.000267

0.000163

0.0012430.0013220.000810.0021080.0002060.0011990.001377

2.6e-05

5.8e-05

0.000259

4e-05

1.9e-05

0.0001125.2e-052.8e-050.0001177.4e-05

0.0001536.1e-056.2e-050.000155

2.4e-05

5.8e-056.9e-055.1e-05

5.8e-056.9e-055.1e-05

5.8e-056.9e-055.1e-05

3.3e-05

3.3e-05

3.3e-05

0.01170.0443530.01490.0830080.0074510.0149390.04765

0.01170.0443530.01490.0830080.0074510.0149390.04765

0.0016450.000736

0.0016450.000736

7.3e-05

7.3e-05

0.0023760.0023390.0025290.002610.0016410.0022980.002348

0.0023760.0023390.0025290.002610.0016410.0022980.002348

0.0093240.0420140.0123710.0803980.0040920.0126410.044566

2e-062e-064e-06

6.1e-05

0.0086380.0414020.0123710.0361340.0035870.0119720.04435

3.2e-05

1.1e-05

2.4e-05

0.0003990.0004720.0442640.0001580.000381

1.6e-051.4e-051.7e-05

1.8e-05

2e-06

2.9e-059e-062.7e-05

3.3e-054.3e-053.4e-05

0.0001720.000148e-050.0001680.000216

3.5e-053.3e-053.8e-05

4e-06

1.2e-05

2e-06

0.0002050.0006210.000169

9.9e-050.0003118.1e-05

1.7e-050.0001331.2e-05

4.9e-05

4.9e-05

1.7e-058.4e-051.2e-05

1.7e-058.4e-051.2e-05

8.2e-050.0001786.9e-05

3e-050.0001112.7e-05

3e-055.6e-052.7e-05

5.5e-05

5.2e-056.7e-054.2e-05

5.2e-056.7e-054.2e-05

0.0001060.000318.8e-05

0.0001060.000318.8e-05

8e-063.5e-053e-06

8e-063.5e-053e-06

5.3e-05

5.3e-05

3.4e-050.000113e-05

2e-054.9e-051.8e-05

1.4e-056.1e-051.2e-05

3e-054.1e-052.6e-05

3e-054.1e-052.6e-05

1.9e-057.1e-051.8e-05

1.9e-057.1e-051.8e-05

1.5e-051.1e-05

1.5e-051.1e-05

5.5e-059.8e-054e-05

5.5e-059.8e-054e-05

5.5e-059.8e-054e-05

3e-055.2e-052.1e-05

3e-055.2e-052.1e-05

2.5e-054.6e-051.9e-05

2.5e-054.6e-051.9e-05

0.0003033.4e-050.0005420.0002973.2e-05

0.0003033.4e-050.0005420.0002973.2e-05

0.0003033.4e-050.0005420.0002973.2e-05

0.0003033.4e-050.0005420.0002973.2e-05

0.0003033.4e-050.0005420.0002973.2e-05

5e-050.0001353.9e-05

5e-050.0001353.9e-05

5e-050.0001353.9e-05

5e-050.0001353.9e-05

6e-05

5e-057.5e-053.9e-05

0.0049850.002570.0037440.002710.005110.0048050.002226

1.8e-055.4e-051.2e-05

1.8e-055.4e-051.2e-05

1.8e-055.4e-051.2e-05

1.8e-055.4e-051.2e-05

1.8e-055.4e-051.2e-05

0.0049670.002570.0037440.002710.0050560.0047930.002226

0.0049670.002570.0037440.002710.0050560.0047930.002226

0.0049570.002570.0037440.002710.0049990.0047830.002226

7e-064.9e-056e-06

7e-064.9e-056e-06

5e-062.8e-054e-06

5e-062.8e-054e-06

0.0049430.002570.0037440.002710.004850.004770.002226

0.0049430.002570.0037440.002710.004850.004770.002226

2e-063.1e-053e-06

2e-063.1e-053e-06

4.1e-05

4.1e-05

1e-055.7e-051e-05

3e-062.9e-053e-06

3e-062.9e-053e-06

7e-062.8e-057e-06

7e-062.8e-057e-06

7.7e-050.0007026.6e-05

8.6e-05

8.6e-05

8.6e-05

8.6e-05

8.6e-05

2e-050.0001791.6e-05

1.4e-050.0001341.1e-05

1.4e-050.0001341.1e-05

1.4e-055.2e-051.1e-05

1.4e-055.2e-051.1e-05

4.7e-05

4.7e-05

3.5e-05

3.5e-05

6e-064.5e-055e-06

6e-064.5e-055e-06

6e-064.5e-055e-06

6e-064.5e-055e-06

2.7e-053.9e-051.9e-05

2.7e-053.9e-051.9e-05

2.7e-053.9e-051.9e-05

2.7e-053.9e-051.9e-05

2.7e-053.9e-051.9e-05

3.2e-05

3.2e-05

3.2e-05

3.2e-05

3.2e-05

3e-050.0003663.1e-05

3e-050.0003663.1e-05

3e-050.0003663.1e-05

3e-050.0003663.1e-05

0.000188

3e-050.0001783.1e-05

3e-064.1e-052e-06

4.1e-05

4.1e-05

4.1e-05

4.1e-05

4.1e-05

3e-062e-06

3e-062e-06

3e-062e-06

3e-062e-06

3e-062e-06

2e-063.5e-050

2e-063.5e-050

2e-063.5e-050

2e-063.5e-050

2e-063.5e-050

2e-063.5e-050

0.0095430.0244170.0150190.029910.0064470.009310.013298

0.0095430.0244170.0150190.029910.0064470.009310.013298

0.0095220.0244170.0150190.029910.0062930.0092860.013298

1.7e-058.3e-051.5e-05

1.1e-054.4e-051.1e-05

3e-062.6e-052e-06

8e-061.8e-059e-06

6e-063.9e-054e-06

2e-068e-060

8e-06

4e-062.3e-054e-06

0.0095050.0244170.0150190.029910.006210.0092710.013298

0.002870.0106030.0027430.0043160.0011370.0027930.00352

6e-067e-066e-06

1e-050.0051525.2e-059e-060.00352

0.0028520.0054510.0027430.0043160.0010530.002775

2e-062.5e-053e-06

0.0066350.0138140.0122760.0255940.0050730.0064780.009778

2.1e-05

1.1e-05

6e-06

0.0056290.004942

0.0003530.0001230.0079294e-060.000352

0.0008060.0010350.0038142.1e-050.0007980.000318

0.0007660.0010980.0009390.0033121.1e-050.0007540.000451

0.0013970.0012290.0048189e-060.0013590.000184

7e-06

0.0033130.0103290.0057080.0057214.1e-050.0032150.008825

1.8e-058.7e-052.2e-05

1.8e-058.7e-052.2e-05

1.3e-052.6e-051.3e-05

1.3e-052.6e-051.3e-05

5e-063.3e-059e-06

5e-063.3e-059e-06

2.8e-05

2.8e-05

3e-066.7e-052e-06

3e-066.7e-052e-06

2e-063.9e-051e-06

2e-062e-051e-06

1.9e-05

1e-062.8e-051e-06

1e-062.8e-051e-06

0.4067040.4148750.4346240.3673620.4278160.4222810.3772

0.0038830.002120.0028790.008030.0037540.0036760.001646

1e-054.6e-059e-06

1e-054.6e-059e-06

2e-062.7e-054e-06

2e-062.7e-054e-06

8e-061.9e-055e-06

8e-061.1e-055e-06

8e-06

0.0038640.002120.0028790.008030.0036710.003660.001646

1.6e-053.2e-051.4e-05

1.6e-053.2e-051.4e-05

1.6e-053.2e-051.4e-05

0.0001190.0056210.0003157.4e-05

5.6e-055.8e-052.7e-05

1.2e-052.1e-051.2e-05

3.7e-052.8e-058e-06

7e-069e-067e-06

5.5e-050.0056210.0002064.2e-05

1.7e-05

3e-06

2.6e-053e-062.6e-05

1e-060.0034131e-061e-06

2e-061.4e-05

4e-06

1.1e-05

8e-06

1e-05

5.1e-05

9e-062.9e-057e-06

8e-061.6e-05

2e-064e-062e-06

7e-060.0022082e-066e-06

2e-05

1.3e-05

8e-065.1e-055e-06

1e-05

1e-05

5e-06

5e-06

1e-068e-061e-06

7e-061.3e-054e-06

0.0037290.002120.0028790.0024090.0033240.0035720.001646

1.4e-052e-057e-06

1.4e-052e-057e-06

0.0036890.002120.0028790.0024090.0032360.0035430.001646

3e-061.6e-052e-06

9e-069e-06

2e-061.1e-052e-06

2.9e-05

000

7e-061e-066e-06

2.3e-05

1e-068e-062e-06

0.0036660.002120.0028790.0024090.0030970.0035220.001646

2.5e-05

1e-062.6e-050

3e-062.6e-053e-06

3e-062.6e-053e-06

2.3e-054.2e-051.9e-05

1.7e-052e-051.3e-05

6e-062.2e-056e-06

9e-063.7e-057e-06

9e-063.7e-057e-06

9e-063.7e-057e-06

9e-063.7e-057e-06

0.1328140.1625810.1833960.1890020.1824730.1588390.187268

6.9e-05

6.9e-05

6.9e-05

6.9e-05

1.8e-057.1e-051.5e-051.5e-05

1.8e-057.1e-051.5e-051.5e-05

1.8e-057.1e-051.5e-05

1.8e-057.1e-051.5e-05

1.5e-05

1.5e-05

0.000113

0.000113

6.7e-05

6.7e-05

4.6e-05

4.6e-05

0.0419650.03820.0652920.0682550.0537190.0527670.041553

0.0001820.0002380.000158

9.6e-05

9.6e-05

0.0001826.2e-050.000158

9e-066e-067e-06

0.0001613e-050.000141

5e-06

1e-06

1.2e-052e-051e-05

8e-05

8e-05

0.0149510.0171590.0083390.0246940.0067970.0191870.020621

4e-054.1e-056.9e-05

4e-054.1e-056.9e-05

4.2e-053.4e-054.6e-05

4.2e-053.4e-054.6e-05

2.9e-05

2.9e-05

0.0067040.0043470.0045780.0031120.0036080.0064790.003315

0.0003453.6e-054.9e-050.0003581.9e-05

0.0063590.0043110.0045780.0031120.0033940.0061210.003084

6.9e-05

6.2e-05

0.000143

6.4e-05

3.9e-05

7.8e-05

5.8e-05

2e-05

0.000161.6e-058.1e-050.0001674.9e-05

9.1e-051.6e-056e-069.2e-052.6e-05

1.3e-05

1.9e-05

6.9e-052e-057.5e-052.3e-05

4e-06

1.9e-05

0

0.0002480.0005440.0004440.0023090.0002650.0049140.002259

0.0001311e-050.000118

0.0001170.0005440.0004440.0023090.0002550.0047960.002259

8.1e-05

8.1e-05

7.4e-05

7.4e-05

9.2e-05

9.2e-05

8.7e-05

8.7e-05

0.0006150.0001960.0005641.5e-05

7.3e-056e-054.9e-05

0.0002875.3e-050.000308

4.5e-055.3e-052.1e-05

0.000213e-050.000186

1.5e-05

8.9e-05

8.9e-05

0.0003030.0001270.000311

0.0003030.0001270.000311

0.0002022.5e-050.000211

0.0002022.5e-050.000211

4.5e-050.0002124.6e-05

2.2e-054.3e-052.5e-05

5.9e-05

6e-062e-066e-06

8e-062e-068e-06

9e-065.5e-057e-06

5.1e-05

4.1e-05

4.1e-05

7.4e-05

7.4e-05

5.3e-05

5.3e-05

0.0031720.0011220.0014550.0019070.0008280.0030318.4e-05

0.0003265e-050.000303

2e-06

1e-06

9e-06

0.0001842.2e-057e-060.0001625e-06

7e-06

0.0002851.3e-050.0002893.1e-05

3e-06

7e-06

1.2e-05

4.2e-05

1e-06

0.0002281.5e-050.000226

0.0021490.00110.0014550.0019070.0006540.002051

5e-06

4.8e-05

6e-05

6e-05

0.0011590.0040960.0018620.0031120.0001530.0011590.003767

9.3e-05

0.0001371.6e-051.2e-050.000133

2e-06

1e-06

3e-052e-062.7e-05

0.0001121.3e-058e-068.1e-053e-06

0.000880.0040670.0018620.0031123.5e-050.0009180.003764

7.3e-05

7.3e-05

0.0002940.0009770.002617.2e-050.0002840.003335

9.6e-050.0009770.002611.1e-059.3e-050.003111

2.2e-05

0.0001983.9e-050.0001910.000224

4.8e-054.8e-05

4.8e-054.8e-05

0.0019190.0060570.0116440.0003240.0018580.007797

0.0001440.0001330.0018071.5e-050.0001450.000616

0.001280.0052650.0045170.0001960.001264

0.0003870.0006370.002615.6e-050.0003930.005442

0.0001082.2e-050.002715.7e-055.6e-050.001739

0.0028050.0015830.002710.0017580.0030640.001182

0.0001220.0001259.3e-05

5.9e-05

0.0001223.5e-059.3e-05

3.1e-05

0.0004851.3e-050.000120.0004177.4e-05

2.2e-05

0.0002717.6e-050.000223

0.0002141.3e-052.2e-050.0001947.4e-05

0.0021980.001570.002710.0015130.0025540.001108

0.0003642.6e-050.000367

0.0006326.4e-059.4e-050.0009936.9e-05

0.0013690.000776

0.0008260.0001561.7e-050.0008190.000263

0.0003760.001350.002717e-060.000375

8.1e-05

8.1e-05

8.1e-05

0.0209140.0192190.0564880.0392450.0428830.0275680.019165

0.001053

0.001053

0.0002390.0001284.4e-050.000230.000116

0.0002136e-051e-050.0001990.000116

2.6e-056.8e-053.4e-053.1e-05

0.0192950.0188560.0564880.0392450.0410790.0260060.018817

0.001279

0.0097960.0086

0.001084

7.2e-05

6e-050.0044720.0078310.0148551.3e-056e-059.3e-05

0.0056640.004973

0.018050.0136510.0053110.018970.001020.0247450.011766

0.0011850.0007330.0006850.005420.0001510.0012010.006958

0.017250.015144

0.0099510.008736

7e-06

0.001380.0002350.0007070.0013320.000232

0.0003772.7e-053.5e-050.000323

9.5e-057e-052.9e-050.0001056.8e-05

3e-05

1e-05

1.7e-05

9e-06

7.7e-05

0.000412

03.3e-05

4e-05

0.0004315.5e-050.0001150.0004632.1e-05

0.0004774.3e-055e-050.000441

3.3e-05

0.000154

0.000154

0.000154

0.0011670.0005110.0010453e-05

9.7e-05

9.7e-05

0.0001782.7e-050.000161

0.0001782.7e-050.000161

0.0003550.0001340.0003171.5e-05

3.7e-05

0.0001384.6e-050.0001341.5e-05

0.0002175.1e-050.000183

0.00026.1e-050.000161

0.00026.1e-050.000161

9.5e-058e-068.4e-05

9.5e-058e-068.4e-05

0

0.0003390.0001840.0003221.5e-05

2.4e-05

4.1e-05

3.6e-05

0.000154.8e-050.000146

0.0001893.5e-050.0001761.5e-05

0.0019460.0002390.0004650.0016060.0012970.0017450.000555

2.1e-052.8e-052.4e-05

2.1e-052.8e-052.4e-05

0.000710.0003470.0006090.000308

0.0002194.7e-050.0001686e-06

0.000206

0.0003418.3e-050.0002840.00028

2.7e-056e-063.1e-051.5e-05

4.4e-055e-064.9e-057e-06

7.9e-0507.7e-05

2e-054.6e-052.4e-05

2e-054.6e-052.4e-05

0.0011950.0002390.0004650.0016060.0008760.0010880.000247

2.2e-05

7.6e-051e-052.4e-056.3e-054.6e-05

5.4e-05

0.0002281.2e-050.0001831e-05

0.0001262.9e-050.0001

8e-05

5.4e-05

0.0007650.0002290.0004650.0016060.0006010.0007420.000185

6e-06

0.0009550.0039720.0008390.0037140.0003660.0008530.004239

2e-065.4e-051e-06

1.6e-05

1.6e-05

2e-063.8e-051e-06

2.9e-05

2e-069e-061e-06

0.0009530.0039720.0008390.0037140.0003120.0008520.004239

3.2e-05

3.2e-05

0.0009530.0039720.0008390.0037140.000280.0008520.004239

2.7e-05

1.7e-05

9e-062.1e-051e-05

0.0009440.0039720.0008390.0037140.0001940.0008420.004239

2.1e-05

0.0178640.0067340.0155320.0056210.0231650.0173530.004152

5.7e-05

5.7e-05

5.7e-05

5.4e-05

5.4e-05

5.4e-05

0.0175280.0067340.0155320.0056210.0226380.0170550.004152

0.0171090.0067340.0155320.0056210.0218630.0166770.004137

0.0003440.000196

0.0064990.005706

0.0163840.0067340.0090330.0056210.015580.0162790.004106

0.0003810.0001650.0003983.1e-05

0.000216

0.0001410.000170.000109

6.9e-058.1e-055.6e-05

7.2e-058.9e-055.3e-05

8.3e-05

8.3e-05

0.000174

0.000174

0.0001510.0002040.0001511.5e-05

0.0001510.0002040.0001511.5e-05

0.0001270.0001440.000118

0.0001270.0001440.000118

5.5e-050.0001194.7e-05

5.5e-050.0001194.7e-05

6e-05

5.5e-055.9e-054.7e-05

0.0002810.0002970.000251

8e-055.4e-057.1e-05

8e-055.4e-057.1e-05

5.9e-05

5.9e-05

5.6e-055.2e-054.9e-05

5.6e-055.2e-054.9e-05

0.0001095.7e-050.000101

0.0001095.7e-050.000101

3.6e-057.5e-053e-05

9e-061.7e-055e-06

1.1e-054.1e-051.1e-05

1.6e-051.7e-051.4e-05

0.0191670.042140.0205040.0299120.009810.0382560.026851

0.0002510.0002520.000195

7.6e-054.5e-055.6e-05

7.6e-054.5e-055.6e-05

9.7e-05

3.9e-05

2.5e-05

3.3e-05

0.0001754.2e-050.000139

0.0001754.2e-050.000139

6.8e-05

6.8e-05

0.0189160.042140.0205040.0299120.0095580.0380610.026851

0.0002657.6e-050.0001120.0002090.000167

1e-06

4.7e-05

0.0002657.6e-051.8e-050.0002090.000167

4.5e-05

1e-06

0.0177320.0419940.0205040.0299120.0092550.0371290.026481

6e-060.002612e-067e-06

5.8e-05

0.0013420.0023740.0019210.0023090.0008210.0031920.002339

0.000114

0.0010740.0039840.0007490.0029110.0002440.0011420.000381

0.0009870.0036890.0007420.0022080.0001880.0010320.000847

0.0048860.00429

3.1e-050.0008390.0024097e-064e-050.00146

0.0008730.0002750.0001680.000924

0.0022440.0005030.0009660.0032120.0003660.002373

0.0111750.030330.011240.0142530.0029970.0284190.021454

0.0009137e-050.0001140.0007170.000203

0.0003796.4e-050.0002876.4e-05

0.0005347e-055e-050.000430.000139

6.6e-05

2.7e-05

2.2e-05

1.7e-05

6e-061.1e-056e-06

6e-061.1e-056e-06

0.0008584.4e-050.0024030.0006950.000103

0.000534.4e-050.0018580.0004277.2e-05

6.8e-051.1e-053e-06

6.8e-051.1e-053e-06

8.4e-05

8.4e-05

4.9e-051.2e-053.8e-05

4.9e-051.2e-053.8e-05

0.0001490.0001130.0001393.2e-05

0.0001490.0001130.0001393.2e-05

4.8e-05

4.8e-05

0.0002644.4e-050.001590.0002474e-05

0.0002644.4e-050.0002060.0002473.2e-05

0.000123

0.001018

0.000118

8e-06

0.000125

0.0001460.0002380.0001123.1e-05

5.8e-05

5.8e-05

9e-050.0001027.1e-053.1e-05

3.4e-052.9e-052.6e-05

2.1e-053e-051.4e-05

3.5e-054.3e-053.1e-053.1e-05

4.6e-05

4.6e-05

5.6e-053.2e-054.1e-05

5.6e-053.2e-054.1e-05

8.8e-050.000138.1e-05

8.8e-050.000138.1e-05

2.4e-052e-062.4e-05

6.4e-057e-055.7e-05

4e-06

5.4e-05

3.4e-054.4e-052.6e-05

3.4e-054.4e-052.6e-05

3.4e-054.4e-052.6e-05

4.5e-05

4.5e-05

4.5e-05

6e-058.8e-054.9e-05

6e-058.8e-054.9e-05

2.9e-05

2.6e-05

6e-053.3e-054.9e-05

9.5e-050.0003348.7e-050.000433

7e-05

7e-05

3.1e-05

3.9e-05

9.6e-05

4.7e-05

4.7e-05

4.9e-05

4.9e-05

9.5e-050.0001688.7e-05

3.4e-055.4e-053.3e-05

3.4e-055.4e-053.3e-05

1.4e-053.5e-059e-06

1.4e-053.5e-059e-06

2.3e-053.5e-052.4e-05

2.3e-053.5e-052.4e-05

2.4e-054.4e-052.1e-05

2.4e-054.4e-052.1e-05

0.000433

0.000433

0.000433

0.0163480.0179680.0125450.0168620.0138050.0162590.009862

0.0163480.0179680.0125450.0168620.0138050.0162590.009862

2.9e-059.5e-052.5e-052e-05

5e-06

2.9e-054.2e-052.5e-05

1.5e-05

5.3e-05

8.6e-058.4e-058.4e-05

5.3e-052.2e-054.9e-05

3.3e-054e-053.5e-05

2.2e-05

0.0162330.0179680.0125450.0168620.0131230.016150.009842

1.1e-05

1.4e-05

0.0024540.0107170.0028260.0062230.0007710.0028180.009505

2.3e-05

4.8e-054e-06

1.7e-05

7e-06

1.2e-05

3.7e-051.4e-05

2e-051.6e-05

3e-06

5.8e-05

5.1e-05

2.2e-056e-062.8e-05

3.3e-054e-062.3e-056.1e-05

6.2e-054e-056.5e-054.9e-05

1.7e-050.0030114e-061.5e-05

1.7e-057e-061.6e-05

3.9e-053.6e-05

2.8e-051.1e-052.6e-05

3.2e-052.7e-059.2e-053.5e-05

3.9e-051.9e-050.0029111.6e-053.4e-054.6e-05

0.0134940.0072320.0097190.0047170.0118070.012998

3.5e-055.6e-053.5e-05

6e-06

0.000104

0.000503

0.000503

0.0064080.0085810.0078060.0140520.0026770.0048230.010262

0.0064080.0085810.0078060.0140520.0026770.0048230.010262

8.2e-050.0001176.4e-05

8.2e-050.0001176.4e-05

0.0001077.3e-050.000106

0.0001077.3e-050.000106

0.0003910.0001910.0003450.0004040.001509

8.3e-05

7.9e-059.1e-056.8e-058e-05

0.001509

0.0002370.00017.4e-050.000248

7.5e-050.000127.6e-05

2.8e-055.3e-050.0018070.0001852.7e-05

0.000111

2.8e-055.3e-050.0018072.1e-052.7e-05

5.3e-05

0.002560.0054360.0059520.0095350.0010140.002180.006089

0.0004370.0001450.0002210.0019070.0001460.0002090.000105

0.0006820.0002480.0006010.0037140.0005660.0007060.000412

0.000266

0.0040560.003561

0.0013050.0050430.0010740.0039140.0002760.001130.00158

0.000165

0.0001362.6e-050.000135

2.6e-053.9e-053.5e-05

2.6e-053.9e-053.5e-05

6.8e-050.0001326.2e-05

6.8e-050.0001326.2e-05

5.4e-054.5e-055.8e-054.5e-05

5.4e-054.5e-055.8e-054.5e-05

0.0030920.0029010.0018540.002710.0007270.0018870.002619

1.7e-05

0.000230.000110.000266

0.0028620.0029010.0018540.002710.0006170.0016210.002602

0.0001540.0004510.000132

0.0001540.0004510.000132

2.1e-050.0001482.1e-05

2.1e-050.0001482.1e-05

0.0001179.5e-059.3e-05

0.0001179.5e-059.3e-05

1.6e-050.0002081.8e-05

1.6e-058.1e-051.8e-05

7e-05

5.7e-05

0.012220.0081730.0088660.0098360.0110770.0116010.006805

3.3e-05

3.3e-05

3.3e-05

0.0002393.2e-050.0007630.000233

0.0002393.2e-050.0007630.000233

3e-06

4.5e-053e-064.4e-05

2.7e-05

6e-06

1.9e-05

2.2e-05

7e-06

3.3e-054e-062.6e-05

2.2e-05

0.0001613.2e-050.000650.000163

1.6e-054.5e-051.6e-05

1.6e-054.5e-051.6e-05

1.6e-052.3e-051.6e-05

2.2e-05

0.0041930.0034890.0032430.0062230.0038650.0039320.003198

0.0009920.0008620.0010490.0019070.0008530.0009390.000865

5.8e-05

3.7e-051.6e-052.9e-05

2.3e-05

4.2e-051.7e-053.1e-05

1.4e-05

0.0009130.0008620.0010490.0019070.0006940.0008790.000865

1.7e-05

1.4e-05

3.1e-058e-052.8e-05

3.1e-058e-052.8e-05

4.8e-05

4.8e-05

4.5e-05

4.5e-05

0.0004660.0010220.0022080.0002710.0003620.001175

0.0004190.000220.000313

2.9e-051.6e-053.3e-05

1.5e-05

7e-06

1e-06

1.8e-050.0010220.0022081.2e-051.6e-050.001175

0

0.0027040.0016050.0021940.0021080.0025680.0026030.001158

0.002690.0016050.0021940.0021080.002520.0025940.001158

1.4e-054.8e-059e-06

0.0076240.0046520.0056230.0036130.006230.0072990.003607

0.0076240.0046520.0056230.0036130.006230.0072990.003607

4.5e-054.1e-05

1.3e-055e-061.2e-05

2.6e-052.9e-052e-05

1.8e-051e-051.4e-05

2.8e-051.3e-053e-05

3e-051.5e-052.8e-05

8e-062e-066e-06

8e-065e-064e-068e-06

5e-058e-064.2e-05

0.0072720.0046470.0056230.0036130.0060470.006980.003607

1.5e-051.1e-051.5e-05

7e-061.7e-051.1e-05

5e-062e-064e-06

4.3e-052.7e-053.6e-05

2.8e-052.1e-052.5e-05

1.8e-051.6e-051.7e-05

1e-053e-061e-05

5.1e-053.8e-054.3e-05

5.1e-053.8e-054.3e-05

5.1e-053.8e-054.3e-05

6.5e-057.8e-056.1e-05

6.5e-057.8e-056.1e-05

2.6e-053.9e-052.1e-05

3.9e-053.9e-054e-05

3.2e-052.5e-051.7e-05

3.2e-052.5e-051.7e-05

3.2e-052.5e-051.7e-05

0.0013310.0059340.0017840.0090330.0009540.002020.007431

0.0010670.0059340.0017840.0063230.0006050.0018260.007431

0.0010670.0059340.0017840.0063230.0005790.0018260.007431

0.0010510.0059340.0017840.0063230.0005360.0018060.007431

2e-062e-065e-06

7e-068e-066e-06

1e-05

1.8e-05

7e-065e-069e-06

2.6e-05

2.6e-05

0.0002640.002710.0002650.000194

4.2e-054.4e-054.1e-05

4.2e-054.4e-054.1e-05

0.000170.0001460.000123

3.8e-055.9e-05

0.0001328.7e-050.000123

6e-06

6e-06

1.6e-050.002712.9e-051.3e-05

1e-061.6e-051e-06

1.5e-050.002711.3e-051.2e-05

3.6e-054e-051.7e-05

3.6e-051.5e-051.7e-05

2.5e-05

8.4e-05

3.6e-05

3.6e-05

4.8e-05

4.8e-05

8.7e-05

8.7e-05

8.7e-05

8.7e-05

0.0001990.0005680.000156

0.0001990.0005680.000156

0.000101

3.7e-05

6.4e-05

4.9e-052.3e-054.5e-05

4.9e-052.3e-054.5e-05

0.000150.0002630.000111

1.2e-055.1e-059e-06

1.4e-053e-052.2e-05

4.3e-05

0.0001247.4e-058e-05

6.5e-05

7.1e-05

7.1e-05

5.1e-05

5.1e-05

5.9e-05

5.9e-05

0.0138730.0301170.0502280.0300110.0624610.0125890.072075

0.0047980.0070120.0021070.0138510.0025670.0043920.010275

6.1e-051.5e-057.3e-055.2e-051.9e-05

4.8e-05

6.1e-051.5e-052.5e-055.2e-051.9e-05

1.5e-05

1.5e-05

0.0047370.0069970.0021070.0138510.0024790.004340.010256

2.5e-05

3.3e-05

1.4e-05

4e-05

6.6e-051.1e-055.8e-05

8.5e-05

5.7e-05

5.4e-05

7.4e-051.4e-056.9e-054e-06

5e-06

8.2e-058e-067.8e-052.7e-05

1.6e-05

0.0002340.0001763.7e-050.0002330.000115

0.0001352.6e-053e-060.000126

0.0001564.9e-050.000155

0.000164

0.0001764.7e-050.00016

4.2e-05

0.0001475.6e-052e-050.0001410.000282

0.0010010.0053570.0009480.005320.0002140.0009620.008051

4.7e-05

0.0002544.3e-053.5e-050.0002324.7e-05

8e-06

7e-06

0.0001853.3e-053.3e-050.000175

2.1e-05

0.0002130.000370.0025094.2e-050.0001920.000782

3.8e-05

5.4e-05

8e-06

1.9e-05

2.5e-05

3.1e-05

1.4e-05

1.4e-05

6.7e-05

6.8e-05

0.001340.0009160.0011590.0020070.0005830.0012870.000938

9.7e-052e-050.0040152.4e-058.8e-05

0.00016

2.8e-05

5e-06

7.5e-051.1e-057.2e-055e-06

3.5e-05

7e-06

2.9e-05

2.4e-05

1.9e-051.8e-051.9e-05

6e-06

0.0001422.5e-050.00014

2.1e-05

0.0001814.7e-050.000153

5.7e-05

7.3e-05

6e-06

1.6e-05

0.0090750.0231050.0481210.016160.0598940.0081970.0618

0.0001310.0001530.0001670.000143

6e-059.8e-053.6e-056.2e-05

4e-05

2.3e-05

2.6e-05

3.3e-051.7e-054e-05

1e-05

1.6e-058e-061.8e-05

2.2e-055.5e-057e-062.3e-05

0.0088480.0227870.0481210.016160.0595730.0079660.0618

0.0048870.0212510.0037030.0137510.0008160.0038390.028617

0.002484

0.0017430.0007180.0010190.0005120.001808

0.001220.0008180.0024090.000170.0012453.8e-05

0.000324

0.000602

0.000812

0.0433990.0540320.029425

2.3e-05

4.7e-05

0.001073

0.0009980.0001880.0010742.9e-05

6.4e-05

0.001004

0.001113

9.6e-050.0001650.0001548.8e-05

7.7e-05

9.6e-050.0001652e-058.8e-05

5.7e-05

0.0013590.0007180.0017060.0003430.0012330.003487

0.0013590.0007180.0017060.0003430.0012330.000543

0.0011260.0007180.0017060.0001920.0010760.000543

5.9e-05

9e-06

0.000122.3e-050.0001111.9e-05

0.000203

0.0001691.2e-050.0001577e-05

0.0008370.0007180.0017068.9e-050.0008080.000251

0.0001368.7e-058.6e-05

0.0001368.7e-058.6e-05

9.7e-056.4e-057.1e-05

9.7e-056.4e-057.1e-05

0.002944

0.002944

0.002944

0.1777360.1858710.1295470.099870.1344060.171120.085161

0.0005250.0012830.0005420.000945

0.0005250.0012830.0005420.000945

0.0001017.4e-059.5e-05

0.0001017.4e-059.5e-05

4.1e-056.3e-053.5e-05

4.1e-056.3e-053.5e-05

7.8e-05

7.8e-05

0.0002030.000920.0001990.000945

0.0002038.2e-050.000199

0.000279

0.000469

9e-05

0.000945

0.000180.0001480.000213

7.3e-053.9e-057e-05

7.6e-053.7e-057.2e-05

3.3e-05

3.1e-053.9e-057.1e-05

0.0001750.0003140.0001587e-05

0.0001750.0003140.0001587e-05

0.0001350.0001050.0001225.4e-05

0.0001350.0001050.0001225.4e-05

2.3e-058.4e-052.1e-05

2.3e-058.4e-052.1e-05

1.6e-05

1.6e-05

1.7e-050.0001251.5e-05

1.7e-050.0001251.5e-05

2e-059.8e-051.7e-05

2e-059.8e-051.7e-05

2e-059.8e-051.7e-05

2e-059.8e-051.7e-05

1e-054.5e-056e-06

1e-054.5e-056e-06

1e-054.5e-056e-06

1e-054.5e-056e-06

5.6e-050.0003434.5e-05

5.6e-050.0003434.5e-05

2.2e-050.0001761.7e-05

8e-069.7e-054e-06

1.4e-057.9e-051.3e-05

3.4e-050.0001672.8e-05

2.1e-058.1e-051.7e-05

1.3e-058.6e-051.1e-05

0.0049830.004460.0043170.0036130.0058010.004770.00362

0.0004013.3e-050.0007250.0003821.5e-05

6.2e-055.8e-055.7e-05

6.2e-055.8e-055.7e-05

5.1e-053.6e-055.4e-051.5e-05

2.9e-051.8e-053.1e-05

2.2e-051.8e-052.3e-051.5e-05

0.000131

0.000131

8.6e-053.3e-058.8e-058.5e-05

8.6e-053.3e-058.8e-058.5e-05

0.0001370.0001830.000129

0.0001370.0001830.000129

6.3e-050.0001085.5e-05

6.3e-055.8e-055.5e-05

5e-05

2e-060.0001212e-06

1.8e-05

0.0001

2e-063e-062e-06

0.0045820.0044270.0043170.0036130.0050760.0043880.003605

0.000228

0.000228

0.0001830.0002050.000163

8.4e-050.0001137.3e-05

9.9e-054.5e-059e-05

4.7e-05

1.9e-057.8e-051.2e-05

1.9e-057.8e-051.2e-05

0.000149

0.000149

4.6e-050.0001154e-05

4.6e-050.0001154e-05

0.0001220.0001270.000124

0.0001220.0001270.000124

0.000153

0.000153

0.0039990.0044150.0043170.0036130.0038640.0038390.003605

0.0001690.0001530.000161

0.003830.0044150.0043170.0036130.0037110.0036780.003605

0.0002131.2e-050.0001570.00021

0.0001065.8e-050.000108

4.6e-05

1.7e-05

0.0001071.2e-053.6e-050.000102

0.0852360.1151640.0662590.0359330.0706790.0819070.041485

0.0851190.1151640.0662590.0359330.0704270.0818050.04083

0.000146

0.000146

4.5e-050.0001184.5e-051.4e-05

2.7e-054e-052.8e-058e-06

1.8e-057.8e-051.7e-056e-06

0.000293

0.000293

0.000294

0.000179

0.000115

0.000147

0.000147

5e-054.5e-054.7e-05

5e-054.5e-054.7e-05

5.2e-05

5.2e-05

0.000122

0.000122

5.6e-057.1e-054.8e-05

2.8e-057e-061.5e-05

2.8e-056.4e-053.3e-05

0.000250.0002080.0002441.7e-05

0.000170.0001170.000171.7e-05

8e-059.1e-057.4e-05

0.000179

0.000179

7.6e-05

7.6e-05

0.0001910.0002030.000197

0.0001390.0001240.000144

4e-05

5.2e-053.9e-055.3e-05

0.000186

0.000186

5.5e-050.0001174.6e-05

5.5e-050.0001174.6e-05

0.000179

0.000179

0.0001260.0001370.000123

0.0001260.0001370.000123

0.0005270.0004420.0004450.0017060.0004090.0005190.002536

0.0001790.0001490.0001821e-06

0.0003480.0004420.0004450.0017060.000260.0003370.000353

0.002182

8.4e-05

8.4e-05

0.000118.5e-050.000106

0.000118.5e-050.000106

0.000194

0.000194

1.5e-05

1.5e-05

0.083690.1147220.0658140.0342270.0673750.0804150.037909

2.7e-050.0009732.8e-05

0.0836630.1147220.0658140.0342270.0660530.0803870.037909

0.000349

4.6e-05

4.6e-05

1.9e-051.5e-05

1.9e-051.5e-05

0.0001170.0002520.0001020.000655

1e-057.7e-051.4e-05

1e-057.7e-051.4e-05

4.5e-056.2e-053.3e-05

4.5e-056.2e-053.3e-05

6.2e-050.0001135.5e-05

6.2e-050.0001135.5e-05

0.000655

0.000655

0.004660.0081380.0050640.0103380.00460.0044670.007256

4.2e-057e-052.6e-05

4.2e-057e-052.6e-05

4.2e-057e-052.6e-05

7.3e-05

7.3e-05

7.3e-05

0.0035640.0075510.0050640.0065240.0040250.0034330.006642

9e-062.8e-051.2e-05

3e-068e-064e-06

4e-06

3e-068e-066e-06

2e-067e-061e-06

1e-061e-061e-06

0.0026140.0066180.0042850.0048180.0036210.0025150.006352

07e-060

5e-060.0001393e-060.00037

0.0026090.0066180.0042850.0048180.0034750.0025120.005982

0.0009360.0009330.0007790.0017060.000330.0009020.00029

3e-062.8e-053e-06

2e-061e-06

0

01.5e-050

3e-062.7e-053e-06

5e-060.0002395e-06

0.0009230.0009330.0007790.0017060.000890.00029

2.1e-05

5e-064.6e-054e-06

1e-063e-061e-06

3.1e-05

4e-061.2e-053e-06

0.0010540.0005870.0038140.0004320.0010080.000614

0.0010520.0005870.0038140.0004170.0010040.000614

4.3e-051.8e-054.6e-05

0.000123

4e-061e-061e-06

0.0001754.4e-050.000194

0.00032

0.0001433.4e-054.8e-051.8e-05

5.9e-050.0018071.4e-056e-05

2.1e-050.0020071e-052.1e-052e-06

1.8e-050.0001681.1e-052e-055.3e-05

1.3e-052.3e-057e-06

1.3e-055e-061.4e-055.6e-05

1.1e-055e-061.1e-05

1.3e-055e-064e-06

4e-061e-066e-06

2.3e-057e-062.4e-05

5e-061e-065e-06

0.0003890.000198.9e-050.0004159.4e-05

9.5e-052e-050.0001025.7e-05

1.1e-052e-061.2e-05

2e-060.0002291e-062e-061.4e-05

1e-053e-061.2e-05

2e-061.5e-054e-06

2e-061.5e-054e-06

0.000225

0.000115

0.000115

0.000115

0.00011

0.00011

0.00011

0.0796480.0571920.0527180.0463720.0482660.0768350.030824

0.0013510.000740.0010820.0018070.0014460.0012870.000504

0.0012940.000740.0010820.0018070.0011840.0012390.000504

0.0012940.000740.0010820.0018070.0011840.0012390.000504

5.7e-058.8e-054.8e-05

5.7e-058.8e-054.8e-05

0.000174

0.000174

0.0002176.4e-05

3.4e-05

3.4e-05

0.000115

0.000115

3e-05

3e-05

0.000102

4.7e-05

5.5e-05

0.0008660.0102070.0018960.0112420.0002360.0008230.010703

0.0007980.0102070.0018960.0112420.000130.0007830.01067

4e-060.001094e-064e-06

4e-061e-064e-06

6e-065e-066e-06

4.7e-05

5e-060.0009056e-065e-06

9e-061.7e-055e-06

2e-052.8e-052e-050.000203

3e-061.1e-053e-06

1e-06

0.0007470.0082120.0018960.0112421e-050.0007360.010467

6.8e-050.0001064e-053.3e-05

1.7e-05

6.8e-053.5e-054e-051.6e-05

7.1e-05

0.0015560.0024060.0015020.002810.0010360.0014930.002419

0.0015560.0024060.0015020.002810.0010360.0014930.002419

2e-05

0.000112

6.2e-053.4e-055.7e-05

0.0012160.0023780.0015020.002810.0005950.0011680.002404

7.7e-057.2e-056.8e-05

7.5e-056.7e-057.6e-051.5e-05

0.0001262.8e-054.4e-050.000124

9.2e-05

6.2e-057.3e-055.7e-05

6.2e-057.3e-055.7e-05

6.2e-057.3e-055.7e-05

0.0120260.0071490.0067890.0069260.0030350.011570.002439

4.4e-05

4.4e-05

6.3e-05

6.3e-05

0.0001533.2e-050.0001660.0001351.4e-05

8.8e-052.1e-056.8e-057.9e-051.4e-05

5e-053.7e-054.4e-05

1.5e-051.1e-051.4e-051.2e-05

4.7e-05

7.5e-05

7.5e-05

0.0116010.0071170.0067890.0069260.0023490.0111790.001653

0.0101090.0059230.0051290.0043160.0019690.009716

3.1e-05

5.3e-05

0

8.2e-05

0.0013030.001170.001660.002612.6e-050.0012570.001587

7.3e-05

0.0001212.4e-054.6e-050.0001456.6e-05

0

6.8e-054.5e-056.1e-05

4e-06

2e-05

0.0002260.0002180.0002140.000772

4.3e-051.5e-054.6e-050.000186

0.000206

4.9e-055e-054.7e-059.8e-05

5.4e-055.3e-054.7e-05

6.5e-05

8e-053.5e-057.4e-050.000282

4.6e-050.000124.2e-05

8e-062.3e-059e-06

1.4e-053.1e-051.3e-05

1.9e-052.3e-051.7e-05

3.5e-05

5e-068e-063e-06

0.0002330.0006150.000213

2.7e-05

2.7e-05

0.000141

6.8e-05

7.3e-05

5.5e-057.7e-055.2e-05

5.5e-057.7e-055.2e-05

0.0001160.0001690.000103

4.9e-056.3e-054.2e-05

3.9e-054.8e-053.4e-05

2.8e-055.8e-052.7e-05

6.2e-057.3e-055.8e-05

6.2e-057.3e-055.8e-05

3.4e-05

3.4e-05

9.4e-05

9.4e-05

0.0587140.033950.0403680.0220810.0377510.0564990.013787

3.4e-053.3e-052.8e-055.7e-05

3.4e-053.3e-052.8e-055.7e-05

0.000325

0.000159

8.7e-05

7.9e-05

0.0001920.0001710.0001740.000125

0.0001110.0001050.0001059.2e-05

8.1e-056.6e-056.9e-053.3e-05

8.3e-05

8.3e-05

0.0563630.0322730.0376580.0184680.0353560.054260.006898

0.0001463.2e-056.9e-050.000151

0.000127

0.000109

0.0555460.0320520.0376580.0184680.0342890.053399

0.0001523.4e-056.6e-050.000166.4e-05

0.000431

0.0002026.5e-050.0001090.0002150.003395

0.0001835.8e-055.8e-050.0001930.003407

0.0001343.2e-059.8e-050.0001423.2e-05

0.0021250.0016770.002710.0036130.0018660.0020370.006624

0.0021250.0016770.002710.0036130.0018660.0020370.006624

7.5e-05

7.5e-05

7.5e-05

7.5e-059.5e-057.4e-05

7.5e-059.5e-057.4e-05

7.5e-059.5e-057.4e-05

0.0004170.0003570.0003992.1e-05

0.0001430.0001530.000133

0.0001430.0001530.000133

0.0001218.4e-050.000117

0.0001218.4e-050.000117

0.0001530.000120.0001492.1e-05

0.0001530.000120.0001492.1e-05

7.7e-050.0001188e-05

7.7e-050.0001188e-05

6e-066e-066e-06

2.8e-05

6e-061.2e-057e-06

1.1e-056e-061.4e-05

9e-062.2e-055e-06

1.1e-056e-061.1e-05

2.6e-052.8e-052.5e-05

4e-062e-069e-06

4e-068e-063e-06

0.0042710.002740.0010810.0015060.0032120.004340.000887

0.00023

0.00023

8.7e-050.0002677.6e-051.5e-05

8.7e-050.0002677.6e-051.5e-05

0.000111

0.000111

0.0041840.002740.0010810.0015060.0026040.0042640.000872

0.0009520.0006210.0002720.0009740.000334

0.001184

0.000880.0006440.0003330.0009040.000245

0.0012920.0008090.0010810.0015060.0005490.001301

0.001060.0006660.0002660.0010850.000293

0.0024230.0009170.0011890.0036140.0027520.0023730.000961

0.0015220.0008510.0011890.0018070.0009410.0014630.000883

0.00018

3.9e-05

2.8e-05

2.9e-05

1.7e-05

3.3e-05

3.4e-05

4e-05

4e-05

0.0015220.0008510.0011890.0018070.0006710.0014630.000883

2.3e-05

0.0015220.0008510.0011890.0018070.0006480.0014630.000883

5e-05

2.2e-05

2.8e-05

0.0009016.6e-050.0018070.0018110.000917.8e-05

1.8e-053.6e-055.4e-05

1.8e-053.6e-055.4e-05

4.3e-05

4.3e-05

3.4e-05

3.4e-05

0.000130.0001360.000117

6.6e-05

1.6e-05

7.4e-053.8e-056.5e-05

5.6e-051.6e-055.2e-05

4.4e-05

4.4e-05

0.0002540.000360.0002437.8e-05

4.7e-05

3.9e-05

0.0001335.5e-050.0001223.9e-05

6.5e-05

0.0001216.5e-050.000121

4.9e-05

7.9e-05

0.000286.6e-050.0018070.0009290.000284

5.5e-05

0.000755

1.5e-05

3.2e-05

0.000286.6e-050.0018074.3e-050.000284

2.9e-05

0.0002190.0002290.000212

2.2e-05

7.6e-051.2e-057.5e-05

1.4e-05

3.3e-05

2.1e-05

7.6e-054.4e-057.2e-05

4.6e-05

6.7e-052e-056.5e-05

1.7e-05

0.0140560.0081160.0106940.0079290.01490.0134380.006377

3.8e-050.0003993.6e-05

1.1e-056.1e-059e-06

1.1e-056.1e-059e-06

4e-064e-053e-06

7e-062.1e-056e-06

2.7e-050.0003382.7e-05

2.7e-050.0003382.7e-05

2.7e-050.0003382.7e-05

0.0102360.0061320.0080820.0057210.0100720.0098250.004902

0.0040290.0024630.0032190.0025090.0040250.0038620.001818

0.0039820.0024630.0032190.0025090.0039060.0038250.001818

0.0039820.0024630.0032190.0025090.0039060.0038250.001818

2.2e-054.1e-051.8e-05

2.2e-054.1e-051.8e-05

5e-063.3e-054e-06

5e-063.3e-054e-06

2e-054.5e-051.5e-05

2e-054.5e-051.5e-05

0.0062070.0036690.0048630.0032120.0060470.0059630.003084

5e-063.3e-054e-06

5e-063.3e-054e-06

8e-062.1e-058e-06

8e-062.1e-058e-06

1e-062.9e-051e-06

1e-062.9e-051e-06

0.0061930.0036690.0048630.0032120.0059640.005950.003084

0.0061930.0036690.0048630.0032120.0059640.005950.003084

2.3e-050.0001591.9e-05

1.7e-050.0001231.4e-05

1.2e-056.4e-059e-06

1.2e-056.4e-059e-06

3e-063e-053e-06

3e-063e-053e-06

2e-062.9e-052e-06

2e-062.9e-052e-06

6e-063.6e-055e-06

6e-063.6e-055e-06

6e-063.6e-055e-06

0.0033760.0019840.0026120.0022080.0032960.0032290.001461

8e-062.1e-058e-06

8e-062.1e-058e-06

8e-062.1e-058e-06

2.6e-057.8e-052.3e-05

2.6e-057.8e-052.3e-05

2.6e-053.2e-052.3e-05

4.6e-05

0.0033420.0019840.0026120.0022080.0031970.0031980.001461

5e-062.1e-055e-06

5e-062.1e-055e-06

0.0033370.0019840.0026120.0022080.0031760.0031930.001461

2.3e-05

2e-062e-06

2.4e-053.1e-052.2e-05

6e-063.1e-057e-06

4.4e-05

2.6e-053e-052.3e-05

2.1e-053.1e-052.1e-05

0.003210.0019840.0026120.0022080.0029440.0030830.001461

8e-064e-06

1.3e-051.6e-051.1e-05

1.6e-051.3e-05

1.1e-052.6e-057e-06

1.9e-058.9e-051.7e-05

1.9e-058.9e-051.7e-05

3.8e-05

3.8e-05

1.9e-055.1e-051.7e-05

1.9e-055.1e-051.7e-05

3.1e-052.8e-052e-05

3.1e-052.8e-052e-05

3.1e-052.8e-052e-05

3.1e-052.8e-052e-05

0.0001540.0003530.000136

0.0001220.0002180.000109

3.7e-05

3.7e-05

0.0001220.0001810.000109

6e-06

1.3e-052.5e-051.1e-05

2e-059e-061.7e-05

7e-067e-064e-06

7e-062.1e-058e-06

2.1e-051.9e-051.7e-05

2.3e-05

1.4e-053.5e-051.3e-05

2.6e-056e-062.3e-05

1.4e-053e-051.6e-05

3.2e-050.0001352.7e-05

3.2e-056.2e-052.7e-05

1.9e-052.7e-051.8e-05

1.3e-053.5e-059e-06

7.3e-05

4e-05

3.3e-05

0.0001480.0004680.000131.4e-05

1.6e-057.8e-051.3e-05

1.6e-053.5e-051.3e-05

1.6e-053.5e-051.3e-05

4.3e-05

4.3e-05

4.8e-059.7e-054.3e-05

3.3e-057.1e-052.9e-05

1.4e-05

1e-051.8e-051e-05

9e-062e-058e-06

1.4e-051.9e-051.1e-05

1.5e-052.6e-051.4e-05

1.5e-052.6e-051.4e-05

4.9e-059.3e-054.5e-051.4e-05

4.9e-059.3e-054.5e-051.4e-05

3e-055.2e-052.7e-05

7e-061.6e-056e-06

1.2e-052.5e-051.2e-051.4e-05

1.3e-057.5e-051.1e-05

2.9e-05

2.9e-05

1.3e-054.6e-051.1e-05

1.3e-054.6e-051.1e-05

4.5e-05

4.5e-05

4.5e-05

3.9e-05

3.9e-05

3.9e-05

2.2e-054.1e-051.8e-05

2.2e-054.1e-051.8e-05

2.2e-054.1e-051.8e-05

3.1e-053.6e-052.6e-05

3.1e-053.6e-052.6e-05

3.1e-053.6e-052.6e-05

3.1e-053.6e-052.6e-05

0.0781530.0561870.1081080.0625310.0921940.0751480.096748

0.0231030.0128950.0157280.0095350.0253130.0220910.010629

0.0009980.000587

9.6e-05

9.6e-05

0.0009020.000587

0.000902

0.000587

0.0216670.0126590.0157280.0095350.0201390.0207980.009501

6e-055.7e-054.5e-05

6e-055.7e-054.5e-05

0.000132

6.4e-05

6.8e-05

0.0215410.0126590.0157280.0095350.0198720.0206960.009501

0.0215080.0126590.0157280.0095350.0197870.0206660.009501

3.3e-058.5e-053e-05

6.6e-051.7e-055.7e-05

2.8e-059e-062.5e-05

3.8e-058e-063.2e-05

6.1e-05

6.1e-05

0.0001290.0003190.000105

2.6e-050.0001052.4e-05

5.2e-05

2.6e-055.3e-052.4e-05

0.0001030.0002148.1e-05

1.2e-051.3e-051.6e-05

2.1e-05

3.8e-054.7e-051.2e-05

4.6e-05

2.2e-054.3e-053e-05

3.1e-054.4e-052.3e-05

0.0010130.0002360.0035640.0009760.000541

0.0010130.0002360.0035640.0009760.000541

0.0010130.0002360.0035640.0009760.000541

0.0002940.0002930.000212

0.0001120.0001247.5e-05

0.0001120.0001247.5e-05

0.0001289.6e-059.7e-05

0.0001289.6e-059.7e-05

5.4e-057.3e-054e-05

5.4e-057.3e-054e-05

0.0081430.010340.0094050.016060.0102240.0078360.030304

0.0075340.010340.0094050.016060.0099290.0073770.030158

0.001763

0.001763

0.000319

0.000319

0.0075340.010340.0094050.016060.0084870.0073770.027225

0.002837

0.000139

0.000734

0.001387

0.0043070.000853

0.003910.0016130.0012530.0094350.0016460.0039010.000277

2.2e-05

0.002549

0.0019510.0012870.0007910.0023090.0001870.0013020.001269

0.001718

0.002183

0.0064640.005675

0.0016730.007440.0008970.0043160.0001640.0021740.009602

0.000163

0.000281

0.000281

0.0011610.000851

0.001161

0.000851

0.0006090.0002950.0004590.000146

0.0002590.0001160.0002013.2e-05

3.2e-05

0.0002590.0001160.000201

0.0001918.9e-050.0001494.8e-05

0.0001918.9e-050.0001494.8e-05

0.0001599e-050.0001096.6e-05

0.0001599e-050.0001096.6e-05

0.0020250.001250.0016020.0019070.0018550.0019290.000816

0.0020250.001250.0016020.0019070.0018550.0019290.000816

6.6e-053.1e-056.3e-05

6.6e-053.1e-056.3e-05

3.9e-051.8e-053.7e-05

3.9e-051.8e-053.7e-05

9.6e-050.0001288.3e-05

1.4e-052.7e-051.4e-05

3.2e-051.6e-052.9e-05

1.3e-051.7e-058e-06

1e-051.9e-057e-06

2.7e-054.9e-052.5e-05

0.0018240.001250.0016020.0019070.0016780.0017460.000816

0.0018240.001250.0016020.0019070.0016780.0017460.000816

0.0019040.0002950.002150.0017220.0003

0.0001160.0001079.3e-05

0.0001160.0001079.3e-05

0.0001160.0001079.3e-05

0.0013680.0002950.0018270.0012780.000269

0.0007230.0002190.0015420.000680.000183

0.0007230.0002190.0003220.000680.000183

0.00122

0.0006457.6e-050.0002850.0005988.6e-05

0.000190.0001690.000179

0.0004557.6e-057e-060.0004198.6e-05

1.3e-05

9.6e-05

0.000420.0002160.0003513.1e-05

0.0001710.0001120.000153

0.0001710.0001120.000153

0.0002490.0001040.0001983.1e-05

0.0002490.0001040.0001983.1e-05

0.0429780.0314070.0813730.0350290.0526520.041570.054699

0.0007620.0188580.008040.0006310.009369

0.000270.0001430.000228e-05

0.000270.0001430.000228e-05

5.7e-05

5.7e-05

0.0188580.0074960.009222

5.4e-05

0.0083530.007334

0.000108

0.0105050.009222

9.3e-057.2e-057.7e-051.6e-05

5.1e-054.2e-058e-06

4.2e-057.2e-053.5e-058e-06

4.4e-05

4.4e-05

0.0002289.8e-050.0001941e-06

0.0002289.8e-050.0001941e-06

5.1e-05

5.1e-05

0.0001717.9e-050.000145e-05

0.0001717.9e-050.00014

5e-05

0.0016770.0029460.0003570.0049180.001310.0014350.002439

4.3e-058.1e-053.6e-05

4.3e-058.1e-053.6e-05

0.0012240.0028990.0003570.0049180.000610.0011560.00237

7e-06

0.000218.4e-050.000173

2.9e-05

8.6e-05

2.9e-05

0.0001114e-059.9e-051.6e-05

5.7e-05

3.2e-05

0.0008140.0028380.0003570.002710.0001530.0007940.000107

8.2e-05

5.4e-053.1e-051e-065.4e-050.00163

3.5e-053e-050.0022081e-053.6e-050.000617

0.0003434.7e-050.000110.0002086.9e-05

3.6e-05

0.0003434.7e-057.4e-050.0002086.9e-05

0.000103

0.000103

0.000115

0.000115

0.000109

0.000109

2.5e-057e-051.1e-05

4e-061.8e-053e-06

2.1e-055.2e-058e-06

4.2e-050.0001122.4e-05

4.2e-054.2e-052.4e-05

7e-05

0.0080030.0039730.004680.0050180.0038440.0076160.003992

0.000163

8.6e-05

7.7e-05

6.7e-050.0002134.7e-05

6.7e-057.5e-054.7e-05

7.9e-05

5.9e-05

9.6e-056.2e-058.2e-05

9.6e-056.2e-058.2e-05

0.0016650.0004970.0007690.0020070.0013290.0015890.000613

0.000914

0.0016650.0004970.0007690.0020070.0002840.0015890.000613

0.000131

0.0061750.0034760.0039110.0030110.0020770.0058980.003379

0.0061750.0034760.0039110.0030110.0019990.0058980.003379

3.4e-05

4.4e-05

0.0165530.014532

0.0165530.014532

0.0165530.014532

0.0193850.0137750.0293350.0137510.0293690.0193040.014229

0.0008270.0001320.0004730.0007850.000125

8.6e-055.1e-057.2e-05

4.4e-05

9.8e-053e-054.8e-059.4e-05

0.000118

0.0001417e-050.0001311e-06

0.0002385.8e-054.9e-050.0002324e-05

0.0002644.4e-059.3e-050.0002568.4e-05

0.0053750.0070140.0031140.0092340.0022130.0052820.006666

3.3e-057e-063.1e-05

0.000282

6.5e-052.4e-058e-066.5e-059e-06

0.0004730.0041770.0011020.002713.4e-050.0004620.003616

1.2e-05

5e-06

0.0001181.4e-054e-060.0001248.2e-05

0.0001010.000150.0021082.6e-050.00010.000672

2.7e-05

1e-06

0.0028520.0024310.0020120.0024090.0007480.0027720.001917

1.9e-055e-061.9e-05

0.00022e-050.000191.6e-05

1.9e-05

4.4e-052.8e-056e-064.3e-058.3e-05

0.000146.5e-050.000147

0.0001291.3e-050.000131

3.2e-05

0.000262.8e-054.1e-050.000265.4e-05

9.2e-052.8e-059.2e-054e-06

0.0002112.9e-050.0020070.0001160.0002080.000105

5.6e-05

5.4e-051.4e-051e-055.6e-05

0.0001282.4e-050.000134

0.000121

2.5e-05

2.3e-05

1.5e-05

5.4e-05

1e-06

6e-06

0

0.0004560.0001190.0001890.0004480.000108

2.2e-05

1e-05

7.1e-05

2.1e-05

5e-05

1.6e-05

1.4e-05

1.4e-05

0.0076160.003870.0251930.0223060.0078790.003998

0.000976

0.0023240.0009020.0016280.0024110.001183

0.0015780.0002320.0011130.0016210.000602

0.0018330.0004130.0009830.0010620.0019040.000736

0.0209140.018361

0.0018810.0023230.0016680.0007940.0019430.001477

0.0019470.0015740.0010280.0022080.0009270.0019020.001944

0.00011

0.0010620.0002830.0003340.0010356.2e-05

0.0008850.0012910.0010280.0022080.0003840.0008670.001882

9.9e-05

0.0034950.0011850.0023090.0029980.0033480.001482

1.5e-05

0.0034650.0029520.003334

3e-050.0011850.0023093.1e-051.4e-050.001482

0.0001250.0004520.000108

0.0001251.4e-050.000108

2.4e-05

0.000167

7.6e-05

2.6e-05

2.5e-05

1.5e-05

8e-05

2.5e-05

0.0131510.0107130.011590.0113420.0100890.0125840.010138

0.000128

7.2e-05

5.6e-05

2.5e-055.2e-052.1e-05

2.5e-055.2e-052.1e-05

0.000155.5e-054.9e-050.0001345.5e-05

0.000155.5e-054.9e-050.0001345.5e-05

7.4e-05

3.8e-05

3.6e-05

0.0002487.3e-050.0002125.2e-05

0.0002487.3e-050.0002125.2e-05

7.9e-057.1e-056.5e-05

7.9e-057.1e-056.5e-05

0.0004436.3e-050.0004130.0004144.6e-05

0.0003116.3e-050.0003250.0003023e-05

0.0001328.8e-050.0001121.6e-05

9.3e-05

9.3e-05

0.0001760.0001580.0001651.5e-05

0.0001760.0001580.0001651.5e-05

0.0001144.4e-050.000105

0.0001144.4e-050.000105

0.0061750.0059370.0073030.0071260.0056460.0059230.008557

0.0061750.0059370.0073030.0071260.0055730.0059230.008557

7.3e-05

0.000111

6.8e-05

4.3e-05

0.0056210.0046580.0042870.0042160.0031290.0054190.001413

0.0024130.0017040.001750.0021080.0007850.0023480.001368

0.0001558.1e-050.0001481.9e-05

0.0028530.0016990.0025370.0021080.0021460.002739

4.6e-05

8.7e-050.0012553.1e-058.4e-05

0.0001134e-050.00012.6e-05

0.000124.8e-050.000126

6.3e-051.5e-056.3e-05

2.1e-05

5.7e-051.2e-056.3e-05

6.2e-058.9e-056e-05

6.2e-058.9e-056e-05

6.2e-058.9e-056e-05

6.2e-058.9e-056e-05

1.4e-053.9e-051.8e-05

1.1e-052.3e-059e-06

3.7e-052.7e-053.3e-05

1.6e-050.0001081.5e-05

1.6e-050.0001081.5e-05

1.6e-050.0001081.5e-05

1.6e-050.0001081.5e-05

1.6e-050.0001081.5e-05

1.6e-050.0001081.5e-05

2.8e-055.1e-052.2e-05

2.8e-055.1e-052.2e-05

2.8e-055.1e-052.2e-05

2.8e-055.1e-052.2e-05

2.8e-055.1e-052.2e-05

2.8e-055.1e-052.2e-05

3e-064.4e-052e-06

3e-064.4e-052e-06

3e-064.4e-052e-06

3e-064.4e-052e-06

3e-064.4e-052e-06

3e-064.4e-052e-06

0.0056460.003490.0049770.0031120.0058320.005428

0.0056460.003490.0049770.0031120.0058320.005428

0.0056460.003490.0049770.0031120.0058320.005428

3e-060.0001163e-06

3e-060.0001163e-06

3e-060.0001163e-06

0.0056430.003490.0049770.0031120.0057160.005425

0.0056430.003490.0049770.0031120.0057160.005425

0.0056430.003490.0049770.0031120.0057160.005425

0.0053860.0032110.003910.002710.0049990.0051290.002282

0.0053860.0032110.003910.002710.0049990.0051290.002282

0.0052220.0032110.003910.002710.004670.0050080.002282

0.0052220.0032110.003910.002710.004670.0050080.002282

1.8e-056e-051.2e-05

7e-063.1e-055e-06

1.1e-052.9e-057e-06

0.0051680.0032110.003910.002710.004530.0049630.002282

0.0051520.0032110.003910.002710.0044460.0049490.002241

7e-062.5e-056e-06

1.8e-054.1e-05

4e-061.2e-053e-06

5e-062.7e-055e-06

2e-06

1.3e-053.3e-051.3e-05

1.3e-053.3e-051.3e-05

2.3e-054.7e-052e-05

2.3e-054.7e-052e-05

0.0001640.0003290.000121

0.0001370.0002779.7e-05

0.0001370.0002779.7e-05

2.1e-051.8e-051.5e-05

1.1e-052.3e-059e-06

1.6e-053.8e-051.2e-05

2.3e-05

1e-05

2e-053.7e-051.3e-05

9e-06

2.8e-053.1e-052.1e-05

3e-053.6e-051.9e-05

1.8e-05

1.1e-053.4e-058e-06

2.7e-055.2e-052.4e-05

2.7e-055.2e-052.4e-05

2.7e-055.2e-052.4e-05

0.0001050.005350.0005459.2e-050.004697

1.6e-057.1e-051.1e-05

1.6e-057.1e-051.1e-05

1.3e-054.8e-059e-06

1.3e-054.8e-059e-06

5e-062.3e-053e-06

8e-062.5e-056e-06

3e-062.3e-052e-06

3e-062.3e-052e-06

0

3e-062.2e-052e-06

1e-06

0.005350.004697

0.005350.004697

0.005350.004697

0.005350.004697

0.005350.004697

2e-056.2e-051.9e-05

1.7e-055.7e-051.5e-05

1.7e-055.7e-051.5e-05

1.7e-055.7e-051.5e-05

1.7e-055.7e-051.5e-05

3e-065e-064e-06

3e-065e-064e-06

3e-065e-064e-06

0

3e-065e-064e-06

9e-060.00011e-05

9e-060.00011e-05

9e-060.00011e-05

6.4e-05

6.4e-05

9e-063.6e-051e-05

9e-063.6e-051e-05

5.3e-050.0002554.7e-05

9e-068.6e-057e-06

9e-068.6e-057e-06

9e-068.6e-057e-06

9e-068.6e-057e-06

4.4e-050.0001694e-05

4.4e-050.0001694e-05

4.4e-050.0001694e-05

4.4e-050.0001694e-05

7e-065.7e-055e-06

7e-065.7e-055e-06

7e-065.7e-055e-06

7e-065.7e-055e-06

7e-065.7e-055e-06

7.4e-050.0002716.5e-05

7e-063.8e-056e-06

7e-063.8e-056e-06

7e-063.8e-056e-06

7e-063.8e-056e-06

7e-063.8e-056e-06

6e-066.5e-055e-06

6e-066.5e-055e-06

6e-066.5e-055e-06

6e-066.5e-055e-06

6e-066.5e-055e-06

6.1e-050.0001685.4e-05

6.1e-050.0001685.4e-05

6.1e-050.0001685.4e-05

1.9e-055.1e-051.8e-05

7e-062.6e-057e-06

1.2e-052.5e-051.1e-05

8e-063.2e-055e-06

8e-063.2e-055e-06

2.1e-055.1e-052e-05

1e-052.5e-051.1e-05

1.1e-052.6e-059e-06

1.3e-053.4e-051.1e-05

1.3e-053.4e-051.1e-05

3e-050.0002733.2e-05

3e-050.0002733.2e-05

3e-050.0002733.2e-05

3e-050.0002733.2e-05

7e-064.3e-054e-06

5e-063.4e-053e-06

5e-06

2e-064e-061e-06

1.3e-055.2e-052.1e-05

1.3e-055.2e-052.1e-05

1e-050.0001787e-06

1e-056e-057e-06

6.6e-05

5.2e-05

0.0001130.0003388.8e-05

0.0001130.0003388.8e-05

0.0001130.0003388.8e-05

0.0001130.0003388.8e-05

4e-055.2e-053e-05

1.2e-05

1.6e-052.2e-051.1e-05

2.4e-051.8e-051.9e-05

4.3e-050.0001063e-05

1.9e-052.2e-051e-05

7e-063e-056e-06

1.3e-052.3e-057e-06

4e-063.1e-057e-06

9e-067.7e-058e-06

4.5e-05

9e-063.2e-058e-06

6e-065.7e-054e-06

6e-065.7e-054e-06

1.5e-054.6e-051.6e-05

5e-062.6e-051e-05

1e-052e-056e-06

0.0061220.0092160.005690.0095350.0048050.0103710.007231

0.0061220.0092160.005690.0095350.0048050.0103710.007231

0.0006690.000370.0002120.0016060.0003010.0006450.000221

0.0006690.000370.0002120.0016060.0003010.0006450.000221

0.0006690.000370.0002120.0016060.0003010.0006450.000221

0.0002420.00013400.00022

0.0002090.0001060.0002120.0016060.0001610.0002070.000158

1.2e-051.9e-051.2e-05

0.0002060.000130.0001210.0002066.3e-05

0.0020.0046770.00220.0032120.0016070.0018930.003645

0.000140.0006120.000129

1.4e-055.8e-051.1e-05

7e-063.2e-055e-06

7e-062.6e-056e-06

4e-063e-054e-06

4e-063e-054e-06

8e-050.0003677.9e-05

4e-063e-054e-06

0

5e-063.8e-054e-06

2.4e-052.4e-052.2e-05

4e-066e-054e-06

1e-06

3e-063.2e-053e-06

5e-063.6e-056e-06

5e-05

3.5e-057.3e-053.6e-05

2.3e-05

1e-053.9e-058e-06

1e-053.9e-058e-06

3.2e-050.0001182.7e-05

9e-065.5e-051e-05

1.7e-053.3e-051.2e-05

6e-063e-055e-06

0.001860.0046770.00220.0032120.0009950.0017640.003645

6.2e-051.6e-053e-05

1e-061e-06

1e-06

2e-06

3.2e-059e-06

1.7e-0501.9e-05

4e-063e-064e-06

8e-067e-06

0.0017980.0046770.00220.0032120.0009790.0017340.003645

2.9e-051.1e-053.5e-05

2e-064e-062e-06

5e-053.9e-054.6e-05

9e-061.5e-059e-06

1.4e-053e-061.3e-05

0.0016940.0046770.00220.0032120.0009070.0016290.003645

0.0034530.0041690.0032780.0047170.0028970.0078330.003365

0.0034530.0041690.0032780.0047170.0028970.0078330.003365

0.0034430.0041690.0032780.0047170.0028540.0078250.003365

0.001601

1e-061.5e-051e-06

0.0009510.0015970.0008910.0022080.0002750.000930.00122

2.2e-05

0.0024910.0025720.0023870.0025090.0009410.0068940.002145

1e-054.3e-058e-06

1e-054.3e-058e-06

2.4e-057.2e-051.1e-05

2.4e-057.2e-051.1e-05

2.4e-057.2e-051.1e-05

2.4e-057.2e-051.1e-05

2.4e-057.2e-051.1e-05

2.4e-057.2e-051.1e-05

2.3e-050.0001351.4e-05

2.3e-050.0001351.4e-05

2.3e-050.0001351.4e-05

2.3e-050.0001351.4e-05

7e-063.6e-056e-06

7e-063.6e-056e-06

1.6e-056.5e-058e-06

3.1e-05

1.6e-053.4e-058e-06

3.4e-05

3.4e-05

0.1698180.2229640.1925240.2345690.1348550.1544040.256614

0.0034740.0017560.0083620.0052190.0042990.0033540.006834

0.0034740.0017560.0083620.0052190.0042990.0033540.006834

0.0034740.0017560.0083620.0052190.0042990.0033540.006834

0.0029090.00150.0024220.0022080.0027840.0028020.001555

0.0029090.00150.0024220.0022080.0027840.0028020.001555

0.000116

0.000116

0.000111

0.000111

0.0005650.0002560.005940.0030110.0012880.0005520.005279

0.005940.005215

0.0005650.0002560.0030110.0012880.000552

6.4e-05

6.7e-05

6.7e-05

6.7e-05

6.7e-05

6.7e-05

0.025870.0328810.0285580.0307140.0298380.0217450.049465

2.9e-050.0002184e-05

1.3e-058.8e-051.5e-05

3e-064.1e-052e-06

3e-064.1e-052e-06

1e-054.7e-051.3e-05

1e-054.7e-051.3e-05

1.6e-050.000132.5e-05

1.2e-057.8e-052.1e-05

8e-063.6e-051.3e-05

4e-064.2e-058e-06

4e-065.2e-054e-06

4e-065.2e-054e-06

6e-063.4e-055e-06

6e-063.4e-055e-06

6e-063.4e-055e-06

6e-063.4e-055e-06

0.0069510.0052320.0053350.0051190.004550.0067060.003509

6e-065.2e-055e-06

6e-065.2e-055e-06

6e-065.2e-055e-06

1.7e-059e-059e-06

1e-055.1e-054e-06

1e-055.1e-054e-06

7e-063.9e-055e-06

7e-063.9e-055e-06

0.0025860.0017550.0020270.0023090.002350.0024790.000959

7e-064.8e-057e-06

7e-064.8e-057e-06

0.0024560.0017550.0020270.0023090.002030.0023480.000959

0.0024560.0017550.0020270.0023090.002030.0023480.000959

9.2e-050.000140.000102

0

2.7e-052e-053e-05

5.4e-05

1.7e-051.8e-052.2e-05

4.8e-054.8e-055e-05

00

1.2e-055.1e-059e-06

1.2e-055.1e-059e-06

1e-054.1e-055e-06

1e-054.1e-055e-06

6e-061.4e-056e-06

6e-061.4e-056e-06

3e-062.6e-052e-06

3e-062.6e-052e-06

0.0043420.0034770.0033080.002810.0020580.0042130.00255

8e-065.5e-056e-06

8e-065.5e-056e-06

1.1e-054.4e-051.6e-05

6e-062.8e-051.1e-05

5e-061.6e-055e-06

0.0043230.0034770.0033080.002810.0019590.0041910.00255

6e-061.6e-056e-06

9e-061.6e-059e-06

0.0042790.0034770.0033080.002810.0018010.0041490.00255

5e-061.4e-055e-06

5e-062.6e-055e-06

7e-062.4e-057e-06

1e-054.7e-058e-06

2e-061.5e-052e-06

0.0188840.0276490.0232230.0255950.0250360.0149940.045956

0.0001970.0006210.0002019.4e-05

1.1e-056.1e-051.5e-05

1.1e-056.1e-051.5e-05

0.0001860.000560.0001869.4e-05

0.0001260.0004710.000127

9.4e-05

6e-058.9e-055.9e-05

0.0012130.0016180.0009090.0061230.0037010.0009870.00966

2.3e-058.8e-052.2e-05

2.3e-058.8e-052.2e-05

0.000747

0.000747

5.7e-050.000189

0.000189

5.7e-05

0.0002012.3e-050.0004776.5e-050.005971

0.0002012.3e-050.0004776.5e-05

0.002075

0.000841

0.003055

0.0004610.0005550.000864

0.000864

0.000269

0.000555

0.000192

1.8e-050.0002242.3e-050.000472

0.000456

1.8e-050.0002242.3e-051.6e-05

0.0002170.000490.0011370.0001350.001417

0.00039

0.001281

2.7e-050.0002493e-05

0.000190.000490.0004980.0001050.000136

0.000166

0.000166

0.0007540.0005870.0009090.0061230.0010540.000742

0.0007540.0004030.0009090.0061230.0010540.000742

0.000184

4e-054.9e-052.9e-05

4e-054.9e-052.9e-05

4e-054.9e-052.9e-05

3.8e-059.3e-052.9e-05

3.8e-059.3e-052.9e-05

3.8e-059.3e-052.9e-05

3.7e-055.7e-052.7e-05

3.7e-055.7e-052.7e-05

3.7e-055.7e-052.7e-05

5.4e-050.0001925.4e-05

5.4e-050.0001925.4e-05

5.4e-050.0001925.4e-05

0.0101560.0132180.0093770.0084310.0059460.0097690.017101

0.0099070.0119040.0093770.0084310.0050560.0095250.009828

1.6e-052.4e-051.7e-05

0.00012

5e-065.7e-056e-06

4e-062.7e-056e-06

0.000241

0.0098820.0119040.0093770.0084310.0045870.0094960.009828

4.7e-05

4.7e-05

0.0001050.0001680.000102

0.0001050.0001680.000102

9.2e-050.0001840.000599e-05

6.4e-056.1e-05

0.000184

2.8e-050.0001612.9e-05

0.000429

0.001130.007226

0.001130.007226

5.2e-050.0001325.2e-05

5.2e-050.0001325.2e-05

0.000174

0.000174

0.000174

0.0012880.0007440.0009180.0050190.0063080.0012820.002472

5.1e-05

5.1e-05

1.6e-053e-051.7e-05

1e-06

9e-062.7e-059e-06

7e-062e-068e-06

1.2e-050.0001121.6e-05

6e-065.3e-058e-06

6e-065.9e-058e-06

0.001260.0007440.0009180.0050190.0061150.0012490.002472

1.8e-058e-062.5e-05

0.000150.0002530.0002190.0015060.0001480.0001450.00016

0.0018320.001423

7e-06

3.7e-053.7e-053.5e-05

2.1e-052.8e-052.3e-05

5.4e-05

3.6e-052e-053.8e-05

0.0008010.000450.0006990.0016060.0010490.0007760.00029

0.002528

1e-053.5e-051e-05

3.2e-052.8e-053.6e-05

2.6e-05

1e-053e-069e-061.4e-058e-06

2.8e-052.8e-052.9e-05

5.9e-05

2e-051.7e-051.4e-059e-06

1.6e-053e-051.8e-05

6.5e-05

1.6e-054.8e-051.9e-05

0.000582

4.4e-053.8e-050.0019077e-064.5e-05

3.1e-05

2e-05

2.1e-051e-062.2e-05

2e-050.0005290.003411.9e-050.00011

2e-057.3e-051.9e-05

2e-057.3e-051.9e-05

0.000446

0.000446

0.000529

0.000529

0.00011

0.00011

0.002891

0.002891

0.0001260.000590.000105

02.1e-050

03e-060

01e-060

1.7e-05

1.5e-059.4e-051.5e-05

7e-063.2e-057e-06

6e-063.2e-055e-06

2e-063e-053e-06

9e-06

9e-06

1.6e-057e-051.3e-05

1.6e-057e-051.3e-05

1e-054.2e-059e-06

1e-054.2e-059e-06

1.2e-055.2e-051.1e-05

1.2e-055.2e-051.1e-05

5.1e-050.0001834.2e-05

7e-064.6e-055e-06

1.4e-054.8e-051.9e-05

1.2e-052.9e-051.1e-05

4e-062.8e-054e-06

5e-063.2e-053e-06

9e-06

1.3e-050.0001281.5e-05

2e-061.8e-053e-06

4e-063.5e-055e-06

5e-05

7e-062.5e-057e-06

1.3e-050.0001021.1e-05

1e-054.7e-059e-06

1e-054.7e-059e-06

3e-065.5e-052e-06

3e-065.5e-052e-06

0.0057020.011540.0120190.0060220.0037930.0024810.016519

8.8e-050.0001838.4e-05

8.8e-050.0001838.4e-05

9.2e-05

9.2e-05

0.0055920.011540.0055190.0060220.0034090.0023760.010813

0.0055920.011540.0055190.0060220.0034090.0023760.010813

2.2e-050.0001092.1e-05

2.2e-050.0001092.1e-05

0.00650.005706

0.00650.005706

0.1341580.1847560.1232380.1941190.0849440.1232430.178087

0.1046630.1538670.1067180.1529650.0576040.1008620.132939

0.0008270.0007380.0007854.7e-05

0.0008270.0007380.0007854.7e-05

3.2e-053.3e-052e-053.1e-05

2.8e-055.2e-052.7e-05

0.0007670.0006020.000738

5.1e-05

1.6e-05

0.0538110.0600580.0397180.0626320.0263860.0476810.046177

0.0537950.0600580.0397180.0603230.0262690.047650.046177

5.1e-052.5e-050.0029118e-050.00013

0.0112530.0058050.0066850.0038140.0071540.0109230.003735

4.9e-051.1e-054.8e-05

0.02210.0258480.0151620.0122450.0058330.0143590.022682

9.1e-053.8e-059.2e-050.00023

0.000133

5.9e-05

0.000215

5.9e-05

1.8e-05

0.0013250.001080.0012510.0022080.0004830.001290.002062

0.000169

7e-062.8e-052e-067e-064.2e-05

0.0002620.0062234.8e-050.0002510.000139

0.0002570.0010840.0004020.0023090.000150.0002190.001899

9e-06

0.000292

0.0010690.0003310.0004550.002710.0002910.0010210.000159

7.8e-058.8e-057.4e-05

8.1e-057.9e-058.2e-05

0.0001355.2e-050.0001370.00011

1.8e-05

0.0019080.0037990.0034140.0030110.0026390.0044090.00361

0.0014250.0023590.0015610.0025090.0007150.001377

3.7e-057e-064.2e-05

0.0007340.0002350.0004060.0035130.0003840.000710.000144

1.4e-050.0021083.9e-053.9e-05

6e-052.2e-055.7e-05

0.0001110.0001220.0021086.2e-050.0003230.000388

6.2e-05

0.0012210.0046220.001120.0037140.0002870.0009290.004171

0.0089890.008360.0065890.0052190.0030610.008678

5.8e-05

3.7e-05

0.0018450.002388

0.001142

0.000578

7.7e-05

0.000303

3.8e-051.2e-053.8e-051.9e-05

1e-058e-060.0021082e-061e-05

0.000270.0001670.0002640.000495

7.5e-053.7e-057.3e-056e-06

8.7e-059.1e-058.8e-05

0.00017

1.7e-05

0.0020580.0045070.0026730.0036130.0012590.001980.003658

1.6e-050.0023090.0001173.1e-05

1e-052.4e-059e-06

6e-060.0023092.7e-052.2e-05

6.6e-05

0.0351550.0846930.0512390.0719660.020530.0380870.07832

9.9e-050.0001089.1e-05

2.4e-05

7.1e-054e-056.2e-05

2.8e-054.4e-052.9e-05

0.0350560.0846930.0512390.0719660.0204220.0379960.07832

0.0006060.0008190.000760.0017060.0003230.0005880.001351

9.1e-05

0.0014190.0005490.0006460.0049180.0001470.0014270.002908

0.001405

8.3e-056e-050.0036134.9e-057.8e-051.6e-05

6.4e-059.6e-056.9e-051.5e-05

0.0211610.0531850.0268670.0286060.0119830.0203360.048232

8.4e-058.9e-05

3.9e-050.0017063.1e-054e-05

8.3e-050.0001379.1e-05

2.3e-05

2.1e-053.1e-050.0060590.005325e-061.9e-05

0.000151

0.000284

7.8e-05

2.9e-053.2e-053.3e-05

2.8e-050.0056020.0049185.8e-052.7e-05

3e-053.5e-053.1e-05

0.0022040.0045860.0039310.0034130.0025780.0063050.004403

0.0038140.0088680.0029180.0058220.0007160.0036760.007828

8.7e-05

5e-065.6e-056e-064.2e-05

2.5e-05

0.00292

9.7e-057.4e-059.4e-05

6.6e-055.5e-050.0024092.5e-056.7e-05

9.4e-05

0.000152

2.6e-05

6.7e-05

2.8e-05

0.0002840.0072020.0005710.0035130.0002450.0002740.003396

5.8e-05

2.9e-050.0001312.4e-05

0.004910.0093380.0038850.0060220.0010730.0047220.007209

5.9e-05

0.0090780.0057620.0110880.0102370.0041210.0087430.005257

1.9e-053.7e-051.9e-05

1.9e-053.7e-051.9e-05

0.0078960.004410.0097370.008230.0030990.0076050.004307

2.4e-051.5e-052.5e-05

0.000684

0.0077660.0043390.0048210.0039140.0022880.007480.004307

1.2e-05

5.3e-056.2e-055.2e-05

3.5e-054.3e-050.0049160.0043162.2e-053.4e-05

1.8e-052.8e-051.6e-051.4e-05

0.0011630.0013520.0013510.0020070.0009850.0011190.00095

0.0011630.0013520.0013510.0020070.0009850.0011190.00095

0.0057190.0033540.0046730.008130.005530.0055080.003138

0.0029210.0018480.0024340.0022080.0027330.0028070.001349

0.0029210.0018480.0024340.0022080.0026720.0028070.001349

4.6e-05

1.5e-05

2.6e-056e-052.1e-05

2.6e-056e-052.1e-05

0.0027720.0015060.0022390.0059220.0027370.002680.001789

0.0026210.0015060.0022390.0021080.0021440.0025330.00149

4.5e-050.0021083.5e-055e-05

3.7e-052.9e-052.5e-05

2e-052.8e-051.8e-05

0.0004620.000299

3.7e-052.8e-054.3e-05

1.2e-050.0017061.1e-051.1e-05

7.3e-050.0002995.8e-05

7.3e-050.0002995.8e-05

5.9e-05

2.7e-05

8e-06

0.000104

6.1e-05

7.3e-054e-055.8e-05

0.0294950.0308890.016520.0411540.027340.0223810.045148

0.0005798.4e-050.002610.0007630.0006130.001937

0.000102

0.000102

0.0005428.4e-050.002610.0005760.0005810.001937

0.001937

8.6e-05

5.9e-051.2e-056.2e-05

4e-052.2e-054.4e-05

0.0002227.4e-050.0002550.000229

2e-06

1e-05

3e-061e-050.002616e-061.8e-05

4.8e-052e-054.8e-05

2.9e-051.5e-053e-05

4.2e-05

1.3e-05

1.1e-05

1.6e-059e-061.9e-05

3.3e-053.6e-053.5e-05

6e-06

4.5e-054e-064.8e-05

4.7e-057e-064.8e-05

1.3e-05

7e-06

3.7e-058.5e-053.2e-05

3.7e-058.5e-053.2e-05

2.7e-055.2e-052.2e-05

2.7e-055.2e-052.2e-05

2.7e-055.2e-052.2e-05

0.0001260.0005220.0001321.6e-05

8.1e-05

8.1e-05

0.0001260.0004410.0001321.6e-05

1.5e-055e-061.3e-05

1.5e-051.4e-051.7e-05

1.3e-051.5e-051.3e-05

5e-06

8.3e-050.0004028.9e-051.6e-05

3.7e-050.0012624.2e-050.000269

0.000145

0.000145

0.000309

1.9e-05

2.9e-05

4.5e-05

5.1e-05

4.4e-05

2.3e-05

2.4e-05

4.8e-05

2.6e-05

0.000269

0.000269

0.0003240.000269

0.000269

0.000324

3.7e-056.7e-054.2e-05

3.7e-056.7e-054.2e-05

0.000148

0.000148

4.5e-052e-060.002613.6e-055.7e-05

4.5e-052e-060.002613.6e-055.7e-05

1e-062e-060.002611e-061e-06

4e-067e-066e-06

1.7e-059e-062.1e-05

1.9e-051.6e-052.3e-05

4e-063e-066e-06

0.0022520.0021430.0030980.0025090.0050210.0043370.00216

0.0022450.0021430.0030980.0025090.0049620.0043320.00216

0.0022450.0021430.0030980.0025090.0049620.0043320.00216

7e-065.9e-055e-06

7e-065.9e-055e-06

0.0052110.0028630.004060.0048180.0073120.0050570.001208

2.4e-050.002062.3e-050.001137

2.4e-058.1e-052.3e-05

0.0019790.001137

0.0001

0.0001

1.7e-055.6e-051.7e-05

1.7e-055.6e-051.7e-05

0.005170.0028630.004060.0048180.0050960.0050177.1e-05

6.8e-05

6e-062.7e-055e-06

1e-05

2.2e-05

1.4e-05

9.8e-05

5.4e-05

2.2e-05

8.2e-05

1.7e-057.5e-051.8e-05

1.5e-05

5.5e-05

2.7e-057.2e-052.6e-05

9e-06

3.6e-05

1.9e-05

0.0001450.0001393.7e-057.5e-057.1e-05

6e-06

8e-06

1.1e-05

2.4e-05

1.2e-05

0.0003670.0001070.0017060.0002440.000458

6.6e-05

2.5e-05

1.7e-05

7.6e-05

0.0046080.0026170.004060.0031120.0038920.004435

0.0212180.0257970.0093620.0286070.0123720.0121210.039558

0.000107

0.000107

0.0016670.0011840.0017460.0018070.0015620.001604

0.0016670.0011840.0017460.0018070.0015620.001604

8e-066e-067e-06

8e-066e-067e-06

9.5e-05

9.5e-05

0.0017440.0005750.0006840.0021080.0027620.0017880.000159

0.0017440.0005750.0006840.0021080.0027620.0017880.000159

5.9e-050.0001966.2e-05

0

5.4e-05

9.7e-05

5.9e-054.5e-056.2e-05

0.0137480.0213570.003680.0222830.0046030.0048010.03924

0.000187.9e-050.0021082e-050.000180.020843

0.000514

6.1e-050.0022081.2e-056.3e-05

0.000247

1e-053e-061.8e-05

0.0003640.0036110.0009970.0024092.8e-050.0005380.002387

5e-06

3.8e-050.0001014e-05

0

2e-050.0001093e-05

4.1e-050.0002115.2e-05

6.3e-050.0014120.0023099e-066.6e-050.001709

0

3.2e-054.9e-054.5e-05

9e-06

3.4e-052.6e-054.1e-05

0.000239

0.0003520.0052910.0043161.5e-050.0003430.001086

0.000282

0.00031

3.2e-05

0.0006510.0001910.002819.1e-050.000655

3.4e-051.6e-054.9e-05

1.2e-05

0.0013790.006180.0006340.002610.0001130.0018750.0074

0.0001351e-060.000191

0.00022.9e-051.8e-050.000217

7.8e-05

0.000174

2.8e-05

2e-05

0.000226

0.0001116e-060.000112

0.000263

0.000278

7.9e-054.8e-058.8e-05

6.9e-05

1.9e-054.1e-052.4e-05

0.0001384.5e-050.0002640.000148

5e-05

2.2e-058.2e-052.6e-05

0.0097850.0045190.0020490.0035130.00050.005815

2e-06

2e-06

0.003980.0026810.0032520.0024090.0024180.0038430.000159

02.8e-05

6e-063e-065e-06

8e-06

2.2e-05

3.5e-054.8e-054.1e-05

2e-06

1e-065e-061e-06

1e-067e-064e-067e-06

4e-06

2e-063e-062e-068e-05

1.2e-05

02.8e-05

2.9e-053e-052.8e-058e-06

9e-06

0.0039020.0026810.0032520.0024090.0022680.003756

4e-065e-066e-06

0.000116

0.000116

0.000144

3e-05

8e-06

0.000106

1.2e-050.0001041.6e-05

1.2e-050.0001041.6e-05

0.00011

0.00011

0.000149

8.5e-05

6.4e-05

0.0002570.0084480.0021590.000250.007432

0.0002570.0084480.0021590.000250.007432

0.0002570.0084480.0021590.000250.007432

1e-057.8e-057e-06

1e-051.6e-057e-06

6.2e-05

0.000155

0.000155

0.0084480.0018360.007416

0.0084480.007416

0.001836

9e-05

9e-05

1.6e-05

1.6e-05

0.0002470.000243

0.0002470.000243

0.0060590.0035710.0239180.0045170.0135480.0058120.014796

0.0057280.0035710.0049770.0045170.0053970.0055040.003144

0.0057280.0035710.0049770.0045170.0053970.0055040.003144

0.0057280.0035710.0049770.0045170.0053970.0055040.003144

0.0036180.0023340.0030040.0024090.0033940.0034770.001745

0.002110.0012370.0019730.0021080.0020030.0020270.001399

4.9e-050.0002964.6e-05

4.9e-050.0002964.6e-05

2e-058e-052.1e-05

2e-058e-052.1e-05

0.000103

0.000103

2.9e-050.0001132.5e-05

2.9e-050.0001132.5e-05

0.0002820.0189410.0078550.0002620.011652

0.001365

0.001365

0.001242

0.000123

0.0002820.0189410.006490.0002620.011652

0.0002820.0189410.006490.0002620.011652

0.000549

6.7e-050.0001367.4e-05

0.0132720.011652

0.0056690.004977

0.000587

0.0002150.0002410.000188

0.0020260.0108910.0039940.0474760.0021420.001890.065025

0.0020260.0108910.0039940.0474760.0021420.001890.065025

1.8e-050.0001161.6e-05

1e-054.5e-059e-06

9e-062.1e-059e-06

9e-069e-06

2.1e-05

1e-062.4e-050

1e-062.4e-050

6e-063.3e-055e-06

6e-063.3e-055e-06

6e-063.3e-055e-06

2e-063.8e-052e-06

2e-063.8e-052e-06

2e-063.8e-052e-06

0.0020080.0108910.0039940.0474760.0020260.0018740.065025

0.0020080.0108910.0039940.0474760.0020260.0018740.065025

0.0020080.0108910.0039940.0474760.0020260.0018740.065025

2e-05

0.000290.0014710.0004820.0024090.0001950.0002480.002108

1.6e-051.2e-051.7e-05

2.6e-05

1e-063e-061e-06

5e-061.9e-052e-06

1e-066e-061e-06

01.2e-051e-06

0.0016920.009420.0035120.0450670.0017230.0015860.062917

3e-061e-051.8e-05

6.3e-050.0003247.1e-05

6.3e-050.0003247.1e-05

6.3e-050.0003247.1e-05

2.2e-056.8e-052e-05

1.4e-052.1e-051.4e-05

4e-06

1.4e-051.7e-051.4e-05

8e-064.7e-056e-06

8e-064.7e-056e-06

4.1e-050.0002565.1e-05

5.7e-05

5.7e-05

3e-064e-057e-06

3e-064e-057e-06

2.1e-055.3e-052.6e-05

2.1e-055.3e-052.6e-05

1.7e-050.0001061.8e-05

3.2e-05

4.6e-05

1.7e-052.8e-051.8e-05

0.0001169.8e-05

0.0001169.8e-05

0.0001169.8e-05

0.0001169.8e-05

0.0001169.8e-05

0.0001169.8e-05

0.0001169.8e-05

0.0853050.056720.0641090.0496830.0811190.0822410.048169

0.0492620.0268190.0355660.0253930.045130.0476150.022866

0.0029530.002220.0027250.0038140.00240.0028390.001665

0.0029530.002220.0027250.0038140.00240.0028390.001665

0.0029530.002220.0027250.0038140.00240.0028390.001665

0.0008380.0010170.0009910.0019070.0007540.0008080.000642

1e-064e-061e-06

3e-06

0.0008360.0010170.0009910.0019070.0007160.0008060.000642

8e-06

8e-060

1e-061.1e-051e-06

4e-06

0.0021150.0012030.0017340.0019070.0016410.0020310.001023

5e-068e-064e-06

4e-06

4e-060

7e-06

3e-06

5e-06

07e-060

7e-06

2e-06

4e-06

03e-060

3e-06

04e-060

2e-065e-062e-06

5e-06

6e-06

0.0021080.0012030.0017340.0019070.0015640.0020250.001023

5e-06

5e-06

0.0019330.0011340.0018990.0019070.0018840.001857

0.0019330.0011340.0018990.0019070.0018840.001857

0.0019330.0011340.0018990.0019070.0018840.001857

0.0019330.0011340.0018990.0019070.0018840.001857

1e-062.2e-051e-06

0.0019320.0011340.0018990.0019070.0018620.001856

3.8e-050.0002253.8e-05

3.6e-050.0001213.6e-05

3.3e-057.1e-053.2e-05

3.3e-057.1e-053.2e-05

5e-062.6e-055e-06

2.8e-054.5e-052.7e-05

1e-061.9e-052e-06

1e-061.9e-052e-06

1e-061.9e-052e-06

2e-061.7e-052e-06

2e-061.7e-052e-06

2e-061.7e-052e-06

01.4e-050

01.4e-050

01.4e-050

3.1e-05

3.1e-05

3.1e-05

3.1e-05

2e-067.3e-052e-06

2e-067.3e-052e-06

2e-062e-052e-06

2e-062e-052e-06

2.4e-05

2.4e-05

02.9e-050

02.9e-050

0.0041090.0024380.0031620.0025090.0040570.0039610.001729

0.0040630.0024380.0031620.0025090.0037080.0039020.001729

0.0040630.0024380.0031620.0025090.0037080.0039020.001729

0.0040630.0024380.0031620.0025090.0037080.0039020.001729

0.0040620.0024380.0031620.0025090.0036870.00390.001729

1e-062.1e-052e-06

1.2e-050.0002291.3e-05

5e-065.6e-055e-06

5e-065.6e-055e-06

2e-062.9e-052e-06

3e-062.7e-053e-06

7e-060.0001738e-06

1e-061.4e-051e-06

1e-061.4e-051e-06

2.2e-050

9e-06

1.3e-050

4e-061.5e-054e-06

4e-061.5e-054e-06

2e-061.9e-052e-06

2e-061.9e-052e-06

06.5e-050

1e-06

9e-06

02e-060

3e-06

02e-060

1.3e-05

1e-05

4e-06

1e-06

05e-060

4e-06

1.1e-05

02.6e-050

02.6e-050

01.2e-051e-06

01.2e-051e-06

3.4e-050.000124.6e-05

1.8e-053.9e-053e-05

1.7e-052.6e-052.9e-05

1.6e-051.4e-051.6e-05

1e-061.2e-051.3e-05

1e-061.3e-051e-06

1e-061.3e-051e-06

01.3e-050

01.3e-050

01.3e-050

7e-062.3e-057e-06

7e-062.3e-057e-06

7e-062.3e-057e-06

9e-064.5e-059e-06

9e-062.8e-059e-06

2e-061e-053e-06

4e-06

7e-061.4e-056e-06

01.7e-050

01.7e-050

4e-067.4e-054e-06

4e-067.4e-054e-06

4e-067.4e-054e-06

2e-05

1.1e-05

9e-06

4e-064.4e-054e-06

1e-061.2e-051e-06

1e-061.3e-051e-06

2e-061.3e-052e-06

06e-060

01e-05

01e-05

0.0322190.01670.0220.0122450.0296310.03120.015939

0.0322190.01670.0220.0122450.0296310.03120.015939

0.0322170.01670.0220.0122450.0296180.0311980.015939

01.9e-05

2e-06

03e-06

1.4e-05

0.0005880.000370.0005090.0016060.0005520.0005660.000207

0.0005880.000370.0005090.0016060.0005520.0005660.000207

3e-064.7e-053e-06

1e-061.1e-051e-06

3e-06

2e-061.1e-052e-06

02.2e-050

0.0316260.016330.0214910.0106390.0290.0306290.015732

1e-053.5e-059e-06

0.000112

3.4e-05

8e-063.3e-058e-06

0.000144

0.0316080.016330.0214910.0106390.0286420.0306120.015732

2e-061.3e-052e-06

2e-061.3e-052e-06

2e-061.3e-052e-06

0.0079950.0043270.005780.0049180.006780.0077040.003533

0.0001010.0002490.000101

0.0001010.0002490.000101

1e-052.6e-051.1e-05

1e-052.6e-051.1e-05

1.3e-054.2e-051.1e-05

1.3e-054.2e-051.1e-05

1.2e-052.1e-051.1e-05

1.2e-052.1e-051.1e-05

9e-062.2e-051e-05

9e-062.2e-051e-05

1.4e-053e-051.4e-05

1.4e-053e-051.4e-05

2.6e-05

2.6e-05

1e-052.4e-051.1e-05

1e-052.4e-051.1e-05

3.3e-055.8e-053.3e-05

1.8e-053e-051.9e-05

1.5e-052.8e-051.4e-05

0.0027580.0014310.0020180.0021080.0018690.0026620.001418

0.0027390.0014310.0020180.0021080.00170.0026420.001418

1e-055.9e-051.1e-05

1e-055.9e-051.1e-05

1.2e-055.1e-051.2e-05

1.2e-055.1e-051.2e-05

0.0027170.0014310.0020180.0021080.001590.0026190.001418

0.000199

0.0026840.0014310.0020180.0021080.0013340.0025870.001418

3.3e-055.7e-053.2e-05

1.9e-050.0001692e-05

1.9e-050.0001692e-05

1.9e-050.0001692e-05

0.0051360.0028960.0037620.002810.0046620.0049410.002115

1.7e-050.0001481.7e-05

7e-064.8e-057e-06

7e-064.8e-057e-06

1e-050.00011e-05

4.1e-05

3.7e-05

1e-052.2e-051e-05

0.0051190.0028960.0037620.002810.0044890.0049240.002115

5.9e-050.0001535.9e-05

2.7e-057.4e-052.7e-05

3.2e-057.9e-053.2e-05

1.2e-052.1e-051e-05

4e-06

5e-061e-064e-06

7e-061.6e-056e-06

1.3e-054.6e-051.6e-05

1.3e-054.6e-051.6e-05

0.0050350.0028960.0037620.002810.0042690.0048390.002115

0.0050250.0028960.0037620.002810.0042290.0048280.002115

1e-054e-051.1e-05

2.5e-05

2.5e-05

2.5e-05

1.1e-057.9e-051.2e-05

1.1e-057.9e-051.2e-05

7e-064.6e-058e-06

7e-063.9e-058e-06

3e-065e-063e-06

01e-051e-06

2e-061.4e-052e-06

2e-061e-052e-06

07e-060

07e-060

4e-063.3e-054e-06

3e-062.7e-053e-06

02e-061e-06

2e-067e-061e-06

01e-060

1e-067e-061e-06

8e-06

2e-06

1e-066e-061e-06

1e-066e-061e-06

5e-060.0001124e-06

5e-060.0001124e-06

8.2e-05

8.2e-05

4.9e-05

4.9e-05

3.3e-05

3.3e-05

5e-063e-054e-06

5e-063e-054e-06

5e-063e-054e-06

5e-062.1e-054e-06

9e-06

0.0360170.0299010.0285430.024290.0357690.0346070.025303

0.0360170.0299010.0285430.024290.0357690.0346070.025303

0.002010.003080.0016320.0042160.002640.0019320.002482

6e-062.1e-054e-06

6e-062.1e-054e-06

1e-06

8e-06

00

6e-061.2e-054e-06

0.0020040.003080.0016320.0042160.0026190.0019280.002482

1.6e-050.0001671.7e-05

5e-066.9e-056e-06

1.1e-059.8e-051.1e-05

1.7e-050.0019470.0023090.0005261.6e-050.001663

1.7e-050.0005261.6e-05

0.0019470.0023090.001663

1e-062e-052e-06

1e-061.1e-051e-06

09e-061e-06

0.001970.0011330.0016320.0019070.0019060.0018930.000819

0.001970.0011330.0016320.0019070.0019060.0018930.000819

0.0323440.0184530.0240220.0145540.0316290.0310760.014975

0.0323440.0184530.0240220.0145540.0315970.0310760.014975

02.8e-051e-06

01.8e-051e-06

01e-050

7e-06

2e-06

5e-06

1e-061.7e-051e-06

1e-061.7e-051e-06

1e-061.8e-051e-06

1e-061.8e-051e-06

0.0323420.0184530.0240220.0145540.0315270.0310730.014975

0.0323420.0184530.0240220.0145540.0315270.0310730.014975

03.2e-050

01e-050

01e-050

09e-060

09e-060

1.3e-05

1.3e-05

1e-062.4e-051e-06

1e-061e-051e-06

1e-061e-051e-06

1e-061e-051e-06

1.4e-05

1.4e-05

1.4e-05

0.0016620.0083680.0028890.005520.0014760.0015980.007846

0.0016620.0083680.0028890.005520.0014760.0015980.007846

01e-05

08e-06

2e-06

0.0016620.0083680.0028890.005520.0014510.0015980.007846

03e-060

1.5e-05

1e-061.4e-051e-06

2e-067e-061e-063.3e-05

0.0016590.0083680.0028890.005520.0014120.0015960.007813

01.5e-050

01.5e-050

2e-057.8e-051.4e-05

2e-057.8e-051.4e-05

2e-057.8e-051.4e-05

2e-057.8e-051.4e-05

2e-057.8e-051.4e-05

2e-057.8e-051.4e-05

1e-063e-051e-06

1e-063e-051e-06

1e-063e-051e-06

1e-063e-051e-06

1e-063e-051e-06

1e-063e-051e-06
