# Supplementary material for: MetaMeta: integrating metagenome analysis tools to improve taxonomic profiling
Source: Microbiome. 2017 Aug 14;5:101. doi: 10.1186/s40168-017-0318-y (PMC5557516; doi:10.1186/s40168-017-0318-y)
Supplement: Supplementary file 2 — Additional File with interactive charts for all CAMI toy set results on default, very-precise and very-sensitive mode. File prefix S, M, and H for low, medium and high complexity, respectively. (TAR 3573 kb) [file 40168_2017_318_MOESM2_ESM.tar › M1_S001__insert_5000_very-precise.html]

Javascript must be enabled to view this page.

magnitude
magnitudeUnassigned

clark.parsed\_profile
dudes.parsed\_profile
final.metametamerge.profile
gottcha.parsed\_profile
kaiju.parsed\_profile
kraken.parsed\_profile
motus.parsed\_profile

1.0000040.9999979999999990.9999989999999990.9999991.0000020.9999971

0.0038840.0018770.001080.032460.0061630.003903

3.8e-050.0002044.1e-05

3.8e-050.0002044.1e-05

3.8e-050.0002044.1e-05

3.8e-050.0002044.1e-05

3.8e-050.0002044.1e-05

3.8e-050.0002044.1e-05

9e-067.4e-059e-06

9e-067.4e-059e-06

4.1e-05

4.1e-05

3.2e-05

3.2e-05

9e-06

9e-06

9e-063.3e-059e-06

9e-063.3e-059e-06

9e-063.3e-059e-06

1e-06

9e-063.2e-059e-06

3.6e-050.0002933.6e-05

3.6e-050.0002933.6e-05

0.000111

7.4e-05

2.6e-05

4e-06

2.2e-05

2e-05

2e-05

8e-06

8e-06

2e-05

5e-06

1.5e-05

3.7e-05

3.7e-05

2.4e-05

1.2e-05

1e-06

9.8e-05

6.4e-05

2.1e-05

1.8e-05

3e-06

3.1e-05

3.1e-05

7e-06

7e-06

2e-06

2e-06

3e-06

1e-06

2e-06

3.4e-05

7e-06

7e-06

1.7e-05

1.7e-05

1e-05

1e-05

3e-053.2e-052.9e-05

1.3e-05

1.3e-05

1.3e-05

3e-051.9e-052.9e-05

3e-051.9e-052.9e-05

3e-051.9e-052.9e-05

6e-065.2e-057e-06

6e-065.2e-057e-06

2.7e-05

2.5e-05

2e-06

4e-062.2e-057e-06

2e-069e-064e-06

2e-06

0

2e-061e-063e-06

1e-05

2e-063e-06

2e-063e-06

2.5e-05

2.5e-05

2.5e-05

2.5e-05

2.5e-05

2.5e-05

0.0038010.0018770.001080.032460.0055670.003817

2.5e-050.000113.1e-05

2.5e-050.000113.1e-05

7e-066.2e-059e-06

3e-062e-053e-06

3e-062e-053e-06

4e-064.2e-056e-06

4e-068e-064e-06

1e-05

1.4e-05

1e-052e-06

1.8e-054.8e-052.2e-05

1.8e-054.1e-052.2e-05

4e-06

1e-06

1e-05

1.1e-051e-068e-06

7e-062.2e-051.4e-05

3e-06

7e-06

7e-06

0.0006430.0004320.010820.0005060.000626

0.0006430.0004320.010820.0005060.000626

0.0006430.0004320.010820.0005060.000626

8e-062.2e-058e-06

8e-063e-068e-06

1e-06

5e-06

1e-06

1e-06

5e-06

6e-06

0.0006350.0004320.010820.0004650.000618

6e-06

2e-06

1e-05

2e-06

2e-06

8e-06

6e-06

4e-06

4e-06

6e-06

3e-06

3e-06

5e-061e-062e-06

7e-06

3e-06

0.0006280.0004320.010820.0003820.000614

2e-061.6e-052e-06

1.9e-05

1.9e-05

0.0009270.0006710.001080.010820.0007330.000906

0.0009270.0006710.001080.010820.0007330.000906

0.0009270.0006710.001080.010820.0007330.000906

0.0009270.0006710.001080.010820.0007330.000906

0.0009270.0006710.001080.010820.0007220.000906

1.1e-05

0.001890.0006350.010820.0031590.001913

0.0005930.0002270.0010750.000611

6.5e-050.0004979.5e-05

6.5e-050.0004979.5e-05

6.5e-050.0004979.5e-05

0.0005280.0002270.0005780.000516

3.3e-050.000143.7e-05

3.3e-050.000143.7e-05

0.0004730.0002270.0002870.000451

4e-05

4.4e-057.6e-054.3e-05

0.0004290.0002270.0001710.000408

2.2e-050.0001512.8e-05

2.2e-050.0001512.8e-05

0.0009560.0004080.010820.0015040.000955

7.8e-05

7.8e-05

7.8e-05

4.6e-050.0003544.7e-05

1.7e-050.0001281.3e-05

1.7e-050.0001281.3e-05

2.9e-050.0002263.4e-05

8.3e-05

8.9e-05

2.9e-055.4e-053.4e-05

0.000910.0004080.010820.0010720.000908

0.0006210.0004080.010820.000510.000613

1.9e-057e-052.1e-05

0.0006020.0004080.010820.000440.000592

4.9e-050.0001143.2e-05

4.9e-050.0001143.2e-05

0.0002030.0003990.00023

7.5e-050.0001919.8e-05

0.0001280.0002080.000132

3.7e-054.9e-053.3e-05

1e-05

1.5e-053e-061.2e-05

2.2e-053.6e-052.1e-05

0.0003410.000580.000347

0.0003410.000580.000347

1.9e-056.9e-052.5e-05

1.9e-056.9e-052.5e-05

6e-05

6e-05

4.9e-058e-055.2e-05

4.9e-058e-055.2e-05

3.8e-050.0001362.7e-05

3.8e-050.0001362.7e-05

7.6e-050.0001018.1e-05

5.3e-055.8e-055.3e-05

2.3e-054.3e-052.8e-05

6.3e-055.9e-057e-05

6.3e-055.9e-057e-05

3.8e-053.7e-053.8e-05

3.8e-053.7e-053.8e-05

5.8e-053.8e-055.4e-05

5.8e-053.8e-055.4e-05

2e-069e-052e-06

2e-069e-052e-06

2e-069e-052e-06

2e-05

2e-05

2e-064e-052e-06

7e-06

9e-06

1.1e-05

2e-061.3e-052e-06

3e-05

1.9e-05

1.1e-05

1.8e-050.0001811.4e-05

1.8e-050.0001811.4e-05

2e-05

2e-05

2e-05

1.8e-050.0001611.4e-05

2e-062.4e-052e-06

2e-069e-062e-06

5e-06

5e-06

5e-06

3e-061.9e-05

8e-06

3e-067e-06

4e-06

6e-061.5e-055e-06

6e-061.5e-055e-06

7e-060.0001037e-06

1.8e-05

3.2e-05

7e-06

1.3e-05

2e-061.3e-052e-06

5e-062e-055e-06

0.0002590.0001390.0005750.00029

1.4e-056.1e-051e-05

1.4e-056.1e-051e-05

1.4e-056.1e-051e-05

6e-063.3e-054e-06

8e-062.8e-056e-06

0.0002160.0001390.0002740.000217

0.0001580.0001390.0001330.000155

0.0001580.0001390.0001330.000155

0.0001580.0001390.0001330.000155

3.2e-056.2e-053.1e-05

5e-063.1e-055e-06

5e-063.1e-055e-06

2.7e-053.1e-052.6e-05

2.3e-051.3e-052.2e-05

4e-061.8e-054e-06

1.1e-052.3e-053e-06

1.1e-052.3e-053e-06

1.1e-052.3e-053e-06

1.5e-055.6e-052.8e-05

1.2e-053.6e-052.6e-05

7e-06

4e-068e-061.2e-05

8e-062.1e-051.4e-05

3e-062e-052e-06

3e-062e-052e-06

2.9e-050.000246.3e-05

1.2e-055.4e-053.6e-05

1.2e-055.4e-053.6e-05

2e-061.6e-052e-06

1e-053.8e-053.4e-05

1.7e-050.0001862.7e-05

6e-066e-068e-06

6e-066e-068e-06

3.7e-057e-06

3.7e-057e-06

2e-05

2e-05

1.8e-05

1.8e-05

9e-065.7e-058e-06

0

1.3e-05

1e-06

2e-06

8e-06

8e-06

3e-06

1.7e-05

7e-062e-066e-06

1e-061e-06

2e-062e-061e-06

2e-061.7e-054e-06

2e-061.7e-054e-06

3.1e-05

1.4e-05

1.7e-05

3.7e-050.0002133.5e-05

3.1e-05

3.1e-05

3.1e-05

3.1e-05

2.8e-057.4e-053.5e-05

1.2e-052.4e-059e-06

1.2e-052.4e-059e-06

3e-061.2e-05

9e-061.2e-059e-06

1.7e-05

1.7e-05

1.7e-05

3e-061.4e-053e-06

3e-061.4e-053e-06

3e-061.4e-053e-06

1.3e-051.9e-052.3e-05

1.3e-051.9e-052.3e-05

1.3e-051.9e-052.3e-05

9e-060.000108

9e-060.000108

3.7e-05

3.7e-05

9e-063.4e-05

9e-063.4e-05

3.7e-05

3.7e-05

0.996120.9981209999999990.9989189999999990.9675390.9938390.9960941

0.1809890.2183960.2975130.2518980.3489460.1787470.342922

0.1270150.1718150.2368870.2047750.2823350.1275720.301158

0.075960.1249060.19570.1009210.2465830.0798330.271413

0.0010.0010530.0017980.010820.0019550.001967

6e-069.1e-053e-06

6e-069.1e-053e-06

0.0009940.0010530.0017980.010820.0018640.001964

0.0009940.0010530.0017980.010820.0018640.001964

0.0470840.0286310.1838920.0154720.2339070.0462280.103772

0.000122

0.000122

0.0007318.1e-050.1524010.2016560.0007630.103772

0.1524010.2014410.103772

0.0007318.1e-050.0002150.000763

7e-059.1e-056.3e-05

7e-059.1e-056.3e-05

0.0462830.028550.0314910.0154720.0320380.045402

3.6e-05

1.7e-05

4.3e-056.9e-054.1e-05

8.1e-056.1e-059e-05

2.1e-053e-052.1e-05

0.0001066e-053.2e-055.1e-05

3.3e-05

4.2e-05

9.5e-05

8e-06

4.4e-05

0.0005310.0001260.0001440.000611

5e-05

8e-06

0.000122

1.6e-05

1.8e-05

1.8e-05

5.7e-05

2.5e-05

6.6e-05

1.7e-05

0.0455010.0283640.0314910.0154720.0309070.044588

7e-05

2.1e-05

6e-06

1.1e-05

1.5e-05

2.5e-054.7e-052.1e-05

2.5e-054.7e-052.1e-05

2.5e-054.7e-052.1e-05

0.0271130.095220.010010.0746290.009243999999999990.0307440.167641

6.6e-05

6.6e-05

0.0074540.0058390.0066860.0112250.0035340.007345

2e-06

0.0073390.0058390.0066860.0112250.0033750.00719

1.9e-05

5e-062.5e-051.2e-05

1.1e-052e-061.7e-05

1.1e-052.5e-052e-05

1e-06

5e-063e-064e-06

8.1e-053.4e-059e-05

2.2e-05

4e-06

1.1e-05

1e-062e-069e-06

1e-069e-063e-06

1.6e-057.2e-052.2e-05

1.6e-057.2e-052.2e-05

0.0172730.0888650.0033240.0634040.0029870.0209360.167365

6.2e-05

7.5e-055.7e-058.2e-05

0.0001268e-06

0.0024320.0325150.0006560.0178990.0001090.0030670.005837

5.3e-05

3e-061e-064e-06

7.7e-051.3e-057.6e-05

8.3e-05

5e-06

6.6e-05

5.8e-056e-055.8e-05

0.0090.053340.0008440.0159770.0001310.0118320.035012

1.2e-05

2.1e-055e-052.9e-05

6.8e-05

0.0001710.0001160.000179

6.7e-051.2e-056.6e-05

0.0009865.1e-050.0011196.1e-050.001027

0.00045.1e-055.4e-050.000427

3e-053.8e-053.8e-05

6.4e-05

8.4e-05

5e-06

0.000102

0.000684

3e-05

0.0002570.001710.0125396e-060.000255

0.0001368.1e-050.000136

0.002010.0009820.0007050.0169890.0001350.002040.126516

8.3e-05

1.7e-052e-061.8e-05

0.0003719.9e-050.000438

0.000770.000180.0001960.000873

0.0001393.2e-053.5e-050.000132

3e-06

6e-064e-063e-065e-06

8.2e-05

0.0001211e-060.000154

3.1e-05

7.1e-05

1e-06

0.00013

9.8e-05

9.8e-05

9.4e-050.0001350.00011

9.4e-050.0001350.00011

4e-066e-064e-06

4e-066e-064e-06

5.1e-05

5.1e-05

6.6e-05

4.1e-05

2e-05

5e-06

0.0021560.0005160.0020150.0022030.000276

0.0021560.0005160.0004270.0022030.000276

7.4e-05

0.001514

6e-05

3.8e-05

2.2e-05

0.0001167.7e-050.000124

0.0001167.7e-050.000124

7.7e-05

7.7e-05

8.4e-050.0006040.000107

0.000146

1.9e-05

1.3e-05

2.5e-05

2.1e-05

1e-05

1.9e-05

9e-06

1.1e-05

1.9e-05

8.4e-053.5e-050.000107

8.4e-053.5e-050.000107

0.000104

0.000104

0.000112

0.000112

0.0001

0.0001

0.000107

0.000107

0.0004110.0004660.000523

0.0003220.0002070.00043

5e-06

1e-06

1e-0601e-06

1.8e-052e-061.9e-05

1.9e-059e-062e-05

2.8e-05

1.5e-05

6.5e-055e-068.2e-05

4e-06

7.8e-051e-050.000105

1e-05

1.9e-051e-056.5e-05

1.3e-05

2.6e-051e-054e-05

1.5e-055e-061.5e-05

1e-05

5.4e-058e-066.2e-05

2.7e-052e-062.1e-05

6e-05

8.9e-050.0001539.3e-05

8.9e-050.0001539.3e-05

0.000106

0.000106

0.0001162e-064.4e-050.0001

0.0001162e-064.4e-050.0001

3.7e-055e-063.4e-05

2e-062e-061e-062e-06

3e-051.4e-052.3e-05

3.3e-051.1e-053.4e-05

1.4e-051.3e-057e-06

0.0001270.0003160.000143

0.000168

0.000168

0.0001270.0001480.000143

6.6e-050.0001066.7e-05

2.4e-052e-052.8e-05

7e-061.6e-057e-06

4e-06

3e-052e-064.1e-05

0.0510550.0469090.0411870.1038540.0357520.0477390.029745

0.0001610.0024550.000132

0.0001610.0024550.000132

0.000102

0.000864

0.0001610.0002730.000132

0.000344

0.000739

0.000133

0.0023420.0040.00120.0334720.0008840.00230.001914

3.9e-050.0001194.8e-05

3.9e-050.0001194.8e-05

0.0022630.0040.00120.0334720.0006540.0022010.001914

8.5e-057.6e-059.3e-05

4.4e-05

2.9e-052.9e-053.5e-05

3.6e-05

0.0013870.0035080.00120.0113260.000320.001360.001645

0.0003080.0002580.0221463.7e-050.000252

0.0004540.0002340.0001120.0004610.000269

4e-050.0001115.1e-05

4e-050.0001115.1e-05

0.0002040.0005880.000176

9.9e-050.0002678.7e-05

8.5e-05

9.9e-050.0001468.7e-05

3.6e-05

4.7e-050.0001114e-05

4.7e-050.0001114e-05

5.8e-050.000214.9e-05

5.3e-05

2e-06

1.3e-054.4e-051.3e-05

2e-06

1.1e-054e-051.4e-05

9e-061.9e-056e-06

2.5e-055e-051.6e-05

0.0065570.0059920.0046320.006431

0.0065570.0059920.0046320.006431

7.4e-050.0001777.2e-05

0.000258

0.006430.0059920.0041160.006307

5.3e-058.1e-055.2e-05

0.0389740.0406030.0331920.059360.0234750.035760.026249

9.7e-050.0005680.000158

7.5e-050.000139.5e-05

2.2e-057.4e-056.3e-05

0.000364

0.0388770.0406030.0331920.059360.0229070.0356020.026249

0.00061

6.8e-053.2e-055.6e-056.4e-05

3.3e-05

0.0005160.000140.000330.000585

0.0001593.1e-050.0001790.000268

0.0001150.0001450.000112

0.0009230.0001960.0001490.0009420.000226

0.0033910.003048

3.9e-05

0.0001352.9e-056e-050.000139

0.0003370.0009310.000355

0.0001960.0002160.000206

0.001553

0.000775

0.000810.0001140.0006950.000837

0.000544

4.7e-057.4e-057.1e-054.3e-05

0.0057540.005171

9.7e-05

8e-06

0.0117860.0147150.0075990.0121350.0025820.007810.007813

0.0003120.000720.000316

0.0008230.0001480.0008090.0008918e-06

0.0003565e-050.000363

0.000859

0.000730.0001850.0001270.0007610.00028

0.0002170.0001340.00021

0.0001999.5e-059e-060.00022

0.000394

9.5e-055e-059.7e-05

4.4e-05

0.0006950.0043570.0016550.011731.7e-050.0006850.00677

0.0001054.8e-050.0120340.0004350.000358

0.0005170.0001030.000450.0005550.000774

0.0002288.7e-050.0006640.00025

2.8e-055.1e-050.000229.1e-05

0.0008280.0001840.0004070.0008468e-06

0.001463

0.0027130.0049260.002970.0113260.0011890.002683

0.000232

3e-050.0001357e-063.2e-050.000316

0.0001630.0002780.0001910.0003310.001452

0.0019340.001739

0.0157460.0146750.0098890.0121350.0035190.015552

0.000488

0.0028170.0023060.0008030.0110220.0037180.002940.001582

0.0026580.0023060.0008030.0110220.0036210.0027910.001582

8.6e-055.6e-058.9e-05

8.1e-05

0.0004040.0002228.8e-050.000409

1.9e-050.0001441.6e-05

2.4e-056e-059.1e-05

0.000927

0.0003510.000357

2.9e-05

3.7e-055.1e-053.7e-05

1.6e-051.1e-051e-05

4e-05

0.0002370.0002260.0001950.000251

2e-05

8.2e-05

0.000455

8.7e-05

8.1e-05

0.0003920.0014080.0008030.0110225.1e-050.0003820.001044

3.3e-05

9.9e-05

0.0001890.0002370.000195

3e-06

7e-05

2e-066e-062e-06

2e-051.8e-051.9e-05

7.9e-054.8e-050.000115

1.1e-053.7e-051e-05

0.000250.0003060.000259

4.5e-053.6e-054.5e-05

2.5e-05

0.0003870.0003770.0001490.0003870.000538

1e-064e-06

0.0001087.3e-054e-050.000117

1.8e-05

3.4e-05

0.0001599.7e-050.000149

9.9e-052.9e-059.7e-05

6e-055.2e-055.2e-05

1.6e-05

0.0008770.0004210.0109210.0014360.000855

8.7e-050.0003918.1e-05

8.7e-050.0002758.1e-05

8.7e-050.0002758.1e-05

5e-05

3.6e-059e-053.2e-05

2.5e-05

1.6e-05

5.1e-059.4e-054.9e-05

0.000116

0.000116

4.5e-05

7.1e-05

7.3e-050.0003498.4e-05

7.3e-050.0003498.4e-05

2.5e-050.0001124.1e-05

2.5e-050.0001124.1e-05

4.8e-050.0001324.3e-05

4.8e-050.0001324.3e-05

0.000105

0.000105

0.0007170.0004210.0109210.0006960.00069

0.0007170.0004210.0109210.0006960.00069

0.0007170.0004210.0109210.0006960.00069

0.0006620.0004210.0109210.000570.000645

5.5e-050.0001264.5e-05

0.0003320.0007780.00036

0.0003320.0007780.00036

0.0003320.0007780.00036

0.000386

0.000386

0.0003070.000337

0.0003070.000337

2.5e-050.0001322.3e-05

9.8e-05

2.5e-053.4e-052.3e-05

0.000111

0.000111

0.000149

0.000149

0.000105

0.000105

0.000105

0.000105

0.000105

0.0505690.0454890.0594730.0362020.0622450.0477720.041764

0.0002330.0008430.000198

1.1e-050.0001188e-06

1.1e-050.0001188e-06

1.1e-050.0001188e-06

8.7e-050.000239.1e-05

4e-067.3e-05

4e-067.3e-05

4.5e-055.6e-055.5e-05

1.6e-053.2e-051.5e-05

2.9e-052.4e-054e-05

3.8e-050.0001013.6e-05

2e-06

1.2e-056e-061.2e-05

1e-06

8e-063.8e-056e-06

1.2e-056e-069e-06

1.5e-05

2e-061e-053e-06

4e-062.3e-056e-06

3.3e-050.00012.7e-05

2.3e-054.7e-052e-05

2.3e-054.7e-052e-05

1e-055.3e-057e-06

1e-055.3e-057e-06

0.0001020.0003957.2e-05

4e-064.2e-053e-06

4e-064.2e-053e-06

4.9e-052.3e-051.6e-05

4.9e-052.3e-051.6e-05

6e-068.2e-054e-06

6e-068.2e-054e-06

4e-063.7e-052e-06

1.8e-05

2e-06

2e-062e-06

2e-061.5e-052e-06

2.7e-058.9e-053.6e-05

2.7e-058.9e-053.6e-05

3e-066.8e-05

3e-066.8e-05

9e-065.4e-051.1e-05

9e-065.4e-051.1e-05

0.0502250.0454890.0594730.0362020.0610360.0474480.041764

0.0001910.0008160.000201

1.9e-058.6e-051.8e-05

1.9e-058.6e-051.8e-05

0.0001720.000730.000183

0.0001320.0006340.000144

4e-059.6e-053.9e-05

0.0001140.0001160.000102

0.0001140.0001160.000102

0.0001140.0001160.000102

0.0001230.0002140.000135

0.0001230.0002140.000135

0.0001230.0002140.000135

0.0427440.0306410.0331050.0138540.0385820.0419090.023098

4.7e-054.3e-055.7e-05

3.2e-054e-053.5e-05

1e-06

1.5e-052e-062.2e-05

0.0426270.0306410.0331050.0138540.0382930.0417650.023098

0.0059950.0050930.00572

3.1e-051.5e-051.3e-054.5e-05

2.1e-052.1e-052.4e-05

0.0417920.0304530.027110.0138540.032260.0408740.016175

5.7e-052.7e-054e-05

9.6e-057.2e-051.7e-050.000104

0.001203

4.8e-053.8e-055.5e-05

1.1e-05

6.1e-052.8e-053.6e-056.9e-05

6e-05

7.1e-05

5.3e-051.4e-055.8e-05

2.4e-05

0.0001093.3e-050.000115

1e-055.6e-051.4e-05

4.4e-053.5e-055e-05

4.5e-058.1e-055.1e-05

0.00013

8.1e-05

7.2e-05

0.0001113.7e-050.000109

2e-05

5.6e-056.2e-056e-05

9.3e-057.3e-051e-069.7e-05

7e-050.0001558.7e-05

3.5e-056.9e-054.5e-05

3.5e-058.6e-054.2e-05

9.1e-05

9.1e-05

0.0002340.0010860.000254

0.0002340.0010860.000254

0.0002340.0010860.000254

0.000347

0.000347

0.000347

0.0006730.0027320.0100590.0039010.0005830.012057

0.0003915.1e-050.0007670.000399

0.0003910.0007670.000399

5.1e-05

3.5e-050.0001412.8e-05

3.5e-050.0001412.8e-05

5.2e-050.0005860.000805

0.000805

5.2e-05

0.000586

0.000223

0.000223

2.6e-050.0039430.0002933.6e-050.003544

2.6e-050.0002933.6e-05

0.0039430.003544

0.0001110.0061160.0004794.1e-050.006764

0.000853

0.0061160.005497

0.000414

0.0001110.0004794.1e-05

0.000110.0024060.0013637.9e-050.000944

0.000405

2.5e-050.0003162.8e-05

8.5e-050.0024060.0005285.1e-050.000539

0.000519

0.000272

0.000272

0.0034650.0078870.0033650.0114270.0020570.0015990.006063

4.7e-050.0001164.8e-05

4.7e-050.0001164.8e-05

0.00012

0.00012

0.0001170.000160.000122

0.0001170.000160.000122

0.0033010.0078870.0033650.0114270.0016610.0014290.006063

0.0033010.0078870.0033650.0114270.0016610.0014290.006063

0.0019340.0012790.0019310.0109210.0020240.001915

0.0017070.0012790.0019310.0109210.0014620.00165

0.0016560.0012790.0019310.0109210.0012720.001619

1e-053.8e-057e-06

1.4e-054.4e-051.2e-05

1.1e-055.3e-056e-06

1e-065.5e-056e-06

1.5e-05

1.8e-05

1.8e-05

4.4e-058.2e-053.7e-05

4.4e-058.2e-053.7e-05

7e-066.7e-052.1e-05

7e-066.7e-052.1e-05

1.7e-05

1.2e-05

2e-06

3e-06

8.1e-050.0002070.000119

3.1e-054.7e-057.2e-05

1.1e-052.8e-059e-06

3.9e-057.8e-053.8e-05

5.4e-05

2.3e-056.9e-053.5e-05

2.3e-056.9e-053.5e-05

5.4e-050.000125.3e-05

4.1e-054.7e-053.8e-05

9e-062.6e-051.1e-05

4e-064.7e-054e-06

6.5e-050.0028370.0110130.0083756.6e-050.000273

0.0078570.007062

0.0078570.007062

6.5e-050.0001216.6e-05

6.5e-050.0001216.6e-05

0.0028370.003156

0.0028370.003156

0.000273

0.000273

0.001192

0.001192

0.0006230.0001130.0033090.0006230.000273

0.000273

0.000273

0.000113

0.000113

0.0002170.0002480.00021

0.0002170.0002480.00021

3.2e-050.0011643.6e-05

4e-064.9e-057e-06

0.000674

0.000264

1e-054.6e-051.2e-05

6e-069.8e-056e-06

1.2e-053.3e-051.1e-05

0.0002790.00160.000276

0.000210.000207

6.9e-050.0003686.9e-05

0.001232

9.5e-050.0002970.000101

9.5e-050.0002970.000101

3.5e-055.9e-053.1e-05

3.5e-055.9e-053.1e-05

3.5e-055.9e-053.1e-05

2.4e-050.000153e-05

2e-055.5e-052.6e-05

2e-055.5e-052.6e-05

4e-069.5e-054e-06

4e-069.5e-054e-06

0.0001020.0003010.000118

5.3e-050.000128e-05

4.5e-055.9e-057.4e-05

4.5e-055.9e-057.4e-05

8e-066.1e-056e-06

8e-066.1e-056e-06

4.9e-050.0001813.8e-05

3e-050.000112.3e-05

1.1e-056.8e-051.1e-05

1.9e-054.2e-051.2e-05

1.9e-057.1e-051.5e-05

1.9e-057.1e-051.5e-05

9e-066.5e-058e-06

9e-066.5e-058e-06

9e-066.5e-058e-06

9e-066.5e-058e-06

0.0021960.0006710.0011530.0020470.002188

0.0021960.0006710.0011530.0020470.002188

0.0021960.0006710.0011530.0020470.002188

0.000331

0.000331

0.000159

0.000159

0.000430.0001250.0004460.000413

0.000430.0001250.0004460.000413

0.0017660.0005460.0011530.0011110.001775

0.0017660.0005460.0011530.0011110.001775

0.0025150.0148230.0035970.0128430.0015170.0021950.016475

0.0025150.0148230.0035970.0128430.0015170.0021950.016475

0.0025030.0148230.0035970.0128430.0013850.0021810.016475

0.0025030.0148230.0035970.0128430.0013850.0021810.016475

0.0025030.0148230.0035970.0128430.0013850.0021810.016475

9e-061.4e-054e-06

5e-062e-064e-06

1.5e-05

0

4e-061.4e-058e-06

1e-067e-062e-06

0.0024710.0148230.0035970.0128430.0012820.002150.016475

9e-063e-069e-06

9e-06

4e-063.9e-054e-06

1.2e-050.0001321.4e-05

2e-061.8e-052e-06

2e-061.8e-052e-06

2e-061.8e-052e-06

2.4e-05

2.4e-05

2.4e-05

1e-059e-051.2e-05

1e-053.8e-051e-05

3.8e-05

1e-051e-05

5.2e-052e-06

5.2e-052e-06

0.0003340.0009320.000336

4e-050.0001013.6e-05

4e-050.0001013.6e-05

3.5e-056.9e-053.4e-05

3.5e-056.9e-053.4e-05

2.3e-053.9e-052.5e-05

1.2e-053e-059e-06

5e-063.2e-052e-06

5e-063.2e-052e-06

0

5e-063.2e-052e-06

2e-050.0002272e-05

3e-061e-053e-06

3e-061e-053e-06

3e-061e-053e-06

3e-061e-053e-06

1.7e-050.0002171.7e-05

1.7e-050.0002171.7e-05

1.7e-050.0002171.7e-05

1.7e-050.0002171.7e-05

3.2e-058.2e-054.3e-05

3.2e-058.2e-054.3e-05

3.2e-058.2e-054.3e-05

3.2e-058.2e-054.3e-05

3.2e-058.2e-054.3e-05

1.3e-050.0002321.1e-05

1.3e-050.0002321.1e-05

1.3e-050.0002321.1e-05

1.3e-058.1e-051.1e-05

1.3e-058.1e-051.1e-05

0.000151

0.000151

0.0002290.000290.000226

7.4e-050.0001255.6e-05

7.4e-050.0001255.6e-05

7.4e-050.0001255.6e-05

7.4e-050.0001255.6e-05

0.0001550.0001650.00017

0.0001550.0001650.00017

0.0001550.0001650.00017

0.0001550.0001650.00017

0.0080890.0099830.006360.0333710.005280.02010.004805

0.0080890.0099830.006360.0333710.005280.02010.004805

0.0006850.0003150.0002350.000695

0.0006850.0003150.0002350.000695

0.0006850.0003150.0002350.000695

0.0002110.0001118e-050.000216

0.0002368.1e-050.0001380.000245

1.6e-051.7e-051.6e-05

0.0002220.0001230.000218

0.0007930.0030650.0008940.0220450.0021160.0007530.001277

0.0003470.0009540.0003320.010820.0001620.0003290.000267

2.7e-051.9e-052.2e-05

6e-06

6e-066e-06

2e-06

2e-06

2.1e-056e-061.6e-05

3e-06

0.000320.0009540.0003320.010820.0001430.0003070.000267

1.1e-055e-063e-062e-05

3e-061e-063e-06

0.0002920.0009540.0003320.010820.000130.0002870.000231

1.6e-05

1.4e-055e-061.4e-05

2e-06

0.0004460.0021110.0005620.0112250.0019540.0004240.00101

4.8e-059e-054.4e-05

1.4e-053.5e-051.3e-05

3.4e-055.5e-053.1e-05

1.5e-050.0002271.1e-05

4e-065.3e-05

4e-067.3e-055e-06

7e-060.0001016e-06

2.9e-054.8e-052e-05

2.9e-054.8e-052e-05

8e-067.2e-056e-06

8e-067.2e-056e-06

0.0003460.0021110.0005620.0112250.0015170.0003430.00101

2e-064.5e-05

5e-066.9e-055e-06

2e-065.9e-054e-06

1.9e-050.0001351.8e-05

2.3e-05

7.8e-054.9e-057.3e-05

0.0002250.0021110.0005620.0112250.0008010.0002230.000883

9e-066.5e-051.4e-05

2e-060.0001572e-06

5.6e-05

4e-065.8e-054e-060.000127

0.0066110.0066030.0054660.0113260.0029290.0186520.003528

0.0066110.0066030.0054660.0113260.0029290.0186520.003528

1e-056.4e-058e-06

1e-056.4e-058e-06

0.0066010.0066030.0054660.0113260.0028650.0186440.003528

5e-063.1e-055e-06

0.0065130.006570.0054660.0113260.0020410.0185530.003528

0.000641

0.000109

8.3e-053.3e-054.3e-058.6e-05

5.8e-050.0002545.2e-05

5.8e-050.0002545.2e-05

5.8e-050.0002545.2e-05

5.8e-050.0002545.2e-05

2.5e-054e-052.5e-05

2e-06

8e-069e-068e-06

1.7e-052.9e-051.7e-05

8e-060.0001433e-06

4.1e-05

8e-064.3e-053e-06

5.9e-05

2.5e-057.1e-052.4e-05

2.5e-057.1e-052.4e-05

1.1e-053.4e-056e-06

1.1e-053.4e-056e-06

1.1e-053.4e-056e-06

1.1e-053.4e-056e-06

1.1e-053.4e-056e-06

1.1e-053.4e-056e-06

0.0476850.0351620.0285850.0142580.0368930.0466280.015375

1.9e-058.2e-051.3e-05

1.9e-058.2e-051.3e-05

1.9e-058.2e-051.3e-05

1.9e-058.2e-051.3e-05

1.9e-058.2e-051.3e-05

0.0476530.0351620.0285850.0142580.0367680.0465970.015375

0.0476530.0351620.0285850.0142580.0367680.0465970.015375

0.0476530.0351620.0285850.0142580.0367680.0465970.015375

3.7e-058.9e-054.2e-05

2.1e-054.5e-051.8e-05

1.6e-054.4e-052.4e-05

1.2e-054.7e-051.3e-05

1.2e-054.7e-051.3e-05

3.1e-055.8e-052.2e-05

3.1e-055.8e-052.2e-05

0.0475730.0351620.0285850.0142580.0365740.046520.015375

2.7e-055.1e-052e-05

0.0475460.0351620.0285850.0142580.0365230.04650.015375

1.3e-054.3e-051.8e-05

1.3e-054.3e-051.8e-05

1.3e-054.3e-051.8e-05

1.3e-054.3e-051.8e-05

1.3e-054.3e-051.8e-05

0.3267890.2021680.1886640.0966730.2666270.3246180.122051

0.0007170.0011570.000672

0.0003320.0006830.000255

0.0002950.0004590.00021

0.000110.0002536.1e-05

0.000112

0.000110.0001416.1e-05

0.0001850.0002060.000149

0.0001850.0002060.000149

3.7e-050.0002244.5e-05

0.00011

0.00011

3.7e-050.0001144.5e-05

3.7e-050.0001144.5e-05

0.0003850.0004740.000417

0.0003850.0004740.000417

6.7e-057.9e-05

6.7e-057.9e-05

1.5e-054.6e-051.2e-05

1.5e-054.6e-051.2e-05

0.00018e-050.000101

0.00018e-050.000101

7.9e-05

7.9e-05

8.4e-050.0001078.2e-05

8.4e-050.0001078.2e-05

0.0001190.0001620.000143

6.1e-058.9e-058e-05

5.8e-057.3e-056.3e-05

0.0002090.0004760.000185

0.0002090.0004760.000185

0.0002090.0004760.000185

0.0002090.0004760.000185

0.0002090.0003030.000185

0.000173

0.3205230.2016550.1877460.0966730.2577070.3182730.121772

0.000190.0001190.000202

0.000190.0001190.000202

0.000190.0001190.000202

0.000190.0001190.000202

0.0123980.0084640.0082160.0116290.0104930.0120360.002944

0.0123980.0084640.0082160.0116290.0104930.0120360.002944

0.0001587.3e-058.5e-05

0.0001587.3e-058.5e-05

5.8e-05

5.8e-05

0.000567

0.000139

0.000139

0.000137

0.000152

0.012240.0084640.0082160.0116290.0096760.0119510.002944

0.012240.0084640.0082160.0116290.0096760.0119510.002944

0.000119

0.000119

0.0002710.0003490.000249

0.0002710.0003490.000249

0.0002710.0001560.000249

0.0002710.0001560.000249

0.000193

0.000193

0.000270.0002180.000289

0.000270.0002180.000289

0.000270.0002180.000289

0.000270.0002180.000289

0.0005210.0009250.0109210.0004990.0005990.001234

0.0005210.0009250.0109210.0004990.0005990.001234

6.4e-05

6.4e-05

2.1e-052.4e-051.9e-05

2.1e-052.4e-051.9e-05

5.2e-05

5.2e-05

0.00050.0009250.0109210.0003590.000580.001234

1e-06

3.6e-05

3.6e-051.4e-053.3e-05

1.2e-05

2.2e-05

8.6e-053.6e-057.3e-05

3e-05

0.0001840.0009250.0109215.6e-050.0002770.001234

7e-065e-061.3e-05

7e-06

5.2e-051.2e-054.6e-05

4.5e-055.8e-054e-05

5.1e-055e-065.1e-05

9e-06

3.9e-051.1e-054.7e-05

4.5e-05

0.0009560.0008470.000991

0.0009560.0008470.000991

0.0009560.0008470.000991

0.0002230.00020.000259

0.0001460.000120.000149

0.000220.0001820.000216

0.0001550.0001660.000166

0.0002120.0001790.000201

0.0016615.3e-050.0019880.001716

0.0016615.3e-050.0019880.001716

0.0002140.0002620.000227

0.0002140.0002620.000227

0.000151

0.000151

0.0003442.9e-050.000364

0.0003442.9e-050.000364

0.0002460.0001430.000228

0.0002460.0001430.000228

0.000152

0.000152

0.0002610.0001180.000278

0.0002610.0001180.000278

0.000330.0005390.000345

2e-06

0.000102

8e-06

8.2e-05

1e-06

0.000330.0002240.000345

0.00012

0.0002662.4e-050.0003520.000274

0.0001745.7e-050.000179

2.9e-05

0.000229

9.2e-052.4e-053.7e-059.5e-05

0.000139

0.000139

0.000132

0.000132

0.0224990.0151160.0152180.0122360.0166220.0220640.007579

0.0224990.0151160.0152180.0122360.0166220.0220640.007579

0.0001740.0001510.000176

0.0001740.0001510.000176

0.0218510.0150430.0152180.0122360.0161390.0213910.007579

0.0212780.0150430.0152180.0122360.0157590.0208170.007579

0.0001750.0001250.000186

0.0001970.0001390.000188

0.0002010.0001160.0002

0.0003957.3e-050.0002820.000403

0.0002547.3e-050.0001710.000265

0.0001410.0001110.000138

7.9e-055e-059.4e-05

3.5e-052.2e-054.3e-05

4.4e-052.8e-055.1e-05

0.0003030.000140.000284

0.0003030.000140.000284

0.0003030.000140.000284

0.0003030.000140.000284

0.0196810.0074570.0235650.0112250.026560.0201020.010366

0.0042170.0008570.0010570.0042240.0043950.000255

0.0011850.0002810.0011690.001255

6.8e-05

3.7e-05

0.0002580.0002373.5e-050.000251

6.3e-05

3e-05

0.000245

4.1e-05

0.000308

0.0001492.1e-054.9e-050.000161

0.0001882.3e-056.7e-050.000197

0.0003080.0001830.00034

0.0002822.7e-050.000306

1.6e-05

0.0030320.0005760.0010570.0030550.003140.000255

0.0016820.0003880.0010570.0021290.00177

0.0007420.0001020.0002280.0007430.000255

0.000239

0.0006088.6e-050.0004590.000627

0.0002510.0001910.000272

0.0002510.0001910.000272

0.0002510.0001910.000272

0.0001310.000110.000108

0.0001310.000110.000108

0.0001310.000110.000108

0.0079440.0047870.0067640.0112250.0059320.007854

0.0078480.0047870.0067640.0112250.0056380.007721

2e-06

5.7e-056e-067.5e-05

7.8e-057.4e-059.7e-05

00

8e-051e-059.4e-05

9.1e-053.7e-059.3e-05

4.5e-05

2.8e-056e-062.6e-05

1.1e-051e-056e-06

0.000124

0.0001766.5e-050.000204

2.1e-05

1.6e-058e-062e-05

6.9e-051.7e-057e-067e-05

7.6e-05

7.1e-05

8e-062e-068e-06

1.1e-053.9e-051.2e-05

7.2e-057.4e-058.9e-05

1.3e-05

8e-05

0.0001469.7e-050.000176

1.5e-05

1e-05

2.9e-052e-053.6e-05

8e-061e-058e-06

0.0066810.004770.0067640.0112250.0045140.006559

1.1e-053e-061.2e-05

1.6e-05

2.6e-051e-052.6e-05

0.000162

1.1e-05

6.8e-05

6.3e-05

8.8e-053.1e-050.00011

9.6e-050.0002940.000133

9.6e-050.0002940.000133

5.8e-05

5.8e-05

5.8e-05

0.006650.0018130.0157440.0155350.0069710.010111

0.006650.0018130.0157440.0155350.0069710.010111

0.0001680.0001240.000167

0.000142

0.0001720.0001520.000202

0.000743

0.000812

0.000291

0.00149

6.1e-050.0001273.5e-056.4e-05

0.00037

3e-067e-062e-064e-06

0.0109830.009872

0.000268

0.000144

0.0031420.002824

0.0005925.8e-050.000350.000622

0.000536

0.000189

2.8e-051e-053.4e-05

5.4e-05

0.0003860.000170.000382

0.00028

0.000134

0.000266

0.0009070.0003440.0004440.000909

0.0004490.0003040.000504

0.0002380.0001170.000229

0.0002130.0001940.000213

0.0003870.0003840.0001870.000409

3.2e-054.3e-054e-063.6e-05

0.0025740.0007420.0016190.0015460.002734

0.000171

0.000102

0.000284

0.0001350.0001410.000137

0.0003

0.000288

0.001309

0.000474

0.000141

0.000162

0.0003050.0001080.0001150.0003250.000105

0.0004880.000510.000502

0.0004880.000510.000502

0.0001725.1e-050.000201

0.0001440.0001360.000136

0.0001720.0001180.000165

0.000134

7.1e-05

0.0359580.0132460.0412590.010820.0312540.0379010.018327

0.0359580.0132460.0412590.010820.0312540.0379010.018327

0.0354470.0132050.0412590.010820.031070.0374470.018327

0.0049840.004479

0.000455

0.0040270.0009110.0007332e-06

0.000303

0.0009830.0001510.0001830.001117

0.000206

0.0056730.0023350.0043970.006467

0.000261

0.0023560.002118

0.0003

0.000468

5e-05

0.0098520.008855

8.6e-05

8.1e-05

0.00012

0.0011773.2e-050.001058

0.000102

0.000232

0.000847

0.001171

0.0127230.011435

0.000574

0.000843

0.0040490.0009030.0010250.000830.0046490.000377

0.0008580.0001260.0001020.001002

0.000234

0.001140.0001630.0003020.001229

0.000418

0.0019020.0034510.001136

0.0014140.0001970.0001020.00155

0.00154

0.000816

0.0004328.5e-050.0001150.000484

0.000236

0.0010350.0001950.0002130.001169

0.00011

0.0038550.001110.0011080.0003810.004359

0.0015680.0002629e-050.001736

0.0021590.0010530.0009640.010820.0009750.0022150.000254

6.5e-05

0.000406

0.000322

8.8e-050.0001173e-059.5e-05

0.000436

0.005950.0024280.0001350.006622

0.0020750.0029450.0007710.0002110.0045990.000697

0.000237

1.7e-05

0.000219

0.0001410.0001225e-050.000152

0.0005114.1e-050.0001840.000454

0.0005114.1e-050.0001840.000454

0.2179190.1542660.0966360.028820.1626150.2140280.079636

0.0004250.0007630.000466

0.000239

0.000239

0.000208

0.000208

0.0004250.0003160.000466

0.0004250.0003160.000466

0.001680.00050.0012690.001715

0.0010720.000320.0009220.001078

0.000365

0.0010720.000320.0005570.001078

0.0006080.000180.0003470.000637

0.0006080.000180.0003470.000637

0.2113340.1530020.0966360.028820.1513020.2072480.079379

0.001412

0.001412

0.2056670.1515430.0958310.028820.1404810.2012940.079117

0.000526

5.4e-05

0.000669

0.00220.001978

0.000887

9e-050.0003870.0001729.6e-05

3.6e-05

0.000707

0.000663

0.001593

0.2055770.1511560.0936310.028820.1331960.2011980.079117

0.0020830.0004760.0034120.002225

0.000934

0.0010640.0002240.0008670.00112

0.0010190.0002520.0016110.001105

0.0005770.0001190.0004040.000618

0.0005770.0001190.0004040.000618

0.0001430.0003850.000190.0001440.000262

0.0001430.0003850.000190.0001440.000262

0.0007657.6e-050.0022740.000755

0.0007657.6e-050.0005260.000755

0.000547

0.001201

0.0007850.0004030.0008050.0017240.000836

0.0007850.0004030.0008050.0017240.000836

0.0010060.0010750.001074

0.0010060.0010750.001074

0.0003080.000330.000302

0.0002450.0001130.00025

6.3e-050.0002175.2e-05

0.00040.0003180.000415

0.00040.0003180.000415

0.00040.0003180.000415

4.1e-055e-06

4.1e-055e-06

4.1e-055e-06

0.0003770.0009070.0003510.000257

0.000347

0.000347

0.000257

0.000257

0.000282

0.000282

0.0003770.0002780.000351

0.0003770.0002780.000351

0.000421

0.000421

0.000421

0.0002540.0003950.00026

0.0002540.0002480.00026

0.0002540.0002480.00026

0.000147

0.000147

0.0013260.0004850.000890.001433

0.0013260.0004850.000890.001433

0.0004960.0001750.000330.000543

0.0004140.0001530.0002880.000446

0.0004160.0001570.0002720.000444

0.0006498e-050.0004950.000644

0.0006498e-050.0004950.000644

0.0006498e-050.0004950.000644

0.0014080.0001990.0054440.001423

0.000750.0001030.0027280.000775

0.000672

0.000289

0.000553

0.000750.0001030.0006480.000775

0.000314

0.000252

0.000581

0.000581

0.000189

0.000189

0.000317

0.000317

0.000335

0.000104

0.000231

0.0002830.0002070.000275

0.0002830.0002070.000275

0.0003759.6e-050.0001650.000373

0.0003759.6e-050.0001650.000373

0.000326

0.000326

0.000273

0.000273

0.000323

0.000323

6.6e-050.000376.8e-05

6.6e-050.000376.8e-05

6.6e-050.000376.8e-05

0.0048540.0021280.0028520.0110220.0032420.0047760.001155

0.0002871.8e-050.0001670.000286

0.0002871.8e-050.0001670.000286

0.0002871.8e-050.0001670.000286

0.000360.0001750.000381

0.000360.0001750.000381

0.000360.0001750.000381

0.0042070.002110.0028520.0110220.00290.0041090.001155

0.0040190.002110.0028520.0110220.0027370.0039340.001155

0.0038660.002110.0028520.0110220.0025890.0037640.001155

0.0001530.0001480.00017

0.0001880.0001630.000175

0.0001880.0001630.000175

0.0005030.0002540.0004680.000263

0.0005030.0002540.0004680.000263

0.0005030.0002540.0004680.000263

0.0005030.0002540.0004680.000263

9.7e-050.0001740.000112

9.7e-050.0001740.000112

9.7e-050.0001740.000112

9.7e-050.0001740.000112

0.0014980.0016190.0015250.000268

0.0007730.0005140.000797

0.0001723.4e-050.00018

0.0001613.1e-050.000171

1.1e-053e-069e-06

0.000120.0001630.000121

0.000120.0001630.000121

0.000168.9e-050.00016

0.000168.9e-050.00016

0.0002070.0001090.00022

2.2e-05

0.0002078.7e-050.00022

0.0001140.0001190.000116

0.0001140.0001190.000116

0.0007250.0011050.0007280.000268

0.000390.0004720.0003660.000268

0.000232

0.000390.000240.0003660.000268

0.0003350.0001870.000362

0.0003350.0001870.000362

0.000232

0.000232

0.000214

0.000214

0.0009440.0007140.000931

0.0009440.0007140.000931

0.0003160.0002190.000309

0.0003160.0002190.000309

0.0003220.0002820.000316

0.0003220.0002820.000316

0.0003060.0002130.000306

0.0003060.0002130.000306

0.0051330.0005130.0009180.0069730.0053170.000279

0.0051330.0005130.0009180.0069730.0053170.000279

0.0051330.0005130.0009180.0069730.0053170.000279

0.0051330.0005130.0009180.0069730.0053170.000279

0.0051330.0005130.0009180.0069730.0053170.000279

0.0002070.0003140.000171

0.0002070.0003140.000171

0.0002070.0003140.000171

9.1e-050.0001437.1e-05

9.1e-050.0001437.1e-05

0.0001160.0001710.0001

0.0001160.0001710.0001

4.6e-050.0002014.5e-05

3.4e-055.2e-051.3e-05

3.4e-055.2e-051.3e-05

3.4e-055.2e-051.3e-05

3.4e-055.2e-051.3e-05

3.4e-055.2e-051.3e-05

1.2e-057.2e-053.2e-05

1.2e-057.2e-053.2e-05

1.2e-057.2e-053.2e-05

1.2e-057.2e-053.2e-05

1.2e-057.2e-053.2e-05

7.7e-05

7.7e-05

7.7e-05

7.7e-05

7.7e-05

7e-050.0005028.7e-05

7e-050.0005028.7e-05

7e-050.0005028.7e-05

2.8e-050.0004373.5e-05

1e-050.0001628e-06

1e-055.2e-058e-06

5.2e-05

5.8e-05

3e-067.2e-051e-06

3e-067.2e-051e-06

0.000102

0.000102

1.5e-050.0001012.6e-05

1.5e-050.0001012.6e-05

4.2e-056.5e-055.2e-05

2.2e-054.6e-053.1e-05

2.2e-054.6e-053.1e-05

2e-051.9e-052.1e-05

2e-051.4e-052.1e-05

5e-06

0.0108190.0642180.0157330.0208310.0091420.0096970.03328

8.4e-05

8.4e-05

8.4e-05

8.4e-05

8.4e-05

0.0108190.0642180.0157330.0208310.0090580.0096970.03328

0.0105530.0642180.0157330.0208310.0084150.0094830.03328

0.0105530.0642180.0157330.0208310.0084150.0094830.03328

5.8e-058e-063.2e-05

3.6e-051e-061e-05

2.2e-057e-062.2e-05

0.0104950.0642180.0157330.0208310.0084070.0094510.03328

8e-062.3e-056e-06

7e-06

6e-062.4e-056e-06

4.2e-05

5e-06

7e-0607e-06

0.0103640.0642180.0157330.0208310.0078930.0093040.03328

1e-05

5e-064e-065e-06

6e-061.1e-05

5e-065e-065e-06

3.1e-05

3e-06

6e-063e-066e-06

3e-063e-061e-06

4.2e-05

2.1e-054.9e-052.1e-05

1.6e-05

2e-062e-06

2.5e-057.4e-052.5e-05

9e-06

5e-065e-065e-06

1e-062e-06

5e-061e-065e-06

7e-067e-067e-06

1e-06

3e-061.5e-053e-06

8e-067e-06

6.7e-05

5e-062.1e-05

2e-062e-062e-06

8e-06

1.5e-05

9e-063.5e-05

0.0001530.0004220.000117

0.0001530.0004220.000117

6.5e-058.5e-054.2e-05

4e-05

2e-06

8e-068e-061e-05

5.7e-053.5e-053.2e-05

8.8e-050.0003377.5e-05

6.3e-058.2e-055.9e-05

5e-060.000113

2e-050.00011.6e-05

4.2e-05

0.0001130.0002219.7e-05

2.1e-052.6e-052.4e-05

2.1e-052.6e-052.4e-05

2.1e-052.6e-052.4e-05

9.2e-050.0001957.3e-05

9.2e-050.0001957.3e-05

4e-061.6e-05

1.8e-05

3.6e-051.8e-052.7e-05

2.9e-05

2.1e-052.5e-052e-05

1.2e-05

1.3e-05

2.2e-05

3e-06

2.8e-05

3.1e-051.1e-052.6e-05

1.7e-056e-051.5e-05

1.7e-051.5e-05

1.7e-051.5e-05

1.7e-051.5e-05

1.7e-051.5e-05

1.7e-051.5e-05

6e-05

6e-05

6e-05

6e-05

6e-05

0.3720260.4308350.345140.4527240.2271520.3664110.408291

0.0491440.0698130.1281870.0582460.0512840.0495390.116014

0.0185460.0072650.0056160.0327630.0130.0190380.002563

0.0008270.0003820.000878

0.0002440.0001280.00025

0.0002440.0001280.00025

0.0002249.9e-050.000251

0.0002249.9e-050.000251

0.0003590.0001550.000377

0.0003590.0001550.000377

0.0002790.0003960.000286

0.0002790.0003960.000286

0.0002790.0003960.000286

0.0001430.0001390.000152

1e-061.8e-051e-06

1e-05

1e-06

5e-06

0

1e-061e-06

0

2e-06

0

0

0.0001420.0001210.000151

0.0001424.1e-050.000151

8e-05

0.0036320.0016430.0020680.0218420.0022950.0037150.0018

8.3e-054.6e-057.8e-05

8.3e-054.6e-057.8e-05

0.00019

4.8e-05

7.5e-05

6.7e-05

0.0002880.0001950.00029

0.0001510.0001120.000152

0.0001378.3e-050.000138

0.0007280.000610.0009170.0110220.0004530.000710.0018

0.0007280.000610.0009170.0110220.0004530.000710.0018

0.0025330.0010330.0011510.010820.0014110.002637

0.0002274.7e-055.9e-050.000242

8.2e-05

7e-05

0.0002948.5e-053.6e-050.000335

0.000435

0.0002645.1e-055.3e-050.000296

0.0002244.4e-059.4e-050.000226

0.0002724.6e-053.6e-050.000284

0.0012520.000760.0011510.010820.0005460.001254

5.8e-050.0002495e-05

5.8e-050.0002495e-05

6e-066.4e-056e-06

2e-061.7e-052e-06

5.3e-05

1.7e-052.1e-051e-05

2e-068e-06

7e-062e-06

1.3e-054.3e-051.3e-05

4e-069e-064e-06

1.4e-052.7e-051.3e-05

0.0006341.4e-050.0003730.000645

0.0006341.4e-050.0003730.000645

1.6e-05

4.9e-05

6e-051.4e-051.5e-055.9e-05

0.0001245.4e-050.000131

0.0001596.4e-050.000155

0.0001674.3e-050.000182

0.0001245.3e-050.000118

7.9e-05

0.0004

0.000246

0.00015

9.6e-05

0.000154

0.000154

0.0001630.0003420.000155

0.0001069.7e-050.000103

0.0001069.7e-050.000103

0.000158

0.000158

5.7e-058.7e-055.2e-05

5.7e-058.7e-055.2e-05

0.000192

0.000192

0.000192

0.0008664.7e-050.0015340.000816

4.2e-05

4.2e-05

0.0003784.7e-050.0005790.000372

0.0003784.7e-050.0005790.000372

0.000320.0002550.000311

7.7e-055.7e-057.7e-05

8.4e-056.4e-058.4e-05

0.0001590.0001340.00015

7e-05

7e-05

8.7e-05

8.7e-05

0.000275

0.00014

0.000135

0.0001680.0002260.000133

0.0001680.0002260.000133

0.0032430.0014590.0011190.0109210.001340.0032330.000254

5.5e-05

5.5e-05

0.0023860.001410.0011190.0109210.0006030.002385

0.0020540.0013840.0011190.0109210.0003150.002018

2e-06

1.1e-05

5e-06

0.0001121.3e-053.7e-050.000138

6.6e-05

0.0001416e-050.000146

9.7e-05

7.9e-051.3e-051e-058.3e-05

7.4e-05

7.4e-05

0.0004610.0002110.0004670.000254

0.0001362.4e-050.000138

9.8e-05

9.4e-053e-060.000118

0.000127

9.6e-056.6e-058.6e-05

0.0001352e-050.0001250.000127

4.9e-05

4.9e-05

0.0003144.9e-050.0001960.000288

2.6e-052.2e-051.5e-052.6e-05

6.8e-05

0.000134.4e-050.000111

0.0001582.7e-056.9e-050.000151

8.2e-050.0001529.3e-05

2.2e-05

2.6e-054.1e-054.2e-05

5e-06

2.9e-055.3e-052.8e-05

2.7e-053.1e-052.3e-05

0.0085970.0041020.0024290.0052340.0090.000509

0.00044

0.00044

0.000229

0.000229

0.0082650.0041020.0024290.0036380.0086810.000509

0.0023520.0010760.0004280.0024570.000248

0.001534

0.0020470.0010420.0012470.0006030.002159

0.0020780.0009920.0004730.0021780.000261

0.0017880.0009920.0011820.00060.001887

0.0003320.0009270.000319

0.0003320.0009270.000319

0.0001040.0001240.000108

0.0001040.0001240.000108

0.0001040.0001240.000108

0.0016251.4e-050.0015780.001605

0.0003460.0003130.0003

1e-066.1e-051e-06

7e-06

5.2e-05

1e-062e-061e-06

6.6e-05

6.6e-05

8.2e-052.4e-056.4e-05

8.2e-052.4e-056.4e-05

6e-054e-055.9e-05

3.3e-051.6e-053.4e-05

2.7e-052.4e-052.5e-05

6.6e-053.8e-054.8e-05

6.6e-053.8e-054.8e-05

9.1e-055.6e-058.2e-05

2.8e-05

9.1e-052.8e-058.2e-05

4.6e-052.8e-054.6e-05

4.6e-052.8e-054.6e-05

0.0012791.4e-050.0012650.001305

0.000128

0.000128

0.000340.0001510.000349

0.0001644.2e-050.000187

3.8e-05

0.0001767.1e-050.000162

0.000143

0.000143

0.0002240.0001740.000253

0.0002240.0001740.000253

0.0001280.0001210.000113

0.0001280.0001210.000113

0.0003191.4e-050.0001530.000333

1.4e-05

0.0001655.3e-050.000167

0.0001541.4e-053.9e-050.000166

4.7e-05

9.8e-05

9.8e-05

0.0002580.0002490.000251

0.000190.0001850.000179

6.8e-056.4e-057.2e-05

1e-054.8e-056e-06

1e-054.8e-056e-06

0.0038860.000290.0839240.0114880.0039830.076458

0.0016420.0001340.0839240.0077320.001730.075431

0.001320.0001340.0065150.001382

0.001320.0001340.0065150.001382

0.0839240.075431

0.0839240.075431

2.6e-050.0006622.9e-05

2.6e-050.0006622.9e-05

0.0002960.0005550.000319

0.0002960.0005550.000319

0.0022440.0001560.0037560.0022530.001027

0.000181

0.000181

6.1e-052.8e-056.1e-05

6.1e-052.8e-056.1e-05

0.000175

0.000175

3.8e-054.1e-05

3.8e-054.1e-05

0.0003673.6e-050.0001980.0003860.000258

0.000220.0001430.000231

0.000258

0.0001473.6e-055.5e-050.000155

0.000143

0.000143

0.0001542.9e-050.0001010.000113

7.7e-052.9e-058.7e-057.9e-05

7.7e-051.4e-053.4e-05

0.000769

0.000769

0.000274

0.000274

0.000184.7e-050.0001280.000169

0.000184.7e-050.0001280.000169

0.0001170.0001190.000118

2.7e-054.7e-053.1e-05

9e-057.2e-058.7e-05

7.5e-05

7.5e-05

3.2e-050.0002194.1e-05

2e-055.9e-052.1e-05

1.2e-054.1e-052e-05

0.000119

0.0004674.4e-050.0004110.000462

0.0003614.4e-050.0002470.000352

0.0001067.2e-050.00011

9.2e-05

0.000161

0.000161

0.000329

0.000329

0.0001090.0001340.000133

0.0001090.0001340.000133

0.000259

0.000135

0.000124

0.00015

0.00015

0.0004310.0002650.00045

0.0001570.0001310.000161

0.0002740.0001340.000289

0.0002880.0001650.000279

0.0002880.0001650.000279

0.000137

0.000137

0.000104

0.000104

6.2e-050.0003326.4e-05

6.2e-050.0003326.4e-05

2.8e-050.0001933.1e-05

1.7e-050.0001281.7e-05

1.1e-056.5e-051.4e-05

3.4e-050.0001393.3e-05

3.4e-054.6e-052.9e-05

9.3e-054e-06

0.0016863.3e-050.0022640.001734

0.0016863.3e-050.0022640.001734

0.0004670.0003220.000472

0.0004670.0003220.000472

0.0006543.3e-050.0004760.000666

0.0002637e-050.000277

0.0002360.0001330.000238

0.000118

0.0001553.3e-050.0001550.000151

0.00046

0.00046

0.0004010.0007740.00042

0.0004010.0002090.00042

0.000201

0.000123

0.000241

0.0001640.0002320.000176

0.0001640.0002320.000176

0.0207040.0621370.0386470.0254830.0179030.0204320.036993

2.6e-05

2.6e-05

2.6e-05

0.0048920.0218760.0191540.001660.0049420.017241

0.0048870.0218760.0191540.0016450.0049390.017241

5.4e-05

0.0001190.0168650.0001320.000121

3.1e-056e-066e-06

0.0009220.0035470.0007840.0003020.001001

0.0002014.8e-050.000232

0.00039.1e-050.000324

0.0001775.3e-050.000177

0.0003490.000130.00036

0.0001415.7e-050.000142

0.0003155.6e-050.000354

1.4e-055e-060

0.0001843.8e-050.000219

0.0001684.2e-058e-06

4.2e-052.2e-051.6e-05

3.3e-050.0006091.2e-053.5e-05

0.0001813.7e-050.000191

0.0006160.0004110.0001880.000629

0.018370.016511

0.0001575.4e-050.000154

0.0001077.3e-050.0001070.00073

0.000830.0004440.0002450.000863

5e-061.5e-053e-06

5e-061.5e-053e-06

0.0158080.0402610.0194930.0254830.0161740.0154860.019752

7.1e-05

2.4e-05

8e-06

3.9e-05

3.9e-059e-064.4e-05

3.7e-052e-064.4e-05

2e-065e-06

2e-06

0.0021310.0015530.0011070.0109210.0014070.0020820.000277

0.002120.0015530.0011070.0109210.0013660.0020730.000277

2.4e-05

5e-068e-065e-06

2e-06

4e-063e-064e-06

2e-064e-06

0.0136380.0387080.0183860.0145620.0146870.013360.019475

4e-060.0001914e-06

0.0136340.0387080.0183860.0145620.0144910.0133560.019475

5e-06

4e-064.3e-054e-06

4e-064.3e-054e-06

4e-064.3e-054e-06

1.9e-054.9e-052.2e-05

1.9e-054.9e-052.2e-05

1.9e-054.9e-052.2e-05

1.9e-054.9e-052.2e-05

0.0004640.0004050.000454

0.0004640.0004050.000454

0.0003530.0001650.000363

0.0003530.0001650.000363

5.4e-057.6e-054.9e-05

5.4e-057.6e-054.9e-05

5.7e-050.0001644.2e-05

5.7e-050.0001644.2e-05

0.000118

4e-05

4e-05

4e-05

7.8e-05

7.8e-05

7.8e-05

0.0004620.0006230.000444

0.0004620.0006230.000444

0.0004620.0006230.000444

0.0004620.0006230.000444

0.001697.4e-050.0035240.001763

0.0001450.0010970.00015

0.000313

0.000313

0.0001450.0001780.00015

0.0001459.6e-050.00015

8.2e-05

0.000497

7.8e-05

9e-05

0.000109

6e-05

0.000111

4.9e-05

0.000109

5.8e-05

5.1e-05

0.0015457.4e-050.0024270.001613

0.0004630.0004720.000472

3.4e-05

4.4e-05

0.0001363.5e-050.000127

4.7e-05

0.000182.4e-050.000194

4.2e-05

5.4e-05

0.0001478.2e-050.000151

0.00011

0.00014

0.00014

8.7e-05

8.7e-05

0.000457.4e-050.0009130.000473

6.2e-05

0.000457.4e-050.0001030.000473

4.6e-05

4.3e-05

0.000634

2.5e-05

1.7e-054.4e-055.8e-05

1.7e-054.4e-055.8e-05

0.0001

0.0001

0.0001680.0001760.000156

3e-05

0.0001042.5e-058.4e-05

5.4e-05

6.4e-056.7e-057.2e-05

0.0004470.0004950.000454

6.5e-05

7.7e-05

8.7e-05

0.0002140.0001120.000232

6.2e-05

0.0002339.2e-050.000222

0.1035530.1630240.0544760.0852460.0166920.1015580.136599

0.1035380.1630240.0544760.0852460.0165950.1015510.136599

8.2e-050.0002725.4e-05

1.7e-050.0001511.1e-05

3.2e-05

1.8e-05

1.4e-05

1e-05

3e-064.2e-05

8e-065e-06

2.1e-05

01e-060

6e-066e-066e-06

7e-06

1.7e-055e-051.2e-05

1.7e-055e-051.2e-05

1.2e-053.3e-051.6e-05

1.2e-053.3e-051.6e-05

3.6e-053.8e-051.5e-05

1.8e-051.9e-054e-06

1.8e-051.9e-051.1e-05

6.3e-053e-054.6e-05

6.3e-053e-054.6e-05

6.3e-053e-054.6e-05

0.1033930.1630240.0544760.0852460.0162930.1014510.136599

4.2e-056.3e-053.4e-05

1.8e-054e-051.4e-05

1.6e-057e-061.5e-05

8e-061.6e-055e-06

0.1033320.1630240.0544760.0852460.0161850.1014170.136599

1.7e-053.1e-05

7.8e-05

9e-06

6.1e-05

4.3e-05

1.6e-05

0.0006880.0013830.0013810.0505615.3e-050.000974

1.3e-05

1.3e-05

2.3e-051.9e-05

8.8e-052.1e-058.6e-05

3.5e-05

4.5e-05

2.1e-051e-063e-05

0.1024580.1616410.0530950.0346850.0157090.1002850.136599

3.7e-053.8e-054.2e-05

1.9e-054.5e-05

5e-06

5e-06

2e-061.5e-05

1.2e-05

6e-06

1.7e-052e-06

1.5e-054.8e-054e-06

1.5e-054.8e-054e-06

3e-062.9e-05

3e-062.9e-05

1.2e-051.9e-054e-06

1.4e-05

1.2e-055e-064e-06

4.9e-053e-06

4.9e-053e-06

4.9e-053e-06

4.9e-053e-06

0.1495070.1406980.1051730.2496710.09876999999999990.1467830.109285

0.000104

0.000104

0.000104

0.000104

1.4e-053.5e-051.4e-05

1.4e-053.5e-051.4e-05

1.4e-053.5e-051.4e-05

1.4e-053.5e-051.4e-05

0.0001690.0164010.0011740.0001430.014741

0.00027

0.00027

0.000142

0.000128

0.000453

0.000231

0.000231

0.000222

0.000222

0.0164010.014741

0.0164010.014741

0.0164010.014741

0.0001690.0004510.000143

5.6e-059.5e-054.4e-05

5.6e-059.5e-054.4e-05

3.9e-058.7e-053.6e-05

3.9e-058.7e-053.6e-05

5.7e-050.0001674.7e-05

5.7e-050.0001674.7e-05

1.7e-050.0001021.6e-05

1.7e-050.0001021.6e-05

7.9e-05

7.9e-05

7.9e-05

7.9e-05

0.0012490.0011590.0006260.010820.0010540.0012190.001322

0.0002390.0003510.000233

0.0001336.5e-050.000125

0.0001336.5e-050.000125

8.1e-05

8.1e-05

0.0001065.6e-050.000108

0.0001065.6e-050.000108

0.000149

7.2e-05

3e-05

4.7e-05

0.001010.0011590.0006260.010820.0007030.0009860.001322

0.0003650.0001410.000371

0.0001448e-050.000157

0.0002216.1e-050.000214

7.2e-050.0001056.1e-05

5.1e-05

7.2e-051.4e-056.1e-05

4e-05

0.000106

2.6e-05

4.3e-05

3.7e-05

0.0004450.0011590.0006260.010820.0002280.0004340.001322

0.0004450.0011590.0006260.010820.0002280.0004340.001322

0.0001280.0001230.00012

4.5e-05

8e-06

2.2e-051.1e-051.7e-05

5e-0606e-06

1e-051e-065e-06

2e-0602e-06

1.2e-058e-068e-06

7e-051.8e-057.7e-05

7e-068e-065e-06

2.4e-05

0.0008770.0024550.0010730.0111230.0004840.0007920.001582

0.0008770.0024550.0010730.0111230.0004840.0007920.001582

9.9e-053.6e-056.5e-05

5.3e-052.5e-055.2e-05

4.6e-051.1e-051.3e-05

1.5e-056.1e-055e-06

1.5e-056.1e-055e-06

0.0006230.0024550.0010730.0111230.0001870.0005640.001582

1.2e-051e-051.1e-05

3.2e-052.4e-051.7e-05

2.4e-056.4e-054.4e-05

0.000349

6.3e-05

0.0005550.0024550.0010730.0111238.9e-050.0004920.00117

3e-062.9e-055e-06

3e-062e-065e-06

2e-05

7e-06

3.8e-053.7e-054.2e-05

3.8e-053.7e-054.2e-05

7e-062.5e-051.3e-05

7e-062.5e-051.3e-05

4e-069e-066e-06

4e-069e-066e-06

7.2e-059e-058e-05

4e-069e-064e-06

4e-057e-063.7e-05

3e-06

2.8e-057.1e-053.9e-05

1.6e-051e-051.2e-05

1.6e-051e-051.2e-05

0.028240.0182570.0071810.1140660.0131120.0293180.038082

0.0003510.0001160.0002460.000332

5e-05

5e-05

5.5e-05

5.5e-05

0.0003510.0001160.0001410.000332

1.8e-055.8e-054e-051.7e-05

1e-05

2.2e-05

0.0002814.7e-050.00026

5.2e-055.8e-052.2e-055.5e-05

0.0005580.0005720.000442

0.0001180.0002680.000105

5.9e-05

6.7e-05

5.8e-055.5e-056.3e-05

6e-053.5e-054.2e-05

5.2e-05

0.0002689.1e-050.000209

0.0002689.1e-050.000209

8.2e-050.0001127.3e-05

3.1e-05

3.2e-053.7e-052.2e-05

5e-054.4e-055.1e-05

5.7e-054.6e-052.9e-05

5.7e-054.6e-052.9e-05

3.3e-052e-062.6e-05

3.3e-052e-062.6e-05

5.3e-05

5.3e-05

0.0212940.0163220.0071810.1031450.0086960.0228990.034691

3.9e-05

3.9e-05

0.0014510.000610.0006050.001510.000517

0.0005950.0004771.8e-050.0006360.000517

0.0008560.0001339.7e-050.000874

0.000101

0.00015

4.1e-05

0.000198

0.000226

0.000226

0.0010030.0003510.0002240.000988

8.2e-050.0001496e-060.000103

0.000132

8e-06

7.3e-057e-066.4e-05

0.0004530.0001133e-050.000392

0.0003958.9e-053.7e-050.000429

4e-06

0.000121

0.000121

0.000112

0.000112

0.000739.3e-050.0001140.000775

0.000739.3e-050.0001140.000775

4.7e-050.000154.6e-05

6e-063e-066e-06

6.3e-05

2.7e-051.6e-053.3e-05

1.4e-051.5e-057e-06

2e-06

5.1e-05

0.000131

0.000131

0.000301

0.00026

4.1e-05

0.0016190.0074090.0014750.0148650.000270.0016480.009908

0.0010326.6e-050.0001750.001081

0.0005870.0073430.0014750.0148653e-050.0005670.009908

6.5e-05

9e-061.9e-059e-06

9e-061.9e-059e-06

5.2e-05

5.2e-05

0.0019310.0001470.0007750.001937

3.4e-055.1e-053.3e-05

0.0012130.0001470.0005840.001223

2.6e-054e-052.1e-05

0.0006580.00010.00066

4e-05

4e-05

0.0055040.0011640.0006880.010820.0008690.0056390.000815

0.0014510.000242.6e-050.001475

3e-06

1.2e-05

7e-06

0.0008338.9e-054.3e-050.000891

0.0016480.0004790.0006880.010820.0002130.001648

0.000239

7e-06

1.5e-05

0.0007340.0002672.4e-050.0007560.000815

9e-06

5e-06

4e-06

0.0008388.9e-050.0002550.000869

7e-06

0.0039790.0054880.0042260.0665390.000390.0033340.021253

0.0009750.0035160.0018770.0210338.6e-050.000987

0.000460.0002690.0156744.2e-050.000479

0.0015080.0002880.0006670.0136520.0002250.0007710.021253

0.0010360.0014150.0016820.016183.7e-050.001097

0.0003760.0003060.0007920.0109210.0001270.0023330.000782

8.1e-050.0002660.0007920.0109217.4e-050.0020250.000782

0.0002954e-055.3e-050.000308

3.6e-05

3.6e-05

2.3e-052e-05

2.3e-052e-05

0.0030280.0005690.0013060.0030950.001416

0.000298

0.0012040.0002250.0002650.001283

0.000848

0.0018240.0003440.0002220.001812

0.000167

0.000568

0.000354

0.00028

0.00028

0.000309

0.000309

0.0015690.0001850.0014780.00154

0.0015690.0001850.0014780.00154

2.5e-051.1e-052.5e-05

2.5e-051.1e-052.5e-05

0.000711

0.000711

0.0023670.0014050.0109210.0009340.0023020.00026

0.000142.3e-052.2e-050.000136

7e-061.8e-051.2e-05

0.0001332.3e-054e-060.000124

0.0014250.000130.00030.001345

4e-06

0.0003311.7e-050.000309

4e-06

0.000142

0.000325e-050.000306

8.6e-053.3e-051e-058.2e-05

2.8e-05

1.6e-05

0.0006889.7e-052.9e-050.000648

0.0008020.0012520.0109210.0004160.0008210.00026

0.0007970.0012390.0109215.7e-050.0008170.00026

4.4e-05

6.4e-05

1e-061.3e-0500

4.9e-05

8.2e-05

6.1e-05

5.6e-05

1e-06

4e-062e-064e-06

0.000196

0.000196

0.0021970.0001320.0017980.001930.002605

1.2e-052.2e-051.1e-05

1.2e-052.2e-051.1e-05

0.0011335.9e-050.0007230.0009920.002605

6.6e-052.2e-057.9e-05

0.000273

0.0004050.0001490.0003440.002605

2.7e-053.1e-052.1e-051.7e-05

0.0004790.0002570.000415

0.0001562.8e-051e-060.000137

1e-054.3e-051e-05

1e-054.3e-051e-05

0.0010427.3e-050.001010.000917

3.8e-05

9e-05

0.0004

4.3e-054e-052.1e-05

0.0004394e-050.000416

0.0001163.3e-055e-050.0001

0.000159

0.0004444e-050.0001930.00038

0.000144

0.000144

0.000144

0.0014730.0002820.0005290.0014130.000526

0.0004110.0002030.000515

0.0001541.8e-050.000136

1.7e-054e-061.6e-05

0.0002160.0001530.000335

2.1e-05

2.4e-057e-062.8e-05

0.0002720.0001420.000212

9e-05

1.7e-05

0.0002723.5e-050.000212

0.000790.0002820.0001840.0006860.000526

0.0002889.9e-050.000236

0.0005020.0002824.2e-050.000450.000526

4.3e-05

0.000193

0.000193

0.000193

0.0001590.0006790.0109210.0002370.0001440.000541

0.0001560.0006790.0109210.000170.0001430.000541

0.0001560.0006790.0109210.0001390.0001430.000541

1.3e-05

0.0001430.0006790.0109212.2e-050.0001280.000541

1.3e-053.6e-051.5e-05

3.5e-05

3.3e-05

3.1e-05

3.1e-05

3e-066.7e-051e-06

2.3e-05

2.3e-05

3e-064.4e-051e-06

3e-068e-061e-06

3.6e-05

0.0269140.0097790.0145750.0125390.0217110.0265770.004002

5.3e-050.0001464.7e-05

5.3e-050.0001464.7e-05

7e-05

5.3e-057.6e-054.7e-05

0.000139

0.000139

0.000139

0.0265190.0097790.0145750.0125390.0209330.0262340.004002

0.0260510.0097790.0145750.0125390.0203140.0258370.004002

0.0028020.002519

0.0002527.3e-05

0.000250.0001050.000227

6.6e-05

0.0255490.0097790.0117730.0125390.0175510.025610.004002

0.0001560.000120.000136

0.0001560.000120.000136

0.0001850.0002240.000163

7.3e-050.0001126.1e-05

0.0001120.0001120.000102

0.0001270.0001189.8e-05

0.0001270.0001189.8e-05

9.4e-05

9.4e-05

6.3e-05

6.3e-05

0.000158

0.000158

0.000158

0.0003420.0003350.000296

0.0001016.6e-059.2e-05

0.0001016.6e-059.2e-05

2.3e-050.0001093.6e-05

3e-064.6e-055e-06

1.9e-051.9e-051.9e-05

1e-064.4e-051.2e-05

0.0001556.7e-050.000115

0.0001556.7e-050.000115

6e-05

6e-05

6.3e-053.3e-055.3e-05

6.3e-053.3e-055.3e-05

0.000145

0.000145

8.2e-05

8.2e-05

6.3e-05

6.3e-05

0.0037440.0022230.0007710.0005670.0035830.000751

0.0037440.0022230.0007710.0005670.0035830.000751

0.00350.0022230.0007710.0003650.00340.000751

0.0026870.0020830.0007710.0002150.0026670.000751

1.4e-05

7.7e-05

0.0004568.3e-052.2e-050.000413

0.0003575.7e-053.7e-050.00032

3.8e-059e-053.5e-05

3.8e-059e-053.5e-05

0.0002060.0001120.000148

0.0002060.0001120.000148

0.0001520.002170.0028720.000139

0.0001520.002170.0028720.000139

0.000110.0004858.5e-05

9.8e-05

4.3e-055.2e-053.3e-05

0.000136

2.4e-050.000122.4e-05

4.3e-057.9e-052.8e-05

0.000165

0.000165

9.1e-05

9.1e-05

6.6e-05

6.6e-05

4.2e-052.4e-055.4e-05

4.2e-052.4e-055.4e-05

0.002170.002041

9.1e-05

0.002170.00195

0.0618510.0891380.0416790.0452020.0354870.0593520.043101

0.0608450.0886440.0416790.0452020.0327850.0583090.043101

0.0266530.0109360.0083850.0268990.0103740.0249420.008119

0.0006798e-050.0001080.000585

4.4e-05

0.0007210.0002170.0001270.0006850.000602

0.0003448.7e-052.6e-050.000331

0.0001711.4e-050.000159

0.0001251.5e-050.000123

0.0001340.0002236e-060.0001370.000353

0.00011

0.000117

4.3e-05

0.0010230.0001427.2e-050.0009340.000662

0.000107

9e-06

3.4e-05

9e-06

4.9e-05

0.0151350.0073890.007890.0145620.004250.01520.003638

0.0006270.0003215.3e-050.0006170.000261

0.0018140.0003150.0004590.0017920.000655

4.4e-05

4e-05

0.0001981e-050.000201

4e-05

0.000153

9.9e-05

4.3e-05

0.000175

0.000131

3.4e-05

8.2e-05

0.000501

0.000107

0.0003474.2e-055e-060.00033

5.3e-05

0.0007860.0012640.0004950.0123370.0001060.0007270.001948

2e-05

4.7e-05

0.0020150.0004610.0003690.002012

0.0004936.7e-056.8e-050.000463

0.000173

5e-06

2.4e-05

1.4e-05

7e-05

4.4e-05

4.6e-052.9e-054.5e-055e-05

0.0003893.7e-052.6e-050.000352

8e-06

0.0013610.000228

1.7e-05

0.00012

0.000947

0.0002453.4e-051.5e-050.000244

6e-06

0.001081

0.0341920.0777080.0332940.0183030.0223730.0333670.034982

0.0341920.0777080.0332940.0183030.0221280.0333670.034982

0.000245

3.8e-05

3.8e-05

0.0010060.0004940.0027020.001043

0.0005630.0001980.0021730.000607

0.000184

0.000318.5e-050.0005070.000338

0.0001285e-059.9e-050.000138

2.3e-056.3e-057e-062e-05

0.000243

0.00025

0.0001022.8e-050.000111

0.000311

0.000295

0.000249

1.8e-050.0001291.8e-05

1.8e-051.4e-051.8e-05

7.1e-05

4.4e-05

0.0004250.0002960.00040.000418

1.4e-05

2.6e-051.1e-052.7e-05

1.4e-056e-061.5e-05

2.6e-05

5e-06

3e-06

0.0003770.0002960.0003310.000367

8e-064e-069e-06

0.0002860.0011490.0109210.0004880.0003060.000187

0.00020.0011490.0109210.000110.0002480.000187

2.4e-05

2.4e-05

0.00020.0011490.0109218.6e-050.0002480.000187

1e-061e-064e-06

3e-06

1.5e-05

0.000170.0011490.0109215.6e-050.0002180.000187

2.9e-057e-062.6e-05

4e-06

8.6e-050.0002765.8e-05

3.8e-050.0001341.1e-05

2.9e-057.1e-051.1e-05

9e-066.3e-05

3.8e-054.2e-052.5e-05

3.8e-052.4e-052.5e-05

1.8e-05

2e-061.5e-052e-06

2e-061e-052e-06

5e-06

8e-067.3e-052e-05

8e-067.3e-052e-05

1.2e-05

1.2e-05

0.000102

4.4e-05

4.4e-05

5.8e-05

5.8e-05

0.0112380.0085240.0091780.0225510.0079480.0108790.004976

0.0106480.0085240.0091780.0225510.0071130.0103850.004976

1.7e-058.8e-051.3e-05

1.7e-058.8e-051.3e-05

8e-05

8e-05

2.9e-054.7e-053.1e-05

7e-069e-066e-06

1e-06

1.9e-051.3e-052.3e-05

3e-063e-062e-06

2e-05

1e-06

0.0031720.0030980.0031560.0112250.0020980.0030720.002106

5.6e-051.3e-055e-05

0.000126

0.003040.0030980.0031560.0112250.0017770.0029650.002106

7.6e-052.6e-055.7e-05

3e-05

3.8e-05

4.1e-05

4.7e-05

0.007430.0054260.0060220.0113260.0047360.0072690.00287

0.0074220.0054260.0060220.0113260.0046480.0072630.00287

8e-068.8e-056e-06

6.4e-05

6.4e-05

1.9e-059.5e-052.8e-05

1.9e-059.5e-052.8e-05

1.3e-055.4e-051.4e-05

6e-064.1e-051.4e-05

1.2e-058.2e-051.2e-05

1.2e-058.2e-051.2e-05

1.2e-054.2e-051.2e-05

4e-05

3.1e-050.0001952.4e-05

3.1e-050.0001952.4e-05

2.8e-05

1.1e-054e-061.2e-05

3.7e-05

8e-06

8e-066e-067e-06

1.2e-053.9e-055e-06

5e-06

3.9e-05

2.6e-05

3e-06

2.9e-054.5e-052.7e-05

2.9e-054.5e-052.7e-05

2.9e-054.5e-052.7e-05

0.0003760.0002740.000317

0.0003760.0002740.000317

1e-062e-063e-06

3.1e-051.7e-052.8e-05

0.0001113.6e-058.3e-05

3.6e-054.1e-051.9e-05

1.2e-052.2e-051.6e-05

2.2e-052.1e-052e-05

1e-062e-061e-06

7e-069e-06

3.6e-051e-052.7e-05

7e-0608e-06

7e-064e-065e-06

1.1e-051.2e-051.2e-05

1e-052.4e-051.1e-05

2e-051.6e-051.8e-05

1.8e-051.7e-05

1.5e-054.3e-051.3e-05

3.1e-052.4e-052.7e-05

0.0001239.1e-058.6e-05

0.0001239.1e-058.6e-05

0.0001239.1e-058.6e-05

5.3e-05

5.3e-05

5.3e-05

0.0009460.0034530.000912

1.7e-050.0001311.9e-05

1.7e-050.0001311.9e-05

1.7e-054.6e-051.9e-05

4.8e-05

3.7e-05

9.8e-05

9.8e-05

9.8e-05

9.7e-050.0005189.4e-05

0.000141

0.000141

2.6e-057.2e-051.8e-05

2.6e-057.2e-051.8e-05

7.1e-050.0001797.6e-05

3.3e-056e-053.5e-05

1.9e-056.3e-051.6e-05

1.9e-055.6e-052.5e-05

0.000126

0.000126

0.0006430.0023430.000634

0.0002550.0001630.000248

0.0002550.0001630.000248

0.000125

0.000125

2.1e-051.6e-052.8e-05

2.1e-051.6e-052.8e-05

4e-05

4e-05

5.2e-05

5.2e-05

0.0003670.0019470.000358

0.001128

0.0003670.0002810.000358

0.000179

0.000168

0.000191

0.0001540.0002730.000132

0.0001540.0002730.000132

0

8.6e-050.0001376.9e-05

6.8e-051e-066.3e-05

0.000135

3.5e-059e-053.3e-05

3.5e-059e-053.3e-05

3.5e-059e-053.3e-05

0.0001250.0002350.000128

0.0001250.0002350.000128

1.7e-057.5e-051.7e-05

2.2e-05

3e-05

1.7e-052.3e-051.7e-05

9.4e-059.1e-050.0001

9.4e-059.1e-050.0001

1.4e-056.9e-051.1e-05

1.4e-056.9e-051.1e-05

0.0135430.0073350.0115190.0115280.0095850.013277

0.0135430.0073350.0115190.0115280.0095850.013277

2.3e-056.2e-051.9e-05

2.7e-05

2.3e-053.5e-051.9e-05

0.0135060.0073350.0115190.0115280.0094080.013234

7e-061e-067e-06

7e-062e-067e-06

2.7e-05

8e-06

3.8e-05

1.2e-051.2e-058e-06

4e-06

7e-067e-061.1e-05

4.2e-058e-063.7e-05

8e-063e-069e-06

1.7e-051e-061.6e-05

7e-06

2e-051.9e-059e-06

5e-062e-063e-06

1.1e-05

1.3e-05

1.6e-056e-061.3e-05

0.0133650.0073350.0115190.0115280.0091930.013114

2.6e-05

2e-05

6.4e-05

6.4e-05

1.4e-055.1e-052.4e-05

1.4e-05

1.3e-052.6e-051.4e-05

1e-061.1e-051e-05

0.0001057.1e-058.5e-05

0.0001057.1e-058.5e-05

0.0001057.1e-058.5e-05

0.0001057.1e-058.5e-05

7.4e-052.7e-055.3e-05

1.2e-053.1e-051.3e-05

1.9e-051.3e-051.9e-05

0.0679560.05730.0573040.0595610.0518540.0667870.046393

0.004040.0113270.0017860.0121350.0022250.003910.013612

0.0035070.0112640.0017860.0121350.0019720.0034910.013612

0.000130.000254

0.000254

0.00013

0.0035070.0112640.0017860.0121350.0017610.0034910.013358

0.000714

0.000161

9.4e-05

9.4e-05

0.0004310.0001793.2e-050.000287

0.000714

0.00084

0.0009780.000879

0.00121

0.000382

0.0019830.0107980.0008080.0121350.0001430.0020850.009366

0.0010930.0002870.0003250.0011190.000165

8.1e-05

8.1e-05

0.0005336.3e-050.0002530.000419

0.0002516.3e-050.000140.00021

0.0002516.3e-050.000140.00021

0.0001527.1e-050.000118

0.0001527.1e-050.000118

0.000134.2e-059.1e-05

0.000134.2e-059.1e-05

0.0007860.0004080.000711

0.0004660.000250.000438

0.0003130.0001430.000293

0.0001316.7e-050.000121

1.7e-05

5.2e-05

0.0001827e-060.000172

0.0001530.0001070.000145

0.0001536.3e-050.000145

4.4e-05

6.2e-055.1e-054.5e-05

6.2e-055.1e-054.5e-05

6.2e-055.1e-054.5e-05

0.0002580.0001070.000228

0.0001426.4e-050.000114

0.0001426.4e-050.000114

0.0001164.3e-050.000114

0.0001164.3e-050.000114

0.0348960.0298520.0239960.0132470.0267380.034110.013619

0.0348960.0298520.0239960.0132470.0267380.034110.013619

7.1e-050.0001237.3e-05

1.2e-052.9e-052e-05

2.3e-056e-06

3.5e-052.2e-051.7e-05

2.4e-052.6e-052.4e-05

2.3e-056e-06

1.1e-052.9e-05

1.1e-052.9e-05

2.6e-051.7e-051.9e-05

2.6e-051.7e-051.9e-05

0.0347880.0298520.0239960.0132470.0265690.0340180.013619

0.0347880.0298520.0239960.0132470.0265690.0340180.013619

0.0004320.0008840.000389

0.0001430.0001830.000108

2.5e-055.1e-052.1e-05

2.5e-055.1e-052.1e-05

6.1e-055.4e-055.1e-05

6.1e-055.4e-055.1e-05

5.7e-057.8e-053.6e-05

5.7e-057.8e-053.6e-05

0.0001157.6e-059.4e-05

0.0001157.6e-059.4e-05

0.0001157.6e-059.4e-05

0.0001230.0002820.000121

0.000108

5.5e-05

5.3e-05

4.5e-051e-053.8e-05

1.8e-057e-061.6e-05

2.7e-053e-062.2e-05

3.5e-058e-053.1e-05

1.8e-054.4e-051.6e-05

1.7e-053.6e-051.5e-05

3.7e-05

3.7e-05

4.3e-054.7e-055.2e-05

4.3e-054.7e-055.2e-05

5.1e-050.0002146.6e-05

1.1e-056e-051.9e-05

2.9e-05

1.1e-053.1e-051.9e-05

4e-050.0001544.7e-05

1.4e-054.3e-051.3e-05

5e-062.9e-055e-06

3.1e-05

6e-06

1.2e-054e-051.9e-05

9e-065e-061e-05

0.000129

5.5e-05

5.5e-05

7.4e-05

7.4e-05

0.0278020.0161210.0315220.0341790.0215990.0276670.019162

0.0072730.0017330.0047970.0046310.0073840.000527

0.0069140.0017330.0047970.0042460.0070560.000527

0.0033030.002969

0.0069140.0017330.0014940.0010390.0070560.000527

0.000238

0.0002336.1e-050.00022

1.9e-05

0.0002332.5e-050.00022

1.7e-05

0.000129

5.5e-05

7.4e-05

7e-053.8e-056.2e-05

7e-053.8e-056.2e-05

5.6e-050.0001574.6e-05

6e-05

4.7e-05

5.6e-055e-054.6e-05

0.0095090.008547

0.0095090.008547

0.0095090.008547

0.010620.0054310.0045180.0110220.005750.0106470.002633

2.1e-055.7e-051.9e-05

1.6e-051.3e-051.2e-05

1.3e-05

5e-063.1e-057e-06

0.0001814e-050.0001030.000193

2e-05

2.4e-05

7.2e-052.5e-051.8e-057.3e-05

0.0001091.5e-054.1e-050.00012

0.0089470.0049730.0045180.0110220.0038440.0089520.002633

5.9e-056.7e-051.6e-056.3e-05

1.5e-05

1.5e-05

1.7e-05

1e-05

1.5e-05

2e-06

6e-06

2.4e-05

3.1e-051.7e-051e-053.6e-05

1.9e-05

0.000104

1.5e-05

2.7e-05

0.0001271.7e-050.000137

8.9e-053.9e-050.000101

1.2e-052e-061.9e-05

2.1e-05

2e-06

0.0001159e-060.000127

5.1e-051e-055.6e-05

3e-064e-063e-06

5.3e-05

5e-061.2e-052e-065e-06

6.2e-053e-066.8e-05

6.7e-051.4e-055.9e-050.000263

0.0001323.8e-050.000134

0.0052040.0021630.0031140.0025160.0051980.001844

1.5e-05

0.0001396e-050.000124

1.5e-057e-061.7e-05

7e-06

4.3e-058e-064.6e-05

9e-06

2.9e-05

0.0027930.0027140.0014040.0110220.0005970.0027590.000526

9e-06

7.8e-05

6e-050.0002124.3e-05

1.6e-05

1.1e-05

1.6e-05

6e-059e-064.3e-05

3.3e-05

2.7e-05

6.1e-05

1.4e-05

2.5e-05

0.000659.9e-050.0004490.000651

3.2e-05

0.0001994.9e-058.8e-050.000188

2.8e-054.6e-054.4e-05

0.000112

0.0002075e-055.6e-050.000201

0.0001245.7e-050.000128

9.2e-055.8e-059e-05

0.0007610.0003190.0010850.000789

0.0002023.7e-050.000217

7e-05

0.0008

0.0002340.000196.9e-050.000226

0.0001719.2e-056.1e-050.000173

0.0001548.5e-050.000173

0.0005020.0034820.0036750.000522

5.6e-057.4e-056.5e-05

3e-053.2e-05

2.6e-057.4e-053.3e-05

0.0001417e-050.00012

0.0001417e-050.00012

3.9e-05

3.9e-05

0.0034820.003242

5.9e-05

5.3e-05

0.0034820.00313

0.0001666.2e-050.000185

0.0001666.2e-050.000185

0.0001398.9e-050.000152

0.0001398.9e-050.000152

4e-05

4e-05

5.9e-05

5.9e-05

0.0090160.008920.0092160.0231570.0068010.008780.007192

7e-05

2.3e-05

4.7e-05

0.0001356.6e-050.000111

0.0001356.6e-050.000111

3.6e-052.2e-054.3e-05

1.3e-054e-061.7e-05

1.4e-05

2.3e-054e-062.6e-05

0.001250.0009010.0011410.0109210.0008070.001217

2.3e-050.0001411.4e-052.1e-05

0.0010580.000760.0011410.0109210.0006470.001023

7.5e-053.7e-057.3e-05

1.6e-051.3e-051.9e-05

5.5e-05

7.8e-054.1e-058.1e-05

9.2e-059.8e-052.7e-058.3e-05

9.2e-059.8e-052.7e-058.3e-05

9.4e-057.4e-058.2e-05

9.4e-057.4e-058.2e-05

8.8e-05

5.3e-05

3.5e-05

0.0070990.007820.0080750.0122360.0052240.0069360.007192

0.0070990.007820.0080750.0122360.0051810.0069360.007192

4.3e-05

0.000111

4.8e-05

6.3e-05

6.6e-054.7e-057e-05

6.6e-054.7e-057e-05

0.000140.000110.000137

7.8e-055.8e-057.2e-05

6.2e-055.2e-056.5e-05

1.5e-054.1e-056e-06

1.5e-054.1e-056e-06

7.6e-05

7.6e-05

8.9e-050.0001013.8e-059.5e-05

8.9e-050.0001013.8e-059.5e-05

0.0003913.7e-050.0007420.0003340.000263

0.0002060.0002910.00020.000263

2.8e-05

4e-062e-063e-06

1.7e-05

4.8e-05

3.8e-05

0.0001336.7e-050.0001350.000263

9e-064e-068e-06

5.4e-052.7e-054.9e-05

5e-06

3.6e-05

6e-0605e-06

1.9e-05

4.9e-05

4.9e-05

2.9e-058.9e-052.3e-05

5.5e-05

2.9e-053.4e-052.3e-05

0.0001213.7e-054.7e-057.8e-05

1.7e-05

0.0001213.7e-053e-057.8e-05

6.8e-05

6.8e-05

8.7e-05

8.7e-05

7e-065.3e-059e-06

6e-063.8e-059e-06

1e-061.5e-05

2.8e-055.8e-052.4e-05

2.8e-055.8e-052.4e-05

0.0017610.0084810.001659

0.0002810.0004730.000241

5.7e-050.0001924.5e-05

0.000105

5.3e-05

5.2e-05

5.7e-058.7e-054.5e-05

4.2e-053.7e-053.3e-05

1.5e-055e-051.2e-05

0.0002240.0002810.000196

0.0002240.0002290.000196

3.3e-052.7e-052.4e-05

2.4e-05

1.2e-056e-061.3e-05

7e-062.5e-059e-06

1.3e-05

2.6e-055.6e-052.8e-05

2.5e-053.3e-052.1e-05

5.7e-052e-055.4e-05

3.2e-051.4e-052e-05

3.2e-051.1e-052.7e-05

5.2e-05

5.2e-05

4e-052.8e-052.6e-05

4e-052.8e-052.6e-05

4e-052.8e-052.6e-05

4e-052.8e-052.6e-05

7.1e-056.9e-056.4e-05

7.1e-056.9e-056.4e-05

7.1e-056.9e-056.4e-05

7.1e-056.9e-056.4e-05

5.3e-050.0001774.5e-05

1.8e-054.9e-051.1e-05

1.8e-054.9e-051.1e-05

1.8e-054.9e-051.1e-05

3.5e-050.0001283.4e-05

1.2e-053.6e-051.3e-05

1.2e-053.6e-051.3e-05

1.5e-056.1e-051.4e-05

1.5e-056.1e-051.4e-05

8e-063.1e-057e-06

8e-063.1e-057e-06

0.0001730.0003630.000149

5.5e-050.0001614.8e-05

1.4e-054.7e-051.3e-05

1.4e-054.7e-051.3e-05

3e-063.3e-052e-06

3e-063.3e-052e-06

9e-064e-051.4e-05

9e-064e-051.4e-05

2.9e-054.1e-051.9e-05

2.9e-054.1e-051.9e-05

0.0001180.0002020.000101

4e-056e-053.7e-05

4e-056e-053.7e-05

5.4e-055.3e-054.4e-05

5.4e-055.3e-054.4e-05

9e-063.8e-059e-06

9e-063.8e-059e-06

1.5e-055.1e-051.1e-05

1.5e-055.1e-051.1e-05

0.0003160.0004740.000322

0.000250.0003660.000255

0.0002460.0003410.000251

1.1e-054e-051e-05

4.2e-052.4e-053.8e-05

3.6e-054.1e-053.1e-05

1.8e-054e-052.4e-05

2.9e-05

4.8e-05

5.3e-054.9e-053.7e-05

1e-061e-06

7e-067e-06

1.7e-052.6e-051.9e-05

4.7e-057.2e-05

1.4e-054.4e-051.2e-05

4e-062.5e-054e-06

4e-062.5e-054e-06

3e-054e-053.8e-05

3e-054e-053.8e-05

3e-054e-053.8e-05

3.6e-056.8e-052.9e-05

3.6e-056.8e-052.9e-05

3.6e-052.7e-052.9e-05

4.1e-05

0.0004390.0059510.000436

4.9e-058.4e-052.1e-05

4.9e-058.4e-052.1e-05

3.9e-054.9e-051.3e-05

1e-053.5e-058e-06

0.000390.0058670.000415

0.000390.0058670.000415

0.000390.0058670.000415

3e-060.000101

3e-060.000101

3e-065.3e-05

3e-065.3e-05

4.8e-05

4.8e-05

0.0003850.0008450.000376

0.0001550.000180.000159

0.0001550.000180.000159

5.2e-055.2e-055e-05

6.5e-059.5e-057.6e-05

3.8e-053.3e-053.3e-05

8.7e-05

8.7e-05

8.7e-05

3.7e-059.1e-053.5e-05

3.7e-055e-053.5e-05

3.7e-055e-053.5e-05

4.1e-05

4.1e-05

3.5e-050.000163.4e-05

3.5e-050.0001083.4e-05

3.5e-050.0001083.4e-05

5.2e-05

5.2e-05

8.1e-05

8.1e-05

8.1e-05

0.0001070.0001510.000104

3.3e-053.9e-053.3e-05

3.3e-053.9e-053.3e-05

7.4e-050.0001127.1e-05

2.1e-052.7e-052e-05

2.7e-053.1e-052.7e-05

2.6e-05

2.6e-052.8e-052.4e-05

5.1e-059.5e-054.4e-05

5.1e-059.5e-054.4e-05

5.1e-059.5e-054.4e-05

5.8e-050.0001285.9e-05

5.8e-050.0001285.9e-05

5.8e-050.0001285.9e-05

5.8e-050.0001285.9e-05

2.8e-053.2e-053.3e-05

2.8e-053.2e-053.3e-05

3e-05

3e-05

3e-056.6e-052.6e-05

3e-054.2e-052.6e-05

2.4e-05

8.5e-05

8.5e-05

8.5e-05

8.5e-05

8.5e-05

8.5e-05

0.0035870.0002870.0263520.0261720.0038530.000255

8.2e-050.0001156e-05

8.2e-050.0001156e-05

8.2e-050.0001156e-05

8.2e-050.0001156e-05

4.5e-056.7e-052.8e-05

3.7e-054.8e-053.2e-05

0.0035050.0002870.0263520.0260570.0037930.000255

0.0001450.0001930.000153

1.4e-053.1e-051.7e-05

1.4e-053.1e-051.7e-05

1.4e-053.1e-051.7e-05

6.3e-059.5e-056.3e-05

4.3e-05

2.1e-05

2.2e-05

6.3e-055.2e-056.3e-05

6.3e-055.2e-056.3e-05

4.4e-055e-054.6e-05

1.3e-052.4e-051.3e-05

1.3e-052.4e-051.3e-05

3.1e-052.6e-053.3e-05

3.1e-052.6e-053.3e-05

2.4e-051.7e-052.7e-05

2.4e-051.7e-052.7e-05

2.4e-056e-062.7e-05

1.1e-05

1.8e-05

1.8e-05

1.8e-05

1.8e-05

0.000780.0001180.0010910.000747

2.7e-053.6e-052.6e-05

2.7e-053.6e-052.6e-05

2.7e-053.6e-052.6e-05

2.3e-052.5e-05

2.3e-052.5e-05

2.3e-052.5e-05

7e-062.4e-057e-06

7e-062.4e-057e-06

7e-061.1e-057e-06

1.1e-05

2e-06

0.000380.000610.000348

4e-054.7e-054.2e-05

2.1e-054.7e-051.9e-05

1.9e-052.3e-05

0.0002220.0004630.000199

2.2e-056.8e-052.1e-05

1.4e-057e-063.1e-05

1.3e-056e-051.8e-05

1.5e-053.2e-051.9e-05

1e-06

2.4e-053e-062.5e-05

1.4e-051.8e-05

1.8e-05

5e-06

1.9e-05

1.9e-055.3e-051e-05

1.5e-052.3e-059e-06

3e-06

4e-062.9e-056e-06

3.4e-053e-061.2e-05

7e-06

2e-06

4e-06

1.3e-056.3e-051.9e-05

1.9e-05

3.5e-054.4e-051.1e-05

1.7e-053.3e-052.2e-05

1.7e-053.3e-052.2e-05

0.0001016.7e-058.5e-05

0.0001016.7e-058.5e-05

3e-061.2e-052e-06

3e-061.2e-052e-06

9e-06

3e-063e-062e-06

8e-060.0001261e-05

8e-060.0001261e-05

5.7e-05

8e-063.5e-051e-05

3.4e-05

0.0003320.0001180.0002830.000329

0.0003320.0001180.0002830.000329

0.0003320.0001180.0002830.000329

7.3e-055.4e-057.7e-05

7.3e-055.4e-057.7e-05

7.3e-055.4e-057.7e-05

7.3e-055.4e-057.7e-05

0.0021130.0001690.0263520.0242450.0023830.000255

0.0005070.0001990.000564

0.000370.000150.000424

0.0002495.4e-050.000285

0.0001214.7e-050.000139

4.9e-05

0.0001374.9e-050.00014

0.0001374.9e-050.00014

0.0016060.0001690.000360.001819

0.0009370.0001340.0002390.001064

3.2e-05

0.0002032.7e-053.6e-050.000237

0.0002362.8e-054.7e-050.000269

0.0002253.4e-054.5e-050.000254

0.0002734.5e-057.9e-050.000304

0.0002020.000237

0.0002020.000237

0.0001281.4e-050.000139

0.0001281.4e-050.000139

0.0003393.5e-050.0001070.000379

2e-05

0.000233.5e-057.3e-050.000248

0.0001091.4e-050.000131

0.000255

0.000255

0.000255

0.0263520.023686

0.0263520.023686

0.0263520.023686

0.0002810.0003630.000306

8.9e-050.0001280.000107

8.9e-050.0001280.000107

5e-062e-06

2.4e-053.7e-052.7e-05

3e-061e-064e-06

2.5e-052.1e-053.1e-05

2.6e-053.3e-052.3e-05

1.1e-053.1e-052e-05

4e-056e-054.4e-05

4e-056e-054.4e-05

1.3e-053.3e-051.4e-05

2.7e-052.7e-053e-05

5.2e-053.8e-056.2e-05

5.2e-053.8e-056.2e-05

5.2e-053.8e-056.2e-05

6.4e-057e-056.4e-05

1.7e-052.6e-051.7e-05

1.7e-052.6e-051.7e-05

2e-051.6e-05

2e-051.6e-05

2.7e-054.4e-053.1e-05

2.7e-054.4e-053.1e-05

3.6e-056.7e-052.9e-05

3.6e-056.7e-052.9e-05

3.6e-056.7e-052.9e-05

0.0001139.3e-050.000127

6.5e-054.1e-057.1e-05

6.5e-054.1e-057.1e-05

6.5e-054.1e-057.1e-05

4.8e-055.2e-055.6e-05

4.8e-055.2e-055.6e-05

4.8e-055.2e-055.6e-05

0.0027830.0019750.0012370.0110220.0019690.0027260.000281

0.0027830.0019750.0012370.0110220.0019690.0027260.000281

0.0027830.0019750.0012370.0110220.0019690.0027260.000281

0.0027830.0019750.0012370.0110220.0019690.0027260.000281

0.0027830.0019750.0012370.0110220.0019690.0027260.000281

0.0027830.0019750.0012370.0110220.0019470.0027210.000281

2.2e-055e-06

6e-058.4e-055.6e-05

6e-058.4e-055.6e-05

6e-058.4e-055.6e-05

6e-058.4e-055.6e-05

6e-058.4e-055.6e-05

6e-058.4e-055.6e-05

0.003240.0016990.0027440.0110220.0028190.0031410.001457

7.3e-050.0001064.7e-05

7.3e-050.0001064.7e-05

7.3e-050.0001064.7e-05

7.3e-050.0001064.7e-05

7.3e-050.0001064.7e-05

0.0031670.0016990.0027440.0110220.0027130.0030940.001457

0.0031670.0016990.0027440.0110220.0027130.0030940.001457

0.0031430.0016990.0027440.0110220.0026190.0030750.001457

1.2e-054.3e-054e-06

1.2e-054.3e-054e-06

0.0031080.0016990.0027440.0110220.0023930.0030520.001457

0.0031080.0016990.0027440.0110220.0023930.0030520.001457

7.4e-05

7.4e-05

3e-065.7e-052e-06

3e-065.7e-052e-06

2e-055.2e-051.7e-05

2e-055.2e-051.7e-05

2.4e-059.4e-051.9e-05

3e-065.7e-052e-06

3e-065.7e-052e-06

2.1e-053.7e-051.7e-05

2.1e-053.7e-051.7e-05

0.0001010.0005938.4e-05

2.6e-054.9e-059e-06

2.6e-054.9e-059e-06

2.6e-054.9e-059e-06

2.6e-054.9e-059e-06

2.6e-054.9e-059e-06

8.1e-05

8.1e-05

8.1e-05

8.1e-05

8.1e-05

3.2e-050.0002092.9e-05

3.2e-050.0002092.9e-05

3.2e-050.0002092.9e-05

3.2e-050.0002092.9e-05

9.6e-05

3.2e-050.0001132.9e-05

3.6e-05

3.6e-05

3.6e-05

3.6e-05

3.6e-05

4.3e-050.0002184.6e-05

8e-066.4e-052e-05

8e-066.4e-052e-05

8e-066.4e-052e-05

8e-066.4e-052e-05

3.5e-050.0001542.6e-05

3.5e-050.0001542.6e-05

3.5e-054.5e-052.6e-05

3.5e-054.5e-052.6e-05

6.7e-05

6.7e-05

4.2e-05

4.2e-05

4.3e-050.0003183.6e-05

4.3e-050.0003183.6e-05

4.3e-050.0003183.6e-05

4.3e-050.0003183.6e-05

1.3e-057.8e-051.8e-05

1.3e-057.8e-051.8e-05

9.1e-05

9.1e-05

1e-055.4e-057e-06

1e-055.4e-057e-06

2e-064.9e-05

2e-064.9e-05

1.8e-054.6e-051.1e-05

1.8e-054.6e-051.1e-05

0.0325680.0158070.081130.0410550.0591730.0331360.054264

8.7e-059.8e-057.8e-05

8.7e-059.8e-057.8e-05

8.7e-059.8e-057.8e-05

1.8e-054.6e-051.6e-05

1.8e-054.6e-051.6e-05

6.9e-055.2e-056.2e-05

6.9e-055.2e-056.2e-05

0.0001480.0011840.000143

0.0001480.0011840.000143

1.7e-050.0001811.8e-05

7.7e-05

7.7e-05

1.7e-054.9e-051.8e-05

1.7e-054.9e-051.8e-05

5.5e-05

5.5e-05

2.4e-050.0002072.4e-05

3.9e-05

3.9e-05

7e-064.3e-056e-06

2.7e-05

7e-061.6e-056e-06

3e-069.2e-053e-06

3e-069.2e-053e-06

1.4e-053.3e-051.5e-05

1.4e-053.3e-051.5e-05

5e-063.1e-056e-06

5e-063.1e-056e-06

5e-063.1e-056e-06

5.9e-05

5.9e-05

5.9e-05

1.3e-050.0001761e-05

5e-060.0001215e-06

5e-060.0001215e-06

8e-065.5e-055e-06

8e-065.5e-055e-06

0.000315

7.3e-05

1.8e-05

3e-05

2.5e-05

0.000176

1.8e-05

2.5e-05

7e-06

2.7e-05

2e-05

3.7e-05

4.2e-05

6.6e-05

3.5e-05

3.1e-05

8.9e-050.0002158.5e-05

4.4e-05

4.4e-05

3.3e-051.2e-05

3.3e-051.2e-05

1.1e-053.7e-058e-06

1.1e-053.7e-058e-06

1.3e-052e-052.2e-05

1.3e-052e-052.2e-05

7e-065.7e-051.1e-05

7e-065.7e-051.1e-05

1.8e-053.3e-052.4e-05

1.8e-053.3e-052.4e-05

7e-062.4e-058e-06

7e-062.4e-058e-06

0.0019270.0003920.0083510.0020320.000256

0.0019270.0003920.0083510.0020320.000256

7e-064.3e-058e-06

7e-064.3e-058e-06

7e-064.3e-058e-06

0.0017970.0003920.0081160.0018940.000256

0.000129

0.000129

0.0001860.0004730.000189

0.0001860.0004730.000189

1.3e-056.9e-052.3e-05

1.3e-056.9e-052.3e-05

0.00168

0.00168

9.4e-059.2e-059.5e-05

9.4e-059.2e-059.5e-05

2.1e-056.6e-051.6e-05

2.1e-056.6e-051.6e-05

0.000394

0.000394

0.000368

0.000368

1.7e-058e-052e-05

1.7e-058e-052e-05

0.000272

0.000272

0.000790.0003920.0003460.0008040.000256

0.0007750.0003928.8e-050.0007830.000256

7.6e-05

1.5e-057e-052.1e-05

0.000112

3.6e-050.0001543.5e-05

3.6e-050.0001543.5e-05

2.6e-059.7e-052.9e-05

2.6e-059.7e-052.9e-05

1.9e-050.0001352.5e-05

7.2e-05

1.9e-056.3e-052.5e-05

3.8e-050.0001844.4e-05

3.8e-058.8e-054.4e-05

9.6e-05

2.3e-056.7e-052.7e-05

2.3e-056.7e-052.7e-05

0.0001060.0001410.000114

0.0001060.0001410.000114

1.4e-05

3e-06

3e-06

8e-06

1.7e-051.1e-051.8e-05

1.7e-051.1e-051.8e-05

0.0001770.0001760.000188

4.6e-055e-054.7e-05

5.1e-051.7e-055e-05

3.1e-051.5e-053.2e-05

2.1e-055.1e-052.8e-05

2.8e-054.3e-053.1e-05

1.5e-059.4e-051.8e-05

1.5e-059.4e-051.8e-05

3.8e-050.0001394.2e-05

3.8e-050.0001394.2e-05

5e-050.0001486.8e-05

5e-050.0001486.8e-05

2.8e-05

2.2e-05

6e-06

0.001812

0.000297

0.000232

0.000401

0.000882

8.4e-05

8.4e-05

0.000111

0.000111

7.6e-050.0001299e-05

3.3e-056.1e-054.5e-05

4.3e-054.4e-054.5e-05

2.4e-05

0.000331

0.000176

0.000155

2e-059.4e-052.1e-05

2.7e-05

2e-054.6e-052.1e-05

2.1e-05

3.5e-050.0001982.8e-05

3.5e-050.0001982.8e-05

8.9e-05

8.9e-05

8.9e-05

0.0001025.3e-050.000116

0.0001025.3e-050.000116

2.4e-057e-062.4e-05

8e-065e-068e-06

8e-068e-06

2e-06

8e-061.2e-058e-06

1.5e-052e-051.5e-05

1.6e-054e-063e-05

2.3e-053e-062.3e-05

2.1e-055e-051.4e-05

2.1e-055e-051.4e-05

2.1e-055e-051.4e-05

3e-050.0004833.2e-05

3e-050.0004833.2e-05

3e-050.0004833.2e-05

3.9e-05

3.9e-05

5e-060.0001785e-06

5e-069.4e-055e-06

8.4e-05

6e-065.9e-051.1e-05

6e-065.9e-051.1e-05

9e-065.9e-057e-06

9e-065.9e-057e-06

1e-050.0001489e-06

4.1e-05

2.6e-05

2.5e-05

1e-055.6e-059e-06

2.5e-058.1e-052.7e-05

2.5e-058.1e-052.7e-05

1.2e-054.4e-051.5e-05

1.2e-054.4e-051.5e-05

1.2e-054.4e-051.5e-05

1.3e-053.7e-051.2e-05

1.3e-053.7e-051.2e-05

1.3e-053.7e-051.2e-05

0.0161820.0051110.0713890.0293250.037630.016970.050305

0.000193

3.5e-05

3.5e-05

3.5e-05

0.000158

0.000158

0.000158

0.0161820.0051110.0713890.0293250.0374370.016970.050305

0.0001290.0005040.000132

0.0001290.000150.000132

6.7e-050.0001147.5e-05

6.2e-053.6e-055.7e-05

0.000354

0.000354

3.7e-050.0002093.9e-05

3.7e-050.0002093.9e-05

3.7e-050.0002093.9e-05

0.0013560.0002650.0038070.0028140.0014280.003421

0.0013560.0002650.0038070.0028140.0014280.003421

0.000136

0.000471

0.0003458.5e-050.0003760.000363

9.5e-050.0004670.000118

0.0038070.003421

0.0001460.0001260.000187

0.0001230.0003230.000112

0.0003046.8e-050.0007240.00031

0.0003430.0001120.0001910.000338

0.0001070.0002130.000108

0.0001070.0002130.000108

0.0001070.0002130.000108

1.6e-050.000281.6e-05

1.6e-050.0001031.6e-05

1.6e-050.0001031.6e-05

0.000177

0.000177

0.000604

0.000604

0.000604

0.0029750.0015890.002370.0124380.000810.0029080.00115

0.0029750.0015890.002370.0124380.0006780.0029080.00115

0.0002675.4e-050.0006780.000255

0.0027080.0015350.002370.0124380.0026530.00115

0.000132

0.000132

0.000610.0017670.000626

0.0001760.0014580.000214

0.0001760.0010640.000214

0.000394

0.0004340.0003090.000412

0.0004340.0001430.000412

0.000166

0.0109520.0032570.0652120.0168870.0302360.0117130.045734

0.0109520.0032570.0652120.0168870.0302360.0117130.045734

0.0024670.0005660.0013750.0014660.0026148.9e-05

0.0009350.0001750.0004910.000978

0.000263

0.0505860.045467

8.9e-05

0.0053660.000910.0029150.014550.005789

0.001649

0.000562

0.0088660.007969

0.0010420.0011540.001470.0168870.0010080.001101

8.9e-05

0.0011420.0001890.0019690.001231

0.000572

0.0141690.0103040.0097410.011730.0113460.0138540.003703

0.0141690.0103040.0097410.011730.0113460.0138540.003703

0.0141690.0103040.0097410.011730.0113460.0138540.003703

0.000116

7.3e-05

4.3e-05

6e-064.4e-053e-06

6e-064.4e-053e-06

4.1e-05

4.1e-05

0.0141630.0103040.0097410.011730.0110930.0138510.003703

0.0141630.0103040.0097410.011730.0110930.0138510.003703

5.2e-05

5.2e-05

5.1e-050.0001913.9e-05

5.1e-050.0001913.9e-05

5.1e-050.0001913.9e-05

5.1e-050.0001913.9e-05

3e-064.2e-05

3e-064.2e-05

1.3e-056.1e-059e-06

1.3e-056.1e-059e-06

2.5e-053.7e-051.1e-05

2.5e-053.7e-051.1e-05

1e-055.1e-051.9e-05

1e-055.1e-051.9e-05

2.2e-050.0001532.9e-05

2.2e-050.0001532.9e-05

2.2e-050.0001532.9e-05

2.2e-050.0001532.9e-05

2.2e-050.0001532.9e-05

2.2e-050.0001532.9e-05

3.1e-059.4e-052.4e-05

3.1e-059.4e-052.4e-05

3.1e-059.4e-052.4e-05

3.1e-059.4e-052.4e-05

3.1e-059.4e-052.4e-05

3.1e-059.4e-052.4e-05

6.1e-05

6.1e-05

6.1e-05

6.1e-05

6.1e-05

6.1e-05

0.0003890.01082

0.0003890.01082

0.0003890.01082

0.0003890.01082

0.0003890.01082

0.0003890.01082

8.2e-050.0003448.9e-05

8.2e-050.0003448.9e-05

2.8e-050.0001572.7e-05

1.3e-057.7e-051.5e-05

4.8e-053e-06

2.6e-053e-06

2.2e-05

1.3e-052.9e-051.2e-05

1e-05

8e-061e-051e-05

5e-069e-062e-06

1.5e-058e-051.2e-05

6e-063.1e-053e-06

1.1e-05

3e-063e-06

3e-061e-06

0

1e-06

3e-06

1.5e-05

9e-064.9e-059e-06

7e-063.6e-057e-06

4e-06

2e-069e-062e-06

4.2e-059.7e-054.6e-05

4.2e-059.7e-054.6e-05

1.6e-053.5e-051.6e-05

1.6e-053.5e-051.6e-05

2.6e-053.4e-053e-05

2.6e-053.4e-053e-05

2.8e-05

2.8e-05

1.2e-059e-051.6e-05

1.2e-059e-051.6e-05

1e-051.6e-051e-05

1e-051.6e-051e-05

2e-067.4e-056e-06

3.1e-05

2e-064.3e-056e-06

0.0001820.0003940.000176

0.0001820.0003940.000176

7.5e-050.0002856.8e-05

3e-060.000151.3e-05

3e-06

3e-06

3e-064.3e-056e-06

3e-064.3e-056e-06

2e-057e-06

2e-057e-06

8.4e-05

3.6e-05

4.8e-05

7.2e-058.9e-055.5e-05

1.2e-053.6e-052.7e-05

1.2e-053.6e-052.7e-05

6e-055.3e-052.8e-05

1.5e-052.2e-051.2e-05

4.5e-053.1e-051.6e-05

4.6e-05

4.6e-05

4.6e-05

0.0001070.0001090.000108

0.0001070.0001090.000108

1.1e-056.7e-051.1e-05

1.1e-056.7e-051.1e-05

9.6e-054.2e-059.7e-05

9.6e-054.2e-059.7e-05

1e-054.1e-059e-06

1e-054.1e-059e-06

1e-054.1e-059e-06

1e-054.1e-059e-06

1e-054.1e-059e-06

1e-054.1e-059e-06

0.000250.0003110.000209

0.000250.0003110.000209

0.000250.0003110.000209

0.000250.0003110.000209

2.3e-054.8e-052.5e-05

1.2e-052.5e-051.4e-05

1.1e-052.3e-051.1e-05

6.4e-058.3e-056.6e-05

1.3e-052.5e-051.2e-05

2.7e-052.2e-053.2e-05

1.5e-051.9e-051.5e-05

9e-061.7e-057e-06

0.000155.2e-050.000102

9.6e-052.2e-055.6e-05

5.4e-052.1e-054.6e-05

9e-06

1e-057.7e-051.3e-05

1e-053.5e-051.3e-05

4.2e-05

3e-065.1e-053e-06

3e-065.1e-053e-06

2e-050.0001391.8e-05

2e-050.0001391.8e-05

2e-050.0001391.8e-05

1.3e-057.8e-051.3e-05

1.3e-057.8e-051.3e-05

1.3e-057.8e-051.3e-05

7e-066.1e-055e-06

7e-066.1e-055e-06

7e-066.1e-055e-06

0.0035840.0023790.0018640.0110220.003230.0034730.000544

0.0035840.0023790.0018640.0110220.003230.0034730.000544

0.0031110.0023790.0018640.0110220.0026340.0030660.000544

0.0031110.0023790.0018640.0110220.0026340.0030660.000544

0.0002690.0001410.0002730.000256

4e-06

1.4e-051.8e-051.4e-05

3.4e-05

0.0002020.0001410.0001630.000193

4.2e-054.3e-054.3e-05

1.1e-051.1e-056e-06

0.0001278e-050.00013

0.0001278e-050.00013

1.9e-058.6e-052.9e-05

1.1e-053.8e-051.9e-05

8e-064.8e-051e-05

0.0026960.0022380.0018640.0110220.0021950.0026510.000544

0.0026960.0022380.0018640.0110220.0021950.0026510.000544

0.0004730.0005960.000407

0.0004190.0004740.000355

0.0004190.0004740.000355

6.1e-054.5e-055.2e-05

5.1e-05

3.6e-05

5.2e-052.9e-054.7e-05

2.8e-05

2.6e-053.9e-051e-05

4.5e-053.9e-054.4e-05

1e-05

3.2e-053.1e-053e-05

0.0001330.0001059.2e-05

7e-056.1e-058e-05

5.4e-050.0001225.2e-05

5.4e-050.0001225.2e-05

5.4e-050.0001225.2e-05
