# Supplementary material for: MetaMeta: integrating metagenome analysis tools to improve taxonomic profiling
Source: Microbiome. 2017 Aug 14;5:101. doi: 10.1186/s40168-017-0318-y (PMC5557516; doi:10.1186/s40168-017-0318-y)
Supplement: Supplementary file 2 — Additional File with interactive charts for all CAMI toy set results on default, very-precise and very-sensitive mode. File prefix S, M, and H for low, medium and high complexity, respectively. (TAR 3573 kb) [file 40168_2017_318_MOESM2_ESM.tar › M1_S002__insert_5000_very-sensitive.html]

Javascript must be enabled to view this page.

magnitude
magnitudeUnassigned

clark.parsed\_profile
dudes.parsed\_profile
final.metametamerge.profile
gottcha.parsed\_profile
kaiju.parsed\_profile
kraken.parsed\_profile
motus.parsed\_profile

0.9999919999999970.9999970.9999959999999991.0000011.0000041.000021

0.07771800000000010.0671410.0513670.0618120.07502200000000010.07462500000000010.040862

2.2e-05

2.2e-05

1e-05

1e-05

3e-06

3e-06

7e-06

7e-06

1.2e-05

1.2e-05

1.2e-05

6e-06

6e-06

4e-061.8e-053e-06

4e-061.8e-053e-06

4e-061.8e-053e-06

4e-061.8e-053e-06

4e-061.8e-053e-06

4e-061.8e-053e-06

0.07770700000000010.0671410.0513670.0618120.0748080.07461400000000010.040862

0.001470.0010550.0014910.0104030.0011480.0014120.001429

0.001470.0010550.0014910.0104030.0011480.0014120.001429

0.001470.0010550.0014910.0104030.0011480.0014120.001429

3e-061.9e-053e-06

1e-06

3e-06

3e-061e-063e-06

1.1e-05

1e-06

2e-06

1.8e-05

1.8e-05

0.0014670.0010550.0014910.0104030.0011110.0014090.001429

9e-06

1e-06

5e-06

4e-06

4e-06

1e-06

1e-06

3e-06

1e-06

3e-06

1e-06

2e-06

1e-06

0.0014670.0010550.0014910.0104030.0010710.0014090.001429

1e-06

1e-06

2e-06

0.0033960.0025440.0036170.0107060.0032390.003263

0.0033960.0025440.0036170.0107060.0032390.003263

0.0033960.0025440.0036170.0107060.0032390.003263

0.0033960.0025440.0036170.0107060.0032390.003263

1.3e-05

0.0033960.0025440.0036170.0107060.0032260.003263

4.2e-05

4.2e-05

4.2e-05

4e-06

4e-06

2.7e-05

4e-06

1.2e-05

4e-06

7e-06

1.1e-05

6e-06

5e-06

4e-060.0001912e-06

4e-060.0001912e-06

4e-060.0001842e-06

3.9e-05

1.1e-05

1.3e-05

1.2e-05

3e-06

2e-060.000112e-06

4.5e-05

1.5e-05

1.4e-05

2e-061.3e-052e-06

1.3e-05

1e-05

1.1e-05

6e-06

1e-06

4e-06

2e-062.4e-05

2e-062.4e-05

7e-06

7e-06

7e-06

1.3e-050.0002891.4e-05

1.3e-059.1e-051.4e-05

9e-06

9e-06

9e-06

3.5e-05

3.5e-05

3.5e-05

1.4e-05

1.4e-05

1.4e-05

1.3e-053.3e-051.4e-05

1.3e-053.3e-051.4e-05

1.3e-05

1.3e-052e-051.4e-05

6.5e-05

6.5e-05

6.5e-05

6.5e-05

0.000133

0.000133

3.7e-05

3.7e-05

7.9e-05

7.9e-05

1.7e-05

1.7e-05

2e-060.0001142e-06

2e-060.0001142e-06

3.8e-05

3e-05

2e-06

2e-06

2e-06

2.4e-05

8e-06

8e-06

2e-067.6e-052e-06

2e-065.9e-052e-06

7e-06

1.5e-05

04e-06

2e-063.3e-052e-06

1.7e-05

1.7e-05

0.0697290.06160.0440420.0195940.0675340.0669530.038071

3e-062.4e-051e-06

3e-062.4e-051e-06

3e-062.4e-051e-06

1e-066e-061e-06

2e-061.8e-05

0.0697050.06160.0440420.0195940.0673450.066930.038071

4e-065.5e-052e-06

4e-063e-051e-06

4e-061.7e-051e-06

5e-06

8e-06

2.5e-051e-06

2.5e-051e-06

0.0696890.06160.0440420.0195940.0671940.0669180.038071

0.0696890.06160.0440420.0195940.0671940.0669180.038071

0.0696890.06160.0440420.0195940.0671940.0669180.038071

2e-063.4e-052e-06

2e-063.4e-052e-06

2e-063.4e-052e-06

1e-056.2e-058e-06

6e-061.4e-054e-06

6e-061.4e-054e-06

4e-064.8e-054e-06

2e-062.9e-052e-06

2e-061.9e-052e-06

2.1e-050.0001652.2e-05

1.1e-053.6e-051e-05

1.1e-053.6e-051e-05

1e-061.9e-051e-06

1e-051.7e-059e-06

1e-050.0001291.2e-05

2e-063.4e-051e-06

1e-06

4e-06

2e-06

1e-062e-061e-06

3e-06

0

0

2e-06

4e-06

1e-063e-06

6e-06

7e-06

6e-061.4e-056e-06

6e-061.4e-056e-06

1.8e-05

1.8e-05

2e-068e-062e-06

2e-068e-062e-06

8e-063e-06

8e-063e-06

1.6e-05

5e-06

1.1e-05

3.1e-05

3.1e-05

0.0030930.0019420.0022170.0211090.0022510.0029680.001362

3.3e-057.4e-053.4e-05

3.3e-057.4e-053.4e-05

5e-061.2e-053e-06

5e-061.2e-053e-06

1e-051.4e-051e-05

5e-067e-064e-06

5e-067e-066e-06

2e-067e-063e-06

2e-067e-063e-06

8e-06

8e-06

4e-069e-065e-06

4e-069e-065e-06

1e-061.1e-054e-06

1e-061.1e-054e-06

7e-065e-066e-06

7e-065e-066e-06

4e-068e-063e-06

4e-068e-063e-06

0.0019770.0012030.0011480.0107060.0011440.001910.000449

0.0019720.0012030.0011480.0107060.0011030.0019020.000449

3e-062.1e-051e-06

3e-062.1e-051e-06

0.0019650.0012030.0011480.0107060.0010720.0018960.000449

1.8e-051.9e-051.7e-05

0.0019470.0012030.0011480.0107060.0009210.0018790.000449

0.000132

4e-061e-055e-06

4e-061e-055e-06

5e-064.1e-058e-06

5e-064.1e-058e-06

5e-064.1e-058e-06

0.0010830.0007390.0010690.0104030.0010330.0010240.000913

0.0010760.0007390.0010690.0104030.0009710.0010180.000913

9e-062e-058e-06

9e-062e-058e-06

0.0010270.0007390.0010690.0104030.0008950.0009880.000913

4e-061.4e-056e-06

0.0010230.0007390.0010690.0104030.0008810.0009820.000913

1.9e-059e-066e-06

2e-06

2e-0601e-06

1.7e-057e-065e-06

2.1e-054.7e-051.6e-05

5e-061.6e-054e-06

1.6e-053.1e-051.2e-05

1.1e-05

1.1e-05

1.1e-05

7e-065.1e-056e-06

5e-063.5e-054e-06

5e-067e-064e-06

1.8e-05

1e-05

2e-061.6e-052e-06

2e-061.6e-052e-06

6e-06

6e-06

6e-06

6e-06

6e-06

6e-06

7e-060.0001688e-06

7e-060.0001688e-06

3.4e-05

2.7e-05

2.7e-05

2.7e-05

7e-06

7e-06

7e-06

4.8e-05

3.5e-05

3e-06

3e-06

0

8e-06

8e-06

1.7e-05

1.2e-05

5e-06

7e-06

7e-06

1.3e-05

6e-06

6e-06

6e-06

6e-06

1e-06

1e-06

1e-062.3e-051e-06

1e-062.3e-051e-06

1e-061.1e-051e-06

1e-06

0

1e-066e-061e-06

000

4e-06

9e-06

8e-06

1e-06

3e-06

3e-06

6e-066.3e-057e-06

8e-06

8e-06

1e-06

5e-06

1e-06

1e-06

6e-065.5e-057e-06

7e-06

7e-06

1.9e-05

1.9e-05

6e-062.2e-056e-06

6e-069e-066e-06

1.3e-05

7e-061e-06

3e-061e-06

4e-06

0.9222739999999970.9328560.9486289999999990.9381890.9249819999999970.9253949999999990.959138

0.0562650.0317340.0744410.0966570.0705310.0549860.042302

3.7e-058.9e-053.5e-05

3.7e-058.9e-053.5e-05

3.7e-058.9e-053.5e-05

2.7e-053.5e-052.3e-05

2.7e-053.5e-052.3e-05

1e-055.4e-051.2e-05

1e-055.4e-051.2e-05

0.0560190.0317340.0739990.0966570.0691280.0547190.042302

5.5e-059.3e-055.5e-05

5.5e-059.3e-055.5e-05

5.5e-054.6e-055.5e-05

5.5e-054.6e-055.5e-05

4.7e-05

4.7e-05

0.0001980.0004990.000390.0104030.0003410.0002330.00116

0.0001980.0004990.000390.0104030.0003410.0002330.00116

0.0001970.0004990.000390.0104030.0002530.0002310.00116

7e-062.5e-059e-06

2.5e-05

1e-06

1.4e-053e-061.4e-05

9.1e-050.0004990.000390.0104033.6e-050.0001350.00116

3.4e-051.3e-052.5e-05

1.4e-05

1.9e-059e-061.6e-05

2.5e-05

3.2e-05

3e-06

1e-06

7e-062e-061.5e-05

2.1e-051e-051.2e-05

5.2e-05

4e-062e-065e-06

2.1e-05

2.1e-05

1e-061.1e-052e-06

1e-061.1e-052e-06

5.6e-05

5.6e-05

0.0011010.0007820.0009590.0104030.0014140.0010710.000462

0.0011010.0007820.0009590.0104030.0014140.0010710.000462

4.7e-05

4.7e-05

0.000149

5e-05

2.3e-05

4.5e-05

3.1e-05

3e-05

3e-05

0.0010870.0007820.0009590.0104030.0011020.0010440.000462

0.0010870.0007820.0009590.0104030.0011020.0010440.000462

1.4e-058.6e-052.7e-05

1.4e-058.6e-052.7e-05

2.8e-055.1e-053e-05

2.8e-055.1e-053e-05

2.8e-055.1e-053e-05

2.8e-055.1e-053e-05

0.0002340.000170.000218

0.0002340.000170.000218

6e-056.3e-055.6e-05

6e-056.3e-055.6e-05

6.9e-055.3e-056.9e-05

6.9e-055.3e-056.9e-05

0.0001055.4e-059.3e-05

0.0001055.4e-059.3e-05

0.0001770.0001870.000175

0.0001770.0001870.000175

0.0001770.0001870.000175

3.8e-054e-053.9e-05

4.3e-053.9e-054.1e-05

2.8e-052.6e-053.1e-05

3.1e-053.4e-053.1e-05

3.7e-054.8e-053.3e-05

7e-057.1e-057.4e-05

7e-057.1e-057.4e-05

7e-057.1e-057.4e-05

7e-057.1e-057.4e-05

0.0173840.0104330.0133920.0118170.0145550.0167350.011393

7.8e-054.4e-058.2e-05

7.8e-054.4e-058.2e-05

7.8e-054.4e-058.2e-05

6.1e-054.6e-055.9e-05

6.1e-054.6e-055.9e-05

6.1e-054.6e-055.9e-05

0.0172450.0104330.0133920.0118170.0144650.0165940.011393

4.9e-056.7e-053.7e-05

4.9e-056.7e-053.7e-05

0.0171960.0104330.0133920.0118170.0143980.0165570.011393

0.0171220.0104330.0132590.0118170.0142660.016480.011393

7.4e-050.0001330.0001327.7e-05

0.0074170.0043230.017050.0210080.0100430.0072680.01112

0.0002535e-050.000240.0002080.000257

0.0002535e-050.000240.0002080.000257

0.0002535e-050.000240.0002080.000257

0.0001580.0001480.0001240.000164

0.0001580.0001480.0001240.000164

0.0001580.0001480.0001240.000164

0.0007820.0002150.0007310.0007280.000803

0.0005440.0001470.0005040.0005270.000565

0.0005440.0001470.0002990.0003240.000565

0.0002050.000203

0.0002386.8e-050.0002270.0002010.000238

0.0002386.8e-050.0002270.0002010.000238

7.1e-05

7.1e-05

7.1e-05

8e-062.8e-056e-06

8e-062.8e-056e-06

8e-062.8e-056e-06

0.0006150.0003040.0003350.0003190.000621

0.0006150.0003040.0003350.0003190.000621

0.0001810.00010.0001798.6e-050.000174

0.0002420.0001090.0001250.000242

0.0001929.5e-050.0001560.0001080.000205

0.0005077.4e-050.0008570.0017530.000494

8.1e-05

8.1e-05

6.5e-05

6.5e-05

7e-05

7e-05

5.3e-05

5.3e-05

9.4e-05

9.4e-05

0.0003285.2e-050.0008570.0011220.000322

0.0002980.000295

0.0003285.2e-050.0003120.0002810.000322

0.00011

0.0002470.000245

9.2e-05

9.9e-05

0.0001112.2e-052.8e-050.000109

0.0001112.2e-052.8e-050.000109

6.8e-054.7e-056.3e-05

6.8e-054.7e-056.3e-05

0.0001

0.0001

9.3e-05

7.1e-05

2.2e-05

0.0003014.3e-050.010110.0009460.0003070.009182

0.0092820.009182

0.0092820.009182

0.0003014.3e-050.0003480.0004710.000307

0.0003014.3e-050.0003480.0004710.000307

0.000280.000277

0.000280.000277

0.00020.000198

0.00020.000198

9.1e-050.0001179.3e-05

9.1e-058.2e-059.3e-05

9.1e-058.2e-059.3e-05

3.5e-05

3.5e-05

2.5e-050.0001212.7e-05

2.5e-050.0001212.7e-05

2.5e-050.0001212.7e-05

0.000160.0006590.0006520.000156

0.000160.000160.0001590.000156

0.000160.000160.0001590.000156

0.0002870.000284

0.0002870.000284

0.0002120.000209

0.0002120.000209

0.0045170.0036370.003970.0210080.0049760.004340.001938

5.8e-053.8e-055.2e-05

5.1e-051.4e-054.9e-05

7e-062.4e-053e-06

0.0033980.0031950.0031350.0107060.0029670.0032690.001692

0.0033980.0031950.0031350.0107060.0029670.0032690.001692

3.8e-054.4e-053.5e-05

3.8e-054.4e-053.5e-05

0.00013

0.00013

0.0001130.0002550.000108

0.0001

0.0001137.4e-050.000108

8.1e-05

0.0006160.0004420.0008350.0103020.0011550.000590.000246

6e-05

7e-06

0.000610.0004420.0005180.0103020.0005150.0005850.000246

6.5e-05

5.6e-05

0.0001790.000177

0.0001380.000136

6e-069e-065e-06

6e-06

5.2e-05

7.2e-05

0.0001040.0001010.000102

0.0001040.0001010.000102

1e-051.6e-051e-05

1e-051.6e-051e-05

0.000180.000270.000174

8.3e-057.9e-058.1e-05

7.3e-05

9.7e-050.0001189.3e-05

0.0005065e-060.0006290.000489

0.0005065e-060.0006290.000489

4.1e-05

4.1e-05

8.5e-050.0001658.2e-05

4.3e-05

8.5e-056.7e-058.2e-05

0

2e-06

2.4e-05

2.9e-05

4.2e-05

4.2e-05

0.0001035e-068.9e-050.000101

5e-05

1.8e-055e-061.1e-051.7e-05

1.1e-05

8.5e-051.7e-058.4e-05

8.3e-057.9e-05

8.3e-057.9e-05

4e-05

4e-05

6.5e-053.4e-056.5e-05

6.5e-053.4e-056.5e-05

5e-05

5e-05

6.6e-054.1e-055.8e-05

6.6e-054.1e-055.8e-05

0.0001040.0001270.000104

0.0001040.0001270.000104

0.0119850.0056680.0180050.0213110.0165650.0118270.002891

0.0014520.0001750.0047040.0034970.0014260.00241

0.0014520.0001750.0047040.0034970.0014260.00241

7.8e-053.7e-057.7e-05

4.8e-05

7.1e-05

0.0002630.00026

3.5e-05

6.4e-05

0.0001740.000172

9.7e-055.5e-059.5e-05

8.3e-055.5e-058.1e-05

5.2e-05

9e-063e-067e-06

6.9e-052.8e-056.7e-05

7.6e-05

6.7e-05

0.0004770.0001390.0002790.0003160.00049

5e-05

9.4e-05

4.8e-054.7e-054.1e-05

3.4e-05

0.000119

5.1e-05

0.0001380.0001318.4e-050.000123

1.1e-052.4e-059e-061.1e-05

0.0001370.000136

6e-05

1e-0601e-06

3.2e-055.1e-053.5e-05

0.0006510.000644

6.5e-052e-055.2e-05

2.1e-05

0.0001390.0001446e-050.000147

5.9e-05

3.4e-053.5e-053.1e-05

6e-061.2e-053e-069e-06

8.1e-05

0.0003520.000349

1.7e-05

3.2e-05

0.0024360.00241

0.0001650.0001370.0001020.000159

7.5e-056e-057.6e-05

7.5e-056e-057.6e-05

7.5e-056e-057.6e-05

2.3e-05

2.3e-05

2.3e-05

0.000160.0001570.0001450.000161

0.000160.0001570.0001450.000161

0.000160.0001570.0001450.000161

0.0066770.0044950.0060440.0109080.0057750.006399

0.0066160.0044950.0058670.0109080.00560.006335

1e-05

7.8e-054.9e-057.7e-05

0.000108

5.2e-05

1e-061e-062e-06

5.6e-05

3.7e-052e-064.1e-05

1.3e-05

6e-06

5e-062e-067e-06

6e-051.7e-056e-05

0.0059270.0044820.0058670.0109080.0049270.005716

1.2e-055e-061.1e-05

0

3.9e-051.3e-054e-05

6.3e-05

7.1e-054.3e-056.7e-05

5e-06

1e-051e-069e-06

6.2e-051.3e-058e-066.7e-05

0.0001081e-06

000

3e-062e-066e-06

2e-061e-063e-06

6e-06

9.5e-053.1e-050.000105

5.3e-05

5e-054e-055.7e-05

5e-061e-064e-06

5e-06

0

1e-055e-061.3e-05

1.1e-054e-068e-06

6.1e-05

6e-06

0

3e-053e-064.2e-05

6.1e-050.0001770.0001756.4e-05

6.1e-050.0001770.0001756.4e-05

0.0031540.0009980.0066370.0104030.0066480.0032790.000481

0.0015360.0003250.0012770.0018980.001590.00024

0.0003586.1e-050.0002490.0001510.0003750.00024

0.0003525e-050.000340.0002960.000371

0.0001730.000171

0.0008260.0002140.0005150.001280.000844

0.0016180.0006730.005360.0104030.004750.0016890.000241

0.0022280.002204

0.0002177.2e-050.0002285.1e-050.000234

1e-05

0.0003390.000335

0.0004380.00010.0002440.000470.000468

0.0002430.000241

2.5e-05

4.7e-05

0.0003377.2e-050.0003484e-050.000353

0.0004010.0003510.0004180.0104035.4e-050.0004010.000241

0.0011380.001126

3.1e-05

0.0002257.8e-050.0001740.0001160.000233

0.0004670.0004630.0004170.000486

0.0004670.0004630.0004170.000486

9.3e-05

0.0001420.000157.8e-050.000155

8.5e-05

0.0001580.0001644e-050.000166

0.0001670.0001490.0001210.000165

0.0004480.0006090.000443

0.0002530.0001650.000253

4.4e-056.8e-054.7e-05

4.4e-056.8e-054.7e-05

6.2e-051.7e-056e-05

2e-061e-062e-06

6e-051.6e-055.8e-05

3.2e-053.7e-052.7e-05

3.2e-053.7e-052.7e-05

5.6e-052.6e-056.2e-05

5.6e-051.8e-056.2e-05

8e-06

5.9e-051.7e-055.7e-05

5.9e-051.7e-055.7e-05

0.0001950.0004440.00019

8.5e-057.8e-058.2e-05

8.5e-057.8e-058.2e-05

8.4e-05

8.4e-05

0.000110.0002040.000108

0.000119.9e-050.000108

0.000105

7.8e-05

7.8e-05

5.9e-054.4e-055.1e-05

5.9e-054.4e-055.1e-05

5.9e-054.4e-055.1e-05

5.9e-054.4e-055.1e-05

0.0001250.0001079.9e-05

0.0001250.0001079.9e-05

0.0001250.0001079.9e-05

0.0001250.0001079.9e-05

5.7e-054.5e-055.4e-05

5.7e-054.5e-055.4e-05

5.7e-054.5e-055.4e-05

5.7e-054.5e-055.4e-05

0.0107560.0077460.0081320.0113120.0097860.0103420.004173

0.0107560.0077460.0081320.0113120.0097860.0103420.004173

0.0003278.6e-050.0002080.0002310.000317

9.4e-056.3e-058.7e-05

0.0002338.6e-050.0002080.0001680.00023

4.9e-054.6e-055.2e-05

4.9e-054.6e-055.2e-05

1.6e-051.7e-051.8e-05

9e-065e-061e-05

7e-061.2e-058e-06

0.0103640.007660.0079240.0113120.0094920.0099550.004173

5e-054.5e-054.9e-05

0.0101980.007660.0079240.0113120.0093750.0097920.004173

5.5e-053.3e-055.1e-05

6.1e-053.9e-056.3e-05

0.0054190.0022780.0160710.0104030.0144180.0055550.011103

0.0054190.0022780.0160710.0104030.0144180.0055550.011103

0.0052840.0022780.0160710.0104030.0143790.0054450.011103

3.8e-050.0001222e-064.1e-05

2.3e-05

8.8e-052.2e-051.9e-059.7e-05

2e-05

2e-05

2.6e-05

2.6e-05

0.0003690.0001230.0002290.000415

0.0003240.0001910.0002732.4e-050.000359

1.4e-05

5.6e-05

3e-05

2.4e-05

0.0003830.0001030.0002077e-060.000426

4.6e-05

2.3e-05

0.0003549.6e-050.0003681.4e-050.000374

3.5e-053e-063.7e-05

0.00012

0.0002430.00024

0.0002426e-060.000239

9.9e-05

1.6e-05

3e-05

0.0007190.00030.0005041e-050.000776

2e-06

0.0004130.0001020.000212.2e-050.00045

1.3e-05

0.0003546.1e-050.0003771e-050.000394

0.0002957.4e-050.0003183.1e-050.000338

0.0001450.000210.0002061.1e-050.000328

0.000860.0006380.0004280.0104030.000570.0008520.000119

3e-05

3.4e-05

3.1e-05

0.0115140.0126130.010385

1e-05

9e-06

0.0003098.7e-050.0003272e-050.000338

0.0003910.0001010.0001633.6e-050

1.4e-05

2e-05

3.2e-05

4e-05

1.1e-05

1e-05

9e-06

0.0002470.000244

0.0002072.8e-050.0002153.9e-050.00022

0.0001353.9e-050.00011

0.0001353.9e-050.00011

1.4e-050.0001031e-05

1.4e-050.0001031e-05

1.4e-050.0001031e-05

1.4e-050.0001031e-05

1.4e-056.6e-051e-05

3.7e-05

4.4e-056.2e-054.1e-05

4.4e-056.2e-054.1e-05

4.4e-056.2e-054.1e-05

4.4e-056.2e-054.1e-05

4.4e-056.2e-054.1e-05

0.0001510.0004420.0011490.000181

5.6e-050.0002890.0005675.7e-05

4.2e-050.0002890.0004234.2e-05

1.9e-050.0002890.0002862.4e-05

1.9e-050.0002890.0002862.4e-05

2.3e-050.0001371.8e-05

2.3e-057e-051.8e-05

6.7e-05

1.4e-050.0001441.5e-05

5e-05

5e-05

1.4e-059.4e-051.5e-05

1.4e-059.4e-051.5e-05

9.5e-050.0001530.0005820.000124

9.5e-050.0001530.0005820.000124

1.9e-050.0001521.9e-05

1.1e-059.6e-051.1e-05

8e-065.6e-058e-06

0.000105

0.000105

1.2e-051.2e-05

1.2e-051.2e-05

4e-050.0001530.0001515.2e-05

4e-050.0001530.0001515.2e-05

2e-057.3e-053.9e-05

2e-057.3e-053.9e-05

4e-060.0001012e-06

4e-060.0001012e-06

2.5e-052.8e-052.2e-05

2.5e-052.8e-052.2e-05

2.5e-052.8e-052.2e-05

2.5e-052.8e-052.2e-05

2.5e-052.8e-052.2e-05

2.5e-052.8e-052.2e-05

0.0280080.0196170.014847

0.0280080.0196170.014847

0.0280080.0196170.014847

0.0280080.0196170.014847

0.0280080.0196170.014847

0.0280080.0196170.014847

0.028420.0166540.0179780.0133320.0270020.0273870.010721

0.0284060.0166540.0179780.0133320.0269750.0273750.010721

0.0284060.0166540.0179780.0133320.0269750.0273750.010721

8e-063.5e-057e-06

4e-061.4e-053e-06

4e-061.4e-053e-06

4e-062.1e-054e-06

4e-062.1e-054e-06

0.0283980.0166540.0179780.0133320.026940.0273680.010721

2.7e-05

2.7e-05

5e-063.1e-053e-06

5e-063.1e-053e-06

0.0283870.0166540.0179780.0133320.0268380.027360.010721

0.0283870.0166540.0179780.0133320.0268380.027360.010721

1e-062.1e-052e-06

1e-062.1e-052e-06

5e-062.3e-053e-06

5e-062.3e-053e-06

1.4e-052.7e-051.2e-05

1.4e-052.7e-051.2e-05

1.4e-052.7e-051.2e-05

1.4e-052.7e-051.2e-05

1.4e-052.7e-051.2e-05

0.0022770.0017070.0023350.0105040.0023050.0021890.001926

0.0022470.0017070.0023350.0105040.0022360.002160.001926

0.0022470.0017070.0023350.0105040.0022360.002160.001926

0.0022470.0017070.0023350.0105040.0022360.002160.001926

1.6e-054.7e-051.4e-05

6e-062.2e-055e-06

1e-052.5e-059e-06

4e-062.6e-054e-06

4e-062.6e-054e-06

0.0022090.0017070.0023350.0105040.0021430.0021210.001926

0.0022030.0017070.0023350.0105040.0021260.0021170.001926

6e-061.7e-054e-06

1.8e-052e-052.1e-05

1.8e-052e-052.1e-05

2.1e-054.6e-052.1e-05

2.1e-054.6e-052.1e-05

2.1e-054.6e-052.1e-05

2.1e-054.6e-052.1e-05

2.1e-054.6e-052.1e-05

9e-062.3e-058e-06

9e-062.3e-058e-06

9e-062.3e-058e-06

9e-062.3e-058e-06

9e-062.3e-058e-06

2e-069.8e-055e-06

2e-069.8e-055e-06

2e-069.8e-055e-06

1e-063.3e-053e-06

1e-063.3e-053e-06

1e-063.3e-053e-06

1e-066.5e-052e-06

1e-066.5e-052e-06

1e-066.5e-052e-06

0.0218670.0430910.0253940.0476720.0246850.0511510.033334

0.0218670.0430910.0253940.0476720.0246850.0511510.033334

0.0017050.0008770.0012470.0007480.001696

0.0017050.0008770.0012470.0007480.001696

0.0017050.0008770.0012470.0007480.001696

0.0005530.0003150.0003990.0002960.000559

0.0005460.0003280.000450.000539

2.5e-053.6e-052.6e-05

0.0005810.0002340.0003980.0004160.000572

0.003250.0276660.0075280.0250480.013690.0032330.023995

0.0004050.0012380.0004330.0106050.0002190.000370.000497

1.6e-058e-061.2e-05

7e-06

1.3e-059e-06

1e-06

3e-063e-06

0.0003890.0012380.0004330.0106050.0002110.0003580.000497

5e-06

1.7e-055e-06

4.7e-05

1e-06

2e-061.1e-052e-06

3e-061e-063e-06

0.0003670.0012380.0004330.0106050.0001880.0003530.00045

0.0028450.0264280.0070950.0144430.0134710.0028630.023498

4.4e-050.0002513.6e-05

1.3e-057.3e-051.2e-05

7e-066.9e-057e-06

2.4e-050.0001091.7e-05

1e-063.6e-052e-06

1e-063.6e-052e-06

1.1e-055e-051e-05

9e-062.1e-059e-06

2e-062.9e-051e-06

2e-063.5e-054e-06

2e-063.5e-054e-06

0.0027870.0264280.0070950.0144430.0130990.0028110.023498

3.8e-050.0001580.0001563.7e-05

9e-064.4e-058e-06

1.6e-050.0001370.0001361.6e-05

5e-061.6e-055e-06

2e-064.1e-055e-06

3.9e-050.0006280.0006214.3e-05

3.2e-05

1.6e-051e-06

3.7e-05

0.0026580.0264280.0061720.0144430.0119610.002680.023498

2e-053.9e-051.6e-05

0.0169120.0145480.0166190.0226240.0102470.0462220.009339

0.0169120.0145480.0166190.0226240.0102470.0462220.009339

0.0169050.0145480.0166190.0226240.0102280.0462170.009339

4e-061.4e-054e-06

0.0034480.003411

0.0011680.0008760.0010030.0106050.0004470.001409

0.0005050.0005

0.0157330.0136720.0116630.0120190.0058560.0448040.009339

7e-061.9e-055e-06

7e-061.9e-055e-06

1e-050.0001389e-06

1e-050.0001389e-06

1e-050.0001389e-06

1e-050.0001389e-06

4e-067.2e-054e-06

3e-05

4e-064.2e-054e-06

6e-064.4e-055e-06

6e-064.4e-055e-06

2.2e-05

2.2e-05

2.7e-052.6e-052.8e-05

2.7e-052.6e-052.8e-05

2.7e-052.6e-052.8e-05

2.7e-052.6e-052.8e-05

2.7e-052.6e-052.8e-05

2.7e-052.6e-052.8e-05

0.0001980.0011890.0002850.0105040.0001960.0001690.00129

0.0001980.0011890.0002850.0105040.0001960.0001690.00129

3e-066e-055e-06

2e-063.9e-054e-06

2e-062.4e-054e-06

2e-064e-06

2.4e-05

1.5e-05

1.5e-05

1e-061.4e-051e-06

1e-061.4e-051e-06

1e-061.4e-051e-06

7e-06

7e-06

7e-06

0.0001950.0011890.0002850.0105040.0001360.0001640.00129

0.0001950.0011890.0002850.0105040.0001360.0001640.00129

0.0001950.0011890.0002850.0105040.0001360.0001640.00129

0

0

6e-06

3e-063e-06

2e-0602e-06

1e-061e-061e-06

2e-06

1e-06

0.0001890.0011890.0002850.0105040.0001230.0001610.00129

3.3e-050.0002783.4e-05

3.3e-050.0002783.4e-05

3.3e-050.0002783.4e-05

1.6e-050.0001871.7e-05

9e-062.9e-059e-06

9e-062.9e-059e-06

6e-067.9e-057e-06

1.9e-05

6e-063.2e-057e-06

2.8e-05

5.3e-05

5.3e-05

1e-062.6e-051e-06

1e-062.6e-051e-06

1.7e-059.1e-051.7e-05

8e-061e-057e-06

2e-06

8e-068e-067e-06

9e-068.1e-051e-05

9e-068.1e-051e-05

0.0046880.029550.0074390.0166650.0051160.0041190.02837

0.0046880.029550.0074390.0166650.0050250.0041190.02837

7.6e-050.0001580.0003796.6e-05

7.6e-050.0001580.0003796.6e-05

5e-050.0001580.0003444.7e-05

2.7e-054.3e-052.4e-05

8e-05

1.8e-056.4e-051.7e-05

5e-060.0001580.0001576e-06

2.6e-053.5e-051.9e-05

3e-06

2e-057e-061e-05

3e-061.7e-057e-06

3e-068e-062e-06

0.004580.029550.0072810.0166650.0045280.0040280.02837

0.004580.029550.0072810.0166650.0045280.0040280.02837

2e-066e-065e-06

6e-063e-06

2e-062e-06

0.0045780.029550.0072810.0166650.0045220.0040230.02837

4e-062.4e-056e-06

4e-062e-06

1e-061e-06

1.1e-055e-065e-06

1e-061e-061e-06

2e-062e-063e-06

2e-06

0.0045360.029550.0072810.0166650.0042980.0039870.02837

2e-06

4e-061.5e-05

2e-0601e-06

2.8e-05

9e-06

4e-062e-063e-06

1e-063e-061e-06

2.4e-05

1e-06

1e-0601e-06

3e-0603e-06

3.3e-05

1.1e-05

6e-06

1.7e-05

6e-063e-06

2e-066e-062e-06

4e-06

5e-06

2e-06

1e-06

3e-06

3e-061e-063e-06

2e-06

6e-063e-06

3.2e-050.0001182.5e-05

3e-050.0001062.3e-05

3e-050.0001062.3e-05

7e-06

1e-05

4e-06

1.6e-052.6e-059e-06

8e-061.9e-058e-06

3e-065e-063e-06

2e-06

8e-06

3e-069e-063e-06

1.4e-05

2e-06

2e-061.2e-052e-06

2e-061.2e-052e-06

2e-061.2e-052e-06

9.1e-05

9.1e-05

9.1e-05

9.1e-05

9.1e-05

3e-063.6e-053e-06

3e-063e-06

3e-063e-06

3e-063e-06

3e-063e-06

3e-063e-06

3.6e-05

3.6e-05

3.6e-05

3.6e-05

3.6e-05

8.1e-050.0081050.000536.7e-050.008018

5e-065.2e-054e-06

5e-065.2e-054e-06

5e-065.2e-054e-06

5e-065.2e-054e-06

5e-065.2e-054e-06

0.0081050.008018

0.0081050.008018

0.0081050.008018

0.0081050.008018

0.0081050.008018

2.2e-050.0001791.8e-05

6e-068.9e-057e-06

6e-068.9e-057e-06

6e-068.9e-057e-06

6e-068.9e-057e-06

1.6e-059e-051.1e-05

1.6e-059e-051.1e-05

1.6e-059e-051.1e-05

1.6e-059e-051.1e-05

9e-060.0001918e-06

9e-060.0001918e-06

9e-060.0001918e-06

9e-066e-058e-06

9e-066e-058e-06

0.000131

0.000131

1.9e-055.6e-051.3e-05

1.9e-055.6e-051.3e-05

1.6e-053.5e-051e-05

1.6e-053.5e-051e-05

1.1e-051.6e-056e-06

5e-061.9e-054e-06

3e-062.1e-053e-06

3e-062.1e-053e-06

0

3e-062.1e-053e-06

2.6e-055.2e-052.4e-05

1.9e-054.7e-051.7e-05

1.9e-054.7e-051.7e-05

1.9e-054.7e-051.7e-05

1.9e-054.7e-051.7e-05

7e-065e-067e-06

7e-065e-067e-06

7e-065e-067e-06

7e-065e-067e-06

0.0084180.0064830.0076510.0214120.0074990.0080670.004852

0.0084180.0064830.0076510.0214120.0074990.0080670.004852

0.0082880.0064830.0076510.0214120.0072490.0079580.004852

0.0082880.0064830.0076510.0214120.0072490.0079580.004852

9e-066.4e-059e-06

6e-063.5e-057e-06

3e-062.9e-052e-06

0.0007810.0006810.0009230.0104030.0007710.00075

0.0007810.0006810.0009230.0104030.0007710.00075

2.2e-055.5e-051.8e-05

2.2e-055.5e-051.8e-05

0.0074760.0058020.0067280.0110090.0063590.0071810.004852

6e-068e-068e-06

0.0074630.0058020.0067280.0110090.0063020.0071650.004852

5e-061.4e-055e-06

1.9e-05

2e-061.2e-053e-06

4e-06

0.000130.000250.000109

2.3e-054.8e-052.5e-05

2.3e-054.8e-052.5e-05

2.3e-054.8e-052.5e-05

0.0001070.0002028.4e-05

0.0001070.0002028.4e-05

2.2e-052e-051.5e-05

9e-06

1e-05

3.2e-052.9e-052.5e-05

1.2e-05

5e-061.6e-054e-06

9e-063.1e-058e-06

5e-068e-067e-06

2e-052.4e-051.2e-05

9e-06

1.4e-053.4e-051.3e-05

3.6e-050.0003233.1e-05

3.6e-050.0003233.1e-05

1e-067.2e-051e-06

1e-067.2e-051e-06

1e-064.8e-051e-06

1e-062.8e-051e-06

2e-05

2.4e-05

2.4e-05

2.3e-050.0001722.3e-05

8e-067.6e-058e-06

6e-063e-056e-06

6e-062.5e-056e-06

4e-06

1e-06

2e-064.6e-052e-06

1.9e-05

2e-06

2e-06

2e-061e-062e-06

1e-06

1e-06

1.8e-05

2e-06

0

1.5e-059.6e-051.5e-05

4e-062.3e-055e-06

5e-06

1e-067e-061e-06

3e-061.1e-054e-06

1.1e-057.3e-051e-05

8e-064.7e-057e-06

3e-062.6e-053e-06

1.2e-057.9e-057e-06

1.2e-057.9e-057e-06

8e-062.4e-056e-06

8e-062.4e-056e-06

2.6e-05

2.6e-05

4e-062.9e-051e-06

4e-062.9e-051e-06

1.6e-050.0001091e-05

1.6e-050.0001091e-05

1.6e-050.0001091e-05

1.6e-050.0001091e-05

2e-062.7e-052e-06

2e-062.7e-052e-06

2e-062.7e-051e-06

2e-062.7e-051e-06

7e-061.8e-056e-06

7e-061.8e-056e-06

5e-063.7e-051e-06

5e-063.7e-051e-06

0.3096280.2296520.1663180.0624180.2665980.2973180.169862

0.3096280.2296520.1663180.0624180.2665980.2973180.169862

0.3096280.2296520.1663180.0624180.2665980.2973180.169862

0.3096280.2296520.1663180.0624180.2665980.2973180.169862

0.3096280.2296520.1663180.0624180.2665980.2973180.169862

2e-060.0006780.0006711e-06

0.3096260.2296520.165640.0624180.2659270.2973170.169862

2.2e-050.0001861.3e-05

2.2e-050.0001861.3e-05

2.2e-050.0001861.3e-05

2.2e-050.0001861.3e-05

5e-060.0001053e-06

3.8e-05

5e-063.4e-053e-06

3.3e-05

6e-065.3e-051e-06

6e-065.3e-051e-06

1.1e-052.8e-059e-06

4e-0602e-06

6e-06

7e-062.2e-057e-06

1.2e-053e-051.1e-05

1.2e-053e-051.1e-05

1.2e-053e-051.1e-05

1.2e-053e-051.1e-05

1.2e-053e-051.1e-05

1.2e-053e-051.1e-05

3.4e-05

3.4e-05

3.4e-05

3.4e-05

3.4e-05

3.4e-05

0.0001070.000218.9e-05

0.0001070.000218.9e-05

0.0001070.000218.9e-05

0.0001070.000218.9e-05

1.4e-053.7e-051.4e-05

8e-062e-057e-06

6e-061.7e-057e-06

3.2e-052.9e-052.7e-05

1.8e-059e-061.6e-05

1.4e-051.1e-051.1e-05

9e-06

1.9e-055.1e-051e-05

1.1e-059e-063e-06

1e-061.2e-051e-06

2e-061.5e-052e-06

5e-061.5e-054e-06

1.5e-052.5e-051.1e-05

1.5e-052.5e-051.1e-05

2.7e-056.8e-052.7e-05

2.7e-051.9e-052.7e-05

4.9e-05

0.0057260.0017010.0130430.0109080.01650.0057630.001397

0.0056990.0017010.0130430.0109080.0163960.0057370.001397

7.2e-050.000530.0005257.4e-05

7.2e-050.000530.0005257.4e-05

7.2e-050.000530.0005257.4e-05

7.2e-050.000530.0005257.4e-05

5e-05

5e-05

5e-05

5e-05

0.0001030.0006690.00090.000117

1.6e-052e-051.5e-05

1.6e-052e-051.5e-05

9e-06

1.6e-051.1e-051.5e-05

5.1e-050.0006690.0007336.2e-05

5.1e-050.0006690.0006626.2e-05

5.1e-050.0006690.0006626.2e-05

7.1e-05

3.7e-05

3.4e-05

2.8e-050.0001133e-05

1.4e-056.1e-051.4e-05

1.4e-056.1e-051.4e-05

1.4e-055.2e-051.6e-05

1.4e-055.2e-051.6e-05

8e-063.4e-051e-05

8e-063.4e-051e-05

8e-063.4e-051e-05

0.000250.0008990.0012390.000261

5.6e-050.0002810.0002787.3e-05

5.6e-050.0002810.0002787.3e-05

5.6e-050.0002810.0002787.3e-05

4.9e-050.000150.0002565.1e-05

4.9e-050.000150.0002565.1e-05

3.6e-050.000150.0001483.6e-05

1.3e-050.0001081.5e-05

2.9e-050.0001480.0001461.5e-05

2.9e-050.0001480.0001461.5e-05

2.9e-050.0001480.0001461.5e-05

5.5e-050.000190.0002435.4e-05

1.4e-051.4e-05

1.4e-051.4e-05

3.2e-050.000190.0001883.1e-05

3.2e-050.000190.0001883.1e-05

9e-065.5e-059e-06

9e-065.5e-059e-06

6.1e-050.000130.0003166.8e-05

6.1e-050.000130.0003166.8e-05

8e-060.000130.0001299e-06

1.5e-054.6e-051.5e-05

1e-066e-061e-06

1.5e-056.5e-052.2e-05

1e-064e-062e-06

2.1e-056.6e-051.9e-05

0.0008430.0079580.0086310.000916

0.0074150.007336

0.0074150.007336

0.0074150.007336

0.0006260.0001410.0007690.000688

7.6e-058.3e-05

7.6e-058.3e-05

4.6e-055.2e-055.4e-05

4.6e-055.2e-055.4e-05

0.0003520.0001410.0005170.000377

9.3e-05

7.5e-058.5e-058.1e-05

0.0001010.0001060.0001

9.4e-059.4e-050.000108

8.2e-050.0001410.0001398.8e-05

0.0001520.00020.000174

5.1e-05

7.6e-050.00018.7e-05

7.6e-054.9e-058.7e-05

0.0002170.0004020.0005260.000228

4.9e-050.0001270.0001255e-05

4.9e-050.0001270.0001255e-05

0.0001680.0002750.0004010.000178

5.9e-050.0001330.0001326.1e-05

0.0001420.000141

0.0001090.0001280.000117

7.4e-050.000238.5e-05

2.6e-050.0001163.2e-05

2.6e-050.0001163.2e-05

2.6e-050.0001163.2e-05

4.8e-050.0001145.3e-05

4.8e-050.0001145.3e-05

4.8e-050.0001145.3e-05

0.0043570.0017010.0029870.0109080.0048210.0042840.001397

2e-063.2e-052e-06

2e-063.2e-052e-06

2e-064e-062e-06

2.8e-05

3.9e-053.9e-05

3.9e-053.9e-05

3.9e-053.9e-05

6e-061.2e-057e-06

6e-061.2e-057e-06

1e-06

1e-06

6e-061e-057e-06

0.004130.0017010.0028310.0109080.0038840.0040370.001397

0.004130.0017010.0028310.0109080.0038840.0040370.001397

0.004130.0017010.0028310.0109080.0038840.0040370.001397

2.7e-056e-052.3e-05

2.7e-056e-052.3e-05

2.7e-056e-052.3e-05

0.0001480.0005490.00017

0.0001080.0003720.000126

6e-062e-062.9e-05

7e-068e-066e-06

1.1e-052.2e-051e-05

1.1e-058.2e-051.1e-05

3e-06

1.2e-050.0001051.4e-05

1.5e-053.5e-051.5e-05

1.3e-05

7e-064e-066e-06

0

1.1e-053.2e-059e-06

1.3e-056e-061.4e-05

8e-061.3e-056e-06

3e-06

0

1.8e-05

1e-06

1.2e-05

4e-06

8e-06

7e-061e-066e-06

1.3e-057.5e-051.7e-05

1.3e-057.5e-051.7e-05

1e-057.3e-051.1e-05

2e-062e-06

8e-067.3e-059e-06

1.7e-052.9e-051.6e-05

1.7e-052.9e-051.6e-05

5e-060.0001560.0002846e-06

5e-060.0001560.0002846e-06

9.1e-05

0.0001560.000154

5e-063.9e-056e-06

2.7e-050.0001042.6e-05

2.7e-050.0001042.6e-05

2.7e-050.0001042.6e-05

2.7e-050.0001042.6e-05

2.1e-056.4e-052.1e-05

6e-064e-055e-06

0.005060.0013530.0170910.0211090.021680.0052570.003514

0.0040820.0013230.011550.0211090.0130160.0041550.003514

8.9e-05

7e-05

7e-05

7e-05

1.9e-05

1.9e-05

1.9e-05

0.0040820.0013230.011550.0211090.0129270.0041550.003514

0.0022780.0007530.0050520.0107060.0035210.0023210.002997

0.0022780.0007530.0050520.0107060.0035210.0023210.002997

0.0005320.000526

0.0001075.5e-050.0001050.000111

0.0001270.000125

0.0001114e-050.0001520.0004550.000114

0.001640.0005310.0007410.0107060.0010010.0016590.00025

0.0002290.000226

0.0002150.0002960.0007890.00023

0.0027770.002747

0.000115

7e-05

0.0002055.7e-050.0001980.0001790.000207

2.2e-050.000150.0002382.6e-05

0.000150.000149

0.000150.000149

2.2e-058.9e-052.6e-05

2.2e-058.9e-052.6e-05

0.0006550.000410.0008570.0104030.0004370.0006250.00027

0.000114

0.000114

0.0006550.000410.0008570.0104030.0003230.0006250.00027

0.0006210.000410.000530.0104030.0005910.00027

3.4e-050.0003270.0003233.4e-05

2e-050.000121.8e-05

2e-050.000121.8e-05

2e-050.000121.8e-05

0.0002620.00026

0.0002620.00026

0.0002620.00026

0.0006868.2e-050.0042430.0041110.0007190.000247

0.0006868.2e-050.0042430.0041110.0007190.000247

0.000250.000247

0.0002030.000201

7.6e-050.0006520.0006458e-05

7.9e-050.0008760.0008678.1e-05

0.0001010.0001380.0002540.000122

0.00038.2e-050.0003260.0003650.00031

0.0009470.000937

6.2e-050.0006310.0006245.2e-05

6.8e-050.000220.0002187.4e-05

1.6e-050.0001031.6e-05

1.6e-050.0001031.6e-05

1.6e-050.0001031.6e-05

4.1e-050.0001710.0002984.2e-05

0.0001710.00017

0.0001710.00017

4.1e-050.0001284.2e-05

1.4e-052.2e-051.2e-05

2.7e-050.0001063e-05

0.0003647.8e-050.0008150.0038390.000388

0.0002887.8e-050.0006040.0035250.000313

0.0002887.8e-050.0004360.0033590.000313

0.0001680.000166

7.6e-050.0002110.0003147.5e-05

7.6e-050.0001067.5e-05

0.0002110.000208

0.000830.0055410.007550.00094

0.000830.0055410.007550.00094

4e-065.5e-056e-06

4e-065.5e-056e-06

4e-065.5e-056e-06

5e-061.7e-055e-06

5e-061.7e-055e-06

5e-061.7e-055e-06

1e-05

1e-05

1e-05

0.0007820.0055410.0074160.000869

0.0002520.00025

0.0002520.00025

2e-059e-052.3e-05

2e-059e-052.3e-05

4.5e-050.0002570.0002544.8e-05

4.5e-050.0002570.0002544.8e-05

0.0002990.000295

0.0001530.000151

0.0001460.000144

8.4e-05

8.4e-05

0.0001540.000335

9.3e-05

4.6e-05

0.0001540.000152

4.4e-05

2.7e-050.0001940.0001923e-05

2.7e-050.0001940.0001923e-05

0.00270.002671

0.00270.002671

0.0001380.0001840.000148

1.6e-054.8e-051.8e-05

1.7e-054.4e-051.7e-05

3.9e-056.1e-054.4e-05

2.7e-051.7e-052.9e-05

3.9e-051.4e-054e-05

7.4e-050.0001588.5e-05

3e-054.3e-053.6e-05

3.4e-05

4.4e-058.1e-054.9e-05

2.6e-050.0001613.1e-05

2.6e-057.8e-053.1e-05

8.3e-05

0.000140.000139

0.000140.000139

5.7e-050.00016.5e-05

4.1e-059e-064.6e-05

1.4e-05

3.3e-05

1.6e-054.4e-051.9e-05

1.1e-055.4e-051e-05

1.1e-055.4e-051e-05

0.0002490.000246

0.0002490.000246

8e-060.0001079e-06

8e-060.0001079e-06

2.8e-050.0001043.2e-05

2.8e-056.8e-053.2e-05

1.4e-05

2.2e-05

1.4e-052.9e-051.2e-05

1.4e-052.9e-051.2e-05

1.9e-050.000172e-05

8.8e-05

1.9e-058.2e-052e-05

6.9e-050.000180.0001788e-05

6.9e-050.000180.0001788e-05

5e-066.2e-056e-06

5e-066.2e-056e-06

0.0001890.000186

0.0001890.000186

1.3e-05

1.1e-05

2e-06

4e-064e-064e-06

4e-064e-064e-06

1.3e-054.4e-051.5e-05

1.3e-054.4e-051.5e-05

0.0001270.000170.0003540.000139

0.0001270.000170.0003540.000139

5.2e-050.0004920.0004866.2e-05

5.2e-050.0004920.0004866.2e-05

0.000118

0.000118

3.5e-050.0002650.0002633.7e-05

3.5e-050.0002650.0002633.7e-05

1.2e-05

5e-06

3e-06

4e-06

1e-057.3e-051.3e-05

1e-057.3e-051.3e-05

3.9e-055.2e-056e-05

3.9e-055.2e-056e-05

1e-058e-061e-05

5e-062e-065e-06

4e-06

1e-052.4e-059e-06

9e-069e-061.8e-05

4e-06

5e-067e-065e-06

2e-065e-06

6.4e-050.0006137.1e-05

6.4e-050.0006137.1e-05

5e-063.3e-055e-06

3e-062.2e-053e-06

3e-062.2e-053e-06

2e-061.1e-052e-06

2e-061.1e-052e-06

3e-050.0001463.3e-05

4e-063.1e-056e-06

4e-063.1e-056e-06

3e-062.7e-054e-06

3e-062.7e-054e-06

9e-063.4e-057e-06

9e-063.4e-057e-06

4e-064e-06

4e-064e-06

2e-062.2e-053e-06

2e-062.2e-053e-06

8e-061.5e-059e-06

8e-061.5e-059e-06

1.7e-05

1.7e-05

7e-062.2e-059e-06

7e-062.2e-059e-06

7e-062.2e-059e-06

6e-069.9e-056e-06

2.9e-05

2.9e-05

6e-062.9e-056e-06

6e-062.9e-056e-06

4.1e-05

4.1e-05

1.6e-050.0001071.8e-05

1.6e-05

1.6e-05

1e-053e-051e-05

1e-053e-051e-05

3e-063.4e-054e-06

3e-061.6e-054e-06

1.8e-05

3e-062.7e-054e-06

3e-062.7e-054e-06

4.6e-05

4.6e-05

4.6e-05

0.00016

8.1e-05

1.2e-05

4e-06

1.1e-05

1.1e-05

7e-06

2.2e-05

1.4e-05

4.2e-05

1.1e-05

1.4e-05

1.7e-05

3.7e-05

2e-05

1.7e-05

4.4e-053e-050.0001854.4e-05

4.4e-053e-050.0001854.4e-05

4.4e-053e-050.0001854.4e-05

6e-05

3.4e-05

2.6e-05

2.1e-05

2.1e-05

4.3e-053e-056.2e-054.3e-05

4.3e-053e-056.2e-054.3e-05

1e-062e-051e-06

1e-062e-051e-06

2.2e-05

2.2e-05

4e-064.5e-053e-06

4e-064.5e-053e-06

3e-062.4e-052e-06

3e-062.4e-052e-06

3e-062.4e-052e-06

1e-062.1e-051e-06

1e-062.1e-051e-06

1e-062.1e-051e-06

1.9e-054.4e-052.5e-05

1.9e-054.4e-052.5e-05

1.9e-054.4e-052.5e-05

1e-051e-051e-05

1e-051e-051e-05

9e-063.4e-051.5e-05

9e-063.4e-051.5e-05

1.7e-050.0002271.9e-05

1.7e-050.0002271.9e-05

1.7e-050.0002271.9e-05

1.9e-05

1.9e-05

1e-066.4e-051e-06

1e-063.7e-051e-06

2.7e-05

4e-063.3e-055e-06

4e-063.3e-055e-06

9e-069.2e-051e-05

1.9e-05

2e-05

9e-063.1e-051e-05

2.2e-05

3e-061.9e-053e-06

3e-061.9e-053e-06

0.1174220.1592130.2258940.2099790.2094510.1152490.279868

0.0756620.1248570.1573220.1743260.1494720.0751340.258676

0.0245920.0406620.0547120.0798910.0280360.0239330.066925

0.0006410.0013520.0005730.0105040.0006170.0006130.001234

0.0006010.0013520.0005730.0105040.0005920.0005810.001234

8e-067e-069e-06

1e-061e-06

1e-063e-060

5.8e-05

4e-064e-064e-06

4e-069.5e-057e-06

0.0001820.0002017.4e-050.000180.000251

8e-065e-066e-06

1.9e-05

4e-06

6e-061e-055e-06

2.8e-05

01.3e-050

2.5e-052e-05

1.4e-05

3.3e-05

1e-060

6e-061.8e-055e-06

9e-06

9e-06

9e-06

2.4e-05

1.1e-05

9e-06

9e-06

6e-063e-064e-06

4.1e-051.4e-053.6e-05

2e-05

1.6e-05

3e-061e-067e-06

2e-06

4e-067e-066e-06

1.3e-05

0.0002990.0011510.0005730.0105044.8e-050.0002870.000983

4e-061e-064e-06

4e-052.5e-053.2e-05

2.6e-051.3e-052e-05

1.4e-057e-061.2e-05

5e-06

1.7e-050.0002061.4e-05

1.7e-050.0002061.4e-05

3.9e-05

1.9e-05

1.5e-05

1.7e-052.2e-051.4e-05

6.8e-05

4.3e-05

0.0014440.0012870.001210.001384

0.0014440.0012870.001210.001384

1.4e-051.8e-051.2e-05

0.001410.0012870.0011030.001352

5e-05

2e-053.9e-052e-05

0.0045570.0035930.0034390.021210.00170.0043880.004572

1.6e-055.7e-051.6e-05

1.6e-055.7e-051.6e-05

2.2e-053e-052.5e-05

2.2e-053e-052.5e-05

0.0045190.0035930.0034390.021210.0016130.0043470.004572

0.0004090.0010730.0004030.0105040.0001190.000390.001544

0.0003070.000303

1.1e-059e-069e-06

1.1e-058e-061.1e-05

4e-054.6e-053.8e-05

0.0040480.002520.0027290.0107060.0011130.0038990.003028

1.5e-05

4.8e-050.00024.9e-05

1.6e-058.2e-051.6e-05

7e-062.6e-055e-06

4e-061.1e-054e-06

2e-068e-061e-06

1e-062e-063e-06

2e-062e-053e-06

2e-06

1.3e-05

5e-062.8e-055e-06

5e-062.8e-055e-06

2.7e-059e-052.8e-05

4.1e-05

2.7e-053.6e-052.8e-05

1.3e-05

0.0178850.0357170.0494130.0481770.0241030.0174850.061119

0.0178460.0357170.0494130.0481770.0238720.0174330.061119

0.0004890.000484

0.0004677.8e-050.0003380.0002110.00048

1.1e-052.8e-059e-054.6e-05

5.3e-053.2e-050.0002940.0003290.000261

0.016750.01657

0.0001374.4e-050.0001497.5e-050.000159

0.0002380.000235

5.2e-058.1e-054.5e-05

0.0010080.000997

0.0010910.0002340.0004289.7e-050.00110.000299

0.0001320.0001360.0001385.7e-050.000141

0.0008580.0002580.0003260.0001610.000869

8.1e-052.6e-050.000340.0003368e-05

5.8e-050.0001099.7e-05

2.8e-051.3e-053.1e-052.4e-05

0.0020780.0024380.0016070.0106050.0005840.0013540.002956

0.0023650.0006390.0009230.017170.0002810.0023590.001867

7e-053.5e-052.7e-057.4e-05

0.0002750.000272

0.0029340.0007880.0006650.0003030.0029590.000425

0.0001037e-060.000112

0.0007960.000787

0.0001420.0001760.0003770.000133

3.9e-054e-056.9e-054e-05

0.000270.000267

0.0009580.0002890.0004090.0003050.0009530.000282

0.0001010.0001190.00011

0.0002040.000202

0.0061690.006102

3.9e-051e-052.9e-053.6e-05

0.0009130.000903

0.0056470.005586

0.0004610.0007760.0005336.5e-050.000448

0.0005177.8e-050.000380.0002420.000522

0.0003930.0002240.0001160.000422

9e-050.0004490.0004539e-069.1e-05

0.004280.0293260.008740.0204026.3e-050.0042080.041849

8.4e-053.5e-058.3e-05

0.0002240.0002720.0004440.000227

0.0002590.000256

3.9e-050.0002315.2e-05

2.7e-055.3e-052.6e-05

1.2e-054.9e-052.6e-05

0.000129

0.051070.0841950.102610.0944350.1214360.0512010.191751

1.3e-053.1e-058e-06

1.3e-053.1e-058e-06

1.3e-053.1e-058e-06

0.0028540.0010550.0716560.0103020.0969440.0029670.054574

8.1e-05

8.1e-05

0.0002762.8e-050.0701620.0947090.0002890.054574

0.0699650.0945880.054574

0.0002762.8e-050.0001970.0001210.000289

0.0025490.0010270.0014940.0103020.0021150.002655

1e-05

1.2e-05

1.1e-05

5.5e-05

3.8e-05

6.4e-05

2e-05

9e-06

0.0015380.0003690.0007990.000850.001898

1e-05

2e-05

1.7e-054.4e-051.8e-05

1.1e-051.5e-059e-06

1.2e-05

4.1e-054.7e-054.5e-05

2.3e-05

3.6e-05

8e-06

0.0005080.0003970.0002450.0001210.000262

1.9e-05

8.9e-05

8e-06

8.4e-05

3.8e-05

5.1e-05

3.3e-05

1e-05

0.0004340.0002610.000450.0103020.0003780.000423

2.9e-053.9e-052.3e-05

2.9e-053.9e-052.3e-05

0.0001350.0001850.000144

4.4e-05

4.4e-05

2.7e-056.7e-052.3e-05

2.7e-056.7e-052.3e-05

0.0001087.4e-050.000121

7e-068e-064e-06

1e-06

4e-06

1e-06

2.2e-054e-063e-05

2e-054e-062.6e-05

2.1e-05

9e-0607e-06

4e-06

1.6e-052e-061.6e-05

5e-06

5e-06

000

3e-063e-062e-06

1.5e-053e-061.3e-05

1e-06

9e-063e-061.1e-05

3e-06

7e-062e-061.2e-05

7.5e-050.00027.1e-05

0.00012

0.00012

7.5e-058e-057.1e-05

1.2e-051e-061.2e-05

1.2e-051.4e-051.8e-05

4.3e-054.7e-053.7e-05

8e-061.4e-054e-06

4e-06

0.0002650.0002920.000460.0104030.0006690.000494

0.0002610.0002920.000460.0104030.000630.000492

0.0002610.0002920.000460.0104030.000630.000492

4e-063.9e-052e-06

4e-063.9e-052e-06

0.0476220.0828480.0304940.073730.0228720.0474160.137177

7e-05

7e-05

5.6e-05

5.6e-05

3e-063e-064e-06

3e-063e-064e-06

4.4e-054.6e-054.3e-05

4.4e-054.6e-054.3e-05

6.2e-05

2.8e-05

3.4e-05

0.0005540.0001260.0006770.0006350.0005650.000259

0.0004490.000444

4.5e-05

0.0005540.0001260.0002280.0001460.0005650.000259

3.8e-055.9e-053.5e-05

3.8e-055.9e-053.5e-05

3.6e-05

3.6e-05

5.2e-05

5.2e-05

5.5e-05

3.6e-05

1.3e-05

6e-06

7e-063.4e-051.1e-05

7e-063.4e-051.1e-05

0.0322210.027010.0231960.0136350.0186610.030960.001027

7e-06

5e-060.0007755e-066e-060.000767

1e-06

3.1e-05

1.2e-055.9e-051.3e-05

1e-061e-058e-06

3.5e-05

2e-060.0002624e-062e-060.00026

2.9e-053.6e-052.8e-05

1.2e-05

2e-069e-062e-06

1.5e-05

0.032170.027010.0221590.0136350.0184240.030901

1.3e-05

0.0147550.0557120.0066210.0600950.0031030.0157980.135891

4.2e-05

6.4e-056e-06

2e-06

4e-061e-064e-06

3.4e-059e-053.1e-05

0.000116

4.2e-059e-064.1e-05

0.0059820.037320.0005980.0158570.0001030.0066650.040657

9.2e-05

8e-065.2e-057e-06

0.0003390.0003628.7e-050.000379

5.2e-05

0.0012980.0007630.0004970.0142410.0001080.0012960.091506

1.9e-052.7e-051.2e-05

4.3e-05

5e-055.3e-054.4e-05

5e-06

0.0039060.0006050.0006070.0002230.003882

9e-06

0.0001880.000186

9.8e-059.8e-050.000102

0.0005460.000541

0.000111

7e-05

9.9e-05

8.5e-05

4.8e-058.5e-053e-064.6e-05

3.9e-051.2e-053.3e-05

8.2e-05

0.0007520.0001620.0003439.9e-050.000759

0.0005070.0001260.0002770.0162610.0001540.000552

1e-052.3e-051.5e-05

5e-061e-066e-06

0.0014460.0166510.0030760.0137369.2e-050.0018160.003728

3.4e-05

0.000106

6.4e-051e-066.7e-05

8e-06

3.8e-055.2e-053.8e-05

2e-061e-063e-06

0.0001270.000125

6.2e-052.1e-055.2e-05

6.2e-052.1e-055.2e-05

1.4e-052e-061e-05

2.8e-056e-062.4e-05

1.2e-051e-061.3e-05

7e-061.2e-054e-06

1e-0601e-06

4.4e-050.0005144.9e-05

0.0001

0.0001

0.000135

0.000135

0.000104

1.8e-05

7e-06

7e-06

1.4e-05

1.2e-05

1.6e-05

5e-06

1.3e-05

1.2e-05

4.4e-052.9e-054.9e-05

4.4e-052.9e-054.9e-05

8.2e-05

8.2e-05

6.4e-05

6.4e-05

0.0367690.0311230.0470240.0247450.0508530.0353230.005855

0.0366890.0311230.0468520.0247450.0496160.0352320.005855

2.6e-054.6e-052.1e-05

2.6e-054.6e-052.1e-05

2.6e-054.6e-052.1e-05

1.9e-050.000980.0045410.0035131.8e-05

0.0008290.00082

0.0008290.00082

0.000980.000991

0.000980.000991

1.9e-050.0001530.0001521.8e-05

1.9e-050.0001530.0001521.8e-05

0.0025680.002541

0.0025680.002541

3.4e-050.0001113.2e-05

3.4e-050.0001113.2e-05

3.4e-050.0001113.2e-05

0.0002650.0010320.0053820.003010.0002290.002321

5e-060.0002750.0002726e-06

5e-060.0002750.0002726e-06

0.0001780.0017390.001544

0.001560.001544

0.0001780.000179

2.5e-050.0008540.0010170.0008613e-05

1.5e-050.0008540.0004510.0003029e-06

1e-050.0002350.0002322.1e-05

0.0003310.000327

0.0006170.000611

0.0006170.000611

5.9e-050.0008280.0002942e-050.000525

0.000270.000267

5.9e-050.0002970.0002942e-05

0.0002610.000258

1.1e-050.0001880.0001861.2e-05

1.1e-050.0001880.0001861.2e-05

0.0001650.0002150.000540.000161

0.0001650.0002150.000540.000161

0.0005030.0002460.000252

0.0002480.000246

0.0002550.000252

0.0004470.0001620.0049940.0044170.0004720.000257

2.9e-050.0024370.0025172.8e-05

2e-065.2e-053e-06

0.0010550.001044

2.1e-050.0001480.0001462e-05

6e-065.4e-054e-06

0.0002430.0002411e-06

0.0009910.00098

5.8e-050.0001990.0001976.3e-05

5.8e-050.0001990.0001976.3e-05

0.0002810.0001250.001640.001250.000294

0.000250.0002510.000247

0.0001250.000126

0.0007080.000701

3.1e-050.0005550.0005494.7e-05

3.7e-05

3.7e-05

0.000260.000257

0.000260.000257

7.9e-050.0004580.0004538.7e-05

7.9e-050.0004580.0004538.7e-05

0.0001140.0006280.000740.00011

0.0001050.0004740.0005880.000103

6.3e-050.0001196.4e-05

4.2e-050.0004740.0004693.9e-05

9e-060.0001540.0001527e-06

9e-060.0001540.0001527e-06

2.5e-056.1e-051.7e-05

2.5e-056.1e-051.7e-05

2.5e-056.1e-051.7e-05

0.0039330.0027930.0051860.0107060.0056290.0038210.003023

4.5e-050.0001345.1e-05

5e-065.4e-051.2e-05

4e-058e-053.9e-05

0.0038750.0027930.0051860.0107060.0053580.0037550.003023

6e-068.7e-053e-06

2.1e-05

0.0012940.00128

0.0005740.000567

8e-067.8e-059e-06

1.1e-058.3e-051.1e-05

1.2e-052.3e-051.2e-05

1.5e-053e-051.6e-05

0.000134

0.0037380.0027880.0033180.0107060.003890.0035980.001743

7e-05

3e-061.8e-054e-06

5.8e-05

5e-069e-068e-06

3e-052.4e-053.1e-05

2.1e-05

7e-061e-069e-06

1.3e-052.6e-059e-06

8e-062.7e-051.2e-05

7e-065e-067e-061.1e-05

7e-05

3.3e-05

6e-06

7e-064.1e-051.7e-05

5e-063.4e-055e-06

8.5e-05

8.5e-05

1.3e-055.2e-051.5e-05

6e-064.8e-058e-06

2e-06

7e-062e-067e-06

4e-060.000163e-06

3e-068.3e-052e-06

3e-068.3e-052e-06

1e-067.7e-051e-06

1e-067.7e-051e-06

5.1e-050.0007930.0007855.5e-05

5.1e-050.0007930.0007855.5e-05

5.1e-050.0007930.0007855.5e-05

0.0008560.000846

0.0008560.000846

0.0008560.000846

0.0001990.0004260.0003560.0005749.8e-050.000254

8e-060.0001510.0001496e-06

8e-060.0001510.0001496e-06

1.9e-050.0002050.0002031.8e-05

1.9e-050.0002050.0002031.8e-05

9.6e-05

9.6e-05

0.0001720.0004260.0001267.4e-050.000254

0.0001720.0004260.0001267.4e-050.000254

0.0315720.025730.0241160.0140390.0297240.030356

2e-055.7e-052.3e-05

2e-055.7e-052.3e-05

6e-06

6e-06

2.5e-050.0001242.4e-05

4e-064.9e-054e-06

2.1e-054e-052e-05

3.5e-05

0.031440.025730.0241160.0140390.0291830.030197

7e-064.8e-058e-06

1.2e-053.9e-051.2e-05

8e-064.7e-051.4e-05

6e-065.1e-056e-06

0.0314050.025730.0241160.0140390.0289980.030157

2e-06

1.2e-05

2e-06

1e-06

9e-06

2.7e-057.2e-053e-05

2.7e-057.2e-053e-05

2e-059.3e-052.2e-05

2e-059.3e-052.2e-05

3.4e-050.0001836e-05

3e-062.9e-051.2e-05

2e-053.4e-053.7e-05

6.3e-05

1.1e-055.7e-051.1e-05

6.6e-050.0001720.0008776.3e-05

4.4e-050.0003723.6e-05

2e-069.3e-051e-06

2e-069.3e-051e-06

3e-066.1e-051e-06

3e-066.1e-051e-06

2.5e-051.5e-052.4e-05

2.5e-051.5e-052.4e-05

6.7e-051e-06

6.7e-051e-06

1e-064.9e-051e-06

1e-064.9e-051e-06

8e-066.9e-054e-06

8e-066.9e-054e-06

5e-061.8e-054e-06

3e-062e-06

0

1.5e-05

2e-062e-06

3e-06

2e-050.0002512.5e-05

4e-065.8e-053e-06

1e-062e-061e-06

1e-065e-061e-06

2e-062.9e-051e-06

4e-06

0

5e-06

1.2e-05

1e-06

1.5e-057.4e-052.1e-05

5e-064.9e-051.1e-05

1e-052.5e-051e-05

1e-060.0001191e-06

1e-060.0001191e-06

8.3e-05

4e-05

4e-05

4.3e-05

4.3e-05

2e-060.0001720.0001712e-06

2e-060.0001720.0001712e-06

2e-060.0001720.0001712e-06

3e-064.8e-053e-06

3e-064.8e-053e-06

3e-064.8e-053e-06

3e-064.8e-053e-06

1.1e-050.0003122.5e-05

3e-060.0001898e-06

1e-060.0001267e-06

1e-066.3e-056e-06

6.3e-051e-06

2e-066.3e-051e-06

2e-066.3e-051e-06

8e-060.0001231.7e-05

3.1e-05

3.1e-05

8e-069.2e-051.7e-05

8e-069.2e-051.7e-05

1.1e-050.0004890.0005971.4e-05

1.1e-050.0004890.0005971.4e-05

1.1e-050.0004890.0005971.4e-05

0.0001810.000179

0.0001810.000179

1e-050.0001131.3e-05

1e-050.0001131.3e-05

1e-060.000160.0001591e-06

1e-060.000160.0001591e-06

0.0001480.000146

0.0001480.000146

5.5e-05

5.5e-05

5.5e-05

5.5e-05

5.5e-05

0.0047460.0032330.0066970.0109080.0052840.0045390.004398

4e-050.0003772.8e-05

8.8e-05

8.8e-05

6.3e-05

2.5e-05

4e-050.0002892.8e-05

4e-050.0002892.8e-05

5e-05

2.6e-050.0001081.4e-05

1.9e-05

4e-06

1.4e-050.0001081.4e-05

0.004640.0032330.0063390.0109080.0044780.0044550.004398

0.004640.0032330.0063390.0109080.0044780.0044550.004398

0.004640.0032330.0063390.0109080.0044780.0044550.004398

0.0046230.0032330.0039720.0109080.0043290.0044390.002206

1.7e-050.0001510.0001491.6e-05

0.0022160.002192

6.6e-050.0003580.0004295.6e-05

6.6e-050.0003580.0004295.6e-05

0.000190.000188

0.000190.000188

4.8e-057.5e-054.4e-05

4.8e-057.5e-054.4e-05

1.8e-050.0001680.0001661.2e-05

1.8e-050.0001680.0001661.2e-05

0.0002340.0143620.003190.0002390.010939

0.0002340.0143620.003190.0002390.010939

0.0002340.0143620.003190.0002390.010939

0.0002240.0002310.000233

0.0002240.0002310.000233

0.0003330.00033

0.0003330.00033

0.0136620.0025760.010939

0.0110580.010939

0.0026040.002576

1e-050.0001496e-06

1e-052.8e-056e-06

0.000121

0.0001360.000135

0.0001360.000135

1.2e-050.0001021.6e-05

1.2e-050.0001021.6e-05

1.2e-050.0001021.6e-05

1.2e-050.0001021.6e-05

1.2e-050.0001021.6e-05

1.2e-050.0001021.6e-05

1.7e-050.0002561.4e-05

6.3e-05

6.3e-05

6.3e-05

6.3e-05

6.3e-05

9e-069.1e-051e-05

9e-069.1e-051e-05

9e-069.1e-051e-05

9e-069.1e-051e-05

9e-069.1e-051e-05

8e-060.0001024e-06

8e-060.0001024e-06

8e-060.0001024e-06

8e-060.0001024e-06

8e-060.0001024e-06

2e-063.1e-052e-06

2e-063.1e-052e-06

2e-063.1e-052e-06

2e-063.1e-052e-06

2e-063.1e-052e-06

2e-063.1e-052e-06

0.3617970.3825210.3630380.4021820.2702659999999990.3532879999999990.373684

0.0418250.034210.0318240.0667610.0261170.0404440.029214

0.0003260.0001280.0001720.0002940.000127

4.6e-050.0001282.8e-054e-050.000127

4.6e-050.0001282.8e-054e-050.000127

4.6e-050.0001282.8e-054e-050.000127

0.0001588.1e-050.000148

6e-053.7e-055.8e-05

6e-052.1e-055.8e-05

1.6e-05

9.8e-054.4e-059e-05

4.4e-052.2e-053.5e-05

5.4e-052e-065.5e-05

1.8e-05

2e-06

0.0001226.3e-050.000106

8.4e-053.4e-057.5e-05

8.4e-053.4e-057.5e-05

3.8e-052.9e-053.1e-05

3.8e-052.9e-053.1e-05

0.0002190.0003530.000224

3.3e-050.0001094.7e-05

2e-063.6e-055e-06

2e-061.3e-055e-06

2.3e-05

3.1e-057.3e-054.2e-05

9e-061.7e-052e-05

8e-065e-069e-06

8e-061.1e-058e-06

2.3e-05

7e-06

6e-061e-055e-06

4.9e-050.0001056.5e-05

3.2e-05

1.4e-05

1.8e-05

1.4e-05

1.4e-05

2e-059e-062e-05

9e-068e-068e-06

1.1e-051e-061.2e-05

7e-063.2e-051.1e-05

5e-061.3e-057e-06

2e-061.9e-054e-06

2.2e-051.8e-053.4e-05

2.2e-051.8e-053.4e-05

9.3e-055.8e-058.3e-05

1.4e-052.7e-051.4e-05

1.4e-052.7e-051.4e-05

4.8e-051.7e-054.1e-05

4.8e-051.7e-054.1e-05

3.1e-051.4e-052.8e-05

3.1e-051.4e-052.8e-05

4.8e-05

2.1e-05

2.1e-05

2.7e-05

2.7e-05

4.4e-053.3e-052.9e-05

4.4e-053.3e-052.9e-05

4.4e-053.3e-052.9e-05

0.0011180.0039860.0018580.011110.0005720.0010810.006985

0.0007140.0037860.0016070.011110.0002890.0007070.006985

0.0007140.0037860.0016070.011110.0002280.0007070.006985

0.0002560.000253

0.0006160.0037860.0013510.011115.6e-050.0006190.006732

3.6e-05

8.2e-05

6.8e-054.5e-057e-05

3e-059e-061.8e-05

3.5e-05

3.5e-05

2.6e-05

2.6e-05

0.0004040.00020.0002510.0002830.000374

4.6e-052.4e-054.8e-05

4.6e-052.4e-054.8e-05

0.0002980.00020.0002510.000240.000279

0.0002980.00020.0002510.000240.000279

6e-051.9e-054.7e-05

6e-051.9e-054.7e-05

0.0282830.0194790.0200980.0441370.0137270.0274530.016584

0.0010410.0005510.0007640.0206040.0009170.0009930.000498

3.9e-05

1.6e-05

2.3e-05

0.000220.0002160.0002830.0103020.0002120.0002070.000498

2.3e-05

0.000220.0002160.0002830.0103020.0001890.0002070.000498

5.1e-053.3e-054.7e-05

5.1e-053.3e-054.7e-05

3.1e-051e-052.9e-05

3.1e-051e-052.9e-05

1.1e-051.8e-059e-06

1.1e-051.8e-059e-06

4.9e-05

2.1e-05

2.8e-05

2.7e-05

1e-05

1.7e-05

1.7e-051e-052.2e-05

6e-064e-068e-06

4e-06

1.1e-052e-061.4e-05

5.5e-052.4e-055e-05

5.5e-052.4e-055e-05

7.8e-054.9e-056.8e-05

3.3e-052.4e-052.6e-05

4.5e-052.5e-054.2e-05

1.6e-051e-051.8e-05

1.6e-051e-051.8e-05

3.2e-052.5e-052.6e-05

3.2e-052.5e-052.6e-05

2e-05

2e-05

0.000530.0003350.0004810.0103020.0003910.000517

1.8e-059e-062e-05

0.0004560.0003350.0004810.0103020.0003410.000434

8e-068e-061.1e-05

8e-063e-068e-06

1.4e-05

4e-051.6e-054.4e-05

0.0003556.1e-050.0002610.0003670.000344

5.1e-05

2.4e-05

2.7e-05

2.2e-051.3e-052.2e-05

2.2e-051.3e-052.2e-05

5.3e-052.5e-054.5e-05

7e-06

5.3e-051e-054.5e-05

8e-06

0.0002586.1e-050.0002610.0002060.000258

3.4e-05

0.0002586.1e-050.0002615.2e-050.000258

0.00012

2.2e-057.2e-051.9e-05

2.3e-05

3.3e-05

2.2e-051.6e-051.9e-05

0.0002320.0014990.0008250.0001910.000878

0.0014990.0006510.000878

0.0008880.000878

0.0006110.000605

2e-05

2.6e-05

5.8e-052.4e-054.8e-05

5.8e-052.4e-054.8e-05

1.6e-05

1.6e-05

2e-05

2e-05

3.4e-052.3e-053.1e-05

1.6e-051.4e-05

1.8e-052.3e-051.7e-05

6e-053.5e-055e-05

6e-053.5e-055e-05

8e-054.4e-056.2e-05

8e-054.4e-056.2e-05

1.2e-05

1.2e-05

0.0265090.0188670.0166860.0235330.0113040.0258070.01433

3.1e-050.0001082.5e-05

8e-06

3.8e-05

7e-06

8e-06

4e-06

1.2e-05

3.1e-054e-062.5e-05

1.4e-05

1.3e-05

6.5e-055.4e-056.1e-05

3.9e-052e-053.7e-05

2.6e-059e-062.4e-05

1e-05

1.5e-05

0.0009510.0002020.0009260.0007870.00089

0.0003287.5e-050.0002470.0001690.0003

0.0001353.1e-050.0001370.0001560.000121

3.3e-053.2e-054.4e-05

5.3e-05

0.00020.000198

0.0001692.3e-050.0001667.8e-050.00016

0.0002867.3e-050.0001760.0001010.000265

0.0001991.6e-050.0002490.000188

3.6e-05

4.9e-052.8e-054.4e-05

5.1e-055.4e-05

0.00013

4.4e-052.5e-054e-05

5.5e-051.6e-053e-055e-05

0.025250.0186490.015760.0235330.0100730.0246330.01433

8.9e-058e-067.9e-05

0.0002212.8e-050.0001610.0001040.000208

4e-051.8e-055e-064.4e-05

4e-06

1.2e-053e-061.4e-05

2.7e-05

6.5e-052.4e-056.8e-05

1e-0601e-06

1.1e-05

0

3.4e-05

3.2e-054e-063.1e-05

4.1e-052e-064.6e-05

7e-06

4.6e-057e-064e-05

4.5e-051.1e-051.1e-054.5e-05

0.0088420.0035640.0055590.0112110.0053430.0087010.003267

1.2e-053e-061.3e-05

4e-06

0.0001750.000173

3e-06

5.1e-05

0.000140.000139

9e-06

7e-06

3e-05

9.9e-055.3e-059.3e-05

3e-06

1.1e-05

1.9e-05

0.0155580.0149880.0097250.0123220.0039240.0150970.011063

0.0001081.5e-050.000113

3.6e-052.7e-051.5e-053.7e-05

4e-06

3e-061.3e-051e-063e-06

9e-06

6e-06

1.3e-053.3e-051e-05

1e-058e-067e-06

3e-061.5e-053e-06

1e-05

0.0008880.000878

0.0008880.000878

0.0008880.000878

0.0001460.0003140.000118

6.6e-050.0001345.8e-05

1.9e-05

5e-062e-065e-06

1.1e-05

1e-0601e-06

9e-06

1e-06

1.4e-05

3.8e-052.5e-052.8e-05

2.2e-05

1.9e-05

2.2e-051.2e-052.4e-05

000

1.7e-054e-051.7e-05

1.7e-054e-051.7e-05

2.1e-05

2.1e-05

3e-05

3e-05

1e-05

6e-06

4e-06

1.8e-052.8e-051.2e-05

1.8e-056e-061.2e-05

2.2e-05

3.6e-05

3.6e-05

4.5e-051.5e-053.1e-05

7e-06

4.5e-058e-063.1e-05

0.0118790.0107450.009740.0115140.0112930.0113920.005518

0.0118790.0107450.009740.0115140.0112930.0113920.005518

1.4e-053.4e-051.7e-05

8e-063e-06

7e-06

2e-06

1.4e-051.3e-051.4e-05

4e-06

2.5e-052e-062e-05

2.5e-052e-062e-05

1.3e-051.6e-05

1.3e-051.6e-05

0.0118270.0107450.009740.0115140.0112410.0113550.005518

0.0118270.0107450.009740.0115140.0112410.0113550.005518

0.0012310.0016880.0007110.0105040.0005590.0011580.001065

9e-061.9e-051e-05

9e-061.9e-051e-05

6e-061.1e-057e-06

6e-068e-067e-06

3e-06

3e-068e-063e-06

3e-068e-063e-06

1.2e-052.4e-059e-06

1.2e-052.4e-059e-06

1.2e-052.4e-059e-06

1.2e-052.4e-059e-06

0.001210.0016880.0007110.0105040.0005160.0011390.001065

2e-050.0001291.5e-05

1.1e-05

1.1e-05

1.3e-057.3e-051.2e-05

4e-066e-065e-06

6e-06

1e-064e-06

000

8e-06

8e-065e-067e-06

2e-05

1.5e-05

9e-06

2.1e-052e-06

2.1e-052e-06

7e-062.4e-051e-06

3e-061.7e-051e-06

4e-067e-06

1.8e-052e-051.9e-05

1.8e-052e-051.9e-05

1.8e-052e-051.9e-05

0.0011720.0016880.0007110.0105040.0003670.0011050.001065

1.3e-053.3e-058e-06

2e-061.2e-052e-06

6e-061.8e-052e-06

5e-063e-064e-06

5e-063.3e-053e-06

1e-061.2e-05

6e-06

2e-06

3e-06

4e-06

4e-066e-063e-06

0.0011540.0016880.0007110.0105040.0003010.0010940.001065

2.2e-05

2.2e-05

2e-06

2e-06

3e-06

1.1e-05

3e-061.2e-05

5e-061e-051e-066e-06

0.0011190.0016780.0007110.0105040.0001970.0010760.001065

1.4e-05

1.4e-055e-06

4e-061e-062e-06

3e-064e-062e-06

3e-06

6e-062e-068e-06

0.0005330.0001770.0013960.000497

3e-052.8e-052.5e-05

3e-052.8e-052.5e-05

3e-052.8e-052.5e-05

3e-052.8e-052.5e-05

5e-063.8e-055e-06

5e-063.8e-055e-06

5e-063.8e-055e-06

5e-063.8e-055e-06

6.2e-050.0002276.4e-05

4.3e-050.0001244.1e-05

2e-062.8e-051e-06

2e-062.8e-051e-06

1.8e-052.4e-051.8e-05

1.8e-052.4e-051.8e-05

2.1e-055.8e-052.1e-05

2.1e-055.8e-052.1e-05

2e-061.4e-051e-06

2e-061.4e-051e-06

1.9e-050.0001032.3e-05

2e-062.2e-057e-06

2e-062.2e-057e-06

3e-062.6e-052e-06

3e-062.6e-052e-06

3e-062.4e-052e-06

3e-062.4e-052e-06

1.1e-053.1e-051.2e-05

1.1e-053.1e-051.2e-05

0.0001780.000270.000173

0.0001520.0001860.000151

0.0001510.0001780.00015

3e-052.3e-052.8e-05

3.1e-052.2e-052.6e-05

1.9e-052.3e-05

1.3e-052.7e-051.4e-05

1.1e-05

1.6e-058e-061.3e-05

3.3e-05

1.7e-052.3e-051.7e-05

1.2e-051.4e-051e-05

1e-061e-06

1e-051.7e-051e-05

2e-068e-06

1e-068e-061e-06

1e-068e-061e-06

2.1e-055.9e-051.8e-05

2.1e-055.9e-051.8e-05

3.4e-05

2.1e-052.5e-051.8e-05

5e-062.5e-054e-06

5e-062.5e-054e-06

5e-062.5e-054e-06

2.2e-050.0001770.0001991.6e-05

1.3e-050.0001770.0001751.2e-05

1.3e-050.0001770.0001751.2e-05

1.3e-050.0001770.0001751.2e-05

9e-062.4e-054e-06

9e-062.4e-054e-06

2e-067e-062e-06

7e-061.7e-052e-06

9.9e-050.0002429.3e-05

2.7e-05

2.7e-05

2.7e-05

1e-053.3e-059e-06

1.8e-05

1.8e-05

1e-051.5e-059e-06

1e-051.5e-059e-06

3.5e-054.1e-052.8e-05

3.5e-054.1e-052.8e-05

2.1e-052.3e-051.4e-05

5e-066e-065e-06

9e-061.2e-059e-06

3.3e-055.7e-053.7e-05

1e-051.4e-051.6e-05

1e-051.4e-051.6e-05

2.3e-054.3e-052.1e-05

8e-061.4e-057e-06

7e-061.1e-056e-06

8e-068e-068e-06

1e-05

2.1e-05

2.1e-05

2.1e-05

9e-064.1e-059e-06

1.7e-05

1.7e-05

9e-062.4e-059e-06

9e-062.4e-059e-06

1.2e-052.2e-051e-05

1.2e-052.2e-051e-05

1.2e-052.2e-051e-05

1.9e-059.6e-051.5e-05

4e-062.3e-053e-06

4e-062.3e-053e-06

4e-062.3e-053e-06

1.5e-057.3e-051.2e-05

5e-061.9e-054e-06

5e-061.9e-054e-06

4e-063.7e-054e-06

4e-063.7e-054e-06

6e-061.7e-054e-06

6e-061.7e-054e-06

0.0001180.0002430.000106

2.6e-059.8e-051.9e-05

5e-05

2.2e-05

2.8e-05

2.6e-054.8e-051.9e-05

1.1e-053.6e-057e-06

1.5e-051.2e-051.2e-05

9.2e-050.0001458.7e-05

2.7e-05

2.7e-05

9.2e-050.0001188.7e-05

2.7e-057e-062.5e-05

1.9e-056e-061.9e-05

7e-066e-067e-06

1e-051e-056e-06

4e-062.9e-053e-06

6e-062e-059e-06

1.9e-05

2e-064e-063e-06

1.7e-051.5e-051.5e-05

2e-06

5.3e-05

5.3e-05

2.8e-05

2.8e-05

2.5e-05

2.5e-05

0.1113280.0743290.1403860.0836280.0844110.1093370.09512

0.0004390.0004550.000387

0.0004390.0004550.000387

0.0001014.5e-058.6e-05

0.0001014.5e-058.6e-05

0.0001640.0001110.000148

6.1e-053.2e-055.3e-05

2.6e-052.2e-052.6e-05

7.7e-052.9e-056.9e-05

2.8e-05

4.1e-05

4.1e-05

4.6e-054.7e-054.5e-05

4.6e-054.7e-054.5e-05

0.0001280.0002110.000108

0.0001284.9e-050.000108

7.6e-05

5.1e-05

3.5e-05

0.0066980.0007080.0564090.0118110.0067650.045388

0.000140.0022070.000170.0001180.002184

5e-064.3e-055e-06

5e-064.3e-055e-06

0.0022070.002184

0.0022070.002184

7.8e-057.7e-056.9e-05

7.8e-057.7e-056.9e-05

5.7e-055e-054.4e-05

5.7e-055e-054.4e-05

0.0065580.0007080.0542020.0116410.0066470.043123

0.0003570.000354

0.0003570.000354

0.0004160.000411

0.0004160.000411

7.3e-05

7.3e-05

9.7e-05

9.7e-05

0.0002740.0002860.0001670.000243

0.0001270.0001291.1e-058e-05

0.0001470.0001570.0001560.000163

0.0005345.6e-050.0003580.0002110.000536

0.0005345.6e-050.0003580.0002110.000536

8e-05

8e-05

0.000140.0003770.0004930.00015

0.0002080.000205

7.8e-050.0001690.0001678.7e-05

6.2e-050.0001216.3e-05

0.0001390.000138

0.0001390.000138

0.0010867.8e-050.0008140.0006590.001067

0.0008597.8e-050.0006410.0004190.000846

0.000124

0.0002270.0001730.0001160.000221

0.0001920.0001490.0001010.000192

0.0001920.0001490.0001010.000192

0.0003540.000351

0.0003540.000351

0.0002390.000237

0.0002390.000237

0.0006510.000644

0.0006510.000644

0.0005176e-050.0005140.0004810.000532

0.0005176e-050.0005140.0004810.000532

0.000840.000832

0.0003540.000351

0.0004860.000481

0.0020840.0004190.0440510.0021950.0021490.042577

0.0430390.042577

0.0006760.0002430.0004970.0008960.000705

0.0014080.0001760.0005150.0012990.001444

0.0002370.0002680.0003170.000255

0.0002370.0002680.0003170.000255

0.000720.000713

0.000720.000713

0.0005340.000528

0.0005340.000528

0.0017380.00172

0.0017380.00172

0.0002350.0001390.0002110.0002330.000156

7.5e-050.0001037.4e-057.8e-05

0.000160.0001390.0001080.0001597.8e-05

0.0012269.5e-050.0010490.0008110.001272

0.0008689.5e-050.0006870.0004650.000901

0.0003580.0003620.0003460.000371

0.0002090.000207

0.0002090.000207

3.3e-051.8e-05

3.3e-051.8e-05

8.1e-05

8.1e-05

8.1e-05

6e-062.8e-054e-06

6e-062.8e-054e-06

6e-062.8e-054e-06

6e-062.8e-054e-06

0.0371940.0434190.0329280.0267650.0307660.0357270.033363

2.5e-05

2.5e-05

2.5e-05

1.8e-052.6e-051.7e-05

1.8e-052.6e-051.7e-05

1.8e-052.6e-051.7e-05

0.0368710.0423240.0311840.0267650.0305850.0354080.032308

7e-062.1e-05

1.9e-05

1e-06

7e-061e-06

0.0313240.0258260.0227950.0137360.0236890.0300810.021336

0.0313220.0258260.0227950.0137360.0235270.0300790.021336

3.7e-05

2.3e-05

1.7e-05

8.5e-05

2e-062e-06

0.0055280.0164980.0083890.0130290.0068620.0053150.010972

7e-060.0003110.0003087e-06

0.0055180.0164980.0080780.0130290.0065510.0053060.010972

3e-063e-062e-06

1.2e-051.3e-051.2e-05

1e-06

7e-065e-067e-06

3e-06

3e-06

5e-061e-065e-06

0.0003050.0010950.0017440.000130.0003020.001055

1e-065e-062e-06

1e-065e-062e-06

0.0003040.0010950.0017440.0001250.00030.001055

5e-06

2.1e-057e-062.2e-05

0.0001950.000193

2.4e-054e-062.5e-05

3.8e-05

2.1e-054e-062e-05

8e-064e-062e-06

1e-061e-06

2.7e-055e-063e-05

1.2e-054e-061.1e-053e-06

1.8e-052e-061.7e-05

9e-06

4e-061e-06

2e-060.0009850.0009961e-052e-065.5e-05

7e-066e-061e-052.1e-05

3.3e-050.0001952e-053.2e-050.000193

3e-067e-06

2e-062e-061.5e-05

2e-060.000110.0001631e-063e-060.000301

7e-064e-067e-063.8e-05

5e-052e-055.3e-05

4.8e-050.0001951.3e-055.1e-050.000193

1.4e-053e-061.3e-05

0.001211.2e-050.0003930.0011860.001122

0.0008811.2e-050.0003930.0007470.000817

0.0001910.0001360.0001420.000181

0.0001360.0001369.1e-050.000133

5.5e-055.1e-054.8e-05

0.0002481.2e-050.0001250.0001160.00023

2.7e-05

1.1e-05

0.0001291.2e-050.0001253.7e-050.000119

0.0001194.1e-050.000111

0.0002420.0001330.000228

0.0001287.9e-050.000111

0.0001142.5e-050.000117

2.9e-05

6.2e-054.5e-055.4e-05

6.2e-054.5e-055.4e-05

7.7e-05

7.7e-05

0.0001380.0001327.3e-050.000124

0.0001380.0001327.3e-050.000124

8.7e-05

8.7e-05

2.4e-05

2.4e-05

5e-05

5e-05

0.0003290.0004390.000305

4.1e-053.4e-054e-05

4.1e-053.4e-054e-05

6.9e-055.8e-057.6e-05

6.9e-055.8e-057.6e-05

7.9e-05

7.9e-05

5e-053.2e-054.9e-05

2.7e-051.9e-052.9e-05

2.3e-051.3e-052e-05

0.0001070.0001078.8e-05

0.0001070.0001078.8e-05

1e-066.7e-052e-06

1e-05

1e-061e-062e-06

5.6e-05

6.1e-056.2e-055e-05

2.4e-05

6.1e-053.8e-055e-05

1.2e-056e-051.1e-05

1.2e-056e-051.1e-05

1.2e-056e-051.1e-05

1.2e-056e-051.1e-05

0.0003550.0002560.0003130.000303

0.0003550.0002560.0003130.000303

0.0003090.0002560.0002090.000263

0.0003090.0002560.0002090.000263

2.2e-057.4e-051.9e-05

2.2e-057.4e-051.9e-05

2.4e-053e-052.1e-05

2.4e-053e-052.1e-05

5.2e-05

2.5e-05

2.5e-05

2.5e-05

2.7e-05

2.7e-05

2.7e-05

0.0022350.0005390.0036060.0126250.0034220.0022420.000498

0.0001910.0001810.0003070.000169

4.3e-05

2.5e-05

1.8e-05

0.000165

4.1e-05

3.9e-05

1.8e-05

1.8e-05

2.3e-05

2.6e-05

3.5e-05

3.5e-05

0.0001910.0001816.4e-050.000169

1.7e-05

0.0001910.0001814.7e-050.000169

0.0020440.0005390.0034250.0126250.0031150.0020730.000498

4.3e-05

4.3e-05

0.0004647.3e-050.0008590.0126250.0004760.0004670.000246

5.4e-05

0.0002490.000246

0.0002864.6e-050.0004260.0126259.8e-050.000282

0.0001782.7e-050.0001847e-050.000185

8.7e-05

4.8e-05

0.000119

6e-061.3e-051.3e-05

6e-061.3e-051.3e-05

0.0012120.0004660.002230.0019110.0012430.000252

0.0001450.000144

0.0005660.0002010.0003597.7e-050.000588

0.0003120.000309

0.0007470.000739

0.0001530.000151

0.0001748.6e-050.0001420.0001020.000175

0.000129

0.0001460.000144

0.0004720.0001790.0002260.0001160.000480.000252

3.7e-05

3.7e-05

5.1e-05

5.1e-05

0.000180.0001840.000340.000184

6.3e-05

0.000180.0001845.6e-050.000184

2.3e-05

4.8e-05

0.000102

4.8e-05

0.0001820.0001520.0002440.000166

0.0001520.00015

9e-052.8e-058.5e-05

2.6e-05

9.2e-054e-058.1e-05

4.7e-050.0001594.4e-05

4.7e-050.0001594.4e-05

3.6e-058.9e-053.3e-05

9e-061.4e-059e-06

2.7e-057.5e-052.4e-05

1.1e-057e-051.1e-05

9e-062.8e-059e-06

2e-064.2e-052e-06

0.0631320.0296510.0467940.0442380.0361590.0627320.015871

0.000357e-060.0002420.0002660.0003340.00024

0.000357e-060.0002420.0002660.0003340.00024

1.2e-05

3.9e-057e-060.0002428e-063.8e-050.00024

2.3e-05

8.9e-055.4e-058.7e-05

6.5e-05

9.1e-052.7e-058.4e-05

6.4e-051.6e-056.5e-05

6.7e-056.1e-056e-05

0.0005790.0003290.0002790.000515

0.0001570.0001537.4e-050.000145

0.0001570.0001537.4e-050.000145

0.0002820.0001760.0001050.000242

0.0002820.0001760.0001050.000242

0.000140.00010.000128

0.000140.00010.000128

0.0054060.0028380.0037820.0107060.0021150.0052170.00097

9.9e-05

9.9e-05

8.9e-05

8.9e-05

5.9e-05

5.9e-05

0.0006130.0007690.0002610.0005840.000733

8.6e-05

0.0002240.0002335.2e-050.0002150.000256

0.0002410.000238

0.0001350.0001271.2e-050.000117

0.0001077.6e-050.000111

0.0001470.0001683.5e-050.0001410.000239

2.5e-055.7e-052.9e-05

1.2e-055e-06

1.8e-051e-051.7e-05

2e-069e-062e-06

4e-06

5e-062.2e-055e-06

0.0043840.0027660.0028360.0107060.0012830.0042620.000237

0.0001390.000138

0.0001572.5e-050.0001552.6e-050.00015

2e-06

0

1.4e-05

1.4e-05

0.000134

0.0037870.0027190.0021310.0107060.0007290.003643

0.0001972.2e-050.0002249.8e-050.0002370.000237

0.0002430.0001870.0001280.000232

0.0003847.2e-050.0001770.0002670.000342

0.0001035.6e-059.4e-05

3.8e-052.3e-051.8e-053.4e-05

7.3e-05

0.0002434.9e-050.0001770.000120.000214

1.4e-050.0001198e-06

1.4e-050.0001198e-06

3e-061.8e-052e-06

1.3e-05

1.9e-05

2e-069e-061e-06

1e-06

2.3e-05

5e-062e-061e-06

7e-06

4e-062.7e-054e-06

0.0004890.0002460.000484

0.0004890.000484

0.0004890.000484

0.000127

0.000127

0.000119

6.5e-05

5.4e-05

0.0001610.0001490.0002910.000139

0.0001490.000148

0.0001490.000148

0.0001165.6e-050.00011

0.0001165.6e-050.00011

4.5e-058.7e-052.9e-05

4.5e-058.7e-052.9e-05

5.2e-05

5.2e-05

5.2e-05

8.1e-056.3e-056.3e-05

8.1e-056.3e-056.3e-05

8.1e-056.3e-056.3e-05

0.0003940.0001570.0006570.0003550.000118

0.0001870.0001270.00016

6.3e-054e-055.6e-05

5.3e-055.6e-054.4e-05

7.1e-053.1e-056e-05

8.9e-056e-058.6e-05

8.9e-056e-058.6e-05

2.9e-05

2.9e-05

7e-05

7e-05

0.0001188.5e-050.0001090.000118

0.0001188.5e-050.0001090.000118

0.0001570.000258

0.0001570.000156

0.000102

2.8e-05

2.8e-05

0.0001510.0001380.0001450.000127

0.000150.0001380.0001410.000125

0.000150.0001385.6e-050.000125

8.5e-05

1e-064e-062e-06

2e-06

0

0

001e-06

1e-06

0

1e-06

0

1e-0601e-06

0.0492570.022330.0343920.0117160.0269130.0494630.006801

0.0006780.000671

0.0006780.000671

0.0002060.0002010.0002360.000167

0.0002060.0002010.0002360.000167

0.0002070.000205

0.0002070.000205

0.0490510.022330.0333060.0117160.0258010.0492960.006801

0.0081590.0030670.0042360.0030350.0083210.003391

0.0235830.0131010.014640.0117160.0101970.023217

0.0089380.0031380.0031060.0021630.0091720.001599

0.0079980.007912

0.0083710.0030240.0033260.0024940.0085860.001811

0.0066420.0044760.0071160.0218160.0049480.0064340.007258

0.0045510.0027090.0048360.0106050.0030010.0044730.000984

0.0002255.8e-050.0002393.5e-050.0002380.000248

0.000213.4e-050.0002833.8e-050.0002180.000736

0.0001793.5e-050.0001878.5e-050.000192

0.0001833.8e-050.0001914.5e-050.000195

6.5e-05

0.0004330.000428

7.7e-05

0.0001913.7e-050.000196.2e-050.000186

0.0035630.0025070.0033130.0106050.0021660.003444

0.000253

7.4e-05

5.8e-05

0.000121

0.0017460.0017670.002280.0112110.0014840.0016680.006274

0.0017460.0017670.002280.0112110.0014840.0016680.006274

5.8e-052.4e-054.6e-05

5.8e-052.4e-054.6e-05

0.0002870.0001860.000247

0.0001188.2e-050.000103

0.0001690.0001040.000144

9.7e-056.5e-057.7e-05

9.7e-056.5e-057.7e-05

9.7e-056.5e-057.7e-05

4.6e-055.2e-054.4e-05

4.6e-055.2e-054.4e-05

4.6e-055.2e-054.4e-05

4.6e-055.2e-054.4e-05

2.2e-052.1e-051.8e-05

1.6e-051.4e-051.8e-05

8e-061.7e-058e-06

0.2068340.2722940.189940.2412890.1577310.2018080.248285

0.0779520.0431150.0436610.0188870.0662210.0752370.000747

2e-05

2e-05

2e-05

0.0779520.0431150.0436610.0188870.0662210.0752370.000727

0.0779330.0431150.0436610.0188870.0660750.0752170.000709

8e-065e-067e-06

7e-061e-057e-06

2.5e-05

0.0778460.0431150.0430150.0188870.0657710.075133

7e-06

9e-065e-069e-06

8e-065e-066e-06

1e-050.0001451.2e-059e-060.000143

9e-067.9e-059e-06

1.3e-05

2e-062e-062e-06

5e-06

1.7e-05

3.5e-05

3.5e-05

0.0005011.5e-050.000496

3e-051.8e-05

6e-061e-067e-06

6e-06

1e-058e-061.1e-05

3.1e-05

1.8e-051e-051.7e-05

3.7e-05

3.7e-05

1.4e-057e-051.6e-05

2.3e-05

6e-061.9e-051e-05

8e-062.8e-056e-06

5e-063.9e-054e-061.8e-05

5e-061.7e-054e-06

2.2e-05

1.8e-05

0.0002170.0012730.0004240.0106050.0002320.0002650.001022

1.9e-059.8e-052.3e-05

3e-061.9e-053e-06

3e-061.1e-053e-06

8e-06

1e-054e-06

9e-06

1e-064e-06

1e-052.4e-051.4e-05

1e-052.4e-051.4e-05

3e-06

3e-06

6e-064.2e-052e-06

3e-061.9e-05

3e-062.3e-052e-06

4.5e-05

2.2e-05

2.2e-05

2.3e-05

2.3e-05

0.0001980.0012730.0004240.0106058.9e-050.0002420.001022

1e-05

1e-05

0.0001980.0012730.0004240.0106057.9e-050.0002420.001022

1.8e-052e-061.7e-05

2e-06

0.0001790.0012730.0004240.0106057.1e-050.0002230.001022

2e-06

1e-0602e-06

2e-06

2.4e-05

2.4e-05

2.4e-05

2.4e-05

0.0213660.0150570.0116770.0129280.0025450.0207280.014037

0.0213660.0150570.0116770.0129280.0025450.0207280.014037

0.021180.0150570.0115450.0129280.0024430.0205960.014037

0.0170820.0141630.0061280.0129280.0016710.0166440.008019

0.0017410.000320.0004780.0001930.0016490.000924

8.3e-05

0.0023570.0005740.0004220.0001190.0023030.001002

0.0003810.000377

0.0041360.004092

3.3e-057.3e-051.8e-05

3.3e-057.3e-051.8e-05

0.0001530.0001322.9e-050.000114

0.0001530.0001322.9e-050.000114

1.1e-052e-051.4e-05

1.1e-052e-051.4e-05

1.1e-052e-051.4e-05

1.1e-052e-051.4e-05

4.5e-05

4.5e-05

4.5e-05

4.5e-05

0.0004210.013010.013260.000417

0.0001740.0001590.0004750.000155

4e-062e-067e-06

4e-062e-067e-06

6.9e-054.1e-056.7e-05

6.9e-054.1e-056.7e-05

8.2e-050.0001590.0003197.8e-05

8.2e-056.1e-057.8e-05

2.8e-05

0.0001590.000158

3.5e-05

3.7e-05

5e-05

5e-05

1.9e-052.7e-053e-06

1.9e-052.7e-053e-06

3.6e-05

3.6e-05

1.3e-052.1e-051.3e-05

1.3e-052.1e-051.3e-05

1.3e-052.1e-051.3e-05

1.6e-053.7e-053.4e-05

1.6e-053.7e-053.4e-05

1.6e-051.3e-053.4e-05

1.1e-05

1.3e-05

0.0001940.0128510.012590.000193

0.0001940.0128510.012590.000193

0.0126940.012558

3.7e-051e-064e-05

0.0001570.0001573.1e-050.000153

3e-05

3e-05

3e-05

2.4e-050.0001072.2e-05

2.6e-05

2.6e-05

1.5e-055.2e-051.5e-05

5e-069e-065e-06

2e-062.4e-053e-06

8e-061.9e-057e-06

2e-05

2e-05

9e-069e-067e-06

9e-069e-067e-06

0.017520.0137770.0322420.067670.0070120.0214360.028728

0.0002130.0002420.000150.000180.000239

3e-05

3e-05

4.2e-055.5e-053.8e-05

1.4e-05

9e-06

2.3e-059e-062.6e-05

1.9e-051.3e-051.2e-05

1e-05

4.9e-053.5e-054.2e-05

1.8e-051.2e-051.7e-05

1.2e-05

3.1e-051.1e-052.5e-05

1.2e-051e-061e-05

1.2e-051e-061e-05

2.1e-050.0002421.3e-051.8e-050.000239

2.1e-051.3e-051.8e-05

0.0002420.000239

8.9e-051.6e-057.2e-05

8.9e-051.6e-057.2e-05

0.0051760.0078410.002810.0114130.0015610.0051290.008462

5.9e-05

5.9e-05

0.000421e-050.0001628e-050.00037

4e-06

0.000111.4e-050.000104

0.0001750.0001626e-060.000148

1e-06

3e-051e-054e-063e-05

8e-06

3e-05

0.0001057e-068.8e-05

6e-06

0.0046610.0078110.0026480.0114130.0014150.0046740.008462

0.0002270.000224

0.0046440.0077760.0018410.0114130.0003950.0046560.008462

0.000133

2e-063.5e-0502e-06

5e-05

0.0002150.000213

1.5e-053e-061.6e-05

0.0003650.000361

3.5e-05

1e-06

0

9.5e-052e-057e-068.5e-05

9.1e-052e-052e-068.2e-05

4e-065e-063e-06

0.000170.0001340.0001090.000151

9e-06

9e-06

1.3e-05

1.3e-05

0.000170.0001348.7e-050.000151

0.000140.0001345.4e-050.000125

1.5e-051.3e-051.3e-05

1.5e-054e-061.3e-05

3e-06

1.3e-05

0.0006710.0008360.0005520.0003090.0006670.001238

0.0004090.0008360.0005520.0001810.0003950.001238

0.0002350.0008360.0003813e-050.000230.001238

5.3e-05

0.0001740.0001719.8e-050.000165

0.0001957.4e-050.000223

9e-061e-05

4.2e-059e-063.9e-05

7.8e-054.8e-050.00011

1e-05

6.6e-057e-066.4e-05

6.7e-055.4e-054.9e-05

6.7e-057e-064.9e-05

1e-05

3.7e-05

0.0104110.0049830.0281470.0562570.0041990.0145170.018705

0.0032860.0007910.0095480.0020580.0032330.007238

0.0010310.00102

9.6e-05

0.000730.000722

0.0023320.0006280.0003820.000140.0023120.000276

0.0009540.0001630.0003678e-050.000921

0.004220.004175

0.0028180.002787

1e-058e-065e-06

1e-058e-065e-06

0.0003525.2e-050.0003519e-050.0003422.4e-05

0.0002090.0002061.3e-050.0001982.4e-05

2.4e-05

0.0001435.2e-050.0001452e-060.000144

9e-06

1.8e-05

0

2.4e-05

4e-067.4e-054e-06

2e-06

4e-069e-064e-06

3.7e-05

8e-06

3e-06

1.5e-05

0.000109

0.000109

8e-067e-06

8e-067e-06

2e-05

2e-05

6.9e-05

6.9e-05

0.0004380.0010340.0018270.0112116.2e-050.0004290.004642

6.1e-05

0.0001190.0009960.0002720.0112119e-060.0001140.003348

8e-06

0.0003193.8e-050.000323.3e-050.0003152e-06

0.0012352e-050.001221

2e-06

8e-05

6.3e-05

1.7e-05

2.9e-05

2.9e-05

0.0004310.0009150.000930.0112110.0001060.0047470.004593

0.0001680.0008520.000660.0112118.9e-050.0044740.004593

0.0002636.3e-050.000271.7e-050.000273

5.4e-05

5.4e-05

0.0003716.1e-050.0003723.4e-050.000366

0.0003716.1e-050.0003723.4e-050.000366

1.1e-053.7e-051.1e-05

1.1e-053.7e-051.1e-05

6.1e-05

6.1e-05

0.0008359.4e-050.0004970.0001880.000817

6e-061.4e-058e-06

0.00069.4e-050.0002770.0001320.000591

8e-061.4e-055e-06

0.0002210.000222.8e-050.000213

3.3e-05

3.3e-05

0.0002870.0002550.0002050.000285

0.0002870.0002550.0002050.000285

0.0002030.0002270.0105580.0103023.6e-050.0001680.000243

7.6e-051.7e-053.8e-055e-05

5.2e-050.000160.0001441e-055.1e-050.000128

2.8e-055e-062.9e-055.1e-05

4.7e-056.7e-050.0104140.0103024e-065e-051.4e-05

0.0036790.0015650.0030830.0104030.0006810.0036550.001747

7e-06

1e-06

4e-06

4.4e-05

0.0005569.3e-050.0005691.1e-050.000569

6e-06

0.0018430.001170.0012250.0104030.0004830.001785

1e-06

7e-06

3e-06

2e-06

0.0003310.0001480.0002981e-050.000340.001189

5e-06

0.0004717.1e-050.0004887.7e-050.000495

0.0004788.3e-050.0005032e-050.0004660.000558

3.1e-05

3.1e-05

2.6e-05

2.6e-05

2.5e-05

2.5e-05

0.0004960.0002440.0007260.013136.2e-050.0004480.000218

0.0001946.3e-050.0001951.3e-050.000193

0.0002328.2e-050.0002058e-060.00018

4.3e-059.9e-050.0003260.013132e-065e-050.000163

2.1e-05

3e-06

3e-06

2.7e-051e-062.5e-053.4e-05

3.2e-05

2.1e-05

2.1e-05

4.2e-05

4.2e-05

4.2e-05

3.6e-05

3.6e-05

3.6e-05

0.0008790.0001170.0003570.0006060.0007928.4e-05

0.0003060.0001360.000150.0002778.4e-05

9.8e-052.6e-058.4e-058.4e-05

4.6e-05

3.7e-0503.4e-05

0.0001370.0001366.8e-050.000132

1e-056e-067e-06

2.4e-054e-062e-05

4e-061e-056e-06

4e-061e-056e-06

0.0005630.0001170.0002210.0004320.000505

3.8e-05

2.6e-05

3.2e-051e-051.2e-052.7e-05

8e-06

4.8e-05

0.0003810.0001070.0002210.0002850.000357

0.0001244e-060.000101

2.6e-051.1e-052e-05

6e-061.4e-054e-06

6e-061.4e-054e-06

6.1e-058.5e-054.8e-05

6.1e-058.5e-054.8e-05

4.8e-053e-053.3e-05

4.8e-053e-053.3e-05

8e-064e-057e-06

2e-05

8e-061.4e-057e-06

6e-06

5e-061.5e-058e-06

5e-061.5e-058e-06

0.0125850.004390.0099640.0113120.0148360.0122690.002506

3.7e-05

3.7e-05

3.7e-05

0.0001990.0001780.000171

3.8e-052.9e-054.2e-05

3.8e-052.9e-054.2e-05

6.6e-053.3e-055.3e-05

6.6e-053.3e-055.3e-05

8.2e-053.1e-056.5e-05

8.2e-053.1e-056.5e-05

3.1e-05

3.1e-05

1.3e-055.4e-051.1e-05

8e-061.3e-058e-06

1e-062.6e-05

4e-061.5e-053e-06

4.2e-05

4.2e-05

4.2e-05

0.0123720.004390.0099640.0113120.0145250.0120920.002506

9.4e-05

9.4e-05

6.5e-057e-056.4e-05

6.5e-057e-056.4e-05

9.6e-050.0001158.6e-05

9.6e-050.0001158.6e-05

0.0001070.0001140.0001

4.9e-058e-054.9e-05

5.8e-053.4e-055.1e-05

4.9e-05

4.9e-05

0.0121040.004390.0099640.0113120.0140830.0118420.002506

0.0004260.000120.0002358.8e-050.000449

0.000210.0001420.000105

0.0114680.004270.0061060.0113120.0103390.0113930.002506

0.0034810.003444

0.000107

1.4e-055.4e-056e-06

1.4e-055.4e-056e-06

3.4e-05

1.4e-052e-056e-06

0.030360.1546830.0207810.0486820.0055780.0269950.17122

0.0303590.1546830.0207810.0486820.0055540.0269940.17122

0.0303590.1546830.0207810.0486820.0055060.0269940.17122

0.0303510.1546830.0207810.0486820.0052910.0269880.17122

8e-066.2e-056e-06

3.8e-05

8.3e-05

3.2e-05

4.8e-05

4.8e-05

1e-062.4e-051e-06

1e-061.8e-051e-06

1e-063e-061e-06

1.5e-05

6e-06

6e-06

0.0001133e-050.000230.0001289.3e-050.000247

0.0001133e-050.000230.0001289.3e-050.000247

3e-063e-063e-06

3e-063e-063e-06

3.1e-053.1e-052.6e-05

2e-06

3e-062e-063e-06

2.2e-052.5e-051.7e-05

6e-062e-066e-06

3e-063e-063e-06

3e-063e-063e-06

4.2e-053e-050.000233.1e-053.8e-050.000247

1.4e-052.1e-051.6e-05

2e-05

7e-061e-068e-06

1.1e-057e-066e-06

1e-053e-050.000232e-068e-060.000227

2e-051e-06

2e-051e-06

1e-067e-060

4e-06

1e-062e-060

1e-06

4e-061.6e-052e-06

4e-061.6e-052e-06

2.3e-058e-061.8e-05

8e-064e-063e-06

1.5e-054e-061.5e-05

6e-069e-062e-06

6e-069e-062e-06

0.006530.0048850.005440.0213110.0052190.0062660.003436

4e-067e-063e-06

4e-067e-063e-06

4e-067e-063e-06

0.0001520.0001240.000145

0.0001520.0001240.000145

1e-061e-061e-06

1.8e-051.4e-051.6e-05

3e-063e-064e-06

3e-067e-063e-06

7e-065e-06

4.8e-053.8e-055.5e-05

2e-062e-062e-06

8e-064e-064e-06

8e-067e-068e-06

3e-061.3e-054e-06

6e-064e-066e-06

3e-061e-063e-06

1.1e-055e-061e-05

1e-061e-06

2.1e-059e-061.5e-05

7e-065e-066e-06

2e-061.1e-052e-06

0.0062970.0048850.005440.0213110.0049270.0060540.003436

0.0005910.0005280.0005790.0104030.0004510.0005750.000495

1e-05

8e-06

2.6e-05

0.0005360.0005280.0005790.0104030.0003750.0005170.000495

2.9e-054e-062.8e-05

2.6e-051.2e-053e-05

8e-06

8e-06

0.0056970.0043570.0048610.0109080.0044060.005470.002941

0.005690.0043570.0048610.0109080.0043910.0054650.002941

7e-061.5e-055e-06

9e-06

9e-06

5e-061.2e-056e-06

1e-06

3e-062e-062e-06

5e-06

02e-060

0

2e-062e-064e-06

4e-062.1e-053e-06

4e-062.1e-053e-06

2.8e-05

2.8e-05

6e-062.3e-055e-06

6e-062.3e-055e-06

1.2e-05

6e-061.1e-055e-06

2e-063.5e-055e-06

2e-063.5e-055e-06

1e-062.3e-051e-06

1e-061.2e-054e-06

2e-056.2e-051.7e-05

2e-056.2e-051.7e-05

8e-06

5e-06

1e-06

3e-06

6e-061.2e-054e-06

1.2e-05

7e-06

1e-0509e-06

4e-062e-064e-06

1.2e-05

2.2e-05

2.2e-05

2.2e-05

4.9e-051.9e-053.7e-05

4.9e-051.9e-053.7e-05

4.9e-051.9e-053.7e-05

0.0001130.0051530.0053790.000102

0.0001130.0051530.0053790.000102

6.9e-050.0001335.5e-05

4.7e-05

1.5e-05

2.2e-053e-051.3e-05

2.1e-052.7e-051.7e-05

2.6e-051.4e-052.5e-05

0.0051530.005137

0.0051530.005097

4e-05

3.2e-05

3.2e-05

4.4e-057e-064.7e-05

4.4e-057e-064.7e-05

2.4e-05

2.4e-05

4.6e-05

4.6e-05

0.0387820.0339050.0462870.0394910.0363620.0371860.025094

0.0378050.0335890.0454680.0288860.0358470.0363190.024604

0.0002599.3e-050.0187110.014140.0022660.0002690.002226

0.0002040.000202

4.2e-051.2e-054.8e-05

0.0001424.8e-050.0001490.0001510.000149

0.000250.000247

0.0020.001979

2.8e-054.5e-050.0142930.014145e-062.3e-05

0.0002490.000247

4.7e-052.3e-054.9e-05

0.0003770.000373

0.0003880.000384

7.7e-05

0.0008010.000792

0.0375380.0334960.0267570.0147460.0334890.0360440.022378

5e-06

1.4e-05

3e-06

8e-069e-068e-06

1.3e-05

9e-066e-066e-06

0.037520.0334960.0267570.0147460.0334350.0360290.022378

1e-064e-061e-06

8e-069.2e-056e-06

2e-05

6e-05

8e-061.2e-056e-06

0.0009770.0003160.0008190.0106050.0005150.0008670.00049

5e-06

5e-06

4.1e-056.7e-054.7e-053.8e-05

2e-05

4.1e-056.7e-052.7e-053.8e-05

0.0009360.0002490.0008190.0106050.0004630.0008290.00049

1.8e-05

1e-06

7e-06

5e-06

3.3e-0503.3e-05

4.3e-051.9e-055e-064e-05

3.8e-056e-063.2e-05

7e-06

8e-06

4e-06

1e-05

9e-06

1.4e-05

4e-06

1e-06

2.4e-055e-062.2e-05

6e-06

6e-053.8e-057e-066e-05

7e-06

9e-06

5.8e-051.5e-056e-05

1.9e-05

8.9e-052.4e-057.8e-05

7e-06

2e-06

6e-06

2.6e-053e-062.4e-05

7e-06

9.5e-050.0001630.0003240.0106051.8e-058.6e-05

1.1e-05

2.6e-05

8.1e-051.8e-051.5e-057.7e-05

4.7e-058e-065e-05

3e-06

4e-06

1.4e-05

2.9e-052e-062.6e-05

1.3e-05

5.7e-05

1e-05

3e-06

6e-06

5e-065e-066e-06

1.4e-05

6e-06

3e-06

2e-05

4.9e-058e-064.9e-05

9e-06

8e-06

4.2e-056e-068e-064.1e-05

6.2e-051.5e-055.9e-05

3e-06

9.2e-051.4e-057.9e-05

6e-065e-060.0004951e-067e-060.00049

0.0007390.0011790.0005620.0104030.0005910.0006940.000745

0.0006450.0011790.0005620.0104030.0004640.0006130.000745

2.7e-054.5e-052.7e-05

1.6e-05

2.7e-056e-062.7e-05

2.3e-05

0.0004290.0011790.0005620.0104030.0002670.0004110.000745

0.0004290.0011790.0005620.0104030.0002670.0004110.000745

2.9e-05

1.3e-05

9e-06

7e-06

0.0001213.5e-050.00011

5.6e-051.5e-055.1e-05

6.5e-052e-055.9e-05

6.8e-058.8e-056.5e-05

9e-06

6e-062e-069e-06

2e-06

1e-051e-067e-06

6e-061e-066e-06

5e-0605e-06

0

1e-060

2.7e-057e-062.8e-05

1.3e-053e-061e-05

6.3e-05

9.4e-050.0001278.1e-05

4.1e-051.8e-052.7e-05

4.1e-051.8e-052.7e-05

5.3e-05

1.7e-05

1.5e-05

2.1e-05

2.3e-05

2.3e-05

5.3e-053.3e-055.4e-05

5.3e-053.3e-055.4e-05

6.4e-050.0005090.0001615.8e-050.000503

4e-05

1e-05

1e-05

3e-05

3e-05

6.4e-057.5e-055.8e-05

1e-051.1e-051.1e-05

1e-051.1e-051.1e-05

2.2e-051.9e-051.9e-05

2.2e-051.9e-051.9e-05

1.1e-052.5e-051e-05

1.1e-052.5e-051e-05

2.1e-052e-051.8e-05

2.1e-052e-051.8e-05

4.6e-05

4.6e-05

2.5e-05

2.1e-05

0.0005090.000503

0.0005090.000503

0.0005090.000503

3.3e-05

3.3e-05

1.7e-05

1.7e-05

1.6e-05

1.6e-05

2.3e-050.0002343.1e-05

2.3e-050.0002343.1e-05

1.2e-055.8e-051.3e-05

1.2e-055.8e-051.3e-05

1e-053.2e-051.1e-05

1e-053.2e-051.1e-05

2e-062.6e-052e-06

2e-062.6e-052e-06

1.1e-050.0001761.8e-05

9e-065.4e-051.6e-05

4e-062.9e-053e-06

1.4e-05

4e-061.5e-053e-06

5e-062.5e-051.3e-05

5e-062.5e-051.3e-05

2e-069.7e-052e-06

5.7e-05

3.4e-05

2.3e-05

2.4e-05

2.4e-05

1.2e-05

1.2e-05

2e-064e-062e-06

2e-064e-062e-06

2.5e-05

2.5e-05

2.5e-05

3.6e-050.0002012.9e-05

2.5e-05

2.5e-05

2.5e-05

2.5e-05

2.5e-05

5e-062.1e-053e-06

5e-062.1e-053e-06

5e-062.1e-053e-06

5e-062.1e-053e-06

5e-067e-063e-06

1.4e-05

1.5e-05

1.5e-05

1.5e-05

1.5e-05

1.5e-05

1.4e-059.8e-051.4e-05

4e-063.8e-056e-06

4e-063.8e-056e-06

4e-063.8e-056e-06

4e-063.8e-056e-06

1e-056e-058e-06

1e-056e-058e-06

2e-05

2e-05

1e-052.2e-058e-06

1e-052.2e-058e-06

1.8e-05

1.8e-05

1.7e-054.2e-051.2e-05

1.7e-054.2e-051.2e-05

1.7e-054.2e-051.2e-05

1.7e-054.2e-051.2e-05

1.7e-054.2e-051.2e-05

1e-063.9e-05

1e-063.9e-05

1e-063.9e-05

1e-063.9e-05

1e-063.9e-05

1e-063.9e-05

4.3e-050.0002643.8e-05

4.3e-050.0002643.8e-05

4.3e-050.0002643.8e-05

4.3e-050.0002643.8e-05

0.000121

0.000121

3.5e-054.4e-053.3e-05

3.5e-054.4e-053.3e-05

1e-064.7e-05

1e-064.7e-05

7e-063.3e-055e-06

7e-063.3e-055e-06

1.9e-05

1.9e-05
