# Supplementary material for: MetaMeta: integrating metagenome analysis tools to improve taxonomic profiling
Source: Microbiome. 2017 Aug 14;5:101. doi: 10.1186/s40168-017-0318-y (PMC5557516; doi:10.1186/s40168-017-0318-y)
Supplement: Supplementary file 2 — Additional File with interactive charts for all CAMI toy set results on default, very-precise and very-sensitive mode. File prefix S, M, and H for low, medium and high complexity, respectively. (TAR 3573 kb) [file 40168_2017_318_MOESM2_ESM.tar › M2_S001__insert_180_default.html]

Javascript must be enabled to view this page.

magnitude
magnitudeUnassigned

clark.parsed\_profile
dudes.parsed\_profile
final.metametamerge.profile
gottcha.parsed\_profile
kaiju.parsed\_profile
kraken.parsed\_profile
motus.parsed\_profile

0.9999919999999990.9999960.9999990000000010.9999991.0000060.9999939999999990.999985999999998

0.9959389999999990.9982750.9977660000000010.9899190.9936619999999990.9959999999999990.999398999999998

0.049650.0323620.0259280.0214720.0371990.0485610.016479

2.3e-058.8e-051.5e-05

2.3e-058.8e-051.5e-05

2.3e-058.8e-051.5e-05

2.3e-058.8e-051.5e-05

2.3e-058.8e-051.5e-05

0.0496150.0323620.0259280.0214720.0370690.0485370.016479

0.0496150.0323620.0259280.0214720.0370690.0485370.016479

0.0496150.0323620.0259280.0214720.0370690.0485370.016479

4.1e-050.0001323.5e-05

2.4e-056.4e-051.4e-05

1.7e-056.8e-052.1e-05

0.0495410.0323620.0259280.0214720.0368360.0484720.016479

0.0495080.0323620.0259280.0214720.0367540.0484450.016479

3.3e-058.2e-052.7e-05

2.2e-055.7e-052e-05

2.2e-055.7e-052e-05

1.1e-054.4e-051e-05

1.1e-054.4e-051e-05

1.2e-054.2e-059e-06

1.2e-054.2e-059e-06

1.2e-054.2e-059e-06

1.2e-054.2e-059e-06

1.2e-054.2e-059e-06

6.1e-05

6.1e-05

6.1e-05

6.1e-05

6.1e-05

6.1e-05

0.0001020.000460.00011

0.0001020.000460.00011

0.0001020.000460.00011

5.1e-050.0003894.9e-05

3e-067e-053e-06

3e-067e-053e-06

2.2e-050.0001431.7e-05

5.3e-05

4.3e-05

2.2e-054.7e-051.7e-05

2.6e-057.2e-052.9e-05

2.6e-057.2e-052.9e-05

0.000104

0.000104

5.1e-057.1e-056.1e-05

2.1e-052.4e-052.2e-05

6e-06

2.1e-051.8e-052.2e-05

3e-054.7e-053.9e-05

3e-054.7e-053.9e-05

0.0002630.0003080.000194

0.0002630.0003080.000194

0.0002630.0003080.000194

0.0002630.0003080.000194

3e-064.3e-052e-06

3e-064.3e-052e-06

4.8e-050.0001014.6e-05

1e-052.7e-056e-06

1.1e-052.5e-059e-06

6e-062.9e-055e-06

2.1e-052e-052.6e-05

0.0001476.4e-058.8e-05

8e-06

9.5e-052.6e-055.2e-05

5.2e-053e-053.6e-05

4.3e-054.4e-053.8e-05

1.7e-052e-051.6e-05

2.6e-052.4e-052.2e-05

2.2e-055.6e-052e-05

2.2e-052.7e-052e-05

2.9e-05

4.8e-050.0002623.9e-05

4.8e-050.0002623.9e-05

4.8e-050.0002623.9e-05

4.8e-050.0002623.9e-05

1.2e-055.8e-051e-05

1.2e-055.8e-051e-05

9e-060.0001565e-06

9e-065e-055e-06

4.7e-05

5.9e-05

2.7e-054.8e-052.4e-05

6e-061.2e-054e-06

2.1e-053.2e-052e-05

4e-06

0.3322990.1951410.2303930.1245970.2693240.3311090.115972

0.3261230.1944650.2295120.1245970.2607590.3250690.115642

0.0008390.0008840.0008622.6e-05

0.0008390.0008840.0008622.6e-05

0.0008390.0008840.0008622.6e-05

0.0001880.0001850.0001812.6e-05

0.0001120.0001260.000113

0.000150.0001730.000152

0.0001840.0001920.000182

0.0002050.0002080.000234

0.0002840.0002320.0002310.000272

0.0002840.0002320.0002310.000272

0.0002840.0002320.0002310.000272

0.0002840.0002320.0002310.000272

0.0001720.0001190.000169

0.0001720.0001190.000169

0.0001720.0001190.000169

0.0001720.0001190.000169

0.0340780.015640.0466120.0021170.0321990.0377980.020696

0.0340780.015640.0466120.0021170.0321990.0377980.020696

0.0004613.1e-050.0002620.0001850.0003854.2e-05

0.0004613.1e-050.0002620.0001850.0003854.2e-05

0.0336170.0156090.046350.0021170.0320140.0374130.020654

7.7e-050.0001162.6e-058.4e-05

4e-06

0.0004248.3e-050.0001190.0004712.6e-05

0.0004030.000452

0.0104460.011723

0.0005050.000567

0.000230.000258

6.2e-05

9.3e-05

0.000137

2.1e-05

0.0004010.00045

0.0011080.0002090.000380.000350.0011965.4e-05

0.0054370.0028920.0038640.00617

0.000177

0.0024130.0036370.002156

0.0007530.000845

0.0038430.004312

0.0004440.000498

0.0018370.002061

0.001360.001526

0.0002190.000246

0.000113

0.0008360.000120.0001030.000953

1.9e-05

0.0002290.000257

6.6e-05

0.0019610.002201

0.0005333.8e-050.000598

0.000120.0001275e-050.000136

0.0013140.0001890.0002540.00010.0014230.000138

0.0002110.000114

5.8e-05

0.0037780.0011270.000930.0008610.0043280.000445

0.000134

0.0004890.000549

4e-06

0.0007670.000861

8.9e-05

4.2e-05

0.0009390.0001410.0002530.0002010.001046

0.0009640.0001870.0002970.0002150.001062

0.0065210.007318

0.0002350.000264

0.0003640.000408

6e-06

0.0035730.0011950.0009140.0003940.004039

0.0038070.0011610.0006590.0007411e-060.000341

0.000107

0.0057050.0030190.0003560.0001520.0063010.000191

0.0007550.000847

0.000225

0.0002860.000321

4e-05

0.0004290.000481

0.0002110.0002362.3e-05

0.0003750.000421

0.0019290.0037110.0006780.0002210.0064090.000903

0.0014850.0002610.0005239.6e-050.001633

0.0002860.000321

0.0021210.0009640.0009470.0021170.0009950.0021610.000456

0.0014750.0007320.001640.0014394.3e-05

0.0007220.0007320.0011110.0006984.3e-05

0.0003930.0002830.000470.0003534.3e-05

0.0003930.0002830.0002460.0003534.3e-05

0.000224

0.0002150.000242

0.0002150.000242

0.0003290.0002340.0001820.000345

0.0003290.0002340.0001820.000345

0.000217

0.000217

0.0007530.0005290.000741

0.000168.9e-050.000143

0.000168.9e-050.000143

0.0001564.1e-050.00015

0.0001473.8e-050.000142

9e-063e-068e-06

0.000210.0001010.000227

2.5e-05

0.000217.6e-050.000227

0.00010.0001870.000111

0.00010.0001870.000111

0.0001270.0001110.00011

0.0001270.0001110.00011

0.0004850.0002780.0004280.000113

0.0004850.0002780.0004280.000113

0.0004850.0002780.0004280.000113

0.0004850.0002780.0004280.000113

0.0009563.7e-050.0007710.0007450.0009356e-06

0.0009563.7e-050.0007710.0007450.0009356e-06

0.0003080.0002460.0002270.000313

0.0003080.0002460.0002270.000313

0.0003180.0002490.0002260.000314

0.0003180.0002490.0002260.000314

0.000333.7e-050.0002760.0002920.0003086e-06

0.000333.7e-050.0002760.0002920.0003086e-06

0.0128340.0078230.0067050.0059480.0106910.0124920.004166

0.0128340.0078230.0067050.0059480.0106910.0124920.004166

0.0001259e-056.7e-05

0.0001259e-056.7e-05

0.0005841.4e-05

0.000149

0.000121

1.4e-05

0.000166

0.000148

5.1e-05

5.1e-05

0.0127090.0078230.0067050.0059480.0098650.0124250.004152

0.0127090.0078230.0067050.0059480.0098650.0124250.004152

0.000101

0.000101

0.0192280.0087210.0291280.0141130.0262460.0194310.011165

0.0001270.0001190.000119

0.0001270.0001190.000119

0.0001270.0001190.000119

0.0002370.0001770.000236

0.0002370.0001770.000236

0.0002370.0001770.000236

0.0005090.0005080.0004831e-06

0.0005090.0005080.0004831e-06

0.0001635.2e-050.000167

0.0001440.000140.00013

1e-06

0.0002020.0001070.000186

0.000139

7e-05

0.0039680.001050.002540.0048390.0041840.0041080.00033

0.0028840.0007710.0016370.0028230.0030430.0029810.000165

0.000232

0.0016630.0005740.0011940.0028230.0021750.001742

0.0006730.0001220.0002230.000689.6e-05

0.0005487.5e-050.0004430.0004130.0005596.9e-05

0.0010840.0002790.0009030.0020160.0011410.0011270.000165

5.4e-05

0.0002510.0002310.0020163.9e-050.0002450.00011

3.9e-05

0.0002630.000295

1.3e-05

0.0002140.00024

6.4e-05

0.0002830.0002130.0001820.000283

4.2e-05

0.0001762.4e-057e-050.0001861.4e-05

0.0002270.0002132.5e-050.000252

2.5e-05

0.0001472.4e-055.3e-050.0001614.1e-05

0.006270.0032590.0221720.0056450.0151080.0065160.010695

0.006270.0032590.0221720.0056450.0151080.0065160.010695

1.9e-051.2e-052.5e-05

0.000240.000269

3.2e-056.5e-054e-063.4e-05

3e-069e-062e-064e-06

0.0002780.000312

0.0002220.000249

0.0002420.000271

0.0003230.000362

0.000178

4.3e-05

0.000290.0001390.0001060.0002941.2e-05

0.000123

0.0001676.9e-050.000140.000183

0.0007750.0004950.0004930.000380.000777

0.0002010.000160.000189

0.0011460.001286

0.0094010.01055

0.000310.0001350.000299

2.7e-05

0.0002240.000251

0.000710.000796

0.000171

0.0013520.001517

0.0002590.00029

0.0006910.000775

0.0005356.3e-050.0004020.0003330.000565

1.6e-05

0.000159

0.000140.0001270.000141

0.0024780.002782

0.0002250.000253

0.000152

0.0004790.0003660.0003080.000504

0.0003590.0008630.0002880.0001670.000413

7.7e-05

0.0002510.0001090.000253

0.0004790.000538

9.7e-05

0.0024780.0013730.0016330.00150.0026041.3e-05

6e-050.0001830.0003150.0056453.6e-056.2e-05

0.0004050.000455

0.000134

0.0001710.0001260.000169

0.0081170.0044120.0044160.0036290.0060970.0079690.000139

8.8e-050.0002750.000122

8.8e-050.0002750.000122

0.0080290.0044120.0044160.0036290.0058220.0078470.000139

2.5e-052.1e-052.8e-05

7.6e-057.8e-058.7e-05

8.9e-05

02.4e-05

3e-069e-063e-06

9.5e-053.3e-050.0001162.8e-05

0.000140

6e-063e-066e-06

2.2e-059e-062.3e-056e-06

1.4e-05

2e-05

1.7e-05

5.8e-056e-067.4e-05

1.5e-059e-061.7e-05

0.000118

1.3e-058e-061.6e-05

02.4e-05

7e-061.1e-057e-06

8e-06

1.3e-05

7.5e-051.2e-058.2e-051.4e-05

9e-054.4e-058.4e-05

6.8e-059e-066.9e-051.4e-05

7e-063.7e-051e-05

0

1.7e-05

1.4e-05

0

7.9e-05

6.5e-05

7.8e-058e-058.8e-05

4.7e-05

0.0069410.0044120.0044160.0036290.0046360.006804

7.5e-05

0.0001450.0001050.000142

0.0001576.5e-050.000184

8e-05

01e-060

0001.5e-05

8e-064e-067e-06

5.3e-05

5.3e-05

5.3e-05

0.0003040.0003530.000233

0.0003040.0003530.000233

0.000192

0.000192

0.0003040.0001610.000233

0.0003040.0001610.000233

0.0232770.0140590.0112580.008770.0166970.0228720.00749

0.0232770.0140590.0112580.008770.0166970.0228720.00749

7.1e-055e-058.4e-05

3.9e-052.6e-054.6e-05

3.2e-052.4e-053.8e-05

0.0003857.7e-050.0002910.0003924.7e-05

0.0002477.7e-050.0001760.0002554.2e-05

0.0001380.0001150.0001375e-06

0.0226690.0139820.0112580.008770.016190.022230.00744

0.000180.0001690.000184

0.0221160.0139820.0112580.008770.0157440.0216530.00744

0.0001730.0001330.000184

0.00020.0001440.000209

0.0001520.0001660.0001663e-06

0.0001520.0001660.0001663e-06

0.0047670.0020680.0022570.0026210.0031680.0046560.001173

0.0002672.3e-050.0001720.00026

0.0002672.3e-050.0001720.00026

0.0002672.3e-050.0001720.00026

0.0003440.0002410.0001870.000348

0.0003440.0002410.0001870.000348

0.0003440.0002410.0001870.000348

0.0041560.0020450.0020160.0026210.0028090.0040480.001173

0.0039680.0020450.0020160.0026210.0026440.0038780.001173

0.0001420.0001420.000148

0.0038260.0020450.0020160.0026210.0025020.003730.001173

0.0001880.0001650.00017

0.0001880.0001650.00017

0.0005290.0008810.0025130.0047380.0005440.0006080.000534

0.0005290.0008810.0025130.0047380.0005440.0006080.000534

2e-052e-052.1e-05

2e-052e-052.1e-05

6.2e-05

6.2e-05

7.4e-05

7.4e-05

0.0005090.0008810.0025130.0047380.0003880.0005870.000534

9e-065e-062e-05

3e-06

2.4e-051.1e-052.9e-05

4.9e-05

3.9e-054.8e-053.3e-05

1e-06

8e-06

0.0001880.0008810.0003570.0023196.2e-050.0002690.000534

7.7e-053.5e-057.2e-05

3.3e-05

6e-055e-065.9e-05

5.3e-05

1.1e-05

4.7e-050.0021560.0024191.6e-054.4e-05

2.2e-05

6.5e-051.2e-056.1e-05

1.4e-05

9.4e-050.0001770.000101

9.4e-050.0001770.000101

9.4e-050.0001770.000101

9.4e-050.0001770.000101

0.0002530.0001510.0002381.4e-05

0.0002530.0001510.0002381.4e-05

0.0002530.0001510.0002381.4e-05

0.0002530.0001510.0002381.4e-05

0.0014945.2e-050.0005020.0020010.0015454.2e-05

0.0014945.2e-050.0005020.0020010.0015454.2e-05

0.000138

0.000138

0.0002842.9e-050.0002580.000296

0.0002842.9e-050.0002580.000296

0.00016

0.00016

0.0002552.3e-050.0003450.000263

0.000216

3e-05

8e-052.3e-054.1e-058.5e-05

0.0001755.8e-050.000178

0.0002350.0001260.000251

0.0002350.0001260.000251

0.000145

0.000145

0.0002220.0001530.0002114.2e-05

0.0002220.0001530.0002114.2e-05

0.000136

0.000136

0.0001840.0002510.00018

0.0001840.0002510.00018

0.0003140.0002440.0005470.000344

0.000109

1e-06

3e-06

1.2e-05

0.000135

8.2e-05

0.0003140.0002440.0002050.000344

0.2250540.1451840.1288020.086290.1646350.220990.070174

0.0004610.000580.0007430.000544

0.0002250.000252

0.0002250.000252

0.000206

0.000206

0.0004610.0003550.0002850.000544

0.0004610.0003550.0002850.000544

9e-063.5e-056e-06

9e-063.5e-056e-06

9e-063.5e-056e-06

0.0003930.0003290.0003180.00041

0.0003930.0003290.0003180.00041

0.0003930.0003290.0003180.00041

0.0016750.0008410.0013670.0013230.0016637e-06

0.0006270.0002990.0003820.0003620.000615

0.0006270.0002990.0003820.0003620.000615

0.0010480.0005420.0009850.0009610.0010487e-06

0.0010480.0005420.0006540.0005890.0010487e-06

0.0003310.000372

0.0002560.0002190.0003940.000243

0.0002560.0002190.000240.000243

0.0002560.0002190.000240.000243

0.000154

0.000154

0.0012990.0007570.0008820.0008590.0012977e-06

0.0012990.0007570.0008820.0008590.0012977e-06

0.000470.0002630.0003240.0003270.000501

0.0004220.0002370.0002830.000290.000389

0.0004070.0002570.0002750.0002420.0004077e-06

0.0003870.0018050.0009030.000350.001076

0.0003870.0003020.0002920.00035

0.0003870.0003020.0002920.00035

0.0002830.000318

0.0002830.000318

0.0002610.000293

0.0002610.000293

0.0009590.001076

0.0009590.001076

0.2185480.1431340.118820.086290.1535940.2144180.068969

0.0005290.0001930.0003120.0003940.000557

0.0005290.0001930.0003120.0003940.000557

0.000170.0007150.00020.0001751.4e-05

0.000170.0007150.00020.0001751.4e-05

0.0012580.001411

0.0012580.001411

0.0007970.0003610.0003890.0020160.0015890.000840.000135

0.0007970.0003610.0003890.0020160.0015890.000840.000135

0.0010319.6e-050.0002820.0010560.0010890.000102

0.0010319.6e-050.0002820.0010560.0010890.000102

0.0006997.2e-050.0021070.0022520.000674

0.0010910.001225

0.0006997.2e-050.0005420.0004950.000674

0.0004740.000532

0.0020410.0007370.0021720.0034080.0022226e-05

0.0008650.00097

0.0010290.0004070.0007170.0015390.0011241.4e-05

0.0010120.000330.000590.0008990.0010984.6e-05

0.2129370.140960.11230.0842740.1429540.2085210.068658

0.0007570.00085

0.0018930.002124

0.0004590.000515

0.000630.000708

6.7e-05

9.3e-050.0006870.0001790.000102

0.2128440.1402730.1060160.0842740.1356240.2084190.068658

3.1e-05

0.0006010.000675

0.0013590.001525

0.0005850.000656

0.0003440.000330.00034

0.0002590.0001390.000276

8.5e-050.0001916.4e-05

0.0006170.0001010.0004430.00062

0.0006170.0001010.0004430.00062

0.0006170.0001010.0004430.00062

7e-050.0002620.0002946.7e-05

7e-050.0002620.0002946.7e-05

7e-050.0002620.0002946.7e-05

0.0013390.0003510.0042380.0053930.0013720.000115

0.0002496.5e-050.0002130.0002140.000261

0.0002496.5e-050.0002130.0002140.000261

0.000260.000292

0.000260.000292

0.0002550.000286

0.0002550.000286

0.000196

0.000196

0.0003510.0001040.000170.0003535.6e-05

0.0003510.0001040.000170.0003535.6e-05

0.0004820.000541

0.0004820.000541

0.0007390.0001820.0022930.0027890.0007585.9e-05

0.0007390.0001820.0003650.0006240.0007585.9e-05

0.0005040.000566

0.000280.000314

0.0002880.000324

0.000210.000236

0.0006460.000725

0.0002310.000338

0.0002310.000259

7.9e-05

0.0002880.000324

0.0002880.000324

0.0002160.000243

0.0002160.000243

0.00030.000336

0.00030.000336

0.00030.000336

0.0049780.0006760.0008810.0066920.0050790.00033

0.0049780.0006760.0008810.0066920.0050790.00033

0.0049780.0006760.0008810.0066920.0050790.00033

0.0049780.0006760.0008810.0066920.0050790.00033

0.0049780.0006760.0008810.0066920.0050790.00033

0.0002010.0004520.000183

0.0002010.0004520.000183

0.0002010.0004520.000183

0.0002010.0004520.000183

0.000173

0.0002010.0002790.000183

0.0007470.0011280.000604

0.0003870.0005140.000379

0.0003870.0005140.000379

0.0001330.0001930.000147

5.6e-057.6e-055.6e-05

7.7e-050.0001179.1e-05

2.3e-055.7e-052.2e-05

2.3e-055.7e-052.2e-05

8.7e-05

8.7e-05

5.2e-055.6e-05

5.2e-055.6e-05

6.6e-050.0001015.6e-05

6.6e-050.0001015.6e-05

0.0001137.6e-059.8e-05

0.0001137.6e-059.8e-05

0.000360.0006140.000225

6.5e-050.0002095.2e-05

6.5e-050.0001185.2e-05

6.5e-050.0001185.2e-05

9.1e-05

9.1e-05

0.0002950.0004050.000173

0.0001740.0001448.5e-05

0.0001740.0001448.5e-05

0.0001210.0002618.8e-05

0.0001210.0001438.8e-05

0.000118

0.000250.0002930.000174

0.000250.0002930.000174

0.000250.0002930.000174

0.0001080.0001588.6e-05

0.0001080.0001588.6e-05

0.0001420.0001358.8e-05

0.0001420.0001358.8e-05

1.1e-056.5e-059e-06

6.5e-05

6.5e-05

6.5e-05

6.5e-05

6.5e-05

1.1e-059e-06

1.1e-059e-06

1.1e-059e-06

1.1e-059e-06

1.1e-059e-06

9.1e-050.0005988.4e-05

4.7e-05

4.7e-05

4.7e-05

4.7e-05

4.7e-05

7.2e-05

7.2e-05

7.2e-05

7.2e-05

7.2e-05

2.5e-050.0002223.2e-05

6e-066.4e-051.8e-05

6e-066.4e-051.8e-05

6e-066.4e-051.8e-05

6e-066.4e-051.8e-05

1.9e-050.0001581.4e-05

1.9e-050.0001581.4e-05

6.8e-05

6.8e-05

4.2e-05

4.2e-05

1.9e-054.8e-051.4e-05

1.9e-054.8e-051.4e-05

1.7e-055.2e-056e-06

1.7e-055.2e-056e-06

1.7e-055.2e-056e-06

1.7e-055.2e-056e-06

1.7e-055.2e-056e-06

4.9e-050.0002054.6e-05

4.9e-050.0002054.6e-05

4.9e-050.0002054.6e-05

4.9e-050.0002054.6e-05

4.9e-059.8e-054.6e-05

0.000107

4e-064.4e-052e-06

4e-064.4e-052e-06

4e-064.4e-052e-06

4e-064.4e-052e-06

4e-064.4e-052e-06

4e-064.4e-052e-06

4.8e-050.0002013e-05

7e-05

7e-05

7e-05

7e-05

7e-05

3.3e-055.8e-051.4e-05

3.3e-055.8e-051.4e-05

3.3e-055.8e-051.4e-05

3.3e-055.8e-051.4e-05

3.3e-055.8e-051.4e-05

1.5e-057.3e-051.6e-05

1.5e-057.3e-051.6e-05

1.5e-057.3e-051.6e-05

1.5e-057.3e-051.6e-05

1.5e-057.3e-051.6e-05

3.9e-050.0001032.5e-05

3.9e-050.0001032.5e-05

3.9e-050.0001032.5e-05

3.9e-050.0001032.5e-05

3.9e-050.0001032.5e-05

3.9e-050.0001032.5e-05

0.0026090.0137690.0029440.0117940.0015860.0022680.014319

0.0026090.0137690.0029440.0117940.0015860.0022680.014319

1.9e-050.000121.6e-05

1e-062.5e-050

1e-062.5e-050

1e-062.5e-050

1.6e-057.7e-051.4e-05

1e-065.3e-050

1e-065.3e-050

1.5e-052.4e-051.4e-05

2.4e-05

1.5e-051.4e-05

2e-061.8e-052e-06

2e-061.8e-052e-06

2e-061.8e-052e-06

0.002590.0137690.0029440.0117940.0014660.0022520.014319

0.002590.0137690.0029440.0117940.0014660.0022520.014319

0.002590.0137690.0029440.0117940.0014660.0022520.014319

1.8e-052e-061.1e-05

4e-062e-063e-06

1e-051.9e-059e-064.9e-05

0.0025470.0137690.0029440.0117940.0013420.0022170.01427

5e-062.8e-055e-06

000

06e-061e-06

6e-063.5e-056e-06

2e-05

1.2e-05

0.0032110.0017110.0017120.0026210.0028940.0031210.000914

7e-050.0001164.7e-05

7e-050.0001164.7e-05

7e-050.0001164.7e-05

7e-050.0001164.7e-05

7e-050.0001164.7e-05

0.0031410.0017110.0017120.0026210.0027780.0030740.000914

0.0031410.0017110.0017120.0026210.0027780.0030740.000914

2.5e-050.0001052e-05

3e-066.4e-052e-06

3e-066.4e-052e-06

2.2e-054.1e-051.8e-05

2.2e-054.1e-051.8e-05

0.0031160.0017110.0017120.0026210.0026730.0030540.000914

7.5e-05

7.5e-05

1e-053.9e-055e-06

1e-053.9e-055e-06

0.0030840.0017110.0017120.0026210.0024530.003030.000914

0.0030840.0017110.0017120.0026210.0024530.003030.000914

1.7e-055.3e-051.6e-05

1.7e-055.3e-051.6e-05

5e-065.3e-053e-06

5e-065.3e-053e-06

0.1785290.2149260.278490.2755050.345150.1752630.34034

0.0521850.0446520.0590230.0346780.061810.0492670.037034

8.1e-050.0003289.8e-05

4.5e-050.0001316e-05

3.6e-055.8e-055.2e-05

3.6e-055.8e-055.2e-05

9e-067.3e-058e-06

9e-067.3e-058e-06

3.6e-050.0001973.8e-05

1.8e-057.5e-051.7e-05

1.8e-057.5e-051.7e-05

1.8e-050.0001222.1e-05

1.1e-057.1e-051.5e-05

7e-065.1e-056e-06

0.0002420.0008420.000235

9.9e-050.0002250.000115

3.7e-059.4e-054.2e-05

01e-051e-06

01.6e-051e-06

1.1e-056e-061.3e-05

6e-061.2e-056e-06

03e-060

1.2e-056e-061.3e-05

7e-063.8e-057e-06

1e-063e-061e-06

5.4e-055.5e-056.8e-05

1.4e-052.7e-051.5e-05

4e-052.8e-055.3e-05

8e-067.6e-055e-06

8e-067.6e-055e-06

1.8e-050.0001091.3e-05

5e-066.1e-052e-06

5e-066.1e-052e-06

1.3e-054.8e-051.1e-05

1.3e-054.8e-051.1e-05

0.00010.0003938.1e-05

1.9e-056.4e-057e-06

1.9e-056.4e-057e-06

7e-063.9e-051e-05

1.8e-05

2e-067e-065e-06

4e-062e-063e-06

1e-061.2e-052e-06

1.3e-055.5e-051.3e-05

1.3e-055.5e-051.3e-05

3e-052.1e-051.5e-05

3e-052.1e-051.5e-05

5e-065e-053e-06

5e-065e-053e-06

2.2e-059.1e-052.9e-05

2.2e-059.1e-052.9e-05

4e-067.3e-054e-06

4e-067.3e-054e-06

2.5e-050.0001152.6e-05

2.5e-050.0001152.6e-05

2.5e-050.0001152.6e-05

1.2e-056.1e-051e-05

1.2e-056.1e-051e-05

1.2e-056.1e-051e-05

1.2e-056.1e-051e-05

0.051850.0446520.0590230.0346780.0605790.0489240.037034

0.0007150.0033840.0126380.0054440.0038210.000590.011542

0.0001140.0028710.0015420.001367.9e-050.000917

8.7e-050.0028710.0008180.0005475.2e-050.000917

0.0004660.000523

2.7e-050.0002580.000292.7e-05

0.0001290.0052820.0004674.5e-050.005929

0.0003320.000373

0.0007760.000871

0.0041740.004685

0.0001290.0004674.5e-05

2.1e-050.0035810.0002952.7e-050.003723

0.0033180.003723

2.1e-050.0002630.0002952.7e-05

3.8e-050.000132.9e-05

3.8e-050.000132.9e-05

0.0002280.000256

0.0002280.000256

0.00030.0002130.000103

0.0002390.000213

6.1e-05

1.5e-05

8.8e-05

7.5e-050.0012320.0005590.000824

0.0007340.000824

7.5e-05

0.0004980.000559

0.0004130.0001380.000560.0054440.0007540.000412.9e-05

0.0004136.7e-050.000560.0054440.0007540.000412.9e-05

7.1e-05

1.7e-05

1.7e-05

0.0443070.0283520.0288510.0222780.0387730.0434290.020443

0.0442050.0283520.0288510.0222780.0384770.0433240.020443

6.7e-05

8e-065.7e-059e-06

0.0024530.002753

4.8e-052.6e-053.3e-051.5e-05

6e-05

2e-05

4.9e-053.9e-055.3e-05

7.7e-050.0001012e-068.1e-05

0.0434540.0280930.0226010.0191530.0324760.0425230.014002

8.2e-05

8e-053.3e-058.4e-05

4.5e-051.6e-054.9e-057e-06

8.6e-050.0001050.0031251.9e-059.1e-05

0.000104

5e-053.9e-055.6e-05

1.8e-052.1e-052e-05

2.9e-052.5e-051.4e-054.4e-05

1e-05

7.2e-05

6.7e-052.8e-053.5e-057.1e-05

2e-05

9.1e-054e-050.0001

3.8e-057e-054.5e-05

6.5e-055.3e-056.5e-05

7e-06

0.0037970.0051020.003659

4.5e-054.9e-054.2e-05

2.9e-054.2e-052.2e-05

1.6e-053e-062e-05

4e-06

8.9e-05

8.9e-05

5.7e-050.0001586.3e-05

2.4e-057e-053.1e-05

3.3e-058.8e-053.2e-05

0.0001160.0001169.8e-05

0.0001160.0001169.8e-05

0.0001160.0001169.8e-05

3.7e-056.6e-053.2e-05

3.7e-056.6e-053.2e-05

3.7e-056.6e-053.2e-05

0.0001050.000230.000117

0.0001050.000230.000117

0.0001050.000230.000117

0.0035610.0075370.0024990.0046370.0020460.001630.004725

0.000104

0.000104

0.0033990.0075370.0024990.0046370.001650.0014720.004725

0.0033990.0075370.0024990.0046370.001650.0014720.004725

0.0001170.000170.000112

0.0001170.000170.000112

4.5e-050.0001224.6e-05

4.5e-050.0001224.6e-05

2.3e-050.0001343e-05

5e-067.9e-053e-06

5e-067.9e-053e-06

1.8e-055.5e-052.7e-05

1.8e-055.5e-052.7e-05

5.8e-050.0040220.0110260.0082255.5e-050.00024

5.8e-050.0001135.5e-05

5.8e-050.0001135.5e-05

0.0061970.006954

0.0061970.006954

0.0010320.001158

0.0010320.001158

0.0040220.003583

0.0040220.003583

0.0002140.00024

0.0002140.00024

0.0019870.001190.0013760.0023190.0020260.001963

9e-066.6e-052e-05

9e-066.6e-052e-05

2e-062.8e-052e-06

1e-062e-061e-06

2.3e-05

1e-063e-061e-06

4.1e-058.5e-052.5e-05

4.1e-058.5e-052.5e-05

6.2e-050.0002089.7e-05

2.7e-055.4e-056e-05

5e-05

7e-062.4e-057e-06

2.8e-058e-053e-05

2.7e-056.8e-053.8e-05

2.7e-056.8e-053.8e-05

1.6e-05

1.6e-05

4.9e-050.0001234.8e-05

4e-063.9e-055e-06

3.5e-055.5e-053.3e-05

1e-052.9e-051e-05

0.0017810.001190.0013760.0023190.0014480.001733

1.1e-053.9e-051e-05

1.2e-054.5e-051.4e-05

1.3e-05

0.0017260.001190.0013760.0023190.0012590.001688

4e-065.6e-051.1e-05

1.5e-054.9e-051e-05

0.0002870.000323

0.0002870.000323

0.0002870.000323

0.0005290.0001070.0020670.003040.0005486.9e-05

6.9e-05

6.9e-05

3.5e-050.0007490.0010533.9e-05

5e-064.9e-056e-06

1.2e-054.8e-051.6e-05

1.2e-053.1e-051.1e-05

0.0005140.000577

0.0002350.000263

6e-068.5e-056e-06

0.000107

0.000107

0.0001240.0002520.000123

0.0001240.0002520.000123

0.000260.0013180.001480.000263

0.0009960.001118

8.1e-050.0003220.0003628.1e-05

0.0001790.000182

0.000110.0002550.000123

0.000110.0002550.000123

0.0001850.0007810.000194

0.0001640.0006850.000174

4.1e-050.0001024.4e-05

0.0001230.0005830.00013

2.1e-059.6e-052e-05

2.1e-059.6e-052e-05

0.0002276e-050.0002790.0009980.0002381.5e-05

0.0002276e-050.0002790.0009980.0002381.5e-05

0.0002276e-050.0002790.0009980.0002381.5e-05

0.1234160.1690580.2164380.238710.2792260.1231230.301997

0.0485290.0461850.0583670.1215720.0359960.0455760.030865

0.0362020.0394810.0469250.0615930.0238260.0333110.026393

0.0001037e-060.0032620.0033270.0005240.0001921.5e-05

7.9e-050.0001169.7e-051.5e-05

0.0002980.000334

2.4e-057e-060.0029640.0033277.4e-059.5e-05

0.0360990.0394740.0436630.0582660.0233020.0331190.026378

0.0026930.004890.0022050.0040320.0012180.002658

0.000230.0001670.000235

1.7e-05

2.5e-056e-050.0002220.000105

0.0009170.0002490.0002430.0001540.0009230.000176

1.4e-05

6.5e-05

0.0001440.0001570.000142

0.0002270.000255

0.0007830.000879

7.9e-05

0.0011620.001304

0.0003250.0002955.2e-050.0003372e-05

0.000530.000595

5.9e-05

0.000108

0.0011760.00132

0.0005020.000190.0002680.0003330.0005510.00022

0.000710.00010.0002540.0007640.000755.9e-05

0.000670.0041160.0012250.0052421.8e-050.0006710.004549

0.0004810.00054

0.0001325.1e-050.000136

3.3e-05

5e-050.0001086.8e-054.5e-05

0.0028230.003168

4.2e-05

0.0003520.0003950.0008430.000366

0.0115720.0144510.0058610.0072580.0026390.0076760.009125

3.3e-05

0.0006440.0002370.0003330.0040320.0001320.0006570.001047

1.6e-05

0.00012

0.0002860.0003260.0006790.00031

0.0136510.0136470.007240.0077620.0037650.013437

0.0052580.005901

0.0007610.0002010.000410.0008310.0008236.4e-05

0.0001550.0003470.0002770.0002880.0004790.000826

9.2e-055.7e-050.0006020.0115930.0004580.000461

0.0001840.0001321.8e-050.000194

0.0005570.0001460.0003010.0004730.0005860.000434

0.0014840.001665

6.3e-053.6e-050.0070960.0079645.5e-056e-05

0.0002120.0002070.000221

0.0015830.001776

3.1e-050.0001880.0003270.0073598e-063.3e-058.9e-05

0.0001713.8e-050.0002250.0030246.2e-050.0001756.3e-05

0.0002120.0001110.0006440.0002268.6e-05

0.0006240.0001380.0002730.0003870.0006292.3e-05

0.0001343.2e-050.0002030.0002331.4e-05

0.0001560.0001320.0016260.0022850.000122

0.0001560.0001320.0016260.0022850.000122

0.0001560.0001320.0002540.000122

8.5e-05

0.000710.000797

0.0006170.000693

0.000121

0.0002990.000335

0.0028470.0027030.0032480.0127010.0038170.0030060.001764

0.0001367.9e-050.000123

7.7e-052.4e-057.2e-05

5.9e-054.2e-055.1e-05

1.3e-05

0.0027110.0027030.0032480.0127010.0037380.0028830.001764

03e-060

4.5e-05

7.6e-05

0.0002770.0002660.0003370.000288

0.0001190.0002673.9e-050.000126

0.000102

2.8e-05

0.0002380.0003630.0001980.0002567.9e-05

1.1e-053.2e-051e-05

2.7e-050.0001552.4e-05

0.0003990.0013320.0006240.002525.3e-050.000390.001083

8.6e-05

7.7e-05

3e-066e-063e-06

1.4e-051e-051e-05

2.8e-05

7.3e-05

0.0002150.0002210.0002284.4e-05

0.0004090.000459

3.7e-05

1e-064e-060

4.5e-053.7e-054.4e-05

2.6e-059.9e-050.000105

7.2e-054.6e-059.7e-05

0.0003630.0003420.0004190.0081658.3e-050.0003688.6e-05

2.8e-05

2.2e-05

1.7e-051.6e-051.6e-05

9.9e-056e-050.000113

0.0003650.0003990.000290.0020160.0001440.0003610.000332

7.8e-05

2.4e-056.3e-052.6e-05

0.0003960.0003620.000418

4.3e-05

1.8e-05

6.6e-05

2.1e-05

0.0008780.000985

0.0023280.0038690.0014680.0472780.0008750.0022890.002574

5.7e-050.000125.7e-05

5.7e-050.000125.7e-05

5.5e-059e-056.2e-05

5.5e-059e-056.2e-05

0.0022160.0038690.0014680.0472780.0006650.002170.002574

3.6e-05

0.0001950.000360.000250.0411293.5e-050.000158

0.0004480.0002330.0002430.0020160.0001150.0004530.000246

0.0014220.0032760.0009750.0041330.0003240.0013930.002328

3.9e-052.8e-055.1e-05

4.5e-05

0.0001128.2e-050.000115

0.0002390.0005590.000208

0.0001140.0002340.000101

3.8e-05

7e-05

0.0001140.0001260.000101

6.1e-050.0002135e-05

1.2e-052.4e-056e-06

7e-063.9e-056e-06

1.1e-053.1e-059e-06

1e-064e-063e-06

1e-062e-061e-06

6.7e-05

2.9e-054.6e-052.5e-05

6.4e-050.0001125.7e-05

6.4e-050.0001125.7e-05

0.0067570.00510.0046340.006640.000134

0.0067570.00510.0046340.006640.000119

9.4e-050.0001820.000104

0.000102

4.3e-058.8e-054.9e-051.7e-05

0.0002260.000254

0.006620.0048740.004110.006487

1.5e-05

1.5e-05

0.07488700000000010.1228730.1580710.1171380.243230.0775470.271132

0.000140.0003250.000169

0.000186

0.000186

0.000140.0001390.000169

8.2e-059.6e-058.5e-05

6e-061.9e-051.3e-05

3.4e-054e-064.3e-05

1.8e-051.7e-052.8e-05

3e-06

0.0270720.0944560.0112840.0896170.0096380.0294630.172636

7.9e-05

7.9e-05

9.2e-050.0001330.000106

9.2e-050.0001330.000106

9.5e-057.3e-059.4e-05

9.5e-057.3e-059.4e-05

7.4e-05

7.4e-05

0.0076490.0054590.0044520.0038310.0036510.0075180.000197

7.5e-053.6e-057.2e-05

9e-062.2e-051.4e-057e-06

9e-06

1.4e-05

7e-06

5e-065e-066e-063.7e-05

01.4e-05

1.7e-053e-062.1e-059.5e-05

2.4e-053.1e-053.1e-057e-06

3e-068e-063e-061.6e-05

1.1e-05

2.2e-05

3e-06

2e-05

3e-064e-061.1e-05

0.0075130.0054590.0044520.0038310.0034770.00736

2e-067e-063e-067e-06

2e-067e-063e-067e-06

0.0171990.0883480.004770.0830640.00310.0196590.17218

0.000150.0001130.000158

0.0006060.00068

3.2e-056.2e-053e-05

0.0091140.0516110.0008460.020060.0001660.0107820.039444

0.0001351.1e-05

5e-06

0.0001523e-060.000176

8.6e-05

5e-06

4.2e-053.7e-054.4e-05

1.5e-05

8.3e-05

0.0009358.7e-050.0008586.8e-050.000993

7.1e-05

0.0018680.0014070.000620.0156250.0001480.0018880.129327

5e-062e-066e-06

7.4e-05

0.0002320.0023650.0003250.007469e-060.000230.000222

0.0003670.0001090.000443

3.6e-05

7.7e-05

6.3e-057e-056.3e-05

7.7e-051.9e-057.4e-05

0.0009840.0003510.0004530.021270.0001980.001133

8e-067e-063e-068e-06

0.0022290.0324080.0005210.0146170.0001110.0028150.003128

1.4e-052e-061.4e-05

6.2e-05

0

6.2e-05

0.000123

3.5e-05

6.8e-05

6.5e-051.3e-056.5e-055.9e-05

2e-06

0.000147.3e-050.000133

0

8.9e-05

0.0001133.7e-053.5e-050.000102

5.9e-056.1e-056.4e-05

0.0004157.5e-050.0005410.0040325.6e-050.000438

8.2e-05

7.6e-05

5.2e-05

5.2e-05

8.3e-05

8.3e-05

7.3e-05

3.4e-05

3.9e-05

6.8e-05

4.1e-05

1.9e-05

8e-06

0.0020110.0006490.0020620.0027220.0021840.0020620.000252

7.6e-05

0.0020110.0006490.0005920.0027220.0004580.0020620.000252

0

0.001470.00165

2.4e-056.1e-052.1e-05

2.4e-056.1e-052.1e-05

0.0001253e-064.7e-050.00012

0.0001253e-064.7e-050.00012

1.5e-051.7e-051.4e-05

2e-063e-061e-062e-06

3.9e-051.2e-053.7e-05

3.9e-055e-063.9e-05

3e-051.2e-052.8e-05

8.4e-050.0005559.4e-05

9.8e-05

9.8e-05

0.000118

0.000118

0.000103

0.000103

8.4e-053.5e-059.4e-05

8.4e-053.5e-059.4e-05

7.1e-05

7.1e-05

0.00013

1e-05

1.5e-05

1.8e-05

1.7e-05

2e-05

1.2e-05

2e-05

7e-06

1.1e-05

0.0010370.0010340.0010730.0023190.0019340.0020440.00063

0.0010320.0010340.0010730.0023190.0018560.002040.00063

0.0010320.0010340.0010730.0023190.0018560.002040.00063

5e-067.8e-054e-06

5e-067.8e-054e-06

2.3e-055e-051.6e-05

2.3e-055e-051.6e-05

2.3e-055e-051.6e-05

0.0004622e-060.0004070.000527

8.1e-050.000147.4e-05

8.1e-050.000147.4e-05

0.0003812e-060.0001750.000453

2.6e-058e-062.7e-05

2e-062e-068e-064e-06

9e-063e-067e-06

2.1e-051e-062.2e-05

1.1e-05

1e-05

6.2e-056e-066.2e-05

8.4e-056e-068.9e-05

0

3e-06

4.7e-05

1.2e-05

9e-06

9e-06

2.8e-054e-062.1e-05

4.5e-051.5e-055.6e-05

6e-06

2.8e-056e-066e-05

7.6e-051.1e-050.000105

9.2e-05

9.2e-05

0.0459440.0273780.1457140.0252020.2302740.0451140.097866

5.5e-050.0001094.8e-05

5.5e-050.0001094.8e-05

0.0007650.0001410.1171410.1992110.0007860.097836

0.1168830.1989690.097836

0.0007650.0001410.0002580.0002420.000786

0.0451240.0272370.0285730.0252020.0308440.044283e-05

6.8e-05

1.6e-05

2.4e-05

3e-058.1e-053.4e-051.5e-05

2e-053.3e-051.8e-05

5.1e-05

9.5e-05

2.4e-05

1.8e-05

1.2e-05

0.0444810.0269940.0285730.0252020.0295930.043609

6.5e-05

1.9e-05

9.3e-057.1e-054e-054.7e-051.5e-05

2.4e-05

8e-06

6e-05

9.1e-054.6e-059.4e-05

1.1e-05

5e-05

0.00014

3.6e-05

7.1e-05

2.2e-05

0.0004090.0001720.0001640.000478

4.3e-05

2.3e-05

7e-06

0.00011

0.00011

0.0017460.0008050.0012060.0018940.001708

0.0017460.0008050.0012060.0018940.001708

0.0017460.0008050.0012060.0018940.001708

0.0013890.0006820.0009140.0010110.001378

0.0013890.0006820.0009140.0010110.001378

0.000157

0.000157

0.0002920.000328

0.0002920.000328

0.0003570.0001230.0003980.00033

0.0003570.0001230.0003980.00033

0.0002910.0014340.0007310.0002880.000984

0.0002910.0014340.0007310.0002880.000984

0.0002910.0014340.0007310.0002880.000984

0.0002610.0002330.000263

0.0002610.0002330.000263

0.000119

0.000119

0.0012010.0003640.000984

0.0003240.000364

0.0008770.000984

3e-050.0001272.5e-05

3e-053e-052.5e-05

9.7e-05

0.000121

0.000121

0.0008910.0004110.0003890.0021170.001380.0008770.000325

7.9e-050.0003688.4e-05

0.000111

0.000111

4.5e-05

6.6e-05

7.9e-050.0002578.4e-05

7.9e-050.0002578.4e-05

4.2e-059.3e-054.4e-05

1.3e-05

3.8e-05

3.7e-059.3e-054e-05

2e-05

7.8e-050.000337.7e-05

7.8e-050.000337.7e-05

9.8e-05

9.8e-05

2.9e-050.0001083e-05

2.9e-050.0001083e-05

4.9e-050.0001244.7e-05

4.9e-050.0001244.7e-05

0.0007340.0004110.0003890.0021170.0006820.0007160.000325

0.0007340.0004110.0003890.0021170.0006820.0007160.000325

0.0007340.0004110.0003890.0021170.0006820.0007160.000325

5.1e-050.0001115.2e-05

0.000162

0.0006830.0004110.0003890.0021170.0005710.0006640.000163

0.000109

0.000109

0.000109

0.000109

0.000109

0.0003150.0003590.000960.0002960.000403

0.0003590.000403

0.0003590.000403

0.0003590.000403

0.0003590.000403

0.0003590.000403

0.0002070.0003110.000191

0.0001550.0001730.00016

0.0001550.0001730.00016

0.0001550.0001730.00016

0.0001550.0001730.00016

5.2e-050.0001383.1e-05

5.2e-050.0001383.1e-05

5.2e-050.0001383.1e-05

5.2e-050.0001383.1e-05

5e-050.0001324.2e-05

5e-050.0001324.2e-05

9e-064.1e-053e-06

9e-064.1e-053e-06

0

0

9e-064.1e-053e-06

4.1e-059.1e-053.9e-05

4.1e-059.1e-053.9e-05

2e-054.8e-051.9e-05

2.1e-054.3e-052e-05

1.2e-050.0002281.3e-05

1.2e-050.0002281.3e-05

1.2e-050.0002281.3e-05

0.000141

0.000141

1.2e-058.7e-051.3e-05

1.2e-058.7e-051.3e-05

2.2e-050.0002111.7e-05

4e-061.1e-053e-06

4e-061.1e-053e-06

4e-061.1e-053e-06

1e-06

4e-061e-053e-06

1.8e-050.00021.4e-05

1.8e-050.00021.4e-05

1.8e-050.00021.4e-05

1.8e-050.00021.4e-05

2.4e-057.8e-053.3e-05

2.4e-057.8e-053.3e-05

2.4e-057.8e-053.3e-05

2.4e-057.8e-053.3e-05

2.4e-057.8e-053.3e-05

0.0001010.0003339.6e-05

0.0001010.0003339.6e-05

5e-059.2e-054.7e-05

5e-059.2e-054.7e-05

1.3e-053.4e-051.3e-05

1.3e-053.4e-051.3e-05

3.7e-053e-053.4e-05

3.7e-053e-053.4e-05

2.8e-05

2.8e-05

9e-069.2e-059e-06

9e-069.2e-059e-06

3e-066.5e-054e-06

3e-064e-054e-06

2.5e-05

6e-062.7e-055e-06

6e-062.7e-055e-06

4.2e-050.0001494e-05

2.1e-057.9e-052e-05

9e-063.7e-058e-06

6e-061.6e-056e-06

3e-061.1e-052e-06

1e-05

1.2e-054.2e-051.2e-05

1e-052.3e-059e-06

2e-061.9e-053e-06

2.1e-057e-052e-05

2e-054.2e-052e-05

1.8e-053e-051.7e-05

00

3e-06

2e-069e-063e-06

1e-062.8e-050

0

1e-06

000

1e-06

1.2e-05

00

1.2e-05

1e-0600

0

2e-06

0.0001440.0004090.000124

0.0001440.0004090.000124

8.2e-050.0003086.4e-05

7e-060.0001728e-06

3e-068.9e-051e-06

3e-068.9e-051e-06

3e-064e-062e-06

1e-060

2e-064e-062e-06

01.5e-054e-06

01.5e-054e-06

1e-066.4e-051e-06

3.8e-05

1e-062.6e-051e-06

7.5e-058.4e-055.6e-05

5.9e-054.6e-052.8e-05

4.8e-052.9e-051.7e-05

1.1e-051.7e-051.1e-05

1.6e-053.8e-052.8e-05

1.6e-053.8e-052.8e-05

5.2e-05

5.2e-05

5.2e-05

6.2e-050.0001016e-05

6.2e-050.0001016e-05

1.3e-055.7e-051e-05

1.3e-055.7e-051e-05

4.9e-054.4e-055e-05

4.9e-054.4e-055e-05

0.0079270.0098560.0053140.0090730.0053710.0196890.004879

0.0079270.0098560.0053140.0090730.0053710.0196890.004879

0.0007790.0028460.0007420.004940.0021270.000730.001756

0.0004380.0019650.0004390.0028230.0019470.0004060.001155

2e-050.000211.9e-05

1.2e-058.9e-051.1e-05

3e-066.1e-053e-06

5e-066e-055e-06

0.0003460.0019650.0004390.0028230.0015320.0003390.001155

1.1e-056.7e-051e-05

6e-065.5e-056e-06

7e-060.0001456e-06

5e-065.9e-055e-06

9e-066.4e-058e-06

7.3e-05

6.5e-055.3e-056.4e-05

4e-064.3e-053e-06

2.3e-059.8e-052.2e-05

0.0002160.0019650.0004390.0028230.0008490.0002150.001155

2.6e-05

1e-057.3e-056e-06

1e-057.3e-056e-06

1.9e-054.4e-051.3e-05

1.9e-054.4e-051.3e-05

4.3e-058.8e-052.9e-05

3.4e-055.4e-052.2e-05

9e-063.4e-057e-06

0.0003410.0008810.0003030.0021170.000180.0003240.000601

2.1e-053.2e-051.3e-05

1e-062e-06

1e-061e-061e-06

06e-06

1.5e-05

3e-06

3e-062e-06

1.6e-055e-061e-05

0.000320.0008810.0003030.0021170.0001480.0003110.000601

2e-063e-062e-06

3e-064e-061e-06

01e-060

4e-061e-064e-06

7e-069e-067e-06

0.0003040.0008810.0003030.0021170.000130.0002970.000601

0.0064860.006730.0043740.0041330.0029930.0182870.003035

0.0064860.006730.0043740.0041330.0029930.0182870.003035

0.0064750.006730.0043740.0041330.0029390.0182780.003035

5e-064.9e-055e-06

0.000103

0.0005970.00067

0.0063810.0066840.0037770.0041330.0020710.0181590.003035

8.9e-054.6e-054.6e-050.000114

1.1e-055.4e-059e-06

1.1e-055.4e-059e-06

0.0006620.000280.0001980.0002510.0006728.8e-05

0.0006620.000280.0001980.0002510.0006728.8e-05

0.0006620.000280.0001980.0002510.0006728.8e-05

0.0002196e-050.00019800.0002258.8e-05

1.4e-051.9e-051.4e-05

0.00027.6e-050.0001330.000202

0.0002290.0001449.9e-050.000231

0.0029010.0018160.0017640.0026210.0020590.0028410.001102

0.0029010.0018160.0017640.0026210.0020590.0028410.001102

0.0029010.0018160.0017640.0026210.0020590.0028410.001102

0.0029010.0018160.0017640.0026210.0020590.0028410.001102

0.0029010.0018160.0017640.0026210.0020590.0028410.001102

0.0028990.0018160.0017640.0026210.0020270.0028370.001102

2e-063.2e-054e-06

5.8e-050.0001523.3e-05

5.8e-050.0001523.3e-05

5.8e-050.0001523.3e-05

5.8e-050.0001523.3e-05

5e-064.1e-053e-06

5e-064.1e-053e-06

3.2e-053.5e-051.3e-05

3.2e-053.5e-051.3e-05

6e-063.5e-051e-06

6e-063.5e-051e-06

1.5e-054.1e-051.6e-05

1.5e-054.1e-051.6e-05

2.3e-050.0001382.5e-05

2.3e-050.0001382.5e-05

2.3e-050.0001382.5e-05

2.3e-050.0001382.5e-05

2.3e-050.0001382.5e-05

2.3e-050.0001382.5e-05

4.7e-056.7e-053.5e-05

4.7e-056.7e-053.5e-05

4.7e-056.7e-053.5e-05

4.7e-056.7e-053.5e-05

4.7e-056.7e-053.5e-05

4.7e-056.7e-053.5e-05

0.0035380.0004010.0214940.0020160.0254490.0036938e-05

8e-050.0001285.9e-05

8e-050.0001285.9e-05

8e-050.0001285.9e-05

8e-050.0001285.9e-05

5e-057.2e-053.3e-05

3e-055.6e-052.6e-05

0.0034580.0004010.0214940.0020160.0253210.0036348e-05

0.0001180.0001060.000124

6.4e-054.7e-056.7e-05

6.4e-054.7e-056.7e-05

6.4e-054.7e-056.7e-05

5.4e-055.9e-055.7e-05

5.4e-055.9e-055.7e-05

5.4e-055.9e-055.7e-05

0.0002840.0003640.000302

4.1e-056.5e-054.2e-05

4.1e-056.5e-054.2e-05

1.8e-053.5e-051.9e-05

2.3e-053e-052.3e-05

6.5e-056.6e-056.4e-05

3.3e-054.2e-053.2e-05

3.3e-054.2e-053.2e-05

2.1e-052e-05

2.1e-052e-05

1.1e-052.4e-051.2e-05

1.1e-052.4e-051.2e-05

8e-050.0001339.5e-05

8e-050.0001339.5e-05

1.9e-053.8e-052.3e-05

2.3e-052.7e-052.5e-05

1.2e-053.3e-052.1e-05

2e-053e-051.7e-05

1e-064e-063e-06

5e-061e-066e-06

3.9e-055.8e-053.3e-05

3.9e-055.8e-053.3e-05

3.9e-055.8e-053.3e-05

5.9e-054.2e-056.8e-05

5.9e-054.2e-056.8e-05

5.9e-054.2e-056.8e-05

0.0006930.0001380.0002450.0020160.0011240.0006676.7e-05

2.1e-053.9e-052e-05

2.1e-053.9e-052e-05

2.1e-053.9e-052e-05

0.0003030.0001380.0002450.0020160.0002620.0003026.7e-05

0.0003030.0001380.0002450.0020160.0002620.0003026.7e-05

0.0003030.0001380.0002450.0020160.0002620.0003026.7e-05

0.0003280.0006620.000304

0.0002040.0004960.000182

3e-055.5e-052.4e-05

1.5e-057.1e-051.9e-05

0

1e-052.4e-053e-06

9e-0601.2e-05

2.8e-054.9e-051.2e-05

1.5e-05

1.2e-055.4e-051.5e-05

6e-063.2e-057e-06

3e-06

2.6e-05

5e-06

2.8e-051e-061.1e-05

1e-051.1e-052.1e-05

3e-05

2e-06

1.9e-053e-062.1e-05

3e-06

6e-06

2.5e-056.2e-052.2e-05

1.2e-053.9e-051.5e-05

5e-06

7.4e-057.4e-057.3e-05

7.4e-057.4e-057.3e-05

1.3e-053.3e-051.4e-05

1.3e-053.3e-051.4e-05

3.7e-055.9e-053.5e-05

1.7e-051.6e-05

2e-055.9e-051.9e-05

7e-062.4e-057e-06

7e-062.4e-057e-06

7e-061.3e-057e-06

8e-06

3e-06

2.4e-052.4e-05

2.4e-052.4e-05

2.4e-052.4e-05

3e-061.2e-052e-06

3e-061.2e-052e-06

3e-064e-062e-06

8e-06

7e-060.0001258e-06

7e-060.0001258e-06

7e-063e-058e-06

3.9e-05

5.6e-05

1.4e-05

1.4e-05

1.4e-05

1.4e-05

6.3e-056.4e-056.9e-05

6.3e-056.4e-056.9e-05

6.3e-056.4e-056.9e-05

6.3e-056.4e-056.9e-05

0.0021760.0002630.0212490.0234410.0023421.3e-05

0.0005030.0002380.0002310.000559

0.0001335.2e-050.000143

0.0001335.2e-050.000143

0.000370.0002380.0001790.000416

0.0002520.0002386.6e-050.000286

0.0001185.3e-050.00013

6e-05

0.0016730.0002630.0006870.0004020.0017831.3e-05

0.0002120.000245

0.0002120.000245

0.000930.0001650.0006870.0002620.0010391.3e-05

0.000233.6e-050.0002124.8e-050.0002451.3e-05

0.0002384.5e-050.0002245e-050.000268

4.3e-05

0.0002665.2e-050.0002517.9e-050.000299

0.0001963.2e-054.2e-050.000227

0.0004129.8e-050.000120.000365

0.0001945e-057.3e-050.000219

2.8e-05

0.0001281.9e-050.000146

9e-054.8e-050

0.0001192e-050.000134

0.0001192e-050.000134

0.0203240.022808

0.0203240.022808

0.0203240.022808

0.0001240.0002080.00013

1.4e-052.5e-051.6e-05

1.4e-052.5e-051.6e-05

1.4e-052.5e-051.6e-05

6e-059.7e-056.2e-05

6e-055.7e-056.2e-05

6e-055.7e-056.2e-05

4e-05

1.6e-05

2.4e-05

2.9e-056.9e-052.9e-05

1.5e-053.3e-051.7e-05

1.5e-053.3e-051.7e-05

1.4e-053.6e-051.2e-05

1.4e-053.6e-051.2e-05

2.1e-051.7e-052.3e-05

2.1e-051.7e-052.3e-05

1e-05

2.1e-057e-062.3e-05

0.0319660.0162680.0772070.048890.0578910.0323110.054955

0.0001610.0011430.000144

0.0001610.0011430.000144

1.5e-050.0001691.7e-05

1.5e-054.5e-051.7e-05

1.5e-054.5e-051.7e-05

5e-05

5e-05

7.4e-05

7.4e-05

6.8e-050.0002457.1e-05

8e-066.2e-051.1e-05

8e-066.2e-051.1e-05

8e-064.5e-059e-06

8e-064.5e-059e-06

1.3e-059e-06

1.3e-059e-06

1e-052.6e-057e-06

1e-052.6e-057e-06

4.1e-05

4.1e-05

1.7e-054.8e-052.1e-05

1.7e-054.8e-052.1e-05

1.2e-052.3e-051.4e-05

1.2e-052.3e-051.4e-05

5.8e-05

5.8e-05

5.8e-05

4e-050.0001191.9e-05

2.7e-057.5e-057e-06

2.7e-057.5e-057e-06

1.3e-054.4e-051.2e-05

1.3e-054.4e-051.2e-05

0.000335

6.3e-05

3.1e-05

3.2e-05

0.000196

2.8e-05

4.3e-05

5e-06

1.7e-05

2.3e-05

5e-05

3e-05

7.6e-05

3.3e-05

1.8e-05

2.5e-05

1.3e-053e-051.2e-05

1.3e-053e-051.2e-05

1.3e-053e-051.2e-05

2.5e-050.0001872.5e-05

5e-064.2e-054e-06

2.5e-05

5e-061.7e-054e-06

3.6e-05

3.6e-05

1.5e-052.8e-051.5e-05

1.5e-052.8e-051.5e-05

5e-068.1e-056e-06

5e-068.1e-056e-06

0.0150440.006250.0652340.0400190.0363950.0156460.050322

0.0150440.006250.0652340.0400190.0362060.0156460.050322

0.0101640.0043440.0575530.032660.0293490.0107950.046136

0.0101640.0043440.0575530.032660.0293490.0107950.046136

0.0014160.001589

2.2e-05

8.6e-05

5e-06

0.0051420.0013570.0034270.0086690.014010.005488

1.4e-05

2.1e-05

4.3e-05

2e-06

0.0408810.045879

0.0003130.0002794e-06

0.0009130.0014470.0011390.0195560.0009670.0009631.4e-05

1e-06

0.0022880.0006750.0013670.0044350.001430.002407

5e-06

5e-06

5e-06

0.0070170.007875

1.4e-05

0.0004730.000531

0.0008060.0002570.0004180.0004550.000842

0.0010150.0002950.0006130.0019050.001095

0.0005230.000587

1.4e-05

2e-06

5.2e-050.0002185.6e-05

5.2e-050.0002185.6e-05

5.2e-050.0002185.6e-05

0.0028250.0015410.0016280.003730.0007750.0027680.000634

0.000128

0.000128

0.0028250.0015410.0016280.003730.0006470.0027680.000634

0.0025680.0014660.0013440.003730.0025160.000634

0.0002577.5e-050.0002840.0006470.000252

0.001120.0003650.0044390.0036290.0025890.0011780.003538

0.001120.0003650.0044390.0036290.0025890.0011780.003538

0.0031520.003538

8.2e-050.0002630.0002957.9e-05

0.0002620.0001840.0002280.000261

0.000111

0.0002779.7e-050.0003430.0036290.0003350.000285

0.0001130.0001270.000132

0.0003570.000401

0.000298.4e-050.0003240.0006460.000308

9.6e-050.0004460.000113

0.0006170.0007780.0016880.0005861.4e-05

0.0001580.0005530.0013960.0001821.4e-05

0.0001580.0002090.001010.000182

0.0003440.000386

1.4e-05

0.0004590.0002250.0002920.000404

0.000153

0.0004590.0002250.0001390.000404

0.0001080.0002250.000116

0.0001080.0002250.000116

0.0001080.0002250.000116

0.0005320.000597

0.0005320.000597

0.0005320.000597

0.0001280.0003040.0004690.000125

0.0003040.000341

0.0003040.000341

0.0001280.0001280.000125

5.9e-053.4e-056e-05

6.9e-059.4e-056.5e-05

3e-050.0002962.2e-05

3e-059e-052.2e-05

3e-059e-052.2e-05

0.000206

0.000206

0.000189

0.000149

0.000149

0.000149

4e-05

4e-05

4e-05

3.5e-050.0004863.6e-05

3.5e-050.0004863.6e-05

3.5e-050.0004863.6e-05

3.8e-05

3.8e-05

1e-050.0001531e-05

3.5e-05

1e-055.3e-051e-05

4.1e-05

2.4e-05

8e-060.0001738e-06

8e-069.4e-058e-06

7.9e-05

9e-065.5e-058e-06

9e-065.5e-058e-06

8e-066.7e-051e-05

8e-066.7e-051e-05

7.7e-059.5e-056.5e-05

7.7e-059.5e-056.5e-05

7.7e-059.5e-056.5e-05

2.2e-054.3e-052.1e-05

2.2e-054.3e-052.1e-05

5.5e-055.2e-054.4e-05

5.5e-055.2e-054.4e-05

2e-058e-052.6e-05

2e-058e-052.6e-05

7e-063.9e-059e-06

7e-063.9e-059e-06

7e-063.9e-059e-06

1.3e-054.1e-051.7e-05

1.3e-054.1e-051.7e-05

1.3e-054.1e-051.7e-05

0.0147540.0095910.0076130.0068550.011510.0144360.004423

0.0147540.0095910.0076130.0068550.011510.0144360.004423

0.0147540.0095910.0076130.0068550.011510.0144360.004423

3.7e-05

3.7e-05

5e-065.5e-055e-06

5e-065.5e-055e-06

5.2e-05

5.2e-05

0.00012

4.3e-05

7.7e-05

0.0147490.0095910.0076130.0068550.0112460.0144310.004423

0.0147490.0095910.0076130.0068550.0112460.0144310.004423

0.0018750.0004270.004360.0020160.0081820.0019580.00021

0.0018750.0004270.004360.0020160.0081820.0019580.00021

9e-058.7e-050.000108

9e-058.7e-050.000108

1.6e-051.3e-051.1e-05

1.2e-053.3e-051.8e-05

1.1e-057e-062.2e-05

1e-053e-069e-06

5e-064e-061.2e-05

1.4e-051.2e-051.5e-05

2e-059e-061.7e-05

2e-066e-064e-06

0.0017540.0004270.004360.0020160.0079180.0018250.00021

8.2e-050.0001299.8e-05

8.2e-050.0001299.8e-05

0.0014010.001801

0.0002560.000287

0.0007610.000854

0.00023

0.0003840.00043

2.1e-056.1e-051.1e-05

2.1e-056.1e-051.1e-05

2.4e-050.0001512.5e-05

2.4e-054.5e-052.5e-05

7.8e-05

2.8e-05

1.9e-055.3e-052.8e-05

1.9e-055.3e-052.8e-05

7.4e-058.6e-057.4e-05

7.4e-058.6e-057.4e-05

2.8e-05

6e-06

2.2e-05

3.6e-050.000174.1e-05

9.2e-05

3.6e-057.8e-054.1e-05

0.0014190.001593

0.0014190.001593

0.0007680.000410.0004020.0020160.0003450.0007640.00021

1.4e-05

0.000109

1.4e-05

0.000750.000410.0004020.0020169.5e-050.0007450.000182

1.8e-057.1e-051.9e-05

7e-05

8.9e-050.0001199.9e-05

2.2e-05

4.7e-053.9e-055.1e-05

4.2e-055.8e-054.8e-05

7e-05

7e-05

0.0003220.000361

0.0003220.000361

1.2e-050.0001031.5e-05

1.2e-050.0001031.5e-05

2.6e-050.0001012.6e-05

2.6e-050.0001012.6e-05

1.6e-05

5e-06

4e-06

7e-06

2.7e-050.0001173e-05

2.7e-050.0001173e-05

1.3e-051.7e-058e-061.5e-05

1.3e-051.7e-058e-061.5e-05

0.0001790.0001980.000188

4.4e-051.9e-054.1e-05

2.3e-054.6e-052.7e-05

5.2e-056.8e-055.4e-05

2.1e-055.1e-052.5e-05

3.9e-051.4e-054.1e-05

0.0003370.000378

0.0003370.000378

0.000331

0.000182

0.000149

0.000117

0.000117

4.4e-050.000144.3e-05

4.4e-050.000144.3e-05

0.0001990.0002230.0004540.00021

0.0001990.0002230.0004540.00021

2.4e-050.0002022.1e-05

2.4e-050.0002022.1e-05

2.7e-055.9e-052.9e-05

2.7e-055.9e-052.9e-05

2.1e-057.8e-052.3e-05

2.1e-057.8e-052.3e-05

2.2e-050.000132.6e-05

7.2e-05

2.2e-055.8e-052.6e-05

0.000101

0.000101

0.0002560.000287

0.0002560.000287

4.7e-050.0001315.9e-05

4.7e-050.0001315.9e-05

1.9e-055.6e-051.4e-05

1.9e-055.6e-051.4e-05

1.9e-055.6e-051.4e-05

7.3e-05

7.3e-05

7.3e-05

1.2e-054.8e-051.1e-05

1.2e-054.8e-051.1e-05

1.2e-054.8e-051.1e-05

5.6e-050.0003094e-05

5.6e-050.0003094e-05

5.6e-050.0003094e-05

5.6e-050.0003094e-05

2.1e-054.1e-059e-06

2.1e-054.1e-059e-06

1.2e-056.3e-059e-06

1.2e-056.3e-059e-06

1e-065e-050

1e-065e-050

2.2e-056.7e-052.2e-05

2.2e-056.7e-052.2e-05

8.8e-05

8.8e-05

0.0003630.0005480.002016

0.0003630.0005480.002016

0.0003630.0005480.002016

0.0003630.0005480.002016

0.0003630.0005480.002016

0.0003630.0005480.002016

2.3e-050.000141.9e-05

2.3e-050.000141.9e-05

2.3e-050.000141.9e-05

1.5e-058.4e-051.1e-05

1.5e-058.4e-051.1e-05

1.5e-058.4e-051.1e-05

8e-065.6e-058e-06

8e-065.6e-058e-06

8e-065.6e-058e-06

0.0036860.0021390.00170.0044350.0033170.003540.000994

0.0036860.0021390.00170.0044350.0033170.003540.000994

0.0004610.000620.000382

6.9e-050.000135.6e-05

6.9e-050.000135.6e-05

6.9e-050.000135.6e-05

0.0003920.000490.000326

0.0003920.000490.000326

4.7e-05

5.2e-054.5e-055.4e-05

4e-055.4e-051.8e-05

4.7e-054.6e-053.7e-05

0.0001078.8e-057.4e-05

3.1e-053.4e-053e-05

5.5e-053.4e-055e-05

3.4e-05

1.7e-05

2.7e-05

6e-056.4e-056.3e-05

0.0032250.0021390.00170.0044350.0026970.0031580.000994

0.0032250.0021390.00170.0044350.0026970.0031580.000994

2.1e-059.5e-052.7e-05

1.3e-053.7e-051.9e-05

8e-065.8e-058e-06

0.0001147.5e-050.000107

0.0001147.5e-050.000107

0.0002680.0001220.0019150.0003090.0002527.3e-05

1.3e-051.3e-059e-06

1.3e-053.1e-051.2e-05

4.2e-05

3.4e-054.3e-052.7e-05

4e-06

0.0002080.0001220.0019150.0001760.0002047.3e-05

0.0028220.0020170.00170.002520.0022180.0027720.000921

0.0028220.0020170.00170.002520.0022180.0027720.000921

0.3669020.4499090.335950.4308470.2293430.3622550.413353

0.1463950.1499390.1229640.2376010.09979200000000010.1445210.112515

0.0008960.0024920.0024070.0029230.000470.0007940.001787

0.0008960.0024920.0024070.0029230.000470.0007940.001787

2.3e-051e-052.9e-05

2.3e-051e-052.9e-05

1.6e-056.2e-055e-06

1.6e-056.2e-055e-06

0.0006650.0024920.0024070.0029230.0001710.0005850.001787

0.0015920.001787

3.9e-051.6e-051.6e-05

2.1e-051e-051.3e-05

1.9e-054.7e-053.7e-05

0.0005860.0024920.0008150.0029239.8e-050.000519

1.1e-052e-051.3e-05

1.1e-052e-051.3e-05

7.6e-058.3e-057.7e-05

3.3e-056.2e-054e-05

8e-06

9e-066e-069e-06

3.4e-057e-062.8e-05

7.6e-053e-055.1e-05

3.5e-059e-061.2e-05

4.1e-052.1e-053.9e-05

6e-062.4e-059e-06

6e-062.4e-059e-06

4e-062.5e-055e-06

4e-062e-065e-06

6e-06

1.7e-05

1.9e-054.5e-052e-05

1.9e-054.5e-052e-05

0.0252390.0109880.0114660.0092740.0219540.0250180.004107

0.000165

0.000165

0.000165

0.000131

0.000131

0.000131

0.0248990.0109880.0114660.0092740.0211620.0247290.004107

7.6e-05

7.6e-05

0.0001180.0001259.2e-052e-05

0.0001180.0001259.2e-052e-05

0.0001510.0002220.000124

8.7e-050.0001196.3e-05

6.4e-050.0001036.1e-05

0.000103

0.000103

0.0001570.0001340.000146

0.0001570.0001340.000146

0.0244730.0109880.0114660.0092740.0205020.0243670.004087

0.0239980.0109880.0091660.0092740.0176520.0241230.004087

0.00028.5e-05

0.00230.002581

0.0002750.0001060.000244

7.8e-05

0.0002880.0003650.000246

5.7e-054.7e-054.2e-05

5.7e-054.7e-054.2e-05

0.0001185.5e-059.2e-05

0.0001185.5e-059.2e-05

8.3e-057.7e-057.9e-05

8.3e-057.7e-057.9e-05

5.5e-05

5.5e-05

3e-050.0001313.3e-05

6e-065.2e-051e-05

1e-053.1e-059e-06

1.4e-054.8e-051.4e-05

5.2e-050.0001314.3e-05

5.2e-050.0001314.3e-05

5.2e-056.8e-054.3e-05

6.3e-05

0.0009765.7e-050.0012480.0032720.0008081.7e-05

0.000101

0.000101

0.000101

0.0007045.7e-050.0012480.0021860.0005881.7e-05

0.0003595.7e-050.0012480.0018660.0003311.7e-05

0.0009780.001098

0.000177

0.0003595.7e-050.000270.0002450.0003311.4e-05

0.000187

3e-06

0.000159

6.7e-051.3e-051e-06

6.7e-051.3e-051e-06

0.000104

0.000104

0.0002530.0001580.000234

0.0002530.0001580.000234

3.7e-05

3.7e-05

2.5e-058e-062.2e-05

2.5e-058e-062.2e-05

0.0001330.0002510.000102

0.0001330.0002510.000102

5e-052e-064.4e-05

0.000123

1e-06

8.3e-050.0001255.8e-05

2.6e-059.2e-052.3e-05

2.6e-059.2e-052.3e-05

2.6e-059.2e-052.3e-05

2.1e-050.0001561.4e-05

2.1e-050.0001561.4e-05

4.8e-05

5.7e-05

2.1e-055.1e-051.4e-05

9.2e-050.0004868.1e-05

0.000109

0.000109

6.6e-050.0001696.5e-05

2.2e-055.7e-052.2e-05

1.9e-055.1e-051.9e-05

2.5e-056.1e-052.4e-05

2.6e-056.1e-051.6e-05

2.6e-056.1e-051.6e-05

0.000147

0.000147

0.0001460.0135890.0012540.000130.015017

0.0001460.000520.00013

5.4e-050.0001955.4e-05

5.4e-050.0001955.4e-05

3.2e-050.0001082.9e-05

3.2e-050.0001082.9e-05

2.7e-050.0001022.3e-05

2.7e-050.0001022.3e-05

3.3e-050.0001152.4e-05

3.3e-050.0001152.4e-05

0.0002080.000449

0.000215

0.000215

0.0002080.000234

0.0002080.000234

0.000285

0.000285

0.000154

0.000131

0.0133810.015017

0.0133810.015017

0.0133810.015017

0.0268890.0297740.0178680.1292340.0132790.0286350.037384

0.000118

0.000118

0.000118

0.00018

0.00018

0.00018

0.0003134e-050.0002130.0002450.0002880.000151

5.4e-05

5.4e-05

0.0003134e-050.0002130.000130.0002880.000151

4.2e-051.8e-054.4e-05

1e-05

2.3e-05

1.8e-054e-053.8e-051.7e-050.000151

0.0002530.0002134.1e-050.000227

6.1e-05

6.1e-05

0.0013440.0002570.0005550.0005340.0012310.000552

0.0003950.0001920.000447

0.0001940.0001430.000278

2.3e-056e-062.5e-05

2.8e-057e-062.5e-05

0.000152.1e-050.000119

1.5e-05

0.0002390.0001460.000181

2e-05

9e-05

0.0002393.6e-050.000181

0.000710.0002570.0005550.0001960.0006030.000552

0.0002880.0002298.9e-050.0002334.6e-05

0.0004220.0002570.0003265.4e-050.000370.000506

5.3e-05

0.0005410.0005280.000393

0.0001130.0002427.9e-05

6.6e-05

5.7e-053.3e-053.1e-05

5e-05

4.5e-05

5.6e-054.8e-054.8e-05

5.9e-05

5.9e-05

9.3e-050.0001037.1e-05

5.4e-054.1e-054.3e-05

3.4e-05

3.9e-052.8e-052.8e-05

5.7e-054.3e-053.7e-05

5.7e-054.3e-053.7e-05

3.7e-052e-062.3e-05

3.7e-052e-062.3e-05

0.0002417.9e-050.000183

0.0002417.9e-050.000183

0.0203650.0279640.0137870.1269150.0090710.0227850.034707

0.0018970.0001680.0006720.0007910.0018480.000165

2.9e-054.5e-052.6e-05

0.0011820.0001680.0003830.0006040.001175

4.1e-055.8e-053.3e-05

0.0006450.0002898.4e-050.0006140.000165

0.0007040.0001020.0001130.000731

0.0007040.0001020.0001130.000731

0.0003860.0003310.0007670.002520.0003050.0032890.001068

0.0003065.1e-050.000284.9e-050.000323

8e-050.000280.0004870.002520.0002560.0029660.001068

0.0038710.0188560.0032130.1068550.0005050.0032420.023895

0.0014730.0004310.0006530.0247980.0002650.0007540.021497

0.0009670.0024110.0014420.0257064.5e-050.001016

0.0009330.0156280.0004810.0319560.0001420.000956

0.0004980.0003860.0006370.0243955.3e-050.0005160.002398

0.0013970.0007520.000450.0006230.0014470.000475

4.5e-05

0.0005590.0006020.000451.8e-050.0005910.000361

0.000207

0.00011

0.000154

0.0008380.000158.9e-050.0008560.000114

0

1.6e-051.8e-05

1.6e-051.8e-05

0.0002420.00032

0.0002420.000272

4.8e-05

0.0052270.0012990.0020880.0020160.0009170.0053290.000847

0.0001

9e-06

6e-060.000174

1.1e-05

0.0007050.0002830.0003962.4e-050.0006860.000384

3e-06

1.2e-05

1.5e-05

0.0007849.7e-050.0004240.0002620.000831.6e-05

0.0016140.000490.0005610.0020160.0002410.001571

1.3e-05

5e-06

0.0007660.0001020.0002174.7e-050.000825.4e-05

0.0002140.00024

0.0013580.0003270.0002762.1e-050.0014220.000119

8e-06

0.0028170.0006040.0032220.0014110.0028490.002119

0.001120.0001850.0003290.0002720.0011495.2e-05

0.0005460.000612

0.0002990.000335

0.000167

0.0012310.001382

0.0003430.000385

0.0016970.0004190.0004740.0002520.00177.3e-05

0.0002850.00032

0.0002850.00032

0.000210.000235

0.000210.000235

0.0002470.000278

0.0002470.000278

3.8e-05

3.8e-05

0.000131

0.000131

3.3e-05

3.3e-05

4e-05

4e-05

0.000135

0.000135

6e-05

6e-05

2.5e-051.7e-052.5e-05

2.5e-051.7e-052.5e-05

4e-050.0001214.7e-05

1e-061e-061e-06

3e-05

2e-061e-063e-06

6.3e-05

2.9e-051.1e-053.3e-05

8e-061.5e-051e-05

2.5e-051.2e-052.7e-05

2.5e-051.2e-052.7e-05

4.4e-05

4.4e-05

0.0006270.000704

0.0006270.000704

0.0015010.0001980.0005020.0014230.00147

0.0015010.0001980.0005020.0014230.00147

0.0015060.0051760.001040.009980.0002780.0015370.005719

0.0009780.0001040.0001640.001013

0.000213

7.8e-050.000125

0.0005280.0050720.001040.009983.6e-050.0005240.005381

0.0009530.0004780.0002220.0055440.0002170.0009260.000419

0.000117

6.3e-058e-066.4e-052e-06

0.0003820.0001054.3e-050.0004010.000237

4e-06

7e-06

0.000136

8e-050.0002290.0055447e-060.0001021.2e-05

0.0004280.0001440.0002223.1e-050.0003593.2e-05

0.001990.0001330.0016720.0016520.0017060.001103

0.0009167.4e-050.0009060.0009240.0007746.7e-05

0.000141

0.0003854e-050.0002370.0001820.0003132e-05

0.0001083.4e-054.6e-059e-051.6e-05

8.5e-05

0.0003940.0003343e-050.000356

0.0003350.000376

3.1e-05

3.7e-05

2.9e-052.7e-051.5e-05

0.0010415.9e-050.0007660.0006740.0009040.001036

0.0004470.0003060.000260.0003797e-05

0.000142.8e-052e-060.000125

0.000220.000247

2.5e-053.1e-051.5e-051.8e-056e-06

0.0003660.000240.0001280.0003110.000955

6.3e-052.2e-057.1e-055e-06

2.1e-053.6e-051.6e-05

2.1e-053.6e-051.6e-05

1.2e-051.8e-051.2e-05

1.2e-051.8e-051.2e-05

0.0023360.001380.0016410.0023190.0009510.0022320.000871

0.0001292.5e-052.2e-050.0001284.3e-05

9e-061.8e-051.4e-054.3e-05

0.000122.5e-054e-060.000114

0.0008240.0012090.0008730.0023190.0004540.0008350.000785

7.4e-05

8.9e-05

6.6e-05

2e-06

5.8e-05

0.0008190.0011920.0008730.0023195.8e-050.000830.000709

0

6.3e-05

4.2e-05

1e-061.7e-0501e-06

4e-062e-064e-067.6e-05

0.000194

0.000194

0.0013830.0001460.0007680.0002810.0012694.3e-05

0.0003080.0002694.5e-050.000297

3e-06

2.7e-05

0.00013

03e-06

7.5e-053.7e-051e-057.1e-058e-06

1.3e-05

0.0006690.0001090.0002162.7e-050.000595

2.9e-05

3e-06

3e-06

0.0003310.0002832.3e-050.000306

0.0002950.0010680.0063920.0092740.0005280.0003470.000819

0.000116

5.7e-05

5.7e-05

5.9e-05

5.9e-05

0.0002050.0010680.0003740.002520.0001180.0002930.000819

1.3e-05

1.3e-05

0.0002050.0010680.0003740.002520.0001050.0002930.000819

0.0001780.0010680.0003740.002526.8e-050.0002670.000819

7e-06

1e-063e-062e-06

3e-061e-063e-06

1.7e-05

2.3e-059e-062.1e-05

9e-050.0060180.0067540.0002945.4e-05

4.1e-050.0060180.0067540.0001539e-06

2e-058.4e-05

2.1e-050.0060180.0067546.9e-059e-06

1.1e-05

1.1e-05

01.8e-051e-06

07e-060

01.1e-051e-06

3.2e-054.4e-051.7e-05

3.2e-052.6e-051.7e-05

1.8e-05

1.7e-056.8e-052.7e-05

1.7e-056.8e-052.7e-05

0.0001180.0002490.0001194e-06

0.0001180.0002490.0001194e-06

1e-059.2e-051.2e-05

1e-053e-051.2e-05

3.5e-05

2.7e-05

9.7e-050.00019.7e-054e-06

9.7e-050.00019.7e-054e-06

1.1e-055.7e-051e-05

1.1e-055.7e-051e-05

1.5e-055.3e-051.2e-05

1.5e-055.3e-051.2e-05

1.5e-055.3e-051.2e-05

1.5e-055.3e-051.2e-05

0.000101

0.000101

0.000101

0.000101

0.0001610.0006620.0002410.0021170.0002060.0001460.000451

0.0001590.0006620.0002410.0021170.0001570.0001450.000451

0.0001590.0006620.0002410.0021170.0001320.0001450.000451

1.1e-053.6e-051.2e-05

1.6e-05

2.4e-05

0.0001480.0006620.0002410.0021173e-050.0001330.000451

2.6e-05

2.5e-05

2.5e-05

2e-064.9e-051e-06

1.8e-05

1.8e-05

2e-063.1e-051e-06

2.3e-05

2e-068e-061e-06

0.0036360.0025820.0012390.0028230.0005980.0034960.001112

0.0036360.0025820.0012390.0028230.0005980.0034960.001112

4.4e-058.5e-053.1e-05

4.4e-058.5e-053.1e-05

0.000180.0001110.000137

0.000180.0001110.000137

0.0034120.0025820.0012390.0028230.0004020.0033280.001112

2.1e-05

0.0003387.2e-050.000294.5e-050.0003144.3e-05

0.0002380.000268

9.4e-05

0.0025890.0024110.0007110.0028230.0002210.0025630.000689

0.0004859.9e-052.1e-050.0004510.000112

0.0136030.0073010.0072330.0045360.009670.0133660.000172

0.0136030.0073010.0072330.0045360.009670.0133660.000172

6.8e-05

6.8e-05

2.3e-056.2e-051.8e-05

2.3e-052.8e-051.8e-05

3.4e-05

1.6e-055e-052.8e-051.8e-05

1.5e-05

3e-061.2e-051.6e-054e-06

1.3e-052.3e-051.2e-051.4e-05

0.0135640.0073010.0072330.0045360.009490.013320.000154

5e-06

1.1e-05

5e-062e-064e-066e-06

8e-063e-068e-06

2.7e-05

9e-067e-061.5e-054e-06

5.4e-051.4e-054.7e-05

1.6e-053e-061.5e-051.4e-05

1.2e-056e-061.1e-058e-06

8e-06

1.4e-051.4e-05

2e-056e-06

7e-06

1.2e-053e-061.6e-05

9e-066e-062e-068e-062.8e-05

2.8e-05

1.4e-05

0.0134020.0072950.0072330.0045360.0092630.013174

2.5e-05

3.8e-05

2.1e-051.9e-051.1e-05

1.6e-051e-051.1e-05

3.5e-05

0.0001520.0017570.0027990.000139

0.0001520.0017570.0027990.000139

0.000178

0.000178

0.0001140.0003438.9e-05

3.1e-050.0001152.7e-05

4.5e-05

2.3e-054e-051.9e-05

6e-055.5e-054.3e-05

8.8e-05

0.0017570.002093

0.0017570.001972

0.000121

9.2e-05

9.2e-05

7.8e-05

7.8e-05

3.8e-051.5e-055e-05

3.8e-051.5e-055e-05

8.8e-05

8.8e-05

8.8e-05

8.8e-05

0.0616210.0858070.0526530.0675410.0358960.0592560.046214

0.0606240.0851790.0490120.0632060.0333330.0582280.04555

0.0355680.0716520.031740.0392140.0224730.0347180.035184

0.0002750.000308

0.0355680.0716520.0314650.0392140.0221650.0347180.035184

0.0250560.0135270.0172720.0239920.0108240.023510.010366

0.00018

8e-06

0.001010.001134

5.2e-05

4.3e-05

0.0009360.0001460.000247.7e-050.0008390.000176

5e-05

0.0005997.3e-050.000240.0001330.000515

0.000108

0.0005690.000360.0004235.6e-050.0005626.2e-05

0.000108

3.9e-05

0.0020140.0005230.0006430.0003780.002039

0.0003463.8e-050.00033.2e-050.000328

0.000128

0.00011

2e-05

4.8e-05

0.000103

0.000181

0.0001891.1e-050.0001779.2e-05

4.4e-05

3.9e-05

8e-06

1.9e-05

1.4e-05

5e-05

0.000126

0.0006550.0001790.0001310.0006250.000201

0.0004426.9e-050.0003847.1e-050.000421

0.0001270.0002517e-060.0001290.000517

1e-05

9e-05

4.2e-05

3.7e-053.1e-050.0035030.0039314.5e-054.2e-05

9e-06

3.7e-05

0.014150.0096290.0072920.0115930.0044220.0142840.005675

0.0017510.0003210.0005120.0004880.0017690.000418

0.0001492e-050.000137

0.00013

0.0003269e-050.0003262.7e-050.0003240.000488

0.0001181.3e-050.000115e-05

0.0002213.4e-051.4e-050.000204

0.0013450.0002080.000321

1.4e-05

4.5e-05

7e-06

0.0009380.001053

6.5e-05

0.0007730.0015360.0004190.0084680.0001120.0007110.002687

0.000127

3.3e-05

0.0004530.000508

0.0003093.9e-050.0002685e-060.000294

3.6e-05

3.6e-05

0.0009970.0006280.0036410.0043350.0025630.0010280.000664

0.0004290.0002890.000260.0020160.0003790.0004210.000123

2.8e-051.2e-052.7e-05

5e-065e-063e-06

8e-064e-061e-05

2.2e-05

2e-05

6e-06

5e-06

0.0003880.0002890.000260.0020160.0003050.0003810.000123

0.0005580.0003390.0033810.0023190.0020550.0005970.000541

0.0001290.0001239.6e-050.00014

0.0002250.000252

0.000196

2e-059e-050.0020660.0023197e-061.7e-05

0.0002470.000277

0.0003030.0001260.0004420.000324

0.000226

0.0002530.000284

0.0003690.000414

0.0001062.7e-050.000116

0.0002210.000248

0.000127

1e-050.0001291e-05

5.4e-05

1e-051.6e-051e-05

5.9e-05

0.0012650.0011680.000460.0023190.001120.0012250.000783

0.0010440.0011680.000460.0023190.000760.0010180.000783

0.000111

2.5e-05

4.2e-05

4.4e-05

0.0004580.0011680.000460.0023190.000220.0004490.000721

0.0004580.0011680.000460.0023190.000220.0004490.000721

0.0001490.0001580.000155

9e-063e-061.7e-05

1e-0601e-06

2.2e-05

9e-06

1.3e-057e-061.1e-05

00

1e-058e-069e-06

8.4e-051.7e-058.2e-05

2.3e-051.5e-052.4e-05

7.5e-05

9e-062e-061.1e-05

6.6e-050.0001325.9e-054e-05

0

0

5.5e-05

6.6e-051.7e-055.9e-054e-05

6e-05

0.0003710.0001390.0003552.2e-05

0.000157.2e-050.000152

0.0002216.7e-050.0002032.2e-05

0.0002210.000360.000207

0.0001375.7e-050.000126

0.0001375.7e-050.000126

8.4e-055.2e-058.1e-05

8.4e-055.2e-058.1e-05

8.7e-05

8.7e-05

0.000164

5.5e-05

8e-05

2.9e-05

0.0113830.008040.0064110.007560.0081020.011030.004648

2.7e-058.9e-052.4e-05

2.7e-058.9e-052.4e-05

4.3e-05

2.7e-054.6e-052.4e-05

1.3e-054.3e-051e-05

1.3e-054.3e-051e-05

1.3e-054.3e-051e-05

4.6e-05

4.6e-05

4.6e-05

0.0108710.008040.0064110.007560.0072530.0106080.004634

2.8e-055.2e-052.4e-05

0

1.6e-051.5e-051.5e-05

1e-06

1e-06

3e-063e-063e-06

2.2e-05

9e-061e-056e-06

1.5e-057.4e-051e-05

1.5e-057.4e-051e-05

0.0031210.0030450.0022780.0035280.0021750.0030330.00194

7.7e-053.5e-056.4e-05

0.000128

0.0029930.0030450.0022780.0035280.001840.0029260.00194

4.5e-05

3.8e-05

4.4e-05

2.6e-05

5.1e-051.9e-054.3e-05

6e-05

6e-05

7.6e-05

7.6e-05

0.0077070.0049950.0041330.0040320.0048160.0075410.002694

0.0077010.0049950.0041330.0040320.0047250.0075370.002694

6e-069.1e-054e-06

3e-050.0001123.4e-05

3e-050.0001123.4e-05

1.4e-055.3e-051.6e-05

1.6e-055.9e-051.8e-05

3.1e-050.0002111.9e-05

3.1e-050.0002111.9e-05

1.3e-052e-068e-06

3.8e-05

2.2e-05

5e-068e-063e-06

7e-06

6e-06

9e-06

1.3e-054.7e-058e-06

3.4e-05

3.8e-05

0.00018e-057.8e-05

0.00018e-057.8e-05

0.00018e-057.8e-05

0.0003110.0002680.0002571.4e-05

0.0003110.0002680.0002571.4e-05

1.2e-051.2e-05

6e-064e-065e-06

1e-051.6e-059e-06

2.2e-052.9e-051.9e-05

1.6e-053.9e-051.2e-05

1e-063e-062e-06

02e-060

9e-062.8e-058e-06

2.8e-051.1e-052.1e-05

1.9e-051.5e-052.5e-05

1.1e-0501.5e-05

3.8e-053e-051.8e-05

1.6e-052.2e-051.2e-05

4e-061e-065e-06

9.1e-053.6e-057e-051.4e-05

1.1e-051e-059e-06

1.7e-052.2e-051.5e-05

0.000153

0.000153

7.3e-05

7.3e-05

8e-05

8e-05

0.0017870.0005440.0082020.0016361.8e-05

0.0003310.00050.0003154e-06

0.0002630.0003870.0002474e-06

0.0002560.0003640.0002414e-06

2.7e-055.1e-052.5e-05

1.1e-051.1e-05

3.4e-054.3e-052.8e-05

3.8e-055.7e-05

4.2e-052.8e-054e-05

5.3e-054.2e-053.7e-054e-06

3.1e-05

5.4e-05

1.8e-052.9e-051.8e-05

1.6e-054.8e-051.3e-05

1.4e-053.8e-051.1e-05

3e-061e-06

7e-062.3e-056e-06

7e-062.3e-056e-06

3.1e-053.8e-053.7e-05

3.1e-053.8e-053.7e-05

3.1e-053.8e-053.7e-05

3.7e-057.5e-053.1e-05

3.7e-057.5e-053.1e-05

4.5e-05

3.7e-053e-053.1e-05

7.1e-057.4e-056e-05

7.1e-057.4e-056e-05

7.1e-057.4e-056e-05

7.1e-057.4e-056e-05

4e-060.0001011e-06

4e-060.0001011e-06

4.6e-05

4.6e-05

4e-065.5e-051e-06

4e-065.5e-051e-06

3.9e-050.0001742.7e-05

2.5e-050.0001322e-05

1e-055.6e-059e-06

1e-055.6e-059e-06

8e-064.4e-056e-06

8e-064.4e-056e-06

7e-063.2e-055e-06

7e-063.2e-055e-06

1.4e-054.2e-057e-06

1.4e-054.2e-057e-06

1.4e-054.2e-057e-06

0.0001870.0003680.000153

0.0001340.0002090.00011

5.7e-056.1e-055e-05

5.7e-056.1e-055e-05

9e-064.2e-058e-06

9e-064.2e-058e-06

1.2e-055.9e-056e-06

1.2e-055.9e-056e-06

5.6e-054.7e-054.6e-05

5.6e-054.7e-054.6e-05

5.3e-050.0001594.3e-05

1.3e-054.6e-051.2e-05

1.3e-054.6e-051.2e-05

7e-063.4e-051e-05

7e-063.4e-051e-05

7e-063.3e-057e-06

7e-063.3e-057e-06

2.6e-054.6e-051.4e-05

2.6e-054.6e-051.4e-05

0.0003860.0009520.00037

0.0001460.0002220.000145

0.0001460.0002220.000145

6.7e-050.0001147.1e-05

3.2e-054.2e-053e-05

4.7e-056.6e-054.4e-05

0.0001130.0001840.00011

2.8e-054.9e-053.4e-05

2.8e-054.9e-053.4e-05

8.5e-050.0001357.6e-05

2.9e-053.3e-052.4e-05

2.7e-053.6e-052.3e-05

3.4e-05

2.9e-053.2e-052.9e-05

9.2e-05

9.2e-05

9.2e-05

8.3e-05

8.3e-05

8.3e-05

3.7e-059.3e-053.3e-05

4.1e-05

4.1e-05

3.7e-055.2e-053.3e-05

3.7e-055.2e-053.3e-05

5.2e-059.3e-054.3e-05

5.2e-059.3e-054.3e-05

5.2e-059.3e-054.3e-05

3.8e-050.0001853.9e-05

6.2e-05

6.2e-05

3.8e-050.0001233.9e-05

3.8e-050.0001233.9e-05

0.0004560.0005440.0055190.000454

4.1e-050.0001142.2e-05

4.1e-050.0001142.2e-05

9e-063.5e-058e-06

3.2e-057.9e-051.4e-05

0.0004150.0005440.0054050.000432

0.0004150.0005440.0054050.000432

0.0004150.0005440.0054050.000432

3e-052.2e-051.6e-05

3e-052.2e-051.6e-05

3e-052.2e-051.6e-05

3e-052.2e-051.6e-05

0.0002830.0004920.000241.4e-05

0.0002230.0002920.0001881.4e-05

5.1e-05

5.1e-05

0.0002230.0002410.0001881.4e-05

1.2e-051.1e-051.5e-05

3.2e-056e-052.6e-05

3.1e-05

1.6e-051.8e-059e-06

1.1e-05

2.9e-052e-051.7e-051.4e-05

3.3e-053.3e-053e-05

1.7e-051.1e-051.3e-05

4.8e-052.4e-053.9e-05

3.6e-052.2e-053.9e-05

6e-050.00025.2e-05

0.000115

6.2e-05

5.3e-05

6e-058.5e-055.2e-05

2.3e-054.8e-052e-05

3.7e-053.7e-053.2e-05

0.00018.8e-059e-05

0.00018.8e-059e-05

0.00018.8e-059e-05

0.00018.8e-059e-05

7.1e-053.4e-056.3e-05

2.1e-051.4e-052e-05

8e-064e-057e-06

0.0477480.0828320.112740.0345770.0512690.0479430.115573

0.0003469.9e-050.0003470.0005760.000324

0.0003469.9e-050.0003470.0005760.000324

0.0003469.9e-050.0003470.0005760.000324

0.0003469.9e-050.0003470.0005760.000324

0.0001

3.6e-05

3.6e-05

3.6e-05

6.4e-05

6.4e-05

6.4e-05

0.0037470.0005370.0675160.0109640.0037860.073596

0.000234

0.000234

0.000234

0.0021180.0002880.002140.003650.0021380.001633

0.0001373e-059.4e-050.000117

7e-053e-058.1e-058.2e-05

6.7e-051.3e-053.5e-05

0.000172

0.000172

0.000157

0.000157

0.0001090.0005990.0001170.0001140.000672

8e-050.0005997.6e-058.2e-050.000672

2.9e-054.1e-053.2e-05

3.2e-050.0001863.5e-05

9.2e-05

1.1e-054.3e-051.4e-05

2.1e-055.1e-052.1e-05

1.4e-05

1.4e-05

0.0004133.4e-050.0002440.0004264.6e-05

0.0002563.4e-050.0001230.0002674.6e-05

0.0001570.0001210.000159

0.000117

0.000117

5.2e-052.7e-055e-05

5.2e-052.7e-055e-05

0.00013

0.00013

0.000177

0.000177

0.000450.0001270.0002770.0004130.0004521.4e-05

9.8e-055.7e-057.3e-050.000101

0.0003527e-050.0002770.0002510.0003511.4e-05

8.9e-05

0.000136

0.000136

0.0001050.0001180.000116

0.0001050.0001180.000116

0.0004910.000551

0.0004910.000551

3.6e-054.2e-05

3.6e-054.2e-05

0.0002850.000319

0.0002850.000319

0.0002410.00027

0.0002410.00027

7.4e-05

7.4e-05

0.0002620.0001530.000245

0.0002620.0001530.000245

7e-06

7e-06

0.00027

0.000151

0.000119

3.1e-05

3.1e-05

0.0001665.1e-050.0001290.000175

0.0001665.1e-050.0001290.000175

0.000163

0.000163

0.0003564.6e-050.0002470.0001840.0003660.000291

0.0001374.6e-055.5e-050.0001381.4e-05

0.0002470.000277

0.0002190.0001290.000228

7e-06

7e-06

0.0016290.0002490.0653760.0073140.0016480.071729

0.0013230.0002490.0006270.0061830.001336

0.0013230.0002490.0006270.0061830.001336

3.1e-050.0005380.0006032.7e-05

3.1e-050.0005380.0006032.7e-05

0.0002750.0002960.0005280.000285

0.0002750.0002960.0005280.000285

0.0639150.071729

0.0639150.071729

0.0016070.000130.0015690.0046370.0034910.0016365.6e-05

0.000140.000220.0009870.00015

0.000481

5.5e-05

8.9e-05

8.7e-05

6e-05

9.4e-05

9.6e-05

0.000220.000247

0.000220.000247

0.000106

5.4e-05

5.2e-05

0.000140.0001530.00015

0.000149.2e-050.00015

6.1e-05

0.0014670.000130.0013490.0046370.0025040.0014865.6e-05

0.0004028.1e-050.0010660.0024190.0009250.000407

5.5e-05

0.0005670.000637

2.6e-05

0.0004028.1e-050.0004990.0024199.1e-050.000407

4.5e-05

7.1e-05

0.0004340.0002830.0022180.0004930.0004392.8e-05

9.4e-05

6.7e-05

0.0002260.0002830.0022188.9e-050.000218

1.4e-05

7e-05

1.4e-05

0.0002080.0001160.000221

5.7e-05

0.0001680.0001860.0001571.4e-05

5.6e-05

2.9e-05

9e-052.8e-057.6e-051.4e-05

7.8e-057.3e-058.1e-05

1.8e-054.7e-054.5e-05

1.8e-054.7e-054.5e-05

0.0004454.9e-050.0005050.0004381.4e-05

0.0001589e-050.000154

6.1e-05

0.000108

5.4e-05

4.5e-05

3.6e-05

4.5e-05

0.0001664.9e-052.6e-050.000169

0.0001214e-050.0001151.4e-05

8.5e-05

8.5e-05

0.000152

0.000152

0.000111

0.000111

0.0014531.8e-050.001630.0013996e-06

0.0011231.8e-050.001310.0011126e-06

0.0002751.8e-050.0001640.000289

0.0001291.8e-054.2e-050.000135

1.6e-05

0.0001465.7e-050.000154

4.9e-05

0.00014

0.00014

5e-064.7e-052e-06

5e-064.7e-052e-06

0.0002060.0001790.000227

0.0002060.0001790.000227

0.0003080.0001750.000286

4.7e-05

0.000168.1e-050.00013

0.0001484.7e-050.000156

0.000115

0.000115

0.0002390.0002590.000236e-06

0.0001840.000190.0001676e-06

5.5e-056.9e-056.3e-05

0.0001

0.0001

9e-050.0001317.8e-05

9e-050.0001317.8e-05

0.000330.000320.000287

6.6e-052.4e-054.5e-05

6.6e-052.4e-054.5e-05

6.5e-053.8e-056.1e-05

2.9e-051.4e-052.9e-05

3.6e-052.4e-053.2e-05

5.6e-053.9e-054.4e-05

5.6e-053.9e-054.4e-05

6.5e-05

6.5e-05

2e-066.3e-052e-06

4.9e-05

1e-05

2e-064e-062e-06

8.4e-056.6e-058.3e-05

8.4e-053.3e-058.3e-05

3.3e-05

5.7e-052.5e-055.2e-05

5.7e-052.5e-055.2e-05

0.0015614.6e-050.0014310.0022780.0017260.000103

0.0015614.6e-050.0014310.0022780.0017260.000103

0.0006244.6e-050.0002130.0004860.0007954.7e-05

0.000129

0.0002270.0002137.6e-050.0002524.7e-05

0.0002560.0001290.000257

0.0001414.6e-050.0001520.000286

0.0001410.0002210.000134

0.0001410.0002210.000134

0.0003760.000422

0.0003760.000422

0.0003880.0005160.0008390.00045.6e-05

0.0002350.000264

5.6e-05

0.000139

0.0003880.0002810.0002260.0004

0.00021

0.0004080.0003260.000310.000397

0.0004080.0003260.000310.000397

0.0206820.075390.0324750.0228830.0185920.0204130.038416

0.0051880.0380630.0151160.0021560.0052320.013413

3e-061.1e-052e-06

3e-061.1e-052e-06

0.0051850.0380630.0151160.0021450.005230.013413

0.0002130.0001966.9e-050.000227

1.5e-055e-061e-06

0.0005720.0004940.0003030.000250.0005910.000191

0.0001813.4e-056e-06

0.000260.0002456.3e-050.000292

3.4e-053.5e-051.1e-05

0.0001120.0177470.0001560.0001185e-06

0.0007970.0005390.0004520.0002810.0008285e-06

0.0001226.5e-050.0001270.000151

0.0002066.4e-050.000218

3.3e-050.0011110.000991.6e-053.4e-05

0.0002530.0002385.6e-050.000282

0.000320.0002180.0001580.0003572.1e-05

0.000340.0003197.2e-050.000378

0.0001380.0001160.0001430.000503

5.1e-057e-063e-06

9.7e-05

0.0010790.0181720.000760.0003550.001122

0.0111660.012531

0.0001337.3e-050.0001392e-06

4e-06

0.0003260.0002290.0001730.000353

4e-05

4e-05

4e-05

1.5e-054.1e-059e-06

1.5e-054.1e-059e-06

1.5e-054.1e-059e-06

0.0154790.0373270.0173590.0228830.0163550.0151720.025003

0.0022160.0014910.0013160.0024190.0012110.0021640.000834

0.0022070.0014910.0013160.0024190.0011570.0021590.000834

1e-066e-061e-06

2e-062e-06

0

3e-06

2e-06

6e-068e-062e-06

3.5e-05

3.2e-051e-053.7e-05

1e-06

1e-062e-061e-06

1e-064e-064e-06

2.8e-052e-063e-05

2e-061e-062e-06

1e-064e-05

1e-061.6e-05

2e-05

04e-06

0.013230.0358360.0160430.0204640.0150940.0129710.024169

1e-065e-060

1.2e-050.0002520.0002831.2e-053.4e-05

0.0132170.0358360.0157910.0204640.0148060.0129590.024135

8.9e-050.0002847.7e-05

8.9e-050.0002847.7e-05

4.5e-050.000154e-05

2.9e-059.3e-052.8e-05

1.6e-055.7e-051.2e-05

4.4e-050.0001343.7e-05

3.6e-054.6e-053.3e-05

8e-068.8e-054e-06

0.0178030.0066120.0091660.0070570.0129050.0181410.003348

0.0008020.0002210.0003880.000843e-05

0.0002240.0001070.000242

0.0002240.0001070.000242

0.0003450.0002210.0001570.0003521.6e-05

0.0003450.0002210.0001570.0003521.6e-05

0.0002330.0001240.0002461.4e-05

0.0002330.0001240.0002461.4e-05

0.000169

0.000169

0.000169

0.000110.0001090.0001071.4e-05

0.000110.0001090.000107

0.000110.0001090.000107

1.4e-05

1.4e-05

0.0005251.6e-050.0004090.0005282.8e-05

0.0005251.6e-050.0004090.0005282.8e-05

2.1e-05

9e-055.8e-058.6e-05

4.4e-05

0.0001364.4e-050.000156

0.0001096.6e-050.000111

8.4e-05

0.0001397.6e-050.000127

5.1e-051.6e-051.6e-054.8e-052.8e-05

0.0007646.9e-050.0003610.0014780.000737

0.0001360.0001870.000109

0.0001360.0001870.000109

6.1e-05

6.1e-05

0.0002750.0002730.000265

8.8e-057.2e-058.5e-05

0.0001340.0001320.000133

5.3e-056.9e-054.7e-05

8.6e-05

8.6e-05

0.000273

0.000133

0.00014

0.0003536.9e-050.0003610.0005530.000363

0.0003536.9e-050.0003610.0005530.000363

4.5e-05

4.5e-05

0.0031930.0013670.0008360.0023190.0013260.0031370.000343

6.1e-05

6.1e-05

0.00040.0002210.0003970.000287

0.000111

0.0001132.1e-050.000104

0.0001172.6e-050.0001180.000124

0.000103

9.3e-056e-060.0001053.8e-05

7.7e-056.5e-057e-051.4e-05

5.3e-050.0001165.5e-05

1.7e-053.3e-052.7e-05

1.2e-053.6e-051.2e-05

4e-063e-062e-06

2e-052.6e-051.4e-05

1.8e-05

0.0024580.0013190.0008360.0023190.0005930.0024322.7e-05

0.000145.6e-050.000137

2e-06

8.8e-05

0

0

8e-052.1e-051.1e-057.9e-05

7e-06

0.0021270.0012790.0008360.0023190.0003160.002084

6.3e-05

1.2e-05

0.0001111.9e-053.8e-050.0001322.7e-05

0

5e-05

5e-05

0.0002824.8e-050.00020.0002532.9e-05

0.0001462.3e-057.5e-050.0001322.9e-05

0.0001134.2e-059.8e-05

2.3e-052.5e-051.6e-052.3e-05

6.7e-05

8.5e-05

8.5e-05

0.000110.0001320.0001141.8e-05

0.0001090.000120.0001141.8e-05

0.0001093.8e-050.0001144e-06

8.2e-05

1.4e-05

1e-061.2e-050

000

01e-060

1e-060

0

1e-0600

9e-06

01e-060

000

000

0.0082810.0035120.0049070.0053070.0086460.00132

0.0003063.6e-050.0003390.0009080.0002858.7e-05

0.0003063.6e-050.0003390.0009080.0002858.7e-05

0.0079750.0034760.0041870.0037720.0083610.001229

0.0019980.001370.0008140.0004940.0020970.000573

0.0017280.000390.0006680.0006090.001814

0.0019750.0008280.0006870.0006040.0020830.00038

0.0022740.0008880.0005610.0004290.0023670.000276

0.0014570.001636

0.0001994e-06

4e-06

0.000199

0.0003810.000428

0.0003810.000428

0.0003616.1e-05

0.000146

0.000146

0.000215

8.6e-05

0.000129

6.1e-05

6.1e-05

0.0002470.0002460.0003710.000242

0.0002470.0002460.0003710.000242

0.0002470.0002460.0003710.000242

0.0001620.0003080.000137

0.0001178.9e-050.0001

0.0001178.9e-050.0001

4.5e-057.8e-053.7e-05

4.5e-057.8e-053.7e-05

0.000141

0.000141

0.0035460.0016480.0025950.0047380.0023350.0036060.001534

0.0002690.0001830.000274

0.0001297.9e-050.000128

0.000140.0001040.000146

0.0024640.0010740.001920.0021170.0014670.0025510.000321

0.0002395.3e-050.0002236.1e-050.0002625.9e-05

0.0002799.7e-053.8e-050.0003050.000163

6.4e-05

0.0002094.8e-059.7e-050.0002115.2e-05

0.0002668.8e-050.0002443.8e-050.0002824.7e-05

0.0003880.000436

8.9e-05

0.0002144.9e-050.0001976.3e-050.000228

0.0012570.0007390.0008680.0021170.0005810.001263

0.000186

7e-05

6.5e-05

5.1e-05

0.0007360.0005740.0006750.0026210.0004540.000710.001213

0.0007360.0005740.0006750.0026210.0004540.000710.001213

7.7e-054.5e-057.1e-05

7.7e-054.5e-057.1e-05

6.3e-050.0002124.7e-05

6.3e-050.0002124.7e-05

9e-068e-068e-06

6e-061.7e-055e-06

3.4e-05

6e-067e-061e-06

5e-065.6e-055e-06

3e-068e-064e-06

1e-053.2e-059e-06

1.2e-053e-051.2e-05

1.2e-052e-053e-06

0.0004350.0002360.0004020.0004164.8e-05

0.0004350.0002360.0004020.0004164.8e-05

4.5e-050.0001442.9e-05

4.5e-050.0001442.9e-05

0.0003370.0002360.0001840.0003434.8e-05

0.0003370.0002360.0001840.0003434.8e-05

5.3e-057.4e-054.4e-05

5.3e-057.4e-054.4e-05

2.5e-054.7e-052.5e-05

2.5e-054.7e-052.5e-05

2.5e-054.7e-052.5e-05

2.5e-054.7e-052.5e-05

0.1024010.1621780.0501130.104940.0177050.1009280.139826

1.4e-054.1e-056e-06

1.4e-054.1e-056e-06

4e-062.5e-053e-06

4e-062.5e-053e-06

1e-051.6e-053e-06

1.2e-05

1e-054e-063e-06

5e-065e-055e-06

5e-065e-055e-06

5e-065e-055e-06

5e-065e-055e-06

0.1023820.1621780.0501130.104940.0176140.1009170.139826

0.1022570.1621780.0501130.104940.0173070.100830.139826

1.7e-055.4e-051.1e-05

7e-06

8e-06

1e-05

4e-06

6e-068e-061e-06

1.1e-051.7e-051e-05

0.1022040.1621780.0501130.104940.0171970.1007870.139826

1.3e-05

4.4e-05

4.1e-052.7e-05

0.0001252.8e-050.000129

3.2e-05

3.9e-052e-05

1e-05

7e-06

8e-05

0.000131

3.4e-05

0.0009320.003210.0017480.0458677.4e-050.0015785.4e-05

2e-052e-062e-05

3.7e-05

0.1010270.1589680.0483650.0590730.0166190.0990410.139772

2e-053.9e-051.9e-05

3.6e-055.6e-053.2e-05

1.3e-053.1e-051.1e-05

8e-061.5e-056e-06

1.5e-051e-051.5e-05

5.2e-052.9e-054.6e-05

5.2e-052.9e-054.6e-05

5.2e-052.9e-054.6e-05

7.3e-050.0002784.1e-05

7e-063e-054e-06

7e-063e-054e-06

1e-053.8e-051.1e-05

1e-053.8e-051.1e-05

2.7e-054.1e-051.4e-05

1.1e-051.9e-059e-06

1.6e-052.2e-055e-06

2.9e-050.0001691.2e-05

9e-067e-062e-06

3.9e-05

1e-061e-061e-06

01.4e-051e-06

2e-061.2e-050

7e-06

8e-061.2e-054e-06

3.2e-05

3e-064.4e-051e-06

1e-061e-061e-06

5e-062e-06

0.0684710.054960.0495890.0537290.0522870.0671370.045421

0.0004780.0008830.000412.3e-05

6.9e-050.000246.9e-05

9e-065.7e-059e-06

9e-063.6e-059e-06

2.1e-05

6e-050.0001836e-05

1.4e-053.9e-051.3e-05

3.4e-05

2e-05

1.9e-051e-051.9e-05

1e-052.6e-051e-05

1.7e-055.4e-051.8e-05

0.0001217.1e-059.3e-052.3e-05

0.0001217.1e-059.3e-052.3e-05

0.0001217.1e-059.3e-052.3e-05

0.00014

7.4e-05

7.4e-05

6.6e-05

6.6e-05

0.000160.0001570.000125

3.3e-054.3e-052.7e-05

3.3e-054.3e-052.7e-05

5.7e-056.7e-054.1e-05

5.7e-056.7e-054.1e-05

7e-054.7e-055.7e-05

7e-054.7e-055.7e-05

0.0001280.0002750.000123

0.000101

4.3e-05

5.8e-05

3.8e-054.6e-054.2e-05

3.8e-054.6e-054.2e-05

3.1e-058.1e-053.5e-05

1.4e-054e-051.3e-05

1.7e-054.1e-052.2e-05

3.7e-05

3.7e-05

5.9e-051e-054.6e-05

1.9e-056e-061.6e-05

4e-054e-063e-05

0.0272260.016290.0260070.0219760.0219110.0269640.020047

0.000540.0061860.0034580.0005230.004089

0.0001576.9e-050.000132

0.0001576.9e-050.000132

0.000176.6e-050.000174

0.000176.6e-050.000174

4e-05

4e-05

0.000159e-050.0001471.4e-05

0.000159e-050.000147

1.4e-05

0.0061860.0030020.004075

7.7e-05

0.0025550.002867

5.8e-05

0.0036310.004075

6.3e-057.8e-057e-05

3.1e-053.3e-05

3.2e-057.8e-053.7e-05

6.5e-05

6.5e-05

4.8e-05

4.8e-05

0.0041490.004657

0.0041490.004657

0.0041490.004657

0.0003449e-060.0007480.000286

7.4e-05

7.4e-05

0.0001610.0002960.000151

3e-0603e-06

5e-06

1.9e-05

3e-052.7e-052.5e-05

0.0001166.3e-050.000112

3e-05

3.9e-05

3e-061e-063e-06

2.4e-05

4.6e-05

3.6e-05

9e-066e-068e-06

2.3e-055.4e-052.2e-05

2.3e-055.4e-052.2e-05

0.000101

0.000101

0.0001249e-065.5e-058e-05

2.1e-05

0.0001249e-063.4e-058e-05

4.5e-05

4.5e-05

9e-064.6e-057e-06

8e-062.7e-056e-06

1e-061.9e-051e-06

2.7e-057.7e-052.6e-05

5.3e-05

2.7e-052.4e-052.6e-05

0.0067540.002390.0039740.0054440.0047120.0068120.000423

0.0064170.002390.0039740.0054440.0043230.0065070.000408

0.0064170.002390.001230.0054440.0010160.0065070.000408

0.000227

0.0027440.00308

5.4e-050.0001514.6e-05

5.4e-054.2e-054.6e-05

5.7e-05

5.2e-05

0.0002187.2e-050.0001921.5e-05

2.2e-05

2e-05

0.0002183e-050.0001921.5e-05

6.5e-053.8e-056.7e-05

6.5e-053.8e-056.7e-05

0.000128

7.2e-05

5.6e-05

0.0092610.0083470.0072260.0108870.0071360.0090160.00759

0.0001316.3e-050.000106

0.0001316.3e-050.000106

0.0001

4.8e-05

5.2e-05

0.00017.9e-058.3e-05

0.00017.9e-058.3e-05

6.9e-05

6.9e-05

7.8e-05

3.2e-05

4.6e-05

9e-065.4e-058e-06

9e-065.4e-058e-06

6.6e-053e-056.1e-051.4e-05

6.6e-053e-056.1e-051.4e-05

0.0073810.0076770.0063890.008770.0054410.0072180.007529

5.8e-05

0.0073810.0076770.0063890.008770.0053830.0072180.007529

0.0001260.0001220.000116

7.4e-056.8e-056.7e-05

5.2e-055.4e-054.9e-05

0.000122

6.4e-05

5.8e-05

5.7e-056.5e-055.7e-05

5.7e-056.5e-055.7e-05

6.9e-053.3e-057.4e-05

6.9e-053.3e-057.4e-05

0.0012780.000670.0008370.0021170.0008510.0012464.7e-05

5.4e-05

0.0010940.000670.0008370.0021170.0006650.001063

2e-059e-062.4e-054.7e-05

2.7e-052.2e-052.6e-05

7.2e-054.9e-057.6e-05

6.5e-055.2e-055.7e-05

4.4e-052.9e-054.7e-05

1.6e-05

2.7e-056e-063e-05

1.7e-057e-061.7e-05

0.0103270.0055440.0044720.0056450.0058570.0103270.003288

0.0086950.0049920.0037740.0056450.0039220.0087180.003025

1.3e-054e-062.3e-05

8.2e-055.1e-058.8e-05

0.0001394.2e-050.000138

1.1e-05

3e-06

8.8e-05

8e-06

4.9e-057.1e-051.5e-055.2e-05

4.7e-05

2.1e-05

3e-05

5.5e-051.7e-055.9e-055e-05

1.9e-05

0.000137e-050.0001361.8e-05

1.2e-05

0.0050910.0026260.0024180.0029230.002560.0050980.001419

4.9e-051e-055.7e-05

0.0001251.8e-050.000123

2.9e-052e-051e-053.3e-05

1.3e-05

0.0027040.0022620.0013560.0027220.0005830.0026690.001485

0

2.8e-05

6.1e-054e-066.6e-052.4e-05

2.4e-05

4.6e-051e-054.6e-051.5e-05

9e-06

5e-061.3e-052e-065e-068e-06

9.7e-05

1.5e-05

1e-06

1.6e-05

1.6e-058e-061.5e-05

1.8e-05

1.6e-05

9.9e-051.3e-050.000108

1.6e-05

9e-06

2e-064e-062e-066e-06

0.0006429.2e-050.0004360.0006088.8e-05

3.5e-05

0.000105

0.0001946e-055.8e-050.0001822.3e-05

4.1e-055e-054.6e-05

0.00023.2e-058.7e-050.0001886.5e-05

0.0001325.3e-050.000118

7.5e-054.8e-057.4e-05

5.6e-050.0002244.3e-05

3.4e-05

3.1e-05

1.4e-05

2.1e-05

5.6e-051.1e-054.3e-05

1e-05

2.2e-05

6.7e-05

1.4e-05

1.4e-05

1.4e-05

1.6e-055.8e-051.6e-05

5e-062.8e-055e-06

1.1e-051.9e-051.1e-05

1.1e-05

0.0001814.4e-050.0001080.0001793.5e-05

7.2e-052.6e-052e-056.9e-053.5e-05

0.0001091.8e-054.1e-050.00011

2.2e-05

2.5e-05

0.0007370.0004160.0006980.0011090.0007630.000126

0.0001378.7e-050.000156

0.0001680.0001416.8e-050.000177.3e-05

0.0006980.000783

0.0002016.1e-050.0002135.3e-05

8.9e-05

0.0002310.0002148.2e-050.000224

0.0363310.0275730.0192420.015020.0268340.0355160.012233

0.0363310.0275730.0192420.015020.0268340.0355160.012233

6.1e-057.4e-055.3e-05

1e-061.2e-054e-06

2.2e-052.3e-051e-05

2.8e-051.4e-052.9e-05

2e-067e-062e-06

8e-061.8e-058e-06

4.2e-058e-063.3e-05

4.2e-058e-063.3e-05

0.0362110.0275730.0192420.015020.0267290.0354190.012233

0.0362110.0275730.0192420.015020.0267290.0354190.012233

1.7e-052.3e-051.1e-05

1.7e-052.3e-051.1e-05

0.0037470.0110970.004340.0167330.0022310.0036470.013118

0.0032280.0110430.004340.0148180.0019730.0032340.013075

0.0001370.000137

0.000137

0.000137

0.000101

0.000101

7.8e-05

7.8e-05

0.0032280.0110430.004340.0148180.0017350.0032340.01286

0.000104

0.0019650.0104650.0006880.0070560.0001550.0020520.010235

0.0008780.000985

0.0002970.000334

0.0006770.000764e-05

0.0003470.00039

0.000201

0.0003710.000416

1.7e-05

0.0009260.0003830.0005740.0040320.0003610.0009524.7e-05

1.9e-05

0.0002720.000305

0.0003370.0001950.0002360.003734.3e-050.000230.000161

2.2e-05

0.0005195.4e-050.0019150.0002580.0004134.3e-05

0.0001065.5e-057.2e-05

0.0001065.5e-057.2e-05

0.0002645.4e-050.0019150.0001370.0002144.3e-05

0.0002645.4e-050.0019150.0001370.0002144.3e-05

0.0001496.6e-050.000127

0.0001496.6e-050.000127

0.0006890.0004280.0006

0.0004080.000260.000377

0.0002550.0001320.000233

9.9e-056.5e-058.8e-05

5e-05

0.0001567e-060.000145

1e-05

0.0001530.0001280.000144

5.6e-05

0.0001537.2e-050.000144

0.0002150.0001130.000176

8.7e-054.6e-058e-05

8.7e-054.6e-058e-05

0.0001286.7e-059.6e-05

0.0001286.7e-059.6e-05

6.6e-055.5e-054.7e-05

6.6e-055.5e-054.7e-05

6.6e-055.5e-054.7e-05

5.1e-050.0001324.3e-05

5.1e-050.0001324.3e-05

5.1e-050.0001324.3e-05

5.1e-050.0001324.3e-05

3.1e-055.8e-052.6e-05

2.1e-05

3.1e-053.7e-052.6e-05

2e-054.2e-051.7e-05

2e-054.2e-051.7e-05

3.2e-05

3.2e-05

0.011270.0596140.0139630.0540320.0092490.0101280.035609

7.7e-05

7.7e-05

7.7e-05

7.7e-05

7.7e-05

0.011270.0596140.0139630.0540320.0091720.0101280.035609

0.0002060.0003680.000138

0.0002060.0003680.000138

0.0001010.0003148.9e-05

2.1e-059.5e-051.1e-05

2e-059.7e-052.5e-05

6e-057.1e-055.3e-05

5.1e-05

0.0001055.4e-054.9e-05

3e-061e-063e-06

9.1e-052.2e-053.6e-05

3e-062.4e-053e-06

0

8e-067e-067e-06

0.0109750.0596140.0139630.0540320.0086160.0098980.035609

0.0109750.0596140.0139630.0540320.0086160.0098980.035609

0.010920.0596140.0139630.0540320.0086090.0098650.035609

7e-068e-065e-06

1e-061e-06

3e-06

02e-062e-06

3.7e-05

4e-0604e-06

2.6e-053.5e-052e-05

6e-062.2e-052e-06

05e-061e-06

1.7e-05

9e-063.6e-058e-06

1.6e-05

3e-064e-064e-06

1.6e-055.5e-051.1e-05

01e-060

1e-063e-06

3.2e-05

2e-064e-051e-06

1e-062e-061e-06

6e-066e-069e-06

5e-068e-067e-06

2e-064e-06

0.0107770.0596140.0139630.0540320.008080.0097110.035609

7e-06

1e-064e-061e-06

6e-062.8e-056e-06

5e-06

1.1e-051.5e-051.1e-05

2e-063e-062e-06

04e-06

6e-063e-066e-06

4e-069.6e-053e-06

2e-061.8e-053e-05

4e-061e-061e-06

1.8e-055e-061.4e-05

8e-06

5.5e-057e-063.3e-05

2.5e-051e-068e-06

3e-056e-062.5e-05

8.9e-050.0001889.2e-05

1.8e-052.1e-051.8e-05

1.8e-052.1e-051.8e-05

1.8e-052.1e-051.8e-05

7.1e-050.0001677.4e-05

7.1e-050.0001677.4e-05

1.3e-05

1.7e-05

4e-061e-053e-06

1.5e-05

6e-063e-065e-06

2.2e-05

1.1e-052.6e-051.3e-05

3.5e-051.4e-053.2e-05

2.2e-05

1.5e-051.3e-052.1e-05

1.2e-05

1.8e-054e-059e-06

1.8e-054e-059e-06

1.8e-054e-059e-06

1.8e-054e-059e-06

1.8e-054e-059e-06

1.8e-054e-059e-06

9e-064.8e-058e-06

9e-064.8e-058e-06

9e-064.8e-058e-06

9e-064.8e-058e-06

9e-064.8e-058e-06

9e-064.8e-058e-06

8.8e-055.5e-05

8.8e-055.5e-05

8.8e-055.5e-05

8.8e-055.5e-05

8.8e-055.5e-05

8.8e-055.5e-05

8.8e-055.5e-05

0.0039650.0017210.0022330.010080.0063440.0039390.000587

3.4e-050.000353.2e-05

3.4e-050.000353.2e-05

2.8e-053.1e-052.7e-05

1.5e-05

1.5e-05

1.5e-05

2.8e-051.6e-052.7e-05

2.8e-051.6e-052.7e-05

2.8e-051.6e-052.7e-05

4e-060.0001294e-06

4e-067.5e-054e-06

01.6e-051e-06

02e-061e-06

1.4e-05

05e-06

4e-06

01e-06

7e-06

7e-06

08e-060

08e-060

4e-063.9e-053e-06

4e-063.9e-053e-06

5.4e-05

2.2e-05

2.2e-05

1.7e-05

1.7e-05

1.5e-05

1.5e-05

1e-065.4e-051e-06

1e-065.4e-051e-06

1e-062.9e-051e-06

1e-061e-061e-06

01.1e-050

03e-060

000

1.4e-05

06e-060

06e-060

01.9e-05

01.7e-05

2e-06

1e-060.0001360

09.2e-050

2.5e-050

9e-060

1.6e-05

1e-05

1e-05

02.6e-050

02.6e-050

03.1e-050

8e-060

02.3e-050

1e-064.4e-050

1e-064.4e-050

0

1e-06

1e-061.2e-050

3.1e-05

4.5e-050.0002054.4e-05

4.5e-050.0002054.4e-05

4.5e-050.0002054.4e-05

4.5e-050.0002054.4e-05

4.5e-050.0002054.4e-05

4.5e-050.0002054.4e-05

3.4e-05

3.4e-05

3.4e-05

3.4e-05

3.4e-05

3.4e-05

6e-067.4e-055e-06

6e-067.4e-055e-06

3.6e-05

3.6e-05

2e-05

2e-05

1.6e-05

1.6e-05

6e-063.8e-055e-06

6e-063.8e-055e-06

6e-063.8e-055e-06

6e-063e-055e-06

8e-06

0.003880.0017210.0022330.010080.0056810.0038580.000587

3.1e-050.0001094.3e-05

3.1e-050.0001094.3e-05

1.6e-055.5e-052.1e-05

2e-068e-062e-06

2e-068e-062e-06

1.4e-054.7e-051.9e-05

1e-06

07e-061e-06

8e-061.6e-051.2e-05

1.4e-05

08e-060

6e-061e-066e-06

1.5e-055.4e-052.2e-05

1.3e-053.1e-052e-05

3e-067e-061.1e-05

3e-065e-062e-06

2e-067e-062e-06

5e-061.2e-055e-06

2e-062.3e-052e-06

2e-062.3e-052e-06

1e-060.0001011e-06

1e-060.0001011e-06

1e-060.0001011e-06

01.8e-050

01.8e-050

1e-064.8e-051e-06

1e-061e-050

01.6e-05

01.3e-051e-06

9e-06

3.5e-05

2.1e-05

1.4e-05

1.9e-050.0001961.9e-05

1.9e-050.0001961.9e-05

02.7e-050

02.7e-050

02.7e-050

1.9e-050.0001691.9e-05

2e-061.7e-051e-06

04e-060

3e-06

2e-061e-051e-06

6e-062.1e-056e-06

6e-062.1e-056e-06

02.9e-051e-06

7e-06

6e-061e-06

09e-060

07e-060

1.1e-050.0001021.1e-05

3e-062.5e-053e-06

1.4e-05

2.3e-05

5e-061e-055e-06

3e-061.4e-053e-06

1.6e-05

0.0006640.0003990.0004030.0020160.0005120.0006490.000218

0.0006640.0003990.0004030.0020160.0005120.0006490.000218

0.0006640.0003990.0004030.0020160.0005120.0006490.000218

4e-062.9e-055e-06

02e-061e-06

07e-060

4e-062e-064e-06

05e-060

05e-060

1e-06

7e-06

1.4e-05

1.4e-05

0.000660.0003990.0004030.0020160.0004690.0006440.000218

2e-06

7e-06

0.0006530.0003990.0004030.0020160.0003890.000640.000218

6e-06

08e-060

06e-060

5e-06

3e-069e-062e-06

3e-06

4e-064e-062e-06

2e-060

07e-060

3e-06

6e-06

5e-06

3e-060

4e-06

2.2e-050.0002082.9e-05

5e-060.0001013e-06

5e-060.0001013e-06

1e-063.9e-051e-06

1e-063.9e-051e-06

2.9e-05

2.9e-05

4e-063.3e-052e-06

4e-063.3e-052e-06

2.6e-05

2.6e-05

2.6e-05

2.6e-05

1.7e-058.1e-052.6e-05

1.1e-052.3e-052.2e-05

1.1e-052.3e-052.2e-05

1.1e-052.3e-052.2e-05

6e-063.2e-054e-06

6e-063.2e-054e-06

2e-061.5e-050

4e-061.7e-054e-06

1.6e-05

1.6e-05

1.6e-05

1e-05

1e-05

1e-05

0.0002690.0001190.0019150.0006340.0002729.6e-05

0.0002090.0001190.0019150.0003280.0002089.6e-05

6e-063e-052e-06

6e-063e-052e-06

6e-063e-052e-06

2.7e-057.8e-051.7e-05

4e-063.2e-052e-06

4e-063.2e-052e-06

2.3e-054.6e-051.5e-05

1.7e-052.3e-059e-06

6e-062.3e-056e-06

0.0001680.0001190.0019150.0001410.0001649.6e-05

0.0001680.0001190.0019150.0001410.0001649.6e-05

0.0001680.0001190.0019150.0001410.0001649.6e-05

8e-067.9e-052.5e-05

1e-062.6e-051e-06

1e-062.6e-051e-06

7e-065.3e-052.4e-05

4e-063e-051e-05

1.1e-05

3e-061.2e-051.4e-05

4.2e-050.0002495.5e-05

1.7e-056e-052.6e-05

1.7e-056e-052.6e-05

1.4e-054e-052.2e-05

3e-062e-054e-06

2.5e-050.0001892.9e-05

1.4e-055.8e-051e-05

2e-06

1e-052e-065e-06

2e-06

1e-06

4e-06

1.4e-05

1e-05

6e-06

1e-06

1.1e-05

4e-063e-064e-06

02e-061e-06

01.5e-050

01.5e-050

1e-058e-061e-05

1e-058e-061e-05

02.5e-050

02.5e-050

1e-063.2e-059e-06

1e-063.2e-059e-06

03.6e-05

01.5e-05

2.1e-05

01.5e-050

01.5e-050

1.8e-055.7e-059e-06

1.8e-055.7e-059e-06

1.8e-055.7e-059e-06

8e-062.7e-055e-06

1e-053e-054e-06

0.0009640.0006040.0008040.0021170.0007680.000943

0.0009640.0006040.0008040.0021170.0007680.000943

0.0009640.0006040.0008040.0021170.0007680.000943

0.0009640.0006040.0008040.0021170.0007680.000943

09e-060

0.0009640.0006040.0008040.0021170.0007590.000943

0.001910.0005990.0010260.0040320.0031530.0019020.000273

0.0003360.0005750.000319

0.0003360.0005750.000319

5.9e-056.6e-056.2e-05

5.9e-056.6e-056.2e-05

3.9e-050.0001232.8e-05

3.9e-050.0001232.8e-05

3.5e-054.9e-053.8e-05

3.5e-054.9e-053.8e-05

4.9e-054.2e-054.2e-05

4.9e-054.2e-054.2e-05

5.2e-05

5.2e-05

8.3e-050.0001047.5e-05

5.2e-055.1e-055.2e-05

3.1e-055.3e-052.3e-05

4.5e-056.6e-054.6e-05

4.5e-056.6e-054.6e-05

2.6e-057.3e-052.8e-05

2.6e-057.3e-052.8e-05

0.0005820.0002240.0006570.0020160.0010820.0006010.000103

5.9e-050.0004420.0004967.6e-05

5.9e-050.0004420.0004967.6e-05

5.9e-050.0004420.0004967.6e-05

0.0005230.0002240.0002150.0020160.0005860.0005250.000103

2.4e-050.0001423.8e-05

2.4e-050.0001423.8e-05

3.4e-050.0001293.6e-05

3.4e-050.0001293.6e-05

0.0004650.0002240.0002150.0020160.0003150.0004510.000103

4.7e-057.8e-054.2e-05

4.8e-05

0.0004180.0002240.0002150.0020160.0001890.0004090.000103

0.0009920.0003750.0003690.0020160.0014960.0009820.00017

3.9e-050.0003464e-05

1.4e-050.0001191.4e-05

1.4e-050.0001191.4e-05

2.5e-050.0002272.6e-05

8.1e-05

8.5e-05

2.5e-056.1e-052.6e-05

0.0009530.0003750.0003690.0020160.0010860.0009420.00017

4.6e-050.0001153.9e-05

4.6e-050.0001153.9e-05

3e-054.7e-052.3e-05

9e-06

1.5e-052e-061e-05

1.5e-053.6e-051.3e-05

0.0002140.0004030.000229

8.7e-050.0001950.000103

0.0001270.0002080.000126

0.0006630.0003750.0003690.0020160.0005210.0006510.00017

0.0006290.0003750.0003690.0020160.0004510.0006140.00017

3.4e-057e-053.7e-05

6.4e-05

6.4e-05

6.4e-05
