# Supplementary material for: MetaMeta: integrating metagenome analysis tools to improve taxonomic profiling
Source: Microbiome. 2017 Aug 14;5:101. doi: 10.1186/s40168-017-0318-y (PMC5557516; doi:10.1186/s40168-017-0318-y)
Supplement: Supplementary file 2 — Additional File with interactive charts for all CAMI toy set results on default, very-precise and very-sensitive mode. File prefix S, M, and H for low, medium and high complexity, respectively. (TAR 3573 kb) [file 40168_2017_318_MOESM2_ESM.tar › M2_S001__insert_180_very-sensitive.html]

Javascript must be enabled to view this page.

magnitude
magnitudeUnassigned

clark.parsed\_profile
dudes.parsed\_profile
final.metametamerge.profile
gottcha.parsed\_profile
kaiju.parsed\_profile
kraken.parsed\_profile
motus.parsed\_profile

0.9999919999999990.9999961.0000020.9999991.0000060.9999939999999990.999985999999998

8.8e-055.5e-05

8.8e-055.5e-05

8.8e-055.5e-05

8.8e-055.5e-05

8.8e-055.5e-05

8.8e-055.5e-05

8.8e-055.5e-05

0.0039650.0017210.0028940.010080.0063440.0039390.000587

3.4e-05

3.4e-05

3.4e-05

3.4e-05

3.4e-05

3.4e-05

0.003880.0017210.0027190.010080.0056810.0038580.000587

0.001910.0005990.0015610.0040320.0031530.0019020.000273

0.0003360.0001050.0005750.000319

0.0003360.0001050.0005750.000319

4.9e-054.2e-054.2e-05

4.9e-054.2e-054.2e-05

5.2e-05

5.2e-05

4.5e-056.6e-054.6e-05

4.5e-056.6e-054.6e-05

3.9e-050.0001050.0001232.8e-05

3.9e-050.0001050.0001232.8e-05

3.5e-054.9e-053.8e-05

3.5e-054.9e-053.8e-05

2.6e-057.3e-052.8e-05

2.6e-057.3e-052.8e-05

8.3e-050.0001047.5e-05

5.2e-055.1e-055.2e-05

3.1e-055.3e-052.3e-05

5.9e-056.6e-056.2e-05

5.9e-056.6e-056.2e-05

0.0005820.0002240.0008630.0020160.0010820.0006010.000103

0.0005230.0002240.0004390.0020160.0005860.0005250.000103

2.4e-050.0001210.0001423.8e-05

2.4e-050.0001210.0001423.8e-05

3.4e-050.0001110.0001293.6e-05

3.4e-050.0001110.0001293.6e-05

0.0004650.0002240.0002070.0020160.0003150.0004510.000103

0.0004180.0002240.0002070.0020160.0001890.0004090.000103

4.8e-05

4.7e-057.8e-054.2e-05

5.9e-050.0004240.0004967.6e-05

5.9e-050.0004240.0004967.6e-05

5.9e-050.0004240.0004967.6e-05

0.0009920.0003750.0005930.0020160.0014960.0009820.00017

0.0009530.0003750.0005930.0020160.0010860.0009420.00017

0.0002140.0002390.0004030.000229

0.0001270.0001240.0002080.000126

8.7e-050.0001150.0001950.000103

0.0006630.0003750.0003540.0020160.0005210.0006510.00017

3.4e-057e-053.7e-05

0.0006290.0003750.0003540.0020160.0004510.0006140.00017

3e-054.7e-052.3e-05

1.5e-052e-061e-05

1.5e-053.6e-051.3e-05

9e-06

4.6e-050.0001153.9e-05

4.6e-050.0001153.9e-05

6.4e-05

6.4e-05

6.4e-05

3.9e-050.0003464e-05

1.4e-050.0001191.4e-05

1.4e-050.0001191.4e-05

2.5e-050.0002272.6e-05

2.5e-056.1e-052.6e-05

8.5e-05

8.1e-05

0.0009640.0006040.0007710.0021170.0007680.000943

0.0009640.0006040.0007710.0021170.0007680.000943

0.0009640.0006040.0007710.0021170.0007680.000943

0.0009640.0006040.0007710.0021170.0007680.000943

09e-060

0.0009640.0006040.0007710.0021170.0007590.000943

0.0002690.0001190.0019150.0006340.0002729.6e-05

0.0002090.0001190.0019150.0003280.0002089.6e-05

6e-063e-052e-06

6e-063e-052e-06

6e-063e-052e-06

0.0001680.0001190.0019150.0001410.0001649.6e-05

0.0001680.0001190.0019150.0001410.0001649.6e-05

0.0001680.0001190.0019150.0001410.0001649.6e-05

2.7e-057.8e-051.7e-05

4e-063.2e-052e-06

4e-063.2e-052e-06

2.3e-054.6e-051.5e-05

1.7e-052.3e-059e-06

6e-062.3e-056e-06

8e-067.9e-052.5e-05

7e-065.3e-052.4e-05

4e-063e-051e-05

3e-061.2e-051.4e-05

1.1e-05

1e-062.6e-051e-06

1e-062.6e-051e-06

4.2e-050.0002495.5e-05

1.7e-056e-052.6e-05

1.7e-056e-052.6e-05

1.4e-054e-052.2e-05

3e-062e-054e-06

2.5e-050.0001892.9e-05

01.5e-050

01.5e-050

1e-058e-061e-05

1e-058e-061e-05

01.5e-050

01.5e-050

02.5e-050

02.5e-050

1.4e-055.8e-051e-05

2e-06

1.4e-05

2e-06

6e-06

1e-06

1.1e-05

1e-05

1e-06

4e-063e-064e-06

1e-052e-065e-06

02e-061e-06

4e-06

1e-063.2e-059e-06

1e-063.2e-059e-06

03.6e-05

01.5e-05

2.1e-05

1.8e-055.7e-059e-06

1.8e-055.7e-059e-06

1.8e-055.7e-059e-06

1e-053e-054e-06

8e-062.7e-055e-06

1e-060.0001011e-06

1e-060.0001011e-06

1e-060.0001011e-06

01.8e-050

01.8e-050

1e-064.8e-051e-06

9e-06

1e-061e-050

01.6e-05

01.3e-051e-06

3.5e-05

1.4e-05

2.1e-05

3.1e-050.0001094.3e-05

3.1e-050.0001094.3e-05

1.5e-055.4e-052.2e-05

1.3e-053.1e-052e-05

2e-067e-062e-06

3e-065e-062e-06

3e-067e-061.1e-05

5e-061.2e-055e-06

2e-062.3e-052e-06

2e-062.3e-052e-06

1.6e-055.5e-052.1e-05

2e-068e-062e-06

2e-068e-062e-06

1.4e-054.7e-051.9e-05

1e-06

8e-061.6e-051.2e-05

07e-061e-06

1.4e-05

6e-061e-066e-06

08e-060

1.9e-050.0001961.9e-05

1.9e-050.0001961.9e-05

02.7e-050

02.7e-050

02.7e-050

1.9e-050.0001691.9e-05

02.9e-051e-06

09e-060

07e-060

6e-061e-06

7e-06

2e-061.7e-051e-06

04e-060

2e-061e-051e-06

3e-06

6e-062.1e-056e-06

6e-062.1e-056e-06

1.1e-050.0001021.1e-05

3e-061.4e-053e-06

2.3e-05

5e-061e-055e-06

3e-062.5e-053e-06

1.6e-05

1.4e-05

0.0006640.0003990.0003870.0020160.0005120.0006490.000218

0.0006640.0003990.0003870.0020160.0005120.0006490.000218

0.0006640.0003990.0003870.0020160.0005120.0006490.000218

1.4e-05

1.4e-05

0.000660.0003990.0003870.0020160.0004690.0006440.000218

4e-06

5e-06

2e-060

5e-06

2e-06

6e-06

4e-064e-062e-06

3e-069e-062e-06

3e-060

06e-060

3e-06

6e-06

3e-06

07e-060

0.0006530.0003990.0003870.0020160.0003890.000640.000218

08e-060

7e-06

4e-062.9e-055e-06

07e-060

1e-06

02e-061e-06

05e-060

7e-06

4e-062e-064e-06

05e-060

2.2e-050.0002082.9e-05

5e-060.0001013e-06

5e-060.0001013e-06

1e-063.9e-051e-06

1e-063.9e-051e-06

4e-063.3e-052e-06

4e-063.3e-052e-06

2.9e-05

2.9e-05

1.7e-058.1e-052.6e-05

6e-063.2e-054e-06

6e-063.2e-054e-06

4e-061.7e-054e-06

2e-061.5e-050

1.1e-052.3e-052.2e-05

1.1e-052.3e-052.2e-05

1.1e-052.3e-052.2e-05

1.6e-05

1.6e-05

1.6e-05

1e-05

1e-05

1e-05

2.6e-05

2.6e-05

2.6e-05

2.6e-05

4.5e-050.0001750.0002054.4e-05

4.5e-050.0001750.0002054.4e-05

4.5e-050.0001750.0002054.4e-05

4.5e-050.0001750.0002054.4e-05

4.5e-050.0001750.0002054.4e-05

4.5e-050.0001750.0002054.4e-05

3.4e-050.000353.2e-05

3.4e-050.000353.2e-05

1e-060.0001360

1e-064.4e-050

1e-064.4e-050

3.1e-05

1e-061.2e-050

0

1e-06

09.2e-050

1e-05

1e-05

2.5e-050

1.6e-05

9e-060

03.1e-050

02.3e-050

8e-060

02.6e-050

02.6e-050

4e-060.0001294e-06

4e-067.5e-054e-06

7e-06

7e-06

08e-060

08e-060

05e-06

4e-06

01e-06

4e-063.9e-053e-06

4e-063.9e-053e-06

01.6e-051e-06

1.4e-05

02e-061e-06

5.4e-05

2.2e-05

2.2e-05

1.7e-05

1.7e-05

1.5e-05

1.5e-05

2.8e-053.1e-052.7e-05

2.8e-051.6e-052.7e-05

2.8e-051.6e-052.7e-05

2.8e-051.6e-052.7e-05

1.5e-05

1.5e-05

1.5e-05

1e-065.4e-051e-06

1e-065.4e-051e-06

01.9e-05

2e-06

01.7e-05

1e-062.9e-051e-06

01.1e-050

000

1e-061e-061e-06

03e-060

1.4e-05

06e-060

06e-060

6e-067.4e-055e-06

6e-067.4e-055e-06

6e-063.8e-055e-06

6e-063.8e-055e-06

6e-063.8e-055e-06

8e-06

6e-063e-055e-06

3.6e-05

3.6e-05

2e-05

2e-05

1.6e-05

1.6e-05

0.9959389999999990.9982750.9971080000000010.9899190.9936619999999990.9959999999999990.999398999999998

0.049650.0323620.0248540.0214720.0371990.0485610.016479

0.0496150.0323620.0248540.0214720.0370690.0485370.016479

0.0496150.0323620.0248540.0214720.0370690.0485370.016479

0.0496150.0323620.0248540.0214720.0370690.0485370.016479

0.0495410.0323620.0248540.0214720.0368360.0484720.016479

3.3e-058.2e-052.7e-05

0.0495080.0323620.0248540.0214720.0367540.0484450.016479

2.2e-055.7e-052e-05

2.2e-055.7e-052e-05

4.1e-050.0001323.5e-05

2.4e-056.4e-051.4e-05

1.7e-056.8e-052.1e-05

1.1e-054.4e-051e-05

1.1e-054.4e-051e-05

2.3e-058.8e-051.5e-05

2.3e-058.8e-051.5e-05

2.3e-058.8e-051.5e-05

2.3e-058.8e-051.5e-05

2.3e-058.8e-051.5e-05

1.2e-054.2e-059e-06

1.2e-054.2e-059e-06

1.2e-054.2e-059e-06

1.2e-054.2e-059e-06

1.2e-054.2e-059e-06

0.3322990.1951410.2333450.1245970.2693240.3311090.115972

0.3261230.1944650.2316970.1245970.2607590.3250690.115642

9.4e-050.000110.0001770.000101

9.4e-050.000110.0001770.000101

9.4e-050.000110.0001770.000101

9.4e-050.000110.0001770.000101

0.0001720.0001270.0001190.000169

0.0001720.0001270.0001190.000169

0.0001720.0001270.0001190.000169

0.0001720.0001270.0001190.000169

0.0128340.0078230.006930.0059480.0106910.0124920.004166

0.0128340.0078230.006930.0059480.0106910.0124920.004166

0.0001250.0001079e-056.7e-05

0.0001250.0001079e-056.7e-05

0.0127090.0078230.0064280.0059480.0098650.0124250.004152

0.0127090.0078230.0064280.0059480.0098650.0124250.004152

0.000101

0.000101

5.1e-05

5.1e-05

0.0003950.0005841.4e-05

1.4e-05

0.0001270.000149

0.0001420.000166

0.000121

0.0001260.000148

0.0014945.2e-050.0019180.0020010.0015454.2e-05

0.0014945.2e-050.0019180.0020010.0015454.2e-05

0.0002842.9e-050.0002480.000296

0.0002842.9e-050.0002480.000296

0.0001160.000136

0.0001160.000136

0.0001840.0001710.0002510.00018

0.0001840.0001710.0002510.00018

0.0001180.000138

0.0001180.000138

0.0001240.000145

0.0001240.000145

0.0002350.0001580.0001260.000251

0.0002350.0001580.0001260.000251

0.0002220.0001620.0001530.0002114.2e-05

0.0002220.0001620.0001530.0002114.2e-05

0.0003140.0003490.0005470.000344

0.0003140.0002330.0002050.000344

3e-06

1e-06

0.0001160.000135

0.000109

1.2e-05

8.2e-05

0.0002552.3e-050.0003350.0003450.000263

3e-05

8e-052.3e-054.1e-058.5e-05

0.0001750.0001515.8e-050.000178

0.0001840.000216

0.0001370.00016

0.0001370.00016

0.0005290.0008810.0024090.0047380.0005440.0006080.000534

0.0005290.0008810.0024090.0047380.0005440.0006080.000534

2e-052e-052.1e-05

2e-052e-052.1e-05

6.2e-05

6.2e-05

0.0005090.0008810.0024090.0047380.0003880.0005870.000534

8e-06

3.9e-054.8e-053.3e-05

6.5e-051.2e-056.1e-05

2.4e-051.1e-052.9e-05

1.1e-05

5.3e-05

4.7e-050.0020670.0024191.6e-054.4e-05

0.0001880.0008810.0003420.0023196.2e-050.0002690.000534

3.3e-05

1.4e-05

2.2e-05

4.9e-05

3e-06

7.7e-053.5e-057.2e-05

6e-055e-065.9e-05

9e-065e-062e-05

1e-06

7.4e-05

7.4e-05

0.0002530.0001730.0001510.0002381.4e-05

0.0002530.0001730.0001510.0002381.4e-05

0.0002530.0001730.0001510.0002381.4e-05

0.0002530.0001730.0001510.0002381.4e-05

0.0008390.0006360.0008840.0008622.6e-05

0.0008390.0006360.0008840.0008622.6e-05

0.0008390.0006360.0008840.0008622.6e-05

0.0002050.0001840.0002080.000234

0.0001120.0001260.000113

0.0001880.0001580.0001850.0001812.6e-05

0.0001840.0001590.0001920.000182

0.000150.0001350.0001730.000152

0.0014750.0016350.001640.0014394.3e-05

0.0007220.0010770.0011110.0006984.3e-05

0.0003290.0002240.0001820.000345

0.0003290.0002240.0001820.000345

0.0003930.0004620.000470.0003534.3e-05

0.0003930.0002710.0002460.0003534.3e-05

0.0001910.000224

0.0002060.000242

0.0002060.000242

0.0001850.000217

0.0001850.000217

0.0007530.0005580.0005290.000741

0.000160.0001298.9e-050.000143

0.000160.0001298.9e-050.000143

0.0001270.0001110.00011

0.0001270.0001110.00011

0.0001560.0001244.1e-050.00015

9e-063e-068e-06

0.0001470.0001243.8e-050.000142

0.000210.0001860.0001010.000227

2.5e-05

0.000210.0001867.6e-050.000227

0.00010.0001190.0001870.000111

0.00010.0001190.0001870.000111

0.0009563.7e-050.0007380.0007450.0009356e-06

0.0009563.7e-050.0007380.0007450.0009356e-06

0.000333.7e-050.0002640.0002920.0003086e-06

0.000333.7e-050.0002640.0002920.0003086e-06

0.0003080.0002360.0002270.000313

0.0003080.0002360.0002270.000313

0.0003180.0002380.0002260.000314

0.0003180.0002380.0002260.000314

0.0003040.000350.0003530.000233

0.0003040.000350.0003530.000233

0.0001640.000192

0.0001640.000192

0.0003040.0001860.0001610.000233

0.0003040.0001860.0001610.000233

0.0232770.0140590.0115590.008770.0166970.0228720.00749

0.0232770.0140590.0115590.008770.0166970.0228720.00749

0.0001520.0001380.0001660.0001663e-06

0.0001520.0001380.0001660.0001663e-06

0.0003857.7e-050.0001880.0002910.0003924.7e-05

0.0001380.0001150.0001375e-06

0.0002477.7e-050.0001880.0001760.0002554.2e-05

0.0226690.0139820.0112330.008770.016190.022230.00744

0.000180.0001510.0001690.000184

0.0221160.0139820.0107920.008770.0157440.0216530.00744

0.00020.0001530.0001440.000209

0.0001730.0001370.0001330.000184

7.1e-055e-058.4e-05

3.2e-052.4e-053.8e-05

3.9e-052.6e-054.6e-05

0.0340780.015640.045750.0021170.0321990.0377980.020696

0.0340780.015640.045750.0021170.0321990.0377980.020696

0.0004613.1e-050.0002520.0001850.0003854.2e-05

0.0004613.1e-050.0002520.0001850.0003854.2e-05

0.0336170.0156090.0454980.0021170.0320140.0374130.020654

4e-06

0.0003840.00045

0.0054370.0028920.0037040.00617

0.0007350.000861

4e-06

7.7e-050.0001162.6e-058.4e-05

0.0001510.000177

0.0005113.8e-050.000598

0.000107

0.000220.000258

0.0009390.0001410.0002430.0002010.001046

1.9e-05

0.0004248.3e-050.0001980.0001190.0004712.6e-05

0.0019290.0037110.000650.0002210.0064090.000903

0.0002740.000321

6.6e-05

0.0014850.0002610.0005019.6e-050.001633

0.0004110.000481

0.0004690.000549

0.0003860.000452

0.0038070.0011610.0006320.0007411e-060.000341

0.0003490.000408

4e-05

0.0009640.0001870.0002850.0002150.001062

2.1e-05

0.0023130.0036370.002156

0.0011080.0002090.0003640.000350.0011965.4e-05

0.0008360.000120.0001690.0001030.000953

0.0002020.0002362.3e-05

0.0004840.000567

0.0013140.0001890.0002440.00010.0014230.000138

8.9e-05

0.0002740.000321

6e-06

0.0007220.000845

0.0057050.0030190.0003420.0001520.0063010.000191

0.0036840.004312

0.0001170.000137

0.0001920.000225

0.0001260.0002110.000114

0.0021210.0009640.0009080.0021170.0009950.0021610.000456

0.0002250.000264

5.8e-05

0.000120.0001275e-050.000136

0.0100140.011723

0.000220.000257

0.0037780.0011270.0008910.0008610.0043280.000445

0.0013030.001526

6.2e-05

0.0035730.0011950.0008760.0003940.004039

0.001880.002201

0.0001140.000134

9.3e-05

0.0003590.000421

0.0062510.007318

0.0004260.000498

4.2e-05

0.0007240.000847

0.000113

0.000210.000246

0.0017610.002061

0.0047670.0020680.0026270.0026210.0031680.0046560.001173

0.0003440.0002310.0001870.000348

0.0003440.0002310.0001870.000348

0.0003440.0002310.0001870.000348

0.0041560.0020450.0022050.0026210.0028090.0040480.001173

0.0001880.0001490.0001650.00017

0.0001880.0001490.0001650.00017

0.0039680.0020450.0020560.0026210.0026440.0038780.001173

0.0001420.0001230.0001420.000148

0.0038260.0020450.0019330.0026210.0025020.003730.001173

0.0002672.3e-050.0001910.0001720.00026

0.0002672.3e-050.0001910.0001720.00026

0.0002672.3e-050.0001910.0001720.00026

0.0192280.0087210.0314620.0141130.0262460.0194310.011165

0.0081170.0044120.0046410.0036290.0060970.0079690.000139

8.8e-050.0001440.0002750.000122

8.8e-050.0001440.0002750.000122

0.0080290.0044120.0044970.0036290.0058220.0078470.000139

7.6e-057.8e-058.7e-05

0

02.4e-05

0.0001570.0001446.5e-050.000184

8.9e-05

0.0069410.0044120.0042330.0036290.0046360.006804

1.3e-05

0001.5e-05

01e-060

0.000140.000120

1.4e-05

4.7e-05

9e-054.4e-058.4e-05

8e-05

7e-061.1e-057e-06

6.8e-059e-066.9e-051.4e-05

9.5e-053.3e-050.0001162.8e-05

2.5e-052.1e-052.8e-05

0

02.4e-05

6.5e-05

8e-064e-067e-06

7.5e-051.2e-058.2e-051.4e-05

1.7e-05

2e-05

0.000118

8e-06

1.4e-05

5.8e-056e-067.4e-05

7e-063.7e-051e-05

7.8e-058e-058.8e-05

0.0001450.0001050.000142

1.7e-05

3e-069e-063e-06

1.5e-059e-061.7e-05

6e-063e-066e-06

2.2e-059e-062.3e-056e-06

1.3e-058e-061.6e-05

7.9e-05

7.5e-05

0.0002370.0001810.0001770.000236

0.0002370.0001810.0001770.000236

0.0002370.0001810.0001770.000236

0.0039680.001050.003330.0048390.0041840.0041080.00033

0.0028840.0007710.0019850.0028230.0030430.0029810.000165

0.0005487.5e-050.0004250.0004130.0005596.9e-05

0.0006730.0001220.0002180.0002230.000689.6e-05

0.0001980.000232

0.0016630.0005740.0011440.0028230.0021750.001742

0.0010840.0002790.0013450.0020160.0011410.0011270.000165

0.0002520.000295

0.0002510.0002310.0001940.0020163.9e-050.0002450.00011

4.2e-05

0.0001472.4e-050.0001315.3e-050.0001614.1e-05

5.4e-05

0.0002270.0002042.5e-050.000252

6.4e-05

0.0002050.00024

0.0002830.0002040.0001820.000283

2.5e-05

3.9e-05

1.3e-05

0.0001762.4e-050.0001557e-050.0001861.4e-05

0.0005090.0005070.0005080.0004831e-06

0.0005090.0005070.0005080.0004831e-06

0.0001630.0001415.2e-050.000167

1e-06

0.0001440.0001180.000140.00013

0.0002020.000130.0001070.000186

7e-05

0.0001180.000139

0.0001270.0001190.000119

0.0001270.0001190.000119

0.0001270.0001190.000119

5.3e-05

5.3e-05

5.3e-05

0.006270.0032590.0228030.0056450.0151080.0065160.010695

0.006270.0032590.0228030.0056450.0151080.0065160.010695

0.0001676.9e-050.0001380.000140.000183

0.0024780.0013730.0015660.00150.0026041.3e-05

0.0003090.000362

0.0002160.000253

0.0002510.000150.0001090.000253

0.0001360.000159

0.0023760.002782

0.0004790.000350.0003080.000504

0.000130.000152

7.7e-05

9.7e-05

3e-069e-062e-064e-06

0.0001520.000178

0.000230.000269

0.0007750.0004950.0004720.000380.000777

0.0001710.000130.0001260.000169

0.000290.0001390.0001060.0002941.2e-05

0.0002140.000251

0.0003590.0008630.0002760.0001670.000413

0.000680.000796

0.000310.0001840.0001350.000299

0.0006620.000775

0.0090120.01055

1.6e-05

0.000140.0001160.0001270.000141

1.9e-051.2e-052.5e-05

0.0005356.3e-050.0003860.0003330.000565

0.0003880.000455

0.0010980.001286

0.0002480.00029

0.0002660.000312

0.0002320.000271

3.2e-056.5e-054e-063.4e-05

0.0002130.000249

4.3e-05

0.0012960.001517

6e-050.0001830.0003020.0056453.6e-056.2e-05

0.000123

0.0004590.000538

0.0001150.000134

2.7e-05

0.0002010.0001550.000160.000189

0.0001460.000171

0.0004850.0002030.0002780.0004280.000113

0.0004850.0002030.0002780.0004280.000113

0.0004850.0002030.0002780.0004280.000113

0.0004850.0002030.0002780.0004280.000113

0.0002840.0002220.0002310.000272

0.0002840.0002220.0002310.000272

0.0002840.0002220.0002310.000272

0.0002840.0002220.0002310.000272

0.2250540.1451840.1248480.086290.1646350.220990.070174

0.0006170.0001010.0002230.0004430.00062

0.0006170.0001010.0002230.0004430.00062

0.0006170.0001010.0002230.0004430.00062

0.0003930.0003160.0003180.00041

0.0003930.0003160.0003180.00041

0.0003930.0003160.0003180.00041

0.0002560.0003410.0003940.000243

0.0001310.000154

0.0001310.000154

0.0002560.000210.000240.000243

0.0002560.000210.000240.000243

0.2185480.1431340.114580.086290.1535940.2144180.068969

0.0003440.0003370.000330.00034

8.5e-050.0001630.0001916.4e-05

0.0002590.0001740.0001390.000276

0.0010319.6e-050.000270.0010560.0010890.000102

0.0010319.6e-050.000270.0010560.0010890.000102

0.0007970.0003610.0003730.0020160.0015890.000840.000135

0.0007970.0003610.0003730.0020160.0015890.000840.000135

0.0012060.001411

0.0012060.001411

0.0020410.0007370.0020820.0034080.0022226e-05

0.0010120.000330.0005660.0008990.0010984.6e-05

0.0008290.00097

0.0010290.0004070.0006870.0015390.0011241.4e-05

0.0006997.2e-050.002020.0022520.000674

0.0004550.000532

0.0010460.001225

0.0006997.2e-050.0005190.0004950.000674

0.2129370.140960.1078030.0842740.1429540.2085210.068658

6.7e-05

0.0007260.00085

9.3e-050.0006870.0001520.0001790.000102

3.1e-05

0.0006040.000708

0.0005760.000675

0.000560.000656

0.0013030.001525

0.2128440.1402730.1016280.0842740.1356240.2084190.068658

0.000440.000515

0.0018140.002124

0.000170.0007150.000190.00020.0001751.4e-05

0.000170.0007150.000190.00020.0001751.4e-05

0.0005290.0001930.0002990.0003940.000557

0.0005290.0001930.0002990.0003940.000557

0.0012990.0007570.0008460.0008590.0012977e-06

0.0012990.0007570.0008460.0008590.0012977e-06

0.000470.0002630.0003110.0003270.000501

0.0004220.0002370.0002710.000290.000389

0.0004070.0002570.0002640.0002420.0004077e-06

0.0013390.0003510.0042310.0053930.0013720.000115

0.0002070.000243

0.0002070.000243

0.0002496.5e-050.0002050.0002140.000261

0.0002496.5e-050.0002050.0002140.000261

0.0001670.000196

0.0001670.000196

0.0007390.0001820.0021980.0027890.0007585.9e-05

0.0007390.0001820.000350.0006240.0007585.9e-05

0.000620.000725

0.0004830.000566

0.0002760.000324

0.0002680.000314

0.0002010.000236

0.0002450.000286

0.0002450.000286

0.0002760.000324

0.0002760.000324

0.000250.000292

0.000250.000292

0.0004620.000541

0.0004620.000541

0.0002210.000338

7.9e-05

0.0002210.000259

0.0003510.0001040.000170.0003535.6e-05

0.0003510.0001040.000170.0003535.6e-05

9e-063.5e-056e-06

9e-063.5e-056e-06

9e-063.5e-056e-06

7e-050.0002510.0002946.7e-05

7e-050.0002510.0002946.7e-05

7e-050.0002510.0002946.7e-05

0.0004610.0007320.0007430.000544

0.0002150.000252

0.0002150.000252

0.0004610.0003410.0002850.000544

0.0004610.0003410.0002850.000544

0.0001760.000206

0.0001760.000206

0.0002870.000336

0.0002870.000336

0.0002870.000336

0.0003870.001730.0009030.000350.001076

0.000250.000293

0.000250.000293

0.0003870.0002890.0002920.00035

0.0003870.0002890.0002920.00035

0.0002720.000318

0.0002720.000318

0.0009190.001076

0.0009190.001076

0.0016750.0008410.0013110.0013230.0016637e-06

0.0006270.0002990.0003660.0003620.000615

0.0006270.0002990.0003660.0003620.000615

0.0010480.0005420.0009450.0009610.0010487e-06

0.0010480.0005420.0006270.0005890.0010487e-06

0.0003180.000372

0.000250.0002270.0002930.000174

0.000250.0002270.0002930.000174

0.000250.0002270.0002930.000174

0.0001080.0001090.0001588.6e-05

0.0001080.0001090.0001588.6e-05

0.0001420.0001180.0001358.8e-05

0.0001420.0001180.0001358.8e-05

0.0007470.0002460.0011280.000604

0.000360.0002460.0006140.000225

0.0002950.0002460.0004050.000173

0.0001210.0001120.0002618.8e-05

0.0001210.0001120.0001438.8e-05

0.000118

0.0001740.0001340.0001448.5e-05

0.0001740.0001340.0001448.5e-05

6.5e-050.0002095.2e-05

9.1e-05

9.1e-05

6.5e-050.0001185.2e-05

6.5e-050.0001185.2e-05

0.0003870.0005140.000379

0.0003870.0005140.000379

5.2e-055.6e-05

5.2e-055.6e-05

0.0001137.6e-059.8e-05

0.0001137.6e-059.8e-05

6.6e-050.0001015.6e-05

6.6e-050.0001015.6e-05

2.3e-055.7e-052.2e-05

2.3e-055.7e-052.2e-05

0.0001330.0001930.000147

7.7e-050.0001179.1e-05

5.6e-057.6e-055.6e-05

8.7e-05

8.7e-05

0.0002010.000330.0004520.000183

0.0002010.000330.0004520.000183

0.0002010.000330.0004520.000183

0.0002010.000330.0004520.000183

0.0002010.0001820.0002790.000183

0.0001480.000173

0.0049780.0006760.0008450.0066920.0050790.00033

0.0049780.0006760.0008450.0066920.0050790.00033

0.0049780.0006760.0008450.0066920.0050790.00033

0.0049780.0006760.0008450.0066920.0050790.00033

0.0049780.0006760.0008450.0066920.0050790.00033

0.011270.0596140.0133850.0540320.0092490.0101280.035609

7.7e-05

7.7e-05

7.7e-05

7.7e-05

7.7e-05

0.011270.0596140.0133850.0540320.0091720.0101280.035609

8.9e-050.0001889.2e-05

7.1e-050.0001677.4e-05

7.1e-050.0001677.4e-05

2.2e-05

1.3e-05

3.5e-051.4e-053.2e-05

1.7e-05

1.5e-05

1.1e-052.6e-051.3e-05

1.2e-05

6e-063e-065e-06

2.2e-05

4e-061e-053e-06

1.5e-051.3e-052.1e-05

1.8e-052.1e-051.8e-05

1.8e-052.1e-051.8e-05

1.8e-052.1e-051.8e-05

0.0109750.0596140.0133850.0540320.0086160.0098980.035609

0.0109750.0596140.0133850.0540320.0086160.0098980.035609

0.010920.0596140.0133850.0540320.0086090.0098650.035609

1.6e-05

2.6e-053.5e-052e-05

3e-064e-064e-06

2e-061.8e-053e-05

3.2e-05

0.0107770.0596140.0133850.0540320.008080.0097110.035609

1e-063e-06

5e-068e-067e-06

1.1e-051.5e-051.1e-05

6e-066e-069e-06

8e-06

6e-063e-066e-06

2e-064e-06

6e-062.8e-056e-06

2e-063e-062e-06

1e-064e-061e-06

01e-060

1e-062e-061e-06

1e-061e-06

7e-068e-065e-06

1.8e-055e-061.4e-05

3.7e-05

2e-064e-051e-06

7e-06

9e-063.6e-058e-06

04e-06

6e-062.2e-052e-06

5e-06

05e-061e-06

1.7e-05

4e-061e-061e-06

3e-06

02e-062e-06

1.6e-055.5e-051.1e-05

4e-069.6e-053e-06

4e-0604e-06

5.5e-057e-063.3e-05

2.5e-051e-068e-06

3e-056e-062.5e-05

0.0002060.0003680.000138

0.0002060.0003680.000138

0.0001055.4e-054.9e-05

3e-061e-063e-06

9.1e-052.2e-053.6e-05

8e-067e-067e-06

0

3e-062.4e-053e-06

0.0001010.0003148.9e-05

5.1e-05

2.1e-059.5e-051.1e-05

2e-059.7e-052.5e-05

6e-057.1e-055.3e-05

5.8e-050.0001523.3e-05

5.8e-050.0001523.3e-05

5.8e-050.0001523.3e-05

5.8e-050.0001523.3e-05

6e-063.5e-051e-06

6e-063.5e-051e-06

5e-064.1e-053e-06

5e-064.1e-053e-06

3.2e-053.5e-051.3e-05

3.2e-053.5e-051.3e-05

1.5e-054.1e-051.6e-05

1.5e-054.1e-051.6e-05

0.0003630.0005260.002016

0.0003630.0005260.002016

0.0003630.0005260.002016

0.0003630.0005260.002016

0.0003630.0005260.002016

0.0003630.0005260.002016

2.3e-050.000141.9e-05

2.3e-050.000141.9e-05

2.3e-050.000141.9e-05

1.5e-058.4e-051.1e-05

1.5e-058.4e-051.1e-05

1.5e-058.4e-051.1e-05

8e-065.6e-058e-06

8e-065.6e-058e-06

8e-065.6e-058e-06

0.0036860.0021390.0019180.0044350.0033170.003540.000994

0.0036860.0021390.0019180.0044350.0033170.003540.000994

0.0004610.0001110.000620.000382

0.0003920.000490.000326

0.0003920.000490.000326

4.7e-054.6e-053.7e-05

3.1e-053.4e-053e-05

5.2e-054.5e-055.4e-05

5.5e-053.4e-055e-05

3.4e-05

0.0001078.8e-057.4e-05

6e-056.4e-056.3e-05

4.7e-05

2.7e-05

1.7e-05

4e-055.4e-051.8e-05

6.9e-050.0001110.000135.6e-05

6.9e-050.0001110.000135.6e-05

6.9e-050.0001110.000135.6e-05

0.0032250.0021390.0018070.0044350.0026970.0031580.000994

0.0032250.0021390.0018070.0044350.0026970.0031580.000994

0.0002680.0001220.0001770.0019150.0003090.0002527.3e-05

0.0002080.0001220.0001770.0019150.0001760.0002047.3e-05

4.2e-05

1.3e-051.3e-059e-06

1.3e-053.1e-051.2e-05

4e-06

3.4e-054.3e-052.7e-05

0.0028220.0020170.001630.002520.0022180.0027720.000921

0.0028220.0020170.001630.002520.0022180.0027720.000921

0.0001147.5e-050.000107

0.0001147.5e-050.000107

2.1e-059.5e-052.7e-05

8e-065.8e-058e-06

1.3e-053.7e-051.9e-05

0.0319660.0162680.0760950.048890.0578910.0323110.054955

3.5e-050.0004863.6e-05

3.5e-050.0004863.6e-05

3.5e-050.0004863.6e-05

9e-065.5e-058e-06

9e-065.5e-058e-06

8e-066.7e-051e-05

8e-066.7e-051e-05

8e-060.0001738e-06

7.9e-05

8e-069.4e-058e-06

3.8e-05

3.8e-05

1e-050.0001531e-05

4.1e-05

3.5e-05

1e-055.3e-051e-05

2.4e-05

7.7e-059.5e-056.5e-05

7.7e-059.5e-056.5e-05

7.7e-059.5e-056.5e-05

2.2e-054.3e-052.1e-05

2.2e-054.3e-052.1e-05

5.5e-055.2e-054.4e-05

5.5e-055.2e-054.4e-05

0.0018750.0004270.0051720.0020160.0081820.0019580.00021

0.0018750.0004270.0051720.0020160.0081820.0019580.00021

9e-058.7e-050.000108

9e-058.7e-050.000108

2e-066e-064e-06

5e-064e-061.2e-05

1.6e-051.3e-051.1e-05

1.2e-053.3e-051.8e-05

1e-053e-069e-06

1.1e-057e-062.2e-05

1.4e-051.2e-051.5e-05

2e-059e-061.7e-05

7.3e-05

7.3e-05

7.3e-05

1.9e-055.6e-051.4e-05

1.9e-055.6e-051.4e-05

1.9e-055.6e-051.4e-05

1.2e-054.8e-051.1e-05

1.2e-054.8e-051.1e-05

1.2e-054.8e-051.1e-05

0.0017540.0004270.0051720.0020160.0079180.0018250.00021

0.0001990.0002140.0004540.00021

0.0001990.0002140.0004540.00021

0.0003230.000378

0.0003230.000378

1.6e-05

7e-06

5e-06

4e-06

0.0003080.000361

0.0003080.000361

2.7e-055.9e-052.9e-05

2.7e-055.9e-052.9e-05

0.0001790.0001980.000188

4.4e-051.9e-054.1e-05

2.3e-054.6e-052.7e-05

3.9e-051.4e-054.1e-05

5.2e-056.8e-055.4e-05

2.1e-055.1e-052.5e-05

7.4e-058.6e-057.4e-05

7.4e-058.6e-057.4e-05

2.1e-057.8e-052.3e-05

2.1e-057.8e-052.3e-05

8.9e-050.0001199.9e-05

2.2e-05

4.7e-053.9e-055.1e-05

4.2e-055.8e-054.8e-05

2.4e-050.0001512.5e-05

2.4e-054.5e-052.5e-05

2.8e-05

7.8e-05

0.000101

0.000101

0.0002820.000331

0.0001550.000182

0.0001270.000149

2.8e-05

6e-06

2.2e-05

7e-05

7e-05

2.2e-050.000132.6e-05

2.2e-055.8e-052.6e-05

7.2e-05

1.3e-051.7e-058e-061.5e-05

1.3e-051.7e-058e-061.5e-05

0.0002450.000287

0.0002450.000287

4.7e-050.0001120.0001315.9e-05

4.7e-050.0001120.0001315.9e-05

0.000117

0.000117

2.7e-050.0001173e-05

2.7e-050.0001173e-05

0.0013610.001593

0.0013610.001593

0.0015390.001801

0.0001960.00023

0.0003680.00043

0.000730.000854

0.0002450.000287

4.4e-050.000120.000144.3e-05

4.4e-050.000120.000144.3e-05

8.2e-050.000110.0001299.8e-05

8.2e-050.000110.0001299.8e-05

0.0007680.000410.0003850.0020160.0003450.0007640.00021

1.8e-057.1e-051.9e-05

7e-05

1.4e-05

1.4e-05

0.000750.000410.0003850.0020169.5e-050.0007450.000182

0.000109

1.9e-055.3e-052.8e-05

1.9e-055.3e-052.8e-05

2.4e-050.0001730.0002022.1e-05

2.4e-050.0001730.0002022.1e-05

3.6e-050.000174.1e-05

9.2e-05

3.6e-057.8e-054.1e-05

1.2e-050.0001031.5e-05

1.2e-050.0001031.5e-05

2.1e-056.1e-051.1e-05

2.1e-056.1e-051.1e-05

2.6e-050.0001012.6e-05

2.6e-050.0001012.6e-05

0.0150440.006250.0636250.0400190.0363950.0156460.050322

0.0150440.006250.0634980.0400190.0362060.0156460.050322

0.000510.000597

0.000510.000597

0.000510.000597

0.001120.0003650.0046080.0036290.0025890.0011780.003538

0.001120.0003650.0046080.0036290.0025890.0011780.003538

0.0030220.003538

0.000111

0.0003430.000401

0.000298.4e-050.0003110.0006460.000308

8.2e-050.0002520.0002957.9e-05

0.0002620.0001840.0001960.0002280.000261

0.0001130.0001270.000132

9.6e-050.0001550.0004460.000113

0.0002779.7e-050.0003290.0036290.0003350.000285

0.0001080.0001150.0002250.000116

0.0001080.0001150.0002250.000116

0.0001080.0001150.0002250.000116

3e-050.0001760.0002962.2e-05

3e-059e-052.2e-05

3e-059e-052.2e-05

0.0001760.000206

0.0001760.000206

5.2e-050.0001870.0002185.6e-05

5.2e-050.0001870.0002185.6e-05

5.2e-050.0001870.0002185.6e-05

0.0101640.0043440.0551720.032660.0293490.0107950.046136

0.0101640.0043440.0551720.032660.0293490.0107950.046136

2e-06

0.0013570.001589

1.4e-05

5e-06

0.0022880.0006750.0013110.0044350.001430.002407

2.2e-05

0.0051420.0013570.0032850.0086690.014010.005488

0.0008060.0002570.0004010.0004550.000842

4.3e-05

5e-06

0.0009130.0014470.0010920.0195560.0009670.0009631.4e-05

2.1e-05

0.0005020.000587

5e-06

0.0010150.0002950.0005870.0019050.001095

1e-06

8.6e-05

5e-06

0.0391890.045879

2e-06

1.4e-05

0.0003130.0002684e-06

0.0004540.000531

1.4e-05

0.0067260.007875

0.0006170.0008770.0016880.0005861.4e-05

0.0001580.000530.0013960.0001821.4e-05

0.000330.000386

0.0001580.00020.001010.000182

1.4e-05

0.0004590.0003470.0002920.000404

0.0001310.000153

0.0004590.0002160.0001390.000404

0.0001280.0002910.0004690.000125

0.0002910.000341

0.0002910.000341

0.0001280.0001280.000125

5.9e-053.4e-056e-05

6.9e-059.4e-056.5e-05

0.0028250.0015410.0015620.003730.0007750.0027680.000634

0.000128

0.000128

0.0028250.0015410.0015620.003730.0006470.0027680.000634

0.0002577.5e-050.0002730.0006470.000252

0.0025680.0014660.0012890.003730.0025160.000634

0.0001270.000189

4e-05

4e-05

4e-05

0.0001270.000149

0.0001270.000149

0.0001270.000149

0.0147540.0095910.0072980.0068550.011510.0144360.004423

0.0147540.0095910.0072980.0068550.011510.0144360.004423

0.0147540.0095910.0072980.0068550.011510.0144360.004423

5.2e-05

5.2e-05

0.0147490.0095910.0072980.0068550.0112460.0144310.004423

0.0147490.0095910.0072980.0068550.0112460.0144310.004423

3.7e-05

3.7e-05

0.00012

4.3e-05

7.7e-05

5e-065.5e-055e-06

5e-065.5e-055e-06

2e-058e-052.6e-05

2e-058e-052.6e-05

1.3e-054.1e-051.7e-05

1.3e-054.1e-051.7e-05

1.3e-054.1e-051.7e-05

7e-063.9e-059e-06

7e-063.9e-059e-06

7e-063.9e-059e-06

0.0001610.0011430.000144

0.0001610.0011430.000144

6.8e-050.0002457.1e-05

8e-064.5e-059e-06

8e-064.5e-059e-06

1e-052.6e-057e-06

1e-052.6e-057e-06

1.7e-054.8e-052.1e-05

1.7e-054.8e-052.1e-05

1.2e-052.3e-051.4e-05

1.2e-052.3e-051.4e-05

8e-066.2e-051.1e-05

8e-066.2e-051.1e-05

1.3e-059e-06

1.3e-059e-06

4.1e-05

4.1e-05

1.5e-050.0001691.7e-05

1.5e-054.5e-051.7e-05

1.5e-054.5e-051.7e-05

7.4e-05

7.4e-05

5e-05

5e-05

4e-050.0001191.9e-05

2.7e-057.5e-057e-06

2.7e-057.5e-057e-06

1.3e-054.4e-051.2e-05

1.3e-054.4e-051.2e-05

0.000335

7.6e-05

2.5e-05

3.3e-05

1.8e-05

6.3e-05

3.2e-05

3.1e-05

0.000196

2.8e-05

5e-06

5e-05

2.3e-05

1.7e-05

3e-05

4.3e-05

2.5e-050.0001872.5e-05

1.5e-052.8e-051.5e-05

1.5e-052.8e-051.5e-05

5e-068.1e-056e-06

5e-068.1e-056e-06

3.6e-05

3.6e-05

5e-064.2e-054e-06

2.5e-05

5e-061.7e-054e-06

5.8e-05

5.8e-05

5.8e-05

1.3e-053e-051.2e-05

1.3e-053e-051.2e-05

1.3e-053e-051.2e-05

6.1e-05

6.1e-05

6.1e-05

6.1e-05

6.1e-05

6.1e-05

1.8e-054e-059e-06

1.8e-054e-059e-06

1.8e-054e-059e-06

1.8e-054e-059e-06

1.8e-054e-059e-06

1.8e-054e-059e-06

0.0001440.0004090.000124

0.0001440.0004090.000124

6.2e-050.0001016e-05

6.2e-050.0001016e-05

4.9e-054.4e-055e-05

4.9e-054.4e-055e-05

1.3e-055.7e-051e-05

1.3e-055.7e-051e-05

8.2e-050.0003086.4e-05

7e-060.0001728e-06

01.5e-054e-06

01.5e-054e-06

3e-064e-062e-06

2e-064e-062e-06

1e-060

3e-068.9e-051e-06

3e-068.9e-051e-06

1e-066.4e-051e-06

3.8e-05

1e-062.6e-051e-06

5.2e-05

5.2e-05

5.2e-05

7.5e-058.4e-055.6e-05

1.6e-053.8e-052.8e-05

1.6e-053.8e-052.8e-05

5.9e-054.6e-052.8e-05

1.1e-051.7e-051.1e-05

4.8e-052.9e-051.7e-05

0.1785290.2149260.2720110.2755050.345150.1752630.34034

0.0002910.0013760.0007310.0002880.000984

0.0002910.0013760.0007310.0002880.000984

0.0002910.0013760.0007310.0002880.000984

0.0011520.0003640.000984

0.0003110.000364

0.0008410.000984

0.000119

0.000119

0.0002610.0002240.000263

0.0002610.0002240.000263

0.000121

0.000121

3e-050.0001272.5e-05

3e-053e-052.5e-05

9.7e-05

0.0521850.0446520.0578030.0346780.061810.0492670.037034

0.0002420.0008420.000235

9.9e-050.0002250.000115

3.7e-059.4e-054.2e-05

7e-063.8e-057e-06

1.1e-056e-061.3e-05

03e-060

01e-051e-06

6e-061.2e-056e-06

1.2e-056e-061.3e-05

1e-063e-061e-06

01.6e-051e-06

8e-067.6e-055e-06

8e-067.6e-055e-06

5.4e-055.5e-056.8e-05

4e-052.8e-055.3e-05

1.4e-052.7e-051.5e-05

0.00010.0003938.1e-05

4e-067.3e-054e-06

4e-067.3e-054e-06

5e-065e-053e-06

5e-065e-053e-06

3e-052.1e-051.5e-05

3e-052.1e-051.5e-05

1.9e-056.4e-057e-06

1.9e-056.4e-057e-06

1.3e-055.5e-051.3e-05

1.3e-055.5e-051.3e-05

7e-063.9e-051e-05

1.8e-05

1e-061.2e-052e-06

2e-067e-065e-06

4e-062e-063e-06

2.2e-059.1e-052.9e-05

2.2e-059.1e-052.9e-05

1.8e-050.0001091.3e-05

1.3e-054.8e-051.1e-05

1.3e-054.8e-051.1e-05

5e-066.1e-052e-06

5e-066.1e-052e-06

2.5e-050.0001152.6e-05

2.5e-050.0001152.6e-05

2.5e-050.0001152.6e-05

1.2e-056.1e-051e-05

1.2e-056.1e-051e-05

1.2e-056.1e-051e-05

1.2e-056.1e-051e-05

8.1e-050.0003289.8e-05

3.6e-050.0001973.8e-05

1.8e-050.0001222.1e-05

1.1e-057.1e-051.5e-05

7e-065.1e-056e-06

1.8e-057.5e-051.7e-05

1.8e-057.5e-051.7e-05

4.5e-050.0001316e-05

9e-067.3e-058e-06

9e-067.3e-058e-06

3.6e-055.8e-055.2e-05

3.6e-055.8e-055.2e-05

0.051850.0446520.0578030.0346780.0605790.0489240.037034

0.0007150.0033840.01240.0054440.0038210.000590.011542

1.7e-05

1.7e-05

2.1e-050.0034320.0002952.7e-050.003723

2.1e-050.0002520.0002952.7e-05

0.003180.003723

0.00030.0002040.000103

0.0002390.000204

8.8e-05

6.1e-05

1.5e-05

0.0002190.000256

0.0002190.000256

0.0004130.0001380.0005370.0054440.0007540.000412.9e-05

7.1e-05

0.0004136.7e-050.0005370.0054440.0007540.000412.9e-05

0.0001290.0052370.0004674.5e-050.005929

0.0007440.000871

0.0040020.004685

0.0001290.0001730.0004674.5e-05

0.0003180.000373

7.5e-050.0011810.0005590.000824

0.0007040.000824

0.0004770.000559

7.5e-05

0.0001140.0028710.0014790.001367.9e-050.000917

0.0004470.000523

8.7e-050.0028710.0007840.0005475.2e-050.000917

2.7e-050.0002480.000292.7e-05

3.8e-050.0001110.000132.9e-05

3.8e-050.0001110.000132.9e-05

2.3e-050.0001343e-05

5e-067.9e-053e-06

5e-067.9e-053e-06

1.8e-055.5e-052.7e-05

1.8e-055.5e-052.7e-05

0.0019870.001190.0013190.0023190.0020260.001963

2.7e-056.8e-053.8e-05

2.7e-056.8e-053.8e-05

1.6e-05

1.6e-05

4.9e-050.0001234.8e-05

3.5e-055.5e-053.3e-05

1e-052.9e-051e-05

4e-063.9e-055e-06

9e-066.6e-052e-05

9e-066.6e-052e-05

0.0017810.001190.0013190.0023190.0014480.001733

4e-065.6e-051.1e-05

1.1e-053.9e-051e-05

1.3e-05

1.2e-054.5e-051.4e-05

0.0017260.001190.0013190.0023190.0012590.001688

1.5e-054.9e-051e-05

6.2e-050.0002089.7e-05

7e-062.4e-057e-06

2.8e-058e-053e-05

2.7e-055.4e-056e-05

5e-05

4.1e-058.5e-052.5e-05

4.1e-058.5e-052.5e-05

2e-062.8e-052e-06

2.3e-05

1e-062e-061e-06

1e-063e-061e-06

3.7e-056.6e-053.2e-05

3.7e-056.6e-053.2e-05

3.7e-056.6e-053.2e-05

0.0005290.0001070.0023830.003040.0005486.9e-05

0.000260.0014180.001480.000263

0.0009550.001118

8.1e-050.0003090.0003628.1e-05

0.0001790.0001540.000182

3.5e-050.0007170.0010533.9e-05

0.0004920.000577

0.0002250.000263

6e-068.5e-056e-06

1.2e-053.1e-051.1e-05

1.2e-054.8e-051.6e-05

5e-064.9e-056e-06

0.000107

0.000107

0.0001240.0001270.0002520.000123

0.0001240.0001270.0002520.000123

6.9e-05

6.9e-05

0.000110.0001210.0002550.000123

0.000110.0001210.0002550.000123

0.0001160.0001169.8e-05

0.0001160.0001169.8e-05

0.0001160.0001169.8e-05

0.0001850.0001460.0007810.000194

2.1e-059.6e-052e-05

2.1e-059.6e-052e-05

0.0001640.0001460.0006850.000174

0.0001230.0001460.0005830.00013

4.1e-050.0001024.4e-05

0.0002750.000323

0.0002750.000323

0.0002750.000323

0.0035610.0075370.0024990.0046370.0020460.001630.004725

0.0033990.0075370.0023950.0046370.001650.0014720.004725

0.0033990.0075370.0023950.0046370.001650.0014720.004725

4.5e-050.0001040.0001224.6e-05

4.5e-050.0001040.0001224.6e-05

0.000104

0.000104

0.0001170.000170.000112

0.0001170.000170.000112

0.0002276e-050.0002670.0009980.0002381.5e-05

0.0002276e-050.0002670.0009980.0002381.5e-05

0.0002276e-050.0002670.0009980.0002381.5e-05

5.8e-050.0040220.0105690.0082255.5e-050.00024

0.0002050.00024

0.0002050.00024

5.8e-050.0001135.5e-05

5.8e-050.0001135.5e-05

0.005940.006954

0.005940.006954

0.0009890.001158

0.0009890.001158

0.0040220.003435

0.0040220.003435

0.0443070.0283520.0278310.0222780.0387730.0434290.020443

4.5e-054.9e-054.2e-05

1.6e-053e-062e-05

4e-06

2.9e-054.2e-052.2e-05

5.7e-050.0001586.3e-05

2.4e-057e-053.1e-05

3.3e-058.8e-053.2e-05

0.0442050.0283520.0278310.0222780.0384770.0433240.020443

4.8e-052.6e-053.3e-051.5e-05

1.8e-052.1e-052e-05

9.1e-054e-050.0001

7.2e-05

0.0023510.002753

3.8e-057e-054.5e-05

1e-05

7.7e-050.0001012e-068.1e-05

8.6e-050.0001050.0001740.0031251.9e-059.1e-05

2.9e-052.5e-051.4e-054.4e-05

6.5e-055.3e-056.5e-05

6.7e-05

4.5e-051.6e-054.9e-057e-06

6.7e-052.8e-053.5e-057.1e-05

7e-06

8.2e-05

0.003640.0051020.003659

8e-053.3e-058.4e-05

2e-05

6e-05

5e-053.9e-055.6e-05

8e-065.7e-059e-06

0.000104

2e-05

4.9e-053.9e-055.3e-05

0.0434540.0280930.0216660.0191530.0324760.0425230.014002

8.9e-05

8.9e-05

0.0001050.0001140.000230.000117

0.0001050.0001140.000230.000117

0.0001050.0001140.000230.000117

0.1234160.1690580.2107170.238710.2792260.1231230.301997

0.0485290.0461850.0579150.1215720.0359960.0455760.030865

0.0023280.0038690.0014070.0472780.0008750.0022890.002574

0.0022160.0038690.0014070.0472780.0006650.002170.002574

0.0001128.2e-050.000115

0.0001950.000360.0002390.0411293.5e-050.000158

4.5e-05

0.0004480.0002330.0002330.0020160.0001150.0004530.000246

0.0014220.0032760.0009350.0041330.0003240.0013930.002328

3.9e-052.8e-055.1e-05

3.6e-05

5.5e-059e-056.2e-05

5.5e-059e-056.2e-05

5.7e-050.000125.7e-05

5.7e-050.000125.7e-05

0.0002390.0005590.000208

6.1e-050.0002135e-05

2.9e-054.6e-052.5e-05

1.1e-053.1e-059e-06

1.2e-052.4e-056e-06

1e-064e-063e-06

6.7e-05

7e-063.9e-056e-06

1e-062e-061e-06

6.4e-050.0001125.7e-05

6.4e-050.0001125.7e-05

0.0001140.0002340.000101

3.8e-05

0.0001140.0001260.000101

7e-05

0.0362020.0394810.046170.0615930.0238260.0333110.026393

0.0001037e-060.0031280.0033270.0005240.0001921.5e-05

7.9e-050.0001169.7e-051.5e-05

0.0002860.000334

2.4e-057e-060.0028420.0033277.4e-059.5e-05

0.0360990.0394740.0430420.0582660.0233020.0331190.026378

0.0007510.000879

1.6e-05

0.0007610.0002010.0003930.0008310.0008236.4e-05

0.0002860.0003130.0006790.00031

4.2e-05

1.4e-05

5e-050.0001086.8e-054.5e-05

0.0004610.00054

0.0005080.000595

1.7e-05

0.000710.00010.0002430.0007640.000755.9e-05

0.0026930.004890.0021130.0040320.0012180.002658

0.0005570.0001460.0002890.0004730.0005860.000434

3.1e-050.0001880.0003130.0073598e-063.3e-058.9e-05

0.000108

0.0009170.0002490.0002330.0001540.0009230.000176

0.0005020.000190.0002570.0003330.0005510.00022

0.0015170.001776

0.0011140.001304

0.0003250.0002835.2e-050.0003372e-05

0.0001320.0001145.1e-050.000136

0.0002180.000255

0.0001343.2e-050.0001540.0002030.0002331.4e-05

0.0001550.0003470.0002650.0002880.0004790.000826

0.0027060.003168

0.0006240.0001380.0002620.0003870.0006292.3e-05

0.0002120.0001820.0002070.000221

0.0001713.8e-050.0002150.0030246.2e-050.0001756.3e-05

0.0001440.0001260.0001570.000142

0.0011270.00132

3.3e-05

0.0003520.0003790.0008430.000366

7.9e-05

0.00012

0.000670.0041160.0011750.0052421.8e-050.0006710.004549

5.9e-05

9.2e-055.7e-050.0005770.0115930.0004580.000461

0.0115720.0144510.0056190.0072580.0026390.0076760.009125

6.5e-05

0.005040.005901

0.0006440.0002370.0003190.0040320.0001320.0006570.001047

0.0002120.0001110.0001730.0006440.0002268.6e-05

0.000230.0001760.0001670.000235

0.0014220.001665

0.0136510.0136470.006940.0077620.0037650.013437

3.3e-05

6.3e-053.6e-050.0068020.0079645.5e-056e-05

2.5e-056e-050.0001220.0002220.000105

0.0001840.0001320.0001411.8e-050.000194

0.0028470.0027030.0037770.0127010.0038170.0030060.001764

0.0027110.0027030.0037770.0127010.0037380.0028830.001764

2.4e-056.3e-052.6e-05

6.6e-05

0.0002150.0001890.0002210.0002284.4e-05

2.8e-05

03e-060

2.7e-050.0001320.0001552.4e-05

0.0003990.0013320.0005980.002525.3e-050.000390.001083

7.3e-05

7.7e-05

3.7e-05

3e-066e-063e-06

2.2e-05

0.0003650.0003990.0002780.0020160.0001440.0003610.000332

0.0003920.000459

0.0002770.0002550.0003370.000288

7.6e-05

4.3e-05

0.0002380.0003630.0002150.0001980.0002567.9e-05

1.8e-05

1.7e-051.6e-051.6e-05

7.2e-054.6e-059.7e-05

0.0003630.0003420.0004010.0081658.3e-050.0003688.6e-05

4.5e-053.7e-054.4e-05

1.1e-053.2e-051e-05

4.5e-05

9.9e-056e-050.000113

1.4e-051e-051e-05

2.8e-05

7.8e-05

2.1e-05

2.6e-059.9e-050.000105

8.6e-05

0.0008420.000985

2.8e-05

1e-064e-060

0.0003960.0003470.000418

0.0001190.0002670.0001283.9e-050.000126

0.000102

0.0001367.9e-050.000123

7.7e-052.4e-057.2e-05

5.9e-054.2e-055.1e-05

1.3e-05

0.0001560.0001320.0015590.0022850.000122

0.0001560.0001320.0015590.0022850.000122

8.5e-05

0.0002860.000335

0.0001560.0001320.0002540.000122

0.0006810.000797

0.000121

0.0005920.000693

0.0067570.0050020.0046340.006640.000134

1.5e-05

1.5e-05

0.0067570.0050020.0046340.006640.000119

0.000102

9.4e-050.0001130.0001820.000104

0.006620.0046720.004110.006487

4.3e-058.8e-054.9e-051.7e-05

0.0002170.000254

0.07488700000000010.1228730.1528020.1171380.243230.0775470.271132

0.0010370.0010340.0010280.0023190.0019340.0020440.00063

0.0010320.0010340.0010280.0023190.0018560.002040.00063

0.0010320.0010340.0010280.0023190.0018560.002040.00063

5e-067.8e-054e-06

5e-067.8e-054e-06

2.3e-055e-051.6e-05

2.3e-055e-051.6e-05

2.3e-055e-051.6e-05

0.0004622e-060.000120.0004070.000527

9.2e-05

9.2e-05

8.1e-050.000120.000147.4e-05

8.1e-050.000120.000147.4e-05

0.0003812e-060.0001750.000453

8.4e-056e-068.9e-05

2e-062e-068e-064e-06

6e-06

9e-06

1.2e-05

2.6e-058e-062.7e-05

0

4.7e-05

2.8e-054e-062.1e-05

1.1e-05

9e-06

9e-063e-067e-06

1e-05

6.2e-056e-066.2e-05

7.6e-051.1e-050.000105

2.8e-056e-066e-05

4.5e-051.5e-055.6e-05

3e-06

2.1e-051e-062.2e-05

8.4e-050.0005559.4e-05

7.1e-05

7.1e-05

0.000103

0.000103

0.00013

1e-05

2e-05

1.1e-05

1.2e-05

1.7e-05

2e-05

1.8e-05

7e-06

1.5e-05

8.4e-053.5e-059.4e-05

8.4e-053.5e-059.4e-05

0.000118

0.000118

9.8e-05

9.8e-05

0.0270720.0944560.0114860.0896170.0096380.0294630.172636

0.0171990.0883480.0052420.0830640.00310.0196590.17218

1.5e-05

0.0018680.0014070.0005950.0156250.0001480.0018880.129327

8.6e-05

6.2e-05

5e-06

0.000140.0001177.3e-050.000133

0

0.0001520.0001393e-060.000176

6.2e-05

5e-062e-066e-06

0.0001133.7e-053.5e-050.000102

0.0003670.0001810.0001090.000443

5.9e-056.1e-056.4e-05

3.5e-05

0.0091140.0516110.0008110.020060.0001660.0107820.039444

7.6e-05

7.1e-05

1.4e-052e-061.4e-05

8e-067e-063e-068e-06

0.000123

0.0022290.0324080.00050.0146170.0001110.0028150.003128

6.5e-051.3e-056.5e-055.9e-05

0.0002320.0023650.0003110.007469e-060.000230.000222

7.7e-05

6.8e-05

6.3e-057e-056.3e-05

5e-06

0.0004157.5e-050.0005180.0040325.6e-050.000438

4.2e-053.7e-054.4e-05

0.0009840.0003510.0004340.021270.0001980.001133

8.9e-05

8.2e-05

7.7e-051.9e-057.4e-05

0.0005810.00068

0.000150.0001170.0001130.000158

0.0001350.0001151.1e-05

2e-06

0

3.2e-056.2e-053e-05

7.4e-05

8.3e-05

0.0009358.7e-050.0008236.8e-050.000993

3.6e-05

2.4e-056.1e-052.1e-05

2.4e-056.1e-052.1e-05

5.2e-05

5.2e-05

0.0020110.0006490.0019760.0027220.0021840.0020620.000252

0.0014090.00165

0.0020110.0006490.0005670.0027220.0004580.0020620.000252

7.6e-05

0

7.4e-05

7.4e-05

6.8e-05

8e-06

4.1e-05

1.9e-05

7.9e-05

7.9e-05

2e-067e-063e-067e-06

2e-067e-063e-067e-06

7.3e-05

3.4e-05

3.9e-05

0.0076490.0054590.0042680.0038310.0036510.0075180.000197

01.4e-05

3e-064e-061.1e-05

3e-06

7.5e-053.6e-057.2e-05

9e-06

1.1e-05

9e-062.2e-051.4e-057e-06

0.0075130.0054590.0042680.0038310.0034770.00736

3e-068e-063e-061.6e-05

2.4e-053.1e-053.1e-057e-06

5e-065e-066e-063.7e-05

2.2e-05

7e-06

1.4e-05

1.7e-053e-062.1e-059.5e-05

2e-05

9.5e-057.3e-059.4e-05

9.5e-057.3e-059.4e-05

8.3e-05

8.3e-05

9.2e-050.0001330.000106

9.2e-050.0001330.000106

0.000140.0001590.0003250.000169

0.000140.0001390.000169

3.4e-054e-064.3e-05

1.8e-051.7e-052.8e-05

8.2e-059.6e-058.5e-05

6e-061.9e-051.3e-05

3e-06

0.0001590.000186

0.0001590.000186

0.0459440.0273780.1400090.0252020.2302740.0451140.097866

0.00011

0.00011

0.0451240.0272370.0277170.0252020.0308440.044283e-05

6.8e-05

9.5e-05

5e-05

6e-05

6.5e-05

5.1e-05

1.2e-05

2.4e-05

7e-06

2.4e-05

8e-06

3e-058.1e-053.4e-051.5e-05

0.0444810.0269940.027390.0252020.0295930.043609

2.3e-05

4.3e-05

1.9e-05

9.3e-057.1e-054e-054.7e-051.5e-05

1.1e-05

2.4e-05

9.1e-054.6e-059.4e-05

2.2e-05

2e-053.3e-051.8e-05

1.6e-05

0.000120.00014

0.0004090.0001720.0002070.0001640.000478

1.8e-05

3.6e-05

7.1e-05

0.0007650.0001410.1122920.1992110.0007860.097836

0.0007650.0001410.0002470.0002420.000786

0.1120450.1989690.097836

5.5e-050.0001094.8e-05

5.5e-050.0001094.8e-05

0.0001253e-064.7e-050.00012

0.0001253e-064.7e-050.00012

3.9e-055e-063.9e-05

3.9e-051.2e-053.7e-05

1.5e-051.7e-051.4e-05

3e-051.2e-052.8e-05

2e-063e-061e-062e-06

0.0008910.0004110.0006180.0021170.001380.0008770.000325

0.0007340.0004110.0005120.0021170.0006820.0007160.000325

0.0007340.0004110.0005120.0021170.0006820.0007160.000325

0.0007340.0004110.0005120.0021170.0006820.0007160.000325

5.1e-050.0001115.2e-05

0.0001390.000162

0.0006830.0004110.0003730.0021170.0005710.0006640.000163

7.9e-050.0003688.4e-05

0.000111

0.000111

4.5e-05

6.6e-05

7.9e-050.0002578.4e-05

7.9e-050.0002578.4e-05

3.8e-05

2e-05

1.3e-05

3.7e-059.3e-054e-05

4.2e-059.3e-054.4e-05

7.8e-050.0001060.000337.7e-05

7.8e-050.0001060.000337.7e-05

4.9e-050.0001060.0001244.7e-05

4.9e-050.0001060.0001244.7e-05

2.9e-050.0001083e-05

2.9e-050.0001083e-05

9.8e-05

9.8e-05

0.000109

0.000109

0.000109

0.000109

0.000109

0.0017460.0008050.0014970.0018940.001708

0.0017460.0008050.0014970.0018940.001708

0.0017460.0008050.0014970.0018940.001708

0.0013890.0006820.0008760.0010110.001378

0.0013890.0006820.0008760.0010110.001378

0.000280.000328

0.000280.000328

0.0001340.000157

0.0001340.000157

0.0003570.0001230.0002070.0003980.00033

0.0003570.0001230.0002070.0003980.00033

0.0001010.0003339.6e-05

0.0001010.0003339.6e-05

5e-059.2e-054.7e-05

5e-059.2e-054.7e-05

3.7e-053e-053.4e-05

3.7e-053e-053.4e-05

1.3e-053.4e-051.3e-05

1.3e-053.4e-051.3e-05

2.8e-05

2.8e-05

9e-069.2e-059e-06

9e-069.2e-059e-06

3e-066.5e-054e-06

2.5e-05

3e-064e-054e-06

6e-062.7e-055e-06

6e-062.7e-055e-06

4.2e-050.0001494e-05

2.1e-057.9e-052e-05

9e-063.7e-058e-06

1e-05

6e-061.6e-056e-06

3e-061.1e-052e-06

1.2e-054.2e-051.2e-05

2e-061.9e-053e-06

1e-052.3e-059e-06

2.1e-057e-052e-05

2e-054.2e-052e-05

00

1.8e-053e-051.7e-05

2e-069e-063e-06

3e-06

1e-062.8e-050

00

000

1.2e-05

2e-06

0

1.2e-05

1e-06

0

1e-0600

1e-06

0.0035380.0004010.0216020.0020160.0254490.0036938e-05

0.0034580.0004010.0216020.0020160.0253210.0036348e-05

0.0002840.0003640.000302

8e-050.0001339.5e-05

8e-050.0001339.5e-05

2.3e-052.7e-052.5e-05

2e-053e-051.7e-05

1.9e-053.8e-052.3e-05

5e-061e-066e-06

1.2e-053.3e-052.1e-05

1e-064e-063e-06

4.1e-056.5e-054.2e-05

4.1e-056.5e-054.2e-05

1.8e-053.5e-051.9e-05

2.3e-053e-052.3e-05

6.5e-056.6e-056.4e-05

3.3e-054.2e-053.2e-05

3.3e-054.2e-053.2e-05

1.1e-052.4e-051.2e-05

1.1e-052.4e-051.2e-05

2.1e-052e-05

2.1e-052e-05

5.9e-054.2e-056.8e-05

5.9e-054.2e-056.8e-05

5.9e-054.2e-056.8e-05

3.9e-055.8e-053.3e-05

3.9e-055.8e-053.3e-05

3.9e-055.8e-053.3e-05

0.0006930.0001380.0002350.0020160.0011240.0006676.7e-05

2.4e-052.4e-05

2.4e-052.4e-05

2.4e-052.4e-05

0.0003030.0001380.0002350.0020160.0002620.0003026.7e-05

0.0003030.0001380.0002350.0020160.0002620.0003026.7e-05

0.0003030.0001380.0002350.0020160.0002620.0003026.7e-05

0.0003280.0006620.000304

7.4e-057.4e-057.3e-05

7.4e-057.4e-057.3e-05

0.0002040.0004960.000182

1e-051.1e-052.1e-05

3e-06

3e-05

1.2e-055.4e-051.5e-05

2.6e-05

3e-06

1.9e-053e-062.1e-05

2.5e-056.2e-052.2e-05

3e-055.5e-052.4e-05

1e-052.4e-053e-06

6e-063.2e-057e-06

0

2.8e-051e-061.1e-05

5e-06

1.5e-057.1e-051.9e-05

2.8e-054.9e-051.2e-05

9e-0601.2e-05

1.5e-05

6e-06

5e-06

1.2e-053.9e-051.5e-05

2e-06

1.3e-053.3e-051.4e-05

1.3e-053.3e-051.4e-05

3.7e-055.9e-053.5e-05

1.7e-051.6e-05

2e-055.9e-051.9e-05

7e-060.0001258e-06

7e-060.0001258e-06

7e-063e-058e-06

5.6e-05

3.9e-05

7e-062.4e-057e-06

7e-062.4e-057e-06

7e-061.3e-057e-06

3e-06

8e-06

2.1e-053.9e-052e-05

2.1e-053.9e-052e-05

2.1e-053.9e-052e-05

3e-061.2e-052e-06

3e-061.2e-052e-06

3e-064e-062e-06

8e-06

1.4e-05

1.4e-05

1.4e-05

1.4e-05

0.0001240.0002080.00013

2.9e-056.9e-052.9e-05

1.5e-053.3e-051.7e-05

1.5e-053.3e-051.7e-05

1.4e-053.6e-051.2e-05

1.4e-053.6e-051.2e-05

1.4e-052.5e-051.6e-05

1.4e-052.5e-051.6e-05

1.4e-052.5e-051.6e-05

6e-059.7e-056.2e-05

6e-055.7e-056.2e-05

6e-055.7e-056.2e-05

4e-05

2.4e-05

1.6e-05

2.1e-051.7e-052.3e-05

2.1e-051.7e-052.3e-05

1e-05

2.1e-057e-062.3e-05

0.0021760.0002630.0213670.0234410.0023421.3e-05

0.0016730.0002630.0014330.0004020.0017831.3e-05

0.0004129.8e-050.0002930.000120.000365

9e-054.8e-050

0.0001945e-050.0001767.3e-050.000219

0.0001280.0001171.9e-050.000146

2.8e-05

0.000930.0001650.0008380.0002620.0010391.3e-05

0.000233.6e-050.0002034.8e-050.0002451.3e-05

0.0002665.2e-050.0002417.9e-050.000299

0.0001963.2e-050.0001794.2e-050.000227

4.3e-05

0.0002384.5e-050.0002155e-050.000268

0.0002120.0001940.000245

0.0002120.0001940.000245

0.0001190.0001082e-050.000134

0.0001190.0001082e-050.000134

0.0005030.0004520.0002310.000559

0.0001330.0001175.2e-050.000143

0.0001330.0001175.2e-050.000143

0.000370.0003350.0001790.000416

0.0002520.0002296.6e-050.000286

6e-05

0.0001180.0001065.3e-050.00013

0.0194820.022808

0.0194820.022808

0.0194820.022808

6.3e-056.4e-056.9e-05

6.3e-056.4e-056.9e-05

6.3e-056.4e-056.9e-05

6.3e-056.4e-056.9e-05

0.0001180.0001060.000124

5.4e-055.9e-055.7e-05

5.4e-055.9e-055.7e-05

5.4e-055.9e-055.7e-05

6.4e-054.7e-056.7e-05

6.4e-054.7e-056.7e-05

6.4e-054.7e-056.7e-05

8e-050.0001285.9e-05

8e-050.0001285.9e-05

8e-050.0001285.9e-05

8e-050.0001285.9e-05

3e-055.6e-052.6e-05

5e-057.2e-053.3e-05

3.9e-050.0001032.5e-05

3.9e-050.0001032.5e-05

3.9e-050.0001032.5e-05

3.9e-050.0001032.5e-05

3.9e-050.0001032.5e-05

3.9e-050.0001032.5e-05

0.0001020.000460.00011

0.0001020.000460.00011

0.0001020.000460.00011

5.1e-057.1e-056.1e-05

2.1e-052.4e-052.2e-05

6e-06

2.1e-051.8e-052.2e-05

3e-054.7e-053.9e-05

3e-054.7e-053.9e-05

5.1e-050.0003894.9e-05

0.000104

0.000104

2.6e-057.2e-052.9e-05

2.6e-057.2e-052.9e-05

2.2e-050.0001431.7e-05

2.2e-054.7e-051.7e-05

4.3e-05

5.3e-05

3e-067e-053e-06

3e-067e-053e-06

0.0002630.0003080.000194

0.0002630.0003080.000194

0.0002630.0003080.000194

0.0002630.0003080.000194

4.3e-054.4e-053.8e-05

2.6e-052.4e-052.2e-05

1.7e-052e-051.6e-05

4.8e-050.0001014.6e-05

1e-052.7e-056e-06

1.1e-052.5e-059e-06

6e-062.9e-055e-06

2.1e-052e-052.6e-05

3e-064.3e-052e-06

3e-064.3e-052e-06

0.0001476.4e-058.8e-05

8e-06

5.2e-053e-053.6e-05

9.5e-052.6e-055.2e-05

2.2e-055.6e-052e-05

2.9e-05

2.2e-052.7e-052e-05

0.0032110.0017110.0016420.0026210.0028940.0031210.000914

0.0031410.0017110.0016420.0026210.0027780.0030740.000914

0.0031410.0017110.0016420.0026210.0027780.0030740.000914

0.0031160.0017110.0016420.0026210.0026730.0030540.000914

1e-053.9e-055e-06

1e-053.9e-055e-06

1.7e-055.3e-051.6e-05

1.7e-055.3e-051.6e-05

0.0030840.0017110.0016420.0026210.0024530.003030.000914

0.0030840.0017110.0016420.0026210.0024530.003030.000914

5e-065.3e-053e-06

5e-065.3e-053e-06

7.5e-05

7.5e-05

2.5e-050.0001052e-05

3e-066.4e-052e-06

3e-066.4e-052e-06

2.2e-054.1e-051.8e-05

2.2e-054.1e-051.8e-05

7e-050.0001164.7e-05

7e-050.0001164.7e-05

7e-050.0001164.7e-05

7e-050.0001164.7e-05

7e-050.0001164.7e-05

4.8e-050.0002623.9e-05

4.8e-050.0002623.9e-05

4.8e-050.0002623.9e-05

4.8e-050.0002623.9e-05

9e-060.0001565e-06

5.9e-05

4.7e-05

9e-065e-055e-06

2.7e-054.8e-052.4e-05

6e-061.2e-054e-06

2.1e-053.2e-052e-05

4e-06

1.2e-055.8e-051e-05

1.2e-055.8e-051e-05

1.1e-056.5e-059e-06

6.5e-05

6.5e-05

6.5e-05

6.5e-05

6.5e-05

1.1e-059e-06

1.1e-059e-06

1.1e-059e-06

1.1e-059e-06

1.1e-059e-06

5.1e-050.0001324.3e-05

5.1e-050.0001324.3e-05

5.1e-050.0001324.3e-05

5.1e-050.0001324.3e-05

3.2e-05

3.2e-05

3.1e-055.8e-052.6e-05

3.1e-053.7e-052.6e-05

2.1e-05

2e-054.2e-051.7e-05

2e-054.2e-051.7e-05

0.0029010.0018160.0016910.0026210.0020590.0028410.001102

0.0029010.0018160.0016910.0026210.0020590.0028410.001102

0.0029010.0018160.0016910.0026210.0020590.0028410.001102

0.0029010.0018160.0016910.0026210.0020590.0028410.001102

0.0029010.0018160.0016910.0026210.0020590.0028410.001102

0.0028990.0018160.0016910.0026210.0020270.0028370.001102

2e-063.2e-054e-06

2.3e-050.0001180.0001382.5e-05

2.3e-050.0001180.0001382.5e-05

2.3e-050.0001180.0001382.5e-05

2.3e-050.0001180.0001382.5e-05

2.3e-050.0001180.0001382.5e-05

2.3e-050.0001180.0001382.5e-05

0.3669020.4499090.3406760.4308470.2293430.3622550.413353

0.00018.8e-059e-05

0.00018.8e-059e-05

0.00018.8e-059e-05

0.00018.8e-059e-05

7.1e-053.4e-056.3e-05

8e-064e-057e-06

2.1e-051.4e-052e-05

0.1463950.1499390.1256080.2376010.09979200000000010.1445210.112515

0.0002950.0010680.0061280.0092740.0005280.0003470.000819

9e-050.0057690.0067540.0002945.4e-05

1.7e-056.8e-052.7e-05

1.7e-056.8e-052.7e-05

1.1e-05

1.1e-05

01.8e-051e-06

07e-060

01.1e-051e-06

4.1e-050.0057690.0067540.0001539e-06

2e-058.4e-05

2.1e-050.0057690.0067546.9e-059e-06

3.2e-054.4e-051.7e-05

3.2e-052.6e-051.7e-05

1.8e-05

0.0002050.0010680.0003590.002520.0001180.0002930.000819

1.3e-05

1.3e-05

0.0002050.0010680.0003590.002520.0001050.0002930.000819

1e-063e-062e-06

1.7e-05

2.3e-059e-062.1e-05

7e-06

0.0001780.0010680.0003590.002526.8e-050.0002670.000819

3e-061e-063e-06

0.000116

5.9e-05

5.9e-05

5.7e-05

5.7e-05

0.0001180.0002490.0001194e-06

0.0001180.0002490.0001194e-06

1.1e-055.7e-051e-05

1.1e-055.7e-051e-05

1e-059.2e-051.2e-05

1e-053e-051.2e-05

2.7e-05

3.5e-05

9.7e-050.00019.7e-054e-06

9.7e-050.00019.7e-054e-06

0.0036360.0025820.00150.0028230.0005980.0034960.001112

0.0036360.0025820.00150.0028230.0005980.0034960.001112

4.4e-058.5e-053.1e-05

4.4e-058.5e-053.1e-05

0.000180.0001170.0001110.000137

0.000180.0001170.0001110.000137

0.0034120.0025820.0013830.0028230.0004020.0033280.001112

0.0004859.9e-050.0001952.1e-050.0004510.000112

9.4e-05

0.0003387.2e-050.0002784.5e-050.0003144.3e-05

0.0002290.000268

0.0025890.0024110.0006810.0028230.0002210.0025630.000689

2.1e-05

0.0012650.0011680.0008630.0023190.001120.0012250.000783

0.0010440.0011680.0007510.0023190.000760.0010180.000783

6.6e-050.0001325.9e-054e-05

6e-05

6.6e-051.7e-055.9e-054e-05

0

0

5.5e-05

0.0004580.0011680.0004410.0023190.000220.0004490.000721

0.0004580.0011680.0004410.0023190.000220.0004490.000721

0.0001490.0001580.000155

2.3e-051.5e-052.4e-05

7.5e-05

9e-062e-061.1e-05

00

1.3e-057e-061.1e-05

9e-063e-061.7e-05

1e-0601e-06

1e-058e-069e-06

8.4e-051.7e-058.2e-05

2.2e-05

9e-06

0.0003710.000310.0001390.0003552.2e-05

0.000150.0001297.2e-050.000152

0.0002210.0001816.7e-050.0002032.2e-05

0.000111

4.2e-05

2.5e-05

4.4e-05

0.0002210.0001120.000360.000207

8.4e-055.2e-058.1e-05

8.4e-055.2e-058.1e-05

0.0001370.0001125.7e-050.000126

0.0001370.0001125.7e-050.000126

8.7e-05

8.7e-05

0.000164

2.9e-05

5.5e-05

8e-05

0.0113830.008040.0061460.007560.0081020.011030.004648

3e-050.0001123.4e-05

3e-050.0001123.4e-05

1.6e-055.9e-051.8e-05

1.4e-055.3e-051.6e-05

2.7e-058.9e-052.4e-05

2.7e-058.9e-052.4e-05

2.7e-054.6e-052.4e-05

4.3e-05

0.0003110.0002680.0002571.4e-05

0.0003110.0002680.0002571.4e-05

3.8e-053e-051.8e-05

02e-060

1e-051.6e-059e-06

1.6e-053.9e-051.2e-05

6e-064e-065e-06

2.2e-052.9e-051.9e-05

1.2e-051.2e-05

9.1e-053.6e-057e-051.4e-05

2.8e-051.1e-052.1e-05

1.9e-051.5e-052.5e-05

9e-062.8e-058e-06

1.1e-051e-059e-06

1.7e-052.2e-051.5e-05

1e-063e-062e-06

4e-061e-065e-06

1.6e-052.2e-051.2e-05

1.1e-0501.5e-05

1.3e-054.3e-051e-05

1.3e-054.3e-051e-05

1.3e-054.3e-051e-05

3.1e-050.0002111.9e-05

3.1e-050.0002111.9e-05

2.2e-05

3.8e-05

7e-06

3.4e-05

6e-06

1.3e-054.7e-058e-06

1.3e-052e-068e-06

9e-06

5e-068e-063e-06

3.8e-05

4.6e-05

4.6e-05

4.6e-05

0.00018e-057.8e-05

0.00018e-057.8e-05

0.00018e-057.8e-05

0.0108710.008040.0061460.007560.0072530.0106080.004634

0.0031210.0030450.0021840.0035280.0021750.0030330.00194

4.5e-05

5.1e-051.9e-054.3e-05

2.6e-05

3.8e-05

7.7e-053.5e-056.4e-05

0.000128

4.4e-05

0.0029930.0030450.0021840.0035280.001840.0029260.00194

6e-05

6e-05

0.0077070.0049950.0039620.0040320.0048160.0075410.002694

0.0077010.0049950.0039620.0040320.0047250.0075370.002694

6e-069.1e-054e-06

1.5e-057.4e-051e-05

1.5e-057.4e-051e-05

7.6e-05

7.6e-05

2.8e-055.2e-052.4e-05

3e-063e-063e-06

1.6e-051.5e-051.5e-05

9e-061e-056e-06

1e-06

0

2.2e-05

1e-06

8.8e-05

8.8e-05

8.8e-05

8.8e-05

0.0001460.0136210.0012540.000130.015017

0.0003840.000449

0.00020.000234

0.00020.000234

0.0001840.000215

0.0001840.000215

0.0128270.015017

0.0128270.015017

0.0128270.015017

0.0002430.000285

0.0002430.000285

0.0001120.000131

0.0001310.000154

0.0001460.0001670.000520.00013

5.4e-050.0001670.0001955.4e-05

5.4e-050.0001670.0001955.4e-05

2.7e-050.0001022.3e-05

2.7e-050.0001022.3e-05

3.2e-050.0001082.9e-05

3.2e-050.0001082.9e-05

3.3e-050.0001152.4e-05

3.3e-050.0001152.4e-05

0.0136030.0073010.0069330.0045360.009670.0133660.000172

0.0136030.0073010.0069330.0045360.009670.0133660.000172

1.6e-055e-052.8e-051.8e-05

1.3e-052.3e-051.2e-051.4e-05

1.5e-05

3e-061.2e-051.6e-054e-06

2.3e-056.2e-051.8e-05

3.4e-05

2.3e-052.8e-051.8e-05

6.8e-05

6.8e-05

0.0135640.0073010.0069330.0045360.009490.013320.000154

1.2e-053e-061.6e-05

3.5e-05

3.8e-05

2.8e-05

1.4e-051.4e-05

2.1e-051.9e-051.1e-05

8e-06

1.6e-053e-061.5e-051.4e-05

7e-06

9e-066e-062e-068e-062.8e-05

5e-062e-064e-066e-06

5e-06

0.0134020.0072950.0069330.0045360.0092630.013174

8e-063e-068e-06

2e-056e-06

1.4e-05

2.5e-05

1.2e-056e-061.1e-058e-06

5.4e-051.4e-054.7e-05

2.7e-05

1.6e-051e-051.1e-05

1.1e-05

9e-067e-061.5e-054e-06

1.5e-055.3e-051.2e-05

1.5e-055.3e-051.2e-05

1.5e-055.3e-051.2e-05

1.5e-055.3e-051.2e-05

0.0008960.0024920.0023070.0029230.000470.0007940.001787

0.0008960.0024920.0023070.0029230.000470.0007940.001787

1.6e-056.2e-055e-06

1.6e-056.2e-055e-06

1.9e-054.5e-052e-05

1.9e-054.5e-052e-05

7.6e-058.3e-057.7e-05

3.4e-057e-062.8e-05

3.3e-056.2e-054e-05

9e-066e-069e-06

8e-06

4e-062.5e-055e-06

4e-062e-065e-06

6e-06

1.7e-05

1.1e-052e-051.3e-05

1.1e-052e-051.3e-05

6e-062.4e-059e-06

6e-062.4e-059e-06

2.3e-051e-052.9e-05

2.3e-051e-052.9e-05

0.0006650.0024920.0023070.0029230.0001710.0005850.001787

3.9e-051.6e-051.6e-05

0.0015260.001787

0.0005860.0024920.0007810.0029239.8e-050.000519

2.1e-051e-051.3e-05

1.9e-054.7e-053.7e-05

7.6e-053e-055.1e-05

4.1e-052.1e-053.9e-05

3.5e-059e-061.2e-05

0.0001520.0018360.0027990.000139

0.0001520.0018360.0027990.000139

0.0016840.002093

0.0016840.001972

0.000121

9.2e-05

9.2e-05

7.8e-05

7.8e-05

3.8e-051.5e-055e-05

3.8e-051.5e-055e-05

0.0001520.000178

0.0001520.000178

0.0001140.0003438.9e-05

4.5e-05

6e-055.5e-054.3e-05

8.8e-05

3.1e-050.0001152.7e-05

2.3e-054e-051.9e-05

0.0616210.0858070.0520060.0675410.0358960.0592560.046214

0.0009970.0006280.0040560.0043350.0025630.0010280.000664

1e-050.0001291e-05

5.4e-05

5.9e-05

1e-051.6e-051e-05

0.0005580.0003390.0038070.0023190.0020550.0005970.000541

0.000127

0.0003540.000414

0.0002370.000277

2e-059e-050.001980.0023197e-061.7e-05

0.0001290.0001239.6e-050.00014

0.0002150.000252

0.0003030.0001260.0002060.0004420.000324

0.0001670.000196

0.0002120.000248

0.0001930.000226

0.0002430.000284

0.0001062.7e-050.000116

0.0004290.0002890.0002490.0020160.0003790.0004210.000123

2.8e-051.2e-052.7e-05

5e-06

8e-064e-061e-05

2.2e-05

5e-065e-063e-06

6e-06

2e-05

0.0003880.0002890.0002490.0020160.0003050.0003810.000123

0.0606240.0851790.047950.0632060.0333330.0582280.04555

0.0355680.0716520.0304260.0392140.0224730.0347180.035184

0.0355680.0716520.0301630.0392140.0221650.0347180.035184

0.0002630.000308

3.6e-05

3.6e-05

0.0250560.0135270.0175240.0239920.0108240.023510.010366

1e-05

0.0001540.000181

0.0002213.4e-050.0001811.4e-050.000204

0.0005997.3e-050.000230.0001330.000515

0.000108

0.0005690.000360.0004065.6e-050.0005626.2e-05

1.9e-05

8e-06

0.0020140.0005230.0006160.0003780.002039

4.8e-05

0.0003269e-050.0003132.7e-050.0003240.000488

0.0001530.00018

0.0008990.001053

4.5e-05

0.0004340.000508

0.0003093.9e-050.0002575e-060.000294

4.2e-05

5e-05

0.0017510.0003210.0004910.0004880.0017690.000418

0.00013

9e-06

0.000103

4.3e-05

0.0013450.0002080.000308

1.4e-05

6.5e-05

3.7e-053.1e-050.0033580.0039314.5e-054.2e-05

3.9e-05

5.2e-05

0.000126

0.0006550.0001790.00020.0001310.0006250.000201

0.000128

0.0007730.0015360.0004010.0084680.0001120.0007110.002687

7e-06

3.9e-05

0.0001890.0001561.1e-050.0001779.2e-05

9e-05

0.0009360.0001460.0002317.7e-050.0008390.000176

0.0003463.8e-050.0002883.2e-050.000328

8e-06

4.4e-05

0.000127

2e-05

1.4e-05

3.7e-05

0.014150.0096290.006990.0115930.0044220.0142840.005675

5e-05

0.0009680.001134

0.0001270.0002517e-060.0001290.000517

0.0004426.9e-050.0003687.1e-050.000421

3.3e-05

0.000108

0.00011

0.0001490.0001222e-050.000137

0.0001181.3e-050.000115e-05

0.0001610.0006620.0002310.0021170.0002060.0001460.000451

0.0001590.0006620.0002310.0021170.0001570.0001450.000451

0.0001590.0006620.0002310.0021170.0001320.0001450.000451

2.6e-05

1.1e-053.6e-051.2e-05

1.6e-05

0.0001480.0006620.0002310.0021173e-050.0001330.000451

2.4e-05

2.5e-05

2.5e-05

2e-064.9e-051e-06

1.8e-05

1.8e-05

2e-063.1e-051e-06

2e-068e-061e-06

2.3e-05

0.000101

0.000101

0.000101

0.000101

0.000153

0.000153

7.3e-05

7.3e-05

8e-05

8e-05

0.0252390.0109880.011680.0092740.0219540.0250180.004107

0.000131

0.000131

0.000131

5.2e-050.0001314.3e-05

5.2e-050.0001314.3e-05

5.2e-056.8e-054.3e-05

6.3e-05

0.0248990.0109880.0115390.0092740.0211620.0247290.004107

0.0001510.0002220.000124

8.7e-050.0001196.3e-05

6.4e-050.0001036.1e-05

0.0001570.0001240.0001340.000146

0.0001570.0001240.0001340.000146

0.000103

0.000103

0.0244730.0109880.0113110.0092740.0205020.0243670.004087

0.0022050.002581

0.0002750.0001490.0001060.000244

0.00020.0001718.5e-05

7.8e-05

0.0239980.0109880.0087860.0092740.0176520.0241230.004087

0.0001180.0001040.0001259.2e-052e-05

0.0001180.0001040.0001259.2e-052e-05

7.6e-05

7.6e-05

0.0001410.000165

0.0001410.000165

0.0001410.000165

0.0002880.0003650.000246

5.7e-054.7e-054.2e-05

5.7e-054.7e-054.2e-05

0.0001185.5e-059.2e-05

0.0001185.5e-059.2e-05

5.5e-05

5.5e-05

3e-050.0001313.3e-05

1e-053.1e-059e-06

1.4e-054.8e-051.4e-05

6e-065.2e-051e-05

8.3e-057.7e-057.9e-05

8.3e-057.7e-057.9e-05

0.0009765.7e-050.0020530.0032720.0008081.7e-05

2.1e-050.0001561.4e-05

2.1e-050.0001561.4e-05

4.8e-05

2.1e-055.1e-051.4e-05

5.7e-05

0.000101

0.000101

0.000101

2.6e-059.2e-052.3e-05

2.6e-059.2e-052.3e-05

2.6e-059.2e-052.3e-05

9.2e-050.0001260.0004868.1e-05

0.000109

0.000109

0.0001260.000147

0.0001260.000147

2.6e-056.1e-051.6e-05

2.6e-056.1e-051.6e-05

6.6e-050.0001696.5e-05

2.2e-055.7e-052.2e-05

2.5e-056.1e-052.4e-05

1.9e-055.1e-051.9e-05

0.0007045.7e-050.001820.0021860.0005881.7e-05

6.7e-051.3e-051e-06

6.7e-051.3e-051e-06

3.7e-05

3.7e-05

0.000104

0.000104

2.5e-058e-062.2e-05

2.5e-058e-062.2e-05

0.0003595.7e-050.0016440.0018660.0003311.7e-05

0.0001510.000177

0.0003595.7e-050.0002590.0002450.0003311.4e-05

0.000160.000187

0.0001360.000159

0.0009380.001098

3e-06

0.0002530.0001760.0001580.000234

0.0002530.0001760.0001580.000234

0.0001330.0001070.0002510.000102

0.0001330.0001070.0002510.000102

5e-052e-064.4e-05

0.000123

8.3e-050.0001070.0001255.8e-05

1e-06

0.0268890.0297740.0203040.1292340.0132790.0286350.037384

0.0003134e-050.0003330.0002450.0002880.000151

6.1e-05

6.1e-05

5.4e-05

5.4e-05

0.0003134e-050.0003330.000130.0002880.000151

4.2e-051.8e-054.4e-05

1.8e-054e-050.0001293.8e-051.7e-050.000151

1e-05

0.0002530.0002044.1e-050.000227

2.3e-05

0.001990.0001330.0018360.0016520.0017060.001103

0.0009167.4e-050.0009880.0009240.0007746.7e-05

0.0001083.4e-054.6e-059e-051.6e-05

2.9e-052.7e-051.5e-05

8.5e-05

0.0003210.000376

0.000120.000141

3.1e-05

0.0003940.000323e-050.000356

3.7e-05

0.0003854e-050.0002270.0001820.0003132e-05

0.0010415.9e-050.0008480.0006740.0009040.001036

0.0004470.0002940.000260.0003797e-05

0.0002110.000247

0.000142.8e-050.0001132e-060.000125

6.3e-052.2e-057.1e-055e-06

2.5e-053.1e-051.5e-051.8e-056e-06

0.0003660.000230.0001280.0003110.000955

2.1e-053.6e-051.6e-05

2.1e-053.6e-051.6e-05

1.2e-051.8e-051.2e-05

1.2e-051.8e-051.2e-05

0.0023360.001380.0017390.0023190.0009510.0022320.000871

0.0001660.000194

0.0001660.000194

0.0013830.0001460.0007370.0002810.0012694.3e-05

0.0003080.0002584.5e-050.000297

2.9e-05

3e-06

3e-06

2.7e-05

3e-06

0.00013

7.5e-053.7e-051e-057.1e-058e-06

0.0003310.0002722.3e-050.000306

1.3e-05

03e-06

0.0006690.0001090.0002072.7e-050.000595

0.0008240.0012090.0008360.0023190.0004540.0008350.000785

2e-06

1e-061.7e-0501e-06

7.4e-05

4.2e-05

0.0008190.0011920.0008360.0023195.8e-050.000830.000709

8.9e-05

5.8e-05

6.3e-05

6.6e-05

4e-062e-064e-067.6e-05

0

0.0001292.5e-052.2e-050.0001284.3e-05

9e-061.8e-051.4e-054.3e-05

0.000122.5e-054e-060.000114

0.0005410.0001780.0005280.000393

0.0002410.0001787.9e-050.000183

0.0002410.0001787.9e-050.000183

0.0001130.0002427.9e-05

4.5e-05

5e-05

5.6e-054.8e-054.8e-05

6.6e-05

5.7e-053.3e-053.1e-05

9.3e-050.0001037.1e-05

3.9e-052.8e-052.8e-05

3.4e-05

5.4e-054.1e-054.3e-05

3.7e-052e-062.3e-05

3.7e-052e-062.3e-05

5.9e-05

5.9e-05

5.7e-054.3e-053.7e-05

5.7e-054.3e-053.7e-05

0.0013440.0002570.0009840.0005340.0012310.000552

0.000710.0002570.0005320.0001960.0006030.000552

0.0002880.000228.9e-050.0002334.6e-05

5.3e-05

0.0004220.0002570.0003125.4e-050.000370.000506

0.0002390.0001760.0001460.000181

9e-05

0.0002390.0001763.6e-050.000181

2e-05

0.0003950.0002760.0001920.000447

2.3e-056e-062.5e-05

2.8e-057e-062.5e-05

0.0001940.0001630.0001430.000278

1.5e-05

0.000150.0001132.1e-050.000119

0.0203650.0279640.015080.1269150.0090710.0227850.034707

0.0002010.000235

0.0002010.000235

3.8e-05

3.8e-05

0.0001150.000135

0.0001150.000135

0.0006010.000704

0.0006010.000704

0.0038710.0188560.003080.1068550.0005050.0032420.023895

0.0009330.0156280.0004610.0319560.0001420.000956

0.0009670.0024110.0013820.0257064.5e-050.001016

0.0014730.0004310.0006260.0247980.0002650.0007540.021497

0.0004980.0003860.0006110.0243955.3e-050.0005160.002398

2.5e-051.2e-052.7e-05

2.5e-051.2e-052.7e-05

3.3e-05

3.3e-05

0.0009530.0004780.0006890.0055440.0002170.0009260.000419

0.0003820.0001050.0001814.3e-050.0004010.000237

6.3e-058e-066.4e-052e-06

7e-06

4e-06

0.0001160.000136

0.0004280.0001440.0002133.1e-050.0003593.2e-05

0.000117

8e-050.0002290.0001790.0055447e-060.0001021.2e-05

0.0018970.0001680.0006440.0007910.0018480.000165

4.1e-055.8e-053.3e-05

0.0011820.0001680.0003670.0006040.001175

0.0006450.0002778.4e-050.0006140.000165

2.9e-054.5e-052.6e-05

0.0003860.0003310.0007350.002520.0003050.0032890.001068

0.0003065.1e-050.0002684.9e-050.000323

8e-050.000280.0004670.002520.0002560.0029660.001068

0.0002370.000278

0.0002370.000278

1.6e-051.8e-05

1.6e-051.8e-05

4e-05

4e-05

0.0052270.0012990.0021490.0020160.0009170.0053290.000847

0.0001486e-060.000174

1.1e-05

0.0007660.0001020.0002084.7e-050.000825.4e-05

1.3e-05

0.0013580.0003270.0002642.1e-050.0014220.000119

0.0007849.7e-050.0004070.0002620.000831.6e-05

0.0002050.00024

0.0016140.000490.0005380.0020160.0002410.001571

1.5e-05

0.0007050.0002830.0003792.4e-050.0006860.000384

0.0001

1.2e-05

9e-06

5e-06

8e-06

3e-06

0.0002730.00032

0.0002730.00032

0.0007040.0001020.0001130.000731

0.0007040.0001020.0001130.000731

0.0015060.0051760.0014790.009980.0002780.0015370.005719

0.0001820.000213

0.0001077.8e-050.000125

0.0005280.0050720.0009970.009983.6e-050.0005240.005381

0.0009780.0001040.0001930.0001640.001013

0.0028170.0006040.0032310.0014110.0028490.002119

0.001120.0001850.0003160.0002720.0011495.2e-05

0.0002860.000335

0.0001430.000167

0.0003290.000385

0.001180.001382

0.0005230.000612

0.0016970.0004190.0004540.0002520.00177.3e-05

6e-05

6e-05

4.4e-05

4.4e-05

0.0013970.0007520.0009330.0006230.0014470.000475

0.00011

4.5e-05

0.0005590.0006020.0004321.8e-050.0005910.000361

0

0.0001770.000207

0.0001320.000154

0.0008380.000150.0001928.9e-050.0008560.000114

4e-050.0001214.7e-05

1e-061e-061e-06

2.9e-051.1e-053.3e-05

8e-061.5e-051e-05

2e-061e-063e-06

6.3e-05

3e-05

0.0015010.0001980.0004810.0014230.00147

0.0015010.0001980.0004810.0014230.00147

0.000131

0.000131

2.5e-051.7e-052.5e-05

2.5e-051.7e-052.5e-05

0.0002320.00032

4.8e-05

0.0002320.000272

0.0001540.00018

0.0001540.00018

0.0001540.00018

0.000118

0.000118

0.000118

0.1024010.1621780.0481470.104940.0177050.1009280.139826

1.4e-054.1e-056e-06

1.4e-054.1e-056e-06

1e-051.6e-053e-06

1.2e-05

1e-054e-063e-06

4e-062.5e-053e-06

4e-062.5e-053e-06

5e-065e-055e-06

5e-065e-055e-06

5e-065e-055e-06

5e-065e-055e-06

0.1023820.1621780.0481470.104940.0176140.1009170.139826

7.3e-050.0002784.1e-05

2.9e-050.0001691.2e-05

1e-061e-061e-06

7e-06

3.9e-05

1e-061e-061e-06

8e-061.2e-054e-06

5e-062e-06

01.4e-051e-06

2e-061.2e-050

9e-067e-062e-06

3.2e-05

3e-064.4e-051e-06

1e-053.8e-051.1e-05

1e-053.8e-051.1e-05

7e-063e-054e-06

7e-063e-054e-06

2.7e-054.1e-051.4e-05

1.1e-051.9e-059e-06

1.6e-052.2e-055e-06

0.1022570.1621780.0481470.104940.0173070.100830.139826

1.7e-055.4e-051.1e-05

4e-06

8e-06

7e-06

1.1e-051.7e-051e-05

1e-05

6e-068e-061e-06

3.6e-055.6e-053.2e-05

1.5e-051e-051.5e-05

8e-061.5e-056e-06

1.3e-053.1e-051.1e-05

0.1022040.1621780.0481470.104940.0171970.1007870.139826

2e-052e-062e-05

0.0001250.0001092.8e-050.000129

3.9e-052e-05

7e-06

1.3e-05

4.1e-052.7e-05

0.1010270.1589680.0463630.0590730.0166190.0990410.139772

0.0009320.003210.0016750.0458677.4e-050.0015785.4e-05

1e-05

0.000131

2e-053.9e-051.9e-05

3.4e-05

8e-05

3.7e-05

4.4e-05

3.2e-05

5.2e-052.9e-054.6e-05

5.2e-052.9e-054.6e-05

5.2e-052.9e-054.6e-05

0.0684710.054960.05090.0537290.0522870.0671370.045421

0.0272260.016290.0271960.0219760.0219110.0269640.020047

0.0039780.004657

0.0039780.004657

0.0039780.004657

0.0067540.002390.0041770.0054440.0047120.0068120.000423

0.0002180.0001747.2e-050.0001921.5e-05

2.2e-05

0.0002180.0001743e-050.0001921.5e-05

2e-05

0.000128

7.2e-05

5.6e-05

0.0064170.002390.0040030.0054440.0043230.0065070.000408

0.0026310.00308

0.0064170.002390.0011790.0054440.0010160.0065070.000408

0.0001930.000227

6.5e-053.8e-056.7e-05

6.5e-053.8e-056.7e-05

5.4e-050.0001514.6e-05

5.4e-054.2e-054.6e-05

5.2e-05

5.7e-05

0.000540.0063270.0034580.0005230.004089

6.3e-057.8e-057e-05

3.1e-053.3e-05

3.2e-057.8e-053.7e-05

4.8e-05

4.8e-05

0.0001570.0001236.9e-050.000132

0.0001570.0001236.9e-050.000132

0.000170.0001476.6e-050.000174

0.000170.0001476.6e-050.000174

4e-05

4e-05

6.5e-05

6.5e-05

0.005930.0030020.004075

5.8e-05

7.7e-05

0.0034810.004075

0.0024490.002867

0.000150.0001279e-050.0001471.4e-05

0.000150.0001279e-050.000147

1.4e-05

0.0003449e-060.0001060.0007480.000286

2.3e-055.4e-052.2e-05

2.3e-055.4e-052.2e-05

0.0001249e-060.0001065.5e-058e-05

2.1e-05

0.0001249e-060.0001063.4e-058e-05

0.000101

0.000101

0.0001610.0002960.000151

5e-06

3.9e-05

2.4e-05

0.0001166.3e-050.000112

3.6e-05

3e-052.7e-052.5e-05

4.6e-05

3e-05

1.9e-05

3e-061e-063e-06

9e-066e-068e-06

3e-0603e-06

9e-064.6e-057e-06

8e-062.7e-056e-06

1e-061.9e-051e-06

2.7e-057.7e-052.6e-05

2.7e-052.4e-052.6e-05

5.3e-05

7.4e-05

7.4e-05

4.5e-05

4.5e-05

0.0103270.0055440.0056820.0056450.0058570.0103270.003288

0.0001814.4e-050.0001080.0001793.5e-05

2.5e-05

7.2e-052.6e-052e-056.9e-053.5e-05

0.0001091.8e-054.1e-050.00011

2.2e-05

0.0006429.2e-050.0004320.0004360.0006088.8e-05

0.0001320.0001065.3e-050.000118

0.00023.2e-050.0001668.7e-050.0001886.5e-05

0.000105

3.5e-05

4.1e-055e-054.6e-05

7.5e-054.8e-057.4e-05

0.0001946e-050.000165.8e-050.0001822.3e-05

1.6e-055.8e-051.6e-05

1.1e-05

5e-062.8e-055e-06

1.1e-051.9e-051.1e-05

5.6e-050.0002244.3e-05

1e-05

3.1e-05

5.6e-051.1e-054.3e-05

1.4e-05

2.1e-05

2.2e-05

1.4e-05

3.4e-05

6.7e-05

0.0086950.0049920.0039550.0056450.0039220.0087180.003025

9e-06

1.3e-054e-062.3e-05

1e-06

4.9e-057.1e-051.5e-055.2e-05

2.1e-05

4.6e-051e-054.6e-051.5e-05

9.9e-051.3e-050.000108

6.1e-054e-066.6e-052.4e-05

2e-064e-062e-066e-06

4.9e-051e-055.7e-05

2.8e-05

1.9e-05

8e-06

0.0001390.0001184.2e-050.000138

1.6e-05

1.8e-05

5.5e-051.7e-055.9e-055e-05

5e-061.3e-052e-065e-068e-06

0

8.2e-055.1e-058.8e-05

0.0001250.0001061.8e-050.000123

1.2e-05

2.9e-052e-051e-053.3e-05

1.1e-05

3e-06

3e-05

1.6e-058e-061.5e-05

1.5e-05

1.3e-05

1.6e-05

9.7e-05

9e-06

0.000130.0001137e-050.0001361.8e-05

0.0027040.0022620.00130.0027220.0005830.0026690.001485

4.7e-05

0.0050910.0026260.0023180.0029230.002560.0050980.001419

2.4e-05

8.8e-05

1.6e-05

1.4e-05

1.4e-05

0.0007370.0004160.0012950.0011090.0007630.000126

0.0001370.0001258.7e-050.000156

0.0002310.0002140.000198.2e-050.000224

8.9e-05

0.0006690.000783

0.0001680.0001410.0001356.8e-050.000177.3e-05

0.0002016.1e-050.0001760.0002135.3e-05

0.0092610.0083470.0069260.0108870.0071360.0090160.00759

0.0073810.0076770.0061240.008770.0054410.0072180.007529

5.8e-05

0.0073810.0076770.0061240.008770.0053830.0072180.007529

0.0001

5.2e-05

4.8e-05

0.0012780.000670.0008020.0021170.0008510.0012464.7e-05

2e-059e-062.4e-054.7e-05

5.4e-05

0.0010940.000670.0008020.0021170.0006650.001063

7.2e-054.9e-057.6e-05

2.7e-052.2e-052.6e-05

6.5e-055.2e-055.7e-05

6.6e-053e-056.1e-051.4e-05

6.6e-053e-056.1e-051.4e-05

7.8e-05

3.2e-05

4.6e-05

0.0001260.0001220.000116

5.2e-055.4e-054.9e-05

7.4e-056.8e-056.7e-05

0.00017.9e-058.3e-05

0.00017.9e-058.3e-05

6.9e-053.3e-057.4e-05

6.9e-053.3e-057.4e-05

5.7e-056.5e-055.7e-05

5.7e-056.5e-055.7e-05

0.000122

5.8e-05

6.4e-05

4.4e-052.9e-054.7e-05

2.7e-056e-063e-05

1.6e-05

1.7e-057e-061.7e-05

6.9e-05

6.9e-05

9e-065.4e-058e-06

9e-065.4e-058e-06

0.0001316.3e-050.000106

0.0001316.3e-050.000106

0.0004780.0008830.000412.3e-05

0.00014

7.4e-05

7.4e-05

6.6e-05

6.6e-05

0.000160.0001570.000125

7e-054.7e-055.7e-05

7e-054.7e-055.7e-05

5.7e-056.7e-054.1e-05

5.7e-056.7e-054.1e-05

3.3e-054.3e-052.7e-05

3.3e-054.3e-052.7e-05

0.0001280.0002750.000123

5.9e-051e-054.6e-05

4e-054e-063e-05

1.9e-056e-061.6e-05

3.7e-05

3.7e-05

0.000101

4.3e-05

5.8e-05

3.1e-058.1e-053.5e-05

1.4e-054e-051.3e-05

1.7e-054.1e-052.2e-05

3.8e-054.6e-054.2e-05

3.8e-054.6e-054.2e-05

0.0001217.1e-059.3e-052.3e-05

0.0001217.1e-059.3e-052.3e-05

0.0001217.1e-059.3e-052.3e-05

6.9e-050.000246.9e-05

6e-050.0001836e-05

3.4e-05

2e-05

1.9e-051e-051.9e-05

1.7e-055.4e-051.8e-05

1.4e-053.9e-051.3e-05

1e-052.6e-051e-05

9e-065.7e-059e-06

9e-063.6e-059e-06

2.1e-05

0.0037470.0110970.0048940.0167330.0022310.0036470.013118

0.0032280.0110430.0045670.0148180.0019730.0032340.013075

0.0032280.0110430.0043330.0148180.0017350.0032340.01286

0.0019650.0104650.0006590.0070560.0001550.0020520.010235

2.2e-05

0.0003330.00039

0.0006490.000764e-05

0.0003370.0001950.0002260.003734.3e-050.000230.000161

0.0008420.000985

0.000104

0.0001720.000201

0.0002610.000305

0.0002850.000334

1.9e-05

0.0009260.0003830.0005510.0040320.0003610.0009524.7e-05

0.0003550.000416

1.7e-05

0.0002340.0001370.000137

0.0001170.000137

0.0001170.000137

0.000101

0.000101

7.8e-05

7.8e-05

0.0005195.4e-050.0003270.0019150.0002580.0004134.3e-05

0.0001065.5e-057.2e-05

0.0001065.5e-057.2e-05

0.0001490.0001176.6e-050.000127

0.0001490.0001176.6e-050.000127

0.0002645.4e-050.000210.0019150.0001370.0002144.3e-05

0.0002645.4e-050.000210.0019150.0001370.0002144.3e-05

0.0363310.0275730.0184450.015020.0268340.0355160.012233

0.0363310.0275730.0184450.015020.0268340.0355160.012233

0.0362110.0275730.0184450.015020.0267290.0354190.012233

0.0362110.0275730.0184450.015020.0267290.0354190.012233

4.2e-058e-063.3e-05

4.2e-058e-063.3e-05

6.1e-057.4e-055.3e-05

2e-067e-062e-06

1e-061.2e-054e-06

2.2e-052.3e-051e-05

8e-061.8e-058e-06

2.8e-051.4e-052.9e-05

1.7e-052.3e-051.1e-05

1.7e-052.3e-051.1e-05

0.0006890.0003650.0004280.0006

0.0002150.0001090.0001130.000176

8.7e-054.6e-058e-05

8.7e-054.6e-058e-05

0.0001280.0001096.7e-059.6e-05

0.0001280.0001096.7e-059.6e-05

0.0004080.0002560.000260.000377

0.0002550.0001290.0001320.000233

0.0001560.0001297e-060.000145

5e-05

1e-05

9.9e-056.5e-058.8e-05

0.0001530.0001270.0001280.000144

5.6e-05

0.0001530.0001277.2e-050.000144

6.6e-055.5e-054.7e-05

6.6e-055.5e-054.7e-05

6.6e-055.5e-054.7e-05

0.0017870.0006270.0082020.0016361.8e-05

0.0002830.0004920.000241.4e-05

0.0002230.0002920.0001881.4e-05

5.1e-05

5.1e-05

0.0002230.0002410.0001881.4e-05

3.2e-056e-052.6e-05

1.7e-051.1e-051.3e-05

1.1e-05

1.6e-051.8e-059e-06

4.8e-052.4e-053.9e-05

2.9e-052e-051.7e-051.4e-05

3.1e-05

3.6e-052.2e-053.9e-05

3.3e-053.3e-053e-05

1.2e-051.1e-051.5e-05

6e-050.00025.2e-05

0.000115

5.3e-05

6.2e-05

6e-058.5e-055.2e-05

2.3e-054.8e-052e-05

3.7e-053.7e-053.2e-05

7.1e-057.4e-056e-05

7.1e-057.4e-056e-05

7.1e-057.4e-056e-05

7.1e-057.4e-056e-05

3.9e-050.0001742.7e-05

1.4e-054.2e-057e-06

1.4e-054.2e-057e-06

1.4e-054.2e-057e-06

2.5e-050.0001322e-05

7e-063.2e-055e-06

7e-063.2e-055e-06

8e-064.4e-056e-06

8e-064.4e-056e-06

1e-055.6e-059e-06

1e-055.6e-059e-06

0.0004560.0005220.0055190.000454

4.1e-050.0001142.2e-05

4.1e-050.0001142.2e-05

9e-063.5e-058e-06

3.2e-057.9e-051.4e-05

0.0004150.0005220.0054050.000432

0.0004150.0005220.0054050.000432

0.0004150.0005220.0054050.000432

0.0001870.0003680.000153

0.0001340.0002090.00011

5.7e-056.1e-055e-05

5.7e-056.1e-055e-05

1.2e-055.9e-056e-06

1.2e-055.9e-056e-06

5.6e-054.7e-054.6e-05

5.6e-054.7e-054.6e-05

9e-064.2e-058e-06

9e-064.2e-058e-06

5.3e-050.0001594.3e-05

2.6e-054.6e-051.4e-05

2.6e-054.6e-051.4e-05

7e-063.4e-051e-05

7e-063.4e-051e-05

7e-063.3e-057e-06

7e-063.3e-057e-06

1.3e-054.6e-051.2e-05

1.3e-054.6e-051.2e-05

0.0003860.0001050.0009520.00037

5.2e-059.3e-054.3e-05

5.2e-059.3e-054.3e-05

5.2e-059.3e-054.3e-05

3.7e-059.3e-053.3e-05

3.7e-055.2e-053.3e-05

3.7e-055.2e-053.3e-05

4.1e-05

4.1e-05

3.8e-050.0001050.0001853.9e-05

6.2e-05

6.2e-05

3.8e-050.0001050.0001233.9e-05

3.8e-050.0001050.0001233.9e-05

8.3e-05

8.3e-05

8.3e-05

9.2e-05

9.2e-05

9.2e-05

0.0001130.0001840.00011

2.8e-054.9e-053.4e-05

2.8e-054.9e-053.4e-05

8.5e-050.0001357.6e-05

3.4e-05

2.9e-053.3e-052.4e-05

2.7e-053.6e-052.3e-05

2.9e-053.2e-052.9e-05

0.0001460.0002220.000145

0.0001460.0002220.000145

3.2e-054.2e-053e-05

4.7e-056.6e-054.4e-05

6.7e-050.0001147.1e-05

3e-052.2e-051.6e-05

3e-052.2e-051.6e-05

3e-052.2e-051.6e-05

3e-052.2e-051.6e-05

0.0003310.00050.0003154e-06

3.7e-057.5e-053.1e-05

3.7e-057.5e-053.1e-05

3.7e-053e-053.1e-05

4.5e-05

3.1e-053.8e-053.7e-05

3.1e-053.8e-053.7e-05

3.1e-053.8e-053.7e-05

0.0002630.0003870.0002474e-06

0.0002560.0003640.0002414e-06

3.1e-05

3.8e-055.7e-05

3.4e-054.3e-052.8e-05

1.4e-053.8e-051.1e-05

1.8e-052.9e-051.8e-05

2.7e-055.1e-052.5e-05

3e-061e-06

4.2e-052.8e-054e-05

5.3e-054.2e-053.7e-054e-06

5.4e-05

1.1e-051.1e-05

1.6e-054.8e-051.3e-05

7e-062.3e-056e-06

7e-062.3e-056e-06

4e-060.0001011e-06

4e-060.0001011e-06

4.6e-05

4.6e-05

4e-065.5e-051e-06

4e-065.5e-051e-06

0.0477480.0828320.1153940.0345770.0512690.0479430.115573

0.0178030.0066120.0110680.0070570.0129050.0181410.003348

0.0002470.0002360.0003710.000242

0.0002470.0002360.0003710.000242

0.0002470.0002360.0003710.000242

0.000110.0001090.0001071.4e-05

1.4e-05

1.4e-05

0.000110.0001090.000107

0.000110.0001090.000107

0.0082810.0035120.0048760.0053070.0086460.00132

0.0003660.000428

0.0003660.000428

0.000170.0001994e-06

4e-06

0.000170.000199

0.0079750.0034760.0040150.0037720.0083610.001229

0.0013970.001636

0.0017280.000390.0006410.0006090.001814

0.0019980.001370.000780.0004940.0020970.000573

0.0019750.0008280.0006590.0006040.0020830.00038

0.0022740.0008880.0005380.0004290.0023670.000276

0.0003063.6e-050.0003250.0009080.0002858.7e-05

0.0003063.6e-050.0003250.0009080.0002858.7e-05

0.0005251.6e-050.0002370.0004090.0005282.8e-05

0.0005251.6e-050.0002370.0004090.0005282.8e-05

0.0001096.6e-050.000111

8.4e-05

5.1e-051.6e-051.6e-054.8e-052.8e-05

4.4e-05

9e-055.8e-058.6e-05

2.1e-05

0.0001360.0001244.4e-050.000156

0.0001390.0001137.6e-050.000127

0.000110.0001320.0001141.8e-05

1e-061.2e-050

9e-06

0

1e-060

000

000

01e-060

01e-060

000

1e-0600

0.0001090.000120.0001141.8e-05

8.2e-05

1.4e-05

0.0001093.8e-050.0001144e-06

0.0001440.000169

0.0001440.000169

0.0001440.000169

0.0008020.000510.0003880.000843e-05

0.0002240.0001430.0001070.000242

0.0002240.0001430.0001070.000242

0.0002330.0001560.0001240.0002461.4e-05

0.0002330.0001560.0001240.0002461.4e-05

0.0003450.0002110.0001570.0003521.6e-05

0.0003450.0002110.0001570.0003521.6e-05

0.0007646.9e-050.0008090.0014780.000737

0.0003536.9e-050.0003460.0005530.000363

0.0003536.9e-050.0003460.0005530.000363

0.0001360.0001170.0001870.000109

0.0001360.0001170.0001870.000109

0.0002330.000273

0.0001190.00014

0.0001140.000133

6.1e-05

6.1e-05

8.6e-05

8.6e-05

4.5e-05

4.5e-05

0.0002750.0001130.0002730.000265

5.3e-056.9e-054.7e-05

8.8e-057.2e-058.5e-05

0.0001340.0001130.0001320.000133

0.0001620.000120.0003080.000137

4.5e-057.8e-053.7e-05

4.5e-057.8e-053.7e-05

0.000120.000141

0.000120.000141

0.0001178.9e-050.0001

0.0001178.9e-050.0001

0.0035460.0016480.0029730.0047380.0023350.0036060.001534

0.000186

6.5e-05

5.1e-05

7e-05

0.0007360.0005740.0006470.0026210.0004540.000710.001213

0.0007360.0005740.0006470.0026210.0004540.000710.001213

0.0002690.000110.0001830.000274

0.0001290.000117.9e-050.000128

0.000140.0001040.000146

0.0024640.0010740.0022160.0021170.0014670.0025510.000321

0.0002799.7e-050.0001973.8e-050.0003050.000163

0.0002144.9e-050.0001896.3e-050.000228

0.0002395.3e-050.0002136.1e-050.0002625.9e-05

0.0012570.0007390.0008320.0021170.0005810.001263

0.0002094.8e-050.0001799.7e-050.0002115.2e-05

8.9e-05

6.4e-05

0.0002668.8e-050.0002343.8e-050.0002824.7e-05

0.0003720.000436

7.7e-054.5e-057.1e-05

7.7e-054.5e-057.1e-05

0.0001250.0003616.1e-05

0.000215

8.6e-05

0.000129

0.0001250.000146

0.0001250.000146

6.1e-05

6.1e-05

0.0031930.0013670.0010380.0023190.0013260.0031370.000343

0.0002824.8e-050.0001190.00020.0002532.9e-05

0.0001134.2e-059.8e-05

2.3e-052.5e-051.6e-052.3e-05

6.7e-05

0.0001462.3e-050.0001197.5e-050.0001322.9e-05

6.1e-05

6.1e-05

5.3e-050.0001165.5e-05

4e-063e-062e-06

1.7e-053.3e-052.7e-05

1.2e-053.6e-051.2e-05

1.8e-05

2e-052.6e-051.4e-05

0.00040.0002210.0003970.000287

0.0001132.1e-050.000104

9.3e-056e-060.0001053.8e-05

0.000103

0.000111

0.0001172.6e-050.0001180.000124

7.7e-056.5e-057e-051.4e-05

0.0024580.0013190.0009190.0023190.0005930.0024322.7e-05

1.2e-05

6.3e-05

0

0.0001111.9e-053.8e-050.0001322.7e-05

0.000140.0001185.6e-050.000137

8e-052.1e-051.1e-057.9e-05

0

7e-06

0.0021270.0012790.0008010.0023190.0003160.002084

8.8e-05

2e-06

0

5e-05

5e-05

8.5e-05

8.5e-05

6.3e-050.0002124.7e-05

6.3e-050.0002124.7e-05

5e-065.6e-055e-06

6e-067e-061e-06

3.4e-05

1.2e-053e-051.2e-05

1.2e-052e-053e-06

3e-068e-064e-06

6e-061.7e-055e-06

1e-053.2e-059e-06

9e-068e-068e-06

2.5e-054.7e-052.5e-05

2.5e-054.7e-052.5e-05

2.5e-054.7e-052.5e-05

2.5e-054.7e-052.5e-05

0.0037470.0005370.0666020.0109640.0037860.073596

0.0016290.0002490.0626710.0073140.0016480.071729

0.0002750.0002840.0005280.000285

0.0002750.0002840.0005280.000285

0.0013230.0002490.0006020.0061830.001336

0.0013230.0002490.0006020.0061830.001336

0.061270.071729

0.061270.071729

3.1e-050.0005150.0006032.7e-05

3.1e-050.0005150.0006032.7e-05

0.0001990.000234

0.0001990.000234

0.0001990.000234

0.0021180.0002880.0037320.003650.0021380.001633

0.0002620.0001770.0001530.000245

0.0002620.0001770.0001530.000245

0.0003564.6e-050.0005080.0001840.0003660.000291

0.0002370.000277

0.0001374.6e-050.0001185.5e-050.0001381.4e-05

0.0002190.0001530.0001290.000228

0.0001160.000136

0.0001160.000136

7.4e-05

7.4e-05

0.0001090.0005740.0001170.0001140.000672

2.9e-054.1e-053.2e-05

8e-050.0005747.6e-058.2e-050.000672

0.0004133.4e-050.0002850.0002440.0004264.6e-05

0.0001570.0001230.0001210.000159

0.0002563.4e-050.0001620.0001230.0002674.6e-05

7e-06

7e-06

0.000117

0.000117

3.1e-05

3.1e-05

0.0001290.00027

0.000119

0.0001290.000151

0.0002310.00027

0.0002310.00027

0.0002730.000319

0.0002730.000319

5.2e-052.7e-055e-05

5.2e-052.7e-055e-05

0.0001470.000172

0.0001470.000172

3.6e-054.2e-05

3.6e-054.2e-05

0.0001510.000177

0.0001510.000177

0.0001373e-059.4e-050.000117

7e-053e-058.1e-058.2e-05

6.7e-051.3e-053.5e-05

0.000450.0001270.0002650.0004130.0004521.4e-05

8.9e-05

0.0003527e-050.0002650.0002510.0003511.4e-05

9.8e-055.7e-057.3e-050.000101

0.0001665.1e-050.0001320.0001290.000175

0.0001665.1e-050.0001320.0001290.000175

0.0001340.000157

0.0001340.000157

7e-06

7e-06

1.4e-05

1.4e-05

0.0004710.000551

0.0004710.000551

3.2e-050.0001863.5e-05

2.1e-055.1e-052.1e-05

9.2e-05

1.1e-054.3e-051.4e-05

0.0001050.0001180.000116

0.0001050.0001180.000116

0.0001390.000163

0.0001390.000163

0.00013

0.00013

0.0004350.0003490.0004020.0004164.8e-05

0.0004350.0003490.0004020.0004164.8e-05

4.5e-050.0001230.0001442.9e-05

4.5e-050.0001230.0001442.9e-05

5.3e-057.4e-054.4e-05

5.3e-057.4e-054.4e-05

0.0003370.0002260.0001840.0003434.8e-05

0.0003370.0002260.0001840.0003434.8e-05

0.0015614.6e-050.0021180.0022780.0017260.000103

0.0015614.6e-050.0021180.0022780.0017260.000103

0.000360.000422

0.000360.000422

0.0006244.6e-050.0005180.0004860.0007954.7e-05

0.0002270.0002047.6e-050.0002524.7e-05

0.0001414.6e-050.0001490.0001520.000286

0.000129

0.0002560.0001650.0001290.000257

0.0001410.0001340.0002210.000134

0.0001410.0001340.0002210.000134

0.0003880.0007940.0008390.00045.6e-05

5.6e-05

0.0001190.000139

0.0002250.000264

0.0003880.000270.0002260.0004

0.000180.00021

0.0004080.0003120.000310.000397

0.0004080.0003120.000310.000397

0.0014531.8e-050.0010520.001630.0013996e-06

0.000330.000320.000287

6.6e-052.4e-054.5e-05

6.6e-052.4e-054.5e-05

2e-066.3e-052e-06

1e-05

4.9e-05

2e-064e-062e-06

5.6e-053.9e-054.4e-05

5.6e-053.9e-054.4e-05

5.7e-052.5e-055.2e-05

5.7e-052.5e-055.2e-05

6.5e-05

6.5e-05

6.5e-053.8e-056.1e-05

3.6e-052.4e-053.2e-05

2.9e-051.4e-052.9e-05

8.4e-056.6e-058.3e-05

8.4e-053.3e-058.3e-05

3.3e-05

0.0011231.8e-050.0010520.001310.0011126e-06

0.000120.00014

0.000120.00014

0.0002390.0001530.0002590.000236e-06

0.0001840.0001530.000190.0001676e-06

5.5e-056.9e-056.3e-05

0.0002751.8e-050.0002410.0001640.000289

4.9e-05

0.0001460.0001285.7e-050.000154

1.6e-05

0.0001291.8e-050.0001134.2e-050.000135

5e-064.7e-052e-06

5e-064.7e-052e-06

9e-050.0001120.0001317.8e-05

9e-050.0001120.0001317.8e-05

0.0003080.0002530.0001750.000286

0.0001480.000134.7e-050.000156

4.7e-05

0.000160.0001238.1e-050.00013

0.0002060.0001730.0001790.000227

0.0002060.0001730.0001790.000227

0.0001

0.0001

0.000115

0.000115

0.0001

3.6e-05

3.6e-05

3.6e-05

6.4e-05

6.4e-05

6.4e-05

0.0016070.000130.0021770.0046370.0034910.0016365.6e-05

0.0014670.000130.0018420.0046370.0025040.0014865.6e-05

0.0001680.0001860.0001571.4e-05

5.6e-05

2.9e-05

7.8e-057.3e-058.1e-05

9e-052.8e-057.6e-051.4e-05

0.0004454.9e-050.0002760.0005050.0004381.4e-05

3.6e-05

6.1e-05

5.4e-05

0.0001214e-050.0001151.4e-05

0.0001664.9e-050.0001432.6e-050.000169

4.5e-05

4.5e-05

0.000108

0.0001580.0001339e-050.000154

0.000111

0.000111

8.5e-05

8.5e-05

0.0004340.0004140.0022180.0004930.0004392.8e-05

1.4e-05

0.0002260.0002710.0022188.9e-050.000218

1.4e-05

7e-05

5.7e-05

0.0002080.0001430.0001160.000221

9.4e-05

6.7e-05

1.8e-054.7e-054.5e-05

1.8e-054.7e-054.5e-05

0.0004028.1e-050.0010220.0024190.0009250.000407

0.0004028.1e-050.0004780.0024199.1e-050.000407

5.5e-05

7.1e-05

4.5e-05

0.0005440.000637

2.6e-05

0.000130.000152

0.000130.000152

0.000140.0003350.0009870.00015

0.000106

5.4e-05

5.2e-05

0.0002110.000247

0.0002110.000247

0.000140.0001240.0001530.00015

6.1e-05

0.000140.0001249.2e-050.00015

0.000481

9.6e-05

9.4e-05

8.7e-05

5.5e-05

8.9e-05

6e-05

0.0003469.9e-050.0003330.0005760.000324

0.0003469.9e-050.0003330.0005760.000324

0.0003469.9e-050.0003330.0005760.000324

0.0003469.9e-050.0003330.0005760.000324

8.9e-050.0002847.7e-05

8.9e-050.0002847.7e-05

4.4e-050.0001343.7e-05

3.6e-054.6e-053.3e-05

8e-068.8e-054e-06

4.5e-050.000154e-05

1.6e-055.7e-051.2e-05

2.9e-059.3e-052.8e-05

0.0206820.075390.0316950.0228830.0185920.0204130.038416

4e-05

4e-05

4e-05

1.5e-054.1e-059e-06

1.5e-054.1e-059e-06

1.5e-054.1e-059e-06

0.0154790.0373270.0166390.0228830.0163550.0151720.025003

0.0022160.0014910.0012610.0024190.0012110.0021640.000834

6e-068e-062e-06

3e-06

3.5e-05

0.0022070.0014910.0012610.0024190.0011570.0021590.000834

2e-062e-06

2e-06

0

1e-066e-061e-06

3.2e-051e-053.7e-05

2e-061e-062e-06

1e-06

1e-062e-061e-06

2.8e-052e-063e-05

1e-064e-064e-06

0.013230.0358360.0153780.0204640.0150940.0129710.024169

1.2e-050.0002410.0002831.2e-053.4e-05

0.0132170.0358360.0151370.0204640.0148060.0129590.024135

1e-065e-060

1e-064e-05

2e-05

1e-061.6e-05

04e-06

0.0051880.0380630.0150560.0021560.0052320.013413

3e-061.1e-052e-06

3e-061.1e-052e-06

0.0051850.0380630.0150560.0021450.005230.013413

0.0005720.0004940.0002910.000250.0005910.000191

0.0001330.0001167.3e-050.0001392e-06

0.0007970.0005390.0004340.0002810.0008285e-06

0.0001810.0001553.4e-056e-06

0.0002130.0001886.9e-050.000227

3.4e-053.5e-051.1e-05

0.000320.0002090.0001580.0003572.1e-05

0.0001120.0177470.0001560.0001185e-06

9.7e-05

0.0003260.0002190.0001730.000353

0.0002530.0002285.6e-050.000282

0.0001380.0001160.0001430.000503

0.000260.0002356.3e-050.000292

1.5e-055e-061e-06

3.3e-050.0011110.0009491.6e-053.4e-05

0.0010790.0181720.0007280.0003550.001122

0.0002060.0001816.4e-050.000218

5.1e-057e-063e-06

0.0107040.012531

4e-06

0.0001220.0001136.5e-050.0001270.000151

0.000340.0003067.2e-050.000378

4.7e-056.7e-053.5e-05

4.7e-056.7e-053.5e-05

4.7e-056.7e-053.5e-05

4.7e-056.7e-053.5e-05

4.7e-056.7e-053.5e-05

4.7e-056.7e-053.5e-05

0.0026090.0137690.0028220.0117940.0015860.0022680.014319

0.0026090.0137690.0028220.0117940.0015860.0022680.014319

1.9e-050.000121.6e-05

1e-062.5e-050

1e-062.5e-050

1e-062.5e-050

2e-061.8e-052e-06

2e-061.8e-052e-06

2e-061.8e-052e-06

1.6e-057.7e-051.4e-05

1e-065.3e-050

1e-065.3e-050

1.5e-052.4e-051.4e-05

2.4e-05

1.5e-051.4e-05

0.002590.0137690.0028220.0117940.0014660.0022520.014319

0.002590.0137690.0028220.0117940.0014660.0022520.014319

0.002590.0137690.0028220.0117940.0014660.0022520.014319

000

6e-063.5e-056e-06

0.0025470.0137690.0028220.0117940.0013420.0022170.01427

1e-051.9e-059e-064.9e-05

06e-061e-06

2e-05

1.8e-052e-061.1e-05

5e-062.8e-055e-06

4e-062e-063e-06

1.2e-05

9e-064.8e-058e-06

9e-064.8e-058e-06

9e-064.8e-058e-06

9e-064.8e-058e-06

9e-064.8e-058e-06

9e-064.8e-058e-06

9.1e-050.0005988.4e-05

2.5e-050.0002223.2e-05

6e-066.4e-051.8e-05

6e-066.4e-051.8e-05

6e-066.4e-051.8e-05

6e-066.4e-051.8e-05

1.9e-050.0001581.4e-05

1.9e-050.0001581.4e-05

4.2e-05

4.2e-05

6.8e-05

6.8e-05

1.9e-054.8e-051.4e-05

1.9e-054.8e-051.4e-05

4.7e-05

4.7e-05

4.7e-05

4.7e-05

4.7e-05

4.9e-050.0002054.6e-05

4.9e-050.0002054.6e-05

4.9e-050.0002054.6e-05

4.9e-050.0002054.6e-05

0.000107

4.9e-059.8e-054.6e-05

7.2e-05

7.2e-05

7.2e-05

7.2e-05

7.2e-05

1.7e-055.2e-056e-06

1.7e-055.2e-056e-06

1.7e-055.2e-056e-06

1.7e-055.2e-056e-06

1.7e-055.2e-056e-06

5.6e-050.0003094e-05

5.6e-050.0003094e-05

5.6e-050.0003094e-05

5.6e-050.0003094e-05

8.8e-05

8.8e-05

1.2e-056.3e-059e-06

1.2e-056.3e-059e-06

1e-065e-050

1e-065e-050

2.2e-056.7e-052.2e-05

2.2e-056.7e-052.2e-05

2.1e-054.1e-059e-06

2.1e-054.1e-059e-06

4.8e-050.0002013e-05

3.3e-055.8e-051.4e-05

3.3e-055.8e-051.4e-05

3.3e-055.8e-051.4e-05

3.3e-055.8e-051.4e-05

3.3e-055.8e-051.4e-05

1.5e-057.3e-051.6e-05

1.5e-057.3e-051.6e-05

1.5e-057.3e-051.6e-05

1.5e-057.3e-051.6e-05

1.5e-057.3e-051.6e-05

7e-05

7e-05

7e-05

7e-05

7e-05

4e-064.4e-052e-06

4e-064.4e-052e-06

4e-064.4e-052e-06

4e-064.4e-052e-06

4e-064.4e-052e-06

4e-064.4e-052e-06

0.0079270.0098560.0055290.0090730.0053710.0196890.004879

0.0079270.0098560.0055290.0090730.0053710.0196890.004879

0.0007790.0028460.0008360.004940.0021270.000730.001756

0.0003410.0008810.0002910.0021170.000180.0003240.000601

0.000320.0008810.0002910.0021170.0001480.0003110.000601

7e-069e-067e-06

0.0003040.0008810.0002910.0021170.000130.0002970.000601

01e-060

3e-064e-061e-06

2e-063e-062e-06

4e-061e-064e-06

2.1e-053.2e-051.3e-05

1.6e-055e-061e-05

1.5e-05

1e-061e-061e-06

1e-062e-06

3e-062e-06

06e-06

3e-06

0.0004380.0019650.0005450.0028230.0019470.0004060.001155

1e-057.3e-056e-06

1e-057.3e-056e-06

2e-050.000211.9e-05

3e-066.1e-053e-06

5e-066e-055e-06

1.2e-058.9e-051.1e-05

4.3e-058.8e-052.9e-05

3.4e-055.4e-052.2e-05

9e-063.4e-057e-06

1.9e-054.4e-051.3e-05

1.9e-054.4e-051.3e-05

0.0003460.0019650.0005450.0028230.0015320.0003390.001155

4e-064.3e-053e-06

7.3e-05

1.1e-056.7e-051e-05

5e-065.9e-055e-06

9e-066.4e-058e-06

0.0002160.0019650.0004210.0028230.0008490.0002150.001155

6e-065.5e-056e-06

2.3e-059.8e-052.2e-05

2.6e-05

6.5e-055.3e-056.4e-05

7e-060.0001240.0001456e-06

0.0064860.006730.0041920.0041330.0029930.0182870.003035

0.0064860.006730.0041920.0041330.0029930.0182870.003035

0.0064750.006730.0041920.0041330.0029390.0182780.003035

5e-064.9e-055e-06

0.000103

0.0063810.0066840.003620.0041330.0020710.0181590.003035

0.0005720.00067

8.9e-054.6e-054.6e-050.000114

1.1e-055.4e-059e-06

1.1e-055.4e-059e-06

0.0006620.000280.0005010.0002510.0006728.8e-05

0.0006620.000280.0005010.0002510.0006728.8e-05

0.0006620.000280.0005010.0002510.0006728.8e-05

0.0002196e-050.0001900.0002258.8e-05

0.00027.6e-050.0001470.0001330.000202

0.0002290.0001440.0001649.9e-050.000231

1.4e-051.9e-051.4e-05

0.0003150.0008940.000960.0002960.000403

0.0003450.000403

0.0003450.000403

0.0003450.000403

0.0003450.000403

0.0003450.000403

5e-050.0001324.2e-05

5e-050.0001324.2e-05

4.1e-059.1e-053.9e-05

4.1e-059.1e-053.9e-05

2.1e-054.3e-052e-05

2e-054.8e-051.9e-05

9e-064.1e-053e-06

9e-064.1e-053e-06

0

0

9e-064.1e-053e-06

1.2e-050.0001210.0002281.3e-05

1.2e-050.0001210.0002281.3e-05

1.2e-050.0001210.0002281.3e-05

0.0001210.000141

0.0001210.000141

1.2e-058.7e-051.3e-05

1.2e-058.7e-051.3e-05

0.0002070.0002570.0003110.000191

5.2e-050.0001180.0001383.1e-05

5.2e-050.0001180.0001383.1e-05

5.2e-050.0001180.0001383.1e-05

5.2e-050.0001180.0001383.1e-05

0.0001550.0001390.0001730.00016

0.0001550.0001390.0001730.00016

0.0001550.0001390.0001730.00016

0.0001550.0001390.0001730.00016

2.2e-050.0001710.0002111.7e-05

4e-061.1e-053e-06

4e-061.1e-053e-06

4e-061.1e-053e-06

4e-061e-053e-06

1e-06

1.8e-050.0001710.00021.4e-05

1.8e-050.0001710.00021.4e-05

1.8e-050.0001710.00021.4e-05

1.8e-050.0001710.00021.4e-05

2.4e-057.8e-053.3e-05

2.4e-057.8e-053.3e-05

2.4e-057.8e-053.3e-05

2.4e-057.8e-053.3e-05

2.4e-057.8e-053.3e-05
