# Supplementary material for: MetaMeta: integrating metagenome analysis tools to improve taxonomic profiling
Source: Microbiome. 2017 Aug 14;5:101. doi: 10.1186/s40168-017-0318-y (PMC5557516; doi:10.1186/s40168-017-0318-y)
Supplement: Supplementary file 2 — Additional File with interactive charts for all CAMI toy set results on default, very-precise and very-sensitive mode. File prefix S, M, and H for low, medium and high complexity, respectively. (TAR 3573 kb) [file 40168_2017_318_MOESM2_ESM.tar › M2_S002__insert_180_very-precise.html]

Javascript must be enabled to view this page.

magnitude
magnitudeUnassigned

clark.parsed\_profile
dudes.parsed\_profile
final.metametamerge.profile
gottcha.parsed\_profile
kaiju.parsed\_profile
kraken.parsed\_profile
motus.parsed\_profile

1.0000031.0000021.0000010.9999950000000010.9999939999999950.9999959999999980.999997

1.5e-051.1e-05

1.5e-051.1e-05

1.5e-051.1e-05

1.5e-051.1e-05

1.5e-051.1e-05

1.5e-051.1e-05

1.5e-051.1e-05

0.9203889999999970.9359869999999990.9353560.9462860000000010.9251429999999970.9235809999999980.961681

0.0554420.0326750.0534590.0349610.07003800000000010.0539160.042141

0.0551920.0326750.0534590.0349610.06879700000000010.0536880.042127

0.0074130.0046190.0134610.004560.0097020.0072130.011566

2e-050.0001012e-05

2e-050.0001012e-05

2e-050.0001012e-05

0.0001520.0001220.000144

0.0001520.0001220.000144

0.0001520.0001220.000144

0.0001520.0006120.000143

0.000284

0.000284

0.000181

0.000181

0.0001520.0001470.000143

0.0001520.0001470.000143

0.0002586.4e-050.0002010.000258

0.0002586.4e-050.0002010.000258

0.0002586.4e-050.0002010.000258

0.00460.003480.0035320.004560.00480.004420.002193

0.0034810.0029920.002940.0028370.0027990.0033440.001821

0.0034810.0029920.002940.0028370.0027990.0033440.001821

4.6e-054.4e-053.8e-05

9e-062.9e-055e-06

3.7e-051.5e-053.3e-05

4.7e-054.4e-053.9e-05

4.7e-054.4e-053.9e-05

0.0001129.6e-050.000112

0.0001129.6e-050.000112

9e-063.2e-051.4e-051e-05

9e-063.2e-051.4e-051e-05

0.0001690.0002480.0001692.3e-05

8.6e-057.6e-058.6e-052.3e-05

7.1e-05

8.3e-050.0001018.3e-05

9.8e-050.0002649.3e-05

0.000119

9.8e-056.4e-059.3e-05

8.1e-05

0.0006380.0004560.0005920.0017230.0011660.0006150.000349

6e-05

5.4e-05

1.1e-051.3e-051.1e-05

0.0006270.0004560.0005920.0017230.0005190.0006040.000349

0.000129

9e-06

6.1e-05

0.000193

6.5e-05

5.8e-05

5e-06

0.000125

0.000125

0.0004680.000110.0017150.0004696.9e-05

9.8e-05

9.8e-05

0.000102

0.000102

6.8e-05

6.8e-05

6.2e-05

6.2e-05

8.3e-05

6.5e-05

1.8e-05

6e-05

6e-05

0.0001073.1e-052.9e-050.0001071.3e-05

0.0001073.1e-052.9e-050.0001071.3e-05

7.3e-05

7.3e-05

6.4e-054.8e-056e-05

6.4e-054.8e-056e-05

0.0002977.9e-050.0010920.0003025.6e-05

0.000103

0.000103

0.000271

0.000247

0.0002977.9e-050.0002690.0003025.6e-05

9.9e-05

0.0005690.0005440.0003220.000576e-06

0.0005690.0005440.0003220.000576e-06

0.0002260.0001920.0001230.000232

0.0001660.000170.0001110.0001686e-06

0.0001770.0001828.8e-050.00017

0.0008040.0003620.0007330.000806

0.0002490.0001090.00020.000241

0.0002490.0001090.00020.000241

0.0005550.0002530.0005330.000565

0.000196

0.0005550.0002530.0003370.000565

0.0003075.9e-050.0099290.0008890.0003040.009298

0.0099290.009298

0.0099290.009298

0.00026

0.00026

0.0003075.9e-050.0004440.000304

0.0003075.9e-050.0004440.000304

0.000185

0.000185

8.2e-050.0001277.5e-05

4.7e-05

4.7e-05

8.2e-058e-057.5e-05

8.2e-058e-057.5e-05

1e-062.2e-054e-06

1e-062.2e-054e-06

1e-062.2e-054e-06

5.8e-05

5.8e-05

5.8e-05

0.0169410.0104890.0120830.0073980.0140460.0163030.009535

7.4e-054.3e-057e-05

7.4e-054.3e-057e-05

7.4e-054.3e-057e-05

5.8e-054.7e-055.3e-05

5.8e-054.7e-055.3e-05

5.8e-054.7e-055.3e-05

0.0168090.0104890.0120830.0073980.0139560.016180.009535

3.5e-057.2e-052.8e-05

3.5e-057.2e-052.8e-05

0.0167740.0104890.0120830.0073980.0138840.0161520.009535

0.0167010.0104890.0120830.0073980.0137380.0160840.009535

7.3e-050.0001466.8e-05

0.0001269.6e-050.0001032.7e-05

0.0001269.6e-050.0001032.7e-05

0.0001269.6e-050.0001032.7e-05

0.0001269.6e-050.0001032.7e-05

5e-054.2e-054.5e-051.3e-05

5e-054.2e-054.5e-051.3e-05

5e-054.2e-054.5e-051.3e-05

5e-054.2e-054.5e-051.3e-05

0.0004685e-060.0006380.000443.9e-05

0.0004685e-060.0006380.000443.9e-05

9.4e-050.0001118.7e-051.3e-05

9.4e-050.0001118.7e-051.3e-05

5.9e-05

5.9e-05

9.4e-055e-069e-059e-05

1e-05

7.7e-051.6e-057.4e-05

1.7e-055e-061.2e-051.6e-05

5.2e-05

4.1e-05

4.1e-05

4.5e-05

4.5e-05

6.5e-053.9e-056.2e-05

6.5e-053.9e-056.2e-05

4.6e-05

4.6e-05

7.5e-057e-05

7.5e-057e-05

6.3e-054.2e-055.8e-05

6.3e-054.2e-055.8e-05

7.7e-050.0001657.3e-052.6e-05

2.8e-05

1e-06

2e-06

4.4e-05

2.5e-05

7.7e-056.5e-057.3e-052.6e-05

0

0.0011260.0007480.0009910.0019250.0013710.0010940.000541

0.0011260.0007480.0009910.0019250.0013710.0010940.000541

1.9e-057.7e-053.3e-05

1.9e-057.7e-053.3e-05

2.1e-05

2.1e-05

0.0011070.0007480.0009910.0019250.0010890.0010610.000541

0.0011070.0007480.0009910.0019250.0010890.0010610.000541

4e-05

4e-05

0.000144

3.8e-05

4.8e-05

3e-05

2.8e-05

0.0109630.007450.0080620.0058780.0097040.010540.005534

0.0109630.007450.0080620.0058780.0097040.010540.005534

0.0105970.007340.0080620.0058780.0094010.0101760.005412

4.8e-056e-054.7e-05

5e-054e-054.8e-05

0.0104420.007340.0080620.0058780.0092530.0100270.005412

5.7e-054.8e-055.4e-05

4.5e-055.2e-054.7e-05

4.5e-055.2e-054.7e-05

1.6e-052e-051.7e-051.3e-05

8e-061.4e-058e-067e-06

8e-066e-069e-066e-06

0.0003050.000110.0002310.00030.000109

0.0002160.000110.0001610.0002140.000109

8.9e-057e-058.6e-05

2.7e-055.1e-052.3e-05

2.7e-055.1e-052.3e-05

2.7e-055.1e-052.3e-05

2.7e-055.1e-052.3e-05

0.0116650.0060520.0063790.0097280.0163380.0114350.004025

0.0029230.0011950.0012210.0038510.0064490.0030280.000769

0.0014040.0004440.0007370.0021280.0018150.0014380.000186

0.0003036.8e-050.0002760.0003158.5e-05

0.000173

0.0007650.0002960.0007370.0021280.0012160.000784

0.0003368e-050.000150.0003390.000101

0.0015190.0007510.0004840.0017230.0046340.001590.000583

0.000256

2.8e-05

2.6e-05

5e-05

0.000331

1.4e-05

0.0003166.5e-053.4e-050.000326

0.002164

0.0003879.3e-050.0004590.00042

0.00105

0.000219.8e-055.2e-050.0002290.000121

0.0002160.0001030.0001110.0002250.0001

0.000390.0003920.0004840.0017235.9e-050.000390.000362

2.5e-05

2.5e-05

2.5e-05

0.0001380.0001260.0001371.3e-05

0.0001380.0001260.0001371.3e-05

0.0001380.0001260.0001371.3e-05

0.0013360.000560.0021280.0034180.0013150.002978

0.0013360.000560.0021280.0034180.0013150.002978

2.3e-05

8.8e-05

6.9e-05

7.6e-053e-057.6e-057e-06

3.2e-054.2e-053.2e-05

5.3e-05

3.5e-05

5e-054e-054.4e-05

5.7e-053.8e-055.8e-052.4e-05

5.7e-05

0.0001137.4e-050.000102

1.2e-053.8e-050.0021289e-061.2e-05

0.000325

0.000142

6e-05

0.000283

3.8e-05

5.4e-05

3.3e-053.5e-053.1e-05

1.6e-05

0.000607

0.000121

6.1e-05

0.0001438.9e-059.5e-050.000138

0.0001

7.8e-050.000155e-057.8e-05

3.8e-05

0.0004510.000270.000330.0004511.3e-05

5.4e-05

3.4e-05

0.002891

5.8e-05

3.8e-05

7.3e-054.5e-057.3e-05

6e-052.2e-055.6e-05

0.0001476.4e-050.000149

7e-05

5e-06

4e-063e-065e-06

6e-061.3e-053e-069e-06

0.000152

1e-0601e-06

0.000450.0004170.0004429.1e-05

0.000450.0004170.0004429.1e-05

0.0001660.0001210.0001591.3e-05

8.8e-05

7.7e-05

0.0001454.3e-050.000145

0.0001398.8e-050.0001382.6e-05

5.2e-05

7.1e-055.3e-056.3e-05

7.1e-055.3e-056.3e-05

7.1e-055.3e-056.3e-05

0.0067470.0042970.0051580.0037490.005850.006450.000174

0.0066960.0042970.0051580.0037490.0057070.0064010.000174

0001.4e-05

0

0

5e-06

6e-063e-066e-06

8e-06

1.2e-057e-061.1e-051.3e-05

5.5e-051.6e-058e-065.7e-05

6e-06

4.2e-051.4e-054.3e-05

2e-061e-063e-06

01.3e-05

6.8e-054.8e-057.1e-05

2.6e-054e-062.9e-05

1.3e-05

4e-061e-064e-06

1.2e-05

1.1e-054e-068e-06

5.2e-052.1e-055.3e-056.2e-05

6e-066e-068e-06

5e-06

4e-061e-065e-06

5.7e-05

3.5e-052e-063.9e-05

9.9e-051e-06

5.5e-05

2e-061e-062e-06

0001.3e-05

8e-054.3e-058.2e-05

000

9.9e-05

1.7e-05

0.0060620.0042810.0051580.0037490.0050370.005834

5.5e-054.5e-055.9e-05

5e-06

4.8e-05

1e-064e-061e-06

7.4e-053.6e-058.6e-054.6e-05

6e-05

4.3e-05

5.1e-050.0001434.9e-05

5.1e-050.0001434.9e-05

0.0001770.000490.0037490.0003330.0002120.000496

0.0001770.000490.0037490.0003330.0002120.000496

2.5e-05

2.5e-05

6.4e-05

6.4e-05

1e-061e-051e-06

1e-061e-051e-06

0.0001760.000490.0037490.0002340.0002110.000496

1.5e-056e-068e-06

4e-062e-067e-06

7e-062.9e-059e-06

2.8e-05

4e-06

1e-06

2e-051.2e-051.6e-05

1e-06

1.8e-053e-061.7e-05

3.4e-05

8e-064e-061.5e-05

1.3e-050.0018241e-051.1e-051.3e-05

1.5e-05

9.1e-050.000490.0019253.9e-050.0001280.000483

1e-05

1e-06

3.5e-05

4.6e-054.2e-054.5e-05

4.6e-054.2e-054.5e-05

4.6e-054.2e-054.5e-05

4.6e-054.2e-054.5e-05

0.0001670.0002030.00016

0.0001670.0002030.00016

0.0001670.0002030.00016

3.4e-054.1e-053.4e-05

3.6e-055.3e-053.4e-05

2.6e-053.2e-052.4e-05

3.6e-053.4e-053.3e-05

3.5e-054.3e-053.5e-05

6.3e-056.6e-056.1e-05

6.3e-056.6e-056.1e-05

6.3e-056.6e-056.1e-05

6.3e-056.6e-056.1e-05

0.000210.0001730.0001931.3e-05

0.000210.0001730.0001931.3e-05

5.6e-056.3e-055.2e-05

5.6e-056.3e-055.2e-05

8.2e-055.8e-057.1e-05

8.2e-055.8e-057.1e-05

7.2e-055.2e-057e-051.3e-05

7.2e-055.2e-057e-051.3e-05

0.0052810.0028220.0124830.0017230.0153070.0053940.010338

0.0052810.0028220.0124830.0017230.0153070.0053940.010338

0.0051660.0028220.0124830.0017230.0152640.00530.010338

3.4e-05

2.2e-05

0.00023.3e-053.9e-050.000213

0.0003420.0002422.2e-050.000374e-06

6.7e-05

3.7e-05

0.000108

0.0001340.0003041.2e-050.00039e-05

0.000212

3e-050.0001244e-063.2e-05

1.3e-05

2.3e-05

5e-061e-05

0.0002855.7e-053.6e-050.0003242e-05

0.0003370.000110.000384

0.000256

2.1e-05

6.6e-05

3e-06

3.1e-050.0001224e-063.4e-05

4.1e-05

0.0008720.0006030.000650.0017230.0005720.0008620.000281

3.6e-05

0.0007150.0004091.5e-050.000761

5.3e-05

0.0003790.0001521.1e-050.0004188e-06

3.3e-05

2.6e-05

7e-06

1.1e-05

3.8e-05

0.0118330.0133320.009481

1.2e-05

0.0003557.8e-051.2e-050.00038

7.1e-05

2.5e-05

3.4e-05

1.7e-05

0.0004020.0001333.6e-050.000434

3.4e-05

2.4e-05

8.7e-052.4e-051.9e-059e-05

0.0002847e-052.5e-050.000317

2.3e-05

3e-06

1.4e-05

0.0003570.0002491.8e-050.00038

1.6e-05

3e-05

0.0003568.9e-053.6e-051e-066.9e-05

1.3e-05

1.8e-05

2.3e-05

0.0001154.3e-059.4e-05

0.0001154.3e-059.4e-05

6.6e-058.2e-055.5e-05

6.6e-058.2e-055.5e-05

3.3e-05

3.3e-05

6.6e-054.9e-055.5e-05

6.6e-054.9e-055.5e-05

0.0004030.0006030.000372

0.0001850.0004410.000167

8.8e-05

8.8e-05

7.8e-057.1e-057.2e-05

7.8e-057.1e-057.2e-05

8.1e-05

8.1e-05

0.0001070.0002019.5e-05

0.0001079.5e-059.5e-05

0.000106

0.0002180.0001620.000205

4.3e-056.7e-054.4e-05

4.3e-056.7e-054.4e-05

4.8e-051.6e-054.4e-05

3e-062e-062e-06

4.5e-051.4e-054.2e-05

3.3e-053.2e-052.8e-05

3.3e-053.2e-052.8e-05

4.8e-051.7e-054.7e-05

4.8e-051.7e-054.7e-05

4.6e-053e-054.2e-05

1e-05

4.6e-052e-054.2e-05

4.9e-058.3e-053.6e-05

4.9e-058.3e-053.6e-05

4.9e-058.3e-053.6e-05

3.3e-053.6e-052.1e-05

3.3e-053.6e-052.1e-05

1.6e-054.7e-051.5e-05

1.6e-054.7e-051.5e-05

1.4e-059.1e-051.1e-05

1.4e-059.1e-051.1e-05

1.4e-059.1e-051.1e-05

1.4e-059.1e-051.1e-05

1.4e-055.5e-051.1e-05

3.6e-05

0.0001440.0010.000146

8.7e-050.0005129.8e-05

8.7e-050.0005129.8e-05

4e-067e-055e-06

4e-067e-055e-06

0.000101

0.000101

2e-058e-053.3e-05

2e-058e-053.3e-05

1.8e-050.000151.4e-05

1e-059.3e-058e-06

8e-065.7e-056e-06

3.3e-050.0001113.7e-05

3.3e-050.0001113.7e-05

1.2e-059e-06

1.2e-059e-06

5.7e-050.0004884.8e-05

1.1e-050.0001278e-06

4.8e-05

4.8e-05

1.1e-057.9e-058e-06

1.1e-057.9e-058e-06

4.6e-050.0003614e-05

1.9e-050.0002391.8e-05

1.9e-050.0002391.8e-05

2.7e-050.0001222.2e-05

2.7e-056.1e-052.2e-05

6.1e-05

4.3e-056.7e-053.5e-051.4e-05

4.3e-056.7e-053.5e-051.4e-05

4.3e-056.7e-053.5e-051.4e-05

4.3e-056.7e-053.5e-051.4e-05

4.3e-056.7e-053.5e-051.4e-05

0.0002070.0011450.00030.0024320.0002130.0001760.001393

0.0002070.0011450.00030.0024320.0002130.0001760.001393

0.0001990.0011450.00030.0024320.0001450.0001690.001393

0.0001990.0011450.00030.0024320.0001450.0001690.001393

0.0001990.0011450.00030.0024320.0001450.0001690.001393

01e-060

0.0001910.0011450.00030.0024320.0001250.0001640.001393

3e-0601e-06

000

2e-06

1e-063e-061e-06

7e-06

1e-062e-060

2e-061e-062e-06

1e-064e-061e-06

8e-066.8e-057e-06

1e-069e-061e-06

1e-069e-061e-06

1e-069e-061e-06

5e-064.5e-055e-06

5e-063.4e-055e-06

5e-065e-06

3.4e-05

01.1e-050

01.1e-050

2e-061.4e-051e-06

2e-061.4e-051e-06

2e-061.4e-051e-06

4e-050.0003283.8e-05

4e-050.0003283.8e-05

2.1e-050.000172e-05

9e-069e-059e-06

7e-064.4e-058e-06

1e-065e-061e-06

6e-063.6e-057e-06

02e-06

1e-06

2e-064.6e-051e-06

0

01e-060

0

1e-05

2e-061e-061e-06

1e-06

0

0

1.7e-05

1.6e-05

1.2e-058e-051.1e-05

1e-056.1e-057e-06

7e-064.5e-055e-06

3e-061.6e-052e-06

2e-061.9e-054e-06

6e-06

1e-068e-063e-06

1e-065e-061e-06

3e-067.5e-053e-06

3e-067.5e-053e-06

2e-065.3e-051e-06

2.9e-05

2e-062.4e-051e-06

1e-062.2e-052e-06

1e-062.2e-052e-06

1.6e-058.3e-051.5e-05

1.6e-058.3e-051.5e-05

2e-063.2e-052e-06

2e-063.2e-052e-06

1.4e-052.5e-051.3e-05

1.4e-052.5e-051.3e-05

2.6e-05

2.6e-05

2.4e-05

2.4e-05

2.4e-05

2.4e-05

2.4e-05

2.4e-05

1.6e-050.0001741.1e-05

1.6e-050.0001741.1e-05

1.6e-050.0001741.1e-05

1.6e-050.0001741.1e-05

4e-068.5e-052e-06

3e-05

4e-062.7e-052e-06

2.8e-05

8e-063e-057e-06

3e-062e-062e-06

5e-062.3e-055e-06

5e-06

4e-065.9e-052e-06

4e-065.9e-052e-06

0.1162970.1605020.2087550.2129110.2052370.1145470.299428

0.07371400000000010.1267870.1619740.1804820.147990.0737110.278657

0.0228310.0416220.0539820.0850210.0285290.0224310.071919

0.0005950.0013130.0005970.0065860.0006180.0005640.00144

0.0005670.0013130.0005970.0065860.0005980.0005380.00144

1.1e-05

1.6e-05

1.5e-05

5.2e-05

1.4e-05

1e-061e-061e-06

01e-060

3.2e-05

1e-05

4e-066e-064e-06

4e-063e-063e-06

1.7e-05

8e-068e-068e-06

1.1e-05

3e-060.0027361e-063e-06

1.2e-051e-05

0.0001630.0001960.0016217e-050.0001570.000179

4e-061e-065e-06

8e-064e-067e-06

2.4e-05

6e-061.1e-052e-06

4e-061.4e-053e-06

03e-060

1e-05

0.00030.0011170.0005970.0022295e-050.0002880.001261

1e-063e-061e-06

3e-05

1.1e-050.0001049e-06

4e-06

5e-067e-063e-06

3e-062.1e-051.1e-05

8e-06

2.9e-051e-052.2e-05

8e-06

1e-061.8e-051e-06

2.8e-052e-052.6e-05

1.4e-051.1e-051.2e-05

1.4e-055e-061.4e-05

4e-06

6.2e-050.0001915.4e-05

1.1e-052.9e-051.3e-05

1.1e-052.9e-051.3e-05

2.5e-057.1e-051.8e-05

5e-061e-053e-06

01e-060

1.1e-051.6e-058e-06

1.4e-05

3e-061.8e-052e-06

5e-069e-064e-06

1e-063e-061e-06

2.6e-059.1e-052.3e-05

3.9e-05

2.6e-054e-052.3e-05

1.2e-05

0.0014710.0012210.00141

0.0014710.0012210.00141

2.4e-055.5e-052.2e-05

1.2e-052.6e-051e-05

0.0014350.0010910.001378

4.9e-05

0.0043720.0035750.0029380.0054720.0017180.0042020.00356

0.0043280.0035750.0029380.0054720.0016450.0041580.00356

8e-061.1e-057e-06

0.000327

5.1e-054.7e-054.6e-05

0.0004140.0010340.0004090.0023310.000120.0003960.001026

0.0038430.0025410.0025290.0031410.0011160.0036980.002534

1.5e-05

1.2e-059e-061.1e-05

2.3e-052.6e-052.5e-05

2.3e-052.6e-052.5e-05

2.1e-054.7e-051.9e-05

2.1e-054.7e-051.9e-05

0.0163120.0367340.0504470.0729630.0245890.016190.066919

4.1e-050.0002295.7e-052.7e-05

1e-055.8e-053.2e-05

3.1e-054.4e-052.5e-052.7e-05

0.000127

0.0162710.0367340.0504470.0729630.024360.0161330.066892

0.0001210.0001790.0016216.2e-050.000126

9e-059e-069.4e-05

1.6e-05

0.000171

0.000820.0005960.000610.0003310.0008150.000623

8.5e-053.4e-059.2e-05

5.7e-058.1e-055.5e-05

3.2e-052.4e-053.3e-052.7e-050.000114

0.0001260.0003520.000137

0.000432

0.0040020.003747

0.000228

0.000106

0.0098170.009193

7.3e-055.4e-050.003040.0003440.000342

7.4e-05

1e-053.2e-050.0001083.7e-05

8.3e-050.0006141.1e-058.4e-050.000117

0.0019320.0025580.0015510.0026350.00060.001260.002627

0.0019710.0009260.0011260.0147950.000320.0019740.002509

0.000224

5.4e-059.7e-059.1e-051.7e-05

1.3e-05

0.0009550.0002730.0001370.0009740.000131

7.4e-05

3.5e-051.2e-050.0045450.0042562.9e-053.3e-051.4e-05

0.0001428.1e-050.000161

0.000268

7.9e-052.8e-050.0003278.2e-05

0.0004140.0006949.3e-050.000412

0.000232

0.000285

0.000702

0.0001840.0004190.000183

0.000265

5.9e-05

0.0027180.0009640.0008850.0073980.0002880.0027440.000539

3.3e-05

0.0040770.0285070.0092810.0373946.8e-050.0040610.045459

0.0181240.016973

0.0005049.4e-050.0002330.000511.4e-05

4.1e-050.0001840.0001050.000133

6.5e-052.9e-052.8e-056.8e-053.9e-05

0.0007240.000810.0005060.0018240.0001670.000721

0.0001260.0001130.000134

0.000273

0.0003577.4e-050.0001030.0003661.4e-05

0.0003968.2e-050.0002450.000417

0.000849

1.9e-050.0001921.1e-05

1.9e-050.0001921.1e-05

3.1e-05

5.6e-05

4.6e-05

1.9e-052.5e-051.1e-05

1.3e-05

2.1e-05

0.0508830.0851650.1079920.0954610.1194610.051280.206738

1e-052.9e-057e-06

1e-052.9e-057e-06

1e-052.9e-057e-06

0.0026510.0013280.0772390.0068910.0946520.0027380.058218

0.0002865.6e-050.0756030.0924190.0002860.057426

0.0756030.09230.057426

0.0002865.6e-050.0001190.000286

0.0023310.0012720.0016360.0068910.0021140.0024260.000792

1.9e-05

3.7e-05

9e-06

9e-061.8e-057e-06

5e-05

1.6e-05

1e-05

1e-05

3.8e-05

4e-054.2e-054.1e-05

0.0004150.0002570.0004440.0017230.0003740.000403

4.8e-05

8.9e-05

0.0004890.0005320.000340.0022290.0001240.0002530.000409

6.7e-05

1.2e-05

7.4e-05

0.0013630.0004830.0008520.0029390.0008520.0017050.000383

1.9e-05

1.1e-05

4e-05

1.5e-054.8e-051.7e-05

1.1e-05

2.8e-05

1.2e-05

1.3e-05

9e-06

3.4e-05

3.4e-054.5e-052.6e-05

3.4e-054.5e-052.6e-05

7.4e-05

7.4e-05

6.1e-052.9e-055.2e-05

6.1e-052.9e-055.2e-05

8e-061e-055e-06

1.7e-052e-061.3e-05

1e-0601e-06

1.9e-051.3e-051.7e-05

1.6e-054e-061.6e-05

0.0476390.0835560.0303540.0868470.0232630.047720.148302

0.0005020.0001710.0018240.0007120.0005038.5e-05

0.0005020.0001710.0018240.0001530.0005038.5e-05

5.9e-05

0.0005

6.6e-05

2.8e-05

3.8e-05

5.6e-05

1.2e-05

3.9e-05

5e-06

4e-065e-063e-06

4e-065e-063e-06

3.8e-05

3.8e-05

0.014570.057150.0056280.0686060.0032130.015930.14666

0

0.000104

5.9e-055.8e-055.3e-05

0.000191

0

0.001220.0010320.0005650.0094240.0001160.0012170.104302

9.9e-05

0.0037450.0007880.0008750.0051680.0002430.003745

0.0003819.6e-050.00042

0.0013280.0173990.0026630.0080069.5e-050.0016620.002136

0.0007410.0002040.0003120.006790.0001060.000753

8.5e-05

4.8e-059e-064.9e-05

4.8e-050.0001040.0026354e-064.7e-050.000183

0.0006430.0002530.0004050.0185450.000160.000725

3.7e-05

6e-057e-06

0.005960.0373660.0008080.0180380.0001330.0069160.039925

3e-06

0.0001318.6e-05

3.4e-058.3e-053.3e-05

4e-062e-065e-06

5.9e-05

3.3e-051.1e-053.1e-052.8e-05

9.3e-05

1.3e-05

5e-06

4e-064e-062e-065e-06

2e-06

2e-052.7e-051.8e-05

1.2e-052.1e-051.5e-05

4e-05

3.1e-055.6e-052.8e-05

8.6e-05

8.3e-050.0001028.6e-05

3e-061e-064e-06

1.5e-054.5e-051.4e-05

8.5e-05

0.000116

0.000557

8e-05

4.7e-05

9.8e-053e-060.000104

0.032470.0262350.0247260.0164170.0188610.0312010.001557

2e-061.4e-055e-06

07.8e-05

2.7e-05

3e-061.6e-053e-06

1e-061.9e-052e-061.5e-05

3.6e-05

5.7e-05

5e-069e-064e-060.000221

9e-06

3.5e-054e-053.2e-051.4e-05

2e-067.7e-05

0.0324070.0262350.0247260.0164170.0185430.031136

2.6e-05

3e-067e-063e-060.000954

1.4e-055.6e-051.6e-050.000198

4.7e-055e-054.2e-05

4.7e-055e-054.2e-05

1.1e-053.9e-059e-06

1.1e-053.9e-059e-06

4.7e-05

4.7e-05

3.5e-055.3e-053.2e-05

3.5e-055.3e-053.2e-05

5.1e-05

5.1e-05

7.2e-05

7.2e-05

3.4e-050.0004813.3e-05

5.4e-05

5.4e-05

0.000121

0.000121

0.000113

1e-05

1.7e-05

1.5e-05

1.7e-05

7e-06

8e-06

1.6e-05

9e-06

1.4e-05

3.4e-052.8e-053.3e-05

3.4e-052.8e-053.3e-05

0.000104

0.000104

6.1e-05

6.1e-05

0.0001480.0001710.000146

0.0001168.5e-050.000124

8e-064e-069e-06

1e-051e-061.6e-05

3e-06

1e-06

7e-066e-067e-06

2.3e-055e-062.7e-05

5e-061e-065e-06

4e-061e-062e-06

2e-06

2.2e-051e-052.1e-05

2e-054e-062.2e-05

3e-06

1e-065e-061e-06

4e-06

7e-06

1.6e-052e-061.4e-05

0

5e-06

2.1e-05

3.2e-054.9e-052.2e-05

3.2e-054.9e-052.2e-05

3.7e-05

3.7e-05

0.0002690.0002810.0003990.0017230.0006530.0005160.000218

0.0002670.0002810.0003990.0017230.0006120.0005140.000218

0.0002670.0002810.0003990.0017230.0006120.0005140.000218

2e-064.1e-052e-06

2e-064.1e-052e-06

7.1e-050.0001836.8e-05

7.1e-056.9e-056.8e-05

3.7e-054e-053e-05

1.1e-051.6e-051.5e-05

2e-06

1.6e-052e-061.8e-05

7e-069e-065e-06

0.000114

0.000114

0.00470.0031450.0039170.0038510.0051510.0045030.002645

0.004620.0031450.0039170.0038510.0043790.0044340.002645

0.004620.0031450.0039170.0038510.0043790.0044340.002645

0.004620.0031450.0039170.0038510.0043790.0044340.002645

1.5e-050.0001491.4e-05

0.0046050.0031450.0039170.0038510.004230.004420.002645

2.5e-050.0003572.1e-05

8.3e-05

8.3e-05

2.6e-05

5.7e-05

2.5e-050.0002742.1e-05

2.5e-050.0002742.1e-05

4e-06

4.8e-05

1e-050.0001079e-06

1.5e-050.0001021.2e-05

1.3e-05

5.5e-050.0004154.8e-05

5.5e-050.0004154.8e-05

3.6e-057.6e-053.4e-05

3.6e-057.6e-053.4e-05

0.000175

0.000175

1.9e-050.0001641.4e-05

1.9e-050.0001641.4e-05

0.0001970.0116580.0028180.0001850.010918

0.0001970.0116580.0028180.0001850.010918

0.0001970.0116580.0028180.0001850.010918

0.0116580.0022880.010918

0.002288

0.0116580.010918

0.000273

0.000273

0.0001820.000179

0.0001820.000179

1.5e-050.000116e-06

8.9e-05

1.5e-052.1e-056e-06

0.000147

0.000147

2.3e-050.0005392.3e-05

2.3e-050.0005392.3e-05

2.3e-050.0005392.3e-05

1.7e-050.0001081.8e-05

1.7e-050.0001081.8e-05

6e-060.0001585e-06

6e-060.0001585e-06

0.000105

0.000105

0.000168

0.000168

5.1e-05

5.1e-05

5.1e-05

5.1e-05

5.1e-05

0.0376630.030570.0312060.0285780.0486880.0361250.007208

2e-050.0002922.2e-05

1.2e-059.9e-051.5e-05

1e-062.8e-051e-06

1e-062.8e-051e-06

1.1e-057.1e-051.4e-05

1.1e-057.1e-051.4e-05

8e-060.0001937e-06

7e-060.0001136e-06

3e-066.8e-054e-06

4e-064.5e-052e-06

1e-068e-051e-06

1e-068e-051e-06

0.037560.030570.0312060.0285780.0475050.036030.007208

0.0323660.0245970.0280240.019660.0287930.031094

1.6e-055.3e-051.4e-05

1.6e-055.3e-051.4e-05

0.0322490.0245970.0280240.019660.0282850.030955

1.8e-056e-051.6e-05

0.0322040.0245970.0280240.019660.0280870.030918

4e-065.2e-054e-06

6e-063.8e-056e-06

6e-06

1.1e-054.8e-051.1e-05

6e-06

6e-06

01.6e-051e-06

05e-061e-06

01e-060

1e-05

1.5e-058.7e-051.8e-05

1.5e-058.7e-051.8e-05

3e-050.0001645.3e-05

5.2e-05

8e-064.7e-059e-06

2e-062.9e-051.1e-05

2e-053.6e-053.3e-05

2.7e-050.0001182.4e-05

1e-063.7e-051e-06

2e-054e-051.8e-05

6e-064.1e-055e-06

2.3e-057e-052.9e-05

2.3e-057e-052.9e-05

6.2e-050.0006946.2e-052.8e-05

6.2e-050.0006946.2e-052.8e-05

6.2e-050.0006946.2e-052.8e-05

0.0002690.0012410.0042560.0028670.0002340.003013

4.6e-050.00031.5e-050.000647

7e-06

4.6e-050.00031.5e-05

0.000197

0.000443

0.0001810.000623

0.000181

0.000623

4e-060.0002685e-061.4e-05

4e-060.0002685e-06

1.4e-05

0.0001780.0042560.0004970.0001751.4e-05

0.0001780.0042560.0004970.0001751.4e-05

0.000559

0.000559

0.0002380.00098

0.000238

0.00098

3.3e-050.001060.0008333.2e-050.000721

8e-060.0002361.8e-05

7e-06

2.5e-050.001060.0002881.4e-050.000714

0.000309

1.4e-05

1.4e-05

8e-060.0001727e-06

8e-060.0001727e-06

6e-060.0001735e-06

1e-066.7e-051e-06

1e-066.7e-051e-06

5e-060.0001064e-06

5e-060.0001064e-06

0.0039950.0026760.0031820.0029390.0054730.0038760.003716

7.1e-05

7.1e-05

9e-064.7e-051e-05

0

4e-062e-064e-06

5e-064.5e-056e-06

0.0039540.0026760.0031820.0029390.0052360.0038320.003716

8e-062.6e-058e-06

1.1e-057.4e-051.2e-05

2.2e-05

7e-061e-068e-06

9e-06

7.1e-05

0.000107

8e-064.1e-058e-06

2.2e-05

7e-062.2e-058e-06

8e-061.1e-058e-06

7e-063.5e-051.7e-05

1.3e-052.7e-051e-05

4e-062e-054e-06

0.001314

6e-066e-068e-061e-05

0.0038220.002670.0031820.0029390.0038210.0036860.001985

0.0005540.000417

6.6e-05

6.2e-05

9e-067.2e-059e-06

2.9e-05

3e-067.4e-052e-06

2.7e-053e-052.7e-05

1.4e-053.2e-051.5e-05

3.2e-050.0001193.4e-05

2.6e-057.3e-052.4e-05

6e-064.6e-051e-05

0.000662

0.000662

0.000662

2.2e-057e-051.6e-05

2.2e-057e-051.6e-05

2.2e-057e-051.6e-05

2.4e-055e-051.8e-05

2.4e-055e-051.8e-05

2.4e-055e-051.8e-05

1e-050.0014220.0033761.2e-058.2e-05

1e-050.0001531.2e-05

1e-050.0001531.2e-05

0.000795

0.000795

0.002428

0.002428

8.2e-05

8.2e-05

0.001422

0.001422

0.0004550.0002150.0039760.0004715.7e-05

2.6e-050.0022722.4e-05

1.8e-050.0001431.7e-05

0.000878

0.000933

3e-064.1e-053e-06

1e-060.0002211e-06

4e-065.6e-053e-06

0.0003120.0001680.0011290.000319

0.000636

0.000168

3.8e-050.0004934.9e-05

0.0002740.00027

7.7e-050.0004178.9e-05

7.7e-050.0004178.9e-05

4e-050.0001583.9e-05

4e-050.0001583.9e-05

1.6e-05

1.6e-05

4.7e-054.1e-05

4.7e-054.1e-05

0.0001090.0007320.000104

0.0001010.0005859.7e-05

5.5e-050.0001035.3e-05

4.6e-050.0004824.4e-05

8e-060.0001477e-06

8e-060.0001477e-06

0.0002070.0004190.0017230.0005490.0001070.000312

0.0001750.0004190.0017230.0001257.5e-050.000312

0.0001750.0004190.0017230.0001257.5e-050.000312

8.9e-05

8.9e-05

1.3e-050.0001481.3e-05

1.3e-050.0001481.3e-05

1.9e-050.0001871.9e-05

1.9e-050.0001871.9e-05

3.5e-059e-053.1e-05

3.5e-059e-053.1e-05

3.5e-059e-053.1e-05

2e-064e-052e-06

2e-064e-052e-06

2e-064e-052e-06

2e-064e-052e-06

8.1e-050.0008517.1e-05

2e-060.0001752e-06

2e-060.0001752e-06

2e-060.0001752e-06

5e-066.4e-051e-06

2e-063.7e-050

2e-063.7e-050

3e-062.7e-051e-06

3e-062.7e-051e-06

4.9e-050.0003813.7e-05

4e-067.3e-052e-06

4e-067.3e-052e-06

2e-065.2e-052e-06

2e-065.2e-052e-06

2e-067.6e-053e-06

2e-067.6e-053e-06

2.4e-051.5e-051.9e-05

2.4e-051.5e-051.9e-05

4e-067.3e-051e-06

4e-067.3e-051e-06

4e-063.3e-054e-06

04e-060

0

0

2e-064e-062e-06

2e-061e-062e-06

2.4e-05

9e-065.9e-056e-06

9e-065.9e-056e-06

2.5e-050.0002313.1e-05

1.4e-057e-052.1e-05

7e-064.1e-051.4e-05

7e-062.9e-057e-06

2e-069.5e-051e-06

2e-069.5e-051e-06

9e-066.6e-059e-06

02.5e-050

1e-061e-061e-06

4e-063e-064e-06

02e-061e-06

1e-06

1e-069e-061e-06

06e-060

3e-061.9e-052e-06

3e-063e-052e-06

3e-05

3e-05

3e-05

3e-05

3e-05

3e-062e-06

3e-062e-06

3e-062e-06

3e-062e-06

3e-062e-06

0.0085350.0062440.0067630.0062830.0076990.008170.00461

0.0085350.0062440.0067630.0062830.0076990.008170.00461

0.0001240.000259.5e-05

0.0001010.0002027.2e-05

0.0001010.0002027.2e-05

1.4e-052.9e-051e-05

1.7e-052.2e-051e-05

1.3e-05

8e-06

1.2e-051.6e-059e-06

1.9e-052.4e-051.3e-05

1e-05

7e-068e-065e-06

1e-052.9e-059e-06

2.2e-052.8e-051.6e-05

1.5e-05

2.3e-054.8e-052.3e-05

2.3e-054.8e-052.3e-05

2.3e-054.8e-052.3e-05

0.0084110.0062440.0067630.0062830.0074490.0080750.00461

0.0084110.0062440.0067630.0062830.0074490.0080750.00461

0.0007910.0006390.0007430.0018240.0007840.0007630.000376

0.0007910.0006390.0007430.0018240.0007840.0007630.000376

2.1e-054.8e-051.6e-05

2.1e-054.8e-051.6e-05

0.0075870.0056050.006020.0044590.0065580.0072840.004234

4e-061.7e-055e-06

6e-062e-054e-06

4e-06

3e-052.4e-05

0.0075740.0056050.006020.0044590.0064730.0072730.00421

3e-061.4e-052e-06

1.2e-055.9e-051.2e-05

4e-062.4e-054e-06

8e-063.5e-058e-06

8.7e-050.0082270.0005256.4e-050.007705

2.8e-050.0001571.8e-05

9e-068.3e-056e-06

9e-068.3e-056e-06

9e-068.3e-056e-06

9e-068.3e-056e-06

1.9e-057.4e-051.2e-05

1.9e-057.4e-051.2e-05

1.9e-057.4e-051.2e-05

1.9e-057.4e-051.2e-05

9e-060.0001977e-06

9e-060.0001977e-06

9e-060.0001977e-06

0.000142

0.000142

9e-065.5e-057e-06

9e-065.5e-057e-06

6e-065.5e-054e-06

6e-065.5e-054e-06

6e-065.5e-054e-06

6e-065.5e-054e-06

6e-065.5e-054e-06

2e-056.3e-051.4e-05

2e-056.3e-051.4e-05

4e-062.1e-053e-06

4e-062.1e-053e-06

4e-062.1e-053e-06

0

0

1.6e-054.2e-051.1e-05

1.6e-054.2e-051.1e-05

7e-062.3e-056e-06

9e-061.9e-055e-06

0.0082270.007705

0.0082270.007705

0.0082270.007705

0.0082270.007705

0.0082270.007705

2.4e-055.3e-052.1e-05

1.6e-054.8e-051.4e-05

1.6e-054.8e-051.4e-05

1.6e-054.8e-051.4e-05

1.6e-054.8e-051.4e-05

8e-065e-067e-06

8e-065e-067e-06

8e-065e-067e-06

0

8e-065e-067e-06

3.9e-050.0002453.2e-05

3.9e-050.0002453.2e-05

3.9e-050.0002453.2e-05

3.9e-050.0002453.2e-05

4e-063.8e-051e-06

4e-063.8e-051e-06

3e-054.4e-052.8e-05

3e-054.4e-052.8e-05

3e-063.3e-052e-06

3e-063.3e-052e-06

2e-061.8e-051e-06

2e-061.8e-051e-06

0.000112

0.000112

0.0277870.0170290.0198380.0130730.0270660.0267880.012961

1.5e-053.4e-051.6e-05

1.5e-053.4e-051.6e-05

1.5e-053.4e-051.6e-05

1.5e-053.4e-051.6e-05

1.5e-053.4e-051.6e-05

0.0277720.0170290.0198380.0130730.0270320.0267720.012961

0.0277720.0170290.0198380.0130730.0270320.0267720.012961

0.0277630.0170290.0198380.0130730.0269920.0267660.012961

6e-063.2e-055e-06

6e-063.2e-055e-06

2e-062.2e-051e-06

2e-062.2e-051e-06

0.0277510.0170290.0198380.0130730.0268880.0267580.012961

0.0277510.0170290.0198380.0130730.0268880.0267580.012961

4e-062.5e-052e-06

4e-062.5e-052e-06

2.5e-05

2.5e-05

9e-064e-056e-06

5e-062.5e-053e-06

5e-062.5e-053e-06

4e-061.5e-053e-06

4e-061.5e-053e-06

0.0267710.0272750.024422

0.0267710.0272750.024422

0.0267710.0272750.024422

0.0267710.0272750.024422

0.0267710.0272750.024422

0.0267710.0272750.024422

0.0052960.0019760.0101990.0033440.0157870.0052790.001347

2.2e-059.9e-051.9e-05

2.2e-059.9e-051.9e-05

2.2e-059.9e-051.9e-05

2.2e-059.9e-051.9e-05

1.6e-055.8e-051.4e-05

6e-064.1e-055e-06

0.0052740.0019760.0101990.0033440.0156880.005260.001347

7.3e-050.0002257.8e-05

4.5e-050.0001164.8e-05

4.5e-050.0001164.8e-05

4.5e-050.0001164.8e-05

2.8e-050.0001093e-05

2.8e-050.0001093e-05

2.8e-050.0001093e-05

0.0039530.0019760.0027070.0033440.0045230.0038690.001347

6e-061.2e-056e-06

6e-061.2e-056e-06

2e-06

6e-068e-066e-06

2e-06

4e-054.1e-05

4e-054.1e-05

4e-054.1e-05

0.0001510.0005420.000145

1.8e-056.9e-051.5e-05

1.1e-056.9e-051e-05

7e-065e-06

1.3e-057.2e-051.4e-05

1.3e-057.2e-051.4e-05

0.0001020.0003730.000104

1.1e-051e-062.6e-05

1.2e-056e-065e-06

1e-061.3e-051e-06

4e-06

9e-064e-068e-06

7e-06

1e-06

3e-06

7e-063.9e-058e-06

1e-053.4e-059e-06

2e-06

1.3e-058.4e-051.1e-05

6e-061e-056e-06

1.4e-05

1e-06

5e-0606e-06

5e-063.2e-054e-06

1.4e-05

4e-06

1.5e-051.1e-051.1e-05

8e-068.9e-059e-06

1.8e-052.8e-051.2e-05

1.8e-052.8e-051.2e-05

2.2e-055.5e-052e-05

2.2e-055.5e-052e-05

2.2e-055.5e-052e-05

0.0037260.0019760.0027070.0033440.0036050.0036480.001347

0.0037260.0019760.0027070.0033440.0036050.0036480.001347

0.0037260.0019760.0027070.0033440.0036050.0036480.001347

3e-063e-053e-06

3e-063e-053e-06

3e-063e-063e-06

2.7e-05

5e-060.0002796e-06

5e-060.0002796e-06

5e-064e-056e-06

0.000151

8.8e-05

0.0008440.0074920.0083120.000888

0.0006420.0007840.000675

0.0001750.0002110.000154

6.3e-055e-056.9e-05

3.7e-050

5.7e-05

7.5e-050.0001048.5e-05

0.0003410.0005220.000379

9.2e-059.7e-050.000106

9.4e-05

8.5e-050.0001389.1e-05

9.1e-050.0001099.7e-05

7.3e-058.4e-058.5e-05

7.7e-058.9e-05

7.7e-058.9e-05

4.9e-055.1e-055.3e-05

4.9e-055.1e-055.3e-05

0.0002020.0005120.000213

5.3e-050.0001215.4e-05

5.3e-050.0001215.4e-05

0.0001490.0003910.000159

0.000133

5.5e-050.0001265.6e-05

9.4e-050.0001320.000103

0.0074920.007016

0.0074920.007016

0.0074920.007016

0.0001030.0008890.000114

5.2e-050.000735.8e-05

5.2e-050.0006595.8e-05

5.2e-050.0006595.8e-05

7.1e-05

3.7e-05

3.4e-05

2e-059.6e-052.2e-05

9e-063.6e-051.1e-05

9e-063.6e-051.1e-05

1.1e-056e-051.1e-05

1.1e-056e-051.1e-05

1.6e-052.4e-051.7e-05

1.6e-052.4e-051.7e-05

1.1e-05

1.6e-051.3e-051.7e-05

1.5e-053.9e-051.7e-05

1.5e-053.9e-051.7e-05

1.5e-053.9e-051.7e-05

0.0002350.0011970.00024

2.3e-050.0001361.2e-05

2.3e-050.0001361.2e-05

2.3e-050.0001361.2e-05

5.3e-050.000235.5e-05

2.9e-050.000183e-05

2.9e-050.000183e-05

1.4e-051.4e-05

1.4e-051.4e-05

1e-055e-051.1e-05

1e-055e-051.1e-05

5.5e-050.0003285.9e-05

5.5e-050.0003285.9e-05

8e-060.0001267e-06

1.2e-056.9e-051.7e-05

2e-066e-063e-06

1e-067e-062e-06

1.9e-056.8e-051.7e-05

1.3e-055.2e-051.3e-05

5.8e-050.000276.6e-05

5.8e-050.000276.6e-05

5.8e-050.000276.6e-05

4.6e-050.0002334.8e-05

4.6e-050.0002334.8e-05

3.3e-050.0001433.4e-05

1.3e-059e-051.4e-05

4.2e-05

4.2e-05

4.2e-05

4.2e-05

6.6e-050.00057.1e-05

6.6e-050.00057.1e-05

6.6e-050.00057.1e-05

6.6e-050.00057.1e-05

4e-068.9e-053e-06

4e-068.9e-053e-06

4e-068.9e-053e-06

3e-063e-052e-06

3e-063e-052e-06

3e-063e-052e-06

1e-065.9e-051e-06

1e-065.9e-051e-06

1e-065.9e-051e-06

0.0048330.0016230.0015330.0132760.0202680.0049680.004274

1.4e-050.0002291.5e-05

1.4e-050.0002291.5e-05

1.4e-050.0002291.5e-05

2.3e-05

2.3e-05

4e-062.2e-054e-06

4e-062.2e-054e-06

3e-066.8e-053e-06

3.5e-05

3e-063.3e-053e-06

3e-063.4e-054e-06

3e-063.4e-054e-06

4e-068.2e-054e-06

4e-062.4e-054e-06

1.9e-05

2e-05

1.9e-05

4.6e-052.9e-050.001520.0001814.4e-051.4e-05

4.6e-052.9e-050.001520.0001814.4e-051.4e-05

4.6e-052.9e-050.001520.0001814.4e-051.4e-05

1.9e-05

1.9e-05

5.4e-05

2.7e-05

2.7e-05

2e-062.2e-052e-06

2e-062.2e-052e-06

2.2e-05

2.2e-05

4.4e-052.9e-050.001526.4e-054.2e-051.4e-05

4.4e-052.9e-050.001526.4e-054.2e-051.4e-05

5e-063.7e-054e-06

5e-063.7e-054e-06

2e-061.8e-051e-06

2e-061.8e-051e-06

2e-061.8e-051e-06

3e-061.9e-053e-06

3e-061.9e-053e-06

3e-061.9e-053e-06

1.9e-053.9e-052e-05

1.9e-053.9e-052e-05

1.9e-053.9e-052e-05

7e-061e-056e-06

7e-061e-056e-06

1.2e-052.9e-051.4e-05

1.2e-052.9e-051.4e-05

0.0037960.0015940.0015330.0117560.012030.0038730.004175

0.0037960.0015940.0015330.0117560.0119390.0038730.004175

0.0003580.0001290.003470.000367

0.0002920.0001290.0031920.00031

0.000148

0.0002920.0001290.0030440.00031

6.6e-050.0002785.7e-05

6.6e-059.8e-055.7e-05

0.00018

2.2e-050.0001142.3e-05

2.2e-050.0001142.3e-05

2.2e-050.0001142.3e-05

4.4e-050.0002464.2e-05

4.4e-050.0001114.2e-05

3.1e-059.4e-053.1e-05

1.3e-051.7e-051.1e-05

0.000135

0.000135

0.0006390.0001220.0002710.0017230.0037450.0006850.00062

0.0006390.0001220.0002710.0017230.0037450.0006850.00062

7.1e-050.0008097.8e-05

5.6e-050.0005315.2e-05

0.000418

0.000838

5.5e-050.0001876.5e-05

0.00010.0002560.000122

0.000280.0001220.0002710.0017230.0003520.0002860.000202

0.000169

7.7e-050.0006038.2e-05

0.000241

0.000241

0.000241

0.0006450.0004070.000640.0018240.0003960.000620.000501

0.000109

0.000109

0.0006450.0004070.000640.0018240.0002870.000620.000501

3.6e-050.0002873.4e-05

0.0006090.0004070.000640.0018240.0005860.000501

1.5e-059.4e-051.5e-05

1.5e-059.4e-051.5e-05

1.5e-059.4e-051.5e-05

0.0020570.0009360.0006220.0082090.0034290.0021010.003054

0.0020570.0009360.0006220.0082090.0034290.0021010.003054

0.0001195e-06

8.9e-050.0004319.7e-05

0.00274

4e-06

0.000214

0.0002074.9e-050.0007730.000221

0.0001756.3e-050.0001750.000179

0.0001261.5e-05

0.0001078.5e-050.005270.0001060.000107

0.0014790.0006870.0006220.0029390.0009780.0014970.000163

5e-06

0.000507

5.2e-05

0.000122

1.6e-050.0002042e-05

0.000138

0.000138

1.6e-056.6e-052e-05

1.6e-056.6e-052e-05

9.1e-05

2.4e-05

2.4e-05

2.4e-05

6.7e-05

6.7e-05

6.7e-05

6.4e-050.0006096.3e-05

6.4e-050.0006096.3e-05

1.1e-052.8e-057e-06

8e-061.5e-055e-06

8e-061.5e-055e-06

3e-061.3e-052e-06

3e-061.3e-052e-06

2.8e-050.0001443.1e-05

6e-063.4e-057e-06

6e-063.4e-057e-06

2e-062.5e-053e-06

2e-062.5e-053e-06

1.8e-05

1.8e-05

6e-061.5e-056e-06

6e-061.5e-056e-06

5e-062.5e-056e-06

5e-062.5e-056e-06

3e-062.7e-054e-06

3e-062.7e-054e-06

6e-065e-06

6e-065e-06

4e-069.9e-054e-06

4e-05

4e-05

3.1e-05

3.1e-05

4e-062.8e-054e-06

4e-062.8e-054e-06

7e-062.1e-058e-06

7e-062.1e-058e-06

7e-062.1e-058e-06

0.000166

3.8e-05

1.4e-05

1.4e-05

1e-05

9.2e-05

3e-06

1.1e-05

1.2e-05

2.5e-05

1.2e-05

2.4e-05

5e-06

3.6e-05

1.6e-05

2e-05

1.4e-050.0001061.3e-05

8e-062.3e-057e-06

8e-062.3e-057e-06

4e-063.7e-054e-06

1.7e-05

4e-062e-054e-06

2e-062.7e-052e-06

2e-062.7e-052e-06

1.9e-05

1.9e-05

4.5e-05

4.5e-05

4.5e-05

0.0008890.0071430.0009498.5e-05

0.0008890.0071430.0009498.5e-05

6e-062.1e-056e-06

6e-062.1e-056e-06

6e-062.1e-056e-06

0.0008040.0070310.0008618.5e-05

1.3e-053.9e-051.3e-05

1.3e-053.9e-051.3e-05

1.2e-052.2e-051.1e-05

1.2e-052.2e-051.1e-05

2e-050.0001522.3e-05

7.7e-05

2e-057.5e-052.3e-05

5.7e-050.0004466.4e-057e-06

5.7e-050.0004466.4e-057e-06

7e-06

7e-06

0.000194

0.000194

0.0001350.0001790.000144

3.1e-051.6e-053.1e-05

2.9e-051.2e-053.2e-05

2e-054.7e-052.1e-05

3.5e-056.4e-053.9e-05

2e-054e-052.1e-05

0.0003441.4e-05

4.4e-05

4.7e-05

8.3e-05

0.00017

1.4e-05

7.6e-050.0001518.7e-05

7.6e-050.0001518.7e-05

1.8e-057.2e-051.7e-05

1.8e-057.2e-051.7e-05

0.000254

0.000254

5.4e-059.6e-055.6e-051.3e-05

1e-05

3.1e-05

1.6e-054.5e-051.7e-05

3.8e-051e-053.9e-051.3e-05

3e-05

3e-05

8e-060.0001128e-06

8e-060.0001128e-06

3e-050.000133e-05

1.6e-05

4.4e-05

3e-057e-053e-05

6e-064.9e-054e-06

6e-064.9e-054e-06

1.2e-053.6e-051.2e-05

1.2e-053.6e-051.2e-05

4.7e-050.0002435.1e-05

4.7e-050.0002435.1e-05

0.000115

0.000115

0.000267

0.000122

0.000145

2.8e-050.000182.8e-05

2.8e-050.000182.8e-05

2.1e-058.4e-052.3e-05

2.1e-058.4e-052.3e-05

1e-05

3e-06

4e-06

3e-06

5e-064e-065e-06

5e-064e-065e-06

0.000273

0.000273

1.6e-05

1.4e-05

2e-06

0.000131

0.000131

0.0001280.0003380.000136

0.0001280.0003380.000136

8e-050.0001458.9e-05

3.1e-05

4.5e-057.2e-055.1e-05

3.5e-054.2e-053.8e-05

1.7e-050.0001322e-05

7.2e-05

1.7e-056e-052e-05

1.4e-05

1.4e-05

3.7e-050.0002314e-05

3.7e-050.0002314e-05

9.1e-05

9.1e-05

0.002495

0.002495

6e-064.2e-056e-06

6e-064.2e-056e-06

6e-064.2e-056e-06

1.1e-05

1.1e-05

1.1e-05

7.3e-053.8e-057.6e-05

7.3e-053.8e-057.6e-05

6e-062e-066e-06

6e-062e-069e-06

2.2e-054e-062e-05

1.2e-055e-061.2e-05

1e-055e-061e-05

1e-056e-069e-06

5e-061.2e-056e-06

2e-062e-064e-06

0.3562670.3935940.3223970.3938930.2683750.3494820.364366

0.0011850.0017560.0007530.0021280.0005720.0011310.001542

5e-063.1e-055e-06

5e-063.1e-055e-06

5e-063.1e-055e-06

5e-063.1e-055e-06

0.0011670.0017560.0007530.0021280.0005210.0011150.001542

1.4e-050.0001261.4e-05

1e-061.4e-051e-06

1e-061.4e-051e-06

5e-068e-057e-06

04e-061e-06

1.3e-05

00

6e-06

1e-061.7e-051e-06

000

1.6e-05

2e-066e-062e-06

01e-060

08e-061e-06

2e-069e-062e-06

7e-062.2e-055e-06

5e-066e-062e-06

2e-061.6e-053e-06

1e-061e-051e-06

1e-061e-051e-06

1.5e-051.3e-051.2e-05

1.5e-051.3e-051.2e-05

1.5e-051.3e-051.2e-05

0.0011380.0017560.0007530.0021280.0003820.0010890.001542

0.0011230.0017560.0007530.0021280.0003070.0010770.001542

4e-06

5e-06

6e-063e-068e-06

4e-06

3e-061.1e-05

1.1e-05

1e-065e-061e-06

1e-052e-061e-05

0.0010830.0017270.0007530.0021280.0002030.0010430.001526

1e-06

2e-05

8e-062.9e-052e-061.5e-051.6e-05

2e-05

2e-06

6e-06

1.2e-058e-06

1.2e-053.8e-051e-05

5e-062.1e-053e-06

3e-061.3e-053e-06

4e-064e-064e-06

3e-063.7e-052e-06

4e-06

1e-067e-061e-06

4e-06

2e-061.3e-051e-06

6e-06

3e-06

1.3e-052e-051.1e-05

1.3e-052e-051.1e-05

6e-061.1e-055e-06

6e-061.1e-055e-06

7e-069e-066e-06

4e-06

7e-065e-066e-06

4.5e-055.6e-054.3e-05

4.5e-055.6e-054.3e-05

4.5e-055.6e-054.3e-05

4.5e-055.6e-054.3e-05

1.9e-052.4e-051.6e-05

1.1e-051.9e-051e-05

1.5e-051.3e-051.7e-05

0.2046770.2784230.1721490.28080.1579730.2017980.239395

0.0395440.0324360.0362140.0324270.0357640.0379050.023349

0.038640.0320790.0362140.0282730.0352160.0371180.022468

0.0384040.0319260.0308030.0232060.0329350.0368720.02001

1e-065e-061e-06

8e-06

0.0383930.0319260.0308030.0232060.0328560.0368620.02001

4e-064e-063e-06

6e-061.8e-056e-06

1.6e-05

8e-06

2e-05

9e-069.4e-058e-06

5.5e-05

2.8e-05

9e-061.1e-058e-06

0.0002270.0001530.0054110.0050670.0021870.0002380.002458

0.00012

0.000224

3.9e-054.1e-052.3e-054.3e-05

0.000364

0.000791

0.0003675.4e-05

0.002254

8.2e-05

1.9e-055.9e-050.0054110.0050674e-061.6e-051.6e-05

1.4e-05

3.9e-051e-054.2e-05

0.000187

0.000135.3e-050.0001350.000137

0.0009040.0003570.0041540.0005480.0007870.000881

4.3e-056.5e-050.0016214.5e-054e-055.5e-05

4.3e-056.5e-050.0016212.6e-054e-055.5e-05

1.9e-05

0.0008610.0002920.0025330.0004950.0007470.000826

1.1e-05

4e-056e-063.7e-05

3.6e-0503.5e-05

2e-06

5.3e-052e-055.1e-052.7e-05

1e-05

3e-06

5.2e-051.5e-055.2e-05

5e-067e-065e-06

8e-06

7e-06

2.5e-053e-062.1e-05

4.3e-059e-064.3e-05

1.3e-05

9e-06

7.1e-052.5e-051.6e-057e-055.6e-05

5e-06

9e-06

9.4e-050.0002080.0025331.8e-058.5e-050.000642

1.5e-05

6e-065e-061e-066e-06

1e-06

7e-06

8.3e-051.9e-056.8e-05

1.5e-05

3.8e-051.1e-055e-063.6e-058.3e-05

6e-06

6e-06

1.9e-05

8e-06

8e-06

2.2e-05

3e-06

1.4e-05

2e-06

4e-06

6e-06

5.6e-05

2.6e-053e-062.4e-05

2.1e-054e-061.8e-05

2e-06

1.4e-05

5.7e-054.3e-058e-065.6e-05

3.2e-05

8e-06

3.9e-058e-063.7e-05

5e-06

1e-06

3.4e-054e-063.4e-05

6e-06

8.2e-051.4e-056.9e-051.8e-05

1.8e-05

2e-05

1e-05

6e-06

8e-06

8e-06

8.3e-050.0054180.0053437.9e-05

8.3e-050.0054180.0053437.9e-05

3.9e-05

3.9e-05

3.5e-05

3.5e-05

5.6e-050.0001194.5e-05

1.1e-051.9e-056e-06

2.2e-051e-052e-05

2.3e-05

2.3e-052.4e-051.9e-05

4.3e-05

0.0054180.005113

3.9e-05

0.0054180.005074

2.7e-051.1e-053.4e-05

2.7e-051.1e-053.4e-05

2.6e-05

2.6e-05

0.0205260.0181350.007650.0200640.0026560.0199550.013364

0.0205260.0181350.007650.0200640.0026560.0199550.013364

2.9e-056.6e-051.7e-051.4e-05

2.9e-056.6e-051.7e-051.4e-05

0.0203410.0181350.007650.0200640.0025610.0198240.013337

0.0017590.0005080.0006860.0054720.0002090.0017040.000798

0.0161580.0169480.0063910.009120.0017240.0157490.007792

0.0024240.0006790.0005730.0054720.0001250.0023710.001401

9.4e-05

0.003346

0.000409

0.0001562.9e-050.0001141.3e-05

0.0001562.9e-050.0001141.3e-05

0.0007450.0012260.0005970.0021280.0006550.0007010.001214

8.9e-050.0001467.7e-05

3.2e-05

3.2e-05

5.9e-053.4e-055.7e-05

5.9e-053.4e-055.7e-05

5.8e-05

1.5e-05

1.8e-05

2.5e-05

3e-052.2e-052e-05

3e-052.2e-052e-05

0.0006560.0012260.0005970.0021280.0005090.0006240.001214

2.9e-054.4e-052.8e-054.2e-05

1.7e-05

2.1e-05

0

0

2.9e-056e-062.8e-054.2e-05

0.000124.2e-050.000101

6.3e-052.3e-055.6e-05

5.7e-051.9e-054.5e-05

7.3e-050.000137.8e-051.3e-05

5e-061e-065e-06

6e-062e-069e-06

5e-063e-066e-06

8e-062e-067e-06

1.2e-056e-061.4e-051.3e-05

3e-06

3.7e-059e-063.7e-05

1e-05

000

000

9.4e-05

3.6e-05

1.1e-05

1.4e-05

1.1e-05

0.0004340.0012260.0005970.0021280.0002570.0004170.001159

0.0004340.0012260.0005970.0021280.0002570.0004170.001159

4.3e-05

4.3e-05

2.1e-05

2.1e-05

2.2e-05

2.2e-05

0.0066390.0047470.0155220.0156050.0052950.0063390.003405

4e-061e-053e-06

4e-061e-053e-06

4e-061e-053e-06

6e-062e-054e-06

6e-062e-054e-06

9e-06

6e-061.1e-054e-06

3.7e-051.7e-052.9e-05

3.7e-051.7e-052.9e-05

3.7e-051.7e-052.9e-05

1.8e-057.4e-051.4e-05

1.8e-057.4e-051.4e-05

9e-061e-067e-06

4e-062.1e-053e-06

4e-06

5e-061e-064e-06

1e-06

1.1e-05

4e-06

1e-05

1.1e-05

1e-05

0.0064140.0047470.0051340.0058770.0049680.0061520.003388

3e-061.8e-053e-06

3e-061.8e-053e-06

2e-05

2e-05

0.0058170.0041690.0045660.0039520.0044230.0055840.00301

4e-061.7e-052e-06

0.0058130.0041690.0045660.0039520.0044060.0055820.00301

8e-061.4e-057e-06

3e-063e-062e-06

1e-06

1e-062e-061e-06

0

0

5e-06

4e-063e-064e-06

2.3e-05

2.3e-05

0.0005860.0005780.0005680.0019250.000470.0005580.000378

0.0005310.0005780.0005680.0019250.0003930.0005090.000378

2.9e-055e-062.7e-05

2.9e-05

9e-06

1.2e-05

2.6e-058e-062.2e-05

7e-06

7e-06

0.0001510.0103880.0097280.000140.0001261.7e-05

0.0001510.0103880.0097280.000140.0001261.7e-05

2e-051.6e-051.1e-05

3e-061.4e-053e-06

4e-060.0103880.0097284e-063e-061e-06

6e-067e-064e-06

1e-0601e-06

2e-051.3e-051.8e-05

5e-061e-055e-06

3.5e-052.4e-053.4e-05

4e-065e-063e-06

1e-062e-061e-061e-06

8e-069e-068e-06

7e-065e-067e-06

7e-066e-06

5e-061.9e-053e-06

2e-061e-062e-061e-06

1e-062e-062e-06

2.2e-059e-061.5e-051.4e-05

9e-063.8e-051.1e-05

9e-063.8e-051.1e-05

4e-062.2e-055e-06

5e-061.6e-056e-06

2.8e-05

2.8e-05

2.8e-05

0.0311140.1556290.0257660.1198820.0065930.0280080.167029

1e-062.1e-050

1e-061.4e-050

1.1e-05

1e-063e-060

7e-06

7e-06

0.0311130.1556290.0257660.1198820.0065720.0280080.167029

6e-05

6e-05

0.0311130.1556290.0257660.1198820.0065120.0280080.167029

3.4e-05

0.000126

0.0311080.1556290.0257660.1198820.0062480.0280.167029

5e-066.5e-058e-06

3.9e-05

7e-062e-057e-06

7e-062e-057e-06

7e-062e-057e-06

7e-062e-057e-06

0.0004580.0134860.0132810.0004062.7e-05

0.0002140.0004250.000163

3.8e-058e-062e-06

3.8e-058e-062e-06

1.3e-05

1.3e-05

4.7e-05

4.7e-05

9.8e-050.000319.1e-05

3.6e-05

0.000158

2.9e-05

9.8e-054.8e-059.1e-05

3.9e-05

8e-063e-065e-06

8e-063e-065e-06

7e-054.4e-056.5e-05

7e-054.4e-056.5e-05

2.9e-050.0001132.3e-05

2.4e-05

2.4e-05

2.3e-055.2e-051.8e-05

5e-062.5e-054e-06

8e-061.3e-057e-06

1e-051.4e-057e-06

6e-061.3e-055e-06

6e-061.3e-055e-06

2.4e-05

2.4e-05

1.2e-053.4e-052.4e-05

1.2e-053.4e-052.4e-05

8e-06

1.2e-051.3e-052.4e-05

1.3e-05

0.000190.0134860.0126630.0001832.7e-05

0.000190.0134860.0126630.0001832.7e-05

2.7e-05

1e-06

0.0001463.1e-050.000142

0.0134860.012629

4.4e-052e-064.1e-05

2.6e-05

2.6e-05

2.6e-05

1.3e-052e-051.3e-05

1.3e-052e-051.3e-05

1.3e-052e-051.3e-05

0.0169530.0146070.0081390.0479310.007340.0227110.025478

0.0006310.0007990.0003060.0006060.001806

5.4e-055e-054.1e-05

5.4e-059e-064.1e-05

3.3e-05

8e-06

0.000390.0007990.0001860.0003630.001806

0.0001599.9e-050.0001411.4e-05

5.1e-05

0.0002310.0007993.6e-050.0002220.001792

0.0001877e-050.000202

6.1e-057e-065.3e-05

9e-061e-069e-06

4.4e-051.1e-054.2e-05

1e-05

7.3e-054.1e-059.8e-05

0.0008180.0001340.0002670.0016210.0006130.0007260.000565

5e-061.3e-054e-061.4e-05

5e-061.3e-054e-061.4e-05

0.000520.0001340.0002670.0016210.0004420.000460.000153

0.0003510.0001340.0002670.0016210.0002930.0003250.000153

3.2e-05

1.2e-051.2e-058e-06

0.0001266e-060.000102

5.3e-05

3.1e-051.1e-052.5e-05

2.6e-05

9e-06

0.0002880.000150.0002570.000398

0.0001346.2e-050.000124

1.9e-054e-061.8e-051.3e-05

3.5e-0503.2e-053.1e-05

9.3e-052.7e-057.8e-050.000354

7e-065e-065e-06

5.2e-05

5e-068e-065e-06

5e-068e-065e-06

4e-05

4e-05

4e-05

0.0001850.0001530.00014

4.2e-056.3e-053.3e-05

1.1e-05

1.5e-05

2.5e-051e-052.1e-05

1.4e-05

1.7e-051.3e-051.2e-05

2e-051.2e-051.6e-05

2e-051.2e-051.6e-05

7.3e-051.8e-055.5e-05

7.3e-051.8e-055.5e-05

3.8e-053.5e-052.8e-05

1.6e-051.3e-051.1e-05

1e-05

2.2e-051.2e-051.7e-05

2.4e-05

2.4e-05

1.2e-051e-068e-06

1.2e-051e-068e-06

0.000174.4e-050.0001040.000159

9e-06

9e-06

1.5e-05

1.5e-05

0.000174.4e-058e-050.000159

8e-06

1.1e-051e-051e-05

1.6e-054e-061.6e-05

5e-06

0.0001434.4e-055.3e-050.000133

0.0099860.0057330.005950.0344540.004490.0159650.016092

0.0003186.3e-059.2e-050.0003139e-05

1.6e-05

2.8e-05

0

2.4e-05

0.0001841.4e-050.000179

6e-06

0.0001346.3e-054e-060.0001349e-05

1.8e-05

1.8e-05

2.2e-05

2.2e-05

0.0008390.0001250.0001820.0007974.1e-05

1e-051e-051e-05

0.0002222.4e-050.0002094.1e-05

7e-061.4e-055e-06

0.00060.0001250.0001340.000573

8e-062.5e-057e-06

8e-062.5e-057e-06

1.7e-051.3e-051.8e-05

1.7e-051.3e-051.8e-05

0.0003366.6e-053.4e-050.000326

0.0003366.6e-053.4e-050.000326

5.9e-05

5.9e-05

0.0004480.0003050.0132757e-050.0004040.000239

3.8e-050.0001310.0132754e-064.5e-053.5e-05

3e-06

2.5e-052e-062.5e-056.4e-05

0.0001768e-051.2e-050.0001773.8e-05

3.6e-05

5e-06

0.0002099.4e-058e-060.0001570.000102

6e-065.7e-058e-06

01e-061e-06

02e-060

3e-069e-062e-06

3e-068e-065e-06

2.4e-05

1.3e-05

0.000280.000180.000271

0.000280.000180.000271

9e-06

9e-06

0.0004310.0009520.0005210.0035470.000250.0067390.003589

0.0001630.000880.0005210.0035470.0002320.0064710.003574

0.0002687.2e-051.8e-050.0002681.5e-05

2.3e-05

2.3e-05

6.7e-05

6.7e-05

2.3e-05

2.3e-05

1.4e-05

1.4e-05

0.0004080.0010380.0039526.3e-050.0003990.00368

2e-050.000376

0.0002964.2e-053.3e-050.0002910.000104

0.0001120.0009960.0039521e-050.0001080.0032

0.0035540.0016850.0012040.0020270.0007050.003450.00051

0.0003210.0001831.1e-050.00030.000251

0.0004340.0001047.7e-050.0004294.5e-05

0.0004310.000112.2e-050.0004296.3e-05

7e-068.2e-05

1.7e-05

2e-06

5e-06

4.3e-05

0.0018420.0011570.0012040.0020270.0005080.001768

0.0005260.0001311e-050.0005245.2e-05

4e-06

3e-06

6e-06

2e-06

3e-06

2e-06

0.0001

0.0001

7e-066e-06

7e-066e-06

3.4e-05

3.4e-05

6.5e-05

6.5e-05

7.5e-05

5.7e-05

1.8e-05

0.0031290.0010850.0042250.0022670.0030660.006937

0.000814

0.0001

0.0042250.003956

0.002584

0.001113

0.0021950.0008870.0001560.0021560.000357

0.0009340.0001988.4e-050.000914e-05

0.0002050.0004140.0116534.3e-050.0001610.001006

4.4e-050.0001060.0027363e-064.4e-05

3e-052.2e-050.0023315e-063e-05

8.7e-052.4e-050.0028372.3e-054.3e-050.00077

4.4e-050.0002620.0037491.2e-054.4e-050.000236

0.0051630.0078970.0019220.0118560.0015880.0051150.007015

0.0047050.0078620.0019220.0118560.001440.0047070.006925

0.000354

4.4e-05

0.000227

2e-06

0.000142

0.000216

4.4e-05

0.0046910.0078180.0019220.0057760.0004070.0046930.006824

1e-06

1.3e-050.003043e-061.3e-050.000101

1e-064.4e-050.0030401e-06

8.1e-052.3e-057e-067.9e-054.6e-05

4e-065e-066e-064.6e-05

7.7e-052.3e-052e-067.3e-05

5.8e-05

5.8e-05

0.0003771.2e-058.3e-050.0003294.4e-05

7e-06

9.8e-059e-068.5e-05

1e-06

2.8e-05

3e-06

2.5e-051.2e-055e-062.4e-051.6e-05

9e-051.5e-058.2e-05

0.0001646e-060.000138

3.3e-05

4e-06

4.6e-05

4.6e-05

4.6e-05

0.0116330.0052790.0059910.0114510.0144730.0113130.00257

3.7e-05

3.7e-05

3.7e-05

3.7e-05

3.7e-05

3.7e-05

0.0114420.0052790.0059910.0114510.0141750.0111510.00257

5.3e-05

5.3e-05

9.1e-05

9.1e-05

8.9e-050.0001058.7e-05

8.9e-050.0001058.7e-05

0.01120.0052790.0059910.0114510.0137310.0109220.00257

0.000112

0.0004170.0001380.0063849.1e-050.000412

0.0105820.0051410.0059910.0050670.0101070.010510.00257

0.0002010.000105

0.003316

8.5e-050.0001227.6e-05

4e-058.9e-053.7e-05

4.5e-053.3e-053.9e-05

6.8e-057.3e-056.6e-05

6.8e-057.3e-056.6e-05

1.6e-054.9e-051e-05

1.6e-054.9e-051e-05

1.6e-051.9e-051e-05

3e-05

0.0001750.0001750.000152

5.3e-053.3e-054.7e-05

5.3e-053.3e-054.7e-05

7.3e-052.8e-055.8e-05

7.3e-052.8e-055.8e-05

2.9e-05

2.9e-05

3.6e-052.7e-053.6e-05

3.6e-052.7e-053.6e-05

1.3e-055.8e-051.1e-05

5e-061.2e-054e-06

3e-062.8e-052e-06

5e-061.8e-055e-06

5.7e-059.1e-054.8e-05

5.7e-059.1e-054.8e-05

3e-061.7e-054e-06

3e-061.7e-054e-06

8e-064.7e-057e-06

8e-061.5e-057e-06

9e-06

2.3e-05

4.6e-052.7e-053.7e-05

4.6e-052.7e-053.7e-05

0.0765310.0451060.0528880.0271580.0658070.073890.00127

0.0765310.0451060.0528880.0271580.0658070.073890.00127

4.1e-05

4.1e-05

7e-064.3e-055e-068.7e-05

1.3e-05

2.4e-05

7e-061.9e-055e-06

4.2e-05

3.2e-05

0.0765130.0451060.0528880.0271580.0656550.0738720.001183

8e-062.8e-05

1.5e-056.6e-05

3.9e-05

3.1e-05

9e-063e-06

2.7e-05

6e-061.1e-058e-066.9e-05

2.5e-05

7e-06

0.0764030.0451060.0528880.0271580.065290.073769

1e-06

4.5e-050.000156

1e-06

1.9e-051.4e-051.7e-053.6e-05

1.9e-059e-051.6e-05

8e-069e-067e-06

1.1e-054e-068e-06

0.000214

2e-055.1e-05

1.1e-059e-061.1e-050.000141

3.5e-05

4.1e-05

2.1e-05

1.4e-056e-061.5e-05

4e-063e-064e-066e-06

1e-052e-069e-066.5e-05

8.1e-05

8e-061.4e-058e-060.000145

1.1e-056.8e-051.3e-05

8e-062.1e-056e-06

1.9e-05

3e-062.8e-057e-06

2.5e-05

2.5e-05

2.5e-05

2.5e-05

5.4e-050.0001564.1e-050.000344

3.8e-05

3.8e-05

1.7e-05

2.1e-05

5.4e-057.7e-054.1e-05

1.7e-052.2e-051.3e-05

1.7e-052.2e-051.3e-05

2e-051.5e-051.5e-05

2e-051.5e-051.5e-05

8e-061.3e-057e-06

8e-061.3e-057e-06

9e-062.7e-056e-06

9e-062.7e-056e-06

0.000344

0.000344

0.000344

4.1e-05

2.5e-05

2.5e-05

1.6e-05

1.6e-05

4.5e-05

4.5e-05

4.5e-05

4.5e-05

0.0001153.4e-050.0016210.000149.7e-056.2e-05

0.0001153.4e-050.0016210.000149.7e-056.2e-05

2.5e-052.7e-052.2e-05

4e-06

8e-061e-066e-06

1.3e-052e-051.2e-05

4e-062e-064e-06

7e-062e-054e-06

7e-062e-054e-06

2.4e-059e-062.1e-051.3e-05

8e-064e-066e-06

1.6e-055e-061.5e-051.3e-05

3e-066e-063e-06

3e-066e-063e-06

6e-062.3e-054e-068e-06

6e-062.3e-054e-068e-06

3e-067e-065e-06

3e-067e-065e-06

8e-069e-065e-06

8e-069e-065e-06

1e-068e-061e-06

1e-061e-061e-06

6e-06

1e-06

3.8e-053.4e-050.0016213.1e-053.2e-054.1e-05

1.1e-051.9e-051.5e-05

1e-053.4e-050.0016213e-068e-06

6e-061e-065e-06

1.1e-058e-064e-06

4.1e-05

0.0002180.0012240.0004780.0025330.0002460.0002980.001283

4.3e-05

2.3e-05

2.3e-05

2e-05

2e-05

0.0001940.0012240.0004780.0025330.0001080.0002780.001283

6e-06

6e-06

0.0001940.0012240.0004780.0025330.0001020.0002780.001283

3e-06

0.0001830.0012240.0004780.0025338.9e-050.0002690.001283

1e-063e-061e-06

4e-06

2e-0601e-06

8e-063e-067e-06

2.4e-059.5e-052e-05

8e-062.3e-056e-06

8e-061.3e-056e-06

1e-05

3e-06

3e-06

8e-064.4e-053e-06

5e-061.9e-05

3e-062.5e-053e-06

1e-067e-062e-06

06e-060

1e-061e-062e-06

7e-061.8e-059e-06

7e-061.8e-059e-06

0.0005120.0013750.0004482e-05

2.4e-050.0001081.9e-05

1.7e-058.8e-051.4e-05

9e-062.7e-058e-06

9e-062.7e-058e-06

6e-062.3e-055e-06

6e-062.3e-055e-06

2e-063.8e-051e-06

2e-063.8e-051e-06

7e-062e-055e-06

7e-062e-055e-06

7e-062e-055e-06

2.8e-052.9e-052.1e-05

2.8e-052.9e-052.1e-05

2.8e-052.9e-052.1e-05

2.8e-052.9e-052.1e-05

2.3e-050.0001811.6e-05

1.2e-050.0001581.1e-05

1.2e-050.0001581.1e-05

1.2e-050.0001581.1e-05

1.1e-052.3e-055e-06

1.1e-052.3e-055e-06

2e-068e-062e-06

9e-061.5e-053e-06

7e-062.5e-054e-06

7e-062.5e-054e-06

7e-062.5e-054e-06

7e-062.5e-054e-06

5.9e-050.0001936.4e-05

4.3e-050.0001124e-05

2e-055.8e-052e-05

2e-055.8e-052e-05

3e-061.7e-052e-06

3e-061.7e-052e-06

1.9e-052.3e-051.7e-05

1.9e-052.3e-051.7e-05

1e-061.4e-051e-06

1e-061.4e-051e-06

1.6e-058.1e-052.4e-05

9e-062.5e-059e-06

9e-062.5e-059e-06

2e-062.3e-052e-06

2e-062.3e-052e-06

2e-061.8e-051e-05

2e-061.8e-051e-05

3e-061.5e-053e-06

3e-061.5e-053e-06

4e-063.9e-052e-06

4e-063.9e-052e-06

1.5e-05

1.5e-05

4e-062.4e-052e-06

4e-062.4e-052e-06

9e-050.000287.5e-057e-06

3.1e-054.5e-052.3e-053e-06

3.1e-054.5e-052.3e-053e-06

5e-068e-063e-06

8e-061.4e-057e-06

1.8e-052.3e-051.3e-053e-06

2.6e-05

2.6e-05

2.6e-05

9e-064.2e-056e-06

2.2e-05

2.2e-05

9e-062e-056e-06

9e-062e-056e-06

8e-064.9e-057e-06

8e-063e-057e-06

8e-063e-057e-06

1.9e-05

1.9e-05

1.3e-052.4e-059e-06

1.3e-052.4e-059e-06

1.3e-052.4e-059e-06

2.3e-05

2.3e-05

2.3e-05

2.9e-057.1e-053e-054e-06

2.1e-055.2e-051.9e-054e-06

7e-061.8e-057e-06

8e-061e-055e-064e-06

1.3e-05

6e-061.1e-057e-06

8e-061.9e-051.1e-05

8e-061.9e-051.1e-05

0.0001510.0002510.0001431.3e-05

0.0001340.0001790.0001261.3e-05

2e-065e-061e-06

2e-065e-061e-06

0.0001320.0001740.000125

2.2e-052.3e-052e-05

1.4e-052.3e-051.5e-05

3.3e-05

1.6e-051.2e-051.3e-05

2e-066e-06

2.4e-052.2e-052e-05

1.1e-051.3e-058e-06

2.4e-052.4e-05

1e-061e-06

1.1e-05

6e-061.6e-056e-06

1.2e-052.1e-051.2e-05

1.3e-05

1.3e-05

4e-062.2e-054e-06

4e-062.2e-054e-06

4e-062.2e-054e-06

1.3e-055e-051.3e-05

1.3e-055e-051.3e-05

2.8e-05

1.3e-052.2e-051.3e-05

0.0001260.0002690.000104

2.8e-059.8e-052.3e-05

5.3e-05

3.3e-05

2e-05

2.8e-054.5e-052.3e-05

1.5e-051.2e-051.1e-05

1.3e-053.3e-051.2e-05

9.8e-050.0001718.1e-05

2.8e-05

2.8e-05

9.8e-050.0001438.1e-05

2.9e-05

4e-065e-063e-06

3e-06

1e-051e-058e-06

2.3e-058e-061.9e-05

1.7e-051.3e-051.3e-05

2.3e-059e-062e-05

9e-068e-068e-06

7e-062.7e-056e-06

5e-063.1e-054e-06

0.0410560.0341190.0274630.0329350.0260780.0396260.029921

0.0002150.0003880.000208

4.4e-053e-053e-05

4.4e-053e-053e-05

4.4e-053e-053e-05

4.7e-050.0001086e-05

8e-063e-059e-06

5e-061.3e-055e-06

3e-061.7e-054e-06

2.1e-051.4e-051.7e-05

8e-061.1e-056e-06

1.3e-053e-061.1e-05

3.2e-05

1.6e-05

1.6e-05

1.8e-051.6e-053.4e-05

1.8e-051.6e-053.4e-05

1.6e-05

1.6e-05

5.2e-05

2.3e-05

2.3e-05

2.9e-05

2.9e-05

8.4e-057.4e-056.6e-05

2.8e-052e-052.2e-05

2.8e-052e-052.2e-05

3.7e-052.7e-052.9e-05

3.7e-052.7e-052.9e-05

1.9e-052.7e-051.5e-05

1.9e-052.7e-051.5e-05

4e-050.0001245.2e-05

3.2e-058.7e-054.6e-05

8e-061.3e-059e-06

1.5e-05

4e-061e-054e-06

1.3e-051e-051e-05

1.7e-05

7e-062.2e-052.3e-05

8e-063.7e-056e-06

1.7e-05

8e-062e-056e-06

0.0011080.0038870.0013540.0074990.0005810.0010710.005827

0.0003790.000180.0016210.0002840.000355.5e-05

0.0002920.000180.0016210.0002390.0002765.5e-05

0.0002920.000180.0016210.0002390.0002765.5e-05

4e-052.2e-053.3e-05

4e-052.2e-053.3e-05

4.7e-052.3e-054.1e-05

4.7e-052.3e-054.1e-05

0.0007290.0037070.0013540.0058780.0002970.0007210.005772

1.8e-05

1.8e-05

0.0007290.0037070.0013540.0058780.0002460.0007210.005772

3.1e-051.2e-052e-052.1e-05

2.5e-05

0.0006120.0037070.0013540.0041556e-050.0006190.005444

4e-06

7.3e-05

7.3e-055.6e-05

4.2e-05

4.7e-05

8.2e-05

9e-06

8.6e-050.0017235.4e-058.2e-051.6e-05

3.3e-05

3.3e-05

0.0272930.020030.0163460.0183420.0137740.0264310.017786

0.0010160.0005390.0007690.0034460.0009730.0009560.000332

4.5e-052.7e-054.1e-05

4.5e-052.7e-054.1e-05

3e-05

3e-05

0.0002220.000220.0002790.0017230.0002190.000210.0003

2.7e-05

0.0002220.000220.0002790.0017230.0001920.000210.0003

2e-051.2e-052.2e-05

1.4e-052e-061.4e-05

6e-064e-068e-06

6e-06

2.7e-052.3e-052.1e-05

2.7e-052.3e-052.1e-05

6e-06

6e-06

2.4e-059e-061.9e-05

2.4e-059e-061.9e-05

0.0005420.0003190.000490.0017230.0004090.0005191e-05

3.2e-051.9e-053.1e-05

9e-065e-068e-067e-06

0.0004710.0003190.000490.0017230.0003510.000451

1.5e-05

1.9e-051.2e-051.7e-05

1.1e-057e-061.2e-053e-06

4.7e-05

2.7e-05

2e-05

9e-062e-057e-06

9e-062e-057e-06

4.7e-053.7e-054.1e-05

4.7e-053.7e-054.1e-05

4.8e-05

2.6e-05

2.2e-05

1.6e-051e-051.7e-051.3e-05

1.6e-051e-051.7e-051.3e-05

6.4e-054.7e-055.9e-053e-06

3e-052.4e-052.7e-053e-06

3.4e-052.3e-053.2e-05

3.5e-05

1.2e-05

2.3e-05

0.0255930.0194120.0155770.0148960.0113240.0248720.015492

2.3e-050.0001171.8e-05

5e-06

9e-06

8e-06

2.3e-054e-061.8e-05

1.1e-05

4.6e-05

1e-05

9e-06

1.5e-05

5.8e-055.7e-055.4e-053e-06

3.4e-052.1e-053.1e-05

1.5e-05

2.4e-059e-062.3e-053e-06

1.2e-05

1e-053.1e-058e-06

4e-069e-063e-06

6e-061.1e-055e-06

1.1e-05

0.0009130.0002340.0007780.0008350.000234

5e-05

0.000196

0.0003048.7e-050.0001620.0002760.000104

0.0001632.6e-057.4e-050.0001461.5e-05

3.8e-053.3e-054.3e-05

0.0002838.7e-050.0001010.0002540.000115

0.0001253.4e-050.0001620.000116

0.0001792.4e-050.0002430.0001664.9e-05

4.9e-052.4e-052.9e-054.6e-052.6e-05

4.9e-054.5e-059e-06

3.4e-052.4e-053.2e-05

3.4e-05

0.000132

4.7e-052.4e-054.3e-051.4e-05

0.024410.0191540.0155770.0148960.0100980.0237910.015206

0.0085220.0045850.0055970.0039520.0053250.0083810.004014

0

7e-06

3.4e-05

6.8e-052.5e-056.9e-056.8e-05

0

9.4e-054.9e-058.9e-05

0.000139

7e-06

4.6e-05

4e-056e-063.9e-056.7e-05

9e-06

1.5e-052e-061.5e-05

7e-06

8.8e-051e-058.4e-05

4e-06

8e-06

1.4e-05

0.0150530.014480.009980.0109440.0039510.014590.010701

2.9e-05

3e-06

1e-061e-0605e-06

3.8e-054e-064.1e-05

4e-051.8e-055e-064.2e-053.1e-05

4.3e-051.2e-054.3e-052.8e-05

2.5e-05

1.2e-05

9.9e-051.4e-059.9e-054e-05

3.6e-052.7e-051.4e-053.7e-053.6e-05

0.000183

5e-06

2.1e-05

1.2e-053e-061.3e-054.6e-05

6e-06

0

3e-061.8e-051e-063e-069e-06

4.6e-052e-064.9e-056.6e-05

6e-06

0.0002122.6e-050.0001090.0001979.5e-05

0.001134

0.001134

0.001134

0.0001550.000320.0001152.7e-05

3e-061.3e-051e-06

2e-068e-061e-06

1e-065e-060

1.9e-05

1.9e-05

7.2e-050.0001465.7e-051.4e-05

1e-0601e-06

2.2e-05

1e-0602e-06

2.2e-05

3.8e-053e-052.9e-05

4e-063e-064e-06

2e-05

2e-06

9e-06

9e-06

2.8e-051.6e-052.1e-051.4e-05

1.3e-05

1.6e-053.2e-051e-05

1.6e-051.1e-051e-05

2.1e-05

4.6e-051.7e-053e-05

4.6e-051.1e-053e-05

6e-06

3e-05

3e-05

1.8e-053.1e-051.7e-051.3e-05

1.8e-053.1e-051.7e-051.3e-05

3.2e-05

3.2e-05

0.0003177.9e-050.0003750.0002991.7e-05

0.0002387.9e-050.0002090.0002321.7e-05

3.4e-05

0.000127

0.0002387.9e-054.8e-050.0002321.7e-05

1.6e-057.3e-051.3e-05

2.1e-05

1.6e-051.9e-051.3e-05

3.3e-05

5e-05

2.1e-05

2.9e-05

4.2e-052.7e-053.4e-05

4.2e-051.1e-053.4e-05

6e-06

1e-05

2.1e-051.6e-052e-05

2.1e-051.6e-052e-05

0.0002120.0007820.0001890.000784

1.9e-05

1.9e-05

0.0005910.000756

0.000545

2e-05

2.6e-05

0.000756

6.9e-054.2e-055.9e-051.4e-05

6.9e-054.2e-055.9e-051.4e-05

1.6e-05

1.6e-05

3.4e-052.9e-053.1e-05

1.6e-052.9e-051.6e-05

1.8e-051.5e-05

2e-05

2e-05

5.7e-053.9e-055.4e-051.4e-05

5.7e-053.9e-055.4e-05

1.4e-05

5.2e-052.6e-054.5e-05

5.2e-052.6e-054.5e-05

0.0002840.0001740.000251.1e-05

0.0001439e-050.00013

9e-054.8e-057.9e-05

5.4e-052e-065.1e-05

6e-06

3.6e-052.3e-052.8e-05

1.7e-05

5.3e-054.2e-055.1e-05

5.3e-052.1e-055.1e-05

2.1e-05

9.6e-055.4e-058.4e-05

6.5e-053e-055.9e-05

6.5e-053e-055.9e-05

3.1e-052.4e-052.5e-05

3.1e-052.4e-052.5e-05

4.5e-053e-053.6e-051.1e-05

4.5e-053e-053.6e-051.1e-05

4.5e-053e-053.6e-051.1e-05

0.0121560.0102020.0097630.0070940.0111610.0116660.006297

0.0121560.0102020.0097630.0070940.0111610.0116660.006297

1.8e-052.5e-051.8e-05

2e-064e-060

8e-068e-068e-06

3e-067e-064e-06

2e-064e-065e-06

3e-062e-061e-06

1.3e-051.1e-051.3e-05

1.3e-051.1e-051.3e-05

1e-053e-067e-06

1e-053e-067e-06

0.0121150.0102020.0097630.0070940.0111220.0116280.006297

0.0121150.0102020.0097630.0070940.0111220.0116280.006297

0.1087920.0792960.1220320.078030.0823210.1064360.093488

0.0379650.0430660.0320240.0312120.0287510.0364620.031832

7e-062.4e-054e-06

7e-062.4e-054e-06

7e-062.4e-054e-06

2.3e-05

2.3e-05

2.3e-05

0.0002870.0022680.0001750.0002850.001241

1e-065e-062e-06

1e-065e-062e-06

0.0002860.0022680.000170.0002830.001241

3e-067e-061e-06

6e-065e-066e-061e-06

4.1e-051.9e-054.1e-053e-06

1e-062e-060

2e-066.9e-051e-062e-068e-06

6e-060.0010831e-057e-062e-06

1.4e-051.5e-051.5e-05

2e-051.3e-052.1e-05

1.4e-055e-061.4e-05

3e-061e-060

5.6e-050.0011162.5e-055.7e-05

1.1e-053e-062e-06

1e-054e-061.1e-059e-06

0.001198

0

8e-069e-068e-062e-06

4e-06

8e-066e-068e-069e-06

8e-06

1.9e-051.1e-052.3e-05

1.8e-055e-062.1e-05

3.1e-051.7e-053.1e-055e-06

1.5e-054e-061.5e-05

0.0376710.0407980.0320240.0312120.0285290.0361730.030591

0.0321110.0242380.0230220.0168220.0211250.0308270.015195

3e-063e-06

2e-062e-061e-06

0.0321060.0242380.0230220.0168220.0207490.0308230.015195

0.00022

1e-06

4.8e-05

07.7e-05

02.8e-050

9e-061.3e-054e-06

6e-061e-061e-06

8e-06

3e-064e-063e-06

0.0055360.016560.0090020.014390.0073790.0053280.015396

0.005530.016560.0090020.014390.0070290.0053220.014105

2e-064e-062e-06

4e-060.0003464e-060.001291

1.5e-051.2e-051.4e-05

3e-061e-062e-06

03e-060

2e-06

00

1.2e-056e-061.2e-05

0.001092.1e-050.0011360.0009775.6e-05

0.0007920.0006980.0007135.6e-05

0.0002060.0001280.0001811.4e-05

9.2e-052.3e-058.3e-051.4e-05

2.6e-05

0.0001147.9e-059.8e-05

0.0001760.0001280.0001631.4e-05

4.6e-054.7e-054.2e-05

0.000138.1e-050.0001211.4e-05

4e-062.6e-052e-06

4e-062.6e-052e-06

7e-05

7e-05

5.1e-05

5.1e-05

0.0002120.0001140.0002012.8e-05

8e-06

0.00013.9e-059.7e-05

3.1e-05

0.0001123.6e-050.0001042.8e-05

6.4e-05

6.4e-05

6.4e-054.5e-054.9e-05

6.4e-054.5e-054.9e-05

0.000137.2e-050.000117

0.000137.2e-050.000117

0.0002982.1e-050.0004380.000264

9.7e-059.6e-058e-05

9.7e-059.6e-058e-05

4.1e-053.2e-054.1e-05

2.3e-052e-052.1e-05

1.8e-051.2e-052e-05

2e-067.2e-052e-06

5.7e-05

2e-063e-062e-06

1.2e-05

5.6e-057.5e-054.4e-05

5.6e-054.3e-054.4e-05

3.2e-05

7.2e-05

7.2e-05

6.6e-052.1e-055.7e-056.3e-05

6.6e-052.1e-055.7e-056.3e-05

3.6e-053.4e-053.4e-05

3.6e-053.4e-053.4e-05

0.0003180.0003040.0002661.3e-05

0.0003180.0003040.0002661.3e-05

0.0002780.0001990.0002341.3e-05

0.0002780.0001990.0002341.3e-05

2.7e-052.9e-052.2e-05

2.7e-052.9e-052.2e-05

1.3e-057.6e-051e-05

1.3e-057.6e-051e-05

0.0004060.0004880.0003760.000109

0.0004060.0004880.0003760.000109

0.0001610.0001260.00017

6.1e-053.4e-055.2e-05

2.9e-05

2.7e-052.6e-055.2e-05

7.3e-053.7e-056.6e-05

5e-054.5e-054.2e-051.5e-05

5e-054.5e-054.2e-051.5e-05

4.5e-05

4.5e-05

8.2e-055.3e-057.1e-05

8.2e-055.3e-057.1e-05

0.0001130.0002199.3e-059.4e-05

3.6e-05

7.8e-05

9.4e-05

5.9e-05

0.0001134.6e-059.3e-05

4.9e-05

2.8e-05

2.8e-05

2.8e-05

2.1e-05

2.1e-05

2.1e-05

0.0062990.0009030.0474610.0112490.0062990.044219

0.0001360.0001590.0001030.000583

5.8e-054.5e-054e-05

5.8e-054.5e-054e-05

7.1e-057.4e-055.7e-05

7.1e-057.4e-055.7e-05

7e-064e-056e-06

7e-064e-056e-06

0.000583

0.000583

0.0061630.0009030.0474610.011090.0061960.043636

0.0005046.2e-050.0004620.000507

0.0005046.2e-050.0004620.000507

0.000805

0.000429

0.000376

0.0001360.0004840.000136

6.4e-050.0001296.2e-05

0.000196

7.2e-050.0001597.4e-05

0.000105

0.000105

0.000315

0.000315

0.000494

0.000494

0.0020050.0005240.0474610.0020830.0020640.043334

0.0006510.0003120.0005780.0008570.000672

0.0013540.0002120.000610.0012260.001392

0.0462730.043334

3.9e-05

3.9e-05

0.000692

0.000692

0.0009790.0001110.0006560.0009821.4e-05

0.00012

1.4e-05

0.0007720.0001110.0004240.000781

0.0002070.0001120.000201

1.3e-05

1.3e-05

0.0001940.0002110.000201

0.0001230.0001120.000133

7.1e-059.9e-056.8e-05

0.000627

0.000627

0.0001633.3e-058.7e-050.000165

0.0001633.3e-058.7e-050.000165

0.000132

0.000132

0.0002810.0001620.000238

0.000131.4e-057.2e-05

0.0001510.0001480.000166

3.2e-051.7e-05

3.2e-051.7e-05

4e-05

4e-05

0.0011270.0001190.0007720.001141

0.0007860.0001190.0004210.0008

0.0003410.0003510.000341

9.5e-051.3e-05

1.3e-05

9.5e-05

0.000403

0.000403

0.0002430.0002980.000246

0.0002430.0002980.000246

0.001571

0.001571

0.0002

0.0002

0.0004995.4e-050.0002080.000499

0.0004995.4e-050.0002080.000499

5.2e-05

5.2e-05

0.000333

0.000333

2.6e-05

2.6e-05

0.0606260.0348140.0425470.0360760.0367630.060010.016972

0.0003350.0002770.0003131.3e-05

0.0003350.0002770.0003131.3e-05

7.4e-055.9e-056.6e-05

8.4e-052.7e-057.8e-051.3e-05

3.7e-058e-063.4e-05

5.8e-052.1e-055.8e-05

1.1e-05

8.2e-055.7e-057.7e-05

6.9e-05

2.5e-05

0.0002430.000154

4.6e-05

4.6e-05

0.000108

0.000108

0.000125

0.000125

0.000118

6.9e-05

4.9e-05

1.5e-059e-051.1e-05

1.5e-059e-051.1e-05

1e-063e-060

3e-062e-061e-06

2e-061.9e-052e-06

1e-061.1e-051e-06

1e-068e-061e-06

1e-061.1e-051e-06

1.5e-05

1e-064e-061e-06

5e-061.7e-054e-06

0.0001330.0002840.000111

9.4e-055.5e-058.3e-05

9.4e-055.5e-058.3e-05

0.000155

0.000155

3.9e-057.4e-052.8e-05

3.9e-057.4e-052.8e-05

4.8e-05

4.8e-05

4.8e-05

0.0003420.0006610.0003051.6e-05

0.000262

0.000164

9.8e-05

0.0001067.4e-059.4e-051.6e-05

0.0001067.4e-059.4e-051.6e-05

2.7e-05

2.7e-05

0.0001590.0001330.0001380

4e-055.2e-053.1e-05

5.5e-054e-054.6e-050

6.4e-054.1e-056.1e-05

6.5e-05

6.5e-05

2.8e-05

2.8e-05

7.7e-057.2e-057.3e-05

7.7e-057.2e-057.3e-05

6.4e-055.8e-055.1e-053.2e-05

6.4e-055.8e-055.1e-05

6.4e-055.8e-055.1e-05

3.2e-05

3.2e-05

0.0066280.0043560.0052190.0073980.0049880.0064120.006765

0.0017680.0016540.0022750.0047630.0014830.0016840.00594

0.0017680.0016540.0022750.0047630.0014830.0016840.00594

0.000258

6e-05

7.1e-05

0.000127

0.0045570.0027020.0029440.0026350.0030170.0044510.000718

0.0001947.4e-053.9e-050.0002020.000429

0.000417

0.0036330.0024280.0029440.0026350.0021680.003503

8.9e-05

5.9e-05

0.0001643.7e-059.1e-050.0001674.3e-05

0.0002258.3e-054.1e-050.0002320.000231

0.000164.1e-055.4e-050.0001691.5e-05

0.0001813.9e-055.9e-050.000178

5.1e-052.6e-054.5e-05

5.1e-052.6e-054.5e-05

2.7e-05

2.7e-05

0.0002520.0002040.0002328e-05

0.0001560.0001160.0001474e-05

9.6e-058.8e-058.5e-054e-05

0.0005440.0002850.000476

0.0001370.0001080.000124

0.0001370.0001080.000124

0.0001487e-050.000137

0.0001487e-050.000137

0.0002590.0001070.000215

0.0002590.0001070.000215

0.0053590.0027320.0020520.0028370.0021740.0050790.00087

0.0044350.0026560.0020520.0028370.0012940.0042585.4e-05

0.000137

0.0002330.0001320.000204

1.1e-05

3e-06

0.00012

1.4e-05

0.0038760.0026020.0020520.0028370.0007470.003716

0.0001812.6e-050.0001020.0002095.4e-05

2e-06

0.0001452.8e-052.6e-050.000129

9.7e-05

9.7e-05

6.2e-05

6.2e-05

2.6e-055.6e-052.6e-05

3e-061.1e-052e-06

8e-06

1.5e-051.1e-051.7e-05

4e-062e-054e-06

4e-066e-063e-06

0.000112

0.000112

0.0003537.6e-050.0002810.00030.000248

0.0002266.4e-050.0001240.0001860.000203

3.2e-051.2e-051.8e-052.8e-054.5e-05

8e-05

9.5e-055.9e-058.6e-05

0.0005450.0002720.0004950.000568

0.0001251.1e-050.0001075.5e-05

0.000169

8.8e-05

0.0001314.3e-050.0001218.7e-05

0.0002015.2e-050.0001820.000257

8.8e-057.8e-058.5e-05

0.0001390.0001370.0001129e-06

0.0001380.0001320.000108

0.0001385.9e-050.000108

7.3e-05

1e-065e-064e-069e-06

0001e-06

0001e-06

0

0000

1e-06

0

0002e-06

0003e-06

0

001e-06

3e-06

1e-061e-061e-060

0

01e-063e-060

0

0.0469830.0277260.0352760.0258410.0274610.0470720.009113

0.0001861.8e-05

0.000186

1.8e-05

0.0001910.0002330.0001522e-06

0.0001910.0002330.0001522e-06

0.000659

0.000659

0.0467920.0277260.0352760.0258410.0263830.046920.009093

0.0078540.0039930.0050230.0069920.002520.0080330.004283

0.007760.0040660.0050390.0070940.0031350.007910.003223

0.0227390.0155420.0129360.006080.0105010.022364

0.0086060.008059

0.0084390.0041250.0036720.0056750.0021680.0086130.001587

8.4e-055.7e-056.8e-05

8.4e-055.7e-056.8e-05

8.4e-055.7e-056.8e-05

0.0020250.0004920.0107420.0033540.0020.000287

0.0018820.0004920.0050670.0030280.0018750.000287

0.0004320.0001120.0050670.0004810.0004076.8e-05

4.7e-05

9.9e-05

5.3e-05

1.4e-05

0.0002636.9e-050.0050678.8e-050.000246

4e-05

0.000122

0.0001694.3e-057.2e-050.0001611.4e-05

0.0001670.0002280.000143

0.000137

7.8e-053.3e-057e-05

2.2e-05

8.9e-053.6e-057.3e-05

5.1e-05

5.1e-05

4.6e-05

4.6e-05

5e-061.9e-051.8e-05

5e-061.9e-051.8e-05

0.0011070.000380.001820.0011420.000219

0.000134

0.000682

0.0004290.0001080.0001140.000449.5e-05

0.0001529.4e-050.000153

0.000293

0.000129

0.00014

0.0005260.0002728e-050.0005490.000124

0.000154

0.0001710.000350.000165

8.5e-05

0.000101

4.7e-05

4.1e-05

0.0001715.3e-050.000165

2.3e-05

3.3e-05

3.3e-05

0.0001430.0056750.0003260.000125

3.9e-05

3.9e-05

0.000174

4.8e-05

2.1e-05

3.4e-05

1.6e-05

2.5e-05

3e-05

0.0001430.0056756.5e-050.000125

2e-05

0.0001430.0056754.5e-050.000125

4.8e-05

2.1e-05

2.7e-05

7e-062.7e-056e-06

7e-062.7e-056e-06

7e-062.7e-056e-06

7e-062.7e-056e-06

4e-050.0001542.8e-05

4e-050.0001542.8e-05

2.8e-059.3e-052e-05

1.3e-057.6e-051.1e-05

1.5e-051.7e-059e-06

1.2e-056.1e-058e-06

5e-063.9e-053e-06

7e-062.2e-055e-06

1.6e-054.6e-051.2e-05

1.6e-054.6e-051.2e-05

1.6e-054.6e-051.2e-05

1.6e-054.6e-051.2e-05

1.7e-059.7e-051.7e-05

1.7e-059.7e-051.7e-05

1.7e-059.7e-051.7e-05

1.7e-059.7e-051.7e-05

1.7e-059.7e-051.7e-05

1.7e-059.7e-051.7e-05

1.7e-050.0002566e-06

4e-069.7e-052e-06

4e-069.7e-052e-06

4e-069.7e-052e-06

4e-069.7e-052e-06

4e-069.7e-052e-06

1.3e-059.9e-054e-06

1.3e-059.9e-054e-06

1.3e-059.9e-054e-06

1.3e-059.9e-054e-06

1.3e-059.9e-054e-06

6e-05

6e-05

6e-05

6e-05

6e-05

9.2e-050.0002096.9e-05

9.2e-050.0002096.9e-05

9.2e-050.0002096.9e-05

9.2e-050.0002096.9e-05

1e-055e-057e-06

1e-061.1e-051e-06

4e-061.6e-053e-06

4e-069e-062e-06

1e-061.4e-051e-06

1.6e-052.8e-051.1e-05

9e-061.6e-055e-06

7e-061.2e-056e-06

2.7e-054.1e-051.9e-05

1.4e-059e-061.2e-05

1.3e-051.5e-057e-06

1.7e-05

1.3e-052.6e-056e-06

1.3e-052.6e-056e-06

2.6e-056.4e-052.6e-05

2.6e-052.1e-052.6e-05

4.3e-05

0.0209410.0446120.0227670.0278680.0254970.0488850.032382

0.0209410.0446120.0227670.0278680.0254970.0488850.032382

0.001630.0011280.0005170.0026350.0007360.0016160.000154

0.001630.0011280.0005170.0026350.0007360.0016160.000154

0.001630.0011280.0005170.0026350.0007360.0016160.000154

2.5e-053.9e-052.4e-05

0.0005430.0004140.00030.0005384.4e-05

0.0005130.0002860.0005170.0026350.0003970.000505

0.0005490.0004280.0005490.00011

0.0161640.0167520.0155250.0097280.0102710.0441610.0086

0.0161640.0167520.0155250.0097280.0102710.0441610.0086

0.0161570.0167520.0155250.0097280.0102530.0441570.0086

4e-061.1e-054e-06

0.0150190.0156680.010970.0072960.0059060.0427720.008073

0.0011340.0010840.000910.0024320.0004530.0013810.000527

0.000469

0.0036450.003414

7e-061.8e-054e-06

7e-061.8e-054e-06

0.0031470.0267320.0067250.0155050.014490.0031080.023628

0.0027480.025520.0062230.0134780.0142650.0027270.022594

2e-063.9e-052e-06

2e-063.9e-052e-06

8e-064.6e-056e-06

2e-062.7e-052e-06

6e-061.9e-054e-06

5.8e-050.0002285.1e-05

2.6e-059.4e-052e-05

1.5e-058e-051.5e-05

1.7e-055.4e-051.6e-05

0.0026770.025520.0062230.0134780.0139260.0026670.022594

2e-061.1e-052e-06

6.3e-050.000815.9e-05

1.9e-053.2e-051.8e-05

2.3e-050.0001222.2e-05

3.1e-05

2e-064.7e-054e-06

2.2e-050.0001252.1e-05

1.5e-054.4e-051.5e-05

2.2e-05

0.0025250.025520.0062230.0134780.0126590.002520.022594

6e-062.3e-056e-06

3e-062.6e-051e-06

3e-062.6e-051e-06

0.0003990.0012120.0005020.0020270.0002250.0003810.001034

0.0003910.0012120.0005020.0020270.000220.0003760.001034

5e-063e-066e-06

1e-051.3e-059e-06

0.0003730.0012120.0005020.0020270.0001990.0003590.001034

2e-063e-061e-06

01e-060

1e-061e-061e-06

8e-065e-065e-06

4e-061e-063e-06

0

0

1e-060

1e-064e-06

2e-0602e-06

00

0.3171210.2197840.243760.1800770.2744650.3044630.164504

0.3171210.2197840.243760.1800770.2744650.3044630.164504

0.3171210.2197840.243760.1800770.2744650.3044630.164504

0.3171210.2197840.243760.1800770.2744650.3044630.164504

0.3171210.2197840.243760.1800770.2744650.3044630.164504

3e-060.0015383e-06

0.3171180.2197840.243760.1800770.2729270.304460.164504

1.2e-053.7e-057e-06

1.2e-053.7e-057e-06

1.2e-053.7e-057e-06

1.2e-053.7e-057e-06

1.2e-053.7e-057e-06

1.2e-053.7e-057e-06

3.9e-050.0002043.2e-05

1.3e-05

1.3e-05

1.3e-05

1.3e-05

1.3e-05

1.5e-059.6e-051.5e-05

4e-063.4e-055e-06

4e-063.4e-055e-06

4e-063.4e-055e-06

4e-063.4e-055e-06

1.1e-056.2e-051e-05

1.1e-056.2e-051e-05

1.1e-052.2e-051e-05

1.1e-052.2e-051e-05

1.8e-05

1.8e-05

2.2e-05

2.2e-05

2.2e-054.2e-051.6e-05

2.2e-054.2e-051.6e-05

2.2e-054.2e-051.6e-05

2.2e-054.2e-051.6e-05

2.2e-054.2e-051.6e-05

2e-062.4e-051e-06

2e-062.4e-051e-06

2e-062.4e-051e-06

2e-062.4e-051e-06

2e-061.1e-051e-06

1.3e-05

2.9e-05

2.9e-05

2.9e-05

2.9e-05

2.9e-05

1.7e-052.2e-051.4e-05

1.7e-052.2e-051.4e-05

1.7e-052.2e-051.4e-05

1.7e-052.2e-051.4e-05

1.7e-052.2e-051.4e-05

1.7e-052.2e-051.4e-05

3.1e-056e-053e-05

3.1e-056e-053e-05

3.1e-056e-053e-05

3.1e-056e-053e-05

3.1e-056e-053e-05

3.1e-056e-053e-05

3.8e-050.0002924.2e-05

3.8e-050.0002924.2e-05

3.8e-050.0002924.2e-05

1.8e-050.0001022.2e-05

1.2e-058.8e-051.5e-05

1.2e-058.8e-051.5e-05

6e-061.4e-057e-06

2e-06

6e-061.2e-057e-06

2e-050.000192e-05

4.6e-05

4.6e-05

2e-063.3e-052e-06

2e-063.3e-052e-06

1.2e-052.4e-051e-05

1.2e-052.4e-051e-05

6e-068.7e-058e-06

6e-062.9e-058e-06

4.2e-05

1.6e-05

1.3e-050.0001126e-06

1.3e-050.0001126e-06

1.3e-050.0001126e-06

1.3e-050.0001126e-06

4e-062.4e-052e-06

4e-062.4e-052e-06

2e-062.6e-052e-06

2e-062.6e-052e-06

4e-062.9e-051e-06

4e-062.9e-051e-06

3e-063.3e-051e-06

3e-063.3e-051e-06

2e-063.3e-051e-06

2e-063.3e-051e-06

2e-063.3e-051e-06

2e-063.3e-051e-06

2e-063.3e-051e-06

2e-063.3e-051e-06

3.4e-050.0002063.7e-05

3.4e-050.0002063.7e-05

2.1e-055.9e-051.9e-05

2.1e-055.9e-051.9e-05

1.5e-053.9e-051.4e-05

1.5e-053.9e-051.4e-05

6e-062e-055e-06

6e-062e-055e-06

1.3e-050.0001471.8e-05

9e-063.2e-051.6e-05

4e-061.3e-059e-06

4e-061.3e-059e-06

5e-061.9e-057e-06

2e-061e-055e-06

3e-069e-062e-06

4e-068.9e-052e-06

1e-061e-051e-06

1e-061e-051e-06

0

04.2e-050

1.4e-05

02.8e-050

1e-068e-060

1e-068e-060

2e-062.9e-051e-06

2e-062.9e-051e-06

2.6e-05

2.6e-05

2.6e-05

3e-062.8e-051e-06

3e-062.8e-051e-06

3e-062.8e-051e-06

3e-062.8e-051e-06

3e-062.8e-051e-06

3e-062.8e-051e-06

0.0048170.0283970.0080460.0314150.0051140.0042580.025191

7.4e-05

7.4e-05

7.4e-05

7.4e-05

7.4e-05

0.0048170.0283970.0080460.0314150.005040.0042580.025191

0.0047140.0283970.0080460.0314150.0045970.0041740.025191

0.0047140.0283970.0080460.0314150.0045970.0041740.025191

7e-062e-066e-06

3e-0602e-06

4e-062e-064e-06

0.0047070.0283970.0080460.0314150.0045950.0041680.025191

3e-06

1e-063e-060

01e-061e-06

1e-061e-061e-06

1e-068e-065e-06

4e-06

2e-065e-052e-06

0.0046460.0283970.0080460.0314150.004330.0041080.025191

3.4e-05

6e-06

2e-061e-062e-06

9e-061.7e-053e-06

02e-060

1e-061e-061e-06

1e-066e-061e-06

1e-05

1.7e-052e-061.4e-05

6e-061e-056e-06

8e-06

01e-062e-06

4e-06

1e-0601e-06

1e-062e-06

2e-063e-061e-06

2.5e-05

3e-0603e-06

4e-065e-063e-06

8e-06

2e-067e-065e-06

2e-061e-051e-06

1e-06

01e-06

4e-062.9e-054e-06

1e-061e-06

3e-06

1e-061e-06

7.1e-050.0003345.8e-05

7.1e-050.0003345.8e-05

2.6e-052.4e-051.7e-05

2.2e-058e-061.4e-05

4e-06

2e-068e-062e-06

1e-06

2e-063e-061e-06

4.5e-050.000314.1e-05

4e-060.0001342e-06

2.5e-055.3e-052.3e-05

6.1e-05

1.6e-056.2e-051.6e-05

3.2e-050.0001092.6e-05

4e-066e-062e-06

4e-066e-062e-06

4e-066e-062e-06

2.8e-050.0001032.4e-05

2.8e-050.0001032.4e-05

6e-063e-063e-06

3e-06

4e-065e-064e-06

0

7e-062.8e-051e-05

4e-06

1.5e-05

6e-06

4e-06

9e-061.8e-057e-06

9e-06

2e-068e-06

1.3e-050.0001351e-05

1.3e-050.0001351e-05

1.3e-050.0001351e-05

1.3e-050.0001351e-05

9e-063.8e-057e-06

9e-063.8e-057e-06

3.2e-05

3.2e-05

4e-066.5e-053e-06

4e-063.9e-053e-06

2.6e-05

0.0023290.0016350.0020370.0023310.0022780.0022270.001379

9e-062.7e-058e-06

9e-062.7e-058e-06

9e-062.7e-058e-06

9e-062.7e-058e-06

9e-062.7e-058e-06

1.8e-053.9e-051.6e-05

1.8e-053.9e-051.6e-05

1.8e-053.9e-051.6e-05

1.8e-053.9e-051.6e-05

1.8e-053.9e-051.6e-05

0.0023020.0016350.0020370.0023310.0022120.0022030.001379

0.0023020.0016350.0020370.0023310.0022120.0022030.001379

0.0023020.0016350.0020370.0023310.0022120.0022030.001379

0.0022670.0016350.0020370.0023310.0021170.0021750.001379

6e-062e-055e-06

0.0022610.0016350.0020370.0023310.0020970.002170.001379

1.6e-051.9e-051.2e-05

1.6e-051.9e-051.2e-05

4e-062.5e-053e-06

4e-062.5e-053e-06

1.5e-055.1e-051.3e-05

8e-062.7e-056e-06

7e-062.4e-057e-06

0.07959900000000010.0640150.0646450.0537090.07485100000000010.07640400000000010.038316

06e-06

06e-06

06e-06

06e-06

06e-06

06e-06

5e-062.2e-054e-06

5e-062.2e-054e-06

5e-062.2e-054e-06

5e-062.2e-054e-06

5e-062.2e-054e-06

5e-062.2e-054e-06

1e-061.9e-051e-06

1e-061.9e-051e-06

1e-069e-061e-06

1e-069e-061e-06

1e-069e-061e-06

1e-063e-061e-06

6e-06

1e-05

1e-05

4e-06

4e-06

6e-06

6e-06

4e-060.0001614e-06

4e-060.0001614e-06

1e-062.3e-051e-06

1e-062.3e-051e-06

8e-06

1e-06

7e-06

5e-06

5e-06

1e-061e-051e-06

1e-065e-061e-06

000

0

01e-060

4e-06

3e-066.3e-053e-06

2e-065.2e-052e-06

6e-06

6e-06

2e-062.3e-052e-06

2e-061.1e-052e-06

1.2e-05

1.7e-05

1.7e-05

6e-060

4e-06

2e-060

1e-061.1e-051e-06

1e-061.1e-051e-06

1e-06

1e-067e-061e-06

0

3e-06

05.4e-050

01.8e-050

07e-060

07e-060

6e-06

6e-06

5e-06

5e-06

03.6e-050

1.3e-05

2e-06

1.1e-05

5e-06

5e-06

1e-05

1e-05

04e-060

04e-060

04e-060

1e-06

03e-060

2.1e-05

1.7e-05

1.7e-05

1.7e-05

4e-06

4e-06

4e-06

0.07958900000000010.0640150.0646450.0537090.07464300000000010.07639500000000010.038316

03.6e-050

03.6e-050

03.6e-050

7e-06

3e-06

4e-06

4e-06

4e-06

02.5e-050

4e-06

06e-060

7e-060

08e-060

0.0015230.0010060.0013030.0020270.0011880.0014590.000858

0.0015230.0010060.0013030.0020270.0011880.0014590.000858

0.0015230.0010060.0013030.0020270.0011880.0014590.000858

2e-063.3e-051e-06

1e-06

1.8e-05

2e-062e-061e-06

01e-060

03e-060

2e-060

06e-060

0.0015210.0010060.0013030.0020270.0011440.0014580.000858

1e-06

0.0015170.0010060.0013030.0020270.001090.0014560.000858

5e-06

6e-06

2e-06

4e-06

02e-060

8e-06

2e-064e-062e-06

3e-06

3e-06

1e-063e-060

2e-06

2e-06

3e-06

1e-062e-060

04e-060

1.1e-05

1.1e-05

7e-060.000175e-06

7e-060.000175e-06

7e-060.0001625e-06

3e-061.9e-051e-06

3e-061.9e-051e-06

1e-05

2e-06

1e-06

7e-06

03.2e-050

5e-060

2e-06

1.1e-050

01.4e-050

4e-060.0001014e-06

1e-061.9e-051e-06

1.6e-05

2e-061e-052e-06

1e-061.2e-051e-06

1.2e-05

3.2e-05

8e-06

8e-06

8e-06

0.0034810.0024170.0032290.0028370.0033440.003342

0.0034810.0024170.0032290.0028370.0033440.003342

0.0034810.0024170.0032290.0028370.0033440.003342

0.0034810.0024170.0032290.0028370.0033440.003342

01.8e-050

0.0034810.0024170.0032290.0028370.0033260.003342

1.3e-050.0002391.2e-05

3.8e-05

3.8e-05

3.8e-05

3.8e-05

1e-060.0001290

1e-060.0001290

2.1e-05

2.1e-05

1e-068.2e-050

1e-068.2e-050

02.6e-050

02.6e-050

1.2e-057.2e-051.2e-05

6e-060

6e-060

6e-060

3e-063.3e-053e-06

3e-063.3e-053e-06

3e-063.3e-053e-06

09e-060

09e-060

09e-060

9e-062.4e-059e-06

9e-062.4e-059e-06

8e-061.7e-058e-06

1e-067e-061e-06

3e-060.0001024e-06

3e-060.0001024e-06

1e-063.5e-052e-06

07e-060

07e-060

1e-062.8e-052e-06

01e-060

03e-061e-06

2e-060

01e-06

1e-062e-051e-06

1e-06

2e-066.7e-052e-06

2e-050

2e-050

2e-064.7e-052e-06

01.5e-050

1.8e-05

1e-064e-061e-06

1e-061e-051e-06

0.0714990.0586940.0578370.0442850.0672840.068640.036187

0.0714750.0586940.0578370.0442850.0670790.0686160.036187

8e-068.3e-056e-06

5e-066.2e-055e-06

4e-062.5e-054e-06

1e-063.7e-051e-06

3e-062.1e-051e-06

3e-062.1e-051e-06

0.0714640.0586940.0578370.0442850.066870.0686080.036187

0.0714640.0586940.0578370.0442850.066870.0686080.036187

0.0714640.0586940.0578370.0442850.066870.0686080.036187

2e-068.9e-051e-06

1e-066.1e-051e-06

8e-06

1e-062.2e-051e-06

03.1e-050

1e-062.8e-050

1e-062.8e-050

1e-063.7e-051e-06

1e-063.7e-051e-06

1e-063.7e-051e-06

6e-063e-054e-06

6e-063e-054e-06

6e-063e-054e-06

4e-062.1e-052e-06

2e-069e-062e-06

1.8e-050.0001752e-05

6e-063.5e-056e-06

6e-063.5e-056e-06

2e-061.9e-052e-06

4e-061.6e-054e-06

1.2e-050.000141.4e-05

5e-061.7e-055e-06

5e-061.7e-055e-06

1e-067e-060

1e-067e-060

5e-064.3e-054e-06

3e-06

1e-05

2e-063e-062e-06

3e-063e-062e-06

04e-060

2e-06

5e-06

1e-06

1e-06

5e-06

5e-06

1e-06

1.4e-05

9e-06

5e-06

07e-063e-06

07e-063e-06

1e-062.9e-051e-06

1e-062.9e-051e-06

02.3e-051e-06

02.3e-051e-06

0.0030630.0018980.0022760.004560.002280.0029330.001271

0.0011010.000710.0009230.0019250.0010150.0010410.000536

9e-06

9e-06

9e-06

8e-065.3e-056e-06

4e-063.9e-053e-06

1.9e-05

4e-066e-063e-06

1.4e-05

4e-061.4e-053e-06

4e-061.4e-053e-06

0.0010930.000710.0009230.0019250.0009530.0010350.000536

0.0010490.000710.0009230.0019250.0008820.0010070.000523

3e-061.3e-054e-06

0.0010460.000710.0009230.0019250.0008690.0010030.000523

7e-061.5e-056e-06

7e-061.5e-056e-06

1.8e-054.6e-051.5e-051.3e-05

8e-062e-058e-061.3e-05

1e-052.6e-057e-06

1.9e-051e-057e-06

5e-06

1.4e-055e-065e-06

5e-0602e-06

0.001920.0011880.0013530.0026350.0011760.0018480.000735

6e-064.5e-056e-06

6e-064.5e-056e-06

6e-064.5e-056e-06

0.0019140.0011880.0013530.0026350.0011310.0018420.000735

3e-061.2e-053e-06

3e-061.2e-053e-06

0.0019060.0011880.0013530.0026350.00110.0018350.000735

0.000138

2.3e-052.6e-052.2e-05

0.0018830.0011880.0013530.0026350.0009360.0018130.000735

5e-061.9e-054e-06

5e-061.9e-054e-06

4.2e-058.9e-054.4e-05

4.2e-058.9e-054.4e-05

5e-061.9e-058e-06

5e-061.9e-058e-06

1.1e-051.9e-051.1e-05

5e-061.1e-055e-06

6e-068e-066e-06

4e-069e-063e-06

4e-069e-063e-06

1e-059e-061e-05

1e-059e-061e-05

6e-068e-066e-06

6e-068e-066e-06

3e-068e-063e-06

3e-068e-063e-06

3e-068e-063e-06

3e-068e-063e-06

9e-06

9e-06
