# Supplementary material for: MetaMeta: integrating metagenome analysis tools to improve taxonomic profiling
Source: Microbiome. 2017 Aug 14;5:101. doi: 10.1186/s40168-017-0318-y (PMC5557516; doi:10.1186/s40168-017-0318-y)
Supplement: Supplementary file 2 — Additional File with interactive charts for all CAMI toy set results on default, very-precise and very-sensitive mode. File prefix S, M, and H for low, medium and high complexity, respectively. (TAR 3573 kb) [file 40168_2017_318_MOESM2_ESM.tar › S_S001__genomes_30__insert_180_default.html]

Javascript must be enabled to view this page.

magnitude
magnitudeUnassigned

clark.parsed\_profile
dudes.parsed\_profile
final.metametamerge.profile
gottcha.parsed\_profile
kaiju.parsed\_profile
kraken.parsed\_profile
motus.parsed\_profile

0.9999911.0000090.99999210.9999939999999980.9999910.999999

0.0112910.0094250.0053080.0168190.011472

1e-060.00023

1e-060.00023

1e-060.00023

1e-060.00023

1e-060.00023

1e-060.00023

0.0001120.0001720.000105

0.0001120.0001720.000105

0.0001120.0001720.000105

0.0001120.0001720.000105

0.0001120.0001720.000105

0.0001120.0001720.000105

4e-060.0002040.00055e-06

4e-060.0002040.00055e-06

0.0002040.00046

0.0002040.00046

0.0002040.000379

0.0002040.000379

8.1e-05

8.1e-05

4e-064e-055e-06

4e-064e-055e-06

4e-064e-055e-06

4e-063.2e-055e-06

8e-06

0.0111530.0094250.0051040.0144370.0113

6.2e-050.0003744.9e-05

6.2e-050.0003744.9e-05

6.2e-050.0003744.9e-05

2.8e-054.8e-051.6e-05

2.8e-054.8e-051.6e-05

3.5e-05

1.9e-05

1.6e-05

3.4e-050.0002913.3e-05

1e-066.6e-051e-06

6.5e-05

3e-05

3.3e-050.000133.2e-05

0.0002120.0004810.000239

0.0002120.0004810.000239

1e-057.1e-051.3e-05

5e-065.1e-057e-06

1e-06

4e-063.9e-055e-06

1e-062e-061e-06

3e-06

4e-06

2e-061e-06

5e-062e-056e-06

5e-062e-056e-06

0.0002020.000410.000226

0.0002010.000380.000201

3.9e-054.7e-054e-05

6e-061.7e-056e-06

0.0001290.0003080.000128

2.7e-058e-062.7e-05

1e-063e-052.5e-05

1e-063e-052.5e-05

0.0016480.0008210.0024920.001725

0.0007580.0004620.0010620.000777

0.0006460.0003980.0005440.000656

0.0003910.0003030.0003230.000406

0.0002160.0002450.0001720.000218

0.0001755.8e-050.0001510.000188

7.1e-055.4e-054.1e-055.2e-05

4e-053.9e-052.9e-053.1e-05

1e-05

3.1e-051.5e-052e-062.1e-05

7.1e-054.1e-058.5e-057.3e-05

7.1e-054.1e-058.5e-057.3e-05

0.0001139.5e-050.000125

6.3e-054.2e-056.2e-05

5e-055.3e-056.3e-05

5.8e-05

5.8e-05

5.8e-05

0.0001126.4e-050.000460.000121

6.2e-053.2e-050.0002116.4e-05

6.2e-053.2e-054.5e-056.4e-05

8.6e-05

8e-05

5e-053.2e-050.0002495.7e-05

5e-053.2e-050.0002495.7e-05

0.0005320.0001030.0006530.000533

0.0005320.0001030.0006530.000533

7.8e-050.0001416.5e-05

7.8e-050.0001416.5e-05

0.0001483.1e-057.9e-050.000144

6.4e-053.9e-056.3e-05

8.4e-053.1e-054e-058.1e-05

4.5e-05

4.5e-05

7.1e-054e-055.5e-05

7.1e-054e-055.5e-05

7.6e-053.6e-050.000158.2e-05

7.6e-053.6e-050.000158.2e-05

7.4e-054.9e-058e-05

7.4e-054.9e-058e-05

3.9e-059e-054.8e-05

3.9e-059e-054.8e-05

4.6e-053.6e-055.9e-055.9e-05

4.6e-053.6e-055.9e-055.9e-05

0.0003580.0002560.0007770.000415

0.0001120.0001640.0004350.000139

0.0001120.0001640.0004350.000139

0.0001120.0001640.0004350.000139

0.0002469.2e-050.0003420.000276

5.4e-053.4e-050.0001137.3e-05

5.4e-053.4e-050.0001137.3e-05

7.1e-053.2e-050.0001087.6e-05

7.1e-053.2e-050.0001087.6e-05

0.0001212.6e-050.0001210.000127

2.2e-05

6.6e-053.9e-057.4e-05

5.5e-052.6e-056e-055.3e-05

0.0088630.0084440.0039730.0062990.008807

9.8e-05

9.8e-05

9.8e-05

9.8e-05

5.2e-050.0004274.6e-05

5.2e-050.0004274.6e-05

7e-067e-051e-06

7e-067e-051e-06

0.000148

0.000148

4.5e-050.0002094.5e-05

4.5e-050.0002094.5e-05

0.0088110.0084440.0039730.0057740.008761

0.0003010.0001870.0001760.0008990.000416

0.0003010.0001870.0001760.0008990.000416

0.0003010.0001870.0001760.0008990.000416

1.3e-050.0001162.4e-05

1.3e-050.0001162.4e-05

1.3e-050.0001162.4e-05

2e-068.1e-050.000380.0007062e-06

2e-068.1e-050.000380.0007062e-06

2e-068.1e-050.000380.0007062e-06

0.0084950.0081760.0034170.0040530.008319

0.0084950.0081760.0034170.0040530.008319

0.0072140.0071210.0027770.002810.007117

0.0012810.0010550.000640.0012430.001202

7e-050.0003430.000121

7e-050.0003430.000121

7e-050.0003430.000121

2.2e-05

2.2e-05

6.8e-050.0002040.000112

8e-064.2e-05

1.9e-05

7e-06

1.2e-052e-06

1e-05

4e-06

1.2e-05

1e-061.4e-051e-06

1e-068e-061e-06

1.5e-05

4e-06

5.7e-052.1e-055.7e-05

2.7e-05

1.1e-05

7e-06

4e-06

9e-062.1e-059e-06

2e-060.0001179e-06

7e-06

1.9e-05

2e-062.3e-053e-06

1.2e-05

7e-066e-06

3.1e-05

1.8e-05

2.2e-050.0002290.0009923.2e-05

2.2e-050.0002290.0009923.2e-05

2e-050.0005673e-05

2e-060.0003162e-06

2.6e-05

1e-063.2e-051e-06

0.000164

3.8e-05

2.9e-05

1e-062.7e-051e-06

1.6e-050.0001862.6e-05

5e-06

6e-061.9e-051.7e-05

8.2e-05

1e-058e-059e-06

2.8e-05

2.8e-05

2e-063.7e-052e-06

2e-061.7e-051e-06

7e-06

1.3e-051e-06

2e-060.0002290.0004252e-06

2e-060.0002290.0004252e-06

2e-060.0002290.0004252e-06

6.1e-050.000160.0005440.0010110.00012

6.1e-050.000160.0005440.0010110.00012

6.1e-050.000160.0005440.0010110.00012

6.1e-050.000160.0005440.0010110.00012

8.5e-050.000250.0004645.8e-05

6.1e-057.5e-050.0002940.0005476.2e-05

0.0002150.0003580.0024450.000207

2.8e-050.0003580.0017235.1e-05

4e-060.0003580.0014042.4e-05

00.0001652e-05

7e-06

1e-06

2e-06

1e-06

8e-05

1e-05

8e-061e-06

2.5e-05

1.5e-05

1e-06

01.2e-050

03e-061.9e-05

0.000102

0.000102

5.1e-05

5.1e-05

1e-060.0003580.0006651e-06

1e-060.0003580.0006651e-06

2.6e-05

2.6e-05

0.000318

4.1e-05

0.000277

3e-067.7e-053e-06

3e-067.7e-053e-06

2.4e-050.0003192.7e-05

2.4e-050.0003192.7e-05

2.3e-057.1e-052.6e-05

1e-060.0002481e-06

5e-069.6e-051.4e-05

5e-069.6e-051.4e-05

5e-069.6e-051.4e-05

1e-063.6e-052e-06

4e-066e-051.2e-05

0.0001820.0006260.000142

6.8e-050.0001555e-05

1e-061.9e-050

1e-061.9e-050

6.7e-050.0001365e-05

4.4e-052.4e-052.9e-05

2.3e-050.0001122.1e-05

8.6e-050.0001575.7e-05

8.2e-050.0001135.3e-05

1.8e-057.4e-053.9e-05

5e-06

6.4e-053.4e-051.4e-05

4e-064.4e-054e-06

4e-064.4e-054e-06

2.8e-057.2e-053.5e-05

2.8e-057.2e-053.5e-05

2.8e-057.2e-053.5e-05

0.000242

0.000242

0.000242

2.1e-050.001486.2e-05

2.1e-050.001486.2e-05

1e-060.000321e-06

1e-060.000321e-06

00.0001440

2e-06

6.7e-05

4.1e-05

07e-060

2.7e-05

8.9e-05

6e-05

2.9e-05

1e-068.7e-051e-06

1e-068.7e-051e-06

0.0004183.8e-05

9.8e-05

9.8e-05

4e-06

6.1e-05

3.2e-05

1e-06

0.000323.8e-05

6.8e-05

6.8e-05

0.0001188e-06

6.4e-058e-06

5.4e-05

3.6e-057e-06

3.6e-057e-06

9.8e-052.3e-05

4.2e-052.3e-05

5.6e-05

2e-060.0004793e-06

0.0002631e-06

0.000216

0.000216

2.6e-05

2.6e-05

2.1e-051e-06

2.1e-051e-06

2e-060.0002162e-06

2.8e-05

1.4e-05

1.4e-05

3.8e-05

1e-05

2.8e-05

1e-067e-051e-06

1e-067e-051e-06

2.3e-05

2.3e-05

1e-065.7e-051e-06

1e-065.7e-051e-06

1.8e-050.0002632e-05

0.0001681e-06

0.0001681e-06

0.0001681e-06

1.8e-059.5e-051.9e-05

1.8e-059.5e-051.9e-05

1.8e-059.5e-051.9e-05

0.98870.9905839999999990.99468410.9831749999999980.9885190.999999

1.8e-050.0002362e-06

1.8e-050.0002362e-06

1.8e-050.0002362e-06

1.8e-050.0002362e-06

1.8e-050.0002362e-06

1.8e-050.0002362e-06

0.0014560.0003640.001870.001334

0.0014560.0001590.0014890.001334

0.0005160.0001590.0007080.000475

0.0005160.0001590.0007080.000475

1.8e-054.3e-051e-05

2e-06

1.2e-051.1e-054e-06

3e-061.6e-055e-06

3e-061.4e-051e-06

0.0004980.0001590.0006650.000465

9.8e-050.000247.4e-05

9.4e-058.5e-058.6e-05

0.0003060.0001590.0002760.000305

6.4e-05

0.0002620.0004270.000206

2.3e-051.2e-051.3e-05

2.3e-051.2e-051.3e-05

2.3e-051.2e-051.3e-05

0.0002390.0004150.000193

0.0002390.0004150.000193

2e-061.3e-057e-06

1.1e-05

2.8e-05

8.7e-05

0.0002

8.2e-052.6e-054.3e-05

5e-069e-062e-06

1.5e-05

1.7e-05

1e-054e-061e-06

0.000145e-060.00014

0.0006780.0003540.000653

0.0006780.0003540.000653

0.0006730.0003480.000508

1.2e-05

1e-06

0.0001015e-064.6e-05

7.7e-051.5e-056.1e-05

0.00013500.000121

4.7e-056e-064.7e-05

2e-061.1e-051e-06

2e-063e-062e-06

3e-06

7e-06

1.1e-05

9e-062.3e-051.5e-05

2.7e-05

1.9e-05

1e-061e-06

1e-062e-06

2.4e-053e-062e-05

5e-062e-064e-06

2e-06

5.2e-054.1e-052.6e-05

1e-066e-061e-06

1e-0600

5e-062e-063e-06

0.0001052.2e-050.000107

4e-060

4.2e-055e-058e-06

1.9e-05

2.8e-054e-063e-05

3e-06

9e-06

2e-060

2e-06

1.1e-05

3.6e-051.4e-05

2.1e-05

5e-066e-060.000145

4e-065e-060.000145

1e-061e-06

0.0002050.000381

0.0002050.000381

0.0002050.000381

0.0002050.000381

0.0002050.000381

0.0004860.0001190.000428

0.0004860.0001190.000428

0.0004860.0001190.000428

0.0004860.0001190.000428

0.0004860.0001190.000428

0.0004860.0001190.000428

0.0069160.0011570.0033520.015260.0070845.7e-05

6.6e-050.0003166.5e-05

6.6e-050.0003166.5e-05

5.1e-050.0001395.2e-05

5.1e-050.0001395.2e-05

5.1e-050.0001395.2e-05

1.5e-050.0001771.3e-05

1.5e-050.0001771.3e-05

1.5e-050.0001771.3e-05

0.0002330.0001660.0015960.000229

0.0002330.0011610.000229

1.4e-050.0002151.5e-05

1.4e-056.5e-051.5e-05

1.4e-055.1e-051.5e-05

1.4e-05

00.000150

04.9e-050

0.000101

6.5e-05

6.5e-05

6.5e-05

3.3e-050.0001623.6e-05

3.3e-050.0001253.6e-05

1.9e-052.3e-05

1.4e-050.0001251.3e-05

3.7e-05

3.7e-05

1.2e-050.0001611.2e-05

1.2e-050.0001611.2e-05

1.2e-050.0001611.2e-05

1.2e-055.2e-051.3e-05

1.2e-055.2e-051.3e-05

1.2e-055.2e-051.3e-05

3.5e-050.0001073.6e-05

3.5e-050.0001073.6e-05

4e-062.9e-054e-06

7e-069e-061e-05

3e-062.1e-052e-06

1e-055e-069e-06

1.2e-05

2e-068e-064e-06

9e-061.5e-057e-06

8e-06

1e-064.1e-051e-06

1e-062e-051e-06

1e-062e-051e-06

2.1e-05

2.1e-05

3.7e-050.0002274.8e-05

3.7e-058.1e-054.8e-05

2.8e-057.7e-054.1e-05

9e-064e-067e-06

0.000146

0.000146

8.9e-050.0001316.8e-05

8.9e-050.0001316.8e-05

4.5e-057e-061.9e-05

2.2e-053.4e-052.2e-05

2e-06

1e-063e-061e-06

1.9e-051.8e-052.3e-05

1.6e-05

5e-06

1.8e-05

2e-062.8e-053e-06

0.0001660.000435

0.000127

0.000127

0.000127

0.0001660.000308

0.0001660.000308

0.0001660.000308

0.0007290.0002240.0011280.000697

0.0007290.0002240.0011280.000697

0.0007290.0002240.0011280.000697

3.7e-050.0002043.5e-05

3.7e-050.0002043.5e-05

6e-060.0003171e-05

2.3e-05

6e-060.0001251e-05

8.6e-05

8.3e-05

1.3e-050.0003351e-05

1.3e-050.0001431e-05

0.000192

0.0006730.0002240.0002410.000642

0.0006730.0002240.0002410.000642

3.1e-05

3.1e-05

0.0004280.0002710.000301

0.0004280.0002710.000301

0.0004280.0002710.000301

1.5e-053.1e-057e-06

1.5e-053.1e-057e-06

0.000115

8.9e-05

2.6e-05

0.0004136.7e-050.000294

0.0004136.7e-050.000294

4e-05

4e-05

1.8e-05

1.8e-05

0.000890.0005030.0001540.000995

0.000890.0005030.0001540.000995

0.000890.0005030.0001540.000995

0.0003490.0002167.7e-050.000471

0.0003490.0002167.7e-050.000471

0.0005410.0002877.7e-050.000524

0.0005410.0002877.7e-050.000524

0.0042140.0011570.0024590.0087820.0044555.7e-05

0.0042140.0011570.0024590.0087820.0044555.7e-05

3.8e-05

3.8e-05

3.8e-05

1.9e-050.0001761.5e-05

1.9e-050.0001761.5e-05

1.9e-050.0001761.5e-05

0.0039140.0011570.0024590.0084420.0041745.7e-05

0.00040.000743

0.00040.000743

0.0003070.00057

0.0003070.00057

0.0002165.1e-050.0005550.000242

0.0002165.1e-050.0005550.000242

2e-050.0001292.3e-05

2e-050.0001292.3e-05

0.0001954.9e-050.0002950.000193

0.0001954.9e-050.0002950.000193

6.9e-058.4e-059.1e-05

1e-06

5e-054.3e-056.2e-05

1.9e-054e-062.9e-05

3.6e-05

6.6e-050.0001517.2e-05

6.6e-050.0001517.2e-05

0.0001010.0001350.000103

0.0001010.0001350.000103

7.9e-050.0001948e-05

7.9e-050.00018e-05

4.9e-05

4.5e-05

8.3e-057.7e-059.2e-05

8.3e-057.7e-059.2e-05

0.000278

0.000278

6.1e-050.000195e-05

6.1e-050.000195e-05

7.6e-052.4e-050.0001328.2e-05

7.6e-052.4e-050.0001328.2e-05

0.0001810.000336

0.0001810.000336

9.3e-050.0001349.8e-05

9.3e-050.0001349.8e-05

0.0004470.00083

0.0004470.00083

0.000460.0004080.0002680.000464

5.7e-054.8e-055.3e-056.2e-05

0.0001010.0001171.6e-050.000102

0.0001368.3e-057.1e-050.000132

8.8e-054.5e-050.0001029.5e-05

7.8e-050.0001152.6e-057.3e-05

0.000137

0.000137

0.0002373.3e-050.0002390.0003

0.0002373.3e-050.0002390.0003

0.0009980.0005360.0004450.0009820.0010565.7e-05

0.0009980.0005360.0004450.0009820.0010565.7e-05

1.9e-05

1.8e-05

1e-06

4.4e-051.9e-054.8e-05

4.4e-051.9e-054.8e-05

1.3e-050.0001431.3e-05

1.3e-050.0001431.3e-05

0.0001030.0002190.0006530.000111

0.0001030.0002470.000111

0.0002190.000406

6e-06

1e-06

2e-06

3e-06

1.1e-059e-061.1e-05

1.1e-059e-061.1e-05

5e-05

1.7e-05

1.1e-05

5e-06

1.7e-05

0.0009425.6e-050.000460.0001470.000994

2.2e-05

0.0001023.3e-057.3e-050.000126

0.000842.3e-050.000465.2e-050.000868

4.7e-050.0003425.1e-05

4.7e-050.0002635.1e-05

7.9e-05

6.2e-05

6.2e-05

0.000533

0.000277

0.000256

0.0002496.5e-050.000235

0.0002496.5e-050.000235

2.4e-053e-062.3e-05

0.0001321.6e-050.000132

1.3e-05

1.6e-059e-062.1e-05

1.6e-054e-061.7e-05

2e-065e-06

6e-067e-064e-06

5.3e-058e-063.8e-05

3.2e-056.1e-053.1e-05

3.2e-056.1e-053.1e-05

3.2e-056.1e-053.1e-05

0.0003560.0030130.000342

0.0003560.0030130.000342

0.000129

0.000129

0.000129

3.9e-050.0004164.1e-05

5e-064.3e-055e-06

5e-064.3e-055e-06

2.8e-050.0001613e-05

2.8e-050.0001613e-05

0.000192

0.000192

6e-062e-056e-06

1e-05

6e-061e-056e-06

2.4e-050.0002232.3e-05

2.4e-050.0002232.3e-05

2.4e-050.0002232.3e-05

0.000961

0.000388

0.000141

0.000172

7.5e-05

0.00029

0.000226

6.4e-05

0.000283

7e-06

9e-06

4.6e-05

6.8e-05

3.1e-05

9.3e-05

2.9e-05

0.0001310.0005680.000151

2e-066.4e-053e-05

2e-066.4e-053e-05

3.6e-05

3.6e-05

4.6e-050.000223.8e-05

4.6e-050.000223.8e-05

2.8e-054.2e-052.5e-05

2.8e-054.2e-052.5e-05

3.1e-052.9e-05

3.1e-052.9e-05

9e-060.0001051.4e-05

9e-060.0001051.4e-05

1.5e-050.0001011.5e-05

1.5e-050.0001011.5e-05

4.6e-050.0002412.9e-05

2.8e-050.0001912e-05

2.8e-050.0001912e-05

1.8e-055e-059e-06

1.8e-055e-059e-06

0.0001160.0004759.8e-05

0.000198

0.000198

6e-05

6e-05

0.0001160.0002179.8e-05

0.0001160.0002179.8e-05

0.000450.0005318.9e-05

6.4e-05

6.4e-05

6.4e-05

6.4e-05

6.4e-05

0.0003830.0003711.6e-05

0.0003830.0003711.6e-05

0.0003830.0003711.6e-05

0.0003830.0003711.6e-05

0.0003830.0003711.6e-05

6.7e-059.6e-057.3e-05

6.7e-059.6e-057.3e-05

6.7e-059.6e-057.3e-05

6.7e-059.6e-057.3e-05

6.7e-059.6e-057.3e-05

0.3000620.4361720.0828940.2380.0757210.2871820.083817

0.3000620.4361720.0828940.2380.0757210.2871820.083817

0.2996380.4361720.0828940.2380.0746150.2868220.083817

0.2996380.4361720.0828940.2380.0746150.2868220.083817

0.298530.4361720.0824140.2380.0737080.2856790.083817

3.1e-050.0003143.9e-05

1.6e-05

0.2984510.436160.0824140.2380.072950.2855870.082392

0.000150.001425

1.3e-050.0001872.2e-05

3.5e-051.2e-059.1e-053.1e-05

0.0001090.0003330.000127

0.0001090.0003330.000127

9.4e-050.0003040.0001

9.4e-050.0003040.0001

0.0009050.000480.000270.000916

1.7e-050.0002112.1e-05

0.0008880.000485.9e-050.000895

0.0004240.0011060.00036

0.000360.0009630.00029

0.000360.0009630.00029

5e-05

1.5e-054.3e-051.2e-05

8.7e-050.0002226.2e-05

8.5e-053.1e-057.3e-05

5e-050.0001664.8e-05

0.000142

5.7e-056.1e-055.7e-05

3.4e-053.6e-052e-05

3.2e-050.0001831.8e-05

1.6e-05

1.3e-05

6.4e-050.0001437e-05

6.4e-050.0001437e-05

6.4e-050.0001437e-05

0.3322160.4379690.3088750.54370.1216140.3386340.385891

0.0137320.0511650.037780.06630.0198050.0122850.021851

0.0122820.0511650.0372390.06630.0176740.0107910.021851

0.0012810.0201920.0114110.0018130.000580.000119

0.000119

0.000119

0.0008960.0001820.0006390.0008040.000139

0.000156

4.3e-055.7e-055e-05

0.000190.000352

1.3e-052.6e-055e-059e-06

5e-060.0001589e-06

0.00013

0.0008350.0004495.7e-057.1e-05

0.0001610.0176470.00950.0003440.000203

2.1e-050.0001044.8e-05

0.0161150.008675

0.00024

0.0015320.000825

0.000140.000155

0.0023630.001272

0.0023630.001272

9.5e-050.0001349.7e-05

9.5e-050.0001349.7e-05

0.0001290.0005310.000141

0.0001290.0005310.000141

0.0008310.0001370.0001650.06630.0005660.000714

0.0005110.0001650.0001770.000453

0.0005110.0001650.0001770.000453

7.2e-058.8e-050.000139

7.2e-058.8e-050.000139

0.0002480.0001370.06630.0001280.000122

0.0002480.0001370.06630.0001280.000122

0.000173

0.000173

0.0014260.0004510.0007380.0027250.0013050.000785

8.9e-055.7e-050.0001595.4e-05

4e-057.4e-053.2e-05

4.9e-055.7e-058.5e-052.2e-05

0.000207

0.000207

0.001280.0003940.0007380.0023080.0011990.000785

1.3e-05

2.3e-052.6e-052.3e-05

0.00026

8e-060.0001555e-06

2.1e-057.1e-052e-05

4e-063.6e-053e-06

4.4e-05

3.6e-051.4e-052.8e-05

0.0009530.0003860.0003160.0004850.000895

1.5e-05

0.0004220.000785

1.3e-058e-051.6e-05

1.5e-052e-061.5e-05

2.8e-053.7e-052.9e-05

0.000279

7.7e-052.2e-056.4e-05

0.000144

5.3e-05

1.4e-050.000161.3e-05

0.000249

3.1e-05

1e-058e-062.3e-051.9e-05

6.2e-053.5e-054.1e-05

08e-060

1.6e-056.6e-052.8e-05

5.7e-055.1e-055.2e-05

2.1e-054.5e-051.7e-05

3.6e-054e-063.5e-05

2e-06

5.3e-050.0002025.9e-05

5.3e-050.0002025.9e-05

5.3e-050.0002025.9e-05

0.001440.0008620.0006350.0011020.001458

8e-060.0002197e-06

8e-060.0002197e-06

0.0014320.0008620.0006350.0008830.001451

0.0006545e-050.0003460.000610.000666

0.0007780.0008120.0002890.0002730.000785

3.4e-050.0003240.0006683.7e-05

8e-060.0003240.0006028e-06

8e-060.0003240.0006028e-06

2.6e-056.6e-052.9e-05

2.6e-056.6e-052.9e-05

0.0048940.0244890.019070.0045460.004340.020769

0.0002620.0065210.0004590.001250.0002190.000573

0.0002730.000508

0.000140.0065010.0001860.000399.1e-050.000573

0.0001222e-050.0003520.000128

0.000257

0.000257

2.6e-050.0002784.9e-05

2.6e-050.0002784.9e-05

0.0023820.001185

0.00018

0.0022020.001185

0.0051270.0030910.000615

0.0006610.000356

0.0044660.002404

0.0003310.000615

0.0037780.0103460.0032780.0015420.003761

0.0037780.0072950.0016360.0015420.003761

0.0030510.001642

0.000101

0.000101

3.8e-050.0001615.6e-05

3.8e-050.0001615.6e-05

0.000791.2e-050.0110570.0004430.0002550.020196

0.000791.2e-050.0002170.0004430.000255

3e-05

3e-05

0.010840.020136

0.001810.0001910.0005820.0018550.00179

4.4e-05

4.4e-05

5.8e-050.000223.7e-05

5.8e-050.000223.7e-05

0.00014.5e-050.0004040.000135

4.7e-050.0001466.8e-05

1.1e-050.0001062.7e-05

4.2e-054.5e-050.0001524e-05

1.1e-050.0001031.1e-05

1.1e-050.0001031.1e-05

0.0011110.0005826.1e-050.001054

0.0011110.0005826.1e-050.001054

0.0003570.0001460.0005610.000387

3.9e-050.0001024.1e-05

8e-060.0001081.3e-05

2.2e-057e-051.4e-05

0.0002180.0001460.000220.000267

3.3e-05

3.7e-056.1e-055.2e-05

02.3e-051e-06

0

04e-061e-06

1.9e-05

0.0001290.0004830.000165

0.000231

2.9e-050.0001525.7e-05

9.1e-055.4e-050.0001

9e-064.6e-058e-06

1.3e-050.004820.0043140.0033759e-060.000178

0.004820.002535

0.0047090.002535

0.000111

0.0013230.002458

0.0013230.002458

0.000178

0.000178

1.3e-056.9e-059e-06

1.3e-056.9e-059e-06

0.0004560.000848

0.0004560.000848

0.000214

0.000214

0.000214

0.0002382.3e-050.0003750.000262

0.0002382.3e-050.0003750.000262

0.0002382.3e-050.0003750.000262

9.7e-058.7e-058e-05

9.7e-058.7e-058e-05

9.7e-058.7e-058e-05

0.0001650.0001460.000157

0.0001650.0001460.000157

0.0001650.0001460.000157

0.0001480.000780.000203

7.8e-050.0001557.1e-05

3e-060.0001031e-06

3e-060.0001031e-06

7.5e-055.2e-057e-05

7.5e-055.2e-057e-05

7e-050.0006250.000132

2.6e-050.0002292.7e-05

2.6e-050.0002292.7e-05

4.4e-050.0003960.000105

1e-050.0003557.7e-05

3.4e-054.1e-052.8e-05

0.0010160.0005417.2e-050.000994

0.0010160.0005417.2e-050.000994

0.0010160.0005417.2e-050.000994

0.0010160.0005417.2e-050.000994

0.0002860.0012790.000297

3.2e-050.0001952.5e-05

1.1e-050.0001275e-06

1.1e-050.0001275e-06

2.1e-056.8e-052e-05

2.1e-056.8e-052e-05

0.0001360.0002220.000113

5.2e-058e-053e-05

8e-062.3e-054e-06

6e-061.6e-056e-06

2.6e-051e-061e-05

1e-06

1e-061e-06

2.3e-053e-06

1.2e-051.3e-056e-06

02e-060

2.8e-057.3e-052.6e-05

2.4e-053.4e-052.3e-05

4e-063.9e-053e-06

5.6e-056.9e-055.7e-05

5.6e-056.9e-055.7e-05

2.4e-058.7e-052.8e-05

2.4e-058.7e-052.8e-05

2.4e-058.7e-052.8e-05

9.4e-050.0007750.000131

2.4e-056.1e-052.2e-05

2.4e-056.1e-052.2e-05

2.7e-052.5e-053.4e-05

2.7e-052.5e-053.4e-05

1e-050.0001141.3e-05

1e-050.0001141.3e-05

1.5e-050.0002171.6e-05

1.5e-050.0002171.6e-05

1.5e-050.0002962.6e-05

1.5e-050.0002962.6e-05

2e-063.3e-058e-06

2e-063.3e-058e-06

1e-062.9e-051.2e-05

4e-061e-06

1e-064e-069e-06

09e-062e-06

1.2e-05

0.000162

0.000162

0.000162

0.000162

0.000162

0.3106570.382140.0778030.47740.0637750.3184790.044607

0.0277620.0358750.0221320.06110.0092410.0269930.006173

0.0018760.000840.0011180.00239

2.7e-054.4e-051.1e-05

1.3e-05

3e-061e-052e-06

2.4e-052.1e-059e-06

0.0018490.000840.0010740.002379

2.2e-051e-061.9e-05

0.000208

3.8e-051.6e-050.00013

1e-05

1.9e-05

0.0001262.1e-050.000123

2.4e-050.0001817e-06

2.9e-05

0.001360.000846e-060.001828

1.1e-051e-052.3e-05

2e-06

1.1e-051e-051.5e-05

1.5e-05

6.1e-051.5e-055.3e-05

4.2e-05

3e-063e-061e-06

5.5e-055.3e-051.2e-05

6e-06

1.1e-052e-061.2e-05

3e-065e-061.5e-05

8e-06

2.1e-05

1.3e-056e-065e-06

3.8e-05

0.000105

1.2e-05

0.000144

1e-06

7.1e-051.9e-058.1e-05

3.4e-05

3e-062.2e-052e-06

2e-053e-061.9e-05

9e-061.2e-05

8e-063e-062.2e-05

04e-060

0.0006221.8e-050.0002170.0002460.000282

8e-062.4e-053.6e-05

8e-062.4e-053.6e-05

2.3e-052.3e-051.4e-05

2.3e-052.3e-051.4e-05

0.0005911.8e-050.0002170.0001990.000232

0.0004020.0002171.3e-054.9e-05

3.4e-051e-053.1e-05

8e-061.2e-054e-06

8e-05

0.0001261.7e-050.000126

4.3e-05

2.1e-051.8e-052.4e-052.2e-05

2.3e-050.0003962.9e-05

2.3e-050.0003962.9e-05

5.1e-05

0.000115

9.8e-05

2.9e-05

7.8e-05

2.3e-052.5e-052.9e-05

0.0007130.0141040.02620.0008980.000837

3.6e-050.0003276.3e-05

3.4e-054.6e-052.6e-05

2e-063.2e-053.7e-05

0.000249

0.0006770.0141040.02620.0005710.000774

0.0002182e-060.000171

1e-062e-064e-05

3e-062.3e-054e-06

7e-066e-067e-06

2e-062.4e-052e-06

4.5e-059.9e-058.6e-05

2.1e-059e-062.1e-05

7e-061e-062e-06

8e-06

7e-063e-066e-06

7e-062e-069e-06

3.2e-05

1e-062.5e-051e-06

3.7e-052.4e-053.4e-05

3.9e-051.2e-055.1e-05

1.4e-05

9e-064e-068e-06

5.8e-051.1e-054.2e-05

1e-063e-061e-06

6e-051e-055.9e-05

4.2e-05

5e-066e-064e-06

4e-061.7e-051.7e-05

1e-061.6e-052e-06

2e-067e-066e-06

1e-063.6e-056e-06

2e-064.5e-05

1.6e-050.0141040.02621.6e-058e-06

4.5e-052.5e-054.2e-05

1.6e-053e-062.6e-05

6.3e-051.1e-057.4e-05

4.3e-05

3.2e-05

1e-0600

1e-06

0.0002660.0001890.000257

0.0002660.0001890.000257

0.0001198.2e-050.000117

4.4e-051.9e-053.8e-05

0.0001032.1e-050.000102

6.7e-05

0.0242620.0358570.0069710.03490.0063940.0231980.006173

0.0241210.0358570.0069710.03490.0062920.0230670.006173

6e-06

3.6e-05

0.0241210.0358570.0069710.03490.006250.0230670.006173

6.1e-055.3e-055.4e-05

3e-061e-052e-06

2e-06

5e-061e-051e-06

1e-068e-062e-05

1.5e-05

1e-061e-061e-06

5.1e-057e-063e-05

8e-054.9e-057.7e-05

8e-054.9e-057.7e-05

0.2828950.3462650.0556710.41630.0545340.2914860.038434

8.8e-050.0006917.5e-05

0.000199

0.000199

5.6e-05

5.6e-05

0.000141

0.000141

0.000101

1.9e-05

9e-06

1.5e-05

1.6e-05

4e-06

8e-06

5e-06

1.5e-05

1e-05

8.8e-054.1e-057.5e-05

8.8e-054.1e-057.5e-05

0.000153

0.000153

0.085060.129490.0230110.07270.0166720.0815060.002378

0.0812590.1290930.0221390.07270.0133850.07790.002378

2e-061.5e-053e-06

1.8e-05

0.0802410.1286820.0218570.07270.0131330.076843

9e-05

1.8e-05

3e-066e-065e-060.000373

2.1e-05

0.0008630.0002180.0002825e-060.0008470.001407

7.7e-053e-050.0001386e-05

1e-064e-061.3e-05

1e-068.9e-05

6.1e-054.5e-053.8e-050.000359

2.6e-05

4.7e-05

1.1e-050.0001931.6e-051.3e-05

5.3e-05

3.1e-05

2.2e-05

5.8e-055.6e-056.3e-05

5.8e-055.6e-056.3e-05

4.3e-05

4.3e-05

0.000114

0.000114

0.0013280.0002260.0002290.001308

3e-05

0.0013280.0002260.0001780.001308

2.1e-05

2.8e-050.0001383.7e-05

2.8e-050.0001383.7e-05

0.000155

0.000155

1.4e-050.000291.8e-05

1.4e-050.000291.8e-05

5.1e-05

6e-06

3.7e-05

8e-06

0.0023690.0003970.0006460.0020180.002178

9.9e-05

8.2e-055.9e-057.5e-05

4e-061.1e-051e-064e-06

5.4e-05

9e-068e-068e-062.2e-05

3.6e-05

7.2e-05

2e-052.3e-051e-062.1e-05

9.2e-05

6e-057e-06

1.1e-050.0001031e-05

0.0002490.0001886e-060.000282

4e-050.0001175.5e-05

0.0001750.0001049.9e-05

1.1e-052.1e-0504e-05

4.3e-05

01.4e-0500

9.4e-051.6e-050.000115

0.000191

1e-06

6e-067.6e-051e-067e-06

3.2e-053.8e-054.7e-05

7e-06

6.1e-051.7e-054.4e-05

0.0001032.6e-050.0001840.000235

0

0

0.000464.9e-050.000137

4.5e-055e-061e-063.3e-05

0.000203

6e-061.6e-059e-06

2.9e-05

0.0001770.000329

2.9e-052.5e-053e-065.4e-05

4.1e-05

9e-0601.1e-05

2.1e-05

0.0008630.0004690.000878

6.9e-05

0

4e-064e-062e-06

4e-064e-062e-06

0.000136

0.000136

0.0001915e-064.9e-050.000203

0.0001915e-064.9e-050.000203

5.9e-051.2e-055.4e-05

2.6e-051.1e-052.5e-05

8e-065e-062e-061.1e-05

9e-066e-062.2e-05

8.9e-051.8e-059.1e-05

9.8e-050.00030.000201

9.8e-050.0002380.000201

2.5e-054e-064.7e-05

4.3e-056e-069.8e-05

1.4e-050.0001881.2e-05

2.1e-05

1.6e-051.9e-054.4e-05

6.2e-05

6.2e-05

0.000410.0001630.0006430.000521

0.0003230.0003020.000422

2e-06

5e-06

1e-061e-061e-06

0.0001381.3e-050.000133

1.1e-052.1e-058e-06

2.4e-056e-062.3e-05

4e-063e-066.5e-05

8.7e-053e-069.8e-05

4e-061.1e-058e-06

2e-066e-061e-06

7e-06

1e-05

7.7e-05

4e-06

1.3e-051e-067e-06

7.8e-05

1.5e-05

3.9e-053.9e-057.8e-05

0.0001630.000303

0.0001630.000303

8.7e-053.8e-059.9e-05

8.7e-053.8e-059.9e-05

0.1969620.216770.0324970.34360.0359050.2088550.036056

0.196580.2166690.0324970.34360.0351650.2084140.036056

0.0006180.0001810.0001660.0002450.000575

4.9e-05

0.0001530.0001740.000211

0.000238

7.9e-05

0.000287

6.6e-05

6.6e-051.8e-055.7e-057.9e-05

0.000271

7.1e-05

0.1418950.0987680.0231620.21910.0266520.1792240.013402

6.6e-05

0.0001

0.0005840.001086

0.0001730.000321

4.2e-05

0.00027

0.0004325.8e-050.0001810.0002140.000518

0.000108

0.000135

0.000194

0.0534160.1176440.0080690.12450.003510.0278070.022654

0.000255

0.0001620.000302

0.000123

5.5e-05

5.9e-05

0.000136

0.000151

0.000151

0.0001310.0002130.000162

0.0001310.0002130.000162

0.0002510.0001010.0003760.000279

0.0002510.0001010.0001940.000279

0.000182

4.9e-050.0002368e-05

1.8e-057.3e-051.3e-05

1.8e-057.3e-051.3e-05

3.1e-050.0001636.7e-05

3.1e-050.0001636.7e-05

3.7e-053.8e-054.5e-05

3.7e-053.8e-054.5e-05

3.7e-053.8e-054.5e-05

0.0072920.0046640.1932920.035570.0073390.319433

0.0072920.0046640.1932920.035570.0073390.319433

0.0072920.0046640.1932920.035570.0073390.319433

0.1900410.0335870.319433

0.171960.319433

0.0180810.033587

0.00022

0.00022

0.000290.0021320.0001660.0004520.000246

0.0001660.000307

0.000290.0021320.0001450.000246

0.0007060.001311

0.0007060.001311

0.0070020.0025320.0023790.007093

0.0070020.0025320.0023790.007093

6.3e-050.0005564.8e-05

6.3e-050.0005564.8e-05

6.3e-050.0005564.8e-05

4.2e-057.7e-054.3e-05

4.2e-057.7e-054.3e-05

0.00018

0.00018

2.1e-050.0001695e-06

2.1e-050.0001695e-06

0.00013

0.00013

0.0004720.0017460.000483

7.3e-050.0002167e-05

7.3e-050.0002167e-05

7.3e-050.0002167e-05

5e-060.0001093e-06

6.8e-050.0001076.7e-05

0.0001740.0007520.000228

0.0001740.0007520.000228

0.000262

0.000262

9.6e-050.0001440.000147

9.6e-050.0001440.000147

7.8e-050.0003468.1e-05

7.8e-050.0003468.1e-05

0.0002250.0007780.000185

0.000219

0.000219

7.6e-05

0.000143

0.0002250.0005590.000185

0.0002250.0005590.000185

0.0001070.0003510.0001

0.0001180.0001228.5e-05

3.7e-05

1e-05

3.9e-05

2.4e-050.0002312.5e-05

2.4e-050.0002312.5e-05

2.4e-050.0002312.5e-05

2.4e-050.0002312.5e-05

2.4e-050.0002312.5e-05

2.4e-050.0002312.5e-05

0.0003420.000220.0016920.000388

0.0003420.000220.0016920.000388

0.0001530.000480.000208

0.0001530.000480.000208

2.7e-050.0001296.3e-05

2.7e-050.0001296.3e-05

7.7e-05

7.7e-05

0.0001260.0002740.000145

0.0001260.0002740.000145

0.0001280.0005580.000126

5.6e-050.0003185.4e-05

2e-060.0001941e-06

1e-061e-05

1e-060.0001631e-06

0

2.1e-05

5.4e-050.0001245.3e-05

8.5e-05

8e-06

1e-06

1e-06

9e-06

4e-06

0

5.4e-051e-065.3e-05

1.5e-05

7.2e-050.000247.2e-05

5.4e-050.0001235.3e-05

2.6e-054.3e-052.6e-05

1.3e-05

2.8e-056.7e-052.7e-05

1.8e-050.0001171.9e-05

9e-068.3e-057e-06

9e-063.4e-051.2e-05

6.1e-050.000220.0006545.4e-05

6.1e-050.000220.0006545.4e-05

4.8e-050.0001994.8e-05

4.8e-050.0001994.8e-05

1.3e-050.000220.0004556e-06

1.3e-050.000220.0004096e-06

4.6e-05

6.8e-050.0005680.0010565.3e-05

6.8e-050.0005680.0010565.3e-05

6.8e-050.0005680.0010565.3e-05

6.8e-050.0005680.0010565.3e-05

6.8e-050.0005680.0010565.3e-05

6.8e-050.0005680.0010565.3e-05

0.0001550.0005860.000171

0.0001550.0005860.000171

0.0001550.0005860.000171

0.0001550.0005860.000171

2e-050.0001022.7e-05

2e-050.0001022.7e-05

6.3e-05

6.3e-05

6.4e-057.7e-059.2e-05

6.4e-057.7e-059.2e-05

3.4e-050.0002842e-05

3.4e-050.0002842e-05

3.7e-056e-053.2e-05

3.7e-056e-053.2e-05

0.0008030.0013090.000687

5.8e-050.0002076.4e-05

3.9e-050.0001433.5e-05

3.9e-050.0001433.5e-05

3.9e-050.0001433.5e-05

3.9e-050.0001433.5e-05

1.9e-056.4e-052.9e-05

1.9e-056.4e-052.9e-05

1.9e-056.4e-052.9e-05

0

1.9e-056.4e-052.9e-05

7.1e-050.0002496.8e-05

7.1e-050.0002496.8e-05

1.5e-050.0001141.5e-05

1.5e-050.0001141.5e-05

2e-06

1.5e-050.0001121.5e-05

0

5.6e-050.0001355.3e-05

5.6e-050.0001355.3e-05

1e-053.4e-051e-05

4.6e-050.0001014.3e-05

1.3e-050.0001611.3e-05

1.3e-050.0001611.3e-05

1.3e-050.0001611.3e-05

9.3e-05

9.3e-05

1.3e-056.8e-051.3e-05

1.3e-056.8e-051.3e-05

0.0002840.0005260.000269

0.0001820.0002520.000202

0.0001820.0002520.000202

0.0001820.0002520.000202

0.0001820.0002520.000202

0.0001020.0002746.7e-05

0.0001020.0002746.7e-05

0.0001020.0002746.7e-05

0.0001020.0002746.7e-05

0.0003770.0001660.000273

0.0003770.0001660.000273

0.0003770.0001660.000273

0.0003770.0001660.000273

0.0003770.0001660.000273

0.0266330.0051850.0028250.0267360.0270560.001062

0.0071530.0020880.0011320.0040540.0071680.000728

0.0071530.0020880.0011320.0040540.0071680.000728

0.0071530.0020880.0011320.0040540.0071680.000728

0.0071530.0020880.0011320.0040540.0071680.000728

0.0071530.0020880.0011320.0040540.0071680.000728

0.0180230.0030970.0012290.0191480.0184380.000334

0.0010990.0002160.0010680.001172

0.0010990.0002160.0010680.001172

0.0001744.5e-050.000172

0.0001744.5e-050.000172

0.0001462.7e-050.0001090.000158

0.0001462.7e-050.0001090.000158

4.2e-05

4.2e-05

7.3e-05

7.3e-05

0.0001482.1e-058.8e-050.000187

0.0001482.1e-058.8e-050.000187

0.000295.7e-050.0003260.000323

0.000295.7e-050.00010.000323

6.4e-05

1e-06

8.4e-05

3e-06

7e-05

4e-06

0.0002336.6e-050.0001660.000222

0.0001691.7e-052.5e-050.000159

6.4e-054.9e-052.6e-056.3e-05

0.000102

1.3e-05

0.0001088.7e-050.00011

0.0001088.7e-050.00011

0.000104

0.000104

7.3e-05

7.3e-05

0.0003614.7e-050.0001080.0004

0.0003614.7e-050.0001080.0004

0.0003614.7e-050.0001080.0004

0.0003614.7e-050.0001080.0004

0.0004951.9e-050.0002648.2e-050.000485

0.0004951.9e-050.0002648.2e-050.000485

0.0004951.9e-050.0002648.2e-050.000485

0.0004951.9e-050.0002648.2e-050.000485

0.0009020.0001940.00040.000921

0.0009020.0001940.00040.000921

0.0002545.6e-050.0001230.000272

0.0002545.6e-050.0001230.000272

0.0003496.8e-050.0001050.000357

0.0003496.8e-050.0001050.000357

0.0002997e-050.0001720.000292

0.0002997e-050.0001720.000292

0.0007534.6e-050.0006470.000804

0.0007534.6e-050.0006470.000804

0.0007534.6e-050.0006470.000804

0.0001340.0001120.000153

7.5e-056.6e-057.1e-05

0.0002073e-050.0001440.000242

0.0001540.0001980.000165

0.0001831.6e-050.0001270.000173

0.0008380.0001110.0006070.000887

0.0008380.0001110.0006070.000887

0.0005626.5e-050.0003910.000587

0.0001271.8e-057.8e-050.000142

0.00021.7e-059.7e-050.0002

0.0001141.4e-050.0001370.000122

0.0001211.6e-057.9e-050.000123

0.0001270.0001190.000132

6.5e-054.6e-056.6e-05

6.2e-057.3e-056.6e-05

9.2e-057.4e-059.8e-05

9.2e-057.4e-059.8e-05

5.7e-054.6e-052.3e-057e-05

2.1e-052.6e-051.1e-052.9e-05

3.6e-052e-051.2e-054.1e-05

0.0001624e-060.0004140.000208

0.0001624e-060.0004140.000208

5.9e-05

5.9e-05

1.1e-052.5e-051.1e-05

1.1e-052.5e-051.1e-05

8.9e-05

8.9e-05

0.0001514e-060.0002410.000197

2.5e-054e-062.5e-05

3.6e-053e-067e-05

3e-06

4.4e-05

7e-06

3.4e-05

2e-064e-062e-062e-06

3.5e-059e-063.7e-05

8e-061.8e-058e-06

2.1e-052.1e-053e-05

1.2e-051e-051.4e-05

0

2.1e-05

3.3e-05

8e-06

1.4e-05

1.2e-051e-051.1e-05

0.0011230.0002020.0011010.001147

0.000547.5e-050.0007880.000559

0.0003034.5e-050.000240.000296

0.0003034.5e-050.000110.000296

0.00013

0.000265

0.000265

0.000197

0.000197

0.0002373e-058.6e-050.000263

0.0002373e-058.6e-050.000263

0.0005830.0001270.0003130.000588

0.0001250.0001480.000116

0.0001250.0001480.000116

0.0001162.7e-056.5e-050.000115

0.0001162.7e-056.5e-050.000115

0.0001133.7e-050.000123

0.0001133e-050.000123

7e-06

3.8e-053.7e-054.4e-05

2e-061.3e-053e-06

3.6e-052.4e-054.1e-05

0.0001910.00012.6e-050.00019

0.0001910.00012.6e-050.00019

0.0001390.0002310.000108

0.0001390.0002310.000108

0.0001390.0002310.000108

0.0001390.0002310.000108

0.0001610.0002080.000166

0.0001610.0002080.000166

0.0001610.0002080.000166

0.0001610.0002080.000166

0.0035010.0007820.0001610.0044940.003625

2.5e-05

2.5e-05

2.5e-05

0.0003030.0003920.000348

0.0003030.0003920.000348

0.000159

0.0001173.4e-050.000131

0.0001177.1e-050.000139

6.9e-056.4e-057.8e-05

6.4e-05

0.0011730.0005470.0015070.001049

3.7e-050.0002324.4e-05

3.7e-050.0002324.4e-05

0.0011360.0005470.0012750.001005

7e-06

0.000258

0.0004145.4e-050.000214

6e-06

6e-06

1e-063e-061e-06

6.4e-051.7e-051.6e-057.7e-05

0

1e-06

3e-060

0

0.0001062e-053.2e-050.000133

8.5e-050.0001830.000104

4.6e-05

1.5e-058e-056e-061.8e-05

0

0.000115

8.8e-05

1.2e-051.3e-051.4e-05

5.3e-058.7e-056.6e-05

5e-062e-065e-06

6.3e-051.3e-052.1e-056.1e-05

0.000175

4e-061.1e-051e-065e-06

3.4e-052e-055e-064e-05

5.4e-05

5e-062e-067e-06

0.0001151.6e-050.0001040.000134

3e-05

4.3e-052e-053.6e-05

3.4e-05

1e-064e-061e-06

4.2e-058e-062e-064.8e-05

8e-06

5e-06

1.6e-05

6e-050

1.4e-054.3e-054.1e-05

0.0001870

0.0008557e-050.0001610.0018110.000936

0.0008557e-050.0001610.0018110.000936

9.9e-05

5.8e-05

9.9e-05

2.3e-05

1e-061e-062e-06

0.0001892.9e-050.000179

9e-06

2.3e-051.6e-057e-06

0.000115

3.9e-05

4.3e-052.6e-052.6e-053.7e-05

3.5e-05

2.9e-05

2.3e-05

2.8e-05

4e-068e-061e-063e-06

3.8e-051.2e-054e-05

4.8e-05

0

2.3e-052.4e-055.6e-05

9e-053e-050.000117

0.0001610.000299

01e-060

5.8e-051.3e-054.9e-05

8.8e-053.6e-055e-050.000103

1.5e-051.9e-057e-06

1.8e-05

0.0001112.8e-050.000137

0.0001073e-050.000151

3.7e-05

8.2e-05

2.3e-05

0.000246

3.1e-05

6.6e-05

2.6e-05

1.4e-05

3.4e-052.9e-053.5e-05

3.4e-05

3.1e-052.1e-051.3e-05

0.0001740.000150.000196

0.0001740.000150.000196

0.0001740.000150.000196

0.0009190.0001650.0005180.001021

0.0003757e-050.0002110.000405

6.9e-051.8e-055e-058e-05

7.1e-05

9.1e-051.4e-055.3e-050.000108

0.0002153.8e-053.7e-050.000217

0.0005449.5e-050.0003070.000616

1e-05

0.0001051.6e-052.8e-050.000122

3.5e-057e-063.5e-05

0.0001362.9e-051.3e-050.000151

6.2e-05

3e-06

6.8e-051.4e-052e-058.1e-05

2e-06

0.00023.6e-056.5e-050.000227

5.1e-05

1.4e-05

1.3e-05

1.9e-05

7.7e-059.1e-057.5e-05

7.7e-059.1e-057.5e-05

7.7e-059.1e-057.5e-05

0.0006438.9e-050.0006010.0006645.7e-05

0.0003097.3e-050.0003360.0003055.7e-05

7.7e-050.0001056.6e-055.7e-05

7.7e-050.0001056.6e-055.7e-05

0.0002327.3e-050.0002310.000239

0.0001053.6e-050.0001040.000111

0.0001273.7e-050.0001270.000128

0.000141.6e-050.0001130.000151

0.000141.6e-050.0001130.000151

0.000141.6e-050.0001130.000151

0.0001940.0001520.000208

0.0001940.0001520.000208

0.0001940.0001520.000208

0.0001960.0004440.000161

0.0001960.0004440.000161

0.00029

8.5e-05

7.1e-05

8.3e-05

5.1e-05

0.0001391.8e-050.000115

0.0001391.8e-050.000115

1.4e-05

1.4e-05

6.4e-05

6.4e-05

5.7e-055.8e-054.6e-05

5.7e-055.8e-054.6e-05

0.0057270.0007310.0008040.0065130.0056110.000167

0.0007510.0001320.0003660.000756

0.0007510.0001320.0003660.000756

0.0002073.6e-056.9e-050.000219

0.000365.5e-050.0001940.000352

0.0001844.1e-050.0001030.000185

0.0002672.3e-050.0001540.0002862.8e-05

0.0002672.3e-050.0001540.0002862.8e-05

0.0002672.3e-050.0001540.0002862.8e-05

2.3e-054.8e-051.1e-05

2.3e-054.8e-051.1e-05

2.3e-054.8e-051.1e-05

0.0007330.0001140.0002620.000739

0.0003275.1e-057.6e-050.000319

0.0003275.1e-057.6e-050.000319

0.0004066.3e-050.0001860.00042

0.0004066.3e-050.0001020.00042

8.4e-05

0.0022320.0002560.0006340.0014830.002036

6.1e-057.6e-057.1e-05

6.1e-057.6e-057.1e-05

0.000140.000220.000127

7e-050.0001166.1e-05

7e-052.8e-056.6e-05

7.6e-05

0.0012590.0006341.5e-050.001108

0.0012590.0006341.5e-050.001108

0.000256.6e-050.0002670.000234

9.6e-05

0.000256.6e-058.6e-050.000234

8.5e-05

0.000106

0.000106

1e-051.4e-058e-06

1e-051.4e-058e-06

0.0003660.0001380.0002850.000356

0.0003660.0001380.0002850.000356

6.9e-050.0004176.9e-05

3.1e-05

6.4e-05

0.00017

9e-069e-064e-06

1.6e-05

3.2e-05

3.5e-05

6e-06

2.4e-05

6e-052.3e-056.5e-05

7e-06

7.7e-055.2e-058.3e-056.3e-05

3.3e-055.2e-055.7e-051.7e-05

4.4e-052.6e-054.6e-05

0.0002875.2e-050.0003590.0002882.7e-05

9.4e-05

9.4e-05

0.0002875.2e-050.0001010.000288

0.0002875.2e-050.0001010.000288

0.0001642.7e-05

2.7e-05

0.000164

0.0002323.2e-050.0001320.000256

0.0002323.2e-050.0001320.000256

0.0002323.2e-050.0001320.000256

0.0015910.000112

0.0015910.000112

0.0015910.000112

0.0002792.6e-050.0002830.000284

0.000139

0.000139

0.0002792.6e-050.0001440.000284

0.0002792.6e-050.0001440.000284

0.0005216.6e-050.0013480.000555

0.00013

0.00013

0.0002332.5e-050.0005590.000229

5.2e-05

0.000181

0.0002332.5e-054.9e-050.000229

6.5e-05

5.4e-05

0.000158

6.4e-05

6.4e-05

7.6e-05

7.6e-05

0.000244

0.000244

4.6e-05

4.6e-05

6.5e-05

6.5e-05

5.1e-05

1.6e-05

3.5e-05

0.0001136.8e-050.000122

0.0001136.8e-050.000122

0.0001754.1e-054.5e-050.000204

0.0001754.1e-054.5e-050.000204

0.0003953e-050.000170.0004470.000394

0.000142

0.000142

0.0003953e-050.000170.0002270.000394

0.0003953e-050.000170.0002270.000394

7.8e-05

7.8e-05

7e-064e-056e-06

7e-064e-056e-06

7e-064e-056e-06

0.0002170.0002370.000241

0.0002170.0002370.000241

0.0002177.9e-050.000241

0.0002177.9e-050.000241

0.000158

0.000158

0.0016170.0006420.0019280.0017410.00011

0.0016170.0006420.0019280.0017410.00011

0.000142.6e-057.8e-050.000156

0.000142.6e-057.8e-050.000156

0.0014770.0006160.001850.0015850.00011

0.0001443.8e-052.1e-050.00016

7.1e-053.1e-055.7e-057.9e-05

1e-05

3.9e-05

0.0001073.8e-054.8e-050.00014

7.5e-05

3.4e-05

2.1e-05

0.000135

1.7e-05

0.0001032.1e-057.8e-050.000115

3.6e-05

7e-055.6e-052.4e-058e-05

2.9e-05

3.3e-05

9e-05

7.1e-05

1.1e-052.7e-058e-061.5e-05

2.2e-05

0.0001514.3e-053.2e-050.000158

5e-06

0.0001373.2e-057e-060.000166

1.8e-05

9.8e-052.9e-053.7e-05

7.4e-05

2.5e-05

0.0001042.9e-050.000118

3e-05

5.5e-05

9.4e-055.3e-054.5e-050.000123

2.1e-05

3.2e-05

3.5e-05

5.8e-05

1.7e-053e-051e-051.7e-05

4.1e-05

8e-055.3e-055.3e-059e-05

3.6e-05

3.5e-05

2.7e-05

5.8e-05

0.0001575.5e-052e-050.000165.5e-05

7e-052.5e-053.3e-057.9e-05

0.000153

2.5e-05

2.7e-05

6.3e-053.8e-052.2e-058.5e-05

6.1e-05

8.9e-051.4e-056.5e-059.7e-05

8.9e-051.4e-056.5e-059.7e-05

8.9e-051.4e-056.5e-059.7e-05

8.9e-051.4e-056.5e-059.7e-05

0.0004130.0002220.0006310.000375

0.0004130.0002220.0006310.000375

0.0004130.0002220.0006310.000375

0.0004130.0002220.0006310.000375

0.0004130.0002220.0004570.000375

0.000174

0.000750.0002420.0020180.000822

0.0003750.0009030.000405

0.0003750.0009030.000405

0.000187

0.000187

6.9e-059.5e-056.7e-05

6.9e-059.5e-056.7e-05

2.2e-050.0001226.8e-05

2.2e-050.0001226.8e-05

0.0001220.000150.000111

0.0001220.000150.000111

6.2e-056.1e-05

6.2e-056.1e-05

0.00010.0003499.8e-05

9e-050.0001538.1e-05

1e-050.0001961.7e-05

0.0003750.0002420.0011150.000417

0.0003440.000330.000395

0.0002350.0001340.000285

0.0002350.0001340.000285

0.0001090.0001960.00011

0.0001098.2e-050.00011

0.000114

3.1e-050.0002420.0007852.2e-05

3.1e-050.0003352.2e-05

3.1e-050.0003352.2e-05

0.0002420.00045

0.0002420.00045

0.0002940.0008850.000253

0.0002940.0008850.000253

0.0002940.0008850.000253

0.0001280.0007180.00013

0.0001280.0007180.00013

0.0001660.0001670.000123

0.0001660.0001670.000123

6.8e-050.0018397e-05

6.8e-050.0018397e-05

5.6e-050.0015235.3e-05

4.7e-050.0005774.1e-05

6e-060.0002833e-06

6e-060.0002833e-06

4.1e-050.0002943.8e-05

4e-050.0002833.7e-05

1e-061.1e-051e-06

9e-060.0008581.2e-05

4e-068.9e-057e-06

3e-068.9e-057e-06

1e-06

1e-060.0002071e-06

1e-060.0002071e-06

2e-060.0003632e-06

2e-060.0003422e-06

2.1e-05

2e-060.0001992e-06

2e-060.0001992e-06

8.8e-05

8.8e-05

8.8e-05

1.2e-050.0003161.7e-05

1.2e-050.0003161.7e-05

8e-064e-051.5e-05

8e-064e-051.5e-05

4e-060.0002762e-06

4e-060.0002762e-06

0.0002070.0003020.0012040.000214

0.0002070.0003020.0012040.000214

0.0002070.0003020.0012040.000214

2.5e-050.0001084.6e-05

1.8e-053.5e-054.1e-05

1.3e-05

1.8e-052.2e-054.1e-05

7e-067.3e-055e-06

7e-067.3e-055e-06

0.0001820.0003020.0010960.000168

0.0001190.0002320.000111

0.0001199.7e-050.000111

7.8e-05

5.7e-05

2e-060.000247e-06

2e-060.000247e-06

6.3e-05

6.3e-05

6.1e-050.0003020.0005615e-05

6.1e-050.0003020.0005615e-05

1.5e-050.0001681.2e-05

1.5e-050.0001681.2e-05

1.5e-050.0001681.2e-05

1.5e-050.0001681.2e-05

1.5e-050.0001681.2e-05

1.5e-050.0001681.2e-05

0.001430.0002080.0005820.001419

0.001430.0002080.0005820.001419

0.001430.0002080.0005820.001419

0.001430.0002080.0005820.001419

4.4e-05

4.4e-05

0.0013920.0001070.001385

0.0013920.0001070.001385

3.8e-050.0002080.0004313.4e-05

3.8e-054.5e-053.4e-05

0.0002080.000386

0.0022050.0007650.0015130.001067

0.0022050.0007650.0015130.001067

0.0022050.0007650.0015130.001067

0.0022050.0007650.0015130.001067

0.0008280.0003170.0003840.000777

0.0008280.0003170.0003840.000777

0.0013080.0002030.0003160.000248

0.0013080.0002030.0003160.000248

5.7e-050.0003583.2e-05

5.7e-050.0003583.2e-05

1.2e-050.0002450.0004551e-05

1.2e-050.0002450.0004551e-05

2e-055.4e-052e-05

2e-052e-05

2e-052e-05

2e-052e-05

2e-052e-05

2e-052e-05

5.4e-05

5.4e-05

5.4e-05

5.4e-05

5.4e-05

6.8e-050.0002122.5e-05

6.8e-050.0002122.5e-05

6.8e-050.0002122.5e-05

6.8e-050.0002122.5e-05

6.8e-050.0002122.5e-05

6.8e-050.0002122.5e-05

0.0035980.0001710.000830.0052880.003285.3e-05

7.3e-050.0003116.9e-055.3e-05

7.3e-050.0003116.9e-055.3e-05

7.3e-050.0003116.9e-055.3e-05

7.3e-050.0003116.9e-055.3e-05

4e-050.0001625.1e-055.3e-05

3.3e-050.0001491.8e-05

0.0035250.0001710.000830.0049770.003211

3.4e-050.0001550.000553.7e-05

4e-060.0001550.0004143e-06

0.0001550.000305

1.6e-05

0.0001550.000289

4e-060.0001093e-06

4e-060.0001093e-06

6e-060.0001021.1e-05

8.1e-052e-06

8.1e-052e-06

6e-062.1e-059e-06

6e-062.1e-059e-06

2.3e-052.4e-052.2e-05

2.3e-052.4e-052.2e-05

2.3e-052.4e-052.2e-05

1e-061e-051e-06

1e-061e-051e-06

7e-06

1e-063e-061e-06

0.0021350.0004910.001020.002008

0.000870.0002520.0002470.000819

0.000870.0002520.0002470.000819

0.000870.0002520.0002470.000819

0.0004450.0002390.0001050.000449

0.0004450.0002390.0001050.000449

0.0004430.0002391.1e-050.000447

2e-069.4e-052e-06

0.0004320.000160.000481

0.0004320.000160.000481

0.0004320.000160.000481

0.0003270.0002010.000192

00.0001181.3e-05

00.0001181.3e-05

0.0003118.3e-050.000167

0.0003118.3e-050.000167

1.6e-051.2e-05

1.6e-051.2e-05

6.1e-050.0003076.7e-05

6.1e-050.0003076.7e-05

3e-068.2e-055e-06

1e-061e-061e-06

3e-066.5e-053e-06

4e-061e-064e-06

1.8e-058.6e-051.9e-05

3.2e-057.2e-053.5e-05

0.0008510.0001710.0001840.0026060.00093

5e-061e-05

5e-061e-05

5e-061e-05

1.6e-056.6e-051.9e-05

1.6e-056.6e-051.9e-05

1.5e-05

7e-06

1.6e-054.4e-051.9e-05

8e-065.5e-055e-06

8e-065.5e-055e-06

8e-065.5e-055e-06

1.9e-050.0003764e-06

1.9e-050.0003764e-06

3.1e-05

6.5e-05

1.9e-050.000284e-06

6e-060.0003254e-06

6e-060.0003254e-06

6e-060.0003254e-06

0.0007870.0001710.0001840.001750.000887

1e-052.1e-053e-06

1e-052.1e-053e-06

6.7e-050.000146.7e-05

6.7e-050.000146.7e-05

0.0007010.0001710.0001840.001430.000808

5e-05

6e-06

7.4e-052e-067.1e-05

1e-06

4e-064e-061.8e-05

4.5e-05

2e-054.5e-051.5e-05

1.4e-051.5e-051.2e-05

5e-060.0001737e-06

2e-06

0.0001840.000342

3e-056.2e-053.2e-05

0.0004050.0001710.0002780.000483

3e-06

2.3e-05

0.0001014e-050.000105

1.1e-0501.1e-05

2e-060.0002092e-06

3e-060.000111e-05

4e-06

3.2e-051.6e-054.2e-05

9e-060.0001599e-06

6e-060.0001595e-06

3e-064e-06

1e-053.4e-051e-06

1e-053.4e-051e-06

2.6e-05

1e-058e-061e-06

4e-060.0001145e-06

4e-060.0001145e-06

4e-060.0001145e-06

4e-060.0001145e-06

7.6e-050.0001457.3e-05

5.1e-053.9e-054.7e-05

5.1e-053.9e-054.7e-05

5.1e-053.9e-054.7e-05

2.5e-050.0001062.6e-05

2.5e-050.0001062.6e-05

2.5e-050.0001062.6e-05

7.3e-05

7.3e-05

7.3e-05

7.3e-05

0.0004250.0004690.000158

1e-05

1e-05

1e-05

0.0003830.0001840.000108

9e-060.0001581e-05

0.00012

4e-061.7e-055e-06

5e-062.1e-055e-06

0.0003742.6e-059.8e-05

0.0003742.6e-059.8e-05

4.2e-050.0002755e-05

2.7e-050.0001831.3e-05

6.6e-05

1.9e-059.1e-055e-06

8e-062.6e-058e-06

1.1e-058.8e-053.1e-05

4e-061.1e-054e-06

1e-061.7e-051.2e-05

3e-062.4e-051e-05

3e-05

3e-066e-065e-06

3e-065e-06

3e-065e-06

1e-064e-061e-06

1e-064e-061e-06

0.0008090.0005260.0014050.000338

0.0008090.0005260.0014050.000338

0.0008090.0005260.0014050.000338

0.0008090.0005260.0014050.000338

1.1e-050.0002370.0004412.2e-05

1.1e-050.0002370.0004412.2e-05

0.0007810.0002890.0007930.000301

0.0002890.000537

8.6e-05

0.0007810.000170.000301

1.7e-050.0001711.5e-05

8e-060.0001619e-06

4e-06

9e-066e-066e-06

0.0027250.0001890.0010710.0033430.002644

0.0027250.0001890.0010710.0033430.002644

0.0012970.0001890.0005050.002520.001325

0.0001552.5e-050.000145

1.6e-051e-061.5e-05

0

0

1.6e-0501.5e-05

00

1e-06

0.0001392.4e-050.00013

1.1e-05

1e-061e-061.2e-05

5e-062e-06

2e-0601e-06

1.1e-051e-05

0.000121e-060.000115

1e-06

0.0011420.0001890.0005050.0024950.00118

0.0010780.0001890.000640.001118

6e-062.4e-053e-06

3.4e-05

1.1e-053.9e-051e-05

1e-055e-051e-05

7e-065.9e-057e-06

6.5e-05

1e-06

5e-069.4e-051.2e-05

7e-06

0.0010370.0001890.0001010.001028

2e-060.0001664.8e-05

1.8e-050.0002741.5e-05

1e-067.2e-051e-06

6e-060.0001223e-06

1.1e-058e-051.1e-05

2.8e-050.0004662.1e-05

1.6e-050.0003561.5e-05

1.2e-050.000116e-06

3e-060.0001763e-06

3e-060.0001763e-06

1.5e-050.0005050.0009392.3e-05

1.5e-050.0005050.0009392.3e-05

0.0002960.000750.000273

0.0002960.000750.000273

0.0002770.0004110.000258

1.1e-05

01e-05

0.0002750.000220.000256

0.000106

2e-066.4e-052e-06

1.9e-050.0003391.5e-05

1.9e-050.0003391.5e-05

0.0011320.0005667.3e-050.001046

0.0011320.0005667.3e-050.001046

0.0011320.0005667.3e-050.001046

0.0010860.00056600.001019

2.5e-052.7e-052.2e-05

1e-068e-061e-06

2e-053.8e-054e-06

0.0014480.0003810.0011450.001095

0.000151

0.000151

0.000151

0.000151

0.000151

7.4e-05

7.4e-05

7.4e-05

7.4e-05

7.4e-05

2.4e-050.0001143.6e-05

2.4e-050.0001143.6e-05

2.4e-050.0001143.6e-05

2.4e-050.0001143.6e-05

2.4e-057.6e-053.6e-05

3.8e-05

1.5e-054e-058e-06

1.5e-054e-058e-06

1.5e-054e-058e-06

1.5e-054e-058e-06

1.5e-054e-058e-06

0.0014090.0003810.0007660.001051

1.8e-050.0001680.0005971.6e-05

1.8e-050.0001680.0005971.6e-05

0.000122

0.000122

0.0001680.000313

0.0001680.000313

1.8e-050.0001621.6e-05

1.8e-050.0001621.6e-05

0.0013910.0002130.0001690.001035

0.0013910.0002130.0001690.001035

0.0013910.0002130.0001690.001035

0.0013910.0002130.0001690.001035

0.0003630.0002190.0007460.000102

0.0003630.0002190.0007460.000102

0.0002953.6e-052.6e-05

0.0002953.6e-052.6e-05

0.0002953.6e-052.6e-05

4e-06

3e-062e-06

1e-066e-061e-06

1e-061.1e-051e-06

2e-0601e-06

0.0002912e-061.9e-05

6e-06

0

04e-062e-06

00

6.8e-050.0002190.000717.6e-05

1e-067.5e-051e-06

1e-067.5e-051e-06

1e-067.5e-051e-06

5.5e-050.0002190.0004876.3e-05

5.4e-058.1e-055.1e-05

5.4e-055.1e-05

8.1e-05

1e-060.0002190.0004061.2e-05

1e-060.0002190.0004061.2e-05

1.2e-050.0001481.2e-05

1.2e-050.0001481.2e-05

1.2e-050.0001481.2e-05

0.000220.0015790.000182

0.0001080.0001288.9e-05

0.0001080.0001288.9e-05

0.0001080.0001288.9e-05

0.0001080.0001288.9e-05

0.0001080.0001288.9e-05

0.0001120.0014519.3e-05

0.0001120.0014519.3e-05

6.9e-050.0011666.5e-05

3e-060.000245e-06

3e-060.000245e-06

5e-060.00024e-06

5e-060.00024e-06

4.2e-050.0002973.5e-05

4.2e-050.0002973.5e-05

1.9e-050.0003692.1e-05

1.9e-050.0003692.1e-05

6e-05

6e-05

4.3e-050.0002852.8e-05

2.7e-050.000152.1e-05

2.7e-050.000152.1e-05

1.6e-050.0001357e-06

1.6e-050.0001357e-06

0.0002050.0013960.000289

0.0001560.0009560.000217

0.0001560.0009560.000217

0.0001560.0009560.000217

1.2e-050.0002464.6e-05

1.2e-050.0002464.6e-05

6.4e-050.0001778.6e-05

3.4e-054.4e-053.7e-05

3e-050.0001334.9e-05

3e-050.0002293.1e-05

3e-050.0002293.1e-05

5e-050.0003045.4e-05

1.8e-050.000133.2e-05

3.2e-050.0001742.2e-05

3.5e-050.0002726e-05

3.5e-050.0002726e-05

3.5e-050.0002726e-05

3.5e-050.0002726e-05

3.5e-050.0002726e-05

1.4e-050.0001681.2e-05

1.4e-050.0001681.2e-05

1.4e-050.0001681.2e-05

1.4e-050.0001681.2e-05

1.4e-050.0001681.2e-05

0.0058260.0050.0229

0.0058260.0050.0229

0.0058260.0050.0229

0.0058260.0050.0229

0.0058260.0050.0229

0.0058260.0050.0229

9e-060.0002841e-05

9e-060.0002841e-05

9e-060.0002841e-05

9e-060.0002841e-05

9e-060.0002841e-05

1e-062.9e-052e-06

8e-060.0002558e-06

0.3033940.1039150.5858840.19540.7130499999999990.3126580.529119

0.0011930.0002250.0006360.00098

0.0011930.0002250.0006360.00098

0.0011930.0002250.0006360.00098

0.0011930.0002250.0006360.00098

0.0010830.0002250.0001970.000864

2.8e-050.0001332.1e-05

8.2e-050.0003069.5e-05

0.227620.0359270.5239680.5709210.2325580.513964

0.000870.001616

0.000870.001616

0.0002950.000547

0.0002950.000547

0.0005750.001069

0.0005750.001069

0.0010730.001992

0.0010730.001992

0.0010730.001992

0.0010730.001992

0.0157790.0021170.3011520.0791430.0150050.51292

0.0070990.013188

0.0070990.013188

0.0034250.006363

0.0036740.006825

0.2755560.511875

0.2755560.511875

0.2755560.511875

0.0152740.0276740.000699

0.0003760.000699

0.0003760.000699

0.0075370.014001

0.0075370.014001

0.0073610.013673

0.0073610.013673

0.0157790.0021170.0032230.0382810.0150050.000346

0.0030480.000260.0003560.0080770.0027550.000346

0.0030480.000260.0003560.0080770.0027550.000346

0.0045080.000420.0007250.0078460.004196

0.0045080.000420.0007250.0078460.004196

0.0044460.0005890.000970.0131860.004536

0.0044460.0005890.000970.0131860.004536

0.0037770.0008480.0011720.0091720.003518

0.0037770.0008480.0011720.0091720.003518

0.0069820.0001860.0026390.0056070.0072475.5e-05

0.0069820.0001860.0026390.0056070.0072475.5e-05

0.002560.0001070.0002020.0019560.002385

0.002560.0001070.0002020.0019560.002385

0.0007570.0006680.0025660.001416

0.0007570.0006680.0025660.001416

0.0036657.9e-050.0017690.0010850.0034465.5e-05

0.0004240.000787

0.0011293.9e-050.0005643.4e-050.000976

0.0010194e-050.000546.7e-050.000986

1.1e-05

0.0015170.0002410.0001860.0014845.5e-05

0.002020.0004650.0043270.0124970.001964

0.002020.0004650.0043270.0124970.001964

3.9e-053.7e-050.0020390.0037874e-05

3.9e-053.7e-050.0020390.0037874e-05

0.0007580.0003620.0003840.0048570.000762

0.0007580.0003620.0003840.0048570.000762

0.0012236.6e-050.0019040.0038530.001162

0.0012236.6e-050.0007040.0016250.001162

0.0006090.001131

0.0005910.001097

0.0492640.0076980.0228220.0593930.050398

0.0040240.0002840.0004810.0022270.003851

0.0040240.0002840.0004810.0022270.003851

0.0040240.0002840.0004810.0022270.003851

0.0016140.002999

0.0016140.002999

0.0016140.002999

0.0026070.0004680.0006490.0021010.003081

0.0026070.0004680.0006490.0021010.003081

0.0026070.0004680.0006490.0021010.003081

0.0036990.0003190.004180.0082360.002161

0.0036990.0003190.004180.0082360.002161

0.000170.000315

0.0027720.000230.000350.0015550.001271

1.7e-05

0.0004550.000845

0.0007050.00131

0.0020360.003781

4e-052.5e-051.2e-055.1e-05

0.0008876.4e-050.0004647e-060.000839

0.000143

0.000251

0.0155290.0041160.0034990.0089240.017486

0.0155290.0041160.0034990.0089240.017486

3.9e-055.2e-050.0002460.000214

0.0008480.0005110.0004270.0013850.000848

6.9e-055.8e-050.0003850.001490.000471

2.1e-050.000390.000214e-062.6e-05

0.0013160.0003680.0004480.0016210.001408

3e-060.0001297e-064e-06

0.0007080.0002460.0002350.000715

1.6e-057.9e-056e-061.7e-05

0.0015440.0002340.0002670.0003730.002093

0.002130.0004590.0005190.0008950.002587

0.001920.0001190.0001770.000380.001678

0.0009240.000150.0001420.000779

0.0003620.0001870.0001510.001122

0.003510.0002010.0003270.0009430.003568

0.0005350.0005020.0001780.0001490.000675

0.0003890.0001553.1e-050.000464

0.0011950.0002760.0003260.0011010.000817

0.0177430.0021860.0107120.0244610.018159

0.0017920.003329

0.0017920.003329

0.0018570.0001090.0010090.0042960.00196

0.0013766.4e-050.0006080.0008380.001358

0.0004814.5e-050.0004010.0034580.000602

0.0018710.003476

0.0018710.003476

0.0001042.9e-050.0023339.8e-05

0.0001042.9e-050.0023339.8e-05

0.0018810.0005350.0006310.0011470.001808

1e-06

0.0001840.000342

0.0002346.2e-050.000174

0.000720.0002830.0002720.0005740.000799

1e-06

1e-06

0.0009270.0002520.0001750.0001660.000835

0.0139010.0015130.0054090.009880.014293

0.0004110.000764

0.0027170.0002780.0003890.0007960.003186

0.0002190.000406

0.0068090.0001870.0002790.0004780.006626

0.0005260.000976

0.0013150.002443

0.0011620.002158

0.0043750.0010480.0011080.0018590.004481

0.0022360.0001960.0004520.0049390.00193

0.0022360.0001960.0004520.0049390.00193

0.0005148.8e-050.0002530.0024650.000249

0.0017220.0001080.0001990.0024740.001681

0.0034260.0001290.0012350.0055060.00373

0.0034260.0001290.0012350.0055060.00373

0.0034260.0001290.0002510.0036770.00373

0.0009840.001829

0.008720.0001530.0029060.0037950.013799

0.008720.0001530.0029060.0037950.013799

2.5e-050.000292.9e-05

2.5e-050.000292.9e-05

6.2e-050.0001610.00284

6.2e-050.0001610.00284

1e-050.0001240.000364

1e-052.3e-050.000364

2.2e-05

7.9e-05

0.000212.7e-050.0002990.000844

5.5e-050.0002410.000807

0.0001552.7e-055.8e-053.7e-05

0.0049150.0001260.0008010.0008890.004005

0.0001014.8e-058e-05

0.0045899.4e-050.0008010.0006530.003746

0.0001420.0001837.4e-05

8.3e-053.2e-055e-060.000105

0.0018380.0014560.0008820.001976

0.0003470.0002197e-060.00049

0.0014470.0007761.8e-050.001437

4.4e-050.0002680.0004984.9e-05

0.0001930.000359

6.1e-050.000470.0009040.000843

6.1e-050.000470.0009040.000843

0.0015670.0001790.0001240.002855

0.0015670.0001790.0001240.002855

3.2e-050.0001224.3e-05

3.2e-050.0001224.3e-05

0.0153210.0016330.0180250.0523160.01524

0.0022380.0013310.0019360.001948

0.0022380.0013310.0019360.001948

0.0005170.00096

0.0022380.0008140.0009760.001948

0.0070790.0011920.0042490.016980.006801

0.005910.0009040.0020540.0075140.005668

0.0022570.00050.0006360.0021070.002144

0.002290.0003650.0005330.0024290.002202

0.0013633.9e-050.0008850.0029780.001322

8.1e-050.0001220.0017517.7e-05

8.1e-050.0001220.0017517.7e-05

9.8e-056.9e-050.0021970.000101

9.8e-056.9e-050.0021970.000101

0.000999.7e-050.0006640.0026750.000955

0.000999.7e-050.0006640.0026750.000955

0.0015310.002843

0.0015310.002843

0.0026560.004933

0.0026560.004933

0.0026560.004933

0.0011180.002077

0.0011180.002077

0.0011180.002077

0.0060040.0004410.0086710.026390.006491

0.0001713.1e-050.000180.0024280.000366

0.0001713.1e-050.000180.0024280.000366

0.0004550.000410.0024660.0112350.001706

0.000750.001394

0.0001560.0001840.0001690.0052920.001492

0.0012450.002313

6.6e-059.1e-050.0003020.000562

0.0002330.0001350.0016740.000214

0.0020940.00389

0.0020940.00389

0.0015930.00296

0.0015930.00296

0.005250.0023380.0038920.004305

0.0031880.0012010.0017590.002161

0.0020620.0011370.0021330.002144

0.0001280.0019850.000114

0.0001280.0019850.000114

0.0101020.0037020.0079420.0339590.006952

0.0090990.0036880.0043870.0279340.005661

0.0002740.00051

0.0002740.00051

0.0016030.0002850.0004310.0043760.001598

0.0016030.0002850.0004310.0043760.001598

0.0071470.0006940.0010760.0130180.00356

0.0037680.0003950.0005530.008093

0.0033790.0002990.0005230.0049250.00356

2.7e-050.0024550.0018070.0069363.2e-05

2.3e-050.0013140.0011470.0056142.3e-05

4e-060.0011410.000660.0013229e-06

0.0003220.0002540.0007990.0030940.000471

0.0003220.0002540.0002230.0020230.000471

0.0005760.001071

0.0010031.4e-050.0007890.0008860.001291

0.0010031.4e-050.0004410.0002390.001291

0.0001095.1e-054.7e-05

0.000159

0.0001231.4e-057e-060.000246

4.8e-051.2e-055.1e-05

0.0007230.0004412e-060.000947

8e-06

0.0003480.000647

0.0003480.000647

0.0027660.005139

0.0009840.001829

0.0009840.001829

0.0017820.00331

0.0017820.00331

0.0184580.0021530.0167940.0277670.01977

0.008620.0008120.0042780.008420.00804

0.0036750.0017190.0026670.003669

0.0007790.001447

0.0036750.000470.0003460.003669

0.000470.000874

0.0047370.0006640.0010470.0024820.004166

0.0002090.000389

0.000410.0001131.5e-050.000343

0.002640.0001170.000172.5e-050.00169

1.5e-05

5e-06

0.000880.0003270.0003770.001490.001179

0.0008070.0001073e-060.000954

0.0002910.00054

0.0002080.0001480.0015120.0032710.000205

0.0001640.000305

0.000158

0.0003360.000625

0.0002580.00048

7.6e-056e-067.3e-05

9.1e-056e-050.000229.3e-05

1.1e-058.8e-056e-068e-06

0.0003860.000716

3e-057.1e-053.1e-05

0.0003680.000684

0.0098380.0013410.0125160.0193470.01173

0.0008830.001641

0.0008830.001641

4.6e-053.4e-050.0006360.0014764.7e-05

4.6e-053.4e-050.0002944.7e-05

0.0006360.001182

0.0097920.0013070.0109970.016230.011683

0.000231

1e-06

0.000133

6.3e-05

00.000251

3.9e-050.0001142.5e-055.4e-05

0.0003360.000624

6.2e-05

4.2e-059.5e-050.0012740.0023674.8e-05

0.0006765.8e-050.000364

0.0002915.1e-050.0002168.1e-050.000648

0.0012010.0007623.4e-050.00172

2e-06

1e-06

0.000116

7e-06

0.000137

6.5e-05

2e-06

9e-06

0.0003570.0001667.1e-050.000341

0.000248

0.0010220.001899

1.3e-054e-059.4e-051.8e-05

9.1e-053.9e-050.0001520.000104

0.0002860.000531

2e-06

0.0001990.000371

0.0006635.7e-050.000352e-050.000637

3e-05

6e-050.00011e-060.000505

0.000191

0.0007933.4e-050.0002210.0001940.001113

0.0002960.000551

0.0009130.0003380.0003310.000640.001129

0.0008190.001522

0.0001820.000338

0.000139

0.000154

0.0006150.001142

0.0021020.0011489.8e-050.002163

0.0001750.000326

0.0006260.001162

2.5e-05

0.0002120.000394

7.4e-052.4e-050.0003088.8e-05

0.000165

1.8e-05

0.0005224.5e-050.0003391.6e-050.000797

4.4e-053.7e-050.0002338.7e-050.000433

0.0005767.7e-050.0003047.7e-050.000554

0.0013353.2e-050.0003390.0003070.001331

7e-06

9.3e-05

0.0003480.000646

0.0002988.8e-050.0030270.0058150.000306

0.0002845.2e-050.0016630.003110.000285

0.0003250.000604

0.0003250.000604

0.0002845.2e-050.0013380.0025060.000285

0.0004920.000915

2.1e-050.0003230.0005994.9e-05

0.0001580.000293

0.0003650.000679

0.0002635.2e-052e-050.000236

1.4e-053.6e-050.0013640.0027052.1e-05

1.4e-053.6e-050.0008330.0017192.1e-05

0.0008330.001547

1.4e-053.6e-050.0001722.1e-05

0.0005310.000986

0.0005310.000986

0.0013870.002577

0.0013870.002577

0.0013870.002577

0.0013870.002577

0.0086150.0016160.0037260.0087540.0088775.6e-05

0.0086150.0016160.0037260.0087540.0088775.6e-05

0.0021633.8e-050.0010060.0012860.002204

0.0021633.8e-050.0007220.0007580.002204

0.0002840.000528

0.0010890.002023

0.0010890.002023

0.0063580.0015170.0014240.0045980.0065585.6e-05

0.0001740.0001110.0005260.00014

6.1e-058.8e-059.1e-055.8e-05

0.0022310.0001770.000110.002218

8.8e-05

0.0005590.001038

0.000225

0.0002860.0001958.2e-050.0002895.6e-05

8.8e-05

0.0003044.2e-058e-050.000234

2.6e-05

0.0004110.0001142e-060.001064

0.0014060.0001370.0001090.001384

0.0002370.000441

0.0001296.4e-050.0002190.000126

0.000268

0.000750.0001540.0001750.0004380.000461

0.000212

0.0002580.00048

0.0002246e-052.1e-050.000251

0.0003820.0003750.0001955.4e-050.000333

9.4e-056.1e-050.0002070.0008470.000115

3.7e-053.1e-050.0002070.0003845e-05

5.7e-053e-050.0003566.5e-05

0.000107

0.0009910.0005710.0013830.000911

0.0009910.0005710.0013830.000911

0.0009910.0005710.0013830.000911

0.0009910.0005710.0013830.000911

0.0395150.006670.0164560.0198650.042570.000933

0.0072450.0002390.0022410.0018330.007151

0.0038167.2e-050.0010810.0005170.003307

0.000254

0.0018177.2e-050.0001740.0001220.001852

5.8e-05

0.0019990.0009076.3e-050.001455

2e-05

0.0008323.7e-050.0003730.0004090.001353

0.000743.7e-050.0003731.1e-050.00065

9.2e-050.0001740.000703

0.000224

0.0012962.8e-050.000410.0004240.001238

0.0012962.8e-050.000410.0004240.001238

0.0012788.3e-050.0002020.0001560.001235

0.0012788.3e-050.0002020.0001560.001235

2.3e-051.9e-052e-061.8e-05

2.3e-051.9e-052e-061.8e-05

0.0001750.000325

0.0001750.000325

0.0059310.0009260.0014340.0025390.0051220.000118

3.3e-050.0003063.9e-05

3.3e-050.0003063.9e-05

0.0035720.0002940.0005820.0005420.0034884.2e-05

0.0002310.0001062.8e-050.0001934.2e-05

9.2e-05

0.002490.0001340.0001951e-050.002467

0.0001430.00019.9e-05

8.1e-05

8.2e-05

5.1e-05

0.0007085.4e-050.0003879.8e-050.000729

0.0022930.0006320.0005620.0011530.001557.6e-05

0.0003520.000654

4e-050.0001734.7e-054.5e-05

0.0009227.8e-050.0001880.0002187.6e-05

0.0005130.0001461e-060.000498

1.3e-050.0001726.8e-053.5e-05

0.0008056.3e-050.000210.0001950.000754

3.3e-050.000290.0005384.5e-05

3.3e-050.000290.0005384.5e-05

0.0191920.0035450.0098080.0118790.0198860.000693

4.7e-050.0010220.0020560.000144

0.0002340.000434

8e-061e-051.7e-05

1e-05

0.0004460.000829

4e-060.0003420.0006353e-06

3.5e-050.0001380.000124

0.0006277.7e-050.0003934.7e-050.000930.000113

1.4e-05

0.0006092.2e-050.0003933.1e-050.000911

1.8e-055.5e-052e-061.9e-050.000113

2.6e-050.0002370.000443.1e-05

2.6e-050.0002370.000443.1e-05

4.2e-05

4.2e-05

0.0023980.0002430.0001810.0019840.000283

5.7e-05

1.5e-05

0.0005760.0001255.6e-050.000707

4.2e-05

0.0018220.0001184.2e-050.0012770.000113

2.6e-05

0.000113

8.3e-05

8.3e-05

0.000247

0.000247

0.0006950.00129

0.0006950.00129

8e-057e-057.9e-05

8e-057e-057.9e-05

0.0007480.0001330.0001622.3e-050.000917

0.0007480.0001330.0001622.3e-050.000917

0.0009340.0003676.5e-050.0012480.000226

6e-06

2.1e-05

0

3.2e-051e-063.2e-05

0.0001628.6e-051.8e-050.000376

0.0001020.0001718e-069.5e-05

0.0006380.000111.1e-050.0007450.000226

0.0016340.0008880.001665

0.0016340.0008880.001665

0.0009180.001705

0.0009180.001705

0.000104

0.000104

0.0003590.000668

0.0003590.000668

5.6e-05

5.6e-05

0.0012910.0007040.0004160.0001970.0013325.7e-05

9e-06

2.6e-05

0.000101

0.0008490.0001230.0001551.5e-050.000883.9e-05

2.5e-05

0.0004420.0005810.0002612.1e-050.0004521.8e-05

0

0.000217

0.00019

2.7e-05

0.000277

0.000277

0.0005190.000964

0.0005190.000964

0.00040.000743

0.00040.000743

0.0033680.0002590.0018680.0014340.003309

2.8e-050.0004120.0007652.1e-05

8.4e-050.0002190.0004089.7e-05

0.0012930.0001880.0001930.0002340.001273

0.0019637.1e-050.0010442.7e-050.001918

2.5e-052.5e-052.8e-051.7e-05

1.6e-051.9e-054e-068e-06

2e-066e-061e-062e-06

2e-062e-062e-06

5e-062.1e-055e-06

0.0055160.001270.0011270.0004290.004429

1e-06

0.0018650.0001680.0002281.3e-050.001753

0.0008380.0004740.0003794e-060.001049

0.0013270.0001890.0002128e-050.000626

0.0002860.0001027e-060.000305

4e-06

9.9e-05

1e-06

3e-06

0.00120.0003370.0003084e-060.000696

2e-06

0

0.000207

3e-06

1e-06

0.0009460.0002060.0005480.0001530.0029441.4e-05

6.4e-050.0001260.0001440.0017411.4e-05

0.0008828e-050.0005489e-060.001203

0.0015520.0002610.0002560.000360.000857

0.0015520.0002610.0002560.000360.000857

0.0003680.000683

0.0003680.000683

0.0003680.000683

0.0005540.00103

0.0005540.00103

0.0005540.00103

0.0030620.0005740.0007480.0005970.004768

0.0010220.0001440.0002620.001092

0.0008715.9e-050.000120.000951

1.7e-05

0.0001518.5e-050.0001250.000141

0.0011069.1e-050.0005890.0001860.001083

0.0011069.1e-050.0005892e-050.001083

1.3e-05

0.000153

0.0009340.0003390.0001590.0001490.002593

7e-060.0001141e-061.2e-05

6.9e-05

0.0008370.0001180.0001596.5e-050.001936

8.4e-050.0001071.3e-050.000638

6e-061e-067e-06

0.0010950.0002520.0002590.0010595.6e-05

7.2e-05

7.2e-05

1.5e-05

1.5e-05

0.0010950.0002520.0001720.0010595.6e-05

5e-06

6e-065.3e-056.8e-056e-063.7e-05

4e-06

0.0008768.2e-059.2e-050.0001871.9e-05

0.0002130.0001173e-060.000866

0.002990.0011340.0013030.0010450.0045846.6e-05

0.000247

0.000247

0.0015340.0003350.0004230.0001330.0014636.6e-05

0.0003270.0001431.6e-050.000301

0.0008085e-050.0004231.7e-050.000766

0.0001453e-058e-060.000148

0

4e-06

1e-0609e-06

0.0002530.0001128e-060.0002483.9e-05

3.5e-05

9e-06

4.4e-05

9e-06

1e-06

0.0006570.0005480.0002345e-060.001205

0.0006510.0002610.0002341e-060.000652

6e-060.0002874e-060.000553

0.0007990.0002510.0006460.000660.001916

2.8e-05

0.000221

0.0002098.8e-051e-060.000189

0.0001720.00032

0.000597.6e-050.0004744e-060.001727

08.7e-0500

0

1.1e-05

1.9e-05

5.6e-05

0.0009870.0003030.001530.0060130.000884

0.0007470.0001580.0004840.0024220.000609

0.0002020.000675

0.000136

0.0002020.000374

0.000165

0.000165e-050.0002820.0009130.0002

0.0001085e-050.0003880.000109

5.2e-050.0002820.0005259.1e-05

0.0001657.7e-050.0004530.00019

8.7e-05

0

01.8e-051e-06

1.8e-051.6e-059e-064.2e-05

7.2e-053.1e-053.3e-057.2e-05

5e-05

0.000115

1e-051.2e-055.1e-051.4e-05

5.2e-054e-065e-05

1e-067e-060

1.2e-059.6e-051.2e-05

6.8e-051e-059.1e-057.3e-05

6.8e-051e-059.1e-057.3e-05

0.0003542.1e-050.000290.000146

0

0.000184

6.1e-05

0.0003542.1e-054.5e-050.000146

0.000240.0001450.0010460.0035910.000275

0.00014.9e-050.0008390.000127

0.00014.9e-050.0008390.000127

0.000149.6e-050.0007190.000148

0.000149.6e-050.0007190.000148

0.0006460.00129

9e-05

0.0003930.00073

0.0002530.00047

0.00040.000743

0.00040.000743

0.0137760.0020680.0804180.150690.013322

0.0137760.0020680.0804180.150690.013322

0.0045250.0015470.0012730.001660.004522

0.0045250.0015470.0012730.001660.004522

0.0092510.0005210.0070970.0151950.0088

0.0048850.0007990.0006230.004745

0.0010950.002035

0.001350.0005210.0005450.0017980.001354

0.0028320.005261

0.0030160.0018260.0054780.002701

0.0064140.011915

0.0064140.011915

0.0033510.006225

0.0033510.006225

0.0031660.00588

0.0031660.00588

0.0591170.109815

0.0564090.104786

0.0027080.005029

0.0367920.0070750.0383030.0977390.035313

0.0036540.006788

0.0036540.006788

0.0036540.006788

0.0059250.0009160.0039470.008270.006822

0.0059250.0009160.0039470.008270.006822

4e-06

0.0029570.005493

0.0057830.0007640.000992.1e-050.006632

0.0001420.0001520.0027520.00019

0.0049240.0004810.0036070.0112850.002831

0.0049240.0004810.0036070.0112850.002831

0.0013440.002497

0.0049240.0004810.0007690.0060120.002831

0.0014940.002776

0.0024790.0001660.0003110.0081230.00267

0.0024790.0001660.0003110.0081230.00267

0.0024790.0001660.0003110.0081230.00267

0.0027380.0001360.0087140.0193150.00247

0.0005667.5e-050.0004080.0035190.000524

0.0005667.5e-050.0004080.0035190.000524

0.0002960.00055

0.0002960.00055

2.1e-055.8e-051.4e-05

2.1e-055.8e-051.4e-05

0.0002230.0001862.2e-05

0.0002230.0001862.2e-05

0.0019286.1e-050.0063530.0119230.00191

0.0019286.1e-050.0010630.0020980.00191

0.0014840.002756

0.0013850.002572

0.00130.002415

0.0011210.002082

0.0016570.003079

0.0016570.003079

0.0207260.0053760.018070.0439580.02052

0.0067650.012566

0.0067650.012566

0.0064620.0042890.0028530.0049860.006015

0.0064620.0042890.0028530.0049860.006015

0.0142640.0010870.0014540.0134060.014505

0.0052040.0001630.0003230.0058950.005613

0.0072920.0001790.0003530.0041730.007104

0.0017680.0007450.0007780.0033380.001788

0.0069980.013

0.0069980.013

0.0030291e-050.001690.0054310.002277

2.4e-050.0002743.7e-05

2.4e-050.0002743.7e-05

2.4e-050.0002743.7e-05

2.4e-050.0002743.7e-05

5e-060.0001929.6e-05

5e-060.0001929.6e-05

3e-060.000119.5e-05

3e-062.8e-059.5e-05

8.2e-05

2e-068.2e-051e-06

2e-068.2e-051e-06

0.0031e-050.001690.0049650.002144

0.000410.000310.000106

0.000410.000310.000106

0.000410.000310.000106

0.001080.0014990.0031730.000565

1.4e-050.0001567.1e-05

1.4e-050.0001567.1e-05

8e-050.0002170.0004027e-05

8e-050.0002170.0004027e-05

1.5e-050.0008210.0018754.4e-05

1.2e-050.0008210.0015241.4e-05

3e-060.0003513e-05

0.0009710.0004610.000740.00038

0.0001910.000355

3e-061.4e-05

0.0009430.000271.2e-050.000342

1.7e-05

2e-063e-061e-06

1e-061e-062e-06

4.9e-05

1e-055.1e-051e-05

6e-060.0001734e-06

4e-067.2e-054e-06

2e-067e-063e-06

0.001511e-050.0001910.0014820.001473

2.4e-050.0003872.6e-05

1e-061.1e-051e-06

2e-050.0002061.8e-05

3e-060.000177e-06

0.0001321e-050.0006977e-05

9e-06

0.000114

8e-063e-061e-05

3e-06

7.4e-05

2e-06

4e-06

4.2e-051.3e-054.2e-05

3e-060.000296

1.5e-05

1.2e-051e-051e-061.1e-05

1.2e-053.6e-056e-06

5.3e-055.7e-05

4.3e-05

2e-063e-061e-06

2.4e-05

0.0013540.0001910.0003980.001377

0.000119

1e-05

7.5e-05

2e-053.3e-054.2e-05

0.0013340.0001910.0001440.001335

1.7e-05

0.0075830.0006350.0049620.015480.007508

2e-065.7e-053e-06

2e-065.7e-053e-06

2e-065.7e-053e-06

2e-065.7e-053e-06

0.0020535e-050.0019910.004090.002531

0.0019425e-050.0013860.002270.002451

0.0008875e-050.0002850.0005380.000372

0.0008875e-050.0002850.0005380.000372

0.0008970.0004280.000650.000893

0.0008970.0004280.000650.000893

6e-050.0002430.0002840.00111

6e-050.0002430.0002840.00111

9.8e-050.000430.0007987.6e-05

9.8e-050.000430.0007987.6e-05

0.0001110.0006050.001828e-05

5.1e-050.0003930.000732.5e-05

5.1e-050.0003930.000732.5e-05

2.9e-050.000373.5e-05

2.9e-050.000373.5e-05

1.2e-050.0003261.4e-05

1.2e-050.0003261.4e-05

1.9e-050.0002120.0003946e-06

1.9e-050.0002120.0003946e-06

0.0003630.0001930.0006630.0028850.000306

0.0002850.0001670.0015750.000245

1.5e-054.9e-052.6e-058e-06

1.5e-054.9e-052.6e-058e-06

0.000270.0001180.0015490.000237

5e-050.0002334.1e-05

1.9e-052.8e-05

7e-061e-05

9e-060.0001371.2e-05

0.000242

7e-05

1e-050.0001151.1e-05

5.3e-053e-050.0002844.8e-05

3.8e-051.6e-05

4.6e-052.9e-050.0001723.6e-05

1.5e-055.9e-055.8e-051.6e-05

2.3e-050.0002381.9e-05

3.5e-050.0002880.0005353.4e-05

3.5e-050.0002880.0005353.4e-05

3.5e-050.0002880.0005353.4e-05

4.3e-052.6e-050.0003750.0007752.7e-05

4.3e-052.6e-050.0003750.0007752.7e-05

7.8e-05

4.3e-052.6e-050.0003750.0006972.7e-05

0.000497.4e-050.0018570.000481

6.1e-050.0002295.2e-05

6.1e-050.0002295.2e-05

6.1e-050.0002295.2e-05

3.1e-050.0001812.9e-05

3.1e-057.7e-052.9e-05

3.1e-057.7e-052.9e-05

0.000104

0.000104

0.00026

0.00026

0.00026

4.9e-059e-060.0001894.6e-05

8.3e-05

8.3e-05

4.9e-059e-060.0001064.6e-05

4.9e-059e-060.0001064.6e-05

0.0002234e-050.0004670.000225

0.0002234e-050.0004670.000225

2.6e-051.9e-050.0001423.9e-05

0.0001440.000210.000137

5.3e-052.1e-050.0001154.9e-05

0.0001262.5e-050.0002560.000129

2.9e-053.9e-053.8e-05

2.9e-053.9e-053.8e-05

9.7e-052.5e-050.0002179.1e-05

3.6e-059.3e-053.6e-05

1.8e-051.2e-054.7e-051.3e-05

1.9e-05

4.3e-051.3e-055.8e-054.2e-05

0.000275

0.000275

0.000275

0.0002350.0006537.4e-05

0.0002080.0004586.3e-05

0.000190.0001414.8e-05

0.000190.0001414.8e-05

1.4e-050.0002391.4e-05

1.4e-050.0002391.4e-05

4e-067.8e-051e-06

4e-067.8e-051e-06

2.7e-050.0001951.1e-05

2.7e-050.0001951.1e-05

2.7e-050.0001951.1e-05

0.0030610.0003180.0016470.0042410.002125

0.0018880.001120.0020110.001263

0.0006460.001201

0.0002990.000556

0.0003470.000645

0.0018880.0004740.000810.001263

0.0018660.0004740.0004830.001245

2.2e-050.0003271.8e-05

0.0011730.0003180.0005270.002230.000862

0.0011730.0003180.0012510.000862

1.5e-054.2e-051.4e-05

0.0008262.6e-050.0001320.000512

0.0001263.8e-050.000141

4.4e-050.0002925.2e-054.6e-05

3.4e-050.0002832.9e-05

1.2e-051.7e-051e-05

0.0001030.0003690.0001

5e-06

1.3e-055.5e-051e-05

0.000258

0.0005270.000979

0.0005270.000979

0.0012420.0003840.0008320.000851

0.0001920.000270.000173

0.0001920.000270.000173

1.6e-059.8e-051.8e-05

0.0001760.0001720.000155

0.001050.0003840.0005620.000678

0.001050.0003840.0005620.000678

0.001050.0003840.0005620.000678

2.4e-050.0002770.0006495.5e-05

2.4e-050.0002770.0006495.5e-05

0.0002770.000515

0.0002770.000515

2.4e-050.0001345.5e-05

2.4e-050.0001345.5e-05

0.0001130.0002160.001082

0.0001130.0002160.001082

0.0001130.0002160.001082

0.0001130.0002160.001082

0.0338260.0409650.0339030.0410.0647510.0375950.009655

0.0004080.0001010.0027780.0053990.000689

4.5e-050.0006120.0011384e-05

4.5e-050.0006120.0011384e-05

4.5e-050.0006120.0011384e-05

0.0001934.6e-050.0016510.0030790.000197

0.0001430.0008490.001590.000135

8e-06

6.5e-050.0004470.000836.4e-05

0.0004020.000747

7.8e-055e-067.1e-05

5e-054.6e-050.0008020.0014896.2e-05

5e-054.6e-050.0004370.0008126.2e-05

0.0003650.000677

0.000175.5e-050.0005150.0011820.000452

8.2e-050.0002510.0004677.9e-05

8.2e-050.0002510.0004677.9e-05

8.8e-055.5e-050.0002640.0007150.000373

8.8e-055.5e-050.0002640.0007150.000373

0.0047560.0001810.0033090.0061830.003658

0.0022070.0001270.0009860.0029160.00134

0.0007660.0004420.0010120.000735

0.0007660.0004420.0010120.000735

0.0006247.5e-050.0001750.0008190.000156

0.0006247.5e-050.0001750.0008190.000156

0.0008175.2e-050.0003690.0010850.000449

0.0008175.2e-050.0003690.0010850.000449

0.0025495.4e-050.0023230.0032670.002318

0.0002570.000478

0.0002570.000478

0.0025495.4e-050.0017090.0021270.002318

0.0003870.000719

0.0015180.0005180.000560.001479

0.0008450.0003561.5e-050.000542

0.0001865.4e-051e-060.000297

0.0004480.000832

0.0003570.000662

0.0003570.000662

0.0030720.0018050.0026770.002921

0.0030720.0018050.0026770.002921

2.4e-050.000140.000103

2.4e-050.000140.000103

0.0004260.0003060.0008588.8e-05

0.0004260.0003060.0008588.8e-05

0.0013950.0007612.5e-050.001432

0.0013950.0007612.5e-050.001432

0.0012270.0007380.0016540.001298

1.9e-050.00022.5e-05

7.5e-050.0005050.0009396.5e-05

5e-060.0001436e-06

0.0011280.0002330.0001920.001202

0.00018

0.0066420.0001690.0116080.0251830.009826

0.0010650.00198

0.0004620.000859

0.0004620.000859

0.0006030.001121

0.0006030.001121

0.0006870.0028510.0051970.001215

0.0006590.0023070.0041860.001125

2.9e-050.0004430.0008232e-05

4.6e-050.0004710.0008765.1e-05

9.2e-05

2.4e-050.0006260.0011633.4e-05

0.0005030.000934

0.000560.0002640.0002980.00102

2.8e-050.0005440.0010119e-05

0.0002070.000385

2.8e-050.0003370.0006269e-05

0.0043330.0001690.005310.0104630.005231

0.0028973.8e-050.0017270.0044880.002745

0.0014483.8e-050.0008970.0025520.001394

0.0014490.000830.0019360.001351

0.0007540.0014

0.0007540.0014

0.0015010.002787

0.0004040.00075

0.0010970.002037

0.0013713.6e-050.0007720.0016690.00131

0.0013713.6e-050.0007720.0016690.00131

6.5e-059.5e-050.0005560.0001190.001176

3e-054e-050.0001120.000144

3.5e-055.5e-050.0005567e-060.001032

6.5e-050.0012560.0023348.5e-05

6.5e-050.0012560.0023348.5e-05

6.5e-050.0012560.0023348.5e-05

0.0015570.0011260.0052090.003295

0.000970.0006090.0020880.000877

0.000970.0006090.0020880.000877

0.0004770.0005170.0016950.002285

0.0004770.0005170.0016950.002285

0.000110.0014260.000133

0.000110.0014260.000133

0.0189480.0405140.0144030.0410.0253090.0205010.009655

0.0048127.9e-050.0029590.0038070.006247

0.001764.5e-050.0013720.002090.001771

0.0002380.000443

0.0006090.001132

0.001764.5e-050.0005250.0005150.001771

9.4e-050.000199e-05

4.8e-05

8e-05

9.4e-056.2e-059e-05

0.0013450.0009020.0005650.001293

0.0013450.000717.3e-050.001293

0.000136

0.0001920.000356

0.0016133.4e-050.0005180.000460.003093

0.0016133.4e-050.0005180.000460.003093

0.0001670.000502

0.0001670.00031

0.000192

0.0004165.1e-050.0027130.0065740.000451

0.000145

0.000145

0.0001412.6e-050.0005140.000164

0.0001412.6e-050.0005140.000164

0.0002140.000397

0.0002140.000397

7.8e-050.000420.000788e-05

7.8e-050.000420.000788e-05

0.000130.0008770.000129

0.000130.0008770.000129

0.0002980.000553

0.0002980.000553

6.7e-052.5e-050.0006320.0011757.8e-05

3.7e-050.0006320.0011753.5e-05

3e-052.5e-054.3e-05

0.0011490.002133

0.0004330.000804

0.0004780.000888

0.0002380.000441

0.0043996.6e-050.0019150.0047320.004271

0.0014420.0003740.0003410.001438

0.0014420.0003740.0003410.001438

3e-056.6e-058.4e-052.7e-05

1.2e-05

2e-053.4e-055e-061.7e-05

1e-053.2e-056.7e-051e-05

3.3e-050.0001883e-05

3.3e-050.0001883e-05

6.8e-050.0002025.9e-05

6.8e-050.0002025.9e-05

0.000237

0.000206

3.1e-05

1.1e-050.0002325e-06

1.1e-050.0002325e-06

0.0014350.000570.0007790.001424

2.1e-055.4e-052.6e-05

0.000210.00039

0.0007030.0001020.000671

4e-060.0001064e-06

0.0006640.000364.3e-050.000674

4.3e-058.4e-054.9e-05

0.0011950.0004320.0005090.001146

0.0005880.0001810.0001850.000559

0.0006070.0002510.0003240.000587

6.5e-050.000194.7e-05

6.5e-050.000194.7e-05

7.6e-050.000315.6e-05

0.000119

7.6e-050.0001915.6e-05

0.000158

0.000158

0.0001850.00063

0.0001850.000344

0.000286

0.0003540.000658

0.000180.000335

0.0001740.000323

4.4e-050.0002143.9e-05

4.4e-050.0002143.9e-05

0.0066680.0402580.004310.0410.0053540.0063790.009655

0.002890.0401510.0030780.0410.001210.0026880.009655

0.0001593e-060.0001684.8e-05

8e-06

0

6e-06

0.0013740.0400660.0016260.0414.3e-050.0013510.007296

7e-06

3.4e-05

1.3e-05

7e-061e-063.2e-05

1.2e-052.2e-051.9e-054e-05

2.3e-05

2.5e-054.4e-052.4e-05

4e-06

2.3e-05

8e-06

1.9e-051.2e-058e-061.3e-05

7.9e-055.8e-057e-057e-06

1.2e-05

2.4e-053.9e-050.001247e-062.4e-050.002304

3.4e-050.0001782.3e-05

2.7e-05

7.9e-05

5.1e-05

0

7e-06

7e-061.9e-052e-06

2.9e-052.1e-051.4e-05

4e-058.5e-053.4e-05

0.000490.0002123.3e-050.000328

0.0002551.7e-050.000239

4.2e-058e-063.7e-05

5e-06

5e-06

2.9e-051e-052.6e-05

0.0002651.2e-051e-050.000263

0.000144

6.3e-05

0.000127

0.0003583e-050.0002740.000308

7.3e-05

0.0002261.8e-059.7e-050.000219

4.9e-05

0.0001321.2e-055.5e-058.9e-05

0.0001780.000360.0011360.000145

5.4e-05

9e-06

4.6e-05

0.0001781.1e-050.000145

0.000211

3e-05

1.1e-05

0.000360.000669

9.5e-05

0.0024732.8e-050.0006940.0012040.002411

6.6e-050.0003427.4e-05

0.0024072.8e-050.0003960.0003090.002337

0.0002980.000553

0.0006376e-060.0001780.0011120.000654

0.000276

2.6e-056e-065.5e-052.5e-05

0.0004960.0001780.0002010.000486

5.6e-050.0003585.7e-05

5.9e-058.6e-05

0.000222

0.0001324.3e-050.0004180.000173

2.6e-051.6e-051.9e-05

3e-053.2e-052.4e-05

2.9e-050.0002047.6e-05

2.8e-05

3.6e-052.9e-054.6e-05

0.000101

1.1e-054.3e-058e-068e-06

0.0026536e-050.0025060.0048420.003153

4.8e-050.0001683e-05

4.8e-050.0001073e-05

6.1e-05

0.0002380.000443

0.0002380.000443

7.1e-055.4e-050.0002930.0005530.000146

7.1e-055.4e-051e-050.000146

0.0002930.000543

6.2e-050.0005450.0010134.3e-05

2e-050.0002180.0004051.8e-05

4.2e-050.0003270.0006082.5e-05

0.0016440.0004930.0004980.00152

0.0016440.0004930.0004980.00152

0.0002790.000518

0.0002790.000518

0.0008286e-060.0003370.0010540.001414

0.000133

1.5e-05

3e-05

4e-0603e-06

0.000205

2.7e-050.0001358.7e-05

2.2e-05

0.000170.000316

0.0007920.0001670.000130.001319

3e-066e-069e-063e-06

5.8e-05

2e-061e-062e-06

0.0003210.000595

0.0003210.000595

0.0301430.0263780.0211360.15440.0558310.031740.0055

0.0016956.1e-050.001350.0070310.002029

0.00094e-050.0011240.0060260.000986

0.0004158e-060.000430.000443

0.0001040.0001070.000161

7e-06

0.0003118e-060.0001250.000282

0.000191

0.0005030.000935

0.0005030.000935

0.0002230.000414

0.0002230.000414

3.2e-053.2e-050.0020370.000146

3.2e-053.2e-050.0020370.000146

0.0001230.0006010.000105

9.4e-050.000298.2e-05

2.9e-050.0003112.3e-05

0.000120.0003350.000121

0.000120.0003350.000121

0.0001880.00035

0.0001880.00035

2e-056.8e-051.5e-05

2e-056.8e-051.5e-05

0.000190.000210.0008560.000156

7.7e-050.000210.000397.7e-05

0.000129

0.0001130.0003377.9e-05

0.0007952.1e-050.0002260.0010050.001043

3.4e-051.4e-050.0001043.4e-05

3.4e-051.4e-050.0001043.4e-05

6.3e-050.0001370.000741

6.3e-050.0001370.000741

0.0005566.8e-050.000133

3.4e-05

0.0005563.4e-050.000133

2.9e-057.7e-052.7e-05

2.9e-057.7e-052.7e-05

0.0002260.000421

0.0002260.000421

2.7e-050.0001421.8e-05

7e-060.0001295e-06

2e-051.3e-051.3e-05

8.6e-057e-065.6e-059e-05

8.6e-057e-061.2e-059e-05

3.4e-05

1e-05

0.0058250.0025660.0063810.0176730.0069140.000425

0.0013440.0005220.001480.001291

0.0007170.0002140.0002110.000709

0.0007170.0002140.0002110.000709

0.0003080.000571

0.0003080.000571

0.0006270.0005720.000582

0.000174

3.5e-054.7e-053.4e-05

0.0004960.0001270.000445

3.1e-056.7e-053.3e-05

6.5e-050.0001577e-05

0.000126

0.000126

1.6e-055.4e-050.0002561.9e-05

03.1e-050.0001230

01.1e-050

0.000121

1e-06

01.1e-0500

0

0

1e-06

0

9e-060

1.6e-052.3e-050.0001331.9e-05

0.000108

1.6e-052.3e-052.5e-051.9e-05

0.0003391.6e-050.0007890.000369

0.0003391.6e-050.0007890.000369

3.6e-051.6e-051e-053.6e-05

4.8e-050.0001815e-05

0.000155

3.8e-05

9.2e-050.0001999.2e-05

0.0001058.7e-050.000138

9e-05

5.8e-052.9e-055.3e-05

3.8e-050.000330.0006143.8e-05

3.8e-050.000330.0006143.8e-05

3.8e-050.000330.0006143.8e-05

0.0001180.0005690.0003560.001181

0.0001180.0005690.0003560.001181

6e-062.6e-051.2e-05

2.9e-051.7e-054.7e-05

3e-066e-065e-06

2e-056e-062.4e-05

1e-063.3e-052e-06

1.3e-056e-061.2e-05

2.6e-050.0005691.1e-050.001057

2e-050.0002242.2e-05

2.7e-05

0.0003598.4e-050.0015790.0037850.000336

0.000210.000467

0.000210.000391

7.6e-05

0.000125

0.000125

0.0001880.000349

0.0001880.000349

0.0001560.0008440.0017480.000149

7.2e-050.000187.1e-05

4.6e-050.0002870.0005334.6e-05

3.8e-050.0005570.0010353.2e-05

5.1e-050.0003370.0006254.4e-05

5.1e-050.0003370.0006254.4e-05

0.0001528.4e-050.0001960.000143

0.0001528.4e-050.0001960.000143

0.000275

0.000275

0.0007387.8e-050.0014650.000717

0.0001330.0003450.000128

5.6e-051.5e-055e-05

2e-056.8e-052.4e-05

2e-050.000111.8e-05

3.7e-051.8e-053.6e-05

0.000134

0.0001090.0004180.000118

0.000217

2e-06

6.7e-05

8e-06

2e-053.9e-052.2e-05

7e-06

3.6e-054.5e-053.9e-05

2.7e-053e-063e-05

2.6e-053e-052.7e-05

3.9e-05

3.9e-05

9.1e-052e-060.0001868.4e-05

9.1e-05

3.3e-053.1e-052.9e-05

6e-062e-062.3e-056e-06

5.2e-054.1e-054.9e-05

5.6e-05

5.6e-05

8.8e-05

8.8e-05

0.0004057.6e-050.0003330.000387

4.5e-05

5.4e-050.0001885e-05

5.1e-053e-054.5e-05

4e-056.5e-054.8e-05

0.000267.6e-055e-060.000244

0.000607

0.000101

0.000101

0.000506

0.000224

0.000282

0.002060.003826

0.002060.003826

0.002060.003826

0.0024180.0023340.0008620.0019330.0025130.000425

0.000655

0.000191

0.000282

0.000182

0.0017720.0021820.0008620.0008730.0019090.0002

0.0002360.0001670.0001010.0002445.7e-05

4.5e-05

0.0002130.000396

5.1e-05

0.0003240.0005170.0002066.7e-050.000353

0.0003420.0005480.0002173.7e-050.0003728.7e-05

0.0002420.0001833.5e-050.000257

0.0002720.0001927.7e-050.0002985.6e-05

0.0003560.0005750.0002266.4e-050.000385

0.0001892.4e-058.7e-050.000195

0.0001892.4e-058.7e-050.000195

0.000253.5e-050.0001580.000222

0.0001327.5e-050.000114

0.0001183.5e-058.3e-050.000108

0.0002079.3e-050.000160.0001870.000225

0.0002079.3e-050.000160.0001870.000225

3.5e-050.0004590.0008534e-05

3.5e-050.0004590.0008534e-05

3.5e-050.0004590.0008534e-05

0.0003630.0010410.000349

0.0001459.9e-050.000141

0.0001459.9e-050.000141

0.0001160.0007470.000106

0.0001160.0007470.000106

0.0001020.0001950.000102

0.0001020.0001950.000102

5.7e-050.0006686.1e-05

4.1e-050.0003644.3e-05

4.1e-050.0003644.3e-05

1.6e-050.000121.8e-05

1.6e-050.000121.8e-05

0.000184

0.000184

0.0113970.0105190.0057620.12740.0123070.0126440.00139

0.0005390.0003990.0004490.05250.0019180.000485

0.000417

0.000139

0.000278

0.0005390.0003990.0002790.05250.0004170.000485

0.0005390.0003990.0002790.05250.000310.000485

0.000107

0.000148

0.000148

0.000170.000936

0.00026

7.2e-05

8.1e-05

5.4e-05

0.000170.000317

0.000152

0.0108580.010120.0053130.07490.0103890.0121590.00139

0.000142

0.000142

0.0002520.000469

0.0002520.000469

0.0001890.000351

0.0001890.000351

0.0026070.0015050.0017690.02780.0033980.0036110.00023

0.0001580.000294

0.000113

0.000246

0.0026070.0015050.0002940.02780.0001890.0036110.00023

0.00011

0.0013170.002446

0.0003144.1e-050.0001710.0003030.000338

0.0003144.1e-050.0001710.0003030.000338

0.0019820.0011240.0008620.04710.001510.0019860.000266

6e-06

0.000225

0.0001870.000347

0.00026

0.0011230.000650.0003760.04710.0002630.001124

0.000155

0.0008590.0004740.0002990.0003630.000862

0.000157

0.0005820.0001340.000730.0005955.8e-05

0.000264

0.0003147e-050.0001560.0002925.8e-05

7.9e-05

0.0002686.4e-050.0002310.000303

0.0053730.0073160.002070.0034860.0056290.000836

0.000258

0.0001660.000308

0.0021630.002780.0002570.000190.002260.000253

0.0002160.000402

0.0026260.0032390.0002720.000140.002770.000583

0.0006470.001202

0.0002670.000496

0.000275

0.0005840.0012970.0002450.0002150.000599

0.0003830.0007480.002520.000374

0.0003830.0007480.002520.000374

0.0002510.0004020.0011970.000246

0.0001960.000450.000196

5.5e-050.0004020.0007475e-05

0.0001320.0003460.0013230.000128

2e-050.0003460.0006432.8e-05

0.0001120.000680.0001

0.0057510.0125530.0048840.0270.0097750.0055740.003602

0.0001420.0001130.000630.0015760.000115

5.8e-050.0001130.0004075.2e-05

5.8e-050.0001130.0004075.2e-05

4.2e-050.000350.0006493.2e-05

4.2e-050.000350.0006493.2e-05

4.2e-050.000280.000523.1e-05

4.2e-050.000280.000523.1e-05

7.4e-05

7.4e-05

7.4e-05

0.0056090.012440.0042540.0270.0081990.0054590.003528

3.2e-050.0002433e-05

3.2e-050.0002433e-05

0.0001970.000468

0.0001970.000366

0.000102

0.000168

0.000168

7.3e-05

7.3e-05

0.0001480.0002710.0008470.000145

7.5e-050.0002710.0005037.3e-05

7.3e-050.0003447.2e-05

0.000840.0003660.0004980.00082

0.000840.0003660.0004980.00082

0.0002290.000425

0.0002290.000425

0.0002610.000484

0.0002610.000484

0.000197

0.000197

0.000169

0.000169

0.0041690.0121420.0014880.0270.0008620.0039910.003248

0.001020.0001950.0001590.000973

0.0031490.0121420.0012930.0270.0007030.0030180.003248

3.6e-051.1e-050.0001462.9e-05

1.8e-051e-051.3e-05

1.8e-051.1e-050.0001361.6e-05

0.000202

0.000202

1.9e-053.9e-051.5e-05

1.9e-053.9e-051.5e-05

6.6e-05

6.6e-05

0.0002220.000413

0.0002220.000413

0.0001940.00036

0.0001940.00036

0.000147

0.000147

0.000107

0.000107

4e-050.0005130.0009533.1e-05

4e-050.0005130.0009533.1e-05

5.1e-050.0004029.7e-05

6e-060.0001245.2e-05

4.5e-050.000114.5e-05

0.000168

0.0002324.4e-050.0002350.0006230.00026

0.0001878.9e-050.000216

4.5e-054.4e-050.0002350.0004374.4e-05

9.7e-05

2.2e-050.0001321.9e-05

2.2e-050.0001321.9e-05

2e-050.0002780.0005572.2e-050.000141

7e-064e-057e-067.1e-05

1.3e-050.0002780.0005171.5e-057e-05

0.0007830.0004460.0009470.000779

0.0007830.0004460.0009470.000779

0.0007830.0004460.0009470.000779

0.0007830.0004460.0009470.000779

0.0002370.0002170.0007620.000272

0.0002170.000402

0.0002170.000402

0.0002170.000402

9.9e-050.0002310.000135

5.9e-054.2e-054.5e-05

2.2e-051e-069e-06

3e-06

2e-06

3.6e-053.4e-05

0

1e-063e-052e-06

6e-06

1.6e-050.0001131.5e-05

6e-06

9.2e-05

1.6e-051.5e-051.5e-05

2.2e-051.6e-057.3e-05

2e-06

7e-063e-061.8e-05

3e-06

2e-061e-062e-06

1.3e-057e-065.3e-05

2e-066e-052e-06

1e-063e-06

1e-063.8e-052e-06

1.9e-05

1.5e-055.2e-051.1e-05

1.5e-055.2e-051.1e-05

1.5e-055.2e-051.1e-05

0.0001237.7e-050.000126

0.0001026.9e-050.000107

1e-06

1.1e-051e-058e-06

3e-06

6e-062e-068e-06

04e-06

1e-063e-061e-06

9e-06

1e-061e-06

6e-0601.1e-05

2.7e-05

3e-067e-061e-06

1e-061e-06

1e-060

0

0

6.4e-052e-066.2e-05

2e-06

9e-0609e-06

1e-061e-061e-06

2.1e-058e-061.9e-05

2.1e-058e-061.9e-05

0.0022510.0004990.0008110.001958.3e-05

0.0022510.0004990.0008110.001958.3e-05

9e-050.0001259.4e-058.3e-05

9e-050.0001259.4e-058.3e-05

0.0014810.0004990.0005390.001418

0.0014810.0004990.0005390.001418

0.000680.0001470.000438

0.000680.0001470.000438

0.000531

0.000285

0.000285

0.000285

0.000246

0.000246

0.000246

0.0014410.0006790.0005530.0024770.001126

0.0014410.0006790.0005530.0024770.001126

9.9e-057.8e-050.0008390.000111

9.3e-05

0.000243

9.9e-057.8e-050.0004350.000111

6.8e-05

0.0003290.000611

0.0003290.000611

0.0009190.0004260.0002240.0002240.00058

0.0009190.0004260.0002240.0002240.00058

0.0002730.0001520.0003920.000286

0.000176

0.0001063.5e-056.5e-050.000111

0.0001124.6e-056.5e-050.000125

5.5e-057.1e-058.6e-055e-05

0.000152.3e-050.0004110.000149

0.000152.3e-050.0004110.000149

0.000380.0002960.0009977.8e-05

0.000380.0002960.0009977.8e-05

0.000380.0002960.0009977.8e-05

0.000380.0002960.0009977.8e-05

1.3e-050.0003872e-05

1.3e-050.0003872e-05

1.3e-050.0003872e-05

7e-060.0001251.2e-05

7e-060.0001251.2e-05

7e-060.0001251.2e-05

6e-060.0002628e-06

6e-060.0002628e-06

6e-060.0002628e-06

0.0001740.000323

0.0001740.000323

0.0001740.000323

0.0001740.000323

0.0001740.000323

0.0001740.000323

0.0022740.0002260.0016960.001941

0.0022740.0002260.0016960.001941

0.0022740.0002260.0016960.001941

0.0022740.0002260.0016960.001941

0.0018120.0002260.0005630.001717

5.7e-050.0003744.5e-05

2.2e-05

0.0017550.0002260.0001670.001672

3e-050.0002453.8e-05

0.000206

3e-053.9e-053.8e-05

1.2e-050.0001931.3e-05

1.2e-050.0001931.3e-05

7.1e-050.0002297.5e-05

1e-050.0001062.3e-05

6.1e-050.0001235.2e-05

0.0003490.0004669.8e-05

0.0002960.0001416e-05

2.6e-050.0001091.6e-05

2e-060.0001672e-06

2.5e-054.9e-052e-05
