# Supplementary material for: MetaMeta: integrating metagenome analysis tools to improve taxonomic profiling
Source: Microbiome. 2017 Aug 14;5:101. doi: 10.1186/s40168-017-0318-y (PMC5557516; doi:10.1186/s40168-017-0318-y)
Supplement: Supplementary file 2 — Additional File with interactive charts for all CAMI toy set results on default, very-precise and very-sensitive mode. File prefix S, M, and H for low, medium and high complexity, respectively. (TAR 3573 kb) [file 40168_2017_318_MOESM2_ESM.tar › S_S001__genomes_30__insert_180_very-sensitive.html]

Javascript must be enabled to view this page.

magnitude
magnitudeUnassigned

clark.parsed\_profile
dudes.parsed\_profile
final.metametamerge.profile
gottcha.parsed\_profile
kaiju.parsed\_profile
kraken.parsed\_profile
motus.parsed\_profile

0.9999911.0000091.00000610.9999939999999980.9999910.999999

0.0112910.0094250.0064180.0168190.011472

1e-060.0001180.00023

1e-060.0001180.00023

1e-060.0001180.00023

1e-060.0001180.00023

1e-060.0001180.00023

1e-060.0001180.00023

0.0001120.0001720.000105

0.0001120.0001720.000105

0.0001120.0001720.000105

0.0001120.0001720.000105

0.0001120.0001720.000105

0.0001120.0001720.000105

2.1e-050.0001970.001486.2e-05

2.1e-050.0001970.001486.2e-05

2e-060.0001110.0004793e-06

0.0001110.0002631e-06

2.1e-051e-06

2.1e-051e-06

0.0001110.000216

0.0001110.000216

2.6e-05

2.6e-05

2e-060.0002162e-06

1e-065.7e-051e-06

1e-065.7e-051e-06

2.8e-05

1.4e-05

1.4e-05

1e-067e-051e-06

1e-067e-051e-06

2.3e-05

2.3e-05

3.8e-05

1e-05

2.8e-05

1e-060.000321e-06

1e-060.000321e-06

00.0001440

2.7e-05

07e-060

2e-06

6.7e-05

4.1e-05

1e-068.7e-051e-06

1e-068.7e-051e-06

8.9e-05

2.9e-05

6e-05

1.8e-058.6e-050.0002632e-05

8.6e-050.0001681e-06

8.6e-050.0001681e-06

8.6e-050.0001681e-06

1.8e-059.5e-051.9e-05

1.8e-059.5e-051.9e-05

1.8e-059.5e-051.9e-05

0.0004183.8e-05

0.000323.8e-05

6.8e-05

6.8e-05

9.8e-052.3e-05

4.2e-052.3e-05

5.6e-05

3.6e-057e-06

3.6e-057e-06

0.0001188e-06

6.4e-058e-06

5.4e-05

9.8e-05

9.8e-05

6.1e-05

1e-06

3.2e-05

4e-06

4e-060.0001940.00055e-06

4e-060.0001940.00055e-06

4e-064e-055e-06

4e-064e-055e-06

4e-064e-055e-06

8e-06

4e-063.2e-055e-06

0.0001940.00046

0.0001940.00046

0.0001940.000379

0.0001940.000379

8.1e-05

8.1e-05

0.0111530.0094250.0059090.0144370.0113

0.0088630.0084440.0038810.0062990.008807

5.2e-050.0001070.0004274.6e-05

5.2e-050.0001070.0004274.6e-05

0.000148

0.000148

7e-067e-051e-06

7e-067e-051e-06

4.5e-050.0001070.0002094.5e-05

4.5e-050.0001070.0002094.5e-05

9.8e-05

9.8e-05

9.8e-05

9.8e-05

0.0088110.0084440.0037740.0057740.008761

0.0003010.0001870.0001680.0008990.000416

0.0003010.0001870.0001680.0008990.000416

0.0003010.0001870.0001680.0008990.000416

0.0084950.0081760.0032450.0040530.008319

0.0084950.0081760.0032450.0040530.008319

0.0012810.0010550.0006080.0012430.001202

0.0072140.0071210.0026370.002810.007117

2e-068.1e-050.0003610.0007062e-06

2e-068.1e-050.0003610.0007062e-06

2e-068.1e-050.0003610.0007062e-06

1.3e-050.0001162.4e-05

1.3e-050.0001162.4e-05

1.3e-050.0001162.4e-05

6.1e-050.000160.0005160.0010110.00012

6.1e-050.000160.0005160.0010110.00012

6.1e-050.000160.0005160.0010110.00012

6.1e-050.000160.0005160.0010110.00012

6.1e-057.5e-050.0002790.0005476.2e-05

8.5e-050.0002370.0004645.8e-05

6.2e-050.0003744.9e-05

6.2e-050.0003744.9e-05

6.2e-050.0003744.9e-05

3.5e-05

1.9e-05

1.6e-05

2.8e-054.8e-051.6e-05

2.8e-054.8e-051.6e-05

3.4e-050.0002913.3e-05

3.3e-050.000133.2e-05

6.5e-05

1e-066.6e-051e-06

3e-05

0.0002128.1e-050.0004810.000239

0.0002128.1e-050.0004810.000239

0.0002028.1e-050.000410.000226

0.0002018.1e-050.000380.000201

2.7e-058e-062.7e-05

0.0001298.1e-050.0003080.000128

3.9e-054.7e-054e-05

6e-061.7e-056e-06

1e-063e-052.5e-05

1e-063e-052.5e-05

1e-057.1e-051.3e-05

5e-062e-056e-06

5e-062e-056e-06

5e-065.1e-057e-06

3e-06

1e-06

4e-06

1e-062e-061e-06

4e-063.9e-055e-06

2e-061e-06

0.0002150.0007320.0024450.000207

0.0001820.0001230.0006260.000142

8.6e-050.0001575.7e-05

8.2e-050.0001135.3e-05

6.4e-053.4e-051.4e-05

1.8e-057.4e-053.9e-05

5e-06

4e-064.4e-054e-06

4e-064.4e-054e-06

6.8e-050.0001555e-05

1e-061.9e-050

1e-061.9e-050

6.7e-050.0001365e-05

4.4e-052.4e-052.9e-05

2.3e-050.0001122.1e-05

0.0001230.000242

0.0001230.000242

0.0001230.000242

2.8e-057.2e-053.5e-05

2.8e-057.2e-053.5e-05

2.8e-057.2e-053.5e-05

2.8e-050.0006090.0017235.1e-05

4e-060.0004820.0014042.4e-05

0.0001420.000318

4.1e-05

0.0001420.000277

2.6e-05

2.6e-05

0.000102

0.000102

00.0001652e-05

1.5e-05

8e-05

1e-06

1e-05

7e-06

03e-061.9e-05

1e-06

8e-061e-06

2.5e-05

2e-06

01.2e-050

1e-06

3e-067.7e-053e-06

3e-067.7e-053e-06

1e-060.000340.0006651e-06

1e-060.000340.0006651e-06

5.1e-05

5.1e-05

2.4e-050.0001270.0003192.7e-05

2.4e-050.0001270.0003192.7e-05

2.3e-057.1e-052.6e-05

1e-060.0001270.0002481e-06

5e-069.6e-051.4e-05

5e-069.6e-051.4e-05

5e-069.6e-051.4e-05

4e-066e-051.2e-05

1e-063.6e-052e-06

2.2e-050.0003010.0009923.2e-05

2.2e-050.0003010.0009923.2e-05

2e-058.4e-050.0005673e-05

1.6e-050.0001862.6e-05

6e-061.9e-051.7e-05

1e-058e-059e-06

5e-06

8.2e-05

2.8e-05

2.8e-05

2e-063.7e-052e-06

7e-06

1.3e-051e-06

2e-061.7e-051e-06

2e-068.4e-050.0003162e-06

2.6e-05

1e-063.2e-051e-06

3.8e-05

8.4e-050.000164

2.9e-05

1e-062.7e-051e-06

2e-060.0002170.0004252e-06

2e-060.0002170.0004252e-06

2e-060.0002170.0004252e-06

0.0016480.0008210.0003980.0024920.001725

0.0003580.0002560.0007770.000415

0.0001120.0001640.0004350.000139

0.0001120.0001640.0004350.000139

0.0001120.0001640.0004350.000139

0.0002469.2e-050.0003420.000276

7.1e-053.2e-050.0001087.6e-05

7.1e-053.2e-050.0001087.6e-05

5.4e-053.4e-050.0001137.3e-05

5.4e-053.4e-050.0001137.3e-05

0.0001212.6e-050.0001210.000127

2.2e-05

6.6e-053.9e-057.4e-05

5.5e-052.6e-056e-055.3e-05

0.0005320.0001037.7e-050.0006530.000533

0.0005320.0001037.7e-050.0006530.000533

7.1e-054e-055.5e-05

7.1e-054e-055.5e-05

4.5e-05

4.5e-05

4.6e-053.6e-055.9e-055.9e-05

4.6e-053.6e-055.9e-055.9e-05

7.6e-053.6e-057.7e-050.000158.2e-05

7.6e-053.6e-057.7e-050.000158.2e-05

7.8e-050.0001416.5e-05

7.8e-050.0001416.5e-05

7.4e-054.9e-058e-05

7.4e-054.9e-058e-05

0.0001483.1e-057.9e-050.000144

6.4e-053.9e-056.3e-05

8.4e-053.1e-054e-058.1e-05

3.9e-059e-054.8e-05

3.9e-059e-054.8e-05

0.0007580.0004620.0003210.0010620.000777

0.0006460.0003980.0001940.0005440.000656

0.0001139.5e-050.000125

5e-055.3e-056.3e-05

6.3e-054.2e-056.2e-05

0.0003910.0003030.0001940.0003230.000406

0.0002160.0002450.0001070.0001720.000218

0.0001755.8e-058.7e-050.0001510.000188

7.1e-054.1e-058.5e-057.3e-05

7.1e-054.1e-058.5e-057.3e-05

7.1e-055.4e-054.1e-055.2e-05

1e-05

3.1e-051.5e-052e-062.1e-05

4e-053.9e-052.9e-053.1e-05

5.8e-05

5.8e-05

5.8e-05

0.0001126.4e-050.0001270.000460.000121

5e-053.2e-050.0001270.0002495.7e-05

5e-053.2e-050.0001270.0002495.7e-05

6.2e-053.2e-050.0002116.4e-05

6.2e-053.2e-054.5e-056.4e-05

8.6e-05

8e-05

7e-050.0003430.000121

7e-050.0003430.000121

7e-050.0003430.000121

6.8e-050.0002040.000112

1.2e-052e-06

1.5e-05

1.2e-05

4e-06

8e-064.2e-05

1.1e-05

4e-06

7e-06

4e-06

5.7e-052.1e-055.7e-05

1e-05

9e-062.1e-059e-06

2.7e-05

7e-06

1e-068e-061e-06

1e-061.4e-051e-06

1.9e-05

2e-060.0001179e-06

1.9e-05

3.1e-05

7e-06

2e-062.3e-053e-06

1.8e-05

1.2e-05

7e-066e-06

2.2e-05

2.2e-05

0.98870.9905839999999990.99358800000000110.9831749999999980.9885190.999999

0.0014560.0006450.001870.001334

0.0014560.000450.0014890.001334

0.0005160.0002740.0007080.000475

0.0005160.0002740.0007080.000475

1.8e-054.3e-051e-05

1.2e-051.1e-054e-06

2e-06

3e-061.6e-055e-06

3e-061.4e-051e-06

0.0004980.0002740.0006650.000465

0.0003060.0001510.0002760.000305

6.4e-05

9.8e-050.0001230.000247.4e-05

9.4e-058.5e-058.6e-05

0.0002620.0001020.0004270.000206

2.3e-051.2e-051.3e-05

2.3e-051.2e-051.3e-05

2.3e-051.2e-051.3e-05

0.0002390.0001020.0004150.000193

0.0002390.0001020.0004150.000193

8.2e-052.6e-054.3e-05

1e-054e-061e-06

0.000145e-060.00014

2.8e-05

1.1e-05

1.7e-05

2e-061.3e-057e-06

5e-069e-062e-06

8.7e-05

0.0001020.0002

1.5e-05

0.0006787.4e-050.0003540.000653

0.0006787.4e-050.0003540.000653

0.0006730.0003480.000508

2e-063e-062e-06

0.0001015e-064.6e-05

9e-06

1.1e-05

1e-061e-06

1.9e-05

0.0001052.2e-050.000107

5.2e-054.1e-052.6e-05

3e-06

7e-06

4.2e-055e-058e-06

2.7e-05

2.4e-053e-062e-05

2e-061.1e-051e-06

5e-062e-063e-06

2e-06

0.00013500.000121

2e-06

1.2e-05

1e-0600

5e-062e-064e-06

1.1e-05

2e-060

2.1e-05

3e-06

1e-066e-061e-06

4e-060

7.7e-051.5e-056.1e-05

1.9e-05

1e-06

9e-062.3e-051.5e-05

4.7e-056e-064.7e-05

2.8e-054e-063e-05

1e-062e-06

3.6e-051.4e-05

5e-067.4e-056e-060.000145

1e-061e-06

4e-067.4e-055e-060.000145

0.0001950.000381

0.0001950.000381

0.0001950.000381

0.0001950.000381

0.0001950.000381

1.3e-050.0001340.0003872e-05

1.3e-050.0001340.0003872e-05

1.3e-050.0001340.0003872e-05

7e-060.0001251.2e-05

7e-060.0001251.2e-05

7e-060.0001251.2e-05

6e-060.0001340.0002628e-06

6e-060.0001340.0002628e-06

6e-060.0001340.0002628e-06

0.0008090.0007280.0014050.000338

0.0008090.0007280.0014050.000338

0.0008090.0007280.0014050.000338

0.0008090.0007280.0014050.000338

0.0007810.0004210.0007930.000301

0.0007810.0001460.000170.000301

0.0002750.000537

8.6e-05

1.7e-058.2e-050.0001711.5e-05

9e-066e-066e-06

8e-068.2e-050.0001619e-06

4e-06

1.1e-050.0002250.0004412.2e-05

1.1e-050.0002250.0004412.2e-05

0.0266330.0051850.0091710.0267360.0270560.001062

0.0180230.0030970.0069290.0191480.0184380.000334

8.9e-051.4e-056.5e-059.7e-05

8.9e-051.4e-056.5e-059.7e-05

8.9e-051.4e-056.5e-059.7e-05

8.9e-051.4e-056.5e-059.7e-05

0.0006438.9e-059.3e-050.0006010.0006645.7e-05

0.0001949.3e-050.0001520.000208

0.0001949.3e-050.0001520.000208

0.0001949.3e-050.0001520.000208

0.0003097.3e-050.0003360.0003055.7e-05

0.0002327.3e-050.0002310.000239

0.0001053.6e-050.0001040.000111

0.0001273.7e-050.0001270.000128

7.7e-050.0001056.6e-055.7e-05

7.7e-050.0001056.6e-055.7e-05

0.000141.6e-050.0001130.000151

0.000141.6e-050.0001130.000151

0.000141.6e-050.0001130.000151

0.0007534.6e-050.0002630.0006470.000804

0.0007534.6e-050.0002630.0006470.000804

0.0007534.6e-050.0002630.0006470.000804

0.0002073e-059.6e-050.0001440.000242

0.0001340.0001120.000153

7.5e-056.6e-057.1e-05

0.0001831.6e-058e-050.0001270.000173

0.0001548.7e-050.0001980.000165

0.0016170.0006420.000390.0019280.0017410.00011

0.0016170.0006420.000390.0019280.0017410.00011

0.000142.6e-057.5e-057.8e-050.000156

0.000142.6e-057.5e-057.8e-050.000156

0.0014770.0006160.0003150.001850.0015850.00011

1.7e-053e-051e-051.7e-05

2.5e-05

7.5e-05

9.8e-052.9e-053.7e-05

7.4e-05

3.4e-05

6.1e-05

4.1e-05

5e-06

3e-05

0.0001042.9e-050.000118

3.5e-05

0.000153

9e-05

0.0001032.1e-057.8e-050.000115

5.5e-05

0.0001575.5e-058.1e-052e-050.000165.5e-05

0.0001073.8e-054.8e-050.00014

2.5e-05

0.0001443.8e-057.8e-052.1e-050.00016

2.1e-05

5.8e-05

0.0001373.2e-057.7e-057e-060.000166

9.4e-055.3e-054.5e-050.000123

2.9e-05

7e-052.5e-053.3e-057.9e-05

2.7e-05

0.0001514.3e-057.9e-053.2e-050.000158

1e-05

2.1e-05

7.1e-053.1e-055.7e-057.9e-05

1.1e-052.7e-058e-061.5e-05

3.6e-05

1.8e-05

3.5e-05

2.7e-05

7e-055.6e-052.4e-058e-05

3.9e-05

6.3e-053.8e-052.2e-058.5e-05

5.8e-05

3.6e-05

3.3e-05

7.1e-05

2.2e-05

8e-055.3e-055.3e-059e-05

0.000135

3.2e-05

1.7e-05

0.0003614.7e-050.0001050.0001080.0004

0.0003614.7e-050.0001050.0001080.0004

0.0003614.7e-050.0001050.0001080.0004

0.0003614.7e-050.0001050.0001080.0004

0.0001624e-060.0004140.000208

0.0001624e-060.0004140.000208

8.9e-05

8.9e-05

1.1e-052.5e-051.1e-05

1.1e-052.5e-051.1e-05

5.9e-05

5.9e-05

0.0001514e-060.0002410.000197

1.2e-051e-051.1e-05

2.1e-05

0

8e-061.8e-058e-06

3.5e-059e-063.7e-05

2e-064e-062e-062e-06

7e-06

2.1e-052.1e-053e-05

3e-06

1.4e-05

1.2e-051e-051.4e-05

3.6e-053e-067e-05

2.5e-054e-062.5e-05

8e-06

3.4e-05

3.3e-05

4.4e-05

0.0008380.0001110.0001020.0006070.000887

0.0008380.0001110.0001020.0006070.000887

0.0001270.0001190.000132

6.2e-057.3e-056.6e-05

6.5e-054.6e-056.6e-05

0.0005626.5e-050.0001020.0003910.000587

0.00021.7e-050.0001029.7e-050.0002

0.0001211.6e-057.9e-050.000123

0.0001271.8e-057.8e-050.000142

0.0001141.4e-050.0001370.000122

5.7e-054.6e-052.3e-057e-05

3.6e-052e-051.2e-054.1e-05

2.1e-052.6e-051.1e-052.9e-05

9.2e-057.4e-059.8e-05

9.2e-057.4e-059.8e-05

0.0001619e-050.0002080.000166

0.0001619e-050.0002080.000166

0.0001619e-050.0002080.000166

0.0001619e-050.0002080.000166

0.0001390.0002310.000108

0.0001390.0002310.000108

0.0001390.0002310.000108

0.0001390.0002310.000108

0.0011230.0002020.0005570.0011010.001147

0.000547.5e-050.000460.0007880.000559

0.0001010.000197

0.0001010.000197

0.0002373e-050.0001278.6e-050.000263

0.0002373e-050.0001278.6e-050.000263

0.0003034.5e-059.7e-050.000240.000296

0.00013

0.0003034.5e-059.7e-050.000110.000296

0.0001350.000265

0.0001350.000265

0.0005830.0001279.7e-050.0003130.000588

3.8e-053.7e-054.4e-05

2e-061.3e-053e-06

3.6e-052.4e-054.1e-05

0.0001133.7e-050.000123

7e-06

0.0001133e-050.000123

0.0001162.7e-056.5e-050.000115

0.0001162.7e-056.5e-050.000115

0.0001910.00019.7e-052.6e-050.00019

0.0001910.00019.7e-052.6e-050.00019

0.0001250.0001480.000116

0.0001250.0001480.000116

0.0004951.9e-050.000258.2e-050.000485

0.0004951.9e-050.000258.2e-050.000485

0.0004951.9e-050.000258.2e-050.000485

0.0004951.9e-050.000258.2e-050.000485

0.0009020.0001940.000320.00040.000921

0.0009020.0001940.000320.00040.000921

0.0002545.6e-059.7e-050.0001230.000272

0.0002545.6e-059.7e-050.0001230.000272

0.0003496.8e-050.0001010.0001050.000357

0.0003496.8e-050.0001010.0001050.000357

0.0002997e-050.0001220.0001720.000292

0.0002997e-050.0001220.0001720.000292

0.0010990.0002160.0004130.0010680.001172

0.0010990.0002160.0004130.0010680.001172

0.000295.7e-050.0001560.0003260.000323

6.4e-05

1e-06

0.000295.7e-050.0001560.00010.000323

8.4e-05

4e-06

7e-05

3e-06

0.0001462.7e-050.0001090.000158

0.0001462.7e-050.0001090.000158

0.0001744.5e-058.9e-050.000172

0.0001744.5e-058.9e-050.000172

0.000104

0.000104

4.2e-05

4.2e-05

0.0002336.6e-058.4e-050.0001660.000222

1.3e-05

6.4e-054.9e-052.6e-056.3e-05

0.0001691.7e-058.4e-052.5e-050.000159

0.000102

7.3e-05

7.3e-05

0.0001482.1e-058.4e-058.8e-050.000187

0.0001482.1e-058.4e-058.8e-050.000187

0.0001088.7e-050.00011

0.0001088.7e-050.00011

7.3e-05

7.3e-05

0.0057270.0007310.0027330.0065130.0056110.000167

0.0001070.0015910.000112

0.0001070.0015910.000112

0.0001070.0015910.000112

0.0002672.3e-050.0001120.0001540.0002862.8e-05

0.0002672.3e-050.0001120.0001540.0002862.8e-05

0.0002672.3e-050.0001120.0001540.0002862.8e-05

0.0007510.0001320.0002510.0003660.000756

0.0007510.0001320.0002510.0003660.000756

0.0001844.1e-050.0001030.000185

0.000365.5e-050.0001420.0001940.000352

0.0002073.6e-050.0001096.9e-050.000219

0.0002875.2e-050.0001750.0003590.0002882.7e-05

9.4e-05

9.4e-05

8.4e-050.0001642.7e-05

2.7e-05

8.4e-050.000164

0.0002875.2e-059.1e-050.0001010.000288

0.0002875.2e-059.1e-050.0001010.000288

0.0002792.6e-050.0001090.0002830.000284

0.0002792.6e-050.0001090.0001440.000284

0.0002792.6e-050.0001090.0001440.000284

0.000139

0.000139

7e-064e-056e-06

7e-064e-056e-06

7e-064e-056e-06

0.0003953e-050.0001620.0004470.000394

7.8e-05

7.8e-05

0.000142

0.000142

0.0003953e-050.0001620.0002270.000394

0.0003953e-050.0001620.0002270.000394

0.0007330.0001140.000270.0002620.000739

0.0003275.1e-050.0001657.6e-050.000319

0.0003275.1e-050.0001657.6e-050.000319

0.0004066.3e-050.0001050.0001860.00042

0.0004066.3e-050.0001050.0001020.00042

8.4e-05

2.3e-054.8e-051.1e-05

2.3e-054.8e-051.1e-05

2.3e-054.8e-051.1e-05

0.0002323.2e-059.7e-050.0001320.000256

0.0002323.2e-059.7e-050.0001320.000256

0.0002323.2e-059.7e-050.0001320.000256

0.0005216.6e-050.0005120.0013480.000555

0.0001754.1e-059.6e-054.5e-050.000204

0.0001754.1e-059.6e-054.5e-050.000204

6.5e-05

6.5e-05

0.00013

0.00013

0.0001136.8e-050.000122

0.0001136.8e-050.000122

7.6e-05

7.6e-05

4.6e-05

4.6e-05

0.0001250.000244

0.0001250.000244

5.1e-05

3.5e-05

1.6e-05

0.0002332.5e-050.0002910.0005590.000229

9.2e-050.000181

8.1e-050.000158

0.0002332.5e-050.0001184.9e-050.000229

6.5e-05

5.4e-05

5.2e-05

6.4e-05

6.4e-05

0.0022320.0002560.0009380.0014830.002036

1e-051.4e-058e-06

1e-051.4e-058e-06

0.000140.000220.000127

7e-052.8e-056.6e-05

7e-050.0001166.1e-05

7.6e-05

0.000256.6e-050.0001240.0002670.000234

9.6e-05

8.5e-05

0.000256.6e-050.0001248.6e-050.000234

0.000106

0.000106

6.9e-058.7e-050.0004176.9e-05

3.5e-05

6e-06

7e-06

6.4e-05

1.6e-05

2.4e-05

9e-069e-064e-06

3.2e-05

6e-052.3e-056.5e-05

3.1e-05

8.7e-050.00017

6.1e-057.6e-057.1e-05

6.1e-057.6e-057.1e-05

0.0003660.0001380.0001250.0002850.000356

0.0003660.0001380.0001250.0002850.000356

0.0012590.0006021.5e-050.001108

0.0012590.0006021.5e-050.001108

7.7e-055.2e-058.3e-056.3e-05

4.4e-052.6e-054.6e-05

3.3e-055.2e-055.7e-051.7e-05

0.0002170.0001980.0002370.000241

0.0002170.0001980.0002370.000241

8.1e-050.000158

8.1e-050.000158

0.0002170.0001177.9e-050.000241

0.0002170.0001177.9e-050.000241

0.0001960.0004440.000161

0.0001960.0004440.000161

0.0001391.8e-050.000115

0.0001391.8e-050.000115

1.4e-05

1.4e-05

6.4e-05

6.4e-05

0.00029

7.1e-05

8.5e-05

5.1e-05

8.3e-05

5.7e-055.8e-054.6e-05

5.7e-055.8e-054.6e-05

0.0035010.0007820.0014150.0044940.003625

7.7e-059.1e-057.5e-05

7.7e-059.1e-057.5e-05

7.7e-059.1e-057.5e-05

0.0001748.8e-050.000150.000196

0.0001748.8e-050.000150.000196

0.0001748.8e-050.000150.000196

0.0011730.0005470.0005810.0015070.001049

3.7e-050.0001190.0002324.4e-05

3.7e-050.0001190.0002324.4e-05

0.0011360.0005470.0004620.0012750.001005

5e-062e-065e-06

8e-06

5.3e-058.7e-056.6e-05

0

1e-063e-061e-06

0

5e-062e-067e-06

1.5e-058e-056e-061.8e-05

0.000115

0.0001759e-05

7e-06

4.3e-052e-053.6e-05

1e-064e-061e-06

1.6e-05

1e-06

3e-05

8.5e-050.0001830.000104

0.0004140.0001445.4e-050.000214

6.4e-051.7e-051.6e-057.7e-05

4e-061.1e-051e-065e-06

0.0001151.6e-050.0001040.000134

0.0001879.6e-050

3.4e-052e-055e-064e-05

3.4e-05

5e-06

6e-050

4.6e-05

6e-06

0.0001320.000258

3e-060

6.3e-051.3e-052.1e-056.1e-05

1.2e-051.3e-051.4e-05

8.8e-05

4.2e-058e-062e-064.8e-05

0

5.4e-05

0.0001062e-053.2e-050.000133

6e-06

1.4e-054.3e-054.1e-05

0.0008557e-050.0003730.0018110.000936

0.0008557e-050.0003730.0018110.000936

8.2e-05

2.3e-05

0

4.3e-052.6e-052.6e-053.7e-05

2.9e-05

2.3e-05

5.8e-051.3e-054.9e-05

4e-068e-061e-063e-06

2.6e-05

0.0001260.000246

3.1e-05

2.3e-05

0.000115

3.7e-05

9e-06

2.3e-051.6e-057e-06

0.0001530.000299

9.9e-05

8.8e-053.6e-055e-050.000103

3.4e-05

6.6e-05

1e-061e-062e-06

3.5e-05

2.3e-052.4e-055.6e-05

1.5e-051.9e-057e-06

1.4e-05

3.1e-052.1e-051.3e-05

9.9e-05

01e-060

2.8e-05

0.0001112.8e-050.000137

9e-053e-050.000117

0.0001073e-050.000151

5.8e-05

4.8e-05

3.4e-052.9e-053.5e-05

3.8e-051.2e-054e-05

1.8e-05

0.0001899.4e-052.9e-050.000179

3.9e-05

2.5e-05

2.5e-05

2.5e-05

0.0009190.0001650.0002920.0005180.001021

0.0005449.5e-050.0001820.0003070.000616

6.2e-05

0.0001051.6e-052.8e-050.000122

1.3e-05

1.9e-05

0.0001362.9e-057.3e-051.3e-050.000151

3.5e-057e-063.5e-05

5.1e-05

6.8e-051.4e-052e-058.1e-05

3e-06

1e-05

1.4e-05

0.00023.6e-050.0001096.5e-050.000227

2e-06

0.0003757e-050.000110.0002110.000405

7.1e-05

0.0002153.8e-050.000113.7e-050.000217

6.9e-051.8e-055e-058e-05

9.1e-051.4e-055.3e-050.000108

0.0003038.1e-050.0003920.000348

0.0003038.1e-050.0003920.000348

0.0001173.4e-050.000131

8.1e-050.000159

6.4e-05

6.9e-056.4e-057.8e-05

0.0001177.1e-050.000139

0.000750.0007760.0020180.000822

0.0003750.0005020.0011150.000417

0.0003440.0001010.000330.000395

0.0002350.0001010.0001340.000285

0.0002350.0001010.0001340.000285

0.0001090.0001960.00011

0.000114

0.0001098.2e-050.00011

3.1e-050.0004010.0007852.2e-05

0.000230.00045

0.000230.00045

3.1e-050.0001710.0003352.2e-05

3.1e-050.0001710.0003352.2e-05

0.0003750.0002740.0009030.000405

0.0003750.0002740.0009030.000405

0.0001220.000150.000111

0.0001220.000150.000111

9.6e-050.000187

9.6e-050.000187

0.00010.0001780.0003499.8e-05

1e-050.00010.0001961.7e-05

9e-057.8e-050.0001538.1e-05

2.2e-050.0001226.8e-05

2.2e-050.0001226.8e-05

6.2e-056.1e-05

6.2e-056.1e-05

6.9e-059.5e-056.7e-05

6.9e-059.5e-056.7e-05

0.0002949.1e-050.0008850.000253

0.0002949.1e-050.0008850.000253

0.0002949.1e-050.0008850.000253

0.0001289.1e-050.0007180.00013

0.0001289.1e-050.0007180.00013

0.0001660.0001670.000123

0.0001660.0001670.000123

0.0071530.0020880.0010750.0040540.0071680.000728

0.0071530.0020880.0010750.0040540.0071680.000728

0.0071530.0020880.0010750.0040540.0071680.000728

0.0071530.0020880.0010750.0040540.0071680.000728

0.0071530.0020880.0010750.0040540.0071680.000728

0.0004130.00030.0006310.000375

0.0004130.00030.0006310.000375

0.0004130.00030.0006310.000375

0.0004130.00030.0006310.000375

8.9e-050.000174

0.0004130.0002110.0004570.000375

2e-055.4e-052e-05

5.4e-05

5.4e-05

5.4e-05

5.4e-05

5.4e-05

2e-052e-05

2e-052e-05

2e-052e-05

2e-052e-05

2e-052e-05

2.4e-050.0001180.0002312.5e-05

2.4e-050.0001180.0002312.5e-05

2.4e-050.0001180.0002312.5e-05

2.4e-050.0001180.0002312.5e-05

2.4e-050.0001180.0002312.5e-05

2.4e-050.0001180.0002312.5e-05

0.0002070.000410.0012040.000214

0.0002070.000410.0012040.000214

0.0002070.000410.0012040.000214

2.5e-050.0001084.6e-05

7e-067.3e-055e-06

7e-067.3e-055e-06

1.8e-053.5e-054.1e-05

1.8e-052.2e-054.1e-05

1.3e-05

0.0001820.000410.0010960.000168

2e-060.0001230.000247e-06

2e-060.0001230.000247e-06

6.3e-05

6.3e-05

6.1e-050.0002870.0005615e-05

6.1e-050.0002870.0005615e-05

0.0001190.0002320.000111

0.0001199.7e-050.000111

7.8e-05

5.7e-05

0.0004860.000120.0001190.000428

0.0004860.000120.0001190.000428

0.0004860.000120.0001190.000428

0.0004860.000120.0001190.000428

0.0004860.000120.0001190.000428

0.0004860.000120.0001190.000428

6.8e-050.0008140.0018397e-05

6.8e-050.0008140.0018397e-05

5.6e-050.0006730.0015235.3e-05

8.8e-05

8.8e-05

8.8e-05

9e-060.0003830.0008581.2e-05

2e-060.0001020.0001992e-06

2e-060.0001020.0001992e-06

4e-068.9e-057e-06

1e-06

3e-068.9e-057e-06

1e-060.0001060.0002071e-06

1e-060.0001060.0002071e-06

2e-060.0001750.0003632e-06

2e-060.0001750.0003422e-06

2.1e-05

4.7e-050.000290.0005774.1e-05

6e-060.0001450.0002833e-06

6e-060.0001450.0002833e-06

4.1e-050.0001450.0002943.8e-05

1e-061.1e-051e-06

4e-050.0001450.0002833.7e-05

1.2e-050.0001410.0003161.7e-05

1.2e-050.0001410.0003161.7e-05

4e-060.0001410.0002762e-06

4e-060.0001410.0002762e-06

8e-064e-051.5e-05

8e-064e-051.5e-05

0.0069160.0011570.0058220.015260.0070845.7e-05

0.0002330.0002390.0015960.000229

0.0001570.000435

0.0001570.000308

0.0001570.000308

0.0001570.000308

0.000127

0.000127

0.000127

0.0002338.2e-050.0011610.000229

3.7e-050.0002274.8e-05

3.7e-058.1e-054.8e-05

2.8e-057.7e-054.1e-05

9e-064e-067e-06

0.000146

0.000146

1.4e-050.0002151.5e-05

1.4e-056.5e-051.5e-05

1.4e-055.1e-051.5e-05

1.4e-05

00.000150

0.000101

04.9e-050

3.5e-050.0001073.6e-05

3.5e-050.0001073.6e-05

9e-061.5e-057e-06

8e-06

1e-055e-069e-06

7e-069e-061e-05

1.2e-05

4e-062.9e-054e-06

2e-068e-064e-06

3e-062.1e-052e-06

8.9e-050.0001316.8e-05

8.9e-050.0001316.8e-05

2e-062.8e-053e-06

1.8e-05

1.6e-05

2e-06

1e-063e-061e-06

4.5e-057e-061.9e-05

1.9e-051.8e-052.3e-05

5e-06

2.2e-053.4e-052.2e-05

3.3e-050.0001623.6e-05

3.7e-05

3.7e-05

3.3e-050.0001253.6e-05

1.4e-050.0001251.3e-05

1.9e-052.3e-05

6.5e-05

6.5e-05

6.5e-05

1.2e-055.2e-051.3e-05

1.2e-055.2e-051.3e-05

1.2e-055.2e-051.3e-05

1.2e-058.2e-050.0001611.2e-05

1.2e-058.2e-050.0001611.2e-05

1.2e-058.2e-050.0001611.2e-05

1e-064.1e-051e-06

1e-062e-051e-06

1e-062e-051e-06

2.1e-05

2.1e-05

0.0007290.0004150.0011280.000697

0.0007290.0004150.0011280.000697

0.0007290.0004150.0011280.000697

0.0006730.0002130.0002410.000642

0.0006730.0002130.0002410.000642

3.1e-05

3.1e-05

6e-060.0003171e-05

8.3e-05

8.6e-05

6e-060.0001251e-05

2.3e-05

3.7e-050.0001040.0002043.5e-05

3.7e-050.0001040.0002043.5e-05

1.3e-059.8e-050.0003351e-05

1.3e-050.0001431e-05

9.8e-050.000192

0.0004280.0001760.0002710.000301

0.0004280.0001760.0002710.000301

0.0004280.0001760.0002710.000301

1.8e-05

1.8e-05

0.000115

8.9e-05

2.6e-05

0.0004130.0001766.7e-050.000294

0.0004130.0001766.7e-050.000294

1.5e-053.1e-057e-06

1.5e-053.1e-057e-06

4e-05

4e-05

0.0042140.0011570.0035380.0087820.0044555.7e-05

0.0042140.0011570.0035380.0087820.0044555.7e-05

1.9e-059e-050.0001761.5e-05

1.9e-059e-050.0001761.5e-05

1.9e-059e-050.0001761.5e-05

3.2e-056.1e-053.1e-05

3.2e-056.1e-053.1e-05

3.2e-056.1e-053.1e-05

3.8e-05

3.8e-05

3.8e-05

0.0039140.0011570.0034480.0084420.0041745.7e-05

0.0004240.00083

0.0004240.00083

7.6e-052.4e-050.0001328.2e-05

7.6e-052.4e-050.0001328.2e-05

0.0009425.6e-050.0004370.0001470.000994

0.000842.3e-050.0004375.2e-050.000868

2.2e-05

0.0001023.3e-057.3e-050.000126

0.000137

0.000137

1.1e-059e-061.1e-05

1.1e-059e-061.1e-05

8.3e-057.7e-059.2e-05

8.3e-057.7e-059.2e-05

1.3e-050.0001431.3e-05

1.3e-050.0001431.3e-05

6.6e-057.7e-050.0001517.2e-05

6.6e-057.7e-050.0001517.2e-05

0.0001720.000336

0.0001720.000336

0.0002920.00057

0.0002920.00057

0.0001954.9e-050.0001120.0002950.000193

0.0001954.9e-050.0001120.0002950.000193

4.4e-051.9e-054.8e-05

4.4e-051.9e-054.8e-05

0.0001030.0002080.0006530.000111

0.0001030.0002470.000111

0.0002080.000406

2e-050.0001292.3e-05

2e-050.0001292.3e-05

0.0001010.0001350.000103

0.0001010.0001350.000103

6e-06

3e-06

2e-06

1e-06

9.3e-050.0001349.8e-05

9.3e-050.0001349.8e-05

0.0002165.1e-050.0001450.0005550.000242

0.0002165.1e-050.0001450.0005550.000242

0.000460.0004080.0002680.000464

7.8e-050.0001152.6e-057.3e-05

0.0001010.0001171.6e-050.000102

5.7e-054.8e-055.3e-056.2e-05

0.0001368.3e-057.1e-050.000132

8.8e-054.5e-050.0001029.5e-05

0.0002373.3e-050.0001310.0002390.0003

0.0002373.3e-050.0001310.0002390.0003

7.9e-050.0001948e-05

4.9e-05

7.9e-050.00018e-05

4.5e-05

1.9e-05

1.8e-05

1e-06

0.000380.000743

0.000380.000743

0.0001420.000278

0.0001420.000278

6.1e-059.7e-050.000195e-05

6.1e-059.7e-050.000195e-05

6.9e-058.4e-059.1e-05

3.6e-05

1.9e-054e-062.9e-05

1e-06

5e-054.3e-056.2e-05

5e-05

1.7e-05

5e-06

1.7e-05

1.1e-05

6.2e-05

6.2e-05

0.0009980.0005360.0004230.0009820.0010565.7e-05

0.0009980.0005360.0004230.0009820.0010565.7e-05

0.0002730.000533

0.0001310.000256

0.0001420.000277

4.7e-050.0001350.0003425.1e-05

7.9e-05

4.7e-050.0001350.0002635.1e-05

0.0002496.5e-050.000235

0.0002496.5e-050.000235

2e-065e-06

1.6e-054e-061.7e-05

6e-067e-064e-06

2.4e-053e-062.3e-05

5.3e-058e-063.8e-05

1.6e-059e-062.1e-05

1.3e-05

0.0001321.6e-050.000132

6.6e-059.1e-050.0003166.5e-05

6.6e-059.1e-050.0003166.5e-05

5.1e-050.0001395.2e-05

5.1e-050.0001395.2e-05

5.1e-050.0001395.2e-05

1.5e-059.1e-050.0001771.3e-05

1.5e-059.1e-050.0001771.3e-05

1.5e-059.1e-050.0001771.3e-05

0.000890.0004770.0001540.000995

0.000890.0004770.0001540.000995

0.000890.0004770.0001540.000995

0.0003490.0002057.7e-050.000471

0.0003490.0002057.7e-050.000471

0.0005410.0002727.7e-050.000524

0.0005410.0002727.7e-050.000524

0.0003560.0008860.0030130.000342

0.0003560.0008860.0030130.000342

0.0001160.0001780.0004759.8e-05

0.0001167.7e-050.0002179.8e-05

0.0001167.7e-050.0002179.8e-05

0.0001010.000198

0.0001010.000198

6e-05

6e-05

3.9e-050.000180.0004164.1e-05

9.8e-050.000192

9.8e-050.000192

2.8e-058.2e-050.0001613e-05

2.8e-058.2e-050.0001613e-05

5e-064.3e-055e-06

5e-064.3e-055e-06

6e-062e-056e-06

6e-061e-056e-06

1e-05

0.000129

0.000129

0.000129

0.0001310.0001120.0005680.000151

4.6e-050.0001120.000223.8e-05

4.6e-050.0001120.000223.8e-05

1.5e-050.0001011.5e-05

1.5e-050.0001011.5e-05

2e-066.4e-053e-05

2e-066.4e-053e-05

3.1e-052.9e-05

3.1e-052.9e-05

2.8e-054.2e-052.5e-05

2.8e-054.2e-052.5e-05

9e-060.0001051.4e-05

9e-060.0001051.4e-05

3.6e-05

3.6e-05

4.6e-059.8e-050.0002412.9e-05

1.8e-055e-059e-06

1.8e-055e-059e-06

2.8e-059.8e-050.0001912e-05

2.8e-059.8e-050.0001912e-05

2.4e-050.0001140.0002232.3e-05

2.4e-050.0001140.0002232.3e-05

2.4e-050.0001140.0002232.3e-05

0.0002040.000961

8.8e-050.000388

7.5e-05

8.8e-050.000172

0.000141

0.000283

6.8e-05

7e-06

2.9e-05

3.1e-05

9.3e-05

9e-06

4.6e-05

0.0001160.00029

6.4e-05

0.0001160.000226

0.0008030.0003060.0013090.000687

0.0002840.0001820.0005260.000269

0.0001027.6e-050.0002746.7e-05

0.0001027.6e-050.0002746.7e-05

0.0001027.6e-050.0002746.7e-05

0.0001027.6e-050.0002746.7e-05

0.0001820.0001060.0002520.000202

0.0001820.0001060.0002520.000202

0.0001820.0001060.0002520.000202

0.0001820.0001060.0002520.000202

7.1e-050.0002496.8e-05

7.1e-050.0002496.8e-05

1.5e-050.0001141.5e-05

1.5e-050.0001141.5e-05

1.5e-050.0001121.5e-05

0

2e-06

5.6e-050.0001355.3e-05

5.6e-050.0001355.3e-05

4.6e-050.0001014.3e-05

1e-053.4e-051e-05

5.8e-050.0002076.4e-05

3.9e-050.0001433.5e-05

3.9e-050.0001433.5e-05

3.9e-050.0001433.5e-05

3.9e-050.0001433.5e-05

1.9e-056.4e-052.9e-05

1.9e-056.4e-052.9e-05

1.9e-056.4e-052.9e-05

1.9e-056.4e-052.9e-05

0

0.0003770.0001240.0001660.000273

0.0003770.0001240.0001660.000273

0.0003770.0001240.0001660.000273

0.0003770.0001240.0001660.000273

0.0003770.0001240.0001660.000273

1.3e-050.0001611.3e-05

1.3e-050.0001611.3e-05

1.3e-050.0001611.3e-05

9.3e-05

9.3e-05

1.3e-056.8e-051.3e-05

1.3e-056.8e-051.3e-05

0.001430.0003390.0005820.001419

0.001430.0003390.0005820.001419

0.001430.0003390.0005820.001419

0.001430.0003390.0005820.001419

0.0013920.0001420.0001070.001385

0.0013920.0001420.0001070.001385

3.8e-050.0001970.0004313.4e-05

3.8e-054.5e-053.4e-05

0.0001970.000386

4.4e-05

4.4e-05

0.0003420.0004780.0016920.000388

0.0003420.0004780.0016920.000388

0.0001538.3e-050.000480.000208

0.0001538.3e-050.000480.000208

0.0001268.3e-050.0002740.000145

0.0001268.3e-050.0002740.000145

7.7e-05

7.7e-05

2.7e-050.0001296.3e-05

2.7e-050.0001296.3e-05

6.1e-050.0003110.0006545.4e-05

6.1e-050.0003110.0006545.4e-05

1.3e-050.0002090.0004556e-06

1.3e-050.0002090.0004096e-06

4.6e-05

4.8e-050.0001020.0001994.8e-05

4.8e-050.0001020.0001994.8e-05

0.0001288.4e-050.0005580.000126

7.2e-050.000247.2e-05

1.8e-050.0001171.9e-05

9e-068.3e-057e-06

9e-063.4e-051.2e-05

5.4e-050.0001235.3e-05

2.8e-056.7e-052.7e-05

2.6e-054.3e-052.6e-05

1.3e-05

5.6e-058.4e-050.0003185.4e-05

2e-068.4e-050.0001941e-06

0

1e-068.4e-050.0001631e-06

1e-061e-05

2.1e-05

5.4e-050.0001245.3e-05

4e-06

1.5e-05

9e-06

8.5e-05

5.4e-051e-065.3e-05

1e-06

1e-06

8e-06

0

0.000220.0006430.0015790.000182

0.0001120.0006430.0014519.3e-05

0.0001120.0006430.0014519.3e-05

4.3e-057.7e-050.0002852.8e-05

2.7e-057.7e-050.000152.1e-05

2.7e-057.7e-050.000152.1e-05

1.6e-050.0001357e-06

1.6e-050.0001357e-06

6.9e-050.0005660.0011666.5e-05

5e-060.0001020.00024e-06

5e-060.0001020.00024e-06

1.9e-050.0001890.0003692.1e-05

1.9e-050.0001890.0003692.1e-05

6e-05

6e-05

3e-060.0001230.000245e-06

3e-060.0001230.000245e-06

4.2e-050.0001520.0002973.5e-05

4.2e-050.0001520.0002973.5e-05

0.0001080.0001288.9e-05

0.0001080.0001288.9e-05

0.0001080.0001288.9e-05

0.0001080.0001288.9e-05

0.0001080.0001288.9e-05

0.0014480.0004450.0011450.001095

7.4e-05

7.4e-05

7.4e-05

7.4e-05

7.4e-05

1.5e-054e-058e-06

1.5e-054e-058e-06

1.5e-054e-058e-06

1.5e-054e-058e-06

1.5e-054e-058e-06

0.000151

0.000151

0.000151

0.000151

0.000151

0.0014090.0004450.0007660.001051

1.8e-050.0002430.0005971.6e-05

1.8e-050.0002430.0005971.6e-05

1.8e-058.3e-050.0001621.6e-05

1.8e-058.3e-050.0001621.6e-05

0.000122

0.000122

0.000160.000313

0.000160.000313

0.0013910.0002020.0001690.001035

0.0013910.0002020.0001690.001035

0.0013910.0002020.0001690.001035

0.0013910.0002020.0001690.001035

2.4e-050.0001143.6e-05

2.4e-050.0001143.6e-05

2.4e-050.0001143.6e-05

2.4e-050.0001143.6e-05

3.8e-05

2.4e-057.6e-053.6e-05

0.0058260.0047480.0229

0.0058260.0047480.0229

0.0058260.0047480.0229

0.0058260.0047480.0229

0.0058260.0047480.0229

0.0058260.0047480.0229

0.0022740.0007940.0016960.001941

0.0022740.0007940.0016960.001941

0.0022740.0007940.0016960.001941

0.0022740.0007940.0016960.001941

0.0018120.0004050.0005630.001717

2.2e-05

5.7e-050.0001910.0003744.5e-05

0.0017550.0002140.0001670.001672

7.1e-050.0002297.5e-05

6.1e-050.0001235.2e-05

1e-050.0001062.3e-05

0.0003490.0001840.0004669.8e-05

0.0002969.8e-050.0001416e-05

2.6e-050.0001091.6e-05

2.5e-054.9e-052e-05

2e-068.6e-050.0001672e-06

1.2e-059.9e-050.0001931.3e-05

1.2e-059.9e-050.0001931.3e-05

3e-050.0001060.0002453.8e-05

0.0001060.000206

3e-053.9e-053.8e-05

0.3033940.1039150.5801770.19540.7130499999999990.3126580.529119

0.0030291e-050.0025360.0054310.002277

2.4e-050.000140.0002743.7e-05

2.4e-050.000140.0002743.7e-05

2.4e-050.000140.0002743.7e-05

2.4e-050.000140.0002743.7e-05

5e-060.0001929.6e-05

5e-060.0001929.6e-05

2e-068.2e-051e-06

2e-068.2e-051e-06

3e-060.000119.5e-05

8.2e-05

3e-062.8e-059.5e-05

0.0031e-050.0023960.0049650.002144

0.001511e-050.0005240.0014820.001473

2.4e-050.0001920.0003872.6e-05

1e-061.1e-051e-06

3e-068.7e-050.000177e-06

2e-050.0001050.0002061.8e-05

0.0013540.0001810.0003980.001377

0.000119

0.0013340.0001810.0001440.001335

7.5e-05

2e-053.3e-054.2e-05

1.7e-05

1e-05

0.0001321e-050.0001510.0006977e-05

4.2e-051.3e-054.2e-05

1.2e-053.6e-056e-06

3e-060.0001510.000296

2.4e-05

4e-06

0.000114

7.4e-05

9e-06

5.3e-055.7e-05

1.5e-05

2e-06

8e-063e-061e-05

1.2e-051e-051e-061.1e-05

3e-06

2e-063e-061e-06

4.3e-05

0.000410.0001010.000310.000106

0.000410.0001010.000310.000106

0.000410.0001010.000310.000106

0.001080.0017710.0031730.000565

1.5e-050.0009580.0018754.4e-05

3e-060.0001790.0003513e-05

1.2e-050.0007790.0015241.4e-05

0.0009710.0005270.000740.00038

6e-068.8e-050.0001734e-06

0.0009430.0002571.2e-050.000342

1e-061e-062e-06

0.0001820.000355

1e-055.1e-051e-05

4e-067.2e-054e-06

2e-063e-061e-06

1.7e-05

4.9e-05

2e-067e-063e-06

3e-061.4e-05

8e-050.0002060.0004027e-05

8e-050.0002060.0004027e-05

1.4e-058e-050.0001567.1e-05

1.4e-058e-050.0001567.1e-05

0.0075830.0006350.0073640.015480.007508

0.0020535e-050.0022450.004090.002531

0.0019425e-050.0013150.002270.002451

9.8e-050.0004080.0007987.6e-05

9.8e-050.0004080.0007987.6e-05

0.0008970.0004060.000650.000893

0.0008970.0004060.000650.000893

0.0008875e-050.000270.0005380.000372

0.0008875e-050.000270.0005380.000372

6e-050.0002310.0002840.00111

6e-050.0002310.0002840.00111

0.0001110.000930.001828e-05

2.9e-050.0001890.000373.5e-05

2.9e-050.0001890.000373.5e-05

1.2e-050.0001670.0003261.4e-05

1.2e-050.0001670.0003261.4e-05

1.9e-050.0002010.0003946e-06

1.9e-050.0002010.0003946e-06

5.1e-050.0003730.000732.5e-05

5.1e-050.0003730.000732.5e-05

0.0002350.0003050.0006537.4e-05

0.0002080.0002050.0004586.3e-05

1.4e-050.0001220.0002391.4e-05

1.4e-050.0001220.0002391.4e-05

0.000198.3e-050.0001414.8e-05

0.000198.3e-050.0001414.8e-05

4e-067.8e-051e-06

4e-067.8e-051e-06

2.7e-050.00010.0001951.1e-05

2.7e-050.00010.0001951.1e-05

2.7e-050.00010.0001951.1e-05

2.4e-050.0002630.0006495.5e-05

2.4e-050.0002630.0006495.5e-05

2.4e-050.0001345.5e-05

2.4e-050.0001345.5e-05

0.0002630.000515

0.0002630.000515

0.000497.4e-050.0004720.0018570.000481

0.0002234e-058.1e-050.0004670.000225

0.0002234e-058.1e-050.0004670.000225

5.3e-052.1e-050.0001154.9e-05

0.0001448.1e-050.000210.000137

2.6e-051.9e-050.0001423.9e-05

6.1e-050.0001170.0002295.2e-05

6.1e-050.0001170.0002295.2e-05

6.1e-050.0001170.0002295.2e-05

4.9e-059e-060.0001894.6e-05

8.3e-05

8.3e-05

4.9e-059e-060.0001064.6e-05

4.9e-059e-060.0001064.6e-05

0.0001410.000275

0.0001410.000275

0.0001410.000275

3.1e-050.0001812.9e-05

3.1e-057.7e-052.9e-05

3.1e-057.7e-052.9e-05

0.000104

0.000104

0.0001330.00026

0.0001330.00026

0.0001330.00026

0.0001262.5e-050.0002560.000129

9.7e-052.5e-050.0002179.1e-05

3.6e-059.3e-053.6e-05

1.9e-05

1.8e-051.2e-054.7e-051.3e-05

4.3e-051.3e-055.8e-054.2e-05

2.9e-053.9e-053.8e-05

2.9e-053.9e-053.8e-05

0.0030610.0003180.0022980.0042410.002125

0.0018880.0012310.0020110.001263

0.0006140.001201

0.0002840.000556

0.000330.000645

0.0018880.0006170.000810.001263

2.2e-050.0001670.0003271.8e-05

0.0018660.000450.0004830.001245

0.0011730.0003180.0010670.002230.000862

0.00050.000979

0.00050.000979

0.0011730.0003180.0005670.0012510.000862

5e-06

1.5e-054.2e-051.4e-05

0.0001263.8e-050.000141

3.4e-050.0001440.0002832.9e-05

1.2e-051.7e-051e-05

0.0001030.0003690.0001

4.4e-050.0002920.0001495.2e-054.6e-05

0.0001320.000258

0.0008262.6e-050.0001420.0001320.000512

1.3e-055.5e-051e-05

0.0003630.0001930.0012250.0028850.000306

4.3e-052.6e-050.0003560.0007752.7e-05

4.3e-052.6e-050.0003560.0007752.7e-05

4.3e-052.6e-050.0003560.0006972.7e-05

7.8e-05

0.0002850.0001670.0005960.0015750.000245

1.5e-054.9e-052.6e-058e-06

1.5e-054.9e-052.6e-058e-06

0.000270.0001180.0005960.0015490.000237

5e-050.0001190.0002334.1e-05

2.3e-050.0001210.0002381.9e-05

3.8e-051.6e-05

0.0001230.000242

1e-050.0001151.1e-05

7e-061e-05

9e-060.0001371.2e-05

5.3e-053e-050.0001450.0002844.8e-05

4.6e-052.9e-058.8e-050.0001723.6e-05

1.5e-055.9e-055.8e-051.6e-05

1.9e-052.8e-05

7e-05

3.5e-050.0002730.0005353.4e-05

3.5e-050.0002730.0005353.4e-05

3.5e-050.0002730.0005353.4e-05

0.0001130.0001060.0002160.001082

0.0001130.0001060.0002160.001082

0.0001130.0001060.0002160.001082

0.0001130.0001060.0002160.001082

0.0012420.000450.0008320.000851

0.0001928.5e-050.000270.000173

0.0001928.5e-050.000270.000173

0.0001768.5e-050.0001720.000155

1.6e-059.8e-051.8e-05

0.001050.0003650.0005620.000678

0.001050.0003650.0005620.000678

0.001050.0003650.0005620.000678

2e-065.7e-053e-06

2e-065.7e-053e-06

2e-065.7e-053e-06

2e-065.7e-053e-06

0.0011930.0003710.0006360.00098

0.0011930.0003710.0006360.00098

0.0011930.0003710.0006360.00098

0.0011930.0003710.0006360.00098

0.0010830.0002140.0001970.000864

8.2e-050.0001570.0003069.5e-05

2.8e-050.0001332.1e-05

0.0301430.0263780.0271230.15440.0558310.031740.0055

0.0058250.0025660.0086840.0176730.0069140.000425

0.0007387.8e-050.0003360.0014650.000717

9.1e-052e-060.0001868.4e-05

3.3e-053.1e-052.9e-05

6e-062e-062.3e-056e-06

9.1e-05

5.2e-054.1e-054.9e-05

8.8e-05

8.8e-05

0.0001090.0001110.0004180.000118

2e-06

6.7e-05

7e-06

0.0001110.000217

2.7e-053e-063e-05

8e-06

2.6e-053e-052.7e-05

2e-053.9e-052.2e-05

3.6e-054.5e-053.9e-05

3.9e-05

3.9e-05

5.6e-05

5.6e-05

0.0001330.0003450.000128

2e-056.8e-052.4e-05

3.7e-051.8e-053.6e-05

0.000134

2e-050.000111.8e-05

5.6e-051.5e-055e-05

0.0004057.6e-050.0002250.0003330.000387

5.1e-053e-054.5e-05

4.5e-05

5.4e-059.6e-050.0001885e-05

4e-056.5e-054.8e-05

0.000267.6e-050.0001295e-060.000244

0.0019560.003826

0.0019560.003826

0.0019560.003826

0.0002580.000607

0.000101

0.000101

0.0002580.000506

0.0001440.000282

0.0001140.000224

0.0003598.4e-050.0018160.0037850.000336

0.00020.000467

7.6e-05

0.00020.000391

0.0001410.000275

0.0001410.000275

0.0001790.000349

0.0001790.000349

0.0001528.4e-058.2e-050.0001960.000143

0.0001528.4e-058.2e-050.0001960.000143

0.0001560.0008940.0017480.000149

3.8e-050.0005290.0010353.2e-05

7.2e-059.2e-050.000187.1e-05

4.6e-050.0002730.0005334.6e-05

5.1e-050.000320.0006254.4e-05

5.1e-050.000320.0006254.4e-05

0.000125

0.000125

3.8e-050.0003140.0006143.8e-05

3.8e-050.0003140.0006143.8e-05

3.8e-050.0003140.0006143.8e-05

0.0013440.000790.001480.001291

0.0007170.0002030.0002110.000709

0.0007170.0002030.0002110.000709

0.000126

0.000126

0.0006270.0002950.0005720.000582

0.0004960.0001260.0001270.000445

3.5e-054.7e-053.4e-05

8.9e-050.000174

6.5e-058e-050.0001577e-05

3.1e-056.7e-053.3e-05

0.0002920.000571

0.0002920.000571

0.0003391.6e-050.0002740.0007890.000369

0.0003391.6e-050.0002740.0007890.000369

7.9e-050.000155

3.8e-05

4.8e-059.3e-050.0001815e-05

9e-05

0.0001058.7e-050.000138

5.8e-052.9e-055.3e-05

9.2e-050.0001020.0001999.2e-05

3.6e-051.6e-051e-053.6e-05

0.0003637.9e-050.0010410.000349

0.0001020.0001950.000102

0.0001020.0001950.000102

0.0001167.9e-050.0007470.000106

0.0001167.9e-050.0007470.000106

0.0001459.9e-050.000141

0.0001459.9e-050.000141

1.6e-055.4e-050.0002561.9e-05

1.6e-052.3e-050.0001331.9e-05

0.000108

1.6e-052.3e-052.5e-051.9e-05

03.1e-050.0001230

0.000121

0

0

0

01.1e-050

1e-06

1e-06

01.1e-0500

9e-060

3.5e-050.0004360.0008534e-05

3.5e-050.0004360.0008534e-05

3.5e-050.0004360.0008534e-05

5.7e-050.000280.0006686.1e-05

4.1e-050.0001860.0003644.3e-05

4.1e-050.0001860.0003644.3e-05

1.6e-050.000121.8e-05

1.6e-050.000121.8e-05

9.4e-050.000184

9.4e-050.000184

0.0001180.0006550.0003560.001181

0.0001180.0006550.0003560.001181

6e-062.6e-051.2e-05

3e-066e-065e-06

2e-050.0001150.0002242.2e-05

2.6e-050.000541.1e-050.001057

1.3e-056e-061.2e-05

2.9e-051.7e-054.7e-05

2.7e-05

1e-063.3e-052e-06

2e-056e-062.4e-05

0.0024180.0023340.001490.0019330.0025130.000425

0.0017720.0021820.0010580.0008730.0019090.0002

4.5e-05

5.1e-05

0.0003560.0005750.0002156.4e-050.000385

0.0002720.0001920.0001257.7e-050.0002985.6e-05

0.0002420.0001830.0001143.5e-050.000257

0.0003240.0005170.0001956.7e-050.000353

0.0003420.0005480.0002063.7e-050.0003728.7e-05

0.0002360.0001670.0001010.0002445.7e-05

0.0002030.000396

0.0003340.000655

9.7e-050.000191

0.0001440.000282

9.3e-050.000182

0.0001892.4e-059.8e-058.7e-050.000195

0.0001892.4e-059.8e-058.7e-050.000195

0.0002079.3e-050.000160.0001870.000225

0.0002079.3e-050.000160.0001870.000225

0.000253.5e-050.0001580.000222

0.0001327.5e-050.000114

0.0001183.5e-058.3e-050.000108

0.000380.0002810.0009977.8e-05

0.000380.0002810.0009977.8e-05

0.000380.0002810.0009977.8e-05

0.000380.0002810.0009977.8e-05

0.0007830.0004240.0009470.000779

0.0007830.0004240.0009470.000779

0.0007830.0004240.0009470.000779

0.0007830.0004240.0009470.000779

0.0002720.000531

0.0001460.000285

0.0001460.000285

0.0001460.000285

0.0001260.000246

0.0001260.000246

0.0001260.000246

0.0016956.1e-050.0022460.0070310.002029

0.0007952.1e-050.0004430.0010050.001043

2.7e-050.0001421.8e-05

7e-060.0001295e-06

2e-051.3e-051.3e-05

6.3e-050.0001180.0001370.000741

6.3e-050.0001180.0001370.000741

0.0002150.000421

0.0002150.000421

0.0005560.000116.8e-050.000133

3.4e-05

0.0005560.000113.4e-050.000133

3.4e-051.4e-050.0001043.4e-05

3.4e-051.4e-050.0001043.4e-05

2.9e-057.7e-052.7e-05

2.9e-057.7e-052.7e-05

8.6e-057e-065.6e-059e-05

8.6e-057e-061.2e-059e-05

1e-05

3.4e-05

0.00094e-050.0018030.0060260.000986

2e-056.8e-051.5e-05

2e-056.8e-051.5e-05

0.0004780.000935

0.0004780.000935

0.0001230.0003070.0006010.000105

9.4e-050.0001480.000298.2e-05

2.9e-050.0001590.0003112.3e-05

0.000190.0002860.0008560.000156

0.0001138.7e-050.0003377.9e-05

0.000129

7.7e-050.0001990.000397.7e-05

0.000120.0003350.000121

0.000120.0003350.000121

0.0002120.000414

0.0002120.000414

0.0004158e-060.0002020.000430.000443

0.0001040.0001070.000161

9.8e-050.000191

7e-06

0.0003118e-060.0001040.0001250.000282

0.0001790.00035

0.0001790.00035

3.2e-053.2e-050.0001390.0020370.000146

3.2e-053.2e-050.0001390.0020370.000146

0.0113970.0105190.0069410.12740.0123070.0126440.00139

0.0108580.010120.006240.07490.0103890.0121590.00139

0.000240.000469

0.000240.000469

0.000142

0.000142

0.0026070.0015050.0018050.02780.0033980.0036110.00023

0.000150.000294

0.000113

0.00011

0.0026070.0015050.0002790.02780.0001890.0036110.00023

0.0001260.000246

0.001250.002446

0.0005820.0001340.0003880.000730.0005955.8e-05

7.9e-05

0.0002686.4e-050.0001350.0002310.000303

0.0003147e-050.0001180.0001560.0002925.8e-05

0.0001350.000264

0.0019820.0011240.0012270.04710.001510.0019860.000266

7.9e-050.000155

0.0008590.0004740.0002840.0003630.000862

0.0001780.000347

6e-06

0.0001150.000225

0.0001330.00026

8e-050.000157

0.0011230.000650.0003580.04710.0002630.001124

0.0003144.1e-050.0001620.0003030.000338

0.0003144.1e-050.0001620.0003030.000338

0.0053730.0073160.0022380.0034860.0056290.000836

0.0002540.000496

0.0021630.002780.0002440.000190.002260.000253

0.0005840.0012970.0002320.0002150.000599

0.0001580.000308

0.0006140.001202

0.0026260.0032390.0002580.000140.002770.000583

0.0002050.000402

0.0001320.000258

0.0001410.000275

0.000180.000351

0.000180.000351

0.0005390.0003990.0007010.05250.0019180.000485

0.000148

0.000148

0.0005390.0003990.0002640.05250.0004170.000485

0.0005390.0003990.0002640.05250.000310.000485

0.000107

0.0001420.000417

0.0001420.000278

0.000139

0.0002950.000936

0.000152

0.0001330.00026

0.0001620.000317

5.4e-05

7.2e-05

8.1e-05

0.0057510.0125530.0055920.0270.0097750.0055740.003602

0.0001420.0001130.0006880.0015760.000115

4.2e-050.0003320.0006493.2e-05

4.2e-050.0003320.0006493.2e-05

5.8e-050.0001139e-050.0004075.2e-05

5.8e-050.0001139e-050.0004075.2e-05

4.2e-050.0002660.000523.1e-05

4.2e-050.0002660.000523.1e-05

0.0056090.012440.0049040.0270.0081990.0054590.003528

2e-050.0002640.0005572.2e-050.000141

7e-064e-057e-067.1e-05

1.3e-050.0002640.0005171.5e-057e-05

0.0041690.0121420.0014130.0270.0008620.0039910.003248

0.0031490.0121420.0012280.0270.0007030.0030180.003248

0.001020.0001850.0001590.000973

8.7e-050.000169

8.7e-050.000169

7.3e-05

7.3e-05

4e-050.0004870.0009533.1e-05

4e-050.0004870.0009533.1e-05

0.0001480.0004330.0008470.000145

7.3e-050.0001760.0003447.2e-05

7.5e-050.0002570.0005037.3e-05

3.2e-050.0002430.0001243e-05

3.2e-050.0002430.0001243e-05

0.0002110.000413

0.0002110.000413

6.6e-05

6.6e-05

5.1e-058.6e-050.0004029.7e-05

4.5e-050.000114.5e-05

8.6e-050.000168

6e-060.0001245.2e-05

0.000840.0003470.0004980.00082

0.000840.0003470.0004980.00082

0.0001870.000468

0.000102

0.0001870.000366

3.6e-051.1e-050.0001462.9e-05

1.8e-051e-051.3e-05

1.8e-051.1e-050.0001361.6e-05

0.0002324.4e-050.0003260.0006230.00026

0.0001870.0001038.9e-050.000216

9.7e-05

4.5e-054.4e-050.0002230.0004374.4e-05

0.000107

0.000107

8.6e-050.000168

8.6e-050.000168

0.0002480.000484

0.0002480.000484

1.9e-053.9e-051.5e-05

1.9e-053.9e-051.5e-05

0.000147

0.000147

2.2e-050.0001321.9e-05

2.2e-050.0001321.9e-05

0.0001030.000202

0.0001030.000202

0.0002170.000425

0.0002170.000425

0.0001840.00036

0.0001840.00036

0.0001010.000197

0.0001010.000197

7.4e-05

7.4e-05

7.4e-05

0.0003830.0009320.002520.000374

0.0003830.0009320.002520.000374

0.0002510.0005050.0011970.000246

5.5e-050.0003820.0007475e-05

0.0001960.0001230.000450.000196

0.0001320.0004270.0013230.000128

0.0001129.8e-050.000680.0001

2e-050.0003290.0006432.8e-05

0.0022510.0006190.0008110.001958.3e-05

0.0022510.0006190.0008110.001958.3e-05

0.0014810.0004740.0005390.001418

0.0014810.0004740.0005390.001418

9e-050.0001259.4e-058.3e-05

9e-050.0001259.4e-058.3e-05

0.000680.0001450.0001470.000438

0.000680.0001450.0001470.000438

0.0014410.0006790.0009260.0024770.001126

0.0014410.0006790.0009260.0024770.001126

0.0003120.000611

0.0003120.000611

0.000152.3e-059.7e-050.0004110.000149

0.000152.3e-059.7e-050.0004110.000149

0.0002730.0001529e-050.0003920.000286

9e-050.000176

0.0001063.5e-056.5e-050.000111

5.5e-057.1e-058.6e-055e-05

0.0001124.6e-056.5e-050.000125

0.0009190.0004260.0002130.0002240.00058

0.0009190.0004260.0002130.0002240.00058

9.9e-057.8e-050.0002140.0008390.000111

9.3e-05

0.0001240.000243

9.9e-057.8e-059e-050.0004350.000111

6.8e-05

0.0002370.0002060.0007620.000272

9.9e-050.0002310.000135

2e-066e-052e-06

1.9e-05

1e-063.8e-052e-06

1e-063e-06

5.9e-054.2e-054.5e-05

2.2e-051e-069e-06

3.6e-053.4e-05

3e-06

1e-063e-052e-06

6e-06

0

2e-06

1.6e-050.0001131.5e-05

1.6e-051.5e-051.5e-05

6e-06

9.2e-05

2.2e-051.6e-057.3e-05

2e-06

7e-063e-061.8e-05

2e-061e-062e-06

1.3e-057e-065.3e-05

3e-06

1.5e-055.2e-051.1e-05

1.5e-055.2e-051.1e-05

1.5e-055.2e-051.1e-05

0.0001237.7e-050.000126

2.1e-058e-061.9e-05

2.1e-058e-061.9e-05

0.0001026.9e-050.000107

2e-06

2.7e-05

9e-0609e-06

6e-0601.1e-05

1e-061e-06

3e-067e-061e-06

9e-06

1e-060

04e-06

1e-063e-061e-06

0

1e-06

6e-062e-068e-06

3e-06

1e-061e-06

1e-061e-061e-06

0

6.4e-052e-066.2e-05

1.1e-051e-058e-06

0.0002060.000402

0.0002060.000402

0.0002060.000402

0.0338260.0409650.0356010.0410.0647510.0375950.009655

0.0189480.0405140.0166810.0410.0253090.0205010.009655

0.0004165.1e-050.0027690.0065740.000451

6.7e-052.5e-050.00060.0011757.8e-05

3e-052.5e-054.3e-05

3.7e-050.00060.0011753.5e-05

0.0002030.000397

0.0002030.000397

0.000139.2e-050.0008770.000129

0.000139.2e-050.0008770.000129

0.0001412.6e-050.0001010.0005140.000164

0.0001412.6e-050.0001010.0005140.000164

0.0002830.000553

0.0002830.000553

7.8e-050.0003990.000788e-05

7.8e-050.0003990.000788e-05

0.000145

0.000145

0.0010910.002133

0.0004110.000804

0.0002260.000441

0.0004540.000888

0.0048127.9e-050.0029090.0038070.006247

0.0002570.000502

9.8e-050.000192

0.0001590.00031

0.0013450.0008560.0005650.001293

0.000136

0.0001820.000356

0.0013450.0006747.3e-050.001293

0.001764.5e-050.0013040.002090.001771

0.0002260.000443

0.001764.5e-050.0004990.0005150.001771

0.0005790.001132

0.0016133.4e-050.0004920.000460.003093

0.0016133.4e-050.0004920.000460.003093

9.4e-050.000199e-05

9.4e-056.2e-059e-05

8e-05

4.8e-05

0.0026536e-050.002560.0048420.003153

4.8e-050.0001683e-05

6.1e-05

4.8e-050.0001073e-05

0.0016440.0004690.0004980.00152

0.0016440.0004690.0004980.00152

6.2e-050.0005180.0010134.3e-05

4.2e-050.0003110.0006082.5e-05

2e-050.0002070.0004051.8e-05

0.0002260.000443

0.0002260.000443

0.0003040.000595

0.0003040.000595

0.0002650.000518

0.0002650.000518

7.1e-055.4e-050.0003530.0005530.000146

7.1e-055.4e-057.5e-051e-050.000146

0.0002780.000543

0.0008286e-060.0004250.0010540.001414

2.7e-050.0001358.7e-05

2e-061e-062e-06

4e-0603e-06

5.8e-05

3e-066e-069e-063e-06

3e-05

0.0007920.0001580.000130.001319

0.000133

0.0001620.000316

0.0001050.000205

2.2e-05

1.5e-05

0.0043996.6e-050.0028930.0047320.004271

0.0003220.00063

0.0001460.000286

0.0001760.000344

0.0001050.000237

3.1e-05

0.0001050.000206

7.6e-059.8e-050.000315.6e-05

7.6e-059.8e-050.0001915.6e-05

0.000119

0.0014350.0006620.0007790.001424

0.0007030.0001210.0001020.000671

4e-060.0001064e-06

4.3e-058.4e-054.9e-05

0.0006640.0003424.3e-050.000674

2.1e-055.4e-052.6e-05

0.0001990.00039

6.8e-050.0001030.0002025.9e-05

6.8e-050.0001030.0002025.9e-05

4.4e-050.000110.0002143.9e-05

4.4e-050.000110.0002143.9e-05

0.0014420.0003550.0003410.001438

0.0014420.0003550.0003410.001438

0.0003360.000658

0.0001650.000323

0.0001710.000335

6.5e-059.7e-050.000194.7e-05

6.5e-059.7e-050.000194.7e-05

1.1e-050.0001180.0002325e-06

1.1e-050.0001180.0002325e-06

3.3e-059.6e-050.0001883e-05

3.3e-059.6e-050.0001883e-05

8.1e-050.000158

8.1e-050.000158

0.0011950.000410.0005090.001146

0.0005880.0001720.0001850.000559

0.0006070.0002380.0003240.000587

3e-056.6e-058.4e-052.7e-05

1.2e-05

2e-053.4e-055e-061.7e-05

1e-053.2e-056.7e-051e-05

0.0066680.0402580.005550.0410.0053540.0063790.009655

0.0001324.3e-050.0001040.0004180.000173

2.6e-051.6e-051.9e-05

3e-053.2e-052.4e-05

2.9e-050.0001040.0002047.6e-05

2.8e-05

0.000101

3.6e-052.9e-054.6e-05

1.1e-054.3e-058e-068e-06

0.0024732.8e-050.0008340.0012040.002411

0.0024072.8e-050.0003760.0003090.002337

6.6e-050.0001750.0003427.4e-05

0.0002830.000553

0.0006376e-060.0006070.0011120.000654

5.6e-050.0001830.0003585.7e-05

0.0001140.000222

2.6e-056e-065.5e-052.5e-05

5.9e-058.6e-05

0.0004960.0001690.0002010.000486

0.0001410.000276

0.002890.0401510.0033590.0410.001210.0026880.009655

2.4e-053.9e-050.0011787e-062.4e-050.002304

5.1e-05

0

3.4e-059.1e-050.0001782.3e-05

7.9e-05

4e-06

2.5e-054.4e-052.4e-05

0.0002651.2e-050.0001351e-050.000263

5e-06

2.7e-05

2.9e-051e-052.6e-05

4e-058.5e-053.4e-05

8e-06

0.000490.0002013.3e-050.000328

1.2e-05

7e-06

0.000144

0.000127

0.0013740.0400660.0015440.0414.3e-050.0013510.007296

2.9e-052.1e-051.4e-05

0.0002550.0001261.7e-050.000239

2.3e-05

1.3e-05

0

4.2e-058e-063.7e-05

1.9e-051.2e-058e-061.3e-05

7.9e-055.8e-057e-057e-06

1.2e-052.2e-051.9e-054e-05

0.0001598.4e-053e-060.0001684.8e-05

6.3e-05

7e-061.9e-052e-06

2.3e-05

8e-06

7e-061e-063.2e-05

7e-06

6e-06

3.4e-05

5e-06

0.0003583e-050.0001140.0002740.000308

0.0001321.2e-055.5e-058.9e-05

7.3e-05

0.0002261.8e-050.0001149.7e-050.000219

4.9e-05

0.0001780.0005320.0011360.000145

3e-05

0.0001788.2e-051.1e-050.000145

1.1e-05

0.0001080.000211

0.0003420.000669

9e-06

9.5e-05

4.6e-05

5.4e-05

0.0030720.0019090.0026770.002921

0.0030720.0019090.0026770.002921

0.0004260.0002910.0008588.8e-05

0.0004260.0002910.0008588.8e-05

0.0013950.0007232.5e-050.001432

0.0013950.0007232.5e-050.001432

0.0012270.0008950.0016540.001298

0.0011280.0002210.0001920.001202

5e-060.0001436e-06

1.9e-050.0001020.00022.5e-05

9.2e-050.00018

7.5e-050.000480.0009396.5e-05

2.4e-050.000140.000103

2.4e-050.000140.000103

0.0047560.0001810.0032580.0061830.003658

0.0025495.4e-050.0023210.0032670.002318

0.0002440.000478

0.0002440.000478

0.0025495.4e-050.0017380.0021270.002318

0.0008450.0003381.5e-050.000542

0.0015180.0004910.000560.001479

0.0004250.000832

0.0001865.4e-050.0001171e-060.000297

0.0003670.000719

0.0003390.000662

0.0003390.000662

0.0022070.0001270.0009370.0029160.00134

0.0006247.5e-050.0001660.0008190.000156

0.0006247.5e-050.0001660.0008190.000156

0.0008175.2e-050.0003510.0010850.000449

0.0008175.2e-050.0003510.0010850.000449

0.0007660.000420.0010120.000735

0.0007660.000420.0010120.000735

0.0004080.0001010.0026390.0053990.000689

4.5e-050.0005820.0011384e-05

4.5e-050.0005820.0011384e-05

4.5e-050.0005820.0011384e-05

0.0001934.6e-050.0015670.0030790.000197

0.0001430.0008060.001590.000135

8e-06

6.5e-050.0004240.000836.4e-05

0.0003820.000747

7.8e-055e-067.1e-05

5e-054.6e-050.0007610.0014896.2e-05

0.0003460.000677

5e-054.6e-050.0004150.0008126.2e-05

0.000175.5e-050.000490.0011820.000452

8.2e-050.0002390.0004677.9e-05

8.2e-050.0002390.0004677.9e-05

8.8e-055.5e-050.0002510.0007150.000373

8.8e-055.5e-050.0002510.0007150.000373

0.0066420.0001690.0111140.0251830.009826

0.0043330.0001690.0050410.0104630.005231

0.0028973.8e-050.001640.0044880.002745

0.0014490.0007880.0019360.001351

0.0014483.8e-050.0008520.0025520.001394

0.0014240.002787

0.0010410.002037

0.0003830.00075

0.0007160.0014

0.0007160.0014

6.5e-059.5e-050.0005280.0001190.001176

3e-054e-050.0001120.000144

3.5e-055.5e-050.0005287e-060.001032

0.0013713.6e-050.0007330.0016690.00131

0.0013713.6e-050.0007330.0016690.00131

0.0006870.0027090.0051970.001215

2.8e-050.0005170.0010119e-05

2.8e-050.000320.0006269e-05

0.0001970.000385

0.0006590.0021920.0041860.001125

2.4e-050.0005950.0011633.4e-05

2.9e-050.0004210.0008232e-05

0.000560.000250.0002980.00102

0.0004780.000934

4.6e-050.0004480.0008765.1e-05

9.2e-05

6.5e-050.0011930.0023348.5e-05

6.5e-050.0011930.0023348.5e-05

6.5e-050.0011930.0023348.5e-05

0.0015570.0011590.0052090.003295

0.000118.9e-050.0014260.000133

0.000118.9e-050.0014260.000133

0.0004770.0004910.0016950.002285

0.0004770.0004910.0016950.002285

0.000970.0005790.0020880.000877

0.000970.0005790.0020880.000877

0.0010120.00198

0.0004390.000859

0.0004390.000859

0.0005730.001121

0.0005730.001121

0.227620.0359270.5071820.5709210.2325580.513964

0.0184580.0021530.0175430.0277670.01977

0.0098380.0013410.0130460.0193470.01173

4.6e-053.4e-050.0007540.0014764.7e-05

0.0006040.001182

4.6e-053.4e-050.000150.0002944.7e-05

0.0008390.001641

0.0008390.001641

0.0097920.0013070.0114530.016230.011683

0.000330.000646

2e-06

4.4e-053.7e-050.0002228.7e-050.000433

0.0005767.7e-050.0002897.7e-050.000554

3e-05

0.0006765.8e-050.000345

0.0003190.000624

0.0007933.4e-050.000210.0001940.001113

0.0009710.001899

7e-06

0.0005840.001142

7.9e-050.000154

0.0001670.000326

0.0003570.0001660.000137.1e-050.000341

4.2e-059.5e-050.001210.0023674.8e-05

6.3e-05

0.0013353.2e-050.0003220.0003070.001331

2e-06

00.0001290.000251

0.0001180.000231

0.0005940.001162

0.0005224.5e-050.0003221.6e-050.000797

6.5e-05

0.000139

0.000137

9e-06

1.8e-05

0.0007780.001522

8.4e-050.000165

0.0012010.0007233.4e-050.00172

0.0001730.000338

9.8e-050.000191

0.000133

0.0002915.1e-050.0002058.1e-050.000648

7e-06

0.0021020.001099.8e-050.002163

0.0001270.000248

0.0006635.7e-050.0003322e-050.000637

7.4e-052.4e-050.0001580.0003088.8e-05

0.0002720.000531

2.5e-05

0.0009130.0003380.0003140.000640.001129

1.3e-054e-059.4e-051.8e-05

0.000116

3.9e-050.0001142.5e-055.4e-05

1e-06

6e-050.00018.6e-051e-060.000505

9.3e-05

0.0001890.000371

1e-06

0.0002020.000394

6.2e-05

0.0002810.000551

2e-06

9.1e-053.9e-050.0001520.000104

0.008620.0008120.0044970.008420.00804

0.0036750.0016340.0026670.003669

0.000740.001447

0.0036750.0004470.0003460.003669

0.0004470.000874

0.0002080.0001480.0016290.0032710.000205

0.0003190.000625

0.0001560.000305

0.0002450.00048

0.000350.000684

8.1e-050.000158

7.6e-056e-067.3e-05

9.1e-056e-050.0001120.000229.3e-05

3e-057.1e-053.1e-05

0.0003660.000716

1.1e-058.8e-056e-068e-06

0.0047370.0006640.0012340.0024820.004166

0.0001990.000389

0.0008070.0001070.0001323e-060.000954

0.000410.0001130.0001081.5e-050.000343

1.5e-05

0.000880.0003270.0003580.001490.001179

0.002640.0001170.0001612.5e-050.00169

0.0002760.00054

5e-06

0.0137760.0020680.0763630.150690.013322

0.0137760.0020680.0763630.150690.013322

0.0045250.0015470.0012090.001660.004522

0.0045250.0015470.0012090.001660.004522

0.0060910.011915

0.0060910.011915

0.0031820.006225

0.0031820.006225

0.0092510.0005210.0067390.0151950.0088

0.001040.002035

0.0030160.0017340.0054780.002701

0.001350.0005210.0005170.0017980.001354

0.0048850.0007590.0006230.004745

0.0026890.005261

0.0030060.00588

0.0030060.00588

0.0561360.109815

0.0025710.005029

0.0535650.104786

0.0395150.006670.0191760.0198650.042570.000933

0.0005260.00103

0.0005260.00103

0.0005260.00103

0.0003490.000683

0.0003490.000683

0.0003490.000683

0.0072450.0002390.0025140.0018330.007151

0.0008323.7e-050.0006110.0004090.001353

0.0001150.000224

0.000743.7e-050.0003541.1e-050.00065

9.2e-050.0001420.0001740.000703

0.0012962.8e-050.0003890.0004240.001238

0.0012962.8e-050.0003890.0004240.001238

0.0001660.000325

0.0001660.000325

0.0012788.3e-050.0001920.0001560.001235

0.0012788.3e-050.0001920.0001560.001235

0.0038167.2e-050.0011560.0005170.003307

2e-05

5.8e-05

0.000130.000254

0.0018177.2e-050.0001650.0001220.001852

0.0019990.0008616.3e-050.001455

2.3e-051.9e-052e-061.8e-05

2.3e-051.9e-052e-061.8e-05

0.002990.0011340.0020510.0010450.0045846.6e-05

0.0001260.000247

0.0001260.000247

0.0006570.0005480.0004155e-060.001205

6e-060.0002870.0001934e-060.000553

0.0006510.0002610.0002221e-060.000652

0.0007990.0002510.0008280.000660.001916

0.0001630.00032

0.000597.6e-050.000454e-060.001727

1.1e-05

5.6e-05

0.0001130.000221

1.9e-05

08.7e-0500

0.0002098.8e-050.0001021e-060.000189

0

2.8e-05

0.0015340.0003350.0006820.0001330.0014636.6e-05

0.0001453e-057.5e-058e-060.000148

0.0003270.0001430.0001151.6e-050.000301

3.5e-05

4.4e-05

0.0008085e-050.0004021.7e-050.000766

0.0002530.0001129e-058e-060.0002483.9e-05

1e-0609e-06

4e-06

0

9e-06

9e-06

1e-06

0.0191920.0035450.0104650.0118790.0198860.000693

0.000380.000743

0.000380.000743

0.0009460.0002060.0006190.0001530.0029441.4e-05

0.0008828e-050.000529e-060.001203

6.4e-050.0001269.9e-050.0001440.0017411.4e-05

0.0015520.0002610.0002430.000360.000857

0.0015520.0002610.0002430.000360.000857

0.0001410.000277

0.0001410.000277

0.0023980.0002430.0002480.0001810.0019840.000283

0.000113

0.0005760.0001250.0001385.6e-050.000707

2.6e-05

4.2e-05

5.7e-05

1.5e-05

0.0018220.0001180.000114.2e-050.0012770.000113

0.0003410.000668

0.0003410.000668

8.3e-05

8.3e-05

0.0012910.0007040.0003950.0001970.0013325.7e-05

2.6e-05

2.5e-05

0

9e-06

0.0004420.0005810.0002482.1e-050.0004521.8e-05

0.000101

0.0008490.0001230.0001471.5e-050.000883.9e-05

4.2e-05

4.2e-05

2.6e-050.0002250.000443.1e-05

2.6e-050.0002250.000443.1e-05

0.0033680.0002590.0017740.0014340.003309

0.0019637.1e-050.0009922.7e-050.001918

8.4e-050.0002080.0004089.7e-05

0.0012930.0001880.0001830.0002340.001273

2.8e-050.0003910.0007652.1e-05

0.0008720.001705

0.0008720.001705

0.0055160.001270.001270.0004290.004429

9.9e-05

1e-06

1e-06

0.0013270.0001890.0002018e-050.000626

0.0001060.000207

3e-06

2e-06

0.0018650.0001680.0002171.3e-050.001753

0.00120.0003370.0002934e-060.000696

4e-06

0.0008380.0004740.000364e-060.001049

0

1e-06

0.0002860.0001029.3e-057e-060.000305

3e-06

8e-057e-057.9e-05

8e-057e-057.9e-05

0.000660.00129

0.000660.00129

2.5e-052.5e-052.8e-051.7e-05

5e-062.1e-055e-06

2e-062e-062e-06

1.6e-051.9e-054e-068e-06

2e-066e-061e-062e-06

4.7e-050.0009710.0020560.000144

0.0002220.000434

8e-061e-051.7e-05

1e-05

0.0004240.000829

4e-060.0003250.0006353e-06

3.5e-050.0001380.000124

0.0009340.0003670.000246.5e-050.0012480.000226

0.0001020.0001718e-069.5e-05

0

3.2e-051e-063.2e-05

0.0006380.000110.0001241.1e-050.0007450.000226

2.1e-05

0.0001628.6e-050.0001161.8e-050.000376

6e-06

0.0007480.0001330.0001542.3e-050.000917

0.0007480.0001330.0001542.3e-050.000917

0.0004930.000964

0.0004930.000964

0.0006277.7e-050.0003734.7e-050.000930.000113

1.4e-05

1.8e-055.5e-052e-061.9e-050.000113

0.0006092.2e-050.0003733.1e-050.000911

0.0016340.0008430.001665

0.0016340.0008430.001665

0.0001260.000247

0.0001260.000247

0.000104

0.000104

5.6e-05

5.6e-05

9.7e-050.000217

9.7e-050.00019

2.7e-05

0.0059310.0009260.0020570.0025390.0051220.000118

3.3e-050.0002750.0005384.5e-05

3.3e-050.0002750.0005384.5e-05

3.3e-050.0001560.0003063.9e-05

3.3e-050.0001560.0003063.9e-05

0.0022930.0006320.0009930.0011530.001557.6e-05

0.0009227.8e-050.000140.0001880.0002187.6e-05

4e-050.0001738.9e-054.7e-054.5e-05

1.3e-050.0001728.8e-056.8e-053.5e-05

0.0005130.0001460.0001421e-060.000498

0.0008056.3e-050.0001990.0001950.000754

0.0003350.000654

0.0035720.0002940.0006330.0005420.0034884.2e-05

0.0002310.0001068.1e-052.8e-050.0001934.2e-05

8.2e-05

0.002490.0001340.0001851e-050.002467

0.0001430.00019.9e-05

0.0007085.4e-050.0003679.8e-050.000729

8.1e-05

5.1e-05

9.2e-05

0.0010950.0002520.0002640.0002590.0010595.6e-05

0.0010950.0002520.0002640.0001720.0010595.6e-05

0.0008768.2e-050.0001579.2e-050.0001871.9e-05

6e-065.3e-056.8e-056e-063.7e-05

0.0002130.0001170.0001073e-060.000866

5e-06

4e-06

1.5e-05

1.5e-05

7.2e-05

7.2e-05

0.0030620.0005740.000950.0005970.004768

0.0011069.1e-050.0005590.0001860.001083

0.000153

1.3e-05

0.0011069.1e-050.0005592e-050.001083

0.0009340.0003390.0002450.0001490.002593

7e-060.0001141e-061.2e-05

8.4e-050.0001079.4e-051.3e-050.000638

6.9e-05

0.0008370.0001180.0001516.5e-050.001936

6e-061e-067e-06

0.0010220.0001440.0001460.0002620.001092

0.0008715.9e-050.0001460.000120.000951

0.0001518.5e-050.0001250.000141

1.7e-05

0.0010180.001992

0.0010180.001992

0.0010180.001992

0.0010180.001992

0.0008260.001616

0.0008260.001616

0.0005460.001069

0.0005460.001069

0.000280.000547

0.000280.000547

0.0069820.0001860.0025040.0056070.0072475.5e-05

0.0069820.0001860.0025040.0056070.0072475.5e-05

0.0036657.9e-050.0016770.0010850.0034465.5e-05

0.0011293.9e-050.0005353.4e-050.000976

0.0015170.0002280.0001860.0014845.5e-05

0.0004020.000787

0.0010194e-050.0005126.7e-050.000986

1.1e-05

0.002560.0001070.0001920.0019560.002385

0.002560.0001070.0001920.0019560.002385

0.0007570.0006350.0025660.001416

0.0007570.0006350.0025660.001416

0.0086150.0016160.0048350.0087540.0088775.6e-05

0.0086150.0016160.0048350.0087540.0088775.6e-05

0.0063580.0015170.0024670.0045980.0065585.6e-05

0.0001090.000212

8.8e-05

0.000530.001038

0.0001740.0001110.0005260.00014

0.0002860.0001950.0001278.2e-050.0002895.6e-05

2.6e-05

0.000750.0001540.0001670.0004380.000461

0.0003044.2e-050.0001358e-050.000234

0.0002450.00048

6.1e-058.8e-059.1e-055.8e-05

8.8e-05

0.0001370.000268

0.0001296.4e-050.0002190.000126

0.0002246e-050.0001212.1e-050.000251

0.0002250.000441

0.0022310.0001770.0001310.000110.002218

0.0014060.0001370.0001140.0001090.001384

0.0003820.0003750.0001855.4e-050.000333

0.0004110.0001140.0001262e-060.001064

0.0001150.000225

0.0010340.002023

0.0010340.002023

9.4e-056.1e-050.0003780.0008470.000115

5.7e-053e-050.0001820.0003566.5e-05

3.7e-053.1e-050.0001960.0003845e-05

0.000107

0.0021633.8e-050.0009560.0012860.002204

0.000270.000528

0.0021633.8e-050.0006860.0007580.002204

0.0367920.0070750.0365820.0977390.035313

0.003470.006788

0.003470.006788

0.003470.006788

0.0024790.0001660.0002950.0081230.00267

0.0024790.0001660.0002950.0081230.00267

0.0024790.0001660.0002950.0081230.00267

0.0207260.0053760.0171580.0439580.02052

0.0142640.0010870.001380.0134060.014505

0.0017680.0007450.0007390.0033380.001788

0.0072920.0001790.0003350.0041730.007104

0.0052040.0001630.0003060.0058950.005613

0.0066450.013

0.0066450.013

0.0064620.0042890.0027090.0049860.006015

0.0064620.0042890.0027090.0049860.006015

0.0064240.012566

0.0064240.012566

0.0059250.0009160.0038540.008270.006822

0.0059250.0009160.0038540.008270.006822

4e-06

0.0001420.0001520.0001060.0027520.00019

0.0028080.005493

0.0057830.0007640.000942.1e-050.006632

0.0049240.0004810.0034260.0112850.002831

0.0049240.0004810.0034260.0112850.002831

0.0014190.002776

0.0012770.002497

0.0049240.0004810.000730.0060120.002831

0.0027380.0001360.0083790.0193150.00247

0.0015740.003079

0.0015740.003079

0.0002810.00055

0.0002810.00055

0.0002230.0001040.0001862.2e-05

0.0002230.0001040.0001862.2e-05

0.0019286.1e-050.0060330.0119230.00191

0.0013150.002572

0.0014090.002756

0.0019286.1e-050.001010.0020980.00191

0.0012350.002415

0.0010640.002082

0.0005667.5e-050.0003870.0035190.000524

0.0005667.5e-050.0003870.0035190.000524

2.1e-055.8e-051.4e-05

2.1e-055.8e-051.4e-05

0.0002988.8e-050.003090.0058150.000306

1.4e-053.6e-050.0013830.0027052.1e-05

1.4e-053.6e-050.0008790.0017192.1e-05

0.0007910.001547

1.4e-053.6e-058.8e-050.0001722.1e-05

0.0005040.000986

0.0005040.000986

0.0002845.2e-050.0017070.003110.000285

0.0003090.000604

0.0003090.000604

0.0002845.2e-050.0013980.0025060.000285

0.000150.000293

2.1e-050.0003060.0005994.9e-05

0.0003470.000679

0.0004680.000915

0.0002635.2e-050.0001272e-050.000236

0.0013170.002577

0.0013170.002577

0.0013170.002577

0.0013170.002577

0.0009910.0005420.0013830.000911

0.0009910.0005420.0013830.000911

0.0009910.0005420.0013830.000911

0.0009910.0005420.0013830.000911

0.0153210.0016330.0175430.0523160.01524

0.0060040.0004410.0084460.026390.006491

0.0001289e-050.0019850.000114

0.0001289e-050.0019850.000114

0.0001713.1e-050.0001710.0024280.000366

0.0001713.1e-050.0001710.0024280.000366

0.005250.002220.0038920.004305

0.0020620.001080.0021330.002144

0.0031880.001140.0017590.002161

0.0015130.00296

0.0015130.00296

0.0019890.00389

0.0019890.00389

0.0004550.000410.0024630.0112350.001706

0.0002330.0001350.000120.0016740.000214

0.0001560.0001840.0001610.0052920.001492

0.0011820.002313

0.0007130.001394

6.6e-059.1e-050.0002870.000562

0.0025220.004933

0.0025220.004933

0.0025220.004933

0.0022380.0012640.0019360.001948

0.0022380.0012640.0019360.001948

0.0022380.0007730.0009760.001948

0.0004910.00096

0.0010620.002077

0.0010620.002077

0.0010620.002077

0.0070790.0011920.0042490.016980.006801

0.005910.0009040.001950.0075140.005668

0.0022570.00050.0006040.0021070.002144

0.0013633.9e-050.000840.0029780.001322

0.002290.0003650.0005060.0024290.002202

0.0014530.002843

0.0014530.002843

0.000999.7e-050.0006310.0026750.000955

0.000999.7e-050.0006310.0026750.000955

9.8e-056.9e-059.9e-050.0021970.000101

9.8e-056.9e-059.9e-050.0021970.000101

8.1e-050.0001220.0001160.0017517.7e-05

8.1e-050.0001220.0001160.0017517.7e-05

0.0009870.0003030.0019470.0060130.000884

0.0007470.0001580.0007420.0024220.000609

6.8e-051e-059.1e-057.3e-05

6.8e-051e-059.1e-057.3e-05

0.0003542.1e-050.0001990.000290.000146

0.0003542.1e-050.0001054.5e-050.000146

6.1e-05

0

9.4e-050.000184

0.0002750.000675

0.0001910.000374

0.000136

8.4e-050.000165

0.000165e-050.0002680.0009130.0002

0.0001085e-050.0003880.000109

5.2e-050.0002680.0005259.1e-05

0.0001657.7e-050.0004530.00019

7.2e-053.1e-053.3e-057.2e-05

5.2e-054e-065e-05

0.000115

1.8e-051.6e-059e-064.2e-05

1e-067e-060

5e-05

01.8e-051e-06

1e-051.2e-055.1e-051.4e-05

1.2e-059.6e-051.2e-05

0

8.7e-05

0.000240.0001450.0012050.0035910.000275

0.0006130.00129

0.0003730.00073

9e-05

0.000240.00047

0.000149.6e-050.00010.0007190.000148

0.000149.6e-050.00010.0007190.000148

0.000380.000743

0.000380.000743

0.00014.9e-050.0001120.0008390.000127

0.00014.9e-050.0001120.0008390.000127

0.0157790.0021170.2859650.0791430.0150050.51292

0.0067420.013188

0.0067420.013188

0.0032530.006363

0.0034890.006825

0.261660.511875

0.261660.511875

0.261660.511875

0.0145030.0276740.000699

0.0069890.013673

0.0069890.013673

0.0071570.014001

0.0071570.014001

0.0003570.000699

0.0003570.000699

0.0157790.0021170.003060.0382810.0150050.000346

0.0044460.0005890.0009210.0131860.004536

0.0044460.0005890.0009210.0131860.004536

0.0045080.000420.0006880.0078460.004196

0.0045080.000420.0006880.0078460.004196

0.0037770.0008480.0011130.0091720.003518

0.0037770.0008480.0011130.0091720.003518

0.0030480.000260.0003380.0080770.0027550.000346

0.0030480.000260.0003380.0080770.0027550.000346

0.0492640.0076980.0225150.0593930.050398

0.0036990.0003190.0040970.0082360.002161

0.0036990.0003190.0040970.0082360.002161

0.0008876.4e-050.0004417e-060.000839

0.0006690.00131

0.0019330.003781

0.0004320.000845

0.0001610.000315

0.0027720.000230.0003330.0015550.001271

4e-052.5e-051.2e-055.1e-05

0.000143

0.0001280.000251

1.7e-05

0.0022360.0001960.000430.0049390.00193

0.0022360.0001960.000430.0049390.00193

0.0017220.0001080.0001890.0024740.001681

0.0005148.8e-050.0002410.0024650.000249

0.0034260.0001290.0011730.0055060.00373

0.0034260.0001290.0011730.0055060.00373

0.0034260.0001290.0002380.0036770.00373

0.0009350.001829

0.0155290.0041160.0038350.0089240.017486

0.0155290.0041160.0038350.0089240.017486

2.1e-050.000390.0001994e-062.6e-05

0.0007080.0002460.0002230.000715

0.0008480.0005110.0004060.0013850.000848

0.001920.0001190.0001680.000380.001678

0.0013160.0003680.0004260.0016210.001408

0.0011950.0002760.000310.0011010.000817

0.0009240.000150.0001270.0001420.000779

6.9e-055.8e-050.0003660.001490.000471

0.0015440.0002340.0002530.0003730.002093

0.003510.0002010.000310.0009430.003568

0.002130.0004590.0004930.0008950.002587

3e-060.0001297e-064e-06

1.6e-057.9e-056e-061.7e-05

3.9e-055.2e-050.0001170.0002460.000214

0.0003890.0001550.0001373.1e-050.000464

0.0005350.0005020.0001690.0001490.000675

0.0003620.0001870.0001310.0001510.001122

0.0026070.0004680.0006160.0021010.003081

0.0026070.0004680.0006160.0021010.003081

0.0026070.0004680.0006160.0021010.003081

0.0040240.0002840.0004570.0022270.003851

0.0040240.0002840.0004570.0022270.003851

0.0040240.0002840.0004570.0022270.003851

0.0177430.0021860.0103740.0244610.018159

0.0139010.0015130.0051340.009880.014293

0.000390.000764

0.0002080.000406

0.0011030.002158

0.0012490.002443

0.0004990.000976

0.0043750.0010480.0010520.0018590.004481

0.0068090.0001870.0002640.0004780.006626

0.0027170.0002780.0003690.0007960.003186

0.0001042.9e-050.0001020.0023339.8e-05

0.0001042.9e-050.0001020.0023339.8e-05

0.0018810.0005350.0007010.0011470.001808

0.0009270.0002520.0001660.0001660.000835

1e-06

0.0001750.000342

0.0002340.0001026.2e-050.000174

0.000720.0002830.0002580.0005740.000799

1e-06

1e-06

0.0018570.0001090.0009580.0042960.00196

0.0004814.5e-050.0003810.0034580.000602

0.0013766.4e-050.0005770.0008380.001358

0.0017770.003476

0.0017770.003476

0.0017020.003329

0.0017020.003329

0.0015330.002999

0.0015330.002999

0.0015330.002999

0.002020.0004650.0041090.0124970.001964

0.002020.0004650.0041090.0124970.001964

0.0007580.0003620.0003650.0048570.000762

0.0007580.0003620.0003650.0048570.000762

3.9e-053.7e-050.0019360.0037874e-05

3.9e-053.7e-050.0019360.0037874e-05

0.0012236.6e-050.0018080.0038530.001162

0.0012236.6e-050.0006690.0016250.001162

0.0005610.001097

0.0005780.001131

0.008720.0001530.00360.0037950.013799

0.008720.0001530.00360.0037950.013799

0.000212.7e-050.0002690.0002990.000844

5.5e-050.000190.0002410.000807

0.0001552.7e-057.9e-055.8e-053.7e-05

6.2e-050.0001560.0001610.00284

6.2e-050.0001560.0001610.00284

0.0015670.000170.0001240.002855

0.0015670.000170.0001240.002855

0.0049150.0001260.0008430.0008890.004005

0.0001014.8e-058e-05

0.0001428.2e-050.0001837.4e-05

8.3e-053.2e-055e-060.000105

0.0045899.4e-050.0007610.0006530.003746

1e-050.0001860.0001240.000364

2.2e-05

7.9e-05

1e-050.0001862.3e-050.000364

6.1e-050.0004460.0009040.000843

6.1e-050.0004460.0009040.000843

3.2e-050.0001224.3e-05

3.2e-050.0001224.3e-05

0.0018380.0013820.0008820.001976

0.0014470.0007371.8e-050.001437

0.0001830.000359

0.0003470.0002087e-060.00049

4.4e-050.0002540.0004984.9e-05

2.5e-050.0001480.000292.9e-05

2.5e-050.0001480.000292.9e-05

0.0101020.0037020.0077070.0339590.006952

0.0026270.005139

0.0016920.00331

0.0016920.00331

0.0009350.001829

0.0009350.001829

0.0090990.0036880.0041650.0279340.005661

0.0016030.0002850.000410.0043760.001598

0.0016030.0002850.000410.0043760.001598

0.0071470.0006940.0010210.0130180.00356

0.0037680.0003950.0005250.008093

0.0033790.0002990.0004960.0049250.00356

0.000260.00051

0.000260.00051

0.0003220.0002540.0007590.0030940.000471

0.0005470.001071

0.0003220.0002540.0002120.0020230.000471

2.7e-050.0024550.0017150.0069363.2e-05

4e-060.0011410.0006260.0013229e-06

2.3e-050.0013140.0010890.0056142.3e-05

0.0010031.4e-050.0009150.0008860.001291

0.0003310.000647

0.0003310.000647

0.0010031.4e-050.0005840.0002390.001291

8.1e-050.000159

0.0001095.1e-054.7e-05

8e-06

4.8e-051.2e-055.1e-05

0.0007230.0004192e-060.000947

0.0001231.4e-058.4e-057e-060.000246

0.000450.0001930.0005318.9e-05

0.0003830.0001930.0003711.6e-05

0.0003830.0001930.0003711.6e-05

0.0003830.0001930.0003711.6e-05

0.0003830.0001930.0003711.6e-05

0.0003830.0001930.0003711.6e-05

6.4e-05

6.4e-05

6.4e-05

6.4e-05

6.4e-05

6.7e-059.6e-057.3e-05

6.7e-059.6e-057.3e-05

6.7e-059.6e-057.3e-05

6.7e-059.6e-057.3e-05

6.7e-059.6e-057.3e-05

0.0001550.0001450.0005860.000171

0.0001550.0001450.0005860.000171

0.0001550.0001450.0005860.000171

0.0001550.0001450.0005860.000171

2e-050.0001022.7e-05

2e-050.0001022.7e-05

6.4e-057.7e-059.2e-05

6.4e-057.7e-059.2e-05

3.7e-056e-053.2e-05

3.7e-056e-053.2e-05

6.3e-05

6.3e-05

3.4e-050.0001450.0002842e-05

3.4e-050.0001450.0002842e-05

0.0027250.0001890.0017940.0033430.002644

0.0027250.0001890.0017940.0033430.002644

0.0002960.00030.000750.000273

0.0002960.00030.000750.000273

0.0002770.0001270.0004110.000258

0.000106

01e-05

2e-066.4e-052e-06

1.1e-05

0.0002750.0001270.000220.000256

1.9e-050.0001730.0003391.5e-05

1.9e-050.0001730.0003391.5e-05

0.0011320.0005387.3e-050.001046

0.0011320.0005387.3e-050.001046

0.0011320.0005387.3e-050.001046

2.5e-052.7e-052.2e-05

1e-068e-061e-06

2e-053.8e-054e-06

0.0010860.00053800.001019

0.0012970.0001890.0009560.002520.001325

0.0001552.5e-050.000145

1.6e-051e-061.5e-05

1e-06

1.6e-0501.5e-05

0

00

0

0.0001392.4e-050.00013

1e-06

2e-0601e-06

1e-061e-061.2e-05

1.1e-05

5e-062e-06

1.1e-051e-05

0.000121e-060.000115

0.0011420.0001890.0009560.0024950.00118

1.8e-050.0002741.5e-05

1.1e-058e-051.1e-05

1e-067.2e-051e-06

6e-060.0001223e-06

3e-069e-050.0001763e-06

3e-069e-050.0001763e-06

1.5e-050.000480.0009392.3e-05

1.5e-050.000480.0009392.3e-05

2.8e-050.0001820.0004662.1e-05

1.2e-050.000116e-06

1.6e-050.0001820.0003561.5e-05

0.0010780.0001890.0002040.000640.001118

7e-06

1e-055e-051e-05

7e-065.9e-057e-06

6.5e-05

1e-06

1.1e-053.9e-051e-05

2e-068.5e-050.0001664.8e-05

5e-069.4e-051.2e-05

6e-062.4e-053e-06

3.4e-05

0.0010370.0001890.0001190.0001010.001028

6.8e-050.000540.0010565.3e-05

6.8e-050.000540.0010565.3e-05

6.8e-050.000540.0010565.3e-05

6.8e-050.000540.0010565.3e-05

6.8e-050.000540.0010565.3e-05

6.8e-050.000540.0010565.3e-05

0.0003630.0004330.0007460.000102

0.0003630.0004330.0007460.000102

0.0002950.0001493.6e-052.6e-05

0.0002950.0001493.6e-052.6e-05

0.0002950.0001493.6e-052.6e-05

1e-061.1e-051e-06

3e-062e-06

2e-0601e-06

0

04e-062e-06

6e-06

0.0002910.0001492e-061.9e-05

1e-066e-061e-06

4e-06

00

6.8e-050.0002840.000717.6e-05

1e-067.5e-051e-06

1e-067.5e-051e-06

1e-067.5e-051e-06

1.2e-057.6e-050.0001481.2e-05

1.2e-057.6e-050.0001481.2e-05

1.2e-057.6e-050.0001481.2e-05

5.5e-050.0002080.0004876.3e-05

1e-060.0002080.0004061.2e-05

1e-060.0002080.0004061.2e-05

5.4e-058.1e-055.1e-05

8.1e-05

5.4e-055.1e-05

0.0035980.0001710.0021260.0052880.003285.3e-05

0.0035250.0001710.0019670.0049770.003211

4e-060.0001145e-06

4e-060.0001145e-06

4e-060.0001145e-06

4e-060.0001145e-06

7.3e-05

7.3e-05

7.3e-05

7.3e-05

0.0004250.0001910.0004690.000158

4.2e-050.0002755e-05

3e-065e-06

3e-065e-06

2.7e-050.0001831.3e-05

6.6e-05

8e-062.6e-058e-06

1.9e-059.1e-055e-06

1e-064e-061e-06

1e-064e-061e-06

1.1e-058.8e-053.1e-05

1e-061.7e-051.2e-05

3e-062.4e-051e-05

4e-061.1e-054e-06

3e-05

3e-066e-065e-06

0.0003830.0001910.0001840.000108

0.0003740.0001912.6e-059.8e-05

0.0003740.0001912.6e-059.8e-05

9e-060.0001581e-05

4e-061.7e-055e-06

5e-062.1e-055e-06

0.00012

1e-05

1e-05

1e-05

7.6e-050.0001457.3e-05

2.5e-050.0001062.6e-05

2.5e-050.0001062.6e-05

2.5e-050.0001062.6e-05

5.1e-053.9e-054.7e-05

5.1e-053.9e-054.7e-05

5.1e-053.9e-054.7e-05

3.4e-050.0001480.000553.7e-05

4e-060.0001480.0004143e-06

0.0001480.000305

0.0001480.000289

1.6e-05

4e-060.0001093e-06

4e-060.0001093e-06

6e-060.0001021.1e-05

6e-062.1e-059e-06

6e-062.1e-059e-06

8.1e-052e-06

8.1e-052e-06

1e-061e-051e-06

1e-061e-051e-06

1e-063e-061e-06

7e-06

2.3e-052.4e-052.2e-05

2.3e-052.4e-052.2e-05

2.3e-052.4e-052.2e-05

0.0021350.0007210.001020.002008

0.000870.0002390.0002470.000819

0.000870.0002390.0002470.000819

0.000870.0002390.0002470.000819

0.0004320.0001440.000160.000481

0.0004320.0001440.000160.000481

0.0004320.0001440.000160.000481

6.1e-050.0003076.7e-05

6.1e-050.0003076.7e-05

3e-066.5e-053e-06

3e-068.2e-055e-06

3.2e-057.2e-053.5e-05

1e-061e-061e-06

1.8e-058.6e-051.9e-05

4e-061e-064e-06

0.0004450.0002270.0001050.000449

0.0004450.0002270.0001050.000449

0.0004430.0002271.1e-050.000447

2e-069.4e-052e-06

0.0003270.0001110.0002010.000192

00.0001181.3e-05

00.0001181.3e-05

0.0003110.0001118.3e-050.000167

0.0003110.0001118.3e-050.000167

1.6e-051.2e-05

1.6e-051.2e-05

0.0008510.0001710.0009070.0026060.00093

1.9e-050.0001430.0003764e-06

1.9e-050.0001430.0003764e-06

6.5e-05

3.1e-05

1.9e-050.0001430.000284e-06

8e-065.5e-055e-06

8e-065.5e-055e-06

8e-065.5e-055e-06

6e-060.0001660.0003254e-06

6e-060.0001660.0003254e-06

6e-060.0001660.0003254e-06

0.0007870.0001710.0005980.001750.000887

0.0007010.0001710.0005170.001430.000808

1.1e-0501.1e-05

3.2e-051.6e-054.2e-05

7.4e-052e-067.1e-05

2e-054.5e-051.5e-05

6e-06

0.0004050.0001710.0001460.0002780.000483

2e-060.0001070.0002092e-06

3e-060.000111e-05

0.0001014e-050.000105

3e-056.2e-053.2e-05

2e-06

0.0001750.000342

5e-068.9e-050.0001737e-06

4e-064e-061.8e-05

3e-06

1e-06

4.5e-05

5e-05

1.4e-051.5e-051.2e-05

4e-06

2.3e-05

6.7e-050.000146.7e-05

6.7e-050.000146.7e-05

1e-052.1e-053e-06

1e-052.1e-053e-06

9e-068.1e-050.0001599e-06

6e-068.1e-050.0001595e-06

3e-064e-06

1.6e-056.6e-051.9e-05

1.6e-056.6e-051.9e-05

1.5e-05

7e-06

1.6e-054.4e-051.9e-05

5e-061e-05

5e-061e-05

5e-061e-05

1e-053.4e-051e-06

1e-053.4e-051e-06

1e-058e-061e-06

2.6e-05

7.3e-050.0001590.0003116.9e-055.3e-05

7.3e-050.0001590.0003116.9e-055.3e-05

7.3e-050.0001590.0003116.9e-055.3e-05

7.3e-050.0001590.0003116.9e-055.3e-05

4e-058.3e-050.0001625.1e-055.3e-05

3.3e-057.6e-050.0001491.8e-05

0.0022050.000910.0015130.001067

0.0022050.000910.0015130.001067

0.0022050.000910.0015130.001067

0.0022050.000910.0015130.001067

1.2e-050.0002330.0004551e-05

1.2e-050.0002330.0004551e-05

0.0008280.0003010.0003840.000777

0.0008280.0003010.0003840.000777

0.0013080.0001930.0003160.000248

0.0013080.0001930.0003160.000248

5.7e-050.0001830.0003583.2e-05

5.7e-050.0001830.0003583.2e-05

0.3322160.4379690.3008020.54370.1216140.3386340.385891

0.0072920.0046640.1837930.035570.0073390.319433

0.0072920.0046640.1837930.035570.0073390.319433

0.0072920.0046640.1837930.035570.0073390.319433

0.000670.001311

0.000670.001311

0.0070020.0025320.0022590.007093

0.0070020.0025320.0022590.007093

0.0001130.00022

0.0001130.00022

0.1804570.0335870.319433

0.1632880.319433

0.0171690.033587

0.000290.0021320.0002940.0004520.000246

0.0001570.000307

0.000290.0021320.0001370.0001450.000246

0.3106570.382140.0769120.47740.0637750.3184790.044607

0.2828950.3462650.0554730.41630.0545340.2914860.038434

9.8e-059.6e-050.00030.000201

6.2e-05

6.2e-05

9.8e-059.6e-050.0002380.000201

1.4e-059.6e-050.0001881.2e-05

1.6e-051.9e-054.4e-05

4.3e-056e-069.8e-05

2.1e-05

2.5e-054e-064.7e-05

0.0001915e-064.9e-050.000203

0.0001915e-064.9e-050.000203

5.9e-051.2e-055.4e-05

8.9e-051.8e-059.1e-05

8e-065e-062e-061.1e-05

9e-066e-062.2e-05

2.6e-051.1e-052.5e-05

4.9e-058.4e-050.0002368e-05

3.1e-058.4e-050.0001636.7e-05

3.1e-058.4e-050.0001636.7e-05

1.8e-057.3e-051.3e-05

1.8e-057.3e-051.3e-05

3.7e-053.8e-054.5e-05

3.7e-053.8e-054.5e-05

3.7e-053.8e-054.5e-05

8.8e-050.000180.0006917.5e-05

0.000101

9e-06

1e-05

1.5e-05

1.6e-05

5e-06

8e-06

1.5e-05

4e-06

1.9e-05

8.8e-054.1e-057.5e-05

8.8e-054.1e-057.5e-05

0.000141

0.000141

7.8e-050.000153

7.8e-050.000153

5.6e-05

5.6e-05

0.0001020.000199

0.0001020.000199

0.1969620.216770.0318980.34360.0359050.2088550.036056

0.196580.2166690.0317220.34360.0351650.2084140.036056

0.1418950.0987680.0219940.21910.0266520.1792240.013402

0.0001470.000287

6.6e-05

0.0534160.1176440.0076620.12450.003510.0278070.022654

5.9e-05

7.1e-05

0.0001

0.0001380.00027

0.000123

9.9e-050.000194

6.6e-05

0.0006180.0001810.0001580.0002450.000575

0.0001380.000271

4.9e-05

0.000136

4.2e-05

0.0001210.000238

0.000108

0.0001539e-050.0001740.000211

0.0004325.8e-050.0001720.0002140.000518

7.9e-05

6.6e-051.8e-055.7e-057.9e-05

0.0001540.000302

0.000130.000255

0.0001640.000321

0.000135

5.5e-05

0.0005550.001086

0.0002510.0001019.3e-050.0003760.000279

0.0002510.0001010.0001940.000279

9.3e-050.000182

0.000151

0.000151

0.0001318.3e-050.0002130.000162

0.0001318.3e-050.0002130.000162

0.085060.129490.023060.07270.0166720.0815060.002378

5.8e-055.6e-056.3e-05

5.8e-055.6e-056.3e-05

0.0023690.0003970.0011210.0020180.002178

4e-050.0001175.5e-05

9e-068e-068e-062.2e-05

0.0001680.000329

7.2e-05

3.2e-053.8e-054.7e-05

4e-061.1e-051e-064e-06

2.9e-05

2e-052.3e-051e-062.1e-05

6e-061.6e-059e-06

4.5e-055e-061e-063.3e-05

5.4e-05

9.9e-05

0.000460.0001084.9e-050.000137

4.3e-05

0

6e-067.6e-051e-067e-06

0.0001032.6e-057.9e-050.0001840.000235

2.1e-05

7e-06

2.9e-052.5e-053e-065.4e-05

0

6.1e-051.7e-054.4e-05

1.1e-050.0001031e-05

6.9e-05

6e-057e-06

8.2e-055.9e-057.5e-05

1e-06

3.6e-05

9.2e-05

9.8e-050.000191

0.0008630.0004450.000878

01.4e-0500

9e-0601.1e-05

0.0002490.0001880.0001196e-060.000282

0.0001040.000203

0

0.0001750.0001049.9e-05

1.1e-052.1e-0504e-05

4.1e-05

9.4e-051.6e-050.000115

4e-064e-062e-06

4e-064e-062e-06

5.1e-05

6e-06

8e-06

3.7e-05

4.3e-05

4.3e-05

0.0812590.1290930.0214970.07270.0133850.07790.002378

1.8e-05

2.1e-05

1e-064e-061.3e-05

1.8e-05

6.1e-050.0001844.5e-053.8e-050.000359

4.7e-05

1.1e-050.0001939.9e-051.6e-051.3e-05

1e-068.9e-05

7.7e-053e-050.0001386e-05

9e-05

2e-061.5e-053e-06

0.0008630.0002180.0002685e-060.0008470.001407

2.6e-05

0.0802410.1286820.0207550.07270.0131330.076843

3e-060.0001916e-065e-060.000373

1.4e-050.0001480.000291.8e-05

1.4e-050.0001480.000291.8e-05

5.3e-05

3.1e-05

2.2e-05

0.000136

0.000136

0.000114

0.000114

2.8e-050.0001383.7e-05

2.8e-050.0001383.7e-05

7.9e-050.000155

7.9e-050.000155

0.0013280.0002150.0002290.001308

0.0013280.0002150.0001780.001308

3e-05

2.1e-05

0.000410.0001550.0006430.000521

8.7e-053.8e-059.9e-05

8.7e-053.8e-059.9e-05

0.0001550.000303

0.0001550.000303

0.0003230.0003020.000422

1.3e-051e-067e-06

7.7e-05

5e-06

4e-063e-066.5e-05

3.9e-053.9e-057.8e-05

4e-06

8.7e-053e-069.8e-05

0.0001381.3e-050.000133

2e-066e-061e-06

4e-061.1e-058e-06

1e-05

1.1e-052.1e-058e-06

2.4e-056e-062.3e-05

1.5e-05

2e-06

1e-061e-061e-06

7.8e-05

7e-06

0.0277620.0358750.0214390.06110.0092410.0269930.006173

0.0002660.0001890.000257

0.0002660.0001890.000257

0.0001032.1e-050.000102

0.0001198.2e-050.000117

6.7e-05

4.4e-051.9e-053.8e-05

0.0007130.0136180.02620.0008980.000837

0.0006770.0134910.02620.0005710.000774

1e-062e-064e-05

4.5e-052.5e-054.2e-05

0.0002189.8e-052e-060.000171

8e-06

1.4e-05

7e-066e-067e-06

2e-064.5e-05

1e-063e-061e-06

2.1e-059e-062.1e-05

6.3e-051.1e-057.4e-05

1.6e-053e-062.6e-05

1e-062.5e-051e-06

1e-06

7e-062e-069e-06

3.2e-05

7e-063e-066e-06

7e-061e-062e-06

3.7e-052.4e-053.4e-05

4.2e-05

5.8e-051.1e-054.2e-05

1e-061.6e-052e-06

3.9e-051.2e-055.1e-05

4e-061.7e-051.7e-05

9e-064e-068e-06

1.6e-050.0133930.02621.6e-058e-06

4.5e-059.9e-058.6e-05

3.2e-05

2e-062.4e-052e-06

1e-063.6e-056e-06

2e-067e-066e-06

4.3e-05

1e-0600

3e-062.3e-054e-06

6e-051e-055.9e-05

5e-066e-064e-06

3.6e-050.0001270.0003276.3e-05

0.0001270.000249

2e-063.2e-053.7e-05

3.4e-054.6e-052.6e-05

0.0006221.8e-050.0002060.0002460.000282

8e-062.4e-053.6e-05

8e-062.4e-053.6e-05

0.0005911.8e-050.0002060.0001990.000232

4.3e-05

0.0001261.7e-050.000126

8e-061.2e-054e-06

3.4e-051e-053.1e-05

2.1e-051.8e-052.4e-052.2e-05

0.0004020.0002061.3e-054.9e-05

8e-05

2.3e-052.3e-051.4e-05

2.3e-052.3e-051.4e-05

0.0018760.0009960.0011180.00239

0.0018490.0009960.0010740.002379

04e-060

3e-062.2e-052e-06

2.2e-051e-061.9e-05

7.1e-051.9e-058.1e-05

2e-06

1.5e-05

3.4e-05

1.9e-05

1.1e-052e-061.2e-05

9e-061.2e-05

6e-06

2.1e-05

2e-053e-061.9e-05

0.000144

3.8e-051.6e-050.00013

1.3e-056e-065e-06

1.2e-05

1.1e-051e-051.5e-05

1e-06

8e-063e-062.2e-05

1e-05

3.8e-05

1.1e-051e-052.3e-05

8e-06

2.4e-059.3e-050.0001817e-06

3e-065e-061.5e-05

0.0001060.000208

0.001360.0007976e-060.001828

3e-063e-061e-06

4.2e-05

6.1e-051.5e-055.3e-05

2.9e-05

5.5e-055.3e-051.2e-05

0.0001262.1e-050.000123

0.000105

2.7e-054.4e-051.1e-05

1.3e-05

3e-061e-052e-06

2.4e-052.1e-059e-06

2.3e-050.0003962.9e-05

2.3e-050.0003962.9e-05

0.000115

5.1e-05

9.8e-05

2.3e-052.5e-052.9e-05

2.9e-05

7.8e-05

0.0242620.0358570.0066190.03490.0063940.0231980.006173

6.1e-055.3e-055.4e-05

1e-061e-061e-06

3e-061e-052e-06

2e-06

5e-061e-051e-06

1.5e-05

5.1e-057e-063e-05

1e-068e-062e-05

8e-054.9e-057.7e-05

8e-054.9e-057.7e-05

0.0241210.0358570.0066190.03490.0062920.0230670.006173

0.0241210.0358570.0066190.03490.006250.0230670.006173

3.6e-05

6e-06

0.0004720.0003850.0017460.000483

7.3e-050.0002167e-05

7.3e-050.0002167e-05

7.3e-050.0002167e-05

6.8e-050.0001076.7e-05

5e-060.0001093e-06

0.0002250.0007780.000185

0.0002250.0005590.000185

0.0002250.0005590.000185

1e-05

0.0001070.0003510.0001

0.0001180.0001228.5e-05

3.7e-05

3.9e-05

0.000219

0.000219

0.000143

7.6e-05

0.0001740.0003850.0007520.000228

0.0001740.0003850.0007520.000228

9.6e-057.4e-050.0001440.000147

9.6e-057.4e-050.0001440.000147

7.8e-050.0001770.0003468.1e-05

7.8e-050.0001770.0003468.1e-05

0.0001340.000262

0.0001340.000262

8.3e-050.000162

8.3e-050.000162

8.3e-050.000162

8.3e-050.000162

8.3e-050.000162

6.3e-050.0001780.0005564.8e-05

6.3e-050.0001780.0005564.8e-05

6.3e-050.0001780.0005564.8e-05

9.2e-050.00018

9.2e-050.00018

4.2e-057.7e-054.3e-05

4.2e-057.7e-054.3e-05

2.1e-058.6e-050.0001695e-06

2.1e-058.6e-050.0001695e-06

0.00013

0.00013

0.0137320.0511650.0394510.06630.0198050.0122850.021851

0.0010160.0005147.2e-050.000994

0.0010160.0005147.2e-050.000994

0.0010160.0005147.2e-050.000994

0.0010160.0005147.2e-050.000994

0.0001480.0002980.000780.000203

7e-050.0002980.0006250.000132

2.6e-050.0001170.0002292.7e-05

2.6e-050.0001170.0002292.7e-05

4.4e-050.0001810.0003960.000105

3.4e-054.1e-052.8e-05

1e-050.0001810.0003557.7e-05

7.8e-050.0001557.1e-05

3e-060.0001031e-06

3e-060.0001031e-06

7.5e-055.2e-057e-05

7.5e-055.2e-057e-05

0.0002860.0002620.0012790.000297

3.2e-050.0001952.5e-05

2.1e-056.8e-052e-05

2.1e-056.8e-052e-05

1.1e-050.0001275e-06

1.1e-050.0001275e-06

2.4e-058.7e-052.8e-05

2.4e-058.7e-052.8e-05

2.4e-058.7e-052.8e-05

9.4e-050.0002620.0007750.000131

1e-050.0001141.3e-05

1e-050.0001141.3e-05

2.4e-056.1e-052.2e-05

2.4e-056.1e-052.2e-05

2e-063.3e-058e-06

2e-063.3e-058e-06

1.5e-050.0001110.0002171.6e-05

1.5e-050.0001110.0002171.6e-05

1e-062.9e-051.2e-05

4e-061e-06

09e-062e-06

1.2e-05

1e-064e-069e-06

1.5e-050.0001510.0002962.6e-05

1.5e-050.0001510.0002962.6e-05

2.7e-052.5e-053.4e-05

2.7e-052.5e-053.4e-05

0.0001360.0002220.000113

5.6e-056.9e-055.7e-05

5.6e-056.9e-055.7e-05

2.8e-057.3e-052.6e-05

4e-063.9e-053e-06

2.4e-053.4e-052.3e-05

5.2e-058e-053e-05

02e-060

2.6e-051e-061e-05

1.2e-051.3e-056e-06

6e-061.6e-056e-06

2.3e-053e-06

1e-061e-06

8e-062.3e-054e-06

1e-06

0.0122820.0511650.0383770.06630.0176740.0107910.021851

9.7e-058.7e-058e-05

9.7e-058.7e-058e-05

9.7e-058.7e-058e-05

0.0002382.3e-050.0001440.0003750.000262

0.0002382.3e-050.0001440.0003750.000262

0.0002382.3e-050.0001440.0003750.000262

0.0001657.9e-050.0001460.000157

0.0001657.9e-050.0001460.000157

0.0001657.9e-050.0001460.000157

0.0012810.0201920.0112870.0018130.000580.000119

0.0001610.0176470.0092190.0003440.000203

0.000147.5e-050.000155

0.0001230.00024

2.1e-050.0001044.8e-05

0.0161150.008238

0.0015320.000783

0.0023630.001208

0.0023630.001208

0.0001299.2e-050.0005310.000141

0.0001299.2e-050.0005310.000141

0.0008960.0001820.0007680.0008040.000139

0.0001568e-05

0.000180.000352

4.3e-055.7e-055e-05

0.0008350.0004275.7e-057.1e-05

5e-068.1e-050.0001589e-06

0.00013

1.3e-052.6e-055e-059e-06

9.5e-050.0001349.7e-05

9.5e-050.0001349.7e-05

0.000119

0.000119

1.3e-050.004820.0041880.0033759e-060.000178

1.3e-056.9e-059e-06

1.3e-056.9e-059e-06

9.1e-050.000178

9.1e-050.000178

0.0012570.002458

0.0012570.002458

0.004820.002407

0.000111

0.0047090.002407

0.0004330.000848

0.0004330.000848

0.0014260.0004510.0014450.0027250.0013050.000785

0.0001060.000207

0.0001060.000207

8.9e-055.7e-050.0001595.4e-05

4.9e-055.7e-058.5e-052.2e-05

4e-057.4e-053.2e-05

5.7e-055.1e-055.2e-05

2.1e-054.5e-051.7e-05

3.6e-054e-063.5e-05

2e-06

0.001280.0003940.0013390.0023080.0011990.000785

1.5e-05

3.1e-05

1.6e-056.6e-052.8e-05

6.2e-053.5e-054.1e-05

0.0001270.000249

4.4e-05

1.3e-05

8e-067.9e-050.0001555e-06

3.6e-051.4e-052.8e-05

0.0009530.0003860.00030.0004850.000895

1e-058e-062.3e-051.9e-05

08e-060

0.0001330.00026

0.0004010.000785

7.7e-052.2e-056.4e-05

5.3e-05

1.3e-058e-051.6e-05

1.5e-052e-061.5e-05

2.3e-052.6e-052.3e-05

2.1e-057.1e-052e-05

4e-063.6e-053e-06

0.0001430.000279

2.8e-053.7e-052.9e-05

7.4e-050.000144

1.4e-058.2e-050.000161.3e-05

0.000110.000214

0.000110.000214

0.000110.000214

0.0008310.0001370.0002440.06630.0005660.000714

0.0005110.0001560.0001770.000453

0.0005110.0001560.0001770.000453

7.2e-058.8e-050.000139

7.2e-058.8e-050.000139

8.8e-050.000173

8.8e-050.000173

0.0002480.0001370.06630.0001280.000122

0.0002480.0001370.06630.0001280.000122

3.4e-050.0003080.0006683.7e-05

2.6e-056.6e-052.9e-05

2.6e-056.6e-052.9e-05

8e-060.0003080.0006028e-06

8e-060.0003080.0006028e-06

0.0048940.0244890.0186360.0045460.004340.020769

2.6e-050.0001420.0002784.9e-05

2.6e-050.0001420.0002784.9e-05

0.0023820.001218

0.0022020.001126

0.000189.2e-05

0.0037780.0103460.0031120.0015420.003761

0.0037780.0072950.0015530.0015420.003761

0.0030510.001559

0.0002620.0065210.0005170.001250.0002190.000573

0.000260.000508

0.000140.0065010.0001760.000399.1e-050.000573

0.0001222e-058.1e-050.0003520.000128

0.000101

0.000101

0.0001310.000257

0.0001310.000257

0.000791.2e-050.0104990.0004430.0002550.020196

0.000791.2e-050.0002060.0004430.000255

0.0102930.020136

3e-05

3e-05

3.8e-058.2e-050.0001615.6e-05

3.8e-058.2e-050.0001615.6e-05

0.0051270.0029350.000615

0.0044660.002283

0.0006610.000338

0.0003140.000615

0.001440.0008620.0007150.0011020.001458

0.0014320.0008620.0006030.0008830.001451

0.0006545e-050.0003280.000610.000666

0.0007780.0008120.0002750.0002730.000785

8e-060.0001120.0002197e-06

8e-060.0001120.0002197e-06

5.3e-050.0001030.0002025.9e-05

5.3e-050.0001030.0002025.9e-05

5.3e-050.0001030.0002025.9e-05

0.001810.0001910.0011180.0018550.00179

0.0001290.0001960.0004830.000165

2.9e-057.8e-050.0001525.7e-05

0.0001180.000231

9e-064.6e-058e-06

9.1e-055.4e-050.0001

0.0011110.0005536.1e-050.001054

0.0011110.0005536.1e-050.001054

4.4e-05

4.4e-05

02.3e-051e-06

04e-061e-06

0

1.9e-05

0.00014.5e-050.0001520.0004040.000135

4.7e-057.4e-050.0001466.8e-05

4.2e-054.5e-057.8e-050.0001524e-05

1.1e-050.0001062.7e-05

0.0003570.0001460.0001040.0005610.000387

3.7e-056.1e-055.2e-05

2.2e-057e-051.4e-05

0.0002180.0001460.0001040.000220.000267

3.9e-050.0001024.1e-05

3.3e-05

8e-060.0001081.3e-05

1.1e-050.0001031.1e-05

1.1e-050.0001031.1e-05

5.8e-050.0001130.000223.7e-05

5.8e-050.0001130.000223.7e-05

1.5e-058.6e-050.0001681.2e-05

1.5e-058.6e-050.0001681.2e-05

1.5e-058.6e-050.0001681.2e-05

1.5e-058.6e-050.0001681.2e-05

1.5e-058.6e-050.0001681.2e-05

1.5e-058.6e-050.0001681.2e-05

1.8e-050.000120.0002362e-06

1.8e-050.000120.0002362e-06

1.8e-050.000120.0002362e-06

1.8e-050.000120.0002362e-06

1.8e-050.000120.0002362e-06

1.8e-050.000120.0002362e-06

9e-060.000130.0002841e-05

9e-060.000130.0002841e-05

9e-060.000130.0002841e-05

9e-060.000130.0002841e-05

9e-060.000130.0002841e-05

8e-060.000130.0002558e-06

1e-062.9e-052e-06

0.0002050.0005570.0013960.000289

3.5e-050.0001390.0002726e-05

3.5e-050.0001390.0002726e-05

3.5e-050.0001390.0002726e-05

3.5e-050.0001390.0002726e-05

3.5e-050.0001390.0002726e-05

1.4e-058.6e-050.0001681.2e-05

1.4e-058.6e-050.0001681.2e-05

1.4e-058.6e-050.0001681.2e-05

1.4e-058.6e-050.0001681.2e-05

1.4e-058.6e-050.0001681.2e-05

0.0001560.0003320.0009560.000217

0.0001560.0003320.0009560.000217

0.0001560.0003320.0009560.000217

1.2e-050.0001260.0002464.6e-05

1.2e-050.0001260.0002464.6e-05

5e-058.9e-050.0003045.4e-05

1.8e-050.000133.2e-05

3.2e-058.9e-050.0001742.2e-05

3e-050.0001170.0002293.1e-05

3e-050.0001170.0002293.1e-05

6.4e-050.0001778.6e-05

3e-050.0001334.9e-05

3.4e-054.4e-053.7e-05

6.8e-050.0001090.0002122.5e-05

6.8e-050.0001090.0002122.5e-05

6.8e-050.0001090.0002122.5e-05

6.8e-050.0001090.0002122.5e-05

6.8e-050.0001090.0002122.5e-05

6.8e-050.0001090.0002122.5e-05

0.3000620.4361720.0795860.2380.0757210.2871820.083817

0.3000620.4361720.0795860.2380.0757210.2871820.083817

0.0004240.0002920.0011060.00036

6.4e-050.0001437e-05

6.4e-050.0001437e-05

6.4e-050.0001437e-05

0.000360.0002920.0009630.00029

0.000360.0002920.0009630.00029

8.7e-050.0001140.0002226.2e-05

3.2e-059.3e-050.0001831.8e-05

5.7e-056.1e-055.7e-05

3.4e-053.6e-052e-05

0.000142

1.3e-05

5e-058.5e-050.0001664.8e-05

8.5e-053.1e-057.3e-05

5e-05

1.6e-05

1.5e-054.3e-051.2e-05

0.2996380.4361720.0792940.2380.0746150.2868220.083817

0.2996380.4361720.0792940.2380.0746150.2868220.083817

0.0001090.0003330.000127

0.0001090.0003330.000127

9.4e-057.7e-050.0003040.0001

9.4e-057.7e-050.0003040.0001

0.0009050.0005630.000270.000916

0.0008880.0004555.9e-050.000895

1.7e-050.0001080.0002112.1e-05

0.298530.4361720.0786540.2380.0737080.2856790.083817

1.6e-05

1.3e-059.6e-050.0001872.2e-05

0.0001390.000150.001425

3.5e-051.2e-059.1e-053.1e-05

3.1e-050.0001610.0003143.9e-05

0.2984510.436160.0782580.2380.072950.2855870.082392

0.0001650.000323

0.0001650.000323

0.0001650.000323

0.0001650.000323

0.0001650.000323

0.0001650.000323
